# Genomic and transcriptomic analysis of the Asian honeybee *Apis cerana* provides novel insights into honeybee biology

**DOI:** 10.1038/s41598-017-17338-6

**Published:** 2018-01-16

**Authors:** Qingyun Diao, Liangxian Sun, Huajun Zheng, Zhijiang Zeng, Shengyue Wang, Shufa Xu, Huoqing Zheng, Yanping Chen, Yuanyuan Shi, Yuezhu Wang, Fei Meng, Qingliang Sang, Lianfei Cao, Fang Liu, Yongqiang Zhu, Wenfeng Li, Zhiguo Li, Congjie Dai, Minjun Yang, Shenglu Chen, Runsheng Chen, Shaowu Zhang, Jay D. Evans, Qiang Huang, Jie Liu, Fuliang Hu, Songkun Su, Jie Wu

**Affiliations:** 1 Institute of Apicultural Research, Chinese Academy of Agricultural Sciences, Beijing, 10093 China; 2 College of Animal Sciences, Zhejiang University, Hangzhou, 310058 China; 3 Molecular Biology and Pharmacology Key Laboratory of Fujian Advanced Education, Quanzhou Normal University, Quanzhou, Fujian, 362000 China; 4 Shanghai-MOST Key Laboratory of Health and Disease Genomics, Chinese National Human Genome Center at Shanghai, Shanghai, 201203 China; 5 Honeybee Research Institute, Jiangxi Agricultural University, Nanchang, Jiangxi 330045 China; 6 USDA-ARS Beltsville Bee Research Laboratory, Beltsville, Maryland 20705 USA; 7 College of Bee Science, Fujian Agriculture and Forestry University, Fuzhou, 350002 China; 8 Bioinformatics Laboratory and National Laboratory of Biomacromolecules, Institute of Biophysics, Chinese Academy of Sciences, Beijing, 100101 China; 9 ARC Centre of Excellence in Vision Science, Research School of Biology, College of Medicine, Biology and Environment, The Australian National University, Canberra, ACT 2601 Australia

## Abstract

The Asian honeybee *Apis cerana* is one of two bee species that have been commercially kept with immense economic value. Here we present the analysis of genomic sequence and transcriptomic exploration for *A. cerana* as well as the comparative genomic analysis of the Asian honeybee and the European honeybee *A. mellifera*. The genome and RNA-seq data yield new insights into the behavioral and physiological resistance to the parasitic mite *Varroa* the evolution of antimicrobial peptides, and the genetic basis for labor division in *A. cerana*. Comparison of genes between the two sister species revealed genes specific to *A. cerana*, 54.5% of which have no homology to any known proteins. The observation that *A. cerana* displayed significantly more vigilant grooming behaviors to the presence of *Varroa* than *A. mellifera* in conjunction with gene expression analysis suggests that parasite-defensive grooming in *A. cerana* is likely triggered not only by exogenous stimuli through visual and olfactory detection of the parasite, but also by genetically endogenous processes that periodically activates a bout of grooming to remove the ectoparasite. This information provides a valuable platform to facilitate the traits unique to *A. cerana* as well as those shared with other social bees for health improvement.

## Introduction

Of the ten honeybee species in the genus *Apis*, only the multiple-comb bee species, the European honeybee *Apis mellifera* and the Asian honeybee *Apis cerana*, have been commercially kept for crop pollination with immense economic and ecological value^1–4^. Nevertheless, in recent decades, there have been escalating concerns that populations of honeybees are in sharp decline as a result of the loss of habitat, introduction of invasive species, emergence of new pathogens and parasites, persistence of chemical residues and other environmental threats^5–9^. One of the most serious threats to honeybees is Colony Collapse Disorder (CCD), a mysterious plague that decimated entire bee colonies during the winter of 2006–2007 in the US and yet remains unsolved^10^. Hence, a genome-based approach to understanding the molecular mechanisms that regulate population dynamics of honeybees has been an integral part of pollinator conservation efforts. The complete genome sequence of *A. mellifera* has provided the first insights into honeybee biology and evolution^11^. To deepen our understanding of the complex social behavioral traits of honeybees, genome sequences of related species will enhance the understanding of the genetic basis associated with adaptive changes within and between honeybee species.

*A. cerana* is endemic to Asia with six morphologically distinct subspecies^3,12,13^ distributed throughout a series of climatic zones on the Asian landscape and has been used for pollination and commercial beekeeping over thousands of years in Asia. As close relatives, *A. cerana* and *A. mellifera* are very similar in morphology and behavior. Despite this, *A. cerana* has several distinct biological characteristics when compared with *A. mellifera*. For example, workers of *A. cerana* ventilate their hive with their heads pointing toward the outside which is the opposite of *A. mellifera* workers which fan with their head toward the entrance; foragers of *A. cerana* are good at collecting nectar from scattered floral resources that are often neglected by foragers of *A. mellifera*; *A. cerana* does not collect propolis, a resinous material used by *A. mellifera* to seal apertures in the hive and defend against pathogens. Moreover, as an indigenous species of Asia, *A. cerana* has evolved a series of striking biological characteristics to combat the adverse climatic conditions of their habitats. Foragers of the Chinese black honeybee (*A. c. cerana*) visit the flowers of *Eurya spp* under cloudy conditions when the air temperature is as low as 7 °C, a temperature at which the *A. mellifera* enters into a torpid state. *A. cerana* also has developed a diverse set of defense mechanisms to combat the invasion of predators, parasites and pathogens^14,15^. Compared to the steady and clumsy flight of *A. mellifera*, the flight of *A. cerana* is rapid, hasty and unpredictably zigzagging, which helps in escaping from hornets and bee-eating birds^3^. *A. cerana* is the native host to the ectoparasitic mite *Varroa destructor*^16^ which is the single most detrimental pest of *A. mellifera*, and has evolved high resistance to this pest over a long period of mutual adaptation^17^. Its workers can effectively remove mites from both adults and brood by performing a series of cleaning behaviors^18,19^.

Now many regions of Asia are suffering from a shortage of pollinators for plants and reduction of biodiversity^3,20,21^. The parasites and pathogens that have caused serious disease problems in *A. mellifera* were also found in *A. cerana*^22^. Therefore, it is urgent to take measures to protect *A. cerana*. Even so, compared to the wealth of genetic information available from *A. mellifera* which is used as a key model animal for social behavior^11,23^, the knowledge of *A. cerana* is limited.

Here we present our complementary efforts of a high-quality assembly and annotation of the southern strain of *A. cerana* genome and detailed analyses of its evolutionary history of domestication and selection. In particular, we identify and describe the genome variations that regulate different aspects of worker behavior and physiology between *A. cerana* and *A. mellifera*. Most importantly, we annotate the genome by coupling RNA-Seq analysis and laboratory assays to test gene functions and provide compelling experimental evidence of the molecular mechanisms underlying the *Varroa* mite resistance characteristics of *A. cerana*. These data provide a novel insight into honeybee biology and might be utilized to improve bee health globally.

## Results

### Genome sequencing and assembly

We sequenced 23.7 giga-bases (Gb) of Illumina pair-end reads, 8.7 Gb of Illumina mate-pair reads, and 636 mega-bases (Mb) of 454 GS FLX shotgun sequences using genomic DNA extracted from two haploid drone pupae from one *A. cerana* colony (Supplementary Table 1). A total of 21,784 contigs of 209.2 Mb, ranging from 500 bp to 174,956 bp with N50 of 21,160 bp were assembled. All contigs were constructed into 879 scaffolds with a total length of 228.8 Mb and N50 of 1.39 Mb (Supplementary Table 2), coinciding with the estimated genome size of 226 Mb calculated by k-mer (Supplementary Fig. 1).

A comparison with the *A. mellifera* genome (V4.5) demonstrated consistency between two genomes (Supplementary Fig. 2). Transcriptome sequencing of a sample of mixed brains of *A. cerana* workers produced 469,162 ESTs with an average length of 390 bp and 99% of the ESTs could be mapped on the *A. cerana* genome, suggesting the *A. cerana* genome covered most genes. The assessment of *A. cerana* genome coverage using 469,162 ESTs showed that the accumulative numbers of mapped EST aligned over 100%, 90%, 80%, 70%, 50%, and 20% of their length was 308128, 451855, 462797, 465583, 467197, and 467952, respectively. The accumulative mapped ratio aligned over 100%, 90%, 80%, 70%, 50%, and 20% of their length was 65.68%, 96.31%, 98.64%, 99.24%, 99.58%, and 99.74%, respectively.

**Figure 1:**
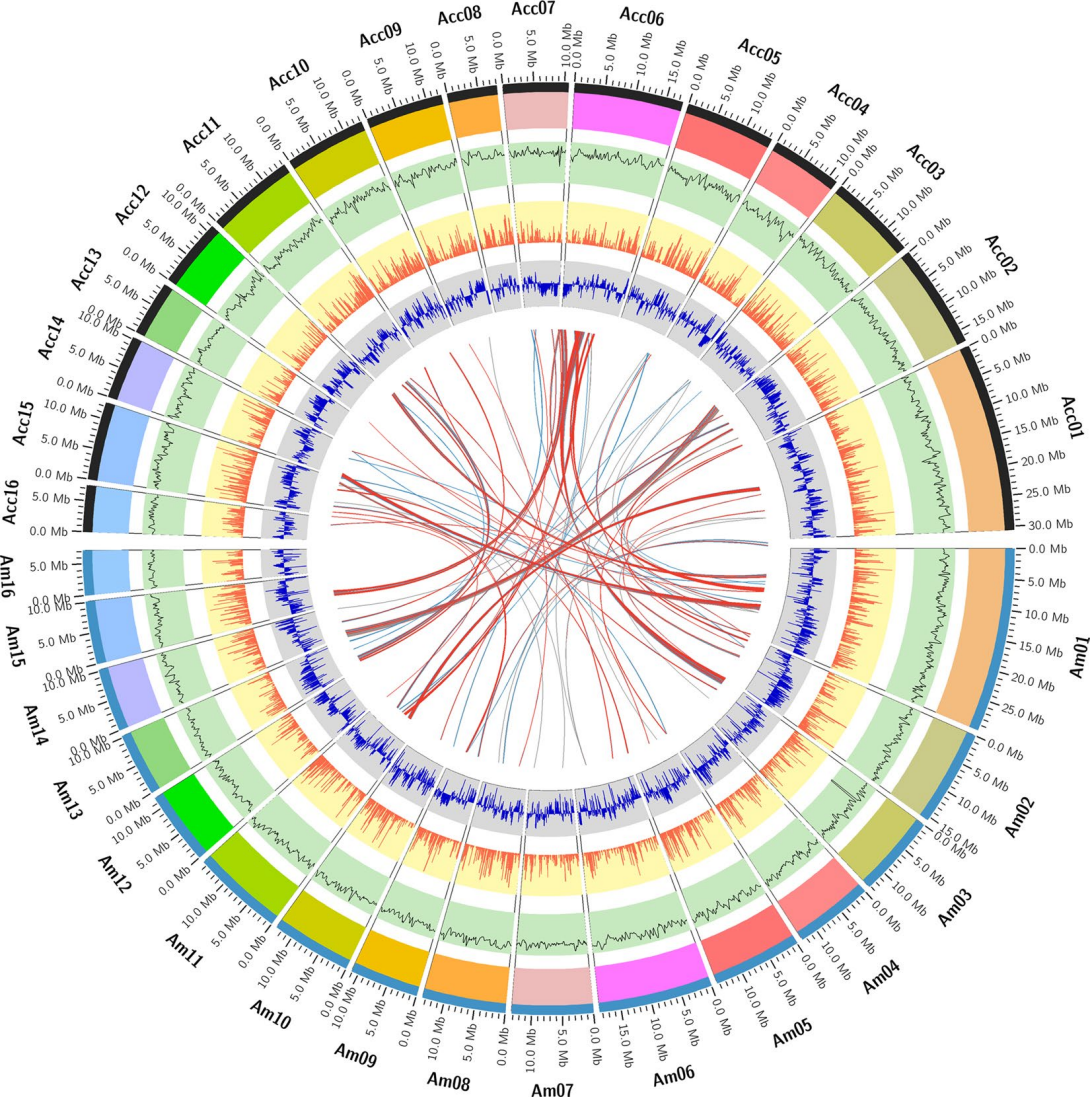
The atlas of *A. cerana* chromosomes and comparison to *A. mellifera*. The 16 chromosomes of *A. cerana* and *A. mellifera* genomes were drawn in the upper and bottom part, respectively. From outer side to inside, each circle represents the chromosome, genome homology, gene density, GC content and the translocation between the two species.

Depending on a SNP-based *A. cerana* linkage map^24^ and *A. mellifera* DH4 linkage group, we linked 370 *A. cerana* scaffolds into 16 chromosomes, with a total size of 214.19 Mb (Fig. 1, Supplementary Table 3, Supplementary Fig. 3).

We found only 4.2% of the genome could be defined as repeat region, mainly composed of microsatellites (2.63%) (Supplementary Fig. 4). A total of 104,983 microsatellites were revealed in the genome, with an average distance of 1,948 bp and 10 repeat units in each microsatellite. Among them, (AT)n and (AG)n occupied 25% and 19%, respectively.

### Genome characterization and annotation

By combining a gene prediction program based on EST alignments (Gnomon) and three *ab initio* prediction programs (GeneMark.hmm, Augustus and Snap) based on the *A. mellifera* model, we identified 10,182 protein-encoding genes in the *A. cerana* genome, with an average gene size of 7,577 bp and an average CDS size of 1,695 bp. The largest CDS was 27,474 bp long (*ACC_02761*), comprising 67 exons and encoding a myosin light chain kinase. The average exon and intron sizes were 237 bp and 988 bp, respectively (Table 1). The intron size of *A. cerana* is 250 bp smaller than that of *A. mellifera*, explaining the contradiction of bigger CDS length but smaller gene size for *A. cerana* (Supplementary Fig. 5). A total of 571 genes were predicted without introns (Supplementary Table 4).

**Table 1:** Gene Character of *A.cerana* vs. *A. mellifera*.

	*A. cerana*	*A. mellifera (Glean3)*
Gene Number	10,182	10,157
Avg. CDS Length	1,695 bp	1,592 bp
GC content	37.96%	39.43%
Total coding size	17,260,536 bp (7.5%)	16,484,776 bp (7.2%)
Avg. Gene Length	7,577 bp	8,288 bp
Avg. Exon Number	7.1	6.4
Avg. Exon Size	237 bp	254 bp
Avg. Intron Size	988 bp	1,234 bp
tRNA	171	213
Orthologs*	9,353	9,428
Unique Gene#	812	711
Identity	96.60%	
Intron Identity	92.50%	

^*^Genes with homology on DNA level.

^#^Genes with no homology on either DNA or Protein level.

BLASTP searches revealed that 9,911 (97.4%) of *A. cerana* proteins have homology with other known proteins. InterproScan assigned IPR domains to 8,767 genes, and 7,343 proteins were assigned GO terms. 3,849 proteins have Kyoto Encyclopedia of Genes and Genomes (KEGG) orthologs with 2,237 of them involved in at least one pathway. Eukaryotic Orthologous Groups (KOG) analysis assigned 7,713 *A. cerana* genes into different KOG categories, and 13.97% of these genes were involved in diverse signaling pathways, a similar ratio as in other insects like ants and bees, coinciding with the complex behavior of *A. cerana*. To study the gene roles of *A. cerana* genes in different stages, we performed RNA-seq for 16 different *A. cerana* samples (Supplementary Table 5).

### CpG and DNA methylation

The *A. cerana* genome had an overall GC content of 32.97%. Based on observed CpG/expected CpG ratio, CpG dinucleotide distribution in *A. cerana* genes showed a di-peak pattern (Supplementary Fig. 6), with 2,577 genes under-represented (0.53-fold, higher methylated genes) and 2,297 genes over-represented (1.15 fold, rarely methylated genes) compared to the expectation from mononucleotide frequencies (Supplementary Information). It is known that the CpG deficiency in mammalian genomes represents a mutational hot spot through deamination of methylcytidine to thymidine.

There were a total of 793 lower CpG content genes and 248 higher CpG content genes that were involved in different pathways and showed significantly different enrichment (Supplementary Table 6). Lower CpG content genes showed significant enrichment in N-Glycan biosynthesis and protein processing in the endoplasmic reticulum (Supplementary Fig. 7) suggesting that genes of the two pathways were subject to germline methylation and subsequent deamination from a genomic history. N-glycans in invertebrate allergens (like bee venom) are a common cause of immunoglobulin (Ig) E cross-reactivity *in vitro* and are commonly known as cross-reactive carbohydrate determinants (CCDs). Higher CpG contentgenes showed significant enrichment in neuro-active ligand-receptor interactions, Cell Adhesion Molecules (CAMs) and insect hormone biosynthesis (Supplementary Fig. 8).

### Comparison with other insects and unique genes

We compared six social insects including *A. cerana, A. mellifera, A. florea, Bombus impatiens, Camponotus floridanus* and *Harpegnathos saltator* with five non-social insects *Drosophila melanogaster, Anopheles gambiae, Megachile rotundata, Bombyx mori* and *Nasonia vitripennis* at the proteome level. By searching the Conserved Domain Database (CDD) with RPS-BLAST at E-value of 1e-5, we uncovered 2,010 domains conserved across all of the insects, and three domains uniquely to *A. cerana* (Table 2, Supplementary Table 7).

**Table 2:** Comparison of domains among insects.

Species	shared by all taxa	shared by ten taxa	shared by nine taxa	shared by eight taxa	shared by seven taxa	shared by six taxa	shared by five taxa	shared by four taxa	shared by three taxa	shared by two taxa	unique
*A. cerana*	2010	580	225	145	71	49	23	14	9	12	3
*A. mellifera*	2010	554	208	126	64	33	19	11	10	25	64
*C. floridanus*	2010	560	215	129	62	43	30	24	25	18	16
*H. saltator*	2010	571	194	136	68	38	28	24	24	23	13
*A. gambiae*	2010	541	198	110	59	32	35	41	48	80	23
*B. mori*	2010	428	138	68	38	28	27	24	35	29	33
*D. melanogaster*	2010	581	217	119	64	36	34	42	53	90	76
*N. vitripennis*	2010	484	163	115	40	36	26	26	26	23	16
*A. florea*	2010	538	203	119	60	35	17	17	11	17	19
*B. impatiens*	2010	583	226	142	68	43	33	20	20	44	166
*M. rotundata*	2010	590	227	135	71	41	28	25	15	13	10

A total of 4,844 genes of *A. cerana* are confirmed as single copy, with less than 30% coverage and 20% identity with other genes in the whole proteome. The gene distribution in pathways was studied in detail (Supplementary Table 8). Non-social insects had more KEGG Orthologies (KOs) (144) than social insects (130) for the immune system, consistent with previous suggestions that the reduction of immune related genes at the individual level in honey bees may be a result of strengthened collective colony-level immunity (social immunity)^11,25,26^. However, the new evidence indicated that xenobiotic detoxification and immune genes are similarly depauperate in both honeybees and bumblebees which have colony lifestyles lying between completely solitary bees and highly eusocial species such as the honey bees^27^. The number of immune genes remains consistent regardless of bees’ degree of sociality suggests that a depauperate immune repertoire precedes evolution of sociality in bees and the differences in selection on immune genes likely reflect divergent pressures exerted by parasites across social contexts^27,28^.

Among the social insects, we only identified one unique KO (K12796) relative to non-social insects, which encoded the erbb2-interacting protein and was involved in the NOD-like receptor signaling pathway. Domain comparison and KOG analysis showed that there is no significant statistical difference between *A. cerana* and *A. mellifera* or *A. florea*.

**Figure 2:**
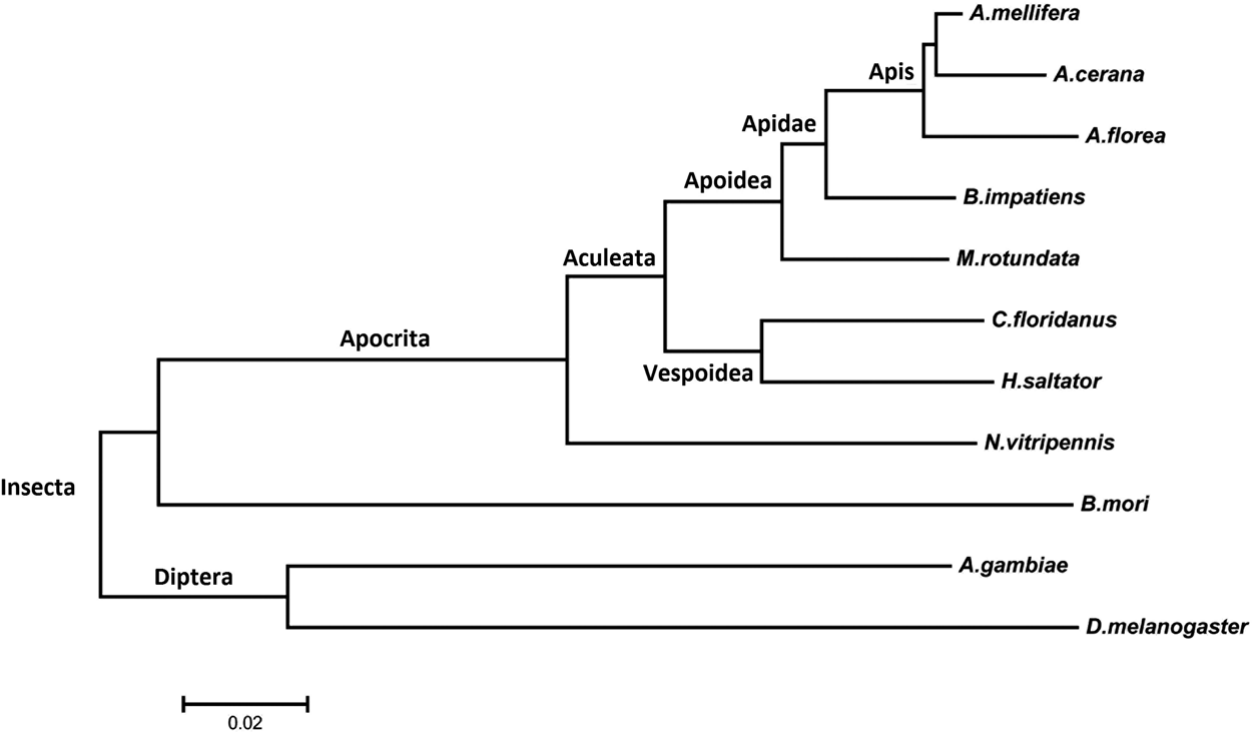
Evolutionary position of *A. cerana*.

To better understand the evolutionary position of *A*. *cerana*, 155 single copy genes with the best hit to another 10 species were chosen to create a phylogenetic tree. It is clear that *A*. *cerana* and *A. mellifera* were located together, and showed a close relationship with the other three species of Apoidae. Relative to ants (*C. floridanus* and *H. saltator*), the parasitoid wasp (*N. vitripennis*) showed a more distant relationship with *A. cerana* (Fig. 2, Supplementary Table 9).

8,202 orthologous gene pairs were identified between *A. cerana* and *A. mellifera* using Bidirectional Best Hit (BBH). By measuring the synonymous substitution rates (Ks) of orthologous gene pairs, we obtained the mean Ks of 0.149 and the peak Ks of 0.095. The mean sequence identity between *A. cerana* and *A. mellifera* homologous genes are 96.6%, with intron regions showing 92.5% identity. In addition, only 19 genes out of these 8,202 orthologs were under positive selection (Supplementary Table 10).

A comparison of *A. cerana* genes against genes of *A. mellifera* and *A. florea* revealed that 433 genes are specific to *A. cerana*, with 408 of them validated by RNA-seq data (Supplementary Table 5), though 236 genes (54.5%) have have no homology to any known proteins in the database like NR, KEGG and KOG (Supplementary Table 11). These *A. cerana* unique genes might contribute to the specific characteristics of the *A. cerana*. For example, *ACC_04782* and *ACC_08219* encode proteins involved in sensory perception of smell and sensory perception of taste, respectively; three NADH-ubiquinone oxidoreductase genes, which participate in oxidative phosphorylation, are unique in *A. cerana* compared with *A. mellifera*.

Three unique genes (*ACC_00190*, *ACC_01436*, and *ACC_04893*) encode MLE (*maleless*) which is one component of the dosage compensation complex, and the encoded MLE proteins have no homology at the amino acid level with *A. mellifera* or *A. florea*. Dosage compensation is a genetic regulatory mechanism, which balances systems of higher eukaryotes’ genome by equalizing the phenotypic expression of an unequal copy of sex chromosomes in the two sexes^29^. Five components are the core subunit of the dosage compensation complex (DCC) in *Drosophila*^30^, including MLE, MSL1, 2, 3 (*male-specific lethal 1*, 2 and 3) and MOF (*males absent on the first*). Unlike *A. mellifera*, lacking some component of X-specific dosage compensation^11^, the *A. cerana* genome has a full set of genes encoding the components of DCC. Genes, *msl1*, 2 and 3 (*ACC_01543*, *ACC_01587* and *ACC_04118*), are all detected in *A. cerana*. We identified five genes (*ACC_04472*, *ACC_00332*, *ACC_05236*, *ACC_01279* and *ACC_02931*) as *Mof* genes with histone acetyltransferase activity according to previous reports by Gu *et al*. (2000) and Hilfiker *et al*. (1997)^31,32^. According to KOG analysis, a total of 18 genes encode for MLE, of which three of them are unique for *A. cerana*. There are three NADH-ubiquinone oxidoreductase genes in *A. cerana*, and all of them are unique genes when compared with *A. mellifera*. The NADH-ubiquinone oxidoreductase gene participates in the process of oxidative phosphorylation.

### Homeobox proteins

A total of 114 homeobox protein genes were revealed in the genome (Supplementary Table 12), including six genes encoding Paired box (Pax) proteins which are involved in the visual system development of the metazoan^33^. Among them, two Pax-6 genes, *eye gone* (*eyg*) and *eyeless* (*ey*) were known to act cooperatively in promoting eye development^34^, with *eyg* controlling eye growth and *ey* controlling eye specification^35^. Their duplicate genes, *twin of eye gone* (*toe*) plays only a subtle role in the *Drosophila* eye imaginal disc^36^, and *twin of eyeless* (*toy*) is required for initiation of *ey* expression in the embryo and acts through *ey* to activate eye development^37^. Phylogenetic analysis (Supplementary Fig. 9) revealed two *A. cerana* proteins (ACC_02837 and ACC_09296) located within the same clade with *Eyg* and *Toe*, indicating a possible role in eye growth by promoting cell proliferation. This is supported by the gene expression profile analysis which revealed that *ACC_02837* only expressed in the pupal stage. Another *A. cerana* protein, ACC_01021, is located in the same branch as *Ey* and *Toy*, indicating its main role might be involved in retinal specification^36^.

### Apitoxin

Apitoxin, or honeybee venom, is a complex mixture of proteins/peptides that cause an allergic reaction in humans. The main components of apitoxin include melittin, phospholipase A2 (PLA2), hyaluronidase, apamin, histamine, dopamine, noradrenaline^38^, acid phosphatase^39,40^, CUB serine protease, and Api m 5^41^. We revealed 73 apitoxin candidate genes in the *A. cerana* genome (Supplementary Table 13), including the above-mentioned compounds and homologues of allergens from other species, like snake venom vascular endothelial growth factor toxin (ACC_00031). Among these components, phospholipase A2 (ACC_09897) is the most lethal^42^, together with hyaluronidase (ACC_10171), acid phosphatase (ACC_02702) and Api m 5 (ACC_09285), mainly contributing to IgE-mediated allergic reactions^39,41^. Melittin (ACC_10176), considered as a minor allergen, is the most abundant, occupying 50% of the venom^38^.

**Figure 3:**
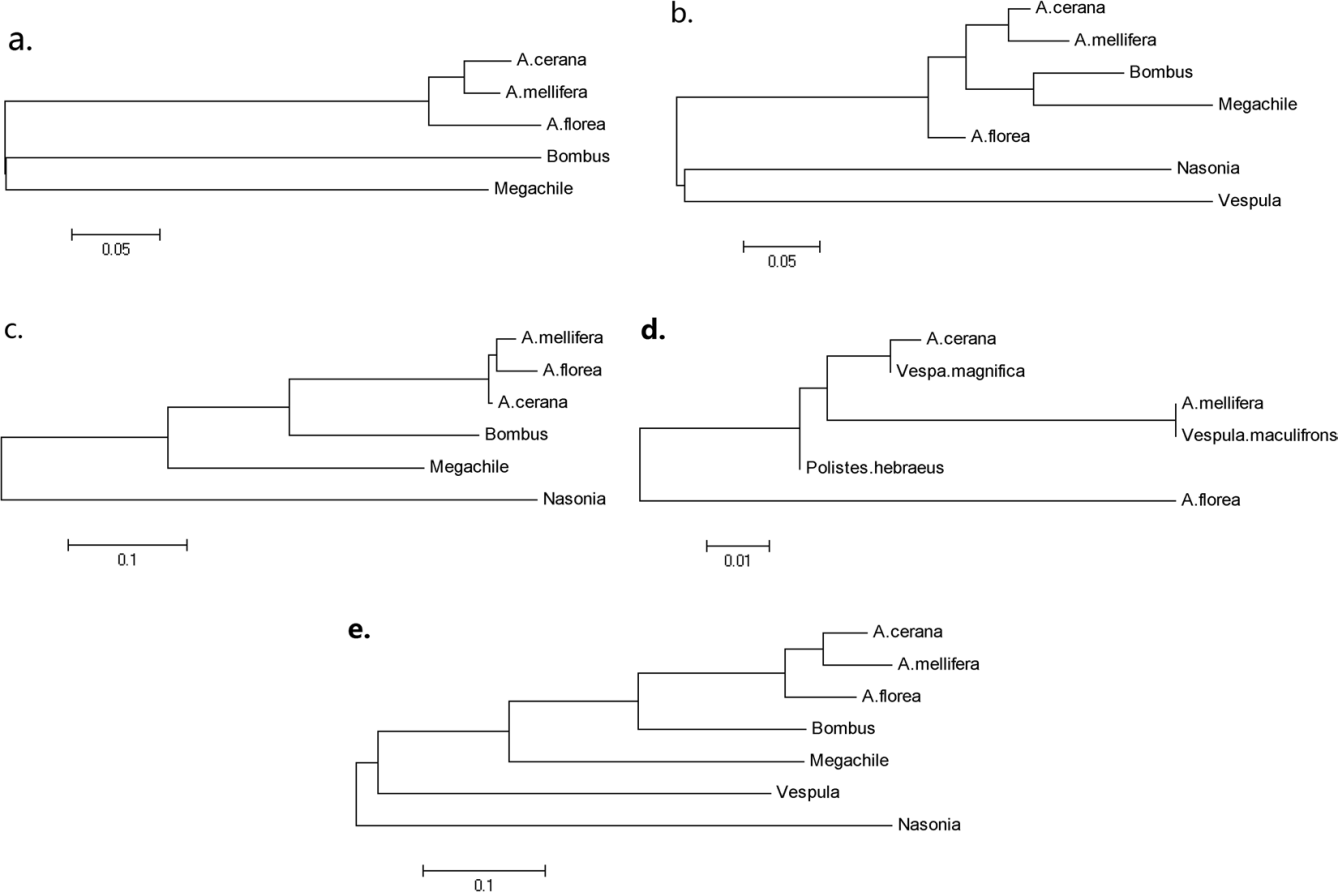
Phylogenetic trees of Allergen genes. The phylogenetic trees were constructed with DNA sequences of *Api m 1* (**a**), *Api m 2* (**b**), *Api m 3* (**c**), *Api m 4* (**d**) and *Api m 5* (**e**), by the neighbor-joining method (1,000 bootstrap replicates) using MEGA v.5.

Though phylogenetic analysis had shown that *A. cerana* is most closely related with *A. mellifera*, the melittin gene (*Api m 4*) of *A. cerana* shows 99% identity with that of *Vespa magnifica* while only 93% identity with *A. mellifera*, which in turn, showed 100% identity with *Vespula maculifrons* prepromelittin (Fig. 3). A test for positive selection was performed on the five genes (*Api m 1*-*Api m 5*) encoding the major allergens and melittin (Supplementary Table 14). Noteworthy, the *Api m 5 gene* experienced position selection (dn/ds = 2.25) during the sequence divergence between *A. cerana* and other bees. We did not identify the complete coding sequence of another minor allergen *Api m 6*^43^ in the *A. cerana* genome. We compared the genomic structure of *Api m 6* locus in *A. mellifera* with the corresponding region of *A. cerana* genome (Supplementary Fig. 10) and found that the 2^nd^ exon of *Api m 6* was disrupted in the *A. cerana* genome, perhaps resulting in a degenerate gene. The residues 37–91 of *Api m 6* encoded a trypsin inhibitor-like cysteine rich (TIL) domain, whose coding region was not affected by the disruption.

### Immune System

Honeybees, like all insects, lack an adaptive immune system and have evolved an “innate immunity” to fight against foreign invaders. The immune system of *A. cerana* consists of 144 genes and 12 pathways (Supplementary Table 15), with six more genes and three more KOs than *A. mellifera* (Supplementary Table 8). Among them, 46 genes constitute the chemokine signaling pathway, which regulates inflammatory immune response by transducing chemokine signals through G-protein-coupled receptors (GPCR) expressed on the immune cells. There are 43 GPCR genes in the *A. cerana* genome (Supplementary Table 16) associated with neuropeptides, vision, odor, etc.

Adenylate Cyclase (AC) → cyclic adenosine monophosphate (cAMP) → Protein Kinase A (PKA) cascade is associated with many intracellular signaling pathways, such as calcium signaling, insulin secretion, circadian entrainment, and chemokine signaling pathway. *A. mellifera* has seven AC genes^44^, while *A. cerana* has eight AC genes, and seven of them are membrane-bound and one soluble (*ACC_10007*). AC8 is prominently expressed in the mushroom bodies^45^ which play important roles in some forms of associative learning and memory formation, such as olfactory memory^45^. Besides PKA-C3 (*ACC_04702*), AC8 (*ACC_01531*, membrane-bound) was highly expressed in worker pupae (p < 1e-4), suggesting that AC8 might be highly expressed in mushroom bodies in the pupal stage of workers for promoting olfactory system formation.

Fc gamma R-mediated phagocytosis plays an essential role in host-defense mechanisms through the uptake and destruction of infectious pathogens. The 36 candidate genes which involve in recognition of infectious pathogens through Fc gamma receptors and gamma R-mediated phagocytosis were identified in *A. cerana*, suggesting phagocytosis could be another important immune defense mechanism of *A. cerana*. Arp2/3 complex is highly conserved in almost all eukaryotes and has the capability to initiate actin filament formation^46,47^. In the *A. cerana* genome, the actin related protein 2/3 complex was intact and included two actin-related proteins, Arp2 (*ACC_00048*) and Arp3 (*ACC_01435*), and five subunits (ARPC1–5, *ACC_01746*, 00828, 08843, 09274, 07187). ARPC3, ARPC5, and Arp2, were all highly expressed in the dance stage (p < 1e-15) and ARPC5 was also highly expressed in the forage stage (p < 1e-25). Knockout of genes encoding ARPC3 leads to a defect of sustained migration directionality and a delay in wound closure in mouse fibroblast^48^. Perhaps the Arp2/3 complex is crucial for damage healing.

Honeybees are affected by a wide array of xenobiotic compounds. Three enzyme families, the cytochrome P450 - (P450), glutathione transferases (GST) and carboxy/cholinesterases (CCE) catalyze a wide range of detoxification reactions. *A. cerana* has 27, 52 and 12 genes encoding CCE, P450 and GST enzymes (Supplementary Table 17), respectively, with a similar number of genes found in *A. mellifera*^44^.

We found seven *A. cerana* genes in *A. cerana* encoding *apidaecin, hymenoptaecin*, *defensin, apisimin*, and *abaecin*, respectively (Supplementary Table 18). While all these antimicrobial peptide (AMPs) genes were also found in *A. mellifera*, it had been reported that *A. cerana* expresses many more transcripts of AMPs than *A. mellifera*^49^, suggesting that *A.cerana* might exhibit a stronger protecting ability against pathogens through encoding variable AMPs.

RNA-seq was performed to determine the AMP gene expression profiling in the brain of *A. cerana* nurses, guard bees, and foraging bees. The newly-emerged workers were set as a control. There were differences in the patterns of the AMP gene expression among *A. cerana* workers specializing in different tasks (Supplementary Fig. 11). For both *abaecin* and *defensin2*, the expression levels increased with age. The remarkable increase happened from nursing to guarding for *abaecin* while that from guarding to foraging for *defensin2* (Supplementary Fig. 11). The mRNA levels of *hymenoptaecin*, *apidaecin* and *defensin1* fluctuated with caste transition. Of them, nearly the same pattern was seen in *hymenoptaecin* and *apidaecin* gene expression and their transcriptions both peaked at the guarding caste then decreased sharply to a low level. However, for *defensin1*, its highest level of expression was seen in the nursing stage (Supplementary Fig. 11).

### Genes underlying complex grooming and body shaking behaviors

Hygienic and grooming behaviors are important defensive mechanisms of honeybees against the parasitic mite *V. destructor*. The grooming behavior involves the ability of bees to shake, swipe, and bite *Varroa* mites from their bodies while the hygienic behavior involves the ability of bees to detect, uncap, and remove mite-infested brood^17,50,51^. Compared with *A. mellifera, A. cerana* possesses desirable behavioral traits including body shaking, rolling and tomb uncapping when they are infested by *Varroa* mites^17,52^, in addition to the behavioral strategies of grooming and hygienic behavior^15^. The results of our bioassay for grooming effectiveness towards *Varroa* mites confirmed that *A. cerana* and *A. mellifera* exhibited significant differences in their behavior responses to *Varroa* mites (Table 3). *A. cerana* workers responded immediately and vigorously to the presence of *Varroa* mites and displayed an average of 122 times of intensive body shaking, 228 times of light to moderate body shaking, 36 times of self-grooming, and 9 times of allogrooming within five minutes. In most of the cases, mites were finally removed from the body of *A. cerana* workers. Meanwhile, *A. mellifera* workers showed insignificant responses to the presence of *Varroa* mites in comparison to *A. cerana*. Their responses were more limited to the light to moderate body shaking and self-grooming, an average of 22.6 times and 5.93 times, respectively. The same, effective behaviors were rarely observed in *A. mellifera* workers.

**Table 3:** Behaviors in response to the exposure of *Varroa* mite.

Groups		Shaking (*Total time in five minutes*)				Grooming (*# of times in five minutes*)			
		Intensive Shaking^1^ (Mean ± SD)		Normal shaking^1^ (Mean ± SD)		Self-grooming^2^ (Mean ± SD)		Allogrooming^2^ (Mean ± SD)	
Apis cerana (without mite)	I	3.00 ± 2.00		20.80 ± 3.01		1.80 ± 0.20		N/A	
	II	1.00 ± 1.00		16.60 ± 1.54		2.00 ± 0.00		N/A	
	III	0.80 ± 0.80		16.00 ± 1.41		2.40 ± 0.24		N/A	
	Ave	1.60 ± 0.97	**a**	17.80 ± 4.90	**a**	2.07 ± 2.40	**a**		
Apis cerana (with mites)	I	120.00 ± 14.00		220.00 ± 14.47		32.80 ± 5.24		8.00 ± 3.40	
	II	117.00 ± 15.09		254.40 ± 13.74		39.40 ± 4.93		10.40 ± 2.02	
	III	130.00 ± 13.16		210.20 ± 17.07		36.20 ± 3.48		10.80 ± 4.32	
	Ave	122.33 ± 19.44	**b**	228.37 ± 14.96	**b**	36.13 ± 6.02	**b**	9.73 ± 4.46	**a**
Apis mellifera (without mite)	I	N/A		26.40 ± 0.93		1.60 ± 0.25		N/A	
	II	N/A		24.00 ± 0.71		1.60 ± 0.25		N/A	
	III	N/A		24.00 ± 1.30		1.00 ± 0.32		N/A	
	Ave			24.80 ± 5.88	**a**	1.40 ± 0.68	**a**		
Apis mellifera (with mite)	I	0.80 ± 0.80		19.80 ± 0.80		5.40 ± 1.03		1.00 ± 0.00	
	II	2.00 ± 2.00		23.60 ± 1.78		6.60 ± 1.66		1.40 ± 0.24	
	III	1.60 ± 1.16		24.40 ± 1.72		5.80 ± 2.06		1.60 ± 0.24	
	Ave	1.47 ± 0.97	**a**	22.60 ± 8.30	**a**	5.93 ± 4.77	**c**	1.33 ± 1.08	**b**

^1^Seconds; ^2^number of times

One-way ANOVA. Tukey’s HSD (honestly significant differences), p < 0.05. Different letters indicate statistically significant difference among different groups (N = 15).

From the transcriptomic study of *A. cerana* workers exhibiting *Varroa-*induced grooming and body shaking behaviors, 502 genes were significantly (FDR < 0.001) up-regulated and 11 were down-regulated in workers displaying grooming behaviors in response to the presence of *Varroa* mites relative to the control bees without *Varroa* (Supplementary Table 19). Meanwhile, 48 genes were up-regulated and 60 were down-regulated in the heads of body-shaking bees (Supplementary Table 20).

GO and pathway enrichment analysis suggested that up-regulated genes in grooming bees were enriched in DNA replication and repair, while down-regulated genes were enriched in response to biotic stimulus (GO:0009607) in both grooming and body-shaking bees (Supplementary Table 21). Six genes were significantly down-regulated in both grooming and body-shaking bees, including genes encoding antimicrobial peptides (*apidaecin* and *hymenoptaecin*), one pheromone-binding protein-related protein, one major royal jelly protein, and protein mab-21. Protein mab-21 is a homeotic regulator found in eukaryotes, which appears to be involved in the determination of cell fate. It is cell-autonomously required for specifying the identity of sensory ray 6 in the *Caenorhabditis elegans* male tail, and also for backward locomotion, normal body morphology, fecundity, and embryonic morphogenesis^53^. Of the 27 candidate genes identified influencing *A. mellifera* grooming behavior^54^, *Ataxin-10* and a gene encoding gamma-tubulin complex component were up-regulated in *A. cerana* allogrooming workers. However, none of these candidate genes were found to be up-regulated in body-shaking bees.

Of 513 genes with altered expression in response to *Varroa* infestation, (Supplementary Table 19), the expression of 40 genes involved in signal recognition, modulation, and transduction as well as immune responses were confirmed by qRT-PCR in *Varroa* -challenged *A. cerana* that exhibited grooming and body shaking behavior and in *A. cerana* without exposure to *Varroa*. The qRT-PCR corroborated with the RNA-seq data (Table 4).

**Table 4:** Gene expression in *A. cerana* exposed to *Varroa* or without exposed to *Varroa* by qRT-PCR assay.

**Signaling Pathway**				
**Gene**	**Product**	**W. Varroa**	**W/O Varroa**	
**Recognition**				
ACC_04148	Peptidoglycan-recognition protein SA	1.000*	2.612	(2.517–2.711)
ACC_00291	Peptidoglycan-recognition protein SC2	1.000*	6.163	(6.031–6.297)
ACC_09093	Scavenger receptor class B member	1.000*	1.995	(1.739–2.288)
**Signal modulation**				
ACC_07089 Malectin		1.000*	3.636	(3.530–3.745)
ACC_07866	Acyl-coenzyme A thioesterase	1.000*	5.736	(5.471–6.014)
ACC_05040	Activating transcription factor of chaperone	1.000*	1.835	(1.790–1.882)
ACC_06604	Calmodulin	1.000*	3.839	(3.767–3.912)
ACC_06157	Sodium-dependent dopamine transporter	1.000*	4.065	(3.990–4.141)
ACC_00864	TRAF-interacting protein	1.000*	5.918	(5.688–6.157)
ACC_03016	Serine protease snake	1.873 (1.844–1.904)		1.000*
ACC_01788	Alaserpin	1.225 (1.136–1.322)		1.000*
ACC_04088	Galectin-8	2.288 (1.106–1.127)		1.000*
**Signal transduction**				
ACC_08842	NF-kappa-B inhibitor cactus	1.000*	2.952	(2.824–3.087)
ACC_09588	Mitogen-activated protein kinase kinase dSOR1	1.000*	9.627	(9.298–9.968)
ACC_03970 E3	ubiquitin-protein ligase CBL	1.000*	1.965	(1.918–2.013)
ACC_10164	Dorsal-ventral patterning protein tolloid	1.000*	5.973	(5.861–6.087)
ACC_02175	Myeloid differentiation response protein	1.000*	4.169	(3.540–4.911)
ACC_04061	Serine/threonine-protein kinase mos	1.000*	3.637	(3.591–3.684)
ACC_05279	CAP-Gly domain-containing linker protein	1.000*	6.057	(5.975–6.140)
ACC_05594	Protein FRA10AC1	1.000*	4.563	(4.455–4.674)
ACC_07547	Programmed cell death protein	1.000*	2.233	(2.149–2.321)
ACC_01488	Caspase-1	-1 3.442	(3.215–3.684)	1.000*
ACC_05279	RAC serine/threonine-protein kinase	1.791	(1.700–1.888)	1.000*
**Effectors**				
ACC_01573	Neurexin-1-alpha (Fragment)	1.000*	3.579	(3.247–3.943)
ACC_06112	Ataxin-10	1.000*	4.064	(3.891–4.244)
ACC_09073	Arrestin domain-containing protein	1.000*	3.708	(3.559–3.863)
ACC_01574	Atlastin	1.000*	4.556	(4.074–5.096)
ACC_05357	Hexamerin	2.454 (2.117–2.844)	1.000*	
ACC_08301	Defensin	2.265 (2.136–2.403)	1.000*	
ACC_03623	Phenoloxidase subunit A3	2.421 (2.272–2.580)	1.000*	
ACC_00645	Apidaecins type	1.978 (1.931–2.026)	1.000*	
ACC_03149	Pheromone-binding protein-related protein	1.000*	3.403	(3.287–3.524)
ACC_03450	Hymenoptaecin	2.135 (2.031–2.244)		1.000*
ACC_0563	Protein mab-21	4.120 (3.885–4.370)		1.000*

### Chemoreceptor genes

Odorant binding proteins (OBPs) and chemosensory proteins (CSPs) are two gene families that transport odorant molecules through the sensillum lymph to the olfactory receptors on the membrane of chemosensory neurons. The *A*. *cerana* genome encodes only 14 OBPs (Supplementary Table 22), less than the 21 OBPs of *A. mellifera*. Correspondingly, 81 olfactory receptor (Or) genes were revealed in *A. cerana*, less than half the number of *A. mellifera* (163) but similar to the number of *A. gambiae* (79). Twenty-one of these tandemly arrayed genes were located in a 109 kb region, and showed 28%-79% identity to each other on the protein level, indicating a possible gene duplication. We also determined two CSP genes (*ACC_03947* and *ACC_09381*) and 10 gustatory receptor (Gr) genes in the *A. cerana* genome.

### Learning and Memory

The learning and memory performance of *A. cerana* is significantly better on both color and grating patterns than that of *A. mellifera*^55^. Protein sequences of learning and memory genes in mice*, Drosophila*, and other species were used to search homologous genes in the honeybees. Sixty-five and 50 homologous genes involved in learning and memory were identified in *A. cerana* and *A. mellifera*, respectively (Supplementary Table 23). Moreover, most of those learning and memory genes in higher mammals have a corresponding homolog in *A. cerana* and *A. mellifera*, indicating that the molecular regulation mechanisms of learning and memory are highly conserved from the lower insects to higher mammals^56,57^. The learning and memory process needs to convert extracellular stimuli into intracellular signals. The formation of long-term memory and synaptic plasticity needs to activate many signal pathways of neurons, such as cAMP-PKA, MAPK, and CaMK IV pathways^58^. Those signal transduction pathways converge on the cyclic AMP response element-binding protein (CREB), which is a transcription factor affected by cyclic AMP in the cell (Supplementary Fig. 12).

### Division of labor

Analysis of RNA-seq data revealed that 66% of the *A. cerana* genes (6,722 of 10,182 genes) had significant expression differences (fold change > 2, FDR > 0.001) in the brain of newly emerged workers, nurses, guards, foragers, and dancers (Supplementary Fig. 13, Supplementary Tables 24 and 25). Enrichment analysis revealed that GO terms namely “energy metabolism”, “carbohydrate metabolism” and “amino acid metabolism” were significantly enriched (FDR < 0.05) in up-regulated genes in the brain of foragers and dancers compared to that of younger nest workers (Supplementary Table 26).

**Figure 4:**
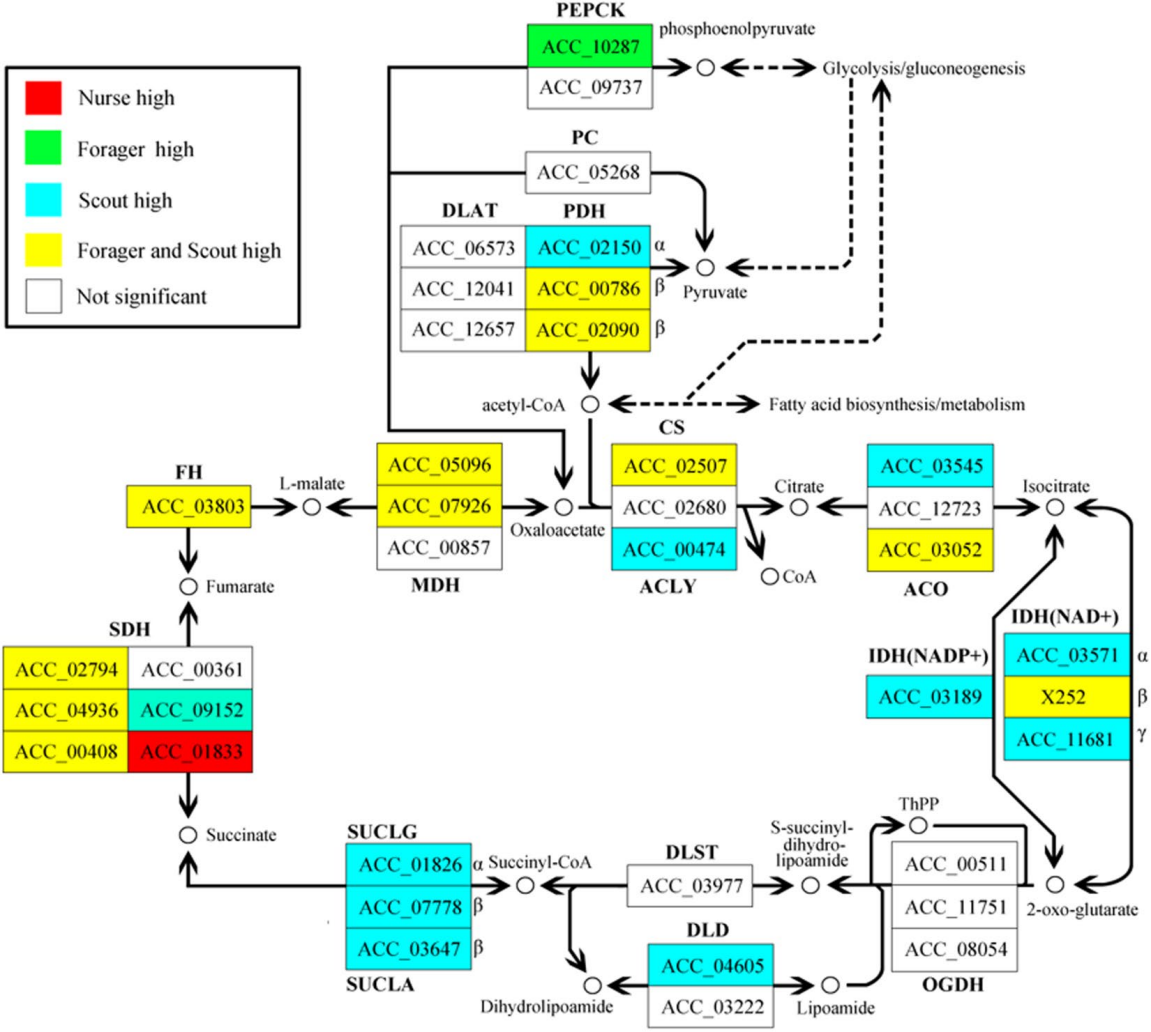
Changes in brain energy metabolism during worker maturation elucidated by pathway analysis of data from brain RNA-seq experiments. Gene expression data from nurse, forager and scout brains were mapped to citrate cycle pathways compiled by the Kyoto Encyclopedia of Genes and Genomes. Genes in the tricarboxylic acid pathway (shown) and other energy metabolism pathways were predominantly upregulated in foragers and scouts brains relative to nurses. Solid lines indicate the predicted enzymatic reactions catalyzed by the product of each gene and dotted lines indicate indirect links to other metabolic pathways. ACLY: ATP citrate (pro-S)-lyase; ACO: aconitate hydratase; CS: citrate synthase; DLAT: Dihydrolipoyllysine-residue acetyltransferase;DLD: dihydrolipoamide dehydrogenase;DLST: dihydrolipoamide succinyltransferase; FH: fumarate hydratase; IDH: isocitrate dehydrogenase; MDH: malate dehydrogenase; OGDH: 2-oxoglutarate dehydrogenase E1 component; PC: pyruvate carboxylase; PDH: Pyruvate carboxylase; PEPCK:phosphoenolpyruvate carboxykinase; SDH: succinate dehydrogenase; SUCLA: succinate–CoA ligase(ADP-forming); SUCLG: succinate–CoA ligase (GDP-forming).

Intriguingly, foraging bees had a higher brain energy metabolism rate than in-hive workers (Fig. 4, Supplementary Information, Supplementary Tables 27 and 28). Of 147 genes belonging to “energy metabolism”, 109 (74%) and 87 (59%) genes were up-regulated in scouts and foragers relative to nurses; whereas 89 (61%) and 58 (39%) of genes were up-regulated relative to guard bees. Pathway analysis of the same data showed that the oxidative phosphorylation pathway, whereby a cell generates most adenosine triphosphate (ATP) during respiration, was significantly enriched (FDR < 0.001) in up-regulated genes in foragers and dancers relative to nurses and guard bees (Supplementary Table 27). The overall brain energy metabolic rate in different phenotypes could be arranged as: dancer > forager > newly-emerged > guard > nurse. Newly-emerged workers had a higher capacity for ATP production than the other in-hive workers, which not only helps to facilitate the bee to chew open the cell cap during emergence but also is required for nerve cell growth.

Pathway analysis of the RNA-seq data revealed that 59% of the insulin/TOR network genes (Supplementary Fig. 14) were up-regulated in the brain during the transition from nurses to foraging workers (18 of the 44 genes were up in foragers, 19 up in dancers, and 11 up in both). Furthermore, the brain expression of the insulin/TOR/PI3K network was positively correlated with main energy metabolism pathways in addition to several transcription and cell cycle related pathways (Supplementary Information). This result indicates that the insulin/TOR signaling played a central role in linking cellular energy status to changes in behavior and in controlling the process of behavioral maturation, perhaps via regulation of cellular energy status and protein synthesis, which consequently affects nerve cell growth. The genome of *A. cerana* contains the same gene set for insulin-related neuropeptides and receptors as in *A. mellifera*, which includes two insulin-like peptides (ILPs), two ILP receptors (InRs), an adipokinetic hormone (AKH), and an AKH receptor (AKHR). The level of mRNA of these genes displayed an age- dependent manner, with foraging bees showing much higher brain expression than nurses (Supplementary Table 28, Supplementary Fig. 14). Brain mRNA levels for ILPs, InRs, and AKH increased ~2.3-fold as nurses turned into guard bees, and then stayed at a higher level thereafter (Supplementary Table 28). The brain expression level of AKH was much higher than its receptor gene, and the mRNA concentration of AKHR was just above detection limits. Because AKH is synthesized and stored in the *corpora cardiaca*, a neurohemal organ adjacent to the brain^59^, we speculated that this neuropeptide hormone might execute biological functions mainly in other tissues after being released into the hemolymph and transported to its target cells. An increase in AKH activity might correlate with loss of internal lipid stores during behavioral maturation from nurses to foraging workers, indicating AKH signaling causally influence lipid mobilization and metabolism (Supplementary Information).

### Comparison with A*cc* genome of Korea

In the preparation of this paper, a Northern strain of *A. cerana* genome with 2,430 scaffolds and 10,631 predicted genes was published by scientists in Korea^60^. The comparison of Northern strain of *A. cerana* genome and South strain of strain of *A. cerana* genome from present study showed that the identity between two strains was 98.8%, with the Korea *A. cerana* genome only 475 kb shorter (Supplementary Table 50). The N50 of the Korea *A. cerana* genome is 1,421,626 bp, while the Chinese *A. cerana* genome is 1,393,515 bp. Though the predicted CDS size of Chinese *A. cerana* (1,696 bp) is 200 bp larger than that of Korea *A. cerana* (1,472 bp), the identity between the genes of two genomes was 99.71%. Meanwhile, we identified 1,618 unique genes (with an average length of 900 bp) in Chinese Southern strain of *A. cerana* genome relative to Korea strain of *A. cerana*, and 2,011 unique genes (with an average length of 438 bp) in the Korea strain of *A. cerana*. For the 2,011 unique genes in the Korea strain, 2,009 had homologous regions in the Chinese *A. cerana* genome, but might be excluded by our gene prediction criteria, since the average size of these genes is only 438 bp and 88.8% (1,786) of them encoded hypothetical proteins. For the 1,618 unique genes in the Chinese strain, 1,564 had homologous regions in the Korea *A. cerana* genome while only 14.9% (241) of them encoded hypothetical proteins. The 54 genes left might be taken as unique Chinese *A. cerana* genes, including odorant binding protein 21 precursor, cytochrome c oxidase subunit, etc. (Supplementary Table 51), suggesting that the Chinese *A. cerana* have evolved unique physiological adaptations in response to different climate and other environmental conditions in China.

## Conclusions

We present a high-quality genome sequence for the Asian honeybee *A. cerana* which constitutes an important resource for further molecular studies of honeybees and other social insects. Our comparative genomic analysis of *A. cerana* and *A. mellifera* deepens our understanding of the relationship between genes, behavior, and genetic adaptations of honeybees and reveals many species-specific genes that are potentially related to specialized biological functions and life history of *A. cerana*.

Using whole-genome sequencing and transcriptome sequencing (RNA-seq) followed by laboratory experiments measuring and comparing behavior response and in *A. cerana* and *A*. *mellifera* induced by *Varroa* infestation, we identified and functionally validated genes underlying the *A. cerana* grooming behavior in cleaning and removing *Varroa* mites. Over the years, the unique biological features of Asian honeybees especially their high degree of resistance to *Varroa* infestation have attracted a great deal of interest from researchers around the world who are committed to understanding the *Varroa* mite and searching for solutions for its control. Previous studies on the mechanisms of honeybee grooming behaviors focused on the induction of the olfactory, visual, and immune responses to the exogenous stimuli from the parasitic mites. Our genome analysis reveals, while *A. cerana* has more immune system genes and encodes many more antimicrobial peptides than *A. mellifera*, *A. cerana* contains less than 34% and 50% of genes encoding odorant binding proteins (OBPs) and olfactory receptors (Ors), respectively, compared to *A. mellifera*. Our observation that *A. cerana* displayed significantly more rapid and vigilant grooming behaviors to the presence of *Varroa* mites than *A. mellifera* suggests that parasite-defensive grooming in *A. cerana* is likely triggered not only by exogenous stimuli through visual and olfactory detection of the parasite but also by genetically endogenous processes, the programmed grooming model that postulates an existence of central programming that periodically activates a bout of grooming to remove ectoparasites before they begin to feed. The difference in levels of grooming behaviors in response to *Varroa* infestation between *A. cerana* and *A. mellifera* may also reflect their genetic difference in DNA methylation-mediated learning and memory. It has been reported previously that the learning and memory performance of *A. cerana* is significantly better on both color and grating patterns than that of *A. mellifera*. The formation of long-term memory and synaptic plasticity needs to activate many signal pathways of neurons, such as cAMP-PKA, MAPK, and CaMK IV pathways. Our transcriptome analysis by RNA-seq following qRT-PCR confirmation showed that the expression level of genes involved in these genetic pathways was significantly upregulated in *A. cerana* when challenged by *Varroa* mites, suggesting that Asian honeybees have evolved species-specific behavior adaptation that helped them survive and thrive in their particular environments. We, therefore, conclude that the Asian honeybee is an excellent model system for studying the cellular and molecular mechanisms of hygienic and grooming behaviors to combat the *Varroa* mites, the greatest pest threat to the European honeybees.

## Materials and Methods

### Bees and Genomic DNA Extraction

A wild colony of *A. cerana cerana* was collected at the Chinese Honeybee Natural Protection Area of the Wufu Mountain, Jiangxi Province, China. It was believed that this place (28°09′, 118°03′) was where Fabricius collected bee samples for his original descriptions of *A. cerana* in 1793. The bee colony was maintained at the Chinese National Human Genome Center at Shanghai. Genomic DNA was extracted from two drone pupae from the colony of *A. cerana* after getting rid of its intestine using the AxyPrep Multisource Genomic DNA Kit (AxyGen, Cat.No. AP-MN- MS-GDNA-50, CA, USA).

### Genome Sequencing and Assembly

A 300 bp paired-end library was constructed using the standard Illumina paired-end protocol, and 99,070,380 pairs of 120 bp reads were produced on the Illumina Genome Analyzer platform (Illumina, San Diego, CA), providing 88 fold coverage of the genome. The shotgun library of 300–800 bp fragments was prepared from 5ug of DNA using the standard GS FLX shotgun library protocol, and a total of 1,641,411 reads with an average length of 388 bp were produced by Roche 454 GS FLX, providing 2.3 fold coverage of the *A. cerana* genome. The 3 kb mate-pair library was constructed combing the GS FLX and Illumina mate-pair protocol, with an adaptor sequence inserted between the mate-pair reads. A total of 46,124,432 mate-pair reads of 120 bp was produced on the Illumina Genome Analyzer platform, providing 32 fold sequencing coverage.

The Illumina pair-end reads and Roche 454 reads were assembled using Velvet V1.2.03^61^, which produced 21,784 contigs with an average length of 9,601 bp. Then mate-pair reads were mapped to the contigs to construct a scaffold of the *A. cerana* genome. Finally, we obtained 879 scaffolds with a total size of 228,791,026 bp, with a maximum length of 4,477,204 bp.

### cDNA sequencing

The honeybee samples were taken from an *A. cerana cerana* colony at the Huajiachi campus of Zhejiang University, Hangzhou, China. The five samples of newly emerging workers, nurse workers, guard workers, dancing workers and foraging workers were collected from the same colony and each sample included thirty individuals. Newly emerging workers were identified as bees emerging from the cells. Nurses were caught when they entered into the cells and were nursing the larvae^62^. Guard workers were identified as bees stinging at the black lint ball which was jumping before the entrance of the hive^63^. Foragers were identified as bees returning to a colony loaded with pollen on their corbicula. Dancers were identified as pollen foragers performing waggle dances on the combs^64^. Each individual was put into liquid nitrogen with a pair of forceps immediately after collection and stored at −80 °C until head dissection.

To prevent mRNA degradation, brains were dissected in a solution of 0.4 M guanidine thiocyanate on an ice bag. The brain tissue was transferred into TRIzol reagent (Invitrogen) immediately and total RNA was prepared according to the manufacturer’s protocols. mRNA [poly(A) RNA] was then purified from total RNA using the Micropoly(A)Purist^TM^ mRNA purification kit (Ambion, Cat.No.1919, Foster, CA, USA).

### Gene Prediction

Four gene prediction sets were independently generated using Augustus^65^, Genemark^66^, Gnomon^67^, and SNAP^68^. Augustus and Genemark predicted using an *A. mellifera* model. Gnomon predicted based on ESTs. For the first step, the longest gene was selected, and if the gene was predicted at the same start or same end locus by two or more software; the second, the gene was selected which had no overlap with other genes; the third, gene was selected if it could be mapped by EST sequences or had homolog with *A. mellifera* genes; the fourth, a gene was abandoned if it had a stop codon in the middle of the sequences. After above steps, we manually checked all of the genes. Finally, a total of 10,182 genes were predicted.

### Bioinformatics analysis

Based on the deduced amino acid sequences, the annotation was performed through BLASTP against the Swiss-Prot database and non-redundant peptide database (NR), with parameters set at E-value 1e-3. Gene ontology analysis was performed using Blast2GO^69^ through BLASTP against the Swiss-Prot database with a parameter of 1e-3. Protein motif and KOG (the assignment was predicted through RPS‐BLAST with the Conserved Domain Database (CDD) with E-value 1e-10^70^. The metabolic pathway was constructed based on the KEGG database by BBH method^71^. Differently expressed genes were identified by the DEGseq package using the MARS (MA-plot-based method with Random Sampling model)^72^ method, with the reads number of each gene transformed into RPKM (Reads Per Kilo bases per Million reads)^73^.

### Candidate species for comparison with *A. cerana*

Seven species were chosen for comparison analysis with *A. cerana*, including *A. mellifera, Camponotus floridanus, Harpegnathos saltator, Bombyx mori, Nasonia vitripennis, Drosophila melanogaster* (release 5) and *Anopheles gambiae*. The set of orthologs were defined by pair-wise comparison of the proteomes using BLASTP with E-value ≤ 1e-50, identity ≥50% and alignment length covering at least 40% of the shorter protein. In addition, proteins matching the same KOG were also taken as orthologs.

### Mite infection experiment

For both *A. cerana* and *A. mellifera*, newly emerged workers were marked on their thorax with non-toxic paint and returned to their original colony consisting of a normal age-structured worker population. Twelve days later, when they were at the age of performing duties within the hive, they were collected and subjected to behavioral tests regarding their behavioral responses to the presence of *Varroa* mites. The cages used to house the bees in the test were plastic containers (diameter = 5.0 cm; height = 5.0 cm), each with a single hole (diameter = 1.0 cm) on the top to allow the introduction of bees and mites. After the introduction, the hole was covered with mesh to prevent bees escaping. Single bees were introduced into each cage. The bees were given ten minutes without any disturbance to calm them down. Using a fine brush, a mite, collected by opening capped brood cells, the mites removed and kept in a petri dish, were transferred onto the abdomen of the bee. Following mite introduction, the bee was observed for 2 minutes. During the observation, two different levels of behavior by individual bees were noticed in response to the presence of the mites. In the first case,, bees groomed themselves using their legs, twisting or pivoting their abdomens while grooming. In the second case, bees immediately moved their bodies rapidly in a side-to-side movement, shaking their bodies repetitively. These two cases were assigned as self-grooming group, and body-shaking group, respectively. As *A. mellifera* did not show significantly triggered behaviors in response to the presence of *Varroa*, only *A. cerana* with behavior responses were used for subsequent molecular analysis. Five bees of each typical behavior of *A. cerana* were sampled. Bees without exposed to *Varroa* mite were used as negative controls. Bees with obscure behaviors were discarded. Five heads of bees in each group were used to extract RNA.

### Caste determination experiment

The newly laid eggs in a comb were used to reveal the mechanism of the caste determination of *A. cerana* by RNA-seq. After two days, 30 larvae of *A. cerana* were moved into artificial queen cells with royal jelly which was collected from the same hive. The remaining larvae were still kept in their own cells. After two days, the larvae were collected into EP tubes and stored immediately in nitrogen for future use. After another two days, the fourth-day larvae were collected from the royal jelly feed cells and the original cells into EP tubes and stored immediately in liquid nitrogen for future RNA extraction.

### Accession Numbers

*A. cerana* genome assembly contigs and scaffolds have been deposited in GenBank under accession number JPYL00000000. Sequences and functional annotations of *A. cerana* protein-encoding genes are available from the NCBI.

## Electronic supplementary material

Supplementary information figures and tables
