# Supplementary material for: Genomic and transcriptomic analysis of the Asian honeybee Apis cerana provides novel insights into honeybee biology
Source: Sci Rep. 2018 Jan 16;8:822. doi: 10.1038/s41598-017-17338-6 (PMC5770391; doi:10.1038/s41598-017-17338-6)
Supplement: Supplementary file 1 — Supplementary information figures and tables [file 41598_2017_17338_MOESM1_ESM.pdf]

Genomic and transcriptomic analysis of the Asian honeybee *Apis cerana* provides novel insights into honeybee biology

Qingyun Diao[1]†, Liangxian Sun[2,3]†, Huajun Zheng[4]†, Zhijiang Zeng[5]†, Shengyue Wang[4]†, Shufa Xu[1]†, Huoqing Zheng[2]† Yanping Chen[6]† , Yuanyuan Shi[5], Yuezhu Wang[4], Fei Meng[2], Qingliang Sang[3], Lianfei Cao[2], Fang Liu[2], Yongqiang Zhu[4], Wenfeng Li[6], Zhiguo Li[7], Congjie Dai[3], Minjun Yang[4], Shenglu Chen[2], Runsheng Chen[8], Shaowu Zhang[9], Jay D. Evans[6], Qiang Huang[6], Jie Liu[6], Fuliang Hu[2]§, Songkun Su[2,7]§, Jie Wu[1]§

†Authors contributed equally to this work.
§Corresponding authors E-mail: apis@vip.sina.com; susongkun@zju.edu.cn; flhu@zju.edu.cn;

[1]Institute of Apicultural Research, Chinese Academy of Agricultural Sciences, Beijing, 10093, China.

[2] College of Animal Sciences, Zhejiang University, Hangzhou, 310058, China.

[3] Molecular Biology and Pharmacology Key Laboratory of Fujian Advanced Education, Quanzhou Normal University, Quanzhou, Fujian, 362000, China.

[4] Shanghai-MOST Key Laboratory of Health and Disease Genomics, Chinese National Human Genome Center at Shanghai, Shanghai, 201203, China.

[5] Honeybee Research Institute, Jiangxi Agricultural University, Nanchang, Jiangxi, 330045, China.

[6] USDA-ARS Beltsville Bee Research Laboratory, Beltsville, Maryland 20705, USA.

[7] College of Bee Science, Fujian Agriculture and Forestry University, Fuzhou, 350002, China

[8]Bioinformatics Laboratory and National Laboratory of Biomacromolecules, Institute of Biophysics, Chinese Academy of Sciences, Beijing, 100101, China

[9]ARC Centre of Excellence in Vision Science, Research School of Biology, College of Medicine, Biology and Environment, The Australian National University, Canberra, ACT 2601, Australia.

1. Supplementary Note

1). Genome sequencing and features

Genome sequencing and assembly. We estimated the genome size based on k-mer frequencies of Illumina reads. A 17-nucleotide depth distribution was used to calculate the size of the genome, suggesting the *A. cerana* genome size of 226 Mb (**Supplementary Fig.1**).

The quality of the sequencing and assembling of the genome was evaluated by comparing with ESTs sequences and *A. mellifera* genome. Transcriptome sequencing was performed for mixed brains of *A. cerana* workers, and produced 469,162 ESTs with average length 390 bp. 98% of the ESTs could be mapped on *A. cerana* genome, suggesting the *A. cerana* genome covered most genes.

The largest scaffold of *A. cerana* reached 5.03Mb, which completely covered group2.18 and group2.19 of *A. mellifera* with a 90.1% identity (**Supplementary Fig.2a**). Meanwhile, we mapped 14 *A. cerana* scaffolds onto chromosome14 of *A. mellifera* with 98.1% coverage (**Supplementary Fig.2b**).

The repetitive sequences. We found only 4.2% of the genome could be defined as repeat region, mainly composed of microsatellites (2.63%) (**Supplementary Fig.4**). A total of 104,983 microsatellites were revealed in the genome, with an average distance of 1,948 bp and 10 repeat units in each microsatellite. Among them, (AT)n and (AG)n occupied 25% and 19%, respectively, coinciding with the high AT content of *A. cerana* genome.

We identified 157 tRNA genes in the genome, representing 20 amino acids. In regards to ribosomal RNA (rRNA), we identified five 18S, eleven 5.8S, and two 28S rRNA genes, respectively.

Gene prediction and annotation. Combining a gene prediction program based on EST alignments (Gnomon) and three *ab initio* prediction programs (GeneMark.hmm,

Augustus and Snap) based on honey bee model, we identified 10,182 protein encoding genes in the *A. cerana* genome, with an average gene density of 53 genes per Mb. Chromosome 15 and Chromosome 13 separately exhibited the highest gene density (59 genes/Mb) and lowest gene density (34 genes/Mb) in *A. cerana*. The average gene size was 7,577 bp, with 7 exons per gene and the largest gene span 390,254 bp in the genome. The average CDS size was 1,695 bp, and the coding regions cover 17.26 Mb, accounting for 7.5% of the genome. The GC content in exons, introns and intergenic regions of *A. cerana* genome was 37.09%, 32.55% and 33.11% respectively.

We revealed 809 extracellular protein and 2,231 transmembrane proteins. 538 paralog families, composed of 2,484 genes, were reveald in *A. cerana* genome.

CpG and DNA methylation. The CpG dinucleotides distribution in *A. cerana* genes showed a surprising di-peak pattern (**Supplementary Fig.6**), with 2,577 genes under-represented (0.53-fold) and 2,297 genes over-represented (1.15) compared to the expectation from mononucleotide frequencies. The observed CpG/expected CpG ratio in genes was one-peak in most insects, with most of observed CpG/expected CpG ratio less than 1 (0.44 in human). It is known that methylated cytosines mutate to thymines at a high rate, which caused the CpG deficiency in mammalian genomes. So the higher CpG content in honey bee might hint that methylation rarely occurred, while the lower CpG content indicated a higher methylation ratio. So we divide the *A. cerana* genes into two groups, and studied the detailed role of these genes affect by methylation.

We found three DNA (cytosine-5-)-methyltransferase genes (K00558) in the genome, including previously reported DNA methyltransferase3 (Dnmt3), an enzyme involved in de novo methylation. The mRNA expression of *Dnmt3* was detected in different developmental stages of worker and queen, and it was expressed significantly higher in 30-day-old worker than in 1- and 7-day-old worker. The expression level of *Dnmt3* was higher in queen pupae than in worker pupae. After feeding with homologous royal jelly, the whole DNA methylation of *dynactin p62* in 6-day-old *A.c.cerana* larvae was significantly lower than 3-day-old *A.c.cerana* larvae.

Gene Expression. To study the gene role of *A. cerana* in different stages, we performed RNAseq for 16 different *A. cerana* samples (**Table S5**). Brains of workers of five different stages (nurse, forager, guard, dancing and newly-emerged workers) were sequenced to study behavior divergence. Antenna and brain of *A. cerana* and *A. mellifera* under Varroa destructor infection were sequenced to find the different mechanism of Varroa destructor resistance of *A. cerana*. Comparing the gene expression level in each stage, we found over 5,000 genes were significantly up- or down-regulated (FDR<0.001, Fold Change >2) in at least one stage compared with the other stages.

Comparison with Other Insects. Comparing social insects' with non-social insects' genome, only one KO related with signal pathway is missed in social insects' genome. A total of 4,844 genes of *A. cerana* are confirmed as single copy, with less than 30% coverage and 20% identity with other genes in the whole proteome. To better understand the evolutionary position *A. cerana*, 155 single copy genes with best hit to other 10 species were choose to create a phylogenetic tree (**Figure. 2, Table S9**). It is clear that *A. cerana* and *A. mellifera* located together, and showed close relationship with the other three species of Apoidae. The mean sequence identity between *A. cerana* and *A. mellifera* homologous genes are 96.6%, with intron regions showing 92.5% identity.

Domain comparison and KOG analysis showed that there is no significant statistical difference between *A. cerana* and *A. mellifera* or *A. florea*.

Telomerase Complex. Telomeres are capping structures that comprise the physical ends of eukaryotic chromosomes and composed of randomly repeated arrays of $(TTAGG)_n$ in insects. Telomerase is a ribonucleoprotein enzyme complex, composed of telomerase reverse transcriptase (TERT), telomerase RNA (TERC) and dyskerin (DKC1), which adds DNA sequence repeats to the 3' end of DNA strands in the telomere regions. TERT has been already confirmed in *A. mellifera*, and its activity

might contribute to ageing differences of worker, drone and queen honeybees. We identify two genes (*ACC_06995* and *ACC_07320*) encoding TERT and one gene (*ACC_03717*) encoding DKC1 in *A. cerana*. But no shelterin complex component was revealed in *A. cerana* genome, which is crucial for both the maintenance of telomere structure and its signaling functions. The trafficking of telomerase to both Cajal bodies and telomeres depends on telomerase Cajal-body protein (ACC_01627), and telomerase-binding protein (ACC_04595 and ACC_04596) is critical for telomerase-telomere associations.

2) **Biological features**

Protease and Inhibitors. Besides participating in intracellular protein digestion and many biological processes, proteolytic activities of honeybee in the hemolymph, gut and body surface played important role in degradation of pathogenic bacteria, fungi and parasitic mites. Using the MEROPS batch BLAST server (E-value<1e-10), we detected 353 peptidases belonging to 85 protease gene families in *A. cerana* genome (**Table S29**). Among them Serine Peptidases and Metallo Peptidases each occupied one third of total peptidases, with 59 being secreted and 52 belonging to S1 family. Serine proteases in the S1 family are involved in digestion, development, and defense response[1].

We revealed 30 peptidase inhibitors in *A. cerana* genome, including 21 serine peptidase (SP) inhibitors and 6 cysteine peptidase inhibitors (**Table S30**). SP inhibitors are present in insect haemolymph to remove excess proteases and maintain homeostasis. There are 11 secreted protease inhibitors in *A. cerana*, and they might participate in cuticle-associated immunity by interacting with secreted protease. Expression profile revealed two pair of interacted proteases-inhibitors (*ACC_08021*/*ACC_09990*, *ACC_01201*/*ACC_08200*), which were all transmembrane proteins and showed similar expression pattern (**Supplementary Fig. 15**).

Heat shock proteins. Four kinds of heat shock proteins (HSPs) are detected in *A. cerana*, including HSP20, HSP40, HSP70 and HSP90. The heat shock 70kDa proteins

(HSP70s), one of the most ubiquitous classes of chaperones, fulfill different biological functions and impact on a range of different intracellular pathways. Often, single HSP70 is driven by multiple HSP40s. As expected, almost five times HSP40 genes (29, IPR001305 and IPR001623, **Table S31**) than HSP70 genes (6, IPR013126), are confirmed in *A. cerana*. Only one HSP70 (coding by *ACC_0453*4) has classical cytosolic HSP70 features (a conserved EEVD motif for substrate binding and a GGMP repeat unit), while the other five can be taken as non-canonical HSP70. Both canonical (*ACC_04534*) and non-canonical HSP70 genes show constitutive expression in different life-cycle stages, with the only exception that *ACC_04534* shows significant down-regulation in nurse bees (p-value=0). Phylogenetic analysis reveals that HSP70s of *A. cerana* is closer to *A. mellifera* and *A.florea* than other species including *N. vitripennis* (**Supplementary Fig. 16**, **Table S32**).

HSP90 showed an alternative splicing-dependent regulation between caste- and age-specific expression. A total of four HSP90 (IPR013126) genes are detected in *A. cerana*, and two (*ACC_01484* and *ACC_04157*) of them encode conserved EEVD motif. It is interesting that the expression of two HSP90s genes with EEVD motif also showed significant down-regulation in nurse stage (p<1e-100).

HSP20 plays an important role in regulation of muscle contraction, apoptosis and metabolism, and in protection against oxidative stress. Ten HSP20 genes (IPR002068) are confirmed in *A. cerana*. Two of HSP20 genes (*ACC_01376* and *ACC_01377*) show significant up-regulation in forage stage, while another three genes (*ACC_00695*, *ACC_01374* and *ACC_04703*) show significant down-regulation in newly-emerged workers.

In structure, we found HSP 67B2 had duplicate genes (*ACC_00316*/*ACC_07746*), and five HSP20 genes (*ACC_01373-ACC_01377*) tandem located in a 15 kb region. The expression profile showed that the five genes had a similar expression tendency in 11 different stages (**Supplementary Fig. 17**)

Metabolic and signaling pathways. The genome of *A. cerana* encodes components of several intact metabolic pathways, including glycolysis/gluconeogenesis, TCA

cycle, fatty acid metabolism, purine and pyrimidine metabolism, but lacks synthesis ability of 8 amino acids. In addition, *A. cerana* has 20 nuclear receptors (**Table S33**) and complete gene sets involved in circadian rhythm, sensory system, nervous system and development (**Table S34-36, S22**).

A total of 391 *A. cerana* genes are involved in 19 signaling pathways (**Table S37**). Basically, the number and kinds of genes involved in signaling pathway of *A. cerana* is similar to *A. mellifera*.

The cAMP-dependent kinase (PKA) is composed by regulatory (R) and catalytic (C) subunits, and plays a crucial part in cellular processes and in long-term memory formation in the honeybee. In the *A. cerana*, two catalytic subunits were encoded by two genes (*ACC_04702* and *ACC_08577*) and two genes (*ACC_00806* and *ACC_02101*) encoded the regulatory subunits of PKA, losing one gene encoding catalytic subunit (PKA-C2) compared with *A. mellifera*. PKA-C3 (also known as DC2) encoded by *ACC_04702* in the *A. cerana*, but the role of PKA-C3 is not known. We speculated that PKA-C3 might be functional at worker development stage, because PKA-C3 showed significant highly expression ($p<0.001$) in worker's chrysalis. Like *A. mellifera*, only one gene (*ACC_03168*) encoded CREB (cAMP response element binding protein).

A diversity of signals accelerates glycogen degradation that is mediated by hosphorylase b kinase (Phk), which has one catalytic (gamma) and three regulatory (alpha, beta and delta) subunit. PKA phosphorylates alpha and beta subunits to relieve inhibition of gamma subunit and activate the enzyme. In the *A. cerana*, alpha/beta subunit (PHKA_B, *ACC_01233*) and gamma subunit (PHKG, *ACC_02557*) were identified and were both highly expressed ($p<0.001$) in worker's chrysalis. So in consideration with PKA also highly expressed in worker's chrysalis, we speculated that development of workers need lots of glycogen as sources.

Hippo signaling pathway is a conserved pathway for growth control. The orthologous lingand/receptor (Eiger/Wengen) axis is first isolated and characterized in *Drosophila*, and forced expression of Eiger or Wengen can active JNK and cause apoptotic cell death. JNK activation makes a crucial contribution to the activiation of

Yorkie (a transcriptional coactivator protein in *Drosophila*) whose activation might contribute to wing disc regeneration. In *A. cerana*, Eiger (*ACC_05309*) was highly expressed ($p<1e-20$) on forage stage and dancing stage，and Wengen (*ACC_07309*) was highly ($p<1e-15$) on forage stage, compared with new-born，nurse and guard. We hypothesized that wing damage might be more often occurred on the bees working far from hives.

Sex determination. Sex determination in *A. mellifera* is governed by heterozygosity at a single locus (the Sex Determination Locus, SDL), which is always heterozygous in females and homo- or hemizygous in males. This SDL harbors two sexual regulating genes, the complementary sex determiner (*csd*) gene and feminizer (*fem*) gene, which were demonstrated by RNAi induced knockdown experiments. In *A. mellifera fem* is downstream of *csd*, which directs its alternative splicing into a non-functional variant in males. The same as *A. mellifera*, only one *fem* and one *csd* were found in *A. cerana*. Gene *doublesex* and *transformer* were also detected in *A. cerana*, which gave a hint that honeybee might have auxiliary sex determination mechanism.

Sex fate of *Drosophila* depends on *Sex-lethal* (*Sxl*) expression which acts as a genetic switch. *Sxl* is on in female and remains off in male by controlling its own alternative splicing. The *Sxl* (*ACC_06351*) and downstream genes are all detected in *A. cerana*, including *transformer* (*tra*, *ACC_06796*), *doublesex* (*dsx*, *ACC_05947*) and *fruitless* (*fru*, *ACC_04409*, *ACC_05182*, *ACC_06237* and *ACC_08734*). Since the ROX sequence is missed and could be compensated by over expression of MSL1 and MSL2, so we speculate that DCC system might be functional at female stages, because MSL2 is a crucial component of *Drosophila* dosage compensation system. Because both DCC and sex determination locus (SDL) are presence in *A. cerana* genome, it is speculative hypothesis that two systems are functional at difference stages to control the sex determination.

Aging. Histone acetyltransferases (HATs) and deacetylases (HDACs) add and remove acetyl groups on histone tails, regulate genes through chromatin structure, and are linked to the aging process. We identified six HATs (**Table S38**) in *A. cerana*. Humans have four classes of HDACs, comprising HDAC1-11 and the NAD^+-dependent sirtuin family proteins (SIRT1–7). In contrast, only seven HDACs and seven sirtuins (SIRT1–2, 4-7) are found in *A. cerana*, similar gene numbers in *A. mellifera*[2].

Insulin signaling/secretion. In *A. cerana*, 63 genes involving insulin signaling pathway have been identified (**Table S37**), whereas one gene, encoding receptor-type tyrosine-protein phosphatase F, is identified as unique for *A. cerana* compared with other seven reference genomes. A total of 28 genes participate in insulin secretion; RYR2 and KCNNN is both ion channel and confirmed as unique genes for *A. cerana*. Adenylate cyclase 5 (ADCY5) is unique for social organisms, but its function can be substituted by other genes in non-social organisms, such as ADCY2, ADCY8, ADCY9.

Circadian rhythms. The clock proteins of *A. cerana* have cryptochrome (Cry), timeless (Tim), clock (Clk) and cycle (Cyc), the kinds of protein is the same as *A. mellifera*. But *A. cerana* has no orthologs to gene encoding timeout (Tim2) **(Table S34)**.

Similar to the genome of *A. mellifera* which encodes ten potassium voltage-gated channels, *A. cerana* genome encodes nine (**Table S39**). A total of 68 and 96 kinds of enzymes involved in carbohydrate (**Table S40**) and lipid metabolism (**Table S41**). About 23 of 68 enzymes performed glycoside hydrolase activity, and 35 kinds of enzymes performed glycosyltransferase activity to format of glycosidic bonds, and 7 kinds of enzymes performed carbohydrate esterase, and three kinds of enzymes (2.4.1.-, 3.1.1.- and 3.5.1.-) performed muti-functional activity.

Learning and memory. Our analysis showed that all genes involved in cAMP-PKA pathway of mammal could be found in *A. cerana*. The neurotrophin family of MAPK pathway in *A. cerana* had no homology with known genes in mammal, such as brain-derived neurotrophic factor (BNDF), nerve growth factor (NGF), beta-nerve growth factor (beta-NGF), ciliary neurotrophic factor gene (CNTF), glial cell line-derived neurotrophic factor (GDNF), neurotrophin-3 (NT-3), neurotrophin-4/5 (NT-4/5), neurotrophin-6 (NT-6). These genes in *Drosophila* also had no homology. This result suggested that neurotrophin family could not play an important role in learning and memory of the insects. However, the other genes in MAPK pathway in *A. cerana* had homology with known genes in mammal. Except for the neurotrophin family, CaMK IV gene of CaMK IV pathway in *A. cerana* had no homology with known genes in mammal. While the upstream and downstream genes of CaMK IV in *A. cerana* had homology with known genes in mammal. Our analysis illustrated that cAMP-PKA, MAPK and CaMK IV pathway may play an important role in learning and memory of *A. cerana*.

Cold resistance of *A. cerana*. It's known that the insects survive low temperature through freeze avoidance by supercooling or freeze tolerance36. Freeze avoidance insects will remove or mask ice-nucleating agents (INAs) to keep body fluid supercool (a condition that solution remains unfrozen below its freezing point), while freeze tolerance insects will produce hemolymph protein ice nucleators (PINs) which induce freezing at high subzero temperatures thereby inhibiting lethal intracellular freezing. Six genes encoding homology of PINs were revealed in *A. cerana*, with two of them were extracellular proteins (**Table S42**). Meanwhile, antifreeze proteins (AFPs) function as cryoprotectants to prevent freeze damage in freeze tolerance and inhibit INAs in freeze avoidance. Though no antifreeze domains (PF 05264 and PF 08666) were revealed in *A. cerana* genome, we identified four genes encoding Giardia variant-specific surface protein (PF 03302) which usually existed in AFPs, and another seven genes encoding homology of AFPs (**Table S42**).

Meanwhile, it's known that the capacity to supercool decreases as body mass increases, so the cold-tolerance capacity of *A. cerana* might be partly explained by its small size than *A. mellifera.*

3) Caste determination and Labor division

Caste determination. Previous findings have led to the conclusion that the influence of food intake on Juvenile hormone (JH) is the key determinant of female caste in honey bees. JH signaling locates at downstream of the insulin/TOR nutrient-signaling pathways in honey bee. JH changes in energy metabolism and increasing brain levels of octopamine (OA) are led by JH. In this study, we discovered that two genes of insect hormone biosynthesis pathway expressed significantly higher in the queen fated larvae (**Table S43**), while JH acid methyl transferase (encoded by *ACC_08841*) was only significantly up-regulated in 2-days queen fated larvae.

Yellow protein and MRJP family are multifunctional proteins with diverse, context-dependent physiological and developmental roles. The MRJP family is required for all major aspects of eusocial behavior in *A. mellifera*, and nutritional MRJP component of RJ is essential for behaviors of the honey bee. The MRJP family in *A. cerana* is encoded by ten genes (MRJP1-6, 8, **Table S44**). Gene *yellow-e3* was proved as an origin of the MRJP subfamily. Yellow genes occur in insects as well as some bacteria and fungi, but they are curiously absent in all noninsect metazoans. Five yellow genes are detected in *A. cerana*, and most of them were expressed in all the 16 stages except *ACC_10025*.

Dynamics of brain transcriptome during behavioral development of worker. The social behavior of honeybees is dramatically featured by the age-related division of labor workers. In general, young workers tend to perform in-nest tasks whereas older individuals forage outside the hive for nectar and pollen. When newly emerged workers progressing through larvae caring and then hive entrance guarding tasks, only relatively small number of genes were up-regulated (78 genes in nurses vs. newly emerged, and 244 genes in guarders vs. nurses, **Table S25**), suggesting that the

behavioral transitions within the hive are not absolute. In contrast, there are 2,844 and 3,783 genes (28% and 37% of the 10172 genes) up-regulated in the brains of foragers and dancers relative to larvae-feeding nurses, indicating the shift from hive activities to foraging is more dramatic. Intriguingly, 13% of the *A. cerana* genes (1363 genes) showed significant difference in brain mRNA abundance between dancers and foragers, the two behavioral phenotypes of a colony's foraging force. These findings are similar to microarray results of *A. mellifera*, which showed that 39% of the analyzed genes were differentially expressed between nurses and foragers, whereas 16% of the genes differed in expression between dancers and non-dancers.

Of the brain differentially expressed genes, 3,308 and 2,237 genes were assigned to GO terms and KEGG pathways, respectively. Enrichment analysis revealed that the up-regulated genes during behavioral development were over-represented in GO terms and molecular pathways related to a number of fundamental biological processes such as metabolism, system development and gene expression (**Table S26-27**). Terms namely "energy metabolism", "carbohydrate metabolism" and "amino acid metabolism" were significantly enriched (FDR < 0.05) in up-regulated genes in brain of foragers and dancers compared to younger nest workers (**Table S26**).

Foraging bees are higher in brain energy metabolism rate than in-hive worker honeybees. Of 147 genes belonging to "energy metabolism", 109(74%) and 87 (59%) genes were up-regulated in dancers and foragers relative to nurses; whereas 89(61%) and 58(39%) gene were up-regulated relative to guarding bees. Pathway analysis of the same data showed that the oxidative phosphorylation pathway, whereby a cell generates most adenosine triphosphate (ATP) during respiration, was significantly enriched (RST < 0.001) in up-regulated genes in foragers and dancers relative to nurses and guarding bees (**Table S27**).

Majority of the 88 oxidative phosphorylation genes were upregulated in the brain of foraging bees relative to nest workers (70% in forager vs. nurse, 81% in dancer vs. nurse, and 63% in dancer vs. guard). More detailed analysis revealed that the

abundance of brain transcripts for ATP synthase was higher in foraging workers than in nest bees (**Table S28**). In addition, genes encoding ADP/ATP carrier protein and phosphate carrier protein, two mitochondrial transport proteins that play an important role in control of oxidative phosphorylation, also exhibited higher transcription level in brains of foraging bees relative to nursing and guarding bees. Furthermore, genes encoding rate limiting enzymes in essential energy metabolism pathways, including the tricarboxylic acid (TCA) cycle, glycolysis, and fatty acid metabolism, showed higher transcripts abundance in brain of dancers and foragers than in nurses and guarding bees (**Table S28**). Although the energy metabolism pathways were not significant enriched in the up-regulated genes in foraging bees, 22 of the 34 (65%) TCA cycle genes (**Figure. 4**), and 15 of the 24 (65%) fatty acid metabolism genes , were up-regulated in the brain of dancers relative to nurses (**Table S27**). All these results suggested that foraging workers were higher in whole-brain energy metabolism rate than nurses and guarding bees. Foraging workers perform complex behaviors such as communicating each other for sites and quality of food resource with dance language, searching for food and at the same time evading flying predators in complex external environments. Carrying out these activities seems requiring more intensive brain activities than performing in-hive works. Consequently the brain of foraging workers needs to produce a higher amount of ATP to sustain the energy demand than in nest workers. The expression pattern of key energy metabolism genes (**Table S28**), the overall brain energy metabolic rate in different phenotypes could be arranged as: dancer > forager≈newly-emerged > guard > nurse. Newly-emerged workers had a higher capacity for ATP production than the other in-hive workers, which not only facilitate the bee to break the cell cap during emergence, but also is required for nerve cell growth.

Enhanced energy metabolism in brain of workers during behavioral development correlates with up-regulation of insulin/TOR signaling. Insulin/TOR signaling senses the energy status of a cell through AMPK, which is activated in

response to high AMP/ATP ratio, and activated AMPK stimulates ATP-generating processes. The brain expression of AMP-activated protein kinase (AMPK) genes was enhanced during transition from in-hive workers to foraging bees (**Table S28**), which parallelized the expression of energy metabolism genes. Another node of insulin/TOR network, the *Akt* gene Akt, was higher expressed in foragers and dancers than in nurses and guarders (**Table S28**). In mammals, Akt maintains a high ATP level by increasing mTOR-dependent nutrient uptake. Therefore, foraging workers might maintain a high energy level in brain Akt-mediated food intake. In fact, workers eat honey before leaving the nest for a foraging trip.

Brain expression of insulin/TOR/ PI3K pathway correlated with transcription and translation pathways during behavioral development. Of the 161 genes belonging to "transcription" class, 83(52%) and 63 (39%) genes were up-regulated in dancers and foragers relative to nurses. Similarly, 47% and 41% of the 318 genes belonging to "translation" class were up-regulated in dancers and guarders relative to nurses (**Table S26**). More detailed analysis revealed that 69% of the ribosome biogenesis pathway genes (44 out of 64 genes) were up-regulated in dancers relative to nurses, and 61% of the ribosome pathway genes (46 out of 76 genes) were up-regulated in foragers relative to guarders (**Table S27**). These results suggested that the transition from nursing workers to forager outside the hive involved a higher rate of protein synthesis. Intriguingly, the brain expression of insulin/TOR/PI3K signaling was found positively correlated with several transcription and cell cycle related pathways ($R_{\text{PI3K-Akt/ Basal transcription factors}}$=0.97, $R_{\text{PI3K-Akt/ Spliceosome}}$=0.94, $R_{\text{PI3K-Akt/ RNA transport}}$=0.96, $R_{\text{Insulin/ mRNA surveillance}}$ =0.99, $R_{\text{mTOR-PI3K/ mRNA surveillance}}$ =0.99, $R_{\text{Insulin/ Cell cycle}}$ =0.96, $R_{\text{mTOR-PI3K/Cell cycle}}$=0.98). The correlation between insulin/TOR/ PI3K-Akt signaling and energy metabolism, gene expression and cell growth related pathways suggested that insulin/TOR signaling may play a central role in controlling the process of behavioral maturation, perhaps via regulation of cellular energy status and protein synthesis, which consequently affects nerve cell growth.

Lipid loss during behavioral maturation is attributable to elevated *Akh* expression. The genome of *A. cerana* contains the same gene set for insulin-related neuropeptides and receptors as in *A. mellifera*, which includes two insulin-like peptides (ILPs), two ILP receptors (InRs), an adipokinetic hormone (AKH), and an AKH receptor (AKHR). The level of mRNA of these genes displayed an age-dependent manner, with foraging bees showing much higher brain expression than nurses (**Table S37**, **Supplementary Fig. 14**). Brain mRNA level for ILPs, InRs and AKH increased ≈2.3-fold as nurses turn into guarding bees, and then stayed at higher level thereafter (**Table S28**). However, the mRNA abundance of insulin-related neuropeptide genes and their receptor genes was unproportionate. The brain mRNA level of *InR* was 5-10 folds higher than *Ilp*, indicating InR could have other ligands besides binding ILPs. On the contrary, the brain expression level of *Akh* was much higher than its receptor gene, and the mRNA concentration of *Akh*R was just above detection limits. Because AKH is synthesized and stored in *corpora cardiaca*, a brain adjacent neurohemal organ, we speculated that this neuropeptide hormone might execute biological functions mainly in other tissues after being released into hemolymph and transported to its target cells.

The expression pattern of AKH and ILP genes was closely coupled with dynamics of internal nutrient stores during the trajectory of behavioral maturation. As described above, brain expression of AKH and ILP genes was up-regulated in guarders and foraging workers relative to nurses. On the other hand, the major storage proteins vitellogenin (Vg) and hexamerin genes were highly expressed in nurses, then dropped significantly in guarders and foraging workers. We also found that the acetyl-CoA carboxylase gene, which encodes the rate limiting enzyme in fatty acid biosynthesis pathway, was more highly expressed in newly-emerged workers and nurses, then decreased to a relatively low level in older workers. These results were in accordance with previous findings in *A. mellifera*, which showed that nurses lose most of their lipid and Vg reserves prior to the onset of foraging and subsequently remain lean for the remaining time of life. The elevated insulin and AKH signaling might have causal effect on internal nutrient stores loss during behavioral maturation. Firstly, studies in

other insect species suggest that ILPs can have catabolic functions. Secondly, AKH is the insect equivalent of glucagon, which can mobilize lipid stores through activating the catabolic enzymes triacylglycerol lipase. As expected, the brain mRNA level of *triacylglycerol lipase* was low in newly-emerged workers and nurses, then enhanced about 3 folds in guarders, and reached the highest level in foragers and dancers (**Table S28**). Thus, increase of AKH activity might correlate with loss of internal lipid stores during behavioral maturation from nurses to foraging workers, indicating AKH signaling causally influence lipid mobilization and metabolism.

Genes for enzymes in the last three steps of JH biosynthesis pathway were more highly expressed in foraging bees relative to nest bees. JH, a multifunctional lipophilic hormone synthesized by the neurohemal organ corpora allata, medicates many fundamental processes in the division of labor in worker bees. It paces behavioral maturation, underlies age-related changes in energy metabolism, and increases brain levels of octopamine (OA). Analysis of RNA-seq data showed that the expression level of genes encoding enzymes in the last three steps of JH biosynthesis pathway (JH esterase-like, JH epoxide hydrolase and JH acid methyltransferase) was higher in foraging workers relative to in-hive workers (**Table S28**), indicating the rate of JH biosynthesis is enhanced during transition from nest workers to foraging workers. Because the JH signaling locates at downstream of the insulin/TOR nutrient-signaling pathways in honey bee, it is clear that the regulation of behavioral maturation involved the coordination of hormonal signaling pathways.

2. Supplementary Figures

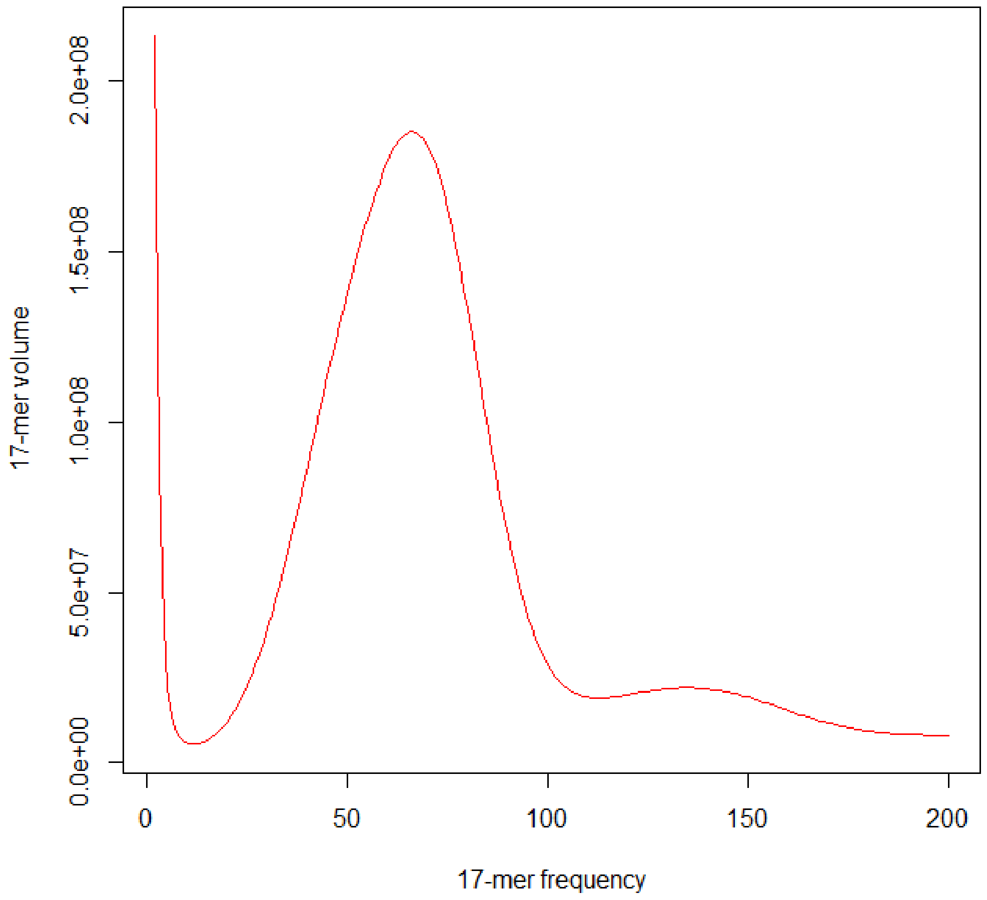

Supplementary Figure 1. 17-mer volume histogram of Illumina reads. The volume of 17-mer is plotted against the frequency at which they occur. The total error-free 17-mer number is 14,969,935,296 and the peak is 66. The genome size ca be estimated as (total K-mer number) / (the volume peak).

a.

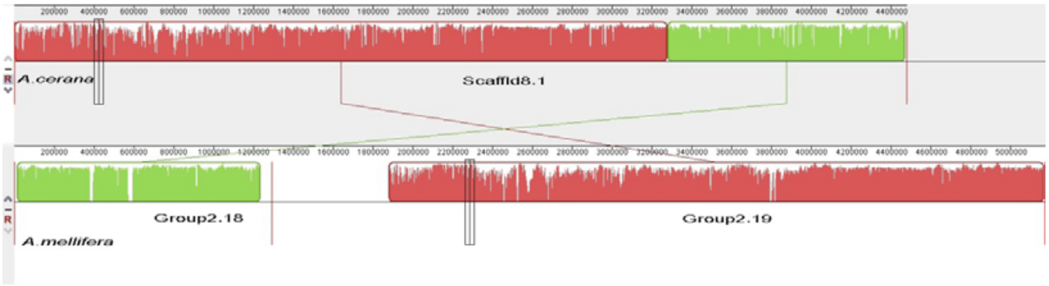

b.

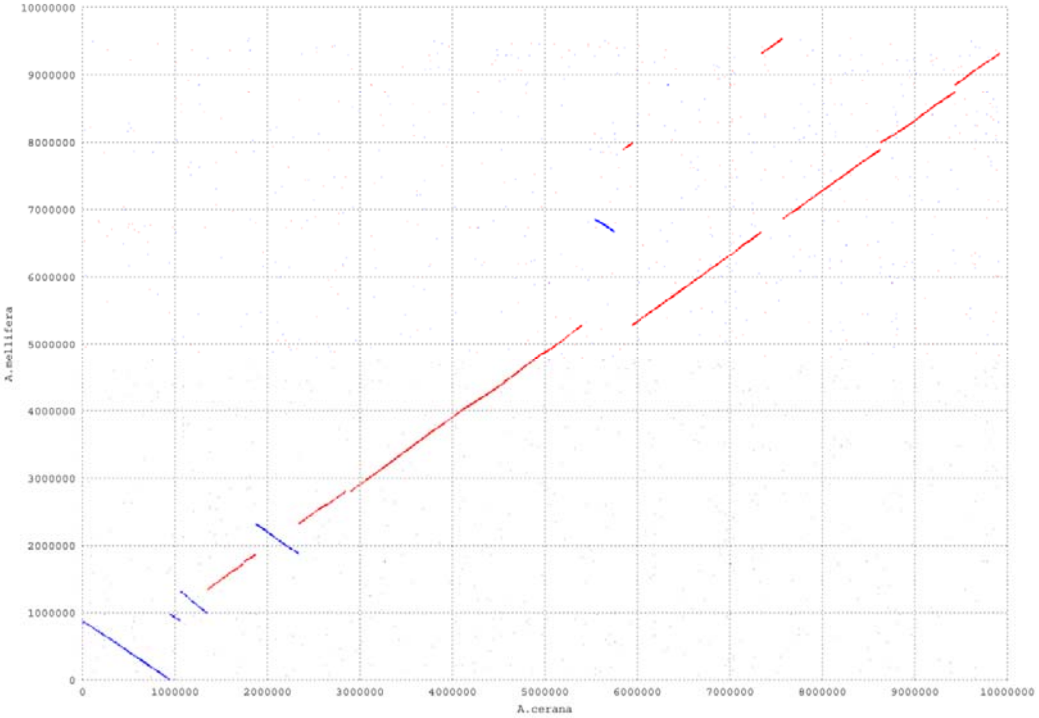

Supplementary Figure 2. Alignment of *A. cerana* scaffolds with *A. mellifera* genomes. a. alignment of *A. cerana* scaffold8.1 with two scaffolds of *A. mellifera* genome V4.5 using progressiveMauve, with weight 15 mers for initial seeds. b. the syntenic relationship between 14 A.cerana scaffolds and the chromosome 14 of *A. mellifera* drawn with M Mmer.

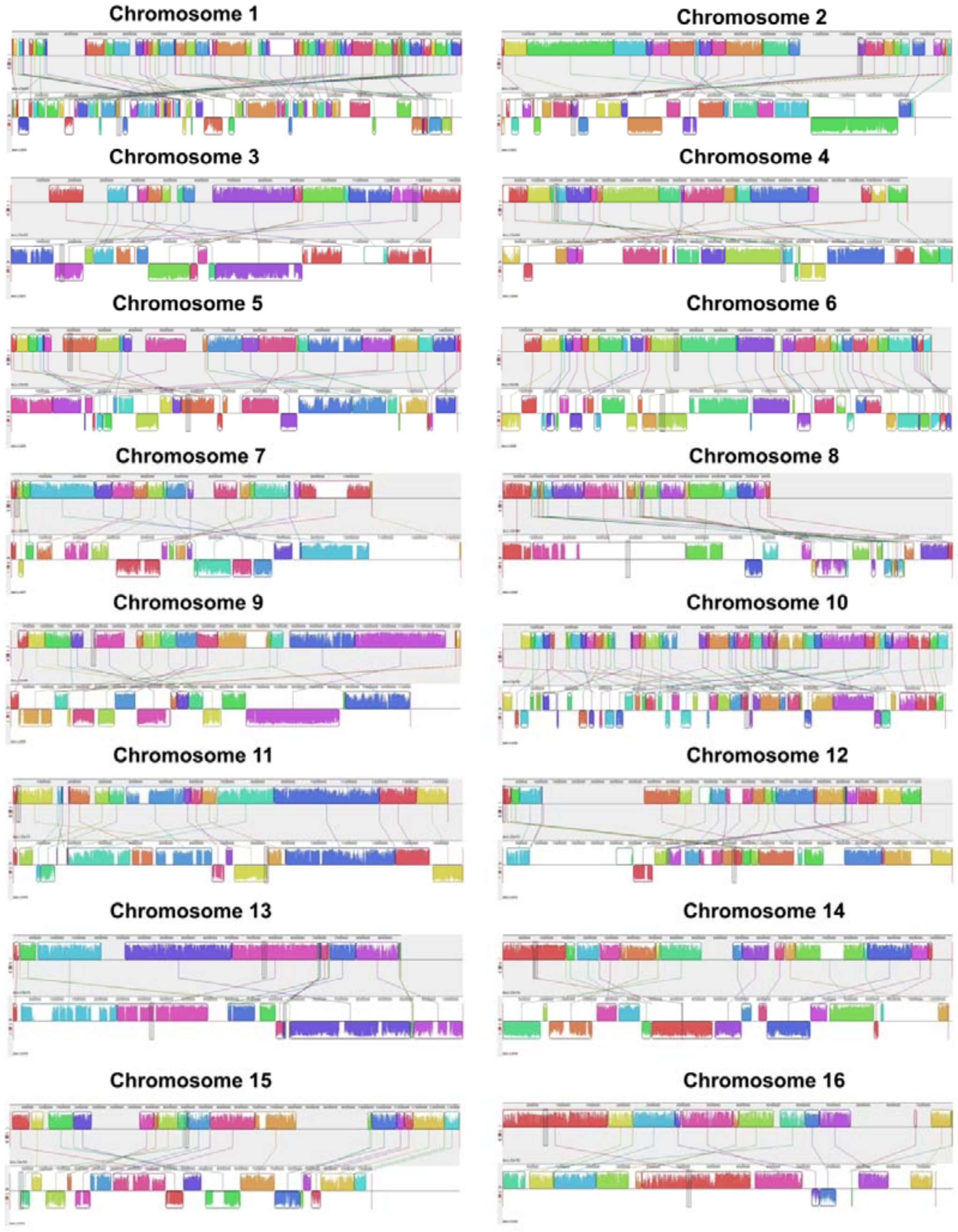

Supplementary Figure 3. Synteny between the *A.cerana* scaffolds and *A.mellifera* chromosomes. The white blocks in each chromosome represent the unique region in *A.cerana* or *A.mellifera*.

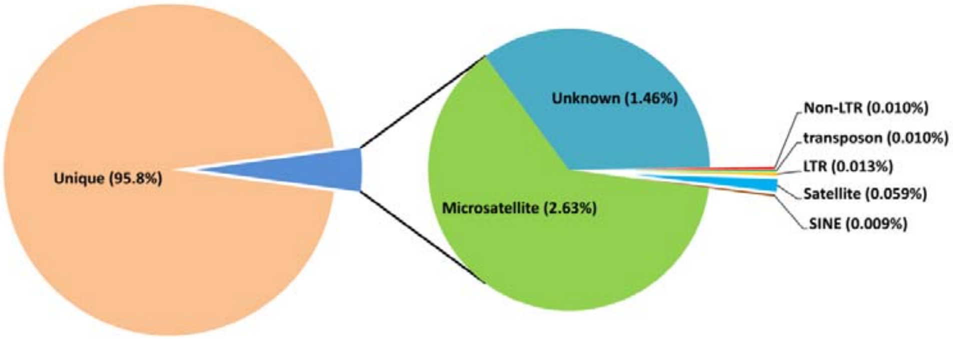

Supplementary Figure 4. Repeats classification in *A.cerana*. The repeat units were defined by RepeatScout, and classification was performed based on RepBase and Censor. The Microsatellite was defined using SciRoKo.

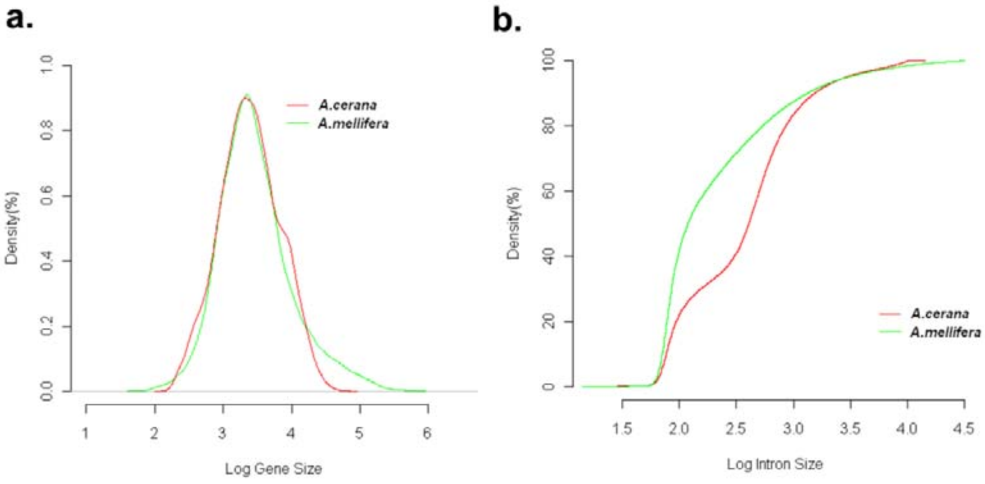

Supplementary Figure 5. Size distribution of CDS (a) and introns (b) in *A.cerana*.

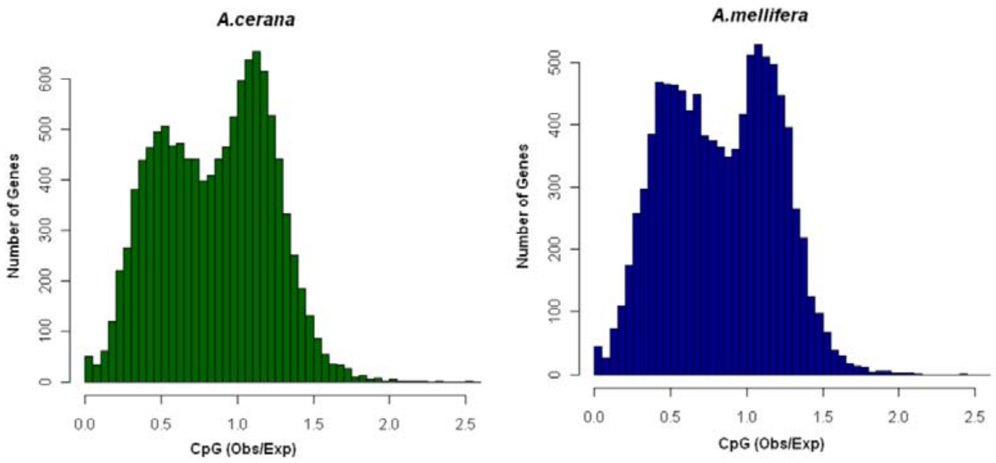

Supplementary Figure 6. Distribution of CpG dinucleotides in exons. The x-axis represents the ratio of observed CpG / expected CpG, and y-axis represents the gene number.

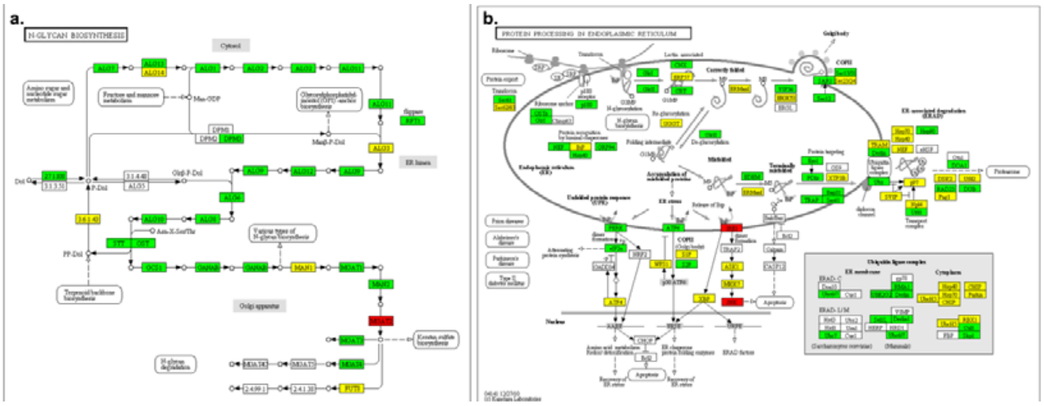

Supplementary Figure 7. Highly methylation genes enriched Pathway. a. N-Glycan biosynthesis pathway, b.Protein processing in endoplasmic reticulum pathway. The pathway maps were drawn based on KEGG database[71]. The green indicates genes with highly methylation (CpG(observed/expected)>1.15), red indicates genes with rare methylation (CpG(observed/expected)<0.53).

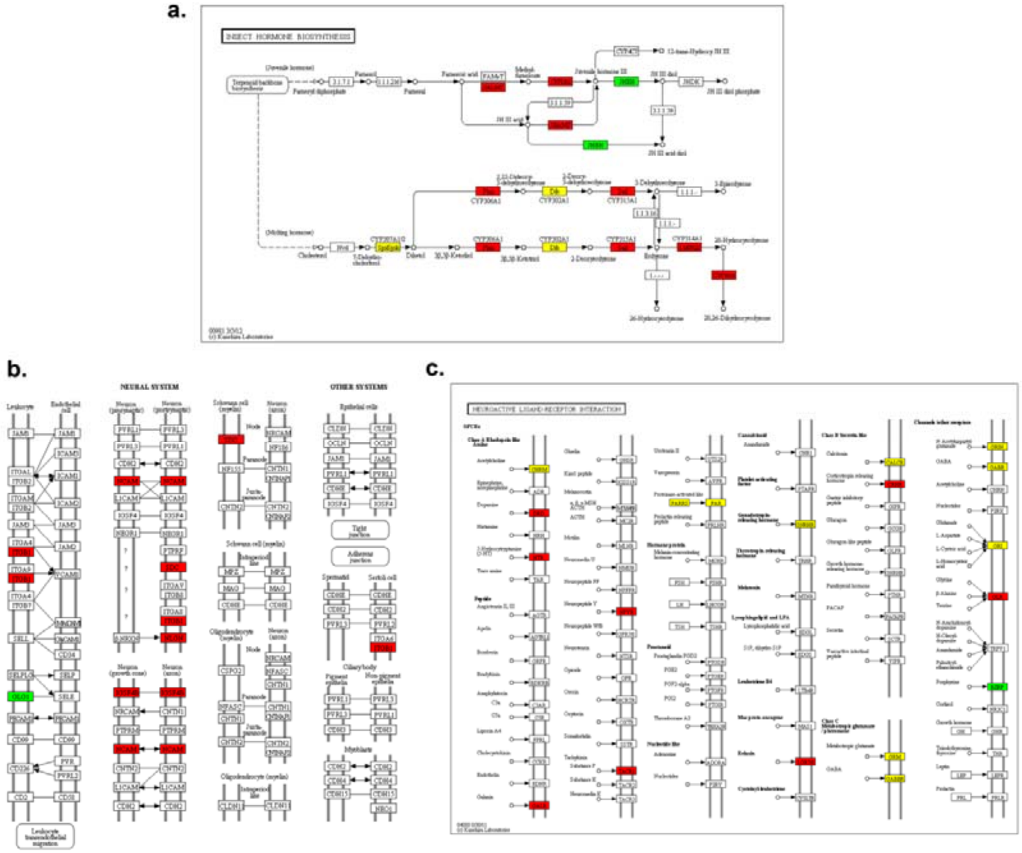

Supplementary Figure 8. Rare methylation genes enriched Pathway. a. Insect hormone biosynthesis pathway (The pathway map was drawn based on KEGG database[71]), **b. Cell adhesion molecules (CAMs), c. Neuroactive ligand-receptor interaction.** The green indicates genes with highly methylation (CpG(observed/expected)>1.15), red indicates genes with rare methylation (CpG(observed/expected)<0.53).

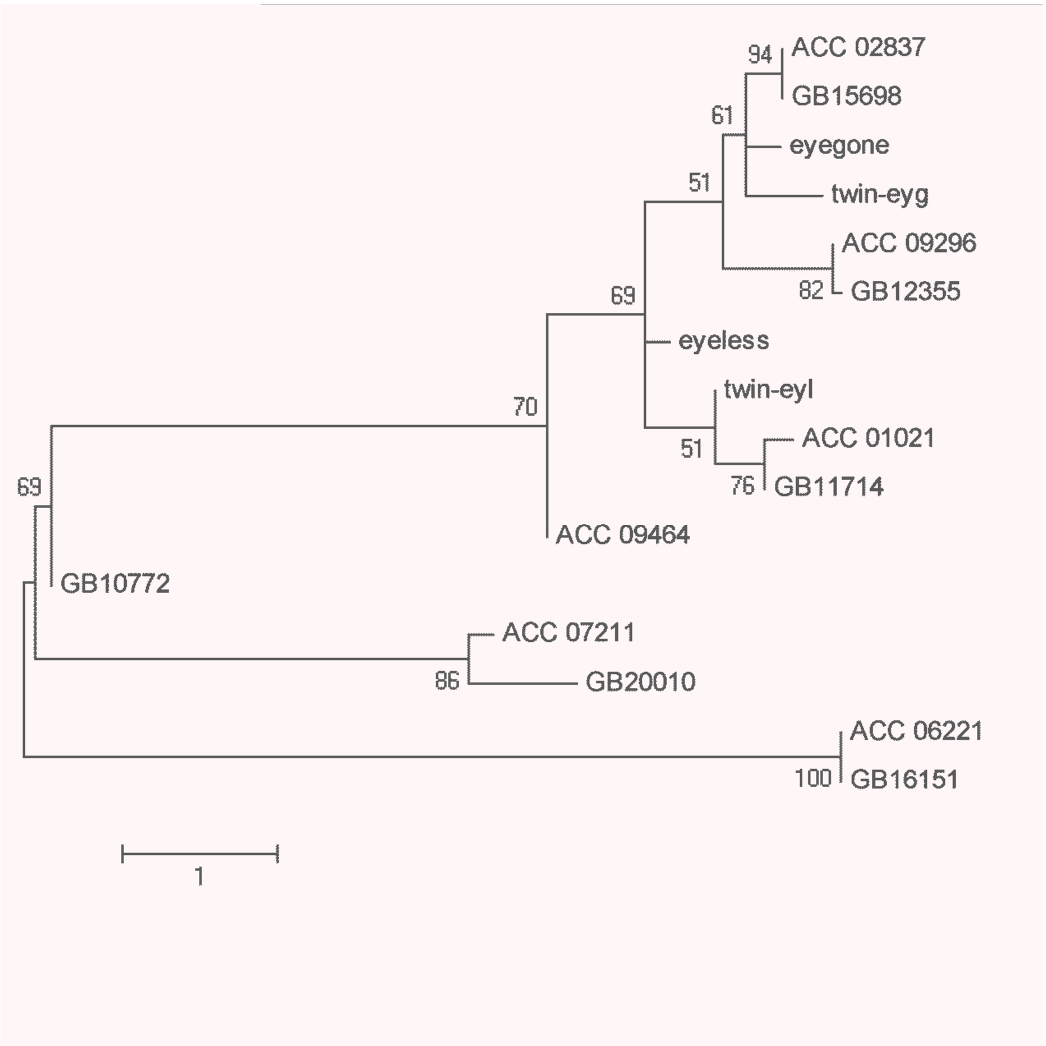

Supplementary Figure 9. Phylogenetic tree of Paired box (Pax) genes. The phylogenetic tree was constructed with protein sequences of Pax from *A.cerana*, *A.mellifera* and *D.melanogaster*, by the neighbor-joining method (1,000 bootstrap replicates) using MEGA v.5 .

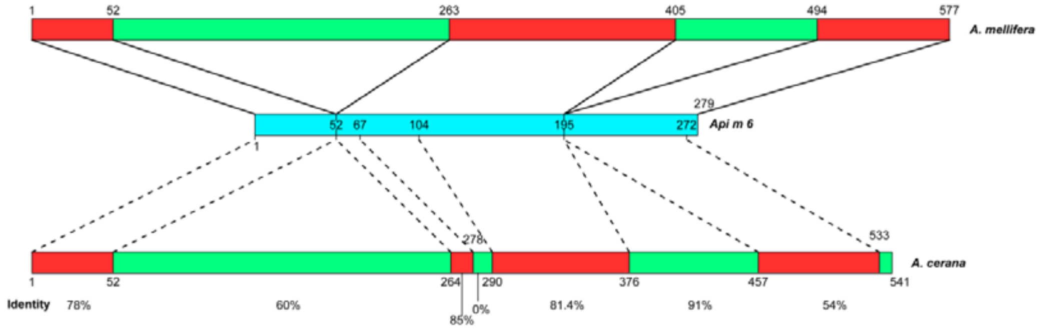

Supplementary Figure 10. Genomic structure comparison of *Api m 6* loci between *A.cerana* and *A.mellifera*. The red blocks represent exons in genome, and green blocks represent introns. The blue bar in the middle represent a complete *Api m 6* CDS of *A.mellifera*.

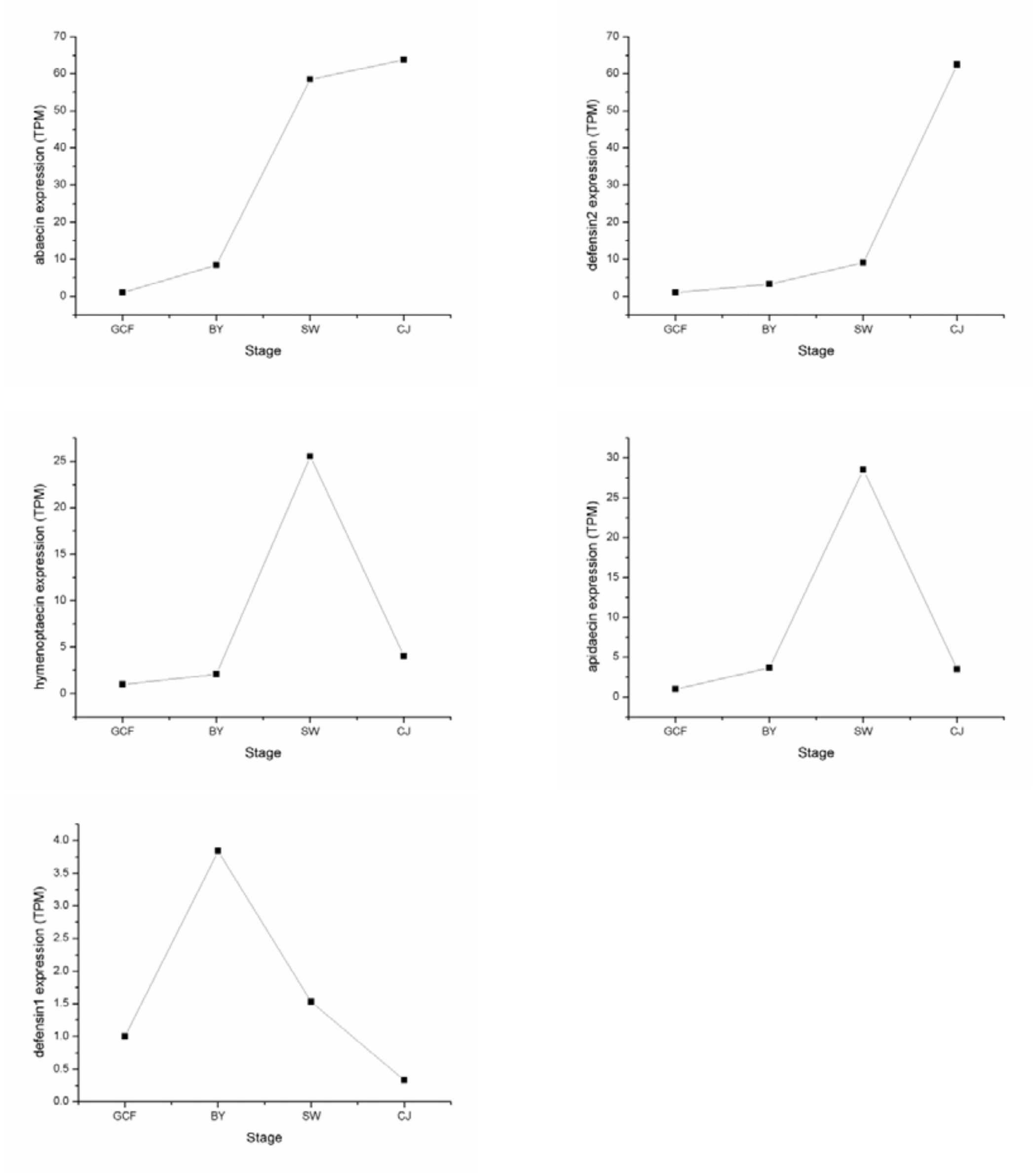

Supplementary Figure 11. Antimicrobial Peptide (AMP) gene expression along with the caste transition. The abbreviations are as follows: GCF, newly-emerged adult; BY, nurse; SW, guard; CJ, forager.

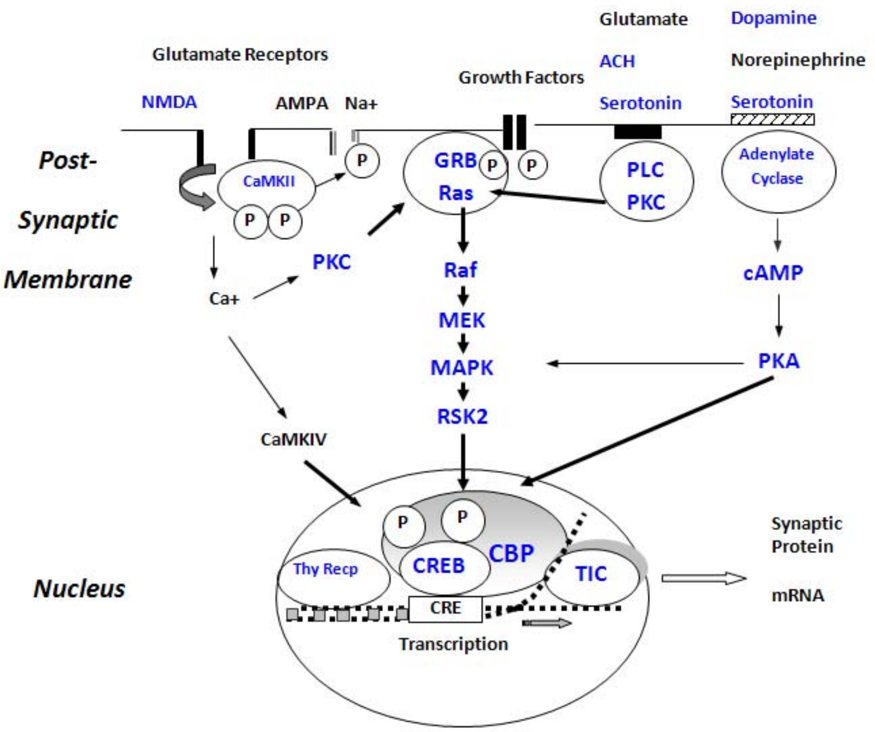

Supplementary Figure 12. Signaling pathways for learning and long-term memory storage described in the text from work in animal models. Gene in blue are homologous gene in *A.cerana*. Three types of neurotransmitter receptors are shown: ionotropic receptors that open ion channels (the NMDA- and AMPA-type glutamate receptors shown at *top left*), metabotropic receptors (glutamate, acetylcholine, and serotonin at *upper right*) linked to synthesis of inositol phosphates and stimulation of PKC, and transmembrane receptors linked to generation of cAMP, shown at *upper far right*.

a.

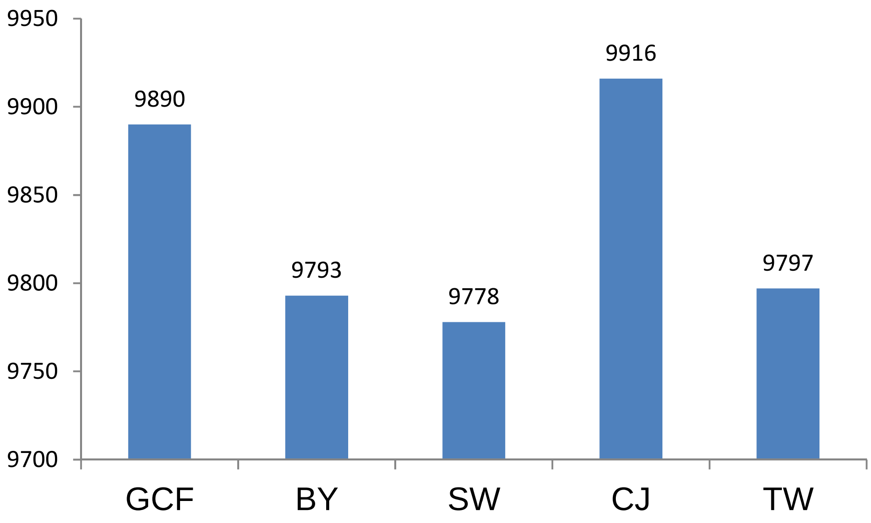

b.

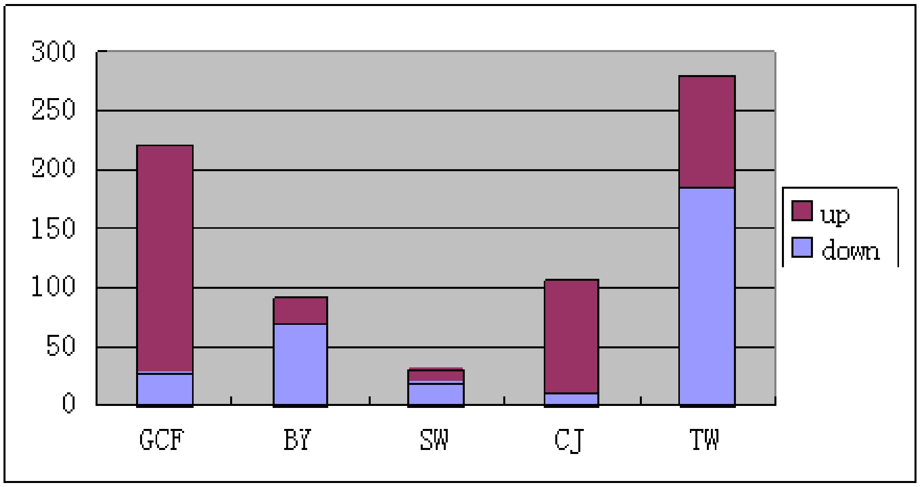

Supplementary Figure 13. The number of expressed genes detected in each stage (a) and the number of genes detected significantly differentially expressed between stages (b). Red represents the up-regulated genes, and purple represents the down-regulated genes.

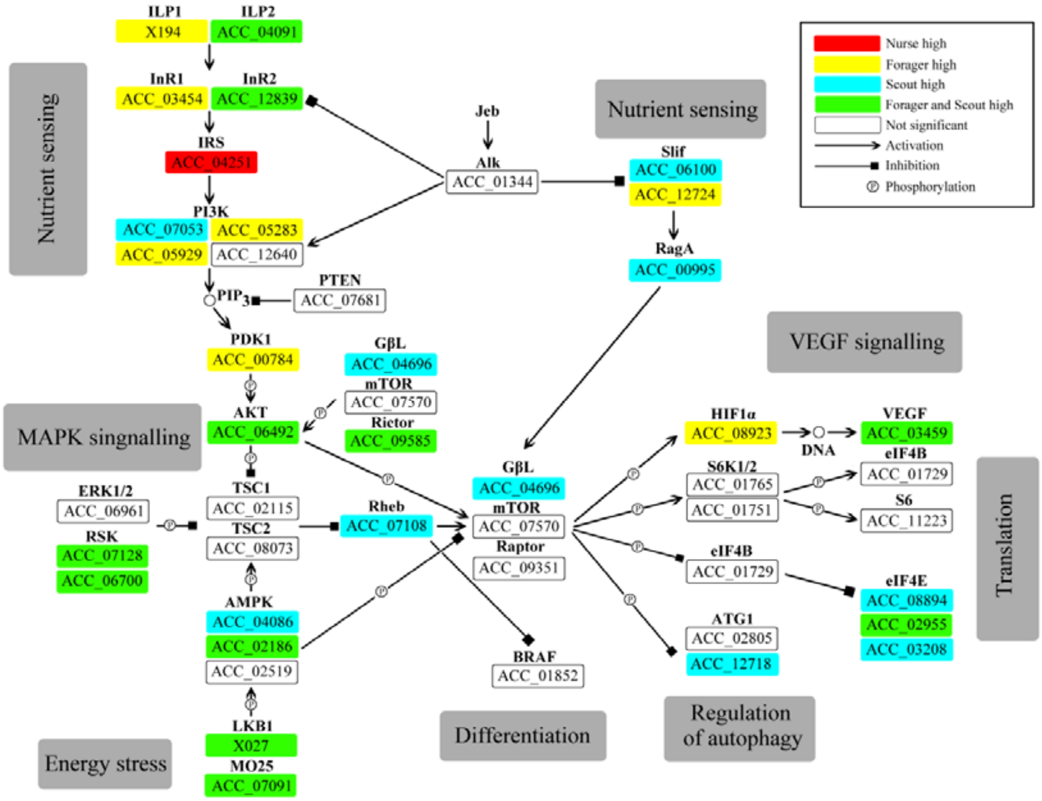

Supplementary Figure 14. The insulin/TOR network in *Apis cerana* showing brain differentially expressed genes between foraging workers and nurses. The pathway map was drawn based on KEGG database[71].

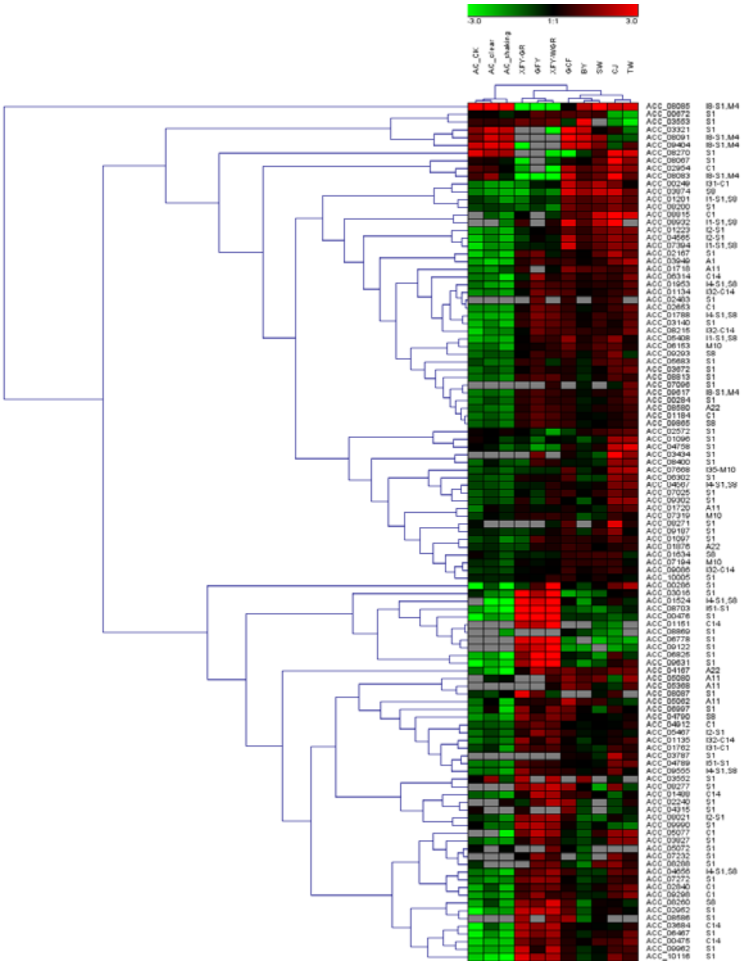

Supplementary Figure 15. Transcription association of protease and protease inhibitor genes in *A.cerana*. The log2-transformed RPKM (reads per kilobase per million sequenced reads) value of each gene from 11 living stages was clustered using Genesis.

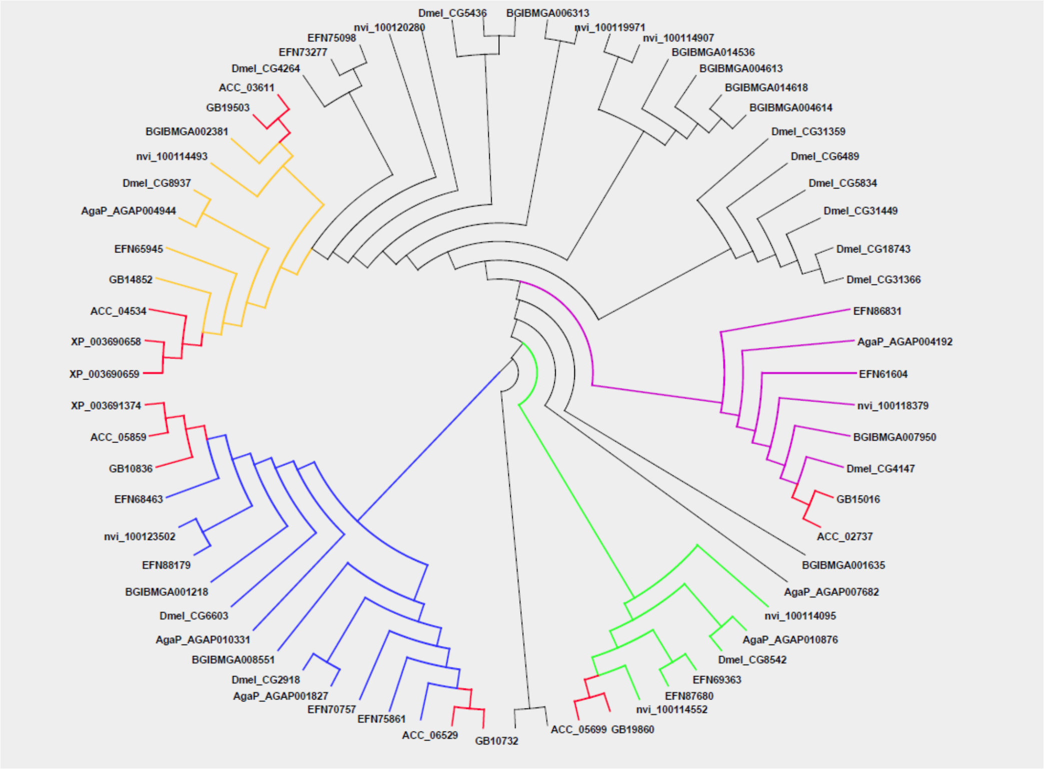

Supplementary Figure 16. Phylogenetic tree of heat shock protein 70 (HSP70) genes. The phylogenetic tree was constructed with protein sequences of HSP70 by the neighbor-joining method (1,000 bootstrap replicates) using MEGA v.5 .

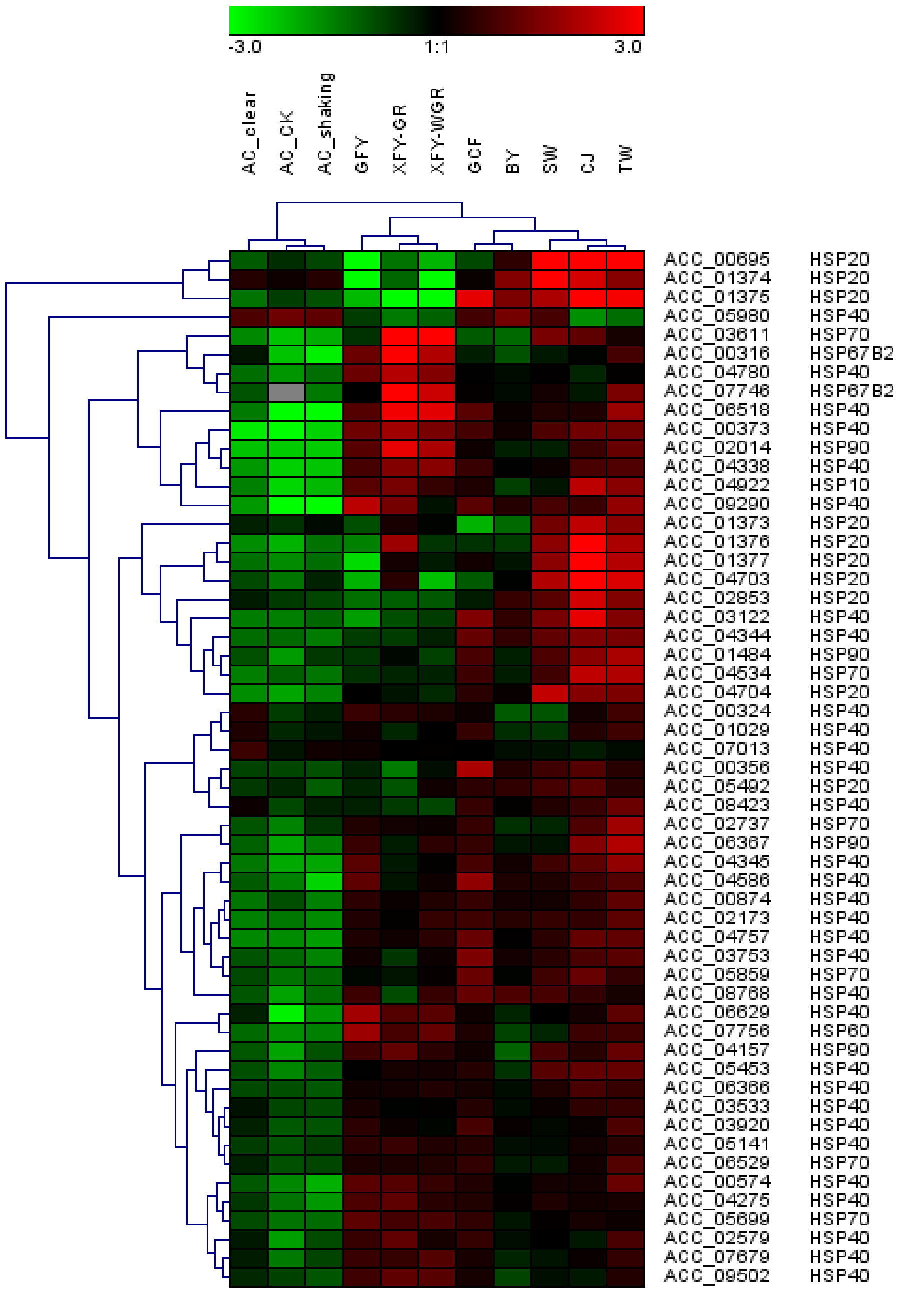

Supplementary Figure 17. Transcription association of heat-shock proteins in *A.cerana*. The log2-transformed RPKM (reads per kilobase per million sequenced reads) value of each gene from 11 living stages was clustered using Genesis.

Supplementary Table 1. Genome Sequencing Statistics

	454 GS FLX	Illumina GA IIx Pair-End (300 bp)	Illumina GA IIx Mate-Pair (3kb)
**Reads number**	1,641,411	99,070,380	46,124,432
**Average length (bp)**	388	2x120	2x120
**Total bases**	636,867,468	23,776,891,200	8,716,583,327
**Coverage (x)***	2.3	88	32

* the coverage counting is base on the scaffold length 229.5 Mb.

Supplementary Table 2. Genome assembly statistics

	Total contigs	Scaffolds
**Number**	21,784	879
**Total size (bp)**	209,159,478	228,791,026
**Average length (bp)**	9,601	260,285
**Largest length (bp)**	174,956	4,477,204
**N50 Length (bp)**	21,160	1,393,515
**N90 Length (bp)**	4,974	290,869

Supplementary Table 3. Chromosome construction of *A.cerana*

Chromosome	*A.cerana* Scaffold Number	NCBI Nucleotide Accession	orientation	Length (bp)
**Chr01**	Scaffold_253	KZ288434.1	-	95123
	Scaffold_003	KZ288187.1	+	1085358
	Scaffold_197	KZ288380.1	-	248208
	Scaffold_094	KZ288278.1	+	224747
	Scaffold_237	KZ288419.1	+	624135
	Scaffold_122	KZ288306.1	+	140905
	Scaffold_174	KZ288357.1	+	2536605
	Scaffold_176	KZ288359.1	+	111647
	Scaffold_181	KZ288364.1	+	1848954
	Scaffold_074	KZ288258.1	+	685032
	Scaffold_030	KZ288214.1	-	433901
	Scaffold_464	KZ288748.1	-	1210
	Scaffold_017	KZ288201.1	+	909270
	Scaffold_089	KZ288273.1	+	263696
	Scaffold_389	KZ288733.1	-	2171
	Scaffold_203	KZ288386.1	+	333260
	Scaffold_381	KZ288732.1	-	2458
	Scaffold_278	KZ288459.1	-	47585
	Scaffold_220	KZ288403.1	-	324334
	Scaffold_369	KZ288541.1	-	10586
	Scaffold_333	KZ288512.1	-	21024
	Scaffold_043	KZ288227.1	+	3426641
	Scaffold_279	KZ288460.1	-	226344
	Scaffold_322	KZ288503.1	+	70043
	Scaffold_137	KZ288321.1	+	234815
	Scaffold_019	KZ288203.1	+	2857148
	Scaffold_160	KZ288343.1	+	226531
	Scaffold_021	KZ288205.1	+	338221
	Scaffold_028	KZ288212.1	-	1879415
	Scaffold_337	KZ288725.1	-	8806
	Scaffold_040	KZ288224.1	+	264826
	Scaffold_023	KZ288207.1	+	319191
	Scaffold_063	KZ288247.1	+	360017
	Scaffold_107	KZ288291.1	+	485334
	Scaffold_091	KZ288275.1	+	245364
	Scaffold_442	KZ288603.1	-	4928
	Scaffold_184	KZ288367.1	+	341811
	Scaffold_216	KZ288399.1	-	79307
	Scaffold_068	KZ288252.1	-	344877
	Scaffold_155	KZ288338.1	-	492317
	Scaffold_054	KZ288238.1	+	988148
	Scaffold_060	KZ288244.1	+	806744
	Scaffold_106	KZ288290.1	+	414836
	Scaffold_185	KZ288368.1	+	419158

	Scaffold_376	KZ288731.1	+	2744
	Scaffold_069	KZ288253.1	+	1386536
	Scaffold_070	KZ288254.1	+	4411227
**Chr02**	Scaffold_345	KZ288522.1	+	86130
	Scaffold_462	KZ288747.1	+	1228
	Scaffold_395	KZ288564.1	+	5525
	Scaffold_049	KZ288233.1	-	338631
	Scaffold_224	KZ288407.1	+	141795
	Scaffold_144	KZ288328.1	-	377744
	Scaffold_008	KZ288192.1	+	4477204
	Scaffold_146	KZ288330.1	-	243217
	Scaffold_005	KZ288189.1	+	4127074
	Scaffold_073	KZ288257.1	-	332195
	Scaffold_140	KZ288324.1	-	595144
	Scaffold_534	KZ288778.1	-	894
	Scaffold_639	KZ288854.1	+	700
	Scaffold_308	KZ288489.1	-	71509
	Scaffold_108	KZ288292.1	+	2595329
	Scaffold_114	KZ288298.1	+	269472
	Scaffold_125	KZ288309.1	+	705758
	Scaffold_067	KZ288251.1	+	645373
	Scaffold_815	KZ288998.1	-	536
	Scaffold_288	KZ288469.1	+	432678
	Scaffold_173	KZ288356.1	-	550742
	Scaffold_139	KZ288323.1	+	775991
	Scaffold_274	KZ288455.1	-	118951
	Scaffold_593	KZ288818.1	-	771
	Scaffold_565	KZ288800.1	-	816
	Scaffold_342	KZ288519.1	+	15706
**Chr03**	Scaffold_020	KZ288204.1	+	3036172
	Scaffold_130	KZ288314.1	+	185149
	Scaffold_116	KZ288300.1	+	440794
	Scaffold_042	KZ288226.1	+	832952
	Scaffold_189	KZ288372.1	-	203605
	Scaffold_222	KZ288405.1	+	339587
	Scaffold_036	KZ288220.1	-	1314320
	Scaffold_048	KZ288232.1	+	2560658
	Scaffold_014	KZ288198.1	+	1578228
	Scaffold_370	KZ288542.1	+	16810
	Scaffold_261	KZ288442.1	+	134219
	Scaffold_072	KZ288256.1	+	2301240
	Scaffold_161	KZ288344.1	-	1214543
**Chr04**	Scaffold_078	KZ288262.1	+	2490196
	Scaffold_097	KZ288281.1	+	171311
	Scaffold_783	KZ288708.1	+	4372
	Scaffold_270	KZ288451.1	-	153887
	Scaffold_013	KZ288197.1	+	2098417

	Scaffold_190	KZ288373.1	-	102544
	Scaffold_483	KZ288755.1	+	1103
	Scaffold_549	KZ288658.1	-	8317
	Scaffold_530	KZ288652.1	+	4368
	Scaffold_022	KZ288206.1	+	1538284
	Scaffold_247	KZ288722.1	+	53246
	Scaffold_085	KZ288269.1	-	2030668
	Scaffold_092	KZ288276.1	+	284976
	Scaffold_234	KZ288416.1	+	1958298
	Scaffold_061	KZ288245.1	+	549877
**Chr05**	Scaffold_121	KZ288305.1	+	552976
	Scaffold_055	KZ288239.1	-	1123706
	Scaffold_045	KZ288229.1	+	1819815
	Scaffold_408	KZ288737.1	-	1773
	Scaffold_152	KZ288720.1	-	115942
	Scaffold_100	KZ288284.1	+	273400
	Scaffold_007	KZ288191.1	+	2290117
	Scaffold_246	KZ288428.1	+	144195
	Scaffold_010	KZ288194.1	-	2866043
	Scaffold_196	KZ288379.1	+	1356105
	Scaffold_204	KZ288387.1	+	759997
	Scaffold_025	KZ288209.1	+	1042630
	Scaffold_192	KZ288375.1	+	631721
	Scaffold_260	KZ288441.1	+	116721
	Scaffold_563	KZ288799.1	-	818
	Scaffold_499	KZ288634.1	+	7912
	Scaffold_356	KZ288728.1	+	4968
	Scaffold_032	KZ288216.1	+	471047
	Scaffold_138	KZ288322.1	-	897170
**Chr06**	Scaffold_109	KZ288293.1	+	2932990
	Scaffold_254	KZ288435.1	-	87262
	Scaffold_202	KZ288385.1	+	88241
	Scaffold_132	KZ288316.1	-	181124
	Scaffold_529	KZ288651.1	-	4660
	Scaffold_090	KZ288274.1	+	283163
	Scaffold_011	KZ288195.1	-	1393515
	Scaffold_143	KZ288327.1	+	350831
	Scaffold_145	KZ288329.1	+	515331
	Scaffold_183	KZ288366.1	+	145038
	Scaffold_083	KZ288267.1	+	1398299
	Scaffold_195	KZ288378.1	-	202818
	Scaffold_018	KZ288202.1	+	2060125
	Scaffold_006	KZ288190.1	-	1599754
	Scaffold_035	KZ288219.1	+	3816228
	Scaffold_076	KZ288260.1	+	838471
	Scaffold_127	KZ288311.1	+	1766480
**Chr07**	Scaffold_257	KZ288438.1	-	337130

	Scaffold_199	KZ288382.1	-	225928
	Scaffold_039	KZ288223.1	+	2418441
	Scaffold_111	KZ288295.1	-	520132
	Scaffold_131	KZ288315.1	+	505814
	Scaffold_168	KZ288351.1	+	314986
	Scaffold_667	KZ288878.1	+	670
	Scaffold_230	KZ288412.1	-	154267
	Scaffold_075	KZ288259.1	+	675395
	Scaffold_235	KZ288417.1	-	206704
	Scaffold_171	KZ288354.1	+	2802284
	Scaffold_267	KZ288448.1	-	38382
	Scaffold_050	KZ288234.1	+	765153
	Scaffold_281	KZ288462.1	+	901458
	Scaffold_136	KZ288320.1	+	701076
	Scaffold_304	KZ288485.1	+	46169
**Chr08**	Scaffold_134	KZ288318.1	+	585574
	Scaffold_296	KZ288477.1	+	48613
	Scaffold_215	KZ288398.1	-	211267
	Scaffold_166	KZ288349.1	+	1603157
	Scaffold_187	KZ288370.1	-	288678
	Scaffold_218	KZ288401.1	+	222079
	Scaffold_436	KZ288598.1	+	1414
	Scaffold_650	KZ288865.1	-	689
	Scaffold_347	KZ288524.1	+	13701
	Scaffold_426	KZ288588.1	-	5317
	Scaffold_227	KZ288410.1	-	80366
	Scaffold_225	KZ288408.1	+	153735
	Scaffold_258	KZ288439.1	-	164276
	Scaffold_262	KZ288443.1	+	111367
	Scaffold_178	KZ288361.1	+	2122269
	Scaffold_034	KZ288218.1	-	614642
	Scaffold_785	KZ288971.1	+	558
	Scaffold_799	KZ288983.1	+	548
	Scaffold_186	KZ288369.1	+	420931
	Scaffold_265	KZ288446.1	+	421065
	Scaffold_096	KZ288280.1	+	815582
	Scaffold_226	KZ288409.1	+	168186
**Chr09**	Scaffold_268	KZ288449.1	-	484001
	Scaffold_147	KZ288331.1	+	471407
	Scaffold_162	KZ288345.1	+	719830
	Scaffold_448	KZ288607.1	+	4901
	Scaffold_179	KZ288362.1	+	344888
	Scaffold_180	KZ288363.1	+	296057
	Scaffold_024	KZ288208.1	+	845429
	Scaffold_153	KZ288336.1	+	511805
	Scaffold_081	KZ288265.1	+	497504
	Scaffold_485	KZ288757.1	-	1076

	Scaffold_165	KZ288348.1	+	422833
	Scaffold_617	KZ288838.1	-	729
	Scaffold_088	KZ288272.1	+	565617
	Scaffold_598	KZ288822.1	-	767
	Scaffold_012	KZ288196.1	+	595523
	Scaffold_170	KZ288353.1	+	1406821
	Scaffold_302	KZ288483.1	-	67836
	Scaffold_177	KZ288360.1	+	525071
	Scaffold_009	KZ288193.1	+	4342787
	Scaffold_295	KZ288476.1	+	256474
	Scaffold_329	KZ288510.1	+	148637
**Chr10**	Scaffold_079	KZ288263.1	+	1192271
	Scaffold_080	KZ288264.1	-	193651
	Scaffold_456	KZ288612.1	-	8395
	Scaffold_447	KZ288743.1	+	1347
	Scaffold_460	KZ288615.1	+	4425
	Scaffold_523	KZ288647.1	+	4690
	Scaffold_807	KZ288990.1	+	544
	Scaffold_361	KZ288536.1	+	5899
	Scaffold_156	KZ288339.1	+	115749
	Scaffold_151	KZ288335.1	+	368175
	Scaffold_228	KZ288411.1	+	117696
	Scaffold_119	KZ288303.1	+	288670
	Scaffold_449	KZ288608.1	-	1344
	Scaffold_559	KZ288796.1	-	823
	Scaffold_602	KZ288826.1	-	764
	Scaffold_120	KZ288304.1	+	143083
	Scaffold_243	KZ288425.1	+	254095
	Scaffold_135	KZ288319.1	+	245044
	Scaffold_248	KZ288429.1	+	141550
	Scaffold_507	KZ288770.1	+	980
	Scaffold_167	KZ288350.1	+	141887
	Scaffold_059	KZ288243.1	-	519194
	Scaffold_256	KZ288437.1	+	51678
	Scaffold_052	KZ288236.1	+	227525
	Scaffold_201	KZ288384.1	+	150065
	Scaffold_263	KZ288444.1	-	232237
	Scaffold_475	KZ288624.1	+	5184
	Scaffold_154	KZ288337.1	+	172578
	Scaffold_205	KZ288388.1	+	108255
	Scaffold_191	KZ288374.1	+	182657
	Scaffold_194	KZ288377.1	-	232894
	Scaffold_206	KZ288389.1	+	638782
	Scaffold_210	KZ288393.1	+	215054
	Scaffold_207	KZ288390.1	+	82289
	Scaffold_098	KZ288282.1	-	1759026
	Scaffold_209	KZ288392.1	+	228087

	Scaffold_182	KZ288365.1	-	368716
	Scaffold_212	KZ288395.1	+	86288
	Scaffold_238	KZ288420.1	+	134564
	Scaffold_297	KZ288478.1	-	169879
	Scaffold_214	KZ288397.1	-	82731
	Scaffold_417	KZ288582.1	+	5460
	Scaffold_115	KZ288299.1	+	251422
	Scaffold_086	KZ288270.1	+	205754
	Scaffold_126	KZ288310.1	-	1278953
	Scaffold_159	KZ288342.1	+	262909
	Scaffold_188	KZ288371.1	+	154106
	Scaffold_888	KZ289062.1	-	500
	Scaffold_658	KZ288871.1	-	679
	Scaffold_401	KZ288735.1	-	1914
	Scaffold_198	KZ288381.1	+	160088
	Scaffold_164	KZ288347.1	+	1546432
	Scaffold_123	KZ288307.1	+	290869
	Scaffold_312	KZ288493.1	-	67398
	Scaffold_315	KZ288496.1	-	46256
	Scaffold_371	KZ288543.1	-	15032
	Scaffold_422	KZ288738.1	+	1583
	Scaffold_444	KZ288604.1	+	8513
**Chr11**	Scaffold_221	KZ288404.1	-	133493
	Scaffold_112	KZ288296.1	+	1161095
	Scaffold_113	KZ288297.1	+	491503
	Scaffold_229	KZ288721.1	-	63981
	Scaffold_169	KZ288352.1	+	245442
	Scaffold_213	KZ288396.1	+	574393
	Scaffold_654	KZ288869.1	+	683
	Scaffold_366	KZ288540.1	+	6097
	Scaffold_379	KZ288550.1	+	2587
	Scaffold_556	KZ288795.1	+	828
	Scaffold_513	KZ288773.1	+	970
	Scaffold_217	KZ288400.1	+	947709
	Scaffold_271	KZ288452.1	-	807100
	Scaffold_004	KZ288188.1	-	642796
	Scaffold_172	KZ288355.1	+	213567
	Scaffold_193	KZ288376.1	-	300089
	Scaffold_443	KZ288742.1	-	1368
	Scaffold_053	KZ288237.1	+	592691
	Scaffold_095	KZ288279.1	-	444502
	Scaffold_341	KZ288518.1	+	40355
	Scaffold_071	KZ288255.1	+	1869179
	Scaffold_566	KZ288801.1	-	811
	Scaffold_057	KZ288241.1	-	808661
	Scaffold_077	KZ288261.1	+	309293
	Scaffold_066	KZ288250.1	+	892110

	Scaffold_031	KZ288215.1	-	3689919
**Chr12**	Scaffold_037	KZ288221.1	+	446687
	Scaffold_056	KZ288240.1	+	610921
	Scaffold_457	KZ288613.1	+	4670
	Scaffold_849	KZ289027.1	-	517
	Scaffold_512	KZ288639.1	+	4491
	Scaffold_351	KZ288527.1	+	21537
	Scaffold_511	KZ288638.1	-	4572
	Scaffold_481	KZ288627.1	-	8314
	Scaffold_536	KZ288780.1	+	888
	Scaffold_002	KZ288186.1	+	4346810
	Scaffold_325	KZ288506.1	+	27673
	Scaffold_328	KZ288509.1	-	39153
	Scaffold_062	KZ288246.1	-	392003
	Scaffold_158	KZ288341.1	-	111387
	Scaffold_047	KZ288231.1	+	597950
	Scaffold_646	KZ288861.1	+	692
	Scaffold_029	KZ288213.1	-	303260
	Scaffold_148	KZ288332.1	-	206311
	Scaffold_242	KZ288424.1	-	112090
	Scaffold_033	KZ288217.1	-	1777032
	Scaffold_208	KZ288391.1	+	879560
	Scaffold_306	KZ288487.1	-	80032
	Scaffold_101	KZ288285.1	+	1093429
**Chr13**	Scaffold_290	KZ288471.1	+	127297
	Scaffold_516	KZ288642.1	-	4924
	Scaffold_554	KZ288660.1	-	12487
	Scaffold_666	KZ288686.1	-	672
	Scaffold_133	KZ288317.1	+	376182
	Scaffold_163	KZ288346.1	+	299386
	Scaffold_414	KZ288579.1	+	5809
	Scaffold_546	KZ288789.1	-	853
	Scaffold_433	KZ288595.1	-	4454
	Scaffold_702	KZ288902.1	+	627
	Scaffold_471	KZ288620.1	+	4323
	Scaffold_407	KZ288573.1	+	8217
	Scaffold_573	KZ288808.1	-	800
	Scaffold_236	KZ288418.1	-	152313
	Scaffold_150	KZ288334.1	+	301776
	Scaffold_065	KZ288249.1	-	351566
	Scaffold_232	KZ288414.1	-	203145
	Scaffold_099	KZ288283.1	+	170780
	Scaffold_046	KZ288230.1	+	2980393
	Scaffold_142	KZ288326.1	+	466443
	Scaffold_093	KZ288277.1	+	314179
	Scaffold_027	KZ288211.1	-	304853
	Scaffold_058	KZ288242.1	-	1113656

	Scaffold_233	KZ288415.1	+	113161
	Scaffold_118	KZ288302.1	+	518149
	Scaffold_454	KZ288745.1	+	1283
	Scaffold_385	KZ288555.1	-	2301
	Scaffold_175	KZ288358.1	+	1027499
**Chr14**	Scaffold_104	KZ288288.1	+	1049202
	Scaffold_044	KZ288228.1	+	401773
	Scaffold_557	KZ288661.1	-	4523
	Scaffold_607	KZ288831.1	+	749
	Scaffold_084	KZ288268.1	+	765910
	Scaffold_334	KZ288513.1	-	10148
	Scaffold_350	KZ288526.1	+	5342
	Scaffold_026	KZ288210.1	+	3052130
	Scaffold_110	KZ288294.1	+	223753
	Scaffold_124	KZ288308.1	+	985629
	Scaffold_157	KZ288340.1	+	815212
	Scaffold_064	KZ288248.1	-	998191
	Scaffold_305	KZ288486.1	+	80804
	Scaffold_105	KZ288289.1	+	1010466
	Scaffold_129	KZ288313.1	+	577833
	Scaffold_241	KZ288423.1	-	353904
	Scaffold_463	KZ288617.1	-	4858
	Scaffold_438	KZ288600.1	-	8858
**Chr15**	Scaffold_016	KZ288200.1	+	552538
	Scaffold_298	KZ288479.1	+	127197
	Scaffold_293	KZ288474.1	-	350643
	Scaffold_015	KZ288199.1	+	761024
	Scaffold_051	KZ288235.1	+	511158
	Scaffold_082	KZ288266.1	+	2686839
	Scaffold_087	KZ288271.1	+	1906270
	Scaffold_149	KZ288333.1	-	117518
	Scaffold_001	KZ288185.1	+	2787216
	Scaffold_103	KZ288287.1	+	1067626
	Scaffold_240	KZ288422.1	-	219168
	Scaffold_200	KZ288383.1	+	236416
	Scaffold_244	KZ288426.1	+	73906
	Scaffold_117	KZ288301.1	+	1236372
**Chr16**	Scaffold_038	KZ288222.1	+	2866834
	Scaffold_141	KZ288325.1	+	1060848
	Scaffold_245	KZ288427.1	+	1105163
	Scaffold_102	KZ288286.1	+	236504
	Scaffold_041	KZ288225.1	-	529373
	Scaffold_128	KZ288312.1	+	1332606
	Scaffold_231	KZ288413.1	-	357473

Supplementary Table 4. Genes without introns in A.cerana genome

Gene	Length	Scaffold	Start	End	Strand
ACC_00001	1293	scaffold1.1	91927	93219	-
ACC_00069	1743	scaffold10.1	1032865	1034607	-
ACC_00074	1011	scaffold10.1	1090556	1091566	-
ACC_00086	354	scaffold10.1	1954895	1955248	-
ACC_00117	504	scaffold20.1	2925581	2926084	-
ACC_00140	360	scaffold101.1	944338	944697	-
ACC_00151	405	scaffold103.1	437277	437681	-
ACC_00222	1077	scaffold108.1	353173	354249	-
ACC_00234	375	scaffold108.1	1131223	1131597	-
ACC_00238	3741	scaffold108.1	1542098	1545838	-
ACC_00254	252	scaffold108.1	2485132	2485383	-
ACC_00260	288	scaffold109.1	1198381	1198668	-
ACC_00275	288	scaffold11.1	159771	160058	-
ACC_00296	1509	scaffold111.1	31477	32985	-
ACC_00314	267	scaffold112.1	319125	319391	-
ACC_00354	627	scaffold117.1	996855	997481	-
ACC_00383	1602	scaffold124.1	27988	29589	-
ACC_00385	1539	scaffold124.1	161062	162600	-
ACC_00404	204	scaffold125.1	105323	105526	-
ACC_00417	243	scaffold126.1	349973	350215	-
ACC_00419	1995	scaffold126.1	425898	427892	-
ACC_00431	1113	scaffold126.1	950655	951767	-
ACC_00456	273	scaffold127.1	416031	416303	-
ACC_00467	2169	scaffold127.1	898952	901120	-
ACC_00468	1191	scaffold127.1	1052272	1053462	-
ACC_00498	1296	scaffold129.1	405926	407221	-
ACC_00514	354	scaffold13.1	1461296	1461649	-
ACC_00516	1605	scaffold13.1	1506515	1508119	-
ACC_00518	228	scaffold13.1	1689827	1690054	-
ACC_00537	201	scaffold134.1	260939	261139	-
ACC_00546	2718	scaffold136.1	351500	354217	-
ACC_00567	1521	scaffold138.1	580963	582483	-
ACC_00578	219	scaffold138.1	851488	851706	-
ACC_00585	204	scaffold139.1	474936	475139	-
ACC_00608	603	scaffold14.1	1447636	1448238	-
ACC_00613	1365	scaffold250.1	56128	57492	-
ACC_00629	1812	scaffold141.1	493090	494901	-
ACC_00640	1170	scaffold141.1	716674	717843	-
ACC_00650	2049	scaffold142.1	445405	447453	-
ACC_00654	993	scaffold143.1	249357	250349	-
ACC_00663	756	scaffold145.1	50206	50961	-
ACC_00677	2109	scaffold185.1	144455	146563	-
ACC_00691	1341	scaffold19.1	360605	361945	-
ACC_00695	525	scaffold19.1	1388412	1388936	-
ACC_00697	651	scaffold19.1	1630007	1630657	-
ACC_00706	2835	scaffold150.1	8545	11379	-

ACC_00729	204	scaffold155.1	273938	274141	-
ACC_00735	1569	scaffold157.1	64123	65691	-
ACC_00765	513	scaffold171.1	1888868	1889380	-
ACC_00784	1269	scaffold162.1	115272	116540	-
ACC_00800	3558	scaffold162.1	461845	465402	-
ACC_00804	813	scaffold162.1	555254	556066	-
ACC_00836	975	scaffold164.1	1237883	1238857	-
ACC_00837	3897	scaffold165.1	105848	109744	-
ACC_00905	753	scaffold1.1	1909169	1909921	-
ACC_00920	360	scaffold170.1	1295657	1296016	-
ACC_00926	375	scaffold171.1	480929	481303	-
ACC_00928	231	scaffold171.1	630169	630399	-
ACC_00930	216	scaffold171.1	1128746	1128961	-
ACC_00937	753	scaffold172.1	65900	66652	-
ACC_00952	237	scaffold174.1	98236	98472	-
ACC_00978	981	scaffold220.1	70461	71441	-
ACC_00982	1008	scaffold175.1	494023	495030	-
ACC_00983	6	scaffold175.1	549297	549302	-
ACC_00986	303	scaffold273.1	13755	14057	-
ACC_01008	339	scaffold178.1	2102035	2102373	-
ACC_01051	438	scaffold181.1	81151	81588	-
ACC_01061	702	scaffold181.1	1317385	1318086	-
ACC_01062	660	scaffold181.1	1325520	1326179	-
ACC_01065	648	scaffold181.1	1368370	1369017	-
ACC_01066	243	scaffold181.1	1415635	1415877	-
ACC_01075	1137	scaffold193.1	177388	178524	-
ACC_01095	186	scaffold187.1	132374	132559	-
ACC_01115	375	scaffold19.1	612226	612600	-
ACC_01116	411	scaffold19.1	624930	625340	-
ACC_01121	411	scaffold19.1	1303531	1303941	-
ACC_01122	381	scaffold19.1	1305358	1305738	-
ACC_01123	375	scaffold19.1	1312076	1312450	-
ACC_01124	678	scaffold19.1	1317999	1318676	-
ACC_01141	1023	scaffold92.1	277234	278256	-
ACC_01142	1863	scaffold191.1	127038	128900	-
ACC_01143	1458	scaffold191.1	133005	134462	-
ACC_01148	1380	scaffold191.1	177089	178468	-
ACC_01149	525	scaffold192.1	296343	296867	-
ACC_01186	468	scaffold196.1	1035108	1035575	-
ACC_01220	2067	scaffold2.1	580127	582193	-
ACC_01246	690	scaffold70.1	2920955	2921644	-
ACC_01292	1386	scaffold20.1	2322203	2323588	-
ACC_01313	327	scaffold200.1	25367	25693	-
ACC_01334	1461	scaffold204.1	189875	191335	-
ACC_01338	1113	scaffold204.1	223221	224333	-
ACC_01360	1704	scaffold208.1	449430	451133	-
ACC_01373	603	scaffold212.1	59969	60571	-
ACC_01374	711	scaffold212.1	63317	64027	-

ACC_01375	582	scaffold212.1	66391	66972	-
ACC_01376	648	scaffold212.1	68684	69331	-
ACC_01377	585	scaffold212.1	74612	75196	-
ACC_01380	1434	scaffold264.1	161652	163085	-
ACC_01384	210	scaffold213.1	232470	232679	-
ACC_01391	390	scaffold217.1	192951	193340	-
ACC_01406	780	scaffold22.1	34929	35708	-
ACC_01416	1860	scaffold22.1	668661	670520	-
ACC_01442	2352	scaffold222.1	121488	123839	-
ACC_01461	837	scaffold225.1	101422	102258	-
ACC_01493	1575	scaffold234.1	1884978	1886552	-
ACC_01501	225	scaffold236.1	63994	64218	-
ACC_01512	249	scaffold24.1	438493	438741	-
ACC_01516	2148	scaffold72.1	1837252	1839399	-
ACC_01523	3369	scaffold241.1	94063	97431	-
ACC_01558	324	scaffold246.1	1103680	1104003	-
ACC_01579	375	scaffold7.1	2117278	2117652	-
ACC_01583	291	scaffold250.1	3888	4178	-
ACC_01584	672	scaffold250.1	8722	9393	-
ACC_01591	396	scaffold252.1	289388	289783	-
ACC_01594	303	scaffold253.1	37018	37320	-
ACC_01596	210	scaffold253.1	52895	53104	-
ACC_01604	552	scaffold254.1	41706	42257	-
ACC_01605	750	scaffold254.1	43841	44590	-
ACC_01612	213	scaffold258.1	123852	124064	-
ACC_01614	297	scaffold258.1	152974	153270	-
ACC_01631	330	scaffold26.1	679319	679648	-
ACC_01633	489	scaffold26.1	774137	774625	-
ACC_01642	495	scaffold26.1	1594969	1595463	-
ACC_01648	228	scaffold26.1	2751500	2751727	-
ACC_01649	339	scaffold26.1	2847215	2847553	-
ACC_01658	204	scaffold262.1	103033	103236	-
ACC_01681	936	scaffold266.1	47152	48087	-
ACC_01696	264	scaffold270.1	72577	72840	-
ACC_01703	240	scaffold272.1	43628	43867	-
ACC_01705	420	scaffold272.1	703722	704141	-
ACC_01711	546	scaffold272.1	803705	804250	-
ACC_01727	372	scaffold28.1	112001	112372	-
ACC_01760	321	scaffold282.1	575831	576151	-
ACC_01767	210	scaffold284.1	317897	318106	-
ACC_01797	477	scaffold289.1	370248	370724	-
ACC_01810	207	scaffold290.1	363958	364164	-
ACC_01816	1326	scaffold292.1	42746	44071	-
ACC_01826	216	scaffold296.1	150332	150547	-
ACC_01832	318	scaffold298.1	148040	148357	-
ACC_01846	228	scaffold301.1	142926	143153	-
ACC_01882	408	scaffold87.1	1209014	1209421	-
ACC_01907	999	scaffold31.1	1605712	1606710	-

ACC_01947	228	scaffold314.1	10252	10479	-
ACC_01948	342	scaffold314.1	26764	27105	-
ACC_01960	306	scaffold324.1	25965	26270	-
ACC_01975	384	scaffold33.1	338398	338781	-
ACC_02038	525	scaffold35.1	2052138	2052662	-
ACC_02043	420	scaffold35.1	2237289	2237708	-
ACC_02046	1518	scaffold35.1	3107622	3109139	-
ACC_02066	3030	scaffold36.1	436687	439716	-
ACC_02079	1077	scaffold127.1	779760	780836	-
ACC_02098	1608	scaffold38.1	1645096	1646703	-
ACC_02105	441	scaffold38.1	2509806	2510246	-
ACC_02128	222	scaffold39.1	2172202	2172423	-
ACC_02129	255	scaffold391.1	5212	5466	-
ACC_02130	405	scaffold391.1	8252	8656	-
ACC_02138	795	scaffold9.1	1196815	1197609	-
ACC_02154	222	scaffold401.1	25	246	-
ACC_02163	735	scaffold42.1	156882	157616	-
ACC_02164	327	scaffold42.1	157986	158312	-
ACC_02174	309	scaffold42.1	576353	576661	-
ACC_02208	210	scaffold43.1	1093498	1093707	-
ACC_02211	498	scaffold43.1	1574675	1575172	-
ACC_02215	6405	scaffold43.1	1822026	1828430	-
ACC_02224	1029	scaffold83.1	185947	186975	-
ACC_02225	1173	scaffold45.1	188043	189215	-
ACC_02241	1698	scaffold45.1	1375593	1377290	-
ACC_02268	480	scaffold69.1	924385	924864	-
ACC_02298	258	scaffold48.1	1625368	1625625	-
ACC_02316	804	scaffold5.1	35041	35844	-
ACC_02355	882	scaffold5.1	1465043	1465924	-
ACC_02371	5340	scaffold5.1	3162663	3168002	-
ACC_02376	1284	scaffold5.1	3216960	3218243	-
ACC_02382	462	scaffold5.1	3296909	3297370	-
ACC_02392	585	scaffold5.1	3765664	3766248	-
ACC_02411	483	scaffold126.1	1108917	1109399	-
ACC_02413	798	scaffold502.1	192	989	-
ACC_02416	585	scaffold51.1	256281	256865	-
ACC_02449	1878	scaffold54.1	313206	315083	-
ACC_02453	1521	scaffold54.1	390618	392138	-
ACC_02470	288	scaffold55.1	1111021	1111308	-
ACC_02471	267	scaffold557.1	297	563	-
ACC_02504	1056	scaffold60.1	692627	693682	-
ACC_02524	357	scaffold633.1	316	672	-
ACC_02579	1866	scaffold69.1	499621	501486	-
ACC_02586	2046	scaffold69.1	916607	918652	-
ACC_02600	237	scaffold7.1	150742	150978	-
ACC_02623	225	scaffold7.1	1947871	1948095	-
ACC_02624	363	scaffold7.1	2004041	2004403	-
ACC_02642	285	scaffold70.1	33047	33331	-

ACC_02644	255	scaffold70.1	118854	119108	-
ACC_02655	960	scaffold70.1	830627	831586	-
ACC_02656	963	scaffold70.1	832517	833479	-
ACC_02671	1791	scaffold70.1	1145357	1147147	-
ACC_02689	1002	scaffold70.1	3270648	3271649	-
ACC_02697	801	scaffold70.1	3541004	3541804	-
ACC_02698	1890	scaffold70.1	3602039	3603928	-
ACC_02700	384	scaffold70.1	4297498	4297881	-
ACC_02733	2931	scaffold72.1	1558696	1561626	-
ACC_02798	645	scaffold78.1	2137726	2138370	-
ACC_02849	285	scaffold8.1	3061172	3061456	-
ACC_02857	756	scaffold8.1	4278907	4279662	-
ACC_02869	2097	scaffold82.1	620868	622964	-
ACC_02901	312	scaffold82.1	2457355	2457666	-
ACC_02942	966	scaffold85.1	685026	685991	-
ACC_02977	921	scaffold86.1	12949	13869	-
ACC_03023	270	scaffold88.1	247419	247688	-
ACC_03060	1179	scaffold9.1	3880508	3881686	-
ACC_03081	3597	scaffold95.1	173061	176657	-
ACC_03108	1056	scaffold98.1	882246	883301	-
ACC_03166	3345	scaffold8.1	1781270	1784614	+
ACC_03190	1890	scaffold8.1	3048946	3050835	+
ACC_03211	1449	scaffold10.1	834495	835943	+
ACC_03232	1851	scaffold10.1	1462638	1464488	+
ACC_03243	936	scaffold10.1	1939661	1940596	+
ACC_03245	1362	scaffold10.1	2012109	2013470	+
ACC_03286	240	scaffold101.1	15769	16008	+
ACC_03294	1059	scaffold101.1	519363	520421	+
ACC_03322	450	scaffold103.1	591563	592012	+
ACC_03327	657	scaffold103.1	636439	637095	+
ACC_03363	1743	scaffold140.1	75918	77660	+
ACC_03416	201	scaffold108.1	1475275	1475475	+
ACC_03422	5394	scaffold108.1	1587815	1593208	+
ACC_03448	276	scaffold109.1	1997668	1997943	+
ACC_03453	1203	scaffold109.1	2526800	2528002	+
ACC_03472	2409	scaffold11.1	446588	448996	+
ACC_03499	6531	scaffold111.1	406688	413218	+
ACC_03506	573	scaffold112.1	310990	311562	+
ACC_03516	543	scaffold112.1	764386	764928	+
ACC_03531	234	scaffold114.1	256421	256654	+
ACC_03545	375	scaffold116.1	88367	88741	+
ACC_03572	471	scaffold118.1	412745	413215	+
ACC_03580	3654	scaffold59.1	6068	9721	-
ACC_03676	732	scaffold127.1	219915	220646	+
ACC_03740	342	scaffold141.1	244152	244493	+
ACC_03748	855	scaffold13.1	631637	632491	+
ACC_03756	483	scaffold141.1	967691	968173	+
ACC_03761	1386	scaffold13.1	1963891	1965276	+

ACC_03768	1731	scaffold38.1	1335758	1337488	+
ACC_03791	225	scaffold133.1	334010	334234	+
ACC_03818	1476	scaffold138.1	191435	192910	+
ACC_03828	405	scaffold138.1	430659	431063	+
ACC_03837	426	scaffold138.1	631091	631516	+
ACC_03844	273	scaffold138.1	873857	874129	+
ACC_03851	1374	scaffold95.1	90911	92284	+
ACC_03864	555	scaffold14.1	800367	800921	+
ACC_03903	55	scaffold141.1	359969	360023	+
ACC_03926	861	scaffold145.1	57857	58717	+
ACC_03945	564	scaffold147.1	189811	190374	+
ACC_03961	411	scaffold19.1	614697	615107	+
ACC_03962	375	scaffold19.1	622671	623045	+
ACC_03965	1749	scaffold19.1	867278	869026	+
ACC_03967	312	scaffold19.1	1304234	1304545	+
ACC_03968	312	scaffold19.1	1310702	1311013	+
ACC_03969	372	scaffold19.1	1314479	1314850	+
ACC_04002	831	scaffold153.1	375995	376825	+
ACC_04067	873	scaffold161.1	781732	782604	+
ACC_04120	948	scaffold165.1	244958	245905	+
ACC_04123	2940	scaffold165.1	412863	415802	+
ACC_04137	3261	scaffold166.1	744762	748022	+
ACC_04157	2154	scaffold68.1	281424	283577	+
ACC_04191	804	scaffold17.1	412375	413178	+
ACC_04193	243	scaffold17.1	423325	423567	+
ACC_04199	1932	scaffold1.1	597409	599340	+
ACC_04220	282	scaffold170.1	848008	848289	+
ACC_04224	711	scaffold170.1	943582	944292	+
ACC_04233	1422	scaffold170.1	1313060	1314481	+
ACC_04238	219	scaffold171.1	94956	95174	+
ACC_04243	432	scaffold171.1	1278598	1279029	+
ACC_04249	261	scaffold172.1	35126	35386	+
ACC_04271	1110	scaffold173.1	517780	518889	+
ACC_04273	231	scaffold174.1	155225	155455	+
ACC_04281	207	scaffold174.1	1248983	1249189	+
ACC_04311	2172	scaffold175.1	589168	591339	+
ACC_04316	261	scaffold177.1	325079	325339	+
ACC_04351	1938	scaffold18.1	761625	763562	+
ACC_04363	381	scaffold82.1	1185181	1185561	+
ACC_04397	1572	scaffold27.1	157344	158915	+
ACC_04407	258	scaffold181.1	1599874	1600131	+
ACC_04408	360	scaffold181.1	1650705	1651064	+
ACC_04422	849	scaffold186.1	327031	327879	+
ACC_04445	312	scaffold19.1	625583	625894	+
ACC_04466	1179	scaffold191.1	140914	142092	+
ACC_04501	2238	scaffold196.1	856841	859078	+
ACC_04532	5373	scaffold70.1	133858	139230	+
ACC_04541	195	scaffold2.1	560558	560752	+

ACC_04571	939	scaffold2.1	2397221	2398159	+
ACC_04587	684	scaffold2.1	3817687	3818370	+
ACC_04602	273	scaffold20.1	1067884	1068156	+
ACC_04604	249	scaffold20.1	1155036	1155284	+
ACC_04645	1587	scaffold200.1	34467	36053	+
ACC_04665	318	scaffold204.1	192932	193249	+
ACC_04667	2265	scaffold204.1	217584	219848	+
ACC_04676	246	scaffold206.1	120564	120809	+
ACC_04692	1995	scaffold21.1	321572	323566	+
ACC_04699	495	scaffold210.1	27872	28366	+
ACC_04704	633	scaffold212.1	76864	77496	+
ACC_04760	504	scaffold22.1	999605	1000108	+
ACC_04780	420	scaffold220.1	185989	186408	+
ACC_04785	309	scaffold241.1	129172	129480	+
ACC_04800	351	scaffold223.1	396060	396410	+
ACC_04821	1338	scaffold39.1	429870	431207	+
ACC_04838	684	scaffold321.1	92090	92773	+
ACC_04845	2247	scaffold234.1	1731952	1734198	+
ACC_04894	2046	scaffold241.1	143472	145517	+
ACC_04898	1806	scaffold99.1	41138	42943	+
ACC_04909	483	scaffold298.1	97383	97865	+
ACC_04917	414	scaffold246.1	199712	200125	+
ACC_04950	378	scaffold25.1	21785	22162	+
ACC_04972	315	scaffold250.1	14469	14783	+
ACC_05013	291	scaffold26.1	325383	325673	+
ACC_05055	1575	scaffold266.1	379535	381109	+
ACC_05080	462	scaffold277.1	335677	336138	+
ACC_05081	300	scaffold278.1	7707	8006	+
ACC_05085	1131	scaffold28.1	30255	31385	+
ACC_05089	2079	scaffold28.1	325955	328033	+
ACC_05108	822	scaffold28.1	1331873	1332694	+
ACC_05111	1086	scaffold28.1	1672234	1673319	+
ACC_05118	2397	scaffold281.1	488127	490523	+
ACC_05178	813	scaffold85.1	1951120	1951932	+
ACC_05197	471	scaffold295.1	397515	397985	+
ACC_05231	306	scaffold302.1	168030	168335	+
ACC_05232	2604	scaffold303.1	28432	31035	+
ACC_05240	258	scaffold308.1	65663	65920	+
ACC_05243	417	scaffold309.1	8863	9279	+
ACC_05247	546	scaffold87.1	11074	11619	+
ACC_05312	972	scaffold31.1	1418521	1419492	+
ACC_05322	1911	scaffold31.1	1575404	1577314	+
ACC_05328	423	scaffold31.1	1902438	1902860	+
ACC_05367	216	scaffold311.1	64493	64708	+
ACC_05372	495	scaffold316.1	35895	36389	+
ACC_05385	297	scaffold32.1	336391	336687	+
ACC_05397	246	scaffold323.1	16021	16266	+
ACC_05404	330	scaffold33.1	225756	226085	+

ACC_05439	213	scaffold341.1	11509	11721	+
ACC_05442	318	scaffold349.1	4164	4481	+
ACC_05453	1065	scaffold45.1	578836	579900	+
ACC_05488	1497	scaffold35.1	2150870	2152366	+
ACC_05497	240	scaffold353.1	16513	16752	+
ACC_05498	216	scaffold357.1	19	234	+
ACC_05546	660	scaffold98.1	885081	885740	+
ACC_05593	336	scaffold4.1	32455	32790	+
ACC_05597	591	scaffold9.1	841594	842184	+
ACC_05618	393	scaffold9.1	3495529	3495921	+
ACC_05630	1098	scaffold6.1	1341801	1342898	+
ACC_05637	2805	scaffold14.1	796235	799039	+
ACC_05674	201	scaffold43.1	212531	212731	+
ACC_05748	327	scaffold46.1	492696	493022	+
ACC_05766	552	scaffold47.1	170091	170642	+
ACC_05800	582	scaffold48.1	1531005	1531586	+
ACC_05812	252	scaffold48.1	2172596	2172847	+
ACC_05833	1191	scaffold5.1	617426	618616	+
ACC_05842	402	scaffold5.1	1098983	1099384	+
ACC_05878	288	scaffold50.1	9334	9621	+
ACC_05894	513	scaffold501.1	148	660	+
ACC_05895	1533	scaffold51.1	320043	321575	+
ACC_05931	2061	scaffold55.1	21978	24038	+
ACC_05933	447	scaffold55.1	207901	208347	+
ACC_05934	840	scaffold55.1	263829	264668	+
ACC_05942	345	scaffold55.1	450884	451228	+
ACC_05943	396	scaffold55.1	458054	458449	+
ACC_05954	912	scaffold58.1	363763	364674	+
ACC_05979	639	scaffold279.1	14574	15212	+
ACC_06029	321	scaffold64.1	117870	118190	+
ACC_06044	1236	scaffold65.1	229230	230465	+
ACC_06053	228	scaffold66.1	254881	255108	+
ACC_06063	357	scaffold67.1	518402	518758	+
ACC_06064	366	scaffold67.1	581685	582050	+
ACC_06085	249	scaffold69.1	403491	403739	+
ACC_06132	1302	scaffold7.1	1762857	1764158	+
ACC_06159	264	scaffold70.1	379192	379455	+
ACC_06161	261	scaffold70.1	588532	588792	+
ACC_06172	216	scaffold70.1	1058179	1058394	+
ACC_06177	1128	scaffold70.1	1149261	1150388	+
ACC_06187	2118	scaffold70.1	3334326	3336443	+
ACC_06195	1131	scaffold138.1	80797	81927	+
ACC_06233	1848	scaffold208.1	356704	358551	+
ACC_06244	216	scaffold75.1	131560	131775	+
ACC_06252	1026	scaffold75.1	311497	312522	+
ACC_06253	300	scaffold75.1	317354	317653	+
ACC_06287	1005	scaffold78.1	499594	500598	+
ACC_06346	294	scaffold81.1	134234	134527	+

ACC_06365	225	scaffold82.1	1046836	1047060	+
ACC_06379	306	scaffold82.1	2201141	2201446	+
ACC_06388	258	scaffold83.1	1309596	1309853	+
ACC_06423	2802	scaffold86.1	5095	7896	+
ACC_06490	1827	scaffold9.1	4331007	4332833	+
ACC_06499	426	scaffold95.1	40736	41161	+
ACC_06501	3102	scaffold287.1	117562	120663	+
ACC_06528	222	scaffold98.1	1028112	1028333	+
ACC_06578	1164	scaffold20.1	661538	662701	-
ACC_06585	1059	scaffold10.1	1409690	1410748	-
ACC_06592	1329	scaffold10.1	2624970	2626298	-
ACC_06633	516	scaffold108.1	434259	434774	-
ACC_06637	327	scaffold108.1	608730	609056	-
ACC_06641	633	scaffold109.1	265957	266589	-
ACC_06708	1548	scaffold124.1	70700	72247	-
ACC_06711	1590	scaffold124.1	156025	157614	-
ACC_06714	609	scaffold125.1	513246	513854	-
ACC_06723	273	scaffold127.1	262227	262499	-
ACC_06767	441	scaffold269.1	163570	164010	-
ACC_06776	579	scaffold142.1	313337	313915	-
ACC_06782	477	scaffold251.1	225548	226024	-
ACC_06787	480	scaffold138.1	343161	343640	-
ACC_06849	246	scaffold19.1	1630823	1631068	-
ACC_06884	414	scaffold16.1	362078	362491	-
ACC_06923	270	scaffold165.1	280365	280634	-
ACC_06958	465	scaffold170.1	1384446	1384910	-
ACC_07001	267	scaffold178.1	1860292	1860558	-
ACC_07031	300	scaffold181.1	658285	658584	-
ACC_07049	498	scaffold123.1	209576	210073	-
ACC_07060	366	scaffold19.1	1167244	1167609	-
ACC_07097	1254	scaffold2.1	2238764	2240017	-
ACC_07111	405	scaffold70.1	4139758	4140162	-
ACC_07128	1416	scaffold20.1	2757335	2758750	-
ACC_07183	2022	scaffold220.1	77247	79268	-
ACC_07264	570	scaffold271.1	229	798	-
ACC_07275	1671	scaffold254.1	93440	95110	-
ACC_07276	114	scaffold330.1	130651	130764	-
ACC_07284	384	scaffold26.1	197393	197776	-
ACC_07290	465	scaffold26.1	1380618	1381082	-
ACC_07311	183	scaffold269.1	423938	424120	-
ACC_07312	369	scaffold27.1	19503	19871	-
ACC_07374	234	scaffold290.1	83084	83317	-
ACC_07429	1311	scaffold87.1	1332892	1334202	-
ACC_07460	3522	scaffold28.1	1055259	1058780	-
ACC_07467	270	scaffold323.1	54652	54921	-
ACC_07485	207	scaffold341.1	24139	24345	-
ACC_07486	33	scaffold341.1	25983	26015	-
ACC_07540	324	scaffold127.1	912772	913095	-

ACC_07546	279	scaffold98.1	893902	894180	-
ACC_07618	309	scaffold692.1	4270	4578	-
ACC_07627	432	scaffold22.1	17296	17727	-
ACC_07635	1989	scaffold43.1	639554	641542	-
ACC_07651	270	scaffold170.1	897922	898191	-
ACC_07689	1281	scaffold48.1	1521108	1522388	-
ACC_07713	1902	scaffold126.1	305104	307005	-
ACC_07718	438	scaffold126.1	752159	752596	-
ACC_07761	2400	scaffold101.1	391966	394365	-
ACC_07796	399	scaffold103.1	477703	478101	-
ACC_07804	1212	scaffold25.1	6590	7801	-
ACC_07839	54	scaffold66.1	684035	684088	-
ACC_07876	1155	scaffold31.1	585092	586246	-
ACC_07903	456	scaffold31.1	2999983	3000438	-
ACC_07943	213	scaffold71.1	816166	816378	-
ACC_07963	261	scaffold758.1	269	529	-
ACC_07979	399	scaffold96.1	804202	804600	-
ACC_07989	1725	scaffold78.1	2178549	2180273	-
ACC_07994	795	scaffold79.1	147712	148506	-
ACC_08005	876	scaffold43.1	798837	799712	-
ACC_08015	486	scaffold8.1	1333046	1333531	-
ACC_08065	1809	scaffold85.1	1241376	1243184	-
ACC_08071	243	scaffold85.1	1932814	1933056	-
ACC_08153	1995	scaffold1.1	65148	67142	+
ACC_08165	198	scaffold1.1	2400150	2400347	+
ACC_08166	1494	scaffold8.1	3178649	3180142	+
ACC_08174	1824	scaffold20.1	251541	253364	+
ACC_08179	1290	scaffold10.1	836720	838009	+
ACC_08180	1494	scaffold10.1	839434	840927	+
ACC_08193	7383	scaffold238.1	200379	207761	+
ACC_08208	156	scaffold104.1	769793	769948	+
ACC_08211	432	scaffold140.1	23069	23500	+
ACC_08225	342	scaffold108.1	2237007	2237348	+
ACC_08229	1269	scaffold109.1	1956441	1957709	+
ACC_08230	237	scaffold109.1	2145205	2145441	+
ACC_08272	432	scaffold118.1	414088	414519	+
ACC_08282	855	scaffold124.1	821622	822476	+
ACC_08290	2388	scaffold127.1	292261	294648	+
ACC_08297	282	scaffold129.1	322683	322964	+
ACC_08315	351	scaffold107.1	277647	277997	+
ACC_08328	465	scaffold136.1	92810	93274	+
ACC_08338	1569	scaffold138.1	424941	426509	+
ACC_08342	807	scaffold95.1	77123	77929	+
ACC_08356	627	scaffold10.1	2418620	2419246	+
ACC_08365	453	scaffold116.1	121247	121699	+
ACC_08366	1602	scaffold116.1	122887	124488	+
ACC_08401	267	scaffold154.1	12238	12504	+
ACC_08439	837	scaffold164.1	85898	86734	+

ACC_08445	1446	scaffold164.1	1143233	1144678	+
ACC_08449	1059	scaffold179.1	49670	50728	+
ACC_08454	666	scaffold166.1	661771	662436	+
ACC_08466	483	scaffold68.1	327558	328040	+
ACC_08494	1155	scaffold1.1	2382325	2383479	+
ACC_08506	1833	scaffold173.1	485080	486912	+
ACC_08510	1260	scaffold174.1	822823	824082	+
ACC_08524	1023	scaffold175.1	963100	964122	+
ACC_08550	288	scaffold82.1	2358177	2358464	+
ACC_08553	735	scaffold181.1	353432	354166	+
ACC_08646	717	scaffold20.1	2034375	2035091	+
ACC_08688	702	scaffold22.1	286060	286761	+
ACC_08689	1407	scaffold22.1	301648	303054	+
ACC_08717	1695	scaffold39.1	771729	773423	+
ACC_08734	276	scaffold24.1	353727	354002	+
ACC_08755	972	scaffold246.1	308376	309347	+
ACC_08764	417	scaffold25.1	450068	450484	+
ACC_08772	372	scaffold7.1	2123651	2124022	+
ACC_08792	909	scaffold26.1	282308	283216	-
ACC_08803	249	scaffold264.1	193348	193596	+
ACC_08822	1524	scaffold275.1	39222	40745	+
ACC_08868	291	scaffold2.1	1094734	1095024	+
ACC_08888	453	scaffold31.1	166472	166924	+
ACC_08893	828	scaffold31.1	490089	490916	+
ACC_08902	1197	scaffold31.1	1971242	1972438	+
ACC_08915	261	scaffold314.1	70420	70680	+
ACC_08919	1011	scaffold28.1	115682	116692	+
ACC_08951	258	scaffold347.1	13111	13368	+
ACC_08966	1056	scaffold36.1	1259920	1260975	+
ACC_09012	393	scaffold9.1	3612582	3612974	+
ACC_09020	1176	scaffold6.1	1337490	1338665	+
ACC_09029	993	scaffold42.1	650751	651743	+
ACC_09045	198	scaffold22.1	997409	997606	+
ACC_09046	333	scaffold43.1	1486325	1486657	+
ACC_09047	627	scaffold43.1	1831181	1831807	+
ACC_09048	189	scaffold43.1	1833298	1833486	+
ACC_09052	1056	scaffold43.1	3240455	3241510	+
ACC_09053	363	scaffold170.1	478538	478900	+
ACC_09057	1614	scaffold83.1	130751	132364	+
ACC_09083	5175	scaffold48.1	575710	580884	+
ACC_09120	438	scaffold117.1	934107	934544	+
ACC_09128	240	scaffold522.1	94	333	+
ACC_09158	303	scaffold101.1	223293	223595	+
ACC_09173	882	scaffold35.1	1812872	1813753	+
ACC_09177	978	scaffold35.1	2084466	2085443	+
ACC_09229	1599	scaffold124.1	20189	21787	+
ACC_09232	1275	scaffold124.1	705656	706930	+
ACC_09236	555	scaffold68.1	289751	290305	+

ACC_09265	1332	scaffold31.1	1105334	1106665	+
ACC_09290	585	scaffold70.1	1220136	1220720	+
ACC_09303	432	scaffold138.1	458511	458942	+
ACC_09340	531	scaffold96.1	282023	282553	+
ACC_09373	717	scaffold60.1	566112	566828	+
ACC_09380	267	scaffold82.1	1052948	1053214	+
ACC_09400	1125	scaffold15.1	109967	111091	+
ACC_09427	2550	scaffold9.1	4246507	4249056	+
ACC_09653	200	scaffold166.1	1598605	1598804	-
ACC_09833	285	scaffold28.1	1096772	1097056	+
ACC_09842	2772	scaffold72.1	1433107	1435878	+
ACC_09863	405	scaffold31.1	976649	977053	-
ACC_09873	1077	scaffold105.1	841890	842966	-
ACC_09882	1884	scaffold135.1	146541	148424	+
ACC_09892	813	scaffold174.1	2215921	2216733	-
ACC_09893	681	scaffold19.1	423408	424088	-
ACC_09895	843	scaffold72.1	70988	71830	-
ACC_09908	1119	scaffold13.1	1524058	1525176	-
ACC_09909	696	scaffold20.1	606534	607229	+
ACC_09911	240	scaffold171.1	145697	145936	-
ACC_09916	105	scaffold45.1	154039	154143	+
ACC_09954	981	scaffold58.1	589081	590061	-
ACC_09960	216	scaffold290.1	509512	509727	+
ACC_09976	117	scaffold210.1	90291	90407	+
ACC_09988	1296	scaffold17.1	865349	866644	+
ACC_09993	309	scaffold2.1	1302883	1303191	-
ACC_09995	1164	scaffold10.1	1088554	1089717	-
ACC_09999	213	scaffold294.1	109832	110044	+
ACC_10017	1095	scaffold250.1	422304	423398	-
ACC_10067	201	scaffold96.1	365658	365858	-
ACC_10073	264	scaffold181.1	477	740	+
ACC_10112	282	scaffold15.1	305883	306164	-
ACC_10115	216	scaffold19.1	1409083	1409298	-
ACC_10119	537	scaffold79.1	265326	265862	+
ACC_10122	1023	scaffold178.1	1781872	1782894	-
ACC_10123	450	scaffold192.1	464119	464568	-
ACC_10124	1524	scaffold13.1	1479746	1481269	-
ACC_10127	318	scaffold641.1	319	636	-
ACC_10128	459	scaffold87.1	1905745	1906203	-
ACC_10132	327	scaffold98.1	671863	672189	+
ACC_10133	654	scaffold10.1	2297200	2297853	+
ACC_10138	234	scaffold128.1	403784	404017	-
ACC_10140	1041	scaffold13.1	1517988	1519028	-
ACC_10149	264	scaffold22.1	1078949	1079212	+
ACC_10159	183	scaffold6.1	1548475	1548657	-
ACC_10172	471	scaffold208.1	439755	440225	-

Supplementary Table 5 Expression profile of *A.cerana* genes

(NEB) Newly Emerged Bees; (NB) Nurse Bees; (GB) Guard Bees; (FB) Foraging Bees; (DB) Dancing bees; (NC) Controls W/O Mites; (SGW) Self-grooming Workers; (BSW) Body-shaking Workers; (VQ) Virgin Queens; (FQ) Fertilized Queens; (WA) Worker Antenna; (DA) Drone Antenna; (L2) 2-day Larvae; (L2) 2-day Larvae; (QL2) 2-day Queen Larvae; (QL4) 4-day Queen Larvae

Gene	Product	KO	KOG	NEW	NB	GB	FB	DB	NC	SGW	BSW	VQ	FQ	WA	DA	L2	L4	QL2	QL4
ACC_00001	WD repeat-containing protein 18-like isoform 1	K14829	KOG0646	76	62	64	125	78	7	15	14	82	57	27	41	289	425	163	73
ACC_00002	40S ribosomal protein S15-like	K02958	KOG0898	1446	938	471	1949	857	221	394	315	734	610	1414	1438	4462	4833	1187	662
ACC_00003	lipoma HMGIC fusion partner homolog		KOG4026	59	26	39	59	13	3	1	0	29	54	7	19	4	0	11	1
ACC_00004	PRADC1-like protein-like		KOG3920	167	148	91	399	327	27	25	39	322	283	127	609	676	719	67	9
ACC_00005	general transcription factor IIH subunit 1-like	K03141	KOG2074	141	96	72	105	87	12	16	14	165	127	37	141	333	327	79	37
ACC_00006	double-stranded RNA-specific editase Adar-like	K13194	KOG2777	370	222	239	391	242	48	96	112	425	288	90	68	146	145	103	42
ACC_00007	phosphatidylinositol transfer protein alpha isoform		KOG3668	1213	432	376	599	553	59	80	81	808	575	1645	9622	1728	1724	243	71
ACC_00008	solute carrier family 25 member 44-like	K15121	KOG0765	125	74	89	204	163	12	13	24	431	234	109	426	529	530	83	17
ACC_00009	pre-mRNA-splicing factor SPF27	K12861	KOG3096	200	175	207	342	249	39	75	72	288	137	171	283	331	618	277	320
ACC_00010	conserved hypothetical protein			8	2	7	24	18	0	4	2	3	7	1	0	13	27	17	2
ACC_00011	hypothetical protein			0	0	2	0	0	0	0	0	0	0	0	0	0	1	1	2
ACC_00012	conserved hypothetical protein		KOG0976	10	5	7	4	3	1	2	0	5	11	0	1	2	3	6	11
ACC_00013	ATP-dependent RNA helicase abstrakt isoform 1	K13116	KOG0341	269	158	179	330	244	65	111	129	338	174	179	186	220	350	284	196
ACC_00014	conserved hypothetical protein			5	5	10	18	13	0	1	1	50	34	50	209	8	3	1	0
ACC_00015	peroxisomal N(1)-acetyl-spermine/spermidine oxidase-like i		KOG0685	1235	888	1269	2499	1809	47	78	114	2134	3349	28	81	862	326	22	15
ACC_00016	probable palmitoyltransferase ZDHHC16-like		KOG1313	200	160	140	295	249	28	35	41	339	118	139	117	211	234	25	12
ACC_00017	mothers against decapentaplegic homolog 4 isofor	K04501	KOG3701	272	113	96	102	68	30	37	36	428	351	99	246	334	380	87	36
ACC_00018	conserved hypothetical protein			10	9	13	16	32	0	6	5	11	4	11	19	9	7	1	0
ACC_00019	conserved hypothetical protein		KOG3229	2201	796	887	2875	986	152	255	371	1537	622	387	825	250	326	117	82
ACC_00020	ubiquitin-conjugating enzyme E2 R2-like	K02207	KOG0425	519	201	217	470	154	65	74	107	786	399	127	481	453	546	244	168
ACC_00021	DDB1- and CUL4-associated factor 10 homolog	K11802	KOG0266	143	68	96	114	79	10	9	15	222	142	34	112	157	161	58	30
ACC_00022	DOMON domain-containing protein CG14681-like		KOG1584	40	21	18	25	20	1	3	3	56	35	15	148	247	540	74	85
ACC_00023	sulfotransferase 4A1-like		KOG1584	3	0	3	8	5	1	2	3	20	0	9	1	27	13	5	1
ACC_00024	dihydroorotate dehydrogenase, mitochondrial-like	K00254	KOG1436	64	47	56	136	93	4	12	14	105	81	99	92	198	262	29	18
ACC_00025	ADP-ribosylation factor-like protein 3-like	K07944	KOG0074	51	90	43	64	51	62	79	126	110	36	440	1056	99	117	24	23
ACC_00026	conserved hypothetical protein			22	18	27	33	20	4	8	10	18	11	18	5	1	0	0	2
ACC_00027	conserved hypothetical protein		KOG0954	1470	1329	1147	1722	990	386	709	761	1856	1434	929	192	424	620	2173	1378
ACC_00028	tyrosine-protein kinase Abl-like	K06619	KOG4278	1101	861	785	1181	611	93	143	200	1348	1288	315	390	733	813	215	82
ACC_00029	dynamin-binding protein-like		KOG3519	361	220	196	147	177	17	40	39	454	328	83	154	374	229	40	22
ACC_00030	zinc transporter 9-like	K14696	KOG2802	407	265	212	374	364	95	177	154	800	379	278	586	456	393	156	88
ACC_00031	conserved hypothetical protein			168	134	118	687	306	69	34	70	148	146	13	121	20	31	9	3
ACC_00032	niemann-Pick C1 protein-like		KOG1934	220	117	123	268	169	23	35	45	606	433	343	594	227	206	47	36
ACC_00033	vesicle-associated membrane protein 7	K08515	KOG0859	191	111	140	326	223	42	50	57	379	149	157	437	221	163	41	5
ACC_00034	conserved hypothetical protein		KOG0162	92	17	21	51	17	15	23	32	272	202	33	242	79	126	92	344
ACC_00035	LOW QUALITY PROTEIN		KOG3882	1435	335	486	796	247	14	21	36	489	340	100	884	68	100	164	122
ACC_00036	UPF0670 protein CG4666-like		KOG4366	278	114	90	467	123	17	25	25	355	241	16	27	60	65	54	20
ACC_00037	conserved hypothetical protein	K16717	KOG0531	462	241	230	476	344	32	51	66	347	270	161	150	245	237	91	35
ACC_00038	protein phosphatase methylesterase 1-like isoform	K13617	KOG2564	188	135	165	267	172	19	16	28	404	235	152	417	287	402	43	24
ACC_00039	conserved hypothetical protein			9	3	5	17	1	1	1	2	21	5	17	21	35	107	29	29
ACC_00040	multidrug resistance protein homolog 49-like	K05658	KOG0055	240	109	79	211	27	26	22	22	1575	1924	320	425	354	367	896	114
ACC_00041	protein Kr-h2-like		KOG4002	716	416	334	606	548	54	71	74	1248	642	820	2797	936	1117	60	15
ACC_00042	RNA polymerase-associated protein Rtf1	K15178	KOG2402	1216	696	533	795	921	320	734	757	777	523	581	411	504	775	2867	2118
ACC_00043	alpha1,3-fucosyltransferase C	K14464	KOG2619	49	99	44	136	59	88	70	67	210	189	198	180	95	113	146	9
ACC_00044	conserved hypothetical protein			211	164	184	293	372	19	37	41	172	104	260	305	209	262	75	23
ACC_00045	COP9 signalosome complex subunit 2	K12176	KOG1464	379	266	290	498	554	17	43	62	341	283	243	452	700	800	76	45
ACC_00046	3-hydroxy-3-methylglutaryl-coenzyme A reductase	K00021	KOG2480	608	366	383	826	609	20	47	48	876	593	189	381	617	637	114	54
ACC_00047	UPF0402 protein CG32590-like		KOG4523	41	18	27	27	49	4	4	10	38	19	33	69	36	30	5	2
ACC_00048	actin-related protein 2-like isoform 1		KOG0677	250	141	115	368	407	24	69	64	287	240	179	391	523	375	72	30
ACC_00049	alkaline ceramidase-like isoform 1	K01441	KOG2329	69	45	73	137	135	4	2	17	59	35	35	58	56	75	6	2
ACC_00050	26S protease regulatory subunit S10B	K03064	KOG0651	280	194	230	494	460	33	47	67	1067	578	592	1179	1543	2106	149	118
ACC_00051	conserved hypothetical protein			315	130	120	295	439	48	110	128	228	135	257	373	253	379	282	285
ACC_00052	U6 snRNA-associated Sm-like protein LSm6	K12625	KOG1783	48	30	32	100	101	0	0	8	23	10	53	43	96	170	22	16
ACC_00053	mitochondrial import inner membrane translocase subunit T		KOG2832	550	366	320	677	487	98	151	157	426	349	264	380	1612	2703	1206	932
ACC_00054	CD151 antigen isoform 1		KOG3882	514	178	202	849	336	83	62	81	1356	603	937	1976	261	268	101	47

ACC_00055	actin-like protein 6A-like isoform 2		KOG0679	75	61	57	134	31	2	10	7	29	17	2	1	2	5	24	13
ACC_00056	hypothetical protein			264	158	265	2327	491	5	10	7	151	65	231	110	543	632	277	54
ACC_00057	fatty acid-binding protein, muscle-like isoform 2		KOG4015	1254	886	885	3302	2179	136	207	325	3712	667	975	5671	280	332	79	58
ACC_00058	probable E3 ubiquitin-protein ligase TRIP12-like			101	74	56	31	11	7	9	11	176	228	20	57	132	116	15	2
ACC_00059	zinc finger protein Xfin-like		KOG2462	312	247	214	229	243	28	69	57	212	169	123	94	134	99	43	24
ACC_00060	conserved hypothetical protein		KOG4646	198	124	135	327	377	6	13	15	165	142	181	447	173	209	34	11
ACC_00061	conserved hypothetical protein			178	122	92	153	187	14	63	46	149	204	188	534	191	189	63	22
ACC_00062	39S ribosomal protein L9, mitochondrial-like	K02939	KOG4607	355	264	301	429	389	33	62	94	270	231	142	558	1011	968	201	102
ACC_00063	autophagy-specific gene 6	K08334	KOG2751	225	152	167	287	230	53	54	61	286	133	139	197	155	164	74	47
ACC_00064	type-1 angiotensin II receptor-associated protein-like			180	136	99	293	211	32	46	67	356	125	260	467	140	126	16	11
ACC_00065	conserved hypothetical protein		KOG2242	1439	1246	1268	1322	906	306	545	645	2221	1558	847	1108	1280	1991	2152	1726
ACC_00066	synaptotagmin-14		KOG1028	260	196	129	180	106	8	9	5	252	168	50	88	12	17	3	6
ACC_00067	JNK1/MAPK8-associated membrane protein-like		KOG3744	211	183	243	263	277	23	40	43	275	104	190	280	158	217	47	11
ACC_00068	RNA-binding protein Nova-2-like	K14944	KOG2191	936	579	446	637	265	107	141	164	1119	1765	190	454	511	369	179	82
ACC_00069	conserved hypothetical protein			191	90	98	213	176	20	25	27	449	317	165	440	1630	254	88	17
ACC_00070	probable ATP-dependent RNA helicase spindle-E		KOG0920	369	276	263	541	415	26	63	57	523	823	115	231	377	454	81	34
ACC_00071	sperm-associated antigen 6-like isoform 1		KOG0166	43	22	33	41	28	4	3	7	26	14	9	16	14	10	6	0
ACC_00072	L-asparaginase-like	K13278	KOG0503	326	151	178	310	235	32	37	48	380	293	132	256	445	530	83	25
ACC_00073	n-acetylated-alpha-linked acidic dipeptidase-like protein-like		KOG2195	401	192	183	211	301	20	27	37	277	138	159	267	181	185	35	5
ACC_00074	zinc finger protein 642-like isoform 2		KOG2462	35	28	32	81	64	7	7	4	49	31	34	30	39	98	47	11
ACC_00075	conserved hypothetical protein			252	200	192	230	350	27	38	36	436	262	188	726	619	676	56	16
ACC_00076	protein RER1-like		KOG1688	330	182	160	464	349	86	150	137	365	251	192	510	732	637	342	260
ACC_00077	e3 ubiquitin-protein ligase UBR3-like	K11978	KOG1139	402	243	232	223	171	29	57	58	1155	1232	128	303	459	312	122	52
ACC_00078	conserved hypothetical protein	K14837	KOG1832	2277	1069	612	649	791	259	694	659	676	665	534	332	6847	14526	27950	30866
ACC_00079	probable nuclear transport factor 2-like isoform 3		KOG2104	255	259	178	584	394	45	65	72	313	269	210	603	972	1349	284	100
ACC_00080	probable dynactin subunit 2	K10424	KOG3958	406	240	270	404	389	36	64	55	451	241	225	555	561	472	83	48
ACC_00081	CAAX prenyl protease 2-like	K08658	KOG4130	96	61	47	60	50	21	16	25	278	237	65	368	400	236	36	16
ACC_00082	conserved hypothetical protein			125	104	94	163	120	2	3	4	62	29	22	20	9	16	0	0
ACC_00083	flavin reductase-like		KOG1203	308	203	182	429	424	24	44	40	853	437	667	1925	1169	1126	117	60
ACC_00084	proteasome activator complex subunit 4-like	K06699	KOG1851	1738	1117	925	1641	1434	242	569	621	2827	3410	1647	2173	3409	3841	1165	639
ACC_00085	conserved hypothetical protein			69	32	62	42	36	0	2	2	27	65	70	266	216	155	6	1
ACC_00086	conserved hypothetical protein			65	44	48	68	85	7	9	10	85	40	60	138	156	182	10	6
ACC_00087	ras-related protein Rab-43	K07976	KOG0084	65	31	24	61	67	8	13	9	153	96	45	240	95	86	15	5
ACC_00088	complement component 1 Q subcomponent-bindir	K15414	KOG4024	852	432	364	942	1093	70	88	128	838	847	490	1656	5321	8911	580	358
ACC_00089	pre-mRNA-splicing factor 38A-like	K12849	KOG2889	121	110	98	90	95	18	38	30	91	64	52	84	249	386	145	163
ACC_00090	serine/threonine-protein phosphatase 4 regulatory subunit :		KOG2175	463	394	336	433	272	42	46	69	734	713	185	339	543	761	167	31
ACC_00091	e3 ubiquitin-protein ligase synoviolin A-like isoform 1		KOG0802	922	859	764	1093	636	171	123	225	2128	1543	671	1443	1989	1590	180	45
ACC_00092	conserved hypothetical protein		KOG2200	284	224	183	265	300	37	108	105	183	161	150	151	243	310	179	87
ACC_00093	DNA replication licensing factor mcm5	K02209	KOG0481	668	518	592	572	464	58	111	109	1108	286	481	355	572	775	191	81
ACC_00094	ADP-ribosylation factor-like protein 16-like		KOG0072	28	17	26	53	40	3	5	3	35	8	126	398	8	3	3	0
ACC_00095	conserved hypothetical protein		KOG4774	52	45	35	93	67	4	10	12	39	26	64	109	108	116	10	4
ACC_00096	conserved hypothetical protein			316	142	141	733	335	13	29	31	285	85	694	516	621	617	59	37
ACC_00097	catalase isoform 1	K08876	KOG1243	2547	1734	2029	1820	1140	352	618	622	2901	994	1061	552	1035	1061	386	183
ACC_00098	splicing factor 3B subunit 2-like	K12829	KOG2330	2026	1125	955	1749	1546	375	730	782	2205	1121	1055	1044	1624	2584	2890	2307
ACC_00099	hairless		KOG3554	182	235	205	195	90	25	29	35	233	172	63	95	198	215	94	18
ACC_00100	adenomatous polyposis coli protein-like		KOG2122	148	103	77	79	77	5	17	21	249	180	40	182	305	183	40	23
ACC_00101	AN1-type zinc finger protein 5-like isoform 1		KOG3173	982	618	514	1071	516	461	664	898	1376	703	668	540	585	710	1365	959
ACC_00102	coiled-coil domain-containing protein 135-like			28	21	33	24	18	2	5	12	16	6	0	3	5	11	8	0
ACC_00103	autophagy-related protein 2 homolog A		KOG2993	553	475	477	595	386	117	130	156	1244	927	267	265	620	454	78	16
ACC_00104	conserved hypothetical protein			33	15	20	35	38	5	4	4	72	56	20	61	29	24	2	0
ACC_00105	dimethyladenosine transferase 2, mitochondrial			162	157	113	204	333	7	37	20	40	104	147	259	141	99	39	13
ACC_00106	luciferin 4-monooxygenase-like		KOG1176	606	415	394	543	474	18	39	49	323	201	153	239	221	252	54	21
ACC_00107	a-kinase anchor protein 10, mitochondrial-like	K16526	KOG3590	169	96	63	106	141	7	25	27	372	185	72	197	174	145	17	13
ACC_00108	rho GTPase-activating protein 100F-like isoform 1		KOG1452	498	468	486	428	195	33	40	40	330	284	122	160	71	66	33	10
ACC_00109	SPRY domain-containing SOCS box protein 3-like	K10345	KOG3953	102	90	80	152	120	18	32	30	188	91	125	173	173	253	65	30
ACC_00110	conserved hypothetical protein	K11831		289	175	207	377	177	36	36	45	571	354	122	214	255	321	188	42
ACC_00111	voltage-dependent calcium channel subunit alpha-2/delta-3-		KOG2353	121	278	167	298	116	113	81	208	347	282	16	50	65	51	39	25

ACC_00112	f-box/WD repeat-containing protein 9-like isoform	K10265	KOG0274	203	125	133	346	280	28	31	45	392	213	207	430	194	224	36	12
ACC_00113	riboflavin transporter 2-A-like	K14620	KOG4255	123	63	59	78	77	18	27	32	305	369	86	451	552	327	23	1
ACC_00114	conserved hypothetical protein			75	96	111	121	10	3	5	7	141	144	15	14	5	4	22	8
ACC_00115	transmembrane emp24 domain-containing protein bai		KOG1691	364	343	308	713	646	87	97	212	430	218	485	888	812	883	107	43
ACC_00116	hypothetical protein			0	0	0	0	1	0	0	0	0	5	0	0	1	0	0	1
ACC_00117	protein Dr1-like		KOG0871	127	51	73	128	147	13	5	12	178	91	69	254	240	347	38	8
ACC_00118	conserved hypothetical protein			200	118	107	204	130	17	29	34	593	234	92	220	185	191	41	14
ACC_00119	alkyldihydroxyacetonephosphate synthase-like	K00803	KOG1233	416	490	371	535	393	79	111	103	4735	4083	532	882	184	355	88	33
ACC_00120	conserved hypothetical protein			229	150	104	116	96	14	7	16	362	212	117	343	168	88	18	4
ACC_00121	neurogenin-3-like		KOG3898	174	156	101	84	42	75	240	304	60	48	25	27	19	14	203	153
ACC_00122	methionine aminopeptidase 1-like	K01265	KOG2738	308	179	158	554	328	46	57	68	1199	654	538	988	779	898	286	112
ACC_00123	ankyrin repeat domain-containing protein 6-like		KOG0508	16	11	6	25	14	0	5	2	28	35	33	28	11	19	34	29
ACC_00124	LOW QUALITY PROTEIN	K14861	KOG1791	1265	740	539	768	1028	48	126	91	567	1004	435	354	1304	1277	314	164
ACC_00125	f-box/LRR-repeat protein 4	K10270	KOG4341	242	136	112	205	310	13	26	36	235	186	118	225	358	272	29	13
ACC_00126	mitochondrial ribonuclease P protein 1 homolog		KOG2967	615	331	299	790	965	31	126	138	567	400	430	806	1061	1519	861	691
ACC_00127	PITH domain-containing protein GA19395-like		KOG1730	231	202	172	234	224	31	42	51	186	116	130	303	518	602	56	46
ACC_00128	protein msta, isoform A-like		KOG2084	29	35	35	58	69	2	3	9	55	41	80	101	23	14	6	2
ACC_00129	ATP synthase mitochondrial F1 complex assembly f	K07556	KOG3015	143	99	120	174	244	9	19	31	130	82	166	195	353	439	25	4
ACC_00130	LOW QUALITY PROTEIN		KOG2970	147	74	104	150	249	11	13	6	166	109	96	174	376	234	15	4
ACC_00131	COP9 signalosome complex subunit 5 isoform 2	K09613	KOG1554	430	285	311	611	464	46	60	65	785	497	469	840	718	936	73	35
ACC_00132	tRNA (adenine-N(1)-)-methyltransferase catalytic s	K07442	KOG2915	264	189	216	372	416	23	51	51	243	151	313	261	272	312	37	22
ACC_00133	tetratricopeptide repeat protein 7B-like		KOG4162	827	460	536	1207	948	83	110	123	1251	795	857	1510	619	657	95	25
ACC_00134	kaptin-like			149	90	120	177	260	19	33	37	163	158	124	215	239	218	17	3
ACC_00135	Golgi apparatus protein 1-like isoform 1	K06816	KOG3648	593	358	255	447	399	35	93	113	1118	1166	316	754	884	665	100	40
ACC_00136	protein msta, isoform A-like		KOG2084	2	2	4	6	5	1	3	1	21	33	2	3	1	0	0	0
ACC_00137	protein msta, isoform B-like		KOG2084	30	26	24	97	65	12	12	12	585	224	57	70	31	13	1	2
ACC_00138	COP9 signalosome complex subunit 4	K12178	KOG1497	361	244	204	393	388	43	51	61	511	425	160	670	973	824	93	37
ACC_00139	conserved hypothetical protein		KOG4400	979	624	563	572	770	133	346	361	597	411	537	570	837	1228	1039	739
ACC_00140	hypothetical protein			91	43	57	206	134	9	16	18	86	64	4	2	0	9	2	0
ACC_00141	CUGBP Elav-like family member 4-like	K13207	KOG0146	678	620	765	1978	403	70	126	173	231	124	213	34	0	10	180	56
ACC_00142	long-chain fatty acid transport protein 4-like	K08745	KOG1179	1069	603	845	3287	1606	97	118	175	1524	1264	164	270	255	181	80	45
ACC_00143	longitudinals lacking protein-like		KOG4441	109	87	59	47	45	10	11	14	475	492	39	313	590	500	64	16
ACC_00144	protein RMD5 homolog A-like		KOG2817	132	96	92	214	177	13	12	19	335	230	61	350	222	176	22	20
ACC_00145	v-type proton ATPase subunit e 2-like	K02153	KOG3500	495	470	453	863	1025	50	70	112	798	260	567	1815	897	717	29	17
ACC_00146	conserved hypothetical protein		KOG3681	97	102	73	182	182	10	15	21	158	101	66	52	102	131	26	4
ACC_00147	transferrin 1 precursor			2230	6090	8516	29534	10894	2144	2201	2644	64100	37686	2186	5053	1328	303	196	344
ACC_00148	semaphorin-5A	K06841	KOG3611	194	139	152	452	113	24	25	30	331	290	44	72	156	164	271	49
ACC_00149	ornithine aminotransferase, mitochondrial-like	K00819	KOG1402	30	24	20	22	19	2	6	5	34	14	6	4	3898	9672	673	72
ACC_00150	ornithine aminotransferase, mitochondrial-like	K00819	KOG1402	133	47	55	80	76	4	3	4	32	33	7	17	50	28	6	1
ACC_00151	conserved hypothetical protein		KOG4595	119	87	102	69	82	2	7	6	298	179	49	336	178	213	6	5
ACC_00152	conserved hypothetical protein			2352	1116	1431	2633	1860	80	132	211	970	364	112	293	175	377	191	76
ACC_00153	protein PTCD3 homolog, mitochondrial-like		KOG4422	422	207	228	227	324	10	29	19	168	250	104	498	1025	746	64	26
ACC_00154	conserved hypothetical protein	K13711		47	44	42	153	75	17	21	12	66	33	33	7	15	29	45	18
ACC_00155	conserved hypothetical protein	K13711	KOG2381	322	240	268	521	376	42	44	73	708	541	233	564	695	674	149	23
ACC_00156	protein farnesyltransferase subunit beta-like	K05954	KOG0365	342	130	129	290	266	34	36	53	500	201	159	488	633	838	102	29
ACC_00157	putative RNA-binding protein 11-like isoform 1	K13188	KOG4454	141	111	93	192	182	8	7	17	123	100	77	190	149	199	17	7
ACC_00158	flavin-containing monooxygenase FMO GS-OX-like 2-like		KOG1399	108	28	17	14	12	28	29	52	138	124	30	31	74	37	5	6
ACC_00159	conserved hypothetical protein			27	23	32	47	61	2	1	1	33	27	13	52	52	55	6	2
ACC_00160	serine/threonine-protein kinase grp isoform 2	K02216	KOG0583	129	107	71	131	188	18	30	38	200	101	115	156	647	1292	217	83
ACC_00161	splicing factor 3A subunit 3	K12827	KOG2636	239	115	101	259	235	20	31	47	420	164	172	402	473	698	109	86
ACC_00162	multidrug resistance-associated protein 4-like		KOG0054	333	236	244	389	435	50	67	81	1128	810	711	849	1612	1427	104	21
ACC_00163	nuclear inhibitor of protein phosphatase 1	K13216	KOG1880	282	261	269	474	301	37	51	64	262	188	160	334	217	247	83	39
ACC_00164	conserved hypothetical protein		KOG3607	68	19	31	54	16	5	5	3	132	153	41	74	28	27	33	18
ACC_00165	hepatic leukemia factor	K09057	KOG3119	776	462	473	617	175	335	584	709	1462	1271	1334	1036	99	216	1666	1322
ACC_00166	conserved hypothetical protein			164	120	140	254	175	11	23	23	246	124	100	201	208	263	62	14
ACC_00167	conserved hypothetical protein			31	46	44	24	31	1	4	2	60	42	64	152	221	216	6	4
ACC_00168	cell cycle regulator Mat89Bb homolog		KOG3711	213	159	144	312	209	22	41	40	309	209	157	399	544	589	185	37

ACC_00169	transmembrane and TPR repeat-containing protein CG4050-		KOG4626	1207	699	634	1364	1121	116	141	161	1221	561	487	633	590	572	152	69
ACC_00170	mannose-1-phosphate guanyltransferase alpha-A-l	K00966	KOG1460	269	181	177	355	279	20	36	34	297	298	139	428	455	530	55	17
ACC_00171	protein CutA homolog	K03926	KOG3338	514	306	329	592	359	27	51	82	227	158	147	532	355	282	110	78
ACC_00172	inositol hexakisphosphate and diphosphoinositol-pentakispl		KOG4029	9	3	6	12	3	0	2	1	7	2	0	2	2	3	8	2
ACC_00173	LOW QUALITY PROTEIN	K13024	KOG1057	852	518	465	680	536	67	98	140	2086	1604	329	565	594	333	92	54
ACC_00174	dolichyldiphosphatase 1-like	K07252	KOG3146	115	74	61	71	98	4	2	4	177	100	31	238	244	306	6	0
ACC_00175	NADH dehydrogenase	K03966	KOG4009	320	212	201	785	743	32	51	80	366	157	546	673	555	644	96	66
ACC_00176	iduronate 2-sulfatase-like	K01136	KOG3731	461	353	335	311	262	39	68	52	550	306	338	466	257	347	66	30
ACC_00177	conserved hypothetical protein		KOG3149	422	252	173	338	358	121	294	299	496	362	286	338	548	575	433	178
ACC_00178	RNA methyltransferase-like protein 1-like		KOG2506	270	124	110	414	450	27	80	88	167	126	178	222	454	668	489	376
ACC_00179	probable ATP-dependent RNA helicase DDX17-like	K13178	KOG0331	296	186	184	331	244	50	67	69	438	245	113	297	338	411	189	132
ACC_00180	endoplasmic reticulum mannosyl-oligosaccharide 1	K01230	KOG2431	427	267	351	541	523	25	40	37	502	472	160	434	520	351	33	4
ACC_00181	prefoldin subunit 5-like	K04797	KOG3048	70	64	51	178	167	13	18	14	136	54	124	155	271	410	79	47
ACC_00182	conserved hypothetical protein			569	299	245	284	249	60	127	144	324	363	147	127	179	139	138	26
ACC_00183	conserved hypothetical protein			8	2	5	6	4	2	4	1	17	5	5	2	8	4	1	4
ACC_00184	DNA primase small subunit-like	K02684	KOG2851	44	35	31	47	40	6	5	7	41	26	45	53	204	371	170	132
ACC_00185	transformation/transcription domain-associated pr	K08874	KOG0889	247	148	150	330	105	24	19	32	392	415	78	72	205	424	196	32
ACC_00186	cytochrome b-c1 complex subunit 8-like	K00418	KOG4116	540	539	635	886	486	33	68	73	264	70	455	527	527	615	128	57
ACC_00187	protein LSM12 homolog		KOG4401	412	366	389	534	156	40	54	58	370	328	153	532	580	840	693	229
ACC_00188	activating signal cointegrator 1 complex subunit 2-like		KOG4501	483	287	327	381	488	46	74	60	316	262	177	194	673	949	108	49
ACC_00189	conserved hypothetical protein			194	108	138	146	83	13	30	25	92	64	31	15	44	48	108	69
ACC_00190	conserved hypothetical protein		KOG0921	38	15	22	51	12	1	0	3	3	7	3	0	1	4	23	27
ACC_00191	ubiquitin carboxyl-terminal hydrolase 48-like	K11858	KOG1863	374	260	212	428	258	91	200	185	290	274	131	99	158	177	339	168
ACC_00192	DET1- and DDB1-associated protein 1-like	K11792	KOG4816	206	128	112	360	222	5	14	17	225	105	67	360	148	260	59	7
ACC_00193	adenine phosphoribosyltransferase	K00759	KOG1712	168	163	89	225	171	23	45	45	386	259	245	639	1153	2135	159	68
ACC_00194	slit homolog 1 protein-like		KOG4194	23	14	18	45	34	9	7	9	57	27	36	28	48	72	15	5
ACC_00195	probable leucyl-tRNA synthetase, mitochondrial-lik	K01869	KOG0435	346	196	206	329	297	17	44	39	442	224	214	372	1050	1132	149	44
ACC_00196	dual specificity protein phosphatase 3-like	K14165	KOG1716	132	102	101	232	298	12	12	21	306	160	123	292	206	110	9	3
ACC_00197	conserved hypothetical protein			238	135	132	485	316	17	7	14	65	66	8	68	6	12	3	1
ACC_00198	pallidin-like			175	148	126	182	170	36	82	94	154	96	121	193	188	249	83	74
ACC_00199	activating signal cointegrator 1-like		KOG2845	920	560	527	657	863	188	381	425	591	326	418	658	682	1072	1091	619
ACC_00200	UPF0539 protein CG14977-like			67	53	57	141	211	2	8	9	69	25	63	153	165	217	21	8
ACC_00201	protein FAM49B-like		KOG3951	1173	850	945	2462	746	162	195	286	805	260	412	414	39	187	246	68
ACC_00202	LOW QUALITY PROTEIN	K13105	KOG3903	455	385	416	415	396	58	119	156	503	264	189	456	346	431	163	72
ACC_00203	myophilin		KOG2046	238	217	94	498	284	97	126	137	931	459	124	420	1036	1042	242	85
ACC_00204	LOW QUALITY PROTEIN	K10366	KOG4286	437	312	334	464	150	78	85	125	554	292	161	154	72	119	298	91
ACC_00205	superoxide dismutase	K04565	KOG0441	1007	790	665	2540	1563	272	250	342	4358	1848	3467	8160	5041	4991	1036	543
ACC_00206	rho GTPase-activating protein 190		KOG4271	1428	869	821	1426	1186	261	487	584	1347	1176	789	375	458	393	758	320
ACC_00207	conserved hypothetical protein		KOG4221	456	250	231	394	227	74	115	124	967	1352	119	309	335	294	191	39
ACC_00208	membrane-associated guanylate kinase, WW and PDZ doma		KOG3209	438	380	265	559	367	78	142	142	868	1024	159	475	540	408	122	47
ACC_00209	n6-adenosine-methyltransferase 70 kDa subunit-like		KOG2098	162	82	89	141	81	15	26	19	227	105	93	141	201	225	55	27
ACC_00210	patched domain-containing protein 3-like		KOG1934	1859	577	561	1130	905	74	146	135	953	325	502	543	143	281	86	42
ACC_00211	cat eye syndrome critical region protein 5-like isoform 1		KOG1618	404	184	188	301	247	9	11	12	404	367	63	282	326	206	15	1
ACC_00212	beta-1,4-N-acetylgalactosaminyltransferase bre-4 isoform 2		KOG3916	153	60	61	121	112	6	7	14	149	107	47	104	58	68	11	3
ACC_00213	conserved hypothetical protein		KOG1793	540	395	444	798	580	75	125	150	748	518	444	533	894	1134	594	351
ACC_00214	conserved hypothetical protein	K04671	KOG3653	1236	707	697	1225	1115	140	234	269	1703	1487	609	1188	1201	999	392	80
ACC_00215	signal recognition particle 9 kDa protein	K03109	KOG3465	131	144	124	177	158	27	51	65	92	57	82	128	204	253	75	46
ACC_00216	conserved hypothetical protein	K06546		1061	1189	712	781	513	168	196	318	1797	1348	1022	2813	671	679	102	58
ACC_00217	LOW QUALITY PROTEIN		KOG0161	351	250	194	197	219	82	211	203	216	199	100	166	430	400	262	144
ACC_00218	d-tyrosyl-tRNA(Tyr) deacylase 1-like isoform 1	K07560	KOG3323	102	93	92	98	89	14	43	43	87	59	91	144	524	405	27	24
ACC_00219	kinesin-like protein KIF3A-like	K10394	KOG4280	625	434	553	536	433	32	37	37	304	217	101	288	57	39	18	8
ACC_00220	conserved hypothetical protein	K14014		53	42	37	81	82	6	11	9	31	17	56	116	49	26	23	13
ACC_00221	WD repeat-containing protein 89-like		KOG1188	206	206	181	420	377	36	63	79	404	207	208	331	320	524	120	38
ACC_00222	LOW QUALITY PROTEIN			539	267	159	161	183	71	106	148	207	374	62	75	827	835	164	34
ACC_00223	conserved hypothetical protein			909	422	248	1009	589	361	653	774	1186	419	495	824	2088	4263	9389	8785
ACC_00224	UPF0451 protein C17orf61 homolog		KOG3472	95	80	71	81	71	12	16	22	77	52	61	157	175	219	22	17
ACC_00225	single-strand selective monofunctional uracil DNA g	K10800		243	173	187	431	399	29	49	36	191	114	149	309	262	409	87	10

ACC_00226	probable G-protein coupled receptor Mth-like 5-lik	K04599	KOG4193	150	62	84	163	148	6	15	14	216	138	233	758	179	189	40	18
ACC_00227	LOW QUALITY PROTEIN		KOG1520	1731	1084	825	1199	1122	91	298	378	3223	3174	1071	2537	4736	3705	683	449
ACC_00228	beta-mannosidase-like	K01192	KOG2230	997	624	555	712	953	84	241	237	904	800	599	954	1838	1372	223	109
ACC_00229	conserved hypothetical protein		KOG3882	0	2	0	0	0	0	0	1	3	1	4	18	0	3	0	0
ACC_00230	conserved hypothetical protein			237	125	110	115	47	47	72	103	225	192	57	92	132	199	434	188
ACC_00231	hypothetical protein			0	0	1	0	1	1	0	0	2	6	0	0	0	0	0	2
ACC_00232	conserved hypothetical protein			1	0	1	0	0	0	0	0	0	3	0	0	0	0	0	0
ACC_00233	conserved hypothetical protein		KOG1214	1099	315	397	862	445	28	43	60	355	241	45	50	143	360	108	21
ACC_00234	hypothetical protein			0	0	1	1	0	0	0	1	0	0	0	0	1	0	0	1
ACC_00235	conserved hypothetical protein		KOG0161	177	138	121	245	107	27	65	41	5228	1634	14	23	222	263	69	62
ACC_00236	kynurenine 3-monooxygenase	K00486	KOG2614	44	24	24	87	61	30	59	58	334	94	8	14	47	67	13	6
ACC_00237	programmed cell death 6-interacting protein	K12200	KOG2220	828	698	639	810	742	132	149	184	2372	1906	745	2071	2291	1811	221	78
ACC_00238	isoleucyl-tRNA synthetase, cytoplasmic	K01870	KOG0434	412	284	231	280	376	19	50	66	427	562	115	384	1385	1242	147	99
ACC_00239	transcription elongation factor B polypeptide 2	K03873	KOG4495	120	97	93	158	156	7	7	8	190	199	67	379	301	241	15	7
ACC_00240	LOW QUALITY PROTEIN		KOG3248	277	144	208	253	100	22	48	41	113	91	20	4	19	37	141	64
ACC_00241	hypothetical protein			29	18	24	16	11	0	7	2	11	7	4	4	3	3	28	11
ACC_00242	hypothetical protein			0	1	2	3	1	0	0	0	1	17	0	1	17	13	3	8
ACC_00243	LOW QUALITY PROTEIN	K08799	KOG0583	42	33	31	66	46	4	9	5	47	26	20	16	74	115	22	8
ACC_00244	peptidyl-prolyl cis-trans isomerase 1-like isoform 1		KOG0546	1619	944	1003	1920	1563	373	964	925	1150	622	929	314	746	1013	1737	1273
ACC_00245	cell division cycle protein 16 homolog	K03353	KOG1173	107	82	80	143	146	12	14	24	159	109	110	148	238	405	32	18
ACC_00246	conserved hypothetical protein		KOG1430	165	107	109	132	157	22	14	28	454	339	106	257	518	395	21	6
ACC_00247	LOW QUALITY PROTEIN		KOG3690	275	193	206	314	261	39	68	74	628	579	128	354	363	368	65	17
ACC_00248	E3 ubiquitin-protein ligase listerin-like		KOG0803	539	348	292	348	339	84	131	130	769	744	291	485	1525	1355	408	121
ACC_00249	testican-1-like		KOG3555	1123	646	780	636	182	48	41	62	1063	859	248	519	34	52	64	35
ACC_00250	tubulin alpha-1C chain-like		KOG1376	12	11	9	18	25	1	6	3	344	90	1538	3205	2	1	6	0
ACC_00251	chromatin assembly factor 1 subunit B	K10751	KOG1009	98	82	71	104	95	2	10	6	83	83	77	135	436	449	69	17
ACC_00252	ral GTPase-activating protein subunit beta		KOG3652	1287	788	805	862	855	135	166	199	1654	994	608	504	492	452	54	20
ACC_00253	acylglycerol kinase, mitochondrial-like	K09881	KOG4435	499	393	370	576	643	66	114	131	766	541	397	716	1338	1623	293	108
ACC_00254	hypothetical protein			49	30	31	26	31	0	3	4	54	98	11	79	41	19	4	2
ACC_00255	transmembrane protein 147-like isoform 1		KOG3236	162	145	136	255	210	16	28	37	274	124	261	441	628	599	34	14
ACC_00256	transmembrane protein 68-like		KOG4321	89	87	104	104	124	9	11	6	139	45	45	133	93	89	4	0
ACC_00257	2-aminoethanethiol dioxygenase-like	K10712	KOG4281	89	61	62	178	156	4	7	9	156	97	80	248	150	168	17	4
ACC_00258	peripheral plasma membrane protein CASK-like iso	K06103	KOG0609	1237	505	573	1762	515	91	139	194	742	393	225	196	26	68	105	53
ACC_00259	hypothetical protein			0	0	0	1	0	0	0	0	0	0	0	0	0	0	0	0
ACC_00260	hypothetical protein			0	0	0	0	0	0	0	0	0	0	0	0	0	0	0	0
ACC_00261	tropomyosin-2-like		KOG1003	15	7	1	8	11	17	29	20	68	31	8	0	7	8	22	16
ACC_00262	t-complex protein 1 subunit gamma	K09495	KOG0364	932	440	479	1269	980	86	145	186	1353	822	712	1455	3364	5346	707	429
ACC_00263	methionyl-tRNA synthetase, cytoplasmic-like isofor	K01874	KOG1247	424	287	257	558	476	83	133	219	500	420	273	340	1201	1494	446	217
ACC_00264	LOW QUALITY PROTEIN	K16055	KOG1050	600	529	670	2822	1548	250	379	484	2211	8282	783	932	2996	1450	1008	204
ACC_00265	poly(U)-specific endoribonuclease homolog		KOG2849	35	31	29	38	49	1	1	1	23	50	34	112	40	31	0	0
ACC_00266	GABA neurotransmitter transporter-1B	K05034	KOG3660	1917	589	681	1456	1467	23	57	66	1043	240	6	14	29	129	27	1
ACC_00267	protein TSSC1-like		KOG1007	133	82	103	176	161	15	17	17	177	84	136	239	268	433	37	14
ACC_00268	cytochrome b-c1 complex subunit Rieske, mitochor	K00411	KOG1671	1133	438	520	2040	1078	143	148	181	2062	679	608	1804	1141	1379	909	464
ACC_00269	LOW QUALITY PROTEIN	K10414	KOG3595	29	7	12	31	18	5	5	3	40	47	338	122	1	4	7	1
ACC_00270	sodium- and chloride-dependent glycine transporte	K05038	KOG3660	413	203	222	217	60	8	7	16	175	132	40	40	1	1	5	10
ACC_00271	chaoptin		KOG2704	190	142	121	391	261	74	91	101	2188	1823	220	1134	774	599	134	18
ACC_00272	conserved hypothetical protein			424	244	306	646	588	34	44	95	280	272	184	252	1273	403	79	26
ACC_00273	LOW QUALITY PROTEIN		KOG3326	182	174	130	259	228	11	30	39	637	157	206	326	146	166	22	16
ACC_00274	ribosomal RNA-processing protein 8-like	K14850	KOG3045	271	230	250	178	257	13	35	46	72	68	183	254	251	311	410	215
ACC_00275	hypothetical protein			0	1	0	6	3	0	1	1	1	1	2	0	0	0	5	0
ACC_00276	protein zyg-11 homolog B-like	K10350	KOG3665	823	368	346	471	487	46	56	60	1133	848	191	579	398	291	31	5
ACC_00277	conserved hypothetical protein			368	271	177	385	397	55	125	147	201	266	31	33	152	112	121	66
ACC_00278	conserved hypothetical protein			389	303	164	320	359	34	101	159	144	196	30	36	97	59	189	117
ACC_00279	sialin-like		KOG2532	195	114	71	223	180	19	21	28	247	213	95	331	525	495	82	12
ACC_00280	rab11 family-interacting protein 4A-like	K12485	KOG0982	522	357	218	317	223	92	152	115	1411	378	491	689	348	329	170	87
ACC_00281	LOW QUALITY PROTEIN	K11583	KOG2562	339	229	266	427	469	23	57	56	237	183	178	302	424	467	110	54
ACC_00282	collagen alpha-2(IV) chain-like	K08131	KOG3546	305	54	53	114	47	4	2	10	78	71	5	14	132	67	18	32

ACC_00283	ATPase family AAA domain-containing protein 2		KOG0732	7	5	12	11	9	0	0	0	219	14	0	4	4	4	2	2
ACC_00284	ATPase family AAA domain-containing protein 2-like		KOG0732	843	502	448	958	869	120	225	267	2040	1576	340	902	735	967	516	326
ACC_00285	probable phospholipid-transporting ATPase ID-like	K01530	KOG0206	141	106	72	116	70	17	32	31	378	447	230	278	334	263	142	32
ACC_00286	trypsin-1		KOG3627	21	13	18	53	63	1	7	2	29	7	7	1	6094	13997	1056	47
ACC_00287	hypothetical protein			6	1	2	3	5	3	5	4	0	4	3	0	0	0	2	2
ACC_00288	conserved hypothetical protein		KOG0995	32	5	13	13	7	2	4	15	20	35	5	2	10	5	7	2
ACC_00289	PAB-dependent poly(A)-specific ribonuclease subui	K12571	KOG1275	361	225	225	230	158	40	72	61	345	373	121	149	324	340	495	350
ACC_00290	alpha-amylase	K01176	KOG2212	36	5841	6355	3862	2596	2529	1787	3664	11	6	8	2	46	701	64	4
ACC_00291	peptidoglycan-recognition protein S2 precursor	K01446		320	206	241	1007	518	48	37	39	1475	3023	558	2519	1116	218	31	14
ACC_00292	peptidoglycan-recognition protein LB			4	9	6	7	6	3	1	0	45	68	23	14	97	40	9	2
ACC_00293	pyridoxine-5'-phosphate oxidase-like	K00275	KOG2586	206	126	127	249	285	20	34	49	213	131	164	499	442	465	46	15
ACC_00294	LOW QUALITY PROTEIN	K12272	KOG0090	217	204	191	277	331	25	49	64	244	154	191	484	523	783	58	34
ACC_00295	NADPH oxidase 5		KOG0039	17	19	29	27	25	7	4	5	81	251	9	6	118	122	28	18
ACC_00296	conserved hypothetical protein			713	490	399	406	683	96	277	322	376	272	417	251	404	578	457	359
ACC_00297	succinate dehydrogenase	K00234	KOG2403	503	294	252	381	292	38	34	40	1095	782	276	927	1283	1875	219	36
ACC_00298	dynamin-like		KOG0446	1810	718	745	1124	637	78	69	146	568	416	135	140	146	181	109	49
ACC_00299	peroxisome biogenesis factor 10-like	K13346	KOG0317	51	26	42	49	62	5	1	8	76	45	33	87	124	126	7	2
ACC_00300	LOW QUALITY PROTEIN		KOG4389	806	381	289	635	354	44	41	54	893	467	203	301	211	271	137	17
ACC_00301	mitogen-activated protein kinase kinase kinase kin	K08833	KOG0576	77	58	54	70	48	8	7	15	209	302	22	68	68	58	9	4
ACC_00302	spectrin beta chain	K06115	KOG0517	1406	1162	1036	1452	695	479	790	829	3294	4306	506	786	2351	2577	1879	599
ACC_00303	aryl hydrocarbon receptor nuclear translocator hor	K09097	KOG3561	277	157	180	134	67	18	5	11	312	271	79	244	238	302	145	22
ACC_00304	putative rRNA methyltransferase 3-like isoform 1	K14857	KOG1098	1269	741	590	582	866	205	532	563	578	542	647	531	1758	2524	2505	2041
ACC_00305	26S protease regulatory subunit 8 isoform 1	K03066	KOG0728	442	264	272	657	538	64	78	102	1410	775	904	1746	1753	2556	388	264
ACC_00306	e3 ubiquitin-protein ligase HECW2-like	K12168	KOG0940	631	407	343	443	269	44	67	66	828	489	252	344	344	351	52	23
ACC_00307	sodium-independent sulfate anion transporter-like	K14708	KOG0236	168	170	210	503	506	24	15	25	1280	1308	1766	3315	2621	1396	83	10
ACC_00308	axin-1-like	K02157	KOG3589	306	233	263	193	102	8	10	17	307	229	42	86	149	193	85	10
ACC_00309	UNC93-like protein-like		KOG3097	34	20	14	12	3	11	11	18	105	122	312	376	228	133	99	56
ACC_00310	nucleoporin Nup37-like	K14302	KOG0315	87	67	56	258	234	12	12	20	70	46	89	117	374	588	65	34
ACC_00311	adenylate cyclase type 9-like	K08049	KOG3618	368	286	242	328	232	20	55	52	646	990	138	360	575	377	71	28
ACC_00312	cytochrome P450 4C1, partial		KOG0157	26	35	28	144	100	6	13	8	1090	4500	115	86	5	2	0	0
ACC_00313	structural maintenance of chromosomes protein 2	K06674	KOG0933	360	296	215	393	442	46	100	123	171	122	274	100	571	1052	1549	872
ACC_00314	hypothetical protein			9	2	8	4	4	0	0	2	25	25	2	4	3	9	8	0
ACC_00315	LOW QUALITY PROTEIN	K02148	KOG2909	785	354	356	414	428	40	56	63	1628	852	349	1481	1498	2025	140	52
ACC_00316	heat shock protein 67B2-like		KOG1530	36	25	31	49	60	5	25	7	25	15	45	48	47	54	15	6
ACC_00317	cytochrome P450 4C1, partial		KOG0157	5	0	2	3	3	0	0	1	3	4	0	8	5	11	5	0
ACC_00318	zinc finger protein 598-like		KOG2231	864	479	376	797	696	139	330	353	864	708	430	274	1291	1690	1185	674
ACC_00319	transcription initiation factor TFIID subunit 12	K03126	KOG1142	236	164	171	368	214	22	29	34	306	142	251	404	332	493	179	47
ACC_00320	conserved hypothetical protein			24	14	11	14	2	0	0	4	15	7	0	1	0	0	6	9
ACC_00321	UPF0364 protein C6orf211 homolog		KOG3870	297	192	187	320	384	40	55	61	616	669	413	1241	2030	1104	129	76
ACC_00322	conserved hypothetical protein	K03004	KOG4134	487	313	296	273	438	29	61	79	203	250	209	338	559	742	408	150
ACC_00323	geranylgeranyl pyrophosphate synthase-like, partia	K00804	KOG0777	87	66	52	98	121	2	7	11	163	81	32	113	294	254	9	4
ACC_00324	dnaJ homolog subfamily C member 8-like isoform 1	K09528	KOG1150	344	155	127	404	392	102	277	261	302	168	233	211	183	281	929	886
ACC_00325	argininosuccinate synthase-like	K01940	KOG1706	15	18	18	53	40	3	3	3	228	362	0	0	1444	188	3	3
ACC_00326	protein ROP-like isoform 2	K15292	KOG1300	3140	1538	1737	2962	2032	166	277	320	1788	1316	393	933	449	360	104	62
ACC_00327	abhydrolase domain-containing protein FAM108C1-like isofo		KOG1552	182	89	49	92	71	33	72	91	242	209	39	119	145	135	147	51
ACC_00328	Na(+)/H(+) exchange regulatory cofactor NHE-RF2-like		KOG3209	275	177	108	203	85	56	54	76	1723	1202	238	1839	389	303	208	91
ACC_00329	probable E3 ubiquitin-protein ligase makorin-1-like	K15687	KOG1039	315	167	123	273	202	14	24	28	410	276	115	290	290	244	57	27
ACC_00330	oxysterol-binding protein-related protein 9-like isoform 2		KOG2210	432	277	253	426	408	28	36	67	447	392	154	375	295	305	54	29
ACC_00331	cullin-5	K10612	KOG2285	153	94	77	126	117	7	5	14	296	274	71	271	235	232	26	11
ACC_00332	histone acetyltransferase Tip60	K11304	KOG2747	124	86	93	131	81	25	28	33	119	68	50	65	63	94	52	26
ACC_00333	excitatory amino acid transporter		KOG3787	1395	873	1118	2688	656	287	431	691	736	680	522	174	2	6	112	73
ACC_00334	aromatic-L-amino-acid decarboxylase	K01593	KOG0628	161	86	125	327	152	7	10	18	590	151	24	329	260	178	49	52
ACC_00335	neural-cadherin		KOG4289	2164	996	1108	1833	396	57	76	136	815	483	236	61	28	44	207	132
ACC_00336	UPF0420 protein C16orf58 homolog		KOG4249	237	156	135	231	240	8	16	18	265	181	118	391	295	274	31	6
ACC_00337	DNA replication complex GINS protein PSF2	K10733	KOG4071	114	93	109	194	156	6	17	35	152	75	91	175	101	159	26	18
ACC_00338	LOW QUALITY PROTEIN			363	31	23	43	15	19	17	22	515	83	8	425	519	526	446	120
ACC_00339	probable ATP-dependent RNA helicase CG8611-like		KOG0348	447	234	179	523	429	62	173	192	355	361	244	200	670	792	980	552

ACC_00340	60S ribosomal protein L37a	K02921	KOG0402	528	297	194	754	249	103	172	272	184	160	527	301	3502	2860	1087	908
ACC_00341	uracil phosphoribosyltransferase homolog isoform	K00761	KOG1017	45	31	28	54	50	3	7	7	75	61	41	58	114	88	10	1
ACC_00342	putative succinate dehydrogenase	K00237	KOG4097	413	255	263	757	872	24	38	65	698	265	480	1210	783	885	65	34
ACC_00343	NADP-dependent malic enzyme	K00029	KOG1257	586	253	223	349	252	17	24	26	604	315	146	500	332	319	65	29
ACC_00344	WD repeat-containing protein 44-like		KOG0283	344	254	224	291	164	26	31	42	533	418	127	226	260	210	120	57
ACC_00345	trafficking protein particle complex subunit 1		KOG3368	53	56	37	87	103	8	7	17	141	75	57	242	247	254	25	9
ACC_00346	proline synthase co-transcribed bacterial homolog	K06997	KOG3157	137	90	83	126	191	4	12	11	349	244	72	392	1082	919	43	11
ACC_00347	vacuolar protein sorting-associated protein 45-like	K12479	KOG1299	756	533	634	790	833	40	79	89	692	469	382	906	721	723	60	14
ACC_00348	28S ribosomal protein S7, mitochondrial	K02992	KOG3291	151	143	141	242	262	9	29	20	92	75	213	352	252	483	94	60
ACC_00349	BRCA1-associated RING domain protein 1-like	K10683	KOG4177	217	166	162	226	219	26	54	48	557	403	265	446	353	313	37	34
ACC_00350	iron-sulfur cluster assembly 1 homolog, mitochond	K13628	KOG1120	242	147	171	310	355	17	16	24	502	275	120	494	176	181	26	8
ACC_00351	eukaryotic translation initiation factor 3 subunit D-	K03251	KOG2479	647	468	423	1007	917	51	69	111	773	1051	435	1366	3563	5592	614	155
ACC_00352	mitochondrial dicarboxylate carrier-like	K13577	KOG0759	157	139	117	175	208	6	15	30	478	364	141	316	887	479	44	9
ACC_00353	protein transport protein Sec24B-like	K14007	KOG1985	316	185	115	213	186	20	32	33	635	738	141	604	1291	1116	62	22
ACC_00354	hypothetical protein			24	19	21	25	22	1	3	5	81	105	41	44	132	136	6	0
ACC_00355	Predicted ubiquitin-protein ligase/hyperplastic discs protein,		KOG0943	520	330	416	333	218	47	142	132	139	80	54	17	17	12	71	23
ACC_00356	cysteine string protein-like isoform 1	K09525	KOG0716	949	337	323	523	246	71	87	133	491	429	110	333	357	341	88	46
ACC_00357	conserved hypothetical protein		KOG1035	1355	868	809	956	892	106	159	168	1528	1368	451	641	1503	1202	198	58
ACC_00358	conserved hypothetical protein		KOG0477	103	91	57	93	85	22	31	31	138	55	61	28	56	116	55	16
ACC_00359	calcium/calmodulin-dependent protein kinase type	K08794	KOG0032	523	374	325	247	127	41	48	52	673	1036	79	225	936	429	147	55
ACC_00360	LOW QUALITY PROTEIN	K09498	KOG0359	1885	960	849	1593	1448	352	646	736	2453	1575	882	2574	5588	6206	2039	1576
ACC_00361	kelch-like protein 10-like		KOG4441	83	24	11	57	12	1	1	3	21	5	0	0	0	0	0	1
ACC_00362	ubiquitin-conjugating enzyme E2 variant 2-like isof	K10704	KOG0896	337	232	213	381	249	38	42	56	511	409	127	674	1020	1215	510	227
ACC_00363	conserved hypothetical protein			0	0	0	0	1	1	0	0	5	1	0	0	0	0	0	0
ACC_00364	hypothetical protein			3	3	3	0	1	0	0	1	1	2	0	0	0	0	1	0
ACC_00365	hypothetical protein			7	5	3	4	3	1	0	2	5	0	3	0	0	0	1	1
ACC_00366	12 kDa FK506-binding protein	K09568	KOG0544	1250	1220	1186	1199	781	253	308	394	1063	676	658	2060	1855	2539	791	349
ACC_00367	40S ribosomal protein S12-like	K02951	KOG3406	939	855	437	1530	1229	242	502	640	1271	1047	1440	1538	9193	9042	1317	569
ACC_00368	immediate early response 3-interacting protein 1-like		KOG4779	88	46	37	162	105	11	16	15	82	53	65	208	264	412	95	38
ACC_00369	zinc finger protein-like 1-like isoform 1		KOG3970	130	148	111	183	156	21	21	42	520	305	83	358	96702	43437	6636	1550
ACC_00370	conserved hypothetical protein		KOG3213	6	4	3	8	5	0	2	1	22	8	2	9	4	3	6	0
ACC_00371	probable ubiquitin carboxyl-terminal hydrolase FAF	K11840	KOG1866	1962	1338	1274	1701	909	329	442	604	3274	4064	600	722	1638	1210	678	209
ACC_00372	eukaryotic translation initiation factor 2A-like	K15026	KOG2315	687	468	365	733	526	92	167	173	801	779	182	352	1548	1411	612	354
ACC_00373	mitochondrial import inner membrane translocase	K09539	KOG0723	246	172	215	387	263	5	13	27	425	126	321	633	165	191	25	12
ACC_00374	leucine-rich repeat-containing protein 49-like	K16606	KOG0531	727	234	172	299	355	41	157	130	593	194	279	563	342	393	252	84
ACC_00375	nucleosomal histone kinase 1-like	K08816	KOG1164	268	159	170	244	249	43	135	133	314	134	210	207	312	503	448	346
ACC_00376	hypothetical protein			0	0	0	2	1	0	0	1	0	5	1	0	0	0	0	0
ACC_00377	obscurin-like			40	7	16	25	6	2	0	7	33	30	12	6	0	0	1	1
ACC_00378	hypothetical protein			5	0	2	3	1	0	1	1	2	3	0	0	0	0	0	0
ACC_00379	hypothetical protein			0	0	0	1	0	0	1	0	0	6	1	0	0	0	0	3
ACC_00380	oxysterol-binding protein-related protein 1-like isoform 1		KOG2209	277	174	210	235	121	8	17	22	292	133	53	58	85	26	5	3
ACC_00381	transcription initiation factor TFIID subunit 9-like is	K03133	KOG3334	155	146	177	253	181	17	45	33	266	143	142	285	309	421	77	34
ACC_00382	pancreatic lipase-related protein 2-like			3	1	1	18	11	0	5	7	4	3	1	4	1604	717	112	7
ACC_00383	cytochrome P450 9e2	K15003	KOG0158	1117	801	1041	3234	1237	66	62	116	2645	2801	1829	1671	1221	724	401	19
ACC_00384	coronin-7-like		KOG1445	288	169	154	244	149	23	39	43	539	499	121	262	269	226	116	34
ACC_00385	cytochrome P450 9e2-like	K15003	KOG0158	1	2	1	2	0	0	0	1	3	2	0	1	7	8	5	0
ACC_00386	membralin-like		KOG2092	322	215	208	208	119	40	40	49	722	760	121	298	345	268	29	11
ACC_00387	ferritin heavy chain		KOG2332	2025	3336	2125	4398	4139	829	1214	1609	9460	4341	3084	7069	11345	8427	795	226
ACC_00388	nuclear valosin-containing protein	K14571	KOG0733	395	212	208	420	290	45	72	100	600	407	198	326	713	1302	304	139
ACC_00389	probable trans-2-enoyl-CoA reductase, mitochondr	K07512	KOG0025	402	275	215	400	461	42	68	75	1089	496	263	853	1943	2267	167	40
ACC_00390	adenylosuccinate synthetase-like	K01939	KOG1355	489	271	253	587	403	72	85	77	1376	615	424	1142	714	743	127	46
ACC_00391	homeobox protein MSX-1-like		KOG0492	0	1	1	1	3	0	0	0	0	0	0	0	0	1	0	0
ACC_00392	sodium-dependent nutrient amino acid transporter	K05038	KOG3660	7	4	0	7	10	4	2	4	127	62	41	334	3	3	4	0
ACC_00393	nuclear distribution protein nudE-like 1-A-like	K16739	KOG1853	316	253	243	417	282	37	53	71	327	226	132	285	270	307	76	41
ACC_00394	conserved hypothetical protein		KOG3598	1552	953	2281	2764	1550	333	855	988	2701	1426	1790	1787	528	517	1601	897
ACC_00395	conserved hypothetical protein			595	378	335	561	624	31	40	72	830	889	234	695	1007	1055	115	42
ACC_00396	ATP-citrate synthase isoform 1	K01648	KOG1254	1060	587	541	1049	1103	113	151	181	5025	5541	630	2002	17411	7380	754	498

ACC_00397	serine/threonine-protein kinase 16-like	K08856	KOG2345	154	83	88	169	151	11	11	21	267	193	82	324	419	387	28	6
ACC_00398	LOW QUALITY PROTEIN		KOG0056	584	444	459	796	712	46	79	87	1159	1059	535	830	652	579	92	20
ACC_00399	probable cation-transporting ATPase 13A3-like		KOG0208	659	761	1285	1913	2376	50	120	106	2646	1899	880	4057	811	352	17	12
ACC_00400	protein DJ-1-like	K05687	KOG2764	290	256	225	962	733	34	55	80	403	231	454	766	522	343	54	42
ACC_00401	thyroid receptor-interacting protein 6-like	K16676	KOG1701	731	385	359	693	471	54	58	90	795	417	186	561	714	731	78	21
ACC_00402	prostatic acid phosphatase-like	K14410	KOG3720	251	150	101	245	319	27	61	82	256	259	240	468	236	132	47	33
ACC_00403	acyl-CoA-binding domain-containing protein 5-like		KOG0817	337	175	139	217	194	71	119	92	1227	599	283	568	526	369	114	69
ACC_00404	hypothetical protein			0	1	0	0	0	0	0	2	0	1	0	0	0	0	0	0
ACC_00405	hypothetical protein			1	0	1	0	0	2	0	0	0	0	0	0	0	0	0	0
ACC_00406	exonuclease 1-like	K10746	KOG2518	155	123	110	185	158	16	17	20	98	50	44	37	686	396	45	19
ACC_00407	conserved hypothetical protein		KOG1550	1051	726	784	990	1022	85	132	137	1328	622	316	743	759	787	197	98
ACC_00408	ATP synthase subunit b, mitochondrial-like	K02127	KOG3976	2355	880	792	1963	2068	290	570	642	3485	993	1330	4233	3980	4322	1088	905
ACC_00409	EF-hand calcium-binding domain-containing protein 2-like		KOG0027	31	31	30	54	60	2	6	10	22	10	25	4	0	3	1	1
ACC_00410	cleavage and polyadenylation specificity factor sub	K14401	KOG1896	386	280	290	427	474	45	52	96	456	428	217	241	523	575	70	28
ACC_00411	cyclin-C	K15161	KOG0794	322	199	245	522	401	16	38	42	307	169	169	338	221	337	55	24
ACC_00412	ferredoxin		KOG3309	121	174	187	216	247	8	13	28	143	47	96	149	161	276	28	14
ACC_00413	mitotic spindle assembly checkpoint protein MAD2	K02537	KOG3285	36	21	18	68	57	2	4	10	19	19	23	11	44	93	15	21
ACC_00414	DNA-directed RNA polymerase III subunit RPC4-like	K03026	KOG3122	190	168	147	310	297	31	74	82	145	106	108	119	234	319	201	136
ACC_00415	protein FAM76A-like		KOG3990	280	228	240	444	213	42	53	73	387	256	111	273	352	309	167	53
ACC_00416	LOW QUALITY PROTEIN		KOG3784	289	268	206	277	318	41	102	94	462	388	229	446	468	523	60	16
ACC_00417	bladder cancer-associated protein-like		KOG4489	99	58	80	116	143	3	4	12	201	84	35	204	118	140	16	7
ACC_00418	probable tubulin polyglutamylase TTLL2-like	K16600	KOG2157	156	56	61	130	141	9	17	32	148	79	83	45	171	339	51	34
ACC_00419	gastrulation defective protein 1 homolog		KOG0772	365	285	307	410	303	55	92	103	769	445	249	691	517	442	102	69
ACC_00420	dihydropyrimidine dehydrogenase	K00207	KOG1799	227	111	153	314	354	10	17	20	355	629	121	254	1881	946	76	6
ACC_00421	3-ketoacyl-CoA thiolase, mitochondrial-like	K07508	KOG1391	495	398	152	369	349	87	94	110	7557	2137	420	1816	14027	12896	1004	364
ACC_00422	DNA repair protein XRCC3-like	K10880	KOG1564	286	201	208	450	524	21	42	51	422	266	238	638	560	365	45	18
ACC_00423	glycerophosphodiester phosphodiesterase 1-like		KOG2258	212	152	162	335	394	17	32	34	251	188	241	284	363	381	22	13
ACC_00424	conserved hypothetical protein	K11841	KOG1871	546	465	371	315	203	48	103	121	832	841	111	316	1745	1991	558	234
ACC_00425	conserved hypothetical protein		KOG2462	217	141	131	256	194	16	22	18	296	194	89	82	173	178	28	10
ACC_00426	DNA-directed RNA polymerase III subunit RPC2 isof	K03021	KOG0215	277	187	216	322	260	30	53	56	360	301	142	138	364	374	45	20
ACC_00427	2-oxoglutarate dehydrogenase, mitochondrial-like	K00164	KOG0450	2249	1136	1205	1535	1344	123	133	153	7545	3848	866	2428	1262	1335	102	37
ACC_00428	protein FAM13B-like		KOG4270	4715	2522	2373	3847	3078	637	1337	1426	4513	2303	1947	932	721	719	1030	555
ACC_00429	protein argonaute-2	K11593	KOG1041	1481	1172	1346	1118	846	258	329	427	2718	2403	678	1066	1264	773	341	93
ACC_00430	conserved hypothetical protein		KOG4188	720	482	500	759	828	230	626	711	730	578	552	481	1068	1169	999	650
ACC_00431	mitochondrial translocator assembly and maintenance prote		KOG2986	86	79	48	144	121	13	26	30	134	70	89	133	127	186	25	15
ACC_00432	conserved hypothetical protein		KOG2064	421	249	234	345	319	55	91	124	490	403	121	133	240	240	77	20
ACC_00433	conserved hypothetical protein	K14569	KOG1980	1010	568	415	775	824	164	322	431	681	540	705	433	1127	1640	2013	1196
ACC_00434	conserved hypothetical protein			1481	941	792	1786	1814	235	652	682	1134	1229	902	709	756	748	885	337
ACC_00435	translocating chain-associated membrane protein :	K14010	KOG1608	2242	1555	712	1067	977	509	993	1485	674	586	781	1409	2611	2019	455	332
ACC_00436	phosphatidate phosphatase LPIN1-like	K15728	KOG2116	1766	983	1015	1466	984	231	492	507	1995	2378	334	394	1401	925	1050	310
ACC_00437	mitochondrial chaperone BCS1-like	K08900	KOG0743	170	156	161	261	210	32	49	55	251	166	100	177	591	732	95	42
ACC_00438	mitochondrial import receptor subunit TOM40 hon	K11518	KOG3296	598	320	244	695	450	100	132	171	647	435	394	569	1616	2676	885	552
ACC_00439	39S ribosomal protein L14, mitochondrial	K02874	KOG3441	155	101	105	170	214	13	12	19	113	67	109	311	403	623	55	39
ACC_00440	PRKCA-binding protein-like		KOG3651	410	278	236	444	388	29	41	54	721	477	176	684	847	690	44	11
ACC_00441	prostaglandin E synthase 2-like	K05309	KOG3029	646	435	443	649	806	38	129	127	626	410	339	969	721	555	81	36
ACC_00442	TGF-beta receptor type-1-like	K13569	KOG2052	745	400	356	812	348	157	296	346	506	373	325	216	175	208	1543	772
ACC_00443	UDP-glucose 6-dehydrogenase-like	K00012	KOG2666	190	91	105	303	154	21	16	24	237	253	62	159	786	810	226	39
ACC_00444	30S ribosomal protein S11	K02948	KOG0408	427	299	310	653	767	39	73	89	324	226	319	450	486	654	142	71
ACC_00445	AP-3 complex subunit mu-1-like	K12398	KOG2740	278	209	190	339	272	39	47	52	317	183	201	341	338	355	29	12
ACC_00446	ankyrin repeat, SAM and basic leucine zipper domain-contai		KOG4412	342	210	213	180	240	19	7	26	315	228	172	182	264	240	25	7
ACC_00447	protein LZIC-like			88	65	69	118	178	1	10	11	131	72	70	151	109	105	6	7
ACC_00448	single-chain bursicon precursor			49	62	50	64	31	3	5	9	98	132	17	68	46	57	14	9
ACC_00449	coiled-coil domain-containing protein 75-like		KOG1994	66	36	32	20	24	8	16	20	30	20	41	32	24	84	91	62
ACC_00450	FAS-associated factor 1 isoform 1		KOG1363	599	325	344	705	654	69	104	115	870	541	349	694	559	621	145	92
ACC_00451	LOW QUALITY PROTEIN			2081	1258	1512	812	966	271	675	520	2271	635	966	174	505	680	352	133
ACC_00452	conserved hypothetical protein		KOG0161	77	37	37	45	72	4	12	10	34	33	75	24	94	29	32	19
ACC_00453	partitioning defective 6 homolog gamma-like	K06093	KOG3606	77	64	46	63	53	5	6	18	210	297	38	200	242	222	30	6

ACC_00454	coiled-coil and C2 domain-containing protein 1-like		KOG3837	908	645	567	658	580	205	323	428	1153	850	500	835	846	872	292	155
ACC_00455	probable tRNA(His) guanylyltransferase-like	K10761	KOG2721	101	99	92	143	112	13	24	25	121	121	43	122	409	577	102	58
ACC_00456	ATP synthase subunit g, mitochondrial-like	K02140	KOG4103	68	38	35	139	168	9	5	14	86	20	99	151	217	276	11	9
ACC_00457	conserved hypothetical protein		KOG2416	358	224	186	348	190	40	48	87	363	283	97	172	292	453	569	394
ACC_00458	UPF0672 protein C3orf58 homolog			97	75	71	81	123	5	12	16	86	85	90	200	113	90	3	0
ACC_00459	conserved hypothetical protein			195	105	74	92	126	11	15	26	99	124	76	201	242	194	42	27
ACC_00460	v-type proton ATPase subunit H isoform 2	K02144	KOG2759	1450	653	684	1469	1602	109	134	170	2173	1078	845	2992	1897	2040	252	75
ACC_00461	nuclear hormone receptor FTZ-F1 beta	K08705	KOG4218	152	143	107	88	51	33	35	39	632	618	91	342	344	289	117	32
ACC_00462	conserved hypothetical protein		KOG1832	966	611	546	705	1034	117	322	266	764	701	827	652	798	976	596	283
ACC_00463	conserved hypothetical protein			107	93	85	63	108	2	9	7	56	63	44	164	200	234	26	18
ACC_00464	LOW QUALITY PROTEIN	K01070	KOG3101	120	64	66	222	171	16	19	28	224	175	83	223	737	627	69	42
ACC_00465	LOW QUALITY PROTEIN		KOG0161	116	90	78	91	126	15	28	29	44	27	40	24	21	26	38	21
ACC_00466	surfeit locus protein 1	K14998	KOG1563	279	209	283	380	337	22	29	50	295	157	197	456	461	599	60	35
ACC_00467	Xaa-Pro dipeptidase			15	17	18	26	21	0	2	0	24	12	1	2	14	13	5	2
ACC_00468	G/T mismatch-specific thymine DNA glycosylase-like		KOG4120	98	81	59	151	189	10	22	28	68	81	104	82	80	77	41	15
ACC_00469	methyltransferase-like protein 5-like		KOG3420	64	67	75	132	85	7	23	17	216	238	56	63	3344	878	144	60
ACC_00470	LOW QUALITY PROTEIN			19	17	11	40	33	3	8	6	25	21	28	45	71	101	8	3
ACC_00471	conserved hypothetical protein			360	257	272	352	276	61	95	112	333	166	156	114	125	156	147	86
ACC_00472	FK506-binding protein 15-like		KOG0161	592	450	427	440	429	55	121	137	574	406	224	438	771	530	147	105
ACC_00473	zinc metalloproteinase nas-13-like		KOG3714	27	11	17	3	5	2	2	2	9	12	4	0	20	37	17	13
ACC_00474	transmembrane protein 17-like		KOG4694	40	22	44	45	77	0	1	3	13	54	48	152	65	49	7	1
ACC_00475	caspase Nc-like		KOG3573	219	186	240	324	322	16	24	36	257	127	122	293	360	533	50	15
ACC_00476	conserved hypothetical protein		KOG3627	11	12	8	8	7	1	3	4	19	5	0	8	29	330	177	57
ACC_00477	conserved hypothetical protein	K12752	KOG0030	1146	711	575	1061	952	114	109	189	2984	1303	709	2218	1822	1834	443	106
ACC_00478	WD repeat-containing protein alr3466-like		KOG0272	497	417	440	754	610	49	70	91	438	321	1026	2119	1	43	8	3
ACC_00479	facilitated trehalose transporter Tret1-like		KOG0254	354	166	92	346	293	15	14	25	1117	1048	478	1476	390	69	8	4
ACC_00480	26S proteasome non-ATPase regulatory subunit 14	K03030	KOG1555	73	63	42	112	96	24	23	22	201	113	112	275	438	545	138	66
ACC_00481	tRNA-splicing endonuclease subunit Sen2-like	K15322	KOG4685	135	93	90	273	335	10	33	50	119	101	160	122	249	265	97	69
ACC_00482	coiled-coil domain-containing protein 164-like		KOG0161	69	33	36	35	44	8	15	17	34	56	16	36	68	73	34	16
ACC_00483	HIV-1 Vpr-binding protein		KOG1832	1270	597	819	687	449	178	345	356	667	330	272	86	241	426	591	265
ACC_00484	frizzled	K02432	KOG3577	81	23	15	47	26	2	4	2	68	38	14	76	117	108	17	11
ACC_00485	putative inorganic phosphate cotransporter-like	K08193	KOG2532	72	42	11	26	26	12	10	17	38	69	6	9	130	99	19	10
ACC_00486	u6 snRNA-associated Sm-like protein LSm3-like	K12622	KOG3460	64	63	60	128	102	10	10	10	56	23	63	79	148	285	79	33
ACC_00487	conserved hypothetical protein		KOG1832	22	15	15	39	17	6	1	4	23	18	13	7	8	14	27	27
ACC_00488	e3 SUMO-protein ligase NSE2-like		KOG2979	107	65	63	119	117	12	35	37	82	42	61	110	48	48	39	18
ACC_00489	UPF0605 protein CG18335-like			98	48	56	113	68	8	30	30	92	44	72	70	238	156	43	13
ACC_00490	protein CLEC16A-like		KOG2219	734	496	438	777	655	131	212	209	1743	763	508	540	433	459	182	85
ACC_00491	transcription factor MafG-like isoform 1	K09037	KOG4196	294	223	213	260	267	18	32	50	266	157	276	646	214	281	104	77
ACC_00492	protein maelstrom 2-like, partial			96	93	72	45	53	2	7	5	28	79	2	27	102	61	6	3
ACC_00493	tetratricopeptide repeat protein 26		KOG3785	33	20	25	69	39	4	7	4	51	18	422	319	6	5	5	3
ACC_00494	uridine 5'-monophosphate synthase	K13421	KOG1377	150	151	126	164	137	20	23	29	369	291	142	551	1081	1410	90	34
ACC_00495	signal recognition particle 68 kDa protein	K03107	KOG2460	355	291	236	485	283	85	91	112	998	566	274	710	1802	2029	285	106
ACC_00496	intraflagellar transport protein 140 homolog		KOG3617	160	88	105	188	130	10	24	18	238	111	280	436	122	144	20	6
ACC_00497	conserved hypothetical protein		KOG0161	70	49	65	84	115	7	19	14	53	18	56	5	7	12	3	3
ACC_00498	protein yellow			74	35	27	52	43	9	11	7	910	981	100	598	93	20	4	6
ACC_00499	conserved hypothetical protein		KOG4829	871	541	571	426	624	81	335	258	256	180	292	430	455	643	1081	836
ACC_00500	phosphatidylinositol N-acetylglucosaminyltransfera	K03860	KOG1183	129	87	96	84	131	8	24	19	118	82	170	137	154	152	12	10
ACC_00501	lysophospholipid acyltransferase 7-like	K13516	KOG2706	666	372	356	498	673	27	81	75	1085	901	437	803	1146	834	67	26
ACC_00502	28 kDa heat- and acid-stable phosphoprotein-like		KOG3375	744	550	388	808	382	223	279	434	543	315	367	784	1015	1159	1190	909
ACC_00503	proactivator polypeptide isoform 1	K12382	KOG1340	3192	2144	2165	3215	3633	211	223	312	10272	8247	2094	3764	4394	2728	85	38
ACC_00504	TEL2-interacting protein 1 homolog		KOG4524	465	297	258	474	575	25	86	81	302	301	206	227	389	322	149	64
ACC_00505	protein toll		KOG4194	45	64	40	74	40	1	12	11	522	1031	23	106	132	87	8	2
ACC_00506	protein TBRG4-like		KOG0934	429	241	241	456	576	20	63	57	311	340	315	594	924	710	280	168
ACC_00507	conserved hypothetical protein		KOG4246	1409	917	799	1080	1157	358	670	733	1249	880	890	441	722	1090	2781	1545
ACC_00508	splicing factor 3A subunit 1 isoform 1	K12825	KOG0007	638	495	663	1118	491	77	100	115	732	519	313	544	652	1115	538	275
ACC_00509	conserved hypothetical protein		KOG4225	1861	1201	1361	2466	734	346	481	642	5978	2610	949	966	470	456	866	305
ACC_00510	tyrosine-protein kinase Btk29A-like	K07364	KOG0197	306	154	175	289	154	14	23	37	369	218	45	113	300	216	70	28

ACC_00511	conserved hypothetical protein			32	14	14	13	1	1	0	2	4	5	1	1	0	0	14	5
ACC_00512	rap guanine nucleotide exchange factor 2	K08018	KOG3542	546	398	444	353	164	45	83	88	1001	892	136	202	135	162	185	82
ACC_00513	U4/U6.U5 tri-snRNP-associated protein 2	K12847	KOG2026	202	178	179	236	224	25	33	28	150	178	77	212	356	495	70	21
ACC_00514	GSK3-beta interaction protein-like		KOG3965	39	28	13	33	46	4	8	9	63	73	40	94	38	47	1	1
ACC_00515	conserved hypothetical protein	K09228	KOG2462	96	49	45	93	44	19	27	36	118	39	99	29	13	35	89	53
ACC_00516	conserved hypothetical protein	K09228	KOG2462	361	320	331	539	150	46	66	100	378	290	122	71	74	116	362	95
ACC_00517	conserved hypothetical protein		KOG3623	11	7	7	36	38	2	2	1	14	10	14	2	1	3	1	1
ACC_00518	hypothetical protein			10	3	4	6	4	0	1	1	2	2	1	0	0	1	5	1
ACC_00519	retinal dehydrogenase 1-like isoform 1	K00128	KOG2450	404	360	413	693	620	42	61	59	683	1046	260	552	2985	1849	201	42
ACC_00520	conserved hypothetical protein		KOG2963	1205	698	688	1081	878	119	259	303	2194	1491	321	554	867	1111	781	737
ACC_00521	TWiK family of potassium channels protein 7-like isoform 1		KOG1418	217	212	182	327	201	20	22	45	176	96	93	360	274	214	37	11
ACC_00522	putative glycogen	K00693	KOG3742	413	241	148	229	181	39	62	58	828	432	91	86	1265	994	116	31
ACC_00523	hypothetical protein			17	3	5	13	9	2	2	4	7	1	3	0	0	1	10	0
ACC_00524	TBC1 domain family member 19-like isoform 2		KOG2058	4	4	2	11	7	1	0	0	6	4	7	6	3	0	1	2
ACC_00525	conserved hypothetical protein		KOG3762	809	200	158	362	265	50	43	81	353	156	77	530	153	192	57	22
ACC_00526	single-stranded DNA-binding protein, mitochondria	K03111	KOG1653	155	221	187	193	263	13	29	34	269	141	117	442	559	740	71	20
ACC_00527	LOW QUALITY PROTEIN		KOG4405	1913	710	617	838	675	114	197	224	1110	743	262	833	601	389	183	119
ACC_00528	elongation of very long chain fatty acids protein 6-like		KOG3072	209	73	61	126	91	11	20	18	354	621	76	289	3357	1506	354	50
ACC_00529	coiled-coil domain-containing protein 86-like	K14822	KOG4538	120	105	59	90	72	39	164	139	62	35	51	40	232	475	640	510
ACC_00530	hypothetical protein			0	0	0	1	1	0	0	0	0	0	0	0	0	0	0	0
ACC_00531	LOW QUALITY PROTEIN	K11647	KOG0386	2982	1932	1498	2467	2153	651	1416	1438	2380	1607	1692	732	1702	2287	6458	4113
ACC_00532	COP9 signalosome complex subunit 7-like	K12180	KOG3250	274	200	161	260	181	24	36	52	340	269	107	319	362	421	164	121
ACC_00533	paired amphipathic helix protein Sin3b		KOG4204	780	587	557	764	405	103	131	202	1485	1198	332	299	459	549	198	59
ACC_00534	structural maintenance of chromosomes protein 5-like		KOG0979	372	242	200	421	465	45	97	113	296	226	231	125	347	401	226	114
ACC_00535	conserved hypothetical protein		KOG2546	1040	774	882	1003	552	47	44	68	655	424	163	260	278	301	78	27
ACC_00536	signal peptidase complex catalytic subunit SEC11C	K13280	KOG3342	570	531	296	275	244	116	90	155	508	428	171	821	1418	1141	140	81
ACC_00537	hypothetical protein			1	3	0	1	0	0	0	0	1	0	0	0	0	0	0	0
ACC_00538	hypothetical protein			0	1	1	3	1	1	1	0	1	2	0	1	0	0	3	0
ACC_00539	T-complex protein 1 subunit epsilon-like	K09497	KOG0357	808	506	510	1540	974	104	132	174	1482	1302	813	1967	4463	6637	825	461
ACC_00540	choline transporter-like protein 1-like		KOG1362	159	44	77	130	96	11	16	15	234	231	59	392	145	107	33	4
ACC_00541	dipeptidyl peptidase 9-like	K08656	KOG2281	211	135	117	198	173	16	24	25	351	242	81	200	598	603	52	11
ACC_00542	hypothetical protein			4	2	6	16	4	1	4	2	2	4	0	0	0	0	0	0
ACC_00543	proteasome subunit alpha type-2-like	K02726	KOG0181	482	257	239	440	327	69	77	102	1220	417	869	2396	1558	2459	379	256
ACC_00544	esterase FE4-like		KOG1029	2	2	3	2	2	1	3	7	23	4	0	1	1	0	10	42
ACC_00545	conserved hypothetical protein			0	2	1	1	0	0	2	3	1	1	0	0	0	0	2	1
ACC_00546	conserved hypothetical protein			887	600	544	810	1147	133	362	391	689	602	600	455	551	656	587	273
ACC_00547	conserved hypothetical protein		KOG4294	6	2	5	9	5	1	0	0	11	27	22	8	5	1	1	0
ACC_00548	conserved hypothetical protein		KOG1065	667	512	787	1048	217	50	54	89	1271	1047	221	445	623	383	730	129
ACC_00549	sorting nexin-27-like		KOG3784	563	380	360	747	203	95	92	154	1338	919	120	346	163	151	217	110
ACC_00550	tyrosine-protein phosphatase non-receptor type 9-	K01104	KOG4228	484	256	247	338	143	34	15	24	492	332	49	158	123	81	34	10
ACC_00551	conserved hypothetical protein	K15601	KOG1356	1605	1482	1256	1778	448	235	245	359	3445	3385	504	366	831	1038	1378	665
ACC_00552	photoreceptor-specific nuclear receptor	K07295	KOG4215	25	21	29	44	8	4	3	2	12	7	1	0	1	1	1	1
ACC_00553	conserved hypothetical protein		KOG3598	47	3	5	19	9	5	6	0	376	6	2	14	42	187	62	11
ACC_00554	LOW QUALITY PROTEIN	K10358	KOG0163	4688	2340	1639	2631	2887	1159	2553	2843	2820	1810	1030	1304	1074	1093	1820	1527
ACC_00555	conserved hypothetical protein			911	380	373	664	401	36	48	49	778	617	222	2021	1107	1377	411	81
ACC_00556	ribonuclease H2 subunit B-like	K10744	KOG4705	126	78	56	132	131	10	31	25	320	124	197	333	243	234	85	18
ACC_00557	N(6)-adenine-specific DNA methyltransferase 2-like		KOG3350	114	98	141	118	97	3	28	22	137	95	131	220	159	255	51	32
ACC_00558	parkin coregulated gene protein homolog		KOG3961	72	52	59	176	165	13	27	31	42	24	69	58	53	103	21	7
ACC_00559	26S proteasome complex subunit DSS1-like	K10881		156	142	115	239	127	10	24	15	159	62	190	233	354	390	80	46
ACC_00560	UPF0536 protein C12orf66-like			257	209	203	328	412	17	43	42	208	127	175	205	154	252	34	14
ACC_00561	abhydrolase domain-containing protein 4-like		KOG4409	373	303	230	470	426	147	131	145	1249	602	383	990	636	392	42	10
ACC_00562	alpha-N-acetylgalactosaminidase-like	K01204	KOG2366	533	422	332	503	407	98	86	163	793	216	265	1126	22808	5095	184	125
ACC_00563	protein FAM161A-like	K16772		454	345	330	551	537	54	138	133	289	197	223	213	230	172	143	58
ACC_00564	conserved hypothetical protein		KOG0510	85	82	83	85	99	6	12	19	100	40	28	11	83	69	54	17
ACC_00565	conserved oligomeric Golgi complex subunit 4-like		KOG0412	302	207	212	509	485	26	56	62	342	281	185	310	416	377	57	38
ACC_00566	enhancer of yellow 2 transcription factor homolog	K11368	KOG4479	37	21	20	52	41	3	7	11	36	22	24	73	112	139	22	12
ACC_00567	suppressor of hairless protein	K06053	KOG3743	44	27	26	36	20	3	6	4	224	140	84	296	56	61	25	5

ACC_00568	gamma-glutamyltranspeptidase 1-like isoform 1	K00681	KOG2410	249	246	269	459	369	71	87	81	2262	878	828	1829	971	787	154	20
ACC_00569	membrane magnesium transporter 1-like		KOG3918	100	69	71	143	149	4	16	17	59	36	157	300	98	85	5	1
ACC_00570	adenosine 3'-phospho 5'-phosphosulfate transport	K15276	KOG1581	377	259	235	529	472	28	34	54	426	287	124	395	189	196	33	4
ACC_00571	immune deficiency			174	164	167	217	192	23	49	47	223	156	146	277	322	282	79	23
ACC_00572	adenosine monophosphate-protein transferase FICD homolc		KOG3824	200	141	144	370	226	19	37	56	287	241	121	194	229	172	65	18
ACC_00573	ADP-ribosylation factor-binding protein GGA1 isofc	K12404	KOG1086	720	517	437	621	592	87	131	202	663	633	249	532	619	475	74	20
ACC_00574	dnaJ homolog subfamily C member 17-like	K09537	KOG0691	277	219	205	271	368	37	66	54	415	165	200	310	343	443	83	49
ACC_00575	39S ribosomal protein L17, mitochondrial	K02879	KOG3280	200	185	212	220	221	13	27	33	203	118	235	402	417	668	120	123
ACC_00576	e3 ubiquitin-protein ligase RNF34-like isoform 1		KOG4275	230	124	117	239	339	14	32	37	309	321	158	601	447	337	28	9
ACC_00577	conserved hypothetical protein		KOG0773	281	148	121	118	91	66	110	81	294	402	76	43	487	1328	3091	1182
ACC_00578	hypothetical protein			43	37	62	87	60	5	7	9	79	25	0	0	1	1	2	0
ACC_00579	LOW QUALITY PROTEIN		KOG0444	934	623	611	812	616	152	206	216	2375	981	696	411	785	1075	165	81
ACC_00580	probable ATP-dependent RNA helicase DDX11-like		KOG1133	101	94	107	126	115	6	27	19	88	87	95	76	158	163	46	18
ACC_00581	hypothetical protein			1	0	0	0	0	0	0	0	0	0	1	0	0	0	0	0
ACC_00582	putative odorant receptor 85b-like			4	1	4	0	1	0	0	0	5	4	13	7	3	1	0	0
ACC_00583	neurofibromin	K08052	KOG1826	700	453	371	246	201	45	53	53	1323	1223	107	522	475	381	49	9
ACC_00584	inositol-pentakisphosphate 2-kinase-like	K10572	KOG4749	78	66	56	116	108	5	3	7	132	123	53	163	95	126	11	5
ACC_00585	hypothetical protein			0	0	0	0	0	0	0	0	0	2	0	0	0	0	0	0
ACC_00586	carnitine O-palmitoyltransferase 1, liver isoform-lik	K08765	KOG3716	512	252	170	311	292	92	151	166	1176	2697	225	807	798	436	87	34
ACC_00587	ubiquitin-protein ligase, putative	K10588	KOG4427	590	256	238	440	327	51	65	54	875	765	177	545	322	265	56	14
ACC_00588	pre-mRNA-processing factor 19	K10599	KOG0289	140	78	72	222	211	5	6	9	171	137	95	426	421	703	78	19
ACC_00589	RING finger protein 185-like isoform 2	K10666	KOG0823	289	119	122	394	255	27	31	29	536	336	142	650	434	387	51	11
ACC_00590	THO complex subunit 6 homolog	K13175	KOG0649	243	224	254	327	319	33	71	83	184	145	160	259	293	433	74	76
ACC_00591	DNA ligase 1-like	K10747	KOG0967	3977	2468	2191	2154	2774	819	1578	1699	1763	1328	1217	952	2339	2705	1481	736
ACC_00592	ubiquitin activating enzyme 1 isoform 1	K03178	KOG2012	1310	626	498	1093	912	119	104	125	2907	3296	756	2689	3116	3459	398	103
ACC_00593	kinesin 12		KOG4280	27	47	46	132	61	4	10	16	141	136	1449	1289	69	37	17	5
ACC_00594	conserved hypothetical protein			117	87	78	234	111	6	28	6	53	43	114	177	156	140	146	87
ACC_00595	conserved hypothetical protein	K13172	KOG0670	6902	4561	4518	6064	3367	1067	2247	2099	3437	1670	1454	543	907	1644	5200	3961
ACC_00596	contactin-5-like isoform 1		KOG3513	2140	689	1062	1992	1317	205	397	494	962	618	765	807	94	78	153	160
ACC_00597	conserved hypothetical protein	K09113	KOG3582	468	489	523	430	220	68	68	80	2259	2405	251	541	1716	2528	808	171
ACC_00598	transmembrane protein 11, mitochondrial			287	83	86	164	139	17	31	42	378	341	128	465	600	514	126	40
ACC_00599	histidine decarboxylase isoform 1		KOG0628	81	109	107	474	146	16	11	27	62	31	2	2	1	1	2	3
ACC_00600	mitochondrial phospholipid hydroperoxide glutathi	K00432	KOG1651	706	403	431	1133	1501	18	25	43	868	577	381	1167	950	640	22	7
ACC_00601	acyl carrier protein, mitochondrial-like isoform 1	K03955	KOG1748	378	176	171	990	678	41	40	69	368	167	401	662	999	1196	108	67
ACC_00602	40S ribosomal protein S21-like isoform 1	K02971	KOG3486	293	181	89	511	146	44	102	93	102	54	498	208	1292	1271	421	312
ACC_00603	condensin-2 complex subunit D3-like		KOG0413	731	606	666	446	509	41	119	87	1000	566	295	243	587	695	265	99
ACC_00604	probable serine hydrolase-like isoform 2		KOG1454	486	242	259	357	331	31	55	49	1087	637	375	1094	642	712	61	21
ACC_00605	tRNA-splicing ligase RtcB homolog	K14415	KOG3833	237	160	130	308	286	34	41	48	356	209	174	302	612	560	57	31
ACC_00606	guanylate cyclase 32E-like		KOG1023	349	200	171	312	213	47	42	45	885	624	132	87	436	685	230	53
ACC_00607	hypothetical protein			5	0	0	1	2	0	0	0	0	0	0	0	0	0	4	0
ACC_00608	conserved hypothetical protein			6	8	9	5	12	0	0	3	29	23	36	56	64	84	2	0
ACC_00609	conserved hypothetical protein		KOG0274	10475	7020	5486	5314	6956	1001	2385	2534	3483	3886	2870	869	1644	1838	1932	909
ACC_00610	neuroglian		KOG3513	1165	438	351	226	166	30	51	60	1465	1012	99	1318	1207	807	144	53
ACC_00611	LOW QUALITY PROTEIN		KOG1828	1015	744	608	1236	719	247	386	462	1002	788	706	760	606	570	1034	568
ACC_00612	DNA polymerase eta	K03509	KOG2095	341	279	304	400	455	26	34	36	285	142	171	267	177	189	72	29
ACC_00613	UPF0760 protein C2orf29-like		KOG4508	99	60	66	117	144	5	3	8	167	139	50	173	194	216	14	4
ACC_00614	serine/threonine-protein kinase Genghis Khan	K16307	KOG0612	1439	953	786	977	869	344	742	859	1864	1685	512	320	756	729	1100	591
ACC_00615	ribonuclease H2 subunit C-like	K10745		118	99	82	135	87	2	19	11	54	33	58	195	132	293	68	16
ACC_00616	epidermal growth factor receptor kinase substrate 8-like prc		KOG3557	295	144	156	159	73	16	29	28	692	956	99	535	431	579	189	36
ACC_00617	THUMP domain-containing protein 3-like		KOG2671	31	34	25	43	53	5	7	10	67	49	61	70	236	278	27	7
ACC_00618	conserved hypothetical protein		KOG0247	277	175	155	176	244	22	48	50	97	128	63	38	361	657	741	308
ACC_00619	brain protein 44-like		KOG1589	191	184	145	251	212	85	101	119	619	245	131	355	259	259	86	82
ACC_00620	apoptotic protease-activating factor 1-like		KOG4658	129	95	85	103	113	2	10	8	137	191	28	27	229	210	30	19
ACC_00621	cytoplasmic dynein 1 light intermediate chain 2	K10416	KOG3905	497	223	213	284	227	14	17	17	700	492	102	632	567	482	58	16
ACC_00622	conserved hypothetical protein			12	3	24	20	28	0	1	1	12	7	10	29	9	13	4	0
ACC_00623	LOW QUALITY PROTEIN	K15199	KOG4560	1266	811	650	957	1131	109	295	340	1239	1082	610	347	617	642	375	148
ACC_00624	sine oculis-binding protein homolog			72	77	44	96	92	8	41	44	133	214	25	39	102	52	56	38

ACC_00625	golgin-45-like		KOG4074	263	157	142	458	453	26	81	85	323	275	279	315	555	622	174	132
ACC_00626	LIM and senescent cell antigen-like-containing domain prote		KOG2272	229	138	144	356	325	29	47	44	495	289	246	438	347	323	57	27
ACC_00627	cyclin-dependent kinases regulatory subunit 1-like	K02219	KOG3484	23	24	21	40	29	0	5	4	23	18	28	47	46	35	7	3
ACC_00628	protein FAM192A-like	K02993	KOG3320	1763	1456	871	2280	1708	451	967	1086	1649	1262	2222	3035	5780	6991	2442	1365
ACC_00629	conserved hypothetical protein			14	7	8	11	16	0	3	6	62	198	4	19	75	16	8	3
ACC_00630	vesicular glutamate transporter 2		KOG2532	27	16	16	30	21	4	9	8	36	29	15	8	20	13	6	1
ACC_00631	SAP domain-containing ribonucleoprotein-like isoform 1		KOG4259	338	222	213	538	497	40	98	116	324	216	366	428	357	544	233	245
ACC_00632	putative hydroxypyruvate isomerase-like	K01816	KOG4518	116	121	100	88	118	12	24	32	535	374	89	209	2052	2491	96	53
ACC_00633	t-complex protein 1 subunit theta-like	K09500	KOG0362	1136	698	614	1290	1086	108	168	200	1588	1320	792	1606	4656	6121	601	319
ACC_00634	LOW QUALITY PROTEIN	K14163	KOG1147	2608	1813	1192	1504	1665	472	1487	1458	1656	1901	910	723	5588	5955	3642	2785
ACC_00635	LOW QUALITY PROTEIN	K00649	KOG3730	345	186	181	230	222	8	9	8	332	405	88	291	342	175	6	4
ACC_00636	conserved hypothetical protein	K15326	KOG4772	389	194	188	327	428	21	78	81	335	308	317	363	667	563	185	79
ACC_00637	conserved hypothetical protein			42	37	45	60	78	1	2	1	43	29	50	189	16	7	2	1
ACC_00638	LOW QUALITY PROTEIN	K01104	KOG4228	205	134	89	176	79	36	47	50	375	712	58	156	299	170	116	53
ACC_00639	nitrilase and fragile histidine triad fusion protein NitFhit-like		KOG0807	267	242	215	437	386	24	71	74	246	157	124	322	362	446	51	22
ACC_00640	slit homolog 2 protein-like		KOG4194	560	105	132	1053	373	28	24	69	435	526	12	11	7	2	11	3
ACC_00641	electron transfer flavoprotein subunit alpha, mitoc	K03522	KOG3954	207	138	103	293	298	12	16	28	667	586	335	1102	2969	2615	186	67
ACC_00642	39S ribosomal protein L22, mitochondrial isoform 1	K02890	KOG1711	319	371	392	434	423	46	86	104	350	192	273	506	933	1095	155	105
ACC_00643	conserved hypothetical protein		KOG3598	588	278	95	149	74	194	339	256	4428	2052	129	142	636	577	532	454
ACC_00644	conserved hypothetical protein		KOG0161	384	308	318	743	703	28	123	127	243	247	218	86	242	351	360	215
ACC_00645	apidaecins type 22 precursor		KOG0566	41	160	959	157	103	287	133	87	19023	27175	29	13	10	10	59	21
ACC_00646	conserved hypothetical protein		KOG0161	12	12	13	50	22	6	16	7	271	171	10	14	118	136	49	73
ACC_00647	conserved hypothetical protein	K15211		86	66	54	213	144	12	12	12	60	67	71	230	234	203	68	39
ACC_00648	DNA primase large subunit-like	K02685	KOG2267	152	91	72	81	126	11	18	16	216	109	67	90	313	545	66	19
ACC_00649	DC-STAMP domain-containing protein 2		KOG3726	21	9	6	13	12	5	6	6	35	36	3	8	4	3	6	6
ACC_00650	conserved hypothetical protein			83	75	75	34	15	3	5	10	47	41	12	4	917	716	120	7
ACC_00651	conserved hypothetical protein		KOG1090	108	98	77	125	96	11	13	18	148	113	85	91	21	5	3	1
ACC_00652	3-phosphoinositide-dependent protein kinase 1-lik	K06276	KOG0592	424	194	318	516	108	44	41	72	780	1012	176	166	95	85	379	154
ACC_00653	zinc transporter ZIP3-like	K14709	KOG1558	27	21	21	45	31	3	3	7	8	105	2	8	287	248	28	0
ACC_00654	pyruvate dehydrogenase E1 component subunit be	K00162	KOG0524	146	72	73	180	148	14	14	23	312	92	129	195	101	140	10	4
ACC_00655	protein polybromo-1	K11757	KOG1827	943	825	662	873	512	134	210	275	1139	1087	461	436	821	1073	373	201
ACC_00656	PAP-associated domain-containing protein 5-like	K03514	KOG1906	103	52	41	92	80	6	2	5	120	120	19	64	225	262	23	3
ACC_00657	Fanconi anemia group D2 protein homolog	K10891	KOG4712	1069	651	597	923	1160	77	225	218	513	600	405	344	740	771	269	69
ACC_00658	transmembrane protein 53-like		KOG2521	543	237	285	673	329	9	15	17	247	109	26	43	3	14	9	2
ACC_00659	inhibitor of nuclear factor kappa-B kinase subunit a	K07209	KOG4250	269	204	195	171	265	12	21	14	193	340	136	395	437	167	13	6
ACC_00660	BUD13 homolog	K13106	KOG2654	2282	1462	1728	2223	2413	438	854	1029	1392	461	699	445	587	1030	1422	919
ACC_00661	conserved hypothetical protein		KOG1454	113	43	48	174	61	10	12	11	96	84	41	134	40	104	54	13
ACC_00662	RNA polymerase II, large subunit		KOG0260	1	1	1	0	1	1	0	1	3	0	0	0	2	1	2	4
ACC_00663	conserved hypothetical protein		KOG2302	1	2	0	0	0	0	0	0	0	6	1	1	0	5	1	16
ACC_00664	conserved hypothetical protein		KOG4788	274	211	250	192	180	20	29	31	421	220	231	1301	204	188	24	9
ACC_00665	aminotransferase SSO0104-like		KOG0634	143	135	117	219	258	30	37	45	532	638	274	1293	503	447	66	33
ACC_00666	conserved hypothetical protein			3	0	2	1	2	0	1	0	16	15	4	7	2	4	2	2
ACC_00667	protein transport protein Sec61 subunit alpha isofc	K10956	KOG1373	671	554	212	396	390	88	87	140	852	859	277	676	4337	3643	144	51
ACC_00668	EF-hand domain-containing protein KIAA0494-like		KOG0161	801	555	488	1015	586	127	182	303	487	353	1088	1275	394	335	229	62
ACC_00669	mitogen-activated protein kinase 14B isoform 1	K04441	KOG0660	309	166	155	358	296	27	33	52	551	291	222	561	410	324	38	17
ACC_00670	plasma membrane calcium-transporting ATPase 3			872	462	477	465	145	83	119	144	362	269	115	65	102	86	250	103
ACC_00671	heparan-sulfate 6-O-sulfotransferase 2		KOG3955	66	40	54	74	27	4	3	9	273	406	12	168	155	79	20	13
ACC_00672	conserved hypothetical protein		KOG3627	1011	1126	707	140	87	311	366	427	1656	1848	45	248	371	614	262	181
ACC_00673	aldehyde oxidase 2-like	K00106	KOG0430	4643	6248	3199	1587	1652	2005	3634	3598	15541	12123	14504	30275	1213	225	28	14
ACC_00674	protein takeout-like			86	2	1	3	7	1	0	3	563	1586	368	3060	649	172	13	3
ACC_00675	VPS33B-interacting protein		KOG4677	232	182	185	215	223	21	45	59	334	154	202	330	167	208	34	22
ACC_00676	60S ribosomal protein L13a isoform 2	K02872	KOG3204	2048	1603	729	2629	1619	557	895	1100	1809	1776	2400	3151	7503	10123	4285	2353
ACC_00677	protein Hook homolog 3-like isoform 1	K16536	KOG0161	420	270	287	428	518	81	110	165	439	349	201	175	196	170	115	57
ACC_00678	protein BTG3-like	K14443	KOG4006	489	257	175	221	115	81	151	184	798	608	152	799	859	889	580	370
ACC_00679	erythroid differentiation-related factor 1-like			362	267	263	550	445	50	99	109	514	379	224	283	293	325	213	87
ACC_00680	alpha-tocopherol transfer protein-like		KOG1471	126	96	98	176	210	9	12	28	204	146	68	148	44	44	2	4
ACC_00681	kinesin F		KOG4280	130	105	162	120	96	35	66	69	344	77	134	60	51	39	17	16

ACC_00682	catenin alpha	K05691	KOG3681	293	158	140	220	167	23	46	45	405	434	100	335	321	268	98	38
ACC_00683	nucleolar complex protein 3 homolog	K14834	KOG2153	477	275	263	424	605	19	97	94	205	238	248	183	749	954	571	403
ACC_00684	tektin-3-like		KOG2685	10	9	20	9	16	0	4	5	35	13	18	4	43	47	11	13
ACC_00685	heparanase-like	K07964		765	447	453	747	867	37	61	100	661	595	308	685	1463	1140	93	41
ACC_00686	conserved hypothetical protein	K11321	KOG1474	743	387	340	573	534	136	262	299	567	403	344	317	448	580	593	350
ACC_00687	AN1-type zinc finger protein 1-like		KOG3183	267	172	163	471	549	22	28	32	565	308	285	786	1022	1093	113	21
ACC_00688	astakine-like			42	74	28	44	46	29	48	54	2500	672	775	1296	164	114	14	12
ACC_00689	myophilin-like		KOG2046	107	67	21	197	96	44	82	74	4297	752	157	206	1252	316	111	75
ACC_00690	protein tipE-like			886	270	325	1449	350	27	46	78	224	96	163	50	0	3	14	5
ACC_00691	conserved hypothetical protein			1167	352	401	1895	379	38	44	134	228	97	209	48	0	2	14	13
ACC_00692	probable nuclear hormone receptor HR3-like	K14033	KOG4216	267	148	123	240	65	11	14	25	49	52	38	13	19	52	179	102
ACC_00693	protein quiver-like			454	189	203	80	42	24	38	41	478	1040	9	24	14	5	5	3
ACC_00694	LOW QUALITY PROTEIN	K13288	KOG3242	92	73	77	136	180	1	10	5	21	35	79	87	83	84	4	4
ACC_00695	protein lethal(2)essential for life-like isoform 1		KOG3591	193	540	2515	5082	2449	129	107	215	3100	1077	277	498	10	15	3	12
ACC_00696	LOW QUALITY PROTEIN		KOG1232	123	110	75	171	128	37	40	56	517	419	161	358	1441	1662	172	34
ACC_00697	DNA-directed RNA polymerase III subunit RPC7-like	K03024	KOG1834	552	394	422	455	477	137	281	257	453	246	345	369	697	711	944	898
ACC_00698	protein disulfide-isomerase A3 isoform 2	K08056	KOG0190	1643	1555	773	1797	1895	351	330	465	1771	1845	625	2362	10783	9399	508	131
ACC_00699	LOW QUALITY PROTEIN	K10413	KOG3595	3330	1780	1606	3075	1698	302	491	522	5121	5896	765	1344	2066	1865	475	188
ACC_00700	conserved hypothetical protein		KOG4818	295	114	132	203	105	4	5	4	206	130	15	42	4	10	2	0
ACC_00701	conserved hypothetical protein	K04700	KOG4637	597	290	283	771	613	31	57	57	1048	651	238	1003	632	739	90	23
ACC_00702	TBC1 domain family member 20-like		KOG2595	249	157	143	220	196	19	19	19	266	169	77	277	200	215	25	6
ACC_00703	RING-box protein 2	K10611	KOG2930	109	66	58	135	109	15	21	24	115	94	33	83	109	166	39	18
ACC_00704	conserved hypothetical protein			68	65	16	29	30	18	22	17	131	45	14	99	391	337	57	26
ACC_00705	CKLF-like MARVEL transmembrane domain-containing protein 4-like			93	15	9	42	22	4	3	3	263	101	80	290	73	97	63	3
ACC_00706	WD repeat-containing protein 63		KOG1587	23	9	3	7	1	2	3	0	154	20	17	4	11	13	5	8
ACC_00707	CKLF-like MARVEL transmembrane domain-containing protein 4-like			53	13	18	21	10	1	3	3	111	55	46	176	24	36	24	2
ACC_00708	conserved hypothetical protein		KOG0379	214	124	166	185	52	11	9	15	219	186	33	10	6	8	17	13
ACC_00709	probable malate dehydrogenase, mitochondrial-lik	K00026	KOG1494	3	1	1	5	6	0	1	1	2	0	1	0	1	0	0	0
ACC_00710	DNA repair protein REV1	K03515	KOG2093	564	375	310	598	659	84	169	178	549	335	388	344	510	521	280	80
ACC_00711	mitochondrial 2-oxoglutarate/malate carrier protein-like		KOG0759	149	81	80	57	53	6	10	5	201	132	39	183	465	267	9	6
ACC_00712	GTP-binding protein CG1354-like	K06942	KOG1491	260	216	162	449	156	21	35	38	289	274	184	267	498	653	251	103
ACC_00713	GTP-binding protein ypt7-like		KOG0098	79	70	74	131	88	5	11	9	113	44	72	209	63	69	18	8
ACC_00714	methylcrotonoyl-CoA carboxylase subunit alpha, m	K01968	KOG0238	684	493	478	755	739	34	81	83	828	1209	220	291	3602	2282	117	37
ACC_00715	conserved hypothetical protein			4818	1524	1855	5664	1834	419	736	972	1359	877	1214	1016	3	23	141	129
ACC_00716	LOW QUALITY PROTEIN	K00604	KOG3082	205	117	110	164	219	17	29	37	215	114	105	300	328	376	65	24
ACC_00717	xanthine dehydrogenase	K00106	KOG0430	1047	853	931	1774	1728	102	154	159	2553	4460	401	695	1433	1273	174	52
ACC_00718	zinc finger protein 568-like		KOG2462	212	136	123	196	157	18	21	32	350	331	122	216	282	250	28	12
ACC_00719	leukocyte receptor cluster member 1-like			374	253	186	262	306	57	141	178	165	117	155	130	212	288	542	412
ACC_00720	transmembrane protein 55B-like	K13084	KOG4684	253	143	156	440	288	10	15	35	310	186	119	466	289	386	104	41
ACC_00721	Putative esterase CG3488	K13697	KOG1838	31	24	19	49	33	2	3	4	324	494	95	355	334	319	9	0
ACC_00722	conserved hypothetical protein	K14856	KOG2229	1839	929	841	1243	1342	222	616	626	1033	926	722	575	2747	3486	2981	2044
ACC_00723	PCTP-like protein-like		KOG2761	32	27	17	49	62	1	1	2	37	29	19	44	106	97	12	5
ACC_00724	conserved hypothetical protein			895	466	429	814	1313	34	126	107	448	442	429	472	400	338	296	126
ACC_00725	hypothetical protein			194	86	87	294	396	13	17	31	185	74	129	405	127	164	23	11
ACC_00726	lysozyme isoform 1			321	493	318	465	336	41	70	81	544	225	554	1454	1339	869	235	147
ACC_00727	general vesicular transport factor p115		KOG0946	1126	713	609	940	935	141	264	311	1438	1060	509	753	1167	1143	176	96
ACC_00728	microfibrillar-associated protein 1	K13110	KOG1425	658	515	548	625	676	154	342	332	538	281	438	509	477	600	625	543
ACC_00729	UPF0729 protein			61	39	32	101	91	1	7	5	19	20	26	116	81	112	9	8
ACC_00730	importin-7		KOG1991	694	505	408	967	739	88	128	175	1225	1521	524	874	3596	4185	587	190
ACC_00731	conserved hypothetical protein			2950	1662	1720	1102	1557	280	930	774	2020	858	1158	364	459	471	432	137
ACC_00732	glyceraldehyde-3-phosphate dehydrogenase 2 isof	K00134	KOG0657	8988	3196	3420	7214	6549	369	275	499	14682	7338	3322	15336	24433	18868	1572	508
ACC_00733	zinc finger with UFM1-specific peptidase domain protein-like		KOG4696	520	209	216	461	308	32	55	84	407	222	146	192	88	119	45	25
ACC_00734	probable ATP-dependent RNA helicase DDX46-like	K12811	KOG0334	815	611	636	1125	545	154	222	300	1270	820	546	480	647	1113	1243	1122
ACC_00735	conserved hypothetical protein			156	161	116	275	243	43	93	111	158	143	149	119	203	268	201	94
ACC_00736	conserved hypothetical protein		KOG0971	194	152	164	314	115	53	65	70	750	488	133	151	98	85	156	85
ACC_00737	PTB domain-containing adapter protein ced-6		KOG3536	1542	1329	1608	4126	2595	107	148	202	4221	9544	868	5638	653	489	96	36
ACC_00738	serine/threonine-protein phosphatase 6 regulatory	K15502	KOG4177	71	58	31	58	37	7	10	16	253	267	38	124	250	254	29	8

ACC_00739	high mobility group protein 20A-like isoform 1		KOG0381	155	88	81	287	184	13	31	38	124	64	81	154	153	159	88	52
ACC_00740	odorant receptor 77			58	41	90	55	79	3	11	8	88	41	31	104	21	31	2	0
ACC_00741	brahma associated protein 55kd	K11652	KOG0679	120	61	67	143	134	6	13	14	162	113	82	198	280	476	27	18
ACC_00742	conserved hypothetical protein			10	11	4	15	5	2	4	2	209	170	27	146	65	49	64	32
ACC_00743	conserved hypothetical protein			4	3	0	9	6	0	1	0	0	0	2	1	27	26	3	0
ACC_00744	tyrosyl-tRNA synthetase, cytoplasmic isoform 1	K01866	KOG2144	805	476	372	639	687	96	157	205	908	563	370	1058	3878	3543	450	197
ACC_00745	potassium/sodium hyperpolarization-activated cyclic nucleo		KOG0498	69	39	28	37	45	3	6	2	67	54	29	118	102	86	6	5
ACC_00746	vacuolar protein sorting-associated protein 41 homolog		KOG2066	198	118	126	191	207	11	10	18	324	251	84	138	169	161	12	7
ACC_00747	ATP synthase subunit beta, mitochondrial-like	K02133	KOG1350	3882	1442	1297	4044	2696	268	308	393	8418	4506	1846	7306	12563	12710	1423	496
ACC_00748	lysosomal acid phosphatase-like isoform 1		KOG3720	261	231	247	306	331	34	56	66	1041	397	879	1754	195	194	32	6
ACC_00749	replication factor C subunit 3	K10756	KOG2035	123	101	110	142	129	6	24	20	146	61	65	124	273	517	51	26
ACC_00750	ras association domain-containing protein 8-like	K09855	KOG1574	318	165	185	332	226	24	46	44	245	164	96	189	175	141	64	43
ACC_00751	conserved hypothetical protein	K10631	KOG4430	368	241	257	430	298	52	123	155	540	329	194	316	411	449	300	136
ACC_00752	protein 5NUC-like	K01081	KOG4419	133	265	133	319	211	221	226	336	240	145	334	497	3001	3011	470	48
ACC_00753	conserved hypothetical protein			1212	337	472	936	295	43	54	78	582	309	108	346	24	101	167	77
ACC_00754	conserved hypothetical protein	K09280	KOG3982	47	22	36	60	16	4	2	8	12	17	0	0	3	7	38	23
ACC_00755	solute carrier family 28 member 3-like		KOG3747	224	113	83	170	203	21	33	52	248	236	642	683	651	585	51	9
ACC_00756	NAD kinase-like isoform 1	K00858	KOG2178	281	120	108	407	188	66	66	56	1983	2495	1787	6457	614	295	117	63
ACC_00757	protein tyrosine phosphatase type IVA 1	K01104	KOG2836	459	279	337	957	431	42	72	105	1412	641	160	882	434	387	184	89
ACC_00758	conserved hypothetical protein			5	2	17	58	31	2	1	1	20	29	6	2	4	58	91	56
ACC_00759	39S ribosomal protein L2, mitochondrial	K02886	KOG0438	179	107	109	454	169	42	65	76	282	165	178	243	577	558	178	126
ACC_00760	negative elongation factor A-like	K15179	KOG0260	298	188	246	257	154	43	27	59	349	274	96	251	293	354	151	53
ACC_00761	conserved hypothetical protein		KOG3002	153	61	56	145	145	9	22	21	86	27	17	44	304	116	15	22
ACC_00762	oxysterol-binding protein-related protein 11-like		KOG1739	144	97	79	157	131	6	2	12	88	76	31	148	235	196	27	2
ACC_00763	conserved hypothetical protein		KOG2039	259	161	147	225	191	7	31	29	196	294	96	117	662	869	215	117
ACC_00764	ubiquitin-conjugating enzyme E2 N	K10580	KOG0417	115	80	57	137	104	11	10	24	269	307	68	415	457	338	61	17
ACC_00765	conserved hypothetical protein	K11095	KOG3454	192	155	173	244	159	21	14	22	106	95	110	288	225	367	135	125
ACC_00766	hypothetical protein			0	0	0	0	0	0	0	0	0	0	0	0	2	0	0	0
ACC_00767	LOW QUALITY PROTEIN	K03164	KOG0355	1632	997	682	1365	1141	285	521	553	965	932	745	453	768	1165	2718	1835
ACC_00768	nucleoside diphosphate kinase 6-like	K00940	KOG0888	65	44	53	72	88	14	12	16	196	85	80	328	140	169	38	7
ACC_00769	neurocalcin homolog		KOG0044	492	286	260	221	108	24	33	31	405	188	90	193	51	63	34	17
ACC_00770	conserved hypothetical protein			130	108	100	154	135	13	14	16	268	149	59	202	119	168	11	11
ACC_00771	guanine nucleotide-binding protein subunit alpha h	K04346	KOG0082	24	23	18	10	15	0	2	1	65	52	9	46	84	54	7	5
ACC_00772	conserved hypothetical protein		KOG0533	275	141	178	357	245	22	32	46	309	224	123	362	501	644	173	64
ACC_00773	conserved hypothetical protein		KOG0307	565	786	793	651	182	74	50	102	553	502	126	78	264	446	281	103
ACC_00774	solute carrier family 17 member 9-like	K12303	KOG2532	177	141	149	201	241	19	28	29	240	99	127	134	82	93	14	2
ACC_00775	sarcoplasmic calcium-binding protein isoform 1		KOG0036	52	54	60	89	82	4	8	5	15	21	11	20	84	84	35	9
ACC_00776	vacuolar protein sorting-associated protein 29-like	K07095	KOG3325	98	62	79	182	219	17	12	25	148	72	95	304	242	300	23	15
ACC_00777	protein crumbs-like	K16681	KOG4289	120	68	60	97	41	14	23	34	559	496	129	140	108	141	71	35
ACC_00778	CDGSH iron-sulfur domain-containing protein 2 homolog		KOG3461	187	103	82	400	374	10	15	28	409	160	317	669	836	774	84	35
ACC_00779	trifunctional purine biosynthetic protein adenosine	K11787	KOG0237	221	171	200	282	232	29	53	46	1056	2003	126	243	2599	2262	266	103
ACC_00780	ubiquitin-conjugating enzyme E2 G1	K10575	KOG0425	433	259	238	478	234	60	73	107	835	527	201	560	1429	1714	873	373
ACC_00781	mediator of RNA polymerase II transcription subun	K15135	KOG3264	45	50	33	120	92	4	6	9	65	47	24	44	180	203	24	22
ACC_00782	G-protein coupled receptor Mth2-like		KOG4193	53	31	33	104	30	5	9	4	148	129	25	95	43	113	27	0
ACC_00783	rotatin-like	K16484		202	151	129	264	278	18	42	45	325	316	109	99	297	258	66	19
ACC_00784	AP-1 complex subunit mu-1-like isoform 1	K12393	KOG0937	345	192	192	399	323	16	25	19	492	212	144	612	353	442	43	9
ACC_00785	la protein homolog	K11090	KOG4213	539	393	248	384	298	56	139	162	313	190	248	335	1523	2583	1733	1639
ACC_00786	ribosomal RNA-processing protein 7 homolog A-like	K14545	KOG4008	547	417	353	415	556	114	302	291	315	134	375	454	419	609	655	347
ACC_00787	FAD-linked sulfhydryl oxidase ALR isoform 1		KOG3355	134	137	145	186	178	15	8	16	130	95	88	212	157	155	35	5
ACC_00788	conserved hypothetical protein			95	66	51	120	110	5	13	12	86	43	36	35	113	83	34	19
ACC_00789	conserved hypothetical protein			49	40	23	39	46	5	9	14	377	122	86	653	469	585	43	31
ACC_00790	gram-negative bacteria-binding protein 1-2 precursor			27	20	14	48	28	15	19	17	4899	3278	277	56	7	47	4	6
ACC_00791	probable histone-lysine N-methyltransferase NSD2	K11424	KOG4442	327	173	129	288	245	46	87	92	564	337	180	246	570	713	419	159
ACC_00792	UPF0470 protein C19orf51			41	28	34	59	45	1	12	6	54	20	77	104	37	59	14	4
ACC_00793	conserved hypothetical protein		KOG0307	428	421	457	574	299	109	148	184	669	378	242	265	504	759	790	352
ACC_00794	myotubularin-related protein 9-like		KOG1089	364	325	331	352	280	62	65	73	395	210	169	166	336	393	103	48
ACC_00795	prolactin-releasing peptide receptor-like	K04209	KOG4219	66	25	17	40	41	11	10	8	40	26	169	175	16	16	33	7

ACC_00796	adenylate cyclase type 2-like	K08042	KOG3619	21	50	41	72	79	18	33	24	482	467	40	54	309	155	26	15
ACC_00797	histone-lysine N-methyltransferase E(z) isoform 1	K11430	KOG1079	622	456	491	792	438	140	225	321	964	596	561	515	554	616	350	231
ACC_00798	retinol dehydrogenase 10-A-like		KOG1201	19	25	16	201	96	17	30	33	703	298	1642	2588	353	157	53	18
ACC_00799	epidermal retinol dehydrogenase 2-like		KOG1201	0	0	0	1	0	0	0	0	3	3	11	15	3	1	0	0
ACC_00800	WD repeat-containing protein 35-like		KOG2041	117	105	76	68	68	7	11	16	125	103	80	131	75	69	9	3
ACC_00801	trafficking protein particle complex subunit 4-like		KOG3369	125	84	91	114	118	5	12	3	71	57	47	146	86	155	9	6
ACC_00802	probable ATP-dependent RNA helicase DDX43-like		KOG0336	271	171	216	258	179	25	58	86	246	131	79	147	174	301	166	112
ACC_00803	cytochrome c oxidase subunit VIb polypeptide 1	K02267	KOG3057	806	574	546	2066	1340	63	88	128	810	279	1271	1830	695	683	257	122
ACC_00804	conserved hypothetical protein		KOG0147	147	77	59	114	100	15	12	12	235	153	78	137	191	238	71	13
ACC_00805	2',5'-phosphodiesterase 12-like		KOG0620	175	113	140	154	230	8	15	18	239	177	202	485	486	622	58	24
ACC_00806	cAMP-dependent protein kinase type I regulatory s	K04739	KOG1113	593	603	540	1247	579	54	46	91	1110	734	260	699	610	625	96	23
ACC_00807	gamma-1-syntrophin-like		KOG3549	556	213	177	298	239	18	36	31	330	176	61	147	73	73	26	16
ACC_00808	gamma-1-syntrophin-like		KOG1519	180	93	100	204	195	4	10	15	165	106	62	233	247	227	23	4
ACC_00809	leucine carboxyl methyltransferase 1-like		KOG2918	535	69	72	78	102	4	7	13	350	221	33	215	411	269	16	4
ACC_00810	mitochondrial intermediate peptidase-like	K01410	KOG2090	393	267	238	451	526	16	18	45	454	360	164	395	489	696	58	8
ACC_00811	solute carrier organic anion transporter family member 5A1-		KOG3626	130	78	79	201	96	11	13	17	133	53	0	12	748	1045	447	43
ACC_00812	solute carrier organic anion transporter family member 5A1-		KOG3626	392	349	489	2654	1261	40	48	59	514	196	191	830	48	44	39	1
ACC_00813	conserved hypothetical protein			2	2	0	3	0	1	0	1	2	2	0	0	12	20	9	2
ACC_00814	mediator of RNA polymerase II transcription subun	K15143	KOG3598	107	109	110	178	62	24	25	44	221	67	58	95	189	299	278	105
ACC_00815	conserved hypothetical protein			118	42	73	320	142	24	39	33	280	286	24	96	859	138	93	55
ACC_00816	tyrosine-protein kinase Dnt	K05128	KOG1024	19	25	25	92	35	8	8	12	32	51	10	5	13	19	55	24
ACC_00817	putative ATP-dependent RNA helicase Pl10	K11594	KOG0335	2336	1517	880	3255	1883	735	1044	1165	3304	2104	2500	3909	11332	13129	4156	2586
ACC_00818	proline-, glutamic acid- and leucine-rich protein 1-like			148	104	94	146	150	6	24	22	110	162	75	103	498	601	116	60
ACC_00819	ras-related GTP-binding protein A	K16185	KOG3886	194	137	136	239	206	18	28	31	271	204	89	345	228	176	18	4
ACC_00820	conserved hypothetical protein			88	69	88	166	84	3	3	7	52	45	0	1	1	0	0	0
ACC_00821	1-phosphatidylinositol-3-phosphate 5-kinase	K00921	KOG0230	670	459	436	640	460	152	227	249	1408	746	517	283	266	210	80	40
ACC_00822	cytochrome c	K08738	KOG3453	777	307	247	1259	975	60	82	90	1147	413	443	1300	1183	2193	861	415
ACC_00823	meiotic nuclear division protein 1 homolog		KOG3433	21	11	16	7	23	5	5	2	22	16	22	21	21	49	24	14
ACC_00824	bifunctional 3'-phosphoadenosine 5'-phosphosulfa	K13811	KOG4238	369	276	264	340	300	49	88	95	577	422	178	401	551	482	112	64
ACC_00825	nuclear nucleic acid-binding protein C1D-like isofor	K12592	KOG4835	175	132	126	273	316	29	102	132	254	102	342	441	233	444	206	152
ACC_00826	tryptophan-rich protein-like		KOG4253	108	53	62	114	182	6	12	9	150	118	77	288	301	311	15	5
ACC_00827	conserved hypothetical protein			14	19	26	24	35	1	5	2	15	2	9	9	3	6	1	0
ACC_00828	probable actin-related protein 2/3 complex subuni	K05758	KOG2826	365	229	209	257	300	18	27	44	557	279	142	664	620	739	40	12
ACC_00829	probable glutamyl-tRNA synthetase, mitochondrial	K01885	KOG1149	126	76	81	127	125	9	11	20	134	84	65	201	308	414	31	19
ACC_00830	programmed cell death protein 5-like	K06875	KOG3431	166	111	114	230	226	17	22	21	141	77	153	240	191	345	117	58
ACC_00831	conserved hypothetical protein			96	58	72	202	133	10	24	35	63	23	101	53	83	297	180	114
ACC_00832	WD and tetratricopeptide repeats protein 1-like	K11807	KOG1310	479	291	256	777	657	55	74	78	492	427	318	302	529	601	61	17
ACC_00833	tRNA selenocysteine 1-associated protein 1-like		KOG0144	211	157	169	327	276	18	28	28	311	184	154	486	416	770	68	20
ACC_00834	leucine-rich repeat protein 1-like	K10348	KOG0532	102	110	85	179	209	5	6	8	62	74	51	145	103	87	13	2
ACC_00835	PTEN-like phosphatase	K14165	KOG1719	247	180	197	275	332	26	43	55	277	151	236	396	412	380	47	21
ACC_00836	mitochondrial GTPase 1-like		KOG2485	203	147	113	202	213	8	34	28	215	126	161	146	170	223	13	6
ACC_00837	conserved hypothetical protein			860	509	417	861	612	130	250	287	1396	691	617	840	1017	484	287	126
ACC_00838	sodium channel protein paralytic	K05388	KOG2301	2317	975	1054	3727	1053	160	268	325	751	357	819	111	21	29	56	12
ACC_00839	probable 28S ribosomal protein S25, mitochondrial-like		KOG4079	206	135	164	323	358	19	42	48	143	88	142	285	279	521	140	88
ACC_00840	aspartate--tRNA ligase, mitochondrial-like	K01876	KOG2411	404	253	306	684	643	32	62	73	593	349	257	430	630	804	86	32
ACC_00841	conserved hypothetical protein		KOG1738	1067	765	586	729	677	194	420	499	1050	970	432	373	777	836	666	307
ACC_00842	cell division control protein 31-like		KOG0028	38	41	34	18	35	4	6	5	26	17	16	31	25	44	8	1
ACC_00843	splicing factor 45-like	K12840	KOG1996	251	208	293	279	198	33	46	52	189	130	139	170	320	562	149	89
ACC_00844	Cytochrome b			3	2	3	2	1	0	1	1	0	2	0	0	0	0	1	0
ACC_00845	jmjC domain-containing protein 4-like isoform 1		KOG2131	86	62	81	232	260	10	7	11	171	94	171	288	140	131	11	6
ACC_00846	activating molecule in BECN1-regulated autophagy protein 1		KOG0266	439	286	282	474	451	40	62	96	284	272	220	344	490	598	77	25
ACC_00847	UTP--glucose-1-phosphate uridylyltransferase isofc	K00963	KOG2638	733	442	401	1076	1040	104	141	186	4169	2259	2302	2853	5447	2213	194	88
ACC_00848	macoilin-like		KOG1821	266	157	134	242	71	27	66	76	285	348	54	86	166	101	124	74
ACC_00849	eukaryotic translation initiation factor 4E-binding p	K07205		415	566	513	552	322	84	115	131	1172	531	309	889	1180	1138	292	124
ACC_00850	eukaryotic initiation factor 4A-like isoform 2	K03257	KOG0327	2868	2096	1475	4255	3079	656	1072	1135	7948	6917	3148	9988	12647	11207	2999	814
ACC_00851	activating signal cointegrator 1 complex subunit 1-like		KOG2814	374	267	246	441	501	15	45	38	611	478	233	714	561	549	90	34
ACC_00852	conserved hypothetical protein	K11423	KOG4442	1913	1517	1415	2070	2035	435	931	956	1333	1113	1112	496	478	538	1087	574

ACC_00853	LOW QUALITY PROTEIN	K12815	KOG0924	650	538	539	671	486	109	199	226	586	443	221	220	460	499	409	291
ACC_00854	CUE domain-containing protein 1		KOG4588	256	157	177	281	233	15	20	21	320	178	105	439	98	103	14	4
ACC_00855	LOW QUALITY PROTEIN	K14546	KOG0366	606	339	372	816	674	64	126	166	785	629	344	741	1608	2524	1075	735
ACC_00856	hexamerin			971	353	59	208	111	265	381	287	688	87	8	41	58701	684	12	15
ACC_00857	Cytidylate kinase			0	0	0	1	0	0	0	0	1	2	0	0	20	2	0	0
ACC_00858	LOW QUALITY PROTEIN			201	83	81	150	96	15	21	31	241	226	63	122	295	715	118	53
ACC_00859	tubulin-specific chaperone D-like		KOG1943	420	280	278	367	381	19	32	33	585	486	191	484	397	257	17	4
ACC_00860	mediator of RNA polymerase II transcription subun	K15156	KOG1875	385	324	297	384	201	35	24	34	357	409	78	115	244	245	50	15
ACC_00861	ubiquinol-cytochrome c reductase complex chaperone CBP3		KOG2873	152	137	138	291	394	13	14	19	116	72	167	229	180	323	14	7
ACC_00862	UPF0551 protein C8orf38, mitochondrial-like		KOG4411	53	42	41	65	87	3	0	0	46	35	26	43	61	49	0	1
ACC_00863	guanine nucleotide-binding protein subunit beta-like proteir		KOG0322	66	50	53	62	127	3	4	10	84	51	73	199	94	87	8	3
ACC_00864	TRAF-interacting protein-like		KOG0827	268	225	188	270	422	29	103	147	93	54	155	133	167	255	326	159
ACC_00865	annexin-B9-like		KOG0819	1249	748	732	3411	2329	130	136	194	2756	1171	1333	3605	1623	1487	293	261
ACC_00866	lachesin-like		KOG3513	73	40	33	24	8	0	2	3	35	37	1	12	41	13	10	0
ACC_00867	conserved hypothetical protein			1	0	1	0	0	0	0	0	1	1	0	0	0	2	1	0
ACC_00868	L-allo-threonine aldolase-like	K01620	KOG1368	95	53	58	123	107	4	7	9	335	235	39	224	145	172	13	2
ACC_00869	26S proteasome non-ATPase regulatory subunit 10	K06694	KOG4412	197	78	63	280	191	13	38	27	179	113	127	365	400	504	148	102
ACC_00870	Dynein beta chain, ciliary		KOG3595	8	4	8	40	11	2	3	3	77	628	7	24	53	223	18	60
ACC_00871	Ubiquitin-conjugating enzyme E2-24 kDa	K06689	KOG0417	81	63	53	53	35	0	3	7	149	152	15	53	100	160	33	5
ACC_00872	Serine/threonine-protein phosphatase 4 catalytic s	K15423	KOG0372	242	204	165	428	346	30	41	57	359	284	127	382	642	665	145	42
ACC_00873	LOW QUALITY PROTEIN		KOG1215	344	242	210	286	168	14	27	44	371	364	73	107	308	346	117	42
ACC_00874	dnaJ homolog subfamily B member 11-like	K09517	KOG0713	492	392	301	510	501	88	77	119	670	548	241	743	1941	2365	272	124
ACC_00875	ribosome maturation protein SBDS-like	K14574	KOG2917	196	91	118	267	254	29	61	61	205	107	245	284	360	581	206	132
ACC_00876	zinc finger HIT domain-containing protein 3-like		KOG2857	75	56	46	93	112	5	14	24	63	23	78	113	106	135	24	16
ACC_00877	conserved hypothetical protein		KOG2412	437	213	215	788	1000	94	168	196	518	322	368	449	242	211	369	220
ACC_00878	ubiquitin carboxyl-terminal hydrolase 30-like	K11851	KOG1867	233	102	109	231	281	12	28	23	378	229	207	215	272	263	20	2
ACC_00879	BTB/POZ domain-containing protein 19-like		KOG4350	34	31	21	25	24	7	6	9	19	8	20	8	35	44	18	14
ACC_00880	hypothetical protein			0	0	0	0	0	0	0	0	0	0	0	0	0	0	0	0
ACC_00881	venom dipeptidyl peptidase 4-like	K01278	KOG2100	362	178	152	349	113	38	83	103	250	146	32	136	88	77	120	30
ACC_00882	heat shock factor-binding protein 1-like isoform 2		KOG4117	208	247	193	376	221	26	38	38	175	74	248	257	346	464	117	106
ACC_00883	serine/threonine-protein phosphatase 2A 56 kDa r	K11584	KOG2085	671	549	584	884	511	56	80	91	843	739	146	336	415	494	243	70
ACC_00884	dynein light chain 1, axonemal-like	K10411	KOG0531	47	37	45	47	62	0	9	8	41	21	25	44	57	85	10	3
ACC_00885	general transcription factor IIH subunit 3	K03143	KOG2487	123	112	124	224	214	13	24	26	166	80	111	362	157	220	49	21
ACC_00886	14-3-3 protein epsilon-like	K06630	KOG0841	4766	1803	1695	8602	2996	570	782	1257	2077	1428	1302	1535	2601	2594	3849	2450
ACC_00887	calcium release-activated calcium channel protein :	K16056	KOG4298	38	8	20	73	33	6	10	19	331	216	75	493	130	71	40	6
ACC_00888	NAD-dependent deacetylase sirtuin-6	K11416	KOG1905	227	153	152	309	184	27	52	74	177	162	60	107	127	181	133	70
ACC_00889	proton-coupled amino acid transporter 4-like	K14209	KOG1304	121	154	72	83	34	112	100	77	511	377	22	146	187	155	70	3
ACC_00890	transcription elongation factor SPT4 isoform 2	K15171	KOG3490	326	219	238	485	393	53	134	168	350	110	189	281	419	571	222	68
ACC_00891	conserved hypothetical protein		KOG0517	347	285	324	438	210	178	308	253	6347	3252	357	312	274	234	217	147
ACC_00892	SEC14-like protein 2-like		KOG1471	47	68	71	135	126	42	55	80	468	258	987	2197	1113	909	203	144
ACC_00893	conserved hypothetical protein			597	567	457	1452	877	165	253	343	1147	518	676	2506	530	339	81	58
ACC_00894	NADH dehydrogenase	K03936	KOG1713	655	359	424	1026	801	43	62	90	2012	458	396	1323	1495	1905	115	58
ACC_00895	MMS19 nucleotide excision repair protein homolog	K15075	KOG1967	572	369	371	977	713	311	526	714	2454	930	175	449	621	695	54	11
ACC_00896	inner nuclear membrane protein Man1-like			392	237	185	453	336	38	111	107	469	447	199	362	478	682	352	277
ACC_00897	conserved hypothetical protein		KOG0960	176	188	191	254	232	14	19	29	188	131	77	203	200	209	39	10
ACC_00898	conserved hypothetical protein		KOG2462	110	83	77	142	39	6	10	10	157	180	23	95	110	118	26	21
ACC_00899	ninjurin-1-like			42	14	18	6	14	3	20	22	57	103	12	14	299	100	93	52
ACC_00900	calpain-7	K08576	KOG0045	142	87	63	153	135	7	20	23	261	260	62	295	315	215	18	2
ACC_00901	checkpoint protein HUS1-like		KOG3999	33	27	27	71	50	2	12	15	23	21	24	24	45	50	12	10
ACC_00902	conserved hypothetical protein			260	172	134	335	201	26	39	55	341	127	195	500	369	394	176	67
ACC_00903	hypothetical protein			101	35	15	28	21	10	63	58	16	22	30	56	107	168	150	115
ACC_00904	diphosphomevalonate decarboxylase-like	K01597	KOG2833	169	112	93	200	221	16	31	30	338	208	113	246	424	409	46	36
ACC_00905	BTB/POZ domain-containing protein KCTD16-like		KOG2723	92	39	41	41	15	3	0	1	43	40	0	0	3	10	1	0
ACC_00906	mitochondrial uncoupling protein 2-like	K15103	KOG0753	2155	1498	1919	5897	2135	335	544	694	2026	1301	2676	1206	675	781	2361	613
ACC_00907	nuclear pore membrane glycoprotein 210-like	K14314	KOG1833	215	185	112	188	156	14	17	34	246	429	78	89	924	1053	77	11
ACC_00908	LOW QUALITY PROTEIN	K15013	KOG1256	260	232	174	332	321	58	83	64	2476	2388	4522	7103	10861	4500	526	320
ACC_00909	conserved hypothetical protein			1202	1542	2972	7028	4136	93	93	116	1170	856	225	1054	4	3	1	0

ACC_00910	conserved hypothetical protein	K14840	KOG2823	1253	749	614	953	1063	226	620	690	775	518	674	500	803	1519	1721	1230
ACC_00911	LOW QUALITY PROTEIN	K12818	KOG0922	284	207	245	431	259	48	69	88	550	395	226	397	321	318	84	44
ACC_00912	SCY1-like protein 2-like		KOG2137	405	303	340	404	338	23	48	51	480	384	99	419	516	473	43	3
ACC_00913	conserved hypothetical protein			169	79	100	150	222	5	9	7	82	16	65	56	4	3	1	1
ACC_00914	conserved hypothetical protein	K11459	KOG2660	3481	2419	2181	3486	2751	539	1315	1330	3010	2199	1721	768	1083	1435	1575	535
ACC_00915	NADH dehydrogenase	K03937	KOG3389	271	146	206	549	504	13	31	38	329	161	366	631	332	433	45	26
ACC_00916	TATA-box-binding protein-like isoform 1	K03120	KOG3302	240	233	197	315	167	41	83	87	176	135	141	165	175	205	342	206
ACC_00917	conserved hypothetical protein		KOG2106	2470	1283	1287	2342	1792	395	887	933	1402	860	665	286	394	579	2529	3188
ACC_00918	alpha N-terminal protein methyltransferase 1A-like	K16219	KOG3178	408	365	307	447	445	79	120	181	544	319	312	542	1139	1680	298	129
ACC_00919	tudor domain-containing protein 12-like		KOG0331	271	152	184	179	261	13	20	19	102	143	180	128	118	207	63	31
ACC_00920	39S ribosomal protein L42, mitochondrial-like		KOG4106	369	240	236	788	616	41	90	115	267	236	429	822	1043	1169	245	88
ACC_00921	kelch domain-containing protein 4-like, partial		KOG1230	585	277	233	570	631	101	239	265	574	513	506	662	926	969	1104	927
ACC_00922	LOW QUALITY PROTEIN	K02212	KOG0478	221	169	177	276	233	9	23	24	228	219	109	275	458	659	77	28
ACC_00923	putative RNA-binding protein 15B-like	K13190	KOG0112	544	381	335	547	333	52	78	101	826	842	216	305	470	559	156	55
ACC_00924	septin-4-like		KOG2655	149	99	129	403	225	11	23	18	87	48	65	36	15	60	32	7
ACC_00925	N-alpha-acetyltransferase 35, NatC auxiliary subunit-like		KOG2343	448	331	321	406	285	56	87	102	643	479	124	402	546	475	197	116
ACC_00926	hypothetical protein			5	4	6	4	4	1	2	1	7	1	0	0	1	1	10	0
ACC_00927	conserved hypothetical protein			0	1	0	3	0	0	0	0	86	0	0	0	0	3	0	1
ACC_00928	hypothetical protein			0	0	0	0	1	0	1	0	1	3	1	0	0	1	3	1
ACC_00929	conserved hypothetical protein	K12603	KOG3103	182	107	95	232	175	11	19	18	268	171	171	498	305	318	30	19
ACC_00930	hypothetical protein			3	1	0	5	1	0	0	0	0	2	2	2	1	0	0	0
ACC_00931	hypothetical protein			2	1	0	17	3	0	0	0	0	3	0	0	0	0	2	2
ACC_00932	hypothetical protein			1	0	1	4	1	0	1	3	4	0	2	0	0	0	1	0
ACC_00933	CCR4-NOT transcription complex subunit 6-like		KOG0620	45	26	22	60	36	0	6	8	51	60	24	42	37	35	15	4
ACC_00934	thyrotropin-releasing hormone-degrading ectoenzyme-like		KOG1046	240	118	99	66	75	59	56	89	272	358	545	555	4	0	1	0
ACC_00935	conserved hypothetical protein			7	6	6	5	4	18	12	11	137	10	1562	1285	1	1	5	5
ACC_00936	ubiquitin specific protease-like	K11832	KOG1864	1194	745	780	1766	1139	197	349	411	1120	901	605	1180	750	1098	617	225
ACC_00937	putative ribosomal RNA methyltransferase CG1144	K02427	KOG4589	98	102	107	162	188	11	19	21	140	107	124	219	234	327	32	17
ACC_00938	conserved hypothetical protein			259	83	113	327	261	10	11	14	127	60	71	123	21	16	6	1
ACC_00939	UPF0406 protein C16orf57 homolog		KOG3102	39	29	38	81	107	3	5	8	54	36	40	100	130	146	8	4
ACC_00940	zinc finger CCHC-type and RNA-binding motif-conta	K13154	KOG0117	82	67	72	88	92	19	25	35	92	39	111	109	66	80	33	10
ACC_00941	conserved hypothetical protein			1	0	0	0	0	0	0	0	2	1	0	2	13	12	5	3
ACC_00942	probable nucleolar GTP-binding protein 1-like	K06943	KOG1490	276	184	166	265	138	37	83	80	542	528	162	258	1106	1844	665	381
ACC_00943	GPI transamidase component PIG-T-like isoform 1	K05292	KOG2407	485	310	339	849	544	45	52	77	489	318	209	356	734	1000	134	23
ACC_00944	importin-9		KOG2274	596	369	390	775	486	87	111	127	702	431	366	282	888	993	497	306
ACC_00945	PHD finger protein 2-like	K11445	KOG1633	143	127	114	169	65	11	26	35	219	159	90	114	165	233	95	58
ACC_00946	ATP synthase subunit alpha, mitochondrial isoform	K02132	KOG1353	4958	2025	1725	4970	4361	398	421	551	12128	5665	2220	10072	17709	16524	1492	476
ACC_00947	UBX domain-containing protein 7-like		KOG1364	466	233	182	248	218	15	37	42	384	237	120	351	562	540	84	36
ACC_00948	LOW QUALITY PROTEIN		KOG0032	405	247	66	234	78	311	670	510	16824	10190	212	148	812	785	904	889
ACC_00949	conserved hypothetical protein		KOG0384	1482	916	768	1394	1626	379	862	953	1820	999	1334	1465	1779	1877	1878	1449
ACC_00950	cell division cycle protein 123 homolog		KOG2983	142	88	100	235	289	11	34	34	157	90	151	322	382	591	53	42
ACC_00951	39S ribosomal protein L19, mitochondrial-like	K02884	KOG1698	217	206	221	218	310	24	45	57	187	176	136	295	546	1059	199	141
ACC_00952	hypothetical protein			2	0	0	1	2	0	0	1	0	1	0	0	0	0	2	0
ACC_00953	conserved hypothetical protein		KOG0161	506	328	259	645	691	69	204	223	420	188	346	336	326	348	237	81
ACC_00954	max-like protein X-like	K09113	KOG1319	90	81	48	131	143	17	10	24	242	168	89	283	477	477	79	32
ACC_00955	F-box only protein 28-like		KOG1924	513	467	436	395	210	22	30	39	764	701	164	376	360	353	142	29
ACC_00956	coiled-coil domain-containing protein 12-like isofor	K12871	KOG3407	196	112	99	311	266	37	93	84	330	167	249	221	313	410	150	188
ACC_00957	conserved hypothetical protein		KOG3598	2	5	2	4	0	0	3	1	28	25	0	15	48	610	162	41
ACC_00958	progestin and adipoQ receptor family member 4-like		KOG0748	94	55	69	248	139	22	33	49	116	92	8	7	16	1	2	4
ACC_00959	NADP-dependent malic enzyme isoform 1	K00029	KOG1257	585	505	567	1506	1299	95	139	168	4936	5145	1093	2160	11081	2168	248	152
ACC_00960	conserved hypothetical protein			121	103	120	134	204	7	16	14	115	78	62	163	159	93	10	1
ACC_00961	protein neuralized	K01931	KOG4625	143	110	86	51	8	9	14	22	145	251	21	59	67	62	145	33
ACC_00962	hypothetical protein			8	3	3	3	2	1	0	3	2	0	2	0	2	1	4	0
ACC_00963	suppressor of fused homolog	K06229		96	65	59	161	134	6	5	9	138	76	74	110	98	105	12	3
ACC_00964	translation initiation factor eIF-2B subunit beta-like	K03754	KOG1465	224	185	173	268	218	41	47	74	317	218	146	373	478	488	90	53
ACC_00965	probable ATP-dependent RNA helicase DDX56 isofo	K14810	KOG0346	201	225	185	225	172	23	48	43	311	319	142	284	1039	1531	267	166
ACC_00966	protein unc-80 homolog			548	273	269	397	155	31	38	63	285	165	0	0	1	5	8	2

ACC_00967	LOW QUALITY PROTEIN			30	8	6	19	2	1	3	2	24	10	3	2	0	3	0	5
ACC_00968	cyclin-dependent kinase 2 isoform 2	K02206	KOG0594	222	205	230	273	205	25	38	34	184	83	133	135	377	442	125	36
ACC_00969	urea transporter 2-like			1	0	1	9	3	0	2	0	28	5	1	2	262	114	5	31
ACC_00970	DNA replication licensing factor Mcm6-like	K02542	KOG0480	72	39	46	67	58	5	10	11	81	37	30	39	206	314	96	56
ACC_00971	hypothetical protein			2	0	1	6	5	1	0	0	0	0	1	0	0	0	5	1
ACC_00972	putative peptidyl-tRNA hydrolase PTRHD1-like		KOG3305	508	251	368	412	253	26	32	38	77	55	56	101	80	102	138	51
ACC_00973	WD40 repeat-containing protein SMU1-like isoform	K13111	KOG0275	276	208	186	392	313	42	32	49	457	236	306	515	287	452	29	22
ACC_00974	seryl-tRNA synthetase, mitochondrial-like	K01875	KOG2509	125	81	48	83	117	10	17	19	168	139	54	174	353	355	43	21
ACC_00975	probable G-protein coupled receptor 125-like		KOG4237	444	313	382	346	314	22	37	34	553	372	177	99	370	443	57	16
ACC_00976	odorant receptor Or2-like			1	0	0	1	1	0	0	0	6	4	27	9	0	0	0	1
ACC_00977	DDB1- and CUL4-associated factor 11-like	K11801	KOG0266	147	81	73	124	80	16	24	12	423	277	93	217	279	220	43	15
ACC_00978	UDP-sugar transporter UST74c	K15281	KOG1444	97	68	54	194	148	8	9	18	166	117	63	82	243	253	37	7
ACC_00979	protein halfway-like		KOG0618	141	76	78	114	185	1	4	12	339	203	68	204	412	266	13	4
ACC_00980	twinkle protein, mitochondrial-like		KOG2373	450	302	308	334	367	54	104	101	416	177	278	237	309	354	65	46
ACC_00981	conserved hypothetical protein			16	21	27	29	34	3	0	3	14	21	27	55	9	3	3	2
ACC_00982	carbohydrate kinase domain-containing protein-like isoform		KOG3974	197	185	115	196	143	23	46	34	350	360	260	739	816	771	118	36
ACC_00983	cytochrome P450 6k1-like	K07424	KOG0158	5	3	1	15	5	0	1	0	9	5	7	56	17	67	22	1
ACC_00984	probable cytochrome P450 6a13		KOG0158	60	16	5	38	39	159	248	296	13331	12354	655	1420	780	563	39	23
ACC_00985	microsomal triglyceride transfer protein large subu	K14463	KOG4337	198	98	83	354	127	16	12	32	627	627	117	168	1019	551	171	50
ACC_00986	cysteine-rich PDZ-binding protein		KOG3476	30	22	21	48	54	2	1	1	14	8	22	25	43	37	2	2
ACC_00987	conserved hypothetical protein			378	193	169	312	339	22	56	68	321	268	268	588	343	303	61	26
ACC_00988	protein FAM114A2-like			1225	649	554	733	931	139	374	337	1173	1091	791	1783	2301	1508	516	250
ACC_00989	LOW QUALITY PROTEIN	K00599	KOG2904	246	161	152	360	257	15	34	34	195	152	127	306	561	649	58	31
ACC_00990	gamma-tubulin complex component 3 homolog	K16570	KOG2000	875	641	656	1254	1216	95	156	152	873	617	420	287	454	587	60	15
ACC_00991	beta-1,3-galactosyltransferase 5-like		KOG3356	418	291	315	683	610	37	63	85	429	224	348	841	913	961	119	51
ACC_00992	Oxysterol-binding protein 1		KOG1737	709	429	334	417	305	49	44	57	1771	1159	372	1373	770	734	105	24
ACC_00993	conserved hypothetical protein			240	115	160	288	70	12	9	16	112	64	28	21	16	6	56	29
ACC_00994	probable rRNA-processing protein EBP2 homolog	K14823	KOG3080	378	212	176	228	204	65	145	166	202	149	208	175	915	1930	2006	1682
ACC_00995	conserved hypothetical protein			5	5	1	2	1	0	0	0	8	0	2	54	10	13	30	21
ACC_00996	conserved hypothetical protein		KOG4006	286	1	0	8	0	1	3	4	110	0	1	844	73	21	101	982
ACC_00997	polypyrimidine tract-binding protein 2-like	K14948	KOG1190	696	566	634	1003	278	50	51	97	474	308	123	70	144	269	334	91
ACC_00998	bifunctional 3'-phosphoadenosine 5'-phosphosulfa	K13811	KOG4238	4	1	5	16	7	0	0	2	37	12	1	5	336	699	239	57
ACC_00999	conserved hypothetical protein		KOG0199	210	164	192	142	53	12	16	25	250	240	30	36	66	92	74	29
ACC_01000	muscle-specific protein 20-like		KOG2046	331	224	79	471	153	278	611	583	26846	6924	784	591	3531	1538	1257	1003
ACC_01001	protein phosphatase 1 regulatory subunit 14B-like			121	74	70	132	129	15	16	12	116	78	34	73	175	179	27	15
ACC_01002	60S ribosomal protein L31 isoform 2	K02910	KOG0893	820	638	362	1313	709	154	237	377	904	670	1466	1131	5247	5813	918	602
ACC_01003	conserved hypothetical protein		KOG1029	153	101	72	121	137	22	52	68	52	60	68	117	154	204	168	82
ACC_01004	carbohydrate sulfotransferase 4-like isoform 3			213	104	118	206	172	19	21	9	558	438	96	591	262	189	64	38
ACC_01005	conserved hypothetical protein		KOG0698	154	126	113	243	100	20	39	39	570	666	57	270	482	589	214	99
ACC_01006	facilitated trehalose transporter Tret1-like		KOG0254	74	219	60	147	76	133	110	149	780	517	151	220	330	224	54	56
ACC_01007	sterol regulatory element-binding protein cleavage-activatin		KOG0274	235	149	150	168	122	22	36	38	840	800	101	172	1630	962	119	69
ACC_01008	hypothetical protein			8	4	9	5	7	2	1	0	5	4	1	0	4	0	1	1
ACC_01009	hypothetical protein			0	0	0	0	1	0	0	0	0	0	0	0	0	0	0	0
ACC_01010	conserved hypothetical protein		KOG4272	73	63	65	231	135	13	12	21	154	71	57	195	191	175	94	15
ACC_01011	calmodulin-binding transcription activator 1-like		KOG0520	2215	1085	1222	1976	539	96	160	207	1073	1033	272	130	57	69	171	67
ACC_01012	AP-2 complex subunit mu-1 isoform 1	K11826	KOG0938	1462	570	559	1609	1456	102	120	123	2056	1220	368	2000	1333	1224	177	32
ACC_01013	tropomyosin-1-like	K10374	KOG1003	403	72	41	313	100	58	99	67	5318	2143	208	246	809	834	623	542
ACC_01014	parafibromin	K15175	KOG3786	481	258	270	458	273	42	52	54	610	426	178	555	530	555	129	28
ACC_01015	RNA-binding protein MEX3B-like isoform 2	K15686		75	46	60	142	36	5	8	11	7	23	1	1	1	3	24	31
ACC_01016	methyl-CpG-binding domain protein 3 isoform 3	K11590	KOG4161	125	131	145	336	149	31	39	49	119	46	126	178	104	146	129	93
ACC_01017	conserved hypothetical protein			33	40	36	70	79	1	10	11	11	19	29	50	50	98	101	68
ACC_01018	Ecdysone-induced protein 78C	K08701	KOG4846	38	30	25	15	6	0	2	3	36	25	12	2	0	8	10	5
ACC_01019	probable deoxyhypusine synthase-like	K00809	KOG2924	119	119	123	179	183	27	31	34	112	56	88	111	154	240	28	21
ACC_01020	acid phosphatase-like protein 2-like		KOG3672	474	253	253	523	473	32	58	75	653	283	180	339	60	72	14	6
ACC_01021	paired box protein Pax-6-like	K08031	KOG3862	43	61	46	34	6	1	3	0	20	35	1	0	2	0	3	1
ACC_01022	calcium-binding mitochondrial carrier protein Arala	K15105	KOG0751	352	181	171	165	59	49	42	52	1285	1033	209	221	495	317	104	49
ACC_01023	tetratricopeptide repeat protein 5-like		KOG4626	126	77	72	71	81	13	27	34	323	154	79	104	229	203	18	9

ACC_01024	interferon-related developmental regulator 1-like			267	185	156	310	283	57	75	88	1671	988	205	1097	1367	1279	114	34
ACC_01025	conserved hypothetical protein		KOG0566	378	480	593	583	218	41	43	78	392	220	81	162	272	414	168	51
ACC_01026	DNA-binding protein K10-like isoform 1		KOG1924	181	113	110	352	197	47	71	117	226	269	148	308	293	478	429	360
ACC_01027	glycerol-3-phosphate dehydrogenase, mitochondri	K00111	KOG0042	659	293	267	841	608	59	86	113	3663	2115	449	1447	2183	2230	264	71
ACC_01028	ankyrin repeat and LEM domain-containing protein 2-like			135	106	121	188	182	9	27	24	118	115	71	125	152	166	48	20
ACC_01029	LOW QUALITY PROTEIN	K09527	KOG0550	1859	873	630	1785	1493	470	999	1084	1190	1202	719	866	1135	1123	2973	1659
ACC_01030	putative neutral sphingomyelinase-like	K12351	KOG3873	184	120	142	209	271	67	55	75	508	266	171	432	500	536	15	10
ACC_01031	WD repeat-containing protein 55 homolog		KOG2444	287	179	173	245	292	30	72	78	291	186	300	454	427	429	77	82
ACC_01032	conserved hypothetical protein	K13206	KOG2117	1215	810	518	708	1095	315	616	717	936	773	894	1085	1203	1507	1410	940
ACC_01033	arginine kinase-like	K00933	KOG3581	5515	2933	3800	5976	2805	526	616	1035	6957	4249	3854	15336	7901	7695	1860	687
ACC_01034	syndecan-2-B-like	K16336		263	132	142	131	44	9	14	20	267	301	36	215	97	78	56	38
ACC_01035	conserved oligomeric Golgi complex subunit 1-like		KOG2033	396	295	308	440	399	37	81	69	567	262	234	287	391	381	54	20
ACC_01036	DNA-directed RNA polymerase III subunit RPC3-like	K03023	KOG2587	219	166	169	311	321	18	33	47	269	162	87	257	341	592	73	20
ACC_01037	odorant binding protein 21 precursor			63	21	6	25	21	22	31	20	1065	343	2934	1731	0	0	0	0
ACC_01038	odorant binding protein 21 precursor			81	69	37	329	254	91	146	145	4146	1199	18468	1692	0	0	0	3
ACC_01039	vacuolar protein sorting-associated protein 62-like			64	30	41	88	44	13	14	18	54	30	30	15	88	111	82	67
ACC_01040	conserved hypothetical protein		KOG0828	30	34	14	34	62	4	4	3	84	55	47	154	86	49	6	0
ACC_01041	protein roadkill-like	K10523	KOG4441	545	365	342	308	112	82	105	98	480	486	148	179	190	201	205	40
ACC_01042	protein cereblon-like	K11793	KOG1400	166	113	114	183	211	16	17	11	372	273	83	391	205	285	19	9
ACC_01043	ras-related protein Rab-39A	K07976	KOG0091	114	67	59	66	89	7	4	8	413	226	46	179	120	106	7	5
ACC_01044	galactose-1-phosphate uridylyltransferase-like	K00965	KOG2958	440	331	302	614	582	44	54	68	897	1304	291	1433	1995	1745	325	85
ACC_01045	stAR-related lipid transfer protein 3-like		KOG3845	112	92	72	99	104	14	13	16	152	169	43	130	139	117	12	3
ACC_01046	exportin-1	K14290	KOG2020	362	248	211	247	189	28	21	37	883	1495	109	491	1345	1364	89	29
ACC_01047	hypothetical protein			0	0	0	1	0	0	1	0	0	1	0	0	1	0	0	0
ACC_01048	nesprin-1-like		KOG0307	35	21	20	58	9	2	2	9	42	64	8	6	1	3	14	2
ACC_01049	transmembrane protein 47-like isoform 2		KOG4671	78	28	19	20	17	1	8	2	103	73	15	57	68	55	8	5
ACC_01050	conserved hypothetical protein		KOG0996	114	86	60	56	66	18	58	54	159	105	146	194	343	444	263	225
ACC_01051	NADH dehydrogenase	K11351	KOG4808	222	150	172	300	388	9	16	19	202	113	234	734	265	386	27	18
ACC_01052	conserved hypothetical protein			72	49	49	77	105	0	22	18	43	35	63	82	150	257	52	61
ACC_01053	protein FAM45A-like			80	52	58	97	110	7	8	7	65	74	55	144	255	263	35	22
ACC_01054	tyrocidine synthase 3		KOG1178	2508	889	1378	3333	2685	128	201	260	2876	1389	1639	6280	73	262	65	57
ACC_01055	conserved hypothetical protein		KOG1056	71	419	509	1514	692	2198	2778	3689	16005	7679	2	23	3	2	1	2
ACC_01056	hypothetical protein			5	2	4	9	0	1	0	2	3	2	1	0	0	0	0	0
ACC_01057	hypothetical protein			6	6	1	2	2	3	1	6	3	0	0	0	0	1	1	2
ACC_01058	conserved hypothetical protein			3	1	0	10	0	0	1	1	4	6	4	3	3	12	6	1
ACC_01059	palmitoyltransferase ZDHHC3-like		KOG1311	110	75	59	116	79	12	22	18	154	94	77	141	169	186	18	8
ACC_01060	syntaxin-17	K08491	KOG0811	122	70	50	98	103	28	57	50	277	159	100	183	181	133	60	44
ACC_01061	conserved hypothetical protein			0	1	0	0	0	0	0	0	0	1	0	0	0	0	0	0
ACC_01062	protein G12-like			0	0	0	0	1	0	0	0	44	2	0	1	28444	16074	555	18
ACC_01063	conserved hypothetical protein			0	1	1	5	1	0	0	0	118	0	2	1	61001	5718	68	5
ACC_01064	conserved hypothetical protein			0	0	0	0	0	1	0	0	5	0	0	0	5335	95	2	0
ACC_01065	conserved hypothetical protein			2	0	0	0	0	0	1	0	5	2	0	2	4912	90	0	1
ACC_01066	hypothetical protein			0	0	0	0	0	0	0	0	0	0	0	0	0	0	0	0
ACC_01067	probable G-protein coupled receptor CG31760-like		KOG4418	598	394	377	723	286	72	111	114	300	185	29	16	10	4	26	12
ACC_01068	dual 3',5'-cyclic-AMP and -GMP phosphodiesterase 11-like		KOG3689	144	124	160	187	116	27	27	43	498	508	115	232	156	176	37	9
ACC_01069	conserved hypothetical protein			606	371	355	1142	529	38	19	42	2646	1624	6170	15628	68	43	13	7
ACC_01070	glyceraldehyde-3-phosphate dehydrogenase 1-like	K00134	KOG0657	9	0	1	4	3	0	0	0	3	2	1	0	0	0	0	4
ACC_01071	conserved hypothetical protein		KOG4788	227	50	29	91	36	10	7	5	698	343	141	834	166	274	65	7
ACC_01072	conserved hypothetical protein			166	74	93	272	112	30	47	53	159	84	141	76	44	50	80	41
ACC_01073	cell division cycle protein 23 homolog	K03355	KOG1155	206	160	151	302	296	40	62	72	403	328	244	334	482	515	144	34
ACC_01074	endoplasmic reticulum-Golgi intermediate compartment prc		KOG2667	202	185	169	285	350	37	62	67	202	216	197	320	501	392	37	12
ACC_01075	conserved hypothetical protein	K09142	KOG3925	155	102	83	182	205	32	112	110	221	125	116	137	266	335	278	259
ACC_01076	trafficking protein particle complex subunit 10-like		KOG1931	203	116	78	78	96	11	13	15	368	345	46	142	365	228	23	1
ACC_01077	PH-interacting protein	K11798	KOG0644	976	858	650	551	433	171	281	324	1106	1046	378	244	584	702	879	484
ACC_01078	centromere protein L-like			62	74	64	59	75	3	9	6	60	49	50	91	199	259	14	11
ACC_01079	conserved hypothetical protein			2	0	2	1	3	0	1	0	2	1	0	0	1	0	0	0
ACC_01080	conserved hypothetical protein			6	12	10	20	16	2	4	1	5	2	5	3	9	7	5	2

ACC_01081	cysteine and histidine-rich protein 1 homolog		KOG3002	254	189	163	775	395	34	35	38	173	161	123	272	161	80	21	2
ACC_01082	EF-hand domain-containing protein KIAA0494-like		KOG0161	339	259	276	507	267	62	75	93	307	247	440	598	248	230	114	26
ACC_01083	LOW QUALITY PROTEIN		KOG2194	326	152	134	204	243	9	13	14	351	345	64	124	643	521	34	9
ACC_01084	conserved hypothetical protein			39	29	24	17	40	1	2	4	37	21	20	8	16	11	1	1
ACC_01085	nicalin-1-like		KOG1359	276	205	189	310	349	13	18	28	479	341	103	442	1063	854	36	9
ACC_01086	plasma membrane calcium-transporting ATPase 3		KOG0204	2470	734	637	940	391	146	233	254	1661	1235	253	307	371	299	363	169
ACC_01087	stomatin-like protein 2-like		KOG2620	289	237	211	529	480	35	46	64	359	240	243	536	629	1113	226	93
ACC_01088	intraflagellar transport protein 81 homolog		KOG0161	1625	1102	1197	739	907	268	680	523	1375	548	1135	519	500	809	1106	607
ACC_01089	GK25335	K10418	KOG3430	741	405	325	926	730	36	46	65	657	480	195	1368	909	757	175	91
ACC_01090	DNA2-like helicase-like	K10742	KOG1805	510	405	332	461	601	35	104	78	774	566	475	630	775	894	157	78
ACC_01091	probable alpha-ketoglutarate-dependent dioxygen	K10769	KOG4176	55	44	38	78	84	3	11	15	101	57	79	150	107	102	19	8
ACC_01092	e3 ubiquitin-protein ligase MIB2-like	K10645	KOG4582	521	282	254	483	363	45	100	114	596	368	205	284	624	735	432	189
ACC_01093	TPPP family protein CG4893		KOG4070	24	7	9	22	34	0	0	1	26	10	25	12	14	10	0	0
ACC_01094	conserved hypothetical protein			50	38	34	59	24	8	7	15	42	47	18	16	12	10	22	5
ACC_01095	hypothetical protein			4	3	1	2	8	0	2	0	3	7	14	15	7	4	5	1
ACC_01096	serine proteinase stubble isoform 1		KOG3627	203	119	154	922	619	144	144	144	12955	6629	1534	2158	1861	679	134	71
ACC_01097	conserved hypothetical protein		KOG3627	54	56	37	84	40	11	17	14	81	49	38	25	29	8	12	5
ACC_01098	conserved hypothetical protein		KOG4441	100	81	84	129	25	14	22	40	111	174	66	29	14	22	158	44
ACC_01099	conserved hypothetical protein		KOG4010	266	145	102	368	229	88	168	211	319	119	230	382	337	221	309	186
ACC_01100	conserved hypothetical protein		KOG1949	311	202	200	248	324	13	11	34	222	419	94	433	509	295	37	20
ACC_01101	conserved hypothetical protein			796	533	630	465	403	78	150	121	505	441	127	199	305	401	68	19
ACC_01102	protein MON2 homolog		KOG1848	456	332	257	363	278	48	65	74	1266	1373	229	736	1006	893	103	26
ACC_01103	e3 ubiquitin-protein ligase parkin-like isoform 1	K04556	KOG0006	134	59	65	118	135	29	36	25	265	163	84	196	444	243	43	37
ACC_01104	conserved hypothetical protein			78	56	64	132	108	6	8	10	166	87	57	187	122	132	22	7
ACC_01105	LOW QUALITY PROTEIN	K03439	KOG3115	63	54	58	107	97	12	29	24	98	67	66	45	276	402	82	35
ACC_01106	conserved hypothetical protein		KOG3139	188	92	136	90	53	4	12	19	237	124	68	83	30	10	0	0
ACC_01107	band 4.1-like protein 5-like		KOG3530	439	329	292	365	235	84	161	234	376	251	205	244	327	340	466	215
ACC_01108	glycerophosphodiester phosphodiesterase domain-containir		KOG2258	144	94	95	102	104	3	9	8	173	172	70	358	139	101	7	3
ACC_01109	UDP-GlcNAc		KOG2287	308	181	168	316	174	14	11	17	330	293	48	197	223	202	77	18
ACC_01110	hypothetical protein			1	0	1	1	0	0	0	0	0	2	2	0	0	0	6	0
ACC_01111	calpain-A-like isoform 4	K08585	KOG0045	872	699	721	2852	1285	191	228	308	2418	1174	724	1284	810	918	105	67
ACC_01112	ALK tyrosine kinase receptor-like	K05119	KOG1095	2065	1312	1712	2175	913	113	145	175	2049	1096	265	621	44	61	51	21
ACC_01113	conserved hypothetical protein			31	11	14	26	5	11	14	20	57	77	46	43	0	4	6	4
ACC_01114	G-protein coupled receptor Mth2-like		KOG4193	225	159	180	385	356	21	37	28	2674	2850	150	251	2005	863	45	4
ACC_01115	GM13182	K11251	KOG1756	20	7	3	15	4	5	6	3	11	14	5	8	8	11	37	35
ACC_01116	predicted protein	K11253	KOG1745	19	20	19	156	50	8	6	7	37	16	19	20	27	21	32	35
ACC_01117	mitochondrial-processing peptidase subunit beta-li	K01412	KOG0960	1818	778	797	2452	1930	116	108	161	5918	1866	1396	4720	4929	5391	418	145
ACC_01118	acyl-CoA Delta(11) desaturase-like	K00507	KOG1600	932	609	551	1313	1033	288	475	450	9572	14192	2469	6217	9776	3796	1180	1295
ACC_01119	shootin-1-like		KOG0161	215	269	414	846	408	56	63	74	723	291	83	198	409	370	149	86
ACC_01120	nucleotide exchange factor SIL1-like		KOG2160	312	234	252	555	563	48	94	178	224	166	200	348	192	203	54	37
ACC_01121	predicted protein	K11253	KOG1745	23	26	14	53	32	11	9	12	33	31	50	44	49	37	87	71
ACC_01122	histone H2B.1/H2B.2-like	K11252	KOG1744	13	10	3	10	11	2	3	2	5	4	4	20	17	17	18	16
ACC_01123	GM13182	K11251	KOG1756	10	2	6	13	6	2	3	2	2	7	3	5	4	6	24	29
ACC_01124	conserved hypothetical protein			1370	1706	1079	684	712	463	635	652	976	1036	1265	1508	2680	4067	2891	1117
ACC_01125	phospholipid hydroperoxide glutathione peroxidas	K00432	KOG1651	446	542	517	1005	937	114	145	177	6011	1526	4364	9625	2817	2179	151	51
ACC_01126	LOW QUALITY PROTEIN	K05767	KOG2128	191	153	126	179	237	17	32	29	224	82	59	30	230	376	118	49
ACC_01127	dysbindin-A-like		KOG0994	370	269	300	372	406	46	139	145	294	119	261	274	269	349	86	34
ACC_01128	integrin alpha-8-like		KOG3637	511	355	240	449	485	79	152	170	470	685	249	151	89	67	39	16
ACC_01129	LOW QUALITY PROTEIN			29	30	40	29	13	1	1	3	20	16	2	0	3	5	13	6
ACC_01130	PDZ domain-containing protein 6-like			335	218	266	398	388	22	40	58	248	225	170	242	297	387	89	36
ACC_01131	cell division cycle protein 20 homolog	K03363	KOG0305	49	39	46	85	74	4	7	2	55	35	46	67	295	633	106	54
ACC_01132	conserved hypothetical protein		KOG1860	1029	655	625	988	1142	66	230	187	1363	1305	945	1068	730	639	242	65
ACC_01133	SON protein		KOG0151	3676	2246	2144	2621	2903	626	1572	1793	1633	1120	1371	602	1001	1444	2472	1653
ACC_01134	e3 ubiquitin-protein ligase IAP-3-like	K04725	KOG1101	660	384	343	575	510	59	86	101	1219	1043	219	1638	1425	1619	184	71
ACC_01135	baculoviral IAP repeat-containing protein 4	K16060	KOG1101	544	355	402	698	682	100	179	201	492	309	382	615	476	556	124	65
ACC_01136	carnitine O-palmitoyltransferase 2, mitochondrial-l	K08766	KOG3719	328	174	175	306	362	29	42	41	503	367	300	732	875	902	80	25
ACC_01137	nucleoporin NUP188 homolog	K14311	KOG4833	388	237	209	436	304	30	46	71	453	442	172	302	568	730	151	40

ACC_01138	UPF0464 protein C15orf44 homolog			240	190	212	334	271	37	74	64	390	258	281	595	473	592	109	35
ACC_01139	clavesin-2-like isoform 1		KOG1471	166	77	74	183	182	36	63	77	354	226	112	182	742	1148	426	353
ACC_01140	trifunctional enzyme subunit beta, mitochondrial-li	K07509	KOG1392	647	409	295	677	576	115	136	201	1807	1183	518	1650	4126	4090	686	439
ACC_01141	KRR1 small subunit processome component homol	K06961	KOG2874	148	122	95	166	117	25	59	96	118	74	69	103	443	664	322	299
ACC_01142	TBC1 domain family member 14-like		KOG2223	71	32	49	81	60	5	7	12	130	137	20	41	36	26	7	2
ACC_01143	succinate-semialdehyde dehydrogenase, mitochondrial-like		KOG2451	277	160	192	430	569	11	14	14	313	346	63	391	1655	850	33	10
ACC_01144	dehydrogenase/reductase SDR family member 4-lik	K11147	KOG0725	281	250	279	295	381	42	51	57	860	688	313	823	2675	2209	165	50
ACC_01145	conserved hypothetical protein	K13092		727	557	517	680	393	173	272	286	699	513	303	228	465	637	1550	1034
ACC_01146	WASH complex subunit strumpellin-like		KOG3666	319	200	172	196	217	23	32	33	426	350	116	236	415	375	30	6
ACC_01147	conserved hypothetical protein			66	68	72	68	64	20	35	28	237	105	60	194	102	127	39	16
ACC_01148	beta-1,3-galactosyltransferase brn-like		KOG2287	123	132	109	181	231	8	20	25	66	85	144	270	95	36	13	7
ACC_01149	conserved hypothetical protein			6	10	6	25	15	0	0	1	20	30	71	178	0	0	0	0
ACC_01150	protein germ cell-less	K10485	KOG4682	201	147	136	188	205	13	27	38	285	297	94	254	596	722	68	25
ACC_01151	caspase-like		KOG3573	0	0	1	1	0	0	0	0	0	0	0	8	222	131	20	3
ACC_01152	LOW QUALITY PROTEIN	K02330	KOG2534	431	288	313	536	691	54	168	187	355	176	233	259	249	221	102	57
ACC_01153	d-aspartate oxidase-like	K00272	KOG3923	108	110	67	116	104	51	50	60	388	190	272	822	511	312	48	14
ACC_01154	conserved hypothetical protein		KOG1015	6357	4224	3923	4165	4500	1267	2783	2966	4724	2820	2283	761	2265	3434	7757	4698
ACC_01155	alpha- and gamma-adaptin-binding protein p34-like isoform		KOG4273	204	122	106	212	212	11	25	43	239	163	129	448	416	445	40	18
ACC_01156	26S protease regulatory subunit 6B isoform 1	K03063	KOG0727	309	182	175	449	373	38	26	45	745	635	460	1574	1349	1965	135	49
ACC_01157	conserved hypothetical protein	K11128	KOG3262	528	207	163	401	328	55	100	96	397	291	175	283	1559	2843	1067	1006
ACC_01158	LOW QUALITY PROTEIN			13	16	9	20	18	1	3	7	9	12	13	6	1	2	0	1
ACC_01159	elongation factor 1-alpha-like	K03231	KOG0052	3285	3229	1250	4882	2781	1120	1055	989	7429	10233	2472	13334	35647	45112	10414	1644
ACC_01160	conserved hypothetical protein			147	143	123	92	29	3	1	6	105	74	1	7	5	5	4	1
ACC_01161	monocarboxylate transporter 10-like	K08187	KOG2504	213	90	55	106	45	16	8	20	148	325	7	35	60	63	21	5
ACC_01162	conserved hypothetical protein			0	3	0	2	3	0	1	0	21	2	0	0	5218	50	0	0
ACC_01163	interferon regulatory factor 2-binding protein 2-like		KOG3579	229	195	218	326	90	25	29	35	705	659	114	274	153	113	181	41
ACC_01164	G patch domain-containing protein 2-like			217	132	140	278	200	17	20	26	172	119	80	156	140	144	13	12
ACC_01165	conserved hypothetical protein			152	86	74	210	172	20	30	41	124	113	172	33	77	252	75	28
ACC_01166	G-protein coupled receptor moody-like		KOG4220	145	2	1	2	5	2	5	2	12	13	3	9	21	17	6	4
ACC_01167	LOW QUALITY PROTEIN	K03350	KOG1126	348	231	202	307	202	53	63	65	1162	673	207	346	654	621	234	121
ACC_01168	LOW QUALITY PROTEIN		KOG4229	267	171	148	238	71	34	67	87	368	568	39	58	81	71	86	35
ACC_01169	pyrroline-5-carboxylate reductase 2-like	K00286	KOG3124	289	229	308	705	539	26	59	63	962	810	328	826	789	416	60	16
ACC_01170	conserved hypothetical protein		KOG2425	290	142	141	207	241	18	40	48	194	218	110	208	785	916	207	127
ACC_01171	WD repeat-containing protein 48-like isoform 1	K15361	KOG0308	349	242	233	296	228	81	146	196	616	499	146	278	396	426	291	120
ACC_01172	acetylcholine receptor protein alpha 1, 2, 3, 4 invertebrate, p		KOG3646	170	95	91	91	29	8	13	11	144	113	26	71	41	23	62	8
ACC_01173	conserved hypothetical protein			109	92	96	142	170	8	21	32	101	55	108	183	98	109	24	20
ACC_01174	ubiquitin thioesterase otubain-like	K09602	KOG3991	164	143	128	372	396	16	25	43	224	112	199	300	397	599	81	38
ACC_01175	putative high mobility group protein B1-like 1-like		KOG0381	1033	615	555	1099	904	85	97	127	1332	671	371	1624	1375	1940	249	72
ACC_01176	histone deacetylase complex subunit SAP30 homolog			169	119	150	413	254	24	51	53	146	75	118	183	128	179	95	30
ACC_01177	fatty-acid amide hydrolase 2-like		KOG1212	250	184	172	349	399	23	23	46	440	476	247	963	378	290	11	17
ACC_01178	dipeptidyl peptidase 3-like isoform 1	K01277	KOG3675	696	619	462	599	449	99	135	222	895	895	288	731	2004	2428	263	87
ACC_01179	DNA repair protein RAD51 homolog 1	K04482	KOG1433	61	51	42	131	103	5	7	10	91	64	42	102	133	192	29	9
ACC_01180	conserved hypothetical protein			441	291	290	336	449	53	172	135	458	289	714	691	453	479	262	112
ACC_01181	protein FAN		KOG1787	322	228	207	243	277	21	61	67	260	295	145	393	244	216	43	13
ACC_01182	zinc finger protein 16-like		KOG2462	314	209	172	483	293	119	259	302	422	465	290	167	268	326	774	385
ACC_01183	spastin-like	K13254	KOG0740	138	81	74	116	81	14	24	36	146	127	36	53	132	90	50	27
ACC_01184	putative cysteine proteinase CG12163-like	K01373	KOG1542	2012	1467	1096	1889	1760	308	473	600	7887	6104	1467	2795	2926	1834	141	49
ACC_01185	hypothetical protein			456	380	570	811	522	77	181	142	378	197	109	12	15	25	49	39
ACC_01186	mitochondrial intermembrane space import and assembly p		KOG4149	119	49	46	173	129	16	17	34	98	55	82	125	304	575	168	118
ACC_01187	conserved hypothetical protein	K14529		83	45	52	110	115	2	8	13	112	91	49	147	313	445	46	11
ACC_01188	paxillin-like isoform 2	K05760	KOG1703	360	237	202	448	169	61	88	82	3438	1671	331	785	1080	622	247	90
ACC_01189	peptidyl-prolyl cis-trans isomerase-like 2-like	K10598	KOG0883	305	249	237	342	309	49	83	82	544	242	312	480	333	320	103	45
ACC_01190	NACHT and WD repeat domain-containing protein	K11887	KOG0319	1012	510	554	1263	613	78	62	85	721	459	407	100	169	247	104	22
ACC_01191	probable tyrosine--tRNA ligase, mitochondrial-like	K01866	KOG2623	365	259	309	530	656	41	59	57	400	208	257	250	438	686	57	30
ACC_01192	conserved hypothetical protein	K02983	KOG0009	934	962	507	1621	1214	455	936	1088	581	624	796	1599	4519	6487	4994	7333
ACC_01193	nitrogen permease regulator 3-like protein-like		KOG3830	107	80	76	171	173	2	2	6	122	130	56	116	128	179	4	2
ACC_01194	tubulin-folding cofactor B-like		KOG3206	318	239	221	421	469	76	142	170	448	227	368	423	362	403	207	158

ACC_01195	exostosin-2	K02367	KOG1022	512	317	338	285	415	42	57	72	574	284	340	450	445	394	25	5
ACC_01196	conserved hypothetical protein	K16479	KOG0161	344	259	268	567	326	99	251	269	305	455	169	56	84	109	434	290
ACC_01197	ubiquitin-conjugating enzyme E2-22 kDa-like isofor	K04649	KOG0418	166	105	94	250	163	18	14	23	277	225	123	341	701	726	197	90
ACC_01198	protein arginine N-methyltransferase 5 isoform 1	K02516	KOG0822	284	219	196	359	324	18	36	41	538	376	165	265	1335	1550	141	53
ACC_01199	15 kDa selenoprotein-like		KOG3384	74	51	68	81	128	9	14	15	79	42	91	253	217	240	12	8
ACC_01200	serine/threonine-protein phosphatase 4 regulatory	K15424	KOG0211	233	120	106	239	145	41	55	66	375	202	136	134	253	481	602	225
ACC_01201	LOW QUALITY PROTEIN		KOG3626	311	243	231	413	187	47	43	40	976	675	355	324	54	57	14	7
ACC_01202	acidic mammalian chitinase		KOG2806	774	413	391	504	424	18	9	38	831	852	125	541	318	213	53	12
ACC_01203	acidic mammalian chitinase-like	K08360	KOG1619	480	381	290	364	475	31	35	39	1031	594	249	1239	923	464	19	2
ACC_01204	protein Mpv17-like	K13348	KOG1944	13	2	6	7	7	0	1	0	6	5	1	4	8	2	0	0
ACC_01205	metallo-beta-lactamase domain-containing protein 1-like		KOG4736	43	38	39	47	60	2	4	3	30	43	29	121	166	168	14	3
ACC_01206	conserved hypothetical protein		KOG3701	1089	931	778	1068	821	315	426	606	1962	1171	731	1362	2748	2401	575	275
ACC_01207	transcription initiation factor IIA subunit 1 isoform	K03122	KOG2652	472	354	276	312	226	39	65	72	666	445	210	469	847	791	199	127
ACC_01208	peroxisomal membrane protein PEX13-like	K13344	KOG3875	237	153	161	287	351	11	28	41	350	388	184	805	1268	811	59	15
ACC_01209	FAM203 family protein GA19338-like		KOG2973	106	93	116	258	182	24	37	41	107	85	74	173	161	268	48	39
ACC_01210	calcium-independent phospholipase A2-gamma-like		KOG4231	366	214	184	386	325	20	68	65	650	517	226	383	138	131	64	19
ACC_01211	FABP-like protein		KOG4015	1046	1727	3219	10936	4986	136	271	507	768	322	2857	7044	25120	5876	1119	622
ACC_01212	ornithine decarboxylase-like	K01581	KOG0622	573	302	275	665	627	49	47	38	1677	964	271	1551	1926	2076	193	45
ACC_01213	UPF0136 membrane protein CG5532-like		KOG4267	291	161	209	521	389	32	36	49	413	193	416	874	517	635	104	60
ACC_01214	LOW QUALITY PROTEIN	K14805	KOG0347	412	262	239	343	398	68	180	169	287	273	217	180	927	1530	913	706
ACC_01215	LOW QUALITY PROTEIN		KOG2069	130	140	107	262	311	12	32	24	212	162	165	344	348	416	47	8
ACC_01216	conserved hypothetical protein		KOG0268	546	393	417	549	512	21	53	34	481	342	179	197	421	290	41	20
ACC_01217	flap endonuclease 1	K04799	KOG2519	159	153	146	166	120	16	17	28	190	87	92	157	429	855	431	143
ACC_01218	LOW QUALITY PROTEIN	K11267	KOG1525	250	250	179	224	122	22	51	60	436	501	110	223	541	672	169	79
ACC_01219	eukaryotic translation initiation factor 2 subunit 1-l	K03237	KOG2916	265	145	107	207	217	29	32	46	328	188	122	445	1790	2303	273	149
ACC_01220	ER degradation-enhancing alpha-mannosidase-like	K10085	KOG2429	440	283	262	259	222	52	109	166	467	552	108	532	681	473	100	34
ACC_01221	RILP-like protein homolog		KOG0161	210	108	116	135	160	12	29	39	348	261	70	215	205	177	41	42
ACC_01222	UPF0518 protein AGAP011705-like		KOG3695	437	297	254	408	372	17	30	58	648	620	196	445	376	263	29	14
ACC_01223	LOW QUALITY PROTEIN		KOG3539	162	118	155	254	162	11	17	20	332	278	19	24	122	115	36	8
ACC_01224	coiled-coil domain-containing protein 94-like		KOG2989	273	191	165	279	277	51	149	147	161	67	128	160	197	292	314	323
ACC_01225	conserved hypothetical protein		KOG4570	998	542	494	459	643	115	290	291	563	455	417	569	2218	3101	1619	923
ACC_01226	conserved hypothetical protein			165	123	97	557	215	34	69	100	106	102	219	50	8	8	100	52
ACC_01227	magnesium transporter protein 1-like	K12669	KOG2603	591	560	361	879	643	131	110	161	1005	739	457	1298	1573	1486	104	36
ACC_01228	phospholipase A2-like	K01047	KOG4087	26	22	33	68	46	8	4	8	16	23	14	6	5	17	5	0
ACC_01229	large subunit GTPase 1 homolog	K14539	KOG1424	693	378	329	829	724	87	175	194	953	589	503	693	1216	1328	529	328
ACC_01230	conserved hypothetical protein	K15542	KOG0284	273	166	151	330	210	39	43	54	391	286	140	254	356	526	140	35
ACC_01231	iodotyrosine dehalogenase 1-like isoform 2		KOG3936	85	47	71	121	109	7	9	15	116	79	49	53	71	82	17	3
ACC_01232	neutral ceramidase-like		KOG2232	43	32	50	145	33	5	5	5	92	108	12	29	243	124	23	11
ACC_01233	probable phosphorylase b kinase regulatory subuni	K07190	KOG3635	696	410	391	547	713	49	55	78	1545	881	461	486	521	417	41	15
ACC_01234	HEAT repeat-containing protein 6-like		KOG4535	377	267	244	349	477	29	44	31	780	727	337	1062	564	528	31	10
ACC_01235	NGFI-A-binding protein homolog		KOG3835	134	191	207	378	183	31	42	38	160	80	97	38	11	26	30	15
ACC_01236	conserved hypothetical protein			0	0	0	1	0	1	0	1	0	3	0	0	2	12	1	5
ACC_01237	conserved hypothetical protein		KOG1847	7	7	15	16	5	2	0	1	1	2	1	0	2	1	5	0
ACC_01238	coatomer subunit beta		KOG1058	1009	463	395	1006	681	173	172	212	1770	989	944	1308	1898	2667	577	224
ACC_01239	Bardet-Biedl syndrome 2 protein homolog	K16747		85	56	49	106	75	7	8	8	59	71	55	128	2	7	2	3
ACC_01240	ubiquitin carboxyl-terminal hydrolase 3-like	K11986	KOG1867	277	195	202	446	135	27	18	31	359	201	21	119	172	122	79	27
ACC_01241	40S ribosomal protein S17	K02962	KOG0187	2071	3276	1650	1617	924	623	1141	1492	2028	1503	1124	3181	12215	12264	3457	1841
ACC_01242	conserved hypothetical protein			8	6	4	17	13	0	3	1	9	5	3	3	12	20	13	10
ACC_01243	cap-specific mRNA (nucleoside-2'-O-)-methyltransf	K14589	KOG3673	266	164	123	210	283	24	56	81	413	254	141	193	292	230	101	69
ACC_01244	protein disulfide-isomerase A4-like		KOG0191	447	250	109	180	203	53	41	45	375	273	232	622	607	417	23	11
ACC_01245	neuronal PAS domain-containing protein 2	K02223	KOG3561	162	187	168	83	52	20	16	32	500	543	46	387	30	10	5	2
ACC_01246	RRP15-like protein-like		KOG2974	395	264	224	282	385	81	219	166	247	93	254	193	340	695	922	964
ACC_01247	hypothetical protein			2	0	0	0	0	0	0	0	1	4	0	0	0	0	0	1
ACC_01248	phosphatidylinositol 4-kinase beta-like	K00888	KOG0903	544	396	290	404	244	64	83	81	896	713	137	210	449	436	151	47
ACC_01249	ATP-dependent RNA helicase DDX54-like	K14808	KOG0337	778	555	495	1013	945	181	454	446	530	494	420	448	1105	1379	1459	899
ACC_01250	lambda crystallin-like protein	K13247	KOG2305	618	584	377	1152	1078	491	655	681	5918	4783	1267	2089	18019	15414	2403	1509
ACC_01251	mediator of RNA polymerase II transcription subun	K15152	KOG1510	112	101	135	216	160	14	20	20	95	57	66	173	111	209	45	27

ACC_01252	conserved hypothetical protein			133	66	85	127	35	13	22	14	418	196	86	82	46	122	109	40
ACC_01253	conserved hypothetical protein			0	0	0	0	0	2	0	0	2	2	58	15	0	0	0	0
ACC_01254	60S acidic ribosomal protein P2-like	K02943	KOG3449	2560	2210	1272	3832	2631	787	1351	1661	4727	2241	3226	4455	19348	20832	12054	6827
ACC_01255	glutathione synthetase-like		KOG2861	162	110	96	195	202	16	34	29	159	115	95	275	609	780	117	37
ACC_01256	mitochondrial inner membrane organizing system protein 1-		KOG4604	1256	553	462	1184	707	148	333	415	984	542	387	1192	1501	1885	579	454
ACC_01257	conserved hypothetical protein			5	4	3	6	1	1	0	2	18	18	1	0	0	5	1	1
ACC_01258	conserved hypothetical protein	K10318	KOG0274	30	20	18	23	13	2	2	4	25	42	2	9	24	23	21	18
ACC_01259	conserved hypothetical protein		KOG3598	328	344	531	514	138	28	59	48	503	494	33	30	20	34	79	67
ACC_01260	conserved hypothetical protein			3	1	0	12	0	7	4	4	24	15	55	46	118	404	269	23
ACC_01261	UPF0415 protein C7orf25 homolog isoform 1		KOG4529	375	261	275	625	562	47	55	75	510	229	152	358	271	294	54	18
ACC_01262	protein APCDD1-like			28	19	30	51	44	2	7	3	31	31	19	7	12	26	5	8
ACC_01263	cytochrome b-c1 complex subunit 2, mitochondrial	K00415	KOG2583	2090	788	767	2608	2581	131	131	224	4154	1777	1057	4122	3356	2754	165	60
ACC_01264	conserved hypothetical protein		KOG3608	877	583	614	1288	922	72	144	189	577	515	224	82	149	188	195	58
ACC_01265	conserved hypothetical protein		KOG4673	844	534	585	606	696	162	378	372	1357	896	461	418	412	393	283	125
ACC_01266	conserved hypothetical protein			0	0	0	0	1	0	0	0	1	1	0	0	1	4	5	2
ACC_01267	cytochrome c oxidase subunit 6A1, mitochondrial	K02266	KOG3469	3860	2105	2184	4810	2148	412	635	760	1663	568	4280	4249	2282	1733	837	499
ACC_01268	conserved hypothetical protein			152	107	90	306	239	9	11	23	224	143	130	345	591	809	57	22
ACC_01269	conserved hypothetical protein		KOG0694	604	397	453	597	173	73	77	105	690	422	150	153	88	105	63	58
ACC_01270	conserved hypothetical protein		KOG4590	288	241	241	142	61	23	32	34	603	721	65	174	277	221	160	85
ACC_01271	conserved hypothetical protein			471	170	185	608	88	17	44	75	257	156	77	62	32	24	124	88
ACC_01272	hypothetical protein			17	5	11	8	10	2	2	2	1	3	2	0	0	0	3	1
ACC_01273	FAM18-like protein CG5021-like		KOG3195	330	371	236	522	415	94	129	201	387	233	332	609	557	409	86	32
ACC_01274	85 kDa calcium-independent phospholipase A2-like	K16343	KOG0513	693	288	298	661	345	47	69	84	588	348	292	386	201	201	103	96
ACC_01275	DNA topoisomerase 3-alpha-like	K03165	KOG1956	510	307	337	519	431	63	96	91	604	346	272	255	507	840	408	109
ACC_01276	DIS3-like exonuclease 2-like		KOG2102	457	285	266	627	533	57	95	86	685	422	301	405	552	614	185	89
ACC_01277	conserved hypothetical protein			266	152	131	240	193	35	34	47	342	416	67	146	9	7	4	4
ACC_01278	cyclin-dependent kinase-like 2-like	K08824	KOG0593	27	18	34	41	26	3	5	7	68	58	45	3	28	25	21	9
ACC_01279	conserved hypothetical protein	K11306	KOG2747	7740	7142	6540	7304	4561	2088	3994	3976	10202	4990	4465	961	1395	2629	11078	7107
ACC_01280	Ras-related protein Rab-26		KOG0083	710	371	399	561	199	184	236	289	366	182	89	184	21	16	26	5
ACC_01281	LOW QUALITY PROTEIN	K11978	KOG1139	104	52	88	94	76	5	12	9	259	450	41	94	92	76	15	5
ACC_01282	conserved hypothetical protein	K15438		421	360	306	529	374	34	66	92	549	365	228	562	912	1183	192	98
ACC_01283	GDP-fucose protein O-fucosyltransferase 1-like	K03691	KOG3849	86	40	51	118	93	1	10	9	73	27	73	140	91	129	17	4
ACC_01284	vigilin-like		KOG2208	3342	2354	1220	1390	1233	1055	2292	2182	5582	7707	992	2000	12079	8375	4663	2578
ACC_01285	hypothetical protein			0	0	2	0	0	0	3	0	1	2	1	0	0	0	0	0
ACC_01286	putative phospholipase B-like lamina ancestor-like		KOG3774	521	529	228	561	500	279	222	265	2327	1169	702	2935	1494	1174	176	19
ACC_01287	guanine nucleotide-releasing factor 2-like	K06277	KOG3417	193	108	140	141	58	17	34	36	423	322	43	94	72	64	28	14
ACC_01288	survival of motor neuron-related-splicing factor 30-	K12839	KOG3026	425	307	315	546	456	106	212	284	597	227	417	420	352	436	497	333
ACC_01289	small nuclear ribonucleoprotein Sm D3	K11088	KOG3172	238	208	201	479	295	59	98	97	297	128	216	333	527	951	503	418
ACC_01290	beta-ureidopropionase-like isoform 1	K01431	KOG0808	455	179	131	218	148	38	73	69	247	332	181	345	15273	692	103	64
ACC_01291	ras-related protein Rab-11A	K07904	KOG0087	527	320	320	793	465	109	96	154	1199	770	404	1348	1014	1035	158	82
ACC_01292	transcriptional adapter 3-B-like	K11315	KOG4191	211	174	208	240	194	29	35	39	290	138	125	175	192	230	76	35
ACC_01293	conserved hypothetical protein			173	148	191	447	317	20	29	46	228	181	83	328	74	62	41	40
ACC_01294	e3 ubiquitin-protein ligase TRIM23-like	K07963	KOG0070	733	500	535	1150	918	72	121	166	547	283	564	646	285	305	158	105
ACC_01295	coiled-coil-helix-coiled-coil-helix domain-containing protein		KOG4618	78	80	74	115	131	0	5	1	5	44	65	293	27	10	9	10
ACC_01296	transmembrane protein 98			43	22	16	61	48	3	1	4	49	21	31	84	48	39	6	1
ACC_01297	conserved hypothetical protein		KOG0161	82	63	57	70	65	11	25	21	532	423	152	291	202	190	23	20
ACC_01298	protein SMG8-like		KOG3692	174	131	110	246	178	32	30	40	323	250	135	176	200	276	55	18
ACC_01299	45 kDa calcium-binding protein-like		KOG4251	309	261	294	279	173	17	26	39	161	72	61	65	13	28	10	3
ACC_01300	conserved hypothetical protein	K14388	KOG2349	677	384	427	667	652	74	177	187	176	179	99	367	12	25	54	9
ACC_01301	conserved hypothetical protein		KOG2349	348	232	223	210	179	13	29	20	442	262	159	503	933	751	63	6
ACC_01302	conserved hypothetical protein			80	67	52	59	26	7	14	8	61	27	23	5	26	48	14	1
ACC_01303	conserved hypothetical protein			3	1	5	4	5	0	0	0	2	11	0	0	13	3	3	3
ACC_01304	conserved hypothetical protein		KOG4029	33	17	33	115	43	0	2	4	14	7	3	0	1	5	5	7
ACC_01305	beta-1,3-galactosyltransferase 6-like		KOG2288	160	118	98	206	245	21	27	47	178	93	65	156	80	129	16	8
ACC_01306	sodium-independent sulfate anion transporter-like	K14708	KOG0236	50	47	50	130	90	22	34	32	100	564	19	3	947	208	25	15
ACC_01307	conserved hypothetical protein			283	254	267	379	387	19	35	35	544	306	241	608	546	374	36	9
ACC_01308	profilin	K05759	KOG1755	947	858	640	1543	420	293	474	567	859	1565	481	1103	1639	1813	2783	1465

ACC_01309	exocyst complex component 8		KOG2215	212	129	146	348	276	16	53	49	246	226	128	259	289	259	79	35
ACC_01310	protein I'm not dead yet-like	K14445	KOG1281	539	311	359	821	567	95	220	231	507	176	213	147	290	338	241	74
ACC_01311	putative gustatory receptor 43a	K08471		71	37	49	67	48	6	21	16	183	65	46	54	91	96	15	3
ACC_01312	protein I'm not dead yet-like	K14445	KOG1281	16	5	5	15	8	0	0	0	4	8	3	3	38	14	9	1
ACC_01313	hypothetical protein			4	3	1	14	6	0	0	1	3	3	0	1	1	1	2	0
ACC_01314	polyadenylate-binding protein 1-like	K13126	KOG0123	6584	5155	2502	4886	2588	3118	3724	4163	7570	7319	1874	4577	32036	30852	29744	7225
ACC_01315	conserved hypothetical protein	K07953	KOG0077	341	196	166	568	500	31	31	32	976	634	306	1751	1362	1429	176	20
ACC_01316	transport and Golgi organization protein 11			277	168	138	295	216	6	16	12	448	263	89	483	434	623	45	15
ACC_01317	transmembrane protein 43 homolog			275	200	169	343	281	28	58	62	429	317	174	550	394	425	45	21
ACC_01318	conserved hypothetical protein		KOG1015	43	33	34	62	42	20	36	27	49	76	37	12	7	8	32	13
ACC_01319	ubiquitin-related modifier 1 homolog	K12161	KOG4146	208	118	112	222	123	53	156	195	234	114	90	151	352	520	474	314
ACC_01320	ubiquitin-conjugating enzyme E2 Q2-like	K10582	KOG0897	184	112	67	113	74	6	19	19	402	464	34	251	324	295	68	21
ACC_01321	conserved hypothetical protein		KOG0307	179	252	209	130	66	18	15	29	339	346	75	150	192	196	83	11
ACC_01322	60S ribosomal protein L17 isoform 3	K02880	KOG3353	1084	726	360	1006	724	454	856	971	1372	612	1269	1323	8072	8404	3412	2018
ACC_01323	sodium/potassium-transporting ATPase subunit alpha-like		KOG0203	179	186	114	95	75	10	22	30	155	181	55	37	58	47	16	3
ACC_01324	f-box/LRR-repeat protein 2-like isoform 2		KOG4341	283	126	99	131	137	14	22	36	406	334	107	302	506	492	81	44
ACC_01325	ubiquitin carboxyl-terminal hydrolase 31-like isofor	K11852	KOG1870	122	93	76	131	54	28	29	34	410	416	45	169	200	210	50	28
ACC_01326	glutamyl-tRNA(Gln) amidotransferase subunit B, mitochondr		KOG2438	382	280	270	355	301	37	57	45	453	395	255	380	904	1018	128	66
ACC_01327	UV excision repair protein RAD23 homolog B-like is	K10839	KOG0011	340	165	133	336	253	16	17	23	683	489	201	655	959	1219	166	54
ACC_01328	BAG family molecular chaperone regulator 2-like	K09556	KOG3633	157	84	76	132	143	12	18	20	225	208	91	287	234	193	30	19
ACC_01329	hypothetical protein			228	167	255	274	332	16	28	19	277	200	217	290	144	152	20	9
ACC_01330	u3 small nucleolar RNA-associated protein 6 homol	K14557	KOG2396	488	318	306	660	769	78	132	152	367	244	329	283	469	547	217	162
ACC_01331	conserved hypothetical protein			283	189	127	130	118	18	27	28	241	269	32	218	177	148	34	8
ACC_01332	Golgi to ER traffic protein 4 homolog		KOG3024	154	117	100	153	111	10	12	14	375	309	91	381	608	583	30	17
ACC_01333	nucleolar protein 58-like	K14565	KOG2572	929	524	450	538	586	178	410	445	488	338	334	272	2239	5022	7267	4915
ACC_01334	fizzy-related protein homolog	K03364	KOG0305	84	44	38	55	43	6	4	12	149	120	22	156	128	137	15	4
ACC_01335	meckelin-like		KOG4611	47	37	61	39	60	2	3	3	29	32	212	455	53	29	0	0
ACC_01336	WD repeat-containing protein 20-like		KOG2394	161	78	74	122	73	15	25	28	340	248	78	144	276	241	64	18
ACC_01337	conserved hypothetical protein			208	158	172	252	204	81	87	162	257	204	16	31	6	11	0	0
ACC_01338	conserved hypothetical protein			88	112	106	468	424	677	1441	1763	1538	1245	1	55	22	15	14	9
ACC_01339	conserved hypothetical protein		KOG1144	801	393	224	1408	520	272	504	443	9339	5101	568	572	739	281	465	432
ACC_01340	probable serine incorporator isoform 1		KOG2592	790	387	440	812	1002	56	54	128	1800	917	452	1369	742	454	23	11
ACC_01341	arylsulfatase B-like		KOG3867	280	277	137	331	143	62	74	119	675	528	49	177	148	20	11	10
ACC_01342	isopentenyl-diphosphate Delta-isomerase 1-like isc	K01823	KOG0142	157	150	104	216	210	27	50	38	995	594	133	333	922	878	36	15
ACC_01343	cancer-related nucleoside-triphosphatase homolog	K06928		62	42	46	48	39	5	5	3	74	40	16	77	137	97	7	0
ACC_01344	conserved hypothetical protein		KOG3587	1161	563	601	1225	1058	91	98	153	3388	1834	1090	3377	593	384	90	60
ACC_01345	tyrosine-protein phosphatase non-receptor type 14	K01104	KOG0792	225	230	207	271	241	20	31	33	257	182	113	236	270	278	77	16
ACC_01346	LOW QUALITY PROTEIN		KOG3685	2224	1576	1444	2464	1106	237	223	300	1200	613	302	100	47	73	49	28
ACC_01347	hypothetical protein			3	3	3	13	6	1	1	2	23	14	0	0	0	0	0	0
ACC_01348	conserved hypothetical protein			4	4	5	5	2	0	0	1	4	6	0	0	3	2	8	3
ACC_01349	conserved hypothetical protein		KOG4395	54	32	19	63	19	16	20	23	162	89	39	98	77	127	209	71
ACC_01350	conserved hypothetical protein			39	35	51	108	41	32	38	25	209	271	263	226	53	234	241	246
ACC_01351	proto-oncogene tyrosine-protein kinase receptor R	K05126	KOG0200	157	291	163	134	89	40	66	77	183	291	105	104	103	112	33	15
ACC_01352	conserved hypothetical protein			20	5	7	7	8	1	5	3	28	22	6	2	12	7	12	5
ACC_01353	sodium-coupled monocarboxylate transporter 1-like		KOG2349	280	102	91	216	180	5	7	16	287	96	92	142	40	45	4	1
ACC_01354	probable cation-transporting ATPase 13A1-like	K14950	KOG0209	415	317	231	308	278	47	57	56	1049	1036	201	537	1482	1147	86	36
ACC_01355	steroid receptor RNA activator 1-like		KOG0307	194	104	121	166	157	11	12	16	232	137	92	448	253	210	27	15
ACC_01356	LOW QUALITY PROTEIN	K01196	KOG3625	337	259	154	131	116	61	77	87	1623	1106	104	388	1822	688	41	7
ACC_01357	LOW QUALITY PROTEIN		KOG0915	1453	937	800	1371	1488	102	203	220	1474	1728	936	1443	1859	1923	214	105
ACC_01358	zinc transporter ZIP9-B-like	K14715	KOG3907	108	103	81	129	129	17	17	23	110	80	36	255	213	251	28	7
ACC_01359	myotrophin-like isoform 2		KOG4214	474	291	318	563	474	49	54	75	919	336	538	2111	592	1025	134	49
ACC_01360	hypothetical protein			29	11	46	213	84	14	18	31	35	14	14	25	13	2	20	16
ACC_01361	anoctamin-1-like		KOG2514	1003	615	506	940	808	81	183	187	2109	2085	445	1563	1844	1941	244	83
ACC_01362	anoctamin-1-like		KOG3142	107	59	79	271	231	9	5	5	232	113	177	320	341	268	21	7
ACC_01363	translocon-associated protein subunit delta	K04571	KOG4088	329	303	192	230	271	32	48	64	342	235	392	1470	1315	890	37	17
ACC_01364	protein crooked neck	K12869	KOG1915	252	162	216	310	304	42	79	108	227	168	145	188	306	296	81	44
ACC_01365	arf-GAP with SH3 domain, ANK repeat and PH dom	K12488	KOG0521	454	294	343	342	148	29	28	41	789	605	124	169	123	159	116	31

ACC_01366	probable N-acetyltransferase san-like		KOG3138	151	105	94	282	254	10	17	26	142	75	90	278	311	559	45	28
ACC_01367	brain protein 44-like		KOG1589	9	12	20	20	11	0	1	1	14	1	5	0	3	3	0	1
ACC_01368	sodium channel protein Nach-like		KOG4294	3	3	1	10	9	0	0	2	12	4	1	0	0	0	0	0
ACC_01369	poly(rC)-binding protein 3-like	K13162	KOG2190	1458	1259	1392	1703	298	107	138	222	743	819	98	136	117	148	397	130
ACC_01370	protein unc-80 homolog		KOG4305	2172	1493	1780	1538	522	86	97	131	1072	557	3	8	44	43	34	23
ACC_01371	tetraspanin-31-like		KOG3882	245	86	82	183	212	7	17	13	193	206	30	131	182	100	8	2
ACC_01372	DCN1-like protein 1-like		KOG3077	164	83	88	130	140	17	25	31	412	304	96	397	302	224	21	14
ACC_01373	protein lethal(2)essential for life-like		KOG3591	446	859	4105	10205	4411	695	952	1928	2338	933	929	1914	514	580	661	776
ACC_01374	protein lethal(2)essential for life-like		KOG3591	925	2622	6397	5302	1902	521	744	1265	5898	1636	4283	5772	598	207	58	47
ACC_01375	protein lethal(2)essential for life-like		KOG3591	2615	1161	1326	3717	2213	128	99	223	1502	974	496	1269	6	2	0	0
ACC_01376	protein lethal(2)essential for life-like		KOG3591	293	285	1139	4214	1326	55	90	189	976	744	264	558	1	5	14	14
ACC_01377	protein lethal(2)essential for life-like		KOG3591	2001	1546	4403	17181	5614	293	438	763	1360	361	1749	2337	182	249	185	184
ACC_01378	conserved hypothetical protein			63	28	16	20	34	5	7	4	43	30	46	183	84	84	2	1
ACC_01379	histidine-rich membrane protein KE4 homolog 2-lik	K14713	KOG2693	622	317	219	352	503	44	86	119	716	556	414	1043	1427	1003	77	35
ACC_01380	pyridine nucleotide-disulfide oxidoreductase domain-contai		KOG2755	912	703	757	708	888	133	268	258	1488	732	480	769	1031	1255	201	83
ACC_01381	28S ribosomal protein S18b, mitochondrial	K16174	KOG4021	287	213	212	124	243	14	25	21	380	328	94	466	556	592	28	12
ACC_01382	zinc finger C4H2 domain-containing protein-like		KOG4451	167	143	117	338	197	11	17	27	193	95	88	159	160	183	63	15
ACC_01383	40S ribosomal protein S19a-like	K02966	KOG3411	1591	1070	667	1914	828	584	834	1279	936	895	944	2033	4978	5419	5230	1975
ACC_01384	hypothetical protein			23	2	12	22	33	2	5	2	0	0	2	0	0	0	2	0
ACC_01385	lysine-specific histone demethylase 1A-like		KOG0029	79	45	45	106	16	15	22	35	13	53	17	6	6	11	195	188
ACC_01386	conserved hypothetical protein	K13356	KOG1221	131	110	73	349	337	45	55	59	2026	1537	225	644	637	1193	124	93
ACC_01387	mediator of RNA polymerase II transcription subun	K15131	KOG4057	317	174	170	506	445	35	42	54	270	201	207	474	518	975	148	65
ACC_01388	conserved hypothetical protein		KOG4689	481	321	261	471	582	112	302	317	669	578	547	478	559	490	459	314
ACC_01389	paraplegin-like	K09552	KOG0731	836	496	545	579	636	59	90	78	1493	996	439	851	1956	2367	196	49
ACC_01390	anaphase-promoting complex subunit 5-like	K03352	KOG4322	336	184	181	463	529	39	73	71	301	253	183	305	328	350	36	9
ACC_01391	DNA polymerase epsilon subunit 3	K02326	KOG0870	104	67	45	83	92	21	54	69	104	39	115	112	166	278	263	199
ACC_01392	conserved hypothetical protein			135	61	40	124	104	21	83	64	59	83	41	46	72	72	167	89
ACC_01393	rho GTPase-activating protein 1-like isoform 2		KOG4406	511	302	291	220	190	33	54	52	639	409	105	306	271	241	43	15
ACC_01394	LOW QUALITY PROTEIN		KOG2006	177	139	111	127	119	20	46	48	387	303	83	116	226	201	51	25
ACC_01395	conserved hypothetical protein		KOG1855	256	163	166	536	225	423	500	650	2993	1956	5	73	2241	334	62	67
ACC_01396	CCR4-NOT transcription complex subunit 10-like	K12607	KOG2471	271	238	250	267	309	28	31	31	300	311	144	336	444	400	35	8
ACC_01397	ankyrin repeat domain-containing protein 40-like		KOG0509	305	155	123	289	276	27	26	65	288	193	229	565	341	584	89	51
ACC_01398	conserved hypothetical protein			9	8	15	14	9	1	3	4	19	6	9	2	168	175	14	2
ACC_01399	conserved hypothetical protein			167	162	155	304	396	14	24	35	194	149	115	322	120	140	20	6
ACC_01400	peptidyl-tRNA hydrolase ICT1, mitochondrial-like	K15033	KOG3429	201	134	144	293	267	22	30	32	306	107	173	383	511	1067	97	53
ACC_01401	serine-threonine kinase receptor-associated protei	K13137	KOG0278	376	185	167	485	310	74	74	130	524	262	270	708	1710	1087	416	186
ACC_01402	xenotropic and polytropic retrovirus receptor 1 homolog		KOG1162	128	106	78	112	101	11	18	23	219	242	57	129	173	144	18	3
ACC_01403	hypothetical protein			1	3	3	1	1	1	1	0	3	0	0	0	0	0	0	0
ACC_01404	hypothetical protein			4	29	1	0	0	7	5	11	0	1	6	0	2	0	1	0
ACC_01405	ubiquitin-40S ribosomal protein S27a	K02977	KOG0004	1758	1258	660	2034	1416	643	1140	1420	1614	1064	1653	1927	12105	15657	4862	3748
ACC_01406	ribosome biogenesis protein NSA2 homolog	K14842	KOG3163	1854	768	523	895	828	751	1724	1525	1407	588	890	1079	2573	5400	9048	7196
ACC_01407	conserved hypothetical protein			42	33	41	93	80	3	2	7	70	33	50	207	71	92	18	8
ACC_01408	LOW QUALITY PROTEIN	K00799	KOG0867	247	205	162	444	365	47	71	75	613	289	679	3465	361	606	59	40
ACC_01409	LOW QUALITY PROTEIN	K03349	KOG2165	296	150	182	295	301	16	19	15	257	223	65	242	257	287	27	11
ACC_01410	protein real-time-like		KOG1471	857	613	592	962	937	50	84	93	992	644	234	611	667	624	75	28
ACC_01411	LOW QUALITY PROTEIN		KOG1121	239	160	142	204	187	3	18	21	271	204	101	245	235	260	33	17
ACC_01412	eukaryotic translation initiation factor 4B-like	K03258	KOG0147	445	262	185	456	321	70	99	98	988	668	178	553	1877	2743	772	462
ACC_01413	cytosolic carboxypeptidase-like protein 5-like		KOG3641	238	112	112	161	146	22	56	56	376	205	103	211	198	152	25	16
ACC_01414	mediator of RNA polymerase II transcription subun	K15133	KOG4512	184	125	136	342	255	22	31	35	424	218	129	200	454	683	148	62
ACC_01415	structural maintenance of chromosomes protein 1/	K06636	KOG0018	1933	1144	991	1271	1466	359	753	732	967	844	842	754	1263	2155	3176	2102
ACC_01416	acyl-CoA dehydrogenase family member 9, mitochondrial-lik		KOG0137	501	247	304	399	612	24	36	37	1045	541	308	836	1132	1585	49	17
ACC_01417	40S ribosomal protein S14	K02955	KOG0407	889	565	361	2196	1096	182	303	457	881	589	1986	1491	2945	3795	2194	1675
ACC_01418	double-strand break repair protein MRE11-like	K10865	KOG2310	284	219	239	331	308	37	111	103	349	180	244	294	577	667	245	174
ACC_01419	conserved hypothetical protein			75	57	52	52	44	12	23	20	74	59	41	43	35	24	19	6
ACC_01420	conserved hypothetical protein	K11343	KOG4051	246	134	142	298	295	32	88	100	251	136	181	268	244	361	221	141
ACC_01421	conserved hypothetical protein		KOG2140	193	114	112	173	138	25	70	87	133	73	66	103	118	165	136	124
ACC_01422	hypothetical protein			1	6	2	2	3	0	0	0	0	0	0	0	0	0	0	0

ACC_01423	conserved hypothetical protein		KOG2462	781	523	460	907	760	203	382	381	748	515	596	620	619	960	1599	1092
ACC_01424	conserved hypothetical protein			463	225	186	643	571	333	378	563	2376	1113	147	198	149	135	60	42
ACC_01425	protein scabrous		KOG2579	30	28	27	56	40	4	2	3	50	15	12	3	36	34	22	19
ACC_01426	type I inositol-3,4-bisphosphate 4-phosphatase-like	K01109	KOG4428	803	497	538	1118	712	68	67	101	1231	642	234	282	436	327	76	39
ACC_01427	ubiquinone biosynthesis protein COQ4 homolog, mitochond		KOG3244	175	95	99	246	184	19	32	27	200	102	124	129	219	190	40	21
ACC_01428	tRNA-dihydrouridine synthase 1-like isoform 2		KOG2335	521	345	360	651	476	80	121	127	551	208	412	243	234	308	158	93
ACC_01429	sesquipedalian-1-like		KOG0930	194	117	110	244	211	19	29	16	335	138	112	158	129	147	28	15
ACC_01430	ribosomal protein S6 kinase beta-1-like	K04688	KOG0598	303	129	134	191	152	8	10	14	245	197	37	125	208	168	12	6
ACC_01431	G-protein coupled receptor Mth-like		KOG4193	27	14	11	28	25	15	14	17	228	148	279	614	77	24	13	5
ACC_01432	F-box/WD repeat-containing protein 7-like	K10260	KOG0274	161	103	92	96	62	27	27	39	234	194	38	89	238	284	122	41
ACC_01433	LOW QUALITY PROTEIN		KOG3933	333	274	246	341	301	32	67	73	388	291	203	484	1338	1303	197	90
ACC_01434	leucine-rich repeat-containing protein 59-like		KOG0473	805	539	391	750	1158	195	445	544	468	254	404	629	643	540	405	298
ACC_01435	actin-related protein 3-like		KOG0678	681	396	377	653	623	65	64	113	1080	573	442	863	1212	1445	165	67
ACC_01436	Dosage compensation complex, subunit MLE		KOG0921	6	3	6	7	3	0	5	1	15	20	5	5	7	7	16	8
ACC_01437	conserved hypothetical protein			622	398	347	478	619	67	176	167	359	291	374	347	832	805	399	203
ACC_01438	alpha-1,6-mannosyl-glycoprotein 2-beta-N-acetylgl	K00736	KOG2791	1574	1363	1111	2267	729	527	663	764	1602	1181	476	504	132	79	124	129
ACC_01439	LOW QUALITY PROTEIN	K04688	KOG0598	350	222	143	163	108	43	60	68	974	774	107	371	996	856	123	34
ACC_01440	MORN repeat-containing protein 3-like		KOG0231	32	32	36	43	66	8	6	7	35	25	14	14	40	37	7	4
ACC_01441	transcription initiation factor TFIID subunit 7 isofor	K03132	KOG4011	237	142	135	414	348	40	71	80	311	146	191	243	466	561	135	110
ACC_01442	LOW QUALITY PROTEIN			257	166	156	215	287	12	56	56	150	221	124	193	160	110	52	40
ACC_01443	LOW QUALITY PROTEIN	K04569	KOG4656	128	124	99	222	203	12	32	39	381	182	254	484	294	310	42	14
ACC_01444	KDEL motif-containing protein 1-like		KOG2458	311	183	185	253	283	27	31	61	289	173	197	266	287	248	29	10
ACC_01445	pre-mRNA-splicing factor SYF1-like isoform 1	K12867	KOG2047	234	198	184	338	313	36	48	72	356	264	202	282	343	452	40	19
ACC_01446	LOW QUALITY PROTEIN	K14440	KOG1000	41	27	42	34	43	6	9	9	30	20	39	31	46	71	22	9
ACC_01447	sorting nexin-12-like isoform 1		KOG2527	330	154	142	434	459	39	46	92	491	222	249	472	348	393	58	30
ACC_01448	COMM domain-containing protein 5-like			61	38	35	57	92	0	3	4	23	33	50	134	94	60	13	3
ACC_01449	tubulin polyglutamylase complex subunit 2-like	K16605		38	21	11	138	71	5	8	13	21	16	89	176	6	5	3	2
ACC_01450	E3 ubiquitin-protein ligase Bre1-like	K10696	KOG0978	1507	1035	988	1858	1498	371	822	938	1684	1388	949	861	1380	1699	2051	1363
ACC_01451	conserved hypothetical protein	K10608	KOG2177	471	227	249	597	461	26	41	51	434	379	235	205	346	342	53	12
ACC_01452	kinase suppressor of Ras 2-like		KOG0193	358	215	215	503	327	37	36	45	719	368	159	323	353	289	73	14
ACC_01453	protein lin-37 homolog			129	87	77	145	134	11	24	25	156	101	85	125	193	255	67	27
ACC_01454	hypothetical protein			0	0	0	0	2	1	0	0	0	2	0	0	0	0	0	0
ACC_01455	conserved hypothetical protein			1	0	0	0	0	0	0	0	0	0	0	0	3	0	0	1
ACC_01456	hypothetical protein			1	2	0	1	0	0	0	0	0	11	0	0	0	1	0	0
ACC_01457	LOW QUALITY PROTEIN	K10592	KOG0939	2996	2337	2236	2469	1280	812	1225	1443	6710	6254	928	1050	2658	3032	2193	1234
ACC_01458	inositol monophosphatase 1-like	K01092	KOG2951	417	261	252	687	519	35	41	48	1067	551	255	1285	818	731	96	44
ACC_01459	probable helicase senataxin-like	K10706	KOG1802	474	296	217	511	542	70	172	189	386	504	220	183	423	509	526	239
ACC_01460	phenylalanyl-tRNA synthetase alpha chain B-like	K01889	KOG2784	231	216	179	348	305	15	32	38	296	317	197	555	1227	1593	138	54
ACC_01461	L-aminoadipate-semialdehyde dehydrogenase-pho	K06133	KOG0945	57	28	37	57	84	5	15	14	342	80	112	215	163	314	30	6
ACC_01462	hydroxysteroid dehydrogenase-like protein 2-like		KOG0725	229	160	146	207	276	16	22	35	852	560	295	1147	1319	1346	28	10
ACC_01463	rabenosyn-5-like	K12481	KOG1842	194	130	119	307	262	20	26	30	243	122	154	172	93	82	25	13
ACC_01464	poly(ADP-ribose) glycohydrolase ARH3-like	K11687		348	292	262	464	376	46	77	89	334	194	308	327	504	481	56	36
ACC_01465	putative inorganic phosphate cotransporter-like isoform 1		KOG2532	27	10	15	30	22	3	1	2	148	100	22	46	1392	550	55	8
ACC_01466	structural maintenance of chromosomes protein 4	K06675	KOG0996	613	453	415	677	774	122	266	296	496	447	442	265	599	840	792	397
ACC_01467	coiled-coil domain-containing protein 65-like			67	25	52	51	75	0	1	0	55	22	17	44	29	28	3	2
ACC_01468	4-aminobutyrate aminotransferase, mitochondrial-	K13524	KOG1405	1411	917	861	1891	1580	84	97	121	719	335	72	51	2238	1347	173	34
ACC_01469	conserved hypothetical protein			151	97	119	271	254	16	32	35	227	127	142	179	175	294	43	16
ACC_01470	lisH domain-containing protein ARMC9-like			145	171	143	250	269	13	35	38	35	100	127	184	46	45	9	6
ACC_01471	THO complex subunit 7 homolog	K13176	KOG3215	102	98	98	150	124	16	23	17	89	60	52	89	182	294	102	75
ACC_01472	40S ribosomal protein S12, mitochondrial-like	K02950	KOG1750	420	407	371	610	529	86	135	117	343	197	309	758	610	772	241	103
ACC_01473	conserved hypothetical protein		KOG0847	34	28	38	26	8	1	6	6	14	28	3	1	22	25	19	6
ACC_01474	ZZ-type zinc finger-containing protein 3-like		KOG4582	161	145	147	306	225	21	48	44	216	110	141	180	309	453	108	42
ACC_01475	sorting nexin-16-like		KOG2101	43	43	29	79	71	6	10	4	64	40	32	75	59	60	7	1
ACC_01476	armadillo repeat-containing protein 6 homolog		KOG4199	144	87	65	146	127	9	16	19	137	66	49	105	181	171	28	14
ACC_01477	LOW QUALITY PROTEIN		KOG1825	1034	662	675	843	637	137	152	201	1746	1594	393	367	614	639	220	66
ACC_01478	v-type proton ATPase 116 kDa subunit a isoform 1-	K02154	KOG2189	580	287	409	590	293	58	71	65	1247	549	555	1466	1127	1273	383	32
ACC_01479	conserved hypothetical protein	K00949	KOG3153	168	77	53	79	91	2	3	7	172	180	55	188	167	138	5	8

ACC_01480	t-complex protein 1 subunit eta isoform 1	K09499	KOG0361	586	356	274	949	733	32	52	71	691	578	304	1019	3310	3927	399	208
ACC_01481	LOW QUALITY PROTEIN	K07976	KOG4348	179	109	113	141	122	18	31	43	230	200	62	143	196	145	79	34
ACC_01482	alpha-1-inhibitor 3		KOG1366	4552	2476	2569	3943	2740	212	349	417	8181	11724	122	129	2717	1124	190	81
ACC_01483	vesicle-associated membrane protein/synaptobrevin-bindin		KOG0439	1167	560	499	1177	641	87	132	134	1623	1280	374	2072	1479	1765	671	284
ACC_01484	heat shock protein 90	K04079	KOG0020	17870	8125	15111	32992	29173	1493	3283	6776	9164	7887	3689	7716	20149	31325	37327	40953
ACC_01485	P protein-like isoform 2	K00274	KOG2639	8	6	5	3	5	0	1	0	8	5	23	7	10	4	2	0
ACC_01486	conserved hypothetical protein	K01104		29	13	20	101	81	0	0	3	35	7	33	59	10	12	8	2
ACC_01487	pyrroline-5-carboxylate reductase 2 isoform 2	K00286	KOG3124	200	135	129	221	272	32	28	49	219	116	117	282	361	402	60	19
ACC_01488	caspase-1-like	K04489	KOG3573	81	87	44	27	44	14	14	15	125	94	25	121	618	654	34	9
ACC_01489	3-hydroxypropionyl-coenzyme A dehydratase-like		KOG1680	164	101	105	191	205	20	74	66	270	119	146	315	525	814	172	148
ACC_01490	LOW QUALITY PROTEIN	K06642	KOG0891	1237	732	566	442	467	77	217	204	1415	1526	503	454	877	713	156	38
ACC_01491	guanosine-3',5'-bis(diphosphate) 3'-pyrophosphoh	K01139	KOG1157	73	82	66	128	107	9	10	15	97	49	69	173	116	129	19	6
ACC_01492	probable palmitoyltransferase ZDHHC6-like		KOG1314	147	98	84	120	136	11	21	21	167	113	101	216	183	215	20	10
ACC_01493	trafficking protein particle complex subunit 12-like		KOG2796	97	55	61	187	155	8	9	17	306	101	106	150	144	138	6	5
ACC_01494	probable RNA 3'-terminal phosphate cyclase-like pı	K11108	KOG3980	246	194	188	516	607	37	52	87	389	213	216	444	423	681	36	17
ACC_01495	succinyl-CoA ligase	K01899	KOG1255	1489	448	397	922	713	110	134	181	1836	803	469	1369	4304	3322	372	228
ACC_01496	luciferin 4-monooxygenase-like		KOG1176	22	21	10	1	2	4	2	1	832	324	23260	50254	3	0	1	0
ACC_01497	conserved hypothetical protein			0	1	0	1	0	0	0	0	2	1	2	4	0	0	0	0
ACC_01498	conserved hypothetical protein			2	1	0	1	0	0	0	0	12	2	6	9	3	3	1	1
ACC_01499	WD repeat domain-containing protein 83-like isofo	K13124	KOG0316	114	89	80	207	242	11	25	20	181	86	193	347	235	322	17	10
ACC_01500	probable cytosolic Fe-S cluster assembly factor AGAP009023		KOG2439	471	247	293	677	603	52	101	118	848	555	360	989	577	550	108	72
ACC_01501	hypothetical protein			175	85	130	240	176	3	9	12	486	475	112	681	550	311	113	16
ACC_01502	succinate dehydrogenase		KOG2403	285	220	62	87	57	53	41	65	531	418	205	614	459	427	80	41
ACC_01503	hypothetical protein			1045	378	414	1107	1433	133	316	253	256	117	112	24	14	75	83	44
ACC_01504	endonuclease III-like protein 1-like	K10773	KOG1921	349	211	239	439	469	22	69	58	207	81	228	106	147	305	148	100
ACC_01505	ubiquitin carboxyl-terminal hydrolase 36-like	K11855	KOG1865	238	148	136	201	83	15	54	42	297	340	74	154	521	781	287	96
ACC_01506	conserved hypothetical protein			60	46	63	35	12	9	1	6	66	69	10	2	6	8	15	11
ACC_01507	7,8-dihydro-8-oxoguanine triphosphatase-like	K03574		129	92	83	154	144	7	20	32	77	42	59	45	54	132	16	4
ACC_01508	28S ribosomal protein S23, mitochondrial-like			72	84	110	138	134	9	23	18	55	47	108	146	125	162	8	5
ACC_01509	conserved hypothetical protein			60	35	39	101	73	3	3	10	29	47	2	15	14	12	5	3
ACC_01510	v-type proton ATPase subunit F 1-like	K02151	KOG3432	373	235	289	820	622	31	65	77	664	270	278	1297	596	762	75	44
ACC_01511	ADP-ribosylation factor GTPase-activating protein 1	K12492	KOG0704	821	567	584	851	717	102	174	204	902	647	269	972	1431	1652	299	133
ACC_01512	hypothetical protein			4	0	1	1	1	0	0	0	0	1	1	0	1	2	1	0
ACC_01513	B(0,+)-type amino acid transporter 1-like	K13868	KOG1287	631	421	484	1229	615	71	83	103	1722	703	717	1520	291	270	201	47
ACC_01514	thymidylate kinase-like	K00943	KOG3327	72	49	42	58	83	2	2	5	115	62	46	186	164	153	5	1
ACC_01515	gamma-aminobutyric acid type B receptor subunit	K04615	KOG1055	730	261	225	405	234	22	25	35	432	212	120	196	3	12	5	3
ACC_01516	raf homolog serine/threonine-protein kinase phl	K02644	KOG0193	105	68	69	55	43	6	11	8	216	216	21	38	117	114	14	6
ACC_01517	deoxynucleotidyltransferase terminal-interacting protein 2-l		KOG3100	291	171	157	214	238	40	57	66	272	131	216	360	590	834	161	95
ACC_01518	high affinity copper uptake protein 1-like		KOG3386	180	134	169	213	245	9	29	20	167	85	388	248	155	181	25	6
ACC_01519	nucleolar complex protein 4 homolog B-like	K14771	KOG2154	386	281	240	720	524	55	64	93	437	345	228	468	885	1072	149	61
ACC_01520	conserved hypothetical protein		KOG3630	528	470	406	602	403	58	131	147	667	410	178	213	528	629	238	131
ACC_01521	spectrin alpha chain-like	K06114	KOG0040	3456	1715	1782	3306	1858	340	498	606	5548	3928	1723	2385	2748	2877	741	316
ACC_01522	hypothetical protein			6	4	3	2	6	1	1	3	8	8	0	0	0	0	0	1
ACC_01523	membrane-associated protein Hem	K05750	KOG1917	311	200	200	345	271	13	31	29	452	364	99	408	492	524	125	12
ACC_01524	conserved hypothetical protein		KOG2392	14	4	9	19	7	0	1	1	22	11	16	26	16	264	62	30
ACC_01525	26S proteasome non-ATPase regulatory subunit 4-l	K03029	KOG2884	1330	927	1080	2018	1068	422	843	922	2505	1271	1405	2458	3211	3554	5257	3036
ACC_01526	short/branched chain specific acyl-CoA dehydroger	K09478	KOG0139	221	172	115	375	429	29	35	38	551	349	154	423	955	731	113	132
ACC_01527	tubulin alpha chain, testis-specific-like	K07374	KOG1376	94	58	67	117	62	3	14	12	1016	290	41	74	159	180	27	7
ACC_01528	Fanconi anemia group I protein homolog	K10895	KOG4553	288	244	170	265	301	19	54	44	192	145	60	141	248	247	72	23
ACC_01529	conserved hypothetical protein			630	9	5	56	12	8	9	14	1403	33	2	1828	1	1	1	3
ACC_01530	conserved hypothetical protein			913	564	638	1705	957	249	360	391	1629	633	1145	1712	470	728	439	188
ACC_01531	adenylate cyclase type 8-like	K08048	KOG3619	1664	1390	1241	1356	688	100	97	150	1683	616	3	0	7	5	3	2
ACC_01532	pseudouridine-5'-monophosphatase-like isoform 1		KOG2914	166	122	143	311	327	14	23	32	191	140	91	206	372	454	44	10
ACC_01533	LOW QUALITY PROTEIN	K16186	KOG3887	316	191	187	605	590	25	50	56	514	357	261	720	876	906	59	16
ACC_01534	WD repeat-containing protein 8		KOG4497	88	42	74	41	77	2	3	5	97	80	62	179	202	126	3	1
ACC_01535	conserved hypothetical protein	K14328	KOG1295	1162	944	775	1011	1082	285	626	746	993	554	708	435	848	1130	1665	1203
ACC_01536	sphingosine-1-phosphate lyase	K01634	KOG1383	130	90	78	130	126	10	19	18	367	266	152	491	1041	977	39	8

ACC_01537	NADH dehydrogenase	K03939	KOG3456	1141	610	619	2235	1194	81	259	154	648	168	917	518	480	425	271	280
ACC_01538	cation transport regulator-like protein 2-like		KOG3182	145	118	106	224	245	19	41	48	91	88	155	209	137	176	60	24
ACC_01539	conserved hypothetical protein	K03960		357	251	243	592	465	17	36	44	428	191	375	759	648	677	103	67
ACC_01540	conserved hypothetical protein			966	778	748	1057	522	257	444	522	1161	486	472	705	1210	2083	4734	4250
ACC_01541	cleavage stimulation factor subunit 1-like	K14406	KOG0640	102	53	56	108	119	9	7	12	113	124	59	183	379	456	33	7
ACC_01542	protein shuttle craft-like	K12236	KOG1952	1446	736	543	968	1107	235	538	538	904	670	714	475	1176	1180	1594	811
ACC_01543	conserved hypothetical protein	K13163		235	151	172	327	151	33	58	62	449	373	115	222	229	273	221	94
ACC_01544	solute carrier family 2, facilitated glucose transporter memb		KOG0569	652	395	367	471	340	27	25	41	1450	857	106	232	776	377	24	40
ACC_01545	something about silencing protein 10-like	K14767	KOG3118	516	353	355	457	655	103	247	247	522	324	544	760	809	1109	819	421
ACC_01546	exosome component 10-like	K12591	KOG2206	975	684	562	821	821	245	592	593	1427	919	845	719	1879	2002	1521	720
ACC_01547	LOW QUALITY PROTEIN		KOG0742	315	191	190	384	210	33	50	59	311	323	121	100	1745	3428	1905	2042
ACC_01548	SET and MYND domain-containing protein 4-like			3	2	1	2	1	0	1	0	17	26	0	4	2	0	6	6
ACC_01549	metabotropic glutamate receptor 7-like	K04611	KOG1056	28	9	10	75	43	3	3	4	24	21	28	31	35	52	30	19
ACC_01550	cell differentiation protein RCD1 homolog	K12606	KOG3036	137	66	79	345	242	15	17	26	276	147	62	196	225	234	45	7
ACC_01551	NADH dehydrogenase	K03935	KOG2870	1633	759	815	2608	1856	178	231	319	3914	1543	978	2443	2244	2588	385	182
ACC_01552	conserved hypothetical protein		KOG4441	106	53	42	84	40	3	3	10	133	189	39	41	108	140	89	9
ACC_01553	phosphoglycerate kinase-like	K00927	KOG1367	1734	808	766	1272	1251	254	506	499	2488	1387	777	1796	5444	3979	584	629
ACC_01554	aldose 1-epimerase-like	K01785	KOG1604	582	356	316	868	627	53	71	82	2222	4169	394	2110	2525	1324	118	80
ACC_01555	conserved hypothetical protein			895	515	397	933	976	266	541	577	687	387	856	1325	684	890	1016	872
ACC_01556	kanadaptin-like		KOG1881	432	358	354	379	339	81	146	176	649	317	330	377	383	437	197	127
ACC_01557	hypothetical protein			116	95	76	91	29	13	30	38	23	279	21	27	9	21	292	181
ACC_01558	non-LTR retrotransposon CATS			11	4	5	6	3	0	0	0	4	8	0	0	1	0	2	3
ACC_01559	myotubularin-related protein 8-like		KOG1089	380	258	294	419	434	30	37	36	699	433	286	525	373	304	36	15
ACC_01560	ribosomal RNA processing protein 36 homolog	K14795	KOG3190	263	118	106	323	359	84	212	244	249	107	330	209	202	315	361	199
ACC_01561	transmembrane protein C3orf1 homolog		KOG4608	569	483	518	477	424	51	124	121	551	317	398	818	877	1000	280	161
ACC_01562	protein timeless homolog	K03155	KOG1974	23	18	8	33	18	3	2	2	26	14	6	16	67	136	55	18
ACC_01563	26S proteasome non-ATPase regulatory subunit 1-like isofor		KOG2062	374	165	177	473	167	49	83	125	315	258	228	138	293	479	722	848
ACC_01564	conserved hypothetical protein			475	304	372	670	1015	39	81	88	317	191	333	470	177	272	127	80
ACC_01565	synaptogyrin-2-like		KOG4016	4389	1868	1936	2744	1761	328	613	905	828	541	365	544	107	123	387	158
ACC_01566	f-actin-capping protein subunit beta-like	K10365	KOG3174	605	308	264	1053	875	75	96	111	765	451	352	932	968	992	141	63
ACC_01567	conserved hypothetical protein			30	21	4	19	13	33	86	70	258	47	3	4	87	26	5	5
ACC_01568	chromatin modifying protein 1	K12197	KOG3232	342	171	165	620	476	66	126	144	509	254	397	767	446	436	360	266
ACC_01569	conserved hypothetical protein	K09880	KOG2630	645	302	239	448	324	45	116	131	220	169	97	100	727	837	1311	693
ACC_01570	zinc finger protein 484-like		KOG2462	109	69	50	169	236	12	19	23	59	88	75	73	50	49	49	17
ACC_01571	vesicle transport protein USE1-like	K08507	KOG2678	57	53	65	115	166	2	10	12	92	64	70	157	98	99	2	1
ACC_01572	integrator complex subunit 11-like	K13148	KOG1136	112	72	60	172	196	5	10	11	135	188	91	223	323	352	56	11
ACC_01573	LOW QUALITY PROTEIN		KOG3514	11	8	12	15	4	1	5	5	7	5	7	2	0	0	0	0
ACC_01574	atlastin		KOG2037	739	497	344	595	466	171	270	288	4207	2231	2114	4231	1518	1460	269	106
ACC_01575	protein YIF1B-like		KOG3094	255	267	228	485	374	48	40	61	331	356	214	550	605	519	54	20
ACC_01576	ADP-ribosylation factor 1-like isoform 1	K07977	KOG0070	858	406	264	853	501	256	275	370	1407	1213	423	1409	2247	2638	841	315
ACC_01577	tectonin beta-propeller repeat-containing protein		KOG3669	536	393	308	245	185	67	79	90	851	632	115	177	321	271	34	21
ACC_01578	putative inositol monophosphatase 3-like	K15759	KOG3853	739	373	293	706	665	57	52	74	1018	524	420	1793	455	389	62	22
ACC_01579	histone H2A-like	K11251	KOG1756	111	38	33	140	90	26	25	28	97	133	39	124	63	84	114	60
ACC_01580	actin-interacting protein 1		KOG0318	452	252	225	366	387	29	43	73	1119	914	279	809	1231	1480	107	59
ACC_01581	60S ribosomal protein L9	K02940	KOG3255	1656	1143	709	2360	1763	407	909	1086	2073	1562	2821	2677	7718	9187	2255	1188
ACC_01582	60S ribosomal protein L23	K02894	KOG0901	1366	1609	904	2481	1334	586	755	1256	2094	1669	1801	3140	8929	11532	3009	1889
ACC_01583	hypothetical protein			0	0	0	3	0	0	0	1	1	7	1	0	0	0	1	0
ACC_01584	sentrin-specific protease 8-like	K08597	KOG3246	66	40	47	74	127	3	3	4	31	19	50	72	54	60	5	4
ACC_01585	probable cytochrome P450 305a1		KOG0156	10	4	3	6	0	1	0	0	11	13	0	39	324	136	15	5
ACC_01586	conserved hypothetical protein			29	18	17	51	27	0	2	0	107	49	11	11	539	3910	202	104
ACC_01587	male-specific lethal 2 homolog			125	84	77	225	156	17	21	43	166	128	80	157	84	91	31	7
ACC_01588	conserved hypothetical protein		KOG3803	716	439	301	487	295	525	1272	903	5162	1198	353	175	128	190	853	607
ACC_01589	EH domain-binding protein 1-like		KOG0035	677	336	281	526	373	61	114	134	888	980	195	441	350	251	131	47
ACC_01590	acyl-CoA Delta(11) desaturase-like		KOG1600	401	310	325	713	443	61	86	142	967	458	97	120	42	317	175	54
ACC_01591	Replicase polyprotein 1a			124	39	37	71	62	14	18	23	37	28	26	29	10	19	113	45
ACC_01592	conserved hypothetical protein		KOG2687	714	435	378	881	712	161	328	361	1543	1101	441	655	665	458	240	101
ACC_01593	transmembrane protein 209-like		KOG4670	599	255	192	468	522	68	96	160	484	462	359	681	637	618	164	90

ACC_01594	hypothetical protein			1	0	1	1	0	0	0	0	7	0	1	0	0	1	0	0
ACC_01595	LOW QUALITY PROTEIN	K02105	KOG4203	810	400	381	1087	535	51	65	76	871	774	269	784	483	619	309	91
ACC_01596	histone-lysine N-methyltransferase SETMAR-like			5	1	1	1	1	0	1	1	0	3	6	7	1	0	3	0
ACC_01597	hypothetical protein			2	1	0	0	0	0	0	0	1	0	0	0	0	0	1	0
ACC_01598	mitochondrial inner membrane protease ATP23 homolog		KOG3314	71	77	50	62	64	9	19	21	136	107	85	227	380	492	81	54
ACC_01599	conserved hypothetical protein			558	233	191	281	464	20	68	60	276	281	252	377	382	245	86	45
ACC_01600	ubiquitin-like protein ATG12-like	K08336	KOG3439	266	288	260	444	522	21	47	40	231	128	267	370	218	200	33	8
ACC_01601	conserved hypothetical protein			274	151	125	398	216	32	23	39	188	130	111	328	192	188	56	24
ACC_01602	gamma-tubulin complex component 6-like	K16573	KOG2001	826	688	611	773	753	68	180	210	631	554	281	271	534	422	195	94
ACC_01603	protein BUD31 homolog	K12873	KOG3404	160	155	160	308	285	13	30	31	266	107	173	235	261	575	84	61
ACC_01604	n-alpha-acetyltransferase 11, NatA catalytic subuni	K00670	KOG3235	630	426	368	847	765	118	227	220	1201	694	388	1124	1440	1668	578	155
ACC_01605	conserved hypothetical protein			233	174	240	328	398	19	41	57	209	147	244	393	382	375	86	43
ACC_01606	conserved hypothetical protein		KOG1015	4579	2541	2080	1478	2713	296	930	1057	813	971	928	432	3498	5942	7655	5928
ACC_01607	conserved hypothetical protein		KOG4737	861	399	383	772	686	25	36	40	913	517	375	1625	1040	784	27	13
ACC_01608	peroxiredoxin-like protein	K00430	KOG0854	1269	1245	1253	4190	2570	285	271	379	3093	3631	1821	3857	6177	3604	273	21
ACC_01609	glutamate	K06890	KOG2322	607	334	405	957	972	33	27	43	1637	1152	523	2207	734	622	24	9
ACC_01610	ubiquitin thioesterase trabid-like	K11862	KOG4345	306	236	195	307	162	22	16	17	676	522	85	149	189	178	41	9
ACC_01611	macrophage erythroblast attacher-like isoform 1		KOG0396	111	85	63	99	129	15	27	26	229	147	82	215	198	256	34	10
ACC_01612	hypothetical protein			5	4	0	3	1	0	1	2	1	4	1	4	2	0	2	2
ACC_01613	protein glass-like	K09214	KOG2462	30	31	34	83	58	14	21	28	209	91	31	21	65	83	22	4
ACC_01614	hypothetical protein			0	1	0	0	1	0	0	0	1	1	0	0	1	0	1	0
ACC_01615	ATP-binding cassette sub-family D member 2-like	K05676	KOG0064	117	115	99	196	127	40	58	67	535	399	254	670	788	1030	254	71
ACC_01616	conserved hypothetical protein		KOG1052	1	0	1	2	1	0	0	0	1	1	18	6	0	1	0	0
ACC_01617	conserved hypothetical protein		KOG4367	777	516	473	846	529	102	151	169	593	401	226	159	174	343	203	62
ACC_01618	RWD domain-containing protein 2A-like			118	170	154	278	276	40	65	64	136	81	116	193	238	242	46	29
ACC_01619	testis-specific serine/threonine-protein kinase 1-lik	K08811	KOG0583	7	4	5	2	3	0	0	0	15	29	3	35	20	11	7	2
ACC_01620	probable DNA-directed RNA polymerases I and III s	K03020	KOG3438	34	50	85	80	63	2	10	5	16	5	25	56	50	118	22	16
ACC_01621	conserved hypothetical protein		KOG3563	176	116	118	358	446	10	19	22	136	96	82	155	197	359	36	6
ACC_01622	bifunctional dihydrofolate reductase-thymidylate s	K00560	KOG0673	508	309	264	571	628	70	208	165	610	306	383	762	842	1322	290	102
ACC_01623	toll-interacting protein	K05402	KOG1030	372	228	226	465	301	39	49	62	1242	537	352	1192	690	642	69	21
ACC_01624	dymeclin-like		KOG2225	139	78	77	75	70	7	4	13	198	119	53	108	268	252	14	1
ACC_01625	dymeclin-like		KOG2225	70	69	32	31	17	4	1	1	102	120	18	108	210	125	5	1
ACC_01626	hypothetical protein			0	0	0	0	0	0	0	0	0	0	0	0	0	0	0	0
ACC_01627	telomerase Cajal body protein 1		KOG2919	277	216	144	325	373	37	79	96	147	109	177	157	214	198	140	55
ACC_01628	dipeptidyl aminopeptidase-like protein 6-like		KOG2100	1130	535	481	582	340	28	43	50	523	193	8	28	12	16	11	3
ACC_01629	xylosyltransferase oxt	K00771	KOG0799	215	137	142	300	333	20	28	34	401	401	66	197	352	341	26	6
ACC_01630	zinc finger protein 643-like		KOG2462	11	48	13	4	2	10	14	14	0	4	2	1	2	1	7	1
ACC_01631	28S ribosomal protein S33, mitochondrial		KOG4104	138	118	180	99	151	13	32	30	161	131	175	458	302	266	23	19
ACC_01632	chaoptin-like		KOG4194	235	116	142	191	128	6	13	9	96	60	27	16	6	10	1	1
ACC_01633	furin-like protease 1, isoforms 1/1-X/2-like			58	38	29	45	18	7	8	14	87	68	56	79	12	22	24	7
ACC_01634	furin-like protease 1, isoforms 1/1-X/2-like	K01349	KOG3526	87	98	80	110	31	34	29	31	307	294	154	282	34	37	33	22
ACC_01635	b-cell receptor-associated protein 31-like	K11353	KOG3300	299	158	147	557	544	17	32	53	527	193	260	745	670	913	46	27
ACC_01636	b-cell receptor-associated protein 31-like		KOG1962	425	216	200	461	537	29	47	66	321	209	312	484	316	227	61	28
ACC_01637	Kazrin			122	65	91	80	21	3	8	9	108	77	18	4	1	8	18	3
ACC_01638	conserved hypothetical protein		KOG0249	203	117	135	140	38	3	5	15	293	209	33	31	8	11	23	6
ACC_01639	28S ribosomal protein S15, mitochondrial-like	K02956	KOG2815	303	204	194	464	410	34	67	68	262	141	199	344	536	979	208	138
ACC_01640	ecdysone receptor isoform A	K14034	KOG4216	1121	493	525	1421	219	82	91	177	813	548	263	121	63	67	128	120
ACC_01641	glutamyl-tRNA(Gln) amidotransferase subunit A homolog		KOG1211	86	55	69	115	117	8	10	14	73	53	49	127	260	387	34	18
ACC_01642	hypothetical protein			0	0	0	0	0	0	0	0	0	0	0	0	0	0	0	4
ACC_01643	zinc transporter foi-like	K14716	KOG2693	234	121	72	133	87	12	52	41	268	289	30	100	297	227	94	49
ACC_01644	40S ribosomal protein S9-like	K02997	KOG3301	1041	926	500	1862	1391	274	494	516	1317	1001	1162	2110	7216	8486	2556	1056
ACC_01645	muscle LIM protein Mlp84B-like isoform 1		KOG1700	212	47	15	72	21	74	166	121	4031	2059	72	95	212	235	861	956
ACC_01646	RNA-binding protein 8A-like	K12876	KOG0130	258	166	159	319	278	54	85	119	222	96	245	262	616	1074	436	411
ACC_01647	putative glycerol kinase 3	K00864	KOG2517	215	132	129	241	216	21	26	26	257	163	90	125	208	264	37	7
ACC_01648	histone-lysine N-methyltransferase SETMAR-like			11	2	2	5	6	1	1	2	1	10	1	9	1	3	2	0
ACC_01649	probable cytochrome P450 6a14		KOG0158	5	3	0	1	1	1	2	1	796	671	1	1	1311	196	1	1
ACC_01650	conserved hypothetical protein			38	31	34	89	55	7	13	22	10	15	14	148	11	4	5	8

ACC_01651	importin-5		KOG2171	342	267	167	196	180	43	64	62	1549	1556	142	589	2959	2932	227	83
ACC_01652	DNA replication licensing factor mcm7-B	K02210	KOG0482	22	29	23	44	35	2	4	9	43	32	34	23	344	610	83	26
ACC_01653	nucleoredoxin-like		KOG2501	186	182	207	557	251	25	28	66	115	49	114	23	38	55	62	17
ACC_01654	INO80 complex subunit B-like			321	182	149	498	429	75	163	209	402	195	346	268	217	269	375	273
ACC_01655	BTB/POZ domain-containing adapter for CUL3-med	K15074	KOG2716	159	87	89	124	80	9	9	14	224	192	49	141	365	318	95	24
ACC_01656	conserved hypothetical protein	K01090	KOG0699	593	363	310	464	380	71	137	163	633	441	311	257	441	579	300	186
ACC_01657	hypothetical protein			41	28	30	12	6	2	3	2	15	21	4	1	1	1	2	2
ACC_01658	hypothetical protein			21	12	20	2	1	0	1	2	8	13	1	0	1	2	2	4
ACC_01659	conserved hypothetical protein			74	60	67	160	131	17	28	46	91	69	64	58	675	1492	195	155
ACC_01660	glutamate decarboxylase-like protein 1-like		KOG0629	139	74	84	142	119	5	11	21	546	395	163	629	126	85	22	6
ACC_01661	intraflagellar transport protein 172 homolog		KOG3616	134	78	79	126	63	7	8	12	103	101	375	269	15	7	14	6
ACC_01662	probable E3 ubiquitin-protein ligase RNF144A-like	K11975	KOG1815	111	52	51	75	80	2	6	7	247	140	28	97	84	79	14	0
ACC_01663	conserved hypothetical protein		KOG0612	1366	944	830	1274	1404	207	394	421	1389	981	848	358	1178	1348	843	307
ACC_01664	trafficking protein particle complex subunit 3-like		KOG3330	152	110	109	195	187	19	18	34	242	99	147	346	330	414	32	14
ACC_01665	decaprenyl-diphosphate synthase subunit 2-like	K12505	KOG0776	315	268	263	386	350	44	58	64	695	413	335	973	1131	1492	140	53
ACC_01666	LOW QUALITY PROTEIN		KOG3860	36	25	27	21	24	3	0	2	29	9	19	33	61	55	5	2
ACC_01667	transmembrane protein C9orf91 homolog			194	117	74	141	100	33	45	57	481	477	239	1262	366	334	62	21
ACC_01668	transmembrane and coiled-coil domain-containing protein 1		KOG3312	236	100	78	181	261	23	113	97	136	45	464	637	195	276	178	156
ACC_01669	DNA (cytosine-5)-methyltransferase PliMCI	K00558		524	296	261	440	383	98	167	172	558	285	296	363	636	1035	733	395
ACC_01670	iron-sulfur protein NUBPL-like	K03593	KOG3022	124	78	82	250	176	18	32	28	145	92	74	212	328	251	100	49
ACC_01671	amidophosphoribosyltransferase-like	K00764	KOG0572	492	421	403	911	753	55	73	112	1089	1991	410	290	2124	1803	232	129
ACC_01672	probable Xaa-Pro aminopeptidase 3-like	K01262	KOG2414	270	167	189	370	309	23	29	39	545	308	203	476	647	1019	64	21
ACC_01673	peptidyl-prolyl cis-trans isomerase-like 3-like	K12734	KOG0884	205	153	195	427	386	36	31	42	289	130	210	500	315	425	53	35
ACC_01674	LOW QUALITY PROTEIN	K03232	KOG1668	1142	696	530	845	751	166	250	275	664	1222	388	1227	6420	6544	1323	1344
ACC_01675	conserved hypothetical protein			115	70	71	127	102	27	57	64	78	68	42	11	25	26	36	17
ACC_01676	putative glutamate synthase	K00264	KOG0399	3964	2424	2215	2749	2471	567	784	814	12564	6877	1116	1153	2435	2163	264	114
ACC_01677	conserved hypothetical protein	K05702	KOG1892	3145	2869	2336	2181	1267	472	724	654	3767	3032	1150	636	1001	821	481	268
ACC_01678	LOW QUALITY PROTEIN			457	554	601	486	290	49	48	73	406	509	142	151	535	692	176	39
ACC_01679	nuclear pore complex protein Nup160 homolog	K14303	KOG4521	213	150	121	170	140	21	25	30	327	314	91	163	799	1102	80	46
ACC_01680	conserved hypothetical protein	K11298	KOG0527	362	332	355	720	432	98	108	131	952	452	395	716	374	474	118	41
ACC_01681	u3 small nucleolar ribonucleoprotein protein IMP4·	K14561	KOG2781	60	35	33	111	89	20	17	20	72	58	80	78	372	791	158	108
ACC_01682	hypothetical protein			1	0	0	1	0	0	1	2	4	1	2	0	2	3	9	0
ACC_01683	transcription factor RFX3-like	K09173	KOG3712	127	92	68	48	32	7	8	6	164	249	53	82	155	106	12	11
ACC_01684	39S ribosomal protein L41, mitochondrial-like		KOG4756	162	177	160	264	235	12	14	30	133	52	181	366	350	458	29	15
ACC_01685	mannose-6-phosphate isomerase-like	K01809	KOG2757	270	167	75	61	68	35	33	48	129	84	94	221	889	729	21	9
ACC_01686	septin-4-like isoform 2		KOG2655	85	49	69	249	81	4	2	7	59	20	10	31	7	9	4	3
ACC_01687	NADH dehydrogenase	K03965	KOG3466	606	400	526	849	890	30	69	72	675	214	589	1311	439	578	98	50
ACC_01688	lipase 3-like		KOG2624	440	277	327	372	319	34	52	46	412	429	123	637	743	317	63	14
ACC_01689	hypothetical protein			0	1	0	2	1	0	1	0	0	3	1	0	2	0	4	0
ACC_01690	elongation of very long chain fatty acids protein 6-like		KOG3072	105	90	38	140	133	5	23	24	1774	1972	1	1	1	2	1	1
ACC_01691	hypothetical protein			4	0	3	1	2	0	0	2	22	1	6	3	0	0	0	0
ACC_01692	protein slowmo-like isoform 1		KOG3336	401	305	251	424	220	108	113	179	975	515	354	1436	641	662	587	283
ACC_01693	ATPase WRNIP1-like		KOG2028	88	66	57	70	72	20	25	37	88	69	69	87	134	170	35	14
ACC_01694	viral IAP-associated factor homolog		KOG3170	178	153	97	189	153	22	41	57	183	140	116	211	484	567	78	65
ACC_01695	membrane metallo-endopeptidase-like 1-like		KOG3624	51	49	54	43	54	46	48	67	541	194	372	243	14	1	1	0
ACC_01696	Transposable element Tc3 transposase			2	2	1	4	2	0	0	0	2	5	0	0	1	2	3	1
ACC_01697	conserved hypothetical protein			17	16	8	13	6	0	3	9	5	2	1	1	1	1	14	3
ACC_01698	solute carrier family 41 member 1-like	K15122	KOG3788	1331	750	900	1086	563	57	49	65	1176	648	98	478	539	391	177	25
ACC_01699	conserved hypothetical protein		KOG4837	137	95	88	94	143	12	63	54	162	78	83	206	290	310	53	36
ACC_01700	ras-related C3 botulinum toxin substrate 1 isoform	K04392	KOG0393	64	35	34	37	24	1	1	3	78	98	16	71	105	87	20	7
ACC_01701	39S ribosomal protein L27, mitochondrial-like	K02899	KOG4600	140	111	119	265	266	26	44	44	125	96	183	337	405	551	68	45
ACC_01702	double-strand-break repair protein rad21 homolog		KOG1213	356	285	255	211	148	35	37	34	1385	830	205	618	963	1820	221	65
ACC_01703	hypothetical protein			0	0	0	1	0	0	0	0	0	0	0	0	0	0	0	0
ACC_01704	hypothetical protein			2	0	0	2	2	0	0	0	1	0	0	0	0	0	0	0
ACC_01705	voltage-dependent T-type calcium channel subunit alpha-1G		KOG2302	28	16	14	44	13	4	4	5	16	23	5	7	2	1	8	5
ACC_01706	spermatogenesis-associated protein 20		KOG2244	331	270	284	381	362	45	44	46	571	489	221	551	673	586	43	9
ACC_01707	conserved hypothetical protein			442	263	259	614	734	32	31	60	629	197	470	1300	1039	1009	51	32

ACC_01708	hypothetical protein			8	2	6	7	3	1	0	3	4	0	2	5	5	5	0	0
ACC_01709	sodium-independent sulfate anion transporter-like		KOG0236	32	25	30	24	24	1	4	1	24	31	9	8	345	156	9	1
ACC_01710	conserved hypothetical protein		KOG0546	674	376	397	1009	549	218	260	348	1144	647	348	242	405	739	861	363
ACC_01711	TP53RK-binding protein-like	K15901	KOG4066	77	80	80	63	111	0	2	7	26	77	62	186	187	188	5	2
ACC_01712	speckle targeted PIP5K1A-regulated poly(A) polymerase-like		KOG2277	542	268	246	355	527	16	67	73	436	362	231	346	431	408	75	36
ACC_01713	division abnormally delayed protein-like			285	130	191	152	35	12	4	22	155	155	17	211	20	26	56	22
ACC_01714	phospholipase B1, membrane-associated-like		KOG3670	10	3	0	0	0	1	2	6	14	27	9	14	0	0	0	0
ACC_01715	zinc finger protein 76-like		KOG2462	640	430	461	733	535	77	121	134	664	433	438	415	551	652	219	55
ACC_01716	HIV-1 Vpr-binding protein		KOG1832	762	391	441	381	321	54	126	93	266	186	78	38	139	277	458	215
ACC_01717	pyruvate dehydrogenase E1 component subunit be	K00162	KOG0524	830	191	189	497	323	51	71	78	1967	372	439	956	2025	2097	551	213
ACC_01718	retrovirus-related Pol polyprotein from transposon TNT 1-94			15	11	8	9	8	1	1	3	4	9	1	0	1	3	7	2
ACC_01719	hemicentin-1-like		KOG3513	32	11	16	31	10	1	3	4	19	13	3	3	1	0	0	0
ACC_01720	conserved hypothetical protein			102	82	50	171	123	16	57	70	32	27	7	7	1	3	27	4
ACC_01721	conserved hypothetical protein		KOG0260	13	3	5	21	6	2	4	0	17	31	6	6	304	211	14	19
ACC_01722	conserved hypothetical protein		KOG2462	647	549	599	591	305	129	186	223	1091	1019	307	864	925	1131	825	385
ACC_01723	TBC1 domain family member 16		KOG2224	610	302	283	495	479	23	25	27	587	314	126	225	221	184	26	8
ACC_01724	ATP-dependent DNA helicase Q1-like	K10899	KOG0353	45	44	42	66	68	2	3	2	41	21	19	34	65	63	6	0
ACC_01725	non-histone protein 10-like		KOG0526	113	81	116	193	140	24	47	38	116	56	80	127	134	234	141	109
ACC_01726	small ubiquitin-related modifier 3 isoform 1	K12160	KOG1769	130	116	126	199	154	24	23	37	314	157	77	368	490	797	82	16
ACC_01727	histone H2B.3-like	K11252	KOG1744	35	31	49	101	67	2	5	3	59	22	17	26	41	39	260	110
ACC_01728	small G protein signaling modulator 3 homolog		KOG2222	261	132	142	241	259	14	31	42	408	295	121	358	233	182	32	13
ACC_01729	DNA polymerase delta small subunit-like	K02328	KOG2732	77	53	66	112	139	10	11	16	247	89	101	117	255	471	39	15
ACC_01730	transducin-like enhancer protein 4-like		KOG0639	134	67	93	191	37	19	18	20	206	223	114	103	81	68	285	140
ACC_01731	conserved hypothetical protein		KOG4248	1903	1269	1055	843	553	172	186	186	3927	3962	554	1942	3359	3639	745	311
ACC_01732	ankyrin repeat and SAM domain-containing protein 1A-like		KOG0507	180	81	84	115	43	19	17	34	119	101	27	29	17	25	57	25
ACC_01733	ADP,ATP carrier protein-like		KOG0749	17	9	22	23	35	0	1	3	11	2	11	5	5	9	1	0
ACC_01734	flotillin-1-like isoform 1	K07192	KOG2668	921	374	322	440	328	27	28	31	733	261	228	929	263	278	75	49
ACC_01735	probable cytochrome P450 305a1	K14937	KOG0156	4	9	41	91	84	0	0	4	52	45	1	3	1652	384	11	2
ACC_01736	conserved hypothetical protein		KOG0260	5	1	1	5	2	0	2	1	15	10	14	1	399	2639	619	1786
ACC_01737	GDP-mannose 4,6 dehydratase-like	K01711	KOG1372	382	238	281	165	201	101	135	105	372	182	141	323	273	294	26	11
ACC_01738	LOW QUALITY PROTEIN	K14319	KOG1909	280	173	162	309	323	26	59	69	251	186	276	271	835	987	503	326
ACC_01739	hamartin	K07206	KOG0161	761	517	472	857	682	131	218	233	1061	640	420	389	610	628	367	131
ACC_01740	probable NADH dehydrogenase	K03940	KOG1687	1045	818	1263	6330	4110	111	172	233	2086	899	1305	1867	1048	1044	323	138
ACC_01741	anoctamin-8-like isoform 1		KOG2513	1981	1459	1984	1567	615	90	120	160	1494	901	1053	735	40	42	31	18
ACC_01742	zinc finger matrin-type protein 2-like	K12848	KOG4727	539	324	268	785	754	197	399	416	461	246	635	440	404	521	926	632
ACC_01743	conserved hypothetical protein			280	197	254	668	357	38	72	48	198	162	92	53	50	44	48	16
ACC_01744	G patch domain-containing protein 1 homolog	K13123	KOG2138	918	502	427	915	911	193	397	431	629	555	495	367	404	442	668	336
ACC_01745	ribosome biogenesis regulatory protein homolog	K14852	KOG1765	285	165	167	371	311	69	155	151	213	147	226	237	556	981	1162	894
ACC_01746	actin-related protein 2/3 complex subunit 1A	K05757	KOG1523	838	379	306	1178	769	167	193	253	1126	644	575	1343	1107	1532	682	185
ACC_01747	conserved hypothetical protein			2	0	0	3	6	1	0	0	18	24	0	2	5	36	5	2
ACC_01748	conserved hypothetical protein			11	7	11	43	8	6	1	11	20	45	2	1	29	68	33	145
ACC_01749	tyrosine-protein phosphatase non-receptor type 61	K05696	KOG0792	545	418	430	792	424	121	214	235	1254	1451	427	744	956	1210	829	317
ACC_01750	tyrosine-protein phosphatase non-receptor type 61F-like iso		KOG2618	69	55	43	77	78	7	12	13	99	36	41	59	48	60	20	8
ACC_01751	ADAMTS-like protein 3-like		KOG3538	1519	575	617	645	315	35	49	87	640	200	180	128	18	42	31	12
ACC_01752	sodium/potassium/calcium exchanger 3-like		KOG1307	71	45	65	48	41	6	13	17	70	25	52	6	1480	1826	111	8
ACC_01753	solute carrier family 35 member B1 homolog	K15275	KOG1580	340	201	115	173	207	17	35	25	641	565	224	887	991	948	49	3
ACC_01754	phospholipase D3-like	K16860	KOG3603	733	495	430	492	560	50	68	77	790	832	244	502	861	669	54	23
ACC_01755	cytochrome b-c1 complex subunit Rieske, mitochondrial-like		KOG1671	20	12	17	20	25	1	3	4	4	10	5	2	14	7	5	0
ACC_01756	cancer susceptibility candidate protein 1 homolog			152	78	66	194	126	9	11	19	52	292	28	134	29	67	9	4
ACC_01757	corticotropin releasing hormone binding protein			224	211	227	505	313	18	9	20	147	98	3	4	1	1	0	0
ACC_01758	1-acyl-sn-glycerol-3-phosphate acyltransferase alpha-like		KOG2848	36	29	14	69	42	17	23	32	497	131	8	0	35	98	35	9
ACC_01759	conserved hypothetical protein		KOG1886	543	485	463	732	238	159	308	367	634	1087	180	141	145	164	1254	736
ACC_01760	hypothetical protein			14	11	12	34	28	7	17	15	17	6	13	2	2	3	119	33
ACC_01761	conserved hypothetical protein		KOG4441	3545	2395	2304	2443	1440	351	620	766	1686	1408	708	297	750	634	376	143
ACC_01762	SPARC-related modular calcium-binding protein 1-like		KOG4578	43	32	20	69	37	11	18	11	56	46	87	107	174	161	134	166
ACC_01763	glutathione S-transferase omega-1-like		KOG0406	422	422	492	780	892	14	46	63	256	244	166	365	264	246	19	6
ACC_01764	conserved hypothetical protein		KOG1117	440	257	231	485	306	39	62	90	1310	1179	163	399	548	639	117	28

ACC_01765	conserved hypothetical protein		KOG3608	75	51	30	58	15	5	7	6	43	73	2	2	4	2	3	2
ACC_01766	conserved hypothetical protein			10	6	3	6	7	0	5	4	8	12	3	1	0	1	12	0
ACC_01767	hypothetical protein			3	0	1	1	0	0	1	0	0	0	1	0	0	0	0	0
ACC_01768	S1 RNA-binding domain-containing protein 1-like		KOG1857	959	732	686	980	1292	80	170	155	1028	549	633	604	671	864	283	155
ACC_01769	suppressor of G2 allele of SKP1 homolog isoform 2	K12795	KOG1309	376	287	222	896	891	41	50	74	765	335	413	1158	750	793	124	112
ACC_01770	sodium leak channel non-selective protein-like		KOG2301	2704	2051	2038	4065	3447	288	496	562	1953	895	790	181	414	504	192	114
ACC_01771	sodium leak channel non-selective protein	K05668	KOG0054	1410	824	647	1425	1363	195	384	417	858	917	386	518	3482	3574	937	256
ACC_01772	LOW QUALITY PROTEIN	K12898	KOG4211	993	613	647	781	503	130	160	204	1811	867	602	558	943	1218	230	118
ACC_01773	probable pyruvate dehydrogenase E1 component s	K00161	KOG0225	1100	423	363	686	751	92	94	130	2831	811	364	1353	3754	3318	433	162
ACC_01774	LOW QUALITY PROTEIN	K03006	KOG0260	819	1018	1165	573	234	81	93	105	1747	1561	198	370	989	1122	400	145
ACC_01775	eukaryotic translation initiation factor 2 subunit 2-l	K03238	KOG2768	1356	647	488	1368	1585	440	708	1325	993	753	805	863	2143	3596	3490	4178
ACC_01776	eukaryotic translation initiation factor 3 subunit L-l	K15029	KOG3677	688	420	389	806	901	60	67	99	1018	711	424	1542	3303	4073	202	62
ACC_01777	conserved hypothetical protein		KOG4635	98	63	52	162	100	1	4	8	202	160	18	197	105	51	13	3
ACC_01778	conserved hypothetical protein		KOG2075	301	172	149	164	129	10	17	27	298	164	59	49	48	64	39	12
ACC_01779	ragulator complex protein LAMTOR2-like		KOG4107	353	272	262	513	397	118	177	279	202	106	182	267	208	182	98	139
ACC_01780	coiled-coil domain-containing protein 25-like		KOG3272	319	346	332	417	458	66	118	137	347	113	270	292	420	596	663	539
ACC_01781	ubiquitin-associated domain-containing protein 1-l	K12174	KOG0944	188	144	134	212	249	8	22	19	166	162	124	303	242	278	23	9
ACC_01782	conserved hypothetical protein		KOG3712	213	200	201	251	72	15	30	25	308	550	40	169	305	281	166	60
ACC_01783	mediator of RNA polymerase II transcription subun	K15129	KOG3583	445	268	310	728	485	35	70	78	383	196	229	409	246	385	113	56
ACC_01784	conserved hypothetical protein			842	391	388	660	360	18	22	18	488	96	49	3193	7142	2436	485	343
ACC_01785	signal transducing adapter molecule 1	K04705	KOG2199	686	539	434	383	244	144	157	212	1032	1073	149	452	638	403	182	73
ACC_01786	vacuolar protein sorting-associated protein 52 homolog isof		KOG1961	461	300	306	547	458	49	70	82	626	437	177	461	546	625	83	21
ACC_01787	kinesin-like protein KIF11-like		KOG0243	86	88	73	117	116	9	38	43	64	81	39	51	385	516	239	140
ACC_01788	leukocyte elastase inhibitor-like		KOG2392	144	101	119	137	131	12	14	21	142	158	177	341	187	152	18	7
ACC_01789	phosphomannomutase 2-like	K01840	KOG3189	159	116	96	175	243	20	30	41	331	190	154	408	674	541	20	7
ACC_01790	conserved hypothetical protein			45	49	58	40	92	1	1	1	21	18	30	68	31	18	0	0
ACC_01791	regulator of gene activity-like	K12605	KOG2151	298	224	273	628	377	34	66	86	459	281	240	340	428	618	178	61
ACC_01792	conserved hypothetical protein		KOG4545	70	41	49	66	99	1	6	4	12	35	31	68	168	292	23	10
ACC_01793	conserved hypothetical protein		KOG2084	58	20	8	75	44	15	19	19	587	426	72	62	150	124	37	29
ACC_01794	sphingomyelin synthase-related 1		KOG3058	106	67	36	84	99	6	5	12	196	126	51	137	170	161	6	2
ACC_01795	conserved hypothetical protein			8	2	5	0	3	2	0	0	1	1	42	5	2	2	0	0
ACC_01796	conserved hypothetical protein			0	2	5	9	10	2	2	0	1	1	0	0	1	0	0	2
ACC_01797	conserved hypothetical protein			2	0	3	6	11	0	1	0	1	4	1	0	71	27	2	0
ACC_01798	guanine nucleotide-binding protein G(s) subunit al	K04632	KOG0099	447	329	309	534	192	181	200	360	332	390	80	52	67	89	151	51
ACC_01799	protein 4.1 homolog	K06107	KOG3527	8256	3635	3649	3880	4226	1223	2536	2897	4938	2296	3716	3443	2094	4355	4344	1549
ACC_01800	NADH dehydrogenase	K03948		2607	2447	3087	4289	2717	317	454	589	3972	1020	2019	3744	1217	1409	256	192
ACC_01801	conserved hypothetical protein	K02877	KOG1678	1281	697	370	1927	1008	353	450	552	1792	1307	1453	2551	8152	8316	2329	978
ACC_01802	tubulin beta-1 chain	K07375	KOG1375	9076	4076	3960	15533	5119	596	748	1279	7632	3935	4567	8770	9206	12713	4440	3217
ACC_01803	anaphase-promoting complex subunit 11-like	K03358	KOG1493	65	27	35	133	51	6	2	5	73	58	31	47	30	28	20	7
ACC_01804	sodium-dependent neutral amino acid transporter	K05048	KOG3659	2529	888	1216	2545	793	131	186	244	1294	825	521	196	42	83	151	100
ACC_01805	5'-AMP-activated protein kinase subunit beta-1 iso	K07199	KOG1616	472	276	333	857	774	41	77	84	620	300	431	853	392	450	49	17
ACC_01806	activated RNA polymerase II transcriptional coactivator p15-		KOG2712	564	416	445	505	747	91	172	184	398	197	410	1055	867	1069	457	393
ACC_01807	probable UDP-glucose 4-epimerase-like	K01784	KOG1371	357	290	194	300	288	123	141	127	730	509	79	172	3571	2126	144	37
ACC_01808	conserved hypothetical protein			2	1	1	4	3	0	0	1	1	0	0	0	267	14	7	2
ACC_01809	vesicle-fusing ATPase 1-like isoform 2	K06027	KOG0741	2438	928	881	1218	1161	106	193	233	1799	1126	176	472	320	268	61	43
ACC_01810	cytohesin-1-like, partial			8	0	2	8	0	0	2	0	29	27	1	14	8	1	3	3
ACC_01811	transmembrane protein 208-like		KOG3269	93	59	62	93	88	3	12	11	121	64	40	157	136	221	20	6
ACC_01812	LOW QUALITY PROTEIN		KOG0156	64	59	46	94	77	6	9	15	165	98	65	16	990	1006	62	65
ACC_01813	laminin subunit alpha-like	K06240	KOG1836	54	24	18	31	35	19	25	27	879	2853	15	170	2600	2498	528	157
ACC_01814	PQ-loop repeat-containing protein 3-like		KOG3211	155	82	116	186	227	5	8	13	83	53	37	50	55	72	8	2
ACC_01815	ubiquitin-conjugating enzyme E2 G2	K04555	KOG0426	253	147	139	476	299	38	59	75	285	143	245	350	222	319	69	67
ACC_01816	chitin-binding domain 3 protein	K03933		13	13	10	22	13	2	1	2	9	26	93	70	10	8	4	0
ACC_01817	conserved hypothetical protein	K02728	KOG0178	349	285	252	492	576	37	55	83	1040	668	533	2726	1715	1933	157	45
ACC_01818	conserved hypothetical protein	K02728	KOG4376	111	78	76	160	231	4	17	17	51	73	67	125	90	82	30	11
ACC_01819	40S ribosomal protein S13	K02953	KOG0400	689	784	403	1082	776	210	284	555	670	578	791	1358	5075	4838	1030	537
ACC_01820	DDB1- and CUL4-associated factor-like 1-like	K11789	KOG1832	550	317	327	487	395	54	94	97	690	711	171	219	897	987	757	431
ACC_01821	conserved hypothetical protein	K13191	KOG0132	1147	780	728	944	546	178	310	320	1652	1272	443	425	903	1138	1899	1533

ACC_01822	DNA excision repair protein ERCC-1	K10849	KOG2841	134	150	141	251	180	22	34	34	109	101	89	159	344	422	36	23
ACC_01823	conserved hypothetical protein	K09188	KOG4443	4379	4630	4188	2971	1470	841	1397	1733	4834	3954	846	467	1328	1590	1989	1017
ACC_01824	sperm-associated antigen 1-like		KOG0548	10	5	10	5	16	1	1	3	3	3	201	401	4	2	0	1
ACC_01825	LOW QUALITY PROTEIN	K10734	KOG1106	30	48	33	47	40	5	6	7	100	35	45	82	95	152	10	5
ACC_01826	hypothetical protein			738	323	368	822	692	107	204	225	251	40	117	64	167	182	396	177
ACC_01827	40S ribosomal protein S26	K02976	KOG1768	688	649	339	883	748	267	347	588	749	560	690	890	4427	4855	1682	934
ACC_01828	conserved hypothetical protein			0	0	0	0	0	0	0	0	1	1	0	0	2	0	0	0
ACC_01829	LOW QUALITY PROTEIN		KOG0480	23	13	17	4	19	1	1	1	11	44	40	20	71	59	8	4
ACC_01830	39S ribosomal protein L37, mitochondrial		KOG4461	292	179	189	295	384	15	33	30	333	215	155	609	967	1313	50	48
ACC_01831	60S acidic ribosomal protein P1	K02942	KOG1762	2406	1567	1099	4396	2716	728	1332	1585	1870	1481	3999	3936	5487	6130	4097	2635
ACC_01832	RNA polymerase I, large subunit		KOG0262	194	107	87	51	52	34	49	73	131	105	28	57	54	62	144	61
ACC_01833	hypothetical protein			4	1	0	0	0	0	0	2	2	1	1	0	0	0	6	1
ACC_01834	protein GPR107-like		KOG2569	614	521	371	491	522	50	74	113	1039	881	194	633	925	570	24	7
ACC_01835	putative serine protease K12H4.7-like		KOG2182	1	0	0	8	2	0	0	0	3	0	6	1	665	960	214	6
ACC_01836	conserved hypothetical protein	K02085	KOG2122	2039	1958	1879	3322	1133	176	223	315	2512	1988	1349	819	293	362	190	55
ACC_01837	conserved hypothetical protein			27	13	29	52	31	1	4	7	29	8	33	13	18	21	4	1
ACC_01838	conserved hypothetical protein		KOG4735	333	75	108	102	88	1	5	5	302	228	26	283	511	120	15	5
ACC_01839	conserved hypothetical protein			132	78	68	89	136	11	25	28	108	58	73	82	220	330	257	153
ACC_01840	allatostatins precursor			67	39	51	179	43	7	13	13	49	42	0	0	3	45	31	3
ACC_01841	probable ATP-dependent RNA helicase DDX17-like	K12823	KOG0331	443	427	364	472	162	80	113	107	1656	2302	127	591	465	342	319	117
ACC_01842	glucose dehydrogenase		KOG1238	5	2	6	21	1	1	4	1	171	399	2	3	10	17	12	2
ACC_01843	glucose dehydrogenase		KOG1238	22	7	4	28	12	7	5	8	1610	1274	1910	530	22	49	42	14
ACC_01844	glucose dehydrogenase		KOG1238	428	109	259	1097	439	79	112	99	8159	15711	89	22	322	795	277	58
ACC_01845	ribonuclease H1	K03469	KOG3752	249	143	163	193	223	35	96	104	241	95	196	286	266	286	164	95
ACC_01846	conserved hypothetical protein			1	1	0	0	0	0	0	1	1	4	0	0	0	0	0	0
ACC_01847	protein RCC2 homolog		KOG1427	134	69	63	159	120	12	27	28	101	102	70	85	206	243	82	45
ACC_01848	protein N-terminal asparagine amidohydrolase-like	K14662		328	189	173	353	179	98	122	142	1126	3044	274	424	1752	693	306	145
ACC_01849	lysosomal alpha-glucosidase-like	K12316	KOG1065	932	579	511	767	909	89	150	150	1913	1171	719	1629	1792	1996	234	61
ACC_01850	cytochrome c oxidase assembly protein COX11, mi	K02258	KOG2540	369	237	235	527	503	40	42	63	867	337	288	715	668	609	61	26
ACC_01851	meteorin precursor			78	50	51	216	125	16	24	36	203	62	108	49	91	99	47	17
ACC_01852	conserved hypothetical protein			1	4	4	3	4	0	0	1	24	43	5	5	122	154	15	6
ACC_01853	LOW QUALITY PROTEIN	K10858	KOG1978	323	216	223	299	358	10	26	19	195	146	167	249	254	210	24	11
ACC_01854	nuclear pore complex protein Nup98-Nup96	K14297	KOG0845	2288	1415	1381	2078	1442	352	398	502	3925	2946	1120	1194	1773	1935	430	168
ACC_01855	conserved hypothetical protein		KOG0161	304	219	203	198	178	46	85	79	273	134	173	99	181	259	150	79
ACC_01856	conserved hypothetical protein		KOG0161	439	256	186	431	655	19	118	97	252	330	383	238	319	230	204	102
ACC_01857	dehydrogenase/reductase SDR family member 11-like		KOG1205	693	186	195	468	286	96	115	183	5844	859	17	337	2	1	1	1
ACC_01858	MAP kinase-activating death domain protein-like		KOG3570	2306	1613	1789	926	339	86	114	140	1825	1282	207	263	37	60	52	12
ACC_01859	transcription termination factor 2	K15173	KOG4439	470	320	216	310	286	52	112	144	537	402	204	422	580	655	221	120
ACC_01860	conserved hypothetical protein			1215	804	945	2082	1574	155	285	379	794	432	465	591	181	209	190	88
ACC_01861	conserved hypothetical protein		KOG3523	151	141	120	275	186	47	55	62	836	581	376	616	433	484	193	72
ACC_01862	probable GDP-fucose transporter	K15279	KOG1442	192	125	147	103	149	9	16	27	129	76	73	102	339	274	21	3
ACC_01863	actin-related protein 6-like	K11662	KOG0680	216	162	173	370	324	16	29	35	208	135	144	287	344	533	63	31
ACC_01864	dynein heavy chain 7, axonemal-like isoform 1		KOG3595	66	87	67	150	80	16	20	21	228	656	7	7	85	54	25	27
ACC_01865	muskelin-like		KOG2437	144	119	111	97	91	6	15	14	319	280	60	155	227	234	30	13
ACC_01866	ubiquitin-protein ligase E3C-like	K10589	KOG0942	269	177	172	350	251	29	48	48	563	618	127	353	640	595	105	43
ACC_01867	conserved hypothetical protein	K08867	KOG0584	7767	5967	5493	5564	3461	1072	2104	2083	8670	6341	2209	1366	2291	2192	2293	1125
ACC_01868	conserved hypothetical protein			21	7	15	80	27	5	7	9	94	39	59	11	16	28	12	9
ACC_01869	conserved hypothetical protein		KOG1215	37	29	25	47	14	13	37	21	173	102	61	39	25	18	28	9
ACC_01870	tRNA (uracil-5-)-methyltransferase homolog A-like	K15332	KOG2187	314	161	175	526	431	31	58	71	302	178	156	215	292	371	113	94
ACC_01871	alpha-actinin, sarcomeric-like isoform 2	K05699	KOG0035	1386	388	283	638	338	375	810	683	4236	2285	312	501	2153	1466	2108	1584
ACC_01872	conserved hypothetical protein			904	449	335	597	944	56	167	210	505	638	676	826	683	575	331	117
ACC_01873	transcription initiation factor TFIID subunit 6	K03131	KOG2549	379	367	381	538	300	45	49	101	429	267	175	283	257	293	100	46
ACC_01874	symplekin	K06100	KOG1895	349	226	204	346	288	60	93	112	613	431	272	290	619	599	464	203
ACC_01875	3'-5' exoribonuclease CSL4 homolog	K07573	KOG3409	74	71	75	199	210	8	4	18	120	42	104	159	113	175	28	12
ACC_01876	minor histocompatibility antigen H13-like	K09595	KOG2443	1161	1103	591	1001	764	273	351	433	1595	1351	541	1965	3560	2785	221	102
ACC_01877	conserved hypothetical protein		KOG0147	2317	2102	2157	2179	1024	369	564	596	2699	1787	726	626	1947	3282	3547	2736
ACC_01878	conserved hypothetical protein	K08818	KOG0663	561	391	396	534	440	92	168	172	721	485	303	339	497	529	440	409

ACC_01879	1D-myo-inositol-trisphosphate 3-kinase isoform C	K00911	KOG1621	621	482	618	1401	881	129	215	245	1012	516	210	306	378	584	293	165
ACC_01880	interference hedgehog-like		KOG3513	694	539	441	621	411	153	223	302	1185	1008	227	463	662	576	367	116
ACC_01881	zinc finger FYVE domain-containing protein 1-like		KOG1818	167	98	86	223	204	15	24	27	500	270	122	203	236	216	18	5
ACC_01882	proline-rich nuclear receptor coactivator 2-like isoform 1			267	212	228	136	59	20	11	24	400	590	32	111	22	21	16	5
ACC_01883	conserved hypothetical protein	K03233	KOG1015	1687	1454	1315	1618	656	321	435	518	1887	1426	365	310	445	776	1305	603
ACC_01884	conserved hypothetical protein	K03233	KOG1627	1857	1202	700	1562	1348	440	790	674	2670	2233	840	2546	14879	15024	6569	3585
ACC_01885	eukaryotic translation initiation factor 3 subunit F-l	K03249	KOG2975	267	174	147	566	390	67	65	81	510	287	381	859	1536	2689	465	162
ACC_01886	conserved hypothetical protein			20	11	25	16	26	2	1	1	27	13	7	4	134	181	10	0
ACC_01887	Williams-Beuren syndrome chromosomal region 16 protein I		KOG1427	116	77	78	200	191	14	21	24	275	136	156	161	222	339	47	14
ACC_01888	E3 ubiquitin-protein ligase hyd-like	K10593	KOG0943	1910	1687	1357	1874	1213	339	503	652	3534	3244	925	1114	2452	2854	809	414
ACC_01889	multidrug resistance-associated protein 4-like		KOG0054	50	27	12	42	31	66	102	82	4843	3025	64	58	36	24	10	6
ACC_01890	multidrug resistance-associated protein 4-like		KOG0054	356	178	173	314	359	25	20	33	1374	960	530	1090	856	771	91	27
ACC_01891	4-hydroxybutyrate coenzyme A transferase-like		KOG2828	1591	873	927	1889	1637	89	100	130	2190	1194	530	2231	3208	3030	395	122
ACC_01892	armadillo repeat-containing protein 3-like			31	26	27	87	94	3	7	7	88	27	37	21	29	57	18	10
ACC_01893	conserved hypothetical protein		KOG1015	997	914	839	1110	1048	164	469	503	420	305	336	88	85	112	297	154
ACC_01894	coatomer subunit epsilon		KOG3081	426	305	196	419	439	57	63	72	797	535	176	1179	2351	1571	130	33
ACC_01895	conserved hypothetical protein		KOG3548	732	442	344	886	815	123	368	427	872	1394	638	578	1256	1461	1376	807
ACC_01896	conserved hypothetical protein		KOG2693	9038	4819	6305	6988	4129	619	748	1070	11388	4137	1297	1170	197	287	163	83
ACC_01897	28S ribosomal protein S2, mitochondrial	K02967	KOG0832	173	96	66	207	252	8	11	13	211	209	117	362	526	534	32	8
ACC_01898	prohormone-4			246	86	82	208	125	7	12	17	566	352	51	212	52	46	7	5
ACC_01899	mannose-1-phosphate guanyltransferase beta-like	K00966	KOG1322	1159	764	440	999	980	317	487	538	940	943	433	1366	3695	6210	1068	593
ACC_01900	H/ACA ribonucleoprotein complex subunit 2-like pr	K11129	KOG3167	93	48	37	78	74	10	16	17	49	64	42	82	658	923	177	150
ACC_01901	ubiquitin-conjugating enzyme E2 L3-like isoform 2	K04552	KOG0422	360	262	224	584	451	57	64	92	484	353	216	749	623	629	110	85
ACC_01902	transmembrane protein 183-like			280	198	186	393	381	49	74	96	270	218	258	404	265	249	71	34
ACC_01903	serine/threonine-protein kinase Ial	K08850	KOG0580	40	23	28	50	50	3	5	5	55	14	26	42	126	207	25	6
ACC_01904	ATP-binding cassette sub-family B member 10, mit	K05657	KOG0058	608	421	421	700	761	60	115	109	1283	696	623	954	1069	1405	185	59
ACC_01905	cytidine deaminase-like	K01489	KOG0833	116	129	126	193	191	22	23	28	144	80	132	161	249	288	38	11
ACC_01906	gametocyte-specific factor 1-like		KOG4376	123	95	68	94	89	24	24	23	151	76	90	167	509	493	77	44
ACC_01907	ubiA prenyltransferase domain-containing protein 1 homolo		KOG4581	308	309	196	277	239	18	35	35	596	479	176	678	1012	785	64	11
ACC_01908	conserved hypothetical protein			863	789	427	737	601	231	254	354	1754	1764	476	1067	1452	1256	251	38
ACC_01909	conserved hypothetical protein			232	173	199	185	238	6	55	40	126	148	142	483	300	250	26	13
ACC_01910	conserved hypothetical protein		KOG4193	1141	749	737	1410	851	63	83	132	809	365	343	505	401	323	100	43
ACC_01911	conserved hypothetical protein		KOG1661	643	425	455	1075	634	148	207	252	1224	656	553	788	453	283	99	33
ACC_01912	replication factor C subunit 5-like	K10756	KOG0990	100	81	80	157	149	7	9	15	77	61	97	139	249	606	46	32
ACC_01913	conserved hypothetical protein			453	304	300	485	450	36	62	72	460	255	262	299	280	354	85	37
ACC_01914	transmembrane protein 64-like isoform 1		KOG3140	198	103	83	112	156	11	8	8	241	246	26	192	222	146	6	0
ACC_01915	26S proteasome non-ATPase regulatory subunit 2	K03028	KOG2005	691	307	285	629	511	76	148	152	1614	1682	662	1657	3049	3228	1682	1074
ACC_01916	putative odorant receptor 63a-like			73	38	59	55	62	4	12	15	26	27	48	218	19	32	18	5
ACC_01917	annexin-B11-like		KOG0819	499	396	375	777	601	41	83	73	1279	761	303	1185	824	881	116	54
ACC_01918	galactosylgalactosylxylosylprotein 3-beta-glucuron	K10812	KOG1476	291	150	164	443	225	20	16	23	649	484	92	285	883	754	184	26
ACC_01919	conserved hypothetical protein			33	22	14	42	30	1	4	5	33	19	13	28	24	160	31	5
ACC_01920	peroxisomal N(1)-acetyl-spermine/spermidine oxidase-like		KOG0685	159	99	66	145	111	21	29	29	277	169	54	150	111	137	48	20
ACC_01921	acyl-CoA synthetase short-chain family member 3,	K01908	KOG1175	63	36	29	65	36	24	66	47	126	104	65	34	157	441	60	24
ACC_01922	conserved hypothetical protein		KOG1031	596	391	311	411	327	65	133	120	987	701	258	188	525	371	140	54
ACC_01923	transmembrane GTPase Marf		KOG0448	554	372	386	591	493	38	51	59	2136	1358	249	789	1092	1329	140	34
ACC_01924	conserved hypothetical protein		KOG3091	263	218	215	369	167	40	49	67	377	219	244	168	305	258	80	36
ACC_01925	cysteine protease ATG4D-like	K08342	KOG2674	138	93	90	160	147	9	13	10	190	118	48	65	74	69	6	4
ACC_01926	glutaryl-CoA dehydrogenase, mitochondrial	K00252	KOG0138	142	74	123	150	98	10	11	12	129	550	164	171	1562	2809	1008	1147
ACC_01927	ubiquitin carboxyl-terminal hydrolase calypso-like	K08588	KOG2778	182	132	88	189	130	18	19	18	188	195	80	246	158	204	62	14
ACC_01928	epidermal retinol dehydrogenase 2-like	K15734	KOG1201	27	21	28	34	30	1	4	7	16	28	35	18	252	84	4	10
ACC_01929	FAS-associated factor 2-B-like		KOG1363	199	101	90	175	120	29	34	61	492	472	80	395	745	612	105	29
ACC_01930	2-hydroxyacyl-CoA lyase 1-like	K12261	KOG1185	186	197	133	142	168	55	85	83	3086	919	263	423	526	204	20	13
ACC_01931	probable Bax inhibitor 1		KOG1629	2824	2037	1811	5751	4028	566	673	1070	11577	4290	6301	15395	8487	6920	544	279
ACC_01932	mitochondrial Rho GTPase-like isoform 2		KOG1707	350	217	241	460	451	33	39	61	362	321	180	393	646	677	123	45
ACC_01933	conserved hypothetical protein		KOG4661	865	920	985	622	281	132	170	196	960	648	168	112	383	380	1146	821
ACC_01934	hypothetical protein			1438	1482	1484	419	199	200	199	263	1117	660	274	126	447	506	359	130
ACC_01935	cation transport regulator-like protein 1-like		KOG3182	62	68	85	137	139	39	30	39	467	152	47	78	101	117	38	6

ACC_01936	UPF0598 protein C8orf82 homolog			150	88	82	137	222	12	21	26	345	141	138	401	254	214	23	8
ACC_01937	protein big brother isoform 1		KOG4785	154	59	115	310	78	8	6	23	71	104	8	40	10	12	63	46
ACC_01938	WD repeat-containing protein 59 isoform 1		KOG0309	646	336	352	715	611	63	98	107	855	449	366	342	473	452	120	67
ACC_01939	ADP-ribosylation factor-like protein 2-binding prote	K16742		114	92	97	180	337	5	9	5	38	93	104	240	145	41	8	3
ACC_01940	endoplasmic reticulum-Golgi intermediate compartment pro		KOG2667	501	359	370	718	700	37	73	64	905	549	334	1313	1228	1575	201	39
ACC_01941	meiotic recombination protein SPO11	K10878	KOG2795	1	0	0	1	0	0	0	0	0	1	2	0	2	0	4	0
ACC_01942	cyclic AMP-dependent transcription factor ATF-6 be	K09054	KOG4343	1363	971	884	924	892	97	242	312	783	930	629	816	1286	1345	483	239
ACC_01943	conserved hypothetical protein			221	242	220	231	271	3	15	8	105	159	124	537	289	203	7	7
ACC_01944	slowpoke-binding protein		KOG0667	748	352	357	756	612	54	123	142	746	343	349	452	243	297	77	16
ACC_01945	melanoma-associated antigen G1-like isoform 2		KOG4562	175	125	129	218	206	4	24	19	128	69	125	288	212	300	68	25
ACC_01946	cdc42 homolog	K04393	KOG0393	227	138	123	458	325	13	20	25	229	156	148	689	452	458	83	22
ACC_01947	hypothetical protein			13	66	20	17	7	2	20	10	11	96	4	0	8	10	18	3
ACC_01948	non-LTR retrotransposon R1Bmks ORF2 protein			4	2	0	0	1	0	0	1	1	2	0	0	1	0	1	1
ACC_01949	HBS1-like protein-like	K14416	KOG0458	798	458	412	870	992	86	180	179	795	919	240	1141	1155	1084	179	73
ACC_01950	plasma alpha-L-fucosidase	K01206	KOG3340	637	749	620	578	594	109	156	159	1236	1048	576	1953	1471	1314	103	14
ACC_01951	lisH domain-containing protein C16orf63 homolog	K16535		89	53	47	94	122	8	10	21	52	46	89	112	223	241	29	13
ACC_01952	structural maintenance of chromosomes protein 6-like		KOG0250	224	175	145	258	393	44	91	109	333	212	266	183	231	222	171	83
ACC_01953	antitrypsin-like		KOG2392	695	505	449	593	633	87	69	93	9307	2960	763	2286	1318	937	79	23
ACC_01954	hypothetical protein			1	0	1	2	2	0	1	0	1	0	0	0	0	0	1	4
ACC_01955	protein SCAI-like			595	286	359	729	118	42	51	67	193	233	47	19	0	12	98	25
ACC_01956	conserved hypothetical protein		KOG0307	979	726	894	1591	486	47	68	93	284	154	162	84	19	55	100	36
ACC_01957	NAD-dependent deacetylase sirtuin-7	K11417	KOG1905	293	150	144	261	165	8	17	26	216	148	79	170	137	122	25	13
ACC_01958	rho GTPase-activating protein 17-like		KOG4270	1396	994	846	577	406	113	201	175	827	780	278	364	822	798	328	119
ACC_01959	conserved hypothetical protein			0	0	0	0	0	0	1	1	4	0	0	0	0	0	2	0
ACC_01960	conserved hypothetical protein			23	16	21	24	30	2	1	6	14	4	4	1	0	3	2	1
ACC_01961	guanine nucleotide-binding protein G(o) subunit alpha-like is		KOG0082	114	42	54	104	39	4	6	12	35	15	9	11	9	9	9	4
ACC_01962	LOW QUALITY PROTEIN	K01456	KOG0909	421	175	164	345	376	35	30	52	543	626	231	687	490	584	48	18
ACC_01963	ribosome production factor 2 homolog, partial	K14847	KOG3031	525	323	237	359	527	56	205	222	346	241	343	677	940	1190	578	343
ACC_01964	very low-density lipoprotein receptor-like		KOG1215	585	242	233	316	260	33	43	65	996	1399	109	200	332	165	31	16
ACC_01965	hypothetical protein			1	1	0	1	0	0	0	0	1	1	0	1	0	0	1	0
ACC_01966	RING finger protein 157-like		KOG4265	726	603	664	915	671	70	104	120	741	403	444	361	427	458	73	38
ACC_01967	ras-related protein Rab-8A-like isoform 2	K07901	KOG0078	384	189	168	299	269	25	99	93	284	260	306	439	391	415	231	139
ACC_01968	conserved hypothetical protein		KOG0921	27	6	13	31	11	1	4	9	25	11	3	23	1525	4257	2050	2828
ACC_01969	putative fatty acyl-CoA reductase CG5065-like		KOG1221	25	26	28	27	47	2	0	4	33	32	53	14	2478	3010	84	45
ACC_01970	conserved hypothetical protein			1314	641	650	1024	946	180	378	428	1457	1189	605	844	822	661	368	168
ACC_01971	conserved hypothetical protein	K14791	KOG0270	232	79	54	138	139	28	81	96	119	214	120	109	643	1003	438	287
ACC_01972	LOW QUALITY PROTEIN	K13806	KOG2088	222	137	101	162	78	81	101	136	364	243	19	9	31	37	53	11
ACC_01973	RNA-binding protein 45-like		KOG0144	183	135	121	288	161	20	36	48	335	221	118	189	211	256	61	23
ACC_01974	6-phosphogluconate dehydrogenase, decarboxylat	K00033	KOG2653	387	241	148	501	439	85	89	111	3157	1643	948	2524	8854	5447	456	187
ACC_01975	phosphatidylinositol N-acetylglucosaminyltransfera	K03861	KOG2257	72	65	56	100	93	7	11	29	62	37	71	152	137	171	26	8
ACC_01976	phosphatidylinositol N-acetylglucosaminyltransfera	K15902		99	89	93	103	102	11	15	26	79	61	79	268	156	180	18	3
ACC_01977	conserved hypothetical protein			2	0	0	0	1	0	0	0	0	4	2	1	3	2	0	0
ACC_01978	alkaline phosphatase, tissue-nonspecific isozyme-like		KOG0971	4	4	8	16	7	1	1	3	5	0	0	0	0	0	4	0
ACC_01979	epsin-2	K12471	KOG2056	755	619	531	339	173	45	57	66	2231	1448	230	514	869	730	119	36
ACC_01980	prefoldin subunit 4-like	K09550	KOG1760	237	142	184	358	334	14	108	105	206	77	258	343	248	383	237	178
ACC_01981	cell division cycle 7-related protein kinase-like	K02214	KOG1167	88	78	61	112	121	8	28	19	133	120	67	196	262	233	55	19
ACC_01982	conserved hypothetical protein		KOG2655	173	139	100	119	95	27	28	47	400	291	78	378	497	559	120	41
ACC_01983	eukaryotic translation initiation factor 4H-like		KOG0108	590	355	376	683	492	44	56	87	1050	788	169	453	1290	1979	691	154
ACC_01984	chromobox protein homolog 1-like	K11585	KOG1911	767	380	337	624	421	77	138	173	500	379	215	703	1008	1970	1447	1226
ACC_01985	probable ATP-dependent RNA helicase DDX10-like	K14776	KOG0343	589	341	272	376	376	138	312	342	488	445	273	291	869	1464	1114	758
ACC_01986	serine/threonine-protein phosphatase 6 regulatory	K15501	KOG2073	750	552	487	596	526	117	158	187	1168	940	343	590	1216	1508	223	73
ACC_01987	valyl-tRNA synthetase, mitochondrial-like		KOG0432	375	256	161	225	282	29	47	64	517	431	175	199	678	473	36	7
ACC_01988	conserved hypothetical protein			0	0	2	0	1	0	0	0	4	1	2	1	3	5	2	1
ACC_01989	venom carboxylesterase-6-like		KOG1516	21	44	21	118	68	102	120	177	2071	566	1831	1713	612	875	646	147
ACC_01990	transcriptional adapter 2-alpha-like	K03012	KOG2351	41	38	31	69	58	4	11	20	13	5	25	25	51	85	33	23
ACC_01991	transcriptional adapter 2-alpha-like	K11314	KOG0457	109	89	85	131	80	19	23	45	61	32	34	42	58	95	23	22
ACC_01992	popeye domain-containing protein 3-like			33	15	20	30	30	0	1	1	26	17	1	9	4	3	0	1

ACC_01993	LYR motif-containing protein 7-like isoform 2			73	92	75	60	88	10	23	32	97	55	53	138	80	144	25	27
ACC_01994	homeobox protein rough-like	K09362	KOG0489	1	0	1	5	3	0	0	0	3	1	1	0	1	3	2	0
ACC_01995	LOW QUALITY PROTEIN	K01529	KOG0952	1584	1129	884	1709	1820	222	520	542	1870	1906	944	831	694	748	554	239
ACC_01996	histidyl-tRNA synthetase, cytoplasmic-like isoform	K01892	KOG1936	914	423	368	1245	948	109	147	212	950	499	281	755	1380	1975	607	230
ACC_01997	transmembrane protein 47-like isoform 2		KOG4671	98	29	34	26	22	2	7	4	160	103	31	84	103	65	9	2
ACC_01998	arginine/serine-rich protein PNISR-like	K13170	KOG4307	933	697	741	2004	837	154	307	346	920	518	592	277	328	460	1003	562
ACC_01999	leucine-zipper-like transcription regulator 1		KOG0379	195	132	143	384	298	17	33	48	135	92	142	31	72	108	54	32
ACC_02000	conserved hypothetical protein			19	17	24	15	17	1	9	4	40	11	12	5	1859	739	9	211
ACC_02001	conserved hypothetical protein			1519	838	730	806	312	36	34	48	1335	602	22	405	501	194	135	66
ACC_02002	proton-coupled amino acid transporter 4	K14209	KOG1304	47	80	36	42	41	12	20	16	445	899	26	171	227	145	17	2
ACC_02003	reverse transcriptase			2	5	0	1	1	0	0	0	0	4	1	0	0	0	1	1
ACC_02004	integrin beta-PS-like		KOG1226	569	328	301	295	274	60	98	120	1532	926	203	469	794	749	152	54
ACC_02005	glyoxylate reductase/hydroxypyruvate reductase-li	K00049	KOG0069	245	184	175	273	292	38	56	41	372	184	169	365	672	736	70	25
ACC_02006	bifunctional methylenetetrahydrofolate dehydrogenase/cyc		KOG0089	135	110	82	140	165	18	31	38	137	77	83	161	202	235	44	18
ACC_02007	conserved hypothetical protein	K08596	KOG0779	100	68	77	155	132	19	39	30	139	97	48	48	197	332	94	28
ACC_02008	hypothetical protein			0	0	0	0	0	0	0	0	0	0	0	0	0	0	0	0
ACC_02009	flavin-containing monooxygenase FMO GS-OX-like 3-like		KOG1399	12	23	12	8	8	9	12	29	261	138	68	298	260	42	17	5
ACC_02010	nucleoporin seh1-A	K14299	KOG2445	104	78	80	183	130	6	7	17	111	77	51	119	205	285	24	13
ACC_02011	elongation factor 2-like isoform 1	K03234	KOG0469	6057	4617	3245	9205	6272	1482	1569	1772	17475	14776	5124	14108	33700	33580	4928	811
ACC_02012	conserved hypothetical protein			287	229	300	456	168	61	112	93	1271	955	164	178	1373	1231	841	407
ACC_02013	carbonic anhydrase 2	K01672	KOG0382	46	24	87	278	145	13	21	24	424	322	158	914	163	76	20	12
ACC_02014	heat shock protein 75 kDa, mitochondrial isoform	K09488	KOG0019	362	265	203	573	553	33	42	66	535	667	224	692	2583	4664	313	88
ACC_02015	putative elongator complex protein 4-like	K11375	KOG3949	152	108	116	162	116	12	14	18	98	69	78	147	260	409	52	17
ACC_02016	conserved hypothetical protein		KOG2504	480	236	257	323	251	31	53	52	1048	878	145	399	766	878	92	4
ACC_02017	conserved hypothetical protein			9	6	9	21	12	1	3	3	7	1	11	24	13	8	1	1
ACC_02018	conserved hypothetical protein			185	132	134	453	364	14	15	26	170	132	130	277	483	546	58	32
ACC_02019	peroxisomal acyl-coenzyme A oxidase 3-like	K00232	KOG0135	304	236	167	177	173	69	89	81	4320	2081	103	175	598	535	50	16
ACC_02020	coiled-coil domain-containing protein 19, mitochondrial-like		KOG1029	121	104	136	92	91	39	132	87	167	201	129	32	53	42	246	101
ACC_02021	glutathione S-transferase theta-1-like	K00799	KOG0867	127	89	93	134	186	9	12	25	208	175	108	163	311	190	10	11
ACC_02022	conserved hypothetical protein		KOG0161	147	83	58	133	105	9	28	33	141	145	83	90	118	59	50	18
ACC_02023	NSFL1 cofactor p47-like	K14012	KOG2086	559	352	326	718	575	100	173	149	1082	538	500	1458	1096	1229	334	306
ACC_02024	conserved hypothetical protein		KOG0670	685	433	312	519	147	55	56	85	545	165	11	16	20	45	21	1
ACC_02025	hypothetical protein			6	3	3	4	0	0	1	1	2	3	0	0	0	0	0	0
ACC_02026	ADP-ribosylation factor-like protein 1-like isoform	K07942	KOG0072	145	85	85	159	154	18	23	38	184	76	98	210	341	434	49	26
ACC_02027	conserved hypothetical protein		KOG3639	95	96	91	83	55	10	10	10	160	50	125	251	84	78	7	1
ACC_02028	e3 ubiquitin-protein ligase RNF14-like	K11971	KOG1814	367	228	169	359	483	16	57	57	346	280	238	318	490	458	84	23
ACC_02029	beta-1,4-mannosyl-glycoprotein 4-beta-N-acetylglu	K00737		234	166	184	256	286	26	58	50	238	147	111	100	131	86	13	3
ACC_02030	carbohydrate sulfotransferase 8-like		KOG4651	15	7	17	105	56	2	2	3	5	8	43	36	13	23	10	7
ACC_02031	conserved hypothetical protein			1146	493	429	871	918	172	383	445	872	1012	545	676	1148	581	719	584
ACC_02032	conserved hypothetical protein			411	280	259	385	234	23	57	51	589	586	96	420	437	450	74	13
ACC_02033	4-hydroxybenzoate polyprenyltransferase, mitochc	K06125	KOG1381	326	183	201	357	357	31	50	50	548	325	171	524	358	473	60	24
ACC_02034	conserved hypothetical protein		KOG1551	115	60	71	155	157	6	17	30	124	95	81	94	464	335	38	17
ACC_02035	transmembrane protein 131-like isoform 1		KOG3620	822	483	456	823	592	140	262	307	787	822	301	172	683	522	292	176
ACC_02036	probable cytochrome P450 301a1, mitochondrial		KOG0159	8	9	4	6	2	1	1	2	14	19	4	42	34	68	14	14
ACC_02037	gamma-tubulin complex component 2	K16569	KOG2001	167	110	101	185	184	15	9	19	116	167	62	216	227	176	16	10
ACC_02038	gonadal protein gdl		KOG4810	39	33	38	81	66	7	15	8	43	22	51	37	45	93	23	16
ACC_02039	adrenodoxin, mitochondrial-like		KOG3309	51	35	32	85	93	2	9	9	97	70	25	70	210	306	37	22
ACC_02040	nitrilase homolog 1-like		KOG0806	70	41	43	107	87	14	29	27	139	92	70	125	238	158	33	17
ACC_02041	nitrilase homolog 1-like		KOG4315	722	497	430	603	691	148	294	370	547	254	433	457	731	1006	836	492
ACC_02042	conserved hypothetical protein		KOG0161	576	330	357	626	428	93	207	174	269	159	136	50	169	211	682	766
ACC_02043	hypothetical protein			0	0	0	0	1	0	0	0	4	0	1	5	2	1	3	0
ACC_02044	AP-2 complex subunit alpha isoform 1	K11824	KOG1077	506	202	200	622	367	27	44	57	523	768	121	384	535	494	71	22
ACC_02045	nischarin-like		KOG1259	107	52	36	83	63	3	8	3	232	156	67	180	178	129	8	2
ACC_02046	serine/threonine-protein kinase PAK 1-like		KOG0578	43	29	22	32	24	3	2	1	95	75	9	80	147	136	6	1
ACC_02047	otoferlin-like		KOG1326	563	404	412	810	566	67	72	86	527	398	147	51	33	57	19	10
ACC_02048	conserved hypothetical protein		KOG1221	11	3	8	50	29	3	6	12	25	33	247	39	236	154	14	14
ACC_02049	conserved hypothetical protein	K03135	KOG3219	195	175	156	192	198	35	80	89	105	86	102	112	282	382	359	367

ACC_02050	lamin Dm0-like	K07611	KOG0977	502	352	313	598	405	34	57	70	1074	945	298	1152	1821	1964	269	92
ACC_02051	prenylcysteine oxidase-like	K05906	KOG0029	273	247	252	356	456	13	26	38	385	531	224	835	1006	808	48	9
ACC_02052	CTL-like protein 1-like	K06515	KOG1362	127	85	68	101	127	2	3	4	322	176	65	271	332	216	7	1
ACC_02053	transmembrane protein 115-like		KOG2890	139	61	47	111	103	11	26	33	253	213	59	149	232	196	60	26
ACC_02054	prostaglandin reductase 1-like	K13948	KOG1196	161	112	68	357	313	15	31	40	532	342	401	949	5288	2114	149	59
ACC_02055	HEAT repeat-containing protein 5B-like isoform 2		KOG1822	984	746	639	507	383	59	81	111	1143	1202	261	356	711	475	58	25
ACC_02056	transcriptional repressor protein YY1-like isoform 2	K09201	KOG2462	317	228	196	496	283	48	27	78	668	483	207	539	602	647	188	35
ACC_02057	sugar phosphate exchanger 2-like isoform 1	K13783	KOG2533	242	163	161	454	390	44	38	49	404	229	182	268	247	360	58	12
ACC_02058	CB1 cannabinoid receptor-interacting protein 1-like			13	5	7	22	12	0	1	4	18	10	38	139	2	1	1	6
ACC_02059	phosphatidylserine decarboxylase proenzyme-like	K01613	KOG2420	81	60	72	100	144	4	8	8	82	111	47	193	246	206	10	10
ACC_02060	proton-coupled folate transporter-like	K14613	KOG2816	350	253	313	346	382	30	48	54	494	309	571	884	405	260	21	3
ACC_02061	conserved hypothetical protein	K10606	KOG3268	191	191	191	198	273	9	20	20	268	198	321	435	261	192	11	8
ACC_02062	26S proteasome non-ATPase regulatory subunit 13	K03039	KOG2908	455	398	328	791	780	49	55	51	1083	796	563	2025	1940	1991	174	39
ACC_02063	probable citrate synthase 2, mitochondrial-like	K01647	KOG2617	1805	316	292	846	805	56	59	74	3893	1311	817	3867	4957	5021	412	177
ACC_02064	putative transmembrane protein 185B-like		KOG3879	103	58	53	50	52	7	13	4	158	117	37	98	146	126	15	7
ACC_02065	putative fatty acyl-CoA reductase CG8306-like		KOG1221	657	250	192	688	957	55	80	85	614	481	610	1844	1176	1820	193	137
ACC_02066	chromatin-remodeling complex ATPase chain Iswi-l	K11654	KOG0385	554	332	286	679	374	78	136	161	571	441	255	414	471	816	971	487
ACC_02067	LOW QUALITY PROTEIN		KOG2514	16	14	6	56	29	25	38	62	485	362	608	800	9	5	4	6
ACC_02068	conserved hypothetical protein			0	2	3	4	0	0	0	3	5	1	0	1	0	0	0	0
ACC_02069	protein transport protein SFT2-like		KOG2887	45	45	30	95	93	6	4	15	60	44	33	156	160	96	23	5
ACC_02070	SPRY domain-containing SOCS box protein 1-like, p	K10343	KOG3953	325	129	186	209	64	15	15	23	435	418	29	74	38	69	178	173
ACC_02071	conserved hypothetical protein			256	135	175	324	354	26	39	62	393	265	224	577	240	198	42	21
ACC_02072	spermatogenesis-associated protein 13-like		KOG3519	835	466	409	626	239	123	156	165	744	464	324	139	21	34	48	45
ACC_02073	conserved hypothetical protein		KOG1764	408	246	204	257	158	28	41	67	1222	1216	51	279	243	240	44	8
ACC_02074	conserved hypothetical protein			170	126	109	127	154	11	27	26	268	155	181	574	552	607	44	27
ACC_02075	facilitated trehalose transporter Tret1-like		KOG0254	1	1	2	1	1	0	1	0	3	1	0	0	0	1	0	2
ACC_02076	elongation of very long chain fatty acids protein 1-like		KOG3071	25	12	9	48	32	16	20	27	364	12	10	2	2643	336	7	53
ACC_02077	hypothetical protein			15	12	8	14	7	0	5	6	7	10	1	1	0	0	0	0
ACC_02078	graves disease carrier protein homolog	K15084	KOG0752	37	22	21	51	58	4	8	3	105	57	33	118	97	98	8	4
ACC_02079	innexin inx2			315	69	42	46	36	12	13	16	864	335	23	351	457	374	48	20
ACC_02080	xaa-Pro dipeptidase-like	K14213	KOG2737	814	451	440	889	687	92	180	204	721	503	447	964	1235	1106	283	144
ACC_02081	LOW QUALITY PROTEIN	K07526	KOG3565	1401	1309	1490	1361	515	139	176	225	2252	1105	207	318	409	396	178	76
ACC_02082	inositol monophosphatase 2-like	K01092	KOG2951	803	414	446	1398	1145	110	128	169	1984	436	963	1794	1215	1584	227	118
ACC_02083	snRNA-activating protein complex subunit 3-like	K15210	KOG2664	133	79	78	156	209	10	28	21	136	72	93	114	139	181	34	12
ACC_02084	ATP synthase-coupling factor 6, mitochondrial	K02131	KOG4634	1235	837	890	1932	1233	96	137	161	1053	245	1328	2060	966	1303	259	274
ACC_02085	LOW QUALITY PROTEIN	K09311	KOG0489	0	0	0	0	0	0	0	0	1	1	0	0	7	3	30	21
ACC_02086	conserved hypothetical protein	K14648	KOG2849	3448	4090	5788	9917	7572	190	228	444	10822	6581	822	7789	1039	673	80	27
ACC_02087	NAD-dependent deacetylase sirtuin-2	K11412	KOG2682	407	182	189	421	450	46	73	91	663	343	265	736	657	785	162	109
ACC_02088	3-hydroxyacyl-CoA dehydratase 2-like	K10703	KOG3187	128	111	67	163	193	17	17	34	265	265	174	917	774	1144	62	30
ACC_02089	conserved hypothetical protein		KOG4026	113	63	73	103	132	5	19	19	200	64	190	549	134	215	16	7
ACC_02090	deoxyribodipyrimidine photo-lyase	K01669	KOG0133	290	291	359	135	169	42	77	81	392	204	149	200	177	138	40	25
ACC_02091	thioredoxin domain-containing protein 5-like	K13984	KOG0191	292	166	174	326	328	23	20	33	367	248	191	585	158	202	28	5
ACC_02092	conserved hypothetical protein			247	128	102	202	297	27	83	98	61	81	109	107	173	184	239	136
ACC_02093	hypothetical protein			0	0	0	0	0	0	0	0	1	1	0	0	3	1	4	2
ACC_02094	purine nucleoside phosphorylase-like	K03783	KOG3984	189	108	169	336	238	37	27	41	1659	411	21	94	311	243	162	147
ACC_02095	TWiK family of potassium channels protein 7-like isoform 1		KOG1418	20	8	11	51	26	2	2	5	23	37	6	19	13	7	3	1
ACC_02096	conserved hypothetical protein			7	4	2	2	6	0	1	1	2	9	4	4	26	53	23	4
ACC_02097	conserved hypothetical protein	K12826	KOG0227	306	326	340	387	203	28	33	80	348	193	152	385	645	1070	307	205
ACC_02098	nuclear RNA export factor 2-like		KOG3763	118	94	124	206	272	8	16	24	122	122	67	87	87	79	12	5
ACC_02099	LOW QUALITY PROTEIN	K13120	KOG3410	1170	407	361	959	777	202	527	556	510	267	489	754	418	675	957	881
ACC_02100	ADP-ribosylation factor-like protein 8B-A-like	K07956	KOG0075	342	193	177	433	390	31	34	41	406	246	210	647	319	217	50	18
ACC_02101	cAMP-dependent protein kinase type II regulatory :	K04739	KOG1113	417	308	225	398	203	15	18	34	538	421	37	148	106	76	63	19
ACC_02102	U6 snRNA-associated Sm-like protein LSm2-like	K12621	KOG3448	45	32	47	120	102	2	7	7	22	17	58	86	113	160	63	29
ACC_02103	conserved hypothetical protein			5	8	5	8	2	0	1	0	10	7	3	8	22	16	8	2
ACC_02104	elongation of very long chain fatty acids protein AAEL00800		KOG3071	13	3	3	19	10	2	9	2	90	81	489	577	106	97	40	10
ACC_02105	hypothetical protein			4	3	2	1	3	1	1	2	0	0	0	0	0	0	0	0
ACC_02106	conserved hypothetical protein			26	20	5	5	3	10	21	24	59	39	10	175	252	112	22	40

ACC_02107	RNA 3'-terminal phosphate cyclase-like	K01974	KOG3980	117	109	125	247	247	16	24	31	160	112	122	197	322	506	38	21
ACC_02108	probable ribosome biogenesis protein RLP24-like	K02896	KOG1723	354	261	186	399	321	108	258	337	529	287	372	459	885	1430	1923	3021
ACC_02109	cyclin-G2-like	K10146	KOG0653	355	243	210	460	389	42	59	63	807	487	339	1093	265	178	50	12
ACC_02110	conserved hypothetical protein	K11478	KOG1246	1759	1031	861	1815	1626	261	580	706	1059	692	683	264	380	362	547	261
ACC_02111	CCR4-NOT transcription complex subunit 1 isoform	K12604	KOG1831	601	530	500	537	404	94	113	176	1302	1565	286	555	1500	1348	274	69
ACC_02112	negative elongation factor B-like	K15180		246	178	159	358	334	32	42	46	430	218	196	375	506	518	92	24
ACC_02113	conserved hypothetical protein	K16530		2	2	3	1	1	1	0	1	0	2	0	0	0	4	0	0
ACC_02114	calpain-C		KOG0045	315	120	122	382	304	14	14	29	352	193	162	264	150	334	61	14
ACC_02115	conserved hypothetical protein		KOG2397	73	63	70	150	109	7	10	14	137	76	75	156	104	132	15	3
ACC_02116	conserved hypothetical protein		KOG0078	177	109	111	136	120	7	7	5	140	96	40	65	4	3	2	0
ACC_02117	phospholipase B1, membrane-associated-like		KOG3670	25	21	16	135	88	7	12	13	320	12	339	4248	10	5	1	0
ACC_02118	embryonic polarity protein dorsal			16	3	20	33	31	0	4	3	11	8	4	27	0	1	0	0
ACC_02119	DTW domain-containing protein 2-like		KOG4382	26	23	20	54	39	4	2	8	40	19	21	42	47	58	8	2
ACC_02120	transmembrane protein nessy-like	K13515	KOG2705	41	25	12	10	16	1	4	1	115	182	20	124	83	34	2	2
ACC_02121	conserved hypothetical protein			479	288	329	601	217	86	141	183	435	264	190	103	58	51	191	76
ACC_02122	conserved hypothetical protein		KOG4441	674	339	328	721	374	70	95	136	1006	699	193	203	150	133	165	75
ACC_02123	CD63 antigen		KOG3882	677	787	245	187	171	257	156	248	517	366	550	957	1241	1564	89	21
ACC_02124	CD63 antigen isoform 1	K06497	KOG3882	249	236	125	398	415	76	80	115	2016	968	503	1514	977	662	38	23
ACC_02125	bifunctional coenzyme A synthase-like	K02318	KOG3220	835	429	302	733	1135	74	255	282	910	893	705	1288	1186	880	336	171
ACC_02126	conserved hypothetical protein		KOG4177	14	24	14	9	6	2	3	3	7	2	9	21	13	9	1	2
ACC_02127	staphylococcal nuclease domain-containing proteir	K15979	KOG2039	1234	967	409	895	708	245	439	476	1252	1852	455	1138	6373	6070	1667	780
ACC_02128	hypothetical protein			23	6	5	14	5	0	0	2	0	0	0	0	0	0	2	1
ACC_02129	hypothetical protein			2	1	1	1	1	0	0	0	1	1	0	0	0	0	0	0
ACC_02130	conserved hypothetical protein			10	8	4	9	4	1	1	1	6	14	4	1	0	1	1	2
ACC_02131	hypothetical protein			6	3	3	2	0	0	0	1	1	3	1	0	0	1	1	0
ACC_02132	kinesin light chain-like	K10407	KOG1840	1331	815	863	1011	434	140	244	289	1117	888	254	504	301	286	288	167
ACC_02133	protein transport protein Sec61 subunit gamma-lik	K07342	KOG3498	163	136	90	459	188	25	41	70	139	83	288	243	979	769	111	75
ACC_02134	sortilin-related receptor-like		KOG1215	1601	1213	1437	1886	948	149	134	197	4919	6758	395	803	965	849	196	70
ACC_02135	equilibrative nucleoside transporter 4 isoform 1	K03323	KOG1479	896	487	460	522	320	20	16	35	1167	1214	161	149	15	13	1	0
ACC_02136	V-type proton ATPase catalytic subunit A-like	K02145	KOG1352	1896	792	778	1315	1487	76	103	138	3000	1834	877	3407	3151	3189	230	60
ACC_02137	DNA/RNA-binding protein KIN17	K13102	KOG2837	152	143	136	196	171	28	47	43	206	103	106	195	243	215	170	151
ACC_02138	transcription factor AP-1	K04448	KOG0837	201	167	224	481	167	294	291	473	657	709	189	250	47	70	558	418
ACC_02139	glutaminase kidney isoform, mitochondrial isoform	K01425	KOG0506	993	611	752	971	439	170	304	329	1302	585	142	111	17	33	64	38
ACC_02140	protein still life, isoform SIF type 1-like	K05731	KOG3519	15653	10444	11258	26863	8812	1400	2053	2871	4601	2771	2422	444	84	131	619	304
ACC_02141	dipeptidase 1-like isoform 2		KOG4127	301	154	182	381	247	30	50	39	211	119	120	123	1	11	5	8
ACC_02142	histidine triad nucleotide-binding protein 3-like		KOG4359	19	16	12	25	17	0	2	2	25	28	16	70	60	32	4	0
ACC_02143	probable cytochrome P450 12a5, mitochondrial-like		KOG0159	4	6	9	5	1	5	6	5	0	2	5	0	3	8	17	2
ACC_02144	protein NPC2 homolog	K13443	KOG4063	92	79	70	147	154	26	26	41	793	224	8412	12547	236	142	24	10
ACC_02145	protein FAM8A1-like		KOG4647	222	146	147	349	248	39	48	59	430	199	234	421	261	279	31	16
ACC_02146	ELKS/RAB6-interacting/CAST family member 1		KOG0161	9603	5377	5933	7827	2611	439	797	979	2909	1089	1040	121	42	192	593	319
ACC_02147	bruchpilot		KOG4809	404	226	280	372	89	22	11	15	129	106	44	9	5	9	49	24
ACC_02148	probable elongation factor 1-delta	K15410	KOG1668	1579	770	557	1901	1484	186	397	416	627	568	596	906	2356	2624	713	300
ACC_02149	transmembrane protein 199-like			363	235	242	476	434	20	43	42	285	222	210	594	360	387	44	13
ACC_02150	Protein Muted-like protein			579	354	284	455	370	80	163	154	784	550	253	1076	656	469	228	132
ACC_02151	transmembrane protein 214-like		KOG4467	3746	1367	927	2135	2755	465	1148	1351	1716	1319	823	1537	2007	2327	2238	1689
ACC_02152	conserved hypothetical protein		KOG3885	264	306	403	1353	561	39	75	83	466	127	220	650	30	37	63	28
ACC_02153	poly(A) polymerase gamma	K14376	KOG2245	420	308	337	643	300	46	71	124	438	323	166	131	157	247	156	41
ACC_02154	conserved hypothetical protein			12394	5638	1776	8319	3074	4514	7736	20245	9386	6820	2697	4915	162	183	67838	69108
ACC_02155	tyrosine aminotransferase-like	K00815	KOG0259	174	20	20	72	25	13	19	15	380	239	22	290	401	489	399	96
ACC_02156	collagen alpha-1(IV) chain-like isoform 1	K06237	KOG3546	905	73	70	154	38	26	39	49	2914	695	60	2843	2318	1477	1235	604
ACC_02157	conserved hypothetical protein		KOG0961	521	295	255	320	301	44	61	77	869	960	260	861	1894	1681	245	112
ACC_02158	pro-resilin		KOG0921	7	5	6	8	8	0	0	0	8	4	4	2	122	45	4	19
ACC_02159	beta-galactosidase-like	K12309	KOG0496	413	455	314	880	514	186	164	262	1518	2140	77	277	2371	1287	292	63
ACC_02160	LDLR chaperone boca		KOG4357	203	215	169	265	269	38	59	53	202	64	149	292	366	348	61	56
ACC_02161	conserved hypothetical protein			691	239	313	1915	1234	33	47	69	1339	791	89	232	253	105	13	28
ACC_02162	conserved hypothetical protein		KOG0921	1319	833	2364	13510	6265	616	817	872	53650	51991	8825	12820	40	7	8	11
ACC_02163	yrdC domain-containing protein, mitochondrial-like		KOG3051	266	196	192	469	414	30	39	45	320	183	153	376	588	1077	188	42

ACC_02164	DNA-directed RNA polymerase III subunit RPC10-lik	K03019	KOG2906	60	91	81	127	105	4	13	17	121	96	45	208	337	319	92	29
ACC_02165	conserved hypothetical protein			37	17	23	44	14	4	2	4	34	8	15	22	1	2	4	0
ACC_02166	zinc finger protein 569-like		KOG2462	417	235	327	572	353	34	46	65	172	78	161	319	2	20	13	6
ACC_02167	serine protease 15		KOG3627	14	11	17	46	16	1	2	2	0	3	0	1	6	2	2	4
ACC_02168	transient receptor potential cation channel CG34123		KOG3614	139	65	41	98	44	7	15	17	280	316	56	116	194	208	49	12
ACC_02169	polyglutamine-binding protein 1-like	K12865	KOG3427	354	222	240	622	507	53	98	146	231	188	217	395	362	474	269	199
ACC_02170	protein strawberry notch-like		KOG1513	1113	688	673	753	421	85	76	120	1256	1383	146	349	481	804	319	134
ACC_02171	conserved hypothetical protein			264	180	208	306	462	5	21	13	215	326	199	430	344	257	31	8
ACC_02172	conserved hypothetical protein	K00774	KOG0653	27	23	8	8	11	0	0	5	33	19	4	21	112	184	34	24
ACC_02173	dnaJ homolog subfamily C member 3-like	K09523	KOG0624	283	247	215	280	269	34	38	59	557	741	114	504	1043	1017	117	34
ACC_02174	hypothetical protein			0	0	0	1	0	0	1	0	1	0	0	0	0	0	0	0
ACC_02175	myeloid differentiation primary response protein M	K04729	KOG1745	258	226	177	328	353	49	112	103	371	233	314	461	388	367	154	83
ACC_02176	DNA-directed RNA polymerase II subunit RPB11	K03008	KOG4392	434	626	747	818	619	26	58	80	207	177	282	562	140	238	47	28
ACC_02177	u4/U6.U5 tri-snRNP-associated protein 1-like isofor	K11984	KOG2217	503	349	363	726	530	117	184	220	689	377	326	290	449	669	482	251
ACC_02178	dual specificity protein phosphatase CDC14A-like	K06639	KOG1720	7	2	5	11	8	3	4	9	16	7	3	1	6	35	7	12
ACC_02179	disks large homolog 5-like isoform 2		KOG0708	399	316	225	247	64	23	38	57	590	633	32	118	156	135	51	29
ACC_02180	RNA-binding protein 4.1-like, partial		KOG0109	1903	1209	1218	1364	468	261	352	451	3140	1498	1171	1023	1584	2225	1144	655
ACC_02181	conserved hypothetical protein			60	20	11	37	19	15	14	13	135	121	92	94	35	80	41	9
ACC_02182	39S ribosomal protein L38, mitochondrial		KOG3346	396	243	224	411	391	23	44	53	495	334	272	459	982	1621	133	54
ACC_02183	zinc finger protein 330 homolog		KOG1832	725	345	439	669	394	70	227	221	484	351	231	362	341	461	423	370
ACC_02184	transaldolase	K00616	KOG2772	626	527	471	1017	988	100	132	145	4166	2741	1068	4505	15321	6031	636	322
ACC_02185	UDP-galactose translocator	K15272	KOG2234	205	125	123	196	264	16	10	14	227	121	88	330	375	374	18	8
ACC_02186	60S ribosomal protein L10a-like	K02865	KOG1570	1659	889	429	1038	608	365	626	637	897	902	1237	1114	7912	10049	5822	2948
ACC_02187	apoptosis-inducing factor 1, mitochondrial isoform 1		KOG1346	529	303	273	607	597	50	68	79	652	703	167	692	1728	2574	241	92
ACC_02188	regulator of telomere elongation helicase 1 homolo	K11136	KOG1132	370	262	206	249	259	15	60	48	444	272	308	307	432	394	83	31
ACC_02189	arylsulfatase B-like		KOG3867	44	3	6	12	7	0	2	2	35	15	14	9	10	301	30	3
ACC_02190	dehydrogenase/reductase SDR family member 11-like		KOG1205	370	307	216	595	364	99	111	178	1918	363	10774	12130	1265	887	94	48
ACC_02191	phosphoglycolate phosphatase-like	K01101	KOG2882	968	774	1063	2847	3332	45	74	114	1525	572	631	2224	1300	1343	112	24
ACC_02192	conserved hypothetical protein		KOG0612	1425	989	834	1696	1647	266	598	615	1123	1449	1158	363	389	466	831	332
ACC_02193	conserved hypothetical protein		KOG0956	777	708	751	1277	766	106	155	219	1604	1007	372	642	898	952	653	250
ACC_02194	conserved hypothetical protein		KOG0107	555	313	326	226	134	51	79	62	1747	779	263	661	1157	1126	209	88
ACC_02195	NEDD8-activating enzyme E1 catalytic subunit-like	K10686	KOG2015	454	282	344	558	560	54	107	110	560	306	310	408	511	816	167	61
ACC_02196	icarapin precursor			6671	8220	8494	17533	6558	1643	1458	2209	19393	10495	4910	11777	1215	738	452	207
ACC_02197	gastrula zinc finger protein XlCGF26.1-like isoform 1		KOG2462	0	0	0	0	0	0	0	0	1	1	0	1	0	0	0	0
ACC_02198	conserved hypothetical protein	K10573	KOG0419	517	401	362	769	377	99	73	129	1588	489	405	984	691	708	246	117
ACC_02199	glutathione synthetase-like	K01920	KOG0021	149	166	162	172	224	16	24	36	262	188	305	479	506	555	39	9
ACC_02200	GMP reductase 2-like isoform 1	K00364	KOG2550	169	69	69	191	226	24	28	36	411	209	111	413	318	411	96	75
ACC_02201	conserved hypothetical protein	K14972	KOG2043	454	412	436	375	164	48	54	53	631	419	139	130	376	454	147	49
ACC_02202	fructose-bisphosphate aldolase-like	K01623	KOG1557	8445	4378	4876	12187	4854	417	487	688	14233	9096	1690	3665	20793	3725	871	330
ACC_02203	fructose-bisphosphate aldolase-like	K01623	KOG1557	12	10	11	18	15	0	2	1	30	32	7	5	26	7	2	3
ACC_02204	ring finger protein, transmembrane 2		KOG4638	155	162	125	287	296	15	13	28	269	241	89	325	317	318	51	7
ACC_02205	citrate synthase-like		KOG2617	56	14	18	24	7	1	2	1	18	9	2	6	6	4	2	0
ACC_02206	centaurin-gamma-1A	K12491	KOG0705	398	300	287	658	605	60	58	69	452	393	171	413	288	275	75	16
ACC_02207	CDP-diacylglycerol--inositol 3-phosphatidyltransfer	K00999	KOG3240	367	139	188	351	392	11	20	30	548	174	98	248	227	106	10	3
ACC_02208	hypothetical protein			546	330	371	236	212	47	140	123	246	210	90	73	168	159	265	104
ACC_02209	laminin subunit alpha-5-like	K05637	KOG1836	679	422	305	120	53	77	85	100	887	518	199	94	300	573	282	172
ACC_02210	conserved hypothetical protein		KOG3608	7	3	1	3	10	1	1	0	2	3	2	0	0	3	5	2
ACC_02211	conserved hypothetical protein			56	37	33	54	45	12	37	48	107	34	29	29	53	54	99	86
ACC_02212	coiled-coil domain-containing protein 56-like		KOG4782	185	84	72	147	160	5	18	25	88	120	58	311	280	188	68	39
ACC_02213	putative tyramine receptor		KOG4220	126	44	43	90	45	3	2	6	50	34	6	2	2	11	6	3
ACC_02214	peritrophin-1-like			14	5	6	32	14	1	1	2	3	5	2	4	42	47	92	0
ACC_02215	putative U5 small nuclear ribonucleoprotein 200 kD	K12854	KOG0951	1533	846	760	1209	1179	259	418	478	1659	1432	677	546	1086	1259	1265	933
ACC_02216	conserved hypothetical protein			12	11	12	13	8	3	2	6	85	44	60	14	22	92	8	1
ACC_02217	hypothetical protein			4	4	4	1	2	0	0	0	0	2	1	0	0	0	2	2
ACC_02218	Zinc finger protein 84		KOG3623	195	118	85	103	46	23	28	37	319	305	64	89	181	196	380	72
ACC_02219	protein DPCD-like			100	67	56	189	109	10	16	14	122	46	124	156	261	341	64	43
ACC_02220	methyltransferase-like protein 10-like		KOG1271	62	43	65	108	101	11	12	22	173	62	73	128	108	88	9	5

ACC_02221	protein SGT1 homolog ecdysoneless-like		KOG2406	703	478	554	614	661	62	122	175	518	361	262	352	472	534	150	76
ACC_02222	conserved hypothetical protein			397	252	271	421	214	28	29	40	207	112	111	209	26	49	31	4
ACC_02223	conserved hypothetical protein			28	18	11	20	20	3	4	6	21	4	7	0	2	0	3	6
ACC_02224	tubulin-specific chaperone C-like isoform 1		KOG2512	268	156	172	432	443	16	41	32	354	217	242	544	729	740	105	24
ACC_02225	conserved hypothetical protein			82	69	83	88	98	1	9	5	70	60	59	48	43	47	9	5
ACC_02226	conserved hypothetical protein	K16512		12	7	14	20	15	3	4	1	19	1	18	1	4	11	5	2
ACC_02227	fibrous sheath-interacting protein 2-like		KOG2072	38	19	26	39	28	8	11	15	35	10	7	1	35	40	149	125
ACC_02228	neutral and basic amino acid transport protein rBAT-like		KOG0471	599	365	352	481	354	31	43	26	1178	633	275	458	253	107	40	14
ACC_02229	LOW QUALITY PROTEIN		KOG4154	650	343	212	549	828	58	179	246	340	371	420	1130	1069	1909	414	295
ACC_02230	DNA repair endonuclease XPF-like	K10848	KOG0442	365	268	237	473	405	34	82	86	400	420	189	219	470	551	101	27
ACC_02231	conserved hypothetical protein	K06265	KOG3781	1289	494	518	377	152	57	85	89	1611	637	157	342	1266	1161	1303	456
ACC_02232	WD repeat-containing protein 65-like		KOG0161	87	63	70	67	71	12	24	12	290	218	50	22	274	237	45	7
ACC_02233	A disintegrin and metalloproteinase with thrombospondin m		KOG3538	39	14	30	53	38	5	4	8	71	45	33	5	19	26	15	5
ACC_02234	conserved hypothetical protein		KOG2504	28	28	18	44	13	5	13	12	86	182	9	1	290	197	150	43
ACC_02235	cyclin-K-like		KOG0834	280	315	324	442	234	36	54	82	399	212	162	226	272	390	134	52
ACC_02236	radial spoke head protein 4 homolog A-like			134	122	162	232	117	19	70	61	70	65	32	6	49	19	97	93
ACC_02237	two pore potassium channel protein sup-9-like		KOG4404	47	34	35	56	16	2	4	4	51	26	21	32	3	4	2	1
ACC_02238	beclin 1-associated autophagy-related key regulator-like		KOG4398	141	88	80	99	100	6	14	13	210	138	59	116	153	154	16	7
ACC_02239	protein FAM117B-like isoform 1			72	65	69	43	11	5	8	10	159	167	29	92	103	118	52	15
ACC_02240	tripartite motif-containing protein 2-like		KOG3627	10	2	0	2	3	0	0	4	4	2	6	5	28	57	15	4
ACC_02241	DNA polymerase iota-like	K03510	KOG2095	66	46	49	54	32	4	12	8	163	124	38	99	91	96	19	2
ACC_02242	nicotinic acetylcholine receptor alpha8 subunit	K05312	KOG3645	649	356	383	555	507	37	54	66	337	193	141	140	29	53	11	1
ACC_02243	cytochrome oxidase subunit I	K02256		1	0	0	1	0	0	0	0	0	0	1	0	0	1	0	0
ACC_02244	magnesium-dependent phosphatase 1-like		KOG4549	52	44	66	83	90	0	0	2	20	38	37	151	95	120	8	6
ACC_02245	serine/threonine-protein kinase SRPK3-like	K15409	KOG1290	566	268	207	574	378	122	232	258	608	519	247	239	516	644	859	528
ACC_02246	protein C9orf140-like		KOG4289	193	160	121	280	271	31	28	49	288	154	176	235	260	282	55	21
ACC_02247	conserved hypothetical protein			1333	688	531	810	1305	288	798	642	791	411	728	832	1202	1922	2641	1852
ACC_02248	conserved hypothetical protein		KOG3558	333	248	182	237	62	33	55	68	614	1001	98	184	170	173	76	33
ACC_02249	LOW QUALITY PROTEIN		KOG3640	337	218	168	448	210	21	31	40	156	184	59	83	182	312	157	59
ACC_02250	conserved hypothetical protein		KOG3415	89	60	48	158	167	12	24	38	149	103	117	157	511	510	61	39
ACC_02251	leucine-rich repeat-containing protein 16A-like isoform 2		KOG4242	448	407	365	271	204	34	69	77	813	816	182	339	611	400	70	18
ACC_02252	LOW QUALITY PROTEIN		KOG0531	401	308	297	381	480	24	76	79	384	213	283	557	369	409	67	31
ACC_02253	LOW QUALITY PROTEIN	K08040	KOG3898	12	15	16	44	37	1	2	4	19	2	1	10	4	5	1	1
ACC_02254	LOW QUALITY PROTEIN		KOG2648	224	143	177	242	320	17	29	36	564	251	212	315	418	398	40	11
ACC_02255	WD repeat-containing protein 13-like		KOG0266	94	55	69	125	129	9	22	18	110	107	68	183	271	364	54	20
ACC_02256	zinc finger protein 235-like		KOG2462	465	379	312	330	151	56	149	130	326	221	70	50	57	60	325	184
ACC_02257	hypothetical protein			1	0	0	0	1	1	1	1	1	0	1	0	0	0	1	0
ACC_02258	UPF0082 protein Pmob_0807-like		KOG2972	192	123	103	189	263	22	41	58	201	96	129	394	577	478	86	58
ACC_02259	conserved hypothetical protein		KOG1029	2523	2068	1812	1065	401	146	199	232	1352	548	38	36	24	74	92	47
ACC_02260	DEP domain-containing protein 5-like		KOG3572	1307	950	976	987	693	130	208	230	1175	791	532	208	615	626	139	47
ACC_02261	pyridoxine-5'-phosphate oxidase-like	K00275	KOG2586	53	38	42	98	83	9	16	20	84	43	70	141	166	195	21	5
ACC_02262	eukaryotic translation initiation factor 3 subunit K-l	K15028	KOG3252	240	258	207	334	286	25	47	55	338	197	219	688	931	1192	115	49
ACC_02263	tetratricopeptide repeat protein 35-B-like		KOG3060	371	210	204	429	429	38	54	56	555	376	260	668	796	962	55	32
ACC_02264	5-oxoprolinase-like	K01469	KOG1939	414	191	183	563	167	63	91	142	419	305	134	109	502	379	363	363
ACC_02265	conserved hypothetical protein		KOG0860	161	79	99	197	98	28	45	48	159	82	34	54	49	55	88	37
ACC_02266	hrp65 protein-like	K13219	KOG0115	2179	1901	1778	1478	806	229	468	466	3331	2078	620	803	2755	4687	2495	2237
ACC_02267	cysteine-rich hydrophobic domain 2 protein-like		KOG4101	142	86	76	76	110	14	23	26	202	134	46	104	51	50	9	4
ACC_02268	60S ribosomal protein L21	K02889	KOG1732	1283	1116	534	2054	1088	289	503	517	1055	600	1283	1477	4118	6581	1976	934
ACC_02269	selT-like protein-like isoform 1		KOG3286	334	157	168	555	408	34	44	77	471	280	294	697	780	758	44	21
ACC_02270	LOW QUALITY PROTEIN	K12589	KOG1612	263	196	196	468	446	37	66	70	289	149	291	259	154	223	32	9
ACC_02271	conserved hypothetical protein	K11137	KOG4346	406	213	155	394	372	35	91	93	485	330	213	359	703	621	154	37
ACC_02272	conserved hypothetical protein		KOG0161	361	194	185	261	259	41	122	91	683	765	200	574	690	452	112	39
ACC_02273	protein phosphatase 1L-like		KOG0698	1559	741	658	1441	1323	271	570	680	1422	854	671	882	921	703	492	264
ACC_02274	zinc finger protein 395-like			105	28	37	105	35	10	9	21	89	116	20	46	10	8	15	3
ACC_02275	lipoyltransferase 1, mitochondrial-like isoform 2	K10105	KOG3159	278	141	126	315	232	20	27	30	1126	740	140	345	874	535	132	32
ACC_02276	toll-like receptor 13-like isoform 1		KOG4194	598	404	379	446	250	30	22	40	3113	12280	196	532	980	291	39	37
ACC_02277	LIN1-like protein-like	K13099	KOG2950	228	168	188	238	202	46	82	108	231	122	125	156	242	303	281	239

ACC_02278	conserved hypothetical protein			227	202	227	317	238	17	48	40	251	174	159	299	199	213	84	45
ACC_02279	conserved hypothetical protein	K13348	KOG1944	74	70	51	199	200	10	18	22	294	118	87	276	85	78	12	1
ACC_02280	transcription factor E2F4-like		KOG2577	223	112	107	197	222	27	61	74	216	110	118	156	195	288	278	243
ACC_02281	neuferricin-like		KOG1108	267	199	202	171	187	25	44	58	137	92	206	224	161	153	19	8
ACC_02282	3-oxoacyl-	K09458	KOG1394	186	112	101	208	232	7	11	31	226	124	106	277	491	791	79	15
ACC_02283	calumenin-like		KOG4223	687	446	344	1034	999	82	113	185	573	766	252	712	2320	1999	304	241
ACC_02284	transmembrane protein 165-like		KOG2881	213	155	144	196	162	23	42	38	341	123	170	304	166	235	49	22
ACC_02285	venom carboxylesterase-6 precursor		KOG1516	1	2	2	0	0	0	0	0	1	37	0	0	6	4	5	6
ACC_02286	venom carboxylesterase-6-like		KOG1516	503	221	87	251	203	35	36	36	2288	4884	5201	25925	24151	4829	182	30
ACC_02287	UPF0454 protein C12orf49 homolog		KOG3136	88	47	34	99	70	9	21	28	93	87	75	141	116	118	58	30
ACC_02288	hypothetical protein			11	3	4	12	2	1	1	0	7	7	4	0	0	0	5	1
ACC_02289	glucose oxidase		KOG1238	37297	39844	78155	16854	5534	30768	31025	76492	68	35	12	13	3	2	14	16
ACC_02290	conserved hypothetical protein			41	23	17	53	25	6	3	6	31	41	4	10	3	3	4	12
ACC_02291	conserved hypothetical protein		KOG4441	69	73	65	119	72	12	13	13	55	31	10	0	5	6	16	9
ACC_02292	zinc finger protein 845-like		KOG3623	125	78	83	118	59	21	29	45	165	201	43	68	110	127	243	103
ACC_02293	conserved hypothetical protein		KOG0933	0	0	2	1	4	0	0	2	5	0	0	1	4	1	3	2
ACC_02294	succinate dehydrogenase	K00235	KOG3049	507	298	334	866	826	57	69	78	1525	939	416	1863	1196	1251	130	54
ACC_02295	conserved hypothetical protein		KOG1674	237	171	180	319	280	21	32	38	474	345	248	745	664	831	57	29
ACC_02296	splicing factor 3B subunit 1-like isoform 1	K12828	KOG0213	3211	2602	2335	2842	2177	315	460	502	3168	1975	921	587	1534	1899	658	380
ACC_02297	cytoplasmic tRNA 2-thiolation protein 1-like	K14168	KOG2840	229	119	122	333	342	19	42	45	279	121	141	192	354	666	89	32
ACC_02298	probable splicing factor 3B subunit 5-like	K12832	KOG3485	59	39	50	96	101	1	8	5	25	30	82	109	94	154	12	9
ACC_02299	conserved hypothetical protein			146	124	105	163	95	7	12	13	118	63	58	143	79	101	39	9
ACC_02300	estrogen sulfotransferase-like		KOG1584	22	27	17	34	36	2	5	4	136	19	1296	8139	180	689	51	64
ACC_02301	zinc finger protein 480-like		KOG2462	781	413	274	450	421	69	144	176	681	478	378	169	387	369	289	91
ACC_02302	DNA excision repair protein ERCC-6-like	K10841	KOG0387	733	402	418	500	622	41	54	104	498	358	337	216	306	291	83	32
ACC_02303	cytochrome P450 315a1, mitochondrial isoform 2	K10722	KOG0159	12	10	13	26	31	3	4	4	59	153	8	28	355	459	79	24
ACC_02304	serine/threonine-protein kinase ULK2	K08269	KOG0595	738	461	478	781	414	120	120	140	2264	1407	464	1315	1185	1173	389	52
ACC_02305	density-regulated protein-like		KOG3239	217	152	144	337	317	54	127	144	285	85	221	192	406	757	846	869
ACC_02306	conserved hypothetical protein		KOG4650	1020	374	349	590	454	13	24	21	395	265	89	265	56	45	11	5
ACC_02307	protein transport protein Sec61 subunit beta	K09481	KOG3457	364	279	124	378	366	55	97	143	258	250	183	903	1661	1515	247	75
ACC_02308	conserved hypothetical protein		KOG3553	80	49	44	80	99	4	10	5	93	53	26	146	94	94	20	3
ACC_02309	fatty-acid amide hydrolase 2-like isoform 1		KOG1212	635	457	326	576	500	84	142	106	642	479	262	646	513	566	57	16
ACC_02310	protein transport protein Sec31A	K14005	KOG0307	890	1034	789	659	453	123	169	208	1292	1330	318	693	2679	2287	341	180
ACC_02311	transmembrane protein 59-like			270	122	120	208	213	3	7	9	296	177	83	244	150	140	19	3
ACC_02312	conserved hypothetical protein			85	51	59	99	73	35	32	49	391	353	27	85	20	31	27	7
ACC_02313	putative odorant receptor 13a-like			22	15	13	31	25	1	0	3	8	10	19	7	0	0	1	0
ACC_02314	putative odorant receptor 13a			97	46	50	80	81	3	2	1	40	19	55	113	1	1	0	0
ACC_02315	putative odorant receptor 22c-like			7	2	6	15	21	0	1	1	9	14	48	30	0	0	0	0
ACC_02316	odorant receptor 27			9	4	6	18	10	0	1	2	21	22	6	36	0	1	0	1
ACC_02317	odorant receptor 26			9	6	8	12	15	2	1	5	18	14	30	55	0	0	0	0
ACC_02318	odorant receptor 25			12	9	12	18	17	0	1	1	9	12	85	76	0	0	0	0
ACC_02319	conserved hypothetical protein			5	4	12	6	5	1	2	0	8	8	39	29	0	0	0	0
ACC_02320	odorant receptor 43a-like			14	13	20	19	10	6	3	3	23	26	300	2826	0	0	1	1
ACC_02321	odorant receptor Or2-like			11	7	9	5	13	1	3	2	11	16	9	9	0	1	0	0
ACC_02322	putative odorant receptor 85b-like	K08471		33	2	10	3	5	1	1	0	21	17	43	13	0	0	0	0
ACC_02323	odorant receptor Or2-like			24	16	16	18	10	0	1	3	26	38	134	128	0	0	0	1
ACC_02324	odorant receptor 14			26	13	13	10	15	2	4	4	13	14	128	137	0	0	0	0
ACC_02325	odorant receptor 13			8	4	12	15	6	0	0	2	14	9	27	26	1	0	0	1
ACC_02326	odorant receptor 12			13	13	12	5	5	0	1	4	18	10	245	468	2	1	0	3
ACC_02327	odorant receptor 11			3	5	6	0	2	0	0	0	5	23	134	3943	1	0	0	0
ACC_02328	odorant receptor 10			1	1	1	5	1	0	0	0	5	4	36	300	4	0	0	0
ACC_02329	odorant receptor 9			2	0	1	2	0	0	0	1	1	6	94	128	1	0	0	0
ACC_02330	odorant receptor 9			5	2	1	9	1	0	1	3	4	13	35	73	1	6	1	0
ACC_02331	odorant receptor 5			2	6	4	3	1	0	0	0	1	5	65	17	1	3	3	0
ACC_02332	odorant receptor 5			3	2	6	12	7	0	2	3	7	18	116	54	0	2	1	1
ACC_02333	conserved hypothetical protein			3	13	13	10	8	0	2	1	15	70	103	41	11	10	4	0
ACC_02334	high affinity cAMP-specific and IBMX-insensitive 3',	K01120	KOG1229	7883	5279	5114	22745	14000	2532	6517	9721	8136	7028	941	839	1030	481	2585	688

ACC_02335	zeta-sarcoglycan isoform 2		KOG3950	64	43	59	66	17	6	3	6	18	8	10	0	3	3	16	12
ACC_02336	WD repeat and HMG-box DNA-binding protein 1-lik	K11274	KOG1274	256	276	232	247	243	36	79	68	284	212	111	142	521	511	283	139
ACC_02337	porphobilinogen deaminase-like	K01749	KOG2892	254	115	103	218	218	13	15	25	788	282	428	1312	434	631	108	33
ACC_02338	BRO1 domain-containing protein BROX-like		KOG2220	229	163	150	189	159	18	26	22	327	275	59	338	154	118	33	14
ACC_02339	TFIIH basal transcription factor complex helicase XF	K10844	KOG1131	558	353	325	707	616	38	78	74	681	320	326	223	398	429	95	25
ACC_02340	probable saccharopine dehydrogenase-like isoform	K01495	KOG2698	61	33	29	71	34	5	5	4	1697	978	65	486	51	65	15	5
ACC_02341	probable saccharopine dehydrogenase-like isoform 1		KOG2733	677	500	402	1240	1072	47	40	45	2093	1288	1097	2821	1083	913	52	15
ACC_02342	LOW QUALITY PROTEIN	K11367	KOG0384	3730	2343	2394	2971	2843	898	1664	1758	3361	1651	1718	642	1587	2216	3061	1815
ACC_02343	hsp90 co-chaperone Cdc37		KOG2260	2172	1191	1712	2255	2542	336	588	893	1061	548	1010	1380	1285	1626	1596	1761
ACC_02344	APOBEC1 complementation factor-like		KOG0117	1	0	0	4	2	0	0	0	0	1	0	0	8	3	1	0
ACC_02345	conserved hypothetical protein	K14031	KOG0604	521	366	314	615	436	69	108	116	1182	1000	241	1107	764	673	171	71
ACC_02346	Predicted ubiquitin-protein ligase/hyperplastic discs protein		KOG0943	44	22	17	42	18	15	16	36	66	29	9	4	9	36	88	43
ACC_02347	coiled-coil-helix-coiled-coil-helix domain-containing protein		KOG4695	50	32	51	56	79	2	2	0	17	24	63	92	142	204	26	16
ACC_02348	protein FAM40A-like		KOG3680	191	130	132	160	159	14	26	37	387	302	92	204	250	255	26	20
ACC_02349	conserved hypothetical protein			35	17	15	15	5	2	2	0	17	28	4	3	1	0	0	3
ACC_02350	conserved hypothetical protein			10	12	7	6	2	0	1	4	10	9	1	1	1	0	0	1
ACC_02351	conserved hypothetical protein			52	10	13	41	6	1	4	3	16	22	1	0	1	0	3	3
ACC_02352	hypothetical protein			43	10	12	15	7	3	3	5	10	34	0	0	0	2	3	5
ACC_02353	conserved hypothetical protein			310	205	125	251	259	69	195	194	364	270	279	222	334	364	358	213
ACC_02354	hypothetical protein			78	20	32	33	32	4	18	18	25	27	22	10	5	5	61	64
ACC_02355	SAGA-associated factor 29 homolog	K11364	KOG3038	119	94	114	310	83	23	28	41	107	64	113	134	54	120	358	280
ACC_02356	hypothetical protein			0	0	0	2	0	0	0	0	0	0	0	0	0	0	1	3
ACC_02357	U3 small nucleolar RNA-interacting protein 2-like	K14793	KOG0299	574	307	230	261	301	145	286	279	325	207	239	194	760	1561	1347	1918
ACC_02358	guanine deaminase-like	K01487	KOG3968	46	41	42	86	133	2	5	7	199	138	53	114	2151	1306	101	57
ACC_02359	transmembrane protein 8A-like			235	167	111	175	211	25	29	37	523	318	84	137	694	128	12	2
ACC_02360	transmembrane protein 39A-A-like		KOG3828	383	232	229	477	416	15	35	29	246	131	192	326	163	177	26	7
ACC_02361	nucleoside diphosphate-linked moiety X motif 18-like		KOG3084	339	233	259	337	353	10	17	21	288	147	123	355	177	203	12	4
ACC_02362	transcription initiation factor IIA subunit 2	K03123	KOG3463	71	49	31	123	145	8	12	21	67	61	61	118	134	149	14	10
ACC_02363	hypothetical protein			0	0	0	0	0	0	0	0	0	0	0	0	0	0	0	0
ACC_02364	conserved hypothetical protein			23	14	22	71	28	7	7	8	80	62	17	39	134	55	62	7
ACC_02365	charged multivesicular body protein 6-like	K12195	KOG2910	151	155	166	263	219	34	79	86	245	120	207	308	233	299	232	147
ACC_02366	hypothetical protein			7	6	13	3	3	1	0	0	0	7	1	0	0	0	1	1
ACC_02367	hypothetical protein			18	17	6	13	6	3	5	6	8	2	1	1	0	0	4	4
ACC_02368	hypothetical protein			0	0	0	0	1	0	0	0	0	0	0	0	0	0	1	0
ACC_02369	t-box transcription factor TBX5-like		KOG3585	8	7	9	9	10	1	0	1	6	13	5	10	9	12	15	5
ACC_02370	CDK5 and ABL1 enzyme substrate 1-like isoform 1		KOG4164	316	237	233	323	239	31	54	74	610	689	83	256	228	242	82	30
ACC_02371	e3 ubiquitin-protein ligase UBR2-like	K10626	KOG1140	601	291	327	537	462	63	45	53	1023	1082	166	440	503	457	54	10
ACC_02372	myotubularin-related protein 13 isoform 1		KOG1090	5356	2745	2557	4865	3852	386	513	630	4007	2482	1269	2057	2423	2320	465	256
ACC_02373	LOW QUALITY PROTEIN		KOG1130	474	338	302	425	297	58	69	94	1103	877	328	418	751	956	238	93
ACC_02374	tricarboxylate transport protein, mitochondrial-like	K15100	KOG0756	149	65	59	145	140	4	11	11	968	645	149	953	1724	1228	99	18
ACC_02375	SWI/SNF-related matrix-associated actin-depender	K11648	KOG1649	289	166	182	496	368	25	38	62	377	256	272	580	591	668	126	75
ACC_02376	polycomb protein eed-A-like	K11462	KOG1034	61	47	48	96	85	2	7	5	117	50	30	103	126	174	45	6
ACC_02377	conserved hypothetical protein			33	25	18	31	29	2	4	9	73	49	11	60	69	91	12	2
ACC_02378	conserved hypothetical protein	K11651	KOG4715	765	715	957	812	294	109	156	166	924	540	253	280	934	1259	1220	712
ACC_02379	vacuolar protein-sorting-associated protein 36-like	K12190	KOG2760	195	138	128	218	205	26	33	63	151	100	119	303	224	213	25	30
ACC_02380	conserved hypothetical protein		KOG1028	157	104	137	73	25	5	4	5	120	86	41	29	2	6	3	0
ACC_02381	hypothetical protein			9	5	10	13	11	0	3	0	1	1	4	1	0	0	1	0
ACC_02382	hypothetical protein			1	0	0	1	0	0	3	2	15	1	4	1	2	30	9	6
ACC_02383	caseinolytic peptidase B protein homolog	K03695	KOG1051	330	263	299	512	496	43	79	92	557	376	285	409	745	681	121	45
ACC_02384	conserved hypothetical protein			2	3	4	7	3	0	1	0	1	0	0	1	2	4	1	0
ACC_02385	conserved hypothetical protein		KOG2710	70	14	44	100	18	11	16	17	517	329	193	79	27	46	110	66
ACC_02386	hypothetical protein			9	1	4	9	4	4	4	6	27	11	41	29	1	1	4	10
ACC_02387	peroxisomal membrane protein 11B-like isoform 2	K13352	KOG4186	231	130	152	304	302	13	20	25	256	145	153	469	211	233	25	6
ACC_02388	THO complex subunit 4-like	K12881	KOG0533	215	129	124	524	225	31	37	57	155	182	118	213	463	937	590	266
ACC_02389	protein canopy-1-like		KOG3782	80	105	55	114	184	7	19	8	59	68	47	308	383	480	15	15
ACC_02390	targeting protein for Xklp2-A-like			3	2	3	4	7	0	0	2	5	3	111	43	4	9	7	7
ACC_02391	conserved hypothetical protein			1	1	0	6	0	0	0	1	3	1	2	2	2	0	2	3

ACC_02392	protein trapped in endoderm-1-like		KOG4219	10	12	3	11	2	1	1	0	9	1	0	3	35	76	51	18
ACC_02393	laminin subunit beta-1-like		KOG3644	137	38	47	101	52	2	1	4	48	26	0	1	0	0	0	0
ACC_02394	conserved hypothetical protein		KOG2109	1563	817	888	1320	564	245	357	465	2471	1289	361	268	187	147	365	250
ACC_02395	conserved hypothetical protein	K02883	KOG1714	815	669	354	1774	867	137	173	145	705	694	966	1422	4187	5315	1637	285
ACC_02396	ankyrin repeat domain-containing protein 54-like		KOG0509	57	28	34	37	28	3	1	4	135	145	21	122	121	125	11	5
ACC_02397	39S ribosomal protein L13, mitochondrial	K02871	KOG3203	231	189	186	339	294	27	50	59	172	121	165	613	689	782	110	48
ACC_02398	GPN-loop GTPase 2-like	K06883	KOG1533	156	99	101	273	362	12	29	29	586	326	333	956	592	758	41	18
ACC_02399	proteasome assembly chaperone, putative		KOG4828	75	59	72	72	127	3	17	9	251	154	95	819	309	259	16	2
ACC_02400	zinc finger RAD18 domain-containing protein C1orf124 homo		KOG3931	274	151	143	265	318	12	28	34	299	219	206	268	244	228	46	11
ACC_02401	DNA-binding protein RFXANK-like		KOG0502	138	66	60	158	120	21	32	47	179	98	121	231	196	220	95	35
ACC_02402	transient receptor potential channel pyrexia		KOG0510	629	459	446	755	667	95	134	141	705	543	307	643	987	1317	374	229
ACC_02403	serine/threonine-protein kinase VRK1-like	K08816	KOG1164	561	437	438	440	397	25	35	44	491	385	127	369	367	392	55	17
ACC_02404	lachesin-like		KOG3513	23	17	11	24	11	3	5	4	5	8	0	1	1	0	13	8
ACC_02405	28S ribosomal protein S21, mitochondrial-like	K02970		73	58	53	85	100	7	4	5	83	34	58	238	248	206	12	5
ACC_02406	acyl-protein thioesterase 1-like	K06130	KOG2112	305	154	172	428	341	20	13	37	550	296	143	403	394	393	44	17
ACC_02407	transmembrane protein 194A-like		KOG3817	81	52	42	40	55	6	6	9	45	25	29	14	91	142	19	7
ACC_02408	aldehyde dehydrogenase, mitochondrial isoform 1	K00128	KOG2450	839	815	536	1875	1223	178	203	193	3481	3012	2327	3439	11922	5555	359	70
ACC_02409	6-phosphofructokinase	K00850	KOG2440	1485	744	738	772	936	94	158	139	3848	1912	477	1302	2642	1377	95	53
ACC_02410	14-3-3 protein zeta-like	K06630	KOG0841	4908	2469	2395	3813	1239	494	532	755	5127	4038	995	2653	3298	3753	2932	877
ACC_02411	UPF0587 protein v1g245604-like		KOG1296	87	50	38	137	169	17	42	37	105	57	87	136	306	286	53	52
ACC_02412	conserved hypothetical protein		KOG1883	535	453	388	47	14	151	213	339	349	518	6	15	378	286	744	647
ACC_02413	hemocyanin subunit 3			0	0	0	0	0	0	0	0	0	0	0	0	0	0	0	0
ACC_02414	conserved hypothetical protein		KOG0970	338	125	169	467	135	32	34	46	409	287	58	167	42	107	147	100
ACC_02415	conserved hypothetical protein			172	135	155	371	371	13	24	31	355	197	172	375	181	233	16	9
ACC_02416	DNL-type zinc finger protein-like		KOG3277	49	58	58	81	83	13	14	26	68	58	74	103	145	198	26	20
ACC_02417	transmembrane emp24 domain-containing protein eca-like		KOG1690	320	273	162	551	480	69	130	205	353	194	411	453	773	851	143	84
ACC_02418	TPPP family protein CG4893-like		KOG4070	599	206	218	323	107	22	32	32	886	820	105	1240	170	146	116	14
ACC_02419	conserved hypothetical protein			26	26	30	75	39	5	8	3	19	19	34	5	4	2	2	1
ACC_02420	conserved hypothetical protein			95	112	86	129	95	14	31	30	74	88	47	29	6	1	12	0
ACC_02421	glycerol-3-phosphate acyltransferase 4-like isoform	K13506	KOG2898	531	242	244	451	360	47	59	63	777	527	300	425	972	869	95	35
ACC_02422	conserved hypothetical protein			0	0	0	0	0	0	0	0	1	0	0	0	12	6	1	2
ACC_02423	DNA mismatch repair protein Mlh1	K08734	KOG1979	516	289	311	511	566	41	97	164	200	165	88	164	304	386	242	73
ACC_02424	nimrod C2		KOG1225	1392	533	657	1941	537	141	182	299	964	1255	53	270	1	5	43	21
ACC_02425	fibrillin-1		KOG1214	159	50	98	444	128	50	33	43	1815	3508	31	118	192	75	79	94
ACC_02426	LOW QUALITY PROTEIN	K10408	KOG3595	353	240	250	139	144	42	78	80	809	437	254	67	216	213	118	48
ACC_02427	WD repeat domain phosphoinositide-interacting protein 2-li		KOG2110	392	185	139	523	348	73	72	141	573	512	168	355	355	248	171	81
ACC_02428	protein FAM188A homolog		KOG2871	901	409	388	781	761	137	204	266	1113	785	383	743	1160	1296	461	248
ACC_02429	bifunctional ATP-dependent dihydroxyacetone kina	K00863	KOG2426	569	554	199	648	424	111	164	109	10466	6382	65	383	5803	2336	175	42
ACC_02430	extracellular sulfatase SULF-1 homolog	K14607	KOG3731	598	151	149	200	57	30	22	31	596	207	58	220	75	180	189	78
ACC_02431	UDP-glucuronic acid decarboxylase 1-like	K08678	KOG1429	149	128	96	150	146	27	59	61	456	326	112	255	316	274	102	44
ACC_02432	sodium/potassium-transporting ATPase subunit be	K01540	KOG3927	8670	2756	3169	5394	1219	198	299	580	1691	1160	608	544	97	53	47	23
ACC_02433	e3 ubiquitin-protein ligase UBR4-like	K10691	KOG1776	3250	2140	2068	2745	1675	425	595	754	5187	4201	903	918	2224	2617	682	264
ACC_02434	conserved hypothetical protein		KOG3017	228	141	146	295	252	22	19	33	168	159	40	110	73	61	35	13
ACC_02435	39S ribosomal protein L46, mitochondrial		KOG4548	684	776	842	961	949	96	282	304	747	368	412	1022	1133	1739	516	301
ACC_02436	hypothetical protein			6	2	3	3	0	0	0	0	4	16	0	0	0	0	0	0
ACC_02437	similar to conserved hypothetical protein	K14530	KOG1947	797	444	448	517	709	17	32	54	978	845	495	1003	1111	988	59	15
ACC_02438	eukaryotic translation initiation factor 4E	K03259	KOG1670	392	302	233	486	381	80	117	159	629	388	205	550	1385	1898	243	145
ACC_02439	protein odr-4 homolog		KOG4703	766	396	479	554	618	47	118	139	1102	472	562	814	859	790	280	146
ACC_02440	hypothetical protein			50	22	29	42	15	4	10	12	142	65	26	84	54	29	35	25
ACC_02441	bifunctional heparan sulfate N-deacetylase/N-sulfo	K02577	KOG3703	397	227	212	200	52	25	25	39	581	1121	67	127	82	85	54	7
ACC_02442	signal peptidase complex subunit 3	K12948	KOG3372	169	187	105	195	204	11	17	22	99	82	149	348	388	393	38	9
ACC_02443	ankyrin repeat and protein kinase domain-containing protei		KOG0107	167	122	96	210	165	21	32	33	268	199	100	182	263	265	73	62
ACC_02444	lysocardiolipin acyltransferase 1-like	K13513	KOG1505	698	304	253	530	515	37	85	84	786	423	117	212	315	296	103	70
ACC_02445	phospholipase DDHD1-like	K13619	KOG2308	156	78	64	79	85	5	11	11	222	216	41	146	105	85	9	3
ACC_02446	hypothetical protein			2	1	2	4	4	1	1	0	4	3	3	0	0	0	2	1
ACC_02447	conserved hypothetical protein			47	26	26	24	6	3	0	1	308	286	34	65	56	37	29	13
ACC_02448	Sip1/TFIP11 interacting protein	K13103	KOG2184	266	159	144	276	197	47	110	119	400	239	160	187	452	498	315	225

ACC_02449	glucose dehydrogenase		KOG1238	8	0	1	2	4	1	1	1	17	14	12	2	16	293	75	24
ACC_02450	glucose dehydrogenase		KOG1238	27	10	23	27	14	1	3	8	27	15	2	2	116	112	74	54
ACC_02451	glucose dehydrogenase		KOG1238	32	27	45	97	21	43	56	83	732	253	10	6	12	9	12	11
ACC_02452	glucose dehydrogenase		KOG1238	19	10	20	53	17	6	2	3	92	17	5	23	28	90	89	31
ACC_02453	glucose dehydrogenase		KOG1238	30	23	33	45	12	2	1	0	23	18	21	7	3	141	54	49
ACC_02454	glucose dehydrogenase		KOG1238	8	5	9	8	1	2	3	1	3	7	6	3	3	34	13	4
ACC_02455	coiled-coil domain-containing protein 93		KOG2701	233	163	149	309	304	48	85	77	331	203	135	225	224	297	156	84
ACC_02456	conserved hypothetical protein			1161	777	703	1109	1264	107	299	341	535	308	384	389	584	627	418	190
ACC_02457	putative RNA-binding protein EEED8.10-like		KOG4341	152	103	78	70	118	10	4	5	64	45	35	91	60	68	6	4
ACC_02458	venom acid phosphatase Acph-1-like		KOG3720	21	21	10	32	21	3	2	2	12	18	3	17	28	27	9	2
ACC_02459	DNA polymerase theta-like	K02349	KOG0950	203	150	116	209	200	13	35	40	128	124	68	69	204	215	70	17
ACC_02460	dynein light chain Tctex-type 1	K10420	KOG4081	149	117	107	148	221	22	33	38	154	66	115	285	204	178	15	7
ACC_02461	u11/U12 small nuclear ribonucleoprotein 25 kDa protein-like			100	59	114	210	170	25	40	43	82	50	135	169	67	99	75	46
ACC_02462	conserved hypothetical protein	K16731	KOG0992	1421	951	521	1103	887	438	992	1121	969	570	844	689	1035	889	1845	1437
ACC_02463	LIM/homeobox protein Lhx3-like	K09374	KOG4577	44	17	19	31	7	0	0	3	15	19	0	1	9	22	38	7
ACC_02464	egl nine homolog 1-like	K09592	KOG3710	194	189	144	250	103	65	76	82	605	343	56	290	257	149	76	29
ACC_02465	conserved hypothetical protein		KOG0922	332	228	197	303	455	63	155	181	154	79	117	113	99	107	109	93
ACC_02466	c-1-tetrahydrofolate synthase, cytoplasmic-like	K00288	KOG4230	412	290	206	516	406	136	207	202	2254	12615	162	297	2304	2047	536	429
ACC_02467	hypothetical protein			0	0	0	0	0	0	0	0	0	0	0	0	0	0	0	0
ACC_02468	hypothetical protein			0	0	0	0	0	0	0	0	0	0	0	0	0	0	0	0
ACC_02469	hypothetical protein			7	8	6	6	2	0	0	0	2	5	0	0	0	0	0	1
ACC_02470	hypothetical protein			0	0	1	3	1	0	0	0	0	0	0	0	0	0	0	0
ACC_02471	hypothetical protein			4	2	0	3	4	0	3	2	1	6	0	0	0	0	1	1
ACC_02472	fumarylacetoacetase-like	K01555	KOG2843	106	81	67	178	122	16	17	21	414	560	206	918	1646	1399	393	143
ACC_02473	UBX domain-containing protein 4-like isoform 1		KOG2507	1275	705	596	1257	818	241	331	414	1545	1061	790	2209	1540	1377	842	524
ACC_02474	conserved hypothetical protein	K13169	KOG2891	1239	689	759	1294	1158	230	521	492	756	326	590	170	336	554	941	583
ACC_02475	something about silencing protein 10-like	K15730	KOG3158	3548	1871	2033	3380	2041	643	1111	1392	2481	1460	1479	3884	5957	7678	5325	5982
ACC_02476	peroxisome biogenesis factor 1-like	K13338	KOG0735	136	79	73	135	70	18	19	30	156	239	39	80	256	178	50	64
ACC_02477	run domain Beclin-1 interacting and cystein-rich containing p		KOG1829	257	177	159	288	274	23	51	54	238	156	107	87	120	113	84	19
ACC_02478	transcription elongation factor S-II-like isoform 1	K03145	KOG1105	462	218	172	693	543	95	160	201	348	179	259	393	392	586	317	248
ACC_02479	crossover junction endonuclease MUS81-like	K08991	KOG2379	348	173	223	228	281	16	48	41	154	155	116	217	242	297	52	20
ACC_02480	Kinesin-like protein unc-104	K10392	KOG0245	9116	4460	4330	7244	3768	544	780	1066	5582	3472	1395	1185	190	184	143	38
ACC_02481	conserved hypothetical protein		KOG2991	462	370	330	399	315	56	87	119	538	300	201	250	477	561	183	99
ACC_02482	conserved hypothetical protein			56	68	77	56	43	7	12	13	72	59	18	40	37	47	12	4
ACC_02483	trypsin-1-like		KOG3627	4	0	3	5	0	0	0	0	4	7	1	1	54	27	16	5
ACC_02484	conserved hypothetical protein		KOG4239	86	24	33	83	34	3	1	5	234	90	19	149	53	51	51	15
ACC_02485	fas apoptotic inhibitory molecule 1-like		KOG4352	7	5	3	4	5	2	5	2	32	22	671	522	12	34	6	9
ACC_02486	sodium- and chloride-dependent GABA transporter	K05034	KOG3660	281	184	170	172	77	21	18	25	270	393	8	4	0	16	6	2
ACC_02487	homeobox protein MSX-1-like	K09360	KOG0485	3	1	4	3	3	0	3	0	10	6	7	156	11	19	10	5
ACC_02488	MAGUK p55 subfamily member 7		KOG0609	1082	608	565	1211	411	71	82	112	476	196	173	145	72	94	40	20
ACC_02489	hypothetical protein			6	7	6	14	6	1	3	2	5	17	1	3	0	0	0	2
ACC_02490	hypothetical protein			16	9	17	46	15	8	3	9	5	32	11	1	0	2	7	7
ACC_02491	conserved hypothetical protein		KOG4194	13	10	2	2	3	1	4	0	16	19	2	1	0	2	8	3
ACC_02492	hypothetical protein			3	3	0	2	1	0	0	1	4	1	1	0	0	0	2	1
ACC_02493	conserved hypothetical protein		KOG0161	793	398	381	553	808	51	218	216	751	608	484	423	682	599	262	134
ACC_02494	soluble guanylyl cyclase alpha 1 subunit	K12318	KOG4171	455	211	267	929	416	32	26	54	191	100	60	32	0	7	8	0
ACC_02495	Putative adenylate kinase 7	K00939	KOG4109	20	20	18	39	17	9	8	14	42	22	2	1	1	1	4	7
ACC_02496	vacuolar H+ ATP synthase 16 kDa proteolipid subur	K02155	KOG0232	2108	633	561	1177	554	60	66	95	1703	1336	594	2469	2515	2212	821	102
ACC_02497	ankyrin repeat domain-containing protein 16-like		KOG0509	56	29	28	93	62	3	12	12	45	20	26	20	30	30	35	35
ACC_02498	conserved hypothetical protein	K08836	KOG0579	767	521	550	473	247	204	367	371	986	856	250	237	323	298	400	222
ACC_02499	COMM domain-containing protein 10-like			109	100	91	101	128	12	25	11	115	136	89	178	151	185	28	10
ACC_02500	CWF19-like protein 1-like isoform 1		KOG2476	121	84	109	143	183	10	20	23	181	250	82	228	289	258	25	12
ACC_02501	long-wavelength sensitive opsin	K04255	KOG4219	968	2844	4539	14302	7792	7478	8095	13768	41725	41855	2	243	11	8	3	2
ACC_02502	transmembrane protein C5orf28-like			56	40	29	89	111	11	12	11	179	96	169	420	239	201	27	4
ACC_02503	prefoldin subunit 3-like		KOG3313	429	311	232	531	405	58	107	124	606	262	311	807	1895	2130	376	326
ACC_02504	conserved hypothetical protein	K13108	KOG1882	339	319	369	406	340	47	85	119	303	161	121	219	289	519	210	279
ACC_02505	epsilon-sarcoglycan-like		KOG4482	439	208	237	460	217	25	44	59	341	201	81	107	38	36	13	7

ACC_02506	upstream activation factor subunit spp27-like	K15223	KOG1946	3669	2496	2035	2499	1668	778	1328	1314	5889	2252	2273	3554	4154	7742	16547	18179
ACC_02507	protein enhancer of sevenless 2B-like	K04364	KOG3601	633	270	266	337	343	39	65	50	1117	857	206	1579	723	733	136	18
ACC_02508	conserved hypothetical protein		KOG3608	578	416	376	550	302	117	141	194	801	636	220	226	375	373	233	74
ACC_02509	ubiquitin-60S ribosomal protein L40 isoform 3	K02927	KOG0003	1157	922	526	1337	754	369	635	1048	1132	837	1260	1336	5726	7438	2337	1317
ACC_02510	conserved hypothetical protein			52	43	51	104	44	2	4	6	74	28	6	8	8	15	5	6
ACC_02511	conserved hypothetical protein		KOG0566	1815	707	648	1490	526	311	673	664	1990	951	219	457	811	1370	5343	5913
ACC_02512	radial spoke head protein 9 homolog			32	47	56	56	59	3	4	2	3	14	25	10	4	6	4	4
ACC_02513	conserved hypothetical protein			31	37	22	27	22	2	7	13	48	28	47	37	36	25	14	8
ACC_02514	aconitate hydratase, mitochondrial-like	K01681	KOG0453	1955	501	611	1792	818	113	124	154	7584	2424	683	2288	1582	2811	1727	279
ACC_02515	Myosin-XV	K10361	KOG4229	1315	1117	699	577	492	354	523	561	2356	1690	494	1162	419	662	494	251
ACC_02516	conserved hypothetical protein			135	70	82	388	359	14	11	23	54	12	29	44	9	29	8	4
ACC_02517	LOW QUALITY PROTEIN		KOG2083	346	232	257	512	445	45	56	50	500	182	159	250	221	241	32	12
ACC_02518	ubiquitin-like protein 7-like		KOG0010	336	225	218	286	238	21	26	35	300	252	140	679	585	567	65	7
ACC_02519	phosphofurin acidic cluster sorting protein 2-like isoform 2		KOG3709	1584	909	874	1254	382	411	921	915	1406	740	603	297	77	109	963	389
ACC_02520	protein FRA10AC1 homolog	K13121	KOG1297	183	117	126	141	151	32	58	86	146	108	95	186	78	116	27	21
ACC_02521	cytoglobin-1-like			504	212	257	830	541	57	121	143	76	53	15	12	0	5	15	0
ACC_02522	synaptic vesicle glycoprotein 2C-like		KOG0255	0	1	0	3	3	0	1	0	30	64	1	0	220	39	7	0
ACC_02523	protein ERGIC-53-like isoform 1	K10080	KOG3838	1278	1475	756	894	698	537	644	725	1892	1642	528	1801	5309	3691	701	308
ACC_02524	hypothetical protein			20	151	122	19	3	11	19	32	42	191	8	1	5	10	38	16
ACC_02525	Phosphatidylinositol-5-phosphate 4-kinase type-2 a	K00920	KOG0229	266	116	93	169	173	19	30	43	655	516	80	426	364	200	35	17
ACC_02526	LOW QUALITY PROTEIN	K15456	KOG3062	145	119	111	212	213	9	29	42	231	111	150	233	424	526	84	37
ACC_02527	methionine aminopeptidase 1D, mitochondrial-like	K01265	KOG2738	162	104	130	247	340	11	37	38	437	205	189	373	225	257	14	9
ACC_02528	enhancer of polycomb homolog 1	K11322	KOG2261	96	73	63	71	50	13	55	53	184	297	28	67	138	119	137	97
ACC_02529	RING finger protein 113A-like	K13127	KOG1813	362	254	242	401	277	75	144	155	443	154	256	265	252	438	309	287
ACC_02530	putative ammonium transporter 3-like		KOG0682	49	23	34	63	61	7	8	15	96	16	25	25	21	21	5	2
ACC_02531	60S ribosomal protein L22 isoform 1	K02891	KOG3434	1278	839	490	1599	721	288	803	589	1011	462	2193	1012	6003	5910	2106	1143
ACC_02532	Splicing factor, arginine/serine-rich 2	K12891	KOG4207	210	159	148	327	172	24	26	27	442	311	167	285	370	625	395	93
ACC_02533	beta,beta-carotene 9',10'-oxygenase isoform 1		KOG1285	7	49	45	326	251	141	160	275	1765	1741	34	107	3	1	1	0
ACC_02534	conserved hypothetical protein	K16830		923	517	553	782	822	142	397	408	435	239	416	529	976	1703	2373	1212
ACC_02535	probable cation-transporting ATPase 13A3	K14951	KOG0208	841	403	343	807	220	45	34	54	1283	1012	115	343	478	386	519	67
ACC_02536	cytosolic Fe-S cluster assembly factor NUBP2 homolog		KOG3022	121	162	143	228	248	66	58	99	497	226	151	456	339	362	41	5
ACC_02537	conserved hypothetical protein		KOG4246	25	6	6	6	2	0	0	1	46	21	1	4	1	2	10	5
ACC_02538	ATP-dependent DNA helicase Q5-like	K10902	KOG0352	818	591	586	794	948	39	117	102	540	448	407	558	788	770	348	134
ACC_02539	116 kDa U5 small nuclear ribonucleoprotein compo	K12852	KOG0468	216	158	154	347	133	27	34	43	245	229	137	170	459	1037	320	202
ACC_02540	39S ribosomal protein L16, mitochondrial-like	K02878	KOG3422	286	221	251	498	370	62	107	144	335	131	324	361	449	549	218	168
ACC_02541	gamma-aminobutyric acid receptor-associated pro	K08341	KOG1654	2150	1421	1286	3923	2369	474	652	797	4145	1514	1253	4497	1633	1357	594	230
ACC_02542	isochorismatase domain-containing protein 1-like		KOG4044	108	124	150	280	236	27	22	41	506	253	288	996	815	733	96	46
ACC_02543	dihydrofolate reductase	K00287	KOG1324	271	116	152	219	241	14	62	40	138	73	201	270	520	777	260	151
ACC_02544	putative gamma-glutamylcyclotransferase CG2811-like		KOG4450	378	238	165	531	499	33	47	76	471	412	201	794	1200	1268	164	49
ACC_02545	MIP18 family protein CG7949-like		KOG3381	151	143	143	298	264	27	43	41	150	77	132	255	193	268	47	48
ACC_02546	conserved hypothetical protein			299	110	134	697	242	105	113	101	1258	551	651	613	384	292	379	81
ACC_02547	LOW QUALITY PROTEIN		KOG3629	740	264	319	606	171	39	67	82	562	442	151	163	36	29	95	33
ACC_02548	hypothetical protein			5	1	2	3	3	1	0	0	3	3	0	3	0	0	0	0
ACC_02549	dynein heavy chain 7, axonemal-like		KOG3595	70	65	66	151	68	9	4	9	38	23	4	1	0	5	3	2
ACC_02550	conserved hypothetical protein			36	29	38	96	49	4	5	7	24	14	10	18	18	31	8	7
ACC_02551	proteasome subunit beta type-7-like	K02739	KOG0173	376	228	225	775	598	72	91	105	1709	913	843	2140	2650	3742	327	97
ACC_02552	cell division control protein 6 homolog	K02213	KOG2227	78	62	62	87	81	2	6	12	64	71	19	53	370	616	130	43
ACC_02553	conserved hypothetical protein			492	265	254	396	291	19	27	31	2258	1265	252	876	443	260	57	16
ACC_02554	neuroguidin-A-like	K14765	KOG3117	243	152	148	168	156	43	109	103	514	429	117	136	444	411	292	203
ACC_02555	conserved hypothetical protein	K13943	KOG2462	264	209	285	517	300	53	71	100	765	861	148	386	351	364	193	54
ACC_02556	Phosphorylase b kinase gamma catalytic chain, ske	K09115	KOG2483	87	94	169	146	46	29	39	45	268	546	66	94	77	112	201	116
ACC_02557	conserved hypothetical protein	K00871	KOG0599	150	97	80	178	162	34	47	46	662	316	168	452	247	211	24	14
ACC_02558	proton-coupled amino acid transporter 4-like	K14209	KOG1304	1015	544	413	791	315	299	219	307	3053	2021	230	254	508	593	323	218
ACC_02559	protein SLC7A6OS-like		KOG4852	93	83	81	176	179	7	21	17	148	107	146	217	287	457	55	28
ACC_02560	protein kinase C and casein kinase substrate in neurons prot		KOG2856	884	439	463	596	348	84	86	116	1530	836	332	723	276	329	144	49
ACC_02561	breast cancer anti-estrogen resistance protein 3-like		KOG4792	582	363	585	977	216	16	22	30	500	319	113	262	75	99	211	45
ACC_02562	conserved hypothetical protein		KOG0921	6754	4132	5731	3281	3416	803	1880	1700	2820	1825	982	1059	1881	2212	249	113

ACC_02563	hypothetical protein			1	0	0	3	0	1	1	0	1	1	0	0	0	0	0	0
ACC_02564	mitochondrial fission 1 protein-like		KOG3364	228	199	165	530	552	67	101	150	227	120	397	574	238	220	81	104
ACC_02565	endocuticle structural glycoprotein SgAbd-1-like			10	3	12	19	2	1	1	0	18	7	9	97	164	209	215	214
ACC_02566	circadian clock-controlled protein-like isoform 1			327	43	19	241	52	89	67	114	3858	1359	18534	44655	357	105	14	51
ACC_02567	TM2 domain-containing protein almondex		KOG4272	71	48	56	173	160	9	19	30	139	47	69	105	69	97	6	4
ACC_02568	eukaryotic translation initiation factor 6 isoform 1	K03264	KOG3185	145	86	76	394	227	14	21	43	240	120	194	411	641	997	183	57
ACC_02569	STIP1 homology and U box-containing protein 1-lik	K09561	KOG4642	511	289	296	502	437	52	122	145	523	226	153	348	392	402	253	148
ACC_02570	transcription factor Dp-1-like	K09394	KOG2829	255	193	145	354	234	59	85	93	494	335	203	370	840	1374	1171	570
ACC_02571	conserved hypothetical protein		KOG2571	135	247	251	286	294	23	50	34	159	227	70	46	942	1020	70	2
ACC_02572	serine protease snake		KOG3627	11	24	24	40	30	11	12	24	221	155	7	13	14	31	15	3
ACC_02573	protein phosphatase 1 regulatory subunit 16A-like		KOG0505	518	368	397	446	289	27	42	48	643	472	118	279	245	175	49	18
ACC_02574	bromodomain adjacent to zinc finger domain protein 2B-like		KOG1245	2421	2169	2429	2150	1008	289	477	551	2483	1306	676	161	192	437	1412	765
ACC_02575	conserved hypothetical protein			224	80	63	201	137	14	18	26	696	427	959	4615	860	1147	78	34
ACC_02576	rho-associated protein kinase 2-like	K04514	KOG0612	880	594	536	638	365	89	191	208	880	671	175	187	428	348	363	151
ACC_02577	protein O-mannosyltransferase 1-like	K00728	KOG3359	254	278	261	358	373	29	28	42	681	273	264	156	234	283	30	6
ACC_02578	UPF0585 protein CG18661-like			66	59	43	85	93	4	5	11	95	34	92	201	297	489	22	23
ACC_02579	dnaJ homolog subfamily C member 21-like		KOG0717	1023	627	549	592	901	97	352	388	560	458	531	585	430	738	620	279
ACC_02580	RING finger and CHY zinc finger domain-containing	K10144	KOG1940	154	61	71	101	81	5	5	11	295	286	27	110	153	163	25	3
ACC_02581	tafazzin homolog isoform 2	K13511	KOG2847	477	249	328	444	306	95	211	214	630	280	234	504	404	371	451	182
ACC_02582	major facilitator superfamily domain-containing protein 1-lik		KOG4686	188	90	89	247	208	16	23	16	331	203	77	249	221	119	24	3
ACC_02583	chromatin complexes subunit BAP18-like isoform 3		KOG4834	72	71	74	220	88	9	13	25	63	27	116	111	91	179	163	148
ACC_02584	probable methyltransferase BCDIN3D-like isoform 1		KOG2899	15	20	7	14	11	1	2	1	23	9	12	15	22	11	5	1
ACC_02585	cleavage and polyadenylation specificity factor sub	K14397	KOG1689	301	235	217	591	372	28	48	60	281	105	164	206	260	405	199	98
ACC_02586	condensin complex subunit 2-like		KOG2328	458	360	332	476	613	74	240	212	307	202	236	164	400	566	384	164
ACC_02587	lipoyl synthase, mitochondrial	K03644	KOG2672	231	126	173	205	221	28	53	59	405	180	139	241	523	490	150	60
ACC_02588	UPF0172 protein CG3501-like		KOG3289	193	96	114	264	366	13	24	34	385	169	202	529	617	635	35	27
ACC_02589	conserved hypothetical protein	K16471	KOG0161	576	385	286	297	486	63	211	190	223	274	306	243	396	350	209	125
ACC_02590	protein FAM122A-like			147	118	120	248	170	11	18	26	127	119	35	79	97	94	33	6
ACC_02591	BTB/POZ domain-containing protein 9	K10481	KOG4350	266	171	181	500	482	25	38	61	358	242	223	349	263	300	38	13
ACC_02592	probable NADH dehydrogenase	K11352	KOG3382	330	174	191	784	621	24	39	69	742	230	367	560	575	649	58	55
ACC_02593	conserved hypothetical protein			0	2	1	0	0	0	0	0	0	1	0	0	0	0	0	0
ACC_02594	exocyst complex component 1		KOG2148	474	354	349	454	480	44	76	86	675	462	370	503	585	541	66	19
ACC_02595	tRNA methyltransferase 112 homolog	K15448	KOG1088	45	25	35	118	124	3	7	9	60	27	77	108	186	205	14	6
ACC_02596	cytochrome c oxidase assembly protein COX16 homolog, mitochondria			130	133	133	311	237	15	78	55	61	18	108	287	157	152	119	111
ACC_02597	vesicular glutamate transporter 2-like	K08193	KOG2532	32	111	38	41	28	22	19	43	302	62	160	804	190	188	28	4
ACC_02598	LOW QUALITY PROTEIN			36	36	44	23	40	1	3	0	12	28	45	45	42	104	13	1
ACC_02599	histone deacetylase 4	K11406	KOG1343	1104	675	686	961	200	104	84	111	1222	908	412	161	183	230	312	134
ACC_02600	SNF2 family DNA-dependent ATPase		KOG0388	473	243	309	205	148	27	50	81	187	163	74	53	82	76	24	16
ACC_02601	probable 26S proteasome non-ATPase regulatory s	K03033	KOG2581	471	260	250	636	583	48	66	89	998	624	542	1068	1848	2379	163	79
ACC_02602	GTP-binding nuclear protein Ran	K07936	KOG0096	320	227	182	541	342	37	20	36	852	641	145	716	2480	2593	885	140
ACC_02603	LOW QUALITY PROTEIN	K03539	KOG2363	254	223	317	268	369	14	26	18	146	159	202	372	359	353	69	17
ACC_02604	FACT complex subunit Ssrp1	K09272	KOG0526	2589	1347	1285	1770	1997	446	1046	997	1615	823	932	1035	3305	5528	6928	4677
ACC_02605	conserved hypothetical protein			435	342	397	1100	525	86	94	150	368	240	240	228	225	141	111	50
ACC_02606	putative inorganic phosphate cotransporter		KOG2532	218	67	113	258	64	10	11	8	313	348	57	212	110	225	274	26
ACC_02607	protein kinase C	K02677	KOG0696	3515	1625	1576	3416	1055	370	519	736	2871	1936	620	295	32	65	308	219
ACC_02608	bestrophin-3-like		KOG3547	124	71	58	87	85	26	55	55	422	456	46	40	1362	544	67	56
ACC_02609	TRAF3-interacting protein 1-like isoform 2		KOG3809	235	156	97	190	228	16	31	48	88	56	567	468	257	245	161	80
ACC_02610	solute carrier family 2, facilitated glucose transporter memb		KOG0254	199	90	81	107	82	7	13	21	107	55	52	104	78	50	12	0
ACC_02611	syntaxin-8-like	K08501	KOG3202	242	140	169	306	283	29	49	50	179	78	148	154	147	172	28	19
ACC_02612	acetylcholinesterase	K01049	KOG4389	286	79	106	172	42	6	4	12	271	378	4	15	7	10	16	12
ACC_02613	iron/zinc purple acid phosphatase-like protein-like		KOG1378	628	504	447	953	990	94	100	181	1240	700	632	1853	748	747	89	25
ACC_02614	LOW QUALITY PROTEIN	K07834	KOG0395	323	208	165	263	201	30	57	82	378	149	147	308	351	416	245	172
ACC_02615	solute carrier family 2, facilitated glucose transporter memb		KOG0569	373	256	165	278	258	83	147	213	1559	1297	443	846	1219	1089	96	25
ACC_02616	valyl-tRNA synthetase isoform 1	K01873	KOG0432	2967	1848	1300	1888	2103	822	1645	2018	2312	2036	1340	1556	3559	4574	2995	2485
ACC_02617	mCG3164-like	K07941	KOG0071	227	103	105	218	144	22	22	30	629	325	98	490	360	380	73	34
ACC_02618	isocitrate dehydrogenase	K00031	KOG1526	420	234	223	463	591	25	44	32	845	1019	399	1346	5201	3032	163	69
ACC_02619	LOW QUALITY PROTEIN		KOG0154	251	163	194	278	156	35	53	58	282	114	98	65	89	105	45	20

ACC_02620	conserved hypothetical protein			35	34	20	79	77	8	6	7	19	15	33	59	42	125	34	7
ACC_02621	conserved hypothetical protein	K09189	KOG1844	1580	1394	1404	2228	733	399	685	705	2592	2767	614	319	489	538	1284	595
ACC_02622	conserved hypothetical protein		KOG1911	1462	990	939	1853	794	244	456	588	893	788	658	398	995	1785	3128	1419
ACC_02623	hypothetical protein			43	30	48	114	135	4	5	8	55	44	80	107	40	51	18	11
ACC_02624	conserved hypothetical protein			2	3	3	5	1	0	0	0	1	1	0	0	1	1	8	7
ACC_02625	conserved hypothetical protein			38	28	20	26	26	1	9	10	83	59	19	12	8873	5557	334	385
ACC_02626	conserved hypothetical protein		KOG4788	175	105	117	174	82	14	19	32	276	169	38	179	370	323	126	50
ACC_02627	armadillo repeat-containing protein 8-like		KOG1293	203	89	117	293	230	8	8	6	242	185	102	260	335	371	50	8
ACC_02628	abhydrolase domain-containing protein 13-like	K06889	KOG4391	191	123	112	171	207	7	6	19	152	132	69	137	401	406	49	5
ACC_02629	calcium-binding mitochondrial carrier protein SCaM	K14684	KOG0036	342	311	268	469	371	54	77	83	1012	493	259	334	581	345	69	15
ACC_02630	nuclear pore complex protein Nup155	K14312	KOG1900	442	318	274	369	377	43	56	60	650	597	307	194	1132	1332	95	34
ACC_02631	eukaryotic translation initiation factor 2-alpha kina	K08860	KOG1035	1036	559	589	780	820	76	138	154	852	1015	445	1063	1275	868	222	67
ACC_02632	eukaryotic translation initiation factor 4E type 3-like		KOG1670	132	102	89	242	253	14	31	25	325	172	132	405	268	180	40	20
ACC_02633	translocon-associated protein subunit gamma-like	K13251	KOG4490	325	289	74	244	252	78	118	137	235	120	333	275	1488	1554	246	178
ACC_02634	conserved hypothetical protein	K06709	KOG0958	772	509	398	659	507	109	254	249	1015	694	411	315	267	257	329	121
ACC_02635	conserved hypothetical protein		KOG0147	90	63	47	139	90	5	9	4	130	89	41	32	112	131	20	6
ACC_02636	protein lap1-like	K12796	KOG0444	169	119	99	104	40	10	15	18	209	206	25	52	137	98	19	11
ACC_02637	conserved hypothetical protein	K10577	KOG0424	554	356	324	824	472	66	79	153	526	382	347	843	791	946	203	98
ACC_02638	serine/arginine-rich splicing factor 4-like isoform 1	K12893	KOG0106	2491	2053	2080	2192	1346	408	486	629	2199	1342	1298	1081	1801	2857	2074	1181
ACC_02639	NADH dehydrogenase	K03942	KOG2658	1573	878	1057	4230	2082	199	241	307	4144	1755	1666	4052	2196	2384	653	263
ACC_02640	adenylate cyclase 3	K08043	KOG3619	892	270	279	584	108	14	30	46	409	177	101	29	13	8	21	19
ACC_02641	dihydrolipoyl dehydrogenase, mitochondrial-like is	K00382	KOG1335	16	5	9	9	8	1	1	3	9	20	0	3	2	3	2	1
ACC_02642	hypothetical protein			0	1	0	1	0	0	0	1	1	6	0	0	1	0	1	1
ACC_02643	phosphopantothenoylcysteine decarboxylase-like	K01598	KOG0672	369	141	164	578	412	71	119	135	546	236	295	431	483	680	253	65
ACC_02644	cytochrome c oxidase assembly protein COX19-like		KOG3477	189	81	66	164	125	5	8	8	275	235	49	309	196	239	40	3
ACC_02645	conserved hypothetical protein	K12580	KOG2150	468	339	287	397	212	42	85	82	465	493	87	278	441	512	244	84
ACC_02646	WD repeat-containing protein 66-like		KOG0266	13	13	13	25	22	3	4	3	23	16	8	2	9	16	6	13
ACC_02647	thioredoxin reductase 1, mitochondrial-like isoform	K00384	KOG4716	520	318	303	856	638	89	103	130	1975	1260	953	3395	4012	5384	605	336
ACC_02648	RING finger protein 10-like		KOG2164	4505	2359	2160	4491	3057	1100	2060	2358	8738	4405	2503	4845	8673	8852	9078	4288
ACC_02649	serine/threonine-protein phosphatase 2A 65 kDa r	K03456	KOG0211	1342	758	717	1712	1319	129	152	203	2044	1696	1144	3480	2474	2826	420	115
ACC_02650	dipeptidase 1-like		KOG4127	567	568	586	662	546	160	151	265	952	1006	160	420	247	141	24	8
ACC_02651	conserved hypothetical protein		KOG2532	148	75	51	89	26	5	5	6	47	88	9	1	2	3	10	5
ACC_02652	TATA-box-binding protein-like		KOG3302	63	48	65	91	80	8	10	8	110	57	68	73	96	160	24	4
ACC_02653	counting factor associated protein D-like		KOG1543	1266	812	850	1580	1497	147	177	252	2889	2341	650	2417	2349	1890	137	66
ACC_02654	conserved hypothetical protein		KOG4323	5	2	3	12	6	4	2	3	10	5	1	1	3	0	8	3
ACC_02655	homocysteine S-methyltransferase ybgG-like	K00547	KOG1579	117	109	92	227	247	20	52	53	610	931	169	139	1765	647	47	19
ACC_02656	homocysteine S-methyltransferase 2-like		KOG1579	96	96	60	147	119	14	14	23	132	102	87	263	160	284	39	7
ACC_02657	proteasome maturation protein-like	K11599	KOG3061	307	442	478	663	593	36	51	60	705	573	929	1651	1523	2065	183	109
ACC_02658	vascular endothelial growth factor receptor 1-like	K05096	KOG0200	199	138	133	202	150	49	82	75	1669	1602	127	436	509	355	103	21
ACC_02659	zinc transporter 1-like		KOG1483	1133	864	645	858	567	119	263	256	457	216	34	92	363	334	122	30
ACC_02660	presqualene diphosphate phosphatase-like		KOG4268	128	91	63	115	124	7	19	11	174	141	59	248	211	159	10	9
ACC_02661	heat shock factor protein 5-like		KOG0627	1	3	1	3	2	0	1	0	23	9	1	0	5	6	5	0
ACC_02662	39S ribosomal protein L3, mitochondrial-like	K02906	KOG3141	371	221	216	539	499	41	59	66	663	403	248	560	997	1589	316	108
ACC_02663	hypothetical protein			0	0	0	0	0	0	0	0	0	0	0	1	1	0	0	0
ACC_02664	PHD finger protein 12-like		KOG4299	311	332	416	885	807	80	147	170	386	200	324	147	231	367	384	238
ACC_02665	transmembrane emp24 domain-containing protein-like		KOG1692	803	922	720	994	718	129	221	231	743	330	458	1056	2147	1958	240	117
ACC_02666	protein unc-119 homolog B		KOG4037	182	138	167	244	264	21	17	27	212	93	229	723	109	111	7	1
ACC_02667	UBX domain-containing protein 6	K14011	KOG2699	878	571	464	815	777	132	337	328	1397	963	833	1805	1092	983	320	140
ACC_02668	conserved hypothetical protein			110	94	68	82	87	6	8	7	283	293	54	439	233	117	7	2
ACC_02669	cytoplasmic protein NCK1-like isoform 2	K07365	KOG4226	471	251	278	708	410	45	74	92	662	350	144	406	410	429	220	56
ACC_02670	e3 ubiquitin-protein ligase RNF25-like isoform 1	K10640	KOG4445	200	80	72	182	128	9	5	5	139	104	46	118	115	97	18	5
ACC_02671	KIF1-binding protein homolog			401	274	319	457	351	39	74	81	499	316	200	516	427	480	76	44
ACC_02672	ribosome biogenesis protein BOP1 homolog	K14824	KOG0650	447	186	138	356	394	75	154	182	336	388	276	300	1258	1801	1758	993
ACC_02673	retrograde Golgi transport protein RGP1 homolog		KOG4469	289	236	309	345	287	17	25	38	398	212	187	401	259	267	55	18
ACC_02674	conserved hypothetical protein		KOG4029	11	4	2	23	17	0	0	1	16	5	12	9	259	153	34	99
ACC_02675	protein Daple-like, partial		KOG4643	963	595	560	897	758	140	347	338	501	369	329	119	380	473	386	201
ACC_02676	adipokinetic hormone receptor	K04280	KOG4219	28	9	6	23	23	1	5	6	85	111	5	47	137	99	11	2

ACC_02677	WAS protein family homolog 1-like		KOG1924	216	214	303	280	174	19	15	32	247	174	68	140	285	353	54	28
ACC_02678	vacuolar protein sorting-associated protein 37B-like	K12185	KOG3270	341	189	188	508	479	69	173	187	517	202	454	501	217	327	200	122
ACC_02679	conserved hypothetical protein		KOG4441	47	11	20	31	9	5	4	7	23	5	9	16	8	17	39	40
ACC_02680	rhomboid-related protein 3-like	K02857	KOG2289	57	34	23	33	33	4	5	6	127	67	27	226	114	118	12	5
ACC_02681	UPF0480 protein C15orf24 homolog		KOG3306	1048	502	504	888	907	61	76	114	2666	998	828	3238	1069	1113	44	30
ACC_02682	conserved hypothetical protein			31	10	8	92	45	3	11	11	68	36	3	4	265	167	70	24
ACC_02683	conserved hypothetical protein			12	8	7	16	9	1	1	0	7	4	6	3	3	2	0	1
ACC_02684	cytoplasmic dynein 2 light intermediate chain 1-like	K10417	KOG3929	23	13	25	16	13	7	9	10	65	22	108	211	25	31	5	3
ACC_02685	vacuolar protein sorting-associated protein 54		KOG2115	198	124	138	183	162	31	72	47	500	267	173	151	296	255	91	25
ACC_02686	LOW QUALITY PROTEIN		KOG0388	375	215	190	299	338	35	90	92	135	123	102	130	203	304	192	70
ACC_02687	conserved hypothetical protein		KOG4225	285	161	162	421	344	64	105	114	633	451	220	773	538	424	297	111
ACC_02688	conserved hypothetical protein			141	75	73	115	52	6	13	16	206	205	27	67	86	67	41	7
ACC_02689	conserved hypothetical protein			230	140	135	409	266	58	123	147	528	306	149	76	107	170	162	65
ACC_02690	UHRF1-binding protein 1-like isoform 1		KOG2955	739	404	333	366	385	53	75	106	774	1121	169	335	845	714	112	48
ACC_02691	clathrin heavy chain-like isoform 1	K04646	KOG0985	3115	1321	1159	1380	1190	223	394	496	4234	3583	640	1962	2556	2247	420	178
ACC_02692	39S ribosomal protein L28, mitochondrial	K02902	KOG3279	221	235	275	235	255	26	46	61	278	123	213	402	579	836	145	112
ACC_02693	conserved hypothetical protein		KOG1013	802	869	882	1663	969	105	137	173	844	446	752	572	82	105	40	23
ACC_02694	two pore calcium channel protein 1		KOG2302	696	406	364	497	574	34	33	47	818	525	232	484	259	232	12	9
ACC_02695	AP-3 complex subunit beta-1-like isoform 2	K12397	KOG1060	5154	3850	2252	4156	3541	1303	2685	2837	4057	3263	3320	5188	17209	22724	7903	4133
ACC_02696	sorting nexin-25-like		KOG2101	536	362	337	392	400	34	50	45	1110	685	236	753	687	579	62	12
ACC_02697	protein kinase C iota type isoform 1			395	168	134	609	165	37	62	76	132	141	94	19	10	5	76	47
ACC_02698	slit homolog 2 protein-like		KOG4194	11	1	5	18	20	1	0	1	139	257	24	6	54	59	5	1
ACC_02699	RING finger and SPRY domain-containing protein 1-like		KOG2242	85	51	49	98	77	5	6	14	129	117	32	106	159	109	11	2
ACC_02700	DNA-binding protein inhibitor ID-2-A-like	K04680		157	51	73	241	77	44	48	62	224	169	114	190	132	268	376	89
ACC_02701	conserved hypothetical protein	K15324		163	72	88	132	200	13	60	64	64	37	94	142	64	115	93	39
ACC_02702	venom acid phosphatase		KOG3720	4	0	59	11	11	1	1	1	67	4	33	47	157	180	33	6
ACC_02703	angiotensin-converting enzyme-like	K01283	KOG3690	1976	552	822	3206	1947	82	126	154	1793	435	271	974	6196	5484	693	89
ACC_02704	hypothetical protein			8	7	6	18	11	2	1	3	21	32	11	5	0	0	0	0
ACC_02705	stromal interaction molecule homolog isoform 1	K16059	KOG4403	415	217	277	493	243	95	151	205	655	477	202	162	310	165	306	169
ACC_02706	NEDD8-like	K12158	KOG0005	510	452	592	635	322	57	132	176	311	153	166	335	322	541	319	233
ACC_02707	cdc42 homolog	K04393	KOG0393	112	76	67	135	106	2	10	12	207	185	51	229	400	420	56	14
ACC_02708	AKT-interacting protein-like		KOG0429	263	177	210	353	424	27	35	53	419	195	178	469	338	396	65	19
ACC_02709	major royal jelly protein 1			6	1	5	28	7	0	2	0	19	9	1	9	2	1	9	2
ACC_02710	conserved hypothetical protein			5	3	6	16	9	0	1	0	12	7	0	3	0	2	1	0
ACC_02711	nudC domain-containing protein 3-like		KOG2265	164	111	98	241	257	18	30	30	256	95	143	301	127	182	7	6
ACC_02712	SET and MYND domain-containing protein 4-like		KOG2084	73	48	50	71	90	5	18	10	152	48	58	54	31	50	13	5
ACC_02713	THUMP domain-containing protein 1-like, partial		KOG3943	144	136	104	193	234	16	51	76	130	126	225	327	214	248	57	39
ACC_02714	conserved hypothetical protein			124	88	81	189	98	14	20	11	165	126	91	182	244	212	80	23
ACC_02715	conserved hypothetical protein		KOG3006	763	530	526	1264	1491	144	268	263	901	497	645	1006	841	965	627	470
ACC_02716	conserved hypothetical protein		KOG1029	1688	1043	1182	2048	1098	113	188	215	1208	511	275	279	235	216	142	59
ACC_02717	u6 snRNA-associated Sm-like protein LSm5-like	K12624	KOG1775	90	37	43	158	132	6	11	14	53	44	67	196	118	184	47	18
ACC_02718	nucleoside diphosphate kinase	K00940	KOG0888	637	404	193	800	688	164	186	220	1376	543	1289	2466	2273	2274	388	227
ACC_02719	methyltransferase-like protein 14 homolog		KOG2097	7819	6922	7737	9911	6730	854	1819	2372	3896	4170	1310	812	1425	2227	909	311
ACC_02720	beta-1,4-N-acetylgalactosaminyltransferase bre-4		KOG3916	100	69	52	89	29	28	33	45	305	389	9	42	56	34	24	7
ACC_02721	DNA-directed RNA polymerase II subunit RPB7-like	K03015	KOG3298	94	81	141	228	179	10	21	18	69	44	84	257	192	358	45	30
ACC_02722	phosphatidylserine synthase 1-like	K08729	KOG2735	184	128	89	133	122	14	13	20	447	528	56	183	688	534	54	15
ACC_02723	cytochrome b5-like		KOG0537	260	334	210	295	220	51	97	75	207	143	222	450	685	716	83	75
ACC_02724	protein vav-like	K05730	KOG2996	647	353	316	537	382	102	124	135	1067	620	294	391	673	634	307	91
ACC_02725	conserved hypothetical protein			55	38	41	39	56	2	17	21	17	10	22	43	31	59	52	53
ACC_02726	1-acyl-sn-glycerol-3-phosphate acyltransferase alpl	K13509	KOG2848	123	55	53	112	132	5	9	18	34	21	25	43	4	10	5	2
ACC_02727	Serine/threonine protein kinase		KOG2268	1	0	3	0	0	0	0	0	1	0	0	0	0	0	0	0
ACC_02728	conserved hypothetical protein			4	4	11	11	5	1	0	0	4	14	5	1	2	2	1	0
ACC_02729	box C/D snoRNA protein 1-like		KOG2858	181	130	134	224	266	11	19	19	203	146	109	399	364	383	55	16
ACC_02730	zinc finger CCCH-type with G patch domain-containing prote		KOG2185	476	413	455	541	473	88	177	194	344	241	178	250	201	215	110	72
ACC_02731	autophagy protein 5	K08339	KOG2976	142	111	123	156	108	5	7	16	161	123	47	224	360	307	36	5
ACC_02732	probable nucleoporin Nup54-like	K14308	KOG3091	111	113	101	170	109	12	13	13	204	135	106	140	417	875	119	35
ACC_02733	conserved hypothetical protein		KOG0566	326	182	199	389	86	32	37	72	390	232	67	114	64	101	116	68

ACC_02734	myotubularin-related protein 2-like		KOG4471	233	171	146	398	324	40	50	56	419	281	212	381	422	580	90	40
ACC_02735	conserved hypothetical protein			310	241	196	321	90	59	72	78	647	675	83	356	234	128	160	28
ACC_02736	26S proteasome non-ATPase regulatory subunit 5-l	K06692	KOG4413	186	135	138	217	230	12	28	30	302	321	245	608	473	513	53	22
ACC_02737	heat shock protein cognate 3	K09490	KOG0100	8434	3812	3176	11120	14555	977	1717	3767	7490	4761	3506	6538	15868	20455	5819	5193
ACC_02738	delta-aminolevulinic acid dehydratase	K01698	KOG2794	180	103	91	292	274	18	21	24	263	134	327	858	551	642	76	30
ACC_02739	probable uridine-cytidine kinase-like isoform 1	K00876	KOG4203	322	186	209	381	360	17	24	46	391	216	158	392	336	330	25	18
ACC_02740	neuropathy target esterase sws	K14676	KOG2968	1000	568	632	1226	842	129	161	154	2173	1549	508	975	1066	1449	282	102
ACC_02741	leucine-rich repeat-containing protein 20-like isoform 1		KOG4579	233	96	100	271	119	17	19	14	1126	422	128	179	43	44	10	5
ACC_02742	PDZ domain-containing protein 8-like		KOG3532	387	237	262	334	256	30	44	47	482	320	107	199	180	131	26	10
ACC_02743	conserved hypothetical protein		KOG1834	254	199	232	289	212	49	90	114	147	113	104	148	180	233	261	479
ACC_02744	ras-related protein Rab-9A-like	K07899	KOG0394	75	48	47	95	76	12	23	11	130	96	47	188	121	91	20	9
ACC_02745	probable tRNA pseudouridine synthase 2-like		KOG2559	78	53	66	53	79	3	4	7	70	70	53	121	208	180	14	7
ACC_02746	phosrestin-1-like		KOG3865	70	49	51	108	43	7	9	9	37	20	9	2	2	7	28	9
ACC_02747	conserved hypothetical protein		KOG3700	27	18	8	8	3	20	35	15	291	329	4	5	15	7	19	18
ACC_02748	conserved hypothetical protein	K09312	KOG0848	4	7	2	7	2	0	0	0	0	5	4	3	48	34	9	3
ACC_02749	chromosome transmission fidelity protein 18 homo	K11269	KOG1969	184	112	90	160	171	21	37	53	168	104	89	95	175	246	118	56
ACC_02750	conserved hypothetical protein		KOG0769	429	429	351	1173	886	101	137	132	8254	4322	89	342	1117	1516	195	74
ACC_02751	conserved hypothetical protein			580	349	409	561	641	37	58	87	533	195	335	1165	691	658	92	99
ACC_02752	sorbitol dehydrogenase-like isoform 1	K00008	KOG0024	299	137	260	558	397	49	81	68	3995	16815	207	912	7454	6608	890	255
ACC_02753	RING finger protein 126-like	K11982	KOG4628	939	551	589	749	398	94	90	148	1447	1000	317	1124	1223	1345	399	128
ACC_02754	mitogen-activated protein kinase kinase kinase 4		KOG4645	381	373	379	761	557	71	139	187	453	613	272	303	457	344	137	75
ACC_02755	LOW QUALITY PROTEIN		KOG1064	1889	1254	1301	1377	847	238	333	376	3125	3687	560	888	1196	750	340	146
ACC_02756	conserved hypothetical protein		KOG1892	485	961	569	158	79	264	200	303	6443	2046	75	75	229	163	46	34
ACC_02757	homeobox protein Hox-A2		KOG0489	2	0	4	5	6	1	0	1	0	3	3	0	4	1	1	3
ACC_02758	ER lumen protein retaining receptor-like	K10949	KOG3106	310	232	118	258	225	27	30	33	205	260	102	596	1088	924	44	16
ACC_02759	seryl-tRNA synthetase, mitochondrial-like	K01875	KOG3408	195	147	78	163	197	16	32	44	211	191	113	219	564	864	144	63
ACC_02760	tetratricopeptide repeat protein 37-like	K12600	KOG1127	193	103	81	162	141	9	20	16	260	413	67	273	440	352	43	16
ACC_02761	conserved hypothetical protein		KOG0032	4395	6134	6085	6134	4150	9370	21787	15912	100776	41881	2797	404	1265	2708	12108	12525
ACC_02762	conserved hypothetical protein	K03968	KOG4516	306	134	123	378	366	38	53	84	276	110	284	616	559	518	35	19
ACC_02763	T-complex-associated testis-expressed protein 1		KOG1909	32	18	16	34	9	0	5	6	66	12	23	15	1	4	1	0
ACC_02764	serine/threonine-protein kinase RIO2-like	K07179	KOG2268	680	468	497	610	697	94	177	195	828	564	324	672	777	1039	307	163
ACC_02765	conserved hypothetical protein			668	374	277	475	508	30	152	158	665	730	271	442	663	598	204	62
ACC_02766	DET1 homolog	K10571	KOG2558	101	59	74	141	158	12	8	10	131	76	62	75	106	131	11	5
ACC_02767	conserved hypothetical protein			322	178	141	137	85	21	25	33	327	345	38	317	245	288	56	11
ACC_02768	conserved hypothetical protein			610	281	222	332	483	38	143	154	207	221	355	496	385	284	374	156
ACC_02769	solute carrier family 25 member 46-like isoform 1	K03454	KOG2954	364	212	204	479	386	24	26	42	500	301	190	557	452	584	49	19
ACC_02770	dynein heavy chain 7, axonemal-like		KOG3595	257	206	215	547	448	31	72	73	131	451	101	86	20	7	21	10
ACC_02771	alpha-1,3-mannosyl-glycoprotein 2-beta-N-acetylgl	K00726	KOG1413	272	122	146	370	404	30	59	80	638	377	230	565	523	545	199	99
ACC_02772	centrosomal protein of 135 kDa-like		KOG0161	160	114	92	198	95	38	52	69	262	157	102	90	90	116	71	26
ACC_02773	centrosomal protein of 135 kDa-like	K16461	KOG0161	840	617	537	697	720	159	295	383	597	468	448	261	275	335	705	357
ACC_02774	LOW QUALITY PROTEIN		KOG0266	180	112	155	170	124	25	30	61	74	48	175	21	24	53	30	19
ACC_02775	protein YIPF6-like		KOG2946	217	137	129	239	199	38	53	64	272	136	138	421	308	322	48	24
ACC_02776	potassium channel subfamily T member 1-like		KOG3193	1446	967	840	1640	539	67	89	143	789	743	279	164	41	41	42	23
ACC_02777	conserved hypothetical protein			47	20	16	24	26	2	5	5	19	25	2	7	11	12	14	6
ACC_02778	actin-related protein 8-like isoform 1	K11673	KOG0797	159	134	130	217	174	14	12	18	116	66	51	120	255	332	71	22
ACC_02779	Otoferlin		KOG1028	0	0	0	0	1	0	0	0	0	1	2	0	0	0	0	0
ACC_02780	protein DDI1 homolog 2-like	K11885	KOG0012	388	216	197	277	169	59	81	102	1064	711	231	806	1793	1253	664	384
ACC_02781	optic atrophy 1-like isoform 2		KOG0447	629	378	340	604	476	60	88	102	798	536	248	273	689	829	168	110
ACC_02782	facilitated trehalose transporter Tret1-like		KOG0254	224	172	124	251	132	32	18	32	575	442	143	916	261	405	183	43
ACC_02783	conserved hypothetical protein		KOG4219	86	58	39	130	27	32	31	38	37	15	15	8	0	3	14	3
ACC_02784	conserved hypothetical protein		KOG1985	990	704	780	429	119	75	76	119	730	555	200	654	258	164	157	52
ACC_02785	aspartyl-tRNA synthetase, cytoplasmic	K01876	KOG0556	754	419	402	777	781	108	156	245	1073	1082	487	1085	2982	3290	560	553
ACC_02786	BTB/POZ domain-containing protein 17		KOG4441	270	184	204	584	449	26	36	69	396	269	185	301	286	341	76	26
ACC_02787	sialin-like	K08193	KOG2532	98	129	74	68	84	29	33	43	601	454	93	313	380	462	46	6
ACC_02788	peroxiredoxin-5, mitochondrial	K11187	KOG0541	298	227	197	599	624	29	59	87	830	232	375	670	510	658	43	20
ACC_02789	conserved hypothetical protein		KOG4177	180	71	56	67	22	10	5	15	251	351	25	46	6	3	12	4
ACC_02790	UDP-glucuronosyltransferase 2B19-like	K13754	KOG2399	13	6	3	17	12	1	0	0	36	39	6	16	241	236	41	4

ACC_02791	hypothetical protein			20	15	10	30	23	4	6	5	7	3	1	0	0	2	23	20
ACC_02792	hypothetical protein			8	4	6	4	1	1	1	1	3	1	1	0	0	0	5	10
ACC_02793	DDB1- and CUL4-associated factor 13-like	K11806	KOG0268	191	165	195	561	512	22	53	47	282	197	210	341	651	775	154	47
ACC_02794	n-acetylneuraminate lyase-like	K01639		35	33	37	148	92	7	22	21	319	725	3	19	4865	1581	112	96
ACC_02795	beta-1,3-glucan-binding protein			18	11	22	75	95	8	10	7	742	516	70	144	77	17	1	2
ACC_02796	protein tailless-like	K08545	KOG4215	0	0	0	0	0	0	0	0	0	0	0	0	0	3	1	0
ACC_02797	zinc finger and SCAN domain-containing protein 22-like		KOG1074	85	70	80	106	34	2	3	5	33	42	6	8	4	14	13	10
ACC_02798	conserved hypothetical protein			0	1	2	4	3	0	1	1	2	4	2	8	2	0	0	1
ACC_02799	GTPase-activating protein and VPS9 domain-containing prot		KOG2319	865	657	524	483	421	73	120	111	1388	898	369	292	718	928	152	63
ACC_02800	60S ribosomal protein L12	K02870	KOG0886	700	574	283	849	662	115	164	219	528	642	425	711	3978	5562	755	449
ACC_02801	ribonuclease UK114-like		KOG2317	37	31	46	70	69	6	9	5	591	607	2	2	2945	1421	48	65
ACC_02802	conserved hypothetical protein		KOG4822	415	289	361	487	256	49	68	60	580	357	156	215	410	465	417	147
ACC_02803	UV sensitive opsin	K04255	KOG4219	220	166	181	675	399	194	257	431	1538	762	82	31	199	273	72	20
ACC_02804	LOW QUALITY PROTEIN		KOG2644	200	100	75	89	96	12	11	25	320	180	53	199	1118	742	75	32
ACC_02805	tRNA (guanine-N(1)-)-methyltransferase-like	K15429	KOG2078	672	367	267	549	652	48	276	310	352	320	522	558	852	980	592	569
ACC_02806	glucose-fructose oxidoreductase domain-containing protein		KOG2742	360	280	285	468	503	23	45	78	381	198	144	412	304	302	43	14
ACC_02807	RNA-binding protein NOB1-like	K11883	KOG2463	487	193	177	463	553	34	135	141	222	219	322	409	592	1044	747	509
ACC_02808	zinc finger protein 271-like		KOG2462	494	315	335	1129	689	84	200	227	219	131	151	42	14	52	409	131
ACC_02809	superoxide dismutase 2, mitochondrial	K04564	KOG0876	350	123	128	526	492	15	21	37	601	172	328	800	1626	1129	59	22
ACC_02810	conserved hypothetical protein		KOG0161	3861	2046	1599	2046	3418	417	1072	1142	2009	1840	2168	1180	1358	1156	937	395
ACC_02811	calcium uniporter protein, mitochondrial-like		KOG2966	105	55	49	84	40	4	22	20	179	81	23	64	84	40	33	22
ACC_02812	hypothetical protein			3	2	0	1	1	0	2	0	2	13	0	0	0	0	0	0
ACC_02813	ras-like protein 1-like	K07827	KOG0395	169	92	82	100	84	22	56	46	224	176	48	172	272	312	259	198
ACC_02814	conserved hypothetical protein	K07750	KOG0873	855	677	598	1333	768	89	67	100	3947	7920	1003	3485	176	112	14	7
ACC_02815	tctex1 domain-containing protein 1-like		KOG4108	1	0	1	5	0	0	1	0	2	1	3	0	2	0	1	2
ACC_02816	molybdenum cofactor biosynthesis protein 1	K03639	KOG2876	690	648	376	443	335	142	198	199	987	877	339	1244	1912	1196	129	45
ACC_02817	DNA fragmentation factor subunit alpha-like			461	191	185	322	257	26	22	34	448	210	69	85	208	179	31	4
ACC_02818	LOW QUALITY PROTEIN	K16482	KOG0266	91	90	80	109	123	7	8	12	77	35	40	48	110	152	20	8
ACC_02819	conserved hypothetical protein		KOG3598	2540	2795	2851	3044	1024	241	293	346	3354	3314	308	1621	6014	6434	2994	1172
ACC_02820	conserved hypothetical protein	K16751		548	340	278	413	448	28	70	81	526	634	301	419	653	503	137	74
ACC_02821	CWF19-like protein 2-like, partial		KOG2477	1767	962	940	1381	1926	308	681	751	1970	1153	1331	1717	1520	1396	772	461
ACC_02822	conserved hypothetical protein			190	163	136	795	657	42	51	71	3409	792	378	635	500	161	24	11
ACC_02823	conserved hypothetical protein			0	2	0	0	0	0	1	0	16	3	0	0	0	1	2	0
ACC_02824	conserved hypothetical protein		KOG3689	506	300	300	424	149	34	39	41	681	520	57	67	169	211	30	26
ACC_02825	endoplasmic reticulum resident protein 29-like	K09586		442	409	276	580	492	74	138	139	134	162	172	688	885	1073	357	180
ACC_02826	conserved hypothetical protein			937	333	429	1760	1410	77	102	136	675	262	631	846	302	426	113	23
ACC_02827	probable ATP-dependent RNA helicase DDX49-like	K14778	KOG0340	371	325	277	416	404	82	103	148	596	403	418	774	759	873	188	116
ACC_02828	conserved hypothetical protein		KOG2589	780	413	409	710	627	80	191	232	531	608	263	203	310	321	323	154
ACC_02829	conserved hypothetical protein			274	209	196	176	225	6	10	11	102	260	112	425	649	343	40	6
ACC_02830	conserved hypothetical protein			10	8	10	35	20	0	1	1	8	7	0	0	1	2	0	0
ACC_02831	rab GDP dissociation inhibitor beta		KOG1439	907	351	337	743	512	31	36	30	1295	1217	295	1512	1680	1576	173	51
ACC_02832	insulin-like receptor-like	K04527	KOG4258	490	151	222	474	132	48	61	75	1142	1213	140	406	187	244	300	197
ACC_02833	maspardin-like		KOG4178	159	78	66	245	177	27	22	27	151	117	42	219	412	404	185	18
ACC_02834	soma ferritin	K00522	KOG2332	1115	419	552	1205	1058	98	172	290	352	90	559	585	34	105	78	35
ACC_02835	conserved hypothetical protein		KOG1029	159	71	66	157	132	16	29	26	123	52	304	371	35	22	41	15
ACC_02836	conserved hypothetical protein			402	397	338	1436	895	154	119	194	271	256	47	172	43	76	26	6
ACC_02837	Pax-6 protein (eyegone)			0	0	0	0	1	0	0	0	9	17	37	80	5	11	47	48
ACC_02838	conserved hypothetical protein			174	107	123	904	339	63	83	52	8338	2959	426	601	513	136	112	71
ACC_02839	conserved hypothetical protein	K15195	KOG0566	594	387	487	760	164	63	88	121	674	995	308	123	73	159	334	109
ACC_02840	cathepsin O-like	K01374	KOG1542	64	41	52	114	106	7	19	12	258	197	139	479	161	79	13	5
ACC_02841	ras-related protein Rab-30-like isoform 1	K07917	KOG0095	60	45	42	111	88	3	4	9	192	107	50	178	51	35	8	2
ACC_02842	hemicentin-1-like		KOG4221	2634	1436	1936	5983	2982	207	276	422	1566	667	130	199	23	78	44	24
ACC_02843	conserved hypothetical protein		KOG3621	400	271	217	397	424	36	93	79	525	521	244	600	560	426	101	29
ACC_02844	cofilin/actin-depolymerizing factor homolog	K05765	KOG1735	664	541	386	936	645	130	187	230	1281	731	458	1460	1794	2362	975	285
ACC_02845	centrosomal protein of 120 kDa-like	K16459	KOG0996	467	343	279	259	282	19	35	42	204	155	100	141	202	249	35	23
ACC_02846	n-acetylglucosaminyl-phosphatidylinositol de-N-ac	K03434	KOG3332	73	36	45	97	142	9	7	12	129	63	106	153	136	120	23	3
ACC_02847	jmjC domain-containing histone demethylation pro	K10276	KOG1633	158	94	95	159	109	16	7	8	484	418	85	219	296	239	49	4

ACC_02848	coiled-coil domain-containing protein 63-like		KOG0161	17	18	7	31	42	4	3	1	9	24	15	21	17	14	9	22
ACC_02849	hypothetical protein			0	0	2	3	0	0	0	3	2	0	1	0	0	2	5	0
ACC_02850	transmembrane protein 93-like		KOG4455	141	73	95	196	188	28	33	38	249	75	188	382	212	412	41	19
ACC_02851	seipin-like		KOG4200	197	100	83	267	256	9	23	17	148	111	103	349	267	225	33	13
ACC_02852	retinoblastoma-binding protein 5	K14961	KOG1273	167	136	133	193	178	20	34	36	235	119	106	190	233	241	42	20
ACC_02853	sequestosome-1	K14381	KOG3591	5891	12354	12521	45095	15829	2452	3778	4552	14117	16375	6345	10786	2350	2424	2934	850
ACC_02854	LOW QUALITY PROTEIN	K14864	KOG1099	173	149	119	332	209	33	49	86	239	126	175	227	304	400	77	55
ACC_02855	conserved hypothetical protein		KOG3513	6	1	0	2	4	0	0	0	30	7	13	107	77	78	18	14
ACC_02856	conserved hypothetical protein			137	89	77	107	154	15	37	28	24	18	41	29	178	362	481	216
ACC_02857	vacuolar-sorting protein SNF8	K12188	KOG3341	129	145	125	208	173	36	80	83	168	111	99	196	194	278	88	42
ACC_02858	10-formyltetrahydrofolate dehydrogenase	K00289	KOG2452	54	19	79	302	238	108	75	46	2081	1019	52	465	1719	1214	137	23
ACC_02859	conserved hypothetical protein	K01444	KOG1593	173	108	110	337	224	19	27	29	277	992	120	370	1070	649	116	16
ACC_02860	probable ATP-dependent RNA helicase kurz	K14780	KOG0926	1898	1276	1038	1575	1714	235	813	686	1573	1122	840	643	2278	3238	2916	1721
ACC_02861	ribonuclease H2 subunit A-like isoform 1	K10743	KOG2299	96	70	79	215	93	6	14	10	109	75	39	61	213	233	64	9
ACC_02862	survival of motor neuron protein-interacting protei	K13130		158	115	139	372	363	30	68	71	173	98	186	235	248	359	117	50
ACC_02863	alpha-mannosidase 2	K01191	KOG1958	214	136	94	99	23	6	15	7	416	297	27	92	41	84	23	14
ACC_02864	cuticular protein analogous to peritrophins 3-C precursor			157	25	44	95	20	37	36	59	406	135	4	736	804	1055	666	785
ACC_02865	aquaporin-like		KOG0223	14	3	3	12	10	2	1	0	34	87	8	11	4	39	18	2
ACC_02866	conserved hypothetical protein		KOG3562	5	0	2	16	4	0	1	2	155	232	1	0	80	38	36	7
ACC_02867	sin3 histone deacetylase corepressor complex component SI		KOG4466	486	382	433	747	597	95	227	238	510	233	299	212	303	418	345	236
ACC_02868	coiled-coil domain-containing protein 50-like			233	155	143	206	142	46	72	83	390	141	162	382	165	210	159	92
ACC_02869	conserved hypothetical protein	K16812		76	50	38	9	20	9	4	6	23	51	23	26	337	1036	895	367
ACC_02870	conserved hypothetical protein			229	143	180	353	140	34	49	58	213	139	72	115	68	95	149	37
ACC_02871	ubiquitin-like modifier-activating enzyme atg7-like	K08337	KOG2337	562	466	469	763	651	46	55	74	687	563	462	930	451	440	41	18
ACC_02872	ATP-binding cassette sub-family G member 5-like		KOG0061	80	38	32	160	142	13	23	26	113	84	45	137	133	181	40	12
ACC_02873	phospholipase A-2-activating protein	K14018	KOG0301	252	160	161	336	282	24	31	36	396	359	130	447	641	634	69	26
ACC_02874	pachytene checkpoint protein 2 homolog		KOG0744	59	44	39	78	91	7	3	8	96	54	71	125	117	203	20	7
ACC_02875	solute carrier family 35 member E1 homolog isofor	K15283	KOG1441	179	189	89	175	132	19	30	38	304	264	54	264	439	339	63	10
ACC_02876	protein RRP5 homolog isoform 1	K14792	KOG1070	491	279	292	451	473	94	190	207	445	226	333	574	426	700	292	165
ACC_02877	protein RRP5 homolog	K09008	KOG3363	74	78	75	93	125	2	6	10	87	41	62	218	213	234	8	5
ACC_02878	interferon-related developmental regulator 1-like		KOG2842	272	261	191	336	265	73	116	126	1207	579	179	847	1153	1165	210	56
ACC_02879	calcyphosin-like protein-like		KOG0032	15	26	36	131	77	15	13	29	171	383	1215	2829	89	104	18	21
ACC_02880	nardilysin isoform 2	K01411	KOG0959	711	459	404	777	775	95	172	206	1098	868	412	785	955	1191	287	182
ACC_02881	chromodomain-helicase-DNA-binding protein Mi-2	K11643	KOG0383	5127	4041	3855	7669	5359	1330	2489	3001	4680	4098	2606	1479	3024	3761	10616	8853
ACC_02882	conserved hypothetical protein			647	332	323	617	577	38	86	82	1091	443	385	1050	438	469	165	74
ACC_02883	conserved hypothetical protein	K09200	KOG2462	466	499	488	466	248	64	71	108	404	291	200	131	352	503	221	73
ACC_02884	vacuolar protein sorting-associated protein 33B-like		KOG1302	292	243	216	244	312	16	40	41	340	334	173	453	462	474	34	9
ACC_02885	sorting nexin-30-like		KOG2273	356	179	173	468	385	32	37	34	471	212	205	438	193	225	28	10
ACC_02886	conserved hypothetical protein			36	45	38	40	76	3	2	1	29	33	66	103	48	38	4	7
ACC_02887	conserved hypothetical protein			3	11	11	24	11	1	0	1	1	0	2	4	2	2	1	0
ACC_02888	conserved hypothetical protein			435	236	209	803	494	51	74	79	278	150	549	908	667	665	247	147
ACC_02889	protein brown-like		KOG0061	2	1	0	2	2	1	0	1	13	5	18	35	5	6	1	0
ACC_02890	conserved hypothetical protein		KOG4423	109	77	55	111	50	66	71	110	1175	560	17	153	190	186	60	6
ACC_02891	signal recognition particle 72 kDa protein-like	K03108	KOG2376	523	295	158	197	198	104	184	204	432	382	168	509	1516	1559	539	378
ACC_02892	conserved hypothetical protein		KOG3119	1	10	8	6	3	0	0	1	1	3	8	3	4	1	1	1
ACC_02893	transferrin-like			102	79	64	238	195	23	25	32	561	345	153	188	412	232	43	16
ACC_02894	cytoplasmic aconitate hydratase-like isoform 1	K01681	KOG0452	432	269	268	445	377	57	53	84	1061	944	452	1353	2725	1413	122	39
ACC_02895	protein RFT1 homolog	K06316	KOG2864	214	149	161	275	305	11	26	18	238	188	115	442	280	281	38	10
ACC_02896	ras-related protein Rab-24-like		KOG0092	167	87	98	187	190	19	34	37	176	93	121	187	223	309	61	19
ACC_02897	conserved hypothetical protein		KOG3934	122	100	99	157	177	12	24	23	155	61	125	140	174	390	66	34
ACC_02898	conserved hypothetical protein			2193	1719	1995	1803	1348	316	629	540	1974	1499	621	467	1144	1028	388	139
ACC_02899	transcription initiation protein SPT3 homolog	K11313	KOG3902	73	64	65	93	113	5	11	11	61	47	49	68	54	95	16	5
ACC_02900	LOW QUALITY PROTEIN	K02604	KOG2928	80	48	32	78	92	6	13	13	63	50	67	62	182	249	84	40
ACC_02901	hypothetical protein			32	17	25	41	31	2	2	1	10	4	10	2	1	2	0	1
ACC_02902	conserved hypothetical protein			245	133	149	267	265	27	31	51	231	172	158	369	176	219	52	9
ACC_02903	conserved hypothetical protein		KOG1960	584	651	696	453	174	51	53	71	741	462	172	165	264	277	119	42
ACC_02904	BTB/POZ domain-containing protein 2-like	K10477	KOG2075	353	233	220	347	334	18	25	46	615	516	170	421	522	533	90	12

ACC_02905	WD repeat-containing protein 26-like		KOG0293	170	81	67	92	76	8	21	22	407	277	39	185	267	264	46	15
ACC_02906	DNA-directed RNA polymerase I subunit RPA1	K02999	KOG0262	752	479	357	582	621	91	203	191	877	882	545	505	1283	1832	933	441
ACC_02907	conserved hypothetical protein			11	8	8	8	7	6	17	15	438	1129	4	6	625	296	210	144
ACC_02908	hypothetical protein			1	0	0	0	0	0	0	0	1	1	0	0	0	0	2	1
ACC_02909	carnitine O-palmitoyltransferase 1, liver isoform-like		KOG3716	761	304	184	413	400	132	237	263	1123	2039	329	755	804	396	133	53
ACC_02910	coiled-coil domain-containing protein 43-like			438	304	232	425	332	112	263	276	283	116	255	216	317	456	2040	1934
ACC_02911	conserved hypothetical protein			2	0	0	2	1	0	0	2	9	7	0	2	2	3	19	6
ACC_02912	conserved hypothetical protein			103	68	50	158	169	19	27	25	419	139	85	173	156	162	30	6
ACC_02913	t-complex protein 1 subunit beta-like isoform 1	K09494	KOG0363	677	400	345	1075	778	73	100	103	1094	1049	535	1240	4106	5083	885	633
ACC_02914	MIT domain-containing protein 1-like		KOG4509	92	54	69	59	65	3	9	5	123	68	68	111	136	187	18	5
ACC_02915	probable protein phosphatase 2C T23F11.1-like	K14803	KOG0697	126	67	56	95	80	2	9	15	347	128	38	178	67	66	6	1
ACC_02916	LOW QUALITY PROTEIN	K10807	KOG1112	116	112	108	177	138	12	24	26	247	208	96	179	960	1465	127	33
ACC_02917	probable isocitrate dehydrogenase	K00030	KOG0785	696	224	268	414	361	34	40	63	1283	425	250	957	894	1240	111	32
ACC_02918	protein fem-1 homolog CG6966-like		KOG0508	553	426	387	374	242	49	51	61	1001	609	169	320	397	720	86	25
ACC_02919	LOW QUALITY PROTEIN	K13140	KOG4262	185	92	93	199	108	34	57	81	411	424	52	126	206	230	275	131
ACC_02920	conserved hypothetical protein	K15293	KOG1011	1922	1376	1471	3967	1435	156	256	331	738	513	371	77	69	71	196	90
ACC_02921	hypothetical protein			7	7	12	22	10	0	3	1	17	85	2	2	3	9	4	8
ACC_02922	protein SZT2-like			9	8	4	15	9	2	2	0	24	57	1	15	29	34	2	0
ACC_02923	probable ATP-dependent RNA helicase DDX17-like	K12823	KOG0331	851	557	471	786	473	130	153	180	801	681	298	376	1249	1888	487	253
ACC_02924	probable ATP-dependent RNA helicase DDX17-like	K12823	KOG0331	1363	666	706	1718	974	89	150	196	397	234	360	371	195	170	81	50
ACC_02925	conserved hypothetical protein			24	21	9	29	20	2	7	10	26	16	165	18	46	29	78	19
ACC_02926	selenium-binding protein 1-like		KOG0918	29	87	85	235	191	3	2	8	375	315	6	48	999	1300	65	7
ACC_02927	multidrug resistance-associated protein 7-like		KOG0054	123	71	75	118	112	12	9	19	235	240	51	58	171	108	32	30
ACC_02928	conserved hypothetical protein		KOG0161	40	30	34	85	48	10	11	15	233	129	48	65	38	15	10	4
ACC_02929	conserved hypothetical protein		KOG1832	1734	1760	1511	1769	719	694	1470	1339	2193	1204	1551	1045	1816	3745	38098	64512
ACC_02930	WASH complex subunit CCDC53-like		KOG4496	124	92	99	215	227	7	14	13	106	83	118	256	104	97	15	10
ACC_02931	conserved hypothetical protein	K11307	KOG2747	699	553	451	640	254	140	227	273	783	676	276	253	394	522	704	371
ACC_02932	NF-kappa-B inhibitor cactus		KOG4177	144	92	109	246	112	24	30	35	1031	725	142	976	128	90	44	13
ACC_02933	phosphatidylinositide phosphatase SAC1 isoform 2		KOG1889	238	119	132	205	255	15	11	19	511	382	219	405	361	380	23	10
ACC_02934	LOW QUALITY PROTEIN	K12231	KOG0170	891	571	470	640	403	108	166	167	2330	2029	249	463	1063	1013	257	113
ACC_02935	conserved hypothetical protein		KOG2177	341	245	190	368	470	53	166	189	253	142	172	167	181	243	382	181
ACC_02936	protein mothers against dpp-like	K04676	KOG3701	194	138	108	260	172	43	73	124	431	299	141	143	270	235	163	60
ACC_02937	integrator complex subunit 8	K13145		230	217	200	231	236	16	20	27	293	281	179	210	388	509	52	23
ACC_02938	conserved hypothetical protein		KOG1029	44	38	41	59	42	7	14	10	46	18	269	40	64	65	182	59
ACC_02939	1-phosphatidylinositol-4,5-bisphosphate phosphod	K05858	KOG1265	1031	935	947	1068	588	292	564	659	881	724	157	90	176	163	320	178
ACC_02940	conserved hypothetical protein		KOG2729	128	88	92	231	254	4	12	20	139	94	102	281	277	323	18	5
ACC_02941	conserved hypothetical protein		KOG4264	643	474	493	504	242	129	228	279	869	655	275	354	829	688	698	663
ACC_02942	mitochondrial import receptor subunit TOM40 homolog 1-lil		KOG3296	4	2	4	13	7	1	1	1	10	0	1	1	2	2	1	0
ACC_02943	conserved hypothetical protein			6	13	15	46	31	2	4	1	14	22	77	61	23	40	16	9
ACC_02944	conserved hypothetical protein		KOG3900	489	172	210	740	227	15	17	60	312	130	8	8	40	82	64	81
ACC_02945	conserved hypothetical protein			77	75	106	85	41	1	8	10	30	13	4	0	2	2	0	0
ACC_02946	39S ribosomal protein L4, mitochondrial	K02926	KOG1624	356	186	262	381	401	16	27	51	484	238	219	607	690	1345	84	59
ACC_02947	transmembrane and TPR repeat-containing protein CG4341-		KOG4626	198	33	46	353	48	6	15	23	54	112	3	11	2	4	20	18
ACC_02948	Down syndrome cell adhesion molecule-like protein 1		KOG3513	575	269	432	331	70	9	18	26	214	192	25	23	14	11	47	73
ACC_02949	tRNA (cytosine-5-)-methyltransferase CG6133-like	K15335	KOG2198	254	193	205	404	377	28	48	56	311	245	136	210	846	1269	248	163
ACC_02950	slit homolog 2 protein-like		KOG4194	47	28	24	74	26	11	11	8	144	83	56	92	162	253	154	82
ACC_02951	phosphopantothenate--cysteine ligase	K01922	KOG2728	243	215	238	459	435	28	52	53	389	238	233	613	834	1025	76	45
ACC_02952	proclotting enzyme-like		KOG3627	53	14	23	36	18	1	5	8	62	38	49	37	63	174	38	15
ACC_02953	SAFB-like transcription modulator-like		KOG4661	1368	1258	1171	1622	914	475	721	890	2191	993	598	295	925	1562	4022	3512
ACC_02954	peptidase C1-like protein F26E4.3-like		KOG1544	162	75	80	205	87	42	43	42	707	170	47	128	141	76	25	21
ACC_02955	probable phenylalanyl-tRNA synthetase, mitochonc	K01889	KOG2783	142	68	117	85	136	6	7	6	186	120	113	311	448	563	27	11
ACC_02956	oxidative stress-induced growth inhibitor 1-like			199	159	148	330	249	45	44	58	973	1102	600	1731	744	440	76	48
ACC_02957	protein-L-isoaspartate(D-aspartate) O-methyltrans	K00573	KOG1661	342	144	163	332	182	10	10	35	175	88	87	172	100	107	35	13
ACC_02958	l-2-hydroxyglutarate dehydrogenase, mitochondria	K00109	KOG2665	360	240	238	695	465	63	88	101	363	257	194	397	375	385	94	19
ACC_02959	ubiquitin-conjugating enzyme E2 C	K06688	KOG0421	136	99	87	178	133	11	12	13	141	87	154	361	253	324	33	13
ACC_02960	probable ATP-dependent RNA helicase DDX28-like		KOG0330	204	165	137	229	310	8	50	36	246	140	230	282	382	785	154	85
ACC_02961	equilibrative nucleoside transporter 3	K15014	KOG1479	278	276	259	193	226	28	53	53	305	161	113	321	470	427	25	13

ACC_02962	probable S-acyltransferase At2g14255-like		KOG0509	1815	1044	1088	2185	1698	102	156	234	1020	494	454	707	65	188	31	13
ACC_02963	peptidyl-prolyl cis-trans isomerase FKBP6-like		KOG0543	173	110	101	223	235	49	68	97	309	162	303	243	298	335	106	62
ACC_02964	zinc finger protein 629-like		KOG2462	224	157	137	226	261	20	55	50	209	206	181	185	242	231	69	36
ACC_02965	ADP-ribosylation factor-like protein 6-like isoform 1	K07951	KOG0070	15	17	21	53	43	1	0	5	25	15	79	202	32	20	4	0
ACC_02966	ribonuclease Oy-like	K01166	KOG1642	200	172	130	211	214	39	80	65	221	123	176	407	280	383	71	45
ACC_02967	ATP-binding cassette sub-family G member 4-like		KOG0061	229	122	83	164	153	70	119	121	1432	1535	1103	2810	1395	513	167	86
ACC_02968	ATP-binding cassette sub-family G member 4-like		KOG0061	502	221	275	554	451	54	79	84	1043	965	258	617	469	552	155	39
ACC_02969	cubilin-like		KOG4292	1117	550	483	776	395	85	181	173	677	504	258	216	416	507	736	437
ACC_02970	brain protein 44-like protein-like isoform 1		KOG1590	89	82	80	244	203	20	23	35	121	73	241	335	189	290	142	196
ACC_02971	Serine/threonine-protein kinase LATS1	K08791	KOG0608	385	415	419	291	140	29	55	55	758	761	140	221	442	391	99	32
ACC_02972	conserved hypothetical protein	K11411	KOG2684	1449	861	753	1500	1397	347	699	810	1076	1011	652	406	338	516	1200	603
ACC_02973	transmembrane protein 181-like			366	210	187	524	576	19	22	27	202	103	93	115	15	33	5	3
ACC_02974	pre-mRNA-processing factor 17	K12816	KOG0282	249	172	200	360	292	36	38	72	460	255	224	403	354	530	76	50
ACC_02975	mediator of RNA polymerase II transcription subun	K15146	KOG4552	85	67	56	60	59	8	5	11	177	126	39	192	170	189	25	11
ACC_02976	sister chromatid cohesion protein DCC1-like	K11271	KOG0798	168	118	84	97	106	9	24	26	210	124	62	159	243	306	49	28
ACC_02977	conserved hypothetical protein	K09062	KOG3119	9	8	4	5	2	1	4	2	14	10	1	5	3	3	0	0
ACC_02978	hypothetical protein			2	0	1	1	1	1	0	0	20	4	6	3	0	0	0	0
ACC_02979	heme oxygenase	K00510	KOG4480	220	170	147	258	304	10	44	50	300	136	179	585	221	182	27	14
ACC_02980	phospholipid scramblase 2-like		KOG0621	106	66	75	180	165	2	7	8	53	32	21	53	138	139	22	2
ACC_02981	ribonucleoside-diphosphate reductase subunit M2	K10808	KOG1567	200	148	129	336	368	25	26	25	338	274	222	507	882	2364	172	44
ACC_02982	conserved hypothetical protein		KOG2886	112	77	76	149	110	3	6	7	282	153	19	90	153	168	29	16
ACC_02983	PCNA-associated factor-like			2	0	2	2	1	0	0	0	1	1	2	3	13	16	4	5
ACC_02984	conserved hypothetical protein	K01900	KOG2799	1720	732	869	1002	1270	55	56	90	3265	1597	317	2906	1533	1107	90	24
ACC_02985	short-chain dehydrogenase/reductase		KOG1205	26	11	14	40	17	3	4	14	173	16	8	1	116008	8347	82	1
ACC_02986	TPR Domain containing protein			244	121	115	212	230	62	104	124	480	73	23	43	6	7	2	3
ACC_02987	Thyroid hormone receptor-associated protein complex, subu		KOG3598	687	507	599	265	167	32	61	60	303	209	46	48	90	192	121	195
ACC_02988	choline/ethanolaminephosphotransferase 1-like	K13644	KOG2877	277	120	114	226	228	11	21	22	681	527	129	597	665	621	43	10
ACC_02989	ATP-dependent DNA helicase Q5-like		KOG0352	85	64	35	71	112	3	12	11	43	42	40	37	91	109	61	17
ACC_02990	UPF0708 protein C6orf162 homolog			28	17	20	28	25	0	1	2	1	12	11	42	54	33	3	2
ACC_02991	phosphoglucomutase-like	K01835	KOG0625	178	99	79	155	129	21	33	38	884	1134	128	757	2428	988	97	47
ACC_02992	transcription initiation factor TFIID subunit 8-like	K14649	KOG4336	422	436	426	694	467	65	95	115	421	169	221	417	332	484	146	91
ACC_02993	conserved hypothetical protein		KOG1040	876	474	456	667	341	153	287	301	1536	841	540	241	655	722	597	357
ACC_02994	tRNA-dihydrouridine(20a/20b) synthase	K05545	KOG2335	141	68	85	120	118	3	11	12	96	65	76	188	99	105	14	6
ACC_02995	conserved hypothetical protein		KOG3620	230	184	214	302	215	46	72	102	193	97	153	213	143	380	1633	623
ACC_02996	U2 small nuclear ribonucleoprotein B''-like		KOG4206	84	99	109	193	117	19	11	18	87	73	106	254	298	687	221	76
ACC_02997	28S ribosomal protein S18c, mitochondrial-like	K02963	KOG3162	135	111	112	218	203	14	24	23	95	64	109	287	324	499	42	12
ACC_02998	prestin-like	K14453	KOG0236	448	273	285	518	634	10	19	20	410	250	138	580	239	218	17	12
ACC_02999	conserved hypothetical protein		KOG2462	656	417	477	662	594	78	130	200	738	566	342	534	498	499	250	170
ACC_03000	LOW QUALITY PROTEIN		KOG2058	81	67	56	111	192	6	11	10	167	72	110	274	207	251	14	5
ACC_03001	putative mitochondrial inner membrane protein-like		KOG1854	2245	1120	1035	2021	855	320	601	715	3317	1302	1198	2096	3008	3545	2578	1369
ACC_03002	attractin-like protein 1-like		KOG1388	159	92	83	88	52	22	18	48	345	314	89	104	178	95	115	87
ACC_03003	conserved hypothetical protein	K14035	KOG4215	342	183	195	309	282	20	29	36	498	369	85	271	299	180	37	15
ACC_03004	conserved hypothetical protein		KOG1264	388	313	359	385	185	44	85	83	298	230	104	211	224	216	205	114
ACC_03005	RRP12-like protein-like	K14794	KOG1248	581	329	292	451	404	134	306	319	489	437	306	195	1352	2098	2357	1468
ACC_03006	hypothetical protein			974	443	432	1994	1092	331	928	700	444	239	609	119	63	96	763	91
ACC_03007	conserved hypothetical protein	K03662	KOG3868	1828	1349	1545	1262	1356	66	101	133	1542	1246	658	2795	2892	2063	77	29
ACC_03008	probable sulfite oxidase, mitochondrial-like	K00387	KOG0535	483	294	349	516	458	51	84	81	709	291	303	302	472	542	92	42
ACC_03009	conserved hypothetical protein			23	26	20	23	33	4	20	13	35	8	26	18	10	6	13	7
ACC_03010	polyadenylate-binding protein-interacting protein 2			442	382	261	280	229	50	34	56	754	615	190	755	727	560	64	3
ACC_03011	cytosolic purine 5'-nucleotidase-like	K01081	KOG2469	567	363	457	584	563	42	54	51	2129	1920	178	765	1928	1000	130	50
ACC_03012	rhomboid domain-containing protein 1-like	K09651	KOG2632	83	92	84	72	70	6	20	8	91	88	63	229	98	87	6	3
ACC_03013	conserved hypothetical protein	K15141	KOG1883	314	183	169	330	189	33	58	80	369	239	126	239	290	296	163	73
ACC_03014	conserved hypothetical protein			250	231	236	406	242	39	38	46	189	118	126	66	84	102	24	9
ACC_03015	LOW QUALITY PROTEIN		KOG4370	1285	996	765	1098	1125	329	716	851	1085	729	737	809	1658	2102	3567	2925
ACC_03016	serine protease snake-like		KOG3627	37	55	58	168	128	60	33	45	4711	3066	70	391	33	10	1	4
ACC_03017	protein phosphatase 1 regulatory subunit 3C-B-like	K07189	KOG3986	11	6	8	18	4	4	2	7	220	180	4	11	62	41	20	3
ACC_03018	tankyrase-1 isoform 2	K10799	KOG4177	107	63	58	38	35	7	6	9	305	253	21	115	187	124	31	16

ACC_03019	conserved hypothetical protein			0	0	0	0	0	0	2	0	7	4	18	8	0	0	0	0
ACC_03020	vacuolar protein sorting-associated protein 13B		KOG1809	539	345	297	430	231	45	77	83	653	896	169	277	555	478	145	56
ACC_03021	conserved hypothetical protein			10	9	5	16	4	0	3	0	23	5	15	8	6	1	0	1
ACC_03022	cytochrome P450 4c3		KOG0157	100	6	11	32	34	1	3	3	1108	473	13	38	110	163	33	31
ACC_03023	hypothetical protein			1	1	0	0	1	0	0	0	1	0	0	0	0	0	0	0
ACC_03024	conserved hypothetical protein			55	36	34	25	25	7	11	9	159	151	51	182	16	10	8	3
ACC_03025	solute carrier family 12 member 6	K14427	KOG2082	6456	2064	1979	2921	1120	386	446	673	2850	1024	488	234	226	547	672	175
ACC_03026	40S ribosomal protein S8-like	K02995	KOG3283	2181	1517	927	4933	2339	589	1053	1418	2798	1653	4284	4131	11626	14930	5066	2548
ACC_03027	epimerase family protein SDR39U1-like	K07071	KOG3019	128	79	89	183	187	15	15	21	329	118	104	276	214	251	24	4
ACC_03028	conserved hypothetical protein		KOG2377	668	352	402	471	482	36	90	88	676	389	307	615	570	541	31	18
ACC_03029	enhancer of split mgamma protein-like	K09090	KOG4304	42	30	47	66	31	3	4	3	30	52	4	20	32	55	53	8
ACC_03030	eukaryotic translation initiation factor 5 isoform 1	K03262	KOG2767	1612	1296	1132	2613	1377	411	611	909	4047	2257	581	960	1785	1731	1105	339
ACC_03031	conserved hypothetical protein			443	179	248	377	305	11	7	13	245	72	72	204	3	14	3	1
ACC_03032	v-type proton ATPase subunit D 1-like isoform 1	K02149	KOG1647	1253	646	708	1084	870	106	147	204	1669	795	516	2424	1920	1541	155	125
ACC_03033	alkylated DNA repair protein alkB homolog 1	K10765	KOG2731	219	194	171	326	351	10	21	20	299	206	180	652	661	595	63	19
ACC_03034	protein transport protein Sec23A-like isoform 1	K14006	KOG1986	515	435	317	725	502	55	68	60	947	1008	236	694	2308	2766	319	93
ACC_03035	2-hydroxyacylsphingosine 1-beta-galactosyltransfe	K02727	KOG0184	377	234	213	544	350	47	77	109	767	418	856	1054	884	1479	375	235
ACC_03036	vam6/Vps39-like protein-like isoform 1		KOG2063	344	223	213	365	350	25	37	55	403	255	114	414	386	294	56	10
ACC_03037	COP9 signalosome complex subunit 3	K12177	KOG2582	286	192	166	388	325	48	90	113	416	333	274	546	648	750	240	97
ACC_03038	PKHD domain-containing transmembrane protein C17orf101 homolog			517	341	343	333	467	82	217	277	325	247	554	533	407	643	177	206
ACC_03039	conserved hypothetical protein			39	28	34	41	38	2	4	5	56	19	24	42	51	36	4	1
ACC_03040	ubiquitin domain-containing protein UBFD1-like		KOG1872	147	79	81	231	104	11	15	8	342	312	134	303	364	313	35	19
ACC_03041	probable beta-hexosaminidase fdl-like		KOG2499	76	37	22	77	10	2	1	3	54	107	73	60	29	27	127	30
ACC_03042	conserved hypothetical protein			305	230	160	514	429	52	114	135	212	180	193	143	349	484	355	154
ACC_03043	conserved hypothetical protein			1448	1061	1131	1451	1216	67	135	192	665	449	323	541	129	79	39	23
ACC_03044	neuralized-like protein 2-like isoform 1	K16782	KOG4625	109	73	53	193	129	3	8	10	128	67	51	139	122	127	23	3
ACC_03045	endothelin-converting enzyme 1-like		KOG3624	77	48	56	100	84	36	42	31	458	346	237	556	90	145	52	38
ACC_03046	histidine triad nucleotide-binding protein 3-like		KOG4359	98	59	47	51	71	8	8	8	113	72	41	270	261	156	9	5
ACC_03047	coatomer subunit zeta-1-like isoform 2		KOG3343	165	184	153	412	304	10	35	41	367	186	228	477	561	643	61	36
ACC_03048	luc7-like protein 3-like		KOG0796	767	622	609	646	412	112	165	213	623	415	256	162	394	447	522	383
ACC_03049	conserved hypothetical protein		KOG2361	110	64	26	100	88	26	29	30	81	56	38	38	115	295	125	73
ACC_03050	conserved hypothetical protein			40	7	28	174	151	2	6	11	2	3	0	2	8	72	14	8
ACC_03051	conserved hypothetical protein			27	5	0	32	7	2	2	5	17	4	156	9	0	1	9	11
ACC_03052	sodium/hydrogen exchanger 3	K12040	KOG1966	921	847	1166	748	287	91	106	150	842	536	174	58	68	45	64	34
ACC_03053	polyamine-modulated factor 1-like isoform 1			102	84	89	155	162	10	17	29	65	40	67	71	112	232	168	84
ACC_03054	ABC transporter A family member 1-like		KOG0059	1000	721	652	1324	1081	163	340	327	1353	1129	1276	830	632	405	122	32
ACC_03055	g1/S-specific cyclin-E	K06626	KOG0655	57	39	32	65	30	2	1	5	46	19	14	10	87	196	36	15
ACC_03056	putative ATP-dependent Clp protease proteolytic s	K01358	KOG0840	208	140	165	210	213	29	46	56	351	187	217	425	354	394	62	29
ACC_03057	LOW QUALITY PROTEIN			355	117	102	95	75	24	54	25	143	49	115	19	8	27	55	11
ACC_03058	solute carrier organic anion transporter family member 3A1-		KOG3626	537	109	80	219	48	15	17	31	273	199	25	29	96	74	146	92
ACC_03059	pre-mRNA branch site p14-like protein-like	K12833	KOG0114	277	141	125	380	277	22	60	71	174	86	179	262	664	779	96	55
ACC_03060	protein artemis-like isoform 1	K10887	KOG1361	113	51	63	108	131	5	19	17	137	59	101	115	190	217	24	18
ACC_03061	LOW QUALITY PROTEIN	K09680	KOG2201	257	143	141	178	208	29	47	46	379	343	131	323	993	515	89	56
ACC_03062	alpha-1,2-mannosyltransferase ALG9-like	K03846	KOG2515	357	295	203	413	450	20	33	54	223	283	153	335	455	353	56	19
ACC_03063	conserved hypothetical protein			565	261	235	263	322	29	49	45	586	398	230	383	401	223	44	24
ACC_03064	probable phospholipid-transporting ATPase IF		KOG0206	20	2	1	5	2	2	4	3	22	386	0	2	18	11	1	1
ACC_03065	lipopolysaccharide-induced tumor necrosis factor-alpha factor homolog			105	88	68	87	43	6	7	7	135	104	20	88	87	73	28	12
ACC_03066	putative ATP-dependent RNA helicase DHX30-like		KOG0920	804	414	475	603	858	33	63	87	988	972	504	1004	3141	2755	255	72
ACC_03067	conserved hypothetical protein		KOG2086	4	0	0	10	4	1	3	2	17	3	25	93	4	4	1	2
ACC_03068	LOW QUALITY PROTEIN	K14616	KOG1217	22	7	11	16	5	6	1	6	125	65	13	2	401	147	93	24
ACC_03069	cytohesin-1-like isoform 1		KOG0930	135	68	75	160	79	8	15	25	302	265	29	141	69	50	20	5
ACC_03070	cytohesin-1-like isoform 2			27	6	9	26	6	7	2	3	14	18	11	27	1	1	3	1
ACC_03071	neprilysin-1-like		KOG3624	520	530	713	1266	493	74	86	97	874	348	26	3	44	60	58	57
ACC_03072	conserved hypothetical protein		KOG2052	75	15	26	57	22	32	33	39	128	99	57	104	252	106	91	69
ACC_03073	splicing factor 3B subunit 4-like	K12831	KOG0131	253	251	234	332	97	12	41	49	211	124	74	106	331	556	662	535
ACC_03074	biogenesis of lysosome-related organelles complex 1 subuni		KOG3390	83	48	65	177	147	4	17	11	54	33	117	135	52	68	57	19
ACC_03075	RNA-binding protein squid-like isoform 1	K03102	KOG4205	2099	1172	1063	679	448	196	273	270	2144	1511	484	1053	3339	5837	4270	1794

ACC_03076	conserved hypothetical protein			0	1	0	9	0	0	0	0	0	4	0	0	0	0	2	0
ACC_03077	NECAP-like protein CG9132-like		KOG2500	401	237	236	356	209	25	54	68	345	373	155	544	214	132	82	61
ACC_03078	5-formyltetrahydrofolate cyclo-ligase-like	K01934	KOG3093	93	99	68	59	98	10	20	22	567	279	197	545	1249	836	39	23
ACC_03079	UDP-glucuronosyltransferase 1-10-like		KOG1192	62	57	69	332	213	10	11	13	595	171	1435	1665	778	198	56	14
ACC_03080	UDP-glucuronosyltransferase 1-10-like	K00699	KOG1192	5	0	4	7	3	2	1	2	170	100	5292	8374	333	542	155	29
ACC_03081	conserved hypothetical protein			216	147	124	459	195	35	38	36	1382	1840	219	450	1532	808	256	23
ACC_03082	transmembrane protein 19-like		KOG4491	94	62	76	189	183	7	10	15	197	98	105	148	236	231	26	8
ACC_03083	conserved hypothetical protein	K16518	KOG2279	120	86	75	105	84	1	14	12	240	311	38	122	919	747	42	19
ACC_03084	conserved hypothetical protein	K12605		1016	546	530	745	940	109	196	295	1099	967	521	1342	943	708	116	33
ACC_03085	conserved hypothetical protein		KOG4701	80	18	20	48	18	3	4	9	75	158	8	329	998	573	142	97
ACC_03086	28S ribosomal protein S22, mitochondrial		KOG3890	475	322	325	694	492	45	85	99	611	434	313	608	1166	2095	499	296
ACC_03087	derlin-2-like	K13989	KOG0858	113	62	50	104	93	10	8	17	313	285	65	398	386	317	22	2
ACC_03088	putative ribosomal RNA methyltransferase NOP2-li	K14835	KOG1122	140	85	106	240	211	20	28	32	154	128	133	100	530	916	331	171
ACC_03089	putative ribosomal RNA methyltransferase NOP2-like		KOG0926	728	343	283	208	347	69	150	132	179	172	187	186	823	1305	1546	1020
ACC_03090	n-acetylglucosaminyl-phosphatidylinositol biosynth	K03857	KOG1111	88	77	66	96	127	13	9	9	130	99	84	98	119	126	6	0
ACC_03091	guanine nucleotide-binding protein subunit beta-2-	K07972	KOG0286	124	105	107	374	287	397	447	682	1810	791	47	79	21	40	10	2
ACC_03092	hemicentin-1-like		KOG3513	88	146	197	760	670	11	13	24	142	51	4	1	13	12	2	1
ACC_03093	contactin-5-like		KOG3513	326	113	102	581	251	19	36	37	174	43	8	4	23	45	9	5
ACC_03094	vacuolar protein sorting-associated protein 37A	K12185	KOG3270	140	94	88	174	128	26	14	39	191	171	76	250	206	175	37	13
ACC_03095	conserved hypothetical protein			202	143	128	129	44	8	7	10	303	506	39	91	314	94	47	17
ACC_03096	zinc finger protein 227-like		KOG2462	187	149	139	209	113	24	29	44	441	226	82	192	233	195	96	33
ACC_03097	POU domain, class 2, transcription factor 3-like	K09364	KOG3802	7	3	1	4	0	2	1	1	8	2	3	12	7	15	18	1
ACC_03098	probable signal peptidase complex subunit 2-like	K12947	KOG4072	378	287	182	376	489	108	74	106	396	280	260	1147	1644	1148	33	12
ACC_03099	conserved hypothetical protein			629	283	388	1096	1065	19	31	35	373	274	710	5269	481	347	44	6
ACC_03100	arginase-2, mitochondrial-like	K01476	KOG2965	83	39	80	135	162	12	23	10	117	562	52	15	4478	1902	75	57
ACC_03101	fumarate hydratase, mitochondrial-like	K01679	KOG1317	777	294	280	800	801	55	43	59	1376	771	491	1966	2401	2334	128	51
ACC_03102	conserved hypothetical protein			54	46	46	63	58	3	4	6	54	24	18	43	66	129	10	3
ACC_03103	meiotically up-regulated gene 71 protein-like isoform 1		KOG2316	231	155	118	187	159	19	36	23	483	383	95	209	401	336	64	14
ACC_03104	Golgi SNAP receptor complex member 2	K08496	KOG3251	136	116	124	173	155	22	46	41	161	79	65	234	277	303	55	59
ACC_03105	pinin-like	K13114	KOG3756	498	376	370	465	338	92	160	157	550	323	310	380	664	856	699	727
ACC_03106	glyoxalase domain-containing protein 4-like		KOG2943	241	197	195	446	478	68	169	200	1103	518	753	1386	860	610	66	67
ACC_03107	f-box/LRR-repeat protein 2-like	K10275	KOG1947	92	43	51	33	47	1	4	8	160	119	55	127	105	62	3	1
ACC_03108	conserved hypothetical protein			35	27	20	37	32	1	2	1	60	34	4	12	8	8	3	0
ACC_03109	conserved hypothetical protein			239	113	122	139	201	7	21	30	86	119	92	143	230	211	31	11
ACC_03110	cell growth regulator with RING finger domain protein 1-like		KOG4265	339	186	185	357	188	25	34	50	670	373	139	369	657	189	64	20
ACC_03111	conserved hypothetical protein			186	162	95	174	154	51	55	73	275	108	257	874	248	290	44	7
ACC_03112	conserved hypothetical protein			118	52	47	95	73	8	15	20	234	82	123	712	170	179	24	8
ACC_03113	conserved hypothetical protein			58	62	54	111	112	6	15	16	87	66	54	123	295	329	57	41
ACC_03114	vascular endothelial growth factor A-A-like			6	5	5	12	7	0	0	0	12	14	50	27	20	17	3	2
ACC_03115	LOW QUALITY PROTEIN	K09291	KOG4674	2507	1637	1486	2289	1575	518	846	982	2182	2026	1316	766	2059	2662	2994	1873
ACC_03116	protein virilizer		KOG4822	668	410	419	496	337	58	77	86	853	767	228	241	912	1222	200	45
ACC_03117	cleavage and polyadenylation specificity factor sub	K14404	KOG1040	40	34	29	87	71	3	7	10	30	27	51	61	55	142	31	6
ACC_03118	conserved hypothetical protein		KOG3971	1206	684	816	1846	643	230	429	493	694	422	409	196	127	190	761	473
ACC_03119	conserved hypothetical protein	K16314	KOG0201	1136	825	1048	2056	449	217	263	444	1731	903	343	259	176	308	645	245
ACC_03120	hypothetical protein			72	47	100	55	18	4	6	9	57	26	6	5	0	6	12	9
ACC_03121	conserved hypothetical protein	K16608	KOG2157	30	20	23	19	8	1	2	3	37	133	26	44	87	101	17	3
ACC_03122	J domain-containing protein-like isoform 2	K09532	KOG0691	710	390	548	1811	528	47	57	116	648	396	173	190	23	24	63	38
ACC_03123	high-affinity choline transporter 1-like		KOG3761	848	798	1498	3165	833	90	109	152	714	742	447	2310	47	99	186	93
ACC_03124	lachesin-like		KOG3513	72	29	28	36	18	1	4	5	45	45	2	10	54	60	49	18
ACC_03125	ras GTPase-activating protein 1-like isoform 1	K04352	KOG3508	679	353	363	1357	786	96	198	229	726	516	310	308	546	665	654	337
ACC_03126	conserved hypothetical protein			135	92	93	102	87	19	20	21	234	209	64	466	301	281	68	8
ACC_03127	UPF0614 protein C14orf102-like		KOG1972	857	521	406	632	929	54	124	124	596	596	562	888	663	533	338	175
ACC_03128	conserved hypothetical protein			142	85	110	129	138	4	11	7	53	36	43	114	71	62	14	4
ACC_03129	d-beta-hydroxybutyrate dehydrogenase, mitochondrial-like		KOG1610	243	281	301	245	199	21	36	69	349	207	160	125	219	129	20	22
ACC_03130	SH2B adapter protein 1-like		KOG0197	124	101	77	161	93	11	6	9	146	152	46	96	160	137	42	8
ACC_03131	xaa-Pro aminopeptidase 1-like	K01262	KOG2413	270	228	186	423	378	29	69	76	361	322	252	571	1834	1907	156	58
ACC_03132	conserved hypothetical protein			86	82	120	190	197	3	14	20	52	39	63	193	7	10	4	0

ACC_03133	ran-specific GTPase-activating protein-like	K15306	KOG0864	305	202	177	288	310	53	96	95	195	159	226	549	600	752	319	205
ACC_03134	short-chain specific acyl-CoA dehydrogenase, mitoc	K00248	KOG0139	194	133	148	300	268	18	19	31	379	248	154	352	352	468	47	22
ACC_03135	LOW QUALITY PROTEIN	K12171	KOG4225	569	516	515	414	239	51	71	73	932	485	202	100	361	447	202	101
ACC_03136	LOW QUALITY PROTEIN	K15101	KOG0763	120	85	101	167	153	15	39	42	171	106	140	220	380	408	66	36
ACC_03137	S-phase kinase-associated protein 1-like	K03094	KOG1724	582	244	223	587	568	48	44	74	1218	559	342	1372	851	895	100	51
ACC_03138	conserved hypothetical protein	K15593	KOG3806	119	95	77	193	94	27	36	36	1586	536	231	255	186	118	51	90
ACC_03139	colorectal mutant cancer protein-like isoform 1		KOG0161	415	159	187	318	166	24	21	42	436	125	103	138	41	83	62	25
ACC_03140	serine protease snake-like		KOG3627	103	67	76	74	96	7	13	13	290	305	105	175	169	148	12	4
ACC_03141	probable 28S ribosomal protein S26, mitochondrial		KOG4691	467	207	180	222	392	30	107	108	147	127	302	494	380	520	260	313
ACC_03142	membrane-associated progesterone receptor component 2-		KOG1110	783	553	346	590	455	123	286	301	2591	2109	427	3689	2633	1798	302	240
ACC_03143	conserved hypothetical protein	K04498	KOG1778	979	991	915	764	197	131	159	238	1513	1790	224	208	653	928	1223	414
ACC_03144	conserved hypothetical protein		KOG1090	209	197	203	270	140	17	18	22	404	272	101	189	172	188	74	22
ACC_03145	conserved hypothetical protein		KOG1015	911	542	514	796	338	185	223	419	1365	818	154	313	190	328	356	161
ACC_03146	OTU domain-containing protein 7B-like	K11860	KOG4345	279	161	153	244	185	28	33	41	961	614	168	659	581	476	121	18
ACC_03147	protein JTB		KOG4084	44	42	45	49	51	2	7	13	105	112	24	152	106	98	15	1
ACC_03148	microsomal glutathione S-transferase 1 isoform 1	K00799		307	157	103	225	212	34	46	43	880	382	1260	2174	2542	1508	94	49
ACC_03149	odorant binding protein 1			27	10	14	18	8	151	63	102	1009	452	71292	109843	0	1	59	29
ACC_03150	putative ankyrin repeat protein L93-like		KOG4412	10	7	7	45	26	2	3	3	9	11	13	7	19	28	16	5
ACC_03151	conserved hypothetical protein	K15213	KOG1592	324	173	163	303	299	22	50	55	193	162	101	107	231	454	172	106
ACC_03152	protein phosphatase PP2A 55 kDa regulatory subur	K04354	KOG1354	613	385	331	509	327	73	121	116	866	864	138	366	728	805	560	214
ACC_03153	protein TRC8 homolog	K15703	KOG0802	314	179	168	303	192	42	48	61	564	411	116	261	504	499	93	38
ACC_03154	probable E3 ubiquitin-protein ligase HERC4-like		KOG1427	183	166	175	444	374	32	51	60	257	132	298	198	253	334	91	59
ACC_03155	Predicted ubiquitin-protein ligase/hyperplastic discs protein,		KOG0943	908	525	637	461	271	155	305	268	492	296	226	57	72	111	178	74
ACC_03156	NTF2-related export protein-like	K14285	KOG4353	190	188	198	336	305	31	52	61	210	163	160	263	210	265	75	24
ACC_03157	conserved hypothetical protein		KOG2744	52	55	36	16	9	15	12	11	77	125	3	3	2	4	10	14
ACC_03158	ras-related protein Rab-2-like	K07976	KOG0098	1114	492	489	829	758	77	81	123	1588	905	371	1311	801	784	59	36
ACC_03159	huntingtin-interacting protein 1	K04559	KOG0980	1207	890	767	933	690	219	434	458	1135	767	620	356	1052	1307	579	288
ACC_03160	troponin I isoform 6a2		KOG3977	704	197	29	271	95	574	1310	934	6723	1984	335	332	1041	751	1003	1184
ACC_03161	tetraspanin-11-like	K06537	KOG3882	645	117	117	444	106	6	13	14	295	182	16	200	8	22	61	8
ACC_03162	protein YIPF1-like		KOG3114	405	284	200	547	369	70	100	105	349	284	257	830	620	491	77	31
ACC_03163	conserved hypothetical protein			140	248	33	226	74	87	76	174	2057	3874	6167	970	44	104	21	38
ACC_03164	leucine-rich repeat and immunoglobulin-like domain-contair		KOG4194	208	97	74	93	64	25	16	29	307	251	73	213	258	209	97	13
ACC_03165	peptidyl-prolyl cis-trans isomerase D-like		KOG0546	296	249	240	177	250	16	34	37	253	185	100	214	259	364	53	23
ACC_03166	DNA polymerase subunit gamma-1, mitochondrial-	K02332	KOG3657	346	180	141	285	315	41	123	97	286	235	159	90	264	382	271	119
ACC_03167	conserved hypothetical protein		KOG4786	931	684	530	1388	989	276	593	684	601	477	436	196	690	695	1738	948
ACC_03168	cAMP-responsive element-binding protein-like 2-like			767	394	456	1052	264	197	217	325	2013	1805	1013	5505	183	211	340	59
ACC_03169	kelch-like protein 5	K10442	KOG4441	48	22	17	39	34	5	5	5	75	36	15	58	53	56	5	3
ACC_03170	40S ribosomal protein S3	K02985	KOG3181	3876	2848	1497	3005	2529	845	1231	1472	4494	4337	2888	9104	13374	18145	5473	2327
ACC_03171	conserved hypothetical protein	K14400	KOG2071	1782	1220	1092	1122	767	274	490	482	1978	1140	809	373	899	1206	970	539
ACC_03172	ATP synthase subunit O, mitochondrial	K02137	KOG1662	1246	521	483	1357	1622	144	242	291	2710	1127	809	3795	3921	2738	373	302
ACC_03173	conserved hypothetical protein			585	413	376	672	444	100	258	268	1116	735	314	262	321	241	229	114
ACC_03174	Neuroblastoma-amplified gene protein		KOG1797	480	284	275	403	399	25	103	83	637	599	171	256	607	603	99	28
ACC_03175	Down syndrome cell adhesion molecule-like protein CG4225		KOG3513	488	346	429	921	262	33	52	97	231	199	29	40	4	10	66	78
ACC_03176	6-pyruvoyl tetrahydrobiopterin synthase-like	K01737	KOG4105	101	134	113	130	141	18	59	67	198	82	68	206	313	223	57	42
ACC_03177	RNA pseudouridylate synthase domain-containing protein 4-		KOG1919	208	114	124	174	206	5	37	30	65	120	96	384	531	613	175	86
ACC_03178	peptidyl-alpha-hydroxyglycine alpha-amidating lya:	K00504	KOG3567	433	219	151	358	321	23	33	32	505	279	163	390	545	622	39	15
ACC_03179	alpha-endosulfine-like isoform 1		KOG4076	288	270	205	397	312	20	30	65	394	293	98	554	548	533	99	34
ACC_03180	LOW QUALITY PROTEIN	K13704	KOG1552	284	187	202	392	518	22	44	41	265	181	242	642	475	501	36	10
ACC_03181	conserved hypothetical protein			138	125	109	205	231	9	14	35	138	128	88	161	239	319	37	18
ACC_03182	ryanodine receptor 44F	K04962	KOG2243	2750	1795	1625	2914	1321	293	341	399	18287	8838	227	133	104	104	75	40
ACC_03183	solute carrier family 35 member F1-like isoform 1	K15287	KOG2766	168	38	42	170	84	4	4	4	23	7	2	12	0	1	0	3
ACC_03184	RNA polymerase-associated protein CTR9 homolog	K15176	KOG2002	358	265	233	392	226	102	178	231	858	675	259	361	646	812	769	520
ACC_03185	probable phospholipid-transporting ATPase IA-like	K14802	KOG0206	1258	489	397	842	695	190	201	328	1285	877	475	440	788	543	105	42
ACC_03186	tudor domain-containing protein 3-like		KOG3683	837	502	523	834	623	132	257	284	960	888	289	729	796	878	370	168
ACC_03187	serine/threonine-protein kinase PLK4 isoform 2	K08863	KOG0575	374	255	271	433	346	45	81	79	703	425	295	343	430	465	163	64
ACC_03188	neuronal membrane glycoprotein M6-b-like		KOG4800	86	37	26	60	48	3	6	2	150	87	41	252	132	133	12	3
ACC_03189	mitochondrial fission process protein 1-like		KOG3945	62	75	68	100	117	5	12	13	36	26	28	95	115	136	33	9

ACC_03190	protein sly1 homolog		KOG1301	422	270	225	265	296	25	46	51	629	375	258	653	542	617	38	10
ACC_03191	bicaudal D-related protein homolog isoform 1	K16756	KOG0161	21	32	35	43	6	14	17	21	124	90	22	47	14	22	32	4
ACC_03192	excitatory amino acid transporter 2-like	K05613	KOG3787	2473	406	526	1580	1180	42	73	88	1372	232	26	56	11	76	30	14
ACC_03193	solute carrier family 41 member 2-like	K15122	KOG3788	46	105	86	117	59	61	66	109	611	226	126	70	630	1478	1027	27
ACC_03194	conserved hypothetical protein			8	1	3	7	3	0	0	1	12	4	4	6	30	27	4	0
ACC_03195	conserved hypothetical protein		KOG4577	267	261	260	265	97	33	34	33	283	215	45	62	129	134	91	32
ACC_03196	cell cycle checkpoint protein RAD1-like	K02830	KOG3194	141	61	85	149	204	5	11	11	73	45	49	152	110	143	28	3
ACC_03197	conserved hypothetical protein			14	5	7	13	5	2	3	1	19	7	8	9	6	13	31	11
ACC_03198	RING-box protein 1A-like	K03868	KOG2930	119	63	62	413	193	23	43	59	280	117	211	341	440	506	224	95
ACC_03199	SUMO-activating enzyme subunit 1	K10684	KOG2014	97	76	78	264	188	13	15	22	128	80	101	136	543	1025	159	130
ACC_03200	HSPB1-associated protein 1-like		KOG2132	135	136	133	122	172	8	25	33	157	98	85	201	192	205	21	12
ACC_03201	ADP-ribosylation factor-like protein 2-like isoform 1	K07943	KOG0073	152	112	106	199	178	24	37	45	135	69	164	236	292	338	76	101
ACC_03202	60S ribosomal protein L34 isoform 1	K02915	KOG1790	1181	829	560	2021	739	414	904	1214	750	638	1932	1315	4116	3705	2310	1662
ACC_03203	nucleolar protein 11-like			309	208	193	357	394	15	28	25	535	388	241	581	571	653	46	24
ACC_03204	ATP synthase subunit d, mitochondrial isoform 5	K02138	KOG3366	953	669	684	1427	1893	67	67	109	1731	526	928	2678	1718	1887	113	77
ACC_03205	serine/threonine-protein kinase SMG1-like	K08873	KOG0891	986	661	438	696	488	170	265	293	1260	1503	284	296	815	875	671	279
ACC_03206	conserved hypothetical protein	K06101	KOG4442	1836	1397	997	3044	1740	666	1292	1309	2222	1630	1170	393	650	742	2710	1341
ACC_03207	conserved hypothetical protein			0	0	1	1	0	0	1	0	3	0	1	3	1895	1401	178	9
ACC_03208	conserved hypothetical protein		KOG4221	294	194	179	136	65	43	58	73	510	498	82	306	495	629	145	51
ACC_03209	pre-mRNA-splicing factor ISY1 homolog	K12870	KOG3068	59	53	60	122	67	14	24	17	61	41	38	59	92	120	77	59
ACC_03210	probable cardiolipin synthase-like	K08744	KOG1617	116	110	102	262	203	15	20	21	80	78	102	141	195	220	55	13
ACC_03211	leucine-rich repeats and immunoglobulin-like domains prote		KOG4194	89	102	130	136	174	8	25	25	90	63	67	26	34	32	3	2
ACC_03212	neutral and basic amino acid transport protein rBA	K06519	KOG0471	807	521	489	1462	987	99	92	125	971	614	243	552	755	1056	223	81
ACC_03213	scaffold protein salvador-like	K16686	KOG1891	188	214	269	385	329	31	36	40	191	125	152	169	208	203	50	14
ACC_03214	probable maleylacetoacetate isomerase 2-like	K01800	KOG0868	166	94	90	109	47	10	7	12	423	451	47	335	755	732	77	17
ACC_03215	molybdopterin synthase catalytic subunit-like	K03635	KOG3307	379	291	298	418	446	51	88	75	660	299	385	901	991	970	166	67
ACC_03216	protein chibby homolog 1-like			130	159	100	179	152	74	108	137	244	127	174	479	307	195	100	79
ACC_03217	E3 ubiquitin-protein ligase MARCH6		KOG1609	1308	627	596	987	597	76	89	118	2406	2024	575	1132	1232	966	106	34
ACC_03218	tubulin polyglutamylase TTLL5-like	K16602	KOG2156	146	95	89	65	57	4	9	11	147	99	42	58	142	132	6	5
ACC_03219	conserved hypothetical protein	K15307		438	400	461	480	227	43	104	100	544	257	168	236	264	362	201	85
ACC_03220	deformed epidermal autoregulatory factor 1		KOG3612	240	204	212	394	198	26	34	40	281	272	53	221	246	215	126	33
ACC_03221	protein O-mannosyl-transferase 2-like	K00728	KOG3359	205	139	109	272	339	8	28	23	177	210	125	207	355	314	12	2
ACC_03222	transmembrane protein 8A-like			267	114	120	274	225	21	34	33	328	358	121	1565	483	415	65	20
ACC_03223	conserved hypothetical protein			15	8	16	34	34	0	3	2	10	20	35	66	9	20	2	1
ACC_03224	conserved hypothetical protein			703	541	405	1492	876	213	378	537	755	624	956	1570	1781	2754	4676	5383
ACC_03225	endothelin-converting enzyme 1 isoform 1	K01415	KOG3624	832	510	433	716	271	54	57	62	992	534	154	87	566	235	94	76
ACC_03226	conserved hypothetical protein	K04667	KOG3900	40	10	31	31	4	0	2	4	9	13	18	22	2	6	9	7
ACC_03227	zinc finger protein 395-like			900	457	652	900	241	101	89	157	538	555	148	179	135	145	253	34
ACC_03228	protein spinster-like isoform 1		KOG1330	470	254	252	329	344	24	31	41	1707	1294	262	1683	1398	805	55	8
ACC_03229	small ubiquitin-related modifier-like	K12160	KOG1769	182	173	155	446	277	24	44	53	135	134	175	468	637	919	266	92
ACC_03230	conserved hypothetical protein			1347	886	1030	2213	2018	72	99	158	1885	1916	874	1281	492	477	36	14
ACC_03231	40S ribosomal protein S15Aa-like isoform 3	K02957	KOG1754	1033	1335	863	1784	1000	192	339	309	818	621	1254	1141	4947	5578	1516	777
ACC_03232	tubulin--tyrosine ligase-like protein 12-like	K16609	KOG2155	116	90	78	130	127	16	10	21	138	139	75	129	287	312	35	7
ACC_03233	conserved hypothetical protein			285	173	127	246	210	26	51	43	231	243	79	112	178	156	91	31
ACC_03234	conserved hypothetical protein	K02901	KOG3418	1789	2042	1215	2465	1411	222	365	542	894	829	1489	2029	4190	5758	1662	1425
ACC_03235	OTU domain-containing protein 6B-like		KOG2606	182	86	53	95	111	28	69	66	140	55	66	101	227	351	239	333
ACC_03236	cysteine-rich secretory protein 1-like		KOG3017	153	43	74	156	48	5	2	6	109	48	9	114	2	1	2	1
ACC_03237	BTB/POZ domain-containing protein KCTD3-like isoform 1		KOG2714	216	160	194	206	179	22	44	48	287	178	139	170	269	331	81	41
ACC_03238	f-box only protein 11-like	K10297	KOG1777	565	340	274	395	197	51	60	78	943	972	139	264	294	355	109	26
ACC_03239	protein turtle homolog A-like		KOG3515	1063	615	541	1172	618	34	50	69	689	366	165	275	47	73	22	5
ACC_03240	hypothetical protein			26	20	22	52	46	2	8	9	10	1	7	1	2	5	2	0
ACC_03241	conserved hypothetical protein		KOG3397	142	81	85	178	145	10	23	17	142	86	71	69	138	93	12	4
ACC_03242	alpha-mannosidase 2	K01231	KOG1958	583	359	353	718	568	45	47	62	675	510	263	276	632	680	66	16
ACC_03243	deoxyribonuclease tatD-like	K03424	KOG3020	222	140	176	317	314	33	40	37	377	191	294	359	352	333	25	5
ACC_03244	signal recognition particle 14 kDa protein isoform 2	K03104	KOG1761	73	60	48	132	130	15	9	19	90	29	64	174	149	167	31	21
ACC_03245	hydroxymethylglutaryl-CoA synthase 1	K01641	KOG1393	130	98	93	138	178	4	22	14	463	489	63	178	680	531	31	12
ACC_03246	vacuolar fusion protein MON1 homolog A-like		KOG0997	318	220	260	322	252	32	42	51	380	300	136	293	423	442	89	29

ACC_03247	conserved hypothetical protein	K11485	KOG4323	148	114	83	97	34	10	12	18	143	114	19	37	93	120	105	20
ACC_03248	LOW QUALITY PROTEIN		KOG1577	482	342	362	533	532	35	39	49	331	180	387	988	286	383	50	15
ACC_03249	dihydrolipoyllysine-residue succinyltransferase con	K00658	KOG0559	950	500	451	757	482	68	63	96	1341	776	255	882	1542	2151	290	124
ACC_03250	26S protease regulatory subunit 4-like	K03062	KOG0726	1218	529	490	1374	1169	157	302	317	2063	1338	1316	2916	2919	3779	2432	2358
ACC_03251	coiled-coil domain-containing protein 61-like	K16755		144	134	169	234	248	12	16	20	198	110	241	67	77	80	6	2
ACC_03252	GPI ethanolamine phosphate transferase 1-like	K05285	KOG2124	546	393	454	601	708	20	53	68	412	309	139	264	448	404	53	13
ACC_03253	eukaryotic initiation factor 4A-III-like isoform 1	K13025	KOG0328	561	345	314	877	641	48	61	68	1023	623	581	1412	2000	3358	277	135
ACC_03254	ribosomal RNA small subunit methyltransferase NE	K14568	KOG3073	94	90	94	219	221	9	13	21	229	141	134	376	560	622	48	33
ACC_03255	peregrin-like	K11348	KOG0955	203	149	153	294	161	31	56	72	250	151	152	122	287	269	158	81
ACC_03256	transmembrane protein 179-like			178	106	133	175	171	11	19	18	140	73	85	307	117	109	13	3
ACC_03257	calreticulin isoform 1	K08057	KOG0674	8556	4629	2147	5878	6207	1529	2264	3642	3726	3521	2577	4746	22587	20170	9403	5348
ACC_03258	putative 28S ribosomal protein S5, mitochondrial	K02988	KOG2646	394	205	203	410	453	47	70	97	738	297	382	675	650	1533	208	139
ACC_03259	G-protein coupled receptor 143-like		KOG4193	135	82	93	119	137	14	24	29	75	70	73	91	239	148	21	11
ACC_03260	conserved hypothetical protein	K13199	KOG2945	1403	836	692	1231	547	125	209	264	1665	1916	265	1314	5956	8368	3934	2536
ACC_03261	conserved hypothetical protein			0	0	0	1	0	0	0	0	0	0	1	1	4	59	18	21
ACC_03262	FGGY carbohydrate kinase domain-containing protein-like is		KOG2517	250	158	108	198	165	50	57	64	1558	1507	136	352	4182	1506	213	88
ACC_03263	ATP-binding cassette sub-family F member 2-like	K06185	KOG0927	321	191	158	378	262	22	33	37	678	534	158	601	806	1309	196	131
ACC_03264	ubiquitin-conjugating enzyme E2 J2-like	K04554	KOG0894	166	131	109	138	140	13	23	23	160	171	47	182	134	96	17	6
ACC_03265	cytochrome c oxidase subunit 5A, mitochondrial	K02264	KOG4077	865	364	362	1267	1376	40	62	68	2063	648	875	1974	1734	1624	96	31
ACC_03266	tRNA-dihydrouridine synthase 3-like	K05544	KOG2333	985	570	427	637	626	130	290	317	887	423	464	240	662	1072	571	361
ACC_03267	serine/threonine-protein kinase OSR1-like	K08835	KOG0582	1175	631	564	1001	652	95	112	130	1500	755	389	480	673	818	232	89
ACC_03268	tetratricopeptide repeat protein 25-like		KOG1144	62	28	37	62	18	6	13	6	63	96	13	19	18	27	37	55
ACC_03269	inhibitor of growth protein 4-like	K11346	KOG1973	794	568	567	755	851	138	308	403	332	136	365	370	203	328	539	349
ACC_03270	translation initiation factor eIF-2B subunit gamma-l	K03241	KOG1462	507	261	189	1093	299	49	113	130	363	300	712	587	2032	2462	1263	486
ACC_03271	mpv17-like protein 2-like	K13348	KOG1944	46	26	33	92	81	11	15	13	262	32	31	11	11	28	10	1
ACC_03272	zinc finger protein 729-like		KOG2462	97	61	53	102	80	12	20	27	59	38	21	8	42	31	21	1
ACC_03273	autophagy-related protein 9A		KOG2173	434	258	207	383	281	36	34	60	1089	688	187	567	345	273	43	9
ACC_03274	conserved hypothetical protein			10	7	7	13	9	1	3	4	11	8	2	2	4	4	1	1
ACC_03275	d-3-phosphoglycerate dehydrogenase-like	K00058	KOG0068	306	366	240	404	353	225	335	307	1021	2597	246	552	250	696	341	380
ACC_03276	nucleolar protein 10			37	21	33	81	57	3	5	11	78	152	18	30	81	49	10	2
ACC_03277	sperm-associated antigen 7-like			190	185	184	270	236	53	90	129	255	108	208	240	224	329	158	161
ACC_03278	protein OPI10 homolog		KOG4067	90	69	60	310	183	12	9	17	146	92	85	373	365	514	82	21
ACC_03279	PAXIP1-associated protein 1-like isoform 2	K14973		178	167	205	250	225	15	28	34	137	79	122	255	95	158	37	17
ACC_03280	conserved hypothetical protein		KOG4304	650	629	831	509	138	66	75	69	309	265	65	42	25	36	80	48
ACC_03281	putative ATP synthase subunit f, mitochondrial-like	K02130	KOG4092	1628	1316	1396	2433	1631	232	276	485	2204	652	1136	3128	1860	2301	656	511
ACC_03282	TATA-binding protein-associated factor 172	K15192	KOG0392	597	419	390	440	308	96	104	120	826	709	202	283	698	701	156	42
ACC_03283	vacuolar protein sorting-associated protein 8 homolog isofo		KOG2079	360	238	284	453	351	21	48	52	483	280	186	192	288	251	32	5
ACC_03284	nuclear pore complex protein Nup85-like isoform 1	K14304	KOG2271	162	90	95	131	184	9	14	13	173	149	69	179	401	514	30	9
ACC_03285	hypothetical protein			0	0	2	0	0	0	0	0	3	6	0	0	1	0	0	3
ACC_03286	hypothetical protein			0	0	1	1	1	0	0	0	0	3	0	0	0	1	2	0
ACC_03287	conserved hypothetical protein		KOG3598	34	25	13	22	10	1	5	4	14	32	8	11	7	585	123	31
ACC_03288	ADP-ribosylation factor-like protein 1-like		KOG0070	72	54	56	122	145	6	9	8	66	44	36	81	116	178	33	19
ACC_03289	molybdenum cofactor sulfurase 1-like isoform 1	K15631	KOG2142	237	286	99	80	122	50	125	116	304	326	160	599	678	291	26	11
ACC_03290	ras-related protein Rab-27A-like isoform 1	K07885	KOG0081	108	182	76	120	101	34	45	61	104	123	24	74	278	180	52	11
ACC_03291	protein CREG1		KOG3374	401	347	248	637	444	107	96	148	1576	655	414	1929	1988	935	118	68
ACC_03292	60S ribosomal protein L36-like isoform 1	K02920	KOG3452	1471	1045	546	1437	974	508	847	1025	1507	979	1738	2303	7448	8245	4140	3548
ACC_03293	CDK5RAP1-like protein-like		KOG2492	339	240	224	480	432	59	154	151	676	325	266	445	478	626	215	120
ACC_03294	conserved hypothetical protein			2	2	3	0	4	0	0	2	1	1	1	0	1	0	0	1
ACC_03295	peroxiredoxin-6	K11188	KOG0854	85	61	57	172	152	14	18	29	179	107	276	339	434	497	61	28
ACC_03296	leucine-rich PPR motif-containing protein, mitochondrial-like		KOG4233	149	104	115	137	209	4	4	8	125	68	74	276	199	198	31	16
ACC_03297	protein eyes shut-like		KOG3509	102	86	101	188	148	9	16	20	109	94	541	145	8	36	10	9
ACC_03298	integrin alpha-PS1		KOG3637	551	523	400	434	377	82	130	118	1144	1118	361	646	1768	1432	180	73
ACC_03299	AP-1 complex subunit gamma-1	K12391	KOG1062	664	498	355	448	480	22	45	54	871	1096	173	491	957	703	64	18
ACC_03300	tubulin gamma-1 chain isoform 1	K10389	KOG1374	193	130	126	201	216	9	17	20	239	142	97	229	250	254	21	8
ACC_03301	protein jagunal		KOG4054	97	79	63	102	156	5	4	14	100	73	83	232	198	252	17	6
ACC_03302	ATP synthase subunit gamma, mitochondrial	K02136	KOG1531	1463	393	442	687	853	82	115	124	3355	604	778	2863	3272	3905	267	84
ACC_03303	LOW QUALITY PROTEIN	K03027	KOG1521	192	108	141	364	349	31	25	35	324	120	219	428	322	619	66	44

ACC_03304	TD and POZ domain-containing protein 2-like			111	55	56	190	117	17	27	29	807	438	33	56	228	142	20	5
ACC_03305	glyoxylate reductase/hydroxypyruvate reductase-like		KOG0069	47	32	24	91	63	5	5	4	548	494	73	257	374	303	25	5
ACC_03306	voltage-dependent anion-selective channel	K15040	KOG3126	1540	643	606	988	864	70	77	88	2254	1072	717	2799	6934	7881	466	167
ACC_03307	protein lin-54 homolog		KOG1171	132	140	122	91	46	4	14	16	225	262	48	120	234	273	67	34
ACC_03308	nidogen-2-like	K06826	KOG1214	1210	395	633	2214	504	43	48	48	1531	3912	248	2697	245	346	807	768
ACC_03309	COMM domain-containing protein 7-like			8	7	19	8	18	0	1	0	0	4	8	17	22	13	0	0
ACC_03310	protein preli-like isoform 1		KOG3337	521	256	245	507	503	27	49	46	938	351	331	1074	548	813	76	31
ACC_03311	conserved hypothetical protein			119	100	64	167	139	67	108	108	2963	1146	337	406	128	17	11	12
ACC_03312	glutamate decarboxylase 1 isoform 1		KOG0629	2599	1615	2025	8163	1995	318	352	691	2175	1456	1143	2829	990	724	355	569
ACC_03313	zinc finger protein with KRAB and SCAN domains 5-like		KOG2462	148	130	117	234	175	14	26	32	181	99	90	192	456	670	74	20
ACC_03314	conserved hypothetical protein			182	115	142	187	161	13	45	52	189	111	139	199	308	642	139	58
ACC_03315	SWI/SNF complex subunit SMARCC2-like	K11649	KOG1279	614	535	528	664	325	81	130	170	709	809	238	526	1242	1727	1251	662
ACC_03316	major facilitator superfamily domain-containing protein 10		KOG2615	274	113	136	176	224	8	14	21	159	97	151	369	204	188	14	4
ACC_03317	signal transducer and activator of transcription 5B		KOG3667	576	405	402	534	318	164	291	341	1417	1416	216	511	654	810	566	267
ACC_03318	exonuclease 3'-5' domain-containing protein 2-like		KOG4373	321	240	224	389	356	43	92	93	411	208	180	278	305	293	86	21
ACC_03319	origin recognition complex subunit 1	K02603	KOG1514	364	199	167	365	504	24	76	63	175	99	252	116	133	197	228	93
ACC_03320	conserved hypothetical protein		KOG4113	149	86	84	125	109	7	10	11	60	24	52	71	111	113	31	16
ACC_03321	trypsin-7-like		KOG3627	205	97	16	29	5	29	68	95	54	61	40	4	4	1	4	4
ACC_03322	mimitin, mitochondrial-like		KOG3382	352	412	538	548	544	55	145	137	355	161	536	615	104	233	117	83
ACC_03323	SET and MYND domain-containing protein 5		KOG2084	109	72	60	136	170	6	12	17	150	99	102	210	325	370	23	18
ACC_03324	LOW QUALITY PROTEIN	K16302	KOG2118	633	291	248	585	551	89	249	235	527	481	257	234	646	859	811	299
ACC_03325	LOW QUALITY PROTEIN			263	242	249	543	521	9	29	28	93	90	194	416	199	250	19	11
ACC_03326	long wavelength sensitive opsin 2	K04255	KOG4219	10	65	55	205	206	6	25	8	480	201	0	0	2	1	0	0
ACC_03327	DNA replication complex GINS protein SLD5-like	K10735	KOG3176	31	43	31	55	55	6	11	7	37	40	30	102	103	136	5	8
ACC_03328	methionine--tRNA ligase, mitochondrial-like	K01874	KOG0436	950	617	588	802	770	176	491	462	1012	477	588	661	728	1100	483	362
ACC_03329	probable small nuclear ribonucleoprotein G	K11099	KOG1780	100	62	66	123	177	25	77	84	67	29	80	169	266	381	190	206
ACC_03330	UPF0704 protein C6orf165 homolog			85	63	62	145	140	4	7	25	61	38	41	30	52	67	14	9
ACC_03331	conserved hypothetical protein		KOG0415	32	17	11	13	5	16	22	45	61	78	3	10	2	5	22	8
ACC_03332	probable 28S ribosomal protein S16, mitochondrial	K02959	KOG3419	352	314	409	412	307	44	84	112	399	231	245	652	845	880	222	132
ACC_03333	conserved hypothetical protein			1297	787	815	1192	1573	58	165	178	1148	994	821	2294	5825	9133	768	451
ACC_03334	glutamate-gated chloride channel			511	219	239	448	127	24	46	41	210	181	35	5	1	5	8	4
ACC_03335	DNA-directed RNA polymerases I, II, and III subunit	K03013	KOG3218	173	150	138	219	220	21	34	40	267	104	158	321	386	449	39	30
ACC_03336	hypothetical protein			0	0	0	0	2	0	0	0	0	1	0	1	1	0	0	1
ACC_03337	40S ribosomal protein SA	K02998	KOG0830	1844	938	556	2580	1189	453	590	703	1457	1754	1092	2161	7415	11965	6309	2498
ACC_03338	neurotactin		KOG1516	135	77	64	261	128	7	11	17	99	53	50	100	65	136	100	27
ACC_03339	leucine-rich repeat-containing protein 15-like		KOG4194	347	243	191	173	156	10	32	26	332	405	125	184	249	205	20	3
ACC_03340	LOW QUALITY PROTEIN	K07198	KOG0583	791	427	402	863	742	76	92	129	1555	1100	602	1644	1229	1486	160	57
ACC_03341	adapter molecule Crk-like isoform 2	K04438	KOG4792	200	107	118	161	168	4	6	17	301	325	45	460	236	217	24	4
ACC_03342	E3 ubiquitin-protein ligase UHRF1	K10638	KOG0957	147	98	89	179	122	7	27	27	125	79	34	71	228	405	171	73
ACC_03343	limkain-b1-like		KOG4206	422	342	292	332	262	39	85	78	762	541	169	145	493	534	80	28
ACC_03344	60S ribosomal protein L3-like	K02925	KOG0746	2804	2374	1583	5380	3338	663	853	1156	2933	3044	1920	4050	21417	26349	5635	2448
ACC_03345	LIRP-like			30	21	18	61	42	1	3	6	48	42	18	62	228	166	22	13
ACC_03346	glutamate receptor delta-2 subunit-like		KOG1052	65	20	27	26	32	2	8	6	63	53	151	217	42	48	11	4
ACC_03347	conserved hypothetical protein			80	59	89	155	100	9	5	18	80	27	42	11	42	37	7	4
ACC_03348	esterase A2		KOG1516	85	105	17	229	216	506	562	539	63604	19139	0	2	11	14	1	0
ACC_03349	esterase E4-like, partial		KOG1516	30	24	16	45	29	14	7	7	1406	177	1	0	196	442	61	90
ACC_03350	conserved hypothetical protein		KOG0307	613	627	903	2419	850	60	41	74	3848	1796	189	893	501	267	69	24
ACC_03351	conserved hypothetical protein		KOG2806	501	784	268	274	126	303	319	531	7022	7256	201	2373	1284	1044	109	63
ACC_03352	hypothetical protein			2	2	0	0	2	3	0	1	12	1	1	1	2	4	0	1
ACC_03353	conserved hypothetical protein			14	4	15	29	20	10	10	15	23	8	19	14	2	4	20	12
ACC_03354	conserved hypothetical protein			77	83	8	50	16	29	31	36	294	126	236	1134	22	2	6	1
ACC_03355	conserved hypothetical protein	K09328	KOG0494	31	23	44	134	32	7	2	12	114	45	35	2	8	8	40	9
ACC_03356	sodium-independent sulfate anion transporter-like	K14708	KOG0236	390	250	349	1058	593	20	30	50	572	180	132	199	34	74	35	14
ACC_03357	conserved hypothetical protein			9	23	13	19	13	1	6	5	17	12	171	237	0	1	0	0
ACC_03358	conserved hypothetical protein			8	5	12	20	27	1	0	1	14	25	39	41	0	0	0	0
ACC_03359	odorant receptor 58			1	3	4	6	8	2	0	0	3	2	5	2	0	0	0	0
ACC_03360	e3 ubiquitin-protein ligase RING2-A isoform 1	K10695	KOG0311	306	216	220	466	207	41	68	99	393	241	215	417	265	363	176	43

ACC_03361	conserved hypothetical protein		KOG1659	177	135	113	212	143	13	22	32	191	114	83	164	220	221	26	25
ACC_03362	PPPDE peptidase domain-containing protein 1-like		KOG0324	138	82	92	135	141	16	28	14	263	239	106	208	268	212	49	11
ACC_03363	protein lines isoform 1			65	50	42	68	83	9	4	8	296	147	69	111	153	165	10	2
ACC_03364	protein FAM98A-like		KOG3973	891	545	510	486	316	73	112	193	1053	609	128	315	1130	1065	244	240
ACC_03365	transmembrane channel-like protein 7-like			241	108	111	123	126	3	16	18	190	171	36	67	223	115	19	3
ACC_03366	dihydropyrimidinase-like	K01464	KOG2584	611	212	201	188	67	17	18	15	765	513	102	335	539	246	77	23
ACC_03367	leucine-rich repeat and WD repeat-containing protein KIAA1		KOG3602	84	54	54	51	53	17	23	27	39	42	74	136	21	12	66	18
ACC_03368	conserved hypothetical protein			16	7	10	17	9	1	2	3	12	5	9	0	9	5	3	4
ACC_03369	conserved hypothetical protein	K11318	KOG4140	76	69	58	88	14	8	5	5	93	183	20	29	32	26	88	31
ACC_03370	conserved hypothetical protein		KOG0514	187	123	145	237	112	34	43	46	1054	987	178	592	378	196	170	108
ACC_03371	BET1 homolog	K08504	KOG3385	71	52	51	125	149	10	20	25	98	55	59	160	157	118	13	11
ACC_03372	coatomer subunit alpha isoform 1	K05236	KOG0292	430	301	257	624	599	46	55	94	868	870	241	903	1755	2040	135	39
ACC_03373	DNA-directed RNA polymerases I, II, and III subunit	K03014	KOG3405	260	344	369	394	316	53	63	77	174	101	155	280	236	356	84	73
ACC_03374	bridging integrator 3 homolog		KOG3771	202	123	104	162	155	16	29	40	133	105	76	207	160	169	67	26
ACC_03375	conserved hypothetical protein			87	51	62	98	149	2	13	8	43	70	70	76	142	156	73	39
ACC_03376	mitotic spindle assembly checkpoint protein MAD2B-like		KOG3186	31	29	28	22	39	2	3	1	24	32	46	45	50	50	5	3
ACC_03377	WD repeat-containing protein 67-like		KOG1093	214	181	191	170	180	13	24	22	284	151	123	169	115	115	12	2
ACC_03378	LOW QUALITY PROTEIN		KOG0740	9	7	13	14	12	1	9	5	16	1	28	3	1	8	10	6
ACC_03379	chaperone activity of bc1 complex-like, mitochondı	K08869	KOG1234	284	171	170	290	320	40	78	75	931	562	242	515	552	659	141	63
ACC_03380	gamma-glutamylcyclotransferase-like		KOG4059	162	149	114	247	232	14	15	14	183	86	130	264	382	364	34	12
ACC_03381	60S ribosomal protein L13 isoform 1	K02873	KOG3295	1838	1581	1016	2094	1512	442	682	855	1895	966	2283	3079	7973	9973	3707	2243
ACC_03382	kinesin-like protein KIF23-like	K10402	KOG0247	104	80	86	100	114	12	21	27	99	72	44	18	206	486	221	155
ACC_03383	liprin-alpha-2-like		KOG0249	405	378	453	611	193	102	146	199	796	620	144	113	160	138	281	176
ACC_03384	protein piwi-like	K02156	KOG1042	280	178	188	364	313	33	68	74	257	261	258	252	482	481	154	79
ACC_03385	heterogeneous nuclear ribonucleoprotein L	K13159	KOG1456	569	398	473	694	318	62	183	170	361	212	190	80	120	346	676	489
ACC_03386	probable 28S ribosomal protein S6, mitochondrial	K02990	KOG4708	236	193	205	341	300	19	35	67	142	65	173	384	335	499	79	42
ACC_03387	conserved hypothetical protein		KOG2483	67	110	36	45	7	6	9	5	72	79	10	8	107	195	287	137
ACC_03388	NADH dehydrogenase	K03951	KOG4630	902	751	1060	1232	741	73	102	146	863	227	559	956	384	563	161	84
ACC_03389	putative adenosylhomocysteinase 3-like isoform 2	K01251	KOG1370	518	248	235	485	375	36	36	53	559	450	106	733	635	668	141	33
ACC_03390	rho guanine nucleotide exchange factor 3-like		KOG4305	177	113	103	143	147	20	30	49	239	236	142	220	283	281	116	65
ACC_03391	conserved hypothetical protein		KOG4032	72	58	58	100	106	8	11	15	74	64	35	139	163	223	27	12
ACC_03392	conserved hypothetical protein			14	11	15	25	17	2	4	2	50	15	12	6	89	312	35	19
ACC_03393	bifunctional protein NCOAT-like	K15719	KOG3698	407	206	167	208	139	32	40	51	539	514	118	274	459	321	48	16
ACC_03394	LOW QUALITY PROTEIN	K15172	KOG1999	370	274	225	303	167	79	94	130	726	478	109	254	670	689	147	94
ACC_03395	conserved hypothetical protein			4	4	6	8	5	1	4	4	6	1	2	4	1	2	1	2
ACC_03396	band 4.1-like protein 4A-like			36	31	27	50	33	20	24	33	19	73	18	15	14	31	39	34
ACC_03397	conserved hypothetical protein			525	455	335	817	824	75	129	145	730	335	1195	1074	254	368	67	15
ACC_03398	conserved hypothetical protein	K16716	KOG0161	550	428	372	510	594	98	219	286	194	165	300	96	110	189	525	167
ACC_03399	lysosomal Pro-X carboxypeptidase-like	K01285	KOG2183	274	224	248	293	418	34	67	78	524	447	488	1145	524	253	10	5
ACC_03400	retinol dehydrogenase 11-like		KOG1208	297	195	200	381	394	18	30	39	323	226	115	422	333	367	39	2
ACC_03401	probable E3 ubiquitin-protein ligase TRIP12-like iso	K10590	KOG0168	1230	803	696	861	545	100	120	130	2305	1792	408	542	1509	1445	280	63
ACC_03402	tubulin polyglutamylase TTLL6-like	K16610	KOG2156	67	42	59	61	61	2	8	6	89	39	56	98	93	131	14	6
ACC_03403	conserved hypothetical protein			141	127	114	211	252	9	19	27	246	144	107	299	262	352	38	21
ACC_03404	ras-related protein Rab-1A-like	K07874	KOG0084	812	462	395	870	721	127	132	153	2204	1615	529	2488	1929	2104	199	53
ACC_03405	exocyst complex component 5-like		KOG3745	277	198	213	270	295	15	38	45	322	251	108	318	409	470	47	14
ACC_03406	ubiquitin carboxyl-terminal hydrolase 34-like	K11853	KOG1866	1303	737	600	1264	837	279	528	598	1497	1914	489	439	800	660	1431	647
ACC_03407	transmembrane protein C9orf5-like		KOG2365	738	553	438	672	761	37	58	74	965	702	565	410	655	561	42	9
ACC_03408	translation initiation factor 2	K02519	KOG1145	744	536	543	376	385	155	387	315	1098	548	781	704	1403	1858	766	449
ACC_03409	conserved hypothetical protein		KOG3598	18	11	13	10	2	1	1	4	16	20	1	7	4	4	22	14
ACC_03410	protein SEC13 homolog	K14004	KOG1332	233	181	130	351	239	30	32	45	350	371	224	600	850	1074	135	87
ACC_03411	condensin complex subunit 3-like	K06678	KOG2025	26	35	23	36	33	1	4	6	19	38	22	22	119	166	25	11
ACC_03412	WD repeat-containing protein 92-like		KOG0269	156	102	97	268	258	14	24	29	169	82	130	236	149	213	31	11
ACC_03413	acyl-CoA Delta(11) desaturase-like	K00507	KOG1600	113	88	75	582	383	6	9	25	204	71	107	270	199	200	25	4
ACC_03414	transcriptional activator cubitus interruptus	K16799	KOG2462	48	43	47	28	16	3	3	5	55	40	2	6	80	133	88	24
ACC_03415	2-acylglycerol O-acyltransferase 1-like	K14457	KOG0831	486	167	123	426	210	22	25	42	199	112	29	167	535	573	165	26
ACC_03416	hypothetical protein			1	0	0	0	0	0	0	0	1	0	0	0	1	0	0	3
ACC_03417	60S ribosomal protein L7	K02937	KOG3184	3285	2272	1369	4171	2381	715	1234	1277	4762	3499	3957	6451	12431	18506	4684	2164

ACC_03418	conserved hypothetical protein	K15200	KOG3262	2114	1527	1414	1542	2186	254	574	626	1144	810	602	439	992	1101	2008	1370
ACC_03419	transmembrane and ubiquitin-like domain-containing protei		KOG0010	425	318	302	284	215	29	30	42	956	692	210	1051	728	613	67	17
ACC_03420	cysteine and histidine-rich domain-containing prot(	K16730	KOG1667	351	214	287	663	675	51	53	93	546	254	335	762	846	912	146	139
ACC_03421	LOW QUALITY PROTEIN		KOG2551	146	88	114	163	164	9	16	21	168	105	88	289	142	163	31	6
ACC_03422	conserved hypothetical protein			6	5	8	11	4	0	0	0	22	17	7	0	9	4	2	4
ACC_03423	motile sperm domain-containing protein 1-like		KOG0439	137	99	68	178	129	11	16	14	130	79	75	162	183	175	24	2
ACC_03424	alpha-2-macroglobulin receptor-associated protein-like		KOG3956	533	396	347	620	569	39	59	64	890	556	565	1183	1029	1027	103	47
ACC_03425	33 kDa inner dynein arm light chain, axonemal-like	K10410	KOG4001	18	11	26	34	22	5	7	6	31	3	22	9	10	10	5	2
ACC_03426	conserved hypothetical protein		KOG4186	63	41	53	57	77	6	11	15	114	19	107	58	43	65	9	6
ACC_03427	protogenin-like		KOG4221	397	260	210	315	258	21	44	49	349	405	112	168	198	212	159	32
ACC_03428	activin receptor type-2B-like	K13597	KOG3653	85	53	35	53	40	5	15	15	245	253	26	125	89	74	8	4
ACC_03429	alcohol dehydrogenase class-3-like	K00121	KOG0022	155	110	75	237	224	23	29	36	403	302	282	517	1011	1007	91	44
ACC_03430	28S ribosomal protein S31, mitochondrial			784	371	432	535	739	45	191	176	360	257	505	634	1027	1426	1082	795
ACC_03431	exosome complex exonuclease RRP41-like isoform	K11600	KOG1068	247	126	171	339	246	18	20	23	347	212	178	462	700	838	104	25
ACC_03432	DDB1- and CUL4-associated factor 8-like	K11804	KOG1334	529	295	358	693	614	92	128	165	1045	687	380	461	885	782	220	101
ACC_03433	lipase member H-like			0	0	0	1	1	0	0	0	3	0	48	51	2	0	0	0
ACC_03434	chymotrypsin-2		KOG3627	3	2	1	29	6	0	0	0	5	1	0	0	3245	1831	110	17
ACC_03435	E3 ubiquitin-protein ligase MARCH5-like	K10660	KOG3053	475	413	328	581	423	68	59	93	718	699	467	1330	760	704	106	20
ACC_03436	conserved hypothetical protein			4	5	4	3	7	0	1	1	6	10	0	4	2	3	0	0
ACC_03437	conserved hypothetical protein		KOG2240	472	360	288	558	473	48	64	85	516	511	248	587	1960	2843	669	267
ACC_03438	apolipoprotein D-like		KOG4824	60	13	17	119	54	6	4	11	403	337	121	201	117	48	21	10
ACC_03439	cell adhesion molecule AbsCAM-Ig7B		KOG3513	19	18	8	24	4	0	3	3	12	5	1	0	0	0	0	0
ACC_03440	conserved hypothetical protein		KOG1052	9	4	4	34	24	2	1	3	332	245	50	76	20	55	8	2
ACC_03441	conserved hypothetical protein		KOG2418	0	0	0	0	0	1	0	0	0	4	0	1	0	1	0	1
ACC_03442	TRAF-interacting protein-like	K11985	KOG0827	166	176	209	305	478	54	232	236	366	254	321	255	69	85	21	8
ACC_03443	zinc finger MYND domain-containing protein 10-like, partial		KOG1710	111	122	111	167	219	54	138	146	352	137	223	325	32	46	18	4
ACC_03444	conserved hypothetical protein			225	173	214	470	316	25	34	35	308	104	187	304	123	149	16	22
ACC_03445	cytochrome c oxidase subunit 6C	K02268		295	93	124	934	259	20	20	50	211	29	699	556	755	649	85	37
ACC_03446	conserved hypothetical protein		KOG3273	602	440	399	661	281	96	170	200	1035	884	190	259	603	739	570	326
ACC_03447	conserved hypothetical protein			571	385	446	230	89	11	16	11	469	275	44	61	112	84	32	7
ACC_03448	hypothetical protein			13	1	7	4	2	0	0	2	1	10	3	1	0	0	1	5
ACC_03449	conserved hypothetical protein			33	22	31	42	37	5	4	4	68	33	29	18	38	41	6	4
ACC_03450	hymenoptaecin			4	13	110	19	67	84	29	14	23255	861	38	2336	5	5	5	3
ACC_03451	mitochondrial import inner membrane translocase subunit T		KOG3489	50	30	36	83	99	5	5	4	28	13	43	41	227	499	57	35
ACC_03452	tyrosine-protein kinase CSK-like	K05728	KOG0197	459	164	181	498	277	75	131	175	657	368	339	325	244	154	275	131
ACC_03453	conserved hypothetical protein			81	56	76	289	106	9	14	16	688	996	472	1827	1045	683	86	22
ACC_03454	hypothetical protein			0	0	0	0	0	0	0	0	0	0	0	0	0	0	0	0
ACC_03455	helicase POLQ-like		KOG0950	656	366	353	505	646	67	147	173	766	450	387	379	601	482	199	80
ACC_03456	conserved hypothetical protein			3	4	0	10	1	0	0	1	1	5	1	0	0	1	0	0
ACC_03457	discoidin domain-containing receptor 2-like isoform 1		KOG1094	350	121	156	418	87	9	11	20	120	71	17	13	7	13	48	13
ACC_03458	polyadenylate-binding protein 2	K14396	KOG4209	464	299	251	422	293	125	201	219	141	165	173	253	407	702	1241	852
ACC_03459	conserved hypothetical protein		KOG3598	2882	2203	1870	1443	962	429	704	781	2574	1635	953	749	1151	1311	1076	450
ACC_03460	LOW QUALITY PROTEIN		KOG2648	189	103	105	245	339	14	45	42	166	122	187	235	353	486	83	51
ACC_03461	sodium/hydrogen exchanger 8-like	K14724	KOG1965	1282	1091	1121	1243	1161	78	150	134	1251	1039	365	602	1041	1003	96	44
ACC_03462	ataxin-3-like isoform 2	K11863	KOG2935	616	423	465	867	839	129	276	335	775	364	690	655	513	674	290	174
ACC_03463	cold shock domain-containing protein E1			1092	532	428	1014	827	117	159	214	2139	1543	334	750	1201	1309	334	80
ACC_03464	u3 small nucleolar RNA-associated protein 15 hom(	K14549	KOG0310	243	205	188	254	246	36	40	60	330	270	163	298	564	617	59	27
ACC_03465	sodium-dependent neutral amino acid transporter	K05048	KOG3659	1197	381	433	1145	438	62	83	161	371	188	47	19	0	2	23	8
ACC_03466	conserved hypothetical protein		KOG3775	333	168	173	412	127	21	22	39	385	183	75	66	8	7	12	7
ACC_03467	hemocyte protein-glutamine gamma-glutamyltransferase-like			116	90	66	241	192	15	21	30	428	844	69	204	791	44	6	12
ACC_03468	TOM1-like protein 2-like		KOG1087	882	440	385	913	626	161	281	310	1108	761	719	1061	307	194	175	106
ACC_03469	conserved hypothetical protein			73	37	28	124	132	5	4	7	298	193	64	230	363	457	79	9
ACC_03470	folliculin	K09594	KOG3715	169	138	123	261	248	37	48	54	658	333	174	303	410	504	104	32
ACC_03471	DNA polymerase delta small subunit-like	K02328	KOG2732	146	161	173	321	279	8	6	17	168	154	49	368	192	129	12	2
ACC_03472	conserved hypothetical protein		KOG3598	359	319	244	245	202	111	204	287	332	263	80	16	116	142	272	241
ACC_03473	epidermal growth factor receptor pathway substrate clone 1		KOG0998	1984	1590	1641	1652	867	213	280	333	1851	2039	368	624	1664	1766	1133	546
ACC_03474	conserved hypothetical protein		KOG1808	562	371	356	717	552	70	92	102	840	940	238	761	1163	1643	339	147

ACC_03475	LOW QUALITY PROTEIN	K16172	KOG4701	290	289	255	172	85	38	33	67	706	515	49	105	244	211	100	21
ACC_03476	conserved hypothetical protein		KOG0197	67	51	65	71	27	1	1	2	60	23	2	4	5	3	13	5
ACC_03477	ecdysteroid UDP-glucosyltransferase-like		KOG1192	18	3	3	21	9	5	6	3	524	59	954	3154	0	10	0	1
ACC_03478	conserved hypothetical protein		KOG0161	1054	690	640	949	1034	200	536	569	1012	652	733	1039	806	1020	584	326
ACC_03479	conserved hypothetical protein	K16804	KOG3971	89	36	46	91	90	6	28	32	33	44	37	32	275	481	624	386
ACC_03480	LOW QUALITY PROTEIN		KOG3637	40	14	15	28	13	15	21	17	103	85	25	28	9	6	39	26
ACC_03481	rac GTPase-activating protein 1-like	K16733	KOG3564	142	124	105	166	125	11	22	26	118	116	51	95	132	177	46	21
ACC_03482	conserved hypothetical protein			327	234	263	496	398	22	43	53	281	208	134	337	408	428	130	27
ACC_03483	conserved hypothetical protein	K16753	KOG1144	130	66	86	179	102	24	46	59	147	65	64	25	34	50	248	194
ACC_03484	conserved hypothetical protein			1574	1017	1187	1245	346	42	29	55	620	375	8	10	1	16	20	3
ACC_03485	conserved hypothetical protein	K09090	KOG4304	19	9	6	13	9	0	2	0	5	0	13	41	6	3	4	2
ACC_03486	myosin-IIIa	K08834	KOG0161	321	387	382	903	347	720	771	1025	11482	7075	86	636	152	289	142	6
ACC_03487	conserved hypothetical protein		KOG2462	119	91	78	89	74	8	11	4	69	61	19	27	163	251	123	90
ACC_03488	eukaryotic translation initiation factor 3 subunit H	K03247	KOG1560	282	253	220	506	494	24	34	40	423	343	209	572	1368	1806	158	58
ACC_03489	sorting nexin-9-like isoform 1		KOG2528	236	223	172	190	146	36	64	83	364	303	56	191	215	151	29	17
ACC_03490	non-structural maintenance of chromosomes element 1 hon		KOG4718	102	64	81	79	114	4	2	5	99	219	61	182	429	269	23	8
ACC_03491	conserved hypothetical protein			520	322	154	260	263	48	168	174	246	231	216	156	1776	3708	5176	3695
ACC_03492	conserved hypothetical protein		KOG1015	4281	2921	2434	4023	3455	1204	1871	2635	5792	4301	1993	794	1243	1311	1921	890
ACC_03493	enoyl-CoA hydratase domain-containing protein 3, mitochor		KOG1682	90	69	60	85	78	10	41	18	373	133	190	327	199	237	47	31
ACC_03494	serine/threonine-protein phosphatase 6 catalytic s	K15498	KOG0373	60	40	34	59	40	5	5	4	201	191	24	134	252	254	21	6
ACC_03495	myotubularin-related protein 14 isoform 1		KOG4471	248	135	125	269	166	29	32	32	245	148	73	87	288	387	129	20
ACC_03496	hemicentin-2-like		KOG3513	21	8	4	18	6	1	0	0	9	18	7	8	3	16	18	6
ACC_03497	jerky protein homolog-like		KOG3105	73	65	52	132	128	2	21	12	22	15	37	13	7	9	4	0
ACC_03498	LOW QUALITY PROTEIN	K14559	KOG2600	687	365	327	451	644	87	197	247	324	277	389	414	786	1273	733	471
ACC_03499	nipped-B-like protein-like	K06672	KOG1020	807	652	592	899	399	192	325	348	1104	1194	308	165	287	393	1257	603
ACC_03500	conserved hypothetical protein		KOG4623	78	26	25	65	58	4	11	6	81	75	32	60	524	684	97	47
ACC_03501	60S ribosomal protein L32-like isoform 1	K02912	KOG0878	488	361	170	603	451	106	113	125	318	313	674	625	4095	4947	985	482
ACC_03502	gem-associated protein 5-like	K13133	KOG0284	303	193	165	340	298	19	71	54	326	307	163	233	544	446	119	49
ACC_03503	exportin-5	K14289	KOG2020	117	81	51	102	78	6	9	19	133	225	27	51	551	480	74	26
ACC_03504	n-acetylgalactosaminyltransferase 6-like isoform 1	K00710	KOG3736	55	30	30	75	42	2	3	4	70	73	10	34	440	453	41	17
ACC_03505	ribosomal protein S6 kinase delta-1-like		KOG0603	669	415	377	505	449	23	60	69	533	512	294	559	473	260	60	18
ACC_03506	conserved hypothetical protein	K15142	KOG1883	82	87	130	165	136	6	3	8	95	77	57	69	72	120	18	9
ACC_03507	conserved hypothetical protein	K04378	KOG0015	26	22	27	37	13	1	1	2	99	105	7	18	16	19	28	21
ACC_03508	growth hormone-regulated TBC protein 1-A-like		KOG2058	182	63	70	95	107	9	10	13	125	42	53	114	68	78	20	8
ACC_03509	histone acetyltransferase type B catalytic subunit-li	K11303	KOG2696	58	46	56	103	104	4	5	9	61	40	42	57	171	315	47	26
ACC_03510	putative S-methyl-5'-thioadenosine phosphorylase-	K00772	KOG3985	229	160	144	328	285	24	46	36	309	188	293	622	1123	1573	141	32
ACC_03511	general transcription factor 3C polypeptide 3-like	K15201	KOG0966	265	163	152	294	347	9	47	39	242	262	168	255	411	369	86	29
ACC_03512	conserved hypothetical protein		KOG3815	29	5	13	23	20	3	9	6	50	33	10	4	7	6	6	2
ACC_03513	conserved hypothetical protein		KOG2812	814	230	247	681	799	320	637	589	1339	388	725	534	400	708	768	814
ACC_03514	conserved hypothetical protein		KOG0994	134	91	80	81	46	13	22	28	422	487	39	75	202	186	50	23
ACC_03515	microtubule-associated serine/threonine-protein k	K16309	KOG0605	91	91	83	133	97	7	8	8	87	64	28	57	188	330	75	13
ACC_03516	ADP-ribosylation factor 2-like	K07977	KOG0070	230	154	92	274	330	39	36	40	646	273	235	751	908	1151	62	11
ACC_03517	e3 ubiquitin-protein ligase SMURF2		KOG0939	79	43	51	84	37	10	20	13	217	264	38	90	122	95	31	14
ACC_03518	THO complex subunit 1-like isoform 1	K12878	KOG2491	413	392	384	534	422	51	121	104	463	303	305	237	404	613	91	42
ACC_03519	Golgi phosphoprotein 3-like	K15620	KOG3983	213	101	94	150	133	21	21	36	362	339	58	485	327	280	79	22
ACC_03520	aryl hydrocarbon receptor nuclear translocator-like	K02296	KOG3561	112	142	110	258	94	58	44	97	808	562	245	250	55	93	62	17
ACC_03521	breast cancer type 2 susceptibility protein-like	K08775	KOG4751	524	333	298	486	499	58	149	129	490	363	396	302	505	434	137	64
ACC_03522	conserved hypothetical protein			155	120	116	202	200	9	17	18	81	53	73	159	101	74	16	5
ACC_03523	leucine-rich repeat-containing protein C10orf11 homolog		KOG1644	175	125	137	335	240	13	21	27	255	170	125	290	458	407	100	51
ACC_03524	tumor protein p73-like	K10148		218	124	113	218	210	16	34	39	225	107	104	169	369	389	77	46
ACC_03525	conserved hypothetical protein		KOG0307	574	561	655	371	131	89	135	161	909	686	140	128	334	459	676	335
ACC_03526	conserved hypothetical protein		KOG4514	179	145	148	246	152	17	24	26	237	99	86	268	112	188	62	31
ACC_03527	TBC1 domain family member 22B-like		KOG1092	226	145	124	191	103	31	39	22	561	297	156	215	280	387	54	18
ACC_03528	RING finger protein 11-like isoform 1	K11980	KOG4628	275	148	147	279	158	25	23	34	700	438	140	686	294	259	110	24
ACC_03529	homer protein homolog 1-like	K15010		401	293	295	380	253	10	18	24	274	201	31	116	174	80	32	8
ACC_03530	methylosome subunit pICln-like	K05019	KOG3238	471	338	329	661	484	25	38	36	313	183	235	427	287	375	61	57
ACC_03531	hypothetical protein			1	0	0	1	0	0	0	0	0	0	0	0	0	1	0	0

ACC_03532	conserved hypothetical protein		KOG4441	216	141	137	168	222	22	37	53	280	236	149	297	501	471	51	23
ACC_03533	dnaJ homolog subfamily C member 2-like	K09522	KOG0724	418	297	275	498	360	94	171	184	426	259	199	159	469	732	1233	1054
ACC_03534	RNA-binding protein squid-like	K03102	KOG4205	264	273	202	198	150	30	48	60	548	567	137	488	1132	1081	496	181
ACC_03535	LOW QUALITY PROTEIN		KOG4019	217	141	168	257	179	37	39	38	185	101	82	296	195	199	48	17
ACC_03536	endoplasmic reticulum oxidoreductin-1-like	K10950	KOG2608	204	163	153	156	197	18	23	27	481	383	115	357	698	490	8	2
ACC_03537	conserved hypothetical protein		KOG4350	389	263	293	834	259	22	36	48	102	43	2	1	0	4	9	5
ACC_03538	protein dachsous-like	K16507	KOG4289	62	18	19	61	10	3	4	6	68	58	78	27	3	5	27	44
ACC_03539	protein croquemort		KOG3776	485	293	293	681	208	43	29	62	844	700	166	228	371	263	235	15
ACC_03540	COMM domain-containing protein 8-like			77	80	75	181	145	12	28	25	92	55	144	180	153	155	37	16
ACC_03541	ankyrin repeat domain-containing protein 49-like		KOG0512	57	42	37	97	74	4	8	10	59	39	46	88	186	199	19	11
ACC_03542	probable palmitoyltransferase ZDHHC23-like		KOG1311	69	33	48	118	115	3	5	9	76	46	39	87	69	77	2	6
ACC_03543	conserved hypothetical protein			0	0	0	1	1	0	0	0	3	0	0	1	0	0	0	1
ACC_03544	RWD domain-containing protein 4-like		KOG4018	186	133	131	248	173	30	50	47	150	119	148	261	419	430	221	130
ACC_03545	hypothetical protein			5	7	8	5	5	0	0	1	2	11	2	0	0	0	0	0
ACC_03546	SH3 domain-binding glutamic acid-rich protein homolog		KOG4023	648	255	265	506	300	102	209	275	678	278	340	709	677	622	874	764
ACC_03547	probable ATP-dependent RNA helicase DHX36-like	K14442	KOG0920	413	246	226	413	352	26	49	59	570	603	239	793	789	836	107	23
ACC_03548	ras-related protein Rab-14 isoform 1	K07881	KOG0097	470	319	318	517	481	60	65	87	804	461	197	1033	475	318	55	19
ACC_03549	conserved hypothetical protein			48	39	61	93	89	5	7	8	46	44	43	57	170	136	7	1
ACC_03550	conserved hypothetical protein		KOG0147	97	56	66	178	151	8	8	9	47	56	38	81	116	88	25	7
ACC_03551	probable pterin-4-alpha-carbinolamine dehydratas	K01724	KOG4073	198	158	124	284	233	23	50	92	185	211	123	513	514	399	54	38
ACC_03552	serine proteinase stubble-like		KOG3627	0	11	0	3	0	2	7	2	181	2	2	5	30	87	12	6
ACC_03553	trypsin-7-like		KOG3627	6	71	0	3	1	8	9	16	140	4	2	3	21	147	36	26
ACC_03554	calpain-D-like	K08582	KOG0045	215	134	117	103	51	19	39	39	520	491	64	181	364	310	113	32
ACC_03555	e3 ubiquitin-protein ligase RNF19A-like			212	143	162	233	111	16	16	24	261	148	65	113	89	89	30	9
ACC_03556	integrator complex subunit 2	K13139		236	201	223	405	370	10	26	27	212	343	53	99	163	157	17	6
ACC_03557	3-hydroxyacyl-CoA dehydratase 3-like		KOG3187	426	238	280	541	552	24	36	35	564	243	280	795	299	368	31	7
ACC_03558	LOW QUALITY PROTEIN	K14688	KOG1483	312	220	211	431	277	71	108	153	539	259	363	731	750	728	253	103
ACC_03559	trans-1,2-dihydrobenzene-1,2-diol dehydrogenase-	K00078	KOG2741	318	228	206	374	392	32	107	99	668	961	207	536	10479	2620	181	89
ACC_03560	transitional endoplasmic reticulum ATPase TER94 i:	K13525	KOG0730	1282	732	518	1163	989	119	144	162	4265	3609	954	3348	5234	5738	678	243
ACC_03561	dual specificity mitogen-activated protein kinase ki	K04430	KOG1006	249	206	207	404	349	36	47	61	472	361	143	518	401	562	103	16
ACC_03562	protein phosphatase 1 regulatory subunit 21-like		KOG4421	511	353	402	603	564	115	243	230	896	481	593	729	336	316	213	109
ACC_03563	iron-sulfur cluster assembly 2 homolog, mitochondrial-like		KOG1119	129	112	138	200	217	6	17	18	185	99	111	211	155	174	10	6
ACC_03564	transducin (beta)-like 3	K14555	KOG0319	340	216	187	313	406	37	80	76	612	576	219	693	656	603	86	69
ACC_03565	UPF0463 transmembrane protein C6orf35 homolog			188	80	91	288	231	19	30	30	276	149	98	402	276	325	33	7
ACC_03566	RAD50-interacting protein 1-like		KOG2218	433	290	284	259	286	17	24	23	255	366	147	336	464	418	38	5
ACC_03567	conserved hypothetical protein			178	94	97	231	288	28	68	69	55	56	34	26	15	37	55	49
ACC_03568	signal recognition particle 54 kDa protein-like	K03106	KOG0780	726	656	709	1120	721	190	246	289	1693	817	706	1199	1284	1472	326	114
ACC_03569	conserved hypothetical protein		KOG0670	733	602	630	391	140	56	86	96	1143	798	213	171	482	580	292	94
ACC_03570	ras-related protein Rap-1b	K04353	KOG0395	1261	806	709	962	400	196	324	530	963	353	433	488	750	1180	2104	1937
ACC_03571	ankyrin repeat and death domain-containing protein 1A-like		KOG4177	212	131	143	324	113	14	12	23	61	38	15	4	1	6	19	6
ACC_03572	lysozyme c-1			175	429	40	136	49	233	247	444	113	33	14	31	15	14	30	7
ACC_03573	6-phosphofructo-2-kinase/fructose-2,6-biphosphat	K01103	KOG0234	1686	1061	1228	4627	1783	282	361	531	4341	2332	1354	1260	679	808	769	274
ACC_03574	conserved hypothetical protein			1	1	0	4	1	1	0	0	4	1	0	0	0	0	0	2
ACC_03575	fasciclin-2 isoform 1	K06491	KOG3513	653	248	265	591	165	62	102	134	311	237	180	85	35	56	467	440
ACC_03576	short-chain type dehydrogenase/reductase y4vI-like		KOG4169	358	202	120	352	228	276	331	454	9925	5900	10	246	9012	2133	200	362
ACC_03577	15-hydroxyprostaglandin dehydrogenase		KOG4169	65	24	27	29	41	16	28	24	1336	707	0	2	2435	788	138	91
ACC_03578	cytochrome c1, heme protein, mitochondrial	K00413	KOG3052	1002	408	358	895	755	35	43	60	1211	478	367	1862	2593	2635	279	60
ACC_03579	tyrosine-protein kinase Src64B-like isoform 1	K05704	KOG0197	393	163	190	815	235	15	31	61	155	115	78	38	54	103	219	62
ACC_03580	slit homolog 2 protein		KOG4194	196	66	93	247	54	16	11	30	83	47	22	30	4	13	78	17
ACC_03581	autophagy-related protein 16-1-like		KOG0288	112	75	67	120	57	17	24	24	161	151	25	71	55	33	50	34
ACC_03582	conserved hypothetical protein		KOG4221	243	91	115	346	160	11	8	22	67	10	2	28	0	2	3	3
ACC_03583	pancreatic triacylglycerol lipase-like isoform 1			9	9	10	89	44	1	6	2	852	670	74	218	63	76	24	8
ACC_03584	ubiquitin-protein ligase E3A	K10587	KOG0941	406	239	224	410	348	32	66	88	895	813	138	386	887	842	134	56
ACC_03585	hymenoptaecin			16	19	222	66	104	195	99	19	33528	1017	113	3491	3	3	36	3
ACC_03586	conserved hypothetical protein			94	32	66	140	82	29	34	45	78	138	24	61	600	905	405	114
ACC_03587	secretory carrier-associated membrane protein 1-like		KOG3088	341	235	235	249	137	29	29	30	540	451	183	693	658	497	89	18
ACC_03588	cirhin-like		KOG2048	79	72	56	130	116	2	16	12	149	153	57	115	809	1036	110	30

ACC_03589	conserved hypothetical protein			1139	680	1080	2862	1466	127	131	144	5295	1499	10833	28145	572	857	344	19
ACC_03590	ribosome-releasing factor 2, mitochondrial-like	K02355	KOG0464	345	157	118	202	172	42	85	81	396	326	87	296	553	671	210	97
ACC_03591	ribosome-releasing factor 2, mitochondrial-like		KOG0464	22	12	8	37	34	5	3	5	35	18	14	16	26	40	13	0
ACC_03592	ribosome-recycling factor, mitochondrial-like	K02838	KOG4759	109	119	96	122	126	9	18	13	29	63	39	192	330	410	58	31
ACC_03593	UPF0549 protein C20orf43 homolog		KOG3113	793	565	576	1168	982	165	324	456	749	369	542	893	955	1311	934	788
ACC_03594	39S ribosomal protein L54, mitochondrial-like		KOG3435	120	129	107	346	229	10	38	62	172	111	245	368	621	582	77	57
ACC_03595	endochitinase-like	K01183	KOG2806	42	19	31	47	33	4	2	4	62	33	37	21	147	68	21	9
ACC_03596	40S ribosomal protein S16	K02960	KOG1753	1360	1292	878	4138	1840	289	455	507	1355	1222	2585	1783	4798	5977	1918	896
ACC_03597	serologically defined colon cancer antigen 1 homolog isoforr		KOG2030	963	547	508	617	679	168	431	447	684	492	384	445	827	1440	1236	877
ACC_03598	PHD finger protein 14-like		KOG0957	692	667	645	881	527	93	127	133	615	408	342	239	483	564	434	245
ACC_03599	ras-related protein Rab-7a-like	K07897	KOG0394	169	79	59	94	103	11	13	12	788	807	93	846	504	248	12	2
ACC_03600	UPF0468 protein CG5343-like		KOG3213	4	3	10	66	21	0	7	7	5	0	7	4	15	3	8	9
ACC_03601	26S proteasome non-ATPase regulatory subunit 1-l	K03032	KOG2062	201	60	58	230	166	9	7	11	495	584	121	301	570	782	45	22
ACC_03602	conserved hypothetical protein		KOG4047	106	74	53	73	106	8	6	18	303	272	57	257	346	215	18	2
ACC_03603	DDRGK domain-containing protein 1-like isoform 1		KOG3054	612	674	691	597	532	185	347	454	858	309	705	623	598	666	940	1169
ACC_03604	aldo-keto reductase family 1 member B10-like		KOG1577	1255	1055	1240	2951	1799	170	238	282	3306	4749	4437	12847	9930	10413	686	549
ACC_03605	aldose reductase-like isoform 1	K00011	KOG1577	1342	958	1168	4563	4120	165	276	344	5761	6038	3993	6393	13066	16845	724	927
ACC_03606	conserved hypothetical protein	K02129	KOG4326	471	214	239	1279	354	69	201	86	333	48	855	539	569	589	472	367
ACC_03607	ribose-5-phosphate isomerase	K01807	KOG3075	58	64	50	90	82	6	12	18	186	87	72	81	458	389	30	13
ACC_03608	conserved hypothetical protein			7	2	7	37	18	0	0	0	6	2	0	1	1	1	0	0
ACC_03609	putative pre-mRNA-splicing factor ATP-dependent	K14305	KOG0925	361	175	149	380	348	28	23	37	645	572	169	813	1031	1343	107	26
ACC_03610	grpE protein homolog, mitochondrial	K03687	KOG3003	443	359	372	559	495	54	69	114	678	369	401	787	1948	3902	492	386
ACC_03611	heat shock protein Hsp70Ab-like	K03283	KOG0100	265	246	1235	1331	497	61	117	147	2134	1496	244	299	76	129	39	42
ACC_03612	plexin-B		KOG3610	529	311	309	217	114	30	38	29	672	711	99	124	381	535	110	45
ACC_03613	protein BCL9 homolog		KOG3598	331	354	334	255	76	24	24	32	553	373	108	107	213	240	129	27
ACC_03614	conserved hypothetical protein	K12162	KOG3483	65	62	45	81	97	4	3	0	76	84	50	256	183	120	9	4
ACC_03615	conserved hypothetical protein			200	150	131	220	179	13	23	31	212	263	64	147	234	191	48	14
ACC_03616	conserved hypothetical protein		KOG0147	3423	2300	2483	3031	1638	402	557	840	1742	804	762	808	1223	1388	1002	607
ACC_03617	cyclin-dependent kinase 1-like isoform 1	K02087	KOG0594	33	18	18	120	67	1	7	2	38	17	24	43	181	233	16	5
ACC_03618	apolipoprotein D-like isoform 1		KOG4824	3110	2464	2513	12739	6754	149	213	331	2754	1267	185	477	643	242	40	20
ACC_03619	putative protein tag-52		KOG4424	42	62	42	53	66	3	8	19	30	21	22	59	77	90	17	8
ACC_03620	1-phosphatidylinositol-4,5-bisphosphate phosphod	K01116	KOG1264	380	230	191	197	167	19	39	41	362	359	105	113	315	283	50	14
ACC_03621	ferritin subunit-like	K00522	KOG2332	1674	1829	1401	3850	4500	384	474	859	6745	4104	2024	5220	13006	7424	550	104
ACC_03622	serine/threonine-protein kinase 6	K11481	KOG0580	58	43	57	182	166	5	8	10	75	27	34	16	208	553	120	126
ACC_03623	phenoloxidase subunit A3			57	27	28	99	42	62	62	61	2887	1830	67	266	233	77	26	32
ACC_03624	carboxypeptidase B-like		KOG2650	70	34	32	38	34	10	10	7	70	30	9	14	2474	1000	325	13
ACC_03625	peroxidase-like isoform 1		KOG2408	2411	1157	1354	4076	1860	530	634	1041	6464	7068	1696	768	281	486	1307	326
ACC_03626	conserved hypothetical protein			217	131	134	298	314	8	5	11	188	49	45	86	26	40	3	0
ACC_03627	multiple inositol polyphosphate phosphatase 1-like		KOG4108	3	0	0	1	5	0	0	0	1	0	3	5	0	0	0	0
ACC_03628	intraflagellar transport protein 46 homolog			2	0	1	8	4	0	2	2	10	15	125	317	2	0	4	0
ACC_03629	WD repeat-containing protein 85-like		KOG0280	87	54	55	85	123	10	34	25	71	47	78	99	170	207	42	40
ACC_03630	histone H3.3	K11253	KOG1745	720	346	349	1219	577	41	34	49	643	439	400	1392	849	1490	266	64
ACC_03631	zinc finger FYVE domain-containing protein 19-like			567	477	482	543	592	125	262	313	576	357	497	707	332	249	127	95
ACC_03632	transmembrane protein C2orf18 homolog isoform 1		KOG3912	308	161	162	329	320	22	18	28	530	364	219	556	447	426	25	10
ACC_03633	testis-expressed sequence 10 protein homolog		KOG2149	135	113	214	175	250	4	9	7	258	241	197	402	337	280	10	6
ACC_03634	conserved hypothetical protein	K05628	KOG3554	1596	1642	1677	1342	492	187	256	321	2428	1305	412	294	778	868	814	282
ACC_03635	conserved hypothetical protein		KOG1924	2337	1513	1558	1819	575	182	268	344	1669	1384	370	242	361	379	602	228
ACC_03636	conserved hypothetical protein			170	97	107	170	268	5	16	17	169	126	100	409	278	309	21	8
ACC_03637	nicotinic acetylcholine receptor alpha6 subunit		KOG3646	256	170	166	400	171	23	18	32	110	75	0	1	1	2	17	8
ACC_03638	LOW QUALITY PROTEIN	K13988	KOG4195	211	191	167	297	252	40	74	94	273	135	148	202	298	368	189	134
ACC_03639	general transcription factor IIH subunit 4-like	K03144	KOG3471	80	79	74	125	117	6	15	20	135	65	55	114	120	211	23	5
ACC_03640	sorting nexin-24-like isoform 1			37	19	25	110	66	5	1	2	41	21	25	52	24	35	8	0
ACC_03641	serine/threonine-protein kinase 3-like isoform 1	K04412	KOG0574	304	229	200	209	143	13	12	9	346	259	31	163	314	277	56	16
ACC_03642	40S ribosomal protein S10-like isoform 2	K02947	KOG3344	4677	4330	2413	5406	4293	765	1273	1721	2871	3219	4398	9962	10978	10471	3206	2289
ACC_03643	DNA repair and recombination protein RAD54B-like	K10877	KOG0390	47	41	38	52	45	8	6	8	62	47	32	51	181	171	51	27
ACC_03644	akirin-2 isoform 2		KOG4330	310	122	103	409	253	73	114	160	311	267	144	366	345	347	292	135
ACC_03645	arrestin homolog isoform 2	K13805	KOG3865	437	1810	3139	12936	8978	4309	4844	7384	38834	24988	44	418	31	39	11	4

ACC_03646	putative deoxyribose-phosphate aldolase-like	K01619	KOG3981	129	148	149	246	200	7	11	15	309	425	47	53	4122	1927	137	20
ACC_03647	40S ribosomal protein S4-like isoform 1	K02987	KOG0378	2212	1847	928	2484	1836	827	1312	1398	4338	2851	2487	3871	14901	20441	4110	1627
ACC_03648	RNA pseudouridylate synthase domain-containing protein 1-		KOG1919	53	63	85	119	109	5	7	20	58	38	54	76	82	183	23	6
ACC_03649	dystrotelin-like		KOG4286	281	233	244	245	248	14	37	35	160	81	128	113	129	177	21	3
ACC_03650	probable UDP-glucose 4-epimerase-like	K01784	KOG1371	266	260	255	773	721	43	49	77	447	357	283	848	446	928	157	87
ACC_03651	conserved hypothetical protein			56	63	53	179	138	3	4	12	41	25	51	159	99	67	6	6
ACC_03652	conserved hypothetical protein		KOG2462	4	5	7	21	10	0	0	0	2	3	0	0	0	1	0	0
ACC_03653	ATP synthase subunit delta, mitochondrial isoform	K02134	KOG1758	297	159	139	549	662	26	28	42	512	178	296	825	943	1265	78	45
ACC_03654	pyridoxal kinase-like	K00868	KOG2599	297	282	247	404	362	21	60	78	396	383	191	725	1151	911	42	11
ACC_03655	isovaleryl-CoA dehydrogenase, mitochondrial-like	K00253	KOG0141	37	51	44	98	82	4	10	9	170	234	49	78	915	798	108	55
ACC_03656	conserved hypothetical protein			1067	618	584	1020	1099	55	183	197	802	466	458	477	398	339	155	67
ACC_03657	conserved hypothetical protein		KOG2177	376	222	182	354	314	67	196	186	440	310	189	195	194	209	293	107
ACC_03658	cryptochrome 2	K02295	KOG0133	262	102	75	129	89	61	60	67	391	212	272	254	582	395	232	74
ACC_03659	COP9 signalosome complex subunit 6-like	K12179	KOG3050	191	117	118	303	337	19	16	33	304	210	223	559	393	557	40	23
ACC_03660	filamin-A-like	K04437	KOG0518	1025	505	335	414	265	257	301	322	2535	1958	1217	1494	1064	1837	344	174
ACC_03661	mRNA turnover protein 4 homolog	K14815	KOG0816	104	114	89	229	218	16	40	42	133	67	124	128	393	833	71	43
ACC_03662	nodal modulator 2-like isoform 1		KOG1948	523	355	309	459	313	30	56	69	890	949	338	324	1376	1275	162	39
ACC_03663	conserved hypothetical protein			610	284	281	589	314	91	144	208	270	154	151	169	161	164	285	138
ACC_03664	UDP-glucose	K11718	KOG1879	849	552	372	810	656	160	184	252	1015	1221	563	591	2233	2014	327	131
ACC_03665	immunoglobulin-binding protein 1b-like		KOG2830	312	231	287	413	370	36	56	66	433	195	203	379	315	415	126	78
ACC_03666	conserved hypothetical protein		KOG2200	422	221	206	376	266	188	280	318	1376	639	515	297	439	495	219	69
ACC_03667	28S ribosomal protein S28, mitochondrial		KOG4078	156	125	121	366	441	13	19	27	177	113	164	398	435	773	61	30
ACC_03668	intraflagellar transport protein 122 homolog		KOG1538	659	423	410	467	574	24	42	52	871	791	531	1261	850	848	48	11
ACC_03669	LOW QUALITY PROTEIN	K14050	KOG0529	186	141	130	157	221	4	11	14	93	124	130	362	230	200	32	19
ACC_03670	60S ribosomal protein L7a	K02936	KOG3166	2386	2137	1356	2048	1245	897	1614	2415	1893	1816	1821	2918	9859	13050	5267	2977
ACC_03671	conserved hypothetical protein			327	393	381	832	309	62	73	185	133	332	85	58	5	10	33	10
ACC_03672	LOW QUALITY PROTEIN	K14841	KOG3881	335	221	231	597	483	57	100	121	276	178	252	244	433	460	187	136
ACC_03673	conserved hypothetical protein	K16589		273	169	173	308	290	11	44	34	170	163	131	201	317	353	83	53
ACC_03674	transcriptional adapter 1-like isoform 1	K11317		141	107	100	335	256	16	23	26	146	85	126	220	103	170	27	7
ACC_03675	conserved oligomeric Golgi complex subunit 5-like isoform 2		KOG2211	482	344	347	586	486	60	106	110	358	271	285	209	302	318	71	16
ACC_03676	phosphoethanolamine/phosphocholine phosphata	K13248	KOG3120	87	71	30	138	142	13	32	15	181	156	88	195	556	456	32	11
ACC_03677	zinc finger MYND domain-containing protein 11-like		KOG3612	324	276	287	383	252	33	36	47	354	283	134	390	427	367	124	79
ACC_03678	ribonuclease kappa-like			229	164	172	490	519	17	24	39	179	73	304	718	560	450	18	16
ACC_03679	eukaryotic translation initiation factor 4 gamma		KOG0401	8221	5289	4113	4794	2998	1686	2352	2752	10811	10564	2482	2630	11049	11683	7668	3916
ACC_03680	superkiller viralicidic activity 2-like 2-like isoform 1	K12598	KOG0948	504	320	272	429	499	40	79	82	840	573	262	540	729	1055	144	73
ACC_03681	NEDD8-conjugating enzyme Ubc12-like	K10579	KOG0420	258	101	126	356	220	20	17	16	545	311	119	498	523	594	97	18
ACC_03682	zinc finger CCCH domain-containing protein 10-like		KOG2494	366	350	287	330	221	48	56	82	481	378	116	225	238	226	172	62
ACC_03683	conserved hypothetical protein		KOG0913	336	205	197	421	399	32	37	37	275	239	161	398	998	1301	267	143
ACC_03684	caspase-8-like		KOG3573	216	199	142	171	185	11	59	43	176	229	187	459	544	426	97	52
ACC_03685	phosphoinositide 3-kinase regulatory subunit 4	K08333	KOG1240	631	444	445	674	401	70	106	83	979	828	340	216	561	627	84	41
ACC_03686	v-type proton ATPase subunit d	K02146	KOG2957	998	451	453	942	967	57	64	88	941	568	578	2025	1363	1259	66	17
ACC_03687	facilitated trehalose transporter Tret1-2 homolog		KOG0254	2	1	0	4	1	1	1	2	15	2	0	2	108	187	47	4
ACC_03688	conserved hypothetical protein		KOG0839	355	235	204	188	244	16	36	33	274	381	159	247	536	425	39	13
ACC_03689	LOW QUALITY PROTEIN		KOG1710	426	229	223	673	484	34	63	97	367	366	229	496	432	633	171	117
ACC_03690	nuclear cap-binding protein subunit 2-like	K12883	KOG0121	266	265	322	373	398	33	53	65	397	181	168	326	345	457	58	44
ACC_03691	eukaryotic translation initiation factor 2-alpha kina	K16194	KOG1035	234	161	176	206	258	27	55	38	231	157	125	231	227	274	72	29
ACC_03692	conserved hypothetical protein		KOG4653	257	189	150	259	324	12	18	20	430	471	197	535	690	376	14	5
ACC_03693	conserved hypothetical protein		KOG4194	122	95	88	141	112	13	32	43	131	87	28	44	103	224	74	21
ACC_03694	centromere protein I-like	K11501		104	87	83	99	142	2	11	9	65	71	59	119	209	140	27	9
ACC_03695	LOW QUALITY PROTEIN		KOG0531	274	188	152	178	326	17	29	36	188	187	70	308	383	294	37	22
ACC_03696	serine/threonine-protein phosphatase 2A catalytic	K04382	KOG0371	811	488	520	577	335	55	61	76	1144	1130	223	1236	1117	1295	311	53
ACC_03697	receptor of activated protein kinase C 1 isoform 1	K14753	KOG0279	1467	859	589	2091	1484	384	432	602	1130	2545	888	1750	6421	9241	1668	818
ACC_03698	DNA-directed RNA polymerase I subunit RPA2	K03002	KOG0216	262	196	151	297	336	34	54	59	328	291	186	218	1002	1137	89	41
ACC_03699	probable arylformamidase-like	K01432	KOG4627	132	150	120	148	144	13	15	21	66	87	83	349	66	93	19	5
ACC_03700	40S ribosomal protein S23-like	K02973	KOG1749	2245	2608	1559	3498	1756	461	803	1064	1986	1440	2975	2973	8953	8331	3201	2907
ACC_03701	presenilins-associated rhomboid-like protein, mito	K09650	KOG2980	311	231	248	466	398	31	54	70	365	244	227	705	379	578	84	28
ACC_03702	peptidyl-prolyl cis-trans isomerase NIMA-interactin	K09578	KOG3259	180	173	118	296	267	8	4	22	237	205	83	184	318	389	44	25

ACC_03703	thrombospondin-3	K04659	KOG1219	363	198	195	405	292	55	80	80	3230	2100	656	829	145	303	110	38
ACC_03704	bolA-like protein 3-like		KOG3348	40	32	44	102	97	7	6	4	99	37	74	195	199	249	33	13
ACC_03705	intraflagellar transport protein 74 homolog		KOG0161	735	510	466	422	749	93	235	225	340	149	795	725	128	121	108	91
ACC_03706	protein lin-7 homolog B-like		KOG3550	251	155	178	634	404	35	29	66	321	164	281	354	229	251	46	39
ACC_03707	peroxisomal multifunctional enzyme type 2	K12405	KOG1206	561	389	294	427	386	43	56	63	1696	1870	702	2244	2254	1712	92	32
ACC_03708	putative aldehyde dehydrogenase family 7 membe	K14085	KOG2453	157	47	75	127	154	19	30	42	1480	2601	25	14	36493	5155	300	89
ACC_03709	serine/threonine-protein kinase fused-like	K06228	KOG0597	99	65	60	93	57	22	25	16	245	221	114	156	176	297	96	50
ACC_03710	conserved hypothetical protein			82	108	99	192	46	9	17	35	483	257	17	70	12	2	4	1
ACC_03711	putative zinc metalloproteinase YIL108W-like		KOG4525	260	119	150	357	143	17	11	14	93	69	27	50	12	41	24	13
ACC_03712	pleckstrin homology domain-containing family A member 8-		KOG3221	97	89	82	204	158	10	16	18	114	94	66	178	252	320	49	9
ACC_03713	26S proteasome non-ATPase regulatory subunit 11	K03036	KOG1463	416	201	184	604	413	51	52	69	905	759	366	1134	1316	2024	168	42
ACC_03714	proteasome activator complex subunit 3-like	K06698	KOG4470	497	267	205	540	494	36	86	100	384	406	234	859	1061	1502	485	240
ACC_03715	conserved hypothetical protein		KOG3608	278	187	214	369	264	13	24	26	474	361	116	250	375	356	78	20
ACC_03716	LOW QUALITY PROTEIN	K01689	KOG2670	2981	1281	1319	2319	2728	121	155	181	8305	3399	1172	4004	16339	10399	568	147
ACC_03717	H/ACA ribonucleoprotein complex subunit 4-like	K11131	KOG2529	547	166	99	328	402	57	105	160	223	190	196	164	1554	3979	3169	2136
ACC_03718	COX assembly mitochondrial protein homolog isoform 2		KOG4624	150	138	91	221	144	7	7	20	170	110	150	285	230	271	31	37
ACC_03719	zinc finger protein 346-like			216	112	105	351	253	21	29	45	146	89	103	130	182	249	79	36
ACC_03720	conserved hypothetical protein		KOG4526	507	385	320	602	451	85	160	151	848	573	788	1782	792	855	436	173
ACC_03721	sperm flagellar protein 1-like			1	5	3	6	9	0	4	1	1	2	0	4	5	12	4	4
ACC_03722	conserved hypothetical protein	K06220	KOG1830	224	177	216	190	67	19	16	18	325	198	150	167	93	106	94	37
ACC_03723	kynurenine--oxoglutarate transaminase 3-like	K00816	KOG0257	335	227	186	267	286	39	69	86	1250	846	638	1922	2345	1926	126	26
ACC_03724	feminizer			925	826	590	2041	2350	238	500	613	1640	1105	531	276	1924	2014	2047	1276
ACC_03725	hepatocyte growth factor-regulated tyrosine kinase	K12182	KOG1818	735	595	601	446	204	130	196	205	1128	836	383	307	689	503	442	280
ACC_03726	tRNA (cytosine-5-)-methyltransferase	K15336	KOG0919	65	48	39	69	75	3	6	12	60	39	30	86	138	136	9	6
ACC_03727	conserved hypothetical protein			15	10	14	10	5	0	2	1	18	8	0	0	1	2	1	0
ACC_03728	prominin-like protein-like	K06532	KOG4331	430	181	174	142	62	14	20	40	190	148	31	70	165	126	89	43
ACC_03729	loss of heterozygosity 12 chromosomal region 1 protein hon		KOG4515	162	133	146	292	183	16	14	35	186	99	134	296	183	170	37	12
ACC_03730	conserved hypothetical protein		KOG3827	2921	1229	1396	2553	685	213	259	412	2173	1344	371	748	219	288	658	528
ACC_03731	LOW QUALITY PROTEIN	K11291	KOG4563	328	221	261	423	274	83	133	163	628	197	424	354	552	1431	2102	2576
ACC_03732	thioredoxin, mitochondrial	K03671	KOG0910	146	85	88	387	366	10	20	39	196	119	306	294	400	617	43	56
ACC_03733	conserved hypothetical protein			389	267	330	396	477	13	91	97	301	131	430	603	283	427	134	114
ACC_03734	conserved hypothetical protein			0	0	2	2	0	1	1	0	1	1	7	4	5	3	2	1
ACC_03735	WW domain-containing oxidoreductase isoform 1		KOG1208	385	200	223	620	534	50	86	90	427	204	238	484	216	260	80	39
ACC_03736	m-phase phosphoprotein 6	K12593	KOG4531	198	114	125	174	148	16	55	65	70	56	115	168	74	117	88	51
ACC_03737	cation-independent mannose-6-phosphate recepto	K06564	KOG4504	337	247	186	259	283	23	47	48	540	484	91	322	1023	667	66	33
ACC_03738	cytochrome c oxidase subunit 5B, mitochondrial	K02265	KOG3352	683	372	326	1836	1366	48	59	75	856	224	948	1324	1017	1132	190	168
ACC_03739	conserved hypothetical protein			136	83	83	78	150	3	22	16	58	67	106	254	187	160	45	17
ACC_03740	protein FAM36A-like			33	24	27	58	62	2	6	4	18	17	49	78	39	64	7	2
ACC_03741	synaptic vesicle glycoprotein 2B-like		KOG0253	9	3	2	10	15	0	1	1	19	8	69	488	22	17	1	2
ACC_03742	peptidyl-prolyl cis-trans isomerase-like	K09565	KOG0111	2160	1558	1289	4523	2785	180	209	323	2660	1995	2046	6007	7380	7601	1786	998
ACC_03743	xylulose kinase-like	K00854	KOG2531	46	62	30	59	54	19	18	14	219	497	41	133	301	107	21	9
ACC_03744	conserved hypothetical protein	K10627	KOG0287	211	157	120	231	224	12	48	42	140	166	149	277	240	242	109	41
ACC_03745	conserved hypothetical protein	K09454	KOG0509	260	184	152	209	109	18	39	34	571	373	121	362	525	644	126	71
ACC_03746	ubiquitin-fold modifier-conjugating enzyme 1-like	K12165	KOG3357	114	108	76	197	227	15	17	32	244	111	169	410	540	559	34	18
ACC_03747	serine/threonine-protein kinase D3 isoform 2	K06070	KOG4236	178	100	80	84	51	9	9	10	327	265	30	137	172	160	13	8
ACC_03748	conserved hypothetical protein			21	42	32	110	48	141	120	198	1637	864	1	71	0	0	3	0
ACC_03749	suppressor of variegation 3-9 isoform 2	K03242	KOG0466	775	464	423	995	651	163	240	282	1126	753	820	1508	2333	3395	734	444
ACC_03750	TBC1 domain family member 10A		KOG2221	422	213	185	476	584	68	173	190	386	193	245	648	326	276	88	47
ACC_03751	transmembrane protein 111-like		KOG3188	813	412	345	593	612	50	31	61	542	470	309	1288	782	573	89	38
ACC_03752	mediator of RNA polymerase II transcription subun	K15148	KOG0570	85	54	73	145	107	14	15	18	94	38	78	130	95	148	17	9
ACC_03753	dnaJ homolog subfamily B member 12-like	K09518	KOG0720	1107	503	459	812	613	90	130	149	1619	1294	394	1618	1017	1004	188	92
ACC_03754	protein spitz-like	K16691	KOG1219	96	84	71	67	14	8	12	18	49	87	29	27	6	21	62	33
ACC_03755	conserved hypothetical protein	K13205	KOG3937	90	67	77	163	207	11	17	18	149	80	87	135	127	168	15	5
ACC_03756	mitochondrial import inner membrane translocase subunit T		KOG1652	276	170	209	419	265	12	20	28	330	191	87	451	502	818	285	80
ACC_03757	hypothetical protein			13	4	4	2	1	3	7	6	6	1	4	1	0	0	1	2
ACC_03758	zinc finger protein 571-like		KOG2462	225	165	127	273	164	46	56	68	368	362	101	272	346	283	97	55
ACC_03759	F-box only protein 39-like			116	79	69	278	100	22	27	32	147	62	293	246	18	10	21	24

ACC_03760	hypothetical protein			1	0	0	1	1	1	0	1	1	1	1	3	0	1	14	18
ACC_03761	conserved hypothetical protein		KOG0254	6	0	1	8	1	0	0	0	6	12	1	0	0	1	4	2
ACC_03762	NADH dehydrogenase (ubiquinone) 1 beta subcom	K03958		239	153	149	749	305	35	40	66	246	94	584	317	433	346	47	21
ACC_03763	leucine-rich repeat-containing protein 40-like		KOG0472	576	374	463	537	628	44	62	70	891	363	638	941	201	216	32	7
ACC_03764	coatomer subunit gamma isoform 1		KOG1078	1101	713	631	1775	1336	138	180	223	1964	1702	676	1658	1938	2322	251	73
ACC_03765	methionine aminopeptidase 2	K01265	KOG2775	3797	1602	1331	3394	2454	951	2257	1644	2188	1580	2107	2291	2452	3523	9374	10587
ACC_03766	dihydrolipoyl dehydrogenase, mitochondrial-like is	K00382	KOG1335	1352	509	396	1033	850	107	127	173	2206	857	660	1547	4445	4700	560	291
ACC_03767	WD repeat and FYVE domain-containing protein 3-like		KOG1788	1401	1043	914	1190	925	205	402	432	2277	1924	862	847	1102	1348	790	308
ACC_03768	conserved hypothetical protein			17	0	5	5	2	0	0	0	54	172	0	3	669	24	5	24
ACC_03769	conserved hypothetical protein	K11498	KOG0242	64	42	38	55	76	10	26	22	28	30	33	17	390	943	1604	639
ACC_03770	integrator complex subunit 1-like	K13138	KOG4596	409	354	259	423	364	39	27	50	409	498	167	178	587	746	76	22
ACC_03771	probable U2 small nuclear ribonucleoprotein A'	K11092	KOG1644	171	153	155	271	189	28	27	42	253	155	137	165	383	659	126	80
ACC_03772	NADH dehydrogenase	K03963	KOG3468	719	909	1150	802	758	158	234	351	758	241	510	1109	603	710	202	176
ACC_03773	elongation of very long chain fatty acids protein AAEL00800		KOG3071	132	96	92	166	134	26	39	55	190	91	85	173	597	442	104	48
ACC_03774	conserved hypothetical protein		KOG1454	95	57	81	159	99	3	5	9	175	145	95	133	228	272	19	15
ACC_03775	synaptotagmin 20		KOG1028	267	406	240	131	95	168	192	288	496	279	311	899	63	91	34	12
ACC_03776	apoptosis regulatory protein Siva-like			20	20	13	24	23	0	0	2	20	11	23	43	111	124	12	4
ACC_03777	conserved hypothetical protein			25	13	14	11	14	0	0	0	22	11	8	7	17	11	0	0
ACC_03778	asparagine-linked glycosylation protein 11 homolo	K03844	KOG1387	275	241	202	300	283	38	55	56	450	242	273	457	525	562	29	10
ACC_03779	hypothetical protein			10	1	3	6	8	0	0	0	8	3	4	1	0	0	0	0
ACC_03780	hypothetical protein			25	4	12	11	8	0	3	4	3	3	3	2	0	0	4	0
ACC_03781	LIM domain kinase 1		KOG1044	22	15	12	15	5	2	0	1	55	102	9	37	30	15	1	0
ACC_03782	ets DNA-binding protein pokkuri-like	K03211	KOG3804	70	54	45	42	27	1	8	14	130	95	18	71	92	77	67	12
ACC_03783	transmembrane protein 41 homolog isoform 2		KOG3140	360	230	200	406	303	33	46	42	460	340	419	756	577	553	45	19
ACC_03784	conserved hypothetical protein			363	287	292	609	557	53	81	91	516	442	312	589	845	829	181	94
ACC_03785	FAD-dependent oxidoreductase domain-containing protein		KOG2853	771	402	390	754	849	93	133	163	2119	3417	426	1768	2896	1571	181	122
ACC_03786	Fanconi anemia group J protein homolog, partial	K15362	KOG1132	64	88	48	42	23	10	39	19	91	71	26	41	78	136	50	28
ACC_03787	trypsin-6-like		KOG3627	2	2	1	8	2	0	0	0	0	3	0	0	0	0	1	1
ACC_03788	26S protease regulatory subunit 7-like	K03061	KOG0729	773	425	407	873	714	105	152	162	1457	758	945	1821	1653	2329	622	422
ACC_03789	arf-GAP with coiled-coil, ANK repeat and PH domai	K12489	KOG0521	124	75	58	95	60	6	13	13	289	329	33	112	97	89	9	3
ACC_03790	mitochondrial tRNA-specific 2-thiouridylase 1-like	K00566	KOG2805	254	171	186	254	322	21	46	45	244	155	233	257	546	562	62	27
ACC_03791	conserved hypothetical protein			23	9	6	20	17	8	35	43	7	1	55	15	1	3	29	11
ACC_03792	probable cytochrome P450 304a1		KOG0156	345	182	199	367	480	10	30	48	718	381	164	330	276	280	18	6
ACC_03793	hypothetical protein			36	9	12	49	52	1	6	11	3	2	4	0	0	0	3	0
ACC_03794	LOW QUALITY PROTEIN	K10357	KOG0160	4225	2706	2961	5308	3407	903	1488	1769	3072	2035	873	575	715	491	375	446
ACC_03795	f-box/LRR-repeat protein 20-like isoform 1	K10268	KOG4341	299	152	123	288	88	50	35	55	363	256	99	77	46	34	39	8
ACC_03796	LOW QUALITY PROTEIN	K12813	KOG0923	111	82	72	187	122	18	26	48	220	161	112	128	201	275	101	83
ACC_03797	hypothetical protein			10	8	7	7	2	0	0	1	2	1	11	4	0	0	0	0
ACC_03798	conserved hypothetical protein			3	4	1	7	4	0	1	0	16	13	25	22	467	759	59	3
ACC_03799	glutathione S-transferase-like isoform 1		KOG1695	1556	1190	1084	6632	4893	170	145	186	1323	968	2522	7689	3800	4764	364	111
ACC_03800	glutathione S-transferase S1		KOG1695	736	918	1090	3058	2792	51	83	139	1346	718	871	4956	7330	7625	812	243
ACC_03801	hypothetical protein			2	2	4	7	1	0	1	0	2	0	0	0	0	0	0	0
ACC_03802	beta-1,4-galactosyltransferase 7-like	K00733	KOG3917	223	89	96	341	312	10	20	35	258	130	151	205	215	224	32	5
ACC_03803	huntingtin-like	K04533		1085	569	535	1096	747	135	256	238	1693	1209	488	456	394	448	273	118
ACC_03804	thrombospondin type-1 domain-containing protein 4-like		KOG4597	257	147	149	336	107	51	35	56	571	123	20	97	57	48	37	17
ACC_03805	DNA-directed RNA polymerase I subunit RPA49-like	K03005	KOG4183	165	111	127	231	238	12	23	45	189	123	106	246	327	497	97	37
ACC_03806	zygotic DNA replication licensing factor mcm3	K02541	KOG0479	230	144	157	359	280	25	58	74	167	175	182	189	488	752	582	323
ACC_03807	conserved hypothetical protein			77	32	17	106	103	4	3	7	107	32	29	211	102	158	19	5
ACC_03808	conserved hypothetical protein			238	162	161	221	230	2	25	26	139	78	126	307	199	232	60	40
ACC_03809	bystin-like	K14797	KOG3871	367	181	164	315	312	46	102	112	375	328	217	273	1056	1370	660	724
ACC_03810	annexin-B9-like		KOG0819	273	177	159	429	406	13	43	38	813	387	315	1692	1197	1026	52	32
ACC_03811	mitochondrial import inner membrane translocase subunit T		KOG1733	49	24	20	90	70	2	10	10	28	21	33	100	280	488	113	88
ACC_03812	probable glutaminyl-tRNA synthetase	K01886	KOG1148	458	253	196	370	345	74	120	118	402	420	320	454	1332	1494	474	347
ACC_03813	replication factor C subunit 1	K10754	KOG1968	928	562	527	862	898	128	319	324	875	520	476	451	1034	864	711	374
ACC_03814	putative odorant receptor 13a-like			35	37	34	84	48	2	7	12	34	45	49	101	33	7	5	0
ACC_03815	conserved hypothetical protein		KOG4395	3	6	1	16	6	0	2	1	2	5	3	3	2	0	4	0
ACC_03816	conserved hypothetical protein		KOG3274	225	120	123	72	48	10	14	14	247	170	61	195	245	262	53	17

ACC_03817	AMMECR1-like protein-like		KOG3274	160	102	93	126	100	13	13	15	219	158	78	169	203	242	33	3
ACC_03818	conserved hypothetical protein		KOG2072	454	389	360	314	430	145	304	394	265	204	156	151	280	264	604	459
ACC_03819	WD repeat-containing protein 3-like	K14556	KOG0306	281	192	208	377	328	54	91	99	378	384	207	253	963	1401	281	142
ACC_03820	conserved hypothetical protein	K15168	KOG3598	389	438	477	630	267	54	67	136	421	421	184	251	848	1088	335	164
ACC_03821	phosphoribosylformylglycinamidine synthase	K01952	KOG1907	203	186	114	180	191	30	44	40	538	2171	77	119	1724	1450	113	63
ACC_03822	DNA topoisomerase 3-beta-1-like isoform 2	K03165	KOG1957	260	182	191	414	290	26	47	58	425	293	195	248	269	401	49	29
ACC_03823	protein cramped-like		KOG4468	339	201	192	332	246	58	93	118	613	288	307	277	303	290	107	45
ACC_03824	ETS homologous factor-like	K09429	KOG3804	38	74	64	140	74	45	27	39	750	613	112	806	90	31	30	10
ACC_03825	conserved hypothetical protein		KOG3621	162	114	111	133	201	4	26	20	66	63	73	93	161	176	21	13
ACC_03826	SAGA-associated factor 11 homolog	K11363	KOG2612	141	112	124	228	213	31	61	74	132	82	109	138	81	103	31	26
ACC_03827	serine protease easter-like		KOG3627	54	45	49	235	154	20	15	22	1944	1275	46	77	598	220	36	10
ACC_03828	conserved hypothetical protein			171	151	157	318	188	34	52	79	329	263	230	48	170	194	60	35
ACC_03829	Broad-complex core protein isoform 6		KOG4441	197	111	126	138	109	11	12	24	256	220	47	93	79	122	36	21
ACC_03830	conserved hypothetical protein		KOG3623	97	60	53	114	111	18	19	26	71	15	68	30	19	60	34	14
ACC_03831	TIM21-like protein, mitochondrial-like		KOG4836	129	125	135	196	179	24	27	40	263	113	156	166	427	677	115	42
ACC_03832	WD repeat and FYVE domain-containing protein 2-like		KOG1409	348	188	177	254	170	42	59	74	684	474	152	576	381	272	56	9
ACC_03833	myosin-VIIa-like	K10359	KOG0161	190	237	73	44	29	38	43	62	379	408	84	142	630	857	166	67
ACC_03834	DNA repair protein complementing XP-G cells hom	K10846	KOG2520	652	467	448	557	403	65	154	169	771	562	510	421	387	479	150	68
ACC_03835	proteasome subunit alpha type-7-1-like isoform 1	K02731	KOG0183	556	569	431	682	493	181	354	362	1296	614	973	1694	1830	2569	866	840
ACC_03836	cyclin-dependent kinase 8-like	K02208	KOG0666	237	228	211	426	268	40	55	61	213	141	86	183	363	507	169	47
ACC_03837	transmembrane protein 216-like		KOG4502	91	55	90	157	148	13	23	16	68	36	88	79	32	62	8	5
ACC_03838	diuretic hormone class 2-like			140	66	128	337	141	3	4	4	40	40	1	0	3	12	3	1
ACC_03839	EH domain-containing protein 1-like	K14410	KOG3720	71	112	58	171	104	49	44	91	191	241	109	261	354	155	17	7
ACC_03840	EH domain-containing protein 1	K12476	KOG1954	607	363	363	529	429	90	97	120	1165	872	356	1422	1596	1505	141	35
ACC_03841	target of rapamycin complex subunit lst8-like	K08266	KOG0315	382	266	274	505	451	73	155	173	352	198	268	370	302	355	114	33
ACC_03842	seryl-tRNA synthetase, cytoplasmic-like isoform 1	K01875	KOG2509	812	513	323	920	880	138	309	308	731	450	386	737	1967	2639	1104	602
ACC_03843	39S ribosomal protein L40, mitochondrial		KOG4778	751	361	344	727	772	126	323	326	474	177	584	513	377	522	917	838
ACC_03844	UPF0466 protein AGAP011291, mitochondrial-like		KOG4542	95	71	45	85	108	3	10	7	129	65	43	152	43	46	5	1
ACC_03845	mitochondrial import receptor subunit TOM22 homolog		KOG4111	276	170	163	744	388	26	24	43	292	194	252	470	495	966	574	161
ACC_03846	conserved hypothetical protein			760	428	318	945	702	282	468	555	2802	1212	1374	1727	695	424	273	110
ACC_03847	conserved hypothetical protein		KOG4507	335	168	196	327	306	22	34	32	619	264	205	374	219	332	37	5
ACC_03848	conserved hypothetical protein	K04417	KOG0192	302	216	194	261	191	36	60	49	613	610	127	194	589	669	226	82
ACC_03849	aldose 1-epimerase-like		KOG1604	52	47	78	64	74	3	12	14	71	37	91	28	35	38	16	5
ACC_03850	RISC-loading complex subunit tarbp2-like		KOG3732	367	285	229	400	155	18	25	37	415	551	78	182	158	148	59	16
ACC_03851	conserved hypothetical protein			956	980	793	1367	499	132	237	245	753	778	258	263	282	271	423	116
ACC_03852	spindle assembly abnormal protein 6 homolog	K16487	KOG0161	155	84	70	125	165	23	67	79	73	85	107	77	82	85	182	72
ACC_03853	gephyrin-like	K15376	KOG2371	758	514	388	343	343	92	176	179	822	643	523	772	2825	2172	359	119
ACC_03854	conserved hypothetical protein		KOG1984	900	535	470	626	363	120	206	218	900	600	300	185	254	224	303	97
ACC_03855	LOW QUALITY PROTEIN	K07151	KOG2292	1385	1155	651	713	700	318	447	608	1105	1218	650	656	4295	3777	451	171
ACC_03856	conserved hypothetical protein	K11668		214	125	137	187	106	55	93	90	386	259	92	96	129	144	81	24
ACC_03857	conserved hypothetical protein		KOG4364	23	33	25	64	26	6	6	11	54	22	23	22	35	47	52	12
ACC_03858	peroxisomal membrane protein 2-like	K13347	KOG1944	218	209	164	322	362	37	50	56	605	381	290	888	1043	950	94	61
ACC_03859	uridine diphosphate glucose pyrophosphatase-like isoform 1		KOG4432	224	233	200	751	505	23	35	66	325	229	291	572	727	677	163	98
ACC_03860	exosome complex exonuclease RRP40	K03681	KOG1004	63	51	90	111	111	5	10	9	79	46	39	80	173	300	56	15
ACC_03861	dehydrogenase/reductase SDR family protein 7-like isoform		KOG1205	401	295	285	468	517	29	52	59	350	328	378	767	426	456	81	37
ACC_03862	conserved hypothetical protein			16	8	13	10	6	3	10	7	6	4	1	3	1	1	31	31
ACC_03863	cGMP-dependent protein kinase, isozyme 1-like		KOG0614	1	0	1	1	1	0	0	0	3	4	0	0	97	82	4	0
ACC_03864	peptidyl-prolyl cis-trans isomerase H-like	K09567	KOG0879	27	30	15	82	53	2	11	14	28	10	48	32	66	172	45	25
ACC_03865	TBC1 domain family member 9		KOG4347	429	246	251	477	381	28	30	59	590	553	153	378	301	251	20	5
ACC_03866	ankyrin repeat domain-containing protein 29-like		KOG4177	185	78	77	234	105	159	281	358	1004	346	349	622	117	85	306	210
ACC_03867	UPF0636 protein C4orf41 homolog		KOG4386	339	341	323	330	393	51	77	73	598	467	195	391	424	395	53	23
ACC_03868	conserved hypothetical protein			15	20	25	21	16	2	7	5	10	4	12	41	11	7	2	0
ACC_03869	Aryl-hydrocarbon-interacting protein-like 1		KOG0545	317	215	219	402	506	51	109	113	402	222	334	571	307	395	121	73
ACC_03870	protein pelota isoform 1	K06965	KOG2869	253	179	198	426	458	27	56	74	464	285	146	320	508	689	150	59
ACC_03871	pescadillo homolog	K14843	KOG2481	746	617	554	874	773	177	418	469	667	556	488	387	2242	3932	3427	2802
ACC_03872	NADH dehydrogenase	K03950	KOG3426	505	538	542	1033	786	58	74	109	485	300	532	1157	470	600	152	113
ACC_03873	protein FAM50 homolog	K13119	KOG2894	640	404	386	477	599	143	251	339	374	178	275	360	290	518	852	572

ACC_03874	neuroendocrine convertase 2 isoform 1	K01360	KOG3526	931	570	644	943	259	30	28	40	546	403	71	79	24	37	45	12
ACC_03875	endothelin-converting enzyme 1-like		KOG3624	527	218	269	504	114	34	37	88	244	137	193	143	0	2	5	3
ACC_03876	40S ribosomal protein S25 isoform 2	K02975	KOG1767	3594	2332	1230	2168	924	964	1474	1929	1910	1575	1130	2255	8070	9810	13089	11748
ACC_03877	testis-specific serine/threonine-protein kinase 1-lik	K08811	KOG0583	49	27	44	133	48	13	17	20	40	30	42	5	9	28	44	45
ACC_03878	zinc finger FYVE domain-containing protein 26-like		KOG1811	898	601	514	887	969	33	77	102	558	878	246	477	640	434	112	32
ACC_03879	protein peanut-like		KOG2655	686	229	207	585	309	114	259	259	437	466	218	212	220	375	988	595
ACC_03880	farnesoic acid o-methyltransferase-like isoform 1 protein			1399	1373	1117	2657	1752	138	185	261	3752	1987	1343	4068	754	710	149	162
ACC_03881	lipoma HMGIC fusion partner-like 4 protein-like		KOG4026	91	136	70	75	29	41	29	56	160	60	67	473	193	139	70	36
ACC_03882	protein HEXIM1-like	K15189		266	200	192	255	149	26	44	62	328	276	69	200	151	182	71	42
ACC_03883	DNA damage-binding protein 1-like isoform 1	K10610	KOG1897	678	389	361	515	508	38	54	55	1143	872	365	607	1480	1869	120	35
ACC_03884	DNA-directed RNA polymerase III subunit RPC6-like	K03025	KOG3233	202	57	56	130	159	44	60	50	281	136	120	254	326	453	108	117
ACC_03885	bromodomain adjacent to zinc finger domain prote	K11655	KOG1245	1880	1185	1110	1485	1705	343	739	850	1536	1303	1047	766	1201	2039	2760	1600
ACC_03886	probable glucosamine 6-phosphate N-acetyltransfe	K00621	KOG3396	133	95	83	139	183	14	19	29	246	141	95	240	338	559	59	37
ACC_03887	FERM domain-containing protein 8 isoform 1		KOG4335	382	197	302	670	511	14	36	46	222	76	157	169	54	88	8	4
ACC_03888	3'(2'),5'-bisphosphate nucleotidase 1-like	K01082	KOG3099	225	165	139	287	213	18	15	30	209	151	128	432	289	309	38	12
ACC_03889	ubiquinone biosynthesis protein COQ7	K06134	KOG4061	439	313	314	845	631	45	92	125	345	134	357	520	349	450	152	98
ACC_03890	conserved hypothetical protein			3	1	3	9	3	0	0	0	1	3	35	14	0	0	0	0
ACC_03891	conserved hypothetical protein			31	89	105	199	145	11	20	22	65	27	64	27	0	0	0	0
ACC_03892	spatacsin		KOG1884	411	345	327	480	683	22	30	30	486	858	487	800	655	540	27	9
ACC_03893	NADH dehydrogenase	K03946	KOG3446	123	92	89	419	172	5	7	12	106	29	139	109	93	88	13	8
ACC_03894	lysine-specific demethylase lid isoform 1	K11446	KOG1246	977	606	527	1582	769	278	654	672	1195	1172	456	344	479	656	1636	867
ACC_03895	plastin-3		KOG0046	575	355	364	704	369	159	429	312	525	401	143	129	127	122	288	248
ACC_03896	host cell factor 1-like	K14966	KOG4152	997	616	505	1169	624	146	327	378	1177	1332	431	422	795	1249	2040	1003
ACC_03897	synapse-associated protein of 47 kDa-like		KOG4310	5528	2440	2450	5051	1485	276	373	566	1788	1020	520	308	132	173	307	207
ACC_03898	GTP-binding protein Rit2-like isoform 1		KOG0395	153	114	91	63	65	7	6	10	118	77	16	48	57	50	6	2
ACC_03899	long-chain fatty acid transport protein 4-like	K08745	KOG1179	910	515	539	1026	783	105	153	169	4054	5658	700	2436	891	1543	220	71
ACC_03900	E3 SUMO-protein ligase RanBP2-like		KOG0546	13	11	8	19	19	1	4	4	5	5	9	10	6	1	3	0
ACC_03901	conserved hypothetical protein			261	159	193	217	101	10	15	16	239	182	60	84	187	130	50	12
ACC_03902	polypeptide N-acetylgalactosaminyltransferase 5-li	K00710	KOG3736	307	217	180	223	145	31	34	48	967	1084	110	170	660	616	128	42
ACC_03903	conserved hypothetical protein			120	67	89	249	139	3	7	13	26	26	17	45	0	3	0	1
ACC_03904	A disintegrin and metalloproteinase with thrombospondin m		KOG3538	124	109	135	409	188	18	16	32	298	329	104	141	18	7	6	4
ACC_03905	conserved hypothetical protein			161	54	55	86	44	3	7	13	280	257	27	163	121	115	9	2
ACC_03906	conserved hypothetical protein	K11657	KOG1984	16286	10764	10600	13225	12246	3410	7591	8571	11044	8903	6191	2263	2848	5816	14703	7039
ACC_03907	LOW QUALITY PROTEIN	K04712	KOG2987	336	161	174	366	457	15	31	37	310	145	231	338	497	657	26	11
ACC_03908	26S proteasome non-ATPase regulatory subunit 7-l	K03038	KOG1556	766	309	238	550	493	88	199	243	821	641	562	1191	2544	2795	1313	904
ACC_03909	l-threonine 3-dehydrogenase, mitochondrial-like	K15789	KOG2774	8	5	6	11	3	2	4	0	130	89	26	104	11	14	5	2
ACC_03910	ras-related protein Rab-18-B	K07910	KOG0080	255	155	175	322	316	29	20	22	416	208	166	294	424	406	30	13
ACC_03911	conserved hypothetical protein			9	7	23	198	90	1	1	2	30	4	1	0	168	137	17	1
ACC_03912	hypothetical protein			31	26	21	28	19	3	11	18	8	8	0	0	0	0	0	0
ACC_03913	conserved hypothetical protein		KOG3245	63	64	84	136	98	14	22	28	61	40	62	112	84	132	46	44
ACC_03914	conserved hypothetical protein			26	23	9	14	18	11	31	22	24	19	25	13	90	157	76	36
ACC_03915	cytochrome c oxidase subunit 4 isoform 1, mitocho	K02263	KOG4075	2561	1097	837	2591	2164	635	1085	1222	4610	1059	1869	4902	2946	4462	1547	1393
ACC_03916	conserved hypothetical protein		KOG2342	264	303	210	339	334	71	145	160	2237	548	243	585	2873	1687	127	36
ACC_03917	vacuolar protein sorting-associated protein 33A		KOG1302	374	205	206	369	373	15	19	25	432	280	163	521	335	329	21	7
ACC_03918	serine/threonine-protein kinase TAO1-like	K04429	KOG0577	746	409	342	558	383	163	310	351	744	559	259	237	327	381	668	417
ACC_03919	structure-specific endonuclease subunit SLX1 homo	K15078	KOG3005	67	46	47	108	96	4	5	17	40	31	35	40	71	64	9	14
ACC_03920	translocation protein SEC63 homolog isoform 1	K09540	KOG4434	988	630	420	657	777	144	271	303	1040	1056	466	703	3273	2781	804	393
ACC_03921	translational activator GCN1-like		KOG1242	615	286	225	421	297	78	210	207	602	965	168	249	955	813	747	541
ACC_03922	tetraspanin-7-like		KOG3882	338	792	211	310	262	179	143	246	231	511	51	174	72	32	5	0
ACC_03923	arrestin domain-containing protein 2		KOG3780	72	45	34	115	65	15	19	29	354	191	245	383	122	107	37	27
ACC_03924	LOW QUALITY PROTEIN		KOG3223	239	132	108	326	207	59	141	167	196	139	132	245	527	512	897	992
ACC_03925	conserved hypothetical protein		KOG1924	74	37	40	53	29	4	5	1	106	139	2	15	93	72	19	10
ACC_03926	Ecdysteroid UDP-glucosyltransferase			1	0	1	2	0	0	0	0	0	3	1	0	13	31	1	15
ACC_03927	protein LLP homolog		KOG4811	255	219	205	215	171	43	112	86	110	82	95	130	414	964	756	747
ACC_03928	alanyl-tRNA synthetase, cytoplasmic-like	K01872	KOG0188	695	485	345	389	434	98	180	212	682	707	170	393	1859	1752	199	127
ACC_03929	transmembrane protein 189-like	K10704	KOG3011	66	59	45	58	44	10	12	17	84	88	85	157	178	138	50	21
ACC_03930	alpha-1,3/1,6-mannosyltransferase ALG2-like	K03843	KOG0853	182	115	97	211	286	8	19	21	370	173	154	419	359	225	18	5

ACC_03931	receptor-type tyrosine-protein phosphatase kappa-like		KOG4228	40	27	27	38	16	5	15	17	224	177	3	1	13	12	15	2
ACC_03932	integrator complex subunit 5-like isoform 1	K13142		344	202	160	329	250	12	35	38	417	327	134	239	683	872	82	20
ACC_03933	regucalcin-like		KOG4499	2506	11450	2996	696	1915	3820	3135	7255	2374	1567	1125	1175	231	194	9	6
ACC_03934	regucalcin-like	K01053	KOG4499	208	278	181	327	378	20	31	26	1188	930	416	895	5249	3318	95	32
ACC_03935	pyrimidine-specific ribonucleoside hydrolase rihA-like		KOG2938	61	58	56	103	148	4	14	11	186	255	205	593	588	499	16	2
ACC_03936	conserved hypothetical protein			67	56	40	84	87	7	17	14	58	44	27	31	144	179	25	6
ACC_03937	d-arabinose 1-dehydrogenase-like		KOG1576	58	35	46	91	78	11	20	21	117	66	48	86	459	334	44	28
ACC_03938	cytochrome c-type heme lyase-like	K01764	KOG3996	903	448	379	904	613	138	245	309	1403	939	490	1242	1557	2089	862	495
ACC_03939	probable tubulin polyglutamylase TTLL1-like	K16599	KOG2157	9	1	2	9	4	0	1	1	3	0	1	10	0	0	2	1
ACC_03940	trimeric intracellular cation channel type B-like		KOG3944	125	63	48	132	128	8	16	14	346	184	45	132	431	331	27	11
ACC_03941	LOW QUALITY PROTEIN	K01551	KOG2825	251	153	136	360	417	41	42	56	811	405	323	880	1042	1071	67	28
ACC_03942	PHD finger and CXXC domain-containing protein C(	K14960	KOG1632	313	213	201	338	234	30	46	58	338	225	115	230	247	276	86	22
ACC_03943	60S ribosomal protein L35a isoform 2	K02917	KOG0887	1494	1513	751	2088	1237	346	821	1151	1432	1103	2189	2021	9735	7613	1916	1742
ACC_03944	SPRY domain-containing protein 7-like		KOG4030	218	118	103	336	248	40	42	66	503	219	302	784	433	528	123	22
ACC_03945	dynactin subunit 3	K10425		211	130	135	306	276	33	81	89	116	73	170	334	196	238	89	57
ACC_03946	protein KRTCAP2 homolog isoform 1		KOG4615	132	98	122	250	266	20	17	34	130	76	230	237	252	236	13	12
ACC_03947	chemosensory protein 1 precursor			701	747	360	1262	1453	71	73	60	13023	3806	27860	172300	17	11	2	0
ACC_03948	transmembrane protein 86A-like		KOG4804	168	83	77	80	85	8	17	18	141	65	53	125	35	27	9	2
ACC_03949	lysosomal aspartic protease	K01379	KOG1339	1315	902	878	2380	2448	116	93	184	2856	1551	1341	2427	2090	1279	69	28
ACC_03950	ATP-binding cassette sub-family D member 3-like	K05677	KOG0060	268	203	173	303	330	37	43	54	598	541	230	617	549	494	49	11
ACC_03951	protein archease-like		KOG4528	38	20	25	186	70	15	11	17	104	66	69	115	189	181	91	28
ACC_03952	transcription factor E2F2-like		KOG2577	210	162	187	231	191	20	24	47	330	271	108	256	450	326	92	42
ACC_03953	serine/threonine-protein kinase PINK1, mitochondrial-like		KOG4158	1922	875	851	2555	2085	231	380	445	3940	2239	1006	3435	3340	2539	458	193
ACC_03954	H/ACA ribonucleoprotein complex subunit 3-like	K11130	KOG3503	48	17	34	150	82	2	2	3	25	19	76	63	237	266	23	15
ACC_03955	dehydrodolichyl diphosphate synthase-like	K11778	KOG1602	176	174	165	157	206	12	58	57	156	189	107	406	430	387	56	23
ACC_03956	snurportin-1-like	K13151	KOG3132	216	139	118	290	323	18	29	27	206	146	194	204	195	208	39	17
ACC_03957	receptor expression-enhancing protein 5-like		KOG1725	243	188	118	315	366	37	28	59	568	339	482	1780	821	736	94	65
ACC_03958	adenylate kinase isoenzyme 6-like	K14535	KOG3347	97	109	104	181	185	17	24	29	180	79	117	225	314	434	74	39
ACC_03959	tetraspanin-33-like isoform 1		KOG3882	93	46	30	82	99	2	7	15	236	104	32	79	96	89	12	6
ACC_03960	mitogen-activated protein kinase kinase kinase 15-like		KOG2046	45	27	20	166	53	3	10	9	2077	512	70	95	468	245	70	57
ACC_03961	predicted protein	K11253	KOG1745	15	23	23	161	55	2	2	7	27	21	16	20	32	34	30	40
ACC_03962	GM13182	K11251	KOG1756	13	9	13	49	28	4	3	12	62	18	11	17	97	40	51	68
ACC_03963	u4/U6 small nuclear ribonucleoprotein Prp4	K12662	KOG0272	188	128	175	504	324	18	42	38	211	99	113	200	284	445	113	63
ACC_03964	nuclear pore complex protein Nup107	K14301	KOG1964	90	69	61	161	134	3	8	12	122	111	60	131	578	912	56	16
ACC_03965	hexosaminidase D-like isoform 1	K14459	KOG2499	441	223	230	199	194	11	14	17	459	438	78	382	404	287	28	3
ACC_03966	nucleoplasmin-like protein-like	K11278		1425	800	599	1200	520	191	284	247	1100	641	528	919	3979	8705	7328	2717
ACC_03967	histone H4	K11254	KOG3467	14	5	11	17	16	2	1	6	7	7	17	18	72	48	37	28
ACC_03968	histone H4	K11254	KOG3467	136	81	86	595	413	22	27	46	234	107	87	193	230	251	76	49
ACC_03969	histone H2B.3-like	K11252	KOG1744	101	91	80	135	93	23	19	48	245	265	49	95	469	455	438	323
ACC_03970	E3 ubiquitin-protein ligase CBL-B-like	K04707	KOG1785	110	41	58	64	53	1	6	5	172	138	29	110	83	105	16	7
ACC_03971	conserved hypothetical protein		KOG1166	1026	800	715	581	390	76	101	102	1263	1058	276	355	1429	1589	374	186
ACC_03972	conserved hypothetical protein			291	129	142	677	403	10	26	24	1078	643	34	45	983	747	76	19
ACC_03973	nuclear RNA export factor 1-like isoform 2	K14284	KOG3763	248	174	194	151	104	12	13	27	540	519	67	213	333	377	42	14
ACC_03974	conserved hypothetical protein		KOG0981	1957	929	1066	1475	336	71	90	154	858	413	655	256	13	21	75	35
ACC_03975	protein ariadne-2-like	K11969	KOG1815	304	197	176	243	208	18	25	27	403	284	91	274	155	134	24	5
ACC_03976	synaptojanin-1	K01099	KOG0566	997	601	704	798	507	53	75	96	649	493	151	309	305	291	68	20
ACC_03977	small glutamine-rich tetratricopeptide repeat-conta	K16365	KOG0553	722	480	415	650	440	86	93	124	887	974	345	1433	2054	1882	348	144
ACC_03978	5-aminolevulinate synthase, erythroid-specific, mit	K00643	KOG1360	1075	741	478	1896	1327	199	266	221	1696	1122	1782	2850	5038	8414	1463	488
ACC_03979	f-box only protein 6-like isoform 1			268	111	105	258	251	13	25	30	353	188	170	527	351	435	43	18
ACC_03980	dual specificity protein phosphatase CDC14A isofor	K06639	KOG1720	252	197	170	185	153	21	22	30	513	522	72	337	319	293	34	9
ACC_03981	dynamin-1-like protein	K01528	KOG0446	383	221	240	361	339	24	25	46	363	379	177	481	848	1079	117	38
ACC_03982	v-type proton ATPase 21 kDa proteolipid subunit-lil	K03661	KOG0233	289	117	113	263	387	20	24	26	348	160	282	1011	545	508	20	12
ACC_03983	conserved hypothetical protein		KOG4329	355	270	264	455	232	38	61	71	500	393	169	432	549	849	472	141
ACC_03984	LOW QUALITY PROTEIN		KOG0578	282	166	176	277	169	18	31	45	364	234	127	313	179	240	46	17
ACC_03985	trehalose transporter 1	K14258	KOG0254	281	215	271	1111	663	57	75	92	3588	4290	328	690	2776	1717	491	84
ACC_03986	methylmalonic aciduria and homocystinuria type D homolog		KOG3994	366	244	303	484	411	54	85	94	764	522	280	953	1311	1439	263	124
ACC_03987	syntenin-1-like		KOG3605	618	424	449	655	666	53	91	121	716	502	408	776	720	681	58	30

ACC_03988	protein brunelleschi-like		KOG1953	502	445	412	856	746	72	96	120	597	616	300	305	600	616	103	27
ACC_03989	acetyl-coenzyme A transporter 1-like	K03372	KOG3574	139	72	67	140	114	20	24	34	161	182	60	238	234	403	79	17
ACC_03990	ATP-dependent RNA helicase DDX42-like	K12835	KOG0339	594	442	453	1142	615	109	158	222	1032	468	418	435	599	765	267	167
ACC_03991	GMP synthase	K01951	KOG1622	562	346	301	688	346	65	91	105	675	586	181	581	1262	1340	445	156
ACC_03992	conserved hypothetical protein	K02961	KOG3447	182	163	149	507	421	11	31	50	375	168	241	423	784	1030	68	37
ACC_03993	conserved hypothetical protein		KOG1555	189	106	118	222	181	13	10	19	177	89	81	190	143	166	28	8
ACC_03994	cysteine desulfurase, mitochondrial-like	K04487	KOG1549	431	312	404	1135	942	47	54	88	767	460	294	758	950	1283	211	67
ACC_03995	N(G),N(G)-dimethylarginine dimethylaminohydrola	K01482		376	106	108	294	225	9	11	14	333	164	122	272	131	137	14	3
ACC_03996	conserved hypothetical protein		KOG0508	210	128	90	210	193	7	12	22	406	372	110	426	512	420	36	8
ACC_03997	tetraspanin 6		KOG3882	772	627	432	1252	1355	108	97	166	1688	757	1640	6176	1236	1033	75	10
ACC_03998	organic cation transporter 1-like		KOG0255	146	57	51	145	71	43	43	49	443	288	77	49	142	210	184	84
ACC_03999	conserved hypothetical protein			10	7	7	12	9	1	3	2	20	114	18	7	101	85	8	2
ACC_04000	zinc finger protein 208-like		KOG2462	1163	759	654	914	680	215	421	488	1380	1153	638	546	1034	1226	1312	715
ACC_04001	LOW QUALITY PROTEIN		KOG0962	1818	1174	981	955	1574	164	523	443	1085	1012	886	707	1305	1258	768	407
ACC_04002	conserved hypothetical protein			690	322	281	486	771	25	201	171	258	250	431	447	192	310	500	404
ACC_04003	conserved hypothetical protein	K14292	KOG2730	1304	747	756	1091	1099	209	403	400	1423	1111	657	568	1220	1277	894	300
ACC_04004	conserved hypothetical protein			12	29	52	177	100	2	4	4	90	112	42	705	42	44	2	5
ACC_04005	conserved hypothetical protein	K14409	KOG2162	889	780	682	658	527	90	153	168	612	565	132	298	444	379	139	33
ACC_04006	LOW QUALITY PROTEIN	K10595	KOG0939	2226	1313	1074	1964	1126	228	401	458	2451	2434	494	516	794	930	616	328
ACC_04007	protein-associating with the carboxyl-terminal domain of ez		KOG1243	818	463	413	757	808	86	197	227	674	465	388	854	922	868	279	113
ACC_04008	protein HIRA homolog	K11293	KOG0973	160	162	128	156	97	11	17	15	225	167	92	122	319	347	57	13
ACC_04009	glucose 1,6-bisphosphate synthase-like	K15779	KOG1220	175	129	115	138	179	4	9	15	297	305	69	250	631	474	21	6
ACC_04010	centrin-1		KOG0028	50	57	56	102	91	10	29	24	42	29	63	77	66	68	21	27
ACC_04011	endoribonuclease Dicer-like		KOG0701	2711	2358	2747	1884	1918	271	520	446	2629	2373	1111	1099	961	801	179	46
ACC_04012	conserved hypothetical protein	K14826	KOG0552	1330	451	310	720	853	162	432	508	438	280	461	336	3373	9077	13423	11911
ACC_04013	LOW QUALITY PROTEIN		KOG0161	121	100	116	178	131	15	28	36	152	70	67	22	86	203	92	70
ACC_04014	methenyltetrahydrofolate synthase domain-containing prot		KOG4410	75	42	46	44	67	6	25	30	88	54	71	122	208	138	46	33
ACC_04015	26S proteasome non-ATPase regulatory subunit 2-like		KOG2005	17	7	11	32	12	4	8	4	50	42	36	4	8	6	15	12
ACC_04016	protein downstream neighbor of son homolog		KOG4734	193	104	100	385	374	6	36	36	256	123	179	207	164	178	54	34
ACC_04017	ring canal kelch homolog	K10443	KOG4441	64	56	48	38	24	8	7	14	154	118	40	173	71	42	16	2
ACC_04018	conserved hypothetical protein		KOG2008	343	207	156	252	160	30	59	46	267	214	81	119	111	87	77	18
ACC_04019	COP9 signalosome complex subunit 8-like	K12181	KOG4414	196	128	134	577	304	45	52	86	287	172	192	374	388	441	286	109
ACC_04020	conserved hypothetical protein			0	1	1	0	1	0	0	0	1	2	1	3	2	1	0	1
ACC_04021	conserved hypothetical protein			117	91	79	84	106	2	13	4	42	35	21	26	520	589	132	32
ACC_04022	serine/threonine-protein kinase atr-like	K06640	KOG0890	433	343	284	282	249	38	76	79	480	423	104	136	419	453	153	61
ACC_04023	neogenin		KOG4221	404	254	214	109	86	18	25	28	583	417	106	142	305	323	51	18
ACC_04024	importin subunit beta-1 isoform 1	K14293	KOG1241	591	327	237	540	229	44	65	86	828	902	112	118	837	1311	725	348
ACC_04025	poly(A) RNA polymerase, mitochondrial-like		KOG2277	239	183	192	172	244	18	18	23	212	246	191	291	577	899	58	23
ACC_04026	thioredoxin domain-containing protein 15-like		KOG0190	363	202	269	698	662	33	30	53	423	313	375	949	423	540	86	24
ACC_04027	zinc finger protein 90-like		KOG2462	134	105	100	144	92	7	10	15	374	206	54	94	152	191	43	24
ACC_04028	conserved hypothetical protein		KOG4219	16	17	21	19	7	4	3	3	23	6	8	14	26	22	5	2
ACC_04029	non-structural maintenance of chromosome element 4-like isoform 2			733	448	426	761	894	110	291	243	497	338	435	582	764	1021	851	501
ACC_04030	protein FAM136A-like		KOG3377	70	32	27	64	55	10	16	13	40	14	37	36	279	592	157	177
ACC_04031	probable ATP-dependent RNA helicase DDX23	K12858	KOG0333	1008	732	633	1072	909	261	553	618	1007	641	727	707	1099	1232	1460	1440
ACC_04032	succinate dehydrogenase cytochrome b560 subuni	K00236	KOG0449	585	346	345	1353	783	40	67	53	1401	387	506	1456	1353	1049	130	53
ACC_04033	probable dimethyladenosine transferase-like	K14191	KOG0820	296	280	320	417	330	18	44	55	294	171	174	368	342	506	61	27
ACC_04034	SET and MYND domain-containing protein 4-like		KOG0548	264	171	136	180	211	28	149	105	395	220	269	238	217	144	52	38
ACC_04035	runt-related transcription factor 1-like		KOG3982	20	17	21	46	7	1	3	10	6	38	0	0	0	2	15	21
ACC_04036	glycolipid transfer protein domain-containing protein 1-like		KOG4189	201	91	72	198	171	17	20	35	111	52	45	268	598	327	39	13
ACC_04037	phosphatidylinositol-4-phosphate 5-kinase type-1 a	K00889	KOG0229	485	278	261	379	181	23	40	63	588	491	109	165	154	145	119	59
ACC_04038	tropomyosin 1 isoform B	K10373	KOG1003	1125	433	387	775	352	196	291	385	5407	2727	634	718	1199	896	1942	1853
ACC_04039	conserved hypothetical protein	K10803	KOG3226	276	131	107	269	375	30	110	135	158	109	254	291	346	351	267	126
ACC_04040	proteasome inhibitor PI31 subunit-like	K06700	KOG4761	395	212	218	673	516	54	42	67	617	273	459	1025	786	692	88	57
ACC_04041	conserved hypothetical protein			0	0	1	0	10	0	2	1	10	25	19	3	5	9	9	3
ACC_04042	conserved hypothetical protein			1	0	3	3	1	1	1	0	22	15	22	2	4	4	7	16
ACC_04043	conserved hypothetical protein			17	11	23	41	14	2	4	9	10	13	1	1	0	0	5	2
ACC_04044	28S ribosomal protein S24, mitochondrial			251	243	299	230	368	12	29	29	325	111	195	583	450	546	35	19

ACC_04045	acetylcholine receptor subunit alpha-like isoform 1		KOG3645	62	38	30	61	42	9	16	11	20	28	0	2	0	0	2	1
ACC_04046	aminopeptidase N-like		KOG1046	15316	13441	19445	41690	26932	531	684	1122	21723	10464	52	93	71	162	11	6
ACC_04047	guanine nucleotide-binding protein G(q) subunit al	K04634	KOG0085	225	252	223	527	217	80	121	188	922	1092	54	88	140	205	135	56
ACC_04048	conserved hypothetical protein		KOG4394	81	82	54	106	128	6	21	20	89	76	85	234	221	316	52	37
ACC_04049	hypothetical protein			9	2	4	15	4	0	2	1	3	0	0	0	0	0	5	0
ACC_04050	late secretory pathway protein AVL9 homolog		KOG3823	205	107	93	151	147	19	42	63	293	228	95	82	228	199	84	31
ACC_04051	tousled-like kinase	K08864	KOG1151	777	476	536	761	358	87	169	167	978	696	217	268	641	714	635	358
ACC_04052	conserved hypothetical protein			175	101	73	176	230	21	82	92	160	175	154	144	130	137	94	56
ACC_04053	NEDD8-activating enzyme E1 regulatory subunit-like		KOG2016	502	331	383	575	475	42	66	67	660	479	283	764	727	944	130	32
ACC_04054	replication factor C subunit 2	K10755	KOG0991	99	46	58	146	116	26	35	35	139	67	94	107	258	520	325	206
ACC_04055	formin-like protein CG32138-like		KOG1923	102	127	99	76	44	45	36	41	1220	972	107	203	189	177	39	21
ACC_04056	u4/U6 small nuclear ribonucleoprotein Prp31-like is	K12844	KOG2574	172	137	163	295	122	19	36	46	207	177	142	252	408	491	123	46
ACC_04057	sodium/potassium-transporting ATPase subunit be	K01540	KOG3927	44	27	22	35	30	8	17	5	394	222	135	195	298	255	57	20
ACC_04058	aspartate aminotransferase, mitochondrial isoform	K14455	KOG1411	805	671	543	1119	1104	89	107	114	2031	1392	458	2275	3677	2809	217	87
ACC_04059	LOW QUALITY PROTEIN	K14016	KOG1816	294	143	150	321	336	26	36	68	322	155	243	600	356	376	53	29
ACC_04060	cell cycle checkpoint control protein RAD9A-like	K10994	KOG2810	103	109	107	176	220	13	34	35	95	52	71	69	52	49	21	14
ACC_04061	endonuclease G, mitochondrial-like	K01173	KOG3721	98	63	66	112	106	8	12	18	261	134	86	200	242	252	17	10
ACC_04062	hypothetical protein			12	10	9	11	5	4	2	2	9	7	3	0	1	0	1	0
ACC_04063	ABC transporter G family member 20-like, partial		KOG0059	139	77	48	151	111	42	59	33	3317	2898	578	2085	349	438	17	21
ACC_04064	STAM-binding protein-like	K11866	KOG2880	342	201	142	304	274	71	127	139	361	252	163	204	391	355	314	228
ACC_04065	conserved hypothetical protein		KOG4674	510	425	377	814	322	130	188	209	958	750	229	292	158	213	298	117
ACC_04066	succinate dehydrogenase		KOG3049	15	23	14	32	23	2	5	6	42	30	9	16	99	59	16	9
ACC_04067	conserved hypothetical protein			18	6	13	14	4	1	0	0	13	5	0	0	0	0	2	0
ACC_04068	inositol polyphosphate 5-phosphatase K-like	K01106	KOG0566	241	119	123	157	195	5	7	13	277	236	86	566	158	117	7	0
ACC_04069	cell cycle checkpoint protein RAD17-like	K06662	KOG1970	267	271	248	466	517	17	38	56	212	114	142	310	165	147	30	16
ACC_04070	ras-related protein M-Ras-like	K07831	KOG0395	113	57	44	45	37	21	40	46	129	104	16	59	61	52	43	30
ACC_04071	neuroligin 5		KOG1516	19	11	7	27	3	1	2	3	10	11	0	0	0	0	0	2
ACC_04072	conserved hypothetical protein			102	85	80	181	150	10	14	14	245	157	127	260	192	263	39	12
ACC_04073	transmembrane protein 145-like		KOG4290	135	107	121	188	197	14	22	38	108	146	136	163	100	122	12	3
ACC_04074	conserved hypothetical protein			182	126	108	224	154	25	30	41	419	325	91	326	250	263	56	15
ACC_04075	calcium-transporting ATPase type 2C member 1-like	K01537	KOG0202	1102	535	488	958	770	28	47	61	1184	878	408	911	946	1024	92	19
ACC_04076	Deoxyuridine 5'-triphosphate nucleotidohyd	K01520	KOG3370	72	51	20	64	45	12	19	19	36	27	14	37	411	682	384	326
ACC_04077	protein N-terminal glutamine amidohydrolase-like		KOG3261	667	418	375	164	103	16	17	28	431	212	36	65	152	117	30	14
ACC_04078	conserved hypothetical protein		KOG4362	320	163	117	181	386	18	48	45	111	139	112	101	226	189	168	72
ACC_04079	transmembrane protein 63A-like		KOG1134	590	363	318	598	485	71	68	84	981	780	385	939	832	720	104	22
ACC_04080	n-alpha-acetyltransferase 30, NatC catalytic subuni	K00670	KOG3139	135	108	91	136	134	14	11	20	425	358	104	467	288	300	53	19
ACC_04081	mediator of RNA polymerase II transcription subun	K15151	KOG3046	69	103	78	161	172	6	16	23	79	41	96	194	88	125	29	27
ACC_04082	conserved hypothetical protein		KOG3702	393	229	253	371	238	51	110	138	289	256	109	197	274	232	254	135
ACC_04083	guanine nucleotide exchange factor for Rab-3A-like	K16779	KOG4324	445	205	218	368	263	19	33	37	799	676	79	572	190	134	34	18
ACC_04084	juvenile hormone epoxide hydrolase 1-like	K10719	KOG2565	325	306	207	557	416	45	54	70	2221	604	3600	4031	6064	5537	299	45
ACC_04085	gem-associated protein 6-like	K13134		55	40	44	34	31	0	1	6	25	33	24	68	96	102	8	0
ACC_04086	Reticulon-1		KOG1792	1210	691	438	1002	791	462	866	1310	470	299	776	522	303	229	569	413
ACC_04087	LOW QUALITY PROTEIN	K00688	KOG2099	1673	1008	714	1918	1769	225	485	470	2966	1694	423	671	5733	3378	1046	1003
ACC_04088	galectin-8-like		KOG3587	161	104	112	282	244	8	9	12	312	210	72	529	269	205	17	7
ACC_04089	conserved hypothetical protein	K11268	KOG3014	59	57	52	100	104	7	26	26	61	37	58	61	140	252	132	64
ACC_04090	conserved hypothetical protein			0	0	0	0	0	0	0	0	0	0	0	1	0	0	0	0
ACC_04091	guanine nucleotide exchange factor DBS		KOG4240	458	316	257	195	169	16	25	28	522	403	90	276	268	188	21	5
ACC_04092	adenylate kinase 2, mitochondrial-like	K00939	KOG3078	489	254	198	394	418	27	50	44	1036	778	257	1585	1319	1490	116	47
ACC_04093	cullin-4B-like	K10609	KOG2167	712	516	465	496	397	103	141	177	1262	921	313	545	1068	1191	175	48
ACC_04094	LOW QUALITY PROTEIN	K11407	KOG1343	789	431	408	776	744	69	123	134	1376	1266	484	1212	992	933	245	78
ACC_04095	protein CLP1 homolog	K14399	KOG2749	122	49	57	322	244	12	11	12	128	48	107	164	202	292	66	30
ACC_04096	solute carrier organic anion transporter family member 1A5-		KOG3626	8	1	1	2	0	0	0	1	1	5	0	0	218	460	153	11
ACC_04097	alpha glucosidase		KOG0471	189	9535	130370	36367	40134	1049	2445	1379	1164	870	230	451	136	439	70	27
ACC_04098	gem-associated protein 7-like	K13135		51	61	41	103	79	16	26	17	112	60	80	165	102	101	20	10
ACC_04099	Dosage compensation complex, subunit MLE		KOG0921	173	99	104	107	27	20	27	34	113	388	46	31	10	18	42	42
ACC_04100	probable ATP-dependent RNA helicase DHX34-like		KOG0922	622	432	344	517	486	30	52	45	465	638	210	355	567	457	58	15
ACC_04101	esterase E4-like		KOG1516	37	6	27	44	42	20	14	26	357	415	7	99	9	8	2	1

ACC_04102	conserved hypothetical protein	K09067	KOG4029	34	26	32	27	8	5	2	7	17	12	4	4	6	4	16	3
ACC_04103	conserved hypothetical protein		KOG2504	67	21	26	111	55	19	5	5	107	38	4	12	178	145	75	11
ACC_04104	conserved hypothetical protein			39	22	33	86	35	0	2	3	29	21	10	4	74	176	48	6
ACC_04105	zinc finger HIT domain-containing protein 1-like	K11663	KOG3362	66	64	52	178	165	3	10	16	48	36	115	132	102	168	37	19
ACC_04106	inositol polyphosphate multikinase-like	K00328	KOG1620	246	167	195	362	222	37	33	30	356	137	117	88	118	104	54	9
ACC_04107	conserved hypothetical protein		KOG3608	919	667	593	799	847	158	457	456	1292	1111	713	591	853	842	616	240
ACC_04108	coiled-coil domain-containing protein 6-like	K09288	KOG2129	538	299	218	393	418	118	181	211	574	535	223	412	365	406	382	269
ACC_04109	diacylglycerol kinase eta-like	K00901	KOG1170	484	318	258	487	235	12	30	38	345	400	77	119	152	147	64	17
ACC_04110	protein disulfide-isomerase	K09580	KOG0190	1818	1413	747	1437	1569	265	357	424	2945	2608	714	3114	13844	10219	664	340
ACC_04111	conserved hypothetical protein			60	42	33	28	60	5	20	23	38	30	61	56	148	89	76	44
ACC_04112	Bardet-Biedl syndrome 5 protein homolog	K16748		160	145	121	248	248	10	20	21	83	42	109	215	21	30	5	0
ACC_04113	conserved hypothetical protein	K15198	KOG2009	1087	799	685	1210	1242	214	496	517	834	444	521	419	376	478	421	221
ACC_04114	conserved hypothetical protein			43	54	50	70	77	1	4	4	24	44	52	118	136	107	19	6
ACC_04115	DNA methyltransferase 1a	K00558	KOG0919	230	228	280	135	182	30	88	82	161	101	146	30	127	197	206	92
ACC_04116	phosphoacetylglucosamine mutase-like	K01836	KOG2537	100	83	37	81	106	19	16	25	137	164	106	272	1055	1001	63	12
ACC_04117	conserved hypothetical protein		KOG0515	121	18	5	12	6	2	6	5	197	14	9	317	38	158	21	27
ACC_04118	male-specific lethal 3 homolog		KOG3001	148	141	123	196	150	16	25	31	154	112	102	110	245	305	58	28
ACC_04119	f-box/WD repeat-containing protein 5-like	K10263	KOG0293	349	189	190	337	266	19	34	41	299	268	91	184	169	114	50	26
ACC_04120	WD repeat-containing protein 82-like	K14962	KOG1446	103	83	44	38	28	11	6	11	194	206	15	139	213	264	37	8
ACC_04121	u6 snRNA-associated Sm-like protein LSm4-like	K12623	KOG3293	229	292	308	461	353	42	95	116	222	85	155	257	324	543	272	304
ACC_04122	protein bicaudal D		KOG0999	81	56	46	108	24	11	14	19	196	215	26	109	120	109	62	32
ACC_04123	conserved hypothetical protein			132	89	99	178	46	14	29	20	672	423	4	7	24	32	52	13
ACC_04124	anamorsin homolog		KOG4020	164	105	153	475	693	4	12	10	134	159	136	271	254	251	14	5
ACC_04125	conserved hypothetical protein			1	1	1	1	2	0	0	0	0	2	0	0	2	1	2	0
ACC_04126	patj homolog			92	48	26	49	21	1	6	4	100	117	18	59	46	45	4	4
ACC_04127	conserved hypothetical protein		KOG0933	51	52	48	21	20	7	7	13	31	11	6	3	1	1	7	3
ACC_04128	conserved hypothetical protein	K10605	KOG4362	118	144	122	135	67	14	16	34	233	317	29	37	126	146	87	60
ACC_04129	ras-like protein 2-like	K07830	KOG0395	247	172	166	294	338	112	183	280	281	190	182	354	214	218	49	23
ACC_04130	monocarboxylate transporter 12-like		KOG2504	186	267	162	246	247	122	187	209	519	308	220	178	37	26	80	29
ACC_04131	thioredoxin domain-containing protein 9-like		KOG1672	150	118	93	236	254	4	21	31	295	212	158	668	480	527	21	13
ACC_04132	phosphorylated CTD-interacting factor 1-like			100	78	67	73	51	7	9	15	147	127	28	55	124	144	55	11
ACC_04133	cyclin-Y-like		KOG1675	43	37	25	53	51	2	8	6	122	117	24	127	83	92	12	2
ACC_04134	scavenger mRNA-decapping enzyme DcpS-like isof	K12584	KOG3969	344	209	181	328	380	15	44	50	464	378	291	776	833	820	101	71
ACC_04135	conserved hypothetical protein			1054	671	525	1253	1174	182	512	521	944	729	903	506	770	946	1509	798
ACC_04136	60S ribosomal protein L23a	K02893	KOG1751	5995	4747	2598	4439	2038	1606	2836	3697	2872	2889	2509	3384	19718	24607	17299	14180
ACC_04137	WASH complex subunit 7-like		KOG3578	422	297	331	435	391	36	57	70	532	558	162	485	505	333	50	14
ACC_04138	protein FAM86A-like, partial		KOG3201	216	179	156	208	223	14	20	21	184	117	126	207	148	168	30	12
ACC_04139	60S ribosomal export protein NMD3	K07562	KOG2613	272	192	196	260	264	27	48	63	460	224	250	438	578	987	53	44
ACC_04140	sphingomyelin phosphodiesterase 1-like		KOG3770	13	9	16	29	35	0	2	1	3	17	4	1	306	420	18	3
ACC_04141	rab5 GDP/GTP exchange factor-like		KOG2319	596	348	341	605	587	71	141	140	461	290	296	384	436	380	178	122
ACC_04142	activator of 90 kDa heat shock protein ATPase homolog 1-lik		KOG2936	846	610	753	1860	1552	76	127	186	625	536	317	1217	1385	2304	407	208
ACC_04143	ATP-dependent RNA helicase DDX51-like	K14807	KOG0350	1029	489	409	721	978	129	211	249	601	534	334	521	1031	1868	893	719
ACC_04144	PHD finger-like domain-containing protein 5A-like	K12834	KOG1705	25	33	30	93	76	0	5	6	30	18	59	60	98	158	20	12
ACC_04145	tweedle motif cuticular protein 2		KOG0260	15	15	5	41	6	2	0	0	47	48	15	11	315	1437	198	861
ACC_04146	trafficking kinesin-binding protein milt	K15369	KOG4360	945	514	516	1346	672	151	199	230	1635	1291	562	686	712	442	276	59
ACC_04147	probable alpha-ketoglutarate-dependent dioxygen	K10766	KOG3959	130	86	94	190	204	16	15	11	154	74	88	228	230	314	26	13
ACC_04148	peptidoglycan-recognition protein SA precursor			24	27	33	123	89	10	13	16	448	640	6	11	14	4	0	0
ACC_04149	LOW QUALITY PROTEIN	K04662	KOG3900	173	67	61	137	106	17	24	23	653	394	93	270	341	338	92	22
ACC_04150	cob(I)yrinic acid a,c-diamide adenosyltransferase, r	K00798		237	123	158	427	536	15	26	36	227	136	127	307	276	359	29	13
ACC_04151	beta-glucuronidase-like	K01195	KOG2024	219	74	61	161	100	10	24	18	103	128	23	14	534	1478	292	50
ACC_04152	protein deltex	K06058		284	169	153	242	204	41	69	90	679	259	164	297	359	418	131	70
ACC_04153	conserved hypothetical protein		KOG3608	156	127	119	237	196	11	25	33	130	76	80	128	155	161	39	13
ACC_04154	G patch domain and ankyrin repeats-containing protein 1 ho		KOG2384	333	246	278	404	393	42	100	121	289	162	169	253	283	346	120	66
ACC_04155	protein misato-like		KOG2530	123	70	73	112	122	8	7	23	81	85	43	73	268	357	37	11
ACC_04156	calcium and integrin-binding family member 3-like		KOG0038	41	32	28	96	82	3	5	15	28	14	32	13	43	61	25	10
ACC_04157	heat shock protein 83-like	K04079	KOG0019	1718	702	2228	2274	2575	210	471	838	949	1048	543	656	5730	10646	14898	19467
ACC_04158	integrin-alpha FG-GAP repeat-containing protein 2-like isofo		KOG1568	527	431	444	660	629	88	163	141	1229	719	404	606	1093	1279	290	80

ACC_04159	DNA-directed RNA polymerase I subunit RPA12-like	K03000	KOG2907	32	21	15	53	68	9	8	14	92	50	32	58	162	207	23	13
ACC_04160	protein Wnt-1 isoform 1	K03209	KOG3913	0	0	0	1	0	0	0	0	1	2	0	0	3	2	23	10
ACC_04161	protein Wnt-10b	K01357	KOG3913	2	1	1	6	1	0	2	4	0	9	1	0	2	2	18	10
ACC_04162	conserved hypothetical protein			34	48	39	63	61	5	7	7	25	23	275	309	40	39	12	2
ACC_04163	e3 ubiquitin-protein ligase Nedd-4	K10591	KOG0939	1016	689	603	508	483	122	161	123	1334	1450	437	487	1178	862	87	37
ACC_04164	zinc finger protein ZPR1	K06874	KOG2703	282	183	148	359	296	34	55	84	495	357	238	550	761	1052	197	67
ACC_04165	malate dehydrogenase, mitochondrial-like	K00026	KOG1494	1700	800	775	2260	1469	206	205	313	2490	1325	1020	2898	9715	7371	1548	557
ACC_04166	conserved hypothetical protein		KOG1924	702	296	337	661	550	22	42	58	441	239	132	68	105	169	55	21
ACC_04167	signal peptide peptidase-like 3-like isoform 1	K09598	KOG2443	70	47	36	73	48	6	4	1	176	174	33	125	178	162	45	10
ACC_04168	Longitudinals lacking protein, isoform G		KOG4441	151	138	129	98	35	15	12	12	300	224	42	120	85	59	27	17
ACC_04169	LIM homeobox transcription factor 1-beta-like		KOG4577	4	7	12	17	5	3	5	5	14	18	2	4	9	2	9	5
ACC_04170	conserved hypothetical protein		KOG0998	444	260	220	553	410	73	66	106	534	416	214	445	677	612	145	57
ACC_04171	DUOXA-like protein C06E1.3-like		KOG3921	36	36	48	126	67	4	6	10	167	82	40	110	162	194	57	16
ACC_04172	thioredoxin domain-containing protein 17		KOG3425	100	91	61	106	145	23	25	24	208	121	86	340	1005	1277	47	21
ACC_04173	calcium release-activated calcium channel protein :	K16056	KOG4298	201	128	108	232	168	20	19	29	423	275	106	271	165	129	29	4
ACC_04174	oxysterol-binding protein-related protein 8-like		KOG2210	1037	587	618	672	468	42	74	83	1355	1171	342	1306	442	328	63	14
ACC_04175	conserved hypothetical protein		KOG0307	571	727	847	387	155	42	37	58	587	511	93	169	646	580	366	152
ACC_04176	conserved hypothetical protein		KOG3158	65	68	47	27	38	2	6	5	10	35	31	50	102	79	10	9
ACC_04177	serine/threonine-protein phosphatase 2A activator		KOG2867	362	298	326	548	444	16	34	32	499	249	111	405	291	356	46	13
ACC_04178	cyclin-related protein FAM58A-like isoform 1		KOG0834	136	87	87	271	243	9	10	17	165	151	97	416	617	970	86	34
ACC_04179	conserved hypothetical protein		KOG0352	241	136	123	198	185	9	23	33	97	68	63	78	118	170	34	13
ACC_04180	monocarboxylate transporter 13-like		KOG2504	528	262	289	1893	1083	114	144	204	614	360	262	230	30	29	42	13
ACC_04181	F-box only protein 21-like	K10301		541	352	393	418	415	57	115	114	1179	964	452	1060	653	633	106	46
ACC_04182	lys-63-specific deubiquitinase BRCC36-like	K11864	KOG1555	165	95	80	219	243	21	25	35	209	124	138	323	274	402	38	16
ACC_04183	multiple inositol polyphosphate phosphatase 1-like	K03103	KOG1382	123	106	77	117	143	11	27	25	77	62	85	148	132	161	34	11
ACC_04184	carbonic anhydrase-related protein 10-like		KOG0382	203	101	151	268	76	21	39	57	38	35	6	2	7	14	35	46
ACC_04185	conserved hypothetical protein		KOG3539	127	35	73	117	15	1	1	3	52	61	2	5	5	5	9	2
ACC_04186	galactoside 2-alpha-L-fucosyltransferase 1-like	K00718		178	101	111	135	104	10	2	18	244	186	59	154	125	71	18	1
ACC_04187	ubiquitin domain-containing protein 2-like		KOG0013	97	47	32	58	31	17	18	14	432	372	56	363	545	291	26	17
ACC_04188	nuclear receptor-binding factor 2-like			154	120	161	174	97	25	26	27	128	105	37	99	84	88	14	1
ACC_04189	conserved hypothetical protein	K05747	KOG3671	560	610	658	765	466	63	106	146	628	483	184	230	386	490	219	98
ACC_04190	argininosuccinate lyase-like	K01755	KOG1316	71	22	30	39	44	5	14	8	375	483	30	10	2808	517	25	13
ACC_04191	estradiol 17-beta-dehydrogenase 12-like	K10251	KOG1014	58	45	24	86	70	4	8	21	76	93	37	55	185	97	8	12
ACC_04192	LOW QUALITY PROTEIN		KOG2787	487	405	362	756	675	47	92	97	715	474	455	740	372	435	66	23
ACC_04193	conserved hypothetical protein			40	42	29	207	119	0	3	8	43	22	99	286	150	1023	39	66
ACC_04194	tyrosine-protein phosphatase non-receptor type 4	K01104	KOG0792	79	55	54	108	93	3	16	15	198	188	27	71	106	90	24	5
ACC_04195	transcription initiation factor IIB isoform 1	K03124	KOG1597	88	55	52	112	77	9	10	10	274	172	92	298	331	258	15	4
ACC_04196	exocyst complex component 4-like	K06111	KOG3691	47	23	25	83	64	1	6	5	21	14	47	79	57	84	7	5
ACC_04197	NIF3-like protein 1-like		KOG4131	121	56	52	64	87	9	17	13	158	120	73	220	335	317	25	8
ACC_04198	CDK-activating kinase assembly factor MAT1-like is	K10842	KOG3800	201	165	140	219	193	42	79	94	209	144	137	255	325	441	290	231
ACC_04199	conserved hypothetical protein			133	110	112	161	118	6	21	18	103	76	28	86	86	83	26	12
ACC_04200	nuclear pore glycoprotein p62	K14306	KOG2196	181	181	151	360	176	20	35	47	304	198	138	309	565	818	156	93
ACC_04201	PX domain-containing protein kinase-like protein-like		KOG0575	713	457	405	760	457	93	137	163	1229	791	215	703	558	426	188	49
ACC_04202	conserved hypothetical protein	K04511	KOG1924	34	24	27	56	15	3	0	7	52	55	35	29	27	49	86	15
ACC_04203	conserved hypothetical protein			56	40	40	36	75	8	19	18	38	48	38	53	162	140	21	2
ACC_04204	f-box only protein 33-like	K10310	KOG3740	870	894	855	580	188	64	69	101	1014	820	153	215	387	485	267	72
ACC_04205	glutathione S-transferase omega-1	K00799	KOG0406	137	112	115	267	223	31	34	26	313	218	148	521	855	914	134	46
ACC_04206	peroxidasin		KOG2408	998	755	376	361	272	175	192	191	838	539	123	299	530	713	118	39
ACC_04207	homeodomain-only protein-like			51	94	113	474	255	29	41	83	666	229	283	319	36	26	13	4
ACC_04208	conserved hypothetical protein		KOG1198	803	294	370	887	440	43	45	67	2519	2396	346	1653	221	264	94	20
ACC_04209	conserved hypothetical protein		KOG1054	398	160	172	300	114	22	30	51	202	79	7	2	4	18	28	23
ACC_04210	DNA damage-regulated autophagy modulator protein 2-like		KOG4320	1	1	1	1	0	1	0	0	0	3	2	2	3	4	5	1
ACC_04211	lachesin-like, partial		KOG3513	36	14	14	31	6	3	1	2	12	27	0	1	6	2	7	8
ACC_04212	DNA repair protein complementing XP-A cells hom	K10847	KOG4017	235	156	178	355	340	31	60	83	272	185	313	405	379	398	86	44
ACC_04213	translocon-associated protein subunit beta isoform	K13250	KOG3317	642	374	179	553	507	74	80	99	860	516	358	939	2597	3861	202	108
ACC_04214	collagen type IV alpha-3-binding protein-like	K08283	KOG1739	223	160	139	329	220	32	58	47	419	321	148	270	470	454	89	45
ACC_04215	conserved hypothetical protein			172	112	146	937	364	70	124	153	557	505	426	522	99	54	328	177

ACC_04216	bolA-like protein DDB_G0274169-like	K05527	KOG2313	33	25	30	37	43	3	9	7	18	33	34	29	53	67	15	14
ACC_04217	lipid storage droplet-1			28	28	50	78	54	0	2	4	793	827	9	34	1073	262	9	12
ACC_04218	lipid storage droplets surface-binding protein 2-like			51	97	98	167	136	4	2	7	116	53	31	127	71	101	12	14
ACC_04219	lipid storage droplets surface-binding protein 2-like			119	48	67	91	134	19	44	44	135	116	73	202	465	449	64	54
ACC_04220	hypothetical protein			0	0	0	0	0	0	0	0	0	0	0	0	0	0	0	0
ACC_04221	LOW QUALITY PROTEIN		KOG3622	1280	936	757	1209	961	79	116	169	1323	1141	397	452	568	463	74	22
ACC_04222	protein fem-1 homolog B-like	K10349	KOG0508	231	164	148	239	196	12	16	19	267	341	88	192	275	223	29	16
ACC_04223	Werner Syndrome-like exonuclease-like, partial		KOG4373	167	124	145	133	221	5	37	23	144	129	144	484	273	247	42	26
ACC_04224	putative lipoyltransferase 2, mitochondrial	K03801	KOG0325	123	64	87	154	198	4	23	11	87	69	95	267	155	150	18	11
ACC_04225	protein TAPT1 homolog		KOG2490	172	96	58	148	128	14	21	11	330	336	113	449	377	268	64	24
ACC_04226	conserved hypothetical protein		KOG3538	185	47	49	75	63	1	3	16	72	44	10	24	7	22	8	3
ACC_04227	COMM domain-containing protein 3-like			94	58	68	99	116	5	8	10	57	82	70	161	255	287	19	4
ACC_04228	conserved hypothetical protein			138	126	119	220	224	11	31	24	67	99	85	294	340	417	94	56
ACC_04229	heparin sulfate O-sulfotransferase	K02513	KOG3922	132	107	128	328	336	20	24	32	131	51	67	118	107	167	30	11
ACC_04230	conserved hypothetical protein		KOG2462	544	325	358	728	437	48	57	75	708	581	208	485	884	818	270	155
ACC_04231	conserved hypothetical protein		KOG4177	218	169	142	244	307	12	44	62	112	163	118	104	216	244	46	29
ACC_04232	LOW QUALITY PROTEIN		KOG0161	794	528	398	478	836	73	193	226	334	243	1026	433	170	165	362	142
ACC_04233	facilitated trehalose transporter Tret1-like isoform 3		KOG0254	244	175	174	320	283	18	38	31	774	435	236	165	543	667	48	11
ACC_04234	phosphoribosyl pyrophosphate synthase-associated protein		KOG1503	230	136	118	219	183	23	25	28	356	177	156	343	247	452	51	19
ACC_04235	HEAT repeat-containing protein 2 isoform 1	K12462	KOG3205	479	222	206	442	371	21	27	52	461	318	164	493	588	919	93	76
ACC_04236	fructose-1,6-bisphosphatase 1-like	K03841	KOG1458	31	18	22	47	25	2	6	8	20	56	15	35	10	7	12	4
ACC_04237	hypothetical protein			1	0	1	3	2	0	0	0	0	0	0	0	0	0	0	0
ACC_04238	Predicted ubiquitin-protein ligase/hyperplastic discs protein,		KOG0943	948	557	637	628	539	130	350	302	635	347	195	83	211	409	568	282
ACC_04239	hypothetical protein			30	59	27	7	4	2	1	6	3	11	2	3	0	1	3	0
ACC_04240	S phase cyclin A-associated protein in the endoplasmic reticu		KOG4722	1004	456	351	744	683	161	355	380	739	644	488	248	96	67	507	235
ACC_04241	insulin gene enhancer protein ISL-1-like	K09370	KOG4577	46	12	14	29	15	0	3	1	36	19	3	0	25	17	19	1
ACC_04242	conserved hypothetical protein			16	6	4	13	4	1	5	7	4	13	8	0	0	0	10	3
ACC_04243	conserved hypothetical protein		KOG0274	40	11	5	12	12	2	5	9	4	7	3	0	0	1	5	0
ACC_04244	hypothetical protein			56	29	34	19	8	1	2	9	10	8	1	0	1	1	0	0
ACC_04245	Acetylcholine receptor subunit alpha-like		KOG3646	29	18	20	18	31	1	6	2	10	5	0	0	0	0	0	0
ACC_04246	amphiphysin-like		KOG3771	397	440	407	306	179	48	48	55	1555	1049	221	860	333	238	35	19
ACC_04247	hypothetical protein			16	15	20	11	11	4	4	6	48	9	17	17	17	9	0	0
ACC_04248	uridine phosphorylase 1-like	K00757	KOG3728	449	155	163	98	71	24	31	37	751	1203	72	273	231	139	27	6
ACC_04249	hypothetical protein			0	1	0	2	1	0	1	2	1	3	2	0	0	0	1	0
ACC_04250	NADPH-dependent diflavin oxidoreductase 1-like		KOG1159	380	179	169	242	212	18	34	26	442	300	114	314	466	396	38	14
ACC_04251	conserved hypothetical protein		KOG3558	16	12	14	53	28	2	8	6	27	20	11	8	10	9	3	8
ACC_04252	conserved hypothetical protein	K15144	KOG0956	411	379	382	323	125	38	23	43	1048	952	71	307	279	271	191	30
ACC_04253	conserved hypothetical protein	K00698	KOG2571	8	2	0	1	0	3	1	6	87	10	1	30	450	271	58	29
ACC_04254	probable 39S ribosomal protein L45, mitochondrial		KOG4599	359	216	217	457	418	48	69	86	466	219	335	472	689	994	240	122
ACC_04255	protein ariadne-1 homolog	K11968	KOG1815	622	284	266	358	301	47	86	92	1394	1052	233	695	848	661	105	73
ACC_04256	synaptic vesicle glycoprotein 2B-like isoform 1		KOG0253	669	322	429	1568	820	28	32	45	1047	495	77	1785	665	757	378	50
ACC_04257	conserved hypothetical protein			234	137	158	248	167	8	21	30	280	239	72	212	377	240	52	14
ACC_04258	leucine-rich repeats and immunoglobulin-like domains prote		KOG4194	538	309	301	512	350	67	123	132	671	469	169	184	248	204	192	72
ACC_04259	conserved hypothetical protein			26	33	39	56	43	9	6	7	33	34	1	1	3	8	4	3
ACC_04260	conserved hypothetical protein		KOG0505	271	282	279	243	90	50	60	75	366	373	56	115	262	185	235	122
ACC_04261	conserved hypothetical protein	K01090	KOG0505	143	86	90	170	166	29	51	58	360	210	64	59	166	125	84	57
ACC_04262	guanine nucleotide-binding protein subunit beta-5	K04539	KOG0286	211	132	150	361	305	16	29	37	265	111	162	386	133	141	28	6
ACC_04263	ubiquitin carboxyl-terminal hydrolase 14-like isofor	K11843	KOG1872	534	198	189	480	377	67	131	141	553	388	260	808	831	1217	398	238
ACC_04264	26S proteasome non-ATPase regulatory subunit 6-l	K03037	KOG0687	319	189	160	444	539	25	32	65	634	409	343	939	1138	1518	105	67
ACC_04265	tyrosine-protein kinase Src42A-like isoform 1	K08892	KOG0197	614	306	237	361	291	22	25	34	588	413	112	323	401	469	46	12
ACC_04266	60S ribosomal protein L38	K02923	KOG3499	731	438	333	1726	441	82	182	220	171	183	1111	485	1570	1593	522	305
ACC_04267	tektin-2-like		KOG2685	158	131	151	158	184	7	25	23	92	33	169	345	25	35	9	0
ACC_04268	conserved hypothetical protein	K12484	KOG1028	423	289	217	589	443	278	513	532	992	567	740	1051	218	232	361	255
ACC_04269	e3 ubiquitin-protein ligase Siah1	K04506	KOG3002	112	63	53	89	82	9	3	5	257	232	51	292	145	80	19	2
ACC_04270	conserved hypothetical protein			96	69	57	122	75	4	11	13	226	277	48	225	265	281	33	13
ACC_04271	conserved hypothetical protein		KOG4784	85	47	64	86	134	1	2	6	33	80	42	109	98	65	13	1
ACC_04272	tubulin polyglutamylase TTLL13-like	K00273	KOG3923	8	3	4	4	1	1	2	0	4	18	1	1	30	4	1	0

ACC_04273	hypothetical protein			1	0	1	0	0	0	0	0	1	1	1	0	0	0	0	0
ACC_04274	39S ribosomal protein L32, mitochondrial	K02911	KOG4080	190	203	226	269	293	28	51	62	238	139	235	404	743	991	196	192
ACC_04275	J domain-containing protein CG6693-like isoform 1	K09529	KOG0719	152	128	122	147	105	24	47	36	1244	786	165	345	110	172	88	44
ACC_04276	tiggy-winkle hedgehog protein-like	K06224	KOG3638	2	2	1	2	0	0	0	0	10	6	1	4	7	3	9	5
ACC_04277	carbonic anhydrase 2-like	K01672	KOG0382	17	2	6	24	14	2	0	1	102	80	312	1307	688	358	31	2
ACC_04278	signal recognition particle 19 kDa protein	K03105	KOG3198	161	174	135	236	162	38	59	77	406	296	93	422	360	287	114	74
ACC_04279	tubulin alpha-2 chain-like		KOG1376	7	8	3	18	5	0	0	1	13	8	8	5	7	9	1	3
ACC_04280	UPF0368 protein Cxorf26-like		KOG4093	104	89	93	144	134	9	16	16	47	45	55	71	131	298	68	30
ACC_04281	hypothetical protein			5	3	3	6	0	1	3	4	3	3	0	0	0	1	3	0
ACC_04282	plasma glutamate carboxypeptidase-like isoform 1		KOG2195	542	373	387	906	657	52	81	96	628	553	102	274	163	353	91	71
ACC_04283	plasma glutamate carboxypeptidase-like		KOG2195	16287	9896	6730	1371	1001	1842	1933	2299	208	48	11	25	11	54	11	15
ACC_04284	plasma glutamate carboxypeptidase-like		KOG2195	1034	556	409	73	70	148	239	291	70	39	3	12	5	10	3	0
ACC_04285	hypothetical protein			85	30	29	81	58	9	17	21	21	5	1	0	0	0	5	1
ACC_04286	microtubule-associated protein 2-like, partial		KOG1144	454	269	377	270	59	20	24	32	554	619	20	115	53	23	20	10
ACC_04287	conserved hypothetical protein	K04380	KOG2418	667	395	322	369	126	36	61	60	736	615	56	213	97	58	60	27
ACC_04288	TPX protein	K03386	KOG0852	726	520	463	2455	2026	245	315	444	1510	873	2541	4447	7668	11012	4383	4632
ACC_04289	conserved hypothetical protein		KOG0254	3	1	4	12	7	6	4	3	157	185	12	29	12	35	29	64
ACC_04290	insulin-degrading enzyme-like	K01408	KOG0959	821	567	531	606	583	41	69	120	1338	1184	546	1607	1976	2200	111	25
ACC_04291	protein extra bases-like		KOG2297	1164	651	509	1212	1135	347	627	605	2253	1477	1269	3265	3072	3192	1881	1216
ACC_04292	transmembrane protein 129-like		KOG3899	141	80	82	170	267	12	16	16	160	123	140	352	384	329	22	8
ACC_04293	putative E3 ubiquitin-protein ligase UBR7-like isofo	K11979	KOG2752	425	385	519	475	386	73	78	130	709	355	256	580	693	818	178	78
ACC_04294	discoidin domain-containing receptor 2-like		KOG1094	563	302	339	450	354	36	69	70	515	263	168	72	120	127	44	10
ACC_04295	protoheme IX farnesyltransferase, mitochondrial	K02257	KOG1380	591	339	352	811	759	16	42	62	912	537	382	1442	875	1155	174	46
ACC_04296	phosphoserine phosphatase	K01079	KOG1615	70	71	54	88	104	6	18	20	140	34	129	232	205	253	25	9
ACC_04297	probable ATP-dependent RNA helicase DDX52-like	K14779	KOG0344	569	283	281	488	526	74	145	192	672	376	344	433	864	1246	298	186
ACC_04298	arf-GAP with dual PH domain-containing protein 1-like		KOG0703	66	37	38	110	79	4	9	5	106	83	31	67	92	85	29	4
ACC_04299	vacuolar protein sorting-associated protein 4B isof	K12196	KOG0739	781	432	365	743	632	119	306	287	1367	674	316	1026	852	861	298	201
ACC_04300	transcription factor 25-like isoform 1		KOG2422	284	139	178	302	336	73	94	119	434	295	224	354	294	276	179	137
ACC_04301	probable methylmalonate-semialdehyde dehydrog	K00140	KOG2450	261	169	179	463	173	16	37	43	570	406	230	296	781	739	369	94
ACC_04302	pyruvate carboxylase, mitochondrial-like	K01958	KOG0369	500	303	320	607	208	41	58	60	1374	2701	258	1174	2230	805	323	136
ACC_04303	bumetanide-sensitive sodium-(potassium)-chloride cotransp		KOG2083	4	2	1	3	1	1	1	1	77	100	12	53	135	57	7	3
ACC_04304	LOW QUALITY PROTEIN		KOG0158	62	58	65	161	98	2	2	4	138	94	42	56	66	74	1	0
ACC_04305	probable cytochrome P450 6a14		KOG0158	59	49	51	240	147	21	37	37	818	378	765	2192	43	28	6	0
ACC_04306	probable cytochrome P450 6a13		KOG0158	140	245	341	927	485	56	74	69	2151	2759	2635	5966	125	342	44	22
ACC_04307	cytochrome P450 6A1		KOG0158	0	0	0	1	0	0	0	0	13	7	3	0	0	7	2	0
ACC_04308	cytochrome P450 6AS5	K07424	KOG0158	20	14	9	68	48	6	7	6	1217	726	1116	4699	11	42	9	1
ACC_04309	probable cytochrome P450 6a14-like		KOG0158	737	538	593	1035	921	51	40	76	1645	1190	602	1424	532	312	28	2
ACC_04310	PI-PLC X domain-containing protein 3-like		KOG4306	100	48	63	200	140	6	5	17	52	56	75	143	39	23	4	4
ACC_04311	kinesin-like protein KIF3B isoform 1	K10394	KOG4280	1021	701	791	1303	859	143	251	284	721	335	302	302	95	114	203	79
ACC_04312	conserved hypothetical protein			270	145	170	145	19	7	8	11	135	181	19	12	4	2	3	3
ACC_04313	conserved hypothetical protein		KOG0670	1175	827	1003	1462	1000	174	289	298	1072	605	546	298	503	829	548	342
ACC_04314	conserved hypothetical protein	K02649	KOG4637	721	448	421	949	373	56	90	110	996	821	289	152	488	374	448	268
ACC_04315	plasma kallikrein		KOG3627	4	1	0	1	1	1	0	0	3	0	0	0	42	11	0	1
ACC_04316	hypothetical protein			0	0	0	0	1	0	0	0	0	0	0	0	0	0	0	0
ACC_04317	nucleoporin p58/p45	K14307	KOG3091	478	417	399	537	350	46	58	66	580	404	316	746	720	788	100	61
ACC_04318	protein kibra-like isoform 1	K16685	KOG1028	101	84	73	87	35	12	14	13	201	215	24	55	91	103	44	13
ACC_04319	conserved hypothetical protein			266	197	165	190	136	37	48	53	318	314	353	276	52	53	16	2
ACC_04320	cyclin-L2-like		KOG0835	67	58	65	143	65	7	11	9	120	90	60	123	190	423	145	39
ACC_04321	conserved hypothetical protein			830	591	502	519	441	49	76	138	599	549	148	250	204	169	46	14
ACC_04322	G patch domain-containing protein 4-like		KOG2809	785	519	474	391	682	69	219	159	551	301	516	574	577	828	591	405
ACC_04323	conserved hypothetical protein	K12399	KOG0936	343	315	323	604	682	18	53	54	275	201	140	315	261	305	56	10
ACC_04324	quinone oxidoreductase-like protein 2-like		KOG1197	152	102	96	127	147	14	25	25	331	141	107	202	728	911	75	32
ACC_04325	UPF0160 protein MYG1, mitochondrial-like		KOG2948	155	115	108	230	250	18	21	34	118	121	106	261	795	1138	126	68
ACC_04326	conserved hypothetical protein			8	11	3	4	3	13	23	47	8	3	5	3	2	4	7	16
ACC_04327	conserved hypothetical protein	K11344	KOG3856	137	115	102	235	198	14	19	30	133	83	71	187	110	174	53	22
ACC_04328	SWI/SNF-related matrix-associated actin-depender	K11650	KOG2570	197	147	156	362	233	30	39	58	325	187	130	192	279	417	118	71
ACC_04329	LOW QUALITY PROTEIN		KOG3765	15	7	9	17	1	1	1	2	25	26	0	13	9	4	10	2

ACC_04330	probable G-protein coupled receptor 158-like			9	6	4	3	0	0	1	1	1	3	12	4	1	0	3	2
ACC_04331	peroxisomal membrane protein PEX14-like	K13343	KOG2629	250	187	119	206	188	40	37	50	446	415	103	519	1314	1049	85	46
ACC_04332	conserved hypothetical protein	K15187	KOG3149	1100	793	625	791	703	218	470	487	585	440	392	225	415	574	1583	926
ACC_04333	thioredoxin-related transmembrane protein 2 homolog		KOG0914	191	97	124	168	151	16	20	30	169	95	104	223	173	193	24	9
ACC_04334	conserved hypothetical protein		KOG4850	37	40	32	92	62	6	5	6	39	25	41	50	18	39	5	5
ACC_04335	GTP-binding protein Di-Ras2-like	K07974	KOG0395	83	91	89	106	38	2	15	20	104	53	7	9	3	4	3	2
ACC_04336	protein C20orf11-like		KOG2659	282	134	156	357	351	34	67	53	517	281	192	570	473	370	48	16
ACC_04337	ubiquitin-like protein 4A-like isoform 2		KOG0010	318	233	199	582	463	52	65	87	749	353	368	918	726	948	259	64
ACC_04338	dnaJ homolog subfamily C member 11-like	K09531	KOG0718	291	191	168	365	264	18	34	40	383	271	88	275	1298	1728	343	156
ACC_04339	conserved hypothetical protein	K11669		274	187	219	496	274	37	83	82	277	107	194	245	409	717	436	229
ACC_04340	conserved hypothetical protein		KOG1015	705	542	568	750	536	142	284	304	927	944	350	292	723	907	535	227
ACC_04341	leucine-rich repeat flightless-interacting protein 2-like		KOG2010	201	139	121	243	219	36	60	57	593	471	109	240	396	472	199	87
ACC_04342	conserved hypothetical protein		KOG0161	30	13	22	24	34	4	8	9	107	15	1	2	88900	2113	33	242
ACC_04343	vacuolar fusion protein CCZ1 homolog		KOG2622	108	68	56	133	193	8	24	26	107	72	96	192	192	212	49	32
ACC_04344	cyclin-G-associated kinase-like	K08855	KOG1989	1236	882	990	1685	1091	122	144	216	1279	1248	340	547	758	616	158	31
ACC_04345	putative tyrosine-protein phosphatase auxilin-like		KOG0431	253	174	195	329	348	19	34	39	293	171	97	113	84	111	11	5
ACC_04346	kelch-like ECH-associated protein 1-like	K10456	KOG4441	541	364	327	509	405	105	100	127	672	471	286	303	419	489	122	44
ACC_04347	glucoside xylosyltransferase 1-like	K13676	KOG3765	84	56	51	86	89	4	9	7	67	30	25	36	105	92	8	2
ACC_04348	ATP-binding cassette sub-family G member 5-like		KOG0061	19	2	1	12	9	0	1	0	71	12	22	151	67	75	29	16
ACC_04349	dual specificity protein phosphatase 7		KOG1717	156	79	63	288	144	14	16	30	184	93	28	96	86	112	79	23
ACC_04350	LOW QUALITY PROTEIN		KOG1809	978	616	433	492	254	56	76	87	1190	1217	177	237	718	621	100	53
ACC_04351	protein regulator of cytokinesis 1-like	K16732	KOG4302	199	177	130	269	206	28	70	78	91	78	76	57	248	465	425	309
ACC_04352	dosage compensation regulator isoform 2	K13184	KOG0921	706	496	438	879	664	70	85	107	762	508	264	264	515	777	207	83
ACC_04353	hypothetical protein			33	17	11	30	12	3	8	6	41	48	13	31	14	10	16	8
ACC_04354	LOW QUALITY PROTEIN		KOG2077	1015	536	475	878	486	131	226	271	1802	1026	448	537	589	437	301	146
ACC_04355	conserved hypothetical protein		KOG0161	2457	1544	1277	1784	1818	592	1276	1398	2410	2043	1461	617	1202	1623	2979	1396
ACC_04356	defensin precursor			3919	15955	4918	1437	1929	12041	15605	44789	17431	3464	124	391	19	8	87	18
ACC_04357	patched-related protein		KOG1934	300	97	100	655	194	10	10	27	207	232	27	83	77	235	41	12
ACC_04358	conserved hypothetical protein	K15304	KOG0866	1270	826	723	1314	1059	393	725	917	1135	903	743	747	1104	1482	3120	2863
ACC_04359	torso-like protein-like	K12377		66	22	6	48	10	9	9	8	166	120	7	20	114	68	36	2
ACC_04360	DNA-binding protein Ets97D homolog		KOG3806	223	167	159	299	214	32	60	71	351	189	169	206	324	403	132	67
ACC_04361	conserved hypothetical protein	K10314	KOG2120	200	144	127	324	371	14	30	20	594	295	143	223	212	152	16	5
ACC_04362	constitutive coactivator of PPAR-gamma-like protein 1 homolog isoforn			992	679	479	1088	447	139	252	284	1211	1111	345	326	611	847	1815	1071
ACC_04363	prefoldin subunit 1-like isoform 1	K09548	KOG3501	169	152	176	288	197	20	38	40	102	62	156	338	149	246	119	88
ACC_04364	N-acetyltransferase 10-like isoform 1	K14521	KOG2036	705	569	479	903	850	94	218	253	890	971	597	975	1972	2223	526	260
ACC_04365	LOW QUALITY PROTEIN	K06627	KOG0654	80	53	49	129	117	15	22	20	49	58	74	66	179	315	314	195
ACC_04366	clathrin light chain		KOG4031	1752	1144	1217	1936	1132	409	845	1162	1005	790	726	940	1327	1443	3691	3969
ACC_04367	eukaryotic translation initiation factor 3 subunit C	K03252	KOG1076	1153	569	460	901	839	271	508	579	1200	1124	607	865	3043	4539	5493	4487
ACC_04368	conserved hypothetical protein	K11791		260	204	224	274	195	34	32	37	344	220	133	91	268	264	71	24
ACC_04369	rho GTPase-activating protein 26-like		KOG1451	898	546	494	832	811	75	117	119	811	618	330	393	432	389	82	32
ACC_04370	cytoplasmic FMR1-interacting protein isoform 1	K05749	KOG3534	197	95	78	97	115	14	24	36	331	380	76	154	334	247	35	13
ACC_04371	conserved hypothetical protein		KOG4609	46	33	30	62	72	6	9	7	48	28	36	43	77	99	13	8
ACC_04372	serine/threonine-protein phosphatase PGAM5, mit	K15637	KOG4609	176	150	141	203	160	15	22	41	370	228	73	271	420	448	54	23
ACC_04373	cysteinyl-tRNA synthetase, cytoplasmic-like	K01883	KOG2007	445	313	253	453	520	55	147	164	317	306	258	425	785	752	346	278
ACC_04374	60S ribosomal protein L19-like	K02885	KOG1696	1931	1680	706	1325	888	601	1109	1450	1610	1115	1670	2215	7361	8876	5821	4470
ACC_04375	conserved hypothetical protein			42	21	28	28	5	0	1	2	46	88	3	4	13	4	55	31
ACC_04376	conserved hypothetical protein			30	16	14	18	5	3	2	7	17	28	9	5	4	2	9	5
ACC_04377	UPF0687 protein C20orf27 homolog			83	46	49	63	56	2	11	8	85	79	30	76	225	207	53	10
ACC_04378	major facilitator superfamily domain-containing protein 6-lik		KOG3762	67	69	50	131	171	5	12	13	299	205	112	267	453	225	16	1
ACC_04379	f-box/WD repeat-containing protein 4-like	K10262	KOG0272	186	111	123	322	340	21	13	29	211	152	107	318	203	163	15	6
ACC_04380	conserved hypothetical protein	K16344		83	78	55	158	170	7	4	6	50	32	79	202	138	171	24	18
ACC_04381	x-ray repair cross-complementing protein 5-like			181	60	45	105	58	5	9	13	352	35	108	1157	169	130	53	62
ACC_04382	TWiK family of potassium channels protein 18-like			34	15	11	14	8	1	0	0	23	29	1	9	17	9	3	2
ACC_04383	WD repeat-containing protein 91-like		KOG1333	543	312	339	468	396	75	118	127	758	570	274	668	415	297	91	26
ACC_04384	huntingtin-interacting protein K-like		KOG3450	400	162	147	309	236	55	66	107	298	212	113	249	877	1716	1380	1358
ACC_04385	conserved hypothetical protein			110	55	41	64	16	9	15	13	524	593	39	146	227	192	205	93
ACC_04386	conserved hypothetical protein			61	42	42	37	13	0	3	1	37	35	7	18	13	8	8	0

ACC_04387	Hermansky-Pudlak syndrome 1 protein homolog			456	290	272	545	562	36	50	65	467	287	225	402	540	502	51	10
ACC_04388	conserved hypothetical protein		KOG1195	90	54	37	69	94	1	4	4	56	81	44	184	170	129	13	2
ACC_04389	conserved hypothetical protein	K09114	KOG2483	211	178	238	441	82	32	54	96	232	174	109	48	20	13	45	30
ACC_04390	adenylyltransferase and sulfurtransferase MOCS3-l	K11996	KOG2017	186	144	161	158	194	48	144	133	293	176	209	344	284	275	76	44
ACC_04391	proteasome subunit alpha type-6-like	K02730	KOG0182	226	117	110	706	653	50	54	87	942	362	630	1284	1515	1757	334	183
ACC_04392	rRNA-processing protein UTP23 homolog	K14773	KOG3164	889	417	423	579	700	160	410	336	395	209	454	572	718	1298	1206	807
ACC_04393	rab3 GTPase-activating protein non-catalytic subunit-like iso		KOG2727	654	389	442	726	732	79	89	133	853	868	402	428	547	507	108	44
ACC_04394	CREB 1 protein	K09050	KOG3584	166	103	109	218	131	52	81	116	420	374	107	372	358	382	306	169
ACC_04395	hyccin-like		KOG4688	146	48	59	140	146	27	60	58	184	243	205	296	100	79	56	29
ACC_04396	Golgi reassembly-stacking protein 1-like		KOG3834	475	477	432	496	355	82	110	124	766	422	196	772	943	1013	210	63
ACC_04397	GPI-anchored wall transfer protein 1-like	K05283	KOG0411	179	79	72	107	150	15	22	37	103	81	87	158	343	440	67	32
ACC_04398	tetratricopeptide repeat protein 4		KOG0551	406	337	344	417	417	60	126	152	260	135	322	222	447	618	364	326
ACC_04399	growth hormone secretagogue receptor type 1		KOG4219	2	1	2	6	2	0	0	1	12	19	1	1	1	4	0	1
ACC_04400	conserved hypothetical protein			255	88	70	120	153	49	85	125	139	47	162	172	395	538	359	464
ACC_04401	acetylcholinesterase-like		KOG1516	21	10	9	40	20	6	14	18	12	6	12	1	0	6	5	2
ACC_04402	vacuolar protein sorting-associated protein 26-like		KOG3063	177	63	51	127	127	10	16	13	319	339	53	390	365	405	36	9
ACC_04403	motile sperm domain-containing protein 2-like		KOG1470	388	264	174	263	320	21	57	44	753	402	266	526	783	794	80	24
ACC_04404	LOW QUALITY PROTEIN			9	5	1	6	2	0	2	1	9	8	3	1	2	3	5	0
ACC_04405	conserved hypothetical protein		KOG0689	262	297	185	293	92	106	79	93	880	437	90	177	109	138	84	20
ACC_04406	conserved hypothetical protein			1	2	0	10	4	0	1	0	109	0	2	10	43598	30236	557	29
ACC_04407	hypothetical protein			4	4	1	4	0	0	0	1	1	1	0	0	0	0	2	0
ACC_04408	hypothetical protein			0	0	0	1	0	1	0	0	0	0	0	0	0	0	0	0
ACC_04409	myoneurin-like		KOG4441	75	61	40	53	23	1	6	5	133	82	12	36	42	18	2	1
ACC_04410	ring canal kelch homolog	K10443	KOG4441	116	83	92	61	43	6	11	13	292	180	84	292	108	95	20	5
ACC_04411	conserved hypothetical protein			72	30	64	136	76	17	23	29	103	46	49	126	43	114	134	98
ACC_04412	conserved hypothetical protein		KOG1955	614	442	452	613	305	41	58	61	667	597	171	301	251	225	106	82
ACC_04413	acetyl-coenzyme A transporter 1-like	K03372	KOG3574	207	90	95	244	193	36	30	46	326	309	124	418	453	667	175	26
ACC_04414	secretion regulating guanine nucleotide exchange factor		KOG1427	154	87	96	231	188	22	49	50	82	80	44	48	240	405	122	77
ACC_04415	Transcription initiation factor TFIID subunit 13	K03127	KOG3901	107	94	104	90	80	11	24	23	228	133	50	208	178	264	34	18
ACC_04416	conserved hypothetical protein			253	121	126	210	262	49	146	128	184	84	185	159	180	320	352	315
ACC_04417	conserved hypothetical protein		KOG0676	521	257	240	286	264	24	59	62	539	337	41	92	203	188	50	12
ACC_04418	conserved hypothetical protein		KOG1418	0	0	0	0	0	0	0	0	1	7	0	0	5	7	3	2
ACC_04419	39S ribosomal protein L23, mitochondrial-like	K02892	KOG4089	113	96	98	141	116	7	36	22	89	58	101	184	331	460	58	40
ACC_04420	atrial natriuretic peptide receptor 1-like	K12323	KOG1023	9	8	14	37	17	0	3	6	15	9	7	4	1	4	1	0
ACC_04421	sugar transporter SWEET1-like	K15382	KOG1623	106	50	47	129	167	5	3	14	110	68	73	273	155	111	10	1
ACC_04422	TAR DNA-binding protein 43-like		KOG4205	690	394	322	649	309	83	220	187	721	518	230	128	152	263	617	234
ACC_04423	conserved hypothetical protein		KOG4305	1450	1223	1371	1165	510	184	216	282	1717	1213	241	333	731	616	586	225
ACC_04424	polyadenylate-binding protein-interacting protein 1-like		KOG0401	645	444	433	583	415	86	163	162	746	423	290	483	776	803	248	130
ACC_04425	conserved hypothetical protein		KOG4308	41	20	17	15	36	6	5	3	101	24	34	23	13	8	3	0
ACC_04426	conserved hypothetical protein		KOG0517	874	687	698	954	580	91	173	154	1788	783	287	394	667	828	257	74
ACC_04427	HIV-1 Vpr-binding protein		KOG1832	419	257	348	212	192	31	63	61	213	136	86	51	110	95	53	23
ACC_04428	fatty acid synthase-like isoform 1	K00665	KOG1202	16	9	18	24	13	0	3	6	64	31	3	3	7404	13592	369	1028
ACC_04429	metaxin-2-like isoform 2		KOG3027	172	71	61	85	128	19	31	47	309	211	114	355	303	299	72	41
ACC_04430	t-complex protein 1 subunit delta-like isoform 1	K09496	KOG0358	713	356	396	1282	1010	49	81	106	1092	856	662	1672	3515	5554	461	171
ACC_04431	conserved hypothetical protein		KOG0527	0	0	1	1	0	1	0	0	1	1	0	0	2	3	0	0
ACC_04432	T-box transcription factor TBX5-A-like	K10172	KOG3585	7	8	17	37	29	2	4	0	16	7	12	3	34	24	18	14
ACC_04433	EF-hand domain-containing protein CG10641-like		KOG0041	962	453	492	624	336	52	70	107	1246	456	515	3344	684	238	49	39
ACC_04434	acylamino-acid-releasing enzyme-like	K01303	KOG2100	325	183	161	251	329	49	126	119	522	446	234	406	1924	1273	528	290
ACC_04435	conserved hypothetical protein			275	199	203	142	179	19	21	30	215	134	266	74	91	83	4	2
ACC_04436	UPF0414 transmembrane protein C20orf30 homolog isoforn		KOG4753	239	119	115	276	251	14	11	8	112	68	67	279	130	121	8	2
ACC_04437	SWI/SNF-related matrix-associated actin-depender	K14439	KOG0389	282	202	179	328	327	40	70	89	270	274	293	421	424	406	134	63
ACC_04438	proton-coupled amino acid transporter 4-like		KOG1304	220	165	151	52	67	155	502	282	272	88	45	70	158	103	40	8
ACC_04439	15-hydroxyprostaglandin dehydrogenase		KOG4169	100	91	67	431	252	416	474	758	6102	981	0	289	4	3	0	0
ACC_04440	band 4.1-like protein 4A-like		KOG3530	73	69	65	224	74	29	37	45	97	388	34	75	34	76	86	48
ACC_04441	conserved hypothetical protein			2	1	0	2	1	0	2	2	22	2	8	3	2	5	12	9
ACC_04442	conserved hypothetical protein			326	175	203	315	162	19	30	35	100	19	45	19	3	0	6	0
ACC_04443	intron-binding protein aquarius	K12874	KOG1806	365	282	286	496	427	40	74	63	543	558	200	364	680	886	110	40

ACC_04444	histidine decarboxylase	K01590	KOG0628	96	48	70	295	103	123	123	169	770	363	6	11	11	20	16	8
ACC_04445	histone H4	K11254	KOG3467	85	116	96	452	212	34	23	70	148	122	100	95	268	168	94	89
ACC_04446	unconventionnal myosin-X-like		KOG0161	408	319	269	372	112	19	31	49	519	710	26	115	93	75	37	22
ACC_04447	Mps one binder kinase activator-like 3 isoform 1		KOG1903	102	81	58	118	93	10	12	15	105	116	44	250	256	291	28	8
ACC_04448	protein BCCIP homolog	K15262	KOG3034	197	173	91	285	292	28	49	61	248	181	181	271	1135	1698	323	233
ACC_04449	neutral alpha-glucosidase AB-like isoform 2	K05546	KOG1066	802	581	406	943	769	65	60	87	1356	2139	265	1340	6937	4985	287	50
ACC_04450	transmembrane protein 145-like		KOG4290	142	83	77	127	175	8	10	9	185	118	54	106	134	128	16	0
ACC_04451	putative glycerol kinase 3-like	K00864	KOG2517	181	127	57	125	97	21	31	45	380	602	27	108	1166	765	147	99
ACC_04452	conserved hypothetical protein		KOG3598	685	492	527	435	161	49	53	82	560	401	134	320	660	1158	1086	553
ACC_04453	microtubule-associated serine/threonine-protein k	K08789	KOG0606	841	696	601	751	376	90	165	159	1512	1672	226	388	427	371	236	76
ACC_04454	prolyl 3-hydroxylase 2-like		KOG4459	696	379	344	1161	869	83	217	272	586	365	221	600	418	307	389	151
ACC_04455	conserved hypothetical protein			1	1	0	0	0	0	0	0	0	6	0	0	2	0	0	0
ACC_04456	LOW QUALITY PROTEIN	K00626	KOG1390	250	128	132	375	445	7	7	20	229	233	146	430	467	436	30	6
ACC_04457	serine/threonine-protein kinase MARK2 isoform 1		KOG0586	497	410	388	358	135	79	116	126	714	574	125	179	301	307	370	134
ACC_04458	bis(5'-nucleosyl)-tetraphosphatase	K01518	KOG2839	165	142	127	287	161	21	27	35	236	127	194	318	464	324	131	36
ACC_04459	pyroglutamyl-peptidase 1-like	K01304	KOG4755	26	28	33	33	42	2	3	4	28	46	31	72	206	178	12	3
ACC_04460	leucine-rich repeat-containing protein 6-like		KOG0531	54	63	57	138	86	19	21	17	62	33	90	111	8	15	52	34
ACC_04461	cleft lip and palate transmembrane protein 1 homolog		KOG2489	822	468	524	690	914	18	59	60	1674	1825	450	1725	2710	2486	179	93
ACC_04462	wings apart-like isoform 1		KOG2152	453	356	307	379	293	56	83	118	766	678	241	377	639	696	98	31
ACC_04463	zinc finger protein 224-like		KOG2462	14	9	10	46	17	2	2	1	2	1	0	0	1	3	10	5
ACC_04464	peptidyl-prolyl cis-trans isomerase E-like isoform 2		KOG2898	340	287	260	470	444	64	48	86	437	404	300	840	679	454	30	20
ACC_04465	peptidyl-prolyl cis-trans isomerase E-like isoform 1		KOG0111	288	194	189	535	472	63	66	123	452	148	275	381	385	727	145	46
ACC_04466	FIT family protein CG10671-like		KOG3750	129	74	117	214	179	15	18	17	290	213	121	357	265	196	25	4
ACC_04467	Sjoegren syndrome nuclear autoantigen 1 homolog	K16780		35	23	31	58	29	3	5	8	15	13	20	33	34	30	18	13
ACC_04468	prolyl 4-hydroxylase subunit alpha-2-like	K00472	KOG1591	117	44	79	183	63	3	3	6	136	99	36	70	95	90	80	19
ACC_04469	conserved hypothetical protein		KOG4597	21	75	128	559	661	4	6	7	143	22	66	86	3	3	2	0
ACC_04470	zinc finger protein Xfin-like		KOG3576	1926	899	920	1845	1470	517	1249	1270	1136	686	759	277	351	550	1604	1175
ACC_04471	heparan-alpha-glucosaminide N-acetyltransferase-	K10532	KOG4683	844	634	505	1073	1055	148	220	322	1476	1306	613	1596	974	706	76	24
ACC_04472	probable histone acetyltransferase MYST1	K11308	KOG2747	199	144	149	414	327	12	19	28	355	229	145	256	215	348	56	14
ACC_04473	la-related protein 7-like	K15191	KOG1855	1145	741	710	703	1142	141	341	432	758	459	871	585	950	1608	1336	825
ACC_04474	hypothetical protein			0	0	0	2	1	0	0	0	1	2	0	0	0	0	0	0
ACC_04475	diphosphoinositol polyphosphate phosphohydrolas	K07766	KOG2839	160	131	114	85	60	15	27	36	216	154	96	192	207	217	101	23
ACC_04476	e3 UFM1-protein ligase 1 homolog		KOG2235	624	425	414	607	618	69	165	160	842	846	441	819	1310	1400	208	105
ACC_04477	conserved hypothetical protein			60	50	66	39	19	0	0	1	3	4	28	41	10	21	9	9
ACC_04478	ribulose-phosphate 3-epimerase-like	K01783	KOG3111	142	101	76	212	176	21	35	42	214	169	176	352	1198	1020	131	55
ACC_04479	mitochondrial import inner membrane translocase subunit T		KOG3479	30	25	18	30	35	1	1	8	17	9	44	83	99	120	16	9
ACC_04480	conserved hypothetical protein		KOG2120	493	345	315	445	293	77	154	181	817	858	241	432	608	854	390	248
ACC_04481	G-protein coupled receptor moody-like		KOG4219	294	28	22	83	48	5	7	15	152	116	29	148	15	21	7	3
ACC_04482	conserved hypothetical protein			561	357	556	1170	286	234	185	420	1822	1005	848	687	51	39	161	154
ACC_04483	short coiled-coil protein B-like		KOG3650	1109	638	774	1076	576	36	49	41	618	294	214	991	314	199	44	7
ACC_04484	putative odorant receptor 9a			22	16	10	58	25	3	5	5	11	9	70	52	2	10	10	1
ACC_04485	conserved hypothetical protein			11	6	8	7	1	0	3	2	10	5	4	0	1	0	1	0
ACC_04486	transmembrane protein 135-like		KOG1398	119	85	73	137	198	14	14	19	626	389	215	785	257	204	16	10
ACC_04487	haloacid dehalogenase-like hydrolase domain-containing prc		KOG3040	217	110	96	250	237	10	19	33	218	60	176	291	168	212	35	13
ACC_04488	splicing factor 1-like	K13095	KOG0119	737	649	853	548	223	83	51	90	1372	649	292	311	763	1008	386	100
ACC_04489	zinc finger protein 91-like		KOG2462	157	91	85	202	174	16	17	19	214	200	65	118	132	137	58	23
ACC_04490	DNA polymerase epsilon subunit 4-like	K03506	KOG1657	480	318	236	336	342	59	147	188	163	78	134	192	236	229	120	138
ACC_04491	CD81 antigen-like		KOG3882	15	5	7	11	3	0	1	0	7	7	0	6	179	350	90	19
ACC_04492	alkylated DNA repair protein alkB homolog 8-like	K10770	KOG1331	194	144	153	238	296	20	37	52	226	156	154	169	264	297	29	13
ACC_04493	exosome complex exonuclease RRP45-like	K03678	KOG1614	232	165	140	245	271	50	66	89	240	151	181	371	355	449	170	115
ACC_04494	cytoskeleton-associated protein 5	K16803	KOG1820	2060	1209	1121	1650	1261	165	299	368	1855	2216	455	974	1626	1337	358	127
ACC_04495	protein SPT2 homolog	K15193	KOG1029	1993	1180	1030	1479	1648	447	1118	1143	1039	672	902	545	919	1255	2904	1790
ACC_04496	conserved hypothetical protein	K06689	KOG0417	237	134	127	288	274	13	29	28	287	260	185	402	315	347	22	8
ACC_04497	probable ribonuclease ZC3H12C-like isoform 1		KOG3777	154	102	116	255	219	7	7	25	194	133	50	152	257	357	86	19
ACC_04498	probable malonyl-CoA-acyl carrier protein transacy	K00645	KOG2926	323	229	253	572	674	24	38	55	322	182	306	185	377	557	67	22
ACC_04499	probable small nuclear ribonucleoprotein Sm D1-lik	K11087	KOG3428	213	147	176	242	174	28	63	58	164	130	134	130	329	834	766	601
ACC_04500	conserved hypothetical protein			59	20	35	66	17	5	4	9	318	164	26	47	13	18	25	41

ACC_04501	neurochondrin homolog		KOG2611	1029	672	758	835	1006	66	91	124	2735	1426	311	388	709	661	84	38
ACC_04502	neurochondrin homolog	K01097	KOG3085	97	57	53	92	110	4	6	6	129	104	54	111	146	100	17	4
ACC_04503	ubiquitin carboxyl-terminal hydrolase 5-like	K11836	KOG0944	688	525	483	869	663	53	105	139	942	722	609	1007	1677	1874	170	109
ACC_04504	ankyrin repeat and zinc finger domain-containing protein 1-l		KOG2505	417	308	231	577	605	79	168	257	458	340	272	344	359	363	299	277
ACC_04505	ester hydrolase C11orf54 homolog		KOG4048	173	144	102	189	195	32	46	39	1021	1829	218	428	9765	6689	354	77
ACC_04506	transmembrane protein 20-like		KOG4510	71	49	57	42	52	4	10	8	77	43	19	17	169	221	20	7
ACC_04507	conserved hypothetical protein			56	46	71	106	158	5	11	15	22	29	49	34	86	111	96	39
ACC_04508	ARF GTPase-activating protein GIT2-like isoform 1		KOG0818	264	173	174	434	359	30	26	42	282	214	114	200	191	219	34	9
ACC_04509	nucleolysin TIAR	K13201	KOG0148	111	107	103	122	39	14	4	9	335	406	41	277	483	682	192	33
ACC_04510	pre-mRNA-processing factor 39-like	K13217	KOG1258	2898	1979	1875	2367	1682	535	906	952	3166	1952	978	936	2417	3335	3565	2562
ACC_04511	conserved hypothetical protein	K09027	KOG4005	1210	1374	1106	2282	1443	283	420	763	2408	2390	3011	6565	2476	1982	780	323
ACC_04512	39S ribosomal protein L11, mitochondrial-like	K02867	KOG3257	310	195	189	329	320	49	115	120	311	135	249	397	631	867	230	185
ACC_04513	tyrosine-protein kinase hopscotch isoform 1	K04447	KOG0197	942	602	557	1096	938	109	186	220	802	709	314	511	756	874	336	191
ACC_04514	endoribonuclease Dicer-1	K11592	KOG0701	623	372	346	324	333	44	87	84	569	483	212	219	377	599	166	42
ACC_04515	pleiotropic regulator 1	K12862	KOG0285	322	187	176	416	312	42	61	62	415	188	231	342	247	433	146	69
ACC_04516	threonine aspartase 1-like	K08657	KOG1592	156	110	113	197	133	20	34	36	225	88	129	179	185	282	81	46
ACC_04517	RNA-binding motif protein, X-linked 2-like	K13107	KOG0126	67	73	77	124	105	6	13	21	40	36	43	131	73	85	15	9
ACC_04518	AMP deaminase 2 isoform 1			27	23	18	29	21	5	8	4	87	86	17	35	51	27	6	4
ACC_04519	conserved hypothetical protein		KOG0391	1152	790	653	1087	748	239	335	346	1744	1305	546	766	1324	1407	574	312
ACC_04520	probable medium-chain specific acyl-CoA dehydrog	K00249	KOG0140	686	340	192	530	450	201	297	415	2215	1125	521	1041	6483	6962	2268	1311
ACC_04521	conserved hypothetical protein		KOG1596	1444	825	811	1440	1154	207	414	455	1251	947	689	665	1416	2504	2203	1525
ACC_04522	conserved hypothetical protein		KOG3538	1	2	2	2	4	0	0	1	6	7	1	0	12	16	11	49
ACC_04523	conserved hypothetical protein			28	23	14	10	17	2	8	12	43	35	6	8	49	23	9	4
ACC_04524	eukaryotic translation initiation factor 3 subunit J-li	K03245	KOG4813	1057	670	472	994	529	361	599	747	1298	629	723	977	1762	2406	3036	3354
ACC_04525	integrator complex subunit 9-like isoform 1		KOG1138	157	123	83	151	154	21	20	39	180	112	127	198	417	454	57	32
ACC_04526	kynurenine/alpha-aminoadipate aminotransferase, mitocho		KOG0634	72	30	47	48	85	11	20	18	288	304	4	3	1052	450	15	9
ACC_04527	CCA tRNA nucleotidyltransferase 1, mitochondrial-l	K00974	KOG2159	174	103	103	162	242	4	10	15	116	155	104	299	702	729	42	29
ACC_04528	V-type proton ATPase subunit E-like isoform 1	K02150	KOG1664	1134	636	656	1442	1362	84	212	216	1693	473	1211	2629	1771	2236	422	291
ACC_04529	CAAX prenyl protease 1 homolog	K06013	KOG2719	284	183	177	208	416	7	8	8	388	327	220	518	934	1036	11	5
ACC_04530	conserved hypothetical protein			9	5	3	16	0	4	6	2	629	304	1	14	104	99	81	18
ACC_04531	conserved hypothetical protein			11	7	8	45	13	0	3	3	40	31	22	22	5	23	3	2
ACC_04532	conserved hypothetical protein		KOG1973	851	542	412	928	574	219	440	496	670	704	388	171	221	299	1845	864
ACC_04533	UPF0545 protein C22orf39 homolog			158	124	101	210	171	42	67	81	159	93	209	303	180	230	204	128
ACC_04534	heat shock protein cognate 4	K03283	KOG0101	21930	11069	18291	69307	42013	3237	3025	5449	36159	39524	17646	76645	55398	53842	39910	4793
ACC_04535	conserved hypothetical protein		KOG3053	333	127	113	342	357	22	18	24	1337	781	153	771	377	312	16	4
ACC_04536	hypothetical protein			7	4	6	9	7	2	2	5	15	29	8	4	4	1	1	3
ACC_04537	glutamate--cysteine ligase regulatory subunit-like	K11205	KOG3023	166	118	102	274	299	12	25	35	328	160	406	698	643	1043	72	27
ACC_04538	39S ribosomal protein L48, mitochondrial		KOG4060	96	47	31	131	178	1	5	7	31	46	99	316	175	156	70	32
ACC_04539	synaptotagmin 1	K15290	KOG1028	2566	1649	1658	3520	1496	261	322	488	1376	818	300	138	18	30	61	22
ACC_04540	probable ATP-dependent RNA helicase DDX27-like	K13181	KOG0338	678	306	243	376	468	93	228	230	432	347	318	213	1080	2020	1653	1090
ACC_04541	40S ribosomal protein S20	K02969	KOG0900	382	324	185	524	285	96	180	188	193	151	629	385	1045	1589	576	251
ACC_04542	conserved hypothetical protein		KOG4187	2088	1828	2265	4826	2225	159	228	352	683	278	297	516	8	40	64	13
ACC_04543	putative homeodomain transcription factor-like			423	293	260	324	383	32	54	56	609	402	222	220	366	411	46	14
ACC_04544	trafficking protein particle complex subunit 2-like protein-lik		KOG3444	63	44	55	100	128	1	2	4	35	31	45	133	85	94	6	1
ACC_04545	protein MAK16 homolog A-like	K14831	KOG3064	573	405	363	600	469	114	289	309	373	222	261	158	719	1696	1410	1197
ACC_04546	conserved hypothetical protein			961	384	335	439	789	57	210	274	284	182	286	489	678	1065	580	481
ACC_04547	conserved hypothetical protein		KOG0161	27	29	28	70	48	1	5	5	3	50	4	8	1	1	1	1
ACC_04548	transcriptional enhancer factor TEF-1	K09448	KOG3841	88	72	65	54	13	12	19	16	164	169	36	58	42	76	156	49
ACC_04549	coiled-coil domain-containing protein 22 homolog		KOG1937	597	500	507	543	549	72	197	219	355	310	345	251	539	532	290	167
ACC_04550	WD repeat-containing protein 61-like	K12602	KOG0272	119	117	112	206	165	9	15	27	126	103	79	241	358	355	51	14
ACC_04551	conserved hypothetical protein		KOG0161	58	41	46	32	32	9	10	14	46	59	32	25	217	429	628	434
ACC_04552	importin subunit alpha-4-like		KOG0166	547	267	281	592	383	54	68	77	861	910	248	981	1487	1924	372	143
ACC_04553	conserved hypothetical protein			266	167	170	268	291	35	72	95	197	116	209	298	138	192	69	41
ACC_04554	LOW QUALITY PROTEIN	K01868	KOG1637	918	584	526	1168	1131	78	128	165	1051	1042	405	1465	2058	2045	386	213
ACC_04555	ribosomal protein S6 kinase alpha-5	K04445	KOG0603	686	315	319	1022	424	157	251	249	1669	1389	764	296	197	307	625	547
ACC_04556	abhydrolase domain-containing protein 11-like	K13703	KOG2382	719	383	383	690	853	92	123	155	648	355	358	845	893	891	209	81
ACC_04557	conserved hypothetical protein		KOG3869	1075	616	644	644	992	218	530	461	563	251	574	579	577	977	1074	606

ACC_04558	conserved hypothetical protein	K15044	KOG0702	527	377	392	796	481	60	95	111	1311	538	431	836	481	510	95	38
ACC_04559	conserved hypothetical protein		KOG0933	559	472	432	487	484	53	130	164	375	217	244	425	396	362	114	38
ACC_04560	conserved hypothetical protein		KOG4292	2	2	0	2	0	0	1	1	13	18	2	6	4	7	12	22
ACC_04561	conserved hypothetical protein		KOG3598	598	582	575	389	98	46	68	78	884	911	81	145	372	329	442	231
ACC_04562	Meckel syndrome type 1 protein-like		KOG4446	104	38	73	87	83	4	7	7	48	20	208	320	9	12	3	2
ACC_04563	mitotic spindle assembly checkpoint protein MAD1		KOG4593	366	283	300	378	263	68	100	134	407	289	171	211	387	441	386	218
ACC_04564	dedicator of cytokinesis protein 7-like		KOG1997	171	83	61	91	56	12	19	15	303	175	35	134	183	149	35	16
ACC_04565	axotactin		KOG3516	1055	402	424	868	428	25	53	55	381	209	132	752	11	25	52	13
ACC_04566	nudC domain-containing protein 1-like		KOG4379	321	225	231	386	444	8	51	44	288	219	208	373	412	415	71	22
ACC_04567	conserved hypothetical protein		KOG2392	167	139	134	421	287	45	52	70	1623	1101	138	242	108	97	18	8
ACC_04568	BTB/POZ domain-containing protein 7-like			24	11	12	13	7	0	0	1	19	20	0	6	41	13	1	0
ACC_04569	transforming growth factor beta regulator 1-like			122	106	90	162	109	10	16	25	143	94	74	169	247	273	48	24
ACC_04570	conserved hypothetical protein			133	106	76	204	99	7	16	32	90	76	39	63	105	118	34	10
ACC_04571	ELMO domain-containing protein 2-like		KOG2998	66	40	41	90	95	2	12	5	68	81	48	163	127	115	10	2
ACC_04572	protein henna-like isoform 2	K00500	KOG3820	105	179	205	738	385	52	51	59	1208	762	347	783	165	548	261	101
ACC_04573	60S ribosomal protein L24	K02896	KOG1722	4403	1883	1144	2732	1355	758	1513	1375	1265	1396	1737	2643	9589	11903	10545	11712
ACC_04574	nuclear factor NF-kappa-B p105 subunit		KOG4177	966	781	634	1059	1054	253	441	472	2400	1700	893	1634	1902	1475	369	169
ACC_04575	vesicle transport protein SFT2B-like		KOG2887	40	46	43	64	77	3	8	12	65	52	47	133	153	141	8	3
ACC_04576	importin subunit alpha-2-like	K15043	KOG0166	308	188	199	403	325	21	37	45	536	452	236	610	1055	1454	212	73
ACC_04577	ubiquitin carboxyl-terminal hydrolase 43-like	K11835	KOG1870	365	231	167	207	131	42	59	55	788	459	153	145	699	1282	260	57
ACC_04578	cytochrome b-c1 complex subunit 6, mitochondrial	K00416	KOG4763	192	84	118	342	371	15	12	22	204	73	242	294	361	381	31	20
ACC_04579	conserved hypothetical protein			179	84	108	241	168	26	46	41	106	151	66	104	57	49	32	6
ACC_04580	ribonuclease P protein subunit p29-like	K03538	KOG4046	68	78	71	138	122	1	8	10	35	39	67	126	103	133	37	17
ACC_04581	probable phosphorylase b kinase regulatory subuni	K07190	KOG3635	550	365	396	778	605	33	46	59	844	599	258	255	450	388	49	14
ACC_04582	proline dehydrogenase 1, mitochondrial-like isofor	K00318	KOG0186	872	335	314	804	407	47	53	85	528	206	231	383	134	239	85	33
ACC_04583	conserved hypothetical protein			124	68	79	122	107	8	17	26	90	23	32	51	15	37	5	7
ACC_04584	Putative dolichyl pyrophosphate Man9GlcNAc2 alp	K03848	KOG2575	164	102	68	125	158	6	11	10	153	154	68	258	366	362	15	7
ACC_04585	mevalonate kinase-like	K00869	KOG1511	341	257	230	465	422	8	31	34	411	360	172	587	605	535	27	16
ACC_04586	dnaJ homolog subfamily C member 16-like	K09536	KOG0713	615	259	199	341	278	35	58	36	820	677	119	424	393	420	53	9
ACC_04587	hypothetical protein			10	3	1	7	4	0	1	0	2	11	1	1	0	0	2	1
ACC_04588	conserved hypothetical protein			62	75	73	76	108	3	7	7	49	18	72	116	167	249	13	16
ACC_04589	laminin subunit beta-1	K05636	KOG0994	513	278	230	407	234	52	74	95	1107	3981	118	468	2747	2187	752	202
ACC_04590	CD109 antigen		KOG1366	182	97	73	45	34	8	16	18	402	440	49	349	364	315	21	6
ACC_04591	CDKAL1-like protein-like	K15865	KOG4355	70	67	72	115	100	5	8	20	72	70	44	78	123	210	42	22
ACC_04592	leucine-rich repeat-containing protein 28-like		KOG0532	308	201	218	481	546	10	16	27	106	75	94	118	83	45	11	2
ACC_04593	conserved hypothetical protein			192	157	147	126	184	24	58	73	107	63	131	171	151	234	155	87
ACC_04594	gametogenetin-binding protein 2-like			387	259	223	404	286	71	134	144	464	256	167	226	404	553	465	265
ACC_04595	telomerase-binding protein EST1A-like	K11124	KOG2162	656	400	409	472	217	121	204	227	579	606	174	101	122	159	668	417
ACC_04596	telomerase-binding protein EST1A-like	K11124	KOG2162	131	100	102	116	36	10	15	23	154	214	27	33	21	45	105	104
ACC_04597	IQ domain-containing protein K-like			15	12	6	27	21	3	2	0	4	6	15	24	33	69	14	4
ACC_04598	REM2- and Rab-like small GTPase 1-like		KOG0084	76	71	62	82	93	10	16	14	112	71	90	144	173	229	34	15
ACC_04599	zinc transporter ZIP11-like	K14717	KOG2474	120	63	42	168	53	25	19	19	168	86	18	93	193	159	103	19
ACC_04600	glutamate dehydrogenase, mitochondrial-like	K00261	KOG2250	1022	344	321	1022	713	155	197	304	2541	1307	262	884	2153	1796	364	250
ACC_04601	band 7 protein AAEL010189-like		KOG2621	221	100	73	184	111	17	26	21	136	162	264	480	23	8	13	2
ACC_04602	conserved hypothetical protein			41	30	26	53	55	3	13	10	20	20	30	55	89	71	16	4
ACC_04603	conserved hypothetical protein			978	376	390	815	557	54	100	102	469	228	158	53	101	129	245	198
ACC_04604	HIV-1 Vpr-binding protein		KOG1832	114	83	85	90	39	10	20	24	41	33	10	2	14	26	73	37
ACC_04605	zinc finger CCHC domain-containing protein 24-like			56	33	22	68	48	18	7	11	144	88	39	9	49	27	9	10
ACC_04606	glutaredoxin-C4-like isoform 2	K03676	KOG1752	276	444	308	326	324	81	119	133	443	176	300	443	790	926	224	202
ACC_04607	group XIIA secretory phospholipase A2-like	K01047		89	84	47	178	148	14	14	15	139	95	100	209	214	230	34	11
ACC_04608	conserved hypothetical protein		KOG0943	1009	755	1067	741	589	153	265	251	824	452	230	176	311	389	126	53
ACC_04609	protein sidekick-like	K16353	KOG3513	307	195	148	171	131	29	30	39	604	407	182	244	425	417	86	26
ACC_04610	Nucleosome-remodeling factor subunit NURF301	K11728	KOG1473	3152	2196	1878	2620	2219	878	1858	2019	3142	2633	1471	643	911	1179	3610	1711
ACC_04611	4-aminobutyrate aminotransferase, mitochondrial-like isofo		KOG1405	58	33	36	32	50	20	42	31	185	68	44	11	25	31	14	1
ACC_04612	conserved hypothetical protein			1	12	14	6	6	5	2	13	2	0	11	18	0	0	0	0
ACC_04613	putative glycerol kinase 3-like	K00864	KOG2517	572	288	268	482	454	79	140	144	693	540	425	939	629	566	146	101
ACC_04614	mitochondrial ubiquitin ligase activator of nfkb 1-lil	K15688	KOG1571	178	122	122	312	246	29	33	48	395	172	169	336	517	680	140	40

ACC_04615	conserved hypothetical protein	K08819	KOG0600	1954	1959	1828	1928	948	400	603	673	1756	1823	472	437	1183	1483	3615	2850
ACC_04616	conserved hypothetical protein			461	306	264	485	644	21	98	66	160	221	286	219	239	290	225	109
ACC_04617	armadillo repeat-containing protein 5-like		KOG4350	101	83	95	171	161	11	11	21	152	122	65	148	164	210	24	6
ACC_04618	transmembrane protein 120 homolog		KOG4758	590	379	346	564	469	75	217	203	823	607	392	965	611	621	404	176
ACC_04619	DNA-directed RNA polymerase III subunit RPC9-like		KOG4168	62	40	54	47	50	1	3	6	28	34	34	61	91	143	39	24
ACC_04620	protein KIAA0664 homolog	K03255	KOG1839	2117	900	735	1623	1544	292	556	665	1706	1778	798	627	2898	4648	7605	7075
ACC_04621	multiple epidermal growth factor-like domains protein 8		KOG0379	556	277	239	229	184	35	66	78	1159	1186	95	293	384	222	38	14
ACC_04622	intraflagellar transport protein 80 homolog		KOG1524	43	65	42	18	28	11	36	48	82	62	284	385	67	41	9	0
ACC_04623	peflin-like		KOG0037	116	71	75	161	168	8	7	12	105	39	93	221	100	72	7	9
ACC_04624	clavesin-2-like		KOG1471	104	90	31	58	45	56	107	95	207	190	398	419	75	93	115	89
ACC_04625	cytochrome b5 reductase 4-like		KOG0536	226	114	128	209	103	39	40	33	223	126	53	136	72	92	45	9
ACC_04626	conserved hypothetical protein			0	1	0	2	2	0	0	0	10	1	5	2	0	0	0	1
ACC_04627	conserved hypothetical protein	K11722	KOG1474	487	373	381	314	122	55	124	142	813	898	141	158	368	385	654	524
ACC_04628	vacuolar protein sorting-associated protein 72 hom	K11664	KOG2897	508	393	302	369	375	107	212	255	339	214	295	331	301	357	372	342
ACC_04629	solute carrier family 25 member 40-like	K15119	KOG0761	3828	1413	1351	4730	2824	260	273	402	3979	2651	1788	6514	7256	7600	2309	880
ACC_04630	LOW QUALITY PROTEIN	K13147		347	216	220	412	349	17	57	60	354	225	141	173	307	476	69	30
ACC_04631	polypeptide N-acetylgalactosaminyltransferase 3-li	K00710	KOG3736	160	117	104	197	165	11	22	16	98	64	50	108	88	113	32	5
ACC_04632	ethanolaminephosphotransferase 1-like	K00993	KOG2877	402	223	234	804	398	13	18	35	444	278	434	474	646	617	26	14
ACC_04633	conserved hypothetical protein			1	1	0	0	0	0	0	0	0	1	1	0	1	2	1	0
ACC_04634	cytoplasmic phosphatidylinositol transfer protein 1		KOG3668	342	146	151	352	304	14	10	22	221	158	109	228	189	203	23	12
ACC_04635	transmembrane protein 26-like		KOG4610	9	9	11	58	30	4	6	11	13	4	9	15	1	0	54	13
ACC_04636	conserved hypothetical protein		KOG3765	89	39	61	76	101	9	10	9	134	68	46	212	161	126	16	4
ACC_04637	NADH dehydrogenase	K03954	KOG3877	1246	716	773	855	899	82	118	171	2352	1084	576	2567	3364	3426	246	122
ACC_04638	spindle and kinetochore-associated protein 1-like		KOG4832	27	22	13	35	30	0	1	0	3	6	13	19	42	69	20	11
ACC_04639	conserved hypothetical protein			187	126	84	131	77	15	23	26	355	446	101	340	410	272	31	11
ACC_04640	LOW QUALITY PROTEIN		KOG1215	1979	1487	1077	1081	990	269	391	450	4665	7562	1578	1550	1561	1109	236	91
ACC_04641	nucleolar protein 10	K14788	KOG2321	609	286	277	514	592	92	231	212	345	321	250	254	1134	1680	1427	671
ACC_04642	vascular endothelial growth factor receptor 3-like		KOG1095	22	27	17	18	30	1	2	4	52	18	8	3	204	71	3	1
ACC_04643	lysine-specific demethylase 6A-like	K11447	KOG1246	558	448	293	234	115	96	104	96	1235	418	398	126	97	120	152	53
ACC_04644	tachykinin-like peptides receptor 99D-like isoform :	K04225	KOG4219	24	11	16	40	14	0	1	0	15	14	2	1	1	1	0	0
ACC_04645	conserved hypothetical protein		KOG4341	5	6	9	57	21	1	0	1	10	8	1	6	1	4	28	3
ACC_04646	cytochrome b-c1 complex subunit 7-like	K00417	KOG3440	611	413	393	1007	938	78	191	233	1200	365	561	985	690	1208	160	160
ACC_04647	sodium-dependent phosphate transporter 1-B-like	K14640	KOG2493	590	269	319	1069	672	37	57	91	289	136	139	159	23	50	33	7
ACC_04648	EF-hand domain-containing protein 1-like		KOG0043	95	125	119	158	164	8	12	21	99	69	35	33	49	35	9	1
ACC_04649	protein disulfide-isomerase A6-like isoform 1	K09584	KOG0191	900	788	598	1393	1349	51	97	142	926	988	271	1019	3087	3487	327	171
ACC_04650	gamma-tubulin complex component 5-like	K16572	KOG4344	376	245	258	324	390	23	62	43	515	453	218	257	436	406	35	15
ACC_04651	conserved hypothetical protein			216	189	156	180	153	19	27	38	271	331	70	110	242	222	71	30
ACC_04652	conserved hypothetical protein			228	553	207	178	97	208	247	466	1957	795	205	854	453	384	127	71
ACC_04653	riboflavin kinase-like	K00861	KOG3110	253	259	190	420	373	58	58	90	707	307	321	936	799	712	59	19
ACC_04654	protocadherin-15-like	K16500	KOG4289	231	119	141	264	85	28	42	59	416	305	106	83	98	131	181	67
ACC_04655	carbonic anhydrase 2-like		KOG0382	0	0	0	0	0	0	0	0	0	0	0	0	0	0	1	1
ACC_04656	LOW QUALITY PROTEIN		KOG2392	528	231	172	816	478	70	72	68	10599	5289	564	3946	2695	1072	193	53
ACC_04657	nuclear hormone receptor FTZ-F1	K08705	KOG4218	850	205	180	242	94	29	45	38	527	502	189	166	401	475	103	77
ACC_04658	39S ribosomal protein L12, mitochondrial	K02935	KOG1715	174	104	102	176	228	10	15	16	248	111	112	423	915	1151	82	24
ACC_04659	n-acetylgalactosamine kinase-like	K00849	KOG0631	83	74	77	227	138	8	9	15	141	350	67	99	640	789	110	10
ACC_04660	conserved hypothetical protein			72	83	70	137	51	23	28	30	49	46	7	5	8	17	14	1
ACC_04661	dolichol-phosphate mannosyltransferase subunit 3	K09659	KOG4841	32	42	44	64	77	0	3	1	9	30	68	212	63	49	2	3
ACC_04662	serine hydroxymethyltransferase, cytosolic-like	K00600	KOG2467	244	165	153	260	357	26	44	38	1005	1930	191	524	3114	2335	157	94
ACC_04663	conserved hypothetical protein			286	169	136	179	151	17	24	30	216	181	48	135	124	136	32	12
ACC_04664	AMP deaminase 2-like isoform 2	K01490	KOG1096	842	476	552	936	923	57	89	98	1652	1106	371	426	673	418	42	14
ACC_04665	NADH dehydrogenase	K03959	KOG4631	222	177	158	420	341	19	21	29	139	55	133	404	230	269	32	20
ACC_04666	translin-associated protein X		KOG3066	185	160	141	240	273	7	20	35	80	70	53	225	226	228	26	11
ACC_04667	digestive organ expansion factor homolog	K14774	KOG2340	285	213	255	380	407	31	89	103	440	423	147	325	532	535	78	33
ACC_04668	conserved hypothetical protein		KOG3700	132	99	125	118	104	19	46	52	282	466	153	100	100	229	20	9
ACC_04669	extended synaptotagmin-3-like isoform 2		KOG1028	533	322	272	356	411	48	66	65	860	834	254	577	588	418	61	20
ACC_04670	EF-hand domain-containing family member C2-like		KOG0043	1	4	3	21	6	1	2	0	6	11	74	45	49	8	3	2
ACC_04671	conserved hypothetical protein	K11800	KOG4227	642	401	372	898	678	107	130	178	762	508	295	345	573	642	294	99

ACC_04672	integrin alpha-8-like		KOG3637	64	41	33	64	73	3	9	9	46	138	36	42	28	10	13	4
ACC_04673	uroporphyrinogen-III synthase-like	K01719	KOG4132	53	33	20	53	60	5	5	12	78	58	47	85	90	121	9	6
ACC_04674	nucleoside diphosphate kinase 7	K00940	KOG0888	93	47	42	134	152	4	13	10	59	21	43	79	88	108	9	4
ACC_04675	conserved hypothetical protein		KOG0260	8	4	2	19	2	1	0	0	17	16	4	1	5	28	9	52
ACC_04676	hypothetical protein			0	0	0	0	0	0	0	0	0	0	0	0	0	0	0	0
ACC_04677	homogentisate 1,2-dioxygenase-like	K00451	KOG1417	30	14	7	68	11	4	6	5	316	676	134	967	274	452	63	34
ACC_04678	conserved hypothetical protein			497	348	323	539	510	48	86	104	555	442	351	609	655	567	102	29
ACC_04679	LOW QUALITY PROTEIN			33	9	7	25	19	1	1	1	51	53	9	174	86	84	4	6
ACC_04680	KH domain-containing, RNA-binding, signal transdu	K14942	KOG1588	548	384	377	367	218	75	96	117	378	325	167	260	474	579	717	413
ACC_04681	LMBR1 domain-containing protein 2 homolog		KOG2296	537	309	253	494	458	34	69	94	1203	909	224	1317	860	549	40	4
ACC_04682	histone-lysine N-methyltransferase SETDB1	K11421	KOG1141	356	291	260	486	433	33	78	73	389	361	180	229	550	741	182	77
ACC_04683	Wee1-like protein kinase	K06632	KOG0601	347	234	217	230	193	41	51	57	373	332	108	216	325	251	74	25
ACC_04684	sodium/bile acid cotransporter 7-like	K14347	KOG4821	272	146	160	265	269	15	24	30	201	119	187	186	63	53	9	3
ACC_04685	inositol-tetrakisphosphate 1-kinase-like	K00913		136	122	88	131	135	13	36	23	449	275	159	581	551	592	117	51
ACC_04686	conserved hypothetical protein		KOG2746	390	231	160	381	309	161	378	373	827	511	329	389	203	143	174	90
ACC_04687	protein Smaug homolog 2-like		KOG3791	366	345	365	565	256	62	99	122	451	335	150	70	315	421	280	62
ACC_04688	3-hydroxyisobutyryl-CoA hydrolase, mitochondrial-	K05605	KOG1684	349	240	254	295	340	35	37	36	502	414	199	755	1895	2622	233	85
ACC_04689	hormone-sensitive lipase-like	K07188	KOG4388	391	244	238	369	306	37	40	53	489	346	265	301	166	204	28	13
ACC_04690	peptidylprolyl isomerase domain and WD repeat-cc	K12736	KOG0882	424	251	277	480	452	57	150	129	632	343	319	313	519	647	94	29
ACC_04691	Voltage-dependent anion-selective channel protein 2		KOG3126	5	4	2	10	2	4	1	4	4	6	0	0	0	0	1	0
ACC_04692	condensin complex subunit 2-like	K06676	KOG2328	192	83	49	149	242	18	37	27	36	49	22	31	186	193	176	74
ACC_04693	nascent polypeptide-associated complex subunit al	K03626	KOG2239	967	643	573	976	594	122	188	182	947	767	468	1712	4741	6670	2481	1139
ACC_04694	conserved hypothetical protein			75	80	132	523	416	11	17	13	35	33	14	102	13	21	10	0
ACC_04695	TM2 domain-containing protein CG11103-like		KOG4272	73	67	58	129	109	5	16	26	125	33	65	142	173	206	42	10
ACC_04696	facilitated trehalose transporter Tret1-like		KOG0254	4	1	1	0	2	0	1	0	4	6	8	1	68	4	1	2
ACC_04697	myosin regulatory light chain 2		KOG0031	1111	158	29	438	189	56	80	53	4580	2506	610	1638	2433	1722	1269	513
ACC_04698	exostosin-3-like	K02370	KOG2264	251	171	139	234	219	21	23	31	415	406	95	223	396	242	16	2
ACC_04699	hypothetical protein			121	117	154	61	5	5	4	5	42	44	2	2	0	0	3	1
ACC_04700	conserved hypothetical protein	K15297	KOG3799	861	369	355	807	162	38	36	58	245	145	31	9	1	3	22	15
ACC_04701	prostaglandin E2 receptor EP4 subtype-like		KOG2087	2	3	3	20	7	0	4	1	4	3	0	4	155	54	90	22
ACC_04702	protein kinase DC2	K04345	KOG0616	16	5	7	55	29	10	18	6	831	259	94	86	31	54	21	16
ACC_04703	protein lethal(2)essential for life-like isoform 1		KOG3591	138	300	1010	2683	1287	59	102	237	954	533	797	911	13	72	33	20
ACC_04704	protein lethal(2)essential for life-like		KOG3591	348	279	977	787	504	34	49	91	158	61	129	237	105	156	52	22
ACC_04705	striatin isoform 2		KOG0642	936	441	412	776	500	45	41	65	1726	914	233	975	1167	1027	147	35
ACC_04706	lysM and putative peptidoglycan-binding domain-containing	protein 1-		91	51	50	82	68	8	10	11	160	113	56	165	110	113	10	8
ACC_04707	ATP-binding cassette sub-family B member 7, mito	K05662	KOG0057	544	272	241	377	438	44	70	61	1075	545	310	591	731	801	67	29
ACC_04708	tetratricopeptide repeat protein 19 homolog, mitochondrial		KOG1840	186	135	138	163	243	8	33	12	197	252	237	665	360	223	29	7
ACC_04709	kinesin 3C		KOG0245	278	225	258	223	250	13	30	34	325	213	171	73	671	558	170	120
ACC_04710	solute carrier family 22 member 21-like		KOG0255	704	199	190	325	125	137	203	227	857	662	110	364	727	518	1437	1001
ACC_04711	protein ETHE1, mitochondrial-like		KOG0814	175	92	98	346	272	12	23	23	214	198	100	276	216	281	44	20
ACC_04712	beta amyloid protein precursor-like isoform 1	K04520	KOG3540	6051	3938	4010	4946	1207	373	398	517	5738	2883	1947	1707	215	349	361	85
ACC_04713	conserved hypothetical protein			385	201	157	392	384	91	192	128	366	207	376	673	398	370	112	54
ACC_04714	conserved hypothetical protein		KOG4217	196	96	86	138	66	20	46	46	183	142	35	90	157	164	102	97
ACC_04715	synaptotagmin-7-like		KOG1028	914	767	609	1573	864	51	85	103	583	424	123	25	10	29	16	12
ACC_04716	probable exonuclease mut-7 homolog		KOG2207	614	399	325	415	489	67	148	120	972	841	498	780	862	846	107	44
ACC_04717	conserved hypothetical protein		KOG4793	171	179	175	217	210	8	30	14	138	126	117	285	300	369	74	29
ACC_04718	e3 ubiquitin-protein ligase LRSAM1-like	K10641	KOG0532	367	167	162	289	270	30	48	66	401	291	139	353	297	186	62	26
ACC_04719	ras-related protein Rab-21-like	K07890	KOG0088	195	118	85	223	231	20	26	28	274	212	100	435	486	320	46	10
ACC_04720	UPF0554 protein C2orf43 homolog		KOG3975	374	221	185	389	420	23	31	40	436	343	271	905	1090	864	44	20
ACC_04721	zinc finger protein 184-like		KOG3608	162	82	93	129	142	18	27	48	110	111	53	84	67	42	34	16
ACC_04722	peptidyl-prolyl cis-trans isomerase B precursor	K03768	KOG0880	949	663	376	1061	1279	116	154	219	1817	921	622	2869	4931	4383	187	117
ACC_04723	ribosomal L1 domain-containing protein CG13096-l	K14775	KOG1685	267	225	174	368	370	43	84	147	304	259	177	341	1059	1758	370	265
ACC_04724	splicing factor U2AF 50 kDa subunit	K12837	KOG0120	338	218	247	715	516	32	66	69	372	295	233	314	544	811	148	98
ACC_04725	LOW QUALITY PROTEIN	K00849	KOG0631	115	71	44	108	104	25	45	53	178	159	71	175	513	324	86	32
ACC_04726	conserved hypothetical protein			37	15	20	27	15	13	13	13	37	156	23	149	175	239	482	118
ACC_04727	putative serine protease K12H4.7-like		KOG2182	367	195	145	318	375	40	48	64	621	488	225	557	1119	701	33	13
ACC_04728	conserved hypothetical protein			4308	2587	2083	2353	3672	282	819	958	940	914	1052	538	444	501	1126	441

ACC_04729	nitric oxide synthase-interacting protein homolog	K13125	KOG3039	244	185	191	319	274	70	104	141	332	166	259	361	250	297	140	105
ACC_04730	endocuticle structural glycoprotein SgAbd-1-like			2	5	3	25	11	1	2	2	11	4	16	33	0	5	0	1
ACC_04731	conserved hypothetical protein			279	56	37	52	42	19	22	29	448	113	328	864	99	56	27	7
ACC_04732	GRAM domain-containing protein 1A-like		KOG1032	639	343	314	504	471	82	119	151	514	500	221	662	559	380	118	48
ACC_04733	conserved hypothetical protein	K07441	KOG2122	1192	726	565	1202	801	347	749	768	1042	769	684	319	671	824	1798	1051
ACC_04734	solute carrier family 35 member F3-like	K15288	KOG4314	283	187	123	233	134	86	77	115	588	371	54	101	22	28	17	3
ACC_04735	calcium and integrin-binding protein 1-like		KOG0038	68	26	28	56	55	5	2	5	26	13	15	59	40	46	8	6
ACC_04736	proteasome subunit beta type-5-like	K02737	KOG0175	516	316	289	1268	750	105	119	175	1518	732	1387	1888	2151	3082	401	239
ACC_04737	death-associated protein 1-like			487	701	425	619	274	347	530	1023	587	346	646	1130	634	497	322	126
ACC_04738	mitogen-activated protein kinase 15-like	K08293	KOG0660	7	5	8	14	4	0	2	0	36	15	672	330	1	4	13	9
ACC_04739	WD repeat-containing protein 37-like		KOG0300	389	221	224	605	496	30	26	45	459	241	288	394	307	376	37	22
ACC_04740	contactin-like		KOG3513	273	154	104	145	123	21	28	31	461	563	107	495	698	704	55	13
ACC_04741	LOW QUALITY PROTEIN		KOG0518	577	268	269	675	209	81	90	86	2819	2064	219	175	1000	534	689	757
ACC_04742	negative elongation factor D-like	K15181		202	120	142	305	316	26	32	47	301	244	146	231	330	418	42	26
ACC_04743	e3 ubiquitin-protein ligase ZNRF1-like	K10694	KOG0801	138	67	59	126	100	12	17	23	459	289	107	261	378	283	56	17
ACC_04744	BRCA1-A complex subunit BRE-like	K12173		404	230	319	451	518	25	46	59	347	222	241	539	798	937	64	35
ACC_04745	dynein light chain roadblock-type 2	K10419	KOG4115	96	78	123	106	138	9	9	7	78	37	57	247	138	146	9	6
ACC_04746	exocyst complex component 6B-like isoform 2		KOG2176	347	250	283	449	390	51	84	69	535	282	221	281	319	415	85	34
ACC_04747	proliferation-associated protein 2G4-like		KOG2776	550	335	244	323	259	63	80	110	869	978	262	1101	3956	4406	725	355
ACC_04748	ankyrin-3-like		KOG4177	816	607	550	669	343	62	112	134	765	472	166	167	17	28	28	17
ACC_04749	probable DNA mismatch repair protein Msh6	K08737	KOG0217	237	144	122	276	272	34	86	68	301	149	164	118	306	493	519	418
ACC_04750	programmed cell death protein 2-like	K14801	KOG2061	535	343	353	991	895	57	79	109	639	301	164	342	373	467	63	16
ACC_04751	dynactin subunit 4-like	K10426	KOG3896	303	271	310	422	339	30	36	51	340	243	109	327	427	459	59	25
ACC_04752	pyruvate kinase-like	K00873	KOG2323	2654	1291	1324	3251	3086	192	202	259	7223	3072	1613	5038	7565	6474	753	245
ACC_04753	Eukaryotic translation initiation factor 1A, X-chrom	K03236	KOG3403	836	421	335	789	753	149	300	254	940	531	581	1139	1261	1839	1209	993
ACC_04754	alanine--glyoxylate aminotransferase 2-like	K14286	KOG1403	1275	1235	1595	5801	4000	207	276	411	1833	1297	223	225	1280	102	5	45
ACC_04755	vitellogenin precursor		KOG4338	833	2623	367	660	256	875	801	623	73970	926501	2	50	252	10	0	0
ACC_04756	glutathione S-transferase S4		KOG1695	29	3	4	29	22	18	33	39	278	13	82	339	843	565	318	152
ACC_04757	dnaJ homolog subfamily A member 1	K09502	KOG0712	2777	1292	1402	2987	1962	203	252	360	4896	3256	1069	3337	4689	6692	1142	642
ACC_04758	venom protease		KOG3627	66	38	47	243	227	25	25	21	1951	1783	2720	7090	412	42	7	15
ACC_04759	stromal membrane-associated protein 1-like	K12486	KOG0703	352	245	263	484	253	35	49	69	545	442	146	419	498	467	126	47
ACC_04760	coiled-coil domain-containing protein 115-like			102	74	82	55	40	20	20	23	95	51	41	83	109	116	15	20
ACC_04761	DNA replication licensing factor Mcm2-like	K02540	KOG0477	419	239	217	261	219	40	42	54	447	152	185	73	345	677	68	38
ACC_04762	protein zwilch homolog			107	73	67	46	88	1	6	5	35	25	26	36	58	49	11	3
ACC_04763	intraflagellar transport protein 88 homolog isoform	K16474	KOG2003	256	198	174	261	171	36	33	48	426	217	238	239	136	124	23	9
ACC_04764	hexokinase-2-like		KOG1369	625	307	406	1010	968	43	60	71	2308	2595	719	2357	2752	1139	105	59
ACC_04765	sestrin-1-like isoform 1	K10141		257	161	153	471	109	39	42	83	308	254	167	83	8	4	7	35
ACC_04766	conserved hypothetical protein		KOG3598	875	801	1042	1267	265	62	79	98	1373	1345	271	210	211	293	583	168
ACC_04767	hypothetical protein			26	19	10	12	1	1	1	1	15	5	2	12	0	0	3	1
ACC_04768	protein FAM162B-like isoform 2			215	158	133	288	333	32	39	46	362	146	272	586	479	479	133	84
ACC_04769	frataxin homolog, mitochondrial		KOG3413	127	76	58	119	118	3	1	4	120	84	64	219	147	204	10	5
ACC_04770	cuticular protein 19		KOG0260	229	77	63	37	15	7	8	9	125	12	7	1189	12	3	4	7
ACC_04771	progestin and adipoQ receptor family member 3-like		KOG0748	459	376	546	937	561	18	19	18	1055	978	128	594	287	372	118	11
ACC_04772	WD repeat-containing protein 36-like	K14554	KOG1539	172	119	105	141	171	9	11	14	218	220	100	193	502	727	45	29
ACC_04773	triple functional domain protein		KOG0032	286	195	216	588	328	41	50	51	386	246	145	104	129	131	56	16
ACC_04774	SCY1-like protein 2-like		KOG2137	111	72	86	75	46	14	28	29	91	93	22	9	14	9	34	11
ACC_04775	zinc finger protein 540-like		KOG2462	146	95	102	243	165	16	28	47	69	49	46	66	7	13	21	1
ACC_04776	UPF0378 protein KIAA0100-like		KOG1910	739	517	454	478	356	105	110	130	1227	863	413	288	496	495	75	38
ACC_04777	corepressor interacting with RBPJ 1-like, partial	K06066	KOG3794	2723	1819	1959	1832	1239	237	519	615	1360	1003	581	398	571	1020	1363	647
ACC_04778	pre-mRNA-processing-splicing factor 8-like	K12856	KOG1795	1650	1134	940	1472	1117	146	215	237	2188	2464	568	1086	2462	3076	628	173
ACC_04779	ecto-NOX disulfide-thiol exchanger 1-like		KOG0120	487	330	387	540	363	66	178	178	747	416	178	245	402	526	449	334
ACC_04780	DPH4 homolog		KOG0715	149	146	128	122	117	24	40	67	186	130	109	309	387	490	102	71
ACC_04781	alpha-parvin-like	K06275	KOG3631	290	196	191	346	274	25	44	30	1007	595	223	869	875	617	137	80
ACC_04782	conserved hypothetical protein			3	6	3	15	13	0	1	1	4	8	9	13	3	2	0	0
ACC_04783	conserved hypothetical protein			399	357	379	581	621	106	175	174	520	259	559	630	803	1166	284	158
ACC_04784	SRR1-like protein-like		KOG3131	249	178	178	282	356	14	28	29	355	294	249	497	700	710	56	18
ACC_04785	conserved hypothetical protein			119	123	96	221	212	14	24	28	112	79	157	229	283	248	60	18

ACC_04786	protein hu-li tai shao-like		KOG3699	5100	2286	1880	5016	3092	824	1539	1606	8070	3164	2550	3507	3439	4437	5359	4565
ACC_04787	conserved hypothetical protein	K12846	KOG0147	146	63	78	192	119	15	22	40	106	48	63	111	139	227	152	79
ACC_04788	regulator of G-protein signaling 12	K16449	KOG3589	1605	1358	1314	1871	1491	110	181	201	1071	800	456	466	542	675	200	76
ACC_04789	conserved hypothetical protein	K16723	KOG3346	571	581	410	1558	961	108	140	174	1584	945	887	1529	4690	4244	664	269
ACC_04790	LOW QUALITY PROTEIN	K01280	KOG1114	666	326	261	308	272	50	82	84	684	608	118	343	1036	946	202	76
ACC_04791	zinc finger CCHC domain-containing protein 8 homo	K13128	KOG2673	216	177	160	189	153	32	36	58	378	351	97	293	339	319	98	64
ACC_04792	f-box only protein 42-like isoform 2	K10317	KOG0379	470	257	275	602	457	35	51	64	548	441	230	695	534	533	111	34
ACC_04793	programmed cell death protein 10-like		KOG4025	270	174	152	268	265	35	49	39	305	120	178	745	252	203	29	5
ACC_04794	ankyrin repeat domain-containing protein 13B-like		KOG0522	369	233	246	425	304	35	66	79	681	368	196	272	335	260	48	10
ACC_04795	WD repeat domain phosphoinositide-interacting protein 3-li		KOG2111	263	165	186	163	138	34	50	52	314	212	70	207	239	214	48	23
ACC_04796	UPF0193 protein EVG1-like			1	1	2	0	0	1	0	1	3	2	2	7	0	0	0	0
ACC_04797	sorting nexin-8-like		KOG2273	317	251	265	374	290	69	83	132	507	666	325	534	264	159	38	7
ACC_04798	conserved hypothetical protein		KOG4441	517	380	331	509	303	62	118	142	604	684	124	333	716	989	493	259
ACC_04799	hypothetical protein			1	2	0	2	0	0	1	1	1	1	0	1	0	0	0	0
ACC_04800	hypothetical protein			6	2	1	5	1	0	0	0	2	3	0	1	10	4	2	2
ACC_04801	hypothetical protein			2	2	1	7	2	0	0	0	0	5	0	0	0	0	1	0
ACC_04802	enolase-like		KOG2670	13	5	3	7	5	1	7	1	53	108	5	6	20	8	2	2
ACC_04803	conserved hypothetical protein			4	9	14	63	33	0	2	3	13	3	15	5	1517	1483	78	239
ACC_04804	CTP synthase-like	K01937	KOG2387	188	165	150	287	298	19	28	36	179	126	109	162	506	762	76	27
ACC_04805	s-adenosylmethionine mitochondrial carrier protei	K15111	KOG0768	204	143	166	173	232	25	54	55	228	144	156	337	256	310	26	19
ACC_04806	asparagine synthetase domain-containing protein 1-like		KOG0573	248	115	122	163	174	13	19	23	425	211	244	337	387	460	32	5
ACC_04807	conserved hypothetical protein			1230	1021	1090	1398	1278	166	355	311	1090	485	792	774	567	620	141	66
ACC_04808	UPF0505 protein C16orf62 homolog		KOG3682	208	159	120	158	181	9	23	23	199	249	90	246	409	434	35	4
ACC_04809	LOW QUALITY PROTEIN		KOG0517	2627	1516	1331	1936	1460	494	1120	1051	1643	960	1102	1121	784	1133	2900	1796
ACC_04810	zinc finger protein 511-like		KOG4173	79	32	24	61	38	5	27	12	36	30	79	153	68	55	54	25
ACC_04811	probable alpha-ketoglutarate-dependent dioxygen	K10768	KOG3200	47	40	41	80	79	5	15	8	61	32	67	101	54	101	7	5
ACC_04812	o-glucosyltransferase rumi homolog, partial	K13667	KOG2458	78	78	67	80	113	17	19	10	95	80	112	156	81	144	32	12
ACC_04813	queuine tRNA-ribosyltransferase subunit QTRTD1 h	K15407	KOG3909	50	41	23	46	48	5	5	5	46	89	22	87	417	631	47	6
ACC_04814	B(0,+)-type amino acid transporter 1-like		KOG1287	65	36	30	94	72	4	6	4	80	86	9	57	126	108	38	14
ACC_04815	conserved oligomeric Golgi complex subunit 6-like		KOG3758	118	95	107	282	245	12	24	20	132	106	90	164	191	205	24	1
ACC_04816	conserved hypothetical protein			27	11	13	23	5	3	3	3	44	35	8	19	17	16	16	10
ACC_04817	carboxyl-terminal PDZ ligand of neuronal nitric oxid	K16513	KOG4815	307	183	174	263	89	15	17	27	130	88	95	80	109	117	118	40
ACC_04818	endoplasmic reticulum resident protein 44		KOG0912	458	333	231	337	282	106	164	230	524	524	233	477	779	927	181	71
ACC_04819	conserved hypothetical protein	K02649	KOG4637	919	502	454	1069	519	116	222	277	1320	952	328	176	710	582	552	219
ACC_04820	BTB/POZ domain-containing protein KCTD15-like		KOG2723	19	1	5	15	3	0	2	0	11	17	1	2	2	1	2	1
ACC_04821	tubulin delta chain-like		KOG1374	35	32	46	61	68	2	6	8	18	49	41	40	65	79	11	4
ACC_04822	solute carrier family 35 member C2-like		KOG3416	34	34	32	97	79	7	9	19	70	50	26	59	88	101	23	6
ACC_04823	solute carrier family 35 member C2-like isoform 1	K15280	KOG1443	113	69	63	95	113	6	8	13	272	116	54	202	199	139	8	1
ACC_04824	LITAF-like protein-like			57	45	27	58	63	7	10	9	140	93	82	387	160	150	17	5
ACC_04825	RNA polymerase II subunit B1 CTD phosphatase Rpap2-like		KOG4780	1145	451	418	758	1365	35	140	146	562	428	657	1050	750	828	254	184
ACC_04826	coiled-coil domain-containing protein 102A-like	K16759	KOG0161	353	238	215	306	365	65	134	149	274	177	186	168	236	210	191	137
ACC_04827	ADP,ATP carrier protein-like	K05863	KOG0749	7324	2536	2153	6034	2975	590	636	777	13438	4221	2419	9750	10235	11632	7842	1180
ACC_04828	protein arginine N-methyltransferase 7-like	K11438	KOG1501	312	202	203	553	535	32	39	54	364	186	192	280	396	631	52	21
ACC_04829	conserved hypothetical protein	K12866	KOG4672	423	334	481	510	250	57	73	102	388	267	160	200	357	583	492	316
ACC_04830	conserved hypothetical protein		KOG3598	1019	848	780	929	517	50	70	75	994	517	321	645	1088	1234	496	260
ACC_04831	conserved hypothetical protein			595	467	412	220	137	27	49	37	428	423	59	135	209	212	72	24
ACC_04832	serine/threonine-protein kinase TBK1	K05410	KOG4250	253	224	233	375	352	22	44	70	315	185	131	208	126	131	34	17
ACC_04833	mediator of RNA polymerase II transcription subun	K15170		99	99	106	121	125	11	11	18	94	64	48	141	128	175	31	12
ACC_04834	methylosome protein 50	K13221	KOG0277	170	132	149	220	224	12	19	24	166	148	154	283	484	776	79	18
ACC_04835	alpha-N-acetylglucosaminidase-like	K01205	KOG2233	64	30	39	113	54	5	14	8	103	54	12	26	1520	1306	51	36
ACC_04836	conserved hypothetical protein			152	118	74	101	91	15	46	51	130	70	70	109	164	189	102	52
ACC_04837	transcription elongation factor B polypeptide 3 isof	K15076	KOG2821	708	428	317	437	372	93	169	176	586	394	391	358	620	557	384	198
ACC_04838	putative ankyrin repeat protein FPV014-like		KOG0817	346	209	197	331	382	20	47	39	473	232	177	377	439	616	60	7
ACC_04839	conserved hypothetical protein			25	13	14	18	12	0	1	4	51	49	2	3	10	8	1	1
ACC_04840	pre-mRNA-splicing factor 18-like	K12817	KOG2808	112	55	62	187	183	16	45	40	138	82	125	180	154	280	82	66
ACC_04841	ADP-ribosylation factor-like protein 13B-like		KOG0073	187	146	132	150	166	8	17	21	179	137	63	176	188	192	48	15
ACC_04842	chorion peroxidase-like			25	42	2	1	3	37	19	36	108	117	3	1	13	9	0	0

ACC_04843	2-oxoisovalerate dehydrogenase subunit beta, mito	K00167	KOG0525	1	1	0	19	11	1	5	4	162	307	185	619	900	465	287	149
ACC_04844	conserved hypothetical protein			0	0	0	0	0	0	0	0	0	0	0	0	0	0	0	0
ACC_04845	exocyst complex component 3	K06110	KOG2286	150	118	92	180	139	7	10	11	217	162	62	138	122	104	2	3
ACC_04846	cell division cycle 5-like protein	K12860	KOG0050	532	416	391	662	358	97	135	167	522	432	247	441	521	727	522	307
ACC_04847	probable phosphoserine aminotransferase-like	K00831	KOG2790	232	139	114	262	324	30	54	60	653	857	258	643	2122	1833	140	71
ACC_04848	conserved hypothetical protein		KOG3130	297	188	204	219	258	21	31	28	191	170	136	308	202	238	35	18
ACC_04849	conserved hypothetical protein		KOG4628	270	164	179	241	190	30	32	42	404	277	110	187	300	289	54	11
ACC_04850	conserved hypothetical protein	K08827	KOG0670	2258	1812	1707	2359	1928	516	1028	1105	1777	1227	999	416	910	1009	2524	1930
ACC_04851	zinc carboxypeptidase A 1-like		KOG2650	1	3	0	2	1	1	1	3	8	6	4	1	7177	2299	177	6
ACC_04852	josephin-like protein-like	K15235	KOG2934	81	57	38	120	135	12	28	27	164	88	99	202	113	112	17	7
ACC_04853	dynein light chain 4, axonemal-like	K10412	KOG3430	83	52	40	134	76	6	4	3	46	15	40	85	11	6	0	0
ACC_04854	GTP-binding protein 1-like		KOG0463	440	241	193	318	275	59	91	88	902	420	213	429	489	446	77	25
ACC_04855	60S ribosomal protein L10-like	K02866	KOG0857	1259	934	539	1592	1321	351	421	558	1714	1120	1508	2575	7514	10075	1663	1031
ACC_04856	sesquipedalian-1-like			62	40	60	63	58	5	6	5	120	49	59	113	60	53	10	2
ACC_04857	conserved hypothetical protein			10	7	4	5	2	2	3	2	5	4	13	2	7	8	12	2
ACC_04858	protein sel-1 homolog 1-like	K14026	KOG1550	1240	1077	1055	1013	586	221	225	320	2663	2246	836	1643	1677	1537	295	124
ACC_04859	LOW QUALITY PROTEIN	K14851		194	104	114	119	158	18	60	38	92	72	123	81	340	657	482	319
ACC_04860	conserved hypothetical protein			45	59	62	66	49	7	28	14	21	18	8	19	6	7	30	16
ACC_04861	Y+L amino acid transporter 2-like		KOG1287	146	113	101	127	125	9	12	24	128	128	58	183	391	397	41	16
ACC_04862	ATPase family AAA domain-containing protein 1-A-like		KOG0737	266	203	167	222	182	44	59	74	518	383	132	732	715	757	94	35
ACC_04863	protein bicaudal C		KOG2208	367	224	90	86	43	76	118	140	540	829	96	166	491	161	139	66
ACC_04864	cleavage stimulation factor subunit 2-like	K14407	KOG0108	289	233	235	465	233	20	38	42	313	250	179	331	364	484	133	38
ACC_04865	conserved hypothetical protein			156	54	108	210	126	42	55	69	155	67	71	34	139	143	361	271
ACC_04866	methylthioribose-1-phosphate isomerase-like	K08963	KOG1468	77	80	106	516	377	17	19	35	154	100	62	118	763	942	153	32
ACC_04867	surfeit locus protein 4 homolog		KOG3998	299	237	119	369	399	49	60	80	597	368	155	751	1266	1232	121	18
ACC_04868	SET and MYND domain-containing protein 3	K11426	KOG2084	114	90	99	136	146	14	33	36	326	139	161	167	232	239	41	19
ACC_04869	putative GTP-binding protein Parf-like		KOG0084	478	357	313	441	351	121	231	283	526	423	266	510	419	252	341	213
ACC_04870	target of rapamycin complex 2 subunit MAPKAP1-like		KOG3739	194	100	124	216	191	7	24	26	148	118	76	134	126	141	15	6
ACC_04871	calcyclin-binding protein-like	K04507	KOG3260	369	222	332	583	669	20	45	56	198	213	169	549	1046	1335	348	202
ACC_04872	conserved hypothetical protein			256	179	204	164	181	8	14	13	192	181	67	225	242	265	51	5
ACC_04873	conserved hypothetical protein			197	115	114	290	308	10	15	19	180	48	199	368	285	323	23	13
ACC_04874	vesicle-trafficking protein SEC22b-B-like	K08517	KOG0862	236	164	120	230	255	16	31	65	577	277	108	623	581	640	48	19
ACC_04875	ATP-binding cassette sub-family F member 1-like is	K06184	KOG0066	483	242	139	472	341	104	218	267	387	374	197	258	1089	1318	1619	1551
ACC_04876	dual specificity mitogen-activated protein kinase ki	K04431	KOG0983	214	197	192	431	164	48	52	78	409	282	160	132	208	154	150	20
ACC_04877	histone-lysine N-methyltransferase EHMT1-like iso	K11420	KOG1082	338	203	185	180	152	20	43	36	459	363	79	142	408	441	149	87
ACC_04878	surfeit locus protein 6 homolog			951	608	610	524	938	199	554	542	552	243	618	488	595	997	1738	1533
ACC_04879	conserved hypothetical protein			3	3	7	18	5	0	0	4	7	7	8	7	1	0	1	3
ACC_04880	mediator of RNA polymerase II transcription subun	K15139	KOG3304	17	14	15	52	58	1	3	6	21	14	21	29	37	52	7	4
ACC_04881	conserved hypothetical protein		KOG4282	16	11	4	32	21	1	3	3	8	3	7	11	6	12	17	15
ACC_04882	conserved hypothetical protein			368	158	184	186	57	18	21	29	314	161	44	36	24	28	11	0
ACC_04883	protein FAM102B-like isoform 2			329	190	280	453	203	49	90	153	291	285	268	151	87	96	227	203
ACC_04884	estradiol 17-beta-dehydrogenase 8-like		KOG1200	188	127	77	179	184	42	83	111	490	422	219	445	1452	1638	218	97
ACC_04885	60 kDa SS-A/Ro ribonucleoprotein-like	K11089	KOG4465	215	72	51	63	71	13	30	32	265	360	38	254	270	139	13	22
ACC_04886	conserved hypothetical protein	K08866	KOG0596	346	239	248	309	276	24	59	61	193	161	96	135	309	361	145	43
ACC_04887	folylpolyglutamate synthase, mitochondrial-like	K01930	KOG2525	147	116	112	109	192	13	23	21	489	475	224	439	923	518	26	7
ACC_04888	integrator complex subunit 4-like	K13141	KOG2259	314	219	218	418	266	26	53	74	362	248	203	250	345	285	42	10
ACC_04889	ribonuclease 3	K03685	KOG1817	410	235	249	376	285	39	76	72	513	311	176	172	294	335	65	38
ACC_04890	sex-regulated protein janus-A-like			84	83	51	272	224	13	17	21	150	71	137	170	311	387	58	22
ACC_04891	conserved hypothetical protein		KOG0231	74	52	42	83	97	7	7	10	42	26	10	28	38	32	7	4
ACC_04892	HIG1 domain family member 2A-like		KOG4431	108	64	93	107	192	4	1	0	87	34	60	219	163	121	4	5
ACC_04893	Dosage compensation complex, subunit MLE		KOG0921	1209	456	305	614	456	203	498	459	760	587	354	423	317	428	2286	2250
ACC_04894	conserved hypothetical protein			151	94	74	130	101	34	117	104	526	209	103	23	143	107	87	35
ACC_04895	pupal cuticle protein C1B-like			34	14	13	26	8	1	3	1	47	10	2	13	3148	1704	80	1877
ACC_04896	60S ribosomal protein L26 isoform 1	K02898	KOG3401	916	1019	426	988	521	196	351	400	695	510	802	1346	5228	5696	1132	821
ACC_04897	conserved hypothetical protein			465	284	270	255	395	52	249	242	286	162	241	326	325	405	536	277
ACC_04898	2-oxoglutarate and iron-dependent oxygenase domain-conta		KOG3844	104	68	69	118	100	26	34	55	163	122	31	48	184	509	128	64
ACC_04899	tetratricopeptide repeat protein 27-like		KOG1128	498	363	267	529	697	40	79	87	848	1157	342	1424	3342	2721	150	72

ACC_04900	mannose-P-dolichol utilization defect 1 protein hor	K09660	KOG3211	178	124	130	226	200	14	12	17	291	244	168	574	646	453	21	3
ACC_04901	condensin complex subunit 1	K06677	KOG0414	676	445	399	509	557	107	248	272	926	579	587	262	884	1345	900	413
ACC_04902	thiamine transporter 2-like	K14610	KOG3810	141	87	54	108	156	5	8	15	72	93	52	111	210	168	22	14
ACC_04903	hypothetical protein			0	0	0	0	0	0	0	0	0	0	0	0	0	0	0	0
ACC_04904	UPF0047 protein yjbQ-like		KOG3267	55	24	13	52	45	3	6	5	150	211	20	156	191	154	20	4
ACC_04905	inositol-3-phosphate synthase 1-B isoform 1	K01858	KOG0693	1969	1265	2051	5789	4630	67	135	214	2511	2358	599	1734	2779	1885	180	51
ACC_04906	conserved hypothetical protein			1	2	6	7	11	0	1	1	2	0	3	0	3	1	0	0
ACC_04907	cyclin-dependent kinase 20-like isoform 1	K08817	KOG0659	81	66	60	60	87	7	12	15	40	34	63	119	113	141	30	16
ACC_04908	LOW QUALITY PROTEIN	K05633	KOG0940	646	386	376	388	367	54	115	112	1101	1248	232	612	983	912	134	43
ACC_04909	protein SYS1 homolog		KOG4697	98	57	77	136	181	14	19	18	112	141	158	484	311	294	16	4
ACC_04910	WD repeat-containing protein YPL183C-like, partial		KOG0974	246	122	111	152	258	13	26	26	353	312	155	241	383	306	19	6
ACC_04911	trafficking protein particle complex subunit 5-like		KOG3315	145	99	98	257	225	21	41	46	202	89	191	327	366	297	45	15
ACC_04912	cathepsin L-like	K01365	KOG1543	1791	1722	1504	1926	1845	499	603	850	5908	3732	1621	3431	3030	2954	337	148
ACC_04913	phosphatidylinositol-glycan biosynthesis class X prc	K07541		42	30	26	54	59	3	3	2	8	17	17	41	112	98	19	5
ACC_04914	THO complex subunit 5 homolog	K13174	KOG2216	267	171	166	248	231	22	34	52	339	297	72	275	390	369	75	47
ACC_04915	conserved hypothetical protein			5	4	5	10	3	1	1	5	3	3	6	5	112	146	34	27
ACC_04916	ribonuclease P protein subunit p20-like	K14527		80	55	50	100	87	1	5	12	71	75	44	159	186	144	11	4
ACC_04917	transmembrane protein 203-like		KOG3879	30	33	30	57	62	3	0	7	44	27	38	74	78	94	5	0
ACC_04918	conserved hypothetical protein		KOG2146	707	477	404	898	532	68	159	166	573	273	99	23	91	137	190	101
ACC_04919	LOW QUALITY PROTEIN		KOG1472	200	95	108	204	193	21	32	49	236	197	117	211	200	281	99	30
ACC_04920	actin, clone 403-like	K05692	KOG0676	18	18	16	30	12	4	3	3	8	3	11	1	11	21	14	1
ACC_04921	GTP-binding protein 10 homolog		KOG1489	367	285	342	453	369	22	38	46	313	239	156	470	732	875	176	59
ACC_04922	10 kDa heat shock protein, mitochondrial-like	K04078	KOG1641	549	266	293	2165	958	39	95	101	249	357	490	753	4261	6040	2479	1755
ACC_04923	39S ribosomal protein L20, mitochondrial	K02887	KOG4707	166	170	209	259	286	32	52	52	174	88	141	282	325	518	88	61
ACC_04924	conserved hypothetical protein	K11667		13	6	14	12	20	0	3	3	7	8	11	15	27	20	4	0
ACC_04925	conserved hypothetical protein		KOG0611	504	353	320	508	228	110	175	141	1102	747	107	140	268	182	444	296
ACC_04926	spermatogenesis-associated protein 6-like			64	24	28	72	35	5	16	15	69	41	189	308	39	53	49	29
ACC_04927	conserved hypothetical protein		KOG0845	380	339	268	314	153	30	38	54	448	507	75	153	1156	1434	382	162
ACC_04928	growth factor receptor-bound protein 14-like			23	11	7	30	17	6	23	9	262	76	10	71	8	37	15	5
ACC_04929	growth factor receptor-bound protein 14-like		KOG3751	188	149	151	608	166	40	53	81	505	333	411	680	38	79	73	62
ACC_04930	sodium-coupled monocarboxylate transporter 2-like isoform		KOG2349	312	191	217	626	344	21	36	51	373	162	39	200	52	80	26	12
ACC_04931	hsp70-binding protein 1-like	K09562	KOG2691	327	293	379	580	456	22	43	70	442	343	281	775	766	1045	106	56
ACC_04932	LOW QUALITY PROTEIN		KOG3133	412	186	197	309	358	38	53	127	767	379	285	776	541	538	91	57
ACC_04933	conserved hypothetical protein			0	0	1	1	1	0	0	1	1	2	0	1	0	0	0	0
ACC_04934	roundabout homolog 2		KOG4222	258	135	129	118	65	9	13	17	384	239	73	76	100	117	129	42
ACC_04935	probable dolichyl pyrophosphate Glc1Man9GlcNAc	K03849	KOG2576	329	269	228	372	435	38	52	66	576	287	297	470	443	515	46	10
ACC_04936	synembryn		KOG4464	622	308	317	867	959	40	54	91	577	322	356	803	287	437	69	26
ACC_04937	calnexin isoform 1	K08054	KOG0675	2089	961	876	2041	1741	359	774	1057	3405	1983	2447	3956	2450	2393	634	362
ACC_04938	conserved hypothetical protein			158	164	110	101	132	8	17	15	132	147	156	119	130	137	18	6
ACC_04939	40S ribosomal protein S2 isoform 2	K02981	KOG0877	2824	1834	1580	7079	3576	579	834	1267	3034	2291	1859	4177	10612	12206	4189	1778
ACC_04940	conserved hypothetical protein		KOG4848	1293	633	566	982	1282	279	644	644	718	451	732	639	1037	1670	2449	2016
ACC_04941	twinfilin	K08870	KOG1747	396	300	239	656	490	22	51	47	431	377	150	365	492	400	54	40
ACC_04942	probable cationic amino acid transporter-like	K13871	KOG1286	788	329	426	1323	626	78	135	172	380	250	61	25	5	13	30	10
ACC_04943	serine palmitoyltransferase 1-like	K00654	KOG1358	698	536	477	754	784	42	63	62	840	612	405	869	1001	1009	98	41
ACC_04944	60S acidic ribosomal protein P0 isoform 1	K02941	KOG0815	2540	2130	1090	3096	2235	671	808	800	2907	3308	1202	5506	20298	21023	5195	1333
ACC_04945	sodium/hydrogen exchanger 7	K12041	KOG1965	455	334	307	337	290	31	37	60	1061	1098	215	776	324	153	12	12
ACC_04946	lysine-specific demethylase 6A-like	K11447	KOG1126	40	27	29	24	14	5	3	1	166	87	41	30	19	29	12	3
ACC_04947	serotonin receptor		KOG4220	4	6	2	6	2	13	7	12	11	2	1	0	2712	893	344	5016
ACC_04948	cytosolic 5'-nucleotidase 3-like	K01081	KOG3128	87	64	65	123	149	2	2	8	90	63	40	121	153	219	12	15
ACC_04949	TP53-regulated inhibitor of apoptosis 1-like		KOG3481	62	62	97	139	158	3	6	5	35	22	44	106	75	141	19	4
ACC_04950	hypothetical protein			5	2	2	1	0	0	1	1	3	5	2	2	0	0	2	2
ACC_04951	uroporphyrinogen decarboxylase-like	K01599	KOG2872	691	416	435	548	584	85	109	114	1129	456	909	1387	1354	1627	146	69
ACC_04952	eukaryotic translation initiation factor 5A-like	K03263	KOG3271	1089	765	431	641	425	423	865	1020	1297	1143	613	1155	4499	5243	9297	5647
ACC_04953	1-acyl-sn-glycerol-3-phosphate acyltransferase gan	K13523	KOG1505	1041	643	664	768	670	76	143	162	1139	872	434	1131	585	689	87	27
ACC_04954	hypothetical protein			1	0	3	3	3	0	0	1	0	0	1	0	0	0	1	0
ACC_04955	LOW QUALITY PROTEIN	K16571	KOG2065	432	316	313	445	531	37	86	108	399	337	320	433	592	755	59	20
ACC_04956	sorting nexin-2-like isoform 2		KOG2273	510	369	324	513	430	89	127	149	792	582	318	830	1112	1251	260	78

ACC_04957	hypothetical protein			9	4	6	9	8	0	0	0	3	2	3	0	0	0	0	0
ACC_04958	retinol dehydrogenase 13-like		KOG1208	130	70	90	115	87	8	20	20	98	58	88	69	379	1192	113	62
ACC_04959	probable Dol-P-Man	K03847	KOG2516	555	408	318	466	598	55	169	205	379	428	297	168	550	434	260	149
ACC_04960	transcriptional adapter 2B-like	K15127	KOG0457	3627	3512	1997	6921	3444	928	1258	1491	19084	10455	12666	32247	8772	14403	4642	6040
ACC_04961	ejaculatory bulb-specific protein 3-like			15	2	2	21	12	2	2	3	269	80	4675	4204	5	8	1	4
ACC_04962	LOW QUALITY PROTEIN	K06225	KOG1935	30	20	11	29	17	5	3	2	65	72	5	16	43	63	61	10
ACC_04963	UPF0692 protein CG33108-like			353	184	138	153	102	20	38	48	698	534	102	651	343	183	63	10
ACC_04964	general transcription factor IIH subunit 2 isoform 1	K03142	KOG2807	124	77	76	172	167	15	8	19	162	99	104	149	214	270	41	27
ACC_04965	LOW QUALITY PROTEIN		KOG1286	220	101	112	159	153	2	10	14	473	386	182	725	386	564	48	4
ACC_04966	CAS1 domain-containing protein 1-like		KOG1699	687	336	391	442	462	41	67	71	1116	405	409	355	321	465	37	15
ACC_04967	39S ribosomal protein L33, mitochondrial-like	K02913		27	28	28	77	44	3	6	6	8	9	49	42	67	51	19	12
ACC_04968	islet cell autoantigen 1-like		KOG3891	292	202	160	245	255	13	23	21	390	249	105	374	207	198	12	9
ACC_04969	6-phosphogluconolactonase-like	K01057	KOG3147	362	247	220	298	242	40	54	65	510	292	170	464	915	774	122	40
ACC_04970	mitochondrial carrier homolog 2		KOG2745	255	129	144	304	239	20	17	30	176	220	89	385	935	993	200	74
ACC_04971	transcription factor GAGA-like isoform 2		KOG4441	220	113	127	286	224	32	55	76	217	116	87	62	103	158	83	28
ACC_04972	conserved hypothetical protein			0	0	0	0	0	0	0	0	2	5	0	0	1	1	0	1
ACC_04973	carboxypeptidase M-like		KOG2649	100	20	27	64	35	1	1	7	60	15	3	109	66	82	18	6
ACC_04974	zinc finger protein 613-like		KOG2462	671	598	594	1028	775	85	198	208	389	352	357	354	649	620	652	233
ACC_04975	u2 snRNP-associated SURP motif-containing protei	K12842	KOG0151	1279	895	835	1285	998	215	374	418	1024	839	456	467	829	1082	1139	773
ACC_04976	FERM domain-containing protein 5-like	K10356	KOG0162	171	139	130	155	79	31	55	40	370	313	233	849	145	118	22	5
ACC_04977	myosin-Ie-like, partial	K10356	KOG0162	322	277	293	335	231	97	219	230	253	275	329	535	270	230	107	39
ACC_04978	dual specificity mitogen-activated protein kinase ki	K04432	KOG0984	166	94	85	229	213	7	13	21	309	186	75	426	315	394	32	6
ACC_04979	diacylglycerol kinase epsilon-like	K00901	KOG1169	237	134	130	135	126	29	26	36	392	264	79	160	165	159	29	15
ACC_04980	tRNA-splicing endonuclease subunit Sen34-like	K15323	KOG4133	340	240	245	444	529	48	121	162	219	134	209	214	216	286	312	153
ACC_04981	t-complex protein 1 subunit alpha-like isoform 1	K09493	KOG0360	675	423	393	942	813	73	132	126	1385	744	659	1504	3650	5246	459	263
ACC_04982	glutathione S-transferase-like	K01830	KOG1695	267	74	88	287	177	3	10	4	43	34	6	46	20	10	2	3
ACC_04983	mRNA export factor-like	K14298	KOG0647	145	98	98	279	224	18	26	40	246	131	167	243	288	478	59	28
ACC_04984	conserved hypothetical protein			11	7	0	4	3	0	1	4	8	4	2	0	0	2	7	0
ACC_04985	superoxide dismutase	K04565	KOG0441	91	136	115	434	281	12	16	12	463	184	346	977	96	105	18	13
ACC_04986	conserved hypothetical protein		KOG1023	1051	1054	947	1303	684	92	127	176	639	367	29	80	65	65	40	14
ACC_04987	vacuolar protein sorting-associated protein VTA1 h	K12199	KOG0917	830	589	637	851	973	92	215	190	1070	639	490	1146	911	1004	246	186
ACC_04988	conserved hypothetical protein			5	0	0	1	0	0	0	0	2	1	2	0	0	1	5	1
ACC_04989	conserved hypothetical protein		KOG3241	209	117	118	113	153	6	65	54	50	91	208	479	146	146	82	39
ACC_04990	histone deacetylase complex subunit SAP18-like	K14324	KOG3391	142	137	155	380	268	17	14	47	91	66	108	260	203	437	76	67
ACC_04991	outer dense fiber protein 3-like, partial			1	0	0	0	1	0	0	0	0	0	0	0	1	0	1	0
ACC_04992	outer dense fiber protein 3-B-like			1	3	0	0	1	0	0	0	0	0	0	0	1	0	0	0
ACC_04993	WD repeat-containing protein 16-like		KOG0266	16	13	17	16	19	1	6	6	15	34	27	38	3	5	4	1
ACC_04994	26S proteasome non-ATPase regulatory subunit 12	K03035	KOG1498	655	390	338	1027	867	101	126	192	1134	879	586	1614	1898	2249	303	117
ACC_04995	isoleucyl-tRNA synthetase, mitochondrial-like	K01870	KOG0433	567	346	270	523	596	39	56	64	702	552	286	536	1351	1034	99	28
ACC_04996	forkhead box protein K2-like	K09404	KOG3563	221	194	211	189	94	18	11	14	385	382	86	215	308	376	134	23
ACC_04997	conserved hypothetical protein			267	181	153	161	230	15	24	39	174	67	102	78	62	78	34	9
ACC_04998	hypothetical protein			0	0	0	0	0	1	0	0	1	2	0	0	0	0	0	0
ACC_04999	conserved hypothetical protein			1	3	1	2	0	0	0	0	2	1	3	2	0	0	1	0
ACC_05000	hypothetical protein			4	0	0	0	0	0	2	1	0	0	0	0	0	0	0	0
ACC_05001	glycerol-3-phosphate dehydrogenase	K00006	KOG2711	938	464	545	617	802	52	66	69	3370	1157	470	1989	2034	1495	116	37
ACC_05002	conserved hypothetical protein	K12741	KOG4205	1654	703	678	1103	701	51	124	144	1619	1573	286	840	1901	3234	636	327
ACC_05003	ras-related protein Rab-10 isoform 2	K07903	KOG0078	467	217	171	311	254	51	55	62	614	296	184	783	665	610	129	47
ACC_05004	LOW QUALITY PROTEIN	K15014	KOG1479	319	199	140	226	229	29	43	32	427	363	91	217	936	769	41	12
ACC_05005	c-terminal-binding protein isoform 1	K04496	KOG0067	617	346	344	424	192	66	95	171	453	626	94	229	305	498	683	470
ACC_05006	hcp beta-lactamase-like protein CG13865-like isoform 2		KOG4014	605	409	350	1368	775	58	51	105	1200	3146	257	4408	2444	2357	315	148
ACC_05007	UPF0609 protein CG1218-like		KOG3952	874	425	364	594	967	78	263	284	508	273	685	1013	623	869	633	427
ACC_05008	anaphase-promoting complex subunit 10-like	K03357	KOG3437	162	180	185	361	246	20	37	54	199	88	120	190	130	217	38	25
ACC_05009	conserved hypothetical protein		KOG4351	340	329	294	499	337	52	29	52	1414	1227	201	1529	909	623	117	22
ACC_05010	peroxisomal membrane protein PEX16-like	K13335	KOG4546	232	172	144	163	281	11	31	30	244	315	149	863	605	592	24	7
ACC_05011	conserved hypothetical protein		KOG4282	189	158	98	166	89	35	67	76	185	237	44	151	487	357	179	90
ACC_05012	IQ domain-containing protein H-like		KOG0759	2	3	3	21	6	0	1	1	4	3	0	0	1	3	1	1
ACC_05013	hypothetical protein			0	0	0	0	0	0	0	0	0	0	0	0	0	0	0	0

ACC_05014	cAMP-specific 3',5'-cyclic phosphodiesterase	K01120	KOG3689	1999	1484	1887	3966	696	108	213	328	2744	1653	462	299	137	132	393	311
ACC_05015	conserved hypothetical protein		KOG3538	96	44	94	423	314	29	40	39	79	49	2	12	3	13	46	5
ACC_05016	conserved hypothetical protein		KOG1492	969	539	533	803	1001	89	300	270	654	458	549	259	271	338	311	135
ACC_05017	methyltransferase-like protein 13-like		KOG2352	418	339	361	483	507	53	121	116	487	485	245	636	728	706	139	50
ACC_05018	protein canopy homolog 3		KOG4052	393	232	202	303	387	51	85	129	139	96	196	367	371	407	183	105
ACC_05019	conserved hypothetical protein		KOG4313	450	212	187	297	253	73	185	184	405	278	129	238	247	252	159	54
ACC_05020	conserved hypothetical protein		KOG4083	433	215	237	487	399	55	100	124	591	271	328	767	649	993	209	126
ACC_05021	b-cell receptor-associated protein 31-like	K14009	KOG1962	521	374	229	658	456	89	260	261	1018	902	490	1780	1108	562	227	132
ACC_05022	conserved hypothetical protein			234	128	147	191	217	4	13	7	212	151	118	50	101	83	9	3
ACC_05023	eukaryotic translation initiation factor 3 subunit B i	K03253	KOG2314	668	388	356	825	713	90	98	102	1224	1182	346	1210	3197	4285	500	200
ACC_05024	endophilin-B1-like	K11248	KOG3725	597	257	287	702	584	47	72	76	790	261	229	451	179	177	14	17
ACC_05025	conserved hypothetical protein			11	6	6	3	6	2	3	3	30	10	0	0	10	5	1	2
ACC_05026	conserved hypothetical protein			509	276	279	653	713	109	222	245	614	290	321	340	481	840	488	248
ACC_05027	solute carrier family 25 member 38-like	K15118	KOG0766	94	68	75	173	162	17	13	21	97	362	71	62	106	176	25	5
ACC_05028	vacuolar protein sorting-associated protein 18 homolog		KOG2034	398	220	257	302	341	27	43	48	294	311	139	308	269	222	26	7
ACC_05029	n-acetyllactosaminide beta-1,3-N-acetylglucosaminyltransfe		KOG3765	57	22	29	53	11	5	4	4	83	106	12	49	69	28	31	10
ACC_05030	ras-related protein Rab-3-like	K07976	KOG0093	1678	1099	805	938	269	501	644	991	483	285	140	206	16	13	84	47
ACC_05031	26S protease regulatory subunit 6A	K03065	KOG0652	710	429	371	761	587	92	114	134	1545	1105	762	2061	1694	2531	308	167
ACC_05032	testis-specific serine/threonine-protein kinase 4-lik	K08811	KOG0583	77	58	56	67	59	10	21	22	65	24	18	16	24	37	8	3
ACC_05033	uridine-cytidine kinase-like 1-like	K00876	KOG4203	288	179	172	492	325	44	70	97	440	349	303	356	493	486	106	53
ACC_05034	conserved hypothetical protein		KOG3209	50	46	22	151	43	8	12	23	191	102	55	121	444	189	120	118
ACC_05035	cell division control protein 45 homolog	K06628	KOG2475	177	116	98	232	204	12	19	23	319	171	173	126	320	600	76	25
ACC_05036	mitochondrial import inner membrane translocase subunit T		KOG3479	16	8	16	18	15	0	0	0	2	3	11	20	50	58	5	6
ACC_05037	LOW QUALITY PROTEIN	K00814	KOG0258	791	578	600	1051	755	50	81	80	1876	1559	522	1613	2208	3671	591	77
ACC_05038	brefeldin A-inhibited guanine nucleotide-exchange protein 2		KOG0929	664	386	307	442	327	47	47	64	1530	1577	275	587	743	547	99	30
ACC_05039	inactive hydroxysteroid dehydrogenase-like protein 1-like		KOG1014	49	5	6	35	17	3	9	10	179	310	48	229	887	400	66	50
ACC_05040	activating transcription factor of chaperone isoforn	K04374	KOG4571	7188	5088	4058	6518	4209	1847	4368	4376	13633	9377	4287	10100	27718	20638	22267	10894
ACC_05041	conserved hypothetical protein		KOG0161	520	265	297	438	549	29	94	74	214	189	697	566	277	174	126	61
ACC_05042	conserved hypothetical protein			81	66	80	104	83	0	7	6	31	34	26	99	72	73	12	6
ACC_05043	conserved hypothetical protein		KOG1144	1702	1361	1398	1423	924	275	605	646	1302	1136	616	294	962	1311	3121	1972
ACC_05044	major facilitator superfamily domain-containing pr	K12307	KOG2325	157	96	72	204	216	25	47	63	169	160	129	207	389	245	37	18
ACC_05045	conserved hypothetical protein	K15203		141	114	95	80	107	7	15	17	144	91	82	357	176	233	29	8
ACC_05046	multifunctional protein ADE2	K01587	KOG2835	127	94	97	215	297	14	17	11	1221	2482	145	591	7589	5911	315	122
ACC_05047	mitochondrial-processing peptidase subunit alpha-	K01412	KOG2067	1212	546	421	625	600	113	168	191	1916	989	498	953	2066	2087	185	102
ACC_05048	NADH-ubiquinone oxidoreductase 75 kDa subunit,	K03934	KOG2282	1939	1251	1458	1742	1473	189	256	313	5191	2083	1033	2555	4008	5561	549	218
ACC_05049	protein charybde-like			97	127	52	69	81	11	34	33	281	324	53	146	307	91	14	3
ACC_05050	ATP-binding cassette sub-family E member 1	K06174	KOG0063	213	161	100	244	212	29	34	38	356	341	118	397	1631	2178	208	39
ACC_05051	pontin protein isoform 1	K04499	KOG1942	388	282	261	555	454	30	46	69	374	345	266	600	1770	2025	305	203
ACC_05052	glycine dehydrogenase	K00281	KOG2040	124	71	79	146	125	22	25	40	478	1325	63	59	920	717	95	50
ACC_05053	intraflagellar transport protein 20 homolog	K16473		189	84	107	122	160	15	32	35	118	61	91	181	110	140	51	49
ACC_05054	endothelial transcription factor GATA-2-like		KOG1601	18	5	12	35	5	0	3	0	8	17	5	1	11	18	104	12
ACC_05055	nuclear RNA export factor 1-like	K14284	KOG3763	92	61	68	87	110	4	8	13	105	72	54	120	59	55	7	0
ACC_05056	conserved hypothetical protein		KOG0260	30	11	24	19	10	2	1	5	122	35	171	1022	1984	506	21	21
ACC_05057	sorting nexin-30-like		KOG2273	204	105	117	125	98	9	8	9	339	244	62	320	158	127	5	4
ACC_05058	conserved hypothetical protein	K16311	KOG0586	495	334	443	523	365	79	117	83	3090	1244	467	775	907	898	221	71
ACC_05059	general transcription factor IIF subunit 2 isoform 1	K03139	KOG2905	115	92	94	138	143	7	8	15	161	76	76	202	123	158	20	6
ACC_05060	60S ribosomal protein L30 isoform 1	K02908	KOG2988	1180	1164	544	904	559	287	551	727	438	531	822	860	2906	3373	1371	1075
ACC_05061	60S ribosome subunit biogenesis protein NIP7 hom	K07565	KOG3492	82	50	40	99	108	3	12	9	171	208	151	337	811	511	92	40
ACC_05062	retrovirus-related Pol polyprotein from transposon TNT 1-94			20	8	6	4	2	1	3	1	8	18	0	1	8	1	5	1
ACC_05063	AGAP012114-PA			3	2	3	2	0	0	1	0	2	8	0	0	1	0	0	0
ACC_05064	FYVE, RhoGEF and PH domain-containing protein 4-like isofo		KOG4424	93	47	60	126	103	5	11	12	150	91	36	92	109	129	29	5
ACC_05065	LOW QUALITY PROTEIN		KOG4256	1072	629	496	506	602	39	101	88	921	538	517	294	894	807	156	38
ACC_05066	venom carboxylesterase-6-like		KOG1516	34	40	93	219	135	8	17	20	4066	1649	81198	275410	0	0	0	0
ACC_05067	beta-1,3-galactosyltransferase 5-like		KOG2287	156	74	68	195	128	10	9	12	324	200	77	327	209	187	30	9
ACC_05068	soluble calcium-activated nucleotidase 1-like	K12304	KOG4494	126	93	106	222	222	13	22	34	186	125	117	213	232	260	21	11
ACC_05069	tetratricopeptide repeat protein 39C-like		KOG3783	277	189	161	311	216	27	32	43	296	137	104	100	2	4	1	0
ACC_05070	nuclear protein localization protein 4 homolog		KOG2834	310	241	267	600	395	29	34	38	357	172	154	440	732	983	230	43

ACC_05071	conserved hypothetical protein			24	16	15	40	18	0	4	2	4	10	2	5	13	14	14	11
ACC_05072	venom serine protease 34 precursor		KOG3627	2	1	0	0	0	1	0	2	1	1	1	6	0	0	0	0
ACC_05073	phospholipase B1, membrane-associated-like		KOG3670	22	9	5	11	21	5	6	7	53	48	3	5	13	8	1	0
ACC_05074	endocuticle structural glycoprotein SgAbd-1-like			9	1	9	13	9	1	3	0	293	17	168	525	2	5	0	0
ACC_05075	FYVE, RhoGEF and PH domain-containing protein 4-like isofo		KOG4424	124	70	66	135	102	4	14	6	164	111	34	117	145	125	25	7
ACC_05076	conserved hypothetical protein	K14570	KOG2248	1033	801	807	979	745	175	290	368	1018	653	352	351	684	677	825	377
ACC_05077	growth/differentiation factor 8-like	K04669	KOG3900	11	7	16	47	33	0	0	2	318	21	0	18	38	30	26	9
ACC_05078	hypothetical protein			0	0	0	1	0	0	0	0	0	0	0	0	0	0	0	0
ACC_05079	FoxP protein	K09409		95	76	87	116	31	40	67	96	154	40	45	30	30	52	271	390
ACC_05080	conserved hypothetical protein			6	4	4	1	5	0	1	2	1	1	2	1	0	0	2	0
ACC_05081	hypothetical protein			0	0	0	0	1	0	0	1	0	1	0	0	0	0	1	1
ACC_05082	NACHT and WD repeat domain-containing protein 1-like			5	2	1	3	3	0	2	1	1	5	0	1	1	0	1	0
ACC_05083	SNARE-associated protein Snapin-like			146	95	112	139	117	21	37	51	380	259	114	287	284	240	27	29
ACC_05084	conserved hypothetical protein		KOG3513	80	24	31	129	42	4	8	6	17	21	0	0	0	1	2	2
ACC_05085	actin related protein 1	K05692	KOG0676	6149	3535	3103	4353	1608	230	250	279	5865	5559	1548	10315	16950	12469	3285	676
ACC_05086	transmembrane protein 104 homolog		KOG3832	85	66	48	80	86	12	15	9	191	144	55	222	168	103	6	0
ACC_05087	hydroxyacylglutathione hydrolase, mitochondrial-li	K01069	KOG0813	205	168	134	377	327	16	36	36	475	272	213	546	726	844	94	20
ACC_05088	conserved hypothetical protein		KOG2504	109	86	80	146	78	26	41	59	312	201	105	61	9	13	19	11
ACC_05089	protein MCM10 homolog	K10736	KOG3056	101	85	71	80	99	11	17	21	131	71	208	66	377	543	421	170
ACC_05090	protein SERAC1-like		KOG2029	205	150	107	204	205	25	48	30	315	187	181	166	311	352	63	14
ACC_05091	dynein light chain 1, axonemal-like	K10411	KOG0531	13	9	16	88	33	0	3	2	8	4	4	5	1	0	3	0
ACC_05092	protein FAM195A-like isoform 2			162	97	67	99	125	13	11	15	124	90	39	243	189	143	19	9
ACC_05093	leucine-rich repeats and immunoglobulin-like domains prote		KOG4194	165	31	37	140	68	8	11	15	256	364	7	56	562	62	31	11
ACC_05094	glutamate			16	4	7	11	4	0	0	0	10	4	0	0	1	0	0	0
ACC_05095	importin-4-like		KOG2171	187	111	93	208	206	18	22	14	320	446	84	182	1927	2003	183	75
ACC_05096	adenylate cyclase type 2-like	K01768	KOG3619	350	242	249	423	308	44	61	60	1362	1127	228	110	715	720	199	70
ACC_05097	conserved hypothetical protein		KOG4007	393	245	281	537	577	70	74	114	532	302	378	852	664	622	77	61
ACC_05098	proteasome subunit beta type-1	K02732	KOG0179	435	323	284	692	413	84	156	166	731	423	756	1143	1815	1868	257	160
ACC_05099	abhydrolase domain-containing protein 16A-like		KOG1553	283	193	174	276	295	22	15	30	350	439	150	360	598	604	43	5
ACC_05100	major facilitator superfamily domain-containing protein 6-lik		KOG3762	241	155	135	131	163	25	22	27	535	296	254	472	389	402	20	5
ACC_05101	negative elongation factor E	K15182	KOG0148	75	61	69	123	71	11	19	24	134	61	59	95	120	179	39	27
ACC_05102	MAU2 chromatid cohesion factor homolog	K11266	KOG2300	122	95	79	164	127	13	16	22	362	432	117	306	296	276	57	14
ACC_05103	conserved hypothetical protein		KOG0161	735	588	546	689	601	215	485	415	1810	1021	342	150	315	287	526	391
ACC_05104	conserved hypothetical protein		KOG4566	257	154	121	188	188	39	53	69	419	334	151	387	507	319	134	101
ACC_05105	conserved hypothetical protein		KOG0161	766	599	520	613	852	90	223	240	452	402	351	182	412	483	590	224
ACC_05106	serine/threonine-protein phosphatase PGAM5, mit	K15637	KOG4609	172	124	108	213	179	10	7	20	183	110	100	266	309	482	44	13
ACC_05107	carboxypeptidase D-like	K07752	KOG2649	1223	834	677	930	893	85	139	162	2507	2571	559	2114	2098	1249	98	42
ACC_05108	hypothetical protein			5	1	0	2	1	2	0	0	6	5	0	0	35	6	2	11
ACC_05109	l-lactate dehydrogenase-like	K00016	KOG1495	35	55	52	120	27	17	6	72	11	44	12	17	709	995	857	828
ACC_05110	conserved hypothetical protein		KOG3598	842	427	474	728	690	155	248	199	13602	16563	762	786	4012	979	141	179
ACC_05111	aminoglycoside phosphotransferase domain-containing protein 1-like			234	186	255	667	797	14	8	17	189	113	8	41	59	80	15	7
ACC_05112	hypothetical protein			23	3	8	20	9	2	2	5	6	4	3	2	0	1	5	1
ACC_05113	N-alpha-acetyltransferase 15, NatA auxiliary subun	K00670	KOG1156	555	261	259	590	320	55	142	127	462	449	171	185	839	1551	1074	546
ACC_05114	growth arrest-specific protein 2-like		KOG2046	63	45	33	66	19	1	3	9	102	74	10	25	15	12	9	6
ACC_05115	putative fatty acyl-CoA reductase CG5065-like		KOG1221	26	14	13	14	17	2	3	8	12	31	1	3	1064	2242	108	16
ACC_05116	conserved hypothetical protein			34	62	180	71	87	29	51	63	131	111	33	69	21	29	5	0
ACC_05117	Rac1 GTPase effector FRL		KOG1923	37	31	41	63	19	4	18	14	16	25	4	3	0	0	101	44
ACC_05118	b-box type zinc finger protein ncl-1	K11997	KOG2177	48	36	40	63	10	9	3	15	35	45	1	4	14	20	42	6
ACC_05119	UPF0428 protein CXorf56 homolog isoform 2		KOG4397	275	192	194	248	193	28	78	61	225	95	148	296	253	344	189	125
ACC_05120	cyclin-dependent kinase 9	K02211	KOG0669	174	100	78	157	162	10	12	18	250	91	74	254	164	156	22	7
ACC_05121	conserved hypothetical protein		KOG4384	77	51	34	75	38	12	7	12	137	161	26	85	154	131	52	21
ACC_05122	selenoprotein K-like			47	63	48	110	148	6	13	9	62	54	113	359	167	139	9	5
ACC_05123	conserved hypothetical protein			227	129	82	214	101	27	33	60	98	77	47	50	18	17	99	92
ACC_05124	histone-lysine N-methyltransferase SETMAR-like			11	14	8	15	6	1	0	5	19	20	3	7	9	4	7	1
ACC_05125	organic cation transporter protein-like	K08202	KOG0255	409	173	150	865	542	25	35	56	109	78	147	158	87	139	81	43
ACC_05126	pre-mRNA-splicing factor RBM22-like	K12872	KOG0153	302	274	269	521	277	54	76	99	602	404	455	697	537	718	145	120
ACC_05127	transmembrane 9 superfamily member 3		KOG1277	491	282	206	418	472	42	68	82	1150	1615	227	1042	2149	1681	117	41

ACC_05128	putative malate dehydrogenase 1B-like		KOG1496	85	42	59	83	82	3	10	9	76	27	15	17	13	15	3	2
ACC_05129	chromosome transmission fidelity protein 18 homolog		KOG1968	1947	995	812	1306	1658	98	344	406	888	996	988	922	1324	1971	1120	520
ACC_05130	conserved hypothetical protein			4	0	0	0	2	0	0	0	1	1	0	0	0	0	0	0
ACC_05131	developmentally-regulated GTP-binding protein 2-l	K06944	KOG1486	141	117	77	197	187	14	21	18	224	158	145	293	369	576	56	54
ACC_05132	ras-related protein Rab-40C-like isoform 2	K07928	KOG0078	84	54	25	82	57	7	5	5	207	148	37	188	135	101	24	2
ACC_05133	conserved hypothetical protein			5	5	1	15	12	0	1	0	31	7	7	11	16	7	0	0
ACC_05134	BTB/POZ domain-containing protein KCTD5-like		KOG2715	79	45	38	55	57	7	7	13	120	72	29	76	194	173	23	2
ACC_05135	probable queuine tRNA-ribosyltransferase-like	K00773	KOG3908	214	125	144	137	161	18	48	47	444	217	167	458	703	914	83	32
ACC_05136	calcium channel flower-like isoform 1		KOG4085	193	55	54	70	55	4	3	6	138	101	14	52	63	43	10	5
ACC_05137	general transcription factor 3C polypeptide 5-like	K15202	KOG2473	193	109	106	261	236	37	79	78	219	159	181	183	346	451	165	57
ACC_05138	X-ray repair cross-complementing protein 6-like	K10884	KOG2327	475	233	222	335	385	20	81	80	361	296	235	569	618	332	96	35
ACC_05139	enoyl-CoA hydratase domain-containing protein 2,	K05607	KOG1679	97	70	57	170	173	10	12	11	297	454	74	309	2170	790	48	15
ACC_05140	n-sulphoglucosamine sulphohydrolase-like	K01565	KOG3867	272	207	248	263	281	25	60	82	603	409	201	628	965	533	40	15
ACC_05141	protein tumorous imaginal discs, mitochondrial-like	K09504	KOG0715	823	566	449	769	637	163	234	388	1549	1185	626	988	2433	3806	1773	739
ACC_05142	N-acetyllactosaminide beta-1,3-N-acetylglucosaminyltransfe		KOG1607	480	347	343	593	526	29	28	47	481	367	276	772	690	885	61	23
ACC_05143	LOW QUALITY PROTEIN	K00700	KOG0470	389	243	219	156	198	26	50	37	1248	820	190	527	2174	760	30	17
ACC_05144	Nuclear transport receptor RANBP7/RANBP8 (importin beta		KOG1991	792	520	705	413	306	74	141	153	352	265	97	69	145	224	406	182
ACC_05145	conserved hypothetical protein		KOG4441	130	113	204	164	25	5	2	7	33	47	0	1	5	4	7	1
ACC_05146	conserved hypothetical protein			1	0	0	0	0	0	0	0	0	4	3	0	0	0	0	0
ACC_05147	60S ribosomal protein L11-like	K02868	KOG0397	1009	959	518	1969	1489	331	398	613	1348	1076	1476	2085	4797	5469	1909	1622
ACC_05148	conserved hypothetical protein			211	149	144	81	61	10	6	12	39	56	30	19	441	476	35	69
ACC_05149	dorsal protein isoform B	K09255		528	514	496	478	377	242	422	293	2541	1182	546	398	811	693	278	137
ACC_05150	60S ribosomal protein L44	K02929	KOG3464	4307	2456	1603	2100	1473	1977	3796	4412	4229	2130	1818	3852	14762	15992	12622	14439
ACC_05151	transportin-3	K15436	KOG2081	298	143	104	154	128	14	21	22	425	406	90	303	454	439	43	8
ACC_05152	intraflagellar transport protein 57 homolog	K04638	KOG0972	379	241	246	212	185	44	94	71	300	138	122	230	148	164	76	33
ACC_05153	protein croquemort		KOG3776	281	186	167	218	285	10	14	22	481	545	146	634	859	361	29	1
ACC_05154	endoplasmic reticulum lectin 1-like	K14008	KOG3394	776	573	590	1164	974	115	138	216	1332	858	672	1220	1452	1743	162	46
ACC_05155	spermatogenesis-associated protein 5-like	K14575	KOG0730	207	174	162	193	217	18	72	82	187	177	116	139	429	596	111	112
ACC_05156	DNA replication complex GINS protein PSF1-like	K10732	KOG3303	40	37	24	43	67	2	4	8	9	8	14	25	46	145	19	11
ACC_05157	conserved hypothetical protein	K16458	KOG2978	818	620	598	1365	812	119	158	215	555	313	277	396	751	685	187	187
ACC_05158	vacuolar protein sorting-associated protein 16 homolog		KOG2280	651	465	487	526	489	86	174	160	932	652	622	570	608	694	80	32
ACC_05159	tyrosine-protein kinase Fps85D-like isoform 1		KOG0194	307	213	173	195	152	31	38	45	644	533	152	371	474	419	47	28
ACC_05160	probable phospholipid-transporting ATPase IIB-like	K01530	KOG0210	1234	800	812	902	807	73	83	97	2080	1628	484	1316	888	639	48	17
ACC_05161	syntaxin-16	K08489	KOG0809	166	100	112	147	108	17	17	46	165	108	61	124	125	124	40	8
ACC_05162	e3 ubiquitin-protein ligase TRIM9	K10649	KOG4367	95	46	47	126	29	1	4	8	40	17	7	6	7	16	73	15
ACC_05163	tubulin beta-1 chain-like	K07375	KOG1375	189	45	49	118	32	7	6	5	60	59	44	81	67	16	34	13
ACC_05164	protein-L-isoaspartate(D-aspartate) O-methyltrans	K00573	KOG1661	736	545	662	1778	1315	77	68	105	1068	507	670	811	316	408	41	25
ACC_05165	conserved hypothetical protein			280	212	214	393	377	21	30	36	488	341	170	354	246	208	32	5
ACC_05166	engulfment and cell motility protein 1	K12366	KOG2999	506	365	362	540	491	31	39	64	588	420	178	610	466	440	37	8
ACC_05167	RNA polymerase II-associated factor 1 homolog iso	K15174	KOG2478	593	399	476	581	375	90	233	266	375	233	215	250	277	411	778	952
ACC_05168	CCAAT/enhancer-binding protein gamma-like	K10049	KOG3119	114	78	101	155	138	13	16	31	94	79	50	172	76	54	23	11
ACC_05169	NF-kappa-B inhibitor-interacting Ras-like protein		KOG3883	148	54	41	93	103	6	4	7	135	42	71	175	54	51	14	5
ACC_05170	microtubule-actin cross-linking factor 1-like		KOG0162	1371	811	1186	2009	446	258	380	414	739	704	670	125	36	100	1779	1551
ACC_05171	f-box only protein 22-like			302	195	213	249	232	40	42	78	285	224	123	303	350	407	78	24
ACC_05172	v-type proton ATPase subunit G	K02152	KOG1772	1613	523	548	1886	917	141	169	178	1269	655	869	2006	1158	1178	739	337
ACC_05173	histone H2A type 1	K11251	KOG1756	446	241	202	1488	665	107	119	202	397	174	257	423	1125	2453	1123	795
ACC_05174	conserved hypothetical protein			574	423	345	471	527	74	83	115	820	680	284	1319	981	810	95	38
ACC_05175	protein fuzzy homolog			140	115	94	171	231	8	16	15	164	125	72	199	236	268	27	13
ACC_05176	conserved hypothetical protein	K16726	KOG4369	10154	7586	6896	9248	5258	1834	3219	3869	7415	6441	2989	1533	2648	4014	11130	6702
ACC_05177	mitochondrial import inner membrane translocase subunit T		KOG3324	295	199	191	519	331	31	53	69	452	242	280	471	769	1117	179	140
ACC_05178	BRISC and BRCA1-A complex member 1-like			183	135	158	324	326	14	18	28	132	132	135	269	144	185	25	6
ACC_05179	transcription elongation factor B polypeptide 1 isof	K03872	KOG3473	253	203	179	506	442	12	20	28	350	194	160	426	323	298	49	18
ACC_05180	probable splicing factor, arginine/serine-rich 7-like	K13165	KOG4676	165	125	111	331	209	33	40	76	209	174	184	236	603	753	356	222
ACC_05181	tumor susceptibility gene 101 protein	K12183	KOG2391	271	154	152	288	274	26	36	62	364	277	177	584	443	378	113	51
ACC_05182	sex determination protein fruitless-like		KOG4441	231	105	105	199	209	25	72	66	205	149	200	337	216	191	80	63
ACC_05183	alpha-1,3-mannosyl-glycoprotein 4-beta-N-acetylgl	K00738		353	294	282	311	327	30	59	47	397	357	176	247	249	252	56	15
ACC_05184	LOW QUALITY PROTEIN		KOG1162	1460	941	898	1001	666	73	110	105	1486	1091	222	386	340	364	53	11

ACC_05185	e3 ubiquitin-protein ligase NRDP1	K11981	KOG0297	51	26	13	41	33	3	4	1	103	79	18	91	155	110	12	8
ACC_05186	suppressor of cytokine signaling 7	K04700	KOG4637	327	166	233	292	171	32	56	65	472	320	125	118	190	161	45	26
ACC_05187	LOW QUALITY PROTEIN	K02903	KOG3412	806	638	419	1247	1112	112	173	229	676	616	872	1064	3495	3898	863	363
ACC_05188	RNA polymerase I-specific transcription initiation fa	K15216	KOG2434	184	148	108	215	229	12	24	36	171	208	133	168	1069	1311	122	42
ACC_05189	conserved hypothetical protein	K08596		337	234	246	383	140	24	65	78	477	476	83	237	659	737	160	76
ACC_05190	sphingomyelin phosphodiesterase-like	K12350	KOG3770	603	179	257	965	627	9	17	29	673	2041	60	940	361	289	36	20
ACC_05191	conserved hypothetical protein			192	166	147	271	348	5	19	31	168	143	119	300	390	430	30	21
ACC_05192	proteasome assembly chaperone 2-like		KOG3112	107	65	69	111	173	1	6	12	179	144	190	429	369	377	15	5
ACC_05193	probable histone-binding protein Caf1	K10752	KOG0264	228	113	114	221	175	15	25	38	390	260	125	546	805	1215	180	47
ACC_05194	conserved hypothetical protein			102	99	136	284	218	14	54	81	124	74	403	262	192	158	55	13
ACC_05195	transmembrane protein 234 homolog		KOG4831	38	32	54	49	75	0	4	6	18	20	24	89	24	24	3	5
ACC_05196	hypothetical protein			3	1	1	2	1	0	1	1	5	0	0	0	0	2	8	4
ACC_05197	conserved hypothetical protein			6	4	3	1	2	1	0	1	2	1	0	0	0	1	1	0
ACC_05198	bone morphogenetic protein receptor type-1B	K13579	KOG2052	274	145	172	306	235	36	42	62	580	450	94	264	209	218	60	16
ACC_05199	transcription factor HNF-4 homolog	K07292	KOG4215	76	39	28	62	45	5	7	9	233	233	35	238	332	163	41	23
ACC_05200	transcription factor HNF-4 homolog		KOG4215	109	55	56	200	110	11	23	20	217	164	70	194	337	147	69	19
ACC_05201	tyrosine-protein kinase transmembrane receptor R	K05122	KOG1026	63	38	27	33	29	7	3	3	90	95	5	6	49	73	24	7
ACC_05202	PEST proteolytic signal-containing nuclear protein-like			151	94	80	203	105	17	27	26	307	86	127	217	296	490	132	94
ACC_05203	LOW QUALITY PROTEIN	K10908	KOG1038	487	258	259	467	590	48	88	65	451	446	353	262	917	1003	224	102
ACC_05204	conserved hypothetical protein		KOG4161	1465	1064	926	1288	1233	258	541	561	2075	1241	780	653	884	1001	730	326
ACC_05205	conserved hypothetical protein	K09326	KOG2251	26	21	33	48	9	9	9	12	118	65	0	8	3	18	36	13
ACC_05206	conserved hypothetical protein			120	62	50	64	116	6	28	16	86	86	67	105	183	157	48	21
ACC_05207	conserved hypothetical protein			34	23	24	29	8	6	4	8	70	72	16	38	46	43	87	45
ACC_05208	conserved hypothetical protein			98	35	24	86	28	9	9	9	78	52	29	69	16	95	35	19
ACC_05209	reticulon-4-interacting protein 1 homolog, mitochondrial-lik		KOG1198	212	119	149	218	204	24	34	52	331	215	153	248	316	472	58	13
ACC_05210	vesicular inhibitory amino acid transporter-like	K15015	KOG4303	556	162	225	416	192	16	11	17	159	57	27	4	8	22	14	8
ACC_05211	microprocessor complex subunit DGCR8-like		KOG4334	142	83	105	181	157	23	67	61	88	55	37	25	67	87	144	68
ACC_05212	peroxidase		KOG2408	10	2	8	7	6	1	1	8	49	68	47	11	1734	127	11	7
ACC_05213	conserved hypothetical protein			302	232	225	417	344	30	65	67	247	244	209	315	350	500	130	46
ACC_05214	conserved hypothetical protein		KOG0579	70	36	51	53	53	2	9	10	115	49	148	491	166	162	41	26
ACC_05215	CDK5 regulatory subunit-associated protein 3-like		KOG2607	619	541	356	570	570	130	237	258	818	736	462	1078	1656	1685	225	93
ACC_05216	RNA polymerase II subunit A C-terminal domain ph	K15544	KOG2424	83	58	57	81	100	6	6	8	114	104	50	192	254	190	19	8
ACC_05217	zinc finger protein DPF3-like	K13196	KOG1244	500	353	301	321	169	38	66	62	582	603	157	252	500	586	414	252
ACC_05218	conserved hypothetical protein		KOG3119	816	801	851	1573	842	181	203	240	2894	1695	1139	2069	2061	2052	904	331
ACC_05219	ras-like protein family member 11B-like		KOG0395	85	156	165	879	243	10	18	40	99	54	17	9	2	2	1	2
ACC_05220	BTB/POZ domain-containing protein 7	K10479	KOG2838	77	62	52	101	65	5	10	7	160	156	70	68	260	87	21	15
ACC_05221	conserved hypothetical protein			2	0	0	2	0	0	0	2	0	2	0	0	0	0	1	0
ACC_05222	39S ribosomal protein L53, mitochondrial-like			372	248	237	323	258	15	17	26	265	204	58	457	536	474	90	20
ACC_05223	conserved hypothetical protein		KOG3598	187	145	158	167	56	19	40	35	389	378	53	82	124	91	183	87
ACC_05224	aprataxin-like	K10863	KOG0562	251	287	338	505	445	39	40	53	275	166	179	470	207	148	38	16
ACC_05225	hypothetical protein			5	2	6	4	4	2	3	0	3	8	3	1	0	2	2	1
ACC_05226	zinc finger protein 830-like		KOG3032	368	298	338	440	378	108	259	260	384	179	305	279	376	458	458	409
ACC_05227	tRNA-specific adenosine deaminase 1-like	K15440	KOG1577	312	210	169	448	475	61	98	121	544	334	291	511	450	445	147	39
ACC_05228	UDP-N-acetylglucosamine transferase subunit ALG	K07432	KOG3349	194	92	122	105	168	13	20	33	117	102	117	301	312	277	33	20
ACC_05229	multiple inositol polyphosphate phosphatase 1-like		KOG1382	11	8	10	77	56	0	0	7	25	31	71	126	13	7	6	3
ACC_05230	pancreatic triacylglycerol lipase-like	K14073		38	31	31	101	69	5	16	13	251	245	94	165	264	152	50	13
ACC_05231	hypothetical protein			0	0	0	0	0	0	0	0	0	0	0	0	0	0	0	0
ACC_05232	conserved hypothetical protein		KOG2462	2351	1431	1155	1540	1117	342	663	684	994	777	396	144	451	667	2011	760
ACC_05233	tektin-3-like		KOG2685	260	182	183	353	298	30	61	66	246	144	218	366	109	144	28	17
ACC_05234	conserved hypothetical protein		KOG3778	427	340	288	490	364	47	49	68	520	376	156	311	387	461	71	19
ACC_05235	adenosine kinase 1-like isoform 1	K00856	KOG2854	1665	986	899	2361	1246	300	365	621	2922	2269	916	1915	2624	3413	1714	1475
ACC_05236	conserved hypothetical protein		KOG1244	351	351	293	306	97	75	81	119	826	527	128	87	89	146	222	135
ACC_05237	protein aveugle		KOG3678	23	34	22	39	42	3	5	3	29	9	25	60	46	70	6	3
ACC_05238	conserved hypothetical protein		KOG0921	374	29	10	35	13	4	8	13	236	17	696	6000	80	56	17	17
ACC_05239	conserved hypothetical protein		KOG3598	88	68	29	38	15	10	14	14	223	117	36	1187	64	196	10	6
ACC_05240	hypothetical protein			15	5	4	2	3	1	3	2	8	26	1	3	1	0	4	0
ACC_05241	NADH dehydrogenase	K03943	KOG3196	653	365	303	1003	826	60	56	86	1043	503	581	1782	972	1362	96	50

ACC_05242	conserved hypothetical protein			17	7	2	20	14	2	1	3	4	4	39	23	0	0	0	0
ACC_05243	odorant receptor Or1-like			9	0	3	5	3	0	1	2	6	0	18	16	1	0	1	0
ACC_05244	odorant receptor Or1-like			3	0	0	6	5	0	0	0	0	1	21	13	0	0	0	0
ACC_05245	conserved hypothetical protein			5	7	1	3	3	1	1	0	0	0	7	5	0	0	0	0
ACC_05246	odorant receptor 50			9	16	12	12	5	2	1	7	24	12	24	23	0	0	0	0
ACC_05247	ras-related protein Rap-2c		KOG0395	57	45	37	69	40	3	8	4	185	168	22	108	79	63	21	0
ACC_05248	DDB1- and CUL4-associated factor 7-like	K11805	KOG0290	192	82	80	195	172	25	28	24	113	102	79	174	69	65	20	16
ACC_05249	LOW QUALITY PROTEIN		KOG0061	139	95	66	185	128	80	88	110	767	435	214	523	45	37	13	3
ACC_05250	conserved hypothetical protein		KOG4407	1586	1011	956	1182	655	218	281	335	3324	2026	494	292	711	920	555	271
ACC_05251	diamine acetyltransferase 2-like	K00657	KOG3216	185	196	145	155	215	13	43	51	260	136	165	270	487	747	72	51
ACC_05252	serine/arginine-rich splicing factor 1-like	K12890	KOG0105	100	102	88	103	86	3	12	18	180	213	41	65	318	429	76	64
ACC_05253	elongation factor Tu, mitochondrial-like	K02358	KOG0460	1851	1151	1179	1764	1433	252	306	406	3293	1867	1076	1895	5657	7631	1402	896
ACC_05254	39S ribosomal protein L21, mitochondrial-like	K02888	KOG1686	194	152	156	286	287	30	57	85	331	171	162	356	637	969	336	274
ACC_05255	CD9 antigen		KOG3882	230	105	71	143	89	10	4	14	507	299	35	192	171	163	44	11
ACC_05256	cyclin-dependent kinase 5	K02090	KOG0662	160	127	142	376	179	16	31	39	184	100	110	124	120	123	75	32
ACC_05257	Monocarboxylate transporter 7		KOG2504	52	63	74	74	72	10	28	12	97	44	72	30	661	751	81	4
ACC_05258	probable aspartate aminotransferase, cytoplasmic-	K14454	KOG1412	645	380	355	603	922	41	49	58	2148	2844	363	3576	4924	3160	131	42
ACC_05259	transcription elongation factor SPT6-like	K11292	KOG1856	1509	1228	1080	1196	971	396	667	698	2286	1803	731	557	1139	1082	1226	1333
ACC_05260	n-alpha-acetyltransferase 38, NatC auxiliary subuni	K12627	KOG1784	83	82	71	61	106	4	10	10	96	73	67	173	237	283	22	12
ACC_05261	conserved hypothetical protein		KOG1978	139	117	78	190	229	12	30	42	120	90	141	144	225	284	70	26
ACC_05262	LOW QUALITY PROTEIN	K03097	KOG0668	426	242	190	464	238	110	203	236	553	612	196	409	903	1172	1206	799
ACC_05263	ubiquitin-like modifier-activating enzyme 5-like	K12164	KOG2336	506	430	387	455	425	113	190	206	507	394	313	787	906	788	84	57
ACC_05264	conserved hypothetical protein			175	126	166	223	277	8	10	18	114	44	171	96	65	75	16	1
ACC_05265	phosphate carrier protein, mitochondrial-like isofo	K15102	KOG0767	2768	834	872	2269	1671	186	123	207	6462	1692	859	4791	4253	6334	917	162
ACC_05266	conserved hypothetical protein		KOG0921	2396	473	487	1410	440	146	202	222	38138	27775	779	1716	61	23	29	86
ACC_05267	serrate RNA effector molecule homolog		KOG2295	1282	915	914	1395	958	337	616	579	1942	1048	868	543	1378	2432	2989	2537
ACC_05268	U6 snRNA-associated Sm-like protein LSm7 isoform	K12626	KOG1781	136	115	118	247	180	34	71	72	172	61	186	152	298	308	178	103
ACC_05269	peptidyl-prolyl cis-trans isomerase-like 4-like	K12735	KOG0415	1115	886	765	955	886	223	500	631	554	384	483	352	648	938	1557	784
ACC_05270	bifunctional polynucleotide phosphatase/kinase-lik	K08073	KOG2134	635	386	376	731	751	71	172	163	629	375	395	657	447	476	237	146
ACC_05271	conserved hypothetical protein		KOG3930	157	142	114	172	144	21	45	47	209	156	126	248	307	296	81	31
ACC_05272	oligopeptidase A-like	K01414	KOG2089	904	514	565	956	967	80	140	158	1445	920	492	1118	1811	2154	251	75
ACC_05273	transmembrane protein 184B-like isoform 1		KOG2641	606	329	366	542	429	84	89	93	959	528	312	663	611	535	92	17
ACC_05274	RCC1 and BTB domain-containing protein 1-like	K11494	KOG1427	359	260	271	359	317	64	75	111	413	247	222	230	203	184	37	15
ACC_05275	fumarylacetoacetate hydrolase domain-containing protein 2		KOG1535	222	157	158	502	263	35	65	78	1541	2244	204	675	2414	2351	528	138
ACC_05276	epidermal retinol dehydrogenase 2-like		KOG1201	143	102	121	196	238	13	25	29	252	97	180	224	242	294	31	11
ACC_05277	ketohexokinase-like	K00846	KOG2947	105	82	90	205	260	14	12	23	264	216	129	423	1255	281	30	5
ACC_05278	cleft lip and palate transmembrane protein 1-like protein-lik		KOG2489	301	154	141	235	259	27	88	97	335	345	171	415	608	490	72	41
ACC_05279	RAC serine/threonine-protein kinase	K04456	KOG0690	233	116	129	373	318	41	27	51	698	557	78	435	355	319	60	29
ACC_05280	ubiquitin-like domain-containing CTD phosphatase	K01090	KOG1605	359	249	277	460	446	56	85	120	610	430	272	575	574	697	195	74
ACC_05281	conserved hypothetical protein		KOG1890	1159	645	705	830	565	103	148	167	1086	667	386	518	202	219	51	17
ACC_05282	proteasome subunit beta type-2-like isoform 1	K02734	KOG0177	257	163	163	432	449	31	58	56	766	334	566	1025	1447	1617	106	44
ACC_05283	conserved hypothetical protein	K10740		140	83	74	173	198	4	9	13	339	180	195	691	511	490	40	11
ACC_05284	solute carrier family 25 member 35-like, partial	K11341	KOG0755	222	158	131	341	277	22	36	57	405	235	350	1359	717	587	75	29
ACC_05285	conserved hypothetical protein			36	38	92	212	103	1	4	2	7	3	0	0	6	4	2	0
ACC_05286	LOW QUALITY PROTEIN		KOG2220	1402	1032	980	1024	781	155	311	316	1774	1257	686	438	814	668	291	114
ACC_05287	nuclear pore complex protein Nup205	K14310	KOG1835	680	443	421	794	713	25	51	63	879	684	197	285	1081	1544	160	38
ACC_05288	NF-X1-type zinc finger protein NFXL1-like	K15683	KOG1952	182	206	253	322	296	24	39	42	436	225	364	170	205	209	48	24
ACC_05289	pre-mRNA-processing factor 6-like	K12855	KOG0495	371	200	176	440	303	58	82	107	300	290	156	229	462	508	188	173
ACC_05290	conserved hypothetical protein		KOG4794	286	135	156	551	273	29	44	57	383	321	167	539	375	490	304	130
ACC_05291	rab GTPase-binding effector protein 1-like		KOG0993	529	346	375	855	706	83	183	229	606	408	449	317	536	554	549	350
ACC_05292	soluble NSF attachment protein	K15296	KOG1586	1130	348	370	438	476	49	34	70	1395	728	280	1299	436	382	23	15
ACC_05293	lysyl oxidase homolog 4	K00280		15	29	31	101	41	4	0	4	103	39	38	191	0	0	0	2
ACC_05294	cGMP-specific 3',5'-cyclic phosphodiesterase-like	K13763	KOG3689	539	387	414	1396	416	57	69	139	424	328	41	12	20	28	46	26
ACC_05295	WD repeat-containing protein 34-like		KOG1587	217	153	131	226	222	19	37	30	290	184	137	232	267	346	50	17
ACC_05296	conserved hypothetical protein			89	65	79	95	41	11	8	16	258	201	54	191	142	86	45	14
ACC_05297	conserved hypothetical protein		KOG4628	1568	1533	1323	677	195	148	171	250	1125	787	184	106	116	142	347	198
ACC_05298	26S proteasome non-ATPase regulatory subunit 8-l	K03031	KOG3151	423	336	391	1148	1071	39	76	118	660	343	718	1667	1453	1787	174	113

ACC_05299	actin-related protein 10-like	K16576	KOG0676	175	90	68	303	312	9	15	24	193	155	143	246	672	852	43	16
ACC_05300	DENN domain-containing protein 1A-like isoform 1		KOG3569	717	449	466	743	578	76	134	164	764	675	278	551	560	591	218	129
ACC_05301	nuclear pore complex protein Nup133	K14300	KOG4121	360	273	227	286	343	26	53	52	606	569	241	339	832	1117	103	30
ACC_05302	conserved hypothetical protein			61	29	32	56	58	3	6	6	162	38	73	15	3490	4069	65	16
ACC_05303	conserved hypothetical protein		KOG4797	539	541	584	527	254	35	38	58	836	658	179	458	791	725	258	59
ACC_05304	o-phosphoseryl-tRNA(Sec) selenium transferase-lik	K03341	KOG3843	332	150	124	138	129	4	3	5	183	88	21	50	16	32	4	0
ACC_05305	mannosyl-oligosaccharide glucosidase-like isoform	K01228	KOG2161	571	302	278	261	269	54	74	89	500	654	243	462	1323	1076	163	91
ACC_05306	LOW QUALITY PROTEIN	K03348	KOG1858	1703	1194	1008	1472	1288	105	164	190	1468	1383	838	1206	1471	1395	161	66
ACC_05307	pre-mRNA-splicing factor Slu7-like	K12819	KOG2560	754	474	551	881	683	224	426	490	999	520	550	568	544	627	487	327
ACC_05308	e3 ubiquitin-protein ligase RFWD2-like	K10143	KOG0264	742	452	419	356	270	49	89	83	1384	1019	172	536	1186	811	150	100
ACC_05309	conserved hypothetical protein	K16678		212	121	141	497	445	19	35	39	936	586	127	262	208	157	83	44
ACC_05310	neurogenic protein big brain-like		KOG0223	164	93	100	174	97	15	22	19	136	87	20	77	16	21	33	23
ACC_05311	regulator of nonsense transcripts 1	K14326	KOG1802	978	764	796	1628	698	210	323	509	2470	1353	617	833	1526	1932	913	407
ACC_05312	syntaxin-18	K08492	KOG3894	448	286	280	555	534	64	147	153	450	309	344	407	378	437	134	65
ACC_05313	conserved hypothetical protein			6	13	22	19	25	1	2	3	7	5	24	36	7	6	0	0
ACC_05314	dehydrogenase/reductase SDR family member 7-like		KOG1205	182	177	106	177	282	110	221	249	4567	847	112	153	513	488	41	17
ACC_05315	NF-kappa-B-repressing factor-like isoform 2		KOG4368	498	435	562	492	571	42	93	68	657	348	238	583	475	564	86	29
ACC_05316	coiled-coil domain-containing protein 132-like		KOG2939	390	257	189	374	332	34	61	71	573	399	236	520	438	504	100	26
ACC_05317	rho-related BTB domain-containing protein 2-like	K07868	KOG0393	315	161	189	382	184	27	39	56	275	164	134	133	10	7	13	9
ACC_05318	RING finger protein 37-like	K10600	KOG2042	107	59	49	87	99	3	14	16	134	63	49	148	139	174	27	14
ACC_05319	conserved oligomeric Golgi complex subunit 2-like		KOG2307	421	300	286	473	590	26	73	59	340	304	243	370	539	412	66	10
ACC_05320	putative tRNA pseudouridine synthase Pus10-like	K07583	KOG2364	291	159	185	329	366	27	64	55	369	209	181	401	365	424	89	25
ACC_05321	calcineurin subunit B type 2-like	K06268	KOG0034	521	257	241	252	416	25	60	47	577	381	142	674	331	206	27	29
ACC_05322	conserved hypothetical protein	K15425	KOG3175	382	164	156	232	219	26	82	86	445	352	141	316	595	558	232	100
ACC_05323	LOW QUALITY PROTEIN	K10597	KOG2042	536	372	345	454	319	57	120	149	1004	892	209	726	814	725	227	127
ACC_05324	LOW QUALITY PROTEIN			607	393	342	578	679	43	57	70	956	703	354	705	497	373	39	7
ACC_05325	nuclear receptor-binding protein homolog	K08875	KOG1266	230	133	105	321	203	44	80	110	227	164	91	117	148	112	102	68
ACC_05326	lethal(3)malignant brain tumor-like protein 3-like		KOG3766	509	349	264	511	330	73	93	125	437	317	220	222	398	416	197	101
ACC_05327	fringe glycosyltransferase-like isoform 1	K05948	KOG2246	64	19	39	79	30	3	4	5	61	51	15	53	30	17	18	5
ACC_05328	hypothetical protein			107	38	55	112	63	10	10	14	36	17	28	5	1	2	1	1
ACC_05329	transcription initiation factor TFIID subunit 5	K03130	KOG0263	136	52	75	188	121	22	36	37	80	61	40	65	205	437	147	73
ACC_05330	hypothetical protein			35	18	18	48	26	8	13	15	35	37	1	3	5	12	6	4
ACC_05331	tRNA-dihydrouridine synthase 2-like	K05543	KOG2334	208	168	116	199	198	53	98	134	231	152	179	235	458	401	160	75
ACC_05332	DNA excision repair protein haywire-like isoform 1	K10843	KOG1123	422	262	279	442	394	36	62	64	513	427	204	395	338	388	91	34
ACC_05333	ell-associated factor Eaf-like isoform 1	K15186	KOG4795	654	482	370	622	558	78	72	93	964	703	369	839	1049	1145	93	28
ACC_05334	alanine--glyoxylate aminotransferase 2, mitochond	K00827	KOG1404	13	16	22	26	22	3	2	7	21	23	9	10	14	33	5	4
ACC_05335	VWFA and cache domain-containing protein 1-like		KOG2353	141	126	104	142	146	9	13	17	203	176	67	121	278	185	32	7
ACC_05336	nuclear cap-binding protein subunit 1 isoform 1	K12882	KOG1104	438	311	280	517	437	51	105	159	476	412	216	306	655	735	170	79
ACC_05337	protein pellino-like	K11964	KOG3842	401	303	320	840	521	64	76	122	681	388	264	387	422	589	209	65
ACC_05338	exosome complex exonuclease RRP44-like	K12585	KOG2102	274	189	175	387	406	23	40	29	342	218	171	237	467	767	96	35
ACC_05339	eukaryotic peptide chain release factor GTP-bindin	K03267	KOG0459	999	668	532	770	483	199	342	372	1487	1397	425	1166	2369	3148	1730	1342
ACC_05340	NCK-interacting protein with SH3 domain-like		KOG4035	821	455	600	678	452	28	36	42	719	521	125	263	510	502	89	33
ACC_05341	conserved hypothetical protein		KOG0161	75	82	100	100	149	4	20	21	25	28	7	9	24	19	2	4
ACC_05342	conserved hypothetical protein			31	27	48	36	60	3	5	7	22	14	15	21	7	12	4	2
ACC_05343	conserved hypothetical protein		KOG3598	1612	1303	1231	1369	746	251	439	473	1738	1374	392	270	1031	961	1089	706
ACC_05344	conserved hypothetical protein			71	65	50	134	72	14	30	37	153	54	105	128	58	66	18	14
ACC_05345	RUN domain-containing protein 1-like		KOG3759	233	110	133	176	149	10	29	20	212	142	51	123	113	121	14	7
ACC_05346	conserved hypothetical protein			25	4	13	9	3	2	1	5	5	10	3	4	29	27	11	7
ACC_05347	epidermal retinol dehydrogenase 2-like isoform 1	K15734	KOG1201	295	107	82	326	229	26	25	30	2580	1351	354	1148	1403	759	76	38
ACC_05348	epidermal retinol dehydrogenase 2-like		KOG0557	2	2	2	2	1	0	2	1	33	7	61	54	26	12	0	1
ACC_05349	integrin beta-nu-like		KOG1226	11	5	4	15	8	4	9	2	148	30	2	0	557	692	110	7
ACC_05350	Dihydrolipoyllysine-residue acetyltransferase comp	K00627	KOG0557	392	175	175	126	99	28	30	32	936	485	65	408	2150	1731	201	68
ACC_05351	u5 small nuclear ribonucleoprotein 40 kDa protein-	K12857	KOG0265	175	81	103	248	253	13	21	29	305	175	127	350	434	718	52	21
ACC_05352	serine/threonine-protein kinase RIO3-like		KOG2269	405	369	381	417	343	57	98	113	457	203	262	495	445	470	94	63
ACC_05353	conserved hypothetical protein			46	51	73	90	86	0	2	2	7	16	36	48	42	67	11	5
ACC_05354	pfs, nacht and ankyrin domain protein		KOG4177	3	0	1	2	1	0	0	0	1	2	2	2	2	4	1	0
ACC_05355	mediator of RNA polymerase II transcription subun	K15137	KOG4043	308	138	155	372	246	85	139	222	253	138	186	333	323	439	731	421

ACC_05356	coiled-coil-helix-coiled-coil-helix domain-containing protein		KOG4090	2703	1695	1752	8225	2995	383	417	838	4392	1479	4134	8138	6215	6545	4510	3480
ACC_05357	hexamerin 110		KOG4274	453	646	128	182	58	534	687	575	832	4440	8	20	183801	5826	330	2798
ACC_05358	conserved hypothetical protein		KOG1311	193	152	131	129	56	21	7	9	255	329	62	96	229	299	137	24
ACC_05359	conserved hypothetical protein	K14325	KOG4209	731	454	395	1102	519	164	303	347	363	388	441	447	1175	1995	4730	3966
ACC_05360	angio-associated migratory cell protein-like		KOG0296	232	168	180	259	283	24	51	55	365	280	166	324	560	540	53	28
ACC_05361	katanin p80 WD40-containing subunit B1		KOG0267	244	182	142	251	135	33	49	55	768	575	90	150	221	216	165	62
ACC_05362	adenosylhomocysteinase-like	K01251	KOG1370	242	152	102	360	212	45	45	50	310	429	150	320	2179	3582	701	385
ACC_05363	repressor of RNA polymerase III transcription MAF1 homolo		KOG3104	271	136	118	269	236	26	29	30	477	318	117	335	125	92	21	11
ACC_05364	SHC SH2 domain-binding protein 1 homolog B-like			84	71	54	100	115	13	22	25	121	101	77	97	130	204	31	15
ACC_05365	conserved hypothetical protein			4	0	4	2	1	0	0	0	1	0	3	3	3	0	0	1
ACC_05366	conserved hypothetical protein			5	13	7	1	2	0	2	2	4	35	0	0	0	3	4	2
ACC_05367	hypothetical protein			0	0	0	0	1	0	0	0	0	0	0	0	0	0	1	0
ACC_05368	conserved hypothetical protein			4	3	1	1	2	0	0	0	3	7	0	1	2	0	0	0
ACC_05369	ARS-binding factor 2, mitochondrial-like	K11830	KOG4715	617	371	367	647	870	44	189	194	227	138	357	416	510	745	402	211
ACC_05370	conserved hypothetical protein		KOG3077	96	48	45	107	103	1	4	6	189	93	19	147	119	89	11	1
ACC_05371	conserved hypothetical protein		KOG1924	4947	4245	4629	4755	3302	406	901	1102	1685	923	811	172	39	99	282	79
ACC_05372	peptidyl-prolyl cis-trans isomerase-like	K09565	KOG0111	23	35	27	44	48	1	4	8	25	28	10	30	124	121	9	4
ACC_05373	conserved hypothetical protein			768	134	78	347	222	223	740	580	170	299	163	39	31	55	7919	7903
ACC_05374	ATP-dependent RNA helicase p62-like	K12823	KOG0331	2444	1377	1106	2571	2193	162	198	245	4907	2926	1365	4328	3924	5642	662	211
ACC_05375	mitochondrial thiamine pyrophosphate carrier-like	K15108	KOG0752	85	65	96	87	83	3	11	11	372	87	89	268	224	222	13	4
ACC_05376	LOW QUALITY PROTEIN	K15001	KOG0157	107	70	22	227	214	13	19	15	274	263	16	54	70	47	5	3
ACC_05377	cytosolic non-specific dipeptidase-like isoform 1	K08660	KOG2276	1062	1004	555	605	720	143	190	197	23200	4686	421	2138	2821	3758	148	67
ACC_05378	ubiquitin carboxyl-terminal hydrolase	K05609	KOG1415	116	114	111	194	275	15	21	30	358	156	179	535	607	682	53	14
ACC_05379	hypothetical protein			5	14	7	3	3	1	4	2	7	6	0	2	3	2	3	0
ACC_05380	conserved hypothetical protein		KOG2389	4689	3876	4315	4088	3035	860	1780	1813	3040	1787	1513	748	1849	3213	5264	2673
ACC_05381	Larval cuticle protein A2B			160	128	159	209	160	9	19	27	167	146	132	243	171	770	22	51
ACC_05382	conserved hypothetical protein		KOG3598	8	3	3	9	2	5	0	1	31	12	4	10	7330	4389	407	2774
ACC_05383	Larval cuticle protein A3A			43	16	27	15	6	0	1	1	49	8	2	304	3324	3796	438	5788
ACC_05384	MLL1/MLL complex subunit KIAA1267-like			763	526	476	913	643	151	281	287	1118	685	445	265	633	976	984	533
ACC_05385	hypothetical protein			0	0	0	0	0	0	0	1	0	0	0	0	0	0	0	0
ACC_05386	GTP-binding protein REM 2-like		KOG0395	37	20	19	40	11	4	1	4	217	29	0	1	8	11	16	3
ACC_05387	syntaxin-12-like	K08488	KOG0811	395	271	301	412	358	32	56	49	388	199	148	513	226	176	22	7
ACC_05388	ubiquitin carboxyl-terminal hydrolase 47-like isofor	K11857	KOG4598	258	159	129	246	199	39	67	92	539	789	76	225	490	474	185	81
ACC_05389	DNA-binding protein D-ETS-3-like	K09436	KOG3806	82	25	35	44	11	1	0	3	27	31	3	15	0	1	2	1
ACC_05390	non-specific lipid-transfer protein-like	K08764	KOG1406	554	334	205	296	207	101	181	199	2483	2466	629	2836	2410	2302	293	167
ACC_05391	nuclear transcription factor Y subunit beta isoform	K08065	KOG0869	243	175	139	314	191	48	71	93	230	143	117	261	588	514	185	116
ACC_05392	DNA-directed RNA polymerase III subunit RPC8-like	K03022	KOG3297	139	101	61	203	127	22	48	61	124	83	107	253	278	448	182	110
ACC_05393	l-lactate dehydrogenase A-like 6A-like		KOG1495	17	24	30	196	23	3	3	3	9	2	10	5	5	2	3	7
ACC_05394	l-lactate dehydrogenase-like		KOG1495	7	4	4	15	7	1	2	3	1	6	1	0	1	2	4	18
ACC_05395	UPF0563 protein C17orf95 homolog		KOG2793	252	162	178	149	148	13	15	20	527	361	99	442	382	438	31	10
ACC_05396	UNC93-like protein MFSD11-like		KOG3098	222	81	62	134	117	71	83	119	190	160	84	86	259	340	178	39
ACC_05397	LOW QUALITY PROTEIN			0	1	0	1	2	0	1	1	1	0	0	1	1	2	0	0
ACC_05398	ubiquitin carboxyl-terminal hydrolase 8-like	K11839	KOG1868	803	490	452	754	767	112	212	265	975	741	436	508	458	453	219	80
ACC_05399	cysteine--tRNA ligase, mitochondrial-like	K01883	KOG2007	216	118	113	168	210	7	19	32	298	136	147	219	445	523	45	40
ACC_05400	conserved hypothetical protein		KOG0161	335	154	188	276	259	95	230	197	181	149	160	134	26	67	362	182
ACC_05401	conserved hypothetical protein			646	261	185	483	659	23	276	122	213	293	267	516	703	986	706	689
ACC_05402	LOW QUALITY PROTEIN	K06071	KOG0694	343	318	300	429	215	47	56	83	1132	619	154	266	546	464	115	57
ACC_05403	YY1-associated factor 2-like	K11468	KOG4477	128	48	58	42	32	13	8	8	142	67	32	183	79	61	37	16
ACC_05404	UPF0139 membrane protein pMsmaA27-like		KOG3462	27	40	31	68	45	10	10	13	40	28	45	65	139	179	13	8
ACC_05405	proteasome subunit beta type-6-like	K02738	KOG0174	623	466	434	1317	895	118	151	196	1481	581	1005	1337	1667	2909	347	411
ACC_05406	sodium-coupled monocarboxylate transporter 1-like		KOG2349	604	438	476	1083	820	28	41	56	586	316	32	579	9	32	3	6
ACC_05407	ADP-ribosylation factor-like protein 5B-like	K07977	KOG0070	66	39	24	25	35	4	6	6	255	119	36	194	251	168	10	0
ACC_05408	LOW QUALITY PROTEIN	K06254	KOG1219	677	280	309	719	189	27	41	56	191	116	101	35	28	48	136	81
ACC_05409	protein yellow-like			20	34	23	17	18	1	1	3	5	6	9	9	444	128	6	26
ACC_05410	l-xylulose reductase	K03331	KOG1207	79	39	28	147	132	2	2	3	176	209	62	198	1414	455	34	17
ACC_05411	protein C10-like			36	28	48	117	87	8	6	10	39	17	42	52	22	26	8	1
ACC_05412	conserved hypothetical protein		KOG0109	245	207	204	395	170	18	35	39	327	264	73	188	320	377	143	38

ACC_05413	protein unc-45 homolog A		KOG4151	348	212	201	279	254	61	96	107	1208	1088	290	654	706	638	97	45
ACC_05414	mitochondrial import inner membrane translocase subunit T		KOG3225	161	115	110	237	213	17	48	38	212	133	172	277	400	538	89	38
ACC_05415	conserved hypothetical protein	K02331	KOG1926	536	322	294	417	489	88	184	246	477	599	239	312	1236	2013	1142	628
ACC_05416	LOW QUALITY PROTEIN		KOG2038	624	463	413	754	943	58	135	188	429	430	382	470	1217	1193	483	232
ACC_05417	Myosin regulatory light chain sqh	K12759	KOG0031	389	289	214	427	328	87	117	135	1153	410	566	1607	993	1065	197	148
ACC_05418	conserved hypothetical protein			477	264	301	562	147	58	66	97	665	566	117	236	48	33	203	80
ACC_05419	conserved hypothetical protein			173	130	95	334	324	43	60	59	507	231	524	1454	381	402	105	19
ACC_05420	anion exchange protein 2-like isoform 1		KOG1172	367	376	402	412	160	16	28	48	569	437	115	244	80	66	76	40
ACC_05421	lipopolysaccharide-induced tumor necrosis factor-alpha fact		KOG1924	91	139	95	152	75	21	17	16	421	359	156	622	384	230	54	15
ACC_05422	UDP-N-acetylglucosamine--peptide N-acetylglucos	K09667	KOG4626	1306	873	886	750	533	154	175	187	3802	2387	624	571	1069	1209	270	170
ACC_05423	U1 small nuclear ribonucleoprotein 70 kDa-like	K11093	KOG0113	1708	1050	1060	2466	1150	238	305	402	1576	929	770	422	1078	1510	627	224
ACC_05424	conserved hypothetical protein			97	72	56	92	37	19	11	15	159	108	55	147	43	34	22	3
ACC_05425	eukaryotic translation initiation factor 3 subunit G-	K03248	KOG0122	448	299	278	579	466	55	72	78	822	392	395	1054	1468	1969	350	134
ACC_05426	potassium voltage-gated channel protein Shaker-lil	K05318	KOG1545	629	208	255	673	153	41	68	104	379	862	91	22	13	7	68	60
ACC_05427	predicted protein			5	0	1	0	0	5	3	0	2	3	1	0	0	0	7	0
ACC_05428	V-type proton ATPase 116 kDa subunit a isoform 1-	K02154	KOG2189	2157	801	745	1976	1878	122	123	164	2535	1721	966	3198	2083	1779	202	77
ACC_05429	zinc finger protein 808-like		KOG2462	305	253	211	225	211	26	84	94	267	230	165	300	366	363	170	77
ACC_05430	conserved hypothetical protein	K10624	KOG0314	349	363	327	242	136	28	29	34	537	541	80	149	344	391	95	57
ACC_05431	conserved hypothetical protein		KOG1418	72	67	57	104	85	9	16	15	95	47	39	95	541	201	82	37
ACC_05432	conserved hypothetical protein		KOG3521	1000	780	759	1107	304	222	272	347	1285	416	659	306	73	207	246	74
ACC_05433	hypothetical protein			57	31	16	60	35	23	31	32	32	24	30	21	43	27	86	70
ACC_05434	tRNA guanosine-2'-O-methyltransferase TRM13 ho	K15446	KOG2811	134	83	67	107	112	2	14	9	153	78	133	648	192	243	39	9
ACC_05435	conserved hypothetical protein	K03007	KOG3497	158	158	160	307	319	15	27	25	173	49	234	390	230	229	39	30
ACC_05436	conserved hypothetical protein		KOG1015	1562	1086	941	1562	1112	328	529	582	2163	1471	920	802	1545	1714	1648	874
ACC_05437	poly	K10798	KOG1037	340	173	136	287	340	35	75	89	329	271	137	342	570	776	311	179
ACC_05438	conserved hypothetical protein		KOG4441	295	172	173	384	315	38	61	74	324	246	161	331	344	389	133	63
ACC_05439	conserved hypothetical protein			0	1	1	0	0	0	0	0	0	0	0	0	0	0	0	0
ACC_05440	sn1-specific diacylglycerol lipase beta-like		KOG2088	293	310	35	73	61	259	544	629	1239	848	795	490	288	272	216	235
ACC_05441	conserved hypothetical protein	K09884		55	35	18	90	55	4	8	12	94	38	16	55	8	2	3	2
ACC_05442	hypothetical protein			0	0	0	0	0	0	0	0	0	0	0	0	0	0	0	0
ACC_05443	hypothetical protein			0	0	0	0	0	0	0	0	0	0	0	0	0	0	0	0
ACC_05444	conserved hypothetical protein		KOG0307	356	348	451	772	245	79	93	112	2859	1354	241	664	185	122	118	148
ACC_05445	E3 ubiquitin-protein ligase mind-bomb-like	K10645	KOG4582	68	40	19	58	22	8	11	7	109	106	14	36	30	30	45	30
ACC_05446	f-actin-capping protein subunit alpha-like isoform 2	K10364	KOG0836	705	396	354	903	612	71	100	153	714	424	555	886	1029	886	225	114
ACC_05447	UPF0553 protein C9orf64 homolog		KOG2524	50	20	22	35	40	7	11	14	65	42	14	36	158	411	83	39
ACC_05448	MFS-type transporter C09D4.1-like isoform 1	K08220	KOG2563	700	324	333	278	202	21	43	57	810	760	77	326	444	160	45	21
ACC_05449	LOW QUALITY PROTEIN		KOG4309	3	2	3	16	10	1	2	0	6	1	15	13	19	55	10	5
ACC_05450	mediator of RNA polymerase II transcription subun	K13528	KOG4309	10	2	4	34	7	2	2	3	9	1	24	9	18	48	22	41
ACC_05451	trans-2,3-enoyl-CoA reductase-like	K10258	KOG1639	341	231	179	519	413	30	38	49	3546	1974	188	775	4188	3661	224	162
ACC_05452	cysteine-rich protein 2-binding protein-like		KOG3138	212	210	235	332	298	21	32	32	170	121	95	105	138	234	30	17
ACC_05453	dnaJ protein homolog 1-like	K09507	KOG0712	967	505	1191	1758	1220	127	242	357	1881	1155	481	924	918	1130	657	390
ACC_05454	40S ribosomal protein S11-like	K02949	KOG1728	2087	2069	1187	2007	1032	606	1118	1107	2294	1053	1644	2122	14071	16522	4044	2245
ACC_05455	conserved hypothetical protein	K11875		140	141	118	197	235	10	18	30	185	94	158	301	299	376	46	24
ACC_05456	ribosomal protein S6 kinase alpha-3 isoform 1	K04373	KOG0603	427	189	161	403	299	10	24	24	307	229	113	219	244	267	34	9
ACC_05457	conserved hypothetical protein		KOG3598	122	69	56	111	41	33	38	66	79	110	35	16	18	24	231	202
ACC_05458	solute carrier family 35 member F5-like	K15289	KOG2765	45	25	20	38	32	1	5	3	184	87	26	66	155	102	13	3
ACC_05459	inosine-5'-monophosphate dehydrogenase isoform	K00088	KOG2550	129	81	79	140	122	31	36	34	548	290	90	173	683	1111	121	33
ACC_05460	eukaryotic translation elongation factor 1 epsilon-1	K15439	KOG0867	109	85	72	249	235	11	28	35	115	92	110	204	436	533	73	59
ACC_05461	probable trafficking protein particle complex subunit 2-like		KOG3487	112	114	107	178	173	5	13	11	179	82	96	192	224	176	16	10
ACC_05462	LOW QUALITY PROTEIN	K13675	KOG2246	121	81	50	150	174	9	16	12	195	117	79	222	181	145	23	15
ACC_05463	nitrogen permease regulator 2-like protein-like isoform 1		KOG3789	369	294	280	452	413	53	76	94	470	260	251	413	501	511	74	30
ACC_05464	conserved hypothetical protein		KOG1710	69	48	62	87	67	18	20	15	121	190	78	60	24	15	23	12
ACC_05465	ras-related protein Rab-5C-like	K07889	KOG0092	331	139	155	429	337	26	40	41	767	468	189	623	521	468	77	18
ACC_05466	conserved hypothetical protein			39	28	29	96	67	4	7	9	40	22	87	27	35	23	3	3
ACC_05467	papilin-like		KOG3513	216	142	118	207	97	33	60	67	834	2380	65	209	456	408	287	85
ACC_05468	tryptophanyl-tRNA synthetase, cytoplasmic-like	K01867	KOG2145	441	345	367	601	550	35	57	74	304	236	194	444	641	710	109	56
ACC_05469	protein phosphatase 1B-like	K04461	KOG0697	378	243	200	374	278	40	53	58	875	892	156	671	725	732	109	42

ACC_05470	probable cleavage and polyadenylation specificity f	K14402	KOG1135	499	344	293	591	564	107	249	271	514	445	380	376	725	928	518	291
ACC_05471	conserved hypothetical protein	K12737	KOG0885	1216	845	810	1101	1036	228	468	460	1561	1346	350	334	709	771	804	383
ACC_05472	putative ferric-chelate reductase 1 homolog isoform 1		KOG4293	83	124	23	47	23	114	97	76	135	93	72	160	85	146	37	7
ACC_05473	DNA polymerase delta catalytic subunit-like	K02327	KOG0969	261	174	135	215	198	29	38	38	455	367	126	203	565	675	89	22
ACC_05474	ankyrin repeat and BTB/POZ domain-containing pr	K10521	KOG4441	136	69	56	139	113	6	7	15	207	158	29	73	181	216	59	22
ACC_05475	eukaryotic translation initiation factor 4 gamma 2-l	K03260	KOG0401	3235	1688	1641	2429	1502	589	680	967	4831	3246	1127	2032	2726	3612	2373	670
ACC_05476	periodic tryptophan protein 2 homolog	K14558	KOG0291	436	307	274	536	531	51	87	108	335	429	166	472	1256	1446	183	75
ACC_05477	elongation of very long chain fatty acids protein 4-l	K10249	KOG3071	35	25	25	46	44	0	0	0	48	20	16	15	33	207	12	0
ACC_05478	TBC1 domain family member 23-like		KOG3636	312	170	131	146	167	14	20	24	719	685	116	324	495	429	35	8
ACC_05479	conserved hypothetical protein		KOG2933	169	190	206	287	155	25	37	49	396	826	453	370	319	324	146	26
ACC_05480	transmembrane protein 62-like		KOG2502	390	175	200	248	236	22	22	28	504	331	93	344	349	347	43	25
ACC_05481	PBAN-type neuropeptides precursor			8	1	8	32	6	3	3	5	18	7	7	1	6	7	3	4
ACC_05482	THO complex subunit 3-like isoform 1	K12880	KOG1407	84	48	53	79	99	5	7	14	179	141	87	200	192	202	19	5
ACC_05483	conserved hypothetical protein			167	117	94	235	210	53	105	112	96	93	220	281	120	140	130	49
ACC_05484	conserved hypothetical protein			14	10	8	3	2	0	1	2	10	10	1	5	1	2	0	1
ACC_05485	nucleosome assembly protein 1-like 1-like	K11279	KOG1507	1079	692	508	1077	953	127	296	358	1332	892	472	918	4702	6904	3015	1761
ACC_05486	ubiquilin-1-like	K04523	KOG0010	701	478	491	450	264	51	51	56	1681	2013	124	1246	1707	1414	281	58
ACC_05487	survival motor neuron protein-like		KOG4327	136	88	94	177	161	10	18	30	164	90	118	160	309	432	122	49
ACC_05488	conserved hypothetical protein			80	11	9	11	6	6	14	16	211	66	5	48	24	21	4	4
ACC_05489	conserved hypothetical protein			3	3	1	6	0	0	0	0	2	12	1	1	2	0	6	5
ACC_05490	palmitoyltransferase ZDHHC17 isoform 1		KOG0509	74	38	35	36	34	4	8	8	230	116	52	51	100	92	11	4
ACC_05491	conserved hypothetical protein			184	85	105	187	164	13	24	39	206	71	97	64	98	144	35	8
ACC_05492	heat shock protein beta-1-like		KOG3591	394	429	399	613	288	106	110	136	4372	3077	287	1360	1183	414	158	55
ACC_05493	branched-chain-amino-acid aminotransferase, cyto	K00826	KOG0975	139	117	140	176	185	6	21	5	413	794	20	113	763	282	19	7
ACC_05494	apisimin			39083	897593	833591	99290	54581	982037	1064186	2016606	27	281	34	100	11	3	9	2
ACC_05495	guanine nucleotide-binding protein-like 1-like		KOG1424	285	230	260	348	285	25	33	59	400	396	109	270	484	506	105	62
ACC_05496	protein cappuccino homolog	K08366		103	111	126	136	103	7	8	12	104	71	64	231	208	126	21	4
ACC_05497	hypothetical protein			0	1	1	2	1	0	1	1	0	2	0	0	1	0	0	0
ACC_05498	actin, indirect flight muscle-like isoform 2		KOG0676	117	28	7	39	11	28	48	60	647	317	25	36	354	251	704	773
ACC_05499	conserved hypothetical protein			410	341	269	343	357	53	111	123	379	404	357	314	670	667	365	146
ACC_05500	conserved hypothetical protein		KOG1244	203	111	115	399	242	54	71	91	404	208	130	76	234	275	340	160
ACC_05501	cystathionine gamma-lyase-like	K01758	KOG0053	264	149	111	113	129	6	8	20	328	497	47	421	1887	1915	96	30
ACC_05502	ribosome biogenesis protein BRX1 homolog	K14820	KOG2971	264	177	141	240	278	35	102	135	250	165	179	340	1121	1293	439	191
ACC_05503	conserved hypothetical protein	K15685	KOG2932	175	204	250	318	119	26	34	36	248	205	106	224	228	273	135	48
ACC_05504	conserved hypothetical protein		KOG1700	1	1	1	2	0	1	0	1	5	26	2	3	0	0	0	0
ACC_05505	gamma-secretase subunit pen-2 isoform 1	K06170	KOG3402	116	99	129	208	238	24	25	30	110	46	92	160	181	258	30	12
ACC_05506	conserved hypothetical protein			224	141	111	175	188	13	13	11	185	294	61	885	1011	215	12	17
ACC_05507	sepiapterin reductase-like	K00072	KOG1204	374	266	223	341	418	31	54	52	330	378	139	809	2166	604	31	10
ACC_05508	NADH-cytochrome b5 reductase-like		KOG0534	219	164	159	214	319	21	64	57	155	79	179	207	79	76	16	6
ACC_05509	LOW QUALITY PROTEIN			501	378	330	472	453	33	76	91	775	537	238	380	848	1063	208	46
ACC_05510	conserved hypothetical protein		KOG1450	1253	433	427	510	346	66	98	91	791	488	62	199	219	160	86	40
ACC_05511	homeobox protein unc-62-like		KOG0773	217	146	121	155	63	28	35	36	343	339	145	240	104	118	140	23
ACC_05512	transcription elongation factor 1 homolog isoform 1		KOG3214	47	34	34	99	65	3	3	4	62	36	49	402	122	141	15	9
ACC_05513	conserved hypothetical protein	K10876	KOG1016	2346	1822	1583	1389	740	272	588	646	1717	1477	355	241	568	630	1063	496
ACC_05514	40S ribosomal protein S3a	K02984	KOG1628	4334	3843	2463	6777	4347	1055	1608	1906	5557	6036	5968	9711	20547	19674	5723	2505
ACC_05515	conserved hypothetical protein			56	11	16	34	18	5	9	17	48	19	930	1278	3	11	10	6
ACC_05516	protein son of sevenless-like		KOG3417	752	596	678	647	378	92	156	173	2131	1175	483	468	705	628	311	83
ACC_05517	lachesin-like		KOG3513	388	208	161	204	233	21	25	18	913	510	306	1030	643	573	30	7
ACC_05518	protein-tyrosine sulfotransferase		KOG3988	205	80	82	305	103	3	11	11	104	74	32	112	50	68	75	18
ACC_05519	DNA topoisomerase 2-binding protein 1-like	K10728	KOG1929	487	295	293	355	290	55	63	69	625	297	259	194	325	540	118	42
ACC_05520	DNA repair protein RAD51 homolog 4-like		KOG1434	33	19	10	15	24	0	1	2	13	10	15	41	100	84	9	9
ACC_05521	conserved hypothetical protein			453	334	283	475	366	58	108	131	449	251	212	311	246	241	156	76
ACC_05522	conserved hypothetical protein			1	3	8	4	1	0	1	1	4	4	7	2	3	3	1	0
ACC_05523	serine/threonine-protein phosphatase 4 regulatory	K15426	KOG0211	957	607	586	560	348	80	112	128	1440	858	263	595	205	108	77	33
ACC_05524	conserved hypothetical protein			72	65	60	98	56	5	7	14	85	62	42	100	166	208	42	30
ACC_05525	elongation factor 1-alpha	K03231	KOG0052	1482	825	973	3725	883	168	225	385	1054	841	360	680	68	42	64	56
ACC_05526	upstream stimulatory factor 1-like		KOG1318	130	109	140	179	159	21	33	41	285	94	132	221	161	150	21	9

ACC_05527	hypothetical protein			0	0	0	0	1	0	0	0	0	1	0	0	0	0	1	0
ACC_05528	polypeptide N-acetylgalactosaminyltransferase 35A	K00710	KOG3736	2264	2118	752	761	935	455	659	1339	864	794	307	537	1262	694	108	52
ACC_05529	exportin-2		KOG1992	221	155	175	403	299	14	31	49	354	250	133	142	540	794	79	20
ACC_05530	large neutral amino acids transporter small subunit 2-like		KOG1287	44	92	45	54	36	27	14	25	265	287	0	23	209	292	35	11
ACC_05531	mediator of RNA polymerase II transcription subun	K15162	KOG3598	1200	1167	1329	1234	493	223	282	343	1709	1060	410	323	740	1048	477	292
ACC_05532	glutamine synthetase 2 cytoplasmic-like isoform 2	K01915	KOG0683	4294	2530	5270	6478	4249	520	723	813	9238	7263	2061	7740	4821	4553	1989	811
ACC_05533	putative sodium-coupled neutral amino acid transp	K14994	KOG1305	163	118	146	171	179	7	14	19	156	178	111	165	312	200	16	1
ACC_05534	nuclear pore complex protein Nup88	K14318	KOG4460	301	231	228	382	277	45	115	113	236	192	140	146	467	713	306	227
ACC_05535	reticulocalbin-2-like		KOG4223	250	211	208	367	240	13	15	28	231	112	38	133	205	152	24	9
ACC_05536	DNA-directed RNA polymerase II subunit RPB3-like	K03011	KOG1522	179	166	153	306	243	25	42	52	259	134	247	372	256	495	88	60
ACC_05537	structural maintenance of chromosomes protein 3	K06669	KOG0964	1210	1006	906	1396	1332	318	531	711	959	742	723	424	1151	1672	2781	1967
ACC_05538	conserved hypothetical protein	K14405	KOG1049	322	220	237	427	215	54	56	83	436	289	188	307	491	864	226	259
ACC_05539	alpha-(1,6)-fucosyltransferase	K00717	KOG3705	172	91	75	108	126	9	15	20	305	296	79	437	327	249	58	22
ACC_05540	fasciculation and elongation protein zeta-2 isoform 1		KOG3919	2332	918	972	2712	1453	143	222	274	1750	784	593	1877	613	692	578	285
ACC_05541	conserved hypothetical protein			289	255	242	338	377	11	45	57	237	96	262	573	329	486	70	44
ACC_05542	acyl-CoA	K13514	KOG1505	50	38	25	64	64	6	4	4	89	119	30	134	151	136	17	6
ACC_05543	beta-catenin-like protein 1-like	K12864	KOG2734	379	291	273	385	359	50	82	103	539	427	198	505	1085	1134	214	99
ACC_05544	hypothetical protein			0	1	0	0	0	0	0	0	0	0	0	1	2	1	9	10
ACC_05545	protein pigeon-like isoform 1			682	389	309	534	468	50	56	53	561	429	227	207	221	242	44	17
ACC_05546	ninjurin-1-like			81	29	25	58	52	10	19	8	117	41	44	92	10	9	6	2
ACC_05547	acylphosphatase-1-like	K01512	KOG3360	90	109	113	269	259	7	26	23	99	33	106	96	51	67	22	9
ACC_05548	pseudouridine-5'-phosphate glycosidase-like	K16330	KOG3009	176	113	101	153	178	21	26	31	275	217	92	59	322	313	67	49
ACC_05549	LOW QUALITY PROTEIN	K13143	KOG3768	206	175	164	278	145	30	70	86	476	372	104	117	193	224	171	76
ACC_05550	LOW QUALITY PROTEIN		KOG3732	789	799	825	1195	447	48	85	87	2368	2588	201	1151	1817	1717	458	127
ACC_05551	UPF0468 protein C16orf80 homolog		KOG3213	94	74	94	125	123	14	14	31	123	121	65	318	182	243	63	21
ACC_05552	nicotinate phosphoribosyltransferase-like isoform :	K00763	KOG2511	507	405	282	469	471	56	101	96	1006	1424	388	814	585	721	42	20
ACC_05553	conserved hypothetical protein			163	94	118	187	244	8	16	8	116	93	101	249	150	178	21	6
ACC_05554	tektin-4-like		KOG2685	138	135	107	219	166	20	16	22	223	124	122	154	94	127	26	13
ACC_05555	dedicator of cytokinesis protein 3-like		KOG1998	236	107	105	283	107	15	11	22	280	152	106	56	32	46	24	5
ACC_05556	conserved hypothetical protein			286	172	128	134	148	16	52	41	72	91	105	81	201	186	98	32
ACC_05557	39S ribosomal protein L47, mitochondrial-like		KOG3331	514	398	425	631	581	30	53	70	658	447	205	566	1055	1441	147	44
ACC_05558	LOW QUALITY PROTEIN	K16469	KOG0161	463	324	264	432	426	74	135	175	283	233	200	61	334	401	568	510
ACC_05559	phosducin-like protein-like isoform 1		KOG3171	184	127	132	293	344	38	50	90	232	108	194	255	318	420	79	63
ACC_05560	conserved hypothetical protein		KOG1945	1239	1151	942	936	657	180	340	372	1529	1092	349	139	643	716	901	320
ACC_05561	LOW QUALITY PROTEIN		KOG3641	84	56	83	125	79	10	16	17	78	27	5	12	5	7	10	12
ACC_05562	cytochrome P450 4G11	K15001	KOG0157	1	31	12	33	23	3	5	9	2545	83	3	10	21251	34285	4217	331
ACC_05563	conserved hypothetical protein	K10369	KOG0445	1780	1134	975	1643	1100	349	584	782	1769	1657	480	667	1500	1183	704	264
ACC_05564	TIP41-like protein-like		KOG3224	108	84	73	172	132	11	20	18	113	84	69	144	219	321	33	19
ACC_05565	60S ribosomal protein L27a-like	K02900	KOG1742	981	1521	528	1684	1296	274	476	800	1337	623	1539	2197	7085	6963	1015	717
ACC_05566	ribonucleases P/MRP protein subunit POP1-like		KOG3322	107	114	75	177	158	8	9	11	95	109	39	33	265	524	77	23
ACC_05567	drebrin-like protein-like		KOG3655	706	549	506	707	483	93	142	195	492	363	216	438	754	768	365	267
ACC_05568	hypothetical protein			0	0	0	0	0	0	0	0	1	1	1	0	0	0	0	0
ACC_05569	DNA fragmentation factor subunit beta-like			696	329	262	290	101	14	22	23	411	224	20	68	11	34	63	52
ACC_05570	nucleostemin 1	K14538	KOG2484	335	224	213	508	389	84	185	198	348	290	356	261	835	1417	1513	1186
ACC_05571	conserved hypothetical protein		KOG0161	8	3	3	2	2	0	3	4	6	8	0	1	0	0	0	0
ACC_05572	conserved hypothetical protein			1	0	0	0	1	0	0	1	0	0	0	0	0	0	0	0
ACC_05573	RuvB-like 2	K11338	KOG2680	165	131	108	228	163	17	17	24	201	146	128	194	807	1278	261	173
ACC_05574	discoidin domain-containing receptor 2-like	K05125	KOG1094	114	56	47	79	19	2	4	7	42	50	43	13	2	4	14	5
ACC_05575	conserved hypothetical protein			226	102	129	187	46	9	6	18	45	68	22	0	7	5	103	35
ACC_05576	mitogen-activated protein kinase kinase kinase 13-	K04422	KOG4721	264	208	146	235	173	15	35	31	416	336	91	142	339	321	88	18
ACC_05577	pyruvate dehydrogenase phosphatase regulatory subunit, m		KOG2844	215	131	122	190	151	27	36	51	277	206	133	154	357	411	71	23
ACC_05578	protein max isoform 2	K04453	KOG2483	182	123	109	299	165	20	24	32	288	159	142	367	278	352	92	45
ACC_05579	conserved hypothetical protein	K15169	KOG1105	198	198	186	255	125	37	41	57	258	266	102	139	315	414	178	76
ACC_05580	conserved hypothetical protein		KOG1830	3	0	3	3	2	0	0	1	1	4	0	0	0	1	28	16
ACC_05581	UPF0565 protein C2orf69 homolog		KOG2800	47	38	39	26	48	1	3	4	53	45	27	83	69	68	7	0
ACC_05582	tetraspanin-1-like		KOG3882	83	42	41	148	137	5	2	3	288	113	90	417	87	86	10	4
ACC_05583	probable adenylate kinase isoenzyme F38B2.4-like	K00939	KOG3079	93	48	42	73	80	4	8	11	205	100	47	133	108	36	7	4

ACC_05584	lithostathine-1-alpha-like			39	27	26	31	32	1	4	2	27	28	36	9	20	7	0	1
ACC_05585	hypothetical protein			4	3	1	4	4	0	0	2	4	0	3	0	0	0	26	13
ACC_05586	CD63 antigen	K06497	KOG3882	249	103	97	124	114	21	15	29	329	203	247	2062	99	64	10	3
ACC_05587	alsin-like	K04575	KOG0231	1102	550	596	834	828	164	253	280	1267	758	317	440	500	544	388	146
ACC_05588	G protein-coupled receptor kinase 1-like	K00910	KOG0986	747	434	483	1003	349	104	140	192	791	420	245	240	229	346	344	113
ACC_05589	conserved hypothetical protein			134	89	79	152	130	14	18	27	110	54	59	103	74	39	20	9
ACC_05590	general transcription factor IIE subunit 2-like	K03137	KOG3095	122	53	51	65	49	9	19	19	43	24	27	81	53	53	17	9
ACC_05591	GTP-binding protein Di-Ras2-like		KOG0395	670	473	480	463	217	50	87	97	296	125	27	12	5	4	16	1
ACC_05592	conserved hypothetical protein		KOG3598	1239	903	800	770	483	173	253	310	1177	904	295	131	621	715	658	364
ACC_05593	hypothetical protein			0	0	0	0	0	0	0	0	0	0	0	0	0	0	1	0
ACC_05594	calcium-binding protein p22	K06268	KOG0034	245	149	139	248	270	19	27	38	399	168	169	525	343	449	62	27
ACC_05595	methyltransferase-like protein 9-like		KOG3987	52	37	32	132	40	6	7	9	69	95	57	306	40	18	38	3
ACC_05596	active regulator of SIRT1-like isoform 2			228	147	180	192	227	29	46	79	96	100	135	265	219	235	164	110
ACC_05597	nicotinamide riboside kinase	K10524	KOG3308	140	135	118	160	206	12	36	49	183	102	107	231	341	334	46	19
ACC_05598	WW domain-binding protein 2-like isoform 2		KOG3294	963	690	772	1287	724	201	184	264	2089	1403	811	1881	904	630	222	99
ACC_05599	upstream stimulatory factor 1-like isoform 1	K09106	KOG1318	156	116	112	252	117	20	25	22	356	192	102	364	225	252	70	17
ACC_05600	conserved hypothetical protein			179	86	133	383	79	4	5	9	98	85	232	586	3	7	16	7
ACC_05601	ufm1-specific protease 1-like		KOG2433	8	7	9	23	15	5	6	6	21	16	16	10	25	32	45	13
ACC_05602	multiple coagulation factor deficiency protein 2 homolog		KOG4065	575	693	352	323	183	126	134	181	619	504	253	1035	2058	1599	191	61
ACC_05603	LOW QUALITY PROTEIN	K12175	KOG0686	312	257	283	609	434	41	60	96	820	382	340	491	674	906	142	64
ACC_05604	inositol hexakisphosphate kinase 2-like		KOG1620	340	194	209	458	336	47	65	78	556	356	123	232	201	180	89	40
ACC_05605	inositol hexakisphosphate kinase 2-like			85	64	70	163	86	62	122	158	347	102	361	361	4	9	24	17
ACC_05606	UPF0293 protein C16orf42-like	K09140	KOG3154	186	84	76	306	220	36	54	51	219	147	200	366	305	280	115	98
ACC_05607	f-box only protein 32-like	K10305	KOG3926	425	168	189	277	149	96	124	150	409	254	150	156	58	58	85	56
ACC_05608	conserved hypothetical protein	K06067	KOG1342	352	164	133	267	281	85	155	192	203	267	154	222	533	685	1283	869
ACC_05609	putative sodium-coupled neutral amino acid transporter 10-		KOG1305	1074	615	452	787	963	59	293	229	851	808	671	1013	1062	990	301	75
ACC_05610	choline-phosphate cytidylyltransferase B-like	K00968	KOG2804	500	203	239	304	270	13	21	19	807	484	86	471	1281	927	81	32
ACC_05611	zinc finger protein 26-like		KOG2462	61	42	47	74	35	3	14	13	24	20	8	11	9	8	17	7
ACC_05612	1-phosphatidylinositol-4,5-bisphosphate phosphod	K05858	KOG1265	911	730	947	2626	757	116	162	201	1217	766	225	223	104	133	288	149
ACC_05613	conserved hypothetical protein			0	0	1	0	0	0	0	0	0	0	0	0	16	1	3	5
ACC_05614	conserved hypothetical protein			133	53	85	45	16	7	12	9	132	42	21	5	6	32	18	2
ACC_05615	visual system homeobox 1-like	K09336	KOG0494	121	72	79	125	21	5	4	12	33	19	0	1	1	5	12	4
ACC_05616	zinc finger protein 512B-like		KOG2418	26	0	1	0	0	0	0	0	4	4	2	31	2	8	2	6
ACC_05617	beta-arrestin-1-like	K04439	KOG3865	182	104	103	183	63	13	16	29	383	397	38	213	57	73	125	21
ACC_05618	cytochrome b5-like isoform 1		KOG0537	745	253	262	1198	556	486	645	764	2883	2361	3161	5531	2379	2091	6434	7317
ACC_05619	conserved hypothetical protein			279	153	149	375	291	30	50	66	500	348	116	301	629	655	222	62
ACC_05620	LIX1-like protein-like	K16673		37	27	24	38	38	7	13	12	54	36	30	72	56	62	29	15
ACC_05621	conserved hypothetical protein			21	0	2	13	5	2	3	3	31	7	14	96	24	24	31	68
ACC_05622	chromatin accessibility complex protein 1-like	K11656	KOG1657	105	58	44	125	94	14	45	41	62	29	73	67	69	133	110	71
ACC_05623	conserved hypothetical protein			38	24	42	38	42	0	2	6	19	27	76	175	68	76	7	1
ACC_05624	mitochondrial folate transporter/carrier-like	K15115	KOG0764	371	223	217	396	241	32	23	48	1266	855	83	375	573	494	129	36
ACC_05625	conserved hypothetical protein			0	0	0	3	0	0	0	0	1	1	0	0	2	3	13	24
ACC_05626	putative deoxyribonuclease TATDN1-like	K03424	KOG3020	177	75	97	251	261	7	18	14	170	71	116	97	303	288	48	18
ACC_05627	GPI mannosyltransferase 4-like	K08098	KOG4123	289	227	199	228	299	19	19	39	167	179	101	220	223	168	20	7
ACC_05628	glycosyltransferase-like protein LARGE1-like	K09668	KOG3765	266	194	199	363	266	49	56	87	217	229	85	167	211	185	60	13
ACC_05629	synaptotagmin-9		KOG1028	662	299	348	1183	307	44	46	66	179	80	11	6	0	4	7	11
ACC_05630	protein mab-21-like isoform 1		KOG3963	174	248	109	64	26	189	102	171	97	99	0	1	23	28	56	20
ACC_05631	hypothetical protein			2	8	2	7	1	0	0	0	1	13	1	0	0	0	5	1
ACC_05632	spermine oxidase-like		KOG0628	1	1	4	3	4	0	0	2	167	236	2	22	317	1071	79	39
ACC_05633	conserved hypothetical protein			1	3	0	1	1	0	1	0	1	2	0	1	0	0	0	0
ACC_05634	protein retinal degeneration B isoform 1		KOG3668	1056	730	660	793	475	79	100	110	2166	1504	356	501	604	812	122	23
ACC_05635	collagen alpha-1(IV) chain-like		KOG3546	1483	225	197	320	117	128	137	152	6124	1283	142	3949	5508	2794	2034	1215
ACC_05636	organic cation transporter protein-like		KOG0255	1817	1490	1948	6925	5181	124	131	195	3890	2331	284	529	267	307	53	34
ACC_05637	conserved hypothetical protein		KOG2462	171	109	89	136	115	11	25	31	151	101	60	108	151	169	70	26
ACC_05638	syntaxin-1B-like			33	42	45	21	25	2	0	1	26	18	22	42	121	145	4	1
ACC_05639	syntaxin-1B-like		KOG0810	26	31	27	28	21	3	8	8	63	17	46	97	176	197	21	4
ACC_05640	probable G-protein coupled receptor AH9.1-like			104	232	235	323	225	11	20	26	98	33	6	0	11	4	0	1

ACC_05641	conserved hypothetical protein		KOG1859	720	415	448	737	861	127	291	373	1319	948	588	509	541	370	278	118
ACC_05642	carbonic anhydrase 2-like	K01672	KOG0382	115	93	108	152	159	3	15	17	117	49	64	70	62	57	3	4
ACC_05643	vacuolar protein sorting-associated protein 13C-like		KOG1809	1616	954	650	1103	945	250	594	612	2763	3498	606	743	1174	812	407	194
ACC_05644	tetratricopeptide repeat protein 21B		KOG1496	295	161	136	210	176	26	29	31	239	229	311	464	525	414	40	8
ACC_05645	protein LMBR1L-like isoform 1		KOG3722	152	86	85	147	163	11	19	20	450	260	65	189	173	114	12	2
ACC_05646	39S ribosomal protein L39, mitochondrial-like		KOG1637	240	206	188	356	335	26	52	43	271	199	207	371	636	1027	118	77
ACC_05647	conserved hypothetical protein			58	54	39	62	88	7	20	19	157	117	54	132	290	308	29	14
ACC_05648	histone-lysine N-methyltransferase pr-set7	K11428	KOG1085	378	250	237	330	247	21	48	63	339	177	118	330	229	273	85	36
ACC_05649	elongator complex protein 1-like	K11373	KOG1920	278	167	125	265	360	24	54	54	196	350	129	257	663	732	108	46
ACC_05650	Down syndrome critical region protein 3 homolog		KOG2717	42	35	52	106	153	5	5	12	94	58	66	118	134	166	8	3
ACC_05651	NADH dehydrogenase	K03952	KOG3458	1076	670	757	1306	1140	110	191	232	1219	362	687	1849	1207	1373	250	118
ACC_05652	conserved hypothetical protein		KOG4448	479	389	640	715	256	99	110	194	784	519	231	260	128	262	373	148
ACC_05653	F-box-like/WD repeat-containing protein ebi isofor	K04508	KOG0273	221	134	128	332	238	20	26	24	358	271	131	446	625	775	187	50
ACC_05654	conserved hypothetical protein			1	0	0	2	1	0	0	1	3	12	1	1	1	2	2	1
ACC_05655	DTW domain-containing protein 1-like		KOG3795	44	48	34	75	75	5	5	12	50	23	73	112	49	46	4	1
ACC_05656	protein FAM100B-like			255	119	117	261	116	42	37	56	192	167	108	136	62	50	84	22
ACC_05657	LOW QUALITY PROTEIN			13	2	8	5	9	4	15	22	28	46	10	14	8	7	20	14
ACC_05658	restin homolog		KOG4568	2246	1620	1430	2036	2740	533	1386	1403	2928	2134	1508	595	620	591	648	429
ACC_05659	mitogen-activated protein kinase 1	K04371	KOG0660	683	305	264	429	345	87	138	156	496	351	182	357	431	483	178	82
ACC_05660	solute carrier family 23 member 2-like	K14611	KOG1292	309	228	259	872	686	14	21	31	658	545	224	178	660	876	147	10
ACC_05661	probable elongator complex protein 3-like	K07739	KOG2535	366	239	252	455	403	50	82	74	396	244	315	282	492	632	93	42
ACC_05662	TBC1 domain family member 4 isoform 2		KOG4436	408	347	250	435	306	75	96	114	1951	1407	468	680	144	163	59	19
ACC_05663	UPF0183 protein CG7083-like		KOG2819	278	197	169	359	240	99	80	170	533	246	106	181	144	77	35	10
ACC_05664	LOW QUALITY PROTEIN	K11463	KOG2350	2606	1539	1869	1238	1120	295	720	589	1155	646	388	392	800	992	159	76
ACC_05665	transmembrane channel-like protein 3-like			0	0	1	0	0	0	0	0	2	4	5	2	3	3	2	1
ACC_05666	locomotion-related protein Hikaru genki-like			660	393	461	939	368	36	53	73	283	183	200	455	8	17	22	3
ACC_05667	conserved hypothetical protein			50	59	77	102	63	6	21	25	6	42	17	3	3	19	26	5
ACC_05668	triple functional domain protein-like	K08810	KOG4240	618	560	420	353	197	97	116	138	1216	1153	256	438	424	310	283	102
ACC_05669	probable ATP-dependent RNA helicase pitchoune-l	K13179	KOG0342	739	326	316	509	774	85	234	199	865	429	542	447	805	1467	660	468
ACC_05670	thioredoxin-like protein 4A-like	K12859	KOG3414	136	112	118	254	317	11	31	35	181	91	247	513	366	333	32	21
ACC_05671	t-complex protein 11-like protein 1-like isoform 1		KOG1981	474	282	245	353	316	37	63	71	486	349	156	524	790	500	126	45
ACC_05672	glucosamine-6-phosphate isomerase isoform 1	K02564	KOG3148	144	116	69	106	148	11	25	20	244	248	126	434	1688	1222	88	22
ACC_05673	conserved hypothetical protein			73	50	59	136	151	3	5	7	160	72	944	5922	4046	7946	345	24
ACC_05674	hypothetical protein			0	0	0	1	0	0	0	0	1	1	0	1	0	0	0	0
ACC_05675	transmembrane protein 70, mitochondrial-like		KOG4478	478	232	260	374	527	4	14	13	155	218	221	660	438	392	58	28
ACC_05676	glutamine:fructose-6-phosphate aminotransferase	K00820	KOG1268	256	181	106	209	164	29	40	44	427	506	197	524	1299	634	38	32
ACC_05677	venom allergen 3-like		KOG3017	34	18	26	71	42	2	3	4	21	15	6	8	3	1	8	2
ACC_05678	calcium/calmodulin-dependent protein kinase II	K04515	KOG0033	2148	1509	1467	743	231	149	206	333	1170	474	94	39	15	24	37	41
ACC_05679	gamma-secretase subunit Aph-1-like	K06172	KOG3972	92	73	55	132	101	13	17	21	317	156	62	357	203	258	46	3
ACC_05680	inhibitor of growth protein 1-like		KOG1973	127	110	102	232	200	21	38	62	123	113	82	166	138	126	115	56
ACC_05681	vitellogenin receptor-like		KOG1215	23	33	14	24	18	1	4	4	9	123	13	17	117	51	7	8
ACC_05682	far upstream element-binding protein 1-like	K13210	KOG1676	2041	1492	1570	5607	666	634	1099	1467	1098	920	546	514	832	1079	940	369
ACC_05683	serine protease gd-like		KOG3627	26	16	28	46	27	6	5	8	79	70	35	13	9	16	8	3
ACC_05684	serine/threonine-protein phosphatase 2B catalytic	K04348	KOG0375	586	360	302	502	135	60	76	150	326	369	77	60	30	33	174	94
ACC_05685	ubiquitin-conjugating enzyme E2 J1-like	K10578	KOG0428	338	300	284	385	213	25	32	48	345	402	103	606	453	455	46	9
ACC_05686	LOW QUALITY PROTEIN		KOG2645	264	135	116	211	282	10	5	16	484	398	141	687	375	321	13	1
ACC_05687	conserved hypothetical protein		KOG0946	29	8	8	23	15	10	13	13	370	366	44	459	118	50	29	6
ACC_05688	glucose dehydrogenase		KOG1238	13	5	7	27	10	1	3	5	117	230	96	211	203	263	44	15
ACC_05689	short spindle protein 4-like isoform 2		KOG3654	295	171	150	222	105	62	119	127	528	430	91	78	102	201	552	438
ACC_05690	hypothetical protein			2396	572	44	175	47	1207	1390	1499	3869	32	251	23575	4	0	0	3
ACC_05691	conserved hypothetical protein			29	14	14	21	26	9	5	7	187	167	5	9	105	57	2	13
ACC_05692	conserved hypothetical protein		KOG0509	20	37	64	22	23	2	2	1	9	2	5	10	35	29	7	1
ACC_05693	conserved hypothetical protein		KOG0004	1925	781	900	2710	2699	65	65	76	1611	1057	412	1541	370	308	32	8
ACC_05694	conserved hypothetical protein		KOG4305	101	62	53	137	63	34	32	32	785	410	272	588	324	245	81	96
ACC_05695	RING finger protein 146-like	K15700	KOG0824	135	77	71	119	102	7	16	15	174	75	72	203	133	170	51	12
ACC_05696	probable RNA helicase armi		KOG1804	476	273	255	475	473	16	33	52	451	462	167	391	612	718	111	27
ACC_05697	U4/U6 small nuclear ribonucleoprotein Prp3		KOG2769	285	228	188	353	216	63	91	106	339	293	146	203	308	471	348	205

ACC_05698	peroxidase		KOG2408	12	5	8	28	13	0	1	1	22	19	24	47	77	33	9	3
ACC_05699	heat shock 70 kDa protein cognate 5-like	K04043	KOG0102	3422	2016	1956	3049	1885	476	794	1026	4966	5046	1581	2830	15010	27554	10502	8282
ACC_05700	protein farnesyltransferase/geranylgeranyltransfer	K05955	KOG0530	200	142	141	283	319	28	35	55	333	320	197	584	920	1448	145	29
ACC_05701	conserved hypothetical protein			1	0	0	7	2	1	0	0	2	9	0	1	6	3	0	0
ACC_05702	Transcription factor collier	K09103	KOG3836	23	17	19	34	14	1	1	0	14	16	1	2	12	6	27	2
ACC_05703	ATP-dependent RNA helicase vasa	K13982	KOG0335	201	133	103	147	98	56	72	100	227	124	64	66	278	500	172	92
ACC_05704	conserved hypothetical protein			16	6	3	6	7	23	28	39	2696	554	122	84	11	2	0	0
ACC_05705	ferrochelatase, mitochondrial-like	K01772	KOG1321	206	106	121	192	178	29	23	28	300	189	131	274	364	376	43	16
ACC_05706	leishmanolysin-like peptidase	K13539	KOG2556	131	126	90	53	36	13	19	31	260	147	30	115	97	73	25	23
ACC_05707	tether containing UBX domain for GLUT4	K15627		471	284	275	412	457	48	111	126	441	315	284	470	650	705	385	250
ACC_05708	conserved hypothetical protein		KOG4676	1223	898	593	1276	1169	213	598	711	477	569	600	286	444	709	1321	646
ACC_05709	conserved hypothetical protein			167	216	198	601	522	20	32	31	692	495	417	1435	630	480	85	27
ACC_05710	H(+)/Cl(-) exchange transporter 3-like isoform 1	K05012	KOG0475	1045	601	584	1087	961	125	141	169	2575	1703	699	1511	1602	1136	106	29
ACC_05711	exosome complex component MTR3-like	K12587	KOG1068	155	145	120	325	270	27	17	27	194	146	203	663	481	467	63	26
ACC_05712	AP-2 complex subunit sigma-like	K11827	KOG0935	159	152	180	274	392	12	26	50	164	80	151	302	211	196	15	9
ACC_05713	HEAT repeat-containing protein 2-like	K12462		83	64	53	58	96	6	7	8	99	37	550	738	31	43	1	2
ACC_05714	protein PRRC1-like isoform 2			341	220	237	400	343	30	36	27	728	410	351	970	338	372	53	18
ACC_05715	excitatory amino acid transporter 3		KOG3787	47	34	38	81	80	9	4	4	452	240	50	125	942	675	50	7
ACC_05716	NEDD8-conjugating enzyme UBE2F-like	K10687	KOG0420	87	50	71	141	141	5	21	29	144	68	62	133	176	332	27	15
ACC_05717	conserved hypothetical protein			0	0	0	3	0	0	1	1	19	1	2	0	2	1	3	2
ACC_05718	protein expanded-like	K16683	KOG4371	101	129	171	93	30	9	10	17	184	130	46	57	72	153	99	46
ACC_05719	protein SHQ1 homolog	K14764	KOG3247	195	129	121	209	336	15	21	16	301	222	161	281	318	301	25	8
ACC_05720	conserved hypothetical protein		KOG2482	275	206	200	241	301	16	36	47	269	247	191	354	401	389	58	19
ACC_05721	conserved hypothetical protein		KOG4114	96	115	128	192	191	16	28	31	53	42	77	177	152	163	52	28
ACC_05722	lamin-B receptor-like		KOG1435	98	111	96	130	151	3	20	20	153	124	153	117	153	277	59	21
ACC_05723	guanylate kinase-like	K00942	KOG0707	347	184	188	193	190	38	50	68	451	253	36	163	425	358	43	18
ACC_05724	anaphase-promoting complex subunit 4-like isofor	K03351	KOG4640	194	127	140	205	195	22	32	42	245	190	109	233	410	377	51	17
ACC_05725	NADH-cytochrome b5 reductase 2-like isoform 1	K00326	KOG0534	355	209	224	538	624	42	30	49	691	652	225	1085	2192	1456	108	60
ACC_05726	conserved hypothetical protein			2	0	0	9	2	0	1	0	1	4	2	2	3	2	4	0
ACC_05727	conserved hypothetical protein	K11316		132	84	78	132	150	10	28	30	105	54	72	126	172	356	68	22
ACC_05728	ADP-ribosylation factor GTPase-activating protein 2	K12493	KOG0706	1057	833	639	1276	1015	281	437	471	1868	1026	889	1947	2040	2126	862	556
ACC_05729	phosphatidylinositol 3-kinase catalytic subunit type	K00914	KOG0906	363	248	245	472	418	46	60	58	909	532	264	537	761	701	63	27
ACC_05730	G kinase-anchoring protein 1-like			740	447	400	696	603	142	334	360	273	219	278	210	284	332	1222	948
ACC_05731	myelin expression factor 2-like		KOG4212	846	414	432	1037	694	43	44	65	891	976	208	553	1141	1827	356	84
ACC_05732	conserved hypothetical protein		KOG0670	5	1	1	0	0	0	0	0	1	4	0	3	0	0	0	2
ACC_05733	protein groucho-like	K04497	KOG0639	145	96	78	112	39	18	22	18	201	215	49	77	37	33	66	45
ACC_05734	conserved hypothetical protein		KOG4219	72	64	64	44	25	3	4	4	52	40	12	33	7	7	4	1
ACC_05735	pikachurin-like			52	26	29	70	8	0	6	4	14	15	12	7	0	1	5	4
ACC_05736	e3 SUMO-protein ligase PIAS3	K16063	KOG2169	307	310	235	305	186	50	71	89	379	344	148	191	373	423	222	93
ACC_05737	cytochrome c oxidase subunit 1			0	0	0	0	0	0	0	0	0	0	0	0	0	0	0	0
ACC_05738	cyclin-dependent kinase 14-like	K08821	KOG0594	214	121	123	276	163	30	34	63	370	204	52	188	240	220	79	32
ACC_05739	lisH domain and HEAT repeat-containing protein KIAA1468 h		KOG0211	608	456	416	491	477	43	55	64	1141	643	382	519	465	366	42	7
ACC_05740	geranylgeranyl transferase type-1 subunit beta-like	K11713	KOG0367	191	97	85	168	146	22	35	34	272	144	139	315	375	520	79	14
ACC_05741	tumor necrosis factor alpha-induced protein 8-like protein-like			370	203	192	563	410	98	178	200	462	269	473	1232	152	94	47	21
ACC_05742	protein prenyltransferase alpha subunit repeat-cor	K14137	KOG0529	150	77	82	290	147	13	8	7	203	140	38	198	155	176	86	6
ACC_05743	conserved hypothetical protein		KOG1032	1136	770	784	1014	318	74	113	147	982	613	5373	2449	391	267	287	119
ACC_05744	conserved hypothetical protein			35	28	13	30	17	8	11	16	21	25	1	9	14	15	6	6
ACC_05745	LOW QUALITY PROTEIN	K08851	KOG3087	167	144	199	204	231	10	17	21	242	189	270	444	292	357	45	17
ACC_05746	enoyl-CoA delta isomerase 1, mitochondrial-like	K13238	KOG1683	647	401	367	268	337	67	98	90	515	500	432	1233	2840	1731	60	26
ACC_05747	conserved hypothetical protein		KOG2426	334	317	370	226	301	47	103	110	403	264	193	199	281	400	89	41
ACC_05748	hypothetical protein			1	0	2	4	0	0	0	0	0	0	1	0	0	1	1	0
ACC_05749	zinc finger protein Gfi-1b-like		KOG2462	114	62	57	94	73	2	1	2	99	66	26	119	52	32	5	0
ACC_05750	probable ATP-dependent RNA helicase YTHDC2		KOG0920	226	154	114	185	166	27	32	38	767	625	112	472	697	600	83	30
ACC_05751	hypothetical protein			0	0	0	0	0	0	0	0	2	4	0	0	0	0	0	0
ACC_05752	argonaute	K02156	KOG1042	110	83	101	104	107	5	8	19	83	91	53	79	106	145	22	9
ACC_05753	protein odd-skipped-like	K09215	KOG3623	2	0	4	0	2	0	1	0	5	0	1	0	5	8	13	2
ACC_05754	conserved hypothetical protein			2	2	1	1	0	0	0	1	3	7	1	0	0	1	3	2

ACC_05755	conserved hypothetical protein			14	11	17	51	45	21	27	9	68	154	66	86	15	9	8	10
ACC_05756	histidine protein methyltransferase 1 homolog		KOG2920	80	72	88	194	225	14	44	41	83	68	116	175	169	213	52	28
ACC_05757	hypothetical protein			0	0	0	0	0	0	0	0	0	0	0	0	0	0	0	0
ACC_05758	hypothetical protein			0	0	0	0	0	0	0	0	0	0	0	0	0	0	0	0
ACC_05759	myeloma-overexpressed gene 2 protein homolog			27	23	33	100	47	6	9	8	17	14	73	68	161	142	13	6
ACC_05760	protein Mo25-like	K08272	KOG1566	418	321	273	748	527	71	91	125	797	333	262	461	610	913	95	31
ACC_05761	palmitoyltransferase ZDHHC3-like		KOG1311	248	159	150	367	299	15	10	15	257	207	100	373	180	151	27	6
ACC_05762	GABA neurotransmitter transporter-1A	K05034	KOG3660	1571	216	183	236	212	11	22	21	846	122	0	2	9	48	11	9
ACC_05763	conserved hypothetical protein		KOG1103	350	176	144	309	252	64	160	156	327	203	172	118	152	195	527	346
ACC_05764	BRCA1-associated protein-like	K10632	KOG0804	230	153	138	309	230	26	27	45	323	159	150	238	262	200	57	12
ACC_05765	neuropilin and tolloid-like protein 2-like		KOG4586	162	89	86	69	23	2	3	6	137	138	12	123	15	25	20	20
ACC_05766	conserved hypothetical protein			37	18	11	29	13	1	1	1	47	35	11	30	1	8	5	0
ACC_05767	LIM and SH3 domain protein Lasp-like		KOG1702	99	35	63	42	13	11	16	20	243	308	21	149	221	216	258	100
ACC_05768	BTB/POZ domain-containing protein 2-like		KOG2075	343	259	305	456	504	27	39	50	605	330	290	354	337	380	30	9
ACC_05769	WD repeat-containing protein 7 isoform 1		KOG0271	983	763	548	538	326	70	99	140	1750	1321	335	441	469	368	89	40
ACC_05770	growth hormone-inducible transmembrane protein-like		KOG1630	1260	836	890	3844	2664	180	156	249	3369	1786	974	2545	2207	3322	340	80
ACC_05771	tribbles homolog 2	K08814	KOG0583	109	43	33	73	23	5	5	6	259	242	35	137	127	68	51	21
ACC_05772	LOW QUALITY PROTEIN	K07197	KOG2588	2112	913	1035	2378	1969	148	165	237	5320	2973	1758	3009	8954	4955	492	101
ACC_05773	anaphase-promoting complex subunit 7	K03354	KOG1174	194	128	102	241	229	8	16	17	380	292	149	350	243	309	25	3
ACC_05774	conserved hypothetical protein			1217	671	535	1077	985	110	224	304	589	675	433	376	496	430	343	107
ACC_05775	GTP-binding protein Rheb homolog	K07208	KOG0395	523	398	525	737	598	68	102	132	784	330	420	1208	524	667	155	42
ACC_05776	conserved hypothetical protein			743	282	349	1357	959	35	49	89	624	149	757	804	423	572	97	66
ACC_05777	conserved hypothetical protein		KOG0614	91	91	95	128	87	4	8	8	131	148	26	55	133	117	26	17
ACC_05778	pro-corazonin preproprotein			2	6	10	31	26	0	0	0	1	0	0	0	0	0	0	1
ACC_05779	solute carrier family 25 member 42-like isoform 1	K15085	KOG0752	334	245	232	225	115	24	21	36	674	270	199	354	380	311	59	23
ACC_05780	nucleolar protein 56	K14564	KOG2573	1149	490	510	771	712	147	315	377	582	475	452	616	1800	3229	2369	1354
ACC_05781	FGFR1 oncogene partner 2 homolog			121	123	157	191	164	9	27	23	162	82	131	267	244	442	60	23
ACC_05782	conserved hypothetical protein			368	186	171	204	101	48	50	90	262	305	43	52	37	40	164	46
ACC_05783	cysteine protease ATG4B-like	K08342	KOG2674	128	95	105	221	218	10	19	34	184	131	90	174	324	320	53	8
ACC_05784	dedicator of cytokinesis protein 9-like		KOG1997	159	110	105	180	109	12	13	26	269	492	41	100	193	168	21	12
ACC_05785	protein CWC15 homolog A-like isoform 1	K12863	KOG3228	333	253	252	538	361	69	77	132	354	211	196	368	369	551	210	234
ACC_05786	protein 60A	K16621	KOG3900	10	1	4	19	2	4	5	5	72	143	9	29	47	38	65	37
ACC_05787	prolyl endopeptidase-like isoform 1	K01322	KOG2237	459	293	227	280	296	45	67	72	633	618	200	641	1216	1260	121	66
ACC_05788	conserved hypothetical protein			494	225	196	683	671	71	161	170	582	270	452	530	197	183	110	62
ACC_05789	UPF0693 protein C10orf32 homolog			21	20	34	24	27	1	5	1	19	9	21	44	3	3	0	0
ACC_05790	high mobility group protein DSP1-like	K10802	KOG3598	848	549	630	604	204	87	121	154	753	746	192	710	551	736	910	458
ACC_05791	conserved hypothetical protein		KOG2043	1667	1171	1173	1731	1730	306	727	799	1282	1037	1126	766	1495	1317	1254	492
ACC_05792	protein NPC2 homolog		KOG4063	418	155	116	809	258	368	366	556	1742	1314	798	1343	437	305	500	287
ACC_05793	ribosomal protein S6 kinase alpha-3-like		KOG0598	455	154	176	590	453	72	127	121	309	119	144	59	72	93	144	112
ACC_05794	nucleolysin TIAR	K13201	KOG0148	138	104	137	135	52	8	7	6	395	483	42	363	559	758	161	29
ACC_05795	liprin-beta-1-like		KOG1899	572	466	381	605	427	74	136	138	1033	755	413	835	1037	1019	267	127
ACC_05796	conserved hypothetical protein			634	242	266	633	336	13	29	29	192	59	120	289	9	23	7	2
ACC_05797	conserved hypothetical protein		KOG2748	148	69	85	84	35	4	4	8	222	170	35	89	59	65	28	8
ACC_05798	WD repeat-containing protein 75-like	K14552	KOG1963	294	194	155	294	309	23	41	44	315	421	106	211	898	1128	127	26
ACC_05799	tRNA pseudouridine synthase-like 1-like		KOG4393	125	100	96	180	176	12	22	19	110	126	95	139	455	529	39	16
ACC_05800	dual specificity protein phosphatase 19-like	K14165	KOG1716	91	70	99	153	156	8	19	24	88	94	75	73	206	224	34	9
ACC_05801	nitric oxide synthase	K13253	KOG1158	822	482	529	556	473	21	32	48	415	253	222	1198	1641	1021	102	25
ACC_05802	coenzyme Q-binding protein COQ10 homolog B, mitochondr		KOG3177	33	44	46	29	50	2	2	10	51	33	66	51	65	97	19	7
ACC_05803	conserved hypothetical protein		KOG4619	153	116	143	348	280	10	22	24	121	65	60	132	89	139	17	13
ACC_05804	actin-binding protein IPP-like	K13956	KOG4441	143	55	58	133	120	18	24	24	412	315	48	218	173	114	26	2
ACC_05805	hypothetical protein			2	3	0	3	8	0	1	1	12	9	2	1	3	1	0	0
ACC_05806	methyltransferase-like isoform 1		KOG2361	296	217	235	241	280	37	56	56	378	238	237	476	961	829	100	68
ACC_05807	UPF0663 transmembrane protein C17orf28-like		KOG2226	251	143	151	151	151	9	12	15	297	265	61	134	208	176	20	5
ACC_05808	GTPase Era, mitochondrial-like		KOG1423	228	212	227	344	392	26	42	58	234	141	321	315	412	559	76	47
ACC_05809	tyramine beta hydroxylase	K00503	KOG3568	48	47	70	105	86	2	6	9	49	20	15	5	4	8	8	1
ACC_05810	conserved hypothetical protein		KOG4244	554	242	228	541	184	52	84	101	262	135	207	419	240	174	543	243
ACC_05811	protein smoothened	K06226	KOG3577	309	216	225	322	304	25	31	43	371	296	107	154	434	441	80	32

ACC_05812	cytochrome b5 reductase 4-like			7	6	5	12	7	5	2	3	13	21	1	9	8	12	1	2
ACC_05813	splicing factor U2af 38 kDa subunit	K12836	KOG2202	281	244	206	511	457	46	78	86	649	287	354	585	702	984	208	132
ACC_05814	RNA polymerase II-associated protein 1-like		KOG4732	778	356	304	578	833	62	174	204	775	585	432	501	589	766	348	163
ACC_05815	rho-related GTP-binding protein RhoU-like		KOG0393	79	33	50	59	46	11	14	15	284	118	70	73	94	75	46	14
ACC_05816	f-box/LRR-repeat protein 21-like	K10269	KOG1947	44	40	70	118	97	15	20	23	386	280	65	134	158	43	12	4
ACC_05817	fatty acyl-CoA reductase 1	K13356	KOG1221	827	395	313	1159	585	176	168	173	4936	5959	75	166	326	474	287	68
ACC_05818	conserved hypothetical protein		KOG4724	167	138	191	308	123	37	41	55	705	407	114	229	137	216	121	35
ACC_05819	aquaporin-4-like		KOG0223	3	17	16	23	15	1	1	2	17	55	53	17	277	331	83	4
ACC_05820	centrosomal protein of 78 kDa-like	K16765	KOG4308	13822	10825	10856	50348	36348	3881	7288	8744	13282	7842	1459	586	779	1962	2319	749
ACC_05821	epoxide hydrolase 4-like		KOG4178	9	16	15	16	13	0	0	0	60	242	3	17	916	506	78	12
ACC_05822	mitochondrial import receptor subunit TOM20 homolog		KOG4056	422	192	134	352	284	54	73	99	608	293	229	870	850	1022	202	94
ACC_05823	tubulin-specific chaperone E-like	K01759	KOG2944	133	118	129	397	425	14	23	32	400	193	245	393	723	545	36	11
ACC_05824	tubulin-specific chaperone E-like		KOG3207	188	165	198	350	386	12	23	32	231	193	134	213	440	453	34	13
ACC_05825	rRNA 2'-O-methyltransferase fibrillarin	K14563	KOG1596	417	218	141	284	211	34	56	84	466	365	167	328	1872	3113	857	502
ACC_05826	conserved hypothetical protein		KOG1144	96	59	75	81	94	16	67	67	61	25	72	9	109	241	414	356
ACC_05827	LOW QUALITY PROTEIN	K13144	KOG1988	220	134	152	388	253	31	30	28	237	202	127	122	216	252	60	18
ACC_05828	leucine-rich repeat transmembrane neuronal protein 2-like		KOG4194	6	12	16	80	67	32	25	32	511	384	64	151	15	11	1	0
ACC_05829	hypothetical protein			31	6	8	10	7	7	10	6	19	3	4	1	2	0	63	37
ACC_05830	conserved hypothetical protein			133	109	121	156	150	11	23	37	198	84	133	216	175	246	29	19
ACC_05831	enhancer of mRNA-decapping protein 3-like	K12615	KOG2585	163	131	123	156	122	12	16	18	184	169	68	201	556	653	81	35
ACC_05832	hypothetical protein			11	4	1	4	2	0	0	2	5	35	1	12	3	2	3	0
ACC_05833	dual specificity tyrosine-phosphorylation-regulated kinase 2-		KOG0667	304	192	235	562	140	30	38	66	244	129	163	70	48	67	240	90
ACC_05834	conserved hypothetical protein			25	22	8	13	26	1	2	0	5	20	15	53	74	74	5	6
ACC_05835	glucose-6-phosphate 1-dehydrogenase, partial	K00036	KOG0563	49	47	32	47	50	11	30	35	832	805	492	1152	4146	1805	243	62
ACC_05836	protein wntless-like			77	64	45	58	62	5	4	13	91	50	40	40	112	145	14	5
ACC_05837	ubiquitin-conjugating enzyme E2 H-like	K10576	KOG0416	487	201	226	824	300	123	146	197	479	244	304	161	63	66	210	112
ACC_05838	inositol 1,4,5-trisphosphate receptor	K04958	KOG3533	2350	2278	1738	2000	1129	284	474	650	3118	2165	341	343	1236	632	373	150
ACC_05839	hypothetical protein			1	0	0	4	0	0	1	0	3	23	0	1	0	0	1	0
ACC_05840	conserved hypothetical protein			2	1	2	1	1	0	0	0	4	5	0	7	8	154	29	151
ACC_05841	protein phosphatase 1H-like		KOG1323	100	54	48	126	123	9	12	11	133	58	53	99	105	125	16	4
ACC_05842	conserved hypothetical protein			0	0	0	12	2	0	0	1	20	1	1	1	8503	8527	2282	71
ACC_05843	conserved hypothetical protein	K05463	KOG3858	29	18	15	45	13	0	3	8	20	24	11	13	8	11	106	79
ACC_05844	low molecular weight phosphotyrosine protein phc	K14394	KOG3217	91	73	66	133	161	5	13	13	133	45	130	181	205	341	43	22
ACC_05845	low molecular weight phosphotyrosine protein phc	K14394	KOG3217	79	70	83	150	141	3	8	4	107	27	71	84	64	88	8	0
ACC_05846	dynein-1-beta heavy chain, flagellar inner arm I1 complex-like			22	18	36	31	31	4	5	8	51	40	29	58	292	107	16	6
ACC_05847	membrane-bound transcription factor site-2 protea	K07765	KOG2921	155	121	116	153	163	12	22	23	303	146	121	369	211	201	24	9
ACC_05848	hypothetical protein			0	0	0	4	1	0	0	1	0	0	0	0	0	0	0	0
ACC_05849	hypothetical protein			0	0	0	0	0	0	0	0	1	0	0	0	0	0	0	0
ACC_05850	conserved hypothetical protein		KOG3803	223	199	237	360	41	17	17	20	105	66	132	120	1	7	26	14
ACC_05851	hypothetical protein			21	14	27	26	10	1	4	1	7	6	8	0	0	1	3	2
ACC_05852	phospholipase A1 member A-like			0	1	1	0	1	0	0	0	0	3	1	2	127	55	36	19
ACC_05853	40S ribosomal protein S27-like	K02978	KOG1779	504	384	166	886	532	174	287	432	206	302	468	437	2785	2964	976	679
ACC_05854	peptidyl-tRNA hydrolase 2, mitochondrial-like isofc	K04794	KOG3282	125	107	98	94	129	7	18	29	118	53	106	271	268	378	39	30
ACC_05855	vang-like protein 1-like	K04510	KOG3814	117	61	63	76	55	8	14	16	121	103	21	52	210	156	46	20
ACC_05856	prefoldin subunit 2-like isoform 1	K09549	KOG4098	424	310	284	430	429	36	97	109	482	464	232	1033	883	1243	270	236
ACC_05857	malignant T cell-amplified sequence 1-like	K07575	KOG2523	95	77	66	163	164	3	23	24	136	59	83	178	486	574	62	59
ACC_05858	conserved hypothetical protein		KOG1028	380	180	222	414	226	42	51	103	129	121	85	9	26	48	51	126
ACC_05859	heat shock 70 kDa protein 4L isoform 1	K09485	KOG0103	2805	1217	1638	3039	1346	250	417	559	2710	2246	809	2144	5083	5324	5020	4363
ACC_05860	hypothetical protein			205	74	116	188	89	11	7	12	81	45	4	11	1	0	4	2
ACC_05861	conserved hypothetical protein			26	11	20	14	6	4	5	4	77	60	39	66	17	26	22	18
ACC_05862	conserved hypothetical protein			1	0	0	3	1	1	0	0	3	1	2	0	0	2	18	25
ACC_05863	ubiquitin-conjugating enzyme E2 S-like	K10583	KOG0423	227	183	150	648	435	72	104	136	210	150	232	316	316	431	266	160
ACC_05864	protein dispatched-like		KOG3664	308	192	151	282	335	20	63	84	312	220	72	26	284	423	223	123
ACC_05865	protein dispatched-like	K16746		4	4	4	4	4	0	1	1	18	4	142	242	8	8	2	1
ACC_05866	hypothetical protein			174	77	80	121	126	21	41	71	63	24	29	8	7	25	65	55
ACC_05867	hypothetical protein			1	2	0	2	1	0	0	0	0	2	1	0	1	0	1	2
ACC_05868	phospholipase D2-like	K01115	KOG1329	886	468	361	323	383	43	61	64	435	163	214	124	201	201	22	6

ACC_05869	conserved hypothetical protein		KOG0907	156	155	133	211	192	18	16	34	144	71	288	407	59	83	12	10
ACC_05870	sphingosine kinase 2-like isoform 2	K04718	KOG1116	161	102	102	146	106	9	23	20	806	898	168	410	251	163	41	9
ACC_05871	39S ribosomal protein L52, mitochondrial-like			70	72	62	86	140	4	15	11	88	48	62	292	384	388	23	7
ACC_05872	UPF0533 protein C5orf44 homolog		KOG2625	256	176	198	342	480	21	27	25	132	85	93	153	149	194	7	6
ACC_05873	conserved hypothetical protein	K13211	KOG4338	658	494	527	613	613	114	172	236	1342	1241	346	283	2221	1269	583	347
ACC_05874	coatomer subunit delta isoform 2		KOG2635	431	292	254	434	297	47	90	69	692	595	254	892	1457	1962	314	157
ACC_05875	probable arginyl-tRNA synthetase, mitochondrial-li	K01887	KOG1195	110	85	80	54	104	5	10	13	54	46	50	77	154	173	24	9
ACC_05876	conserved hypothetical protein	K12822	KOG2253	1627	1468	1477	1770	1295	440	862	806	2437	1050	1320	568	1295	2152	4004	3452
ACC_05877	hypothetical protein			0	0	1	1	1	0	0	0	0	2	4	1	6	4	2	1
ACC_05878	hypothetical protein			0	0	0	0	0	0	0	0	0	0	0	0	0	0	0	0
ACC_05879	conserved hypothetical protein	K13171	KOG2146	177	151	104	140	105	42	78	83	193	166	64	64	150	231	575	485
ACC_05880	conserved hypothetical protein		KOG1703	231	103	67	270	41	45	57	65	5134	3537	181	578	669	288	390	165
ACC_05881	exostosin-1	K02366	KOG1022	310	139	119	246	169	19	27	31	484	925	68	535	309	217	69	7
ACC_05882	armadillo repeat-containing protein 4-like		KOG4224	0	0	0	0	0	1	0	1	1	3	14	30	0	5	1	0
ACC_05883	60S ribosomal protein L29-like	K02905	KOG3504	454	307	218	1570	296	36	92	42	121	89	858	332	1003	1410	547	220
ACC_05884	phosphomevalonate kinase-like	K13273		204	238	172	151	173	22	30	36	144	131	102	326	161	220	29	8
ACC_05885	mRNA-decapping enzyme 2	K12613	KOG2937	281	191	189	265	253	20	45	55	253	315	102	435	499	549	106	31
ACC_05886	putative ATPase N2B-like	K06916	KOG2383	170	154	149	248	251	5	24	14	354	198	112	351	391	317	39	8
ACC_05887	conserved hypothetical protein			8	7	17	8	7	1	4	4	6	9	2	1	2	4	9	1
ACC_05888	hypothetical protein			66	14	31	102	27	8	14	0	14	3	2	1	2	8	10	39
ACC_05889	tetratricopeptide repeat protein 1-like		KOG4234	375	252	218	603	462	80	174	181	502	213	289	564	394	491	450	412
ACC_05890	lariat debranching enzyme		KOG2863	154	110	114	196	266	15	35	28	194	113	96	287	331	294	23	11
ACC_05891	probable RNA-binding protein orb2-like		KOG0129	1446	1142	1434	971	679	259	433	657	876	699	200	103	174	369	816	401
ACC_05892	WD repeat-containing protein 24-like		KOG0269	186	112	144	193	167	14	23	36	199	107	107	110	155	205	34	8
ACC_05893	PDZ and LIM domain protein 3			128	99	63	188	64	35	57	48	1035	380	60	51	163	85	53	83
ACC_05894	ferritin	K00522	KOG2332	0	0	0	0	0	0	0	0	0	0	0	0	0	0	0	0
ACC_05895	probable tRNA (guanine(26)-N(2))-dimethyltransferase-like		KOG1253	247	115	105	212	213	22	55	68	264	197	173	245	486	772	217	122
ACC_05896	alcohol dehydrogenase		KOG1577	131	93	115	233	176	28	35	39	2422	2635	883	1952	1759	3970	387	70
ACC_05897	Cytochrome P450 4C1			46	50	40	53	39	1	4	8	58	53	38	37	11	15	3	0
ACC_05898	39S ribosomal protein L43, mitochondrial-like		KOG3445	234	231	205	317	336	25	38	61	903	309	339	805	947	1169	42	30
ACC_05899	e3 ubiquitin-protein ligase KCMF1-like		KOG1280	220	190	152	78	46	12	21	17	561	661	27	335	302	319	79	23
ACC_05900	fragile X mental retardation syndrome-related prot	K15516		445	292	280	343	174	18	27	35	513	529	130	309	761	1032	302	93
ACC_05901	NADH dehydrogenase	K03941	KOG3256	660	393	435	1480	1492	38	68	130	1779	443	779	1704	624	985	86	68
ACC_05902	metaxin-1-like		KOG3028	285	221	237	403	397	9	23	27	169	166	127	237	400	602	73	22
ACC_05903	monocyte to macrophage differentiation factor 2-li	K11064	KOG4243	174	92	89	121	156	4	23	21	285	282	51	447	196	156	32	8
ACC_05904	slit homolog 2 protein		KOG4194	63	30	27	68	9	4	5	14	24	43	22	4	7	2	61	69
ACC_05905	ATP synthase subunit s, mitochondrial-like	K07554	KOG3864	42	40	34	45	71	4	10	9	43	23	45	69	39	54	8	3
ACC_05906	conserved hypothetical protein		KOG4221	250	92	102	149	24	1	5	8	92	77	101	40	3	3	5	5
ACC_05907	conserved hypothetical protein		KOG0161	1353	1240	1559	2815	1321	220	253	353	3477	2127	1240	1632	1232	1092	497	225
ACC_05908	LOW QUALITY PROTEIN	K12275	KOG2927	3114	1347	889	2069	2539	842	1633	1917	1919	1495	1358	2180	2094	2151	2027	1933
ACC_05909	methyltransferase WBSCR22-like		KOG1541	386	334	344	817	684	75	169	149	516	271	360	461	424	708	413	154
ACC_05910	probable O-sialoglycoprotein endopeptidase	K15900	KOG2708	568	365	417	867	945	51	93	118	817	290	443	789	363	629	84	36
ACC_05911	tRNA dimethylallyltransferase, mitochondrial-like,	K00791	KOG1384	119	145	153	235	132	47	150	165	155	64	68	29	123	255	316	288
ACC_05912	phosphatidate cytidylyltransferase, photoreceptor-	K00981	KOG1440	675	375	350	349	598	13	23	31	814	553	150	524	212	133	7	6
ACC_05913	conserved hypothetical protein			80	42	42	61	74	2	1	5	208	174	32	240	395	175	17	4
ACC_05914	dehydrogenase/reductase SDR family member 4-lik	K11147	KOG0725	42	28	36	39	44	4	6	9	204	83	259	2978	369	178	1	4
ACC_05915	kinesin-associated protein 3		KOG1222	1013	516	469	1319	1744	52	139	173	1021	840	625	997	858	774	198	97
ACC_05916	proliferating cell nuclear antigen	K04802	KOG1636	156	106	71	225	223	15	29	33	248	147	143	330	785	1177	208	137
ACC_05917	conserved hypothetical protein	K16675	KOG1311	306	114	118	185	164	9	14	27	513	285	120	423	380	315	24	5
ACC_05918	signal sequence receptor beta		KOG0682	3	1	3	1	3	2	1	0	0	4	0	7	27	9	1	0
ACC_05919	conserved hypothetical protein		KOG1028	330	179	185	397	221	80	128	105	427	238	233	655	302	475	249	91
ACC_05920	conserved hypothetical protein		KOG4047	72	60	57	55	37	3	6	8	118	108	19	56	67	72	19	9
ACC_05921	ribosomal RNA processing protein 1 homolog	K14849	KOG3911	897	560	459	519	778	104	357	345	615	493	687	758	1443	2070	1334	768
ACC_05922	glucose-6-phosphate isomerase-like	K01810	KOG2446	1673	1045	982	1428	1588	120	214	236	3224	1831	731	1850	8953	5385	508	253
ACC_05923	potassium voltage-gated channel subfamily KQT member 1-like			11	5	3	3	1	0	3	3	6	2	12	5	0	0	0	1
ACC_05924	protein UBASH3A homolog		KOG3734	211	131	137	167	157	13	14	22	284	156	104	277	82	80	13	6
ACC_05925	LOW QUALITY PROTEIN	K07192	KOG2668	256	89	101	142	52	13	15	12	271	132	64	207	68	63	52	41

ACC_05926	autophagy-related protein 13 homolog		KOG3874	292	160	194	426	288	22	26	34	328	221	106	434	214	162	22	8
ACC_05927	ubiquitin carboxyl-terminal hydrolase isozyme L5	K05610	KOG2778	558	353	323	669	687	85	132	195	1096	646	478	1136	1702	2378	415	248
ACC_05928	alpha-tocopherol transfer protein-like		KOG1471	57	50	28	99	86	35	48	40	807	493	1592	11828	241	281	40	108
ACC_05929	alpha-tocopherol transfer protein-like		KOG1471	627	461	511	1501	1325	28	39	53	2079	513	14283	44140	797	86	1	0
ACC_05930	conserved hypothetical protein			7	0	2	3	3	1	0	1	21	4	64	64	213	194	23	22
ACC_05931	cleavage and polyadenylation specificity factor sub	K14403	KOG1137	131	95	102	141	154	16	16	19	177	118	64	158	273	384	28	13
ACC_05932	RNA polymerase II transcription elongation factor DSIF/SUPT		KOG1999	289	140	142	179	87	33	51	46	207	154	39	20	62	78	489	626
ACC_05933	conserved hypothetical protein		KOG3885	15	1	3	11	1	1	0	0	6	19	0	1	9	6	22	40
ACC_05934	ATP synthase subunit s-like protein-like		KOG3864	109	98	93	93	125	1	12	15	62	57	40	87	142	195	24	16
ACC_05935	integral membrane protein 2C-like		KOG4681	2255	837	841	2295	2906	69	70	129	3108	1354	427	2017	601	499	24	5
ACC_05936	LOW QUALITY PROTEIN	K08745	KOG1179	718	473	507	560	459	109	151	208	1131	585	578	579	1150	122	30	10
ACC_05937	centrosomin-like	K16718	KOG0161	2688	1799	1967	3869	1627	412	727	859	12148	2838	325	309	429	501	892	730
ACC_05938	cyclin-dependent kinase-like 1-like	K08824	KOG0593	144	113	148	232	168	4	18	22	188	135	403	463	233	562	193	55
ACC_05939	tetratricopeptide repeat protein 30A-like, partial		KOG4340	138	110	100	117	182	10	20	18	83	60	117	239	102	80	12	4
ACC_05940	conserved hypothetical protein			1307	503	372	1088	798	0	2	9	2302	336	32	42	26	33	0	3
ACC_05941	probable S-adenosyl-L-methionine-dependent methyltransfe		KOG2782	443	344	311	621	523	24	48	57	626	449	283	671	980	1153	154	27
ACC_05942	hypothetical protein			0	0	0	0	0	0	0	0	0	0	0	0	0	0	1	1
ACC_05943	conserved hypothetical protein			0	0	0	1	0	0	0	0	0	0	0	0	0	2	0	0
ACC_05944	conserved hypothetical protein			528	583	805	2706	1095	176	345	534	6273	4039	669	1397	1168	404	329	166
ACC_05945	voltage-dependent calcium channel type D subunit alpha-1-l		KOG2301	102	64	73	170	78	1	7	5	77	103	16	1	5	6	8	12
ACC_05946	conserved hypothetical protein		KOG3779	1169	804	1049	2437	1300	126	292	322	354	161	99	16	14	28	239	147
ACC_05947	doublesex isoform 1		KOG3815	17	34	26	14	5	13	12	26	177	382	28	29	11	2	5	1
ACC_05948	protein UXT homolog		KOG3047	24	21	20	72	83	2	2	4	19	9	40	60	77	98	6	5
ACC_05949	cysteine sulfinic acid decarboxylase-like isoform 2		KOG0629	1438	827	1805	2119	1714	92	172	158	1251	254	14	9	49	47	12	10
ACC_05950	elongation of very long chain fatty acids protein 4-like, partia		KOG3071	82	41	56	112	81	2	3	7	51	28	2	27	4	3	1	0
ACC_05951	hypothetical protein			22	16	31	39	27	1	6	5	14	11	13	1	1	0	6	9
ACC_05952	conserved hypothetical protein			90	24	23	58	13	5	5	7	24	17	1	4	4	4	24	3
ACC_05953	lysosomal thioesterase PPT2 homolog isoform 2	K01074	KOG2541	67	49	36	103	83	6	9	11	98	49	45	70	128	107	24	6
ACC_05954	conserved hypothetical protein			360	123	129	238	292	34	71	93	176	131	114	141	210	252	162	88
ACC_05955	cyclin-H	K06634	KOG2496	101	89	77	163	183	12	10	12	77	33	89	48	105	169	46	13
ACC_05956	hypothetical protein			0	0	0	0	0	0	0	0	0	0	0	0	0	0	0	0
ACC_05957	G-protein-signaling modulator 2	K15837	KOG1130	177	108	73	136	67	33	37	50	361	244	84	252	285	325	149	62
ACC_05958	calpain-7-like protein-like		KOG0045	3	1	2	4	1	3	4	2	3	36	0	0	2	1	9	13
ACC_05959	fibrillin-2-like		KOG1219	45	4	1	10	2	0	4	1	124	44	5	34	29	9	20	23
ACC_05960	conserved hypothetical protein		KOG4193	206	182	82	115	44	69	74	137	117	134	26	60	20	16	13	0
ACC_05961	DEAD-box helicase Dbp80		KOG0332	313	214	223	442	425	12	34	36	480	344	272	595	883	1087	108	66
ACC_05962	INO80 complex subunit C-like	K11667	KOG4137	162	117	139	395	305	14	29	51	156	100	141	318	288	331	39	36
ACC_05963	g2/mitotic-specific cyclin-B3	K05868	KOG0653	144	87	75	139	100	17	29	26	153	115	39	70	215	275	149	102
ACC_05964	odorant receptor 98			4	4	3	12	12	4	3	5	17	14	451	99	5	0	0	1
ACC_05965	SHC-transforming protein 1-like	K06279	KOG3697	174	156	107	112	119	15	30	35	501	416	86	483	566	493	47	23
ACC_05966	protein FRG1-like	K13122	KOG3962	242	213	199	340	309	60	90	91	308	137	285	341	180	243	131	140
ACC_05967	exportin-4-like isoform 2		KOG4541	137	90	108	188	234	11	18	20	151	85	89	44	111	167	10	3
ACC_05968	LOW QUALITY PROTEIN	K04372	KOG0607	912	846	502	1726	934	585	705	839	4259	3425	607	896	1199	661	807	186
ACC_05969	s-phase kinase-associated protein 2	K03875	KOG2120	71	61	67	123	126	7	11	7	99	55	89	31	232	585	90	30
ACC_05970	pre-mRNA-processing factor 40 homolog A-like	K12821	KOG0152	743	526	486	780	633	141	326	354	613	357	411	297	636	1013	1506	783
ACC_05971	conserved hypothetical protein	K08815	KOG1164	1073	628	509	616	327	44	52	67	1143	1003	159	289	399	431	112	32
ACC_05972	choline/ethanolamine kinase-like	K14156	KOG2686	1093	655	657	998	764	100	209	277	1964	1437	664	660	1416	1416	402	177
ACC_05973	protein trachealess-like isoform 1	K09098	KOG3558	128	58	62	92	26	12	24	19	245	176	57	28	134	301	355	149
ACC_05974	ADP-ribosylation factor-related protein 1-like	K07952	KOG0076	63	58	84	208	164	5	14	14	67	43	81	130	72	108	26	7
ACC_05975	BTB/POZ domain-containing protein 3-like		KOG2075	325	137	134	266	172	32	22	33	441	266	86	274	196	212	28	2
ACC_05976	LOW QUALITY PROTEIN	K08059	KOG3160	445	421	319	280	297	61	154	166	443	262	266	1058	630	505	61	15
ACC_05977	conserved hypothetical protein		KOG3206	235	148	167	390	74	34	22	61	438	435	322	153	7	6	13	8
ACC_05978	hypothetical protein			0	0	0	0	0	0	0	0	0	0	0	0	0	0	0	0
ACC_05979	hemK methyltransferase family member 2-like		KOG3191	135	105	127	191	140	39	72	85	216	139	157	142	206	171	63	43
ACC_05980	dnaJ homolog subfamily C member 22-like		KOG0715	634	1002	535	123	110	480	437	869	89	71	25	23	691	679	316	102
ACC_05981	BTB/POZ domain-containing protein KCTD9-like		KOG1665	120	40	54	100	135	3	2	8	125	64	61	112	71	48	5	2
ACC_05982	octopamine receptor beta-2R isoform 5		KOG4220	70	45	48	56	10	1	0	8	157	103	15	20	3	0	4	3

ACC_05983	conserved hypothetical protein			1	2	1	0	0	0	1	0	1	1	0	2	0	0	0	1
ACC_05984	CCR4-NOT transcription complex subunit 7-like	K12581	KOG0304	185	115	95	352	241	17	34	32	321	201	237	409	461	731	143	62
ACC_05985	extracellular domains-containing protein CG31004-like		KOG4291	8	11	9	20	7	2	6	2	25	26	15	18	775	1499	107	6
ACC_05986	extracellular domains-containing protein CG31004-like			0	4	6	1	6	0	0	0	0	3	2	1	25	43	10	1
ACC_05987	glutamate receptor delta-2 subunit-like		KOG1052	76	124	99	21	33	32	61	50	241	128	18	79	89	38	11	2
ACC_05988	protein rogdi		KOG3992	246	124	111	250	271	6	20	22	343	268	101	351	391	343	28	12
ACC_05989	sialin-like		KOG2532	505	287	350	524	385	28	35	38	899	452	191	450	244	243	37	6
ACC_05990	LOW QUALITY PROTEIN	K14855	KOG0271	256	212	206	339	316	22	39	43	375	264	175	454	457	569	83	23
ACC_05991	teneurin-3-like isoform 1		KOG4659	3332	1384	1370	2479	842	166	232	369	1570	867	116	192	23	112	291	95
ACC_05992	GL12416	K07374	KOG1376	1735	1535	1368	6583	3816	264	324	512	2746	1714	1683	3485	6838	11367	4721	1699
ACC_05993	UMP-CMP kinase-like	K13800	KOG3079	253	221	149	352	359	36	49	56	511	301	464	771	650	608	28	16
ACC_05994	conserved hypothetical protein	K09071	KOG4029	0	1	1	1	0	2	1	0	2	10	1	2	16	23	40	5
ACC_05995	putative N-acetylglucosamine-6-phosphate deacety	K01443	KOG3892	187	95	95	305	242	15	13	22	312	294	99	372	781	710	52	13
ACC_05996	lon protease homolog, mitochondrial-like	K08675	KOG2004	911	453	346	567	561	145	287	327	1280	1482	397	687	2128	2294	890	552
ACC_05997	protein SMG5-like	K11125	KOG2162	470	272	290	605	568	49	93	122	805	593	230	356	547	778	218	107
ACC_05998	conserved hypothetical protein			34	16	21	42	10	1	2	1	14	14	5	11	4	4	5	1
ACC_05999	conserved hypothetical protein			2026	1295	1558	3416	2101	105	164	142	1307	162	1627	2282	797	934	101	80
ACC_06000	glucosylceramidase-like	K01201	KOG2566	372	1441	2530	418	509	1246	945	1043	2213	472	114	19	15142	181	5	1
ACC_06001	serine/threonine-protein kinase tricorner	K08790	KOG0605	731	325	394	973	425	182	285	374	1500	873	305	195	149	242	367	208
ACC_06002	conserved hypothetical protein			5181	3759	5184	24995	3651	907	1357	2118	3467	990	3439	6692	149	77	387	2290
ACC_06003	LOW QUALITY PROTEIN		KOG0629	1463	523	507	1564	898	100	117	153	1081	843	178	393	1606	975	311	75
ACC_06004	conserved hypothetical protein			58	114	128	490	249	27	55	45	416	503	252	265	36	18	2	2
ACC_06005	coiled-coil domain-containing protein 111-like			156	102	88	150	156	6	32	45	281	179	146	324	273	288	53	11
ACC_06006	glucose dehydrogenase		KOG1238	174	145	156	177	192	13	13	13	221	182	88	266	231	240	14	4
ACC_06007	probable methylthioribulose-1-phosphate dehydra	K08964	KOG2631	240	228	178	387	292	60	94	134	314	241	233	687	741	767	219	154
ACC_06008	prefoldin subunit 6-like	K04798	KOG3478	279	257	264	287	272	40	60	76	250	127	255	579	608	781	116	88
ACC_06009	tubulin alpha chain-like		KOG1376	17	21	15	40	20	0	1	3	22	14	48	66	92	48	17	15
ACC_06010	dynactin subunit 1-like	K04648	KOG0971	1784	1131	992	1850	1317	358	665	740	2137	1614	1003	970	1242	1567	1014	524
ACC_06011	vesicular integral-membrane protein VIP36-like	K10082	KOG3839	278	189	148	398	394	21	30	36	601	444	129	469	972	698	71	25
ACC_06012	conserved hypothetical protein	K13136		262	154	148	301	301	76	164	192	209	161	204	221	458	593	507	404
ACC_06013	LOW QUALITY PROTEIN	K16507	KOG1219	593	296	378	806	296	76	148	171	445	404	255	69	34	72	364	257
ACC_06014	conserved hypothetical protein		KOG4790	445	331	313	605	375	41	65	75	973	554	107	312	564	493	281	73
ACC_06015	conserved hypothetical protein	K10779	KOG1015	2698	1559	1321	1914	2815	292	825	891	1380	1622	1121	781	922	914	941	464
ACC_06016	conserved hypothetical protein			58	32	32	54	61	4	8	6	79	43	24	25	62	99	12	2
ACC_06017	OTU domain-containing protein 5-A-like	K12655	KOG0589	295	193	191	274	179	35	34	38	628	364	133	183	301	358	69	32
ACC_06018	PR domain zinc finger protein 10-like		KOG2462	442	218	169	252	238	46	77	110	349	232	121	120	215	144	95	27
ACC_06019	dynein intermediate chain 2, ciliary-like isoform 2	K10409	KOG1587	163	122	131	184	176	15	24	34	79	56	115	128	26	23	17	7
ACC_06020	kinectin	K14000	KOG0161	10094	5771	4268	5151	5515	1772	3250	3995	6503	4872	4006	3383	7757	6496	5967	4165
ACC_06021	DC-STAMP domain-containing protein 1-like		KOG3726	1119	1278	609	2854	1395	359	474	501	5154	2885	5101	10525	2025	4255	1567	2354
ACC_06022	hypothetical protein			0	0	0	0	0	0	0	0	0	0	0	0	0	0	1	0
ACC_06023	echinoderm microtubule-associated protein-like 1-like			85	36	43	90	19	11	14	22	172	167	23	115	91	44	54	13
ACC_06024	protein bowel-like	K09215	KOG2462	6	41	11	13	0	9	6	3	80	54	30	69	47	47	58	20
ACC_06025	vesicle transport through interaction with t-SNARE:	K08493	KOG1666	231	188	177	254	153	38	50	49	322	129	105	344	160	107	54	48
ACC_06026	kinesin 6B		KOG0247	629	420	311	507	510	31	107	99	177	161	85	45	262	386	184	117
ACC_06027	UPF0586 protein C9orf41 homolog		KOG2798	123	91	70	140	140	11	11	12	246	181	107	288	201	281	22	6
ACC_06028	proteasome subunit beta type-4-like	K02736	KOG0185	341	306	236	738	662	78	90	176	1056	589	994	2111	2062	2176	208	136
ACC_06029	synaptobrevin-like isoform 1		KOG0860	372	278	165	489	343	174	267	336	748	293	428	766	514	659	276	174
ACC_06030	cullin-2-like	K03870	KOG2284	517	501	459	751	463	97	141	160	1458	1034	383	648	1219	1252	299	63
ACC_06031	hexosaminidase D-like		KOG2499	127	99	93	99	99	5	10	17	164	89	55	99	36	35	7	1
ACC_06032	conserved hypothetical protein			126	51	59	210	74	7	10	15	92	64	52	146	5	10	61	14
ACC_06033	conserved hypothetical protein			656	310	302	550	565	96	248	228	341	190	315	191	470	1140	2062	1592
ACC_06034	protein suppressor of forked-like	K14408	KOG1914	133	70	71	210	184	13	25	18	143	79	81	117	141	215	41	6
ACC_06035	sodium-independent sulfate anion transporter-like	K14708	KOG0236	219	154	114	229	199	34	42	70	256	140	155	310	158	177	19	7
ACC_06036	LOW QUALITY PROTEIN		KOG3596	1450	1041	736	842	445	146	209	240	2450	2310	459	369	502	347	189	65
ACC_06037	LOW QUALITY PROTEIN		KOG4583	327	242	263	234	154	30	22	39	844	508	143	777	650	486	19	7
ACC_06038	charged multivesicular body protein 4b-like	K12194	KOG1656	540	326	344	655	586	79	143	208	791	456	304	1131	490	620	121	87
ACC_06039	conserved hypothetical protein			38	33	53	67	72	1	5	2	15	19	28	11	3	12	1	1

ACC_06040	GDP-fucose protein O-fucosyltransferase 2	K03691		223	147	179	222	314	16	33	32	147	132	117	129	213	185	32	7
ACC_06041	conserved hypothetical protein			228	141	109	371	297	29	33	54	483	400	145	305	174	161	63	18
ACC_06042	cyclic AMP-dependent transcription factor ATF-2-lil	K04450	KOG1414	308	219	198	302	230	44	85	87	226	162	123	212	284	286	201	100
ACC_06043	origin recognition complex subunit 4	K02606	KOG2228	95	50	50	84	84	3	8	12	130	76	45	83	142	170	17	1
ACC_06044	tRNA-specific adenosine deaminase-like protein 3-l	K15442	KOG2771	104	88	92	144	81	22	32	66	127	124	33	91	99	123	102	51
ACC_06045	conserved hypothetical protein	K02089	KOG0594	230	124	156	949	520	30	24	56	518	264	409	719	544	776	195	79
ACC_06046	protein CDV3 homolog A-like			278	204	174	201	91	51	85	102	287	232	44	156	704	705	1633	1636
ACC_06047	conserved hypothetical protein		KOG4377	252	145	169	223	31	4	9	15	254	186	5	3	32	85	211	135
ACC_06048	fasciclin-3-like isoform 1		KOG3515	182	85	81	182	36	20	23	38	225	117	219	223	29	61	571	413
ACC_06049	scavenger receptor class B member 1		KOG3776	12	3	2	11	1	11	13	10	1225	610	52	138	403	141	40	25
ACC_06050	mitochondrial inner membrane protein OXA1L-like	K03217	KOG1239	742	406	460	781	660	42	113	123	1028	595	493	1430	2321	2441	262	120
ACC_06051	HEAT repeat-containing protein 1	K14550	KOG1837	506	322	324	505	317	40	52	69	601	618	165	263	935	1332	252	38
ACC_06052	potassium voltage-gated channel subfamily KQT m	K04926	KOG1419	522	216	218	311	70	92	98	196	222	174	427	37	4	3	22	12
ACC_06053	hypothetical protein			2	0	0	1	2	0	0	0	1	2	2	0	0	1	0	1
ACC_06054	conserved hypothetical protein		KOG3212	114	121	100	128	169	4	15	12	51	70	137	212	171	214	20	11
ACC_06055	mRNA cap guanine-N7 methyltransferase-like	K00565	KOG1975	522	292	300	541	638	32	90	91	624	407	360	818	766	966	145	56
ACC_06056	transmembrane 9 superfamily member 4-like isoform 1		KOG1278	566	308	286	569	519	42	45	61	1043	721	235	609	709	705	46	23
ACC_06057	trifunctional enzyme subunit alpha, mitochondrial-	K07515	KOG1683	711	339	225	523	570	136	249	278	978	1012	445	1038	5897	4711	883	476
ACC_06058	transcription elongation factor SPT6-like	K11292	KOG1856	1041	617	601	823	803	195	437	512	1009	749	380	340	944	1152	1875	1870
ACC_06059	conserved hypothetical protein		KOG2123	360	217	182	242	268	36	26	33	562	445	513	1343	361	323	38	11
ACC_06060	solute carrier family 12 member 9-like	K14429	KOG1288	306	277	258	222	256	19	36	21	1214	507	232	467	620	492	34	2
ACC_06061	facilitated trehalose transporter Tret1-2 homolog isoform 1		KOG0254	120	61	23	117	95	22	12	21	533	520	101	234	733	771	112	21
ACC_06062	slit homolog 1 protein-like		KOG4194	83	60	65	35	12	6	9	9	80	108	9	45	11	15	18	2
ACC_06063	endocuticle structural glycoprotein SgAbd-2-like			0	0	1	1	0	0	0	0	1	1	0	1	4	0	2	1
ACC_06064	endocuticle structural glycoprotein SgAbd-1-like			0	0	0	1	0	0	0	0	5	0	0	0	1134	326	508	443
ACC_06065	thioredoxin-like protein 1-like		KOG0908	387	284	273	571	471	68	58	91	1143	788	615	1830	1472	1697	243	122
ACC_06066	H(+)/Cl(-) exchange transporter 7	K05016	KOG0474	567	333	418	615	575	57	133	134	1465	870	605	611	489	525	74	17
ACC_06067	conserved hypothetical protein			1047	513	518	376	189	36	45	50	1796	870	109	311	197	178	25	11
ACC_06068	GF18028		KOG1595	277	163	151	451	143	89	136	152	433	525	180	147	129	80	546	327
ACC_06069	similar to Histone H3.3B CG8989-PA isoform 2	K11253	KOG1745	327	146	178	278	204	15	20	30	210	116	104	436	497	453	94	27
ACC_06070	serine/threonine-protein phosphatase PP1-beta ca	K06269	KOG0374	2057	1150	1196	2956	1252	328	501	636	1865	862	455	412	183	149	206	85
ACC_06071	sodium/potassium/calcium exchanger Nckx30C-like	K13750	KOG1307	1972	588	742	1772	373	25	58	73	336	273	61	8	1	1	8	10
ACC_06072	conserved hypothetical protein		KOG1585	541	262	330	726	570	23	24	38	563	366	202	451	402	352	51	15
ACC_06073	conserved hypothetical protein	K08498	KOG3202	548	376	386	900	635	34	46	59	405	202	233	258	97	94	17	15
ACC_06074	28S ribosomal protein S36, mitochondrial-like			171	156	195	453	216	25	31	55	138	75	94	213	188	282	102	59
ACC_06075	conserved hypothetical protein			1404	958	988	657	512	177	250	371	1450	666	272	258	178	102	81	82
ACC_06076	conserved hypothetical protein		KOG4735	137	96	109	174	53	27	44	36	382	183	91	241	101	177	225	32
ACC_06077	conserved hypothetical protein			100	47	35	37	15	1	2	2	16	31	2	2	3	1	1	0
ACC_06078	cytosolic endo-beta-N-acetylglucosaminidase-like	K01227	KOG2331	257	173	132	181	186	37	44	56	623	511	132	450	449	282	24	9
ACC_06079	nuclear pore complex protein Nup153-like	K14296	KOG3091	807	545	476	730	428	117	250	296	886	1201	259	288	600	626	657	249
ACC_06080	conserved hypothetical protein			179	96	85	243	189	7	21	22	121	80	124	322	196	142	38	26
ACC_06081	conserved hypothetical protein		KOG1103	297	167	186	262	175	41	99	118	211	156	105	70	113	120	369	234
ACC_06082	conserved hypothetical protein			133	105	142	422	294	83	115	102	459	231	32	58	88	334	426	66
ACC_06083	tRNA guanosine-2'-O-methyltransferase TRM11 ho	K15430	KOG2671	155	90	96	174	214	9	14	14	127	124	75	180	186	265	29	11
ACC_06084	sorting nexin-6-like		KOG1660	530	380	292	501	416	54	87	81	758	637	306	1205	1216	1280	189	114
ACC_06085	hypothetical protein			2	2	2	2	1	0	0	0	5	1	1	1	1	5	6	4
ACC_06086	3-hydroxyisobutyrate dehydrogenase, mitochondri	K00020	KOG0409	24	9	12	37	28	1	3	2	61	126	70	232	248	213	30	15
ACC_06087	probable cytosolic iron-sulfur protein assembly protein Ciao		KOG0645	104	84	78	188	147	7	22	23	101	72	54	94	361	519	66	36
ACC_06088	ATPase WRNIP1-like isoform 1		KOG2028	277	138	136	313	299	36	38	80	460	248	219	374	280	309	48	31
ACC_06089	conserved hypothetical protein		KOG4225	480	477	419	477	343	128	214	240	1166	861	353	578	551	428	216	132
ACC_06090	proteasome subunit alpha type-1-like	K02725	KOG0863	311	233	238	369	341	50	58	70	810	433	582	1269	1683	1844	166	63
ACC_06091	LOW QUALITY PROTEIN		KOG2267	59	41	33	57	67	9	21	16	108	92	110	464	229	183	16	4
ACC_06092	conserved hypothetical protein			238	251	232	166	247	0	3	3	16	77	57	328	105	46	9	4
ACC_06093	ubiquitin-like-conjugating enzyme ATG3-like	K08343	KOG2981	543	340	338	628	525	42	63	87	385	239	203	482	570	599	122	69
ACC_06094	conserved hypothetical protein	K03962	KOG4633	464	367	392	739	687	41	97	131	962	260	1131	1783	652	797	79	44
ACC_06095	HIG1 domain family member 1C-like		KOG4431	43	24	31	41	55	0	1	1	95	24	48	81	121	126	5	5
ACC_06096	LOW QUALITY PROTEIN	K10086	KOG2430	988	775	508	390	394	82	170	194	1242	1613	224	657	1199	702	137	47

ACC_06097	u6 snRNA-associated Sm-like protein LSm1-like	K12620	KOG1782	106	100	70	136	134	6	8	13	118	63	54	263	242	270	28	5
ACC_06098	LOW QUALITY PROTEIN	K14938		0	3	0	1	0	0	0	0	1	1	1	7	2	36	18	18
ACC_06099	histone H4 transcription factor-like		KOG3608	328	292	306	361	357	43	99	99	468	199	197	152	213	221	75	24
ACC_06100	mitochondrial glutamate carrier 1-like	K15107	KOG0750	578	230	190	483	436	39	58	77	597	346	175	462	392	300	62	22
ACC_06101	conserved hypothetical protein		KOG1074	969	591	476	619	761	222	468	479	1148	956	465	330	662	767	1637	1065
ACC_06102	growth arrest-specific protein 1-like	K06232		26	20	19	51	40	2	3	6	21	11	5	10	16	29	4	9
ACC_06103	golgin subfamily A member 2-like		KOG0161	1446	867	733	943	1330	245	540	646	1284	940	1164	973	1306	1345	687	407
ACC_06104	conserved hypothetical protein			2	6	2	5	1	0	0	1	4	8	23	29	12	0	0	0
ACC_06105	conserved hypothetical protein		KOG2726	393	297	333	672	654	40	110	162	426	323	436	327	416	420	181	85
ACC_06106	transmembrane emp24 domain-containing protein 5		KOG3287	154	153	81	240	208	22	40	61	240	172	93	231	785	670	58	10
ACC_06107	conserved hypothetical protein	K14997	KOG1305	593	297	316	532	433	50	94	131	597	590	189	250	436	494	180	86
ACC_06108	RNA polymerase II-associated protein 3		KOG4648	515	356	326	384	561	88	218	253	418	270	323	333	531	790	674	499
ACC_06109	conserved hypothetical protein			459	304	281	476	252	44	44	64	437	281	245	270	199	194	84	19
ACC_06110	sarcoplasmic calcium-binding protein 1-like			150	87	78	260	205	94	142	153	711	367	358	332	169	224	112	53
ACC_06111	zinc finger protein 512B		KOG0307	2	0	0	2	2	0	0	0	11	0	17	17	14	27	3	8
ACC_06112	conserved hypothetical protein		KOG2676	136	89	89	65	103	3	13	12	135	112	176	111	168	99	12	3
ACC_06113	conserved hypothetical protein			119	79	70	212	96	15	18	16	263	196	114	325	92	121	72	21
ACC_06114	tubulin-specific chaperone cofactor E-like protein-like		KOG2982	595	259	269	586	207	222	209	319	1379	791	291	390	79	77	77	7
ACC_06115	conserved hypothetical protein			985	426	455	579	103	24	27	46	678	669	52	236	29	25	31	25
ACC_06116	zinc finger protein 26-like	K09228	KOG2462	166	163	140	193	174	24	42	47	263	209	102	189	172	187	69	20
ACC_06117	ADP-dependent glucokinase-like		KOG4184	501	226	215	492	440	15	14	24	753	812	243	964	1221	1106	53	16
ACC_06118	nuclear factor related to kappa-B-binding protein	K11671	KOG1927	1842	1595	2000	2136	786	327	427	555	2083	1351	745	725	931	1138	1098	490
ACC_06119	zinc finger protein 706-like isoform 8		KOG4118	424	214	152	467	230	25	36	66	178	111	104	552	432	579	296	147
ACC_06120	glycine N-methyltransferase-like	K00552	KOG1270	24	23	27	106	108	8	6	11	518	1358	13	4	1170	220	26	58
ACC_06121	probable 39S ribosomal protein L49, mitochondrial-like		KOG4034	241	238	216	495	407	16	32	50	180	138	124	455	396	386	145	61
ACC_06122	splicing factor 3B subunit 3 isoform 1	K12830	KOG1898	1340	630	464	660	487	62	96	104	1333	852	359	304	2596	3887	638	154
ACC_06123	conserved hypothetical protein		KOG0921	1293	917	951	744	476	76	166	200	1578	717	558	404	1156	1689	1075	620
ACC_06124	conserved hypothetical protein		KOG0621	109	48	50	122	111	14	11	14	409	95	78	236	188	235	75	21
ACC_06125	UDP-N-acetylhexosamine pyrophosphorylase	K00972	KOG2388	334	239	148	278	289	39	72	57	395	423	154	474	1774	1707	171	57
ACC_06126	conserved hypothetical protein	K11653	KOG2510	2613	2163	1896	2266	1049	513	770	977	3111	2542	867	354	995	1225	2504	1256
ACC_06127	cullin-3-like	K03869	KOG2166	704	461	398	654	603	79	119	134	1291	821	324	475	919	833	181	72
ACC_06128	serine/threonine-protein kinase mTOR isoform 1	K07203	KOG0891	591	398	370	617	484	61	77	103	839	805	157	182	589	771	156	46
ACC_06129	myeloid leukemia factor		KOG4049	521	293	350	678	508	42	47	69	521	288	213	616	460	596	172	133
ACC_06130	nuclear receptor-binding protein homolog	K08875	KOG1266	329	170	174	398	248	53	95	146	333	251	136	159	219	182	137	63
ACC_06131	zinc finger protein 808-like		KOG2462	500	283	259	243	310	33	73	61	264	263	116	212	285	278	117	57
ACC_06132	histone deacetylase 3-like	K11404	KOG1342	275	253	263	560	468	34	55	64	345	180	285	342	252	316	73	26
ACC_06133	conserved hypothetical protein			290	364	495	456	322	28	48	54	124	77	96	189	72	65	15	10
ACC_06134	cell growth-regulating nucleolar protein-like	K15263	KOG2186	455	222	170	192	219	39	148	178	291	171	166	160	857	1515	1131	1051
ACC_06135	GPI ethanolamine phosphate transferase 3-like	K05288	KOG2126	769	543	457	885	851	42	48	75	429	473	205	271	696	689	46	9
ACC_06136	conserved hypothetical protein			39	26	39	22	34	4	2	4	21	36	11	9	47	30	3	1
ACC_06137	histone-arginine methyltransferase CARMER-like is	K05931	KOG1500	97	82	64	106	66	10	9	16	93	179	14	26	123	167	44	13
ACC_06138	DNA polymerase delta subunit 3-like	K03504		490	229	188	565	614	29	69	107	185	197	220	245	249	252	168	92
ACC_06139	slit homolog 1 protein-like		KOG4194	107	53	58	93	100	1	5	9	51	21	33	63	169	168	11	3
ACC_06140	excitatory amino acid transporter 1-like		KOG3787	3192	1228	1160	2470	1928	419	922	819	1939	1814	969	745	454	345	1020	839
ACC_06141	SCO-spondin-like		KOG1480	117	80	108	67	95	7	20	11	87	89	214	106	66	65	18	7
ACC_06142	ATP-binding cassette sub-family G member 1-like		KOG0061	505	549	294	475	356	116	102	91	1583	916	894	1477	673	555	43	9
ACC_06143	LOW QUALITY PROTEIN	K01008	KOG3939	814	683	737	1413	956	68	82	117	1387	1387	359	1267	4179	5934	1861	352
ACC_06144	hepatocyte nuclear factor 6-like		KOG2252	420	293	388	565	83	14	11	25	132	274	77	30	1	5	49	42
ACC_06145	chitinase domain-containing protein 1-like		KOG2091	448	275	197	406	371	55	79	109	212	175	170	484	829	681	95	36
ACC_06146	conserved hypothetical protein	K13098	KOG1995	3653	1752	1883	1127	927	499	774	849	5222	2739	1088	2048	3006	5403	1993	1509
ACC_06147	conserved hypothetical protein		KOG2549	244	158	159	215	209	24	38	37	192	165	130	182	291	315	35	15
ACC_06148	conserved hypothetical protein		KOG1311	60	32	35	34	12	5	3	1	90	126	10	30	102	105	25	4
ACC_06149	nuclear pore complex protein Nup93-like	K14309	KOG2168	72	48	54	218	154	8	6	11	174	106	59	123	1088	1616	179	52
ACC_06150	conserved hypothetical protein			86	24	35	48	6	1	0	0	12	24	23	37	0	0	1	0
ACC_06151	transmembrane protein 18-like			217	159	104	178	177	63	60	97	404	167	529	1540	276	158	18	7
ACC_06152	matrix metalloproteinase-25-like		KOG1565	39	13	15	59	20	2	11	5	24	32	11	13	26	20	52	33
ACC_06153	matrix metalloproteinase-15-like		KOG1565	702	446	508	713	291	63	88	82	895	384	143	181	369	388	277	98

ACC_06154	matrix metalloproteinase-24-like			1	0	0	0	2	0	0	0	7	5	0	0	75	41	3	2
ACC_06155	c-factor		KOG1611	127	101	96	142	100	12	13	8	361	248	189	372	363	271	36	26
ACC_06156	conserved hypothetical protein	K01090	KOG0698	4131	3861	4746	4365	2388	623	1043	1110	8584	5340	955	1755	3342	3085	3891	2657
ACC_06157	sodium-dependent dopamine transporter-like	K05036	KOG3659	55	21	17	36	12	2	6	3	346	342	34	97	4	8	3	6
ACC_06158	homologous-pairing protein 2 homolog	K06695	KOG4603	125	70	65	57	69	24	58	62	83	43	85	156	89	110	93	47
ACC_06159	hypothetical protein			1	0	2	4	1	3	2	2	3	2	1	0	0	0	0	1
ACC_06160	intraflagellar transport protein 52 homolog		KOG3861	143	159	139	256	294	30	49	44	142	121	127	338	144	130	14	5
ACC_06161	hypothetical protein			13	7	15	89	24	4	2	1	11	7	12	5	9	12	6	5
ACC_06162	eyes absent homolog 4		KOG3107	252	193	222	721	418	111	174	208	830	369	44	59	62	34	34	19
ACC_06163	post-GPI attachment to proteins factor 2-like		KOG3979	212	80	72	123	102	6	6	14	112	104	21	52	49	47	5	1
ACC_06164	LOW QUALITY PROTEIN	K15166	KOG1883	556	431	395	714	627	37	52	47	859	709	344	434	719	906	98	27
ACC_06165	TNF receptor-associated factor 6	K03175	KOG0297	133	112	73	136	177	7	4	6	88	109	63	228	209	179	16	4
ACC_06166	probable polyprenol reductase-like	K12345	KOG1640	41	34	38	46	81	1	2	1	41	26	25	57	69	59	5	0
ACC_06167	replication protein A 32 kDa subunit-like	K10739	KOG3108	20	30	19	31	33	1	1	2	13	51	24	88	64	28	0	0
ACC_06168	GPN-loop GTPase 1-like	K06883	KOG1532	375	327	315	428	378	86	142	152	489	205	272	420	509	758	273	199
ACC_06169	replication protein A 32 kDa subunit	K10739	KOG3108	120	103	105	146	188	7	15	15	132	58	134	148	147	284	39	17
ACC_06170	GPN-loop GTPase 3-like	K06883	KOG1534	89	62	74	161	184	10	31	14	73	49	106	105	237	398	34	20
ACC_06171	39S ribosomal protein L18, mitochondrial	K02881	KOG3333	154	142	122	230	238	17	29	38	215	85	163	286	355	569	83	55
ACC_06172	hypothetical protein			2	3	2	4	1	0	0	1	0	4	0	0	1	0	1	0
ACC_06173	synaptic vesicle 2-related protein-like		KOG0253	794	464	512	1143	1079	45	72	79	614	348	546	344	289	309	35	10
ACC_06174	PRKR-interacting protein 1 homolog		KOG4055	825	541	464	453	579	144	409	343	281	124	494	418	359	579	1124	594
ACC_06175	60S ribosomal protein L14	K02875	KOG3421	2309	2183	1469	3333	1712	703	1573	2367	2016	1942	2401	2617	10639	12386	5653	4354
ACC_06176	protein AATF-like, partial			106	75	73	93	105	2	25	32	56	55	104	159	116	130	42	21
ACC_06177	LOW QUALITY PROTEIN		KOG2773	323	213	208	205	344	21	113	82	215	165	227	207	390	611	303	224
ACC_06178	conserved hypothetical protein		KOG2942	78	59	45	74	67	2	3	7	98	128	23	138	179	159	20	4
ACC_06179	DNA repair protein XRCC4-like			377	246	189	144	300	9	24	20	49	125	218	422	160	62	110	38
ACC_06180	n-acetylgalactosaminyltransferase 7	K00710	KOG3737	225	243	136	201	169	50	49	80	164	277	64	167	720	624	83	59
ACC_06181	39S ribosomal protein L44, mitochondrial		KOG3769	506	320	334	546	493	82	183	221	290	196	269	300	648	1170	725	694
ACC_06182	conserved hypothetical protein		KOG0260	88767	49161	54762	2919	2443	15064	9218	20424	1623	23	1626	10	42898	1980	118	268
ACC_06183	LOW QUALITY PROTEIN		KOG0462	336	211	251	352	393	20	41	54	246	214	212	385	431	590	90	26
ACC_06184	conserved hypothetical protein			255	223	191	401	375	27	65	64	166	184	104	137	144	114	48	26
ACC_06185	conserved hypothetical protein		KOG2008	115	78	73	144	142	9	18	9	183	153	54	167	103	96	14	4
ACC_06186	beta-1,4-mannosyltransferase egh-like			277	158	138	181	224	8	15	12	259	211	119	206	258	455	24	3
ACC_06187	nucleolar GTP-binding protein 2		KOG2423	490	352	311	439	480	72	222	209	403	358	259	449	868	1002	555	345
ACC_06188	polymerase delta-interacting protein 2-like isoform 1		KOG4408	615	487	462	1087	815	137	230	277	1605	896	699	1314	704	838	264	88
ACC_06189	protein spinster homolog 1-like		KOG1330	71	19	4	30	19	7	11	6	1124	80	126	926	3	6	3	2
ACC_06190	lipase 3-like		KOG2624	4	14	17	44	32	1	5	2	16	41	3	13	31	13	1	0
ACC_06191	conserved hypothetical protein			73	53	60	90	73	5	3	4	96	53	29	95	52	36	10	0
ACC_06192	probable E3 ubiquitin-protein ligase MYCBP2-like		KOG1428	235	164	157	159	83	32	69	103	327	261	53	30	83	104	120	70
ACC_06193	puromycin-sensitive aminopeptidase isoform 1	K08776	KOG1046	506	326	243	907	432	74	65	91	809	1108	332	1231	2297	4444	961	193
ACC_06194	craniofacial development protein 1		KOG4776	277	186	207	298	259	80	144	191	270	142	130	101	212	268	883	719
ACC_06195	actin, clone 205-like isoform 1	K05692	KOG0676	911	160	47	282	54	43	42	48	6401	4878	175	353	1841	1234	2077	1903
ACC_06196	sugar transporter SWEET1-like	K15382	KOG1623	244	264	306	741	892	26	31	35	567	463	333	1687	847	557	34	25
ACC_06197	soluble guanylyl cyclase beta-3		KOG4171	33	21	36	63	24	0	3	2	87	65	6	27	21	2	2	0
ACC_06198	protein spire-like	K02098		2625	1912	1949	2374	1246	157	217	275	1921	1450	494	403	840	647	290	127
ACC_06199	retinoblastoma-family protein	K04681	KOG1010	183	102	108	119	103	7	13	17	275	196	60	137	328	362	42	14
ACC_06200	conserved hypothetical protein		KOG1331	714	312	253	430	191	12	38	39	609	361	138	86	157	153	37	9
ACC_06201	UPF0667 protein C1orf55 homolog		KOG2827	216	140	130	346	395	65	129	146	291	117	391	332	163	259	160	131
ACC_06202	LOW QUALITY PROTEIN		KOG1924	813	396	488	658	200	108	99	148	1775	1026	389	543	540	228	219	128
ACC_06203	conserved hypothetical protein	K05961	KOG4235	384	225	197	367	295	32	55	47	221	189	209	222	271	446	124	69
ACC_06204	yellow-e3 precursor			13	102	5	16	9	38	47	31	58	52	8	84	75	55	3	10
ACC_06205	40S ribosomal protein S5 isoform 2	K02989	KOG3291	1569	1153	508	1742	1174	441	636	802	1233	996	1072	1948	9319	9132	3275	2016
ACC_06206	NADH dehydrogenase	K03949	KOG3365	1193	1074	1117	1805	1175	164	221	271	2322	508	992	2144	1109	1413	563	339
ACC_06207	major royal jelly protein MRJP4 precursor			12818	78461	1061	347	733	55005	32487	74013	7	31	2	12	136	2	0	0
ACC_06208	major royal jelly protein 6 precursor			6664	103641	45681	12486	13798	60950	30518	70390	34	36	4	4	18	3	0	1
ACC_06209	major royal jelly protein 2-like			38508	335648	34381	2395	6673	196972	126878	255579	20	191	16	41	17	5	0	2
ACC_06210	major royal jelly protein 8 precursor			8639	324108	11880	973	2522	195618	182263	386588	873	795	522	74	95	16	7	0

ACC_06211	neuroparsin-A-like			112	80	160	350	205	8	14	19	131	40	18	8	0	12	4	0
ACC_06212	protein twisted gastrulation-like			34	13	14	23	18	1	6	2	211	147	32	91	184	142	11	11
ACC_06213	serine/threonine-protein kinase mos-like		KOG0192	56	46	30	95	68	6	15	10	51	33	22	35	39	93	30	9
ACC_06214	phosphatase and tensin-like	K01110	KOG2283	196	130	119	123	73	20	20	20	360	205	46	75	181	242	95	23
ACC_06215	stress-activated protein kinase JNK-like	K04440	KOG0665	473	337	302	770	206	60	77	112	353	280	114	75	76	142	287	221
ACC_06216	conserved hypothetical protein			55	27	21	56	26	6	15	9	40	21	18	7	6	18	32	13
ACC_06217	elongation factor Ts, mitochondrial-like	K02357	KOG1071	357	150	180	414	354	22	30	41	448	295	244	552	1008	1292	83	87
ACC_06218	conserved hypothetical protein			227	130	131	178	206	16	23	34	191	123	97	139	367	358	100	33
ACC_06219	DNA topoisomerase 1 isoform 1	K03163	KOG0981	4363	2926	2689	2666	3004	895	1899	2112	2255	1597	1759	1162	2329	4029	9547	10517
ACC_06220	39S ribosomal protein L30, mitochondrial	K02907	KOG4799	159	134	124	231	313	4	15	24	117	56	146	377	255	384	32	25
ACC_06221	homeobox protein aristaless-like 4-like		KOG0484	20	6	18	22	22	4	4	9	54	42	78	96	6	8	6	2
ACC_06222	zinc finger protein 341-like		KOG3608	244	180	210	320	215	21	22	19	282	211	122	266	278	278	84	31
ACC_06223	conserved hypothetical protein		KOG0161	334	86	102	172	111	4	4	10	179	102	123	483	131	210	49	21
ACC_06224	deoxyribonuclease-2-alpha	K01158	KOG3825	63	40	60	136	95	4	4	5	63	22	5	71	22	33	3	7
ACC_06225	hypothetical protein			33	12	11	9	14	1	2	0	6	1	0	0	0	0	1	0
ACC_06226	hypothetical protein			4	0	4	5	3	0	0	0	4	0	0	2	0	0	0	0
ACC_06227	conserved hypothetical protein			2	2	1	1	0	1	0	1	1	5	0	0	1	2	0	0
ACC_06228	nose resistant to fluoxetine protein 6-like		KOG3700	28	25	21	60	53	5	16	9	78	161	2	20	158	133	7	4
ACC_06229	nose resistant to fluoxetine protein 6-like		KOG3700	66	43	58	84	100	3	8	12	64	99	44	27	611	513	56	12
ACC_06230	casein kinase II subunit beta	K03115	KOG3092	303	139	116	383	303	23	27	36	356	399	131	633	677	746	182	56
ACC_06231	WD repeat-containing protein 46-like	K14768	KOG1272	329	210	161	408	443	59	140	136	334	217	217	352	566	843	454	318
ACC_06232	high affinity copper uptake protein 1-like isoform 1	K14686	KOG3386	163	192	91	130	79	61	65	98	203	131	84	245	269	214	37	17
ACC_06233	protein penguin-like	K14844	KOG2050	626	400	243	418	591	23	105	97	356	296	399	454	603	892	377	234
ACC_06234	serine/threonine-protein kinase SIK3-like	K16311	KOG0586	168	180	163	119	64	8	13	19	506	331	115	73	232	231	56	22
ACC_06235	La-related protein 4	K03113	KOG1770	944	972	847	2768	1417	116	149	251	1359	963	652	2140	1572	1965	1387	483
ACC_06236	conserved hypothetical protein		KOG2405	423	226	177	340	272	30	85	62	749	676	205	442	671	585	179	47
ACC_06237	conserved hypothetical protein		KOG4441	1430	1266	1294	1784	463	144	171	287	1141	992	304	245	256	250	429	203
ACC_06238	putative protein arginine N-methyltransferase 10-like		KOG1501	180	111	98	112	142	3	13	14	139	91	146	107	217	205	27	7
ACC_06239	polycomb protein Sfmbt-like		KOG3766	142	126	110	75	44	9	8	11	308	372	59	172	254	245	46	22
ACC_06240	TBC1 domain family member 13-like isoform 1		KOG4567	220	177	154	226	237	12	26	31	215	212	115	170	376	316	30	8
ACC_06241	conserved hypothetical protein			49	27	18	64	75	8	7	6	85	50	37	40	97	59	6	2
ACC_06242	conserved hypothetical protein			957	1201	2295	7555	7514	147	246	282	1099	1114	74	48	130	100	42	21
ACC_06243	conserved hypothetical protein			12	8	15	66	21	1	1	4	26	33	2	3	15	28	7	4
ACC_06244	hypothetical protein			2	2	0	3	1	0	0	0	1	0	0	0	0	0	0	0
ACC_06245	estradiol 17-beta-dehydrogenase 12-like		KOG1014	578	298	348	1203	639	55	54	85	2051	2121	126	115	813	1518	449	289
ACC_06246	elongation factor Tu GTP-binding domain-containir	K14536	KOG0467	392	238	255	693	317	48	74	81	553	465	120	273	563	807	468	182
ACC_06247	ATP-dependent Clp protease ATP-binding subunit c	K03544	KOG0745	356	237	185	338	314	26	24	35	1821	742	190	759	728	449	64	11
ACC_06248	LOW QUALITY PROTEIN	K01893	KOG0555	508	421	288	804	631	146	346	489	840	562	380	469	1500	1698	672	460
ACC_06249	zinc finger protein 543-like		KOG2462	176	135	170	197	148	12	22	25	220	153	86	295	179	281	27	25
ACC_06250	conserved hypothetical protein		KOG1984	2963	3072	3131	2834	1093	271	371	462	2131	1801	475	283	1157	1030	1277	880
ACC_06251	MKI67 FHA domain-interacting nucleolar phosphop	K14838	KOG4208	258	166	121	271	323	28	82	94	218	186	274	419	986	957	202	179
ACC_06252	potassium channel subfamily K member 10-like		KOG1418	9	11	13	53	17	3	1	0	22	33	9	2	4	4	2	2
ACC_06253	GatC-like protein		KOG4247	82	60	81	58	75	2	8	8	61	111	65	406	232	126	10	3
ACC_06254	alpha-aminoadipic semialdehyde synthase, mitocho	K14157	KOG0172	8	4	4	10	14	0	0	4	362	520	4	0	1906	1962	261	100
ACC_06255	protein msta, isoform A-like		KOG2084	89	57	51	132	183	8	12	14	69	107	51	93	52	23	4	1
ACC_06256	conserved hypothetical protein	K11765	KOG2312	633	657	728	752	265	93	121	143	747	631	215	140	342	429	375	164
ACC_06257	cGMP-dependent 3',5'-cyclic phosphodiesterase-lik	K01120	KOG3689	338	193	221	264	325	25	37	34	317	275	203	188	231	167	31	20
ACC_06258	tetratricopeptide repeat protein 8-like isoform 1	K16781	KOG1129	81	86	108	134	139	8	12	13	42	45	69	141	5	16	5	1
ACC_06259	conserved hypothetical protein			125	78	46	79	64	14	10	21	77	93	41	423	609	465	72	45
ACC_06260	SEC14 domain and spectrin repeat-containing protein 1-like		KOG0517	44	10	6	17	1	16	21	23	153	168	6	70	579	285	77	101
ACC_06261	conserved hypothetical protein		KOG3017	380	338	264	623	294	66	104	107	827	409	306	562	165	167	49	23
ACC_06262	Kv channel-interacting protein 1-like		KOG0044	824	315	268	779	325	67	127	170	289	152	711	240	4	8	69	44
ACC_06263	conserved hypothetical protein			63	34	39	70	95	12	5	10	66	78	60	185	172	135	10	2
ACC_06264	conserved hypothetical protein		KOG2579	21	6	6	11	9	2	1	2	56	20	7	95	28	54	15	4
ACC_06265	dynein heavy chain 1, axonemal	K10408		11	5	6	20	11	0	2	1	1	12	2	0	0	3	4	0
ACC_06266	myocyte-specific enhancer factor 2	K09263	KOG0014	243	302	261	276	63	21	28	51	679	970	87	114	272	229	230	85
ACC_06267	LOW QUALITY PROTEIN		KOG3678	160	158	169	167	80	42	35	43	1261	975	109	335	310	415	105	13

ACC_06268	sphingomyelin phosphodiesterase 4-like	K12353	KOG4396	99	97	85	90	79	8	9	17	116	85	41	47	218	296	36	7
ACC_06269	conserved hypothetical protein		KOG1427	431	298	239	378	236	38	57	72	786	659	116	197	184	115	24	16
ACC_06270	aminoacylase-1-like	K14677	KOG2275	1007	454	595	1877	1693	66	71	95	909	1575	615	5008	6102	2541	118	57
ACC_06271	GTP-binding protein 128up-like isoform 1		KOG1487	158	121	108	270	251	14	23	28	217	186	98	255	885	1117	142	53
ACC_06272	protein phosphatase PTC7 homolog		KOG1379	153	84	74	364	248	16	19	30	205	88	83	158	143	171	62	19
ACC_06273	pre-rRNA-processing protein TSR1 homolog	K14799	KOG1980	487	336	311	673	634	77	157	180	702	463	428	619	1016	1505	490	380
ACC_06274	conserved hypothetical protein			35	28	27	26	37	6	3	8	52	81	53	49	69	91	13	1
ACC_06275	transcription initiation factor TFIID subunit 10-like	K03134	KOG3423	465	299	335	557	346	22	39	45	620	330	356	1190	371	415	122	48
ACC_06276	LOW QUALITY PROTEIN		KOG1144	4288	2848	2937	3105	2183	989	1846	2087	3651	1822	1302	385	2210	3781	8699	9765
ACC_06277	pancreas transcription factor 1 subunit alpha-like is	K09073	KOG4029	8	4	4	12	5	0	1	0	7	6	147	106	2	1	1	1
ACC_06278	solute carrier family 46 member 3-like	K14613	KOG2816	2608	1719	1432	2802	2441	520	885	761	913	699	704	378	1163	1056	184	31
ACC_06279	transmembrane protein 184C-like		KOG2641	368	171	184	198	182	58	70	99	416	260	199	365	225	173	63	31
ACC_06280	cytochrome c oxidase copper chaperone-like	K02260	KOG3496	79	56	57	146	91	7	4	3	29	9	125	72	98	153	22	8
ACC_06281	leucine-rich repeat transmembrane protein FLRT1-like		KOG0027	419	296	244	930	347	119	150	128	5464	987	504	722	7	13	24	6
ACC_06282	pre-mRNA-splicing factor Syf2-like	K12868	KOG2609	269	224	208	590	400	63	111	167	268	136	309	305	221	373	291	219
ACC_06283	alcohol dehydrogenase		KOG1577	72	52	50	76	108	4	10	11	282	314	24	226	7551	646	29	32
ACC_06284	contactin-like	K01900	KOG1447	200	123	84	116	175	23	31	38	305	340	145	457	1964	1552	87	28
ACC_06285	conserved hypothetical protein			82	52	57	102	105	7	4	8	69	29	29	21	299	98	10	1
ACC_06286	lysine-specific demethylase 8-like		KOG2132	90	73	72	189	314	4	6	9	129	73	88	196	137	155	16	2
ACC_06287	conserved hypothetical protein			145	77	68	196	132	251	406	640	1603	711	43	57	2	6	20	6
ACC_06288	conserved hypothetical protein			103	95	60	163	106	18	36	29	1087	526	74	98	201	135	28	15
ACC_06289	LOW QUALITY PROTEIN	K12311	KOG1959	364	782	376	637	650	357	197	516	1000	615	40	148	21503	30196	1190	50
ACC_06290	conserved hypothetical protein			78	82	77	121	100	6	7	12	110	114	65	184	57	57	6	5
ACC_06291	conserved hypothetical protein		KOG1144	1260	393	476	1910	597	93	237	245	552	280	235	56	19	34	242	242
ACC_06292	protein timeless homolog	K03155	KOG1974	28	20	16	31	16	2	7	11	25	15	3	14	64	105	145	86
ACC_06293	conserved hypothetical protein		KOG1930	155	82	107	162	49	7	6	14	115	171	38	78	116	109	131	51
ACC_06294	conserved hypothetical protein			289	184	187	476	351	20	33	49	118	65	76	50	10	14	5	3
ACC_06295	sensory neuron membrane protein 2-like		KOG3776	27	39	1	6	4	8	15	12	140	30	29	12	114	94	17	0
ACC_06296	ubiquitin-conjugating enzyme E2-17 kDa-like	K06689	KOG0417	984	872	850	1147	463	175	244	328	1383	1098	652	1666	2041	2346	1456	570
ACC_06297	sec1 family domain-containing protein 2-like			264	297	220	429	553	15	38	46	237	190	169	226	339	332	31	11
ACC_06298	exportin-4-like isoform 2		KOG4541	147	79	111	179	192	11	14	9	230	116	112	79	194	214	14	8
ACC_06299	conserved hypothetical protein		KOG1832	532	342	370	833	579	41	74	83	273	183	113	113	356	479	235	141
ACC_06300	LOW QUALITY PROTEIN	K00294	KOG2455	1093	421	405	803	678	68	118	134	2054	1235	330	1535	719	555	67	57
ACC_06301	conserved hypothetical protein			1	1	0	0	0	1	0	0	4	1	0	0	0	0	0	0
ACC_06302	prothrombin		KOG3627	99	32	27	189	92	16	17	25	105	43	98	189	164	289	269	184
ACC_06303	TBC domain-containing protein kinase-like protein-like		KOG1093	688	450	458	815	870	76	238	226	616	481	338	368	396	465	232	105
ACC_06304	copper transport protein ATOX1-like	K07213	KOG4656	88	48	43	80	81	9	19	8	65	31	56	133	184	246	72	51
ACC_06305	protein midA homolog, mitochondrial-like		KOG2901	140	113	111	155	196	13	41	44	337	155	210	303	328	254	31	29
ACC_06306	conserved hypothetical protein		KOG1015	577	372	286	569	833	108	283	292	452	262	423	304	361	542	1176	572
ACC_06307	transmembrane protein 53-like		KOG2521	499	455	349	429	550	90	148	191	1166	573	541	1650	1070	956	81	28
ACC_06308	acyl-CoA synthetase family member 4-like		KOG4649	373	200	214	263	369	13	43	47	261	205	209	194	267	314	36	14
ACC_06309	probable phospholipid-transporting ATPase VD-like	K01530	KOG0206	874	493	319	499	430	127	182	153	1837	921	433	287	553	756	134	22
ACC_06310	bestrophin-2-like		KOG3547	319	132	150	177	65	24	32	49	318	174	376	179	100	112	112	46
ACC_06311	conserved hypothetical protein	K15009	KOG4177	1622	1229	1289	926	178	149	159	234	601	606	330	260	51	175	545	102
ACC_06312	WD repeat-containing protein 19-like		KOG2247	434	333	362	388	359	34	73	72	377	301	360	232	284	347	79	26
ACC_06313	conserved hypothetical protein			142	70	75	164	84	10	1	6	75	90	39	121	167	161	96	47
ACC_06314	mucosa-associated lymphoid tissue lymphoma trar	K07369	KOG3513	56	38	40	47	61	6	7	5	33	61	28	119	129	94	8	0
ACC_06315	probable 39S ribosomal protein L24, mitochondrial	K02895	KOG1708	456	388	362	693	613	79	95	127	838	349	320	643	998	1344	132	73
ACC_06316	conserved hypothetical protein		KOG0526	1728	1366	1254	1471	1560	303	781	766	829	510	550	472	1015	1395	3222	2370
ACC_06317	alpha-tocopherol transfer protein-like		KOG1471	5	2	1	6	3	2	3	3	70	108	88	446	103	291	18	25
ACC_06318	cytochrome c oxidase subunit 6B1-like		KOG3057	8	10	5	13	17	2	2	1	2	5	10	2	8	9	8	3
ACC_06319	leucine-rich repeat-containing protein 47-like		KOG2472	296	196	199	253	246	20	37	37	253	220	139	428	548	547	157	117
ACC_06320	conserved hypothetical protein			9205	5348	4118	6581	4938	2268	4560	4717	6733	4306	3044	1321	1967	2578	7644	3955
ACC_06321	conserved hypothetical protein		KOG0921	135	67	37	129	60	19	31	20	1559	227	1605	3108	377	906	232	308
ACC_06322	zinc finger CCCH domain-containing protein 15 homolog		KOG1763	1167	607	545	1354	1341	261	632	621	770	508	610	499	1019	1579	2586	3179
ACC_06323	conserved hypothetical protein		KOG0905	396	182	238	487	130	10	9	11	114	93	17	1	4	9	17	15
ACC_06324	leucine-rich repeat and immunoglobulin-like domain-contair		KOG4194	292	166	103	178	91	51	59	91	468	495	129	359	406	339	383	193

ACC_06325	NEDD4 family-interacting protein 1-like		KOG4812	611	316	297	560	470	51	65	65	762	597	274	1330	779	597	62	15
ACC_06326	probable RNA-binding protein EIF1AD-like	K15025	KOG2925	81	73	51	87	64	11	26	30	100	65	73	132	276	439	68	46
ACC_06327	neuropeptides capa receptor-like		KOG4219	33	28	23	126	106	4	6	10	19	26	16	8	97	51	24	1
ACC_06328	CG14575-like G protein-coupled receptor		KOG4219	2	7	1	4	1	0	1	1	4	7	0	11	7	5	4	2
ACC_06329	conserved hypothetical protein		KOG3854	94	78	65	86	88	11	25	28	96	57	121	676	135	209	171	67
ACC_06330	LOW QUALITY PROTEIN	K00453	KOG3906	35	6	9	75	50	6	17	16	657	1109	23	90	282	261	45	5
ACC_06331	retinol dehydrogenase 12-like		KOG1208	159	114	116	202	235	9	7	18	602	222	860	1947	146	182	7	3
ACC_06332	conserved hypothetical protein		KOG0811	84	23	25	55	25	3	1	4	22	11	14	4	0	1	1	0
ACC_06333	nucleolar protein 6-like	K14544	KOG2054	1605	785	788	1040	1309	76	157	161	764	630	511	597	1137	1353	547	318
ACC_06334	conserved hypothetical protein			8	6	7	3	2	1	2	7	56	62	8	36	1	13	7	11
ACC_06335	conserved hypothetical protein			2	1	1	16	2	2	1	2	5	22	2	3	1	0	1	0
ACC_06336	conserved hypothetical protein			119	92	82	175	184	17	20	27	140	97	77	97	123	237	61	17
ACC_06337	probable phospholipid-transporting ATPase IA-like	K14802	KOG0206	942	360	269	520	454	142	143	228	823	641	330	317	585	359	81	32
ACC_06338	LIM/homeobox protein Lhx5-like	K09372	KOG4577	69	35	60	216	91	9	7	18	23	50	18	7	8	21	65	52
ACC_06339	conserved hypothetical protein		KOG0161	173	142	93	195	150	217	570	474	6379	1776	204	61	168	174	239	241
ACC_06340	coiled-coil domain-containing protein 42 homolog		KOG1029	190	136	156	297	264	25	43	38	90	39	85	60	16	23	13	3
ACC_06341	bicaudal D-related protein homolog isoform 1	K16756	KOG0161	21	26	29	18	4	8	10	15	98	73	21	32	23	39	26	7
ACC_06342	acetyl-CoA acetyltransferase, cytosolic-like	K00626	KOG1390	426	184	223	664	711	20	31	30	1238	974	190	1037	282	506	47	13
ACC_06343	28S ribosomal protein S29, mitochondrial isoform 1		KOG3928	320	286	280	400	436	19	34	48	418	240	288	403	653	1079	54	37
ACC_06344	protein lin-9 homolog		KOG1019	231	133	108	240	170	39	58	73	280	203	131	216	364	454	273	186
ACC_06345	conserved hypothetical protein		KOG0161	169	89	89	85	123	9	37	23	155	198	40	51	151	245	190	89
ACC_06346	conserved hypothetical protein			1	48	37	11	16	16	4	21	277	128	101	149	4	0	0	1
ACC_06347	latrophilin Cirl-like isoform 1		KOG4193	222	90	98	236	66	5	18	30	154	146	41	28	28	17	73	42
ACC_06348	conserved hypothetical protein		KOG3513	169	320	303	122	79	192	195	286	343	236	867	1003	116	48	51	41
ACC_06349	c-Myc-binding protein-like			99	90	70	135	150	16	29	21	204	115	74	252	474	589	58	32
ACC_06350	UPF0431 protein C1orf66 homolog		KOG2651	94	73	69	117	129	11	30	38	93	93	56	91	128	127	24	14
ACC_06351	sex-lethal homolog		KOG0145	662	400	354	787	370	71	131	135	933	509	210	507	482	674	408	149
ACC_06352	doublesex- and mab-3-related transcription factor A2-like		KOG3815	7	11	16	85	26	1	1	5	7	10	0	0	8	3	7	2
ACC_06353	breast cancer metastasis-suppressor 1-like protein-A-like		KOG4466	92	71	81	109	132	8	22	26	105	93	56	145	180	221	51	28
ACC_06354	actin-related protein 5	K11672	KOG0681	244	199	174	303	229	48	111	127	384	209	160	213	274	372	261	184
ACC_06355	importin-13		KOG2022	196	118	99	314	222	15	30	42	341	293	112	215	399	483	94	29
ACC_06356	conserved hypothetical protein		KOG1609	36	26	36	43	15	5	18	21	24	19	13	16	30	33	22	9
ACC_06357	protein-S-isoprenylcysteine O-methyltransferase-lil	K00587	KOG2628	143	86	100	317	331	6	10	12	113	66	77	154	127	138	12	4
ACC_06358	conserved hypothetical protein		KOG1015	1244	1365	1531	1398	251	153	149	383	2553	2112	282	175	308	479	786	466
ACC_06359	sorting nexin-13-like		KOG2101	617	555	438	499	417	58	71	104	894	662	153	436	524	388	52	6
ACC_06360	conserved hypothetical protein		KOG1053	1022	391	305	639	202	65	91	149	431	234	73	32	25	63	97	21
ACC_06361	PDZ domain-containing protein GIPC1-like		KOG3938	178	142	123	359	310	48	69	84	321	253	166	341	779	933	281	149
ACC_06362	LOW QUALITY PROTEIN	K01027	KOG3822	563	394	375	574	573	78	75	77	2157	1338	599	1596	3356	3273	238	75
ACC_06363	dual specificity protein phosphatase 7		KOG1717	299	99	117	472	258	20	29	25	388	185	51	180	178	233	136	34
ACC_06364	peroxiredoxin-5, mitochondrial-like		KOG0541	22	21	10	5	5	0	5	4	68	22	7	6	12	7	2	0
ACC_06365	hypothetical protein			9	10	5	5	2	1	0	0	9	4	2	1	0	6	0	0
ACC_06366	dnaJ homolog subfamily B member 6-like	K09512	KOG0716	686	546	620	1153	670	160	202	301	1517	939	503	1026	603	720	400	172
ACC_06367	endoplasmin-like isoform 1	K09487	KOG0020	2003	1147	906	4187	4171	181	378	509	2158	2790	629	2325	10853	10945	1877	1569
ACC_06368	ATP-binding cassette sub-family F member 3-like is	K06158	KOG0062	662	373	339	640	553	73	190	191	601	340	208	346	998	1217	585	522
ACC_06369	conserved hypothetical protein		KOG4636	115	120	99	133	97	17	27	28	266	277	90	274	263	243	42	6
ACC_06370	retinal homeobox protein Rx2		KOG0484	1332	1207	1293	1447	563	75	100	111	752	336	197	348	15	62	111	81
ACC_06371	probable peroxisomal acyl-coenzyme A oxidase 1-li	K00232	KOG0136	152	122	130	246	203	21	36	33	516	364	387	1299	315	324	23	11
ACC_06372	protein scarlet-like		KOG0061	4	1	0	3	0	4	1	3	24	6	20	187	1	2	0	0
ACC_06373	hypothetical protein			2	5	6	2	7	2	0	3	2	11	0	0	19	9	2	0
ACC_06374	conserved hypothetical protein		KOG3779	3638	2200	2104	2646	1995	755	1592	1802	1886	1339	1121	403	1197	1639	3307	1705
ACC_06375	E3 ubiquitin-protein ligase MARCH2-like		KOG1609	3	6	8	13	13	0	0	0	2	9	1	4	31	20	8	2
ACC_06376	polycomb group RING finger protein 3-like	K11488	KOG2660	42	29	28	71	76	3	3	9	87	38	11	55	61	73	15	0
ACC_06377	s-adenosylmethionine mitochondrial carrier protei	K15111	KOG0768	113	89	78	133	152	17	35	35	66	90	51	101	232	290	60	16
ACC_06378	sodium channel protein Nach-like		KOG4294	5	7	1	10	5	1	0	2	16	14	20	5	7	1	0	0
ACC_06379	hypothetical protein			3	0	1	0	0	0	0	0	0	0	0	0	0	0	1	0
ACC_06380	conserved hypothetical protein			130	60	72	47	33	6	3	4	72	73	20	16	25	23	21	3
ACC_06381	LOW QUALITY PROTEIN	K00844	KOG1369	1167	1037	603	653	357	446	437	615	2629	1546	408	1550	822	721	200	71

ACC_06382	protein FAM160B1-like		KOG3695	498	470	453	1095	503	86	97	129	762	625	246	539	324	284	110	20
ACC_06383	conserved hypothetical protein		KOG0262	13	20	27	48	59	48	95	94	193	142	4	11	0	0	1	5
ACC_06384	thioredoxin-2 isoform 2	K03671	KOG0907	280	297	235	642	654	42	49	88	840	304	556	1105	2603	2494	245	127
ACC_06385	LOW QUALITY PROTEIN		KOG0196	2	0	0	1	0	1	0	0	21	78	3	3	7	4	1	0
ACC_06386	conserved hypothetical protein		KOG0996	6541	3537	3150	2655	3901	849	2096	2176	3002	1634	2201	794	1179	1648	2626	1139
ACC_06387	conserved hypothetical protein			125	58	80	191	116	11	29	24	247	170	76	170	54	72	25	9
ACC_06388	hypothetical protein			27	17	25	22	25	2	4	4	8	7	5	0	0	0	3	2
ACC_06389	hypothetical protein			1	1	1	0	1	0	1	2	0	1	0	0	0	0	0	0
ACC_06390	maternal protein exuperantia			59	55	11	27	25	39	41	43	166	274	75	256	141	54	12	21
ACC_06391	conserved hypothetical protein			1227	513	542	801	504	49	71	90	915	384	127	236	64	85	46	19
ACC_06392	probable calcium-binding protein CML13-like			6	0	6	10	8	1	2	1	15	5	27	11	12	5	0	8
ACC_06393	LOW QUALITY PROTEIN		KOG0939	1585	438	459	845	495	41	42	53	1416	778	457	333	85	110	20	13
ACC_06394	ATP-dependent RNA helicase Ddx1 isoform 1	K13177	KOG0349	450	291	320	562	430	32	31	52	653	399	240	475	758	858	68	45
ACC_06395	malate dehydrogenase, cytoplasmic-like isoform 1	K00025	KOG1496	722	415	379	1174	1081	77	90	119	2313	1160	910	2616	5375	2841	292	164
ACC_06396	leucine-rich repeat-containing protein 67-like		KOG1259	14	17	14	9	11	2	1	1	10	3	15	27	2	2	1	1
ACC_06397	conserved hypothetical protein			3	5	6	12	3	0	0	3	7	11	27	61	52	31	5	7
ACC_06398	RNA-binding protein 40-like	K13157	KOG1144	4388	2086	1154	595	826	903	1842	2281	1089	573	644	590	1877	1809	1391	917
ACC_06399	hypothetical protein			19	8	7	16	11	2	0	3	5	7	5	6	8	5	2	2
ACC_06400	hypothetical protein			218	172	168	130	99	14	28	26	103	75	39	122	60	57	8	3
ACC_06401	conserved hypothetical protein			522	359	321	501	678	41	93	99	219	228	317	403	333	330	147	69
ACC_06402	conserved hypothetical protein	K16472		127	83	80	119	136	6	25	36	69	27	77	75	85	203	232	104
ACC_06403	conserved hypothetical protein	K09553	KOG0548	1363	808	1059	1663	1110	135	245	383	1591	1350	541	1756	4251	5289	1623	1328
ACC_06404	conserved hypothetical protein			349	240	247	431	299	33	56	43	205	95	866	442	7	19	7	10
ACC_06405	protein bcn92-like		KOG3801	46	39	40	114	86	0	9	5	20	26	144	53	53	61	7	5
ACC_06406	facilitated trehalose transporter Tret1-like		KOG0254	2	5	10	6	8	1	1	1	17	7	3	0	6	10	5	7
ACC_06407	post-GPI attachment to proteins factor 2-like		KOG3979	529	367	337	905	828	46	134	155	752	424	320	924	546	444	76	27
ACC_06408	menin-like	K14970		225	122	158	187	84	16	18	28	251	177	34	50	109	149	72	18
ACC_06409	coiled-coil domain-containing protein 123, mitochc	K16543	KOG0161	227	156	125	187	219	34	91	100	145	94	90	74	86	137	139	93
ACC_06410	Plectin-1		KOG0517	69	23	26	137	46	21	30	29	147	116	34	19	9	5	131	94
ACC_06411	transient receptor potential-gamma protein-like isoform 2		KOG3609	20	29	39	64	27	11	14	16	65	134	36	8	29	31	11	4
ACC_06412	short transient receptor potential channel 6-like		KOG3609	6	5	15	35	33	4	5	3	14	32	25	1	12	14	0	1
ACC_06413	PIH1 domain-containing protein 1		KOG4356	72	41	52	105	153	5	7	6	22	42	71	180	171	196	17	8
ACC_06414	conserved hypothetical protein			431	216	199	129	224	5	70	75	128	93	186	242	216	306	242	147
ACC_06415	protein LTV1 homolog	K14798	KOG2637	401	305	297	498	492	90	232	229	244	216	223	269	737	1069	792	559
ACC_06416	conserved hypothetical protein		KOG3685	170	126	78	110	32	17	13	24	200	239	25	37	122	132	52	35
ACC_06417	UBX domain-containing protein 1-like		KOG2689	428	269	191	545	447	109	272	294	466	214	385	521	544	699	1579	824
ACC_06418	TBC1 domain family member 15		KOG2197	240	129	140	252	315	34	29	46	527	490	169	389	332	275	21	8
ACC_06419	conserved hypothetical protein			403	266	201	330	201	41	53	76	408	348	133	149	342	291	126	34
ACC_06420	magnesium transporter NIPA2		KOG2922	242	194	209	264	361	8	31	25	241	166	205	277	197	256	17	4
ACC_06421	probable phenylalanyl-tRNA synthetase beta chain	K01890	KOG2472	668	537	510	709	746	35	78	102	501	427	476	736	1049	1398	83	33
ACC_06422	conserved hypothetical protein	K05725	KOG4257	874	736	639	434	245	48	58	74	1129	808	111	170	193	290	61	37
ACC_06423	conserved hypothetical protein		KOG4364	7	8	11	4	11	8	7	8	50	20	140	25	4	14	6	4
ACC_06424	conserved hypothetical protein			91	45	34	67	86	4	17	13	52	69	67	97	325	403	56	66
ACC_06425	conserved hypothetical protein		KOG1015	10073	6163	6349	8686	2479	602	789	1144	5801	3367	382	241	50	143	208	220
ACC_06426	tubulin epsilon chain	K10391	KOG0129	631	312	387	989	357	46	73	118	415	541	163	67	114	200	260	151
ACC_06427	1,2-dihydroxy-3-keto-5-methylthiopentene dioxyge	K08967	KOG2107	253	242	141	239	285	46	102	78	231	132	193	518	848	605	89	62
ACC_06428	conserved hypothetical protein		KOG4194	121	73	80	98	87	8	14	13	122	64	160	58	59	47	5	5
ACC_06429	copper homeostasis protein cutC homolog	K06201	KOG4013	30	22	20	25	11	13	23	10	58	33	11	54	34	37	18	13
ACC_06430	conserved hypothetical protein	K16719	KOG3253	267	212	249	237	157	30	45	48	437	278	154	229	277	292	54	17
ACC_06431	gamma-glutamyltransferase 7-like		KOG2410	173	131	111	190	212	10	17	20	232	213	129	276	237	212	34	11
ACC_06432	swi5-dependent recombination DNA repair protein 1 homolog			124	57	80	72	64	0	19	14	4	17	46	40	71	58	34	19
ACC_06433	hippocampus abundant transcript 1 protein-like isoform 1		KOG2816	1319	286	262	982	605	128	268	302	852	524	381	682	778	670	730	369
ACC_06434	conserved hypothetical protein		KOG4399	364	270	252	290	314	93	134	153	362	236	198	328	692	1304	585	275
ACC_06435	n-acetyl-D-glucosamine kinase-like	K00884	KOG1794	188	132	98	286	162	38	66	66	169	187	73	165	2239	1546	433	318
ACC_06436	glutaredoxin 3		KOG0911	449	221	242	467	413	42	66	98	649	465	208	674	1594	1443	263	166
ACC_06437	phosphatidate phosphatase PPAPDC1A-like		KOG3030	112	86	122	175	245	17	20	33	136	75	102	261	146	130	10	3
ACC_06438	UPF0668 protein C10orf76 homolog isoform 1		KOG4654	225	107	132	324	367	13	25	30	201	188	148	202	297	340	38	5

ACC_06439	protein FRA10AC1 homolog	K13121	KOG1297	118	82	81	132	151	15	61	68	76	42	92	85	55	58	40	23
ACC_06440	LOW QUALITY PROTEIN	K11142	KOG2597	1707	826	731	1274	930	172	296	260	2211	1666	610	1681	3171	3507	870	733
ACC_06441	TGF-beta-activated kinase 1 and MAP3K7-binding p	K04403	KOG0698	490	274	288	483	485	32	55	75	859	369	320	529	489	520	55	12
ACC_06442	v-type proton ATPase subunit B-like	K02147	KOG1351	1470	663	672	1308	1525	39	24	61	2360	1510	903	5127	4684	3006	113	20
ACC_06443	interleukin enhancer-binding factor 2 homolog isof	K13089	KOG3793	288	182	177	531	407	14	28	33	227	167	111	289	527	739	61	36
ACC_06444	conserved hypothetical protein			0	0	1	0	0	0	0	0	0	0	0	0	0	0	4	0
ACC_06445	clavesin-1-like		KOG1471	51	39	7	42	29	24	37	34	673	166	489	744	7	283	14	49
ACC_06446	dynein heavy chain 8, axonemal-like		KOG3595	9	1	1	7	3	1	0	1	5	7	0	1	10	4	3	1
ACC_06447	conserved hypothetical protein		KOG4400	171	116	120	211	334	79	171	131	927	530	964	1118	724	465	150	101
ACC_06448	72 kDa inositol polyphosphate 5-phosphatase-like i	K01099	KOG0566	402	289	265	464	362	32	46	56	354	297	129	203	99	57	9	6
ACC_06449	ubiquitin carboxyl-terminal hydrolase 7-like	K11838	KOG1863	588	396	335	393	265	58	89	108	1213	1454	133	505	1436	1391	114	32
ACC_06450	transmembrane protein 192-like			237	247	243	192	251	23	24	28	213	160	67	236	275	235	16	4
ACC_06451	serine/threonine-protein phosphatase 5	K04460	KOG0376	299	148	146	281	247	20	56	60	561	367	193	447	641	703	146	51
ACC_06452	conserved hypothetical protein			122	56	74	86	38	6	18	27	507	228	734	1854	131	123	105	29
ACC_06453	conserved hypothetical protein	K04459	KOG1716	26	15	17	43	12	5	9	5	352	249	9	89	29	42	31	7
ACC_06454	conserved hypothetical protein		KOG3612	898	482	504	850	662	120	228	263	1133	746	592	521	768	1030	826	291
ACC_06455	LOW QUALITY PROTEIN		KOG1792	2609	1393	1009	3357	2353	730	1309	1890	1966	1158	2034	1790	948	1036	1730	1583
ACC_06456	leukocyte receptor cluster member 8 homolog		KOG1861	1639	1580	1597	1027	611	98	99	154	2531	1948	532	392	875	935	253	141
ACC_06457	LOW QUALITY PROTEIN	K09479	KOG0137	774	440	467	488	505	69	131	151	1059	797	458	1000	1727	1390	213	127
ACC_06458	guanine nucleotide exchange factor DBS		KOG4240	309	225	170	130	129	14	19	19	366	323	67	196	234	153	13	5
ACC_06459	protein kinase shaggy-like	K03083	KOG0658	937	757	772	1386	382	138	197	264	1120	984	204	216	250	289	389	160
ACC_06460	protein sprint-like		KOG2320	760	263	314	309	106	40	32	55	906	490	152	165	120	117	109	34
ACC_06461	hypothetical protein			78	88	91	68	26	10	10	17	328	235	19	85	139	104	58	16
ACC_06462	zinc finger protein 726-like		KOG2462	104	24	19	67	42	3	1	2	17	2	0	0	1	6	3	1
ACC_06463	conserved hypothetical protein			115	70	79	79	32	9	14	18	160	174	26	29	50	79	63	43
ACC_06464	n-acetyltransferase 15-like		KOG3138	96	52	40	51	34	9	9	7	128	115	26	67	99	104	16	5
ACC_06465	cytochrome c oxidase assembly factor-like			96	112	92	186	131	11	14	24	167	58	78	134	253	414	60	36
ACC_06466	lin-52 protein		KOG4402	64	77	71	135	144	16	25	32	209	59	110	150	300	515	104	43
ACC_06467	conserved hypothetical protein		KOG3627	113	73	97	126	167	7	19	18	117	116	44	94	111	119	16	2
ACC_06468	guanine nucleotide-binding protein subunit gamma	K04547		610	341	501	975	176	185	384	585	247	123	76	96	19	48	261	174
ACC_06469	IQ and AAA domain-containing protein 1-like		KOG0740	17	16	17	32	41	10	11	15	69	24	1101	184	10	8	18	5
ACC_06470	fatty acid synthase-like	K00665	KOG1202	1771	1094	926	1445	1450	552	927	920	17765	39735	511	736	43421	11893	2000	1339
ACC_06471	serine/threonine-protein kinase ICK-like	K08828	KOG0661	143	65	116	134	74	13	17	19	275	302	511	631	186	528	327	111
ACC_06472	HD domain-containing protein 2-like	K07023	KOG3197	380	270	280	586	612	48	119	135	420	133	261	499	313	473	90	73
ACC_06473	conserved hypothetical protein		KOG3878	1204	1007	926	1378	839	308	512	574	1568	786	1022	1692	826	746	746	748
ACC_06474	nicotinic acetylcholine receptor beta2		KOG3645	20	8	10	56	34	1	7	3	53	25	8	12	95	38	10	3
ACC_06475	conserved hypothetical protein		KOG1999	1409	762	613	1616	1111	297	590	656	809	635	667	764	1536	1695	5801	9142
ACC_06476	mitochondrial enolase superfamily member 1-like			95	72	68	74	62	16	19	19	182	628	33	20	7339	2426	108	56
ACC_06477	conserved hypothetical protein		KOG0116	291	212	215	108	58	22	28	37	396	771	35	162	779	865	661	378
ACC_06478	conserved hypothetical protein		KOG1883	213	145	121	172	115	46	107	78	99	93	51	13	27	25	154	117
ACC_06479	putative zinc finger protein 727-like		KOG3623	328	247	235	329	229	62	115	117	76	74	66	13	26	39	364	127
ACC_06480	zinc finger protein 283-like		KOG3623	155	93	105	152	89	12	16	21	55	53	36	17	17	26	79	35
ACC_06481	zinc finger protein 845-like		KOG2462	426	307	290	369	321	93	196	166	791	463	315	365	499	554	308	231
ACC_06482	odorant receptor 49b-like			9	2	4	1	2	0	1	3	1	2	15	13	2	1	0	0
ACC_06483	conserved hypothetical protein			23	15	17	72	28	4	1	10	8	3	1	0	7	18	24	34
ACC_06484	serine/threonine/tyrosine-interacting protein-like		KOG1716	143	121	96	189	138	12	20	10	137	99	107	176	133	115	24	8
ACC_06485	cytochrome b5-like		KOG0537	45	17	25	38	30	4	9	17	36	33	135	27	38	257	106	82
ACC_06486	conserved hypothetical protein		KOG0921	4	2	1	3	3	1	1	0	5	7	1	15	43	2	1	166
ACC_06487	LIX1-like protein-like	K16673		95	69	32	93	49	21	29	20	116	48	52	145	126	128	44	17
ACC_06488	beta-hexosaminidase subunit beta-like	K12373	KOG2499	481	228	292	856	252	28	49	52	629	418	178	252	994	2003	1702	269
ACC_06489	2-oxoglutarate dehydrogenase, mitochondrial-like		KOG0450	17	10	7	8	5	1	0	2	6	25	0	2	8	5	2	1
ACC_06490	dolichyl-diphosphooligosaccharide--protein glycosy	K12666	KOG2291	950	615	334	575	631	114	189	226	884	1102	449	1353	5858	5226	259	94
ACC_06491	Paramyosin, short form		KOG0161	717	270	76	575	146	475	925	726	15192	5161	443	333	901	927	1773	1819
ACC_06492	sprouty-related, EVH1 domain-containing protein 2	K04703	KOG4590	188	94	103	137	79	3	2	14	206	187	19	100	94	71	28	10
ACC_06493	conserved hypothetical protein			0	0	1	1	1	0	0	0	2	0	1	1	0	0	0	0
ACC_06494	probable splicing factor, arginine/serine-rich 7-like	K13165	KOG4676	235	155	152	452	306	48	66	122	294	218	240	343	544	713	497	335
ACC_06495	conserved hypothetical protein	K12611	KOG2868	188	213	218	301	176	27	14	47	327	263	78	270	408	507	97	29

ACC_06496	phosphatidylinositol-4-phosphate 3-kinase C2 dom	K00923	KOG0905	287	232	197	209	189	12	27	13	384	526	78	173	572	531	46	14
ACC_06497	probable E3 ubiquitin-protein ligase HERC4-like	K10615	KOG0941	679	507	442	316	335	69	102	111	1596	1387	304	698	1689	1142	238	128
ACC_06498	esterase FE4-like		KOG1516	18	38	639	60	34	1	3	3	37	5	1	0	302	350	57	26
ACC_06499	thioredoxin, mitochondrial-like		KOG0910	51	24	45	35	63	0	2	2	13	38	8	64	48	40	0	0
ACC_06500	conserved hypothetical protein			158	115	132	206	202	4	4	13	163	142	38	29	38	32	12	1
ACC_06501	presequence protease, mitochondrial	K06972	KOG2019	554	364	344	565	637	32	80	71	692	824	171	527	2298	2827	214	92
ACC_06502	calcitonin receptor-like			6	2	4	5	1	0	0	0	9	24	4	16	2	4	5	0
ACC_06503	tuberin	K07207	KOG3687	840	591	512	619	572	59	94	104	1054	656	315	221	585	502	55	30
ACC_06504	calcium channel flower-like isoform 2		KOG4085	351	75	61	141	85	9	4	10	200	152	28	91	75	64	11	7
ACC_06505	calcitonin receptor	K04577	KOG4564	63	25	44	102	58	1	12	9	89	154	54	52	33	37	35	9
ACC_06506	hypothetical protein			0	0	0	0	1	0	0	0	0	0	0	0	1	0	1	0
ACC_06507	ecdysone-induced protein 75			13	0	1	13	3	0	0	1	1	6	0	0	2	9	11	21
ACC_06508	conserved hypothetical protein		KOG2294	612	542	586	879	550	108	212	216	314	268	202	117	145	255	1960	993
ACC_06509	conserved hypothetical protein			31	43	40	29	32	4	2	16	31	15	19	24	63	72	14	11
ACC_06510	bluestreak	K16777	KOG4625	506	283	317	336	317	36	57	65	596	454	127	159	245	279	30	10
ACC_06511	procollagen-lysine,2-oxoglutarate 5-dioxygenase 3-like		KOG1971	120	56	44	172	160	16	20	19	179	101	90	107	186	231	113	47
ACC_06512	oxidoreductase yrbE-like	K00010	KOG2741	45	26	51	135	126	8	23	15	392	3527	98	140	29488	1136	9	7
ACC_06513	conserved hypothetical protein			165	186	236	946	416	18	21	26	380	203	56	343	101	76	92	25
ACC_06514	adenylate cyclase type 5-like	K08045	KOG3619	168	129	105	107	38	18	38	24	193	400	28	25	8	14	9	6
ACC_06515	sodium- and chloride-dependent GABA transporter	K05039	KOG3660	345	139	205	185	136	19	20	28	517	370	122	546	264	91	57	18
ACC_06516	1-phosphatidylinositol-4,5-bisphosphate phosphod	K05857	KOG1265	45	100	66	104	63	148	164	250	2692	1660	0	58	0	1	1	0
ACC_06517	reticulocalbin-2-like		KOG4223	381	398	356	606	456	24	28	53	434	249	94	247	348	272	21	15
ACC_06518	chaperone protein DnaJ-like		KOG0715	141	78	73	96	176	1	16	6	72	103	81	272	68	42	5	2
ACC_06519	acyl-CoA	K13514	KOG3234	181	109	84	190	251	27	26	36	266	200	223	331	411	530	48	30
ACC_06520	conserved hypothetical protein		KOG0515	444	242	320	308	111	62	95	120	763	494	217	217	258	383	679	397
ACC_06521	ESF1 homolog		KOG2318	553	357	294	459	577	81	225	269	322	269	387	301	745	1364	1348	878
ACC_06522	REST corepressor 3-like isoform 1		KOG1194	156	101	99	143	89	10	16	10	173	187	39	130	210	188	53	17
ACC_06523	transmembrane and TPR repeat-containing protein 4-like		KOG4626	199	156	143	304	349	14	37	46	238	160	178	108	213	210	27	8
ACC_06524	DNA polymerase epsilon catalytic subunit A	K02324	KOG1798	216	123	146	172	170	20	45	42	293	184	120	84	430	439	226	102
ACC_06525	conserved hypothetical protein	K02678	KOG3806	765	373	527	1274	503	113	179	186	876	564	449	288	135	305	762	457
ACC_06526	potassium/sodium hyperpolarization-activated cyclic nucleo		KOG0498	95	40	59	64	62	3	3	7	67	39	23	27	19	8	0	1
ACC_06527	pseudouridine-metabolizing bifunctional protein C1861.05-li		KOG3009	82	64	73	184	166	7	14	16	71	49	65	71	68	72	10	6
ACC_06528	Putative palmitoyltransferase ZDHHC22			3	3	6	6	10	0	0	1	3	2	10	4	8	7	0	0
ACC_06529	hypoxia up-regulated protein 1-like	K09486	KOG0104	3753	2099	1596	3138	3571	685	1195	1505	2084	2077	1522	2211	4933	6293	2791	1455
ACC_06530	SUMO-activating enzyme subunit 2	K10685	KOG2013	217	143	146	259	226	15	16	25	284	311	109	437	864	1104	92	29
ACC_06531	dyslexia susceptibility 1 candidate gene 1 protein homolog		KOG0553	38	19	33	69	42	6	11	9	28	12	38	43	10	10	10	6
ACC_06532	pentatricopeptide repeat-containing protein 1-like			493	296	324	332	439	40	45	63	217	192	267	474	508	502	96	53
ACC_06533	conserved hypothetical protein			103	79	113	123	108	7	20	24	99	63	95	183	223	315	73	47
ACC_06534	carbonyl reductase	K00079	KOG1208	116	92	86	224	268	9	9	7	261	157	149	380	474	293	15	6
ACC_06535	UPF0430 protein CG31712-like	K13173	KOG1029	621	572	544	587	277	151	209	243	1231	566	227	232	379	513	715	446
ACC_06536	conserved hypothetical protein		KOG0147	6376	4154	3556	5423	3619	1181	2210	2240	5052	3725	2959	1420	2813	4886	13969	11697
ACC_06537	prolyl 4-hydroxylase subunit alpha-2-like	K00472	KOG1591	133	77	82	209	61	8	8	13	137	108	45	64	124	111	95	21
ACC_06538	waprin-Phi1-like isoform 2		KOG4802	115	628	1034	3232	2253	38	50	84	685	176	108	210	32	22	0	4
ACC_06539	receptor-binding cancer antigen expressed on SiSo cells-like			105	131	125	177	112	17	13	17	180	83	47	109	133	159	47	12
ACC_06540	protein dopey-1 homolog		KOG3613	1076	767	478	904	627	342	635	773	1318	1262	327	302	830	706	464	180
ACC_06541	metallophosphoesterase 1 homolog		KOG3662	140	87	59	126	124	13	12	10	630	351	115	605	184	134	13	3
ACC_06542	conserved hypothetical protein			7	5	20	35	13	1	1	1	7	2	0	0	0	3	3	7
ACC_06543	conserved hypothetical protein		KOG4805	700	697	692	850	372	144	207	245	755	738	195	174	453	458	443	212
ACC_06544	conserved hypothetical protein			41	32	35	76	50	21	47	47	138	57	73	133	39	22	22	10
ACC_06545	LOW QUALITY PROTEIN	K14809	KOG0345	375	195	190	273	303	46	134	161	210	170	130	188	448	494	272	134
ACC_06546	conserved hypothetical protein		KOG2375	1275	903	1048	2082	1163	395	554	603	12986	15991	681	949	34190	4233	1117	874
ACC_06547	conserved hypothetical protein			6	6	3	11	8	1	0	0	143	5	5	13	8	13	3	0
ACC_06548	conserved hypothetical protein	K13118	KOG2627	262	235	272	393	296	38	37	67	261	180	182	254	233	331	108	44
ACC_06549	t-box transcription factor TBX10-like		KOG3586	52	39	45	68	20	3	9	12	79	43	16	1	6	4	11	7
ACC_06550	glucosidase 2 subunit beta-like	K08288	KOG2397	1376	853	445	693	492	198	238	283	1087	1001	428	899	2754	3506	1053	768
ACC_06551	conserved hypothetical protein	K15309	KOG4217	344	264	347	405	65	96	124	200	147	146	112	45	24	69	709	555
ACC_06552	major facilitator superfamily domain-containing protein 12-l		KOG4830	16	6	10	47	23	0	4	10	15	10	9	3	22	58	40	5

ACC_06553	adenosine deaminase	K01488	KOG1097	82	56	73	56	69	4	9	10	68	63	77	187	206	243	23	12
ACC_06554	programmed cell death protein 6-like isoform 2		KOG0037	154	85	88	443	437	8	13	15	414	217	196	823	395	412	13	6
ACC_06555	conserved hypothetical protein			1758	1289	1135	1255	1085	376	771	907	1337	1088	650	247	470	644	897	435
ACC_06556	probable tyrosyl-DNA phosphodiesterase-like	K10862	KOG2031	465	332	260	582	486	55	117	110	531	230	327	278	333	470	216	121
ACC_06557	arylsulfatase J-like		KOG3867	379	310	369	284	84	171	189	272	148	234	67	64	210	304	260	119
ACC_06558	dipeptidase 1-like	K01273	KOG4127	50	14	20	52	12	4	1	8	28	13	4	9	0	5	11	8
ACC_06559	conserved hypothetical protein			17	2	1	7	3	0	0	2	11	0	1	29	142	873	198	654
ACC_06560	probable asparagine--tRNA ligase, mitochondrial-lil	K01893	KOG0554	128	102	94	133	216	7	6	6	242	122	126	410	279	351	15	3
ACC_06561	homeobox protein orthopedia-like		KOG0484	1	1	1	2	0	0	0	0	0	0	0	0	0	0	1	5
ACC_06562	conserved hypothetical protein		KOG3209	730	540	409	986	288	308	417	716	557	412	524	152	29	86	428	534
ACC_06563	conserved hypothetical protein			239	188	188	644	290	34	58	65	896	131	102	88	62	73	59	21
ACC_06564	conserved hypothetical protein		KOG2072	5175	3342	3474	4207	1673	836	1433	1865	7686	4015	2013	1820	1523	1284	1800	1753
ACC_06565	periostin-like		KOG2469	523	366	389	307	355	33	53	61	450	216	158	419	269	435	122	67
ACC_06566	irregular chiasm C-roughest protein-like isoform 1		KOG3513	92	72	87	148	31	5	6	6	76	48	41	16	12	9	44	28
ACC_06567	conserved hypothetical protein			11	0	2	1	2	2	0	0	15	3	0	1	6	65	73	6
ACC_06568	Down syndrome cell adhesion molecule-like protein CG4225		KOG3513	63	39	42	64	15	2	3	2	29	15	0	0	4	1	17	13
ACC_06569	Down syndrome cell adhesion molecule-like protein CG4225		KOG3513	37	10	13	36	7	0	1	4	8	9	0	0	3	1	3	3
ACC_06570	nuclear pore complex protein Nup50	K14295	KOG2724	518	319	302	553	412	80	113	148	393	401	276	673	512	697	340	178
ACC_06571	fatty acid binding protein			184	186	160	1111	254	26	57	87	271	34	249	664	9	25	11	22
ACC_06572	conserved hypothetical protein			85	37	38	52	15	3	3	4	104	271	20	36	11	13	7	13
ACC_06573	otopetrin-3-like		KOG4740	11	5	6	15	6	1	3	4	6	1	1	0	0	0	0	0
ACC_06574	phospholipase A2 inhibitor subunit B-like		KOG4194	118	60	60	145	97	12	12	18	415	883	41	52	44	17	10	2
ACC_06575	conserved hypothetical protein		KOG4506	309	252	230	712	483	42	65	76	396	258	216	325	494	561	106	53
ACC_06576	protein jagged-1	K06052	KOG1219	14	7	4	19	2	1	0	0	44	40	1	17	23	37	36	13
ACC_06577	tubulin beta-4 chain-like	K07375	KOG1375	1	0	0	0	0	0	0	0	0	0	0	0	3	2	2	9
ACC_06578	serine/threonine-protein kinase SBK1-like		KOG1345	194	127	191	842	186	14	15	35	55	56	4	2	9	1	25	30
ACC_06579	conserved hypothetical protein		KOG3513	97	30	34	191	43	4	6	6	34	28	18	9	4	8	15	9
ACC_06580	probable complex I intermediate-associated protein 30, mitc		KOG2435	106	68	68	121	209	7	13	19	158	91	112	235	370	460	19	12
ACC_06581	U3 small nucleolar RNA-associated protein 18 hom	K14553	KOG2055	343	217	229	467	455	41	104	122	444	268	313	803	640	925	298	140
ACC_06582	sperm-associated antigen 6-like isoform 1			6	4	5	7	9	0	1	0	4	2	4	3	7	2	1	0
ACC_06583	translocase of outer membrane 7		KOG4449	79	81	76	143	38	7	11	20	32	19	77	88	173	194	47	39
ACC_06584	golgi-specific brefeldin A-resistance guanine nucleotide exch		KOG0928	1472	787	632	1364	1240	307	644	628	1755	1471	796	569	723	751	1182	678
ACC_06585	zinc transporter 7-like	K14692	KOG1484	84	80	31	52	64	3	6	11	145	130	25	88	419	332	28	3
ACC_06586	bcl-2-related ovarian killer protein homolog A-like		KOG4728	42	67	25	38	33	31	36	45	65	97	104	65	111	59	32	7
ACC_06587	N-alpha-acetyltransferase 40-like		KOG2488	91	57	90	157	138	9	14	26	131	62	83	172	172	212	33	9
ACC_06588	coiled-coil domain-containing protein 135-like isoform 1			42	26	38	45	38	1	4	5	55	51	10	21	184	131	36	8
ACC_06589	merlin-like	K16684	KOG3529	473	384	298	308	219	35	45	55	752	409	182	262	365	355	114	64
ACC_06590	conserved hypothetical protein			98	83	108	241	225	13	31	37	54	55	254	120	31	14	19	7
ACC_06591	conserved hypothetical protein		KOG0250	1107	641	670	759	682	152	359	354	687	619	320	359	822	874	517	251
ACC_06592	riboflavin transporter 2-like		KOG4255	232	176	259	127	187	4	9	9	203	236	101	526	509	326	27	1
ACC_06593	conserved hypothetical protein			104	97	113	175	84	11	42	39	99	51	42	14	12	4	23	6
ACC_06594	conserved hypothetical protein			2331	1549	1325	1853	1695	871	1697	1991	2756	2092	1423	798	369	375	370	122
ACC_06595	conserved hypothetical protein		KOG0199	637	441	499	524	215	42	84	78	798	688	122	231	379	498	152	41
ACC_06596	synaptobrevin homolog YKT6-like	K08516	KOG0861	282	112	79	260	375	7	10	20	408	136	140	543	329	414	24	3
ACC_06597	conserved hypothetical protein			14	35	25	197	89	9	1	10	23	15	0	0	13	17	2	4
ACC_06598	facilitated trehalose transporter Tret1-like		KOG0254	48	21	22	74	40	3	4	10	70	145	21	44	19	21	13	7
ACC_06599	leucine-rich repeat-containing protein 57-like		KOG0444	78	71	33	76	67	1	5	0	50	80	36	44	124	77	1	4
ACC_06600	protein KRI1 homolog	K14786	KOG2409	615	463	407	579	704	96	252	249	233	205	301	255	370	626	830	528
ACC_06601	nucleolar protein 16-like isoform 2		KOG4706	279	250	165	249	274	29	68	90	279	181	88	373	1294	1670	275	216
ACC_06602	slit homolog 3 protein-like		KOG4194	192	85	77	372	89	9	13	18	103	97	10	101	8	23	31	8
ACC_06603	conserved hypothetical protein	K14437	KOG0384	13882	10567	9642	10616	7855	2600	4685	5223	9883	7505	4204	1197	3805	5343	9410	4523
ACC_06604	AGAP010957-PA	K02183	KOG0027	7025	3109	2755	3066	1849	653	1414	1396	7630	4129	684	3198	2829	2754	2028	970
ACC_06605	conserved hypothetical protein			19	18	23	106	34	6	9	3	204	197	141	113	4	11	7	6
ACC_06606	tudor and KH domain-containing protein-like			73	36	38	85	50	8	4	18	308	317	42	67	89	97	30	6
ACC_06607	tudor and KH domain-containing protein-like isoform 2		KOG2279	94	51	37	100	78	5	11	16	241	236	38	104	159	162	16	6
ACC_06608	slit homolog 1 protein-like		KOG4194	490	301	296	551	193	15	20	36	677	1254	137	192	37	21	14	15
ACC_06609	major royal jelly protein 1-like			17	24	33	207	127	22	16	9	1904	2353	28	257	169	37	1	9

ACC_06610	hemicentin-2-like		KOG3513	121	111	115	174	51	39	75	66	237	226	4	10	10	9	27	15
ACC_06611	vesicle-associated membrane protein 4-like isoform 1		KOG0860	121	98	82	251	67	37	37	47	216	105	101	142	82	103	176	63
ACC_06612	conserved hypothetical protein			51	18	13	66	23	9	9	12	103	101	30	66	8	37	39	9
ACC_06613	atrial natriuretic peptide receptor 1-like	K12323	KOG1023	849	316	437	777	184	37	45	60	185	89	16	11	116	205	290	29
ACC_06614	stAR-related lipid transfer protein 7, mitochondrial-like		KOG2761	146	136	129	155	136	12	31	32	307	283	77	216	926	1187	124	40
ACC_06615	ligand-gated ion channel pHCl isoform 3		KOG3644	554	227	258	441	177	28	63	50	200	128	228	36	26	17	71	22
ACC_06616	conserved hypothetical protein	K08836	KOG0579	1071	783	712	668	354	271	525	619	1335	1220	312	293	430	350	573	365
ACC_06617	casein kinase I isoform alpha	K08957	KOG1163	307	161	140	156	115	40	52	71	621	807	64	432	718	583	256	85
ACC_06618	stargazin related protein STG-1			53	37	29	110	54	4	12	12	35	17	5	0	1	0	5	2
ACC_06619	adenylosuccinate lyase-like	K01756	KOG2700	141	87	104	278	136	75	58	112	439	266	179	257	867	892	337	205
ACC_06620	conserved hypothetical protein			101	56	55	69	19	2	2	2	42	16	2	7	0	7	4	0
ACC_06621	conserved hypothetical protein		KOG1923	104	210	86	39	30	83	94	94	223	217	36	45	12	71	20	9
ACC_06622	conserved hypothetical protein	K09048	KOG0709	507	456	371	406	153	72	84	121	841	895	172	536	940	765	358	215
ACC_06623	conserved hypothetical protein			7662	4391	6989	30878	10622	494	613	1038	5313	2024	3759	14320	20	75	61	64
ACC_06624	conserved hypothetical protein		KOG0498	2802	1948	2244	4400	859	113	134	209	831	770	524	129	15	27	103	53
ACC_06625	conserved hypothetical protein		KOG0501	22	20	35	32	6	0	0	0	15	28	24	8	1	0	0	0
ACC_06626	conserved hypothetical protein		KOG0161	668	441	433	613	626	104	250	285	349	177	327	487	427	348	236	197
ACC_06627	facilitated trehalose transporter Tret1-2 homolog		KOG0254	40	23	18	145	54	10	16	12	285	318	259	330	433	298	276	197
ACC_06628	HEAT repeat-containing protein 3-like			209	130	127	180	189	17	34	39	173	149	89	126	343	426	55	42
ACC_06629	iron-sulfur cluster co-chaperone protein HscB, mitochondria		KOG3192	46	32	33	53	64	3	20	13	45	24	69	58	87	84	35	27
ACC_06630	potassium voltage-gated channel protein Shal	K05321	KOG4390	690	218	280	1109	198	19	24	77	171	147	121	17	11	10	28	23
ACC_06631	conserved hypothetical protein		KOG0532	12	2	6	7	6	2	1	3	6	20	66	64	8	3	10	3
ACC_06632	sodium bicarbonate cotransporter 3-like isoform 1		KOG1172	1302	976	1059	1945	901	203	267	366	1330	933	397	2153	195	169	44	22
ACC_06633	tRNA-specific adenosine deaminase 2-like	K15441	KOG1018	169	80	95	271	221	15	57	78	118	39	138	140	69	142	106	15
ACC_06634	conserved hypothetical protein			455	360	247	508	504	110	263	286	519	243	333	595	260	152	88	44
ACC_06635	conserved hypothetical protein			116	85	71	147	110	9	11	9	170	116	59	166	213	153	17	5
ACC_06636	UPF0676 protein C1494.01-like		KOG0143	287	158	151	291	150	77	73	81	680	323	488	1184	326	460	255	116
ACC_06637	conserved hypothetical protein			0	0	0	0	0	0	0	1	0	1	0	0	0	2	0	0
ACC_06638	conserved hypothetical protein			1	0	0	0	2	0	0	0	3	20	0	0	2	0	0	0
ACC_06639	LOW QUALITY PROTEIN	K16175	KOG0472	647	413	357	665	397	127	183	181	1167	914	216	255	415	526	399	163
ACC_06640	conserved hypothetical protein			2601	1693	1699	2209	1260	509	772	796	3090	2362	719	977	922	915	487	185
ACC_06641	serotonin receptor 7		KOG4220	32	6	5	30	15	0	2	4	15	8	2	0	1	0	0	0
ACC_06642	serotonin receptor 7	K04163	KOG4220	31	13	8	20	5	0	0	2	15	7	0	2	1	0	4	0
ACC_06643	coiled-coil domain-containing protein 147-like		KOG0161	2	3	4	6	9	2	5	1	5	5	35	41	0	1	1	1
ACC_06644	tropomyosin-2-like		KOG1003	9	8	14	6	4	0	0	1	1	1	0	0	0	0	0	0
ACC_06645	sodium- and chloride-dependent glycine transporter 2		KOG3659	56	28	27	51	12	1	3	4	28	26	2	1	0	0	0	0
ACC_06646	conserved hypothetical protein		KOG1144	39	23	36	61	42	8	13	12	93	27	30	8	32	24	37	44
ACC_06647	LOW QUALITY PROTEIN	K00894	KOG4720	135	84	97	110	199	6	9	13	120	97	81	350	147	114	8	3
ACC_06648	conserved hypothetical protein	K08770	KOG0004	1764	947	1141	4168	3447	173	174	275	10024	6274	3464	15090	8112	6920	424	116
ACC_06649	1-acylglycerophosphocholine O-acyltransferase 1-li	K13510		125	77	61	83	28	11	9	13	170	95	184	138	29	17	6	4
ACC_06650	LOW QUALITY PROTEIN	K00502	KOG3820	38	27	42	123	45	4	1	10	52	29	3	0	1	6	1	0
ACC_06651	conserved hypothetical protein			3	2	5	5	2	0	1	2	10	13	70	461	1	4	9	2
ACC_06652	semaphorin-2A		KOG3611	501	122	103	213	128	28	58	77	118	69	36	34	43	75	172	215
ACC_06653	conserved hypothetical protein			3064	1510	1888	4475	1707	273	424	525	1243	319	122	44	1	6	27	21
ACC_06654	conserved hypothetical protein		KOG3581	65	51	85	139	87	17	16	22	294	194	2162	1578	24	34	34	16
ACC_06655	protein hairy	K09090	KOG4304	82	58	120	136	35	28	13	24	151	408	37	156	110	108	243	86
ACC_06656	conserved hypothetical protein		KOG3940	15	12	9	10	1	1	2	1	54	77	22	18	1	1	9	4
ACC_06657	conserved hypothetical protein		KOG0516	366	362	393	361	71	71	63	188	1659	1372	92	296	166	73	239	107
ACC_06658	conserved hypothetical protein			84	45	60	73	84	8	14	12	122	74	52	219	166	160	23	21
ACC_06659	kinesin heavy chain isoform 1	K10396	KOG0240	2131	1246	1188	1778	936	234	399	526	1949	1896	370	877	1054	733	565	253
ACC_06660	conserved hypothetical protein		KOG1819	13	6	16	15	0	1	1	3	17	40	7	5	3	4	43	60
ACC_06661	conserved hypothetical protein		KOG3546	3	1	1	10	4	1	0	0	11	19	0	2	2	6	6	4
ACC_06662	alpha-2B adrenergic receptor-like		KOG4220	108	26	34	122	41	2	2	5	16	23	1	2	1	2	10	6
ACC_06663	conserved hypothetical protein			15	8	24	33	37	5	5	11	59	37	22	25	5	16	3	3
ACC_06664	tyrosine kinase receptor Cad96Ca-like		KOG0200	54	43	36	87	42	15	19	30	354	317	101	143	238	204	64	42
ACC_06665	conserved hypothetical protein			0	0	0	0	0	0	0	0	1	0	0	0	0	588	1293	903
ACC_06666	conserved hypothetical protein			0	0	0	0	0	0	0	0	0	0	0	0	0	900	1397	353

ACC_06667	conserved hypothetical protein			1	3	6	6	1	1	0	2	10	28	0	6	28	239	54	6
ACC_06668	conserved hypothetical protein			1	1	0	0	0	0	0	1	2	17	6	1	0	456	1283	275
ACC_06669	conserved hypothetical protein			1	0	1	2	0	0	1	0	0	1	3	0	0	511	1006	122
ACC_06670	conserved hypothetical protein			0	0	0	0	0	0	0	0	2	1	1	1	0	404	502	16
ACC_06671	conserved hypothetical protein			0	1	0	5	1	4	3	4	10	7	3	0	1	80	66	6
ACC_06672	39S ribosomal protein L2, mitochondrial	K02886	KOG0438	195	149	126	534	226	59	72	88	302	180	198	267	592	574	189	155
ACC_06673	thioredoxin-dependent peroxide reductase, mitoch	K03386	KOG0852	436	319	295	619	560	63	71	108	338	232	329	870	1595	1448	152	83
ACC_06674	tetratricopeptide repeat protein 39B-like		KOG3783	324	190	172	414	342	21	41	37	845	327	576	1405	596	391	26	3
ACC_06675	LOW QUALITY PROTEIN		KOG3513	548	420	87	194	95	883	2304	1699	9278	3025	250	50	167	747	3332	5699
ACC_06676	lysosomal-trafficking regulator-like		KOG1786	780	536	444	593	478	362	473	508	3081	4079	189	251	1249	1579	271	81
ACC_06677	coiled-coil domain-containing protein 85C-like isoform 1		KOG3819	106	58	49	144	63	36	30	39	174	101	142	73	370	463	292	167
ACC_06678	neural-cadherin		KOG4289	590	368	369	560	118	19	40	32	263	272	86	31	8	10	132	97
ACC_06679	conserved hypothetical protein		KOG1427	64	114	122	183	158	22	37	33	114	37	1198	759	15	25	78	18
ACC_06680	RUN and FYVE domain-containing protein 2-like		KOG4381	1129	678	788	703	362	32	59	77	1005	828	109	141	78	116	42	24
ACC_06681	conserved hypothetical protein	K04134	KOG4220	404	192	197	274	62	11	18	27	57	39	2	1	0	0	3	1
ACC_06682	conserved hypothetical protein			6	4	0	0	3	1	1	1	3	3	0	0	0	0	2	3
ACC_06683	conserved hypothetical protein			1039	647	731	1825	911	43	70	81	727	335	163	293	88	138	42	20
ACC_06684	conserved hypothetical protein	K12495	KOG0931	553	435	373	704	353	210	400	595	1159	1092	456	543	226	330	399	103
ACC_06685	galactosylgalactosylxylosylprotein 3-beta-glucuron	K10158	KOG1476	324	202	179	318	308	28	48	61	442	295	205	727	369	273	61	20
ACC_06686	conserved hypothetical protein			467	395	348	784	344	422	612	752	940	589	307	293	101	95	1322	1007
ACC_06687	serotonin receptor 7			4	3	1	5	2	1	0	2	3	1	0	0	0	0	0	0
ACC_06688	death-associated protein kinase 1-like isoform 2	K08803	KOG4177	229	157	132	99	100	9	10	10	154	109	70	43	103	118	5	2
ACC_06689	homeobox protein goosecoid isoform B	K09324	KOG2251	3	2	3	7	1	0	1	0	1	0	0	0	0	1	5	1
ACC_06690	DNA-binding protein D-ETS-6-like		KOG3806	0	0	2	1	0	0	1	0	2	2	2	0	1	2	1	0
ACC_06691	cytochrome b-c1 complex subunit Rieske, mitocho	K00411	KOG1671	1087	439	508	1881	1231	125	159	216	2447	736	736	2040	1446	1597	489	271
ACC_06692	probable Ufm1-specific protease 2-like isoform 1		KOG2433	204	120	92	156	185	16	23	21	416	371	69	530	433	394	17	6
ACC_06693	heterogeneous nuclear ribonucleoprotein 27C-like	K14411	KOG4205	1830	908	984	2399	727	161	271	382	976	990	541	476	991	1637	1762	956
ACC_06694	gastrin-releasing peptide receptor-like			8	7	7	13	7	3	5	3	49	17	1	4	9	52	62	7
ACC_06695	gastrin-releasing peptide receptor-like		KOG4219	16	5	4	15	3	0	1	2	31	10	8	1	2	14	14	0
ACC_06696	myrosinase 1-like		KOG0626	15	20	28	46	37	22	14	17	37	43	0	3	6786	11173	764	34
ACC_06697	ecdysteroid UDP-glucosyltransferase-like		KOG1192	316	310	396	887	1042	11	16	19	623	327	4074	7090	20	95	5	16
ACC_06698	charged multivesicular body protein 7-like		KOG2911	184	140	96	264	410	18	105	118	179	127	170	306	259	222	93	53
ACC_06699	conserved hypothetical protein			26	24	23	27	33	4	3	6	17	11	3	13	43	49	17	12
ACC_06700	probable RISC-loading complex subunit BRAFLDRAFT_24288		KOG3732	90	69	55	98	81	10	16	12	127	71	78	119	108	88	28	11
ACC_06701	hydroxyacid oxidase 1-like	K11517	KOG0538	31	4	8	44	23	3	4	7	209	378	0	6	1643	1253	212	225
ACC_06702	protein grainyhead	K09275	KOG4091	17	22	15	31	8	10	2	3	110	122	9	27	27	34	90	64
ACC_06703	conserved hypothetical protein			197	110	196	144	123	26	22	36	276	172	104	79	380	70	12	3
ACC_06704	conserved hypothetical protein			2	1	1	1	1	0	4	1	9	1	2	18	1	13	10	2
ACC_06705	dihydropteridine reductase-like	K00357	KOG4022	180	82	76	137	65	21	14	22	572	232	120	616	274	223	25	26
ACC_06706	integrator complex subunit 12-like	K13149	KOG4323	278	187	181	227	193	30	55	62	227	152	101	203	270	321	154	39
ACC_06707	cytochrome P450 9e2		KOG0158	11	11	14	42	20	2	2	2	136	215	35	34	31	24	6	1
ACC_06708	cytochrome P450 9e2		KOG0158	14	14	33	181	39	9	15	12	139	423	22	2	1122	514	470	61
ACC_06709	membralin-like		KOG3629	360	251	266	238	45	42	41	74	405	396	161	122	99	111	333	90
ACC_06710	membralin-like			50	64	36	36	24	10	5	12	122	122	20	36	27	41	22	5
ACC_06711	cytochrome P450 9e2-like		KOG0158	12	14	12	51	9	9	2	5	61	359	7	3	21	1	2	0
ACC_06712	zinc finger protein 423 homolog		KOG3623	21	31	21	34	9	4	5	5	111	72	53	104	8	38	7	12
ACC_06713	peroxisomal biogenesis factor 3-like	K13336	KOG4444	270	204	162	219	309	19	46	56	529	346	210	572	430	424	138	59
ACC_06714	semaphorin-1A-like			87	48	43	55	15	9	6	11	28	52	19	16	1	3	46	11
ACC_06715	conserved hypothetical protein			0	0	0	0	0	0	0	0	0	0	0	0	212	199	67	9
ACC_06716	conserved hypothetical protein			0	0	0	1	0	0	0	0	0	0	1	0	170	171	27	2
ACC_06717	general transcription factor IIE subunit 1	K03136	KOG2593	58	59	68	149	131	15	16	25	97	66	68	76	124	196	17	18
ACC_06718	conserved hypothetical protein		KOG0612	270	89	75	274	76	32	75	63	631	188	106	33	19	112	206	193
ACC_06719	conserved hypothetical protein		KOG3598	1383	394	417	963	207	19	30	55	281	166	86	18	6	6	31	32
ACC_06720	pheromone-binding protein-related protein 3-like			683	110	128	526	202	14	33	54	230	42	1465	41	61	89	42	17
ACC_06721	5'-AMP-activated protein kinase subunit gamma-2			82	30	11	25	28	9	8	16	59	53	15	13	3	4	14	1
ACC_06722	exosome complex exonuclease RRP43-like	K12586	KOG1613	105	113	89	151	163	14	24	19	206	123	76	265	432	471	45	19
ACC_06723	conserved hypothetical protein			11	12	5	13	7	2	5	5	51	40	2	7	101	35	8	2

ACC_06724	conserved hypothetical protein			11	7	7	6	8	2	0	0	24	43	17	67	139	334	152	44
ACC_06725	sodium channel protein Nach-like		KOG4294	21	18	32	26	39	1	5	5	51	63	43	144	69	84	12	4
ACC_06726	serine/threonine-protein kinase 32B-like	K08793	KOG0598	282	120	167	412	132	29	33	68	308	67	42	77	38	54	108	59
ACC_06727	conserved hypothetical protein		KOG4196	40	35	62	48	9	4	0	4	132	153	0	0	0	0	1	0
ACC_06728	actin-binding LIM protein 1-like	K07520	KOG1044	287	183	195	211	88	18	37	58	245	141	50	51	117	172	112	35
ACC_06729	Krueppel homologous protein 1		KOG2462	14	20	50	26	21	12	30	29	221	423	6	72	363	317	81	27
ACC_06730	rab3 GTPase-activating protein catalytic subunit		KOG2390	173	121	125	185	154	13	28	30	93	63	79	97	80	90	21	6
ACC_06731	TATA box binding protein-related factor 2 isoform :	K03120	KOG3302	399	189	156	306	242	63	130	154	251	196	85	90	272	373	935	924
ACC_06732	innexin inx7			6	5	5	20	13	0	0	0	33	9	8	4	121	76	5	0
ACC_06733	xaa-Pro dipeptidase-like		KOG2737	211	126	132	172	166	20	22	24	321	307	124	555	724	591	19	7
ACC_06734	conserved hypothetical protein		KOG2462	2	1	1	1	0	0	0	0	1	1	0	2	0	0	1	0
ACC_06735	inositol monophosphatase 2-like	K01092	KOG2951	121	70	59	85	52	8	16	17	883	199	199	658	127	7	2	3
ACC_06736	conserved hypothetical protein		KOG1015	529	378	330	507	189	68	59	92	1075	1067	163	394	248	283	167	115
ACC_06737	cytochrome P450 18a1	K14985	KOG0156	3	1	3	7	4	2	1	1	2	5	5	2	51	87	28	78
ACC_06738	conserved hypothetical protein		KOG0489	156	69	82	90	73	16	23	20	101	72	16	26	86	161	217	29
ACC_06739	conserved hypothetical protein			143	81	123	141	32	7	5	15	57	43	15	7	3	3	26	29
ACC_06740	conserved hypothetical protein			684	527	692	494	95	64	74	137	785	896	197	115	60	79	286	136
ACC_06741	conserved hypothetical protein		KOG0384	406	215	286	448	123	65	80	98	1005	1165	137	103	87	74	208	164
ACC_06742	dentin matrix protein 4-like		KOG3829	395	271	239	400	109	145	222	272	1351	1372	321	269	297	393	681	541
ACC_06743	conserved hypothetical protein			0	0	0	1	0	0	0	0	0	0	0	0	0	1	0	1
ACC_06744	ornithine aminotransferase, mitochondrial-like		KOG1402	2	1	1	1	2	0	0	2	3	2	1	1	89	250	24	7
ACC_06745	protein QIL1-like isoform 2			206	193	151	229	257	11	15	24	221	101	159	524	225	296	32	20
ACC_06746	apoptosis inhibitor 5-like		KOG2213	506	387	314	320	273	29	32	33	626	432	166	169	810	1203	132	64
ACC_06747	glutamate receptor, ionotropic kainate 2-like		KOG1052	19	5	8	20	8	4	4	3	30	70	135	54	13	35	28	0
ACC_06748	homeobox protein OTX-like		KOG0489	0	0	2	0	0	0	0	0	0	0	0	0	1	0	7	0
ACC_06749	conserved hypothetical protein	K09304	KOG0489	220	263	274	29	8	75	81	99	46	61	4	0	1	0	26	13
ACC_06750	membrane metallo-endopeptidase-like 1-like		KOG3624	75	66	73	46	25	50	69	76	815	390	343	391	18	2	2	4
ACC_06751	innexin inx3 isoform 2			357	76	139	245	112	27	32	62	248	55	11	101	75	119	155	156
ACC_06752	protocadherin-like wing polarity protein stan-like	K04600	KOG4289	288	217	235	371	139	22	17	29	147	119	76	31	75	145	107	31
ACC_06753	probable glutamine-dependent NAD(+) synthetase·	K01950	KOG2303	236	149	134	314	207	53	41	53	353	269	164	235	246	352	66	41
ACC_06754	conserved hypothetical protein			192	39	65	98	17	6	5	4	210	407	23	50	48	60	94	55
ACC_06755	conserved hypothetical protein		KOG0242	464	429	282	388	605	69	238	234	309	447	291	126	549	689	897	497
ACC_06756	elongation of very long chain fatty acids protein AAEL00800		KOG3071	128	90	40	119	30	7	18	10	257	154	115	578	1132	558	203	39
ACC_06757	elongation of very long chain fatty acids protein AAEL00800		KOG3071	16	9	4	8	3	1	1	3	56	33	11	124	272	123	9	3
ACC_06758	elongation of very long chain fatty acids protein AAEL00800		KOG3071	92	18	14	51	42	32	60	44	410	251	28	139	25	155	192	110
ACC_06759	elongation of very long chain fatty acids protein 4-like		KOG3071	0	0	1	0	0	0	0	0	2	0	1	0	45	79	19	7
ACC_06760	conserved hypothetical protein			35	21	5	13	13	8	14	24	93	59	38	301	39	25	8	5
ACC_06761	potassium voltage-gated channel protein Shal-like, partial		KOG4390	223	76	129	278	37	8	17	27	57	47	38	4	2	2	19	21
ACC_06762	RNA exonuclease 4-like		KOG2249	89	78	75	169	115	11	23	21	76	46	35	77	181	305	60	13
ACC_06763	ELAV-like protein 2-like	K13208	KOG0145	1695	1081	1423	4208	1121	134	208	283	601	390	198	19	1	40	294	134
ACC_06764	conserved hypothetical protein			2450	1112	1115	1413	799	435	975	1071	1134	774	644	2701	1979	1301	3590	2486
ACC_06765	elongation factor G, mitochondrial-like isoform 1	K02355	KOG0465	601	317	333	635	692	59	64	68	830	742	237	833	1552	1860	194	47
ACC_06766	sodium bicarbonate cotransporter 3-like isoform 1		KOG1172	53	67	42	70	16	5	4	7	113	132	19	389	17	13	4	3
ACC_06767	vanin-like protein 1-like			11	6	6	3	2	0	1	2	10	4	0	3	3	3	3	0
ACC_06768	cys-loop ligand-gated ion channel subunit 8916	K05175	KOG3642	61	42	53	271	202	4	3	4	26	8	0	0	0	2	0	1
ACC_06769	elongation of very long chain fatty acids protein 6-like		KOG3072	4	3	3	0	1	0	0	0	288	53	1	0	1	0	0	0
ACC_06770	cytochrome P450 302a1, mitochondrial		KOG0159	9	10	5	7	5	4	5	4	40	5	6	36	53	47	6	8
ACC_06771	adenosine deaminase CECR1-like		KOG1838	7	14	7	15	5	1	2	0	92	234	25	137	88	95	4	0
ACC_06772	nephrin-like isoform 1			25	9	12	45	15	0	2	3	12	3	1	0	17	5	1	1
ACC_06773	nephrin-like isoform 1		KOG3515	55	20	24	77	17	3	0	4	24	14	1	0	46	29	3	2
ACC_06774	major antigen		KOG0161	5	6	12	12	8	1	3	3	5	101	1	0	2	5	2	2
ACC_06775	protein l(2)37Cc-like		KOG3083	453	248	221	960	581	52	77	110	506	393	526	703	2996	5836	887	528
ACC_06776	inosine triphosphate pyrophosphatase-like isoform	K01519	KOG3222	53	29	27	66	89	3	15	13	41	37	44	103	301	372	32	28
ACC_06777	protein ACN9 homolog, mitochondrial-like		KOG4100	47	52	47	62	69	4	12	7	80	60	34	106	124	138	20	7
ACC_06778	conserved hypothetical protein		KOG3627	2	0	1	2	1	0	0	0	4	6	0	5	8	162	89	22
ACC_06779	histone-lysine N-methyltransferase SETMAR-like			15	15	9	12	14	0	2	4	18	11	6	3	6	3	4	0
ACC_06780	choline transporter-like protein 1-like			8	5	4	8	3	1	3	1	26	44	8	61	11	12	3	3

ACC_06781	conserved hypothetical protein	K08471		0	0	1	0	2	0	0	0	0	2	0	0	0	0	0	0
ACC_06782	pyrokinin-like receptor 2		KOG4219	3	2	2	5	1	0	0	0	11	7	4	2	14	17	5	0
ACC_06783	conserved hypothetical protein		KOG3522	219	169	148	224	59	12	23	31	767	789	20	41	77	75	78	67
ACC_06784	conserved hypothetical protein		KOG1303	85	38	47	141	168	18	27	37	270	117	64	33	69	71	9	5
ACC_06785	conserved hypothetical protein		KOG0147	245	121	105	137	24	3	9	14	121	105	13	14	0	0	9	3
ACC_06786	venom acid phosphatase Acph-1-like		KOG3720	76	669	357	55	102	259	206	363	9	18	14	221	7	14	1	0
ACC_06787	conserved hypothetical protein			32	20	19	55	13	2	13	9	28	40	5	2	62	21	32	19
ACC_06788	protein arginine N-methyltransferase 3	K11436	KOG1499	64	43	39	39	50	7	20	18	37	84	15	30	118	126	37	17
ACC_06789	fidgetin-like protein 1-like		KOG0740	327	235	236	316	297	39	63	45	479	319	336	575	222	213	67	14
ACC_06790	conserved hypothetical protein			56	28	29	67	43	2	4	2	14	17	21	4	4	9	4	6
ACC_06791	NADH dehydrogenase	K03938	KOG4110	728	754	789	1361	1419	73	133	198	749	250	771	1543	1377	1455	142	126
ACC_06792	cytoplasmic tRNA 2-thiolation protein 2-like	K14169	KOG2594	93	79	52	89	106	4	5	11	113	130	82	280	248	254	17	5
ACC_06793	conserved hypothetical protein			0	0	0	0	0	0	0	0	0	0	12	39	1	1	0	0
ACC_06794	pecanex		KOG3604	1221	721	504	505	352	104	202	166	1208	898	217	220	463	372	201	93
ACC_06795	sulfhydryl oxidase 1-like		KOG1731	368	264	245	274	271	16	23	37	632	652	194	686	624	425	49	30
ACC_06796	conserved hypothetical protein	K12897	KOG0108	399	276	258	599	267	24	23	48	212	200	129	167	360	738	461	174
ACC_06797	protein BTG2-like	K14443	KOG4006	643	439	458	294	143	152	213	232	963	914	168	362	224	238	651	255
ACC_06798	pleckstrin homology domain-containing family M member 1		KOG1829	152	157	120	214	244	79	103	147	562	340	108	304	117	152	53	19
ACC_06799	conserved hypothetical protein		KOG4308	5	3	1	11	7	1	3	4	1	0	0	0	0	2	6	2
ACC_06800	conserved hypothetical protein			29	49	59	158	44	5	7	11	13	11	33	39	3	8	16	15
ACC_06801	exosome complex exonuclease RRP46	K12590	KOG1069	21	13	18	63	42	2	0	1	9	6	6	10	54	167	34	20
ACC_06802	conserved hypothetical protein			0	2	1	6	1	0	0	0	1	0	0	0	1	1	1	1
ACC_06803	threonine dehydratase catabolic-like	K01754	KOG1250	89	8	7	42	26	1	3	5	656	4378	0	2	193	5	0	1
ACC_06804	conserved hypothetical protein		KOG3054	24	7	5	29	5	2	2	1	48	172	10	4	8	21	54	38
ACC_06805	conserved hypothetical protein		KOG0241	3	0	0	3	0	1	1	1	4	18	1	0	1	2	2	2
ACC_06806	putative odorant receptor 13a-like			1	2	2	2	2	2	2	1	0	10	48	11	3	2	3	5
ACC_06807	mediator of RNA polymerase II transcription subun	K15128	KOG3169	171	180	208	194	162	26	54	51	194	96	119	290	351	311	128	42
ACC_06808	conserved hypothetical protein			649	369	320	378	261	36	72	76	390	191	241	147	75	95	17	7
ACC_06809	lysophospholipase-like protein 1-like		KOG2112	175	126	115	248	271	16	24	30	235	137	86	304	355	372	41	7
ACC_06810	putative OPA3-like protein CG13603-like		KOG3335	148	107	100	159	183	7	23	27	189	128	124	371	290	305	58	30
ACC_06811	LOW QUALITY PROTEIN	K11660	KOG3554	1073	785	852	1423	779	108	117	146	1242	894	395	470	1007	1123	631	142
ACC_06812	autophagy-related protein 2 homolog B		KOG1809	30	18	22	14	12	4	4	8	87	128	11	24	47	20	5	0
ACC_06813	conserved hypothetical protein		KOG0161	421	249	313	455	216	49	72	94	1335	674	67	196	108	95	74	35
ACC_06814	probable ribonuclease P/MRP protein subunit POP!	K03537	KOG4639	80	44	34	90	80	8	9	15	41	28	78	28	69	114	22	9
ACC_06815	conserved hypothetical protein			44	30	17	58	37	1	3	8	138	78	24	120	121	128	7	5
ACC_06816	protein yippee-like 1-like isoform 2		KOG3399	240	66	106	195	91	58	77	141	196	90	79	75	12	17	23	17
ACC_06817	zinc finger protein 84-like		KOG3598	287	307	354	408	109	41	35	49	455	445	100	223	309	532	657	171
ACC_06818	cysteine-rich with EGF-like domain protein 1-like		KOG4260	472	409	400	840	905	53	80	113	420	253	235	707	752	826	122	58
ACC_06819	ornithine aminotransferase, mitochondrial-like		KOG1402	3	3	3	2	2	0	1	3	5	6	2	0	156	419	51	6
ACC_06820	60S ribosomal protein L37a		KOG0402	78	51	25	131	70	15	27	31	26	26	95	47	200	213	219	162
ACC_06821	conserved hypothetical protein		KOG4225	857	631	767	1120	297	148	210	290	2466	878	412	379	261	277	391	121
ACC_06822	tyrosine-protein phosphatase corkscrew	K07293	KOG0790	1253	814	776	1013	914	111	156	195	1811	935	424	962	1357	1280	208	85
ACC_06823	slit homolog 1 protein-like, partial		KOG4194	17	16	20	20	20	2	7	4	86	56	25	101	26	25	19	24
ACC_06824	conserved hypothetical protein		KOG3530	1393	850	822	985	561	146	256	267	1903	1049	369	332	625	712	405	279
ACC_06825	conserved hypothetical protein		KOG3627	14	12	4	32	8	2	3	2	14	12	6	9	31	432	155	16
ACC_06826	conserved hypothetical protein		KOG0161	186	114	147	160	83	2	11	15	289	291	20	76	132	127	25	5
ACC_06827	conserved hypothetical protein			45	33	27	24	12	4	2	7	56	77	4	29	32	26	25	16
ACC_06828	long-chain-fatty-acid--CoA ligase 3-like isoform 1	K01897	KOG1180	1297	648	585	1161	1005	155	274	268	8013	4471	539	2536	6116	3185	619	218
ACC_06829	conserved hypothetical protein	K03979	KOG1489	330	223	224	301	257	34	54	46	343	221	176	225	624	743	147	45
ACC_06830	protein NDRG3-like isoform 1		KOG2931	1913	780	901	2014	837	180	284	383	998	617	2243	1622	248	183	314	228
ACC_06831	calcineurin-binding protein cabin-1-like		KOG2992	818	628	438	801	798	124	307	331	483	650	254	138	467	540	1218	549
ACC_06832	protein takeout-like			544	519	279	79	114	643	912	1304	48	97	130	210	413	112	42	83
ACC_06833	dystrophin, isoforms A/C/F/G/H-like		KOG0517	1191	724	455	281	171	403	863	871	1470	1319	290	206	160	81	1048	461
ACC_06834	poly(A)-specific ribonuclease PARN-like	K01148	KOG1990	337	188	162	440	315	37	68	71	412	311	131	226	632	840	392	226
ACC_06835	dystrophin, isoforms A/C/F/G/H-like isoform 1		KOG4286	526	334	325	555	137	65	66	113	708	490	216	229	95	111	227	80
ACC_06836	cytochrome b		KOG4663	13	5	8	6	2	1	3	2	3	14	3	0	0	0	9	2
ACC_06837	BCL2/adenovirus E1B 19 kDa protein-interacting pr	K15464		1015	839	837	1664	715	743	1061	1626	1946	951	658	683	941	488	368	145

ACC_06838	delta-1-pyrroline-5-carboxylate synthase-like	K12657	KOG4165	170	152	316	785	449	22	30	29	1442	2334	318	2260	2038	3224	1023	59
ACC_06839	conserved hypothetical protein			1741	1085	1036	3631	1622	501	792	979	4916	1825	1007	1923	1646	1101	1424	439
ACC_06840	protein Skeletor, isoforms B/C-like		KOG4731	406	301	365	1157	313	43	83	104	307	283	36	354	142	108	275	236
ACC_06841	ras-related protein Rab-28-like	K07915	KOG0078	31	19	27	44	26	5	3	4	35	45	116	224	27	34	3	2
ACC_06842	f-box/LRR-repeat protein 2-like isoform 1		KOG4341	15	6	4	15	7	8	3	5	73	109	10	26	42	24	5	8
ACC_06843	Golgi pH regulator-like		KOG2417	349	250	252	494	409	39	70	70	312	209	271	313	604	785	123	37
ACC_06844	bipolar kinesin KRP-130	K10398	KOG0243	354	222	206	243	333	16	61	56	230	160	130	157	383	596	271	172
ACC_06845	coiled-coil-helix-coiled-coil-helix domain-containing	K04794	KOG3282	138	95	88	141	182	9	29	25	141	110	106	172	216	291	48	12
ACC_06846	peroxisome assembly factor 2-like	K13339	KOG0736	387	260	284	397	387	25	35	42	472	398	243	453	482	318	34	9
ACC_06847	cadherin-related tumor suppressor	K16507	KOG1219	0	0	0	8	1	1	0	1	3	0	0	1	1	5	44	39
ACC_06848	rRNA-processing protein FCF1 homolog	K14566	KOG3165	122	79	76	90	90	8	36	33	124	76	34	96	196	347	82	90
ACC_06849	DPH3 homolog isoform 1	K15455	KOG2923	126	132	142	89	130	21	33	38	162	100	58	176	251	198	55	25
ACC_06850	guanine nucleotide-binding protein G(i) subunit alp	K04630	KOG0082	72	45	48	77	77	6	3	7	169	135	28	202	401	392	46	16
ACC_06851	probable protein-cysteine N-palmitoyltransferase p	K00181	KOG4312	56	41	33	83	95	2	1	2	99	68	66	211	172	145	8	0
ACC_06852	SET and MYND domain-containing protein 4-like		KOG2084	56	63	78	114	75	4	17	25	259	95	32	73	106	73	25	12
ACC_06853	protein PAT1 homolog 1	K12617	KOG4592	395	321	394	405	193	23	71	68	660	749	116	381	605	446	144	107
ACC_06854	43 kDa receptor-associated protein of the synapse-like		KOG1941	1001	397	339	1214	607	50	59	89	283	122	50	48	39	31	15	12
ACC_06855	ATP-dependent DNA helicase PIF1-like	K15255	KOG0987	61	43	49	83	48	7	7	16	128	38	27	25	141	270	29	13
ACC_06856	protein kintoun-like		KOG4356	309	167	153	307	140	31	51	59	407	271	175	361	312	294	106	44
ACC_06857	fukutin-related protein-like			158	82	64	78	116	3	5	6	205	126	18	105	48	40	4	1
ACC_06858	dolichyl-diphosphooligosaccharide--protein glycosy	K12667	KOG2447	990	809	446	893	829	102	157	155	1337	1859	561	1028	7043	6883	506	101
ACC_06859	conserved hypothetical protein		KOG1634	3094	2909	2568	3184	2232	1069	1894	2205	2853	2708	1266	488	1004	1417	5199	3455
ACC_06860	conserved hypothetical protein			211	164	133	205	179	34	61	63	523	402	100	328	268	143	15	9
ACC_06861	conserved hypothetical protein			139	85	55	167	162	38	150	139	192	192	99	120	116	37	102	57
ACC_06862	conserved hypothetical protein			6438	4060	4436	3715	4528	641	1558	1460	2775	1986	1426	1590	2211	1987	819	355
ACC_06863	conserved hypothetical protein		KOG1090	158	83	97	246	98	11	19	17	237	107	126	228	121	155	74	23
ACC_06864	zinc finger matrin-type protein 5-like	K13152	KOG3454	130	127	109	228	175	14	30	38	211	78	147	277	190	269	53	20
ACC_06865	UPF0235 protein C15orf40 homolog	K09131	KOG3276	24	20	19	43	34	4	3	9	23	9	17	44	61	90	11	13
ACC_06866	Y+L amino acid transporter 2-like			4	0	0	1	2	0	0	0	0	1	0	0	0	0	0	0
ACC_06867	segment polarity protein dishevelled homolog DVL-	K02353	KOG3571	231	180	165	183	96	21	19	36	358	412	87	224	324	298	86	24
ACC_06868	adenosine deaminase CECR1-like	K13697	KOG1097	14	9	13	69	48	2	4	2	49	16	48	62	466	656	21	7
ACC_06869	iron-sulfur protein NUBPL-like	K12260	KOG3388	11	13	11	21	24	1	1	2	25	10	26	45	67	104	6	5
ACC_06870	iron-sulfur protein NUBPL-like	K03593	KOG3022	123	96	103	251	188	20	26	30	173	110	111	314	478	596	93	38
ACC_06871	probable G-protein coupled receptor CG31760-like		KOG4418	807	520	568	880	475	88	178	178	344	212	19	16	5	4	35	25
ACC_06872	probable hydroxyacid-oxoacid transhydrogenase, mitochon		KOG3857	13	5	5	10	13	1	1	2	15	26	8	11	59	57	3	4
ACC_06873	glycoprotein 150		KOG4194	546	429	493	1009	777	67	83	85	6661	5280	1431	5088	8694	6551	332	53
ACC_06874	conserved hypothetical protein			12	7	6	5	3	1	1	3	20	16	2	2	20	11	23	3
ACC_06875	chloride intracellular channel exc-4		KOG1422	294	174	172	330	131	33	20	45	1187	1179	191	1109	1101	1160	227	52
ACC_06876	facilitated trehalose transporter Tret1-like		KOG0254	228	156	121	273	176	34	50	47	489	375	162	877	269	367	183	25
ACC_06877	RNA polymerase II subunit A C-terminal domain ph	K15732	KOG0323	497	397	420	498	462	66	167	166	827	548	394	430	622	806	290	161
ACC_06878	conserved hypothetical protein			1103	667	868	3258	957	94	146	195	508	250	460	368	20	48	126	94
ACC_06879	protein dumpy-19-like		KOG4587	326	174	175	482	251	41	39	67	399	209	216	183	153	217	77	48
ACC_06880	conserved hypothetical protein	K09280	KOG3982	50	20	30	78	17	3	7	9	16	18	0	0	1	2	15	12
ACC_06881	conserved hypothetical protein			124	63	77	224	25	5	9	9	162	119	17	59	31	31	41	25
ACC_06882	conserved hypothetical protein		KOG3898	0	0	0	0	2	0	0	0	0	0	0	0	0	7	34	7
ACC_06883	conserved hypothetical protein			1	2	3	4	2	1	0	3	23	38	14	1	3	16	9	6
ACC_06884	conserved hypothetical protein			0	0	0	0	0	0	0	0	0	1	0	0	1	8	7	13
ACC_06885	conserved hypothetical protein			0	1	0	0	0	0	0	0	0	0	1	0	0	7	8	6
ACC_06886	conserved hypothetical protein			0	0	0	0	0	0	0	0	2	0	1	0	4	783	2579	197
ACC_06887	ras-specific guanine nucleotide-releasing factor 1-li	K04349	KOG3417	175	183	116	192	100	10	18	14	88	67	14	31	1	0	0	0
ACC_06888	forkhead box protein O		KOG2294	17	6	7	7	2	1	1	4	68	61	8	13	22	10	18	13
ACC_06889	forkhead box protein O	K12358	KOG2294	9	3	1	7	1	2	0	2	43	34	4	10	13	11	14	11
ACC_06890	thyrotropin-releasing hormone-degrading ectoenzyme-like		KOG1046	73	39	47	18	12	15	13	37	71	93	175	140	1	0	0	0
ACC_06891	thyrotropin-releasing hormone-degrading ectoenzyme-like		KOG1046	104	38	22	23	12	21	9	24	85	144	119	238	0	0	0	0
ACC_06892	vanin-like protein 1-like		KOG0806	1	0	1	2	2	0	0	0	5	10	0	0	8	4	1	0
ACC_06893	vanin-like protein 1-like		KOG0806	6	8	2	7	5	0	0	1	35	44	2	4	616	1202	27	5
ACC_06894	neuronal calcium sensor 2-like		KOG0044	1235	366	398	690	339	32	65	67	324	125	109	98	84	71	60	48

ACC_06895	sodium/potassium-transporting ATPase subunit be	K01540	KOG3927	1083	448	602	1102	679	12	17	23	1718	658	65	2161	496	372	45	16
ACC_06896	ABC transporter G family member 20-like, partial			2	3	1	2	2	0	0	1	12	2	7	4	12	3	3	0
ACC_06897	ABC transporter G family member 20-like, partial			3	6	1	5	4	0	1	1	14	1	9	11	14	12	1	0
ACC_06898	TWiK family of potassium channels protein 7-like		KOG1418	44	18	10	20	12	0	0	1	12	32	3	1	0	0	3	2
ACC_06899	sperm-associated antigen 1-like		KOG0548	6	4	6	6	5	1	3	1	4	7	121	287	5	1	1	0
ACC_06900	heparan sulfate glucosamine 3-O-sulfotransferase !	K08104	KOG3704	145	59	65	118	46	13	30	32	87	110	24	31	4	7	28	20
ACC_06901	ultraspiracle	K14030	KOG4215	272	144	204	307	116	55	88	165	359	343	328	176	44	42	113	138
ACC_06902	conserved hypothetical protein			127	160	20	25	26	46	40	68	37	30	20	18	742	337	21	9
ACC_06903	tonsoku-like protein-like		KOG4177	897	425	405	766	987	102	248	241	1132	638	493	430	393	461	252	115
ACC_06904	serine palmitoyltransferase 2-like	K00654	KOG1357	704	209	212	235	139	26	40	45	375	197	55	134	715	803	146	41
ACC_06905	ras-related protein Rab-8A-like isoform 2			57	44	29	74	32	8	20	17	59	48	29	70	53	59	157	63
ACC_06906	conserved hypothetical protein		KOG1090	133	93	82	175	115	21	14	20	172	137	78	104	19	9	7	2
ACC_06907	apolipoprotein D-like		KOG4824	12	6	5	21	15	0	0	0	5	3	1	8	4	161	26	18
ACC_06908	apolipoprotein D-like isoform 2		KOG4824	20	5	2	19	7	1	3	2	13	21	14	9	21	1245	2309	6880
ACC_06909	protein NipSnap-like		KOG2883	476	354	366	701	499	54	63	89	663	408	354	929	1417	991	202	75
ACC_06910	conserved hypothetical protein			163	44	12	39	7	23	13	37	12	35	10	17	51	194	611	126
ACC_06911	beta carbonic anhydrase 1-like		KOG1578	60	50	48	35	47	7	1	8	408	190	48	149	179	58	10	3
ACC_06912	yellow-like protein			47	15	17	57	12	6	12	10	50	7	30	9	7	1	8	6
ACC_06913	monocarboxylate transporter 9-like		KOG2504	16	14	14	29	16	24	51	53	97	148	2	6	148	177	146	137
ACC_06914	rap1 GTPase-activating protein 2		KOG3686	317	159	135	205	100	35	39	63	671	433	73	93	141	147	71	47
ACC_06915	Cytochrome c-2			394	67	73	275	118	21	17	28	159	86	95	197	53	32	29	8
ACC_06916	lipid phosphate phosphohydrolase 3-like	K01080	KOG3030	165	63	84	102	83	2	10	8	180	135	34	200	110	52	13	4
ACC_06917	conserved hypothetical protein			265	149	219	251	91	47	60	88	433	183	164	148	235	308	323	173
ACC_06918	zinc finger protein Noc-like		KOG0956	2864	1818	2086	1662	968	309	595	594	1022	745	309	161	310	461	382	242
ACC_06919	sodium channel protein para-like			174	103	121	142	51	11	16	26	61	34	28	5	1	0	6	4
ACC_06920	lysosomal protein NCU-G1-A-like			703	211	202	344	400	24	33	43	406	168	352	760	201	216	19	6
ACC_06921	caiB/baiF CoA-transferase family protein C7orf10-like		KOG3957	385	240	199	328	373	47	92	115	415	242	211	349	852	712	176	91
ACC_06922	ATP-dependent RNA helicase WM6-like		KOG0329	262	171	165	283	216	20	49	34	244	103	158	129	649	1280	219	98
ACC_06923	vesicle transport through interaction with t-SNARE:	K08493	KOG1666	86	103	157	112	105	7	34	22	51	36	67	149	75	94	30	4
ACC_06924	conserved hypothetical protein			0	2	2	7	4	0	3	1	101	24	118	159	11	21	12	7
ACC_06925	conserved hypothetical protein		KOG3589	1552	970	834	1606	1415	160	282	324	1705	1554	584	894	861	694	239	57
ACC_06926	polycomb protein Scm		KOG3766	79	79	66	107	67	5	8	13	111	103	26	85	119	117	21	14
ACC_06927	muscarinic acetylcholine receptor DM1-like	K04131	KOG4220	1986	1427	1025	2399	947	282	493	579	838	395	60	63	69	86	197	89
ACC_06928	microspherule protein 1-like	K11674	KOG2293	176	82	66	123	127	15	21	28	264	88	121	262	270	398	154	57
ACC_06929	myosin heavy chain, muscle-like isoform 4		KOG0161	109	21	6	40	13	22	40	22	2107	1362	33	74	59	50	60	37
ACC_06930	chondroitin sulfate proteoglycan 4		KOG3597	243	120	113	112	42	13	2	11	205	158	3	23	44	64	72	37
ACC_06931	DNA methyltransferase 1-associated protein 1	K11324	KOG2656	320	228	224	406	264	64	112	122	229	114	168	158	189	262	162	109
ACC_06932	mitochondrial import inner membrane translocase subunit T		KOG3480	209	116	173	322	370	28	62	97	115	72	111	181	443	877	331	210
ACC_06933	conserved hypothetical protein		KOG4194	10	1	1	6	1	1	2	0	1	2	0	0	0	0	0	0
ACC_06934	proton-coupled amino acid transporter 1-like isofo	K14209	KOG1304	1026	342	310	958	585	30	55	51	1052	619	137	640	12	21	22	15
ACC_06935	protein mago nashi	K12877	KOG3392	84	48	63	165	136	7	10	13	171	82	97	253	326	574	78	25
ACC_06936	conserved hypothetical protein		KOG3942	173	132	127	302	190	41	45	46	2548	1336	227	578	126	82	26	16
ACC_06937	alpha-methylacyl-CoA racemase-like	K01796	KOG3957	159	115	99	237	254	15	24	40	434	332	242	663	524	377	48	19
ACC_06938	NMDA receptor 1	K05208	KOG4440	1207	616	593	2056	1372	79	110	105	562	233	64	56	43	65	18	17
ACC_06939	cytochrome c oxidase assembly protein COX15 hon	K02259	KOG2725	154	106	87	277	274	29	28	42	165	151	147	254	614	733	53	18
ACC_06940	calmodulin-like protein 4-like		KOG0027	62	61	32	76	57	4	9	24	77	53	69	101	140	107	24	17
ACC_06941	neurotrimin-like		KOG3513	119	60	59	93	29	8	4	3	65	63	28	35	7	3	8	4
ACC_06942	Kv channel-interacting protein 2-like		KOG0044	523	308	294	631	344	15	18	34	185	99	16	18	2	4	2	2
ACC_06943	protein fat-free homolog		KOG2346	179	108	114	189	188	10	27	28	222	155	127	191	199	185	13	12
ACC_06944	basigin-like		KOG3513	2784	1290	1365	2475	987	322	515	688	2372	894	965	810	1384	2032	1820	442
ACC_06945	conserved hypothetical protein			44	26	16	96	56	5	15	17	139	64	82	325	35	12	10	2
ACC_06946	conserved hypothetical protein			183	126	143	250	114	15	20	19	250	171	57	79	141	155	78	11
ACC_06947	conserved hypothetical protein			3	1	7	0	1	0	0	0	0	1	0	2	1	3	0	0
ACC_06948	dihydroorotate dehydrogenase, mitochondrial-like	K00254	KOG1436	98	75	81	211	170	9	13	32	142	131	86	108	239	328	43	18
ACC_06949	LOW QUALITY PROTEIN		KOG4306	1029	219	254	943	344	33	34	58	503	85	2	6	39	36	25	29
ACC_06950	LOW QUALITY PROTEIN		KOG4287	5	3	2	3	1	4	3	0	37	61	0	2	3	2	7	1
ACC_06951	glutamate receptor, ionotropic kainate 2-like		KOG1054	22	3	3	14	5	10	3	9	409	208	4	33	49	58	54	35

ACC_06952	conserved hypothetical protein			217	1	15	27	4	0	0	4	99	25	4	25	214	399	1537	1024
ACC_06953	UDP-glucuronosyltransferase 1-8-like		KOG1192	0	2	6	1	0	3	2	1	25	19	16	3	243	202	119	1
ACC_06954	39S ribosomal protein L34, mitochondrial-like	K02914	KOG4612	211	95	155	341	220	10	25	60	62	54	215	342	280	304	63	36
ACC_06955	kynurenine/alpha-aminoadipate aminotransferase,	K00825	KOG0634	1	9	2	9	10	3	2	8	1754	425	1	5	34	140	99	14
ACC_06956	sterile alpha motif domain-containing protein 5-like		KOG4384	7	3	6	7	3	0	0	0	2	3	1	2	2	2	1	1
ACC_06957	glycerate kinase-like		KOG3935	127	80	69	182	131	17	22	38	69	39	22	18	5	7	17	3
ACC_06958	ubiquitin-conjugating enzyme E2 T-like	K13960	KOG0417	126	90	114	133	135	13	13	23	52	41	58	186	140	238	35	14
ACC_06959	sodium channel protein Nach		KOG4294	1	0	1	0	1	0	0	0	0	3	4	0	0	0	1	2
ACC_06960	IQ and ubiquitin-like domain-containing protein-like		KOG2018	2	0	4	5	1	0	1	2	4	4	2	2	0	3	5	4
ACC_06961	sorting nexin-4-like		KOG2273	471	245	250	347	328	22	22	24	536	224	115	315	217	233	44	12
ACC_06962	LOW QUALITY PROTEIN		KOG3598	51	53	37	39	9	9	7	10	300	289	61	65	65	96	157	47
ACC_06963	forkhead box protein O	K12358	KOG2294	31	5	12	27	9	18	31	25	99	145	18	23	22	29	137	88
ACC_06964	conserved hypothetical protein			124	52	52	122	18	4	4	6	142	386	15	65	28	11	77	13
ACC_06965	cadherin-87A-like		KOG1219	704	401	339	334	239	36	34	36	579	559	205	747	223	235	67	16
ACC_06966	SPARC isoform 2		KOG1757	4929	949	788	2492	808	140	200	247	3761	2276	525	4993	1910	1246	1268	365
ACC_06967	d-glucuronyl C5-epimerase-like	K01793	KOG3760	133	89	74	68	104	6	8	17	253	160	99	225	163	154	13	3
ACC_06968	sterol O-acyltransferase 1-like	K00637	KOG0380	208	163	176	664	453	97	148	160	2646	2706	14	67	778	693	209	71
ACC_06969	osmotic avoidance abnormal protein 3		KOG4280	13	9	12	17	10	5	3	3	59	65	104	144	92	25	6	7
ACC_06970	conserved hypothetical protein			172	96	60	108	179	2	1	7	96	80	40	179	158	120	9	3
ACC_06971	conserved hypothetical protein	K14766	KOG2147	690	449	299	756	874	133	359	341	631	609	584	404	818	1065	882	679
ACC_06972	apoptosis regulator R1-like		KOG4728	102	61	81	162	103	4	15	25	170	86	41	88	152	225	81	24
ACC_06973	protein tweety-like		KOG4433	161	162	127	231	77	16	40	49	265	376	67	70	141	71	80	55
ACC_06974	trehalase precursor	K01194	KOG0602	1408	832	1103	3045	1435	254	370	388	1236	1288	599	409	202	160	628	689
ACC_06975	facilitated trehalose transporter Tret1-like		KOG0254	3	1	2	6	1	0	1	0	5	9	1	2	18	4	0	3
ACC_06976	ubiquitin carboxyl-terminal hydrolase 38-like	K11854	KOG1864	179	131	135	141	115	7	8	7	505	463	73	286	202	225	13	6
ACC_06977	cell cycle control protein 50A-like isoform 1		KOG2952	440	211	172	361	445	20	47	67	558	334	189	551	398	352	24	10
ACC_06978	voltage-dependent calcium channel type D subunit	K05315	KOG2301	551	373	417	713	241	33	50	47	622	690	108	14	54	63	72	67
ACC_06979	oxysterol-binding protein-related protein 9-like isoform 2		KOG1739	72	47	39	62	38	4	4	3	108	117	29	116	88	112	14	5
ACC_06980	conserved hypothetical protein			0	0	0	2	0	0	0	0	2	1	0	1	6	44	12	18
ACC_06981	conserved hypothetical protein			2	0	1	0	0	0	0	1	6	2	0	22	60	81	23	39
ACC_06982	conserved hypothetical protein			73	46	36	93	31	14	22	19	488	361	31	251	160	152	174	105
ACC_06983	NADP-dependent malic enzyme isoform 1			67	51	55	132	76	6	6	2	563	912	105	460	2629	293	29	12
ACC_06984	protein unc-80 homolog			140	66	80	59	19	6	3	8	63	30	0	0	0	1	1	3
ACC_06985	conserved hypothetical protein		KOG4217	445	211	219	197	66	6	6	18	85	56	19	23	244	101	10	120
ACC_06986	extracellular domains-containing protein CG31004-like		KOG4291	35	13	16	32	22	6	9	13	7	16	10	2	6	7	29	24
ACC_06987	putative odorant receptor 67c-like			1	1	1	0	1	1	0	0	0	1	9	4	0	1	1	1
ACC_06988	putative odorant receptor 30a-like			4	3	0	5	1	0	0	0	2	3	49	165	0	0	0	7
ACC_06989	cadherin-89D-like			1	3	2	5	1	0	0	0	4	0	71	93	4	10	13	1
ACC_06990	conserved hypothetical protein		KOG4135	282	225	212	330	348	39	74	90	262	173	124	204	170	134	56	21
ACC_06991	Pyruvate dehydrogenase		KOG0700	204	119	144	242	185	16	31	19	254	164	133	264	530	821	147	52
ACC_06992	glycosyltransferase 25 family member-like	K11703	KOG4179	93	57	65	116	112	8	19	11	118	107	44	72	119	115	16	4
ACC_06993	potassium voltage-gated channel protein Shaw	K05320	KOG3713	141	49	51	69	39	11	15	28	42	46	22	11	1	1	19	8
ACC_06994	long-chain-fatty-acid--CoA ligase 1-like	K01897	KOG1256	91	39	42	108	37	17	14	17	611	312	108	216	89	56	39	15
ACC_06995	telomerase reverse transcriptase	K11126	KOG1005	137	80	66	80	79	2	5	7	50	104	66	172	119	93	14	9
ACC_06996	armadillo repeat-containing protein 6 homolog		KOG4199	102	44	39	101	80	8	5	14	73	43	25	78	108	119	6	9
ACC_06997	proclotting enzyme isoform 1		KOG3627	12	6	2	9	4	1	4	1	29	0	0	1	19	91	22	6
ACC_06998	conserved hypothetical protein			941	323	418	1363	974	524	1084	875	1292	603	864	183	142	241	540	354
ACC_06999	ecdysone receptor isoform A			413	220	271	431	118	41	88	118	259	223	108	26	11	20	24	36
ACC_07000	DNA repair protein complementing XP-C cells hom	K10838	KOG2179	1210	789	573	1015	1083	210	583	633	769	746	767	529	670	636	628	228
ACC_07001	conserved hypothetical protein			13	28	21	34	32	12	19	16	810	315	203	161	0	1	0	0
ACC_07002	conserved hypothetical protein		KOG1999	20	10	13	15	6	3	8	4	23	55	5	0	14	15	77	124
ACC_07003	protein cueball-like		KOG1214	144	313	79	87	75	79	118	160	1372	2728	66	239	1115	593	142	52
ACC_07004	putative odorant receptor 94b-like			0	0	0	1	0	3	4	3	37	32	73	209	0	1	5	1
ACC_07005	odorant receptor 46a, isoform A-like			0	0	0	0	1	0	0	0	0	0	12	3	0	0	0	0
ACC_07006	conserved hypothetical protein	K01444	KOG2498	431	386	380	821	513	132	257	316	663	337	390	375	365	457	602	395
ACC_07007	3-ketodihydrosphingosine reductase-like isoform 1	K04708	KOG1210	414	279	228	503	459	37	74	83	467	329	330	1009	743	710	61	17
ACC_07008	alpha-mannosidase 2		KOG1958	206	123	65	62	50	4	6	5	276	178	20	83	19	80	12	3

ACC_07009	protein groucho isoform 1		KOG0639	106	77	79	98	20	21	16	22	101	95	39	33	40	70	147	24
ACC_07010	aquaporin AQPcic-like		KOG0223	62	25	24	123	35	12	7	13	474	1102	86	68	366	196	227	96
ACC_07011	conserved hypothetical protein			155	102	114	315	252	9	24	38	169	118	139	355	352	424	61	28
ACC_07012	5'-3' exoribonuclease 1 isoform 1	K12618	KOG2045	874	701	716	793	455	103	164	186	984	791	289	310	670	792	274	132
ACC_07013	dnaJ homolog subfamily C member 1-like	K09521	KOG0713	716	667	498	615	475	320	742	888	631	465	513	585	967	878	742	577
ACC_07014	phospholipase A-2-activating protein	K14018	KOG0301	321	168	198	531	413	28	48	54	518	413	201	467	858	820	107	23
ACC_07015	solute carrier family 25 member 36-A-like isoform	K15116	KOG0757	94	80	77	81	39	5	7	6	394	202	53	50	85	79	22	9
ACC_07016	putative acyl-CoA-binding protein-like isoform 2	K08762	KOG0817	83	83	51	173	98	23	16	15	708	322	187	778	575	733	109	17
ACC_07017	conserved hypothetical protein	K11833	KOG1868	769	414	396	763	282	106	122	170	1349	985	324	604	623	748	644	332
ACC_07018	sarcalumenin-like		KOG1954	303	134	51	154	70	135	233	194	5131	1564	94	221	476	310	194	220
ACC_07019	coronin-1C-like isoform 1		KOG0303	516	310	231	490	409	48	89	88	1602	1048	351	410	1338	1437	257	106
ACC_07020	conserved hypothetical protein		KOG1984	31	31	22	39	16	0	2	0	141	145	29	89	182	158	21	9
ACC_07021	conserved hypothetical protein			292	142	209	388	79	15	19	27	748	489	29	300	33	70	68	27
ACC_07022	odorant binding protein 14 precursor			140	3	10	22	18	6	8	6	1635	251	8036	1430	50230	3767	98	1054
ACC_07023	phosphoglycerate mutase	K01834	KOG0235	1438	767	767	1531	1720	97	122	225	1873	695	547	1887	3577	2953	227	135
ACC_07024	zinc finger protein 347-like		KOG2462	639	406	391	724	372	138	222	289	594	451	356	271	349	604	1530	466
ACC_07025	inhibitor of growth protein 3		KOG3627	89	57	63	149	74	14	22	27	303	148	99	238	123	137	115	42
ACC_07026	acylphosphatase-2-like	K01512	KOG3360	25	3	3	10	9	0	0	0	19	3	2	8	2	2	1	0
ACC_07027	conserved hypothetical protein			360	98	156	604	223	15	5	16	242	110	3	24	16	22	19	7
ACC_07028	glycosyltransferase AER61-like		KOG4698	164	99	75	154	97	21	29	38	93	69	73	153	201	220	82	36
ACC_07029	conserved hypothetical protein			80	74	65	262	209	9	8	19	124	85	51	65	29	22	2	7
ACC_07030	proton-coupled amino acid transporter 4	K14209	KOG1304	37	66	19	26	33	12	6	20	355	599	12	118	165	134	11	2
ACC_07031	conserved hypothetical protein			3	0	3	11	11	0	0	1	2	7	2	9	19	9	3	3
ACC_07032	probable hydroxyacid-oxoacid transhydrogenase, n	K11173	KOG3857	98	75	60	121	111	6	7	10	337	490	112	311	2366	2176	193	72
ACC_07033	insulin-like growth factor-binding protein complex acid labile		KOG4194	2	1	0	4	3	0	0	1	22	16	2	6	352	737	721	71
ACC_07034	serine/threonine-protein kinase 17A-like		KOG0032	37	25	26	62	17	4	3	4	179	128	13	57	49	76	34	11
ACC_07035	mitochondrial 2-oxoglutarate/malate carrier protei	K15104	KOG0759	196	95	69	74	103	7	11	11	298	224	77	336	522	300	10	2
ACC_07036	potassium voltage-gated channel subfamily H member 8		KOG0501	449	276	317	281	85	22	44	56	111	79	7	0	36	52	102	87
ACC_07037	conserved hypothetical protein		KOG4441	196	111	108	192	121	43	60	58	201	95	101	118	82	91	95	45
ACC_07038	transmembrane protein 170A-like		KOG4349	135	92	92	122	138	5	9	15	128	67	122	322	94	99	13	2
ACC_07039	protoporphyrinogen oxidase-like	K00231	KOG1276	304	250	223	252	237	24	29	39	419	205	159	153	273	398	50	24
ACC_07040	conserved hypothetical protein		KOG1144	172	101	127	268	144	30	67	59	407	65	44	15	55	67	273	290
ACC_07041	tyrosine-protein kinase-like otk-like	K05127	KOG1026	79	36	39	71	55	10	11	13	53	43	4	5	35	57	195	108
ACC_07042	NADH dehydrogenase subunit 4		KOG4845	0	1	0	2	0	0	1	0	4	2	0	0	0	0	0	0
ACC_07043	probable small nuclear ribonucleoprotein Sm D2-lil	K11096	KOG3459	149	72	59	177	195	28	63	83	83	56	106	186	301	440	142	157
ACC_07044	enhancer of mRNA-decapping protein 4	K12616	KOG1916	531	332	331	659	460	71	202	225	513	497	250	230	731	869	660	391
ACC_07045	conserved hypothetical protein		KOG0517	2416	2060	1341	2880	947	399	437	650	5370	4282	430	298	979	671	554	108
ACC_07046	conserved hypothetical protein		KOG0161	2106	1983	1220	2673	564	476	538	911	5735	3443	398	179	179	273	403	384
ACC_07047	slit homolog 2 protein-like		KOG4237	547	376	324	1011	798	57	146	119	2111	1740	246	1001	2	3	0	0
ACC_07048	conserved hypothetical protein		KOG3139	102	63	87	66	43	3	6	6	143	59	50	67	32	5	0	1
ACC_07049	conserved hypothetical protein			0	5	5	3	1	1	0	5	23	12	3	22	7	11	9	2
ACC_07050	protein LSM14 homolog B-B		KOG1073	1294	767	628	1726	885	210	337	447	1627	919	893	826	1520	1924	1567	934
ACC_07051	conserved hypothetical protein			186	106	166	420	285	39	46	55	525	232	152	476	196	216	132	23
ACC_07052	conserved hypothetical protein			29	13	13	30	19	3	5	4	85	32	7	15	12	13	9	4
ACC_07053	LOW QUALITY PROTEIN		KOG0161	279	203	166	216	228	52	138	148	176	155	105	106	339	365	153	105
ACC_07054	conserved hypothetical protein		KOG2896	369	242	255	330	256	41	72	79	388	248	118	178	169	164	59	19
ACC_07055	conserved hypothetical protein		KOG4551	1213	651	633	978	1515	81	157	193	638	625	838	598	633	622	431	118
ACC_07056	protein arginine N-methyltransferase 8-like isoforn	K11434	KOG1499	361	219	205	529	471	24	24	32	390	382	79	339	1249	2138	195	85
ACC_07057	conserved hypothetical protein			151	93	83	296	199	6	7	17	139	100	49	135	55	91	3	5
ACC_07058	conserved hypothetical protein	K10392	KOG0245	629	376	297	542	266	80	110	106	1485	894	278	569	550	368	166	98
ACC_07059	helicase SKI2W	K12599	KOG0947	315	230	231	326	326	35	79	107	331	385	165	374	632	672	190	109
ACC_07060	conserved hypothetical protein		KOG0488	0	0	0	0	0	0	1	0	3	3	54	45	2	1	1	0
ACC_07061	cadherin-related tumor suppressor	K16507	KOG1219	4	1	3	13	4	0	6	1	2	3	2	0	2	11	273	225
ACC_07062	ADAM 17-like protease-like isoform 2	K06059	KOG3658	346	180	185	422	370	27	49	54	443	194	183	166	279	263	43	17
ACC_07063	LOW QUALITY PROTEIN			15	11	16	21	5	0	0	2	8	7	2	1	1	3	4	5
ACC_07064	transient receptor potential channel pyrexia		KOG0510	11	6	5	20	3	2	4	6	53	84	21	16	8	22	25	7
ACC_07065	eukaryotic translation initiation factor 4E type 2	K03259	KOG1669	160	144	139	243	217	24	30	37	121	107	142	216	233	292	72	34

ACC_07066	cytoplasmic polyadenylation element-binding prot	K02602	KOG0129	40	34	41	46	36	4	4	8	36	41	13	11	23	19	6	3
ACC_07067	proton-coupled folate transporter-like isoform 1		KOG2816	143	70	77	184	76	10	7	29	289	162	969	4123	7	16	4	5
ACC_07068	conserved hypothetical protein			6	6	9	20	12	1	2	3	12	1	4	2	5079	5234	1133	321
ACC_07069	testican-1-like			131	89	128	65	36	8	4	10	194	172	25	107	12	11	3	1
ACC_07070	testican-2-like			188	83	103	63	32	6	9	7	151	179	36	90	5	6	5	0
ACC_07071	inorganic pyrophosphatase-like	K11726	KOG1626	400	260	283	947	902	23	30	31	583	789	273	1934	1785	1302	99	40
ACC_07072	conserved hypothetical protein	K16449	KOG3589	493	262	291	1000	152	16	36	76	61	79	24	6	4	7	127	127
ACC_07073	probable helicase with zinc finger domain-like		KOG1804	251	160	152	385	115	19	31	38	334	312	108	176	142	241	241	93
ACC_07074	conserved hypothetical protein			84	84	57	73	70	26	43	60	105	66	43	18	41	56	69	32
ACC_07075	cytochrome b5 type B-like		KOG0537	16	3	6	6	12	49	72	32	394	39	3	7	78	296	132	87
ACC_07076	conserved hypothetical protein	K14072	KOG4219	15	22	8	54	27	2	0	1	7	4	0	0	2	5	0	1
ACC_07077	conserved hypothetical protein			4	0	3	9	10	0	1	1	4	5	3	6	7	4	9	2
ACC_07078	conserved hypothetical protein		KOG3608	73	64	44	78	54	5	10	8	85	90	29	37	64	75	11	2
ACC_07079	regulator of nonsense transcripts 2	K14327	KOG2051	503	293	251	300	184	34	38	70	484	371	121	229	615	438	111	54
ACC_07080	leucine rich repeat G protein coupled receptor		KOG2087	2	2	3	3	6	0	1	2	3	8	20	5	0	1	0	1
ACC_07081	conserved hypothetical protein			22	15	18	17	7	1	3	0	28	25	18	69	111	176	135	60
ACC_07082	glucose dehydrogenase		KOG1238	3748	2371	2962	1214	547	2922	1687	4935	407	204	137	59	23	59	76	86
ACC_07083	conserved hypothetical protein	K08268	KOG3558	1527	817	830	1928	454	110	93	211	1926	1894	323	585	1173	1132	1240	431
ACC_07084	paxillin-like isoform 1		KOG1703	52	26	14	79	88	6	15	20	198	103	87	116	88	95	26	7
ACC_07085	solute carrier organic anion transporter family mem	K14353	KOG3626	268	148	105	177	104	20	54	49	617	471	196	189	462	277	183	73
ACC_07086	WD repeat-containing protein 69-like		KOG0272	9	2	7	10	5	0	1	0	15	1	4	1	1	3	5	0
ACC_07087	adenylyl cyclase-associated protein 1-like isoform 1		KOG2675	294	205	209	216	173	37	60	79	522	491	56	217	443	346	255	116
ACC_07088	fatty acid-binding protein, adipocyte-like		KOG4015	90	47	72	145	76	9	11	15	128	61	59	121	176	212	33	14
ACC_07089	malectin-like		KOG3593	228	189	188	251	280	22	29	30	196	98	187	310	222	214	15	3
ACC_07090	A disintegrin and metalloproteinase with thrombospondin m		KOG3538	379	434	377	596	253	115	122	157	933	516	236	155	531	286	105	23
ACC_07091	n-alpha-acetyltransferase 25, NatB auxiliary subunit		KOG2053	86	46	42	88	91	10	8	12	98	113	48	116	260	327	54	17
ACC_07092	protein Wnt-5b	K00444	KOG3913	46	34	26	94	37	4	4	6	76	40	21	53	7	12	5	2
ACC_07093	allatostatins precursor			41	32	54	143	81	3	5	10	40	19	0	0	0	19	20	1
ACC_07094	conserved hypothetical protein			93	49	91	140	34	22	31	42	45	148	31	3	5	15	282	438
ACC_07095	conserved hypothetical protein		KOG0843	2	2	0	0	2	0	0	0	1	1	0	0	0	1	0	1
ACC_07096	trypsin 3A1		KOG3627	0	1	0	1	2	0	0	0	0	5	1	0	1	0	0	1
ACC_07097	conserved hypothetical protein			159	201	283	664	902	6	15	25	4226	2837	4248	21763	628	78	9	0
ACC_07098	LOW QUALITY PROTEIN		KOG4338	7441	2601	2789	9633	4318	440	509	632	15601	78354	1180	907	23880	7949	3006	1683
ACC_07099	etoposide-induced protein 2.4-like	K10134	KOG3966	76	54	26	96	77	8	7	6	377	392	55	226	395	219	24	0
ACC_07100	conserved hypothetical protein			6	2	1	1	6	7	0	1	508	238	11308	9056	2	3	0	0
ACC_07101	radial spoke head 10 homolog B-like		KOG0231	4	2	5	8	3	0	2	1	4	1	0	2	3	0	1	0
ACC_07102	protein numb-like		KOG3537	466	454	549	900	194	34	62	98	358	170	187	177	127	109	274	48
ACC_07103	kelch-like protein 10	K10448	KOG4441	3	2	6	15	4	3	4	3	47	173	13	34	189	37	4	3
ACC_07104	inositol polyphosphate 5-phosphatase OCRL-1-like	K01099	KOG0566	342	192	200	218	244	28	24	37	330	200	118	114	185	249	20	7
ACC_07105	importin-11		KOG1993	406	276	217	578	616	39	51	81	691	608	331	523	1061	1355	144	46
ACC_07106	conserved hypothetical protein	K11422	KOG1080	899	676	564	1326	806	209	407	405	928	596	435	273	645	826	1062	433
ACC_07107	ankyrin repeat and FYVE domain-containing protein 1-like		KOG4591	101	59	62	75	86	5	14	20	184	108	35	95	99	108	5	5
ACC_07108	conserved hypothetical protein			0	0	0	0	0	0	0	0	3	1	85	35	0	0	0	0
ACC_07109	conserved hypothetical protein			106	76	57	140	22	20	22	31	196	239	29	86	89	147	431	115
ACC_07110	conserved hypothetical protein			472	172	182	438	88	77	105	175	910	546	211	291	78	93	724	493
ACC_07111	conserved hypothetical protein		KOG3623	21	11	38	70	6	2	2	8	8	6	3	1	0	0	60	25
ACC_07112	transcription factor 21-like		KOG4029	0	0	3	2	0	0	0	0	5	2	0	1	3	10	38	9
ACC_07113	aminomethyltransferase, mitochondrial-like	K00605	KOG2770	351	295	521	463	204	56	109	125	466	450	56	306	1131	788	534	142
ACC_07114	Protein daughterless	K15603	KOG3910	276	198	261	349	60	12	24	29	283	357	64	62	78	65	129	57
ACC_07115	conserved hypothetical protein			165	66	43	134	51	8	21	31	316	150	171	281	376	239	340	356
ACC_07116	slit homolog 1 protein-like		KOG4194	8	1	1	3	2	1	1	2	13	9	0	55	82	88	104	126
ACC_07117	conserved hypothetical protein			86	68	60	155	134	4	4	13	222	144	21	48	147	112	24	9
ACC_07118	LOW QUALITY PROTEIN	K00681	KOG2410	3219	2511	1422	5887	2880	503	543	513	2235	1448	6703	16425	3524	2244	312	73
ACC_07119	conserved hypothetical protein	K09270	KOG0527	7	5	7	13	2	0	0	1	8	5	11	1	15	14	87	24
ACC_07120	cullin-1-like isoform 1	K03347	KOG2166	567	401	342	501	393	72	122	149	1023	791	238	367	971	909	227	167
ACC_07121	ras-related protein Rab-26 isoform 1			159	50	52	94	21	1	4	5	34	24	20	31	4	1	3	0
ACC_07122	receptor-type tyrosine-protein phosphatase N2-like isoform		KOG0793	1406	866	1041	909	547	41	52	68	1149	1009	96	205	55	103	24	6

ACC_07123	active breakpoint cluster region-related protein-like		KOG4269	130	79	81	158	97	27	36	56	246	148	63	123	106	109	69	23
ACC_07124	putative methyltransferase METT10D-like		KOG2912	351	169	212	244	298	29	69	88	384	204	158	425	304	400	68	17
ACC_07125	LOW QUALITY PROTEIN		KOG3513	11	5	3	3	1	0	1	0	2	5	0	2	0	0	2	0
ACC_07126	guanine nucleotide-releasing factor 2-like			74	76	84	21	14	4	9	7	175	193	14	40	31	26	19	18
ACC_07127	solute carrier family 12 member 8-like	K14428	KOG2083	259	150	148	223	276	40	96	80	753	334	264	263	633	574	95	27
ACC_07128	tRNA (adenine-N(1)-)-methyltransferase non-cataly	K03256	KOG1416	148	116	116	207	252	13	33	37	86	73	79	77	199	313	108	81
ACC_07129	conserved hypothetical protein			27	32	20	55	52	1	3	7	16	10	29	52	51	59	4	7
ACC_07130	tudor domain-containing protein 7-like		KOG2039	972	560	388	611	538	105	189	193	991	992	164	307	428	254	77	39
ACC_07131	YTH domain family protein 1-like		KOG1901	508	317	292	439	210	55	74	73	927	591	193	296	634	636	282	119
ACC_07132	carbonic anhydrase 3	K01672	KOG0382	19	18	21	60	16	3	14	9	31	30	23	7	4	4	47	16
ACC_07133	conserved hypothetical protein			2	3	0	1	0	0	0	0	1	2	0	0	0	0	0	0
ACC_07134	MYCBP-associated protein-like			0	0	0	0	0	0	0	0	0	0	1	3	0	0	0	0
ACC_07135	metallophosphoesterase domain-containing protein 1-like is		KOG3947	191	111	151	205	166	12	18	18	158	98	86	182	242	238	36	12
ACC_07136	serine/threonine-protein phosphatase 4 regulatory	K15424	KOG0211	291	147	145	263	198	58	99	82	432	202	189	161	301	695	782	226
ACC_07137	zinc finger protein 268-like		KOG2462	357	255	224	173	63	41	67	53	946	1224	100	374	600	565	337	208
ACC_07138	UDP-xylose and UDP-N-acetylglucosamine transpo	K15278	KOG1583	153	126	109	147	229	17	12	29	350	266	259	1176	476	317	12	4
ACC_07139	conserved hypothetical protein		KOG2589	338	209	256	336	107	30	77	84	219	411	54	67	38	64	207	243
ACC_07140	conserved hypothetical protein		KOG3139	54	58	48	45	41	6	10	9	41	33	25	32	89	96	8	8
ACC_07141	conserved hypothetical protein	K09180	KOG3811	101	45	63	79	56	30	42	50	92	68	18	17	11	60	363	257
ACC_07142	LOW QUALITY PROTEIN			313	203	178	205	153	36	74	50	910	364	99	192	951	504	41	8
ACC_07143	conserved hypothetical protein			151	23	21	69	21	13	15	13	277	82	76	1904	940	1284	159	262
ACC_07144	conserved hypothetical protein			34	27	25	145	35	4	17	26	40	12	18	17	4	2	17	13
ACC_07145	IQ and ubiquitin-like domain-containing protein-like		KOG2018	4	1	2	7	2	0	1	2	5	6	9	8	1	1	3	4
ACC_07146	extracellular domains-containing protein CG31004-like		KOG4291	7	2	5	4	2	1	0	1	2	9	3	1	4	2	14	5
ACC_07147	DNA-directed RNA polymerase II subunit RPB2-like	K03010	KOG0214	463	305	252	473	472	21	65	69	919	795	261	619	1121	1263	122	46
ACC_07148	putative odorant receptor 67c			0	0	0	0	1	0	0	1	5	5	74	36	0	0	0	0
ACC_07149	putative odorant receptor 13a-like			0	0	1	0	0	0	2	0	9	2	79	43	0	0	3	1
ACC_07150	odorant receptor Or2-like			0	0	0	0	0	0	0	0	9	0	35	3	0	0	0	0
ACC_07151	cadherin-89D-like		KOG1219	26	4	18	23	2	1	1	0	21	10	138	156	20	19	21	7
ACC_07152	photoreceptor-specific nuclear receptor	K08546	KOG4215	48	35	52	146	28	3	9	8	12	1	0	0	1	1	1	0
ACC_07153	LOW QUALITY PROTEIN		KOG4423	324	34	34	162	107	13	16	32	73	39	32	39	168	145	88	18
ACC_07154	phosphotriesterase-related protein-like	K07048		16	6	13	41	39	4	8	2	19	15	19	26	786	219	44	5
ACC_07155	conserved hypothetical protein	K15708		49	55	59	37	41	2	9	14	40	32	33	87	63	99	11	7
ACC_07156	transmembrane protein C9orf91 homolog			37	27	19	31	24	4	5	7	55	26	63	132	55	53	6	2
ACC_07157	sodium channel protein Nach		KOG4294	4	3	2	8	9	1	0	2	13	4	1	2	0	1	0	1
ACC_07158	lysine-specific histone demethylase 1A	K11450	KOG0029	49	24	24	55	29	3	8	8	28	37	4	16	51	62	34	22
ACC_07159	conserved hypothetical protein			128	131	141	99	127	1	25	28	213	107	247	206	308	214	22	15
ACC_07160	nudix hydrolase 8-like		KOG0648	70	53	33	95	143	15	20	16	136	109	54	188	274	182	21	10
ACC_07161	conserved hypothetical protein			2711	1369	1115	1228	1124	434	841	808	3994	1684	371	478	806	898	874	440
ACC_07162	cystathionine-beta-synthase	K01697	KOG1252	85	48	52	83	115	2	12	6	160	84	45	173	271	277	13	6
ACC_07163	protein ARV1-like		KOG3134	64	36	56	78	79	2	4	8	87	92	35	48	112	159	6	4
ACC_07164	zinc finger protein 23-like isoform 1		KOG2462	151	105	98	174	134	26	57	58	189	204	73	113	135	129	99	41
ACC_07165	LOW QUALITY PROTEIN	K01942	KOG1536	955	468	465	681	735	60	57	67	414	306	233	265	447	426	44	10
ACC_07166	alkylglycerol monooxygenase-like	K15537	KOG0872	14	26	14	33	34	3	5	1	256	115	8	11	8	1	0	0
ACC_07167	fibril-forming collagen alpha chain-like	K06236	KOG3546	2599	36	27	27	18	3	2	3	159	40	26	81	38	56	16	10
ACC_07168	conserved hypothetical protein			75	18	13	39	17	1	3	8	51	103	11	15	26	16	9	14
ACC_07169	conserved hypothetical protein		KOG4177	508	258	250	366	78	26	41	56	586	696	122	101	69	71	85	28
ACC_07170	conserved hypothetical protein	K16279	KOG4177	1238	683	584	653	203	166	237	273	1529	1682	320	275	90	94	215	111
ACC_07171	conserved hypothetical protein		KOG1046	0	0	0	0	0	1	1	0	1	10	0	0	14	3	1	0
ACC_07172	40S ribosomal protein S24-like	K02974	KOG3424	3356	3749	2745	2743	2174	761	1301	1634	3424	2938	1745	4716	7977	9770	4917	2806
ACC_07173	UDP-glucuronosyltransferase 2B7-like		KOG1192	2	0	1	8	1	2	0	0	20	19	6	7	114	78	20	1
ACC_07174	glutamate receptor, ionotropic kainate 1-like isoform 1, part		KOG1054	153	68	62	91	18	7	3	11	83	65	3	72	12	9	7	3
ACC_07175	conserved hypothetical protein			174	42	21	65	17	17	23	20	862	98	48	4759	0	1	1	0
ACC_07176	coiled-coil domain-containing protein 58-like		KOG4613	149	87	101	239	154	9	9	9	106	76	118	268	935	1069	165	88
ACC_07177	1-phosphatidylinositol-4,5-bisphosphate phosphodiesterase epsilon-1			79	59	50	225	45	6	7	12	36	24	0	0	0	0	0	0
ACC_07178	conserved hypothetical protein			41	44	62	229	102	10	16	20	33	9	12	8	4	11	2	1
ACC_07179	pyruvate kinase-like		KOG2323	0	0	0	1	2	0	0	0	1	0	0	2	0	2	0	0

ACC_07180	conserved hypothetical protein			53	6	1	2	3	1	0	1	61	8	3	241	15	125	23	55
ACC_07181	proton-coupled amino acid transporter 4-like	K14209	KOG1304	39	87	3	2	3	23	25	20	522	203	14	24	356	164	22	3
ACC_07182	conserved hypothetical protein			0	0	0	1	1	0	3	2	1	0	2	0	0	0	0	1
ACC_07183	conserved hypothetical protein		KOG1311	320	167	143	222	186	39	101	93	354	193	224	81	88	76	70	35
ACC_07184	NHL repeat-containing protein 2			705	393	465	621	622	68	116	154	733	367	329	454	421	482	58	28
ACC_07185	rhotekin-like		KOG3640	192	165	156	362	324	19	27	39	345	178	201	411	443	523	64	24
ACC_07186	conserved hypothetical protein			320	339	210	248	163	80	128	129	355	406	175	378	336	270	73	35
ACC_07187	actin-related protein 2/3 complex subunit 5	K05754	KOG3380	203	197	165	543	325	34	38	60	328	203	294	748	581	559	84	58
ACC_07188	protein FAM73B-like		KOG3831	73	51	54	84	72	5	4	7	125	60	47	45	70	75	14	5
ACC_07189	protein FAM73B-like			53	22	36	79	89	4	8	4	65	36	31	32	31	21	6	2
ACC_07190	luciferin 4-monooxygenase-like		KOG1176	21	21	9	0	0	2	1	0	239	96	9075	11678	2	2	0	0
ACC_07191	myosin heavy chain, muscle isoform 1		KOG0161	377	100	32	145	23	100	193	136	9922	8130	75	260	327	239	275	365
ACC_07192	putative inorganic phosphate cotransporter-like		KOG2532	83	80	46	58	35	17	19	21	1220	2197	62	122	418	978	165	29
ACC_07193	conserved hypothetical protein	K16449	KOG3589	504	252	248	352	154	25	30	43	535	387	115	106	50	65	68	22
ACC_07194	matrix metalloproteinase-14-like	K07763	KOG1565	31	31	26	35	14	8	9	10	88	121	35	180	59	39	37	6
ACC_07195	kinesin 3A		KOG0241	1633	1105	1056	1024	325	212	188	287	1737	2042	333	337	563	498	353	82
ACC_07196	transcription factor Sp3-like		KOG2462	8	3	3	3	2	0	3	1	11	14	14	18	2	3	15	31
ACC_07197	calmodulin-like isoform 2		KOG0027	459	220	278	595	201	32	52	69	291	155	29	36	7	11	10	14
ACC_07198	LOW QUALITY PROTEIN	K03129	KOG2341	288	257	339	309	170	31	32	46	442	344	94	144	285	416	251	91
ACC_07199	armadillo repeat-containing protein 1-like			132	98	96	114	120	4	13	13	117	92	43	183	172	147	14	6
ACC_07200	zinc finger protein 615-like		KOG2462	5	9	7	26	8	8	7	4	133	46	4	9	25	16	8	10
ACC_07201	vesicle transport protein GOT1B-like isoform 2		KOG1743	41	27	30	65	73	5	8	12	66	39	41	96	115	176	14	1
ACC_07202	aminopeptidase N-like isoform 1	K11140	KOG1046	470	266	268	441	165	29	31	31	1929	774	192	619	1251	312	72	27
ACC_07203	phospholipase B1, membrane-associated-like		KOG3670	396	289	62	296	191	62	130	89	3469	2574	14	15	2	18	1	0
ACC_07204	UPF0489 protein C5orf22 homolog			6	8	7	8	4	0	3	1	12	6	2	2	22	25	22	13
ACC_07205	glucose dehydrogenase		KOG1238	3	2	4	4	0	0	1	0	0	3	0	0	2	0	3	8
ACC_07206	LOW QUALITY PROTEIN	K15305	KOG0212	390	345	327	305	310	22	27	35	362	289	141	272	256	252	34	8
ACC_07207	GABA-gated chloride channel	K05195	KOG3643	643	209	192	761	160	18	29	40	98	50	43	6	0	2	26	11
ACC_07208	conserved hypothetical protein			1	2	2	25	12	1	0	0	2	2	5	0	3	0	60	35
ACC_07209	scavenger receptor class B member 1		KOG3776	43	19	23	54	29	19	24	23	55	54	457	835	481	285	232	310
ACC_07210	conserved hypothetical protein	K08706	KOG4216	1	2	0	3	1	0	2	1	25	26	6	4	4	4	23	7
ACC_07211	conserved hypothetical protein		KOG0494	2	2	1	9	0	0	0	2	0	1	0	0	0	0	2	2
ACC_07212	conserved hypothetical protein			4	1	2	5	3	0	0	0	1	1	0	0	0	0	6	0
ACC_07213	trafficking protein particle complex subunit 8-like		KOG1938	1526	1454	1055	892	547	209	335	348	1328	1765	356	643	1480	1089	194	58
ACC_07214	succinate dehydrogenase	K00244	KOG2403	311	187	159	287	262	16	32	25	563	418	227	517	654	980	162	29
ACC_07215	conserved hypothetical protein			66	27	35	37	27	4	6	7	39	27	16	9	2	9	12	5
ACC_07216	ras-related protein Rab-8A-like isoform 2	K07901	KOG0078	142	61	57	141	82	25	42	57	227	121	42	192	278	281	148	67
ACC_07217	conserved hypothetical protein			685	261	260	871	474	33	74	99	173	126	92	35	2	8	27	8
ACC_07218	conserved hypothetical protein	K13092		477	345	309	508	267	105	188	209	503	355	219	116	301	442	864	590
ACC_07219	RING finger protein 17-like		KOG2039	66	51	27	39	38	4	22	26	60	60	19	36	76	64	43	26
ACC_07220	conserved hypothetical protein		KOG3536	45	47	46	46	19	1	1	3	89	52	0	7	22	17	8	9
ACC_07221	conserved hypothetical protein		KOG3522	764	411	584	900	364	56	75	128	635	265	12	35	25	61	48	24
ACC_07222	dual specificity protein phosphatase 12	K14819	KOG1716	125	83	71	139	131	6	12	19	67	62	38	36	49	70	13	6
ACC_07223	conserved hypothetical protein		KOG4003	78	53	54	123	153	4	4	7	51	19	66	163	26	23	1	3
ACC_07224	Exosome complex exonuclease RRP4	K03679	KOG3013	87	57	56	116	98	7	5	9	76	51	59	113	250	379	22	25
ACC_07225	FMRFamide receptor		KOG4219	94	40	68	194	118	3	6	6	252	110	59	71	7	11	7	2
ACC_07226	conserved hypothetical protein		KOG0670	608	479	387	373	221	126	271	250	726	993	134	239	493	315	324	191
ACC_07227	homeobox protein extradenticle-like	K09355	KOG0774	248	180	212	299	116	36	68	101	254	215	94	58	40	49	217	138
ACC_07228	conserved hypothetical protein			30	9	16	39	19	4	6	2	28	3	2	3	0	1	0	0
ACC_07229	nose resistant to fluoxetine protein 6-like		KOG3700	3	0	1	2	2	0	0	0	0	0	0	1	6	12	4	0
ACC_07230	rab GTPase-activating protein 1		KOG1102	274	162	137	134	149	11	24	23	428	314	70	211	292	166	32	6
ACC_07231	ABC transporter G family member 22-like isoform 1, partial		KOG0061	60	26	28	40	10	6	4	8	84	168	5	9	24	25	67	54
ACC_07232	transmembrane protease serine 9-like		KOG3627	0	1	0	6	2	0	0	0	1	1	1	0	0	0	3	0
ACC_07233	POU domain, class 2, transcription factor 3-like	K09364	KOG3802	4	0	0	2	0	0	0	0	5	7	0	1	3	10	19	1
ACC_07234	conserved hypothetical protein		KOG0161	898	454	464	496	371	35	63	48	1499	2727	129	586	3391	1911	205	137
ACC_07235	conserved hypothetical protein		KOG1249	719	420	315	543	705	66	156	165	529	294	372	347	564	623	349	219
ACC_07236	SET and MYND domain-containing protein 4-like			3	2	2	5	4	3	4	2	28	5	3	1	1	0	3	7

ACC_07237	scavenger receptor class B member 1-like		KOG3776	322	135	97	391	235	126	196	153	752	1316	1954	1747	254	149	417	535
ACC_07238	conserved hypothetical protein			203	1	2	2	0	2	0	7	348	8	2	1555	0	19	5	1
ACC_07239	insulin-like growth factor-binding protein complex acid labil		KOG4194	339	169	144	385	415	8	10	16	148	129	54	210	41	60	12	8
ACC_07240	conserved hypothetical protein		KOG4441	38	31	22	16	11	1	2	4	100	84	13	27	9	14	8	3
ACC_07241	adenylyl cyclase-associated protein 1-like isoform 1		KOG2675	197	167	176	203	146	25	71	49	363	352	45	154	336	250	179	79
ACC_07242	FUN14 domain-containing protein 1-like isoform 1		KOG4099	294	182	185	415	483	52	153	157	804	254	358	531	222	209	83	60
ACC_07243	zinc finger protein 649-like		KOG2462	83	58	52	99	48	13	28	30	90	70	35	42	102	131	120	37
ACC_07244	tRNA modification GTPase GTPBP3, mitochondrial-	K03650	KOG1191	162	99	140	165	217	20	36	43	570	319	244	467	441	429	87	32
ACC_07245	LOW QUALITY PROTEIN		KOG2027	633	363	412	642	495	58	91	103	732	426	277	638	609	675	138	70
ACC_07246	protein phosphatase inhibitor 2-like	K16833	KOG4041	412	228	266	345	217	76	173	214	486	339	164	390	412	604	384	325
ACC_07247	conserved hypothetical protein			3	1	4	5	1	0	0	0	4	0	6	1	1	0	0	0
ACC_07248	neurexin 1 precursor		KOG3514	22	5	7	21	4	1	1	3	9	11	7	1	0	0	0	0
ACC_07249	regulator of microtubule dynamics protein 1-like			171	139	119	127	121	24	50	31	152	160	117	213	340	366	31	21
ACC_07250	conserved hypothetical protein			13	5	8	32	19	1	3	3	9	7	9	21	18	40	9	0
ACC_07251	dorsal-ventral patterning protein Sog	K04657		180	109	126	234	93	35	25	35	244	380	29	18	26	39	6	4
ACC_07252	conserved hypothetical protein			198	130	123	194	147	21	63	37	124	114	62	37	79	97	188	99
ACC_07253	mpv17-like protein 2-like	K13348	KOG1944	111	96	113	130	185	9	15	13	134	76	95	148	143	269	25	7
ACC_07254	thioredoxin domain-containing protein 12-like	K05360		0	0	0	0	1	0	0	1	0	0	0	0	0	0	0	0
ACC_07255	roquin isoform 2	K15690	KOG3161	166	125	135	115	48	28	64	64	554	797	63	263	412	277	126	62
ACC_07256	USP6 N-terminal-like protein-like		KOG1102	453	185	181	197	214	32	63	75	471	233	54	182	330	341	212	146
ACC_07257	rhythmically expressed gene 5 protein-like			59	42	46	87	69	5	5	6	57	29	58	52	21	46	25	16
ACC_07258	rhythmically expressed gene 5 protein-like			31	35	28	39	23	6	7	7	65	28	59	56	30	60	11	13
ACC_07259	rhythmically expressed gene 5 protein-like			15	12	17	26	20	0	1	6	45	26	20	27	19	40	3	1
ACC_07260	ATP-binding cassette sub-family G member 4-like		KOG0061	120	51	57	161	148	18	33	36	606	472	459	817	359	626	179	54
ACC_07261	conserved hypothetical protein		KOG1605	444	316	278	306	299	15	36	38	328	203	77	140	75	143	34	13
ACC_07262	LOW QUALITY PROTEIN	K12380	KOG2059	371	212	176	380	296	33	99	101	394	386	110	149	154	146	85	44
ACC_07263	glucose-6-phosphate 1-epimerase-like	K01792	KOG1594	55	43	27	27	27	6	2	4	223	334	81	449	258	219	32	13
ACC_07264	conserved hypothetical protein			260	164	124	320	248	52	99	127	264	236	128	98	97	110	183	93
ACC_07265	conserved hypothetical protein			162	97	76	146	140	27	72	56	141	119	48	62	85	56	94	40
ACC_07266	organic cation transporter 1-like isoform 1		KOG0255	97	86	77	284	171	16	15	32	270	260	31	280	124	110	40	26
ACC_07267	xaa-Pro aminopeptidase 1-like	K01262	KOG2413	135	114	168	529	296	8	12	7	409	167	323	856	29	26	16	5
ACC_07268	acyl-CoA Delta(11) desaturase-like	K00507	KOG1600	873	818	498	2325	1959	97	89	83	38551	30381	38	954	101	250	51	5
ACC_07269	glutamyl aminopeptidase-like	K11141	KOG1046	322	227	191	443	413	41	46	67	396	530	329	875	1611	1839	140	15
ACC_07270	poly(ADP-ribose) glycohydrolase ARH3-like			324	268	259	276	276	46	61	59	403	255	214	382	549	416	40	15
ACC_07271	pyridoxine/pyridoxamine 5'-phosphate oxidase-like		KOG2586	26	39	25	72	58	7	16	15	116	96	54	128	4992	483	25	5
ACC_07272	atrial natriuretic peptide-converting enzyme		KOG3627	180	105	109	306	176	36	32	64	485	219	100	230	276	414	74	29
ACC_07273	conserved hypothetical protein			3	4	2	4	2	1	0	0	9	11	17	19	2	9	3	0
ACC_07274	conserved hypothetical protein		KOG3686	621	532	453	1129	214	41	40	60	454	347	173	139	24	63	25	20
ACC_07275	conserved hypothetical protein			2665	1052	980	1070	1915	142	460	368	482	470	618	306	1709	2832	2766	1825
ACC_07276	homeobox protein rough-like			24	23	22	13	16	1	3	2	8	7	5	2	5	8	1	0
ACC_07277	conserved hypothetical protein		KOG0711	634	694	474	494	504	102	125	156	1890	1351	158	1986	2390	1692	193	69
ACC_07278	conserved hypothetical protein			31	34	35	149	62	1	3	10	9	6	20	20	1	5	7	7
ACC_07279	octopamine receptor beta-1R		KOG4219	157	77	74	256	102	21	22	41	86	66	28	3	6	7	14	1
ACC_07280	osmotic avoidance abnormal protein 3		KOG4280	19	23	11	35	15	9	15	9	111	117	180	272	160	38	6	6
ACC_07281	peroxisomal targeting signal 1 receptor-like	K13342	KOG1125	376	226	204	477	437	40	61	76	929	739	315	1083	1124	1014	84	29
ACC_07282	GATA zinc finger domain-containing protein 1-like			91	73	56	210	140	4	14	18	131	49	67	145	120	155	40	8
ACC_07283	partner of Y14 and mago-like isoform 1	K14294	KOG4325	736	299	201	438	366	162	310	319	328	204	447	473	771	1264	3160	1891
ACC_07284	conserved hypothetical protein			5	0	0	3	2	0	0	1	4	2	1	4	1	0	9	1
ACC_07285	conserved hypothetical protein	K11138	KOG0161	3357	2077	1812	1908	2540	451	958	1080	2190	2263	1527	880	1063	1377	1991	704
ACC_07286	LOW QUALITY PROTEIN		KOG4263	164	97	146	125	118	7	13	9	213	112	91	225	317	296	39	20
ACC_07287	furin-like protease 1			44	18	27	54	6	9	9	9	67	62	60	37	10	4	18	9
ACC_07288	polypyrimidine tract-binding protein 2-like		KOG1190	8	14	16	19	5	1	1	1	10	7	2	2	2	0	1	2
ACC_07289	nicotinic acetylcholine receptor alpha7 subunit pre	K05312	KOG3646	230	111	97	132	29	2	3	2	85	45	1	5	1	4	1	1
ACC_07290	conserved hypothetical protein			53	46	47	27	5	0	2	3	46	47	6	10	0	2	4	0
ACC_07291	conserved hypothetical protein			3	1	4	14	2	1	0	2	4	7	7	2	0	2	1	2
ACC_07292	conserved hypothetical protein			9	7	15	28	12	3	1	4	15	5	1	4	7	21	9	15
ACC_07293	ecdysone receptor-like isoform 1			281	53	53	191	36	31	48	70	152	106	35	27	8	23	169	175

ACC_07294	CDP-diacylglycerol--glycerol-3-phosphate 3-phosph	K00995	KOG3964	238	193	214	484	452	16	40	49	160	211	114	228	195	277	38	19
ACC_07295	40S ribosomal protein S18	K02964	KOG3311	1283	1652	940	1806	1613	258	450	462	1181	589	1497	1596	6268	8087	1561	1416
ACC_07296	segmentation protein even-skipped	K09320	KOG0844	1	1	0	0	0	1	0	0	0	2	0	0	2	9	26	19
ACC_07297	protein maelstrom homolog			18	21	27	18	16	2	2	4	9	27	2	31	32	13	4	2
ACC_07298	conserved hypothetical protein			8	20	8	28	23	9	10	16	863	382	144	173	2	0	0	0
ACC_07299	conserved hypothetical protein			0	0	0	1	0	0	0	0	0	3	1	0	2	1	2	0
ACC_07300	probable cytochrome P450 6a14		KOG0158	31	29	7	62	40	49	74	88	4836	2329	2	3	3858	865	49	15
ACC_07301	conserved hypothetical protein		KOG3551	83	67	67	111	24	3	4	22	105	98	31	40	24	45	50	18
ACC_07302	conserved hypothetical protein			13	14	16	68	54	1	2	3	2	3	7	85	1	1	2	0
ACC_07303	carbohydrate sulfotransferase 11-like	K01017	KOG4651	58	11	16	38	27	0	2	1	27	19	24	209	91	247	83	5
ACC_07304	recombination repair protein 1	K10771	KOG1294	128	152	127	263	230	19	34	36	65	49	121	99	285	458	200	131
ACC_07305	lachesin-like			21	12	9	30	13	1	2	3	16	14	6	2	1	3	22	6
ACC_07306	60S ribosomal protein L5	K02932	KOG0875	1702	1374	813	1324	1405	656	1032	1240	2521	1470	1875	2724	15300	19160	2136	1150
ACC_07307	LOW QUALITY PROTEIN		KOG1984	399	266	211	332	103	36	43	83	512	660	159	198	290	250	150	102
ACC_07308	farnesyl pyrophosphate synthase-like	K00787	KOG0711	324	288	199	324	270	58	90	99	655	395	127	697	698	564	145	68
ACC_07309	conserved hypothetical protein	K16679		190	77	118	481	252	9	12	13	214	106	74	173	92	140	140	15
ACC_07310	conserved hypothetical protein			143	94	114	113	135	6	26	16	197	65	249	228	179	243	18	7
ACC_07311	histone-lysine N-methyltransferase SETMAR-like			23	7	3	4	9	2	2	7	10	9	43	58	2	4	14	1
ACC_07312	conserved hypothetical protein			48	10	17	65	34	2	17	24	17	3	18	15	2	2	14	17
ACC_07313	transmembrane and coiled-coil domains protein 1-like			127	53	36	67	30	13	24	25	145	119	65	259	82	28	20	5
ACC_07314	conserved hypothetical protein		KOG2462	320	284	284	349	140	52	59	63	800	648	232	498	365	376	184	81
ACC_07315	two pore potassium channel protein sup-9-like	K05323	KOG4404	309	154	146	603	262	23	50	73	72	52	12	0	2	9	32	6
ACC_07316	conserved hypothetical protein			207	150	101	280	337	23	98	110	140	113	80	33	8	10	28	11
ACC_07317	membrane metallo-endopeptidase-like 1-like		KOG3624	52	36	38	23	13	47	25	42	496	284	191	260	7	0	3	4
ACC_07318	mannosyl-oligosaccharide alpha-1,2-mannosidase i	K01230	KOG2204	123	78	46	55	17	25	50	55	194	452	44	137	53	64	213	165
ACC_07319	matrix metalloproteinase-14-like	K07763	KOG1565	17	7	8	22	11	4	8	11	74	88	24	97	30	21	17	3
ACC_07320	telomerase reverse transcriptase	K11126	KOG1005	112	74	74	61	62	1	5	4	75	92	54	149	131	85	13	6
ACC_07321	alkaline phosphatase 4-like	K01077	KOG4126	441	230	411	814	393	48	50	85	821	546	139	257	1053	2476	1269	79
ACC_07322	calcium-dependent secretion activator		KOG3543	1741	1106	1064	1015	453	67	145	181	1554	1080	240	199	81	82	39	14
ACC_07323	mariner transposase			13	12	14	35	20	6	4	3	18	13	19	22	8	16	2	3
ACC_07324	peptidyl-prolyl cis-trans isomerase-like 1-like	K12733	KOG0881	83	50	34	129	91	6	9	11	122	113	49	133	258	419	63	31
ACC_07325	transcription elongation regulator 1 isoform 1	K12824	KOG0155	2028	1899	1931	1983	1191	393	850	875	1993	1066	932	360	1257	2011	6181	5175
ACC_07326	conserved hypothetical protein			169	106	122	172	214	3	4	4	148	78	54	99	85	64	4	0
ACC_07327	ankyrin repeat and SAM domain-containing protein 1A-like		KOG0507	30	12	7	12	10	0	3	5	49	76	10	22	5	8	11	2
ACC_07328	conserved hypothetical protein		KOG1030	101	103	119	161	31	3	0	10	159	164	11	26	24	10	35	9
ACC_07329	conserved hypothetical protein			539	482	500	770	385	69	90	116	661	443	234	159	317	349	110	33
ACC_07330	conserved hypothetical protein		KOG2669	434	342	332	476	198	50	78	90	482	370	135	98	142	225	232	82
ACC_07331	F-box/LRR-repeat protein 16-like	K10282	KOG1947	1085	480	559	1776	603	104	133	199	763	276	122	91	12	35	71	25
ACC_07332	conserved hypothetical protein			29	15	16	90	45	11	11	14	692	691	7	133	61	115	25	41
ACC_07333	protein krueppel	K09231	KOG2462	35	54	52	44	7	5	5	6	53	41	7	1	15	45	102	23
ACC_07334	deoxycytidylate deaminase-like isoform 1	K01493	KOG3127	39	35	31	44	34	1	5	7	29	44	16	28	173	251	27	9
ACC_07335	septin-2		KOG3859	622	314	308	887	553	74	176	193	457	275	349	925	390	538	462	295
ACC_07336	conserved hypothetical protein			320	119	157	285	336	62	161	125	151	77	101	18	48	182	263	120
ACC_07337	vanin-like protein 1-like		KOG0806	211	102	81	85	78	19	71	76	97	162	47	187	4296	1966	695	94
ACC_07338	MIP18 family protein CG30152-like		KOG3381	165	77	104	318	243	14	16	18	402	264	109	342	415	422	92	19
ACC_07339	conserved hypothetical protein			0	0	1	0	0	0	0	0	1	0	0	0	115	101	33	4
ACC_07340	1-acyl-sn-glycerol-3-phosphate acyltransferase alpha-like		KOG2848	161	130	103	390	361	39	56	40	3287	1380	16	647	598	194	23	16
ACC_07341	1-acyl-sn-glycerol-3-phosphate acyltransferase alpha-like		KOG2848	43	16	13	48	17	12	11	13	75	81	48	118	306	341	74	77
ACC_07342	conserved hypothetical protein			4	9	8	16	12	2	4	2	7	17	2	7	15	9	7	5
ACC_07343	protein rhomboid-like, partial	K02857	KOG2289	27	6	2	26	7	2	13	9	5	5	59	23	1	1	26	6
ACC_07344	conserved hypothetical protein			696	433	336	281	463	69	232	235	180	146	183	61	430	837	1623	1288
ACC_07345	conserved hypothetical protein			44	22	28	23	38	2	8	10	8	24	10	11	58	61	28	11
ACC_07346	adenylate kinase isoenzyme 5-like		KOG3079	108	144	116	228	161	23	29	28	172	91	1069	1569	69	179	98	20
ACC_07347	putative phosphatidate phosphatase-like, partial	K01080	KOG3030	83	101	55	115	99	24	42	34	122	138	52	137	107	65	21	8
ACC_07348	LOW QUALITY PROTEIN	K07204	KOG1517	681	426	344	508	407	29	42	40	1004	989	126	256	592	624	49	27
ACC_07349	conserved hypothetical protein		KOG1418	43	14	15	30	12	1	2	4	41	67	1395	1919	54	17	1	1
ACC_07350	radial spoke head 10 homolog B2-like		KOG0231	289	268	272	435	439	34	67	73	268	365	408	693	156	180	47	30

ACC_07351	conserved hypothetical protein			6	3	7	21	18	0	0	0	1	6	1	1	0	1	0	0
ACC_07352	multidrug resistance-associated protein 7-like		KOG0054	140	87	69	149	192	14	21	21	327	286	78	82	273	166	11	3
ACC_07353	transmembrane and TPR repeat-containing protein CG4341-		KOG4626	251	98	137	311	33	26	38	60	44	75	5	6	2	2	102	90
ACC_07354	Down syndrome cell adhesion molecule		KOG3513	116	47	49	105	11	4	3	3	65	53	6	4	6	2	6	7
ACC_07355	translation initiation factor eIF-2B subunit alpha	K03239	KOG1466	50	28	29	68	63	12	8	7	60	50	41	81	87	102	14	6
ACC_07356	conserved hypothetical protein		KOG3284	322	225	220	532	421	49	67	82	430	238	247	583	441	498	157	59
ACC_07357	Tubulin beta-3 chain	K07375	KOG1375	82	29	43	148	35	5	2	7	33	32	11	6	114	179	467	724
ACC_07358	valacyclovir hydrolase-like		KOG2984	95	54	70	93	99	24	42	30	1829	856	38	16	204	417	48	11
ACC_07359	conserved hypothetical protein			291	164	204	542	170	30	25	24	540	335	263	717	110	142	132	26
ACC_07360	fatty acid 2-hydroxylase-like		KOG0539	107	50	74	136	144	4	12	10	38	13	64	91	292	208	8	3
ACC_07361	conserved hypothetical protein		KOG1924	1221	1303	1607	1837	1488	243	436	529	1223	838	295	178	230	397	405	283
ACC_07362	conserved hypothetical protein			95	39	38	182	30	6	2	6	40	19	14	3	2	0	3	6
ACC_07363	acyl-CoA synthetase family member 2, mitochondrial-like, pa		KOG1177	21	5	4	25	3	8	7	4	1765	828	31	21	538	216	85	55
ACC_07364	sphingosine-1-phosphate phosphatase 1-like	K04716	KOG2822	222	148	139	244	260	19	32	45	379	335	116	342	622	634	47	13
ACC_07365	conserved hypothetical protein			599	232	336	257	67	3	3	12	124	96	3	16	17	119	177	15
ACC_07366	ATP-binding cassette sub-family G member 1-like		KOG0061	312	239	180	276	239	53	78	72	851	547	308	190	192	140	33	3
ACC_07367	40S ribosomal protein S28	K02979	KOG3502	104	99	84	200	115	23	30	30	134	82	167	308	42	28	16	7
ACC_07368	cubilin-like		KOG4292	340	113	103	238	98	18	25	32	250	189	59	86	75	58	29	11
ACC_07369	conserved hypothetical protein	K11411	KOG2684	686	365	321	580	649	127	347	371	487	482	282	165	139	204	559	251
ACC_07370	LOW QUALITY PROTEIN		KOG1989	4469	3270	3119	3562	3794	954	2131	2230	3886	2656	1957	1254	1659	1576	2425	1394
ACC_07371	lipase maturation factor 2-like isoform 1			608	401	374	730	611	41	65	87	979	683	543	764	1080	910	63	19
ACC_07372	biogenesis of lysosome-related organelles complex	K16750	KOG4559	132	99	119	156	132	11	7	20	177	91	69	326	116	128	13	3
ACC_07373	LOW QUALITY PROTEIN			5	7	4	8	8	0	2	0	9	10	2	0	6	3	1	0
ACC_07374	Histone-lysine N-methyltransferase SETMAR			0	1	0	1	0	0	0	0	0	1	0	1	0	0	0	0
ACC_07375	conserved hypothetical protein			197	75	86	103	40	13	14	22	155	88	13	59	21	17	14	4
ACC_07376	melanoma-associated antigen G1-like isoform 2		KOG4562	94	54	48	75	87	3	10	13	81	46	69	150	113	172	27	10
ACC_07377	probable tubulin polyglutamylase TTLL9-like isoforr	K16603	KOG2157	2	2	4	15	3	2	0	1	6	9	1	4	0	0	4	0
ACC_07378	conserved hypothetical protein			129	84	61	154	97	34	43	55	432	139	113	107	77	75	123	77
ACC_07379	conserved hypothetical protein			723	612	455	1131	474	608	920	1068	1506	884	476	408	131	136	2013	1367
ACC_07380	serine/threonine-protein kinase/endoribonuclease	K08852	KOG1027	1024	866	776	1468	801	196	273	341	1703	1230	787	965	1279	1591	640	159
ACC_07381	meteorin precursor			199	107	114	382	263	36	50	58	405	149	162	178	173	196	81	29
ACC_07382	guanine nucleotide-binding protein G(o) subunit al	K04534	KOG0082	369	185	168	305	131	21	16	29	305	137	26	69	78	81	32	9
ACC_07383	conserved hypothetical protein			1	2	1	1	1	0	0	1	31	59	6	4	31	94	9	7
ACC_07384	conserved hypothetical protein	K16220		676	471	445	840	994	48	84	105	1191	542	691	1924	1155	1000	61	31
ACC_07385	conserved hypothetical protein		KOG0509	152	85	137	636	201	20	34	32	245	196	112	262	106	46	97	40
ACC_07386	corazonin receptor		KOG4219	4	5	3	28	3	2	2	2	136	76	42	20	1	0	0	1
ACC_07387	DENN domain-containing protein 5B-like		KOG2080	325	221	191	181	164	22	23	30	960	893	84	189	443	169	20	14
ACC_07388	UPF0510 protein INM02-like		KOG4827	366	397	404	722	477	74	90	119	445	275	453	841	637	735	177	104
ACC_07389	conserved hypothetical protein			5	2	3	9	6	0	1	0	2	2	0	1	1	2	4	7
ACC_07390	conserved hypothetical protein		KOG0843	1	0	1	1	0	0	0	0	1	0	1	0	0	0	0	0
ACC_07391	GTP-binding protein 2-like		KOG1143	258	205	209	305	233	53	65	105	1008	464	261	337	207	176	61	19
ACC_07392	chaoptin-like		KOG4194	669	178	259	517	141	7	12	18	124	72	63	67	2	1	8	7
ACC_07393	conserved hypothetical protein		KOG4412	226	182	160	159	52	23	24	26	413	327	54	36	124	132	63	60
ACC_07394	follistatin-related protein 5-like		KOG4221	1208	324	367	723	452	19	44	65	474	278	155	228	97	187	40	16
ACC_07395	odorant receptor 109			0	0	0	1	0	0	0	0	6	2	240	56	0	0	0	0
ACC_07396	odorant receptor 109			0	0	0	0	0	0	0	0	4	2	94	52	0	0	0	0
ACC_07397	lysine-specific demethylase NO66-like		KOG3706	71	68	43	193	178	5	13	29	64	61	80	66	496	753	245	197
ACC_07398	protein argonaute-2	K11593	KOG1041	516	289	231	300	161	53	63	98	573	586	112	145	144	141	82	19
ACC_07399	conserved hypothetical protein		KOG2462	44	54	51	89	44	22	25	41	145	142	213	321	30	28	4	6
ACC_07400	conserved hypothetical protein			0	0	0	1	0	0	0	0	0	0	0	0	0	0	0	0
ACC_07401	conserved hypothetical protein			2	1	1	4	1	1	1	2	11	7	0	1	1	1	3	0
ACC_07402	LOW QUALITY PROTEIN		KOG2169	441	247	220	401	136	30	56	68	755	1077	313	627	259	197	89	39
ACC_07403	conserved hypothetical protein		KOG0161	178	118	106	170	108	42	60	80	145	67	234	75	119	126	142	84
ACC_07404	conserved hypothetical protein			434	283	330	327	109	22	31	34	144	82	243	105	2	7	18	3
ACC_07405	importin subunit alpha-7 isoform 1		KOG0166	310	171	136	283	228	19	16	26	872	767	123	546	930	841	92	37
ACC_07406	conserved hypothetical protein		KOG2462	196	126	115	204	133	23	53	54	337	606	25	71	146	135	146	105
ACC_07407	UPF0568 protein C14orf166 homolog	K15433	KOG4380	335	270	246	386	395	29	43	52	390	168	284	677	620	631	87	30

ACC_07408	conserved hypothetical protein			1041	687	560	755	598	34	52	84	716	855	77	885	721	715	83	20
ACC_07409	serine/threonine-protein kinase minibrain-like	K08825	KOG0667	1765	815	741	2123	967	210	394	509	1384	1017	490	213	480	1011	1960	1045
ACC_07410	voltage-dependent calcium channel subunit alpha-1	K05316	KOG2353	864	734	797	823	295	51	41	67	592	415	48	69	43	62	22	10
ACC_07411	cullin-associated NEDD8-dissociated protein 1-like isoform 1		KOG1824	838	629	628	1257	987	47	87	99	1009	1101	288	616	1522	1481	140	49
ACC_07412	conserved hypothetical protein			1078	561	517	992	536	160	221	312	1027	539	269	548	988	1382	1030	826
ACC_07413	transmembrane protein 222-like		KOG3150	128	108	113	211	156	25	21	30	216	145	117	393	194	137	20	16
ACC_07414	multiple epidermal growth factor-like domains protein 10-lik		KOG0994	587	301	254	360	326	58	79	84	1403	1516	316	702	510	613	101	23
ACC_07415	dynactin subunit 5-like	K10427	KOG3121	140	67	68	177	44	23	24	32	148	78	101	166	69	104	218	102
ACC_07416	conserved hypothetical protein			671	492	449	882	932	77	205	258	445	450	353	370	456	517	402	161
ACC_07417	Rad54 protein isoform 1	K10875	KOG0390	469	360	303	457	562	31	70	57	655	604	282	620	930	1098	127	53
ACC_07418	conserved hypothetical protein		KOG0260	287	203	212	456	246	32	36	45	3311	1285	336	1700	746	75	41	66
ACC_07419	conserved hypothetical protein		KOG1215	96	84	69	210	89	19	9	22	331	166	48	145	93	52	102	20
ACC_07420	conserved hypothetical protein			140	202	301	748	268	72	96	176	174	110	31	27	67	45	52	80
ACC_07421	conserved hypothetical protein			1011	855	938	919	921	103	162	147	892	491	558	166	272	303	49	18
ACC_07422	acetylcholinesterase	K01049	KOG4389	350	207	331	247	205	15	33	47	236	278	5	18	8	10	11	4
ACC_07423	organic cation transporter protein-like		KOG0255	0	0	0	0	0	0	0	0	4	2	0	0	1	1	1	1
ACC_07424	conserved hypothetical protein			239	120	116	259	134	13	14	23	80	116	8	21	108	605	159	78
ACC_07425	WD repeat-containing protein mio-B		KOG1008	335	236	223	446	436	45	44	60	657	482	333	680	550	548	62	20
ACC_07426	potassium voltage-gated channel protein eag-like			487	182	223	413	183	78	159	194	319	211	169	59	9	18	22	21
ACC_07427	golgin subfamily A member 7B-like isoform 1		KOG4069	71	38	36	138	60	6	6	5	59	56	15	24	57	33	27	4
ACC_07428	conserved hypothetical protein		KOG0992	1033	646	502	667	778	132	353	403	517	524	521	336	414	320	291	164
ACC_07429	f-box/LRR-repeat protein 7-like isoform 2	K10273	KOG4341	35	47	25	82	91	19	12	14	196	99	73	188	213	169	30	5
ACC_07430	serine/threonine-protein kinase polo	K06631	KOG0575	221	89	76	81	128	19	38	26	217	146	76	253	247	337	48	27
ACC_07431	4-hydroxybutyrate coenzyme A transferase-like isoform 1		KOG2828	251	145	173	271	227	21	37	34	252	57	96	30	122	123	27	5
ACC_07432	conserved hypothetical protein		KOG3950	477	277	277	730	593	18	30	40	325	248	146	356	129	111	15	5
ACC_07433	vacuolar protein sorting-associated protein 13C-like		KOG1809	3042	1989	1926	2909	2434	248	434	576	1463	1265	791	552	304	301	116	35
ACC_07434	conserved hypothetical protein			77	30	39	78	25	1	6	9	21	79	9	18	3	0	10	3
ACC_07435	conserved hypothetical protein			776	236	253	701	201	27	28	49	190	132	33	18	32	43	39	34
ACC_07436	chondroitin sulfate synthase 2-like	K03419	KOG3708	313	187	191	197	290	8	30	27	448	468	240	548	436	392	24	8
ACC_07437	putative RNA-binding protein Luc7-like 2-like		KOG0796	1221	768	786	1952	982	173	298	338	1085	566	476	493	740	883	732	363
ACC_07438	ceramide kinase-like	K04715	KOG1115	789	617	642	487	342	94	134	131	910	572	228	292	179	173	69	21
ACC_07439	ceramide kinase-like			84	63	59	49	26	15	17	30	88	130	44	117	22	17	8	2
ACC_07440	serine/arginine-rich splicing factor 4-like isoform 1	K12893	KOG0106	1120	623	498	592	197	149	130	200	736	856	312	809	836	1165	448	235
ACC_07441	conserved hypothetical protein			178	15	29	79	10	4	4	6	154	35	19	571	859	791	1320	727
ACC_07442	conserved hypothetical protein		KOG1215	148	13	31	34	7	5	4	5	182	202	10	333	1064	738	1267	895
ACC_07443	clavesin-1-like		KOG1471	93	244	88	200	125	131	114	125	5259	4707	981	2874	1442	775	201	63
ACC_07444	dolichyl-diphosphooligosaccharide--protein glycosy	K12668	KOG1746	113	117	114	284	211	15	18	25	82	71	219	225	486	352	28	12
ACC_07445	farnesyl pyrophosphate synthase-like		KOG0711	18	10	7	8	9	0	1	4	8	17	3	2	12	9	7	2
ACC_07446	sodium/potassium/calcium exchanger 3-like		KOG1307	35	39	37	67	47	3	14	10	20	24	8	10	1154	1750	83	9
ACC_07447	reverse transcriptase			66	294	165	94	26	22	43	66	83	593	30	1	54	94	186	77
ACC_07448	alpha-tubulin N-acetyltransferase-like		KOG4601	361	214	204	432	243	48	56	91	881	286	157	214	193	121	36	17
ACC_07449	conserved hypothetical protein		KOG4425	597	305	284	463	423	132	282	308	516	483	218	130	160	210	345	138
ACC_07450	conserved hypothetical protein		KOG4425	195	167	106	235	76	17	36	40	294	344	43	130	178	164	48	13
ACC_07451	Down syndrome cell adhesion molecule homolog isoform 1			176	147	266	365	71	22	54	61	86	106	26	18	8	6	90	39
ACC_07452	protein couch potato-like		KOG1457	654	393	498	966	182	91	147	213	232	156	1220	146	17	17	218	268
ACC_07453	eukaryotic peptide chain release factor subunit 1-li	K03265	KOG0688	165	123	96	152	107	21	36	45	331	502	55	203	1052	1160	480	233
ACC_07454	nephrin-like isoform 1	K06781	KOG3515	22	6	8	30	8	0	2	0	8	6	0	0	36	12	4	0
ACC_07455	LOW QUALITY PROTEIN	K15621	KOG1328	544	236	280	850	133	28	27	32	237	137	200	193	2	12	10	6
ACC_07456	conserved hypothetical protein			44	34	21	54	76	2	6	3	22	23	34	64	65	47	12	6
ACC_07457	conserved hypothetical protein			1	3	2	2	1	1	2	3	2	4	1	1	2	1	2	0
ACC_07458	conserved hypothetical protein		KOG3345	470	442	430	472	581	118	252	285	390	257	351	245	300	365	410	280
ACC_07459	conserved hypothetical protein	K03627	KOG3398	634	527	544	997	598	74	74	136	799	235	267	1208	703	746	179	86
ACC_07460	conserved hypothetical protein			404	241	244	592	190	41	67	46	2871	1790	99	51	97	59	49	23
ACC_07461	conserved hypothetical protein		KOG1029	224	239	224	69	33	53	40	57	228	87	44	162	36	179	249	70
ACC_07462	multiple C2 and transmembrane domain-containing protein		KOG1030	415	268	316	792	227	47	34	59	585	544	94	139	110	78	83	30
ACC_07463	Ubiquinone biosynthesis protein COQ9, mitochondrial		KOG2969	436	254	253	584	498	46	68	71	726	316	316	648	516	549	130	54
ACC_07464	conserved hypothetical protein			9	6	7	40	20	4	4	0	17	11	5	0	2	3	1	0

ACC_07465	conserved hypothetical protein			1	4	4	4	0	0	0	2	0	7	0	0	0	1	1	0
ACC_07466	similar to CG41536 CG41536-PA, partial			264	82	38	314	92	72	104	222	200	207	107	336	4	5	968	820
ACC_07467	LOW QUALITY PROTEIN			10	3	5	4	3	0	1	0	7	4	1	5	2	1	3	0
ACC_07468	conserved hypothetical protein			987	209	90	815	230	242	308	671	630	930	319	983	21	10	4942	7683
ACC_07469	LOW QUALITY PROTEIN			147	110	152	448	146	14	16	29	99	80	8	11	8	19	12	3
ACC_07470	conserved hypothetical protein	K08960	KOG1163	486	350	294	522	279	119	160	241	546	496	171	223	172	224	443	255
ACC_07471	lethal(2) giant larvae protein-like isoform 2	K06094	KOG1983	249	128	116	143	87	16	22	38	350	347	54	187	1296	1025	181	46
ACC_07472	glutaminyl-peptide cyclotransferase-like	K00683	KOG3946	320	237	186	401	387	13	31	29	603	292	302	1072	262	268	24	8
ACC_07473	conserved hypothetical protein	K10482	KOG3840	100	41	28	135	106	18	14	22	138	58	80	72	68	110	32	16
ACC_07474	ATP-dependent DNA helicase Q4	K10730	KOG0351	925	440	372	717	950	68	244	238	446	458	349	307	584	745	711	311
ACC_07475	conserved hypothetical protein			7	9	4	0	0	0	1	2	0	5	1	1	14	4	19	21
ACC_07476	farnesyl pyrophosphate synthase-like		KOG0711	16	10	6	10	10	0	2	3	5	13	1	1	9	4	5	2
ACC_07477	transcription initiation factor TFIID subunit 3-like		KOG1973	261	142	140	354	278	68	109	137	191	96	154	64	111	133	621	278
ACC_07478	histone chaperone asf1	K10753	KOG3265	122	88	52	190	145	13	17	35	178	123	93	254	375	512	109	34
ACC_07479	eukaryotic translation initiation factor 3 subunit A-l	K03254	KOG2072	1445	1208	800	789	412	233	449	463	2067	1429	428	585	3357	5801	4534	3977
ACC_07480	PAB-dependent poly(A)-specific ribonuclease subui	K12572	KOG3741	544	387	407	641	399	69	72	85	660	299	334	330	336	376	80	14
ACC_07481	eukaryotic translation initiation factor 3 subunit I	K03246	KOG0643	260	172	150	403	304	33	57	77	403	229	216	408	1369	2331	229	119
ACC_07482	conserved hypothetical protein		KOG2462	133	169	231	120	13	4	4	4	52	69	15	10	0	2	4	2
ACC_07483	palmitoyltransferase ZDHHC3-like		KOG1311	55	34	39	52	50	5	7	7	69	64	22	83	95	119	18	4
ACC_07484	conserved hypothetical protein			20	33	33	104	47	7	5	8	228	60	27	52	7	7	4	1
ACC_07485	conserved hypothetical protein			1236	530	283	2053	373	433	486	711	1975	895	346	1133	210	78	5156	3813
ACC_07486	reverse transcriptase			1358	210	99	3013	972	429	805	1276	697	1972	845	2133	23	24	15023	9794
ACC_07487	farnesyl pyrophosphate synthase-like		KOG0711	18	15	8	29	21	1	2	4	10	19	4	5	12	20	11	4
ACC_07488	cholecystokinin receptor-like		KOG4219	4	2	3	3	2	0	0	1	7	4	0	0	1	0	0	0
ACC_07489	tctex1 domain-containing protein 2-like		KOG4108	60	49	49	107	87	6	7	8	202	76	76	119	76	71	7	8
ACC_07490	conserved hypothetical protein		KOG2370	649	489	310	706	638	157	300	354	597	424	474	471	477	632	1004	756
ACC_07491	vacuolar protein-sorting-associated protein 25	K12189	KOG4068	158	139	150	194	198	13	31	28	298	102	151	406	247	350	36	13
ACC_07492	conserved hypothetical protein	K16550	KOG0161	52	37	52	106	37	6	9	10	83	16	45	33	39	77	33	9
ACC_07493	conserved hypothetical protein			231	145	171	372	158	59	83	71	569	269	96	58	643	855	1117	553
ACC_07494	facilitated trehalose transporter Tret1-2 homolog		KOG0254	127	33	28	91	31	2	7	15	37	34	2	4	3	3	33	8
ACC_07495	conserved hypothetical protein		KOG3882	282	67	88	171	114	9	15	14	91	18	2	8	7	51	41	17
ACC_07496	histone-lysine N-methyltransferase, H3 lysine-79 sp	K11427	KOG3924	425	377	393	408	122	27	63	78	700	559	77	143	414	332	241	123
ACC_07497	kinesin 4A isoform 1	K10395	KOG0244	1507	1042	1070	925	596	224	379	418	1726	1146	349	453	520	494	535	282
ACC_07498	lysM and putative peptidoglycan-binding domain-containing		KOG2850	57	56	53	75	90	8	12	21	97	85	48	151	164	91	11	1
ACC_07499	conserved hypothetical protein			15	8	2	41	26	2	6	7	367	778	36	95	199	109	14	7
ACC_07500	conserved hypothetical protein		KOG1052	1	0	0	1	1	0	0	0	5	0	3	0	0	1	2	0
ACC_07501	potassium/sodium hyperpolarization-activated cyclic nucleo		KOG0498	0	0	2	0	0	0	0	0	1	0	1	0	2	1	4	1
ACC_07502	tripartite motif-containing protein 2-like	K11997	KOG2177	459	196	201	291	264	40	37	78	430	207	88	117	130	141	27	7
ACC_07503	conserved hypothetical protein		KOG0811	60	35	36	22	19	2	3	2	59	61	9	51	26	27	0	0
ACC_07504	glycine receptor subunit alpha-2		KOG3644	21	6	9	23	13	1	3	1	69	39	4	2	56	55	7	2
ACC_07505	katanin p60 ATPase-containing subunit A-like 2-like		KOG0738	4	11	7	11	15	2	2	4	3	3	27	26	22	8	2	1
ACC_07506	A disintegrin and metalloproteinase with thrombospondin m		KOG3538	14	13	6	45	14	6	3	4	65	80	73	49	14	15	41	24
ACC_07507	A disintegrin and metalloproteinase with thrombospondin m		KOG3538	39	15	23	29	14	8	7	13	21	27	7	13	12	11	42	23
ACC_07508	A disintegrin and metalloproteinase with thrombospondin m		KOG3538	31	20	27	34	6	1	3	1	9	15	9	22	1	5	6	2
ACC_07509	conserved hypothetical protein			1976	410	203	2674	512	477	656	1365	939	1980	761	2059	37	14	12601	16654
ACC_07510	conserved hypothetical protein			57286	9958	4163	20515	4580	11573	26900	43852	18821	32874	6451	35793	852	442	270712	303629
ACC_07511	probable GDP-L-fucose synthase	K02377	KOG1431	179	140	100	283	269	13	18	27	209	175	157	453	471	404	59	22
ACC_07512	rapamycin-insensitive companion of mTOR-like	K08267	KOG3694	585	391	404	988	548	67	82	112	542	335	184	183	372	459	137	46
ACC_07513	methylenetetrahydrofolate reductase-like	K00297	KOG0564	53	71	46	61	77	5	8	7	117	68	124	427	24	15	3	0
ACC_07514	nicastrin	K06171	KOG2657	190	90	80	112	103	8	9	16	282	324	78	216	293	296	24	0
ACC_07515	conserved hypothetical protein		KOG3598	407	340	324	237	146	48	60	57	482	368	90	116	858	1336	536	111
ACC_07516	eukaryotic translation initiation factor 4H-like		KOG0108	579	401	347	340	261	45	71	98	752	970	78	529	1007	1295	376	98
ACC_07517	conserved hypothetical protein	K13761	KOG3689	259	71	109	271	46	10	10	5	156	189	56	26	39	28	85	60
ACC_07518	conserved hypothetical protein	K09091	KOG4304	106	104	121	158	41	22	14	23	207	237	63	166	120	183	352	38
ACC_07519	conserved hypothetical protein	K12841	KOG4368	277	226	232	243	106	18	27	31	406	245	104	135	291	399	153	50
ACC_07520	conserved hypothetical protein			16	3	3	10	3	1	3	1	7	5	1	0	17	14	20	21
ACC_07521	conserved hypothetical protein			1494	605	684	383	247	204	434	378	640	441	84	60	276	190	285	199

ACC_07522	DNA mismatch repair protein Msh2	K08735	KOG0219	400	262	207	294	322	13	34	37	217	156	108	292	568	548	54	15
ACC_07523	facilitated trehalose transporter Tret1-like, partial		KOG0254	4	1	0	0	1	1	1	0	4	1	0	0	120	30	2	1
ACC_07524	conserved hypothetical protein			5	2	2	2	1	0	3	2	5	3	0	0	0	0	2	1
ACC_07525	dephospho-CoA kinase domain-containing protein-like		KOG3220	176	120	114	231	254	20	31	37	262	133	143	335	244	270	32	18
ACC_07526	ATP synthase subunit g, mitochondrial-like	K02140	KOG4103	105	45	56	380	328	8	11	15	128	35	290	202	368	389	36	14
ACC_07527	small nuclear ribonucleoprotein-associated protein	K11086	KOG3168	201	87	105	394	253	26	17	30	205	171	203	472	414	811	344	251
ACC_07528	conserved hypothetical protein			116	97	86	78	120	5	10	7	73	86	80	149	19	18	2	0
ACC_07529	ras-like GTP-binding protein RhoL-like		KOG0393	70	29	25	53	46	23	51	51	185	114	272	1113	272	397	204	38
ACC_07530	rab3 GTPase-activating protein catalytic subunit		KOG2390	152	122	121	197	116	22	31	23	125	56	72	89	66	71	15	9
ACC_07531	rab3 GTPase-activating protein catalytic subunit		KOG2390	115	93	81	94	83	13	14	15	163	143	33	179	125	89	4	3
ACC_07532	ubiquitin-conjugating enzyme E2Q-like protein CG4	K10582	KOG0897	382	152	166	268	126	20	25	27	419	126	367	51	175	205	58	16
ACC_07533	39S ribosomal protein L15, mitochondrial-like	K02876	KOG0846	276	196	191	252	242	23	54	62	427	292	167	614	886	1038	102	65
ACC_07534	NFU1 iron-sulfur cluster scaffold homolog, mitochondrial-lik		KOG2358	330	258	272	496	451	35	67	98	343	169	242	477	558	731	83	48
ACC_07535	conserved hypothetical protein			306	187	188	135	50	27	16	28	1091	862	123	1002	587	260	159	57
ACC_07536	innexin inx7-like			21	24	30	96	70	0	0	2	154	24	24	17	401	345	61	3
ACC_07537	chromosome-associated kinesin KIF4A	K10395	KOG0244	169	124	102	121	160	25	59	61	171	125	132	74	349	512	563	262
ACC_07538	c-Maf-inducing protein-like isoform 1			164	110	93	208	91	21	20	40	276	224	66	63	18	17	15	9
ACC_07539	xaa-Pro dipeptidase		KOG2737	96	64	50	53	66	11	8	8	142	151	32	269	341	222	6	0
ACC_07540	GSK-3-binding protein-like			266	179	189	254	162	47	78	107	358	243	67	63	84	104	159	88
ACC_07541	e3 ubiquitin-protein ligase RNF123-like	K12169	KOG4692	739	505	480	820	750	61	75	108	975	576	397	490	375	339	28	7
ACC_07542	reverse transcriptase			59	293	214	81	26	27	52	85	66	450	20	6	7	26	145	74
ACC_07543	pumilio homolog 2		KOG1488	866	664	684	1350	585	139	216	333	754	735	191	91	143	222	1068	448
ACC_07544	thyroid adenoma-associated protein homolog		KOG1810	152	120	124	150	159	3	17	17	117	164	58	96	350	337	26	17
ACC_07545	ultrabithorax	K09311		0	0	1	0	0	0	0	0	0	1	0	0	18	8	65	27
ACC_07546	CDC42 small effector protein homolog			77	42	30	41	17	3	3	2	41	13	18	57	96	103	24	2
ACC_07547	programmed cell death protein 2	K14801	KOG2061	385	241	232	346	451	33	139	126	443	289	434	697	682	835	304	194
ACC_07548	LSM domain-containing protein 1-A-like		KOG3168	64	30	25	127	143	2	10	9	52	45	61	150	108	148	19	4
ACC_07549	carbohydrate sulfotransferase 11-like		KOG4651	15	14	20	29	26	0	0	0	24	36	11	45	32	28	1	1
ACC_07550	YTH domain-containing protein 1-like		KOG1902	244	134	131	199	135	18	29	24	320	185	66	91	239	305	74	28
ACC_07551	conserved hypothetical protein		KOG0018	2086	1327	1025	753	1410	275	675	704	1089	899	895	570	675	1005	1286	591
ACC_07552	eukaryotic translation initiation factor 5B		KOG1144	2228	1379	1384	1677	1708	508	1217	1268	1604	1165	832	513	1805	3727	8005	8879
ACC_07553	elongation of very long chain fatty acids protein AAEL0080(		KOG3071	13	7	6	9	6	2	1	3	27	28	10	74	203	95	11	1
ACC_07554	conserved hypothetical protein		KOG1187	21	25	15	24	7	1	7	1	144	149	49	56	45	72	32	3
ACC_07555	LOW QUALITY PROTEIN	K01533	KOG0207	306	241	153	270	248	31	37	57	612	741	80	257	472	534	60	17
ACC_07556	similar to CG41536 CG41536-PA, partial			8528	3011	1336	10271	3228	2768	4618	9097	4925	5389	2471	8716	183	119	88498	58425
ACC_07557	conserved hypothetical protein		KOG3598	184	137	160	363	104	9	9	18	72	76	44	27	0	1	14	3
ACC_07558	midasin-like	K14572	KOG1808	169	131	125	300	225	13	31	39	152	168	88	121	183	268	65	34
ACC_07559	conserved hypothetical protein			314	36	30	70	57	2	21	13	78	243	5	21	8888	3101	372	311
ACC_07560	polycomb protein Scm		KOG3766	58	43	40	82	51	4	3	10	70	79	14	53	78	61	18	7
ACC_07561	UTP--glucose-1-phosphate uridylyltransferase isofc	K00963	KOG2638	479	276	268	697	644	77	139	166	2989	1795	1468	2220	4103	1596	166	96
ACC_07562	NADPH--cytochrome P450 reductase-like	K00327	KOG1158	1117	815	893	1378	1393	211	328	349	4331	2830	5484	12243	2447	1745	263	176
ACC_07563	NADPH--cytochrome P450 reductase-like	K07921	KOG0094	0	2	0	0	2	0	0	0	1	1	0	3	0	0	0	0
ACC_07564	hexamerin 70b precursor			36	38	59	139	53	4	7	5	1523	42	22	33	545041	41534	501	1291
ACC_07565	phospholipase B1, membrane-associated-like		KOG3670	124	13	10	108	52	4	2	4	57	15	5	25	94	70	15	3
ACC_07566	conserved hypothetical protein		KOG4566	72	59	57	92	74	5	11	10	626	359	42	252	158	164	52	25
ACC_07567	FMRFamide receptor-like		KOG4219	11	7	3	15	13	0	1	1	5	8	4	13	21	9	6	2
ACC_07568	conserved hypothetical protein		KOG0285	2028	1152	1470	1506	935	224	500	383	1219	842	325	176	493	864	1857	859
ACC_07569	myosin heavy chain, muscle isoform 1		KOG0161	68	11	3	30	9	14	39	17	1594	1009	28	41	123	104	46	35
ACC_07570	glucose dehydrogenase		KOG1238	2	5	0	4	3	0	0	0	4	2	1	0	0	4	3	7
ACC_07571	conserved hypothetical protein			143	43	68	110	42	10	17	12	74	40	22	19	1	3	9	1
ACC_07572	glucose transporter type 1-like	K07299	KOG0569	1247	727	724	760	332	93	104	142	928	443	341	467	65	70	79	33
ACC_07573	gamma-aminobutyric acid receptor subunit beta		KOG3643	431	143	135	436	195	12	23	22	77	29	54	6	1	3	14	7
ACC_07574	GE21259		KOG3643	477	290	349	608	171	28	34	40	109	90	36	9	9	20	49	22
ACC_07575	conserved hypothetical protein			517	258	329	630	342	36	82	89	80	55	54	6	5	7	59	18
ACC_07576	ammonium transporter Rh type B-like	K06580	KOG3796	407	109	112	292	143	24	39	39	339	66	135	248	72	75	32	26
ACC_07577	leucine-rich repeat-containing protein 58-like		KOG0444	131	60	58	172	107	16	24	20	334	811	147	660	408	140	57	25
ACC_07578	PI-PLC X domain-containing protein 1		KOG4306	2	4	2	9	2	1	4	0	58	8	83	36	1	4	4	8

ACC_07579	2-hydroxyacylsphingosine 1-beta-galactosyltransferase-like i		KOG1192	19	17	15	35	15	4	6	8	14	14	16	9	13	14	18	14
ACC_07580	conserved hypothetical protein			185	5	14	7	3	0	5	0	47	14	4	40	82	367	505	231
ACC_07581	LOW QUALITY PROTEIN	K06115	KOG0517	4223	1799	1889	3109	827	171	257	362	2869	2315	379	919	693	892	984	672
ACC_07582	conserved hypothetical protein			149	121	115	165	32	4	6	12	99	80	6	8	1	4	0	1
ACC_07583	conserved hypothetical protein		KOG4536	312	217	273	281	167	55	54	60	977	465	137	284	312	333	122	11
ACC_07584	conserved hypothetical protein			1904	1643	1032	875	656	775	1411	1538	3640	3493	840	600	792	592	1106	405
ACC_07585	putative glycerol kinase 5-like isoform 2		KOG2517	170	92	82	375	195	40	42	58	1000	526	171	669	228	204	59	12
ACC_07586	conserved hypothetical protein		KOG4556	164	110	126	90	36	9	5	14	203	234	32	68	127	88	51	10
ACC_07587	niemann-Pick C1 protein-like	K12385	KOG1933	16	11	16	32	16	1	5	4	15	38	15	14	727	935	92	17
ACC_07588	tryptophanyl-tRNA synthetase, mitochondrial-like	K01867	KOG2713	162	110	114	225	260	12	28	42	243	81	167	216	190	334	49	32
ACC_07589	mini-chromosome maintenance complex-binding protein isc		KOG2545	257	159	175	298	386	20	40	62	243	196	243	350	328	424	88	56
ACC_07590	conserved hypothetical protein			478	325	370	440	492	59	166	167	623	578	365	375	428	464	150	74
ACC_07591	conserved hypothetical protein			0	0	0	2	1	0	0	0	1	0	0	0	0	3	1	0
ACC_07592	conserved hypothetical protein			335	2	27	121	18	1	3	1	30	4	1	68	4868	1202	187	1112
ACC_07593	h2.0-like homeobox protein-like	K09339	KOG0488	12	7	7	2	2	4	7	9	0	1	2	0	1	0	6	2
ACC_07594	conserved hypothetical protein	K16462	KOG0161	882	769	778	658	188	168	156	232	1635	504	357	442	91	644	358	27
ACC_07595	neprilysin-2		KOG3624	7	2	4	34	10	19	21	12	633	265	3586	1332	4	3	3	4
ACC_07596	conserved hypothetical protein		KOG4389	6	1	2	5	0	0	1	2	38	28	2	1	4	7	21	11
ACC_07597	ras-related protein Rab-6A-like isoform 2	K07976	KOG0094	763	395	326	416	329	68	67	117	732	431	169	793	441	436	101	30
ACC_07598	conserved hypothetical protein		KOG0996	1564	924	962	1940	1494	253	454	412	4014	2810	821	1599	1648	1461	843	349
ACC_07599	bruchpilot	K16072		231	138	158	181	40	5	10	25	72	64	23	4	2	4	37	12
ACC_07600	conserved hypothetical protein			176	42	40	211	46	4	6	5	77	45	6	31	1	6	28	7
ACC_07601	conserved hypothetical protein			68	0	0	0	0	1	0	0	0	0	0	0	1	0	1	0
ACC_07602	conserved hypothetical protein			6	4	1	8	0	0	0	0	6	0	0	0	0	6	5	14
ACC_07603	lipopolysaccharide-induced tumor necrosis factor-alpha factor homolog			55	29	40	47	16	6	2	2	82	38	6	47	43	42	13	3
ACC_07604	conserved hypothetical protein	K06084	KOG1029	387	304	310	306	135	91	94	101	1086	453	808	1625	408	412	327	141
ACC_07605	leucine rich repeat G protein coupled receptor	K04307	KOG2087	7	2	4	6	1	1	1	0	3	1	20	6	1	1	1	2
ACC_07606	conserved hypothetical protein		KOG1074	43	26	30	37	4	4	3	3	41	44	10	8	6	39	94	57
ACC_07607	neurobeachin-like, partial		KOG1787	763	448	333	948	208	84	86	160	498	459	108	41	77	105	144	52
ACC_07608	conserved hypothetical protein	K11255	KOG3561	1596	1592	1701	1127	370	168	187	241	1576	1850	357	630	357	450	897	362
ACC_07609	ankyrin repeat domain-containing protein 39-like isoform 3			42	24	25	57	40	3	4	7	81	57	26	101	95	91	11	0
ACC_07610	conserved hypothetical protein			11	0	1	9	3	2	0	3	51	32	0	2	3643	2862	921	359
ACC_07611	aromatic-L-amino-acid decarboxylase	K01593	KOG0628	10	9	7	13	13	2	0	2	27	6	332	148	0	1	7	1
ACC_07612	mps one binder kinase activator-like 4-like isoform 2		KOG1852	180	67	45	65	67	5	4	9	187	133	46	233	186	195	20	7
ACC_07613	lymphokine-activated killer T-cell-originated protei	K08865	KOG0192	59	37	33	94	84	1	4	4	142	65	61	78	199	380	35	22
ACC_07614	guanylate cyclase 32E-like		KOG1023	11	0	1	5	1	1	3	1	24	0	0	0	21	90	78	33
ACC_07615	conserved hypothetical protein			156	119	114	148	152	15	24	32	85	90	79	184	331	305	102	89
ACC_07616	GTP-binding protein Rhes-like		KOG0395	99	34	28	56	14	0	2	1	16	10	0	0	0	4	8	0
ACC_07617	protein MTO1 homolog, mitochondrial-like isoform	K03495	KOG2311	588	432	474	705	560	53	74	94	602	333	309	263	641	684	66	32
ACC_07618	reverse transcriptase			2	0	0	0	0	0	0	0	1	6	1	1	0	0	3	0
ACC_07619	carbohydrate sulfotransferase 11-like		KOG4651	327	214	200	323	305	17	19	31	822	668	413	1754	71	107	42	4
ACC_07620	tyrosine-protein kinase Drl		KOG1024	8	4	3	3	0	1	1	0	2	4	0	6	0	2	1	2
ACC_07621	putative ATP-dependent RNA helicase Pl10	K11594	KOG0335	225	130	116	84	81	29	46	54	653	429	49	168	270	323	251	75
ACC_07622	methionine-R-sulfoxide reductase B1 isoform 1	K07305	KOG0856	297	129	133	515	296	25	20	33	355	276	109	335	1407	833	209	77
ACC_07623	yellow-like protein			33	10	6	39	11	5	7	14	23	6	27	3	2	0	8	4
ACC_07624	phosphoenolpyruvate carboxykinase	K01596	KOG3749	1	1	2	8	3	1	1	0	456	17	17	4	6	3	0	0
ACC_07625	methyltransferase-like protein 22-like		KOG2497	102	72	56	61	119	0	1	9	72	107	120	507	140	143	9	2
ACC_07626	abaecin			1	9	48	70	55	35	28	9	2441	1004	3	204	3	1	24	0
ACC_07627	conserved hypothetical protein			579	370	317	729	709	87	205	292	323	258	64	89	80	28	98	103
ACC_07628	cystathionine-beta-synthase	K01697	KOG1252	70	58	49	103	97	6	10	14	149	88	41	147	237	245	14	14
ACC_07629	protein angel-like		KOG2338	393	278	310	487	422	28	33	37	1000	799	316	1253	712	539	35	7
ACC_07630	lysosome-associated membrane glycoprotein 1-like	K06528	KOG4818	2273	1401	1405	3210	3158	222	231	338	4097	2283	1984	3723	2838	2585	92	54
ACC_07631	ATP synthase subunit epsilon, mitochondrial-like is	K02135	KOG3495	242	169	182	285	271	15	25	23	100	49	170	456	302	321	19	11
ACC_07632	icarapin precursor			2419	3426	3482	5809	2427	707	518	914	8284	6643	1608	6343	453	215	112	54
ACC_07633	conserved hypothetical protein	K11795	KOG1310	696	411	390	601	401	163	225	302	1832	1091	251	358	457	369	193	95
ACC_07634	microtubule-associated protein Jupiter-like			391	166	210	304	143	26	20	31	373	601	114	541	281	184	203	30
ACC_07635	leucine-rich repeat-containing protein 24		KOG4194	291	99	91	268	70	6	18	8	102	60	33	19	0	0	15	7

ACC_07636	FK506-binding protein 59	K09571	KOG0543	1514	544	878	1534	1295	92	148	273	1571	588	521	2004	4063	7125	1090	881
ACC_07637	synaptosomal-associated protein 29	K08509	KOG3065	149	69	73	225	221	33	51	104	146	121	131	202	175	100	43	44
ACC_07638	conserved hypothetical protein		KOG0859	0	3	0	1	1	0	2	1	1	10	0	0	15	4	1	0
ACC_07639	leucine-rich repeat protein soc-2 homolog		KOG0444	554	394	404	338	194	49	85	144	573	652	40	351	295	127	94	66
ACC_07640	zinc metalloproteinase nas-15-like isoform 2		KOG3714	23	1	3	13	5	1	2	7	13	53	0	12	241	38	11	5
ACC_07641	Lipase member H-A			148	20	44	51	10	1	6	10	114	442	65	157	44	54	235	6
ACC_07642	conserved hypothetical protein		KOG4441	104	84	58	93	71	4	7	8	119	62	38	74	87	92	32	7
ACC_07643	endoplasmic reticulum aminopeptidase 2-like	K11141	KOG1046	902	515	509	1895	497	63	97	153	1797	653	1088	928	57	64	30	17
ACC_07644	trace amine-associated receptor 8b-like		KOG4219	240	123	122	130	55	3	4	6	171	127	26	38	0	6	9	2
ACC_07645	conserved hypothetical protein			303	218	192	425	401	17	29	37	377	253	224	624	226	224	21	5
ACC_07646	set1/Ash2 histone methyltransferase complex subu	K14964	KOG2626	199	146	123	206	215	23	31	52	144	103	101	115	212	294	126	44
ACC_07647	conserved hypothetical protein		KOG4384	66	69	52	68	10	1	1	2	54	43	10	4	10	19	7	8
ACC_07648	sterile alpha motif domain-containing protein 5-like			7	10	7	14	4	1	0	1	10	12	1	4	5	8	3	2
ACC_07649	sterile alpha motif domain-containing protein 5-like		KOG4384	8	6	7	9	1	0	0	2	9	7	1	3	4	1	4	2
ACC_07650	conserved hypothetical protein		KOG0260	6	1	2	4	0	0	0	0	61	2	0	0	28456	18892	3572	4028
ACC_07651	glycerate kinase-like			6	10	2	7	4	2	2	3	10	2	5	2	0	0	4	4
ACC_07652	similar to Ras-related protein Rac1	K04392	KOG0393	468	277	220	433	332	36	55	66	741	631	253	882	799	707	98	21
ACC_07653	pro-resilin			29	26	45	167	89	5	6	12	15	13	94	177	1	8	22	4
ACC_07654	conserved hypothetical protein		KOG0921	76	47	51	109	31	9	7	5	344	252	101	51	7	5	5	2
ACC_07655	LOW QUALITY PROTEIN		KOG0548	105	84	65	164	150	6	9	13	106	126	76	121	281	258	47	21
ACC_07656	conserved hypothetical protein			6	4	11	42	10	0	3	4	6	13	3	2	0	0	7	0
ACC_07657	fibrillin-1		KOG1214	2	3	2	15	3	0	0	3	8	7	0	2	4	0	2	1
ACC_07658	zinc finger RNA-binding protein	K13203	KOG3792	1211	966	928	1061	473	129	263	278	1807	1628	497	688	1823	3132	2967	1430
ACC_07659	DDB1- and CUL4-associated factor 12-like	K11803	KOG0266	56	19	20	47	67	6	7	7	142	106	15	83	140	50	5	1
ACC_07660	tubulin-specific chaperone A-like		KOG3470	296	193	149	191	203	48	79	82	235	161	157	311	371	400	300	224
ACC_07661	conserved hypothetical protein			1	0	1	10	1	1	2	0	2	0	0	0	214	225	166	77
ACC_07662	UNC93-like protein-like		KOG3097	44	21	19	59	30	2	12	10	296	80	113	510	620	84	11	40
ACC_07663	conserved hypothetical protein	K15177	KOG2428	840	609	559	1283	1010	162	215	294	1168	837	507	955	1904	2817	620	547
ACC_07664	methionine sulfoxide reductase A	K07304	KOG1635	218	135	156	545	832	7	8	21	361	232	96	257	623	825	60	12
ACC_07665	conserved hypothetical protein		KOG2462	284	204	207	255	108	17	31	48	330	424	49	96	354	346	122	32
ACC_07666	long-chain fatty acid transport protein 4-like			104	25	49	139	83	19	23	24	65	76	21	29	15	17	39	21
ACC_07667	conserved hypothetical protein	K07847	KOG0395	154	96	77	108	33	4	11	7	83	58	4	7	1	4	28	20
ACC_07668	leucine-rich repeat-containing protein 15-like		KOG4194	1379	514	601	2821	2072	172	281	319	865	377	138	144	46	102	163	54
ACC_07669	TBC1 domain family member 25 isoform 1		KOG2197	314	258	191	363	254	49	48	54	317	219	84	139	218	152	33	11
ACC_07670	MOXD1 homolog 2-like		KOG3568	59	19	12	25	5	0	0	3	34	23	4	6	2	8	11	5
ACC_07671	putative ATP-dependent RNA helicase DHX33-like		KOG0922	73	64	67	286	70	20	17	27	53	99	30	55	40	79	172	72
ACC_07672	LOW QUALITY PROTEIN		KOG0522	345	286	286	451	347	50	52	58	325	169	181	294	318	403	106	26
ACC_07673	phosphorylated adapter RNA export protein	K14291	KOG3948	248	161	147	232	284	46	85	120	369	207	183	206	241	297	209	139
ACC_07674	mitotic checkpoint protein BUB3	K02180	KOG1036	154	92	85	99	97	13	21	29	114	71	67	150	208	251	46	41
ACC_07675	clathrin interactor 1		KOG2057	384	240	177	260	234	116	302	292	374	471	258	406	547	374	460	438
ACC_07676	von Hippel-Lindau disease tumor suppressor	K03871	KOG4710	44	24	13	35	3	1	2	2	17	47	9	16	10	8	30	14
ACC_07677	abhydrolase domain-containing protein 3 isoform 2	K13696	KOG1838	1100	528	769	1627	1307	24	33	47	1329	605	94	942	2232	1134	135	11
ACC_07678	transcription initiation factor TFIID subunit 2, partia	K03128	KOG1932	752	443	367	694	602	137	297	280	786	378	374	228	436	554	634	340
ACC_07679	conserved hypothetical protein	K09534	KOG0720	429	276	245	419	393	69	179	218	577	277	258	128	298	362	376	181
ACC_07680	hemicentin-1-like		KOG3515	3	2	3	8	0	0	0	0	2	2	0	0	1	0	1	0
ACC_07681	nephrin-like		KOG3515	31	30	31	73	16	6	8	12	141	241	10	11	30	46	198	95
ACC_07682	DNA polymerase alpha subunit B	K02321	KOG1625	32	25	25	38	27	5	9	10	62	33	32	67	135	216	28	9
ACC_07683	dipeptidyl peptidase 3-like isoform 1	K01277	KOG3675	311	230	177	296	239	46	57	66	349	441	121	406	1246	1549	114	53
ACC_07684	myosin-IA	K10356	KOG0164	369	210	170	209	230	37	75	77	871	538	169	724	869	671	58	33
ACC_07685	conserved hypothetical protein		KOG4171	970	610	679	1842	825	91	80	126	400	457	11	12	103	209	129	131
ACC_07686	f-box only protein 7-like	K10293	KOG0274	60	33	37	74	84	9	5	9	129	63	65	221	172	160	10	8
ACC_07687	sentrin-specific protease 1-like	K08592	KOG0778	261	128	157	308	345	9	35	45	281	243	125	301	281	397	158	46
ACC_07688	katanin p60 ATPase-containing subunit A-like 1-like	K07767	KOG0738	144	71	68	281	257	23	58	53	194	197	202	240	218	211	208	144
ACC_07689	conserved hypothetical protein			35	20	26	27	36	2	4	3	50	31	12	2	85	40	4	0
ACC_07690	protein alan shepard-like		KOG0145	1024	809	749	1050	406	127	167	187	1336	1296	311	457	836	1147	1055	379
ACC_07691	active breakpoint cluster region-related protein-like		KOG4269	182	147	112	219	128	21	50	52	344	213	90	143	166	185	117	38
ACC_07692	androgen-induced gene 1 protein-like isoform 1		KOG3989	213	198	121	210	264	30	46	61	815	869	717	4087	1115	615	95	18

ACC_07693	conserved hypothetical protein			2	0	1	3	1	0	0	1	2	3	0	0	1	0	3	0
ACC_07694	solute carrier family 35 member E2-like	K15284	KOG1441	308	229	193	263	256	22	46	38	303	297	166	487	745	819	161	40
ACC_07695	LOW QUALITY PROTEIN		KOG1187	307	172	150	455	588	24	77	106	294	228	179	354	303	279	118	60
ACC_07696	acetyl-CoA carboxylase-like isoform 2	K11262	KOG0368	974	590	284	385	273	146	182	199	5761	6740	325	694	5858	3877	500	203
ACC_07697	brain-specific homeobox protein homolog		KOG0491	47	37	57	188	31	1	4	13	22	9	3	1	3	5	3	1
ACC_07698	slit homolog 3 protein-like		KOG4194	1	0	0	1	1	0	0	0	3	2	0	1	489	603	179	15
ACC_07699	putative fatty acyl-CoA reductase CG5065-like		KOG1221	22	20	11	33	32	8	7	5	195	67	659	1717	632	1422	76	81
ACC_07700	putative fatty acyl-CoA reductase CG5065-like	K13356	KOG1221	14	23	9	21	13	2	12	14	36	74	9	188	436	137	106	48
ACC_07701	n-acetylglucosamine-1-phosphotransferase subunits alpha/b		KOG1221	71	59	49	89	65	6	6	13	102	96	720	982	211	219	18	10
ACC_07702	sorbitol dehydrogenase-like		KOG0024	234	140	160	604	273	78	87	113	2268	4124	254	97	6181	2393	467	566
ACC_07703	conserved hypothetical protein	K11320	KOG0391	1868	1646	1328	992	625	306	525	618	2198	2143	353	412	3495	4568	2404	1102
ACC_07704	conserved hypothetical protein			506	432	437	875	556	173	273	328	1536	859	274	345	165	165	103	45
ACC_07705	kelch domain-containing protein 10-like isoform 2		KOG0379	90	30	27	44	36	8	8	13	263	165	36	139	216	188	7	5
ACC_07706	conserved hypothetical protein	K11716	KOG3932	1358	592	744	1427	201	34	34	76	487	406	35	35	1	10	34	10
ACC_07707	neurotrimin		KOG3513	932	219	232	344	196	15	34	35	1162	598	30	195	438	615	178	59
ACC_07708	conserved hypothetical protein			163	52	54	94	37	4	3	6	61	73	13	7	9	8	9	16
ACC_07709	conserved hypothetical protein			148	54	74	114	64	8	16	25	67	43	20	12	1	3	16	12
ACC_07710	vacuolar ATPase assembly integral membrane protein VMA2		KOG4783	71	48	85	125	102	11	15	18	82	54	64	137	136	151	50	19
ACC_07711	conserved hypothetical protein	K09442	KOG3805	16	15	7	27	6	4	16	21	60	33	10	21	32	12	74	40
ACC_07712	lachesin-like		KOG3513	32	8	12	9	1	0	1	1	11	22	3	1	1	13	122	6
ACC_07713	protein hunchback	K09213	KOG3608	1	4	2	0	1	1	0	3	5	2	3	0	2	2	3	9
ACC_07714	yorkie homolog	K16687		332	181	201	509	198	98	238	243	439	414	281	223	136	232	1711	1365
ACC_07715	peroxisomal 3,2-trans-enoyl-CoA isomerase-like	K13239	KOG0016	76	47	35	66	98	5	9	8	122	63	76	247	149	178	8	6
ACC_07716	LOW QUALITY PROTEIN	K12172	KOG0864	2222	1836	1695	1752	1220	230	504	541	2866	2503	825	637	1776	1972	1032	459
ACC_07717	LOW QUALITY PROTEIN	K07178	KOG2270	173	109	115	183	223	34	59	58	446	343	276	580	386	499	72	30
ACC_07718	POU domain protein CF1A-like	K09365	KOG3802	136	74	100	96	12	58	62	132	189	319	22	43	31	32	227	235
ACC_07719	conserved hypothetical protein		KOG3598	979	280	290	809	179	14	23	48	249	135	78	22	1	10	26	17
ACC_07720	e3 ubiquitin-protein ligase RNF13-like	K15692	KOG4628	151	83	67	79	68	11	7	15	528	565	80	248	219	151	4	0
ACC_07721	pheromone-binding protein-related protein 3-like			3383	579	598	2812	1148	72	123	153	388	56	58	143	16	59	91	51
ACC_07722	transcription factor GATA-5	K09183	KOG1601	33	42	27	29	3	14	12	22	39	12	8	0	2	4	31	4
ACC_07723	conserved hypothetical protein		KOG1601	5	2	0	0	0	2	2	0	20	12	1	0	310	469	365	47
ACC_07724	conserved hypothetical protein		KOG0921	165	128	100	39	16	41	33	56	205	525	6	10	197	96	366	241
ACC_07725	conserved hypothetical protein			182	112	202	362	220	12	20	33	172	33	14	8	2	0	2	3
ACC_07726	nimrod A		KOG3690	87	38	45	61	17	8	8	11	205	106	60	192	15	12	17	12
ACC_07727	acid sphingomyelinase-like phosphodiesterase 3a-like		KOG3770	383	255	305	1197	225	25	40	38	112	72	87	36	2	8	43	19
ACC_07728	vacuole membrane protein 1-like		KOG1109	420	210	174	203	180	60	83	81	928	707	296	577	1592	1491	99	23
ACC_07729	leptin receptor overlapping transcript-like 1-like		KOG2174	121	89	82	258	171	8	16	29	217	113	205	585	205	172	15	7
ACC_07730	nephrin-like		KOG3515	262	129	109	257	119	15	12	30	64	47	30	40	5	21	36	19
ACC_07731	conserved hypothetical protein		KOG2643	817	529	563	1365	1309	176	327	363	1675	515	603	681	1406	1521	398	236
ACC_07732	eukaryotic translation initiation factor 2-alpha kinase 4		KOG1035	36	27	24	38	30	8	18	17	47	36	20	60	39	36	26	7
ACC_07733	glycosaminoglycan xylosylkinase-like		KOG3829	267	121	126	301	235	13	38	73	154	137	127	339	129	163	83	37
ACC_07734	ovarian-specific serine/threonine-protein kinase Lo	K06641	KOG0615	138	112	109	162	190	12	10	19	110	92	45	105	153	170	15	6
ACC_07735	TWiK family of potassium channels protein 7-like		KOG1418	21	5	9	6	1	2	2	1	12	19	1	1	0	0	1	1
ACC_07736	cuticular protein analogous to peritrophins 3-B precursor			281	103	65	209	43	13	11	18	143	33	40	306	579	283	272	654
ACC_07737	sodium/potassium-transporting ATPase subunit alpha-like		KOG0203	4	2	1	6	1	3	1	1	5	2	2	2	13	2	3	4
ACC_07738	aquaporin AQPAn.G-like	K09884	KOG0223	84	28	40	180	98	11	5	10	972	484	55	654	55	53	11	6
ACC_07739	inositol-trisphosphate 3-kinase A-like	K00911	KOG1621	72	47	81	144	85	24	26	24	253	128	37	56	47	148	21	6
ACC_07740	bifunctional heparan sulfate N-deacetylase/N-sulfotransferase-like			77	42	27	50	11	11	18	27	26	97	27	35	7	6	11	9
ACC_07741	conserved hypothetical protein			82	47	31	77	43	7	8	21	95	65	48	166	126	222	42	10
ACC_07742	enhancer of rudimentary homolog		KOG1766	303	206	237	617	424	31	66	74	369	242	205	794	522	690	177	103
ACC_07743	synaptotagmin-4-like		KOG1028	625	373	486	299	82	11	5	15	452	406	83	142	8	7	3	0
ACC_07744	lipase 1-like		KOG2624	63	53	44	73	56	2	9	8	100	141	13	55	42	32	5	2
ACC_07745	electron transfer flavoprotein subunit beta-like	K03521	KOG3180	267	224	132	356	274	39	50	66	792	496	214	787	3185	2819	359	236
ACC_07746	heat shock protein 67B2-like		KOG1530	13	12	12	11	25	0	4	5	8	7	16	15	14	27	4	1
ACC_07747	glucose dehydrogenase		KOG1238	7	6	11	15	6	2	2	1	15	14	4	0	2	5	11	16
ACC_07748	CUGBP Elav-like family member 4-like		KOG0146	497	496	566	1089	337	77	141	202	212	95	121	23	1	12	115	40
ACC_07749	venom serine carboxypeptidase precursor	K09645	KOG1282	319	230	237	446	535	47	67	96	2593	1969	576	666	1055	1033	30	10

ACC_07750	protein PIEZO2-like		KOG1893	1193	1024	880	979	567	600	928	847	2701	1613	215	147	1020	1263	653	387
ACC_07751	conserved hypothetical protein		KOG2650	2020	1407	1643	2823	693	780	791	1126	3373	2656	1580	1312	32020	15844	4158	1137
ACC_07752	conserved hypothetical protein		KOG3623	83	72	128	94	26	10	19	22	97	82	18	9	8	13	288	333
ACC_07753	conserved hypothetical protein	K10630	KOG1814	43	22	31	72	33	6	12	15	882	360	29	6	35	60	53	50
ACC_07754	aldose 1-epimerase-like	K01785	KOG1604	5	0	0	5	1	1	0	1	4	3	0	0	0	1	3	1
ACC_07755	probable O-sialoglycoprotein endopeptidase 2-like	K01409	KOG2707	211	159	146	280	332	11	40	47	220	112	126	181	389	485	67	34
ACC_07756	60 kDa heat shock protein, mitochondrial-like	K04077	KOG0356	1570	747	731	2188	1547	201	327	463	2118	3535	623	1458	30323	46651	13016	7251
ACC_07757	triosephosphate isomerase	K01803	KOG1643	1228	887	881	1753	1783	143	204	283	1883	688	591	1466	4196	2259	269	182
ACC_07758	kinesin 3B isoform 1		KOG0245	131	84	92	82	37	5	3	7	115	115	13	38	4	1	1	0
ACC_07759	scavenger receptor class B member 1, partial		KOG3776	354	251	188	551	155	44	70	85	1158	2294	1874	3194	572	231	384	130
ACC_07760	conserved hypothetical protein		KOG2462	73	45	73	275	106	31	26	49	40	127	4	2	13	4	8	9
ACC_07761	conserved oligomeric Golgi complex subunit 3		KOG2604	190	193	140	145	136	14	18	22	355	337	113	348	277	245	21	4
ACC_07762	conserved hypothetical protein		KOG4441	99	74	85	152	45	13	18	26	107	116	23	9	15	29	294	346
ACC_07763	actin-binding Rho-activating protein-like isoform 2		KOG3376	12	3	5	71	29	11	11	9	1838	278	92	142	56	116	44	20
ACC_07764	myelin gene regulatory factor-like		KOG3661	200	185	230	188	99	23	37	39	793	848	195	549	158	158	69	25
ACC_07765	conserved hypothetical protein		KOG4701	17	15	20	32	12	3	2	9	195	13	8	4	41964	30426	22720	1065
ACC_07766	u2 small nuclear ribonucleoprotein auxiliary factor 35 kDa su		KOG2202	104	106	96	196	123	27	48	54	127	52	43	73	137	203	169	216
ACC_07767	nucleobindin-2-like		KOG3866	2372	2026	1710	2130	1557	541	902	1008	1720	1589	797	1451	2985	2605	1140	806
ACC_07768	conserved hypothetical protein		KOG1644	1462	1190	1498	3895	1657	102	120	225	1162	711	119	263	1866	1437	291	53
ACC_07769	mitoferrin-1 isoform 2	K15113	KOG0760	70	41	31	72	49	5	6	4	320	220	88	823	251	241	15	1
ACC_07770	conserved hypothetical protein		KOG1874	1717	1355	1165	794	1072	277	698	725	1148	959	819	487	995	1284	1181	580
ACC_07771	conserved hypothetical protein			6	0	1	4	3	1	3	1	4	0	0	0	1272	622	1463	13042
ACC_07772	conserved hypothetical protein		KOG1074	45	20	22	44	10	4	7	0	41	52	12	9	13	23	106	84
ACC_07773	glycoprotein-N-acetylgalactosamine 3-beta-galacto	K00731	KOG2246	209	179	164	266	270	28	34	46	333	174	107	219	778	783	111	14
ACC_07774	protein CIP2A-like		KOG0161	400	299	298	324	352	15	36	29	176	115	155	51	211	317	124	51
ACC_07775	conserved hypothetical protein		KOG0811	87	72	58	39	36	3	5	6	136	97	19	94	65	66	11	2
ACC_07776	mitochondrial import receptor subunit TOM70		KOG0547	229	153	107	307	310	9	23	27	494	407	136	337	1061	1452	146	90
ACC_07777	probable cytochrome P450 301a1, mitochondrial		KOG0159	6	3	6	4	5	1	0	1	6	5	5	41	20	52	5	10
ACC_07778	conserved hypothetical protein			485	426	549	896	711	101	186	138	2818	1945	920	1236	1082	611	83	80
ACC_07779	gamma-tubulin complex component 2			12	10	5	20	29	0	2	1	9	5	10	12	21	13	1	2
ACC_07780	katanin p60 ATPase-containing subunit A-like 2-like			0	4	6	3	10	0	3	1	1	3	11	13	9	7	0	3
ACC_07781	nitrilase homolog 1-like	K13566	KOG0806	109	100	66	140	117	25	33	27	402	229	142	398	719	446	31	17
ACC_07782	conserved hypothetical protein		KOG4364	295	199	191	206	270	52	130	146	149	94	96	54	226	452	1195	916
ACC_07783	mps one binder kinase activator-like 2-like		KOG0440	319	159	179	667	211	53	82	89	736	388	373	510	187	155	174	41
ACC_07784	conserved hypothetical protein			392	293	314	287	261	60	144	156	353	123	217	319	524	699	793	758
ACC_07785	A disintegrin and metalloproteinase with thrombospondin m		KOG3538	27	7	9	27	19	6	7	15	13	13	6	5	7	8	26	9
ACC_07786	28S ribosomal protein S30, mitochondrial		KOG4461	552	327	300	701	612	46	182	164	751	581	346	772	1543	2224	444	237
ACC_07787	ADAMTS-like protein 3-like		KOG3538	3	0	5	4	4	0	0	0	6	2	0	0	7	5	2	0
ACC_07788	steroid receptor seven-up, isoforms B/C-like		KOG4215	82	39	41	117	10	4	5	7	170	365	48	32	64	77	373	172
ACC_07789	conserved hypothetical protein	K14032	KOG4215	101	38	36	65	12	4	3	4	161	472	39	40	88	77	299	105
ACC_07790	dual serine/threonine and tyrosine protein kinase	K16288	KOG0192	232	102	103	169	221	19	21	23	515	324	89	339	212	179	17	2
ACC_07791	conserved hypothetical protein			62	45	13	40	28	39	43	44	245	175	113	303	31	6	11	12
ACC_07792	atrial natriuretic peptide receptor 1-like		KOG1023	362	126	191	410	128	15	32	31	112	50	7	6	26	51	92	5
ACC_07793	conserved hypothetical protein			70	41	87	263	106	0	1	1	167	25	11	6	7	12	9	7
ACC_07794	DE-cadherin-like		KOG4289	216	129	106	138	51	44	30	54	877	581	145	193	168	299	239	91
ACC_07795	conserved hypothetical protein		KOG2651	101	106	93	137	210	7	22	20	188	93	199	286	140	147	17	10
ACC_07796	mitochondrial import inner membrane translocase subunit T		KOG3442	180	173	237	358	221	40	55	102	249	83	167	234	237	393	114	65
ACC_07797	dopamine receptor 1-like	K04148	KOG4220	338	230	265	296	143	27	24	34	124	95	8	1	2	3	7	8
ACC_07798	conserved hypothetical protein		KOG3598	80	79	74	43	14	9	16	12	84	151	24	20	512	1157	394	288
ACC_07799	conserved hypothetical protein			500	295	283	458	212	115	165	185	687	524	185	403	962	1245	1729	1019
ACC_07800	wolframin	K14020		1052	376	325	677	620	71	150	139	779	318	140	295	245	216	72	18
ACC_07801	conserved hypothetical protein		KOG2462	309	271	192	318	172	37	98	108	192	143	168	122	154	155	160	71
ACC_07802	b9 domain-containing protein 1-like	K16744	KOG4027	0	0	2	0	0	0	0	2	7	3	26	21	4	9	4	5
ACC_07803	RNA-binding protein 47-like		KOG0117	2	0	0	2	1	0	2	1	3	3	2	2	19	9	10	6
ACC_07804	conserved hypothetical protein		KOG1883	78	131	136	77	24	14	16	22	192	343	17	49	42	40	92	26
ACC_07805	conserved hypothetical protein		KOG0200	106	57	77	103	31	14	11	15	400	452	277	214	12	19	22	21
ACC_07806	conserved hypothetical protein			98	48	46	65	16	5	4	8	224	210	41	22	21	49	29	5

ACC_07807	neurexin 1 precursor		KOG3514	18	6	7	20	1	1	0	0	5	9	5	4	0	0	0	0
ACC_07808	coiled-coil domain-containing protein 47-like		KOG2357	591	327	292	368	335	71	116	107	610	449	136	706	1244	1001	135	81
ACC_07809	conserved hypothetical protein		KOG0670	408	287	307	488	230	76	94	162	552	402	162	125	238	305	496	224
ACC_07810	potassium voltage-gated channel protein Shaw		KOG3713	11	0	6	9	1	2	1	2	8	12	1	2	0	1	2	3
ACC_07811	potassium voltage-gated channel protein Shaw-like		KOG3713	7	2	3	5	2	1	1	0	7	17	4	9	95	93	71	92
ACC_07812	probable cytochrome P450 6a14 isoform 1		KOG0158	87	28	4	161	211	442	413	491	267	588	0	0	1	0	0	0
ACC_07813	microsomal triglyceride transfer protein large subu	K16804	KOG3971	364	225	209	249	308	21	93	85	161	144	159	100	302	322	312	167
ACC_07814	conserved hypothetical protein	K13504	KOG0860	3615	2179	2767	4496	1787	305	554	826	940	621	644	371	1	27	307	204
ACC_07815	proteasome subunit beta type-3-like	K02735	KOG0180	172	156	134	284	293	16	32	36	383	220	492	983	1162	1312	90	62
ACC_07816	conserved hypothetical protein		KOG2408	12	3	6	7	2	0	0	0	4	14	0	2	12	108	69	22
ACC_07817	conserved hypothetical protein			2	1	1	8	1	0	0	0	7	2	2	0	1	0	2	0
ACC_07818	conserved hypothetical protein			33	22	34	53	9	2	3	1	16	23	32	12	2	1	4	2
ACC_07819	vacuolar protein sorting-associated protein 11 homolog		KOG2114	409	303	324	353	281	36	45	36	518	350	120	273	379	279	23	7
ACC_07820	zinc finger protein squeeze isoform 2		KOG2462	258	172	189	293	74	40	82	67	408	330	257	132	105	117	1416	1010
ACC_07821	probable leucyl-tRNA synthetase, mitochondrial-lik	K01869	KOG0435	33	17	18	24	32	2	6	2	35	30	20	46	85	85	9	6
ACC_07822	HEAT repeat-containing protein 3-like			249	165	158	234	269	26	54	64	243	225	111	179	611	603	65	40
ACC_07823	fanconi-associated nuclease 1-like	K15363	KOG2143	391	263	215	260	340	28	51	68	379	422	301	380	567	463	141	64
ACC_07824	translin		KOG3067	87	76	75	139	180	10	13	22	264	126	153	334	327	337	17	11
ACC_07825	synaptic vesicle glycoprotein 2B-like	K06258	KOG0253	281	143	130	166	137	26	24	30	383	277	93	99	198	146	36	18
ACC_07826	beta,beta-carotene 9',10'-oxygenase isoform 1		KOG1285	5	34	25	171	150	99	113	147	1091	1115	16	64	7	2	0	1
ACC_07827	conserved hypothetical protein		KOG0484	32	18	26	38	24	11	10	15	168	91	18	9	23	12	8	5
ACC_07828	retinal rod rhodopsin-sensitive cGMP 3',5'-cyclic pł	K13758	KOG4038	50	32	38	99	94	3	13	16	75	25	92	144	106	126	21	4
ACC_07829	conserved hypothetical protein			111	39	30	81	21	17	21	31	373	320	211	434	150	149	374	321
ACC_07830	glycylpeptide N-tetradecanoyltransferase 1	K00671	KOG2779	933	411	407	1106	626	130	193	242	1154	1116	335	1458	1638	1484	554	410
ACC_07831	conserved hypothetical protein		KOG4701	40	22	33	37	8	4	5	5	17	15	7	6	81	275	241	112
ACC_07832	alkaline phosphatase, tissue-nonspecific isozyme-li	K01077	KOG4126	273	153	164	465	299	36	74	80	630	301	545	834	526	552	125	51
ACC_07833	conserved hypothetical protein			454	216	251	474	327	32	52	52	206	79	126	20	2	7	19	2
ACC_07834	glucose dehydrogenase		KOG1238	21	8	10	22	5	2	4	5	5	5	8	2	4	11	52	11
ACC_07835	conserved hypothetical protein			12	5	13	9	5	3	0	2	27	43	4	11	13	14	27	13
ACC_07836	conserved hypothetical protein			5	6	2	2	2	0	0	0	14	5	20	63	5	53	6	5
ACC_07837	mediator of RNA polymerase II transcription subun	K15153	KOG4086	38	25	32	112	123	2	8	3	92	45	135	185	150	123	21	4
ACC_07838	teneurin-3 isoform 1		KOG4659	765	410	353	713	247	58	76	118	634	747	122	108	102	155	435	180
ACC_07839	conserved hypothetical protein			284	115	128	205	117	9	28	27	77	57	11	8	2	5	27	3
ACC_07840	membralin-like			63	55	35	47	22	13	16	18	68	88	21	23	25	17	29	6
ACC_07841	sugar transporter ERD6-like 18-like		KOG0254	16	9	13	51	32	5	6	9	72	53	13	1	19	11	9	1
ACC_07842	conserved hypothetical protein			107	78	115	147	47	26	42	46	246	327	17	41	3	1	22	12
ACC_07843	GPI ethanolamine phosphate transferase 2-like	K05310	KOG2125	310	197	221	218	310	18	25	26	186	159	122	192	238	206	12	6
ACC_07844	sodium-dependent transporter CG3252	K05038	KOG3660	24	6	6	27	9	11	7	7	369	195	265	759	4	4	3	3
ACC_07845	conserved hypothetical protein			383	220	186	288	401	21	55	43	283	328	435	620	337	125	76	48
ACC_07846	MFS-type transporter C6orf192 homolog		KOG3764	202	180	140	80	20	98	153	221	65	1223	12	9	991	702	2010	917
ACC_07847	protein takeout-like			468	521	246	153	90	472	442	657	47	105	196	213	324	72	47	57
ACC_07848	circadian clock-controlled protein-like			23	5	1	2	1	2	3	3	171	26	1235	2560	176	2	1	1
ACC_07849	circadian clock-controlled protein-like			0	0	0	1	0	0	1	1	2	1	4	4	36	437	125	27
ACC_07850	homeobox protein Nkx-2.5-like	K08029	KOG0842	0	0	0	1	0	0	0	0	0	1	0	2	18	52	61	2
ACC_07851	rhythmically expressed gene 2 protein-like		KOG3085	51	39	31	74	66	11	11	13	133	66	63	60	43	60	7	5
ACC_07852	conserved hypothetical protein			154	108	53	227	138	35	117	114	101	162	73	58	129	95	258	174
ACC_07853	conserved hypothetical protein		KOG1700	1226	824	800	945	439	175	299	309	5559	2614	719	886	685	1066	626	271
ACC_07854	conserved hypothetical protein		KOG1700	131	67	69	70	15	16	24	20	805	641	66	118	59	89	108	32
ACC_07855	peptidyl-prolyl cis-trans isomerase FKBP8-like isofo	K09574	KOG0543	423	260	191	609	449	103	222	241	651	561	676	1255	1015	1278	715	461
ACC_07856	glycoprotein 3-alpha-L-fucosyltransferase A		KOG2619	10	4	2	8	8	0	0	1	33	16	22	43	7	27	6	1
ACC_07857	conserved hypothetical protein	K10581	KOG0895	1042	665	507	1149	757	208	331	434	1085	678	389	393	314	205	284	158
ACC_07858	sodium-independent sulfate anion transporter-like	K14708	KOG0236	42	32	58	86	40	20	30	22	251	139	268	268	191	158	151	74
ACC_07859	conserved hypothetical protein			2	1	1	1	1	0	0	0	1	3	0	0	1	5	5	1
ACC_07860	conserved hypothetical protein			247	203	236	288	208	21	49	44	298	138	108	148	208	214	117	25
ACC_07861	hemicentin-1-like		KOG3513	221	113	116	128	31	5	7	16	81	57	5	6	0	3	5	4
ACC_07862	conserved hypothetical protein		KOG1052	333	232	168	328	292	48	141	135	681	689	939	719	175	47	19	31
ACC_07863	FACT complex subunit spt16		KOG1189	742	442	368	525	468	157	296	337	793	669	344	331	1156	1733	1754	895

ACC_07864	conserved hypothetical protein		KOG1192	163	72	79	114	81	6	6	10	129	234	16	81	407	312	37	10
ACC_07865	neuroligin-1		KOG4389	973	625	847	1414	293	43	57	104	184	332	17	5	1	5	67	47
ACC_07866	LOW QUALITY PROTEIN		KOG3328	80	117	105	176	136	3	13	15	60	35	92	172	106	132	18	6
ACC_07867	ejaculatory bulb-specific protein 3-like			9	7	11	26	15	0	1	2	16	17	123	216	155	214	7	28
ACC_07868	tetratricopeptide repeat protein 25-like		KOG0548	308	167	166	323	297	141	257	308	261	173	227	190	21	13	54	22
ACC_07869	clathrin interactor 1		KOG2057	302	185	131	303	130	96	247	287	290	357	230	298	406	273	393	400
ACC_07870	conserved hypothetical protein		KOG2200	252	187	208	311	270	18	47	37	265	283	92	294	322	341	57	12
ACC_07871	T-cell immunomodulatory protein precursor		KOG4550	1062	616	795	2174	959	114	81	135	1555	903	731	1191	521	466	127	54
ACC_07872	conserved hypothetical protein		KOG4139	1342	912	876	1214	951	259	552	548	1305	924	557	330	548	784	1010	384
ACC_07873	carbohydrate sulfotransferase 9-like		KOG4651	525	382	441	682	708	29	43	49	265	125	148	53	121	41	2	1
ACC_07874	niemann-Pick C1 protein-like isoform 2	K12385	KOG1933	865	590	465	355	329	82	105	153	2955	3292	1012	2208	1413	839	85	37
ACC_07875	Full=Prohormone-1; Contains			191	68	91	444	112	9	4	17	30	13	7	11	0	0	2	0
ACC_07876	conserved hypothetical protein			1278	1043	955	840	774	89	114	123	1304	807	514	252	466	429	66	12
ACC_07877	probable multidrug resistance-associated protein lethal(2)0		KOG0054	394	233	146	387	302	88	141	168	639	557	309	236	3024	5421	1659	156
ACC_07878	biglycan-like		KOG4237	21	14	11	20	5	6	2	3	76	52	8	21	41	88	123	15
ACC_07879	conserved hypothetical protein	K03945		283	187	217	839	207	21	18	16	153	30	554	201	148	194	15	14
ACC_07880	exocyst complex component 2		KOG2347	347	201	208	421	303	39	47	63	503	310	168	206	589	348	95	26
ACC_07881	LOW QUALITY PROTEIN	K01183	KOG2806	124	20	16	60	23	8	11	8	191	195	3	72	1251	805	393	103
ACC_07882	dolichyl-diphosphooligosaccharide--protein glycosy	K07151	KOG2292	542	418	253	394	335	118	241	255	373	471	155	238	2392	2306	376	157
ACC_07883	amyloid protein-binding protein 2		KOG1840	473	301	353	377	400	51	46	58	554	385	252	535	346	274	32	6
ACC_07884	glutaredoxin-related protein 5, mitochondrial-like	K07390	KOG0911	146	105	119	177	144	14	15	19	101	38	106	170	196	309	62	49
ACC_07885	conserved hypothetical protein			31	22	30	17	25	1	4	1	19	9	8	7	185	217	4	2
ACC_07886	mitochondrial import inner membrane translocase subunit T		KOG2580	180	89	91	201	155	38	51	51	320	231	93	227	605	1156	308	300
ACC_07887	conserved hypothetical protein			86	78	75	94	172	6	15	11	100	75	126	181	168	136	36	26
ACC_07888	conserved hypothetical protein			56	15	15	9	10	2	1	1	27	13	2	34	147	269	70	20
ACC_07889	neuroligin-4, Y-linked		KOG4389	215	154	125	154	148	13	12	22	156	107	45	156	249	336	33	6
ACC_07890	poly(A) RNA polymerase gld-2 homolog A-like		KOG2277	444	290	220	475	320	48	87	126	440	238	219	111	300	365	158	45
ACC_07891	zinc finger protein 729-like		KOG3608	584	415	412	506	387	42	57	66	761	506	213	181	324	351	99	22
ACC_07892	conserved hypothetical protein	K05290	KOG1349	161	160	168	292	253	16	33	37	176	128	109	348	295	249	33	10
ACC_07893	conserved hypothetical protein	K04661	KOG4004	19	12	18	18	10	2	5	9	29	72	9	9	3	2	114	195
ACC_07894	conserved hypothetical protein			57	26	23	34	29	4	10	5	9	92	7	3	14	2	1	2
ACC_07895	troponin C, isoform 3-like		KOG0027	246	43	21	150	44	343	571	451	1591	744	195	116	1117	818	1372	1322
ACC_07896	conserved hypothetical protein	K15190	KOG2899	429	321	348	462	152	68	108	125	918	664	121	195	278	366	209	146
ACC_07897	ceramide kinase-like	K04715	KOG1115	103	106	78	114	58	35	30	27	179	175	112	247	56	33	27	7
ACC_07898	tyrosine-protein kinase transmembrane receptor R	K05129	KOG1026	45	37	27	21	39	2	3	7	169	115	22	68	106	82	6	1
ACC_07899	ubiquitin carboxyl-terminal hydrolase calypso-like		KOG2778	106	104	96	168	142	11	8	24	119	73	76	110	66	79	26	8
ACC_07900	arginyl-tRNA--protein transferase 1	K00685	KOG1193	323	276	274	315	288	26	41	35	443	334	184	352	270	227	45	13
ACC_07901	mRNA-capping enzyme	K13917	KOG2386	495	370	336	515	433	71	99	122	737	416	292	453	902	982	136	82
ACC_07902	conserved hypothetical protein	K14570	KOG2248	92	52	44	57	20	4	5	8	92	123	19	37	124	86	117	91
ACC_07903	troponin C-like		KOG0027	96	15	4	28	10	19	28	28	304	134	32	77	18	19	18	23
ACC_07904	vitamin-K epoxide reductase			84	61	82	118	105	5	9	7	129	62	174	178	276	108	16	6
ACC_07905	conserved hypothetical protein		KOG3732	65	42	59	146	196	15	22	23	77	57	109	37	201	206	73	37
ACC_07906	ubiquitin carboxyl-terminal hydrolase CYLD isoform	K08601	KOG3556	84	59	38	77	66	10	12	16	170	172	40	72	207	150	44	12
ACC_07907	ubiquitin carboxyl-terminal hydrolase CYLD isoform 1			38	23	15	27	20	2	8	12	62	87	10	47	103	79	18	6
ACC_07908	adenylate cyclase 3	K08043	KOG3619	254	73	87	152	17	8	5	5	181	96	46	70	11	7	3	8
ACC_07909	alpha-tocopherol transfer protein-like		KOG1471	89	49	24	65	20	26	24	23	948	186	773	2201	55	18	20	27
ACC_07910	alpha-tocopherol transfer protein-like		KOG1471	14	7	3	13	6	13	15	13	180	126	450	489	194	221	20	13
ACC_07911	endoplasmic reticulum-Golgi intermediate compartment pro		KOG2667	83	40	32	98	101	13	16	14	94	44	80	112	121	121	17	6
ACC_07912	MAGUK p55 subfamily member 6		KOG0609	263	177	173	424	194	116	150	137	388	323	319	833	81	194	186	168
ACC_07913	b-cell CLL/lymphoma 7 protein family member B-like		KOG4095	74	51	58	124	115	3	6	8	75	54	39	146	97	84	19	3
ACC_07914	out at first protein-like			61	39	19	55	71	8	10	15	127	91	57	156	76	96	20	2
ACC_07915	phospholipase A1 member A-like isoform 1			411	317	379	713	752	74	169	203	233	115	87	88	36	130	31	32
ACC_07916	vesicular glutamate transporter 3-like		KOG2532	155	83	80	158	41	7	18	14	63	57	2	0	0	1	1	1
ACC_07917	conserved hypothetical protein			1	3	0	8	4	0	0	1	2	8	1	6	10	4	0	1
ACC_07918	Zinc transporter 1		KOG1483	55	41	26	29	19	7	7	8	18	30	1	11	28	19	1	0
ACC_07919	high affinity nerve growth factor receptor-like		KOG1026	13	4	12	23	5	4	13	6	340	308	9	3	117	99	84	26
ACC_07920	conserved hypothetical protein		KOG3700	2	0	2	4	6	0	0	2	2	3	2	1	2	22	4	1

ACC_07921	conserved hypothetical protein			1	2	0	0	0	1	0	0	1	1	1	1	3	1	0	1
ACC_07922	protein numb-like		KOG3537	549	501	598	1031	304	87	122	173	410	211	230	167	102	130	346	82
ACC_07923	conserved hypothetical protein		KOG2494	226	113	147	132	54	24	45	59	198	126	45	24	14	20	53	39
ACC_07924	ankyrin repeat and FYVE domain-containing protein 1-like		KOG4177	271	178	176	338	322	12	43	47	424	344	167	245	309	332	53	13
ACC_07925	pre-mRNA-splicing factor CWC22 homolog	K13100	KOG2140	345	165	184	461	473	38	81	100	310	212	349	313	327	459	426	263
ACC_07926	conserved hypothetical protein		KOG1676	7	3	6	13	4	0	1	7	10	10	2	1	3824	947	801	1225
ACC_07927	conserved hypothetical protein		KOG4791	616	413	423	363	164	44	54	63	827	596	87	361	758	463	178	50
ACC_07928	conserved hypothetical protein			199	182	185	266	255	15	22	43	155	124	192	355	296	360	49	19
ACC_07929	conserved hypothetical protein		KOG4220	52	44	24	31	24	2	5	0	212	291	18	75	305	173	12	5
ACC_07930	protein kinase C iota type		KOG0695	253	144	119	230	124	16	34	36	272	247	53	88	98	90	17	16
ACC_07931	conserved hypothetical protein	K07189	KOG3986	62	25	34	54	11	3	10	7	196	243	7	6	7	3	7	10
ACC_07932	conserved hypothetical protein			7	6	14	8	2	1	1	0	11	11	1	21	0	3	2	3
ACC_07933	conserved hypothetical protein	K16546		84	41	36	69	46	18	44	44	98	86	35	96	138	150	102	43
ACC_07934	DNA-directed RNA polymerase III subunit RPC1-like	K03018	KOG0261	360	335	286	340	335	56	80	92	663	486	167	189	697	779	133	57
ACC_07935	conserved hypothetical protein			58	18	4	20	6	4	16	7	618	214	37	54	272	212	245	271
ACC_07936	conserved hypothetical protein			215	171	168	273	104	15	18	23	332	389	39	143	258	257	68	19
ACC_07937	protein arginine N-methyltransferase 3	K11436	KOG1499	93	72	47	99	79	14	36	38	77	153	39	63	224	196	60	28
ACC_07938	conserved hypothetical protein			45	21	32	73	16	4	10	11	21	112	17	11	1	1	32	25
ACC_07939	ecdysone 20-monooxygenase-like	K10723	KOG0159	94	54	66	176	59	11	11	7	115	75	14	16	148	87	90	17
ACC_07940	conserved hypothetical protein			180	63	68	253	53	9	19	29	37	81	41	7	22	18	56	78
ACC_07941	conserved hypothetical protein	K09268	KOG0527	190	86	205	120	44	98	65	132	616	441	20	106	135	92	101	67
ACC_07942	SAP30-binding protein-like isoform 2		KOG2959	321	187	192	468	290	40	48	69	314	218	133	306	215	301	204	37
ACC_07943	conserved hypothetical protein			18	17	13	36	43	0	0	1	52	28	11	43	21	14	1	0
ACC_07944	conserved hypothetical protein		KOG1163	397	226	240	415	222	74	131	175	343	316	138	114	96	167	495	354
ACC_07945	Sperm surface protein Sp17			651	522	577	1164	751	170	231	304	397	237	489	80	17	10	104	85
ACC_07946	diphthine synthase	K00586	KOG3123	269	201	201	474	374	52	121	130	360	138	254	315	225	322	125	70
ACC_07947	LOW QUALITY PROTEIN		KOG3629	216	57	83	216	60	34	17	39	368	351	49	193	42	30	53	12
ACC_07948	conserved hypothetical protein		KOG3813	1536	596	638	1711	678	133	168	267	1068	590	324	240	326	306	411	168
ACC_07949	conserved hypothetical protein			119	83	80	90	98	7	14	12	106	60	27	108	125	133	16	5
ACC_07950	metallophosphoesterase 1-like		KOG3662	258	150	164	264	324	13	17	25	209	176	128	367	304	263	30	7
ACC_07951	beta-lactamase-like protein 2 homolog		KOG0813	343	264	267	465	578	58	85	129	757	248	809	1428	647	735	158	29
ACC_07952	conserved hypothetical protein			5	3	3	5	4	2	0	0	1	0	0	0	1	3	8	3
ACC_07953	adenylate kinase 8-like		KOG3078	23	8	8	44	28	1	1	8	4	10	0	0	1	0	0	0
ACC_07954	conserved hypothetical protein			212	124	134	233	73	34	53	93	92	64	39	33	24	40	270	141
ACC_07955	WD repeat domain phosphoinositide-interacting protein 4-li		KOG2111	142	90	109	223	224	13	21	27	181	135	72	229	145	156	28	8
ACC_07956	RNA-binding protein 26	K13192	KOG2135	502	395	386	725	493	71	105	122	612	491	176	276	578	807	360	166
ACC_07957	excitatory amino acid transporter 3		KOG3787	98	63	92	111	106	3	9	12	53	84	28	40	402	151	12	9
ACC_07958	ankyrin repeat domain-containing protein 50		KOG4177	994	497	616	1349	558	82	102	129	865	568	318	297	298	278	216	64
ACC_07959	serine hydrolase-like protein-like		KOG1454	59	56	47	70	97	2	10	8	194	148	124	479	220	184	11	6
ACC_07960	origin recognition complex subunit 3-like	K02605	KOG2538	97	53	69	98	98	7	17	19	117	194	74	98	92	180	33	14
ACC_07961	moesin/ezrin/radixin homolog 1		KOG3529	521	385	283	232	162	32	53	57	1183	838	248	1583	595	591	125	68
ACC_07962	conserved hypothetical protein			55	25	22	100	161	6	8	10	8	6	73	10	3	1	14	7
ACC_07963	conserved hypothetical protein			14147	3599	1439	4334	2077	7374	6731	11375	6523	8978	1608	8709	249	147	57261	44452
ACC_07964	conserved hypothetical protein			0	0	0	0	0	0	0	0	1	1	0	0	0	0	0	0
ACC_07965	proton-associated sugar transporter A-like isoform	K15378	KOG0637	69	51	61	133	66	43	59	78	691	349	25	31	2	6	1	6
ACC_07966	conserved hypothetical protein	K00774		86	55	35	151	154	8	23	25	118	81	124	175	137	118	44	13
ACC_07967	conserved hypothetical protein			810	465	415	662	840	53	178	177	324	347	394	293	256	308	287	60
ACC_07968	potassium channel subfamily T member 1-like			37	23	30	85	49	16	26	34	8	8	0	0	2	1	16	4
ACC_07969	conserved hypothetical protein			344	282	284	260	266	79	161	180	352	238	187	177	271	319	349	219
ACC_07970	conserved hypothetical protein			125	83	62	318	75	12	21	29	30	46	5	0	0	0	5	0
ACC_07971	phosphoenolpyruvate carboxykinase	K01596	KOG3749	4	1	0	12	6	2	1	0	890	33	19	7	5	8	0	0
ACC_07972	LON peptidase N-terminal domain and RING finger protein 2		KOG4159	32	14	16	41	7	1	5	4	59	70	11	22	16	24	5	1
ACC_07973	5-azacytidine-induced protein 1-like	K16540	KOG0161	28	14	18	14	13	6	8	10	56	53	744	63	76	59	12	18
ACC_07974	LIM/homeobox protein Lhx9-like		KOG4577	78	23	33	100	26	1	2	3	22	9	1	0	0	0	4	1
ACC_07975	lipase 3-like		KOG2624	69	32	51	49	50	25	49	46	51	63	78	24	688	974	60	8
ACC_07976	conserved hypothetical protein		KOG0956	756	759	799	563	209	76	83	108	1015	857	167	156	397	630	449	172
ACC_07977	conserved hypothetical protein			3	1	2	9	3	0	1	3	19	10	56	60	941	350	34	37

ACC_07978	conserved hypothetical protein		KOG2996	502	218	194	269	108	26	49	62	482	240	59	28	27	33	77	44
ACC_07979	conserved hypothetical protein			17	3	4	7	1	1	2	1	46	29	2	5	2	0	1	5
ACC_07980	conserved hypothetical protein	K16809	KOG0161	365	260	223	260	183	44	123	120	237	185	58	63	150	147	197	82
ACC_07981	conserved hypothetical protein			3	0	1	2	0	0	0	0	0	0	0	1	10	12	0	0
ACC_07982	conserved hypothetical protein			12	3	3	9	4	7	6	15	22	18	2	3	0	1	8	9
ACC_07983	conserved hypothetical protein	K09280	KOG3982	39	8	14	54	12	0	1	5	6	9	0	0	1	2	8	3
ACC_07984	conserved hypothetical protein			544	333	236	535	822	18	80	89	188	226	268	357	183	186	186	53
ACC_07985	1-phosphatidylinositol-4,5-bisphosphate phosphod	K05860	KOG1265	5310	6068	5279	10785	2323	417	674	1054	2528	1971	14	8	20	15	35	47
ACC_07986	glycoprotein 3-alpha-L-fucosyltransferase A-like	K00753	KOG2619	142	109	107	66	50	4	20	25	107	56	11	8	48	37	14	0
ACC_07987	proton-coupled amino acid transporter 4-like	K14209	KOG1304	107	129	27	59	38	28	35	25	569	274	62	34	401	217	34	12
ACC_07988	conserved hypothetical protein			3	2	1	3	6	0	5	4	5	2	2	0	0	0	0	0
ACC_07989	nucleoporin NDC1-like	K14315	KOG4358	161	121	114	175	210	6	14	15	170	162	110	122	399	446	41	10
ACC_07990	zinc finger and BTB domain-containing protein 24		KOG3623	5611	3929	4509	2881	1618	352	656	766	2441	2285	549	346	735	1610	1010	391
ACC_07991	conserved hypothetical protein		KOG4441	345	260	315	373	70	42	39	64	161	566	80	28	5	5	33	42
ACC_07992	IRP30, partial		KOG4194	3	1	6	38	17	6	2	0	2153	1268	1	6	1	0	1	0
ACC_07993	serine/arginine-rich splicing factor 7-like		KOG0107	146	121	113	268	175	31	43	52	121	94	92	145	159	221	116	105
ACC_07994	DNA repair protein XRCC2-like	K10879	KOG2859	48	43	42	68	103	4	7	8	38	63	84	73	72	61	9	8
ACC_07995	carbonic anhydrase-related protein 10-like		KOG0382	62	22	21	37	7	1	0	2	21	15	6	10	4	5	2	0
ACC_07996	conserved hypothetical protein		KOG2462	46	49	94	303	82	28	20	49	43	92	7	1	8	7	15	6
ACC_07997	nuclear migration protein nudC-like		KOG1999	174	79	67	206	108	35	86	94	148	75	112	226	446	517	1715	2205
ACC_07998	netrin receptor UNC5B-like		KOG1480	498	158	133	346	165	25	27	28	140	26	76	55	2	10	39	35
ACC_07999	dehydrogenase/reductase SDR family member 11-like		KOG1205	16	195	108	569	200	97	101	104	176	71	2958	117	2303	2055	195	53
ACC_08000	dehydrogenase/reductase SDR family member 11-like		KOG1205	454	225	187	657	376	144	113	145	2686	607	10814	17320	1823	1112	75	43
ACC_08001	syntaxin-5-like	K08490	KOG0812	205	163	113	309	242	34	55	51	537	254	228	542	570	535	100	43
ACC_08002	arginine kinase-like		KOG3581	13	5	8	12	3	2	2	2	5	12	5	6	2	2	7	3
ACC_08003	putative ATP-dependent RNA helicase me31b-like i	K12614	KOG0326	887	548	429	631	287	147	233	273	1171	1158	296	370	770	1155	778	330
ACC_08004	squamous cell carcinoma antigen recognized by T-cells 3		KOG0128	416	291	319	410	261	52	120	126	540	372	174	266	407	559	267	127
ACC_08005	conserved hypothetical protein		KOG0500	39	38	26	34	4	2	4	4	33	34	3	0	8	4	6	9
ACC_08006	conserved hypothetical protein		KOG1984	219	235	319	230	39	53	74	121	161	203	110	40	16	38	443	315
ACC_08007	conserved hypothetical protein	K15309		116	91	91	153	28	19	20	45	93	107	646	506	19	26	70	29
ACC_08008	conserved hypothetical protein			158	103	112	156	168	7	25	24	135	82	80	264	368	439	43	25
ACC_08009	odorant receptor 2	K08471		5	3	12	25	11	1	2	3	58	58	845	965	2	1	1	1
ACC_08010	zinc metalloproteinase nas-15-like isoform 2		KOG3714	11	0	2	5	3	1	1	3	11	20	1	5	138	19	13	7
ACC_08011	laminin subunit alpha-1-like		KOG1836	0	1	0	1	0	0	0	0	1	2	0	0	0	0	0	0
ACC_08012	homeodomain-interacting protein kinase 2	K08826	KOG0667	753	813	791	864	214	172	181	201	2768	2638	542	539	212	245	902	289
ACC_08013	lipase member H-A-like			36	6	13	29	4	1	3	7	51	227	46	97	41	67	178	4
ACC_08014	conserved hypothetical protein			15	13	16	25	18	2	11	11	21	36	17	27	4	1	0	1
ACC_08015	eyegone			0	0	0	0	0	0	0	0	2	1	69	57	4	7	14	17
ACC_08016	otopetrin-2-like		KOG4740	551	610	961	915	498	71	142	188	457	291	23	44	10	37	105	64
ACC_08017	conserved hypothetical protein		KOG1170	259	67	47	142	47	43	77	76	578	307	106	448	255	380	228	791
ACC_08018	2-methoxy-6-polyprenyl-1,4-benzoquinol methylas	K06127	KOG1540	102	79	80	141	164	8	16	8	129	42	106	116	143	245	17	19
ACC_08019	eukaryotic translation initiation factor 3 subunit E-l	K03250	KOG2758	324	202	164	310	303	15	35	33	391	405	162	631	1654	2388	220	39
ACC_08020	dimethyladenosine transferase 1, mitochondrial-lik	K15266	KOG0821	609	438	405	724	608	42	83	96	571	507	339	838	1418	1466	255	65
ACC_08021	spondin-1		KOG3539	141	49	37	131	54	39	37	35	1266	2205	14	112	362	336	476	209
ACC_08022	putative oxidoreductase GLYR1 homolog		KOG0409	106	78	62	81	59	11	7	7	342	386	80	322	315	380	101	29
ACC_08023	putative oxidoreductase GLYR1 homolog		KOG1904	132	102	72	134	96	22	27	20	416	508	143	351	344	491	81	47
ACC_08024	peritrophin-1-like			3	1	1	17	4	0	0	0	0	0	2	0	12	10	54	0
ACC_08025	flap endonuclease GEN-like	K15338	KOG2519	267	157	137	275	299	34	54	82	179	94	106	113	131	157	101	64
ACC_08026	conserved hypothetical protein			11	7	5	15	8	2	2	10	138	44	72	26	28	91	29	3
ACC_08027	dipeptidase 1-like		KOG4127	40	13	17	45	12	1	1	2	20	9	2	9	0	3	6	3
ACC_08028	tyrosine-protein phosphatase 99A-like		KOG4228	784	348	333	1356	309	52	41	68	928	682	84	150	191	196	379	127
ACC_08029	hematopoietically-expressed homeobox protein hh	K09310	KOG0489	0	0	0	1	0	0	0	0	1	0	5	0	0	1	15	2
ACC_08030	conserved hypothetical protein		KOG0612	272	176	139	187	129	39	63	54	750	402	167	147	197	155	66	27
ACC_08031	zinc finger protein 184-like	K12845	KOG3387	122	98	69	208	170	7	20	23	56	57	66	179	546	976	154	52
ACC_08032	conserved hypothetical protein		KOG2072	556	261	312	352	99	44	59	106	714	680	173	238	117	89	181	157
ACC_08033	Actin-like protein 6A		KOG0679	264	102	93	257	222	62	111	130	165	99	35	19	72	79	74	65
ACC_08034	actin-like protein 6A-like isoform 2	K11652	KOG0679	129	67	66	186	36	7	7	9	26	33	6	1	2	3	17	23

ACC_08035	Down syndrome cell adhesion molecule-like protein CG4225		KOG3513	1262	629	775	1033	240	70	81	93	410	201	23	1	55	76	186	104
ACC_08036	conserved hypothetical protein			890	222	293	573	177	46	58	71	658	1362	155	175	92	75	138	45
ACC_08037	LOW QUALITY PROTEIN			458	318	382	875	288	31	50	103	86	44	53	15	48	21	67	78
ACC_08038	spermatogenesis-associated protein 20		KOG2244	205	171	180	212	224	21	20	20	371	279	123	389	437	418	27	7
ACC_08039	probable RNA-binding protein CG14230-like			297	149	110	206	373	12	35	19	38	124	217	301	187	147	199	146
ACC_08040	ankyrin repeat domain-containing protein 16-like		KOG0509	25	12	17	64	23	5	3	6	22	20	16	16	31	22	21	8
ACC_08041	26S proteasome non-ATPase regulatory subunit 14	K03030	KOG1555	121	95	80	184	174	38	29	36	308	203	176	666	937	976	182	102
ACC_08042	cuticular protein analogous to peritrophins 3-D precursor			63	11	24	45	7	5	7	8	1074	1244	7	424	93	196	333	285
ACC_08043	basement membrane-specific heparan sulfate proteoglycan		KOG1215	103	36	17	23	12	2	10	9	72	25	10	4	15	14	86	60
ACC_08044	conserved hypothetical protein		KOG3612	178	84	116	76	33	7	11	11	167	104	16	16	9	10	15	8
ACC_08045	conserved hypothetical protein	K12186	KOG4000	396	211	179	449	397	41	66	81	256	217	158	218	717	762	145	21
ACC_08046	solute carrier family 25 member 36-A-like isoform	K15116	KOG0757	50	42	33	45	20	10	5	8	244	164	20	46	48	37	8	1
ACC_08047	calcium-binding mitochondrial carrier protein Aralar1		KOG0751	105	54	43	49	26	13	11	9	439	429	43	85	166	115	20	3
ACC_08048	putative acyl-CoA-binding protein-like isoform 2	K08762	KOG0817	106	115	101	159	126	19	20	21	1139	474	102	823	736	814	119	18
ACC_08049	voltage-gated potassium channel subunit beta-1 isoform 2			194	97	76	123	68	15	12	22	232	102	102	91	10	8	7	0
ACC_08050	conserved hypothetical protein			44	1	1	1	0	0	1	0	26	1	880	197	7626	713	21	228
ACC_08051	sideroflexin-2-like		KOG3767	461	111	105	300	186	16	9	14	275	196	55	231	419	435	75	24
ACC_08052	electron transfer flavoprotein-ubiquinone oxidorec	K00311	KOG2415	215	119	80	226	200	8	22	23	502	525	115	572	2429	2254	194	69
ACC_08053	putative sodium-dependent multivitamin transporter-like		KOG2349	135	91	65	186	103	7	10	20	124	80	52	272	171	166	60	10
ACC_08054	neutral and basic amino acid transport protein rBAT-like			37	23	26	15	13	2	5	4	74	86	10	43	18	3	0	2
ACC_08055	pleckstrin homology domain-containing family M member 1		KOG1829	117	77	87	129	162	46	52	72	337	205	58	182	88	79	22	10
ACC_08056	LOW QUALITY PROTEIN	K15183	KOG4796	451	485	274	539	154	288	308	516	625	463	110	115	254	172	359	174
ACC_08057	protein SZT2-like, partial			1	2	0	3	0	0	0	1	1	7	9	10	0	2	0	0
ACC_08058	protein SZT2-like, partial			373	244	237	424	417	25	47	48	247	369	115	122	434	409	57	5
ACC_08059	protein SZT2-like, partial			18	11	4	6	4	1	1	4	34	67	4	11	20	14	3	1
ACC_08060	regulation of nuclear pre-mRNA domain-containing	K15559	KOG2669	155	123	120	355	205	64	118	168	197	136	102	166	259	253	483	443
ACC_08061	dipeptidase 1-like			23	5	5	10	4	0	0	0	9	5	0	0	0	0	1	2
ACC_08062	leucine-rich repeat-containing protein 26-like		KOG4237	34	41	55	233	102	63	55	129	150	333	31	91	47	24	27	26
ACC_08063	calmodulin-lysine N-methyltransferase-like		KOG3201	282	216	256	573	211	36	60	79	482	147	100	141	89	131	153	100
ACC_08064	conserved hypothetical protein			81	54	51	136	135	20	28	34	107	78	64	91	108	87	60	21
ACC_08065	pumilio domain-containing protein C14orf21-like	K14790	KOG2188	309	180	156	336	259	80	122	128	376	388	163	276	368	428	384	306
ACC_08066	signal-induced proliferation-associated 1-like protein 2-like		KOG3686	212	128	135	245	48	87	55	95	507	255	69	107	68	186	215	67
ACC_08067	venom serine protease 34		KOG3627	35	30	46	529	63	19	21	25	224	130	58	41	5	3	12	8
ACC_08068	conserved hypothetical protein		KOG1924	753	862	1042	1439	890	163	292	359	767	606	262	91	157	209	605	493
ACC_08069	conserved hypothetical protein	K00666	KOG1176	97	30	24	81	30	73	76	93	6076	2656	145	75	1945	979	441	311
ACC_08070	peptidylglycine alpha-hydroxylating monooxygenase		KOG3567	390	214	205	455	395	42	38	54	427	181	115	158	143	140	12	4
ACC_08071	conserved hypothetical protein		KOG4702	81	79	56	109	120	5	11	9	82	37	129	174	101	77	9	8
ACC_08072	laccase-1-like		KOG1263	15	19	20	18	24	5	6	6	1096	200	130	2050	282	46	6	3
ACC_08073	conserved hypothetical protein			118	74	95	147	79	7	22	25	134	79	27	26	31	16	37	28
ACC_08074	COMM domain-containing protein 4-like			122	114	81	240	140	22	48	31	95	73	73	173	292	300	97	89
ACC_08075	tachykinins precursor			875	553	698	1772	1035	68	145	195	436	156	27	12	10	29	7	2
ACC_08076	charged multivesicular body protein 5-like	K12198	KOG1655	357	294	275	705	440	56	74	170	545	212	321	540	388	521	138	111
ACC_08077	apoptosis-inducing factor 3		KOG1336	397	346	361	421	400	50	67	82	537	349	246	496	627	607	70	47
ACC_08078	gamma-aminobutyric acid type B receptor subunit	K04615	KOG1055	586	334	330	565	153	27	37	47	328	300	451	371	81	51	32	15
ACC_08079	protein apterous	K09373	KOG0488	56	19	31	50	6	2	0	1	26	27	2	3	3	5	48	11
ACC_08080	exportin-7		KOG1410	176	108	113	129	121	4	16	14	376	444	62	168	572	519	32	12
ACC_08081	f-box/WD repeat-containing protein 1A	K03362	KOG0281	267	169	144	144	132	22	23	32	481	283	98	197	334	344	43	12
ACC_08082	protein FAM173B-like		KOG4058	115	77	82	227	210	9	12	17	148	108	207	374	215	172	9	3
ACC_08083	chymotrypsin inhibitor-like			1849	570	609	2760	501	292	661	261	758	2056	55	62	2251	668	1944	1119
ACC_08084	conserved hypothetical protein			3	0	1	1	0	1	1	3	13	9	1055	2903	0	0	2	0
ACC_08085	conserved hypothetical protein			680	3079	5928	3902	2935	3313	4271	14236	12	12	3	0	1001	264	19	4
ACC_08086	organic cation transporter protein-like		KOG0255	0	0	0	0	0	0	0	0	0	1	0	0	0	0	0	1
ACC_08087	anionic trypsin-2		KOG3627	0	0	2	5	0	1	1	3	4	0	1	0	328	662	133	5
ACC_08088	octopamine receptor	K04165	KOG4220	319	124	166	789	171	23	35	50	151	50	81	19	13	1	20	5
ACC_08089	headcase protein-like		KOG3816	91	55	55	61	19	18	18	17	52	53	20	34	38	28	93	29
ACC_08090	facilitated trehalose transporter Tret1-like		KOG0254	1152	2707	1386	405	251	1928	2976	4769	635	335	529	729	33	29	113	72
ACC_08091	chymotrypsin inhibitor-like			64	34	15	3	2	8	21	27	3	3	1	3	11	16	5	1

ACC_08092	conserved hypothetical protein			2216	8741	5914	2060	2739	12322	19401	40145	61	19	7	9	14	47	47	1
ACC_08093	dynein beta chain, ciliary-like		KOG3595	1	7	3	11	2	1	0	1	5	7	18	4	8	16	37	4
ACC_08094	transmembrane protein 161B-like		KOG3978	211	163	169	221	269	17	32	36	517	257	153	409	584	809	53	33
ACC_08095	glycerol-3-phosphate acyltransferase 1, mitochond	K00629	KOG3729	751	406	360	430	537	64	120	96	4689	5315	194	659	9734	3191	304	127
ACC_08096	F-box/LRR-repeat protein 7-like isoform 1	K10273	KOG4341	48	57	26	67	79	7	8	8	127	105	47	160	157	128	18	2
ACC_08097	conserved hypothetical protein		KOG2408	31	34	21	158	52	106	96	174	1230	438	235	633	1	0	2	0
ACC_08098	conserved hypothetical protein		KOG1084	1136	760	779	1779	1098	282	506	602	887	1002	481	274	569	698	1257	618
ACC_08099	conserved hypothetical protein			56	39	58	92	93	0	4	5	34	45	47	43	121	112	13	6
ACC_08100	conserved hypothetical protein			10	3	9	5	10	2	2	4	7	14	6	3	58	54	7	3
ACC_08101	conserved hypothetical protein		KOG1144	46	23	13	68	18	13	20	11	611	233	19	8	41	26	42	22
ACC_08102	protein Wnt-11b		KOG3913	39	20	15	52	18	4	3	3	58	57	8	21	10	4	12	4
ACC_08103	peripheral plasma membrane protein CASK-like		KOG0609	209	79	102	213	62	7	21	23	187	122	46	53	17	11	9	2
ACC_08104	PRKC apoptosis WT1 regulator protein-like			437	457	504	553	277	108	88	126	617	314	173	274	177	147	68	23
ACC_08105	autophagy-related protein 101-like isoform 1		KOG4493	216	174	172	239	206	31	20	28	145	156	44	291	197	183	55	7
ACC_08106	conserved hypothetical protein	K00750		776	384	215	414	421	82	553	357	1198	1094	301	295	2597	1628	883	442
ACC_08107	glycogenin-1-like	K00750		37	28	20	36	22	3	0	2	72	54	6	32	72	37	8	4
ACC_08108	conserved hypothetical protein			193	163	152	191	230	45	107	111	534	833	218	708	224	112	40	8
ACC_08109	conserved hypothetical protein			19	15	26	18	37	1	3	8	43	45	18	33	24	9	4	4
ACC_08110	odorant binding protein 7			30	441	183	217	130	69	87	111	3801	2849	38277	4578	17	0	1	4
ACC_08111	odorant binding protein 5 precursor			4	0	0	2	1	0	0	1	79	22	4036	3409	0	0	0	0
ACC_08112	conserved hypothetical protein			40	17	23	32	10	0	1	0	32	11	0	0	3	4	1	0
ACC_08113	odorant receptor 115			2	0	1	0	0	0	1	0	2	2	21	62	0	1	1	1
ACC_08114	dynein heavy chain 10, axonemal-like			5	1	5	6	3	1	1	0	13	7	5	0	0	0	13	12
ACC_08115	aldehyde dehydrogenase family 3 member B1		KOG2456	533	384	341	797	637	80	90	120	1939	1412	1196	4559	3834	2613	175	53
ACC_08116	sulfide		KOG3851	196	118	141	276	293	15	30	60	213	187	130	273	552	439	50	20
ACC_08117	conserved hypothetical protein	K15188	KOG0834	1364	1144	980	1138	519	321	573	638	1258	1554	430	203	322	316	2319	1167
ACC_08118	DNA-directed RNA polymerase III subunit RPC5	K14721	KOG2354	73	58	53	113	76	8	11	18	129	110	39	86	261	382	38	23
ACC_08119	LOW QUALITY PROTEIN	K04512	KOG1924	590	241	253	690	171	45	80	95	918	367	293	92	35	114	128	150
ACC_08120	histone-lysine N-methyltransferase SETMAR-like			11	10	5	5	1	1	2	0	8	12	3	12	1	1	1	0
ACC_08121	conserved hypothetical protein		KOG4389	48	16	17	40	23	2	5	12	40	33	5	4	2	8	18	10
ACC_08122	conserved hypothetical protein		KOG1925	172	133	193	189	74	26	36	30	1148	878	32	41	37	60	51	37
ACC_08123	golgin subfamily A member 5		KOG4677	398	347	350	394	365	55	128	139	354	240	200	350	420	432	140	70
ACC_08124	semaphorin-1A-like		KOG3611	18	6	1	13	1	1	2	3	13	45	1	10	0	1	8	0
ACC_08125	conserved hypothetical protein		KOG1055	7	3	1	2	0	0	0	0	4	2	5	2	0	0	0	0
ACC_08126	conserved hypothetical protein			289	179	190	758	337	60	87	97	560	382	531	689	387	388	272	28
ACC_08127	conserved hypothetical protein			8	4	9	16	11	0	3	1	46	49	23	67	25	22	0	0
ACC_08128	esterase E4-like		KOG1516	17	85	28	277	102	90	86	25	22835	8154	165	756	19	11	8	11
ACC_08129	UPF0197 transmembrane protein C11orf10 homolog		KOG4452	224	215	170	466	212	19	34	23	127	66	340	355	374	310	22	22
ACC_08130	protein RSM22 homolog, mitochondrial-like		KOG2539	193	156	175	183	188	31	52	47	153	152	102	175	249	387	84	92
ACC_08131	laminin subunit alpha, partial		KOG1836	13	3	1	7	3	11	19	21	141	879	1	40	416	162	307	253
ACC_08132	CTD nuclear envelope phosphatase 1 homolog		KOG1605	86	57	52	119	79	13	13	22	166	159	20	63	156	216	66	10
ACC_08133	conserved hypothetical protein		KOG3700	115	71	26	55	12	38	102	54	1314	1098	36	23	36	41	58	58
ACC_08134	dexamethasone-induced Ras-related protein 1-like		KOG0395	35	29	28	85	29	5	13	9	260	163	5	9	3	3	6	2
ACC_08135	obscurin-like			13	5	3	14	4	1	0	0	3	5	1	0	1	0	0	0
ACC_08136	moesin/ezrin/radixin homolog 1		KOG3529	429	304	236	231	125	24	49	41	1028	724	235	1326	492	466	98	43
ACC_08137	s-adenosylmethionine synthase-like isoform 2	K00789	KOG1506	389	187	195	301	242	13	15	23	996	942	104	375	1873	2292	307	58
ACC_08138	chitobiosyldiphosphodolichol beta-mannosyltransf	K03842	KOG2941	290	163	167	142	236	6	32	25	158	168	184	280	277	268	15	9
ACC_08139	RNA (guanine-9-)-methyltransferase domain-conta	K15445	KOG2967	184	90	111	147	170	26	65	73	300	218	150	343	345	307	167	80
ACC_08140	conserved hypothetical protein		KOG4635	154	100	116	171	154	6	15	8	249	275	40	260	149	89	12	5
ACC_08141	pecanex		KOG3604	1768	1152	775	702	611	166	282	296	1902	1359	309	365	711	576	263	147
ACC_08142	UDP-glucuronosyltransferase 2C1-like		KOG1192	358	271	347	1439	933	28	19	39	886	1838	82	258	77	178	46	5
ACC_08143	putative phosphatidate phosphatase-like	K01080	KOG3030	117	88	50	156	104	45	71	88	116	98	65	112	97	47	63	21
ACC_08144	N-acetylserotonin O-methyltransferase-like proteir	K06287	KOG1509	63	58	90	142	156	4	13	16	99	83	69	91	165	160	15	9
ACC_08145	endocuticle structural glycoprotein SgAbd-8			73	5	1	14	5	10	6	7	554	570	256	2093	404	10	65	50
ACC_08146	conserved hypothetical protein		KOG4226	842	389	399	437	215	35	53	84	569	354	75	40	79	67	94	62
ACC_08147	synaptic vesicle glycoprotein 2B-like		KOG0254	91	69	61	170	82	12	6	16	161	87	51	71	3	6	10	4
ACC_08148	conserved hypothetical protein		KOG3882	609	373	530	625	175	31	55	77	289	199	192	102	11	32	57	23

ACC_08149	conserved hypothetical protein		KOG0016	2886	1796	1180	2844	2852	644	1434	1666	2627	2230	2355	1418	2057	2477	2782	954
ACC_08150	conserved hypothetical protein			48	33	33	59	65	4	4	10	41	48	7	16	194	54	10	3
ACC_08151	probable helicase with zinc finger domain-like		KOG1804	161	104	109	241	90	23	15	29	182	195	70	113	93	180	163	51
ACC_08152	28S ribosomal protein S14, mitochondrial-like	K02954	KOG1741	456	350	339	904	704	42	55	60	396	173	515	737	386	497	83	75
ACC_08153	leucine-rich repeat-containing protein 24 isoform 1		KOG4194	48	26	30	36	4	1	1	4	16	53	0	0	2	2	2	2
ACC_08154	major facilitator superfamily domain-containing protein 6-lik		KOG3762	852	359	449	993	360	71	86	128	1898	8900	86	107	740	478	2597	284
ACC_08155	conserved hypothetical protein		KOG3632	1523	1695	1824	988	244	69	99	120	1496	1302	352	170	106	68	181	61
ACC_08156	neuronal acetylcholine receptor subunit alpha-10, partial		KOG3646	127	20	24	121	51	5	6	3	29	8	0	1	2	6	8	15
ACC_08157	conserved hypothetical protein			87	55	49	50	9	4	4	9	27	35	4	18	2	6	27	14
ACC_08158	glutathione S-transferase C-terminal domain-containing prot		KOG2811	247	139	164	306	300	26	45	49	334	228	155	386	313	384	88	22
ACC_08159	conserved hypothetical protein			50	33	39	53	19	8	8	9	235	457	31	53	21	5	30	57
ACC_08160	MOSC domain-containing protein 2, mitochondrial-like		KOG2362	313	144	111	257	85	102	187	213	375	533	119	234	638	335	454	275
ACC_08161	conserved hypothetical protein		KOG0962	12	11	13	26	10	2	1	3	33	30	31	5	4	2	9	7
ACC_08162	conserved hypothetical protein			21	14	6	27	8	1	1	1	188	18	113	318	1214	1069	333	1604
ACC_08163	transient receptor potential-gamma protein isofor	K05328	KOG3609	120	44	49	79	20	1	4	2	47	44	4	1	11	36	68	23
ACC_08164	cytosolic carboxypeptidase 1-like		KOG3641	515	195	161	668	199	22	33	44	1680	281	20	13	37	28	151	135
ACC_08165	peptidyl-alpha-hydroxyglycine alpha-amidating lyase 1-like isoform 1			26	23	7	34	26	0	5	2	16	5	19	8	17	18	6	3
ACC_08166	cytochrome P450 9e2		KOG0158	1099	942	1585	3566	1513	69	104	108	3286	1968	655	738	995	363	86	16
ACC_08167	mature T-cell proliferation 1 neighbor protein-like			251	147	168	309	264	3	14	29	451	308	116	675	617	541	51	19
ACC_08168	conserved hypothetical protein	K05284	KOG3893	324	162	186	272	314	13	24	28	468	305	190	496	571	428	30	8
ACC_08169	conserved hypothetical protein		KOG0943	147	8	13	17	6	0	3	2	57	6	11	27	5	688	841	407
ACC_08170	conserved hypothetical protein			0	1	2	1	2	1	0	0	3	0	1	0	1	4	2	0
ACC_08171	LIM domain only protein 3-like			29	15	26	60	14	2	2	6	24	30	1	2	0	0	1	0
ACC_08172	calmodulin-like isoform 1		KOG0027	81	16	25	64	15	2	1	4	15	12	0	0	22	4	13	3
ACC_08173	RIB43A-like with coiled-coils protein 1-like		KOG1029	51	37	33	59	36	7	9	12	52	29	15	15	24	41	63	71
ACC_08174	ATP-binding cassette sub-family G member 1-like		KOG0061	65	14	38	94	24	8	12	12	168	82	875	1876	241	16	35	24
ACC_08175	methylcrotonoyl-CoA carboxylase beta chain, mito	K01969	KOG0540	28	33	22	52	31	11	21	37	237	374	43	88	980	835	227	124
ACC_08176	conserved hypothetical protein			4	0	1	2	0	1	0	0	0	3	1	10	1	1	1	1
ACC_08177	Eph receptor tyrosine kinase precursor	K05110	KOG0196	398	94	147	270	50	11	11	19	183	133	37	50	47	79	336	215
ACC_08178	conserved hypothetical protein			34	68	43	277	97	8	13	11	311	172	129	150	48	80	72	37
ACC_08179	leucine-rich repeats and immunoglobulin-like domains prote		KOG4194	8	17	11	16	6	0	0	2	4	7	3	4	14	20	6	0
ACC_08180	leucine-rich repeat-containing protein 15-like		KOG4194	5	4	2	7	8	5	6	11	33	8	0	34	54	52	3	1
ACC_08181	serine/threonine-protein phosphatase 2A 56 kDa r	K11584	KOG2085	438	225	233	433	287	44	45	49	623	521	134	373	415	512	415	166
ACC_08182	conserved hypothetical protein			37	32	40	39	50	1	2	5	8	4	26	26	48	61	20	12
ACC_08183	conserved hypothetical protein		KOG3249	179	86	77	109	115	8	26	44	251	101	118	173	153	171	10	8
ACC_08184	Nucleosome-remodeling factor subunit NURF301	K11728	KOG1473	1924	1370	1181	1628	1452	542	1172	1230	1950	1653	895	408	521	733	2220	1135
ACC_08185	conserved hypothetical protein			7	1	3	7	7	2	3	1	3	2	2	2	3	60	23	30
ACC_08186	conserved hypothetical protein			47	40	55	73	29	139	239	220	160	90	84	318	29	45	413	122
ACC_08187	n-glycosylase/DNA lyase-like		KOG2875	28	17	33	36	31	3	8	8	98	72	38	30	140	152	28	15
ACC_08188	conserved hypothetical protein			229	64	55	82	64	10	15	22	31	24	5	3	3	1	0	1
ACC_08189	PDF receptor		KOG4564	1019	314	274	438	294	25	38	55	227	134	17	24	1	2	0	1
ACC_08190	protein SMG9-like isoform 1		KOG4181	157	101	92	153	172	7	14	36	156	159	122	317	388	280	55	27
ACC_08191	GPI transamidase component PIG-S-like	K05291	KOG2459	392	308	282	564	447	77	106	100	748	499	280	831	940	690	142	41
ACC_08192	myotubularin-related protein 4-like		KOG4471	527	379	364	791	506	86	106	126	1237	869	266	595	715	694	184	61
ACC_08193	leucine-rich repeat serine/threonine-protein kinase 1-like		KOG0192	517	280	286	432	357	46	85	90	1149	1185	128	407	295	184	46	16
ACC_08194	conserved hypothetical protein			104	64	98	120	112	19	30	34	57	38	2	3	10	2	49	19
ACC_08195	tolkin	K13046	KOG3714	189	76	70	102	42	18	8	20	428	394	2	20	110	64	33	10
ACC_08196	GL12416	K07374	KOG1376	5736	3767	4675	15642	6075	520	647	908	7450	2455	3466	8220	6386	7787	4423	1460
ACC_08197	conserved hypothetical protein		KOG0670	1081	707	573	1275	848	176	393	435	1032	745	706	362	670	787	1104	642
ACC_08198	conserved hypothetical protein			2	8	3	1	1	0	0	4	3	18	0	0	0	0	4	4
ACC_08199	conserved hypothetical protein		KOG2058	2459	1577	1685	1305	290	330	396	384	697	532	237	85	10	15	39	40
ACC_08200	trypsin-1-like		KOG3627	24	19	15	29	14	3	4	6	9	12	12	1	1500	860	2956	46
ACC_08201	conserved hypothetical protein			41	25	18	47	42	4	6	12	54	33	20	21	50	124	34	8
ACC_08202	conserved hypothetical protein	K01106	KOG1976	755	544	384	651	420	92	214	219	1843	1065	101	202	148	181	78	29
ACC_08203	glutamate-gated chloride channel	K05273	KOG3644	4045	2434	3355	3058	719	80	110	137	1845	1451	118	79	36	47	78	31
ACC_08204	conserved hypothetical protein		KOG0384	884	355	491	858	262	133	380	307	684	498	177	210	236	465	1711	979
ACC_08205	conserved hypothetical protein		KOG3771	413	493	697	3580	1263	33	27	56	2190	1007	157	553	584	164	27	15

ACC_08206	conserved hypothetical protein		KOG4221	446	190	217	659	376	18	17	28	157	32	7	67	5	10	3	4
ACC_08207	conserved hypothetical protein			0	3	1	4	1	2	2	3	13	2	2	2	107	189	29	3
ACC_08208	conserved hypothetical protein			27	8	9	20	16	6	6	6	87	27	43	143	10	10	0	3
ACC_08209	odorant receptor 41			14	5	6	16	12	6	3	1	16	11	60	47	0	0	0	0
ACC_08210	odorant receptor 55			0	4	2	3	6	0	0	2	0	5	5	6	0	0	0	0
ACC_08211	conserved hypothetical protein			37	76	69	146	65	2	6	5	18	12	15	14	0	0	0	0
ACC_08212	odorant receptor 58			1	12	14	28	20	0	0	2	4	4	5	1	0	0	0	0
ACC_08213	plastin-3		KOG0046	324	334	293	626	198	141	163	220	597	493	121	127	120	101	245	186
ACC_08214	probable ribosome production factor 1-like	K14846	KOG2780	267	144	112	396	344	47	131	120	360	202	214	258	445	863	673	387
ACC_08215	e3 ubiquitin-protein ligase IAP-3-like	K04725	KOG1101	73	63	46	59	75	4	9	15	136	130	51	252	191	254	27	10
ACC_08216	conserved hypothetical protein			591	540	739	1516	1319	160	357	382	4488	2408	1596	1805	973	1060	594	213
ACC_08217	cytosolic carboxypeptidase 6-like		KOG3641	211	121	166	204	89	18	31	62	26	39	11	5	2	3	34	22
ACC_08218	conserved hypothetical protein		KOG1904	454	293	241	523	367	123	199	226	973	685	363	753	763	806	496	250
ACC_08219	conserved hypothetical protein	K08471		152	103	124	313	349	18	41	40	44	34	20	9	2	8	4	1
ACC_08220	18-wheeler precursor		KOG4237	9	5	3	28	3	2	0	1	17	15	4	3	0	2	2	2
ACC_08221	conserved hypothetical protein	K09186	KOG1080	3398	3005	2233	2610	1553	579	1104	1308	3827	3420	729	660	732	653	1655	738
ACC_08222	acyl-CoA desaturase 1-like	K00507	KOG1600	6	5	4	16	21	2	0	4	8	18	7	8	548	1943	137	12
ACC_08223	protein GDAP2 homolog		KOG2633	317	110	95	182	100	16	31	28	469	387	85	223	127	109	54	8
ACC_08224	phosphatidylcholine	K04714	KOG3058	1095	554	578	1185	597	165	241	285	1910	587	916	677	658	358	201	74
ACC_08225	phosphatidylinositol-glycan biosynthesis class F prc	K05287	KOG3144	39	38	41	64	60	8	8	7	35	14	40	72	39	57	6	2
ACC_08226	protein lethal(2)denticleless-like	K11790	KOG0321	109	57	63	118	91	8	15	14	116	94	65	82	200	267	74	16
ACC_08227	conserved hypothetical protein			10	5	5	6	5	0	2	1	8	2	0	3	0	0	2	0
ACC_08228	n-acetylglucosamine-6-sulfatase-like isoform 2	K01137	KOG3731	98	73	78	89	85	13	19	15	80	92	61	110	97	101	11	7
ACC_08229	conserved hypothetical protein			3	3	5	19	10	1	1	0	33	19	27	11	9	7	5	0
ACC_08230	conserved hypothetical protein			6	0	0	7	5	0	4	3	24	10	4	23	0	1	0	0
ACC_08231	n-acetyllactosaminide beta-1,3-N-acetylglucosamir	K00741	KOG3765	40	22	21	29	8	0	0	3	24	24	19	41	3	4	2	1
ACC_08232	conserved hypothetical protein		KOG4260	558	497	638	971	248	32	39	64	509	1010	48	200	32	58	79	38
ACC_08233	PCI domain-containing protein 2		KOG2688	239	204	244	377	446	22	82	80	293	162	221	328	480	686	90	43
ACC_08234	conserved hypothetical protein			0	0	0	0	0	0	0	0	0	0	0	1	8	5	1	6
ACC_08235	conserved hypothetical protein	K11135	KOG2809	955	525	506	433	617	78	226	258	586	313	584	483	818	1078	736	393
ACC_08236	integrin alpha-PS2		KOG3637	488	190	157	231	95	47	57	80	1346	567	196	309	173	165	75	38
ACC_08237	alpha-catulin-like		KOG3681	215	130	137	153	43	9	13	17	191	258	13	54	21	17	48	12
ACC_08238	conserved hypothetical protein			27	16	8	34	13	14	21	33	52	51	10	10	11	4	90	94
ACC_08239	conserved hypothetical protein			139	76	65	123	59	8	15	11	60	39	2	4	10	6	9	3
ACC_08240	sideroflexin-3-like isoform 1		KOG3767	202	79	108	337	111	69	62	140	196	258	28	39	1328	171	77	32
ACC_08241	bone morphogenetic protein 2-B	K04384	KOG3900	37	20	23	110	34	3	11	6	162	242	19	24	111	88	220	233
ACC_08242	polynucleotide 5'-hydroxyl-kinase NOL9-like		KOG2750	274	140	140	303	159	60	73	95	554	448	183	365	492	662	286	104
ACC_08243	conserved hypothetical protein	K03649	KOG4120	286	300	487	168	38	19	33	40	306	431	47	78	63	44	103	98
ACC_08244	transketolase isoform 2	K00615	KOG0523	319	209	99	311	293	50	53	59	5089	4737	350	1879	20854	12661	809	206
ACC_08245	tubulin polyglutamylase TTLL13-like	K16582	KOG2158	36	23	12	37	30	5	6	4	34	60	13	6	54	23	7	4
ACC_08246	conserved hypothetical protein	K02270		2475	862	894	3647	1220	142	283	221	1105	227	1035	2261	1457	1726	916	483
ACC_08247	polyphosphoinositide phosphatase		KOG1888	1008	866	1052	1322	1220	146	236	241	1622	721	452	498	362	421	85	48
ACC_08248	protein turtle-like		KOG3513	1394	396	515	2393	448	33	30	46	502	467	280	58	110	97	149	67
ACC_08249	protein turtle-like		KOG4221	5049	1166	1900	3996	1241	319	414	499	5312	1306	206	3734	21	29	64	36
ACC_08250	protein FAM151B-like		KOG3748	1412	525	567	1380	303	62	42	97	452	588	144	324	557	497	461	138
ACC_08251	LOW QUALITY PROTEIN	K06176	KOG2339	650	409	340	596	631	118	249	255	582	412	218	276	974	1267	589	348
ACC_08252	protein kibra-like isoform 1	K16685	KOG1028	345	278	293	355	189	47	92	96	445	397	81	121	161	233	465	353
ACC_08253	myosin-XVIIIa		KOG0161	91	103	86	105	35	20	26	27	183	100	40	33	67	112	46	42
ACC_08254	myosin-XVIIIa	K10362	KOG0161	679	610	565	1049	378	146	220	248	1480	681	314	224	375	789	472	327
ACC_08255	glycine receptor subunit alpha-3-like		KOG3644	178	135	109	163	193	16	12	23	299	187	91	380	438	325	38	4
ACC_08256	NAD-dependent ADP-ribosyltransferase sirtuin-4 is	K11414	KOG2683	57	48	43	56	66	4	4	6	101	65	55	97	138	167	14	8
ACC_08257	puff-specific protein Bx42-like	K06063	KOG2441	423	270	304	650	311	48	109	146	599	436	260	328	531	733	442	444
ACC_08258	conserved hypothetical protein			21	13	7	12	4	3	2	0	10	18	1	0	0	3	8	7
ACC_08259	conserved hypothetical protein		KOG3518	187	103	158	282	176	20	51	38	648	381	134	164	164	277	112	46
ACC_08260	neuroendocrine convertase 1-like isoform 1	K01359	KOG3526	14	3	7	9	4	1	3	2	10	5	2	3	61	38	55	49
ACC_08261	protein EFR3 homolog cmp44E isoform 1		KOG1877	1229	480	562	723	637	52	68	74	1291	493	154	253	278	276	34	9
ACC_08262	conserved hypothetical protein			54	39	36	54	70	4	6	10	75	68	22	46	60	64	10	4

ACC_08263	RNA-binding protein pno1-like	K11884	KOG3273	123	82	75	131	133	9	26	20	94	52	69	126	167	307	108	76
ACC_08264	u11/U12 small nuclear ribonucleoprotein 35 kDa pı	K13155	KOG0113	116	92	99	150	118	12	37	28	125	79	45	76	88	116	68	26
ACC_08265	troponin T isoform 2	K12046	KOG3634	1104	225	56	273	90	235	505	325	11114	4899	199	283	2625	2251	1831	2293
ACC_08266	eye-specific diacylglycerol kinase			31	22	29	23	9	0	6	7	18	39	2	0	0	0	8	6
ACC_08267	eye-specific diacylglycerol kinase	K00901	KOG0782	169	97	136	179	57	28	52	55	441	722	71	121	75	46	68	19
ACC_08268	eukaryotic translation initiation factor 3 subunit M-	K15030	KOG2753	244	210	119	306	352	36	44	38	637	316	380	730	1465	1880	129	50
ACC_08269	Bloom syndrome protein homolog, partial	K10901	KOG0351	739	398	330	672	618	80	173	185	593	466	455	383	682	816	466	175
ACC_08270	trypsin-3	K01312	KOG3627	1	7	14	84	46	37	28	44	11	121	2	1	2	1	19	1
ACC_08271	trypsin-1		KOG3627	4	0	2	14	2	1	0	0	2	11	1	0	37	83	51	2
ACC_08272	lysozyme c-1			43	45	36	44	23	9	8	9	893	795	12	40	20	0	3	1
ACC_08273	gastrin-releasing peptide receptor-like isoform 2		KOG4219	102	56	94	228	65	4	3	12	53	23	10	28	94	75	39	11
ACC_08274	conserved hypothetical protein		KOG1192	65	26	6	29	52	20	31	35	4464	1664	1	9	11	7	3	0
ACC_08275	conserved hypothetical protein			57	31	19	77	19	25	43	70	89	89	22	19	34	9	179	176
ACC_08276	alpha-2B adrenergic receptor-like		KOG4220	86	32	48	91	17	4	1	5	8	17	1	0	0	1	2	6
ACC_08277	conserved hypothetical protein		KOG3627	16	5	1	14	2	0	0	0	8	6	0	3	0	3	16	7
ACC_08278	conserved hypothetical protein			23	7	18	55	33	2	4	11	218	215	113	148	143	437	117	39
ACC_08279	cytochrome P450 9e2 isoform 4		KOG0158	26	38	62	599	106	24	15	16	304	46	721	4	153	50	47	9
ACC_08280	LIM domain kinase 1	K05743	KOG0192	128	111	71	128	106	16	16	33	348	352	66	198	133	129	29	7
ACC_08281	conserved hypothetical protein		KOG3660	1	0	0	0	1	1	3	0	19	9	28	5	9	4	0	1
ACC_08282	conserved hypothetical protein			74	47	44	41	67	3	15	11	16	15	25	12	36	54	84	40
ACC_08283	protein Malvolio	K12347	KOG1291	445	231	233	513	634	51	66	57	4805	875	266	635	1718	2694	98	15
ACC_08284	conserved hypothetical protein			16	6	13	11	0	3	1	2	15	19	2	2	1	79	64	14
ACC_08285	conserved hypothetical protein		KOG1703	53	12	6	53	13	11	12	15	1281	734	26	87	123	54	64	18
ACC_08286	conserved hypothetical protein		KOG2461	283	358	398	710	133	18	27	33	117	151	21	4	3	37	163	138
ACC_08287	kin of IRRE-like protein 2-like			108	25	25	81	33	6	9	9	37	21	14	5	3	15	18	10
ACC_08288	transmembrane protease serine 9		KOG3627	2	1	5	9	3	1	0	0	7	2	6	1	174	18	10	10
ACC_08289	conserved hypothetical protein			28	26	31	14	4	4	4	8	29	112	2	9	27	18	59	30
ACC_08290	conserved hypothetical protein		KOG0161	412	214	200	293	282	72	130	168	591	384	159	175	181	143	145	55
ACC_08291	conserved hypothetical protein		KOG0262	7194	3994	2549	1788	2371	957	3079	2708	2590	2045	1583	1290	14605	39574	67005	68110
ACC_08292	conserved hypothetical protein			21	15	6	16	10	16	16	14	12	11	6	3	46	66	27	5
ACC_08293	conserved hypothetical protein		KOG4675	479	343	359	321	247	77	125	102	994	503	296	202	569	661	121	39
ACC_08294	lissencephaly-1 homolog	K16794	KOG0295	2216	1135	1132	3352	983	198	227	386	1569	1146	723	1322	454	487	582	236
ACC_08295	conserved hypothetical protein			169	146	148	253	121	27	24	31	253	325	98	197	202	244	115	34
ACC_08296	conserved hypothetical protein		KOG4690	27	23	29	66	68	4	3	6	40	24	49	31	19	31	4	1
ACC_08297	conserved hypothetical protein			167	87	85	119	59	23	30	42	149	50	20	26	11	3	42	36
ACC_08298	ADP-ribosylation factor-like protein 4C-like isoform 1		KOG0070	106	56	43	78	15	4	3	18	77	137	8	2	5	13	18	16
ACC_08299	e3 ubiquitin-protein ligase MIB1-like isoform 6	K10645	KOG4412	36	24	34	24	16	3	6	8	83	66	10	24	25	27	9	5
ACC_08300	conserved hypothetical protein			503	358	502	869	204	32	49	61	366	482	75	154	208	153	224	106
ACC_08301	defensin			2	7	15	137	54	19	12	19	1311	386	12	59	1	3	2	0
ACC_08302	palmitoyltransferase ZDHHC15-like		KOG1315	164	89	69	133	135	13	8	9	286	213	57	216	293	223	29	5
ACC_08303	palmitoyltransferase ZDHHC15-like			43	15	32	43	40	3	3	3	20	18	9	16	13	17	2	0
ACC_08304	mannosyl-oligosaccharide alpha-1,2-mannosidase isoform A		KOG2204	26	14	14	21	5	6	10	9	19	34	8	8	8	9	53	26
ACC_08305	plexin domain-containing protein 2-like		KOG3848	790	245	243	398	313	36	42	42	2606	2219	314	737	1687	592	104	30
ACC_08306	conserved hypothetical protein			44	32	32	38	49	5	5	10	30	20	24	17	29	32	7	1
ACC_08307	conserved hypothetical protein			122	55	56	189	33	2	6	5	46	49	6	2	1	4	10	6
ACC_08308	conserved hypothetical protein			793	384	492	995	178	31	43	88	176	159	43	8	7	2	162	67
ACC_08309	cystinosin homolog		KOG4616	81	44	37	187	266	4	4	11	85	53	53	167	154	209	14	5
ACC_08310	conserved hypothetical protein			236	258	363	932	500	71	71	93	1671	2681	1073	6255	1755	728	199	79
ACC_08311	cardioacceleratory peptide receptor	K08376	KOG4219	109	76	41	10	6	12	17	15	19	65	0	3	4	2	0	0
ACC_08312	conserved hypothetical protein		KOG0161	2	2	4	3	3	0	1	1	1	5	0	0	0	0	1	0
ACC_08313	conserved hypothetical protein		KOG1408	502	401	348	252	109	61	87	90	1446	1377	188	336	212	191	92	35
ACC_08314	eukaryotic translation initiation factor 2D-like	K15027	KOG2522	282	174	178	289	239	42	71	51	484	247	178	221	413	619	125	47
ACC_08315	cytosolic carboxypeptidase 6-like			4	5	3	6	5	0	0	2	1	3	1	0	0	1	1	0
ACC_08316	RNA-binding protein 10-like	K13094	KOG0154	721	514	471	632	431	118	181	239	1166	838	264	435	947	1082	754	406
ACC_08317	mothers against decapentaplegic homolog 3-like	K04500	KOG3701	90	84	92	81	32	6	7	10	195	211	36	98	116	112	61	13
ACC_08318	integrin beta-PS-like	K05719	KOG1226	7	5	4	8	4	4	3	5	107	95	3	23	16	15	3	4
ACC_08319	phospholipase A2 isozymes PA3A/PA3B/PA5	K01047		498	427	748	1124	696	20	22	21	1363	1506	43	340	67	19	7	2

ACC_08320	ets DNA-binding protein pokkuri-like		KOG3804	68	80	98	52	13	9	16	10	103	135	10	103	63	79	266	90
ACC_08321	retinol dehydrogenase 11-like		KOG1208	3	7	10	5	6	0	0	0	23	9	10	130	33	16	1	0
ACC_08322	metabotropic glutamate receptor B precursor		KOG1056	55	23	20	36	13	2	1	2	20	6	1	1	0	0	3	2
ACC_08323	metabotropic glutamate receptor B precursor		KOG1056	217	86	91	196	59	8	11	18	72	20	9	8	0	1	4	1
ACC_08324	endothelin-converting enzyme 1		KOG3624	5198	2551	3388	9408	5952	359	574	744	3500	895	285	773	42	82	78	57
ACC_08325	conserved hypothetical protein			3	4	0	8	5	0	0	1	4	2	1	0	1	2	1	0
ACC_08326	transcription factor SOX-2-like	K16796	KOG0527	448	347	493	597	98	14	40	48	94	82	3	7	3	14	169	181
ACC_08327	tyrosine-protein kinase transmembrane receptor ROR1		KOG4194	13	15	7	17	12	4	5	7	17	15	0	3	2	4	17	11
ACC_08328	39S ribosomal protein L51, mitochondrial		KOG4045	118	98	73	160	186	24	40	69	121	80	164	273	425	581	80	68
ACC_08329	nucleoside diphosphate kinase homolog 5-like	K00940	KOG0888	177	91	132	257	206	65	122	131	352	123	66	63	82	83	96	56
ACC_08330	mob as tumor suppressor	K06685	KOG0440	294	140	101	249	227	17	29	26	338	392	46	367	537	376	49	11
ACC_08331	conserved hypothetical protein	K14709	KOG1558	80	45	61	156	105	40	36	34	499	136	238	372	200	172	130	27
ACC_08332	stress-associated endoplasmic reticulum protein 2-like		KOG3491	258	116	42	209	151	22	39	32	164	112	117	419	591	478	89	57
ACC_08333	venom acid phosphatase Acph-1-like		KOG3720	4	1	42	6	8	0	0	0	3	0	2	12	91	73	22	2
ACC_08334	actin, clone 205-like isoform 1	K05692	KOG0676	1610	179	59	356	67	50	88	56	12488	10175	252	532	3725	2254	3260	3085
ACC_08335	actin		KOG0676	36	7	4	13	6	7	7	6	344	193	32	25	91	77	185	129
ACC_08336	conserved hypothetical protein			413	283	281	527	535	38	80	106	360	360	222	321	575	484	101	42
ACC_08337	synaptic vesicle membrane protein VAT-1 homolog-like		KOG1197	1467	1378	740	1768	939	781	1689	1717	797	539	517	415	487	630	1015	641
ACC_08338	aarF domain-containing protein kinase 1-like isofor	K08869	KOG1235	231	147	139	198	223	16	22	36	445	312	124	465	719	783	91	15
ACC_08339	LOW QUALITY PROTEIN	K10393	KOG0246	290	195	198	367	100	18	39	36	346	311	111	96	93	90	130	102
ACC_08340	cohesin subunit SA-1-like	K06671	KOG2011	405	282	260	440	326	54	64	89	722	627	287	326	940	1326	137	33
ACC_08341	conserved hypothetical protein			89	47	48	65	122	0	1	0	11	36	25	108	50	37	3	0
ACC_08342	probable palmitoyltransferase ZDHHC24-like		KOG1311	79	55	47	62	56	3	4	7	37	29	43	95	186	245	10	5
ACC_08343	ecdysone-induced protein 75	K08701	KOG4846	186	262	484	622	73	65	65	106	351	929	177	82	61	87	368	301
ACC_08344	serine/threonine-protein kinase PLK3-like			17	4	5	7	2	0	2	0	37	62	2	23	6	4	1	3
ACC_08345	protein outspread			8	2	3	3	0	1	1	1	10	12	0	6	6	4	3	2
ACC_08346	sodium/calcium exchanger 3	K05849	KOG1306	113	12	14	92	25	8	8	7	99	39	34	11	2	2	16	18
ACC_08347	conserved hypothetical protein			315	280	283	405	433	55	110	98	316	207	336	432	305	291	131	59
ACC_08348	endothelin-converting enzyme 1-like			67	14	20	67	11	1	2	5	46	24	20	24	0	0	0	1
ACC_08349	putative transferase C1orf69, mitochondrial-like	K06980	KOG2929	151	130	152	218	282	21	26	32	201	154	128	206	186	295	50	19
ACC_08350	zinc finger protein 808-like		KOG2462	218	160	162	322	311	16	29	40	299	229	139	204	225	242	17	3
ACC_08351	replication protein A 70 kDa DNA-binding subunit i	K07466		705	473	498	660	754	69	108	144	1189	719	629	1062	1693	2321	215	114
ACC_08352	coiled-coil domain-containing protein 104-like		KOG4511	53	32	32	73	75	18	23	14	60	39	1224	1616	13	18	61	27
ACC_08353	conserved hypothetical protein			1900	1131	1180	1122	1686	125	459	506	855	748	1174	1327	864	854	929	470
ACC_08354	5'-3' exoribonuclease 2 homolog	K12619	KOG2044	426	392	373	363	176	63	74	95	608	519	157	422	686	738	225	98
ACC_08355	5'-3' exoribonuclease 2 homolog			41	44	38	51	37	4	2	5	64	36	14	46	60	72	23	1
ACC_08356	conserved hypothetical protein			71	47	79	98	74	203	335	293	212	96	151	394	34	54	598	164
ACC_08357	peptide chain release factor 1-like, mitochondrial-li	K02835	KOG2726	207	149	168	248	278	22	54	70	125	125	119	151	193	300	54	41
ACC_08358	protein rolling stone-like isoform 2			133	87	126	360	347	19	28	28	222	111	74	300	92	79	4	0
ACC_08359	odorant receptor 55			1	0	0	2	2	0	0	0	4	2	8	10	0	0	0	0
ACC_08360	odorant receptor 57			6	4	10	15	7	0	0	0	5	2	51	10	0	0	0	0
ACC_08361	odorant receptor 58	K08471		21	41	60	49	28	1	1	4	20	12	126	150	0	0	0	0
ACC_08362	conserved hypothetical protein		KOG3571	586	300	265	407	307	200	437	411	1490	1171	1143	541	168	240	380	161
ACC_08363	conserved hypothetical protein			21	10	36	359	268	28	20	22	3627	2145	79	287	11	3	1	0
ACC_08364	TNF receptor-associated factor 4-like	K09848	KOG0297	70	39	54	148	97	12	18	18	202	100	100	72	79	81	56	18
ACC_08365	conserved hypothetical protein			3	0	0	0	0	0	1	0	0	0	0	0	0	0	0	2
ACC_08366	conserved hypothetical protein		KOG1924	1	4	1	1	0	1	0	0	0	3	0	2	0	0	7	7
ACC_08367	conserved hypothetical protein		KOG4798	640	356	419	833	579	88	127	167	853	380	499	1047	783	639	193	85
ACC_08368	palmitoyltransferase ZDHHC15-like		KOG1315	74	30	49	63	88	3	8	8	146	94	30	100	149	118	8	3
ACC_08369	DNA polymerase zeta catalytic subunit-like	K02350	KOG0968	1715	1056	1022	1566	1776	78	225	208	1062	1221	704	835	980	854	253	88
ACC_08370	heparan-alpha-glucosaminide N-acetyltransferase-	K10532	KOG4683	45	26	24	103	56	10	14	18	40	26	38	16	151	106	65	34
ACC_08371	transcription factor SOX-2-like			154	125	165	283	69	16	18	25	43	22	6	3	7	26	143	113
ACC_08372	AN1-type zinc finger protein 2B-like		KOG3183	40	24	38	60	58	1	5	2	104	58	51	76	97	55	9	4
ACC_08373	fibronectin type-III domain-containing protein C4orf31 hom		KOG4806	0	0	1	1	0	0	0	3	20	7	0	0	8	7	0	0
ACC_08374	conserved hypothetical protein			16	15	6	10	12	0	1	1	27	26	4	13	58	30	3	1
ACC_08375	60S ribosomal protein L11-like	K02868	KOG0397	456	436	228	881	646	129	150	246	445	390	648	900	1976	2413	812	658
ACC_08376	conserved hypothetical protein			1	1	2	4	4	0	0	1	2	14	0	0	0	0	1	2

ACC_08377	CCHC-type zinc finger protein CG3800-like	K09250	KOG4400	239	122	117	175	81	14	18	8	675	746	40	393	1323	1659	867	116
ACC_08378	protein bicaudal D		KOG0999	445	225	167	667	372	95	155	147	637	528	187	391	892	1752	1977	304
ACC_08379	conserved hypothetical protein		KOG1418	5	1	0	3	1	0	1	0	1	37	3	5	36	26	9	5
ACC_08380	potassium channel subfamily K member 18-like		KOG1418	449	138	133	223	74	4	8	19	235	87	57	18	12	32	14	20
ACC_08381	sugar transporter SWEET1-like	K15382	KOG1623	20	10	11	49	62	0	1	3	22	27	21	67	29	33	0	2
ACC_08382	protein takeout-like isoform 3			16	3	5	59	23	10	14	6	316	99	359	5531	245	33	29	13
ACC_08383	ABC transporter G family member 20-like		KOG0059	159	261	88	165	115	115	80	109	1225	1700	488	1243	214	328	56	9
ACC_08384	dynein beta chain, ciliary-like		KOG3595	3	3	1	3	0	1	0	0	4	2	7	2	0	2	1	0
ACC_08385	headcase protein-like		KOG3816	109	84	56	117	18	8	9	17	102	120	37	25	60	66	81	27
ACC_08386	glutamate receptor, ionotropic kainate 1-like	K05313	KOG1052	25	7	16	21	11	1	6	4	41	11	33	11	5	8	5	1
ACC_08387	LETM1 and EF-hand domain-containing protein anon-60Da,		KOG1043	1398	791	676	1005	993	244	455	433	2287	1787	770	1081	4100	5621	2342	1536
ACC_08388	conserved hypothetical protein		KOG0996	589	152	100	479	417	85	234	279	262	109	316	215	847	2532	3160	1793
ACC_08389	conserved hypothetical protein			5	2	2	3	5	0	1	0	17	76	0	0	39	29	82	89
ACC_08390	e3 ubiquitin-protein ligase CBL-B-like			52	36	43	39	21	1	2	1	38	25	6	10	10	14	10	3
ACC_08391	conserved hypothetical protein		KOG0307	862	836	741	858	392	47	64	84	601	518	286	207	351	350	130	36
ACC_08392	conserved hypothetical protein			108	112	87	186	190	4	11	8	103	59	64	115	71	88	7	5
ACC_08393	cysteine-rich motor neuron 1 protein-like			10	6	3	18	3	0	1	1	19	20	1	0	0	0	1	1
ACC_08394	cysteine-rich motor neuron 1 protein-like, partial			241	133	136	228	71	23	20	45	557	532	68	163	52	70	67	12
ACC_08395	MACRO domain-containing protein 2-like isoform 2		KOG2633	879	660	680	1165	750	161	261	283	1100	651	800	1874	1348	1153	636	422
ACC_08396	group XV phospholipase A2-like	K06129	KOG2369	315	194	211	281	401	13	23	22	496	264	173	476	281	223	11	3
ACC_08397	heparin sulfate O-sulfotransferase-like		KOG3922	3	0	0	2	5	0	0	0	5	9	9	8	5	2	3	6
ACC_08398	probable 4-coumarate--CoA ligase 3-like		KOG1176	80	37	68	159	105	25	27	22	1206	1218	551	2581	600	1351	289	187
ACC_08399	activator of basal transcription 1-like	K14785	KOG3152	237	132	126	207	284	30	60	82	134	91	164	232	184	260	89	107
ACC_08400	chymotrypsin-1-like		KOG3627	15	16	10	64	38	4	10	10	72	37	21	33	642	321	515	3
ACC_08401	Small nuclear ribonucleoprotein F	K11098	KOG3482	238	177	170	218	194	27	69	66	173	93	194	291	470	674	201	181
ACC_08402	conserved hypothetical protein		KOG4575	31	14	7	26	4	4	0	8	175	141	55	186	53	55	52	15
ACC_08403	metabotropic glutamate receptor B precursor	K04611	KOG1056	555	220	229	482	181	22	62	56	130	38	15	19	1	3	12	2
ACC_08404	conserved hypothetical protein		KOG4817	1298	774	748	1793	866	342	701	845	1363	1359	684	269	417	458	3512	2666
ACC_08405	conserved hypothetical protein			1965	1186	1043	3727	2562	679	1377	1611	1734	1464	1494	286	666	940	5937	3880
ACC_08406	conserved hypothetical protein		KOG1984	3740	3502	3508	3566	1339	600	742	870	3993	3366	897	830	1978	2698	2118	806
ACC_08407	F-box only protein 28-like	K10306		210	147	117	196	189	29	29	43	355	208	121	347	266	265	60	26
ACC_08408	protein Star-like			29	8	20	25	12	0	0	3	28	4	13	10	15	23	72	15
ACC_08409	facilitated trehalose transporter Tret1-like		KOG0254	61	23	33	173	73	37	41	39	796	882	107	289	412	127	49	29
ACC_08410	conserved hypothetical protein			10098	2554	3378	10239	3517	424	602	1043	1731	1184	842	2688	345	259	858	423
ACC_08411	conserved hypothetical protein		KOG4237	399	128	155	371	116	10	43	37	272	659	169	318	141	206	539	421
ACC_08412	reversion-inducing-cysteine-rich protein with kazal motifs		KOG3626	430	179	197	626	210	20	46	40	330	332	57	63	72	159	272	113
ACC_08413	conserved hypothetical protein		KOG3568	460	454	735	2306	886	22	30	58	460	289	8	2	31	48	90	41
ACC_08414	probable 4-coumarate--CoA ligase 3-like		KOG1176	93	64	128	226	160	25	32	30	1677	2063	823	4291	1050	2080	429	267
ACC_08415	apyrase precursor		KOG4419	22	145	187	331	127	25	31	60	94	51	207	196	879	1263	1073	114
ACC_08416	brefeldin A-inhibited guanine nucleotide-exchange protein 3		KOG1846	1782	891	1027	2082	456	70	108	143	758	696	243	169	18	22	67	57
ACC_08417	cadherin-23-like		KOG1219	1088	177	211	1383	390	31	58	81	198	70	10	131	161	226	273	163
ACC_08418	sodium-coupled monocarboxylate transporter 2-like		KOG2349	25	30	25	128	45	14	10	13	407	250	38	82	331	148	96	41
ACC_08419	conserved hypothetical protein	K12597	KOG4400	789	372	265	726	760	84	196	216	620	510	579	309	1151	1327	1229	713
ACC_08420	conserved hypothetical protein			51	17	18	63	47	5	15	21	28	21	19	7	3	4	54	24
ACC_08421	UPF0485 protein C1orf144 homolog isoform 1			120	72	79	102	70	21	36	37	139	63	38	101	99	118	48	41
ACC_08422	nicotinamide mononucleotide adenylyltransferase	K06210	KOG3199	62	41	36	35	39	7	2	3	146	150	23	143	338	215	12	6
ACC_08423	dnaJ homolog subfamily C member 28-like		KOG0568	729	510	508	818	833	140	337	390	491	355	389	622	178	244	179	100
ACC_08424	conserved hypothetical protein		KOG3608	916	637	513	638	440	129	274	252	857	811	554	448	843	1260	1611	1053
ACC_08425	SET and MYND domain-containing protein 4-like	K09873	KOG0223	54	48	45	264	91	29	19	35	1073	890	237	622	121	121	63	34
ACC_08426	conserved hypothetical protein		KOG1883	1099	668	1005	842	127	19	50	71	550	672	172	181	48	63	229	112
ACC_08427	conserved hypothetical protein			20	8	9	14	5	4	5	1	6	8	2	0	1	1	29	24
ACC_08428	neuroligin 5		KOG4389	124	85	65	100	22	4	4	6	43	58	1	5	2	6	7	6
ACC_08429	translationally-controlled tumor protein homolog isoform 1		KOG1727	1185	914	426	1877	1720	241	426	479	1820	1445	1846	1832	5815	5417	603	217
ACC_08430	conserved hypothetical protein			15	10	20	20	4	3	0	3	10	9	1	0	0	0	5	6
ACC_08431	conserved hypothetical protein		KOG2302	120	116	119	191	21	4	10	16	51	27	0	1	7	36	160	54
ACC_08432	conserved hypothetical protein			14	5	7	26	10	6	8	6	647	216	58	4	14	125	15	77
ACC_08433	conserved hypothetical protein			6	8	9	31	20	12	14	10	70	25	313	176	74	41	24	1

ACC_08434	arginine-glutamic acid dipeptide repeats protein-like		KOG3554	1	1	0	1	1	0	0	2	1	0	1	1	11	11	4	0
ACC_08435	LOW QUALITY PROTEIN		KOG1792	558	184	158	1036	699	110	107	223	586	425	335	440	350	152	107	99
ACC_08436	guanine nucleotide exchange factor DBS-like			299	250	273	892	721	40	87	116	167	120	205	218	131	165	136	94
ACC_08437	conserved hypothetical protein			33	23	11	24	21	2	3	7	98	67	37	178	27	18	2	1
ACC_08438	alpha-glucosidase isozyme I		KOG0471	11	9	61	91	52	2	19	1	97	47	176	146	0	1	0	1
ACC_08439	conserved hypothetical protein			90	104	134	324	193	33	47	60	192	98	126	167	100	124	56	28
ACC_08440	solute carrier family 35 member G1-like isoform 1		KOG4510	187	72	66	197	224	2	10	12	194	94	60	124	134	198	14	0
ACC_08441	conserved hypothetical protein		KOG2062	210	79	86	182	60	17	24	45	111	77	40	20	8	23	82	47
ACC_08442	serine/threonine-protein kinase PAK 1 isoform 1	K04409	KOG0578	347	276	260	506	234	66	106	115	453	510	178	203	354	407	742	370
ACC_08443	LOW QUALITY PROTEIN	K14567	KOG0788	2093	1173	1265	1399	1577	216	551	553	1531	1150	840	750	1567	1992	1480	940
ACC_08444	ras-related protein Rab-35-like	K07876	KOG0079	129	85	103	107	73	10	19	12	168	114	22	178	173	194	48	17
ACC_08445	f-box/LRR-repeat protein 14	K10280	KOG4341	68	29	37	144	74	24	22	36	145	239	31	11	7	10	82	52
ACC_08446	zinc finger protein Noc-like		KOG1126	268	216	328	262	108	44	78	58	394	535	52	115	82	62	95	48
ACC_08447	probable RNA-binding protein 18-like			112	91	87	147	86	23	36	47	98	37	73	115	127	169	91	81
ACC_08448	conserved hypothetical protein		KOG3535	570	577	538	389	115	25	33	47	485	377	88	34	3	13	13	4
ACC_08449	forkhead box protein D3-A-like	K09397	KOG3562	169	77	92	176	22	4	6	16	25	24	0	0	1	0	2	2
ACC_08450	ATP-binding cassette sub-family B member 8, mito	K05655	KOG0058	246	194	188	296	313	28	53	53	502	228	170	375	304	516	55	25
ACC_08451	conserved hypothetical protein			20	2	5	11	1	0	0	1	4	1	1	0	1	1	2	4
ACC_08452	patj homolog	K06095	KOG3209	235	145	138	536	138	26	29	67	293	370	146	152	101	66	65	25
ACC_08453	zinc finger protein 567-like		KOG3623	223	188	215	236	255	19	61	76	394	238	207	322	159	138	21	2
ACC_08454	transmembrane protein 173-like			47	21	40	63	61	2	2	9	28	20	28	71	95	61	8	1
ACC_08455	luciferin 4-monooxygenase-like		KOG1176	110	356	258	299	180	196	281	425	7027	3590	365	156	369	1560	1280	63
ACC_08456	calcium and integrin-binding family member 3-like		KOG0038	0	1	4	6	2	0	1	2	60	16	1	2	8	8	9	0
ACC_08457	myosin heavy chain, non-muscle	K10352	KOG0161	2633	1671	1327	1465	1511	881	1898	1904	3445	2981	1533	1093	1709	1379	3574	2202
ACC_08458	trafficking kinesin-binding protein milt			28	17	15	13	7	6	6	7	33	46	11	23	20	19	15	7
ACC_08459	conserved hypothetical protein			216	94	85	132	89	1	10	13	142	81	2	53	415	155	12	11
ACC_08460	LOW QUALITY PROTEIN		KOG0986	367	242	241	469	226	71	69	107	331	150	156	84	103	137	191	62
ACC_08461	conserved hypothetical protein		KOG2893	322	228	174	159	70	37	32	41	389	445	67	246	303	255	149	61
ACC_08462	conserved hypothetical protein		KOG4232	315	134	140	421	219	53	106	76	2213	1351	126	277	4435	4276	1364	903
ACC_08463	conserved hypothetical protein			309	187	162	330	254	41	77	69	524	366	187	223	315	328	105	45
ACC_08464	regulator of chromosome condensation	K11493	KOG0783	145	92	88	122	80	22	22	23	170	90	82	59	481	886	257	121
ACC_08465	sodium-coupled monocarboxylate transporter 2-like		KOG2349	3	4	1	20	8	0	1	3	17	1	4	0	940	613	57	21
ACC_08466	orthodenticle 1			6	6	3	2	1	0	0	0	2	3	0	0	1	1	1	2
ACC_08467	orthodenticle 1		KOG2251	33	31	37	22	6	2	1	1	18	12	0	0	3	3	15	6
ACC_08468	transmembrane protein 164-like isoform 1			175	150	122	94	167	50	36	70	342	197	98	259	151	67	4	0
ACC_08469	neither inactivation nor afterpotential protein C isc	K08834	KOG4229	112	84	83	89	59	204	300	322	1116	732	9	67	8	9	16	10
ACC_08470	conserved hypothetical protein			2	4	4	6	3	13	3	14	3	24	65	242	1	9	8	1
ACC_08471	conserved hypothetical protein	K10749		454	329	396	545	361	153	256	308	661	244	281	402	514	911	1458	888
ACC_08472	oxysterol-binding protein-related protein 8-like			47	47	88	16	11	2	4	3	49	58	17	102	17	11	2	1
ACC_08473	ATP-binding cassette sub-family A member 2-like	K05648	KOG0059	1612	1144	878	1198	843	85	126	195	1869	1521	1029	967	701	294	38	15
ACC_08474	tektin-1		KOG2685	44	37	45	51	37	2	7	5	22	38	14	23	43	36	29	8
ACC_08475	protein kinase C-binding protein NELL1-like		KOG1214	206	88	129	159	43	3	4	11	156	189	38	43	11	12	25	12
ACC_08476	conserved hypothetical protein			43	26	21	22	28	5	8	9	41	19	19	31	20	9	8	6
ACC_08477	discoidin domain-containing receptor 2-like		KOG1094	124	64	78	129	32	3	4	13	35	23	11	1	2	4	32	15
ACC_08478	tubulin alpha chain-like		KOG1376	63	33	42	247	67	38	46	72	415	3142	19	16	225	132	1540	153
ACC_08479	LOW QUALITY PROTEIN		KOG0161	473	193	232	344	186	21	25	41	490	146	125	159	43	85	84	28
ACC_08480	60S ribosomal protein L4 isoform 1	K02930	KOG1475	2215	1538	811	2854	1464	492	917	850	2140	2104	1764	3461	11999	15902	7053	2400
ACC_08481	neuronal acetylcholine receptor subunit alpha-7 isoform 1		KOG3646	227	29	33	193	54	3	2	11	33	17	2	1	2	5	10	11
ACC_08482	disintegrin and metalloproteinase domain-containing protein 10-like			47	8	13	33	5	0	1	9	44	44	2	6	1	3	6	4
ACC_08483	disintegrin and metalloproteinase domain-containi	K06704	KOG3658	671	221	245	836	139	63	166	275	479	324	96	50	14	37	217	210
ACC_08484	conserved hypothetical protein			36	32	29	8	14	2	2	2	31	44	2	3	2	0	0	0
ACC_08485	conserved hypothetical protein			90	161	226	55	28	88	75	128	13	29	16	9	58	363	245	128
ACC_08486	putative GTP-binding protein 6-like		KOG0410	332	200	197	390	301	50	101	85	254	217	226	285	482	667	339	209
ACC_08487	plexin-A4		KOG3611	53	31	24	115	20	5	5	8	31	96	8	4	5	5	32	44
ACC_08488	plexin-A4	K06820	KOG3610	426	300	235	549	115	25	34	48	495	609	62	67	154	180	193	51
ACC_08489	conserved hypothetical protein		KOG4735	148	46	89	488	129	18	15	43	130	51	55	81	45	68	293	252
ACC_08490	conserved hypothetical protein		KOG4320	468	279	356	444	135	30	26	47	863	521	79	244	82	76	109	43

ACC_08491	mucolipin-3 isoform 2	K05329	KOG3733	188	84	109	142	204	12	21	23	488	364	79	387	755	1002	59	2
ACC_08492	conserved hypothetical protein			10	4	3	25	2	0	1	2	35	17	178	887	206	188	66	50
ACC_08493	cytosolic carboxypeptidase 1-like		KOG3641	128	49	49	219	68	6	21	18	192	44	10	7	17	7	15	10
ACC_08494	tRNA (guanine-N(7)-)-methyltransferase subunit W	K15443	KOG3914	69	43	45	55	111	2	3	1	63	65	54	142	115	147	6	1
ACC_08495	TIMELESS-interacting protein-like	K10904	KOG3004	354	299	215	560	629	38	99	118	263	268	344	508	664	739	282	138
ACC_08496	calcium-activated potassium channel slowpoke-like		KOG1420	450	244	263	351	136	35	58	90	215	296	113	48	1	7	46	57
ACC_08497	calcium-activated potassium channel slowpoke-like isoform		KOG1420	53	37	27	68	28	5	8	8	57	41	16	11	1	0	3	2
ACC_08498	calcium-activated potassium channel slowpoke-like	K04936	KOG1420	1214	930	853	1257	500	113	188	239	1041	737	343	224	26	32	97	72
ACC_08499	conserved hypothetical protein		KOG4221	99	33	39	80	18	4	10	8	40	28	6	2	1	1	24	13
ACC_08500	conserved hypothetical protein		KOG2739	1038	992	716	663	503	174	244	324	1674	1328	396	975	2044	1821	916	655
ACC_08501	UPF0193 protein EVG1-like			1	0	0	1	0	0	0	0	2	0	1	0	0	0	1	0
ACC_08502	conserved hypothetical protein			1360	1127	989	1057	424	197	317	357	2115	1391	550	549	427	679	937	880
ACC_08503	dual specificity mitogen-activated protein kinase ki	K04368	KOG0581	46	34	20	31	24	1	2	2	35	23	7	26	23	29	8	2
ACC_08504	conserved hypothetical protein	K15682	KOG0312	199	267	330	595	117	42	51	51	144	206	88	67	45	58	214	124
ACC_08505	ras-related protein Rab-23 isoform 2	K06234	KOG4252	29	22	14	32	21	11	18	14	161	77	47	234	67	60	31	18
ACC_08506	conserved hypothetical protein			506	331	327	665	158	43	48	95	531	525	118	41	94	149	397	102
ACC_08507	LOW QUALITY PROTEIN		KOG3548	643	428	418	680	658	53	131	153	612	580	350	289	604	722	328	160
ACC_08508	dnaJ homolog subfamily C member 9-like			22	21	12	59	54	5	9	5	180	55	54	56	31	75	32	15
ACC_08509	sonic hedgehog protein A-like		KOG3638	1	0	0	0	1	1	2	1	9	3	0	1	1	1	1	3
ACC_08510	conserved hypothetical protein			80	52	20	56	29	14	12	16	444	225	19	10	5	3	26	10
ACC_08511	conserved hypothetical protein		KOG0671	348	224	241	617	290	37	74	85	865	400	134	196	264	334	270	120
ACC_08512	ras suppressor protein 1		KOG0617	172	94	69	120	119	11	25	31	285	116	93	289	396	479	63	17
ACC_08513	conserved hypothetical protein		KOG0030	245	37	22	167	53	26	27	26	1687	738	107	302	503	271	366	153
ACC_08514	triple functional domain protein		KOG0032	201	122	132	371	240	14	34	52	233	115	99	51	72	83	30	16
ACC_08515	echinoderm microtubule-associated protein-like 1-like		KOG2106	310	147	129	489	300	53	75	105	911	483	377	647	237	201	88	25
ACC_08516	kinesin 8	K10401	KOG0242	21	35	48	28	24	2	12	5	61	31	30	22	258	475	230	112
ACC_08517	conserved hypothetical protein		KOG0225	5	1	5	10	9	1	2	2	28	340	38	61	20	16	12	6
ACC_08518	conserved hypothetical protein			276	204	262	513	312	46	70	72	738	793	222	438	151	164	108	54
ACC_08519	conserved hypothetical protein		KOG1677	498	189	255	194	89	68	171	162	402	888	114	81	93	155	1016	480
ACC_08520	conserved hypothetical protein			153	102	127	84	29	9	2	8	50	76	21	30	8	2	2	1
ACC_08521	conserved hypothetical protein		KOG0792	177	91	80	101	20	12	23	28	425	270	62	102	39	53	35	10
ACC_08522	probable cytochrome P450 6a14		KOG0158	3	0	8	11	6	0	0	1	44	155	0	4	9	244	7	0
ACC_08523	conserved hypothetical protein			8	5	2	13	3	0	2	1	7	3	5	0	1	16	12	7
ACC_08524	bone morphogenetic protein 5-like		KOG3900	2	2	1	9	2	2	1	1	9	4	4	0	0	2	16	8
ACC_08525	conserved hypothetical protein		KOG3605	239	107	98	228	141	21	24	44	163	211	52	107	123	102	39	14
ACC_08526	conserved hypothetical protein			519	474	460	303	124	62	100	137	347	400	50	50	131	177	327	231
ACC_08527	papilin-like		KOG4597	143	54	60	116	47	6	3	7	54	42	25	44	32	44	34	20
ACC_08528	myosin-XVIIIa		KOG4217	108	92	87	95	29	14	18	30	159	179	35	52	51	67	45	40
ACC_08529	borealin-like			99	79	59	191	104	38	60	71	140	83	102	131	226	440	923	552
ACC_08530	broad-complex	K02174	KOG4441	559	355	452	601	130	18	30	57	171	134	16	12	107	26	57	205
ACC_08531	cAMP-specific 3',5'-cyclic phosphodiesterase			37	27	21	94	9	2	5	1	157	84	14	17	7	4	5	13
ACC_08532	conserved hypothetical protein	K06971		531	243	350	807	215	84	97	203	854	634	397	184	107	105	319	164
ACC_08533	conserved hypothetical protein			6	9	9	12	8	2	1	4	13	141	2	73	2	8	3	3
ACC_08534	n-acetyllactosaminide beta-1,3-N-acetylglucosaminyltransfe		KOG3765	16	10	7	11	3	1	0	2	9	54	4	9	0	0	7	4
ACC_08535	conserved hypothetical protein			1	0	0	1	0	0	0	0	1	6	0	0	5	1	0	2
ACC_08536	forkhead box protein D3-A-like	K09397	KOG3563	156	61	77	166	43	4	14	10	19	18	0	0	0	0	6	1
ACC_08537	conserved hypothetical protein	K13804	KOG3209	1889	1477	1743	2412	694	321	625	870	2179	1215	530	235	40	29	106	63
ACC_08538	coatomer subunit beta'-like		KOG0276	604	424	370	887	718	97	111	174	860	942	414	1453	1737	2206	248	75
ACC_08539	dolichyl-diphosphooligosaccharide--protein glycosy	K12670	KOG2754	403	348	196	471	448	58	62	84	503	370	329	590	2126	2338	110	57
ACC_08540	conserved hypothetical protein			29	17	19	37	14	4	2	6	27	22	4	12	4	3	10	1
ACC_08541	venom allergen 5-like		KOG3017	4	2	7	20	9	2	2	4	24	3	8	2	2	2	2	0
ACC_08542	glutamate receptor-interacting protein 1		KOG3209	61	45	49	100	79	19	21	43	258	82	56	59	64	83	37	28
ACC_08543	cytoplasmic FMR1-interacting protein isoform 1	K05749	KOG3534	301	194	202	303	299	29	48	31	446	288	160	192	374	426	38	5
ACC_08544	translation initiation factor eIF-2B subunit delta-like	K03680	KOG1467	759	518	559	538	537	104	181	223	1322	648	481	1139	1276	1526	588	323
ACC_08545	potassium/sodium hyperpolarization-activated cyclic nucleo		KOG0498	0	0	0	0	0	0	0	0	0	0	0	0	0	0	0	0
ACC_08546	conserved hypothetical protein		KOG4483	491	540	448	762	491	146	218	273	1208	1020	417	732	625	724	258	128
ACC_08547	conserved hypothetical protein			96	51	47	100	111	1	7	8	110	82	47	205	174	183	12	4

ACC_08548	x-ray repair cross-complementing protein 5-like	K10885	KOG2326	218	157	125	188	258	12	29	35	252	167	121	334	662	568	27	11
ACC_08549	huntingtin-interacting protein K-like			367	77	106	212	193	91	150	117	124	42	162	30	65	98	201	58
ACC_08550	anaphase-promoting complex subunit CDC26-like	K03359		125	161	223	260	111	18	30	20	90	55	82	84	54	79	44	31
ACC_08551	conserved hypothetical protein			160	45	43	135	57	21	32	30	131	132	88	73	55	65	80	46
ACC_08552	conserved hypothetical protein			0	0	0	0	0	0	0	0	0	0	0	0	0	0	0	0
ACC_08553	growth hormone secretagogue receptor type 1		KOG4219	12	11	8	33	16	2	1	1	11	29	2	0	2	7	3	6
ACC_08554	conserved hypothetical protein		KOG2462	556	491	615	1209	166	62	58	157	117	214	85	14	1	2	77	43
ACC_08555	prestin-like		KOG0236	60	73	44	28	20	34	27	41	25	7	2	2	85	150	9	2
ACC_08556	conserved hypothetical protein	K12946		0	0	0	2	2	0	0	0	0	0	0	0	1	246	6	38
ACC_08557	transcriptional repressor CTCFL-like		KOG2462	834	591	612	743	419	97	163	238	1376	1159	244	495	838	758	280	165
ACC_08558	conserved hypothetical protein			11	1	3	22	3	0	1	2	9	11	6	4	0	0	4	1
ACC_08559	dehydrogenase/reductase SDR family member 11-like		KOG1205	7	6	5	8	2	2	0	0	162	27	1	3	4209	488	0	0
ACC_08560	MOXD1 homolog 2-like		KOG3568	38	6	7	17	9	2	7	11	18	11	6	3	1	2	106	39
ACC_08561	peptidyl-prolyl cis-trans isomerase FKBP14-like		KOG0549	11	6	6	18	3	1	1	1	20	5	6	2	82	93	45	9
ACC_08562	peptidyl-prolyl cis-trans isomerase FKBP14-like		KOG0034	27	20	13	43	13	1	2	1	69	15	5	17	515	390	129	41
ACC_08563	conserved hypothetical protein			4	1	1	1	0	0	0	0	1	0	0	0	0	0	1	1
ACC_08564	neural/ectodermal development factor IMP-L2		KOG3513	153	231	333	1468	920	11	11	18	222	182	58	383	82	117	30	6
ACC_08565	pancreatic triacylglycerol lipase-like			2	3	265	183	81	7	4	6	449	706	150	1	12	1	0	0
ACC_08566	potassium channel subfamily K member 18-like		KOG1418	6	0	2	2	6	1	5	5	23	20	0	0	6	10	38	42
ACC_08567	TAR DNA-binding protein 43		KOG0149	849	616	621	768	383	109	176	216	874	572	305	208	220	298	465	170
ACC_08568	LOW QUALITY PROTEIN	K12460	KOG4177	658	667	658	966	705	68	97	87	2199	1645	224	314	795	761	129	30
ACC_08569	conserved oligomeric Golgi complex subunit 7		KOG4182	200	115	119	227	199	7	19	23	242	239	89	250	504	425	72	16
ACC_08570	citron Rho-interacting kinase-like isoform 2	K16308	KOG0976	400	251	198	357	300	78	228	199	550	576	222	163	670	849	953	643
ACC_08571	conserved hypothetical protein		KOG2205	1201	606	718	1880	374	294	326	609	469	312	78	52	313	184	767	254
ACC_08572	conserved hypothetical protein		KOG4169	60	32	24	52	17	10	7	14	143	56	2	35	15	5	3	0
ACC_08573	15-hydroxyprostaglandin dehydrogenase		KOG4169	111	63	38	98	79	66	89	89	3556	3641	1	2	5886	1414	139	233
ACC_08574	15-hydroxyprostaglandin dehydrogenase		KOG4169	145	146	227	85	60	185	300	278	1462	775	26	29	2245	1465	194	65
ACC_08575	conserved hypothetical protein		KOG2746	591	550	591	427	131	72	116	116	866	889	138	129	173	244	244	139
ACC_08576	tricalbin-1-like		KOG1030	143	89	153	255	137	5	10	25	170	110	221	180	18	199	30	15
ACC_08577	cAMP-dependent protein kinase catalytic subunit i	K04345	KOG0616	743	560	464	1266	442	159	294	389	515	315	137	32	19	49	187	188
ACC_08578	conserved hypothetical protein			74	62	90	166	138	1	7	6	43	40	135	157	139	142	17	5
ACC_08579	conserved hypothetical protein	K07874	KOG2744	6650	4272	3752	5447	5679	1484	3130	3752	5275	3809	2729	1513	1794	2173	4346	2560
ACC_08580	presenilin homolog	K04505	KOG2736	391	241	267	399	324	67	70	95	833	402	311	690	801	612	109	50
ACC_08581	tyramine receptor	K04153	KOG4220	102	59	70	128	18	1	6	7	55	48	3	4	3	4	4	2
ACC_08582	glutamate receptor, ionotropic kainate 1-like	K05313	KOG1052	14	14	18	27	18	5	10	6	38	14	49	10	12	10	9	2
ACC_08583	glutamate receptor 1-like, partial		KOG1052	0	2	0	0	0	0	0	0	10	23	166	545	28	8	0	0
ACC_08584	UPF0534 protein CG15027-like			202	148	116	202	235	30	71	119	222	132	140	217	308	514	183	152
ACC_08585	conserved hypothetical protein	K09341	KOG0492	3	1	4	8	1	1	1	1	17	88	2	1	44	35	118	73
ACC_08586	serine protease 31		KOG3627	8	1	2	0	0	0	0	0	0	4	4	11	0	1	11	0
ACC_08587	e3 ubiquitin-protein ligase CBL-B-like	K04707	KOG1785	140	97	154	128	57	7	6	2	371	151	79	275	210	166	37	10
ACC_08588	conserved hypothetical protein		KOG0307	714	655	569	706	307	32	54	60	491	411	176	199	247	307	101	21
ACC_08589	Ras-like GTP-binding protein Rho1 isoform 1	K04513	KOG0393	1272	811	677	867	522	246	371	420	1941	1725	385	2238	1806	1707	1543	917
ACC_08590	cysteine-rich protein 1-like		KOG1700	61	114	69	160	64	92	157	256	1939	787	501	1259	159	84	121	117
ACC_08591	serine/threonine-protein kinase MARK2 isoform 1	K08798	KOG0586	502	237	206	472	185	101	132	145	1130	1030	180	294	480	504	176	55
ACC_08592	conserved hypothetical protein		KOG3960	84	46	63	84	45	4	12	13	281	125	37	6	112	107	110	46
ACC_08593	vesicular glutamate transporter 2-like		KOG2532	287	197	257	350	204	183	267	245	5246	1109	317	190	2940	5614	5079	924
ACC_08594	paramyosin, long form-like		KOG0161	400	98	26	240	67	59	79	68	12192	6109	200	455	873	593	166	177
ACC_08595	conserved hypothetical protein		KOG3576	380	268	423	684	113	19	23	32	189	171	73	71	6	7	57	21
ACC_08596	conserved hypothetical protein	K12230	KOG0818	20	17	19	22	8	0	1	2	73	78	11	29	27	18	25	7
ACC_08597	protein dopey-1 homolog			20	9	5	14	20	1	2	4	20	12	7	8	8	14	0	1
ACC_08598	conserved hypothetical protein			1117	214	166	290	87	39	40	65	817	206	57	261	4	5	1	5
ACC_08599	conserved hypothetical protein			56	25	27	33	6	3	5	11	39	60	8	9	5	15	11	5
ACC_08600	feline leukemia virus subgroup C receptor-related p	K08220	KOG2563	11	3	4	4	4	1	2	0	7	6	0	0	159	174	48	2
ACC_08601	conserved hypothetical protein			3	2	5	1	0	0	0	0	2	5	0	0	104	527	52	409
ACC_08602	glutamate-rich WD repeat-containing protein 1-like	K14848	KOG0302	357	206	189	439	431	44	58	91	304	288	331	394	997	1039	327	246
ACC_08603	conserved hypothetical protein			4	0	0	2	2	1	0	2	21	80	0	3	12	21	50	102
ACC_08604	conserved hypothetical protein			276	171	248	360	159	56	92	146	33	214	43	19	22	12	253	313

ACC_08605	conserved hypothetical protein			535	145	199	342	117	32	78	92	1872	321	179	921	174	209	234	228
ACC_08606	nephrin-like		KOG3513	171	73	94	116	19	20	17	25	65	61	16	3	3	3	11	6
ACC_08607	neurochondrin homolog			59	36	39	29	34	5	4	8	91	83	30	65	43	51	3	0
ACC_08608	synaptosomal-associated protein 25-like isoform 1		KOG3065	846	533	740	1120	540	124	197	247	608	411	102	92	35	32	113	87
ACC_08609	synaptosomal-associated protein 25-like	K08508	KOG3065	3417	4123	5742	4933	1576	274	320	469	1885	1165	312	234	128	177	312	173
ACC_08610	conserved hypothetical protein		KOG0996	647	173	237	345	82	7	5	17	227	210	29	34	2	6	13	8
ACC_08611	E3 ubiquitin-protein ligase RFWD3	K15691	KOG1645	233	158	168	443	355	32	49	47	402	295	167	370	272	263	46	24
ACC_08612	PHD and ring finger domains 1		KOG0825	10072	6391	6362	9270	8127	1657	3729	3983	6153	4390	3117	1019	2848	4947	10849	6591
ACC_08613	transcription factor HNF-4 homolog		KOG4215	26	19	23	41	34	5	3	7	64	42	12	56	85	36	19	9
ACC_08614	conserved hypothetical protein		KOG0527	48	13	30	52	14	5	8	12	65	105	19	48	8	11	7	4
ACC_08615	LYR motif-containing protein 5-like			14	6	18	47	16	4	3	12	8	8	38	54	79	25	6	15
ACC_08616	LOW QUALITY PROTEIN	K01887	KOG4426	480	305	250	361	319	42	112	123	383	374	158	367	1432	2219	445	332
ACC_08617	leucyl-tRNA synthetase, cytoplasmic-like isoform 1	K01869	KOG0437	1178	612	461	907	989	123	170	286	966	1085	443	727	2059	1969	435	234
ACC_08618	LIM/homeobox protein Awh	K09375	KOG4577	8	1	7	12	11	0	0	0	3	1	4	0	4	8	3	4
ACC_08619	conserved hypothetical protein		KOG0226	207	197	242	316	166	54	90	100	299	112	209	182	149	279	281	253
ACC_08620	CUGBP Elav-like family member 2-like		KOG0144	46	23	23	35	6	8	19	19	33	41	20	7	8	10	61	28
ACC_08621	CUGBP Elav-like family member 2-like		KOG0144	289	190	186	182	85	97	217	270	276	478	251	138	38	45	363	110
ACC_08622	alpha-glucosidase isozyme II	K01187	KOG0471	47	38	26	74	19	12	19	11	119	761	10	1	6515	3241	728	62
ACC_08623	disks large 1 tumor suppressor protein-like	K12076	KOG0708	2377	924	1041	2124	769	171	344	355	1535	772	480	463	108	175	732	282
ACC_08624	protein ST7 homolog isoform 1		KOG3807	136	79	83	126	91	3	8	11	120	112	26	119	174	187	14	3
ACC_08625	probable mitochondrial import inner membrane translocase		KOG1652	0	0	0	0	0	0	0	0	0	0	0	0	0	0	0	0
ACC_08626	40S ribosomal protein S6-like	K02991	KOG1646	4556	3985	2230	4949	2537	1218	2050	2435	4296	3125	4146	5520	16118	22265	15270	7233
ACC_08627	conserved hypothetical protein		KOG1883	43	39	59	59	10	2	2	3	14	18	1	3	6	4	10	12
ACC_08628	lipase 3-like		KOG2624	9	11	15	45	31	2	3	4	5	16	11	8	30	16	0	1
ACC_08629	conserved hypothetical protein		KOG3598	259	1462	226	151	57	472	352	757	5672	4340	2032	4514	673	739	308	181
ACC_08630	heparan sulfate 2-O-sulfotransferase pipe-like		KOG3922	89	35	28	69	40	12	22	30	49	41	19	26	8	33	69	31
ACC_08631	multiple coagulation factor deficiency protein 2 homolog		KOG4065	58	58	65	199	98	3	4	4	26	19	29	39	21	14	5	1
ACC_08632	immunoglobulin superfamily member 10-like		KOG4237	191	210	167	192	172	14	26	25	146	94	87	202	255	146	3	3
ACC_08633	methylcrotonoyl-CoA carboxylase beta chain, mito	K01969	KOG0540	15	12	11	28	12	6	7	11	130	151	26	25	428	323	116	75
ACC_08634	conserved hypothetical protein			0	0	0	4	0	0	0	0	1	5	3	5	14	31	57	18
ACC_08635	conserved hypothetical protein			1	1	0	1	0	0	0	0	2	1	1	0	20	665	596	91
ACC_08636	conserved hypothetical protein			40	22	24	32	13	9	12	12	41	7	61	5	4	18	31	27
ACC_08637	conserved hypothetical protein		KOG0670	117	60	58	254	32	6	1	5	45	107	18	234	384	255	401	384
ACC_08638	phospholipase D6-like			175	77	127	594	307	34	56	63	32	41	5	5	11	30	95	64
ACC_08639	blue sensitive opsin	K04255	KOG4219	41	110	144	677	268	348	408	732	1291	703	1	18	2	2	4	1
ACC_08640	tyrosine-protein phosphatase Lar-like, partial	K05695	KOG3513	1584	819	803	1285	247	67	122	196	584	601	232	92	70	99	628	349
ACC_08641	conserved hypothetical protein		KOG4006	1305	922	1266	1004	173	109	102	192	972	1210	106	170	44	61	134	40
ACC_08642	conserved hypothetical protein		KOG4564	85	87	96	199	159	16	36	36	36	14	15	37	26	40	43	26
ACC_08643	diuretic hormone receptor-like	K04578	KOG4564	64	59	70	195	76	9	10	10	70	35	8	30	31	21	12	3
ACC_08644	actin-binding protein IPP-like	K13956	KOG4441	75	33	36	66	76	8	6	8	213	181	28	125	113	79	13	3
ACC_08645	conserved hypothetical protein		KOG3777	1346	678	597	1048	1090	226	462	508	962	584	679	662	624	763	605	272
ACC_08646	leucine-rich repeat-containing protein 57-like		KOG0532	76	74	43	195	164	7	18	11	399	134	127	387	192	155	25	9
ACC_08647	conserved hypothetical protein			131	97	124	106	141	4	5	5	39	54	105	76	55	61	4	1
ACC_08648	protein SMG9-like isoform 1		KOG4181	128	115	115	163	195	7	18	26	138	150	156	249	374	331	58	20
ACC_08649	GPI transamidase component PIG-S-like	K05291	KOG2459	302	199	242	450	375	77	102	106	617	344	239	486	744	491	234	77
ACC_08650	conserved hypothetical protein	K14615	KOG4719	1043	320	334	990	581	47	42	62	476	251	219	528	12	61	14	4
ACC_08651	carbonic anhydrase 2-like		KOG0382	17	16	31	62	23	5	9	8	20	30	34	25	7	8	27	12
ACC_08652	nuclear hormone receptor FTZ-F1-like			132	58	67	70	22	7	17	17	133	128	25	46	39	25	26	28
ACC_08653	pyridoxal-dependent decarboxylase domain-containing prot		KOG0630	470	339	240	553	383	61	63	81	560	544	183	381	664	555	80	28
ACC_08654	conserved hypothetical protein			16	13	25	33	5	0	0	7	10	25	6	7	2	3	18	4
ACC_08655	conserved hypothetical protein	K04459	KOG1716	68	21	30	98	85	39	75	46	625	233	72	110	51	71	120	82
ACC_08656	protein tipE			106	26	12	21	4	0	0	3	11	20	19	8	0	1	0	0
ACC_08657	conserved hypothetical protein			15	11	10	47	22	7	12	16	86	299	105	86	40	154	20	5
ACC_08658	conserved hypothetical protein			439	187	197	607	286	51	71	78	176	195	116	216	240	262	100	67
ACC_08659	tachykinin-like peptides receptor 99D-like isoform	K04225	KOG4219	27	7	14	24	11	0	1	2	16	13	0	0	1	0	6	0
ACC_08660	50S ribosomal protein L1	K02863	KOG1569	396	294	335	545	507	45	84	80	759	271	365	572	1044	2111	236	126
ACC_08661	motor neuron and pancreas homeobox protein 1-like		KOG0488	0	0	0	0	0	0	0	0	0	0	0	0	1	1	2	1

ACC_08662	f-box only protein 9-like	K10295	KOG2997	300	210	190	292	242	55	79	97	419	457	96	293	573	323	95	22
ACC_08663	serine/threonine-protein kinase Doa-like	K08823	KOG0671	409	278	315	711	314	46	54	95	930	490	173	279	332	340	332	99
ACC_08664	myosin light chain alkali-like isoform 4		KOG0030	387	82	27	215	77	44	68	49	2772	894	139	375	974	578	556	288
ACC_08665	conserved hypothetical protein			5	0	2	1	2	0	0	0	2	3	0	0	13	9	5	1
ACC_08666	collagen alpha chain CG42342-like			527	258	336	258	89	13	35	45	57	37	11	16	2	17	231	128
ACC_08667	conserved hypothetical protein			64	23	14	32	16	11	13	17	592	545	28	17	56	75	90	191
ACC_08668	coproporphyrinogen-III oxidase-like	K00228	KOG1518	115	116	70	81	102	14	9	11	259	306	57	267	770	779	57	15
ACC_08669	conserved hypothetical protein		KOG2462	483	290	202	400	377	73	170	196	309	321	321	227	217	170	232	104
ACC_08670	conserved hypothetical protein		KOG4676	1900	1653	1804	1292	842	352	661	695	1326	1275	511	226	805	1020	2201	1397
ACC_08671	protein yippee-like 5-like		KOG3399	324	203	246	1236	490	72	69	118	694	227	321	459	139	130	62	25
ACC_08672	slit homolog 2 protein		KOG4194	51	14	27	36	7	5	5	10	18	26	8	5	14	7	53	37
ACC_08673	voltage-gated potassium channel subunit beta-1-like isoform		KOG1575	899	516	461	848	577	87	147	194	735	361	422	337	68	36	24	4
ACC_08674	sulfatase-modifying factor 1-like isoform 2	K13444		246	147	148	336	261	37	63	72	390	207	250	458	769	394	51	15
ACC_08675	conserved hypothetical protein			152	89	89	101	99	24	36	51	274	325	89	173	359	259	62	26
ACC_08676	conserved hypothetical protein	K07847	KOG0395	8805	6183	6551	6279	3232	578	1097	1305	4737	3038	716	467	1384	2594	1096	397
ACC_08677	rho guanine nucleotide exchange factor 7		KOG2070	525	466	607	644	515	49	57	62	1078	750	350	889	646	596	107	20
ACC_08678	conserved hypothetical protein		KOG4177	185	268	717	227	288	142	157	270	408	629	24	41	21	33	18	3
ACC_08679	dipeptidase 1-like	K01273	KOG4127	193	87	86	349	93	6	5	11	98	56	3	68	4	8	7	3
ACC_08680	conserved hypothetical protein			8	5	5	9	4	1	0	0	19	5	14	31	2	7	6	4
ACC_08681	sodium-independent sulfate anion transporter-like	K14708	KOG0236	3	3	7	3	3	1	1	0	19	64	45	128	38	37	2	0
ACC_08682	conserved hypothetical protein	K15617	KOG2181	281	234	214	322	92	57	63	115	215	231	86	36	54	61	163	49
ACC_08683	retinol dehydrogenase 11-like		KOG1208	83	56	43	39	60	5	4	8	251	115	161	1130	336	92	7	6
ACC_08684	endocuticle structural glycoprotein SgAbd-2-like			9	6	1	7	6	2	0	2	43	2	147	174	4443	1864	512	921
ACC_08685	organic cation transporter protein-like	K10106	KOG0255	458	419	373	865	442	45	48	70	618	356	128	244	66	83	49	15
ACC_08686	protein held out wings-like isoform 2	K14945	KOG1588	438	191	187	468	201	59	70	81	1293	1005	110	153	266	143	83	45
ACC_08687	laccase-4		KOG1263	24	19	9	24	3	1	3	7	15	38	0	17	1	6	49	76
ACC_08688	yjeF N-terminal domain-containing protein GH12525-like iso		KOG2585	54	34	38	65	91	4	9	9	67	55	72	178	164	209	15	3
ACC_08689	epithelial splicing regulatory protein 2	K05286	KOG1771	123	117	146	124	216	3	5	6	63	117	73	89	30	31	5	2
ACC_08690	epithelial splicing regulatory protein 2		KOG1365	99	93	85	94	44	19	21	26	347	353	74	90	233	174	107	25
ACC_08691	metabotropic glutamate receptor 7	K04608	KOG1056	58	35	33	79	36	0	4	5	44	43	0	0	3	2	1	2
ACC_08692	nucleolar complex protein 2 homolog	K14833	KOG2256	319	149	117	247	339	42	116	139	151	253	154	146	837	1047	948	630
ACC_08693	zinc finger SWIM domain-containing protein KIAA0913-like		KOG3615	649	612	625	632	234	104	100	123	1283	1336	313	238	294	296	307	124
ACC_08694	conserved hypothetical protein			15	15	21	12	24	2	1	2	27	20	80	86	117	190	10	1
ACC_08695	conserved hypothetical protein			1	1	0	1	1	1	0	0	4	1	0	1	1	1	0	2
ACC_08696	conserved hypothetical protein	K04348	KOG0375	94	46	48	45	37	6	20	20	88	67	21	9	9	11	63	81
ACC_08697	conserved hypothetical protein		KOG0375	78	37	40	67	18	9	13	19	32	35	16	0	1	5	43	22
ACC_08698	sestrin-1-like isoform 1	K10141	KOG3746	444	333	314	399	124	133	86	139	1445	914	99	219	491	524	381	50
ACC_08699	conserved hypothetical protein		KOG2187	1227	915	734	1077	1049	188	446	471	977	846	618	420	795	960	800	385
ACC_08700	28S ribosomal protein S10, mitochondrial-like	K02946	KOG3321	121	78	87	98	137	4	15	9	107	94	150	286	392	517	58	46
ACC_08701	transcription factor 25-like isoform 1		KOG2422	463	281	262	450	498	88	194	226	677	435	411	563	480	436	352	269
ACC_08702	disintegrin and metalloproteinase domain-containi	K06704	KOG3658	45	32	35	21	10	2	4	3	75	95	9	22	20	20	10	6
ACC_08703	OV-16 antigen-like		KOG3346	51	40	24	57	54	13	10	10	155	115	98	765	1739	831	86	157
ACC_08704	conserved hypothetical protein	K15297		1469	886	1030	1815	315	78	94	162	482	217	93	15	2	6	41	29
ACC_08705	unzipped precursor			7260	5029	6112	12680	8032	625	995	1715	3474	2393	1776	4116	57	98	264	119
ACC_08706	iron-sulfur cluster assembly enzyme ISCU, mitocho	K04488	KOG3361	859	778	1051	3027	2892	112	121	221	2493	799	1388	2556	993	830	74	43
ACC_08707	monocarboxylate transporter 12-like		KOG2504	485	261	371	760	497	47	73	97	1055	843	204	644	619	930	298	51
ACC_08708	conserved hypothetical protein		KOG4286	133	112	152	326	111	18	21	35	174	116	90	160	52	39	111	54
ACC_08709	conserved hypothetical protein	K00698	KOG2571	3	1	0	0	0	5	3	1	62	9	0	40	386	150	15	17
ACC_08710	conserved hypothetical protein			121	70	68	85	65	6	7	4	66	35	7	17	9	16	2	1
ACC_08711	troponin C type IIb		KOG0027	50	11	2	7	5	5	14	6	39	78	11	125	292	137	106	151
ACC_08712	conserved hypothetical protein		KOG0696	272	134	157	295	134	19	29	64	281	274	121	55	130	96	196	148
ACC_08713	conserved hypothetical protein			20	7	8	16	3	1	2	1	27	27	2	4	11	8	23	13
ACC_08714	aminopeptidase N-like		KOG1046	5	3	1	1	2	0	0	0	15	2	1	0	432	670	125	5
ACC_08715	aminopeptidase N-like		KOG1046	1	2	0	10	3	0	2	2	47	3	1	4	14088	22450	3322	129
ACC_08716	conserved hypothetical protein		KOG1046	11	11	6	2	3	9	15	0	52	11	1	3	6953	13079	3136	93
ACC_08717	organic cation transporter protein-like		KOG0255	19	13	23	55	15	11	19	24	192	485	8	41	79	57	77	8
ACC_08718	chitotriosidase-1-like		KOG2806	25	14	13	18	8	1	0	2	10	4	1	0	13	54	1	1

ACC_08719	SET and MYND domain-containing protein 4-like		KOG0553	86	35	38	73	69	5	11	18	21	15	0	4	0	0	14	2
ACC_08720	general transcription factor IIE subunit 2-like	K03137	KOG3095	64	50	42	53	76	4	18	19	49	38	56	110	84	87	25	24
ACC_08721	4-nitrophenylphosphatase-like	K01101	KOG2882	110	33	42	131	70	17	24	26	56	17	33	53	15	17	18	8
ACC_08722	conserved hypothetical protein			21	5	8	15	5	19	16	21	159	20	1	8	148	206	175	84
ACC_08723	conserved hypothetical protein			6	1	1	10	2	1	1	0	5	1	0	0	123	78	6	15
ACC_08724	take-out-like carrier protein precursor			212	178	28	474	239	45	52	56	581	1083	5818	5217	2067	1980	490	586
ACC_08725	troponin C type IIb		KOG0027	585	284	351	659	450	82	160	146	280	187	37	166	316	174	248	394
ACC_08726	conserved hypothetical protein			16	8	11	17	9	0	1	0	98	44	4	27	2	0	2	2
ACC_08727	protein FAM46A-like isoform 4			10	25	18	16	8	9	15	21	38	52	10	4	19	23	43	22
ACC_08728	conserved hypothetical protein		KOG3623	371	162	305	637	154	23	44	59	554	613	43	68	82	115	408	122
ACC_08729	organic cation transporter-like protein-like		KOG0255	7	4	6	8	5	1	3	5	118	208	302	592	151	128	10	4
ACC_08730	conserved hypothetical protein			330	225	257	419	236	26	58	37	748	1033	162	1365	1095	506	251	28
ACC_08731	conserved hypothetical protein			59	34	18	44	14	30	52	36	267	228	17	12	188	69	36	10
ACC_08732	globin-like		KOG3378	477	139	80	348	157	58	57	68	573	1142	86	318	239	158	115	88
ACC_08733	transmembrane protein 223-like			19	14	16	13	11	1	0	0	2	9	20	49	9	19	0	0
ACC_08734	conserved hypothetical protein			36	15	35	35	4	0	0	1	18	11	2	3	2	1	3	2
ACC_08735	tropomodulin isoform 1	K10370	KOG3735	431	202	251	393	291	36	45	78	1269	649	131	280	325	286	67	41
ACC_08736	conserved hypothetical protein		KOG4457	541	407	439	921	886	15	42	34	368	450	392	1085	1226	1333	128	91
ACC_08737	conserved hypothetical protein			611	396	322	245	414	91	285	246	330	123	422	554	308	561	582	398
ACC_08738	torsin-like protein		KOG2170	158	105	115	175	220	12	17	25	223	110	83	185	256	294	22	8
ACC_08739	fasciclin-1			251	55	58	83	42	8	11	4	182	117	14	21	21	11	14	7
ACC_08740	conserved hypothetical protein			2	1	0	5	1	0	3	6	24	30	2	2	0	1	1	0
ACC_08741	conserved hypothetical protein			11	14	10	30	30	0	6	2	7	97	30	29	5	4	3	1
ACC_08742	voltage-dependent calcium channel type A subunit alpha-1			7	1	2	4	2	0	0	1	2	3	0	0	0	0	0	0
ACC_08743	zinc finger protein 76-like		KOG2462	171	124	120	312	167	27	23	32	885	1038	122	246	822	440	164	37
ACC_08744	conserved hypothetical protein			113	58	51	93	40	8	16	21	87	52	9	9	2	5	7	6
ACC_08745	conserved hypothetical protein		KOG1045	34	25	37	22	13	4	0	3	57	58	12	38	47	44	2	1
ACC_08746	ubiquitin like	K13113	KOG3493	87	60	64	99	68	5	22	19	24	22	121	106	85	113	79	51
ACC_08747	formin-binding protein 1 homolog		KOG3565	393	230	253	264	124	33	37	63	940	570	89	301	321	305	111	48
ACC_08748	u7 snRNA-associated Sm-like protein LSm11-like			46	25	31	102	33	9	14	18	23	27	35	44	24	42	58	38
ACC_08749	transmembrane and TPR repeat-containing protein 3-like			13	5	7	13	2	0	1	0	5	4	0	2	0	0	0	0
ACC_08750	disintegrin and metalloproteinase domain-containi	K06704	KOG3658	70	56	27	21	15	4	7	6	127	185	11	43	37	22	15	18
ACC_08751	conserved hypothetical protein			355	249	282	438	282	86	195	196	567	157	256	147	245	344	397	293
ACC_08752	conserved hypothetical protein		KOG4575	24	5	15	22	7	4	6	2	164	109	55	129	48	77	50	16
ACC_08753	nicotinamide mononucleotide adenylyltransferase	K06210	KOG3199	163	102	70	117	83	10	13	15	304	404	64	376	755	469	71	20
ACC_08754	DNA polymerase alpha catalytic subunit-like	K02320	KOG0970	257	267	175	304	334	29	80	78	283	269	196	93	720	857	508	238
ACC_08755	conserved hypothetical protein			33	12	13	28	9	8	12	9	99	89	52	63	118	27	15	10
ACC_08756	conserved hypothetical protein		KOG4429	216	119	143	207	116	33	47	46	555	317	169	278	4397	1158	296	120
ACC_08757	nuclease-sensitive element-binding protein 1-like		KOG3070	3423	2131	1567	3535	777	924	1797	1913	3777	4282	1376	2376	4674	5037	54413	37017
ACC_08758	conserved hypothetical protein			6	19	5	12	5	5	13	14	63	63	19	72	19	28	26	13
ACC_08759	NADH dehydrogenase	K03953	KOG2865	1803	1052	1057	2188	2300	204	303	398	4371	1330	1137	3533	4086	4344	295	209
ACC_08760	glutamate--cysteine ligase catalytic subunit isoform	K11204	KOG3754	324	162	187	184	169	14	20	28	718	535	266	1395	1040	858	53	19
ACC_08761	protein kinase C-binding protein NELL1-like			326	234	220	270	58	8	19	20	115	195	74	77	37	20	60	25
ACC_08762	MOSC domain-containing protein 1, mitochondrial-like		KOG2362	110	83	96	307	146	33	49	46	184	79	11	18	233	328	88	78
ACC_08763	golgin subfamily A member 2-like		KOG0161	909	502	401	580	812	159	375	381	752	598	614	634	831	835	451	239
ACC_08764	conserved hypothetical protein			4	0	0	2	0	0	0	0	90	2	1	2	133	433	257	4
ACC_08765	conserved hypothetical protein			96	63	50	12	12	1	1	4	3	73	6	104	0	0	5	2
ACC_08766	70 kDa peptidyl-prolyl isomerase-like		KOG0543	327	234	197	214	225	22	71	75	258	158	131	246	209	208	38	21
ACC_08767	DC-STAMP domain-containing protein 1-like		KOG3726	2	0	2	6	5	2	1	1	28	33	1	8	11	8	9	5
ACC_08768	dnaJ homolog subfamily C member 13	K09533	KOG1789	324	279	206	234	126	20	45	63	497	846	69	174	391	279	47	20
ACC_08769	serine--pyruvate aminotransferase, mitochondrial-	K00830	KOG2862	7	4	6	9	16	7	6	3	278	733	0	2	2570	726	160	94
ACC_08770	DNA replication factor Cdt1	K10727	KOG4762	251	165	154	246	174	19	12	30	439	227	117	286	399	636	76	27
ACC_08771	slit homolog 1 protein-like		KOG4194	72	36	38	56	70	0	4	3	24	27	18	35	102	110	1	0
ACC_08772	histone H2B.3-like	K11252	KOG1744	83	22	9	88	65	9	18	27	111	86	54	111	151	150	136	78
ACC_08773	ATP-binding cassette sub-family G member 4-like		KOG0061	79	48	52	81	93	2	11	10	110	144	69	86	519	239	7	1
ACC_08774	cytochrome P450 6k1		KOG0158	6	5	8	18	22	5	3	5	1239	4082	16501	5633	76	22	2	4
ACC_08775	conserved hypothetical protein		KOG0994	4	6	5	14	6	0	1	0	5	11	0	3	3	1	1	4

ACC_08776	acyl-CoA Delta(11) desaturase-like, partial		KOG1600	1	1	0	1	1	1	3	0	7	22	104	1	34	4457	854	165
ACC_08777	conserved hypothetical protein			57	38	47	54	44	2	4	4	96	60	15	8	32	66	18	6
ACC_08778	conserved hypothetical protein		KOG1892	100	39	41	87	43	27	45	51	259	151	51	60	46	49	78	39
ACC_08779	conserved hypothetical protein			7	1	1	27	13	3	1	2	29	18	43	128	119	49	35	17
ACC_08780	conserved hypothetical protein		KOG3963	20	8	12	29	16	2	4	4	6	14	9	12	211	445	468	32
ACC_08781	conserved hypothetical protein			0	0	0	0	1	0	0	0	0	6	0	0	0	0	0	0
ACC_08782	breast cancer anti-estrogen resistance protein 1-lik	K16832	KOG0162	44	25	50	50	14	7	2	5	70	54	37	47	20	39	197	73
ACC_08783	conserved hypothetical protein	K14570	KOG2248	548	281	235	476	510	82	131	101	611	453	441	489	528	604	424	245
ACC_08784	peptidyl-prolyl cis-trans isomerase FKBP14-like		KOG0549	34	16	14	57	16	0	4	2	98	56	8	33	654	409	126	42
ACC_08785	peptidyl-prolyl cis-trans isomerase FKBP14-like	K09577	KOG0549	91	54	52	119	30	6	9	12	253	109	15	58	1333	1131	621	123
ACC_08786	sodium/potassium-transporting ATPase subunit alp	K01539	KOG0203	28045	7228	10770	20863	5317	1174	1914	3054	7071	3902	3136	2601	827	1151	3310	2620
ACC_08787	sodium/potassium-transporting ATPase subunit alp	K03016	KOG3400	277	153	158	590	307	45	66	101	360	153	269	273	127	134	92	47
ACC_08788	transcriptional activator protein Pur-alpha-like isoform 2		KOG3074	2662	1116	1185	1887	683	229	300	328	2180	1578	257	413	467	429	850	502
ACC_08789	blood vessel epicardial substance-like			176	71	81	100	84	6	17	20	235	229	40	163	133	73	24	9
ACC_08790	conserved hypothetical protein			299	184	248	460	110	4	15	30	97	52	3	1	1	0	8	3
ACC_08791	enhancer of split mbeta protein-like		KOG4304	71	38	127	179	61	7	11	14	88	63	7	12	3	24	47	16
ACC_08792	tubulin polyglutamylase TTLL4-like	K16601	KOG2156	74	40	28	126	62	17	25	37	120	113	49	81	265	311	420	248
ACC_08793	juvenile hormone esterase-like, partial		KOG1516	37	22	17	49	28	5	11	16	106	20	18	6	818	121	25	22
ACC_08794	conserved hypothetical protein	K06971		36	20	24	35	31	1	3	4	32	12	22	17	38	31	3	4
ACC_08795	tumor protein p53-inducible nuclear protein 2-like			292	129	212	444	81	47	64	99	505	430	205	112	49	38	179	87
ACC_08796	endothelin-converting enzyme 2 isoform 1	K08635	KOG3624	236	226	230	688	247	26	23	42	281	270	51	33	8	16	9	15
ACC_08797	THO complex subunit 2	K12879	KOG1874	1053	889	783	1253	919	246	518	578	1305	886	586	461	1141	1332	2198	1514
ACC_08798	rhomboid family member 1			47	36	33	38	7	1	1	2	17	17	12	7	0	4	5	4
ACC_08799	muscle M-line assembly protein unc-89-like, partial		KOG3513	12	3	5	10	4	1	1	1	23	36	0	0	0	1	0	0
ACC_08800	conserved hypothetical protein			92	61	73	166	29	4	4	8	28	31	15	17	7	14	19	6
ACC_08801	knirps-related protein	K08706	KOG4216	1	0	1	0	0	0	0	0	13	16	6	21	12	10	14	4
ACC_08802	ribosomal protein L35	K02918	KOG3436	616	548	336	1175	700	148	239	401	569	597	1454	936	2879	3508	935	558
ACC_08803	conserved hypothetical protein			49	62	41	78	65	0	4	5	30	15	44	119	60	85	6	2
ACC_08804	laccase-5-like		KOG1263	99	99	81	1294	293	80	117	144	1116	145	21	11	4	8	9	14
ACC_08805	NADH dehydrogenase subunit 5			6	6	0	3	5	0	0	0	6	3	3	0	0	0	0	0
ACC_08806	ligand-gated chloride channel homolog 3 precursor	K05181	KOG3643	466	124	121	302	167	12	16	14	134	42	0	0	1	1	3	1
ACC_08807	conserved hypothetical protein	K09220	KOG3608	385	300	224	277	136	53	52	74	669	547	112	256	549	534	165	24
ACC_08808	conserved hypothetical protein		KOG2103	860	527	480	622	467	61	88	80	1252	1361	877	1582	2182	2097	239	42
ACC_08809	cation-independent mannose-6-phosphate receptor			33	26	20	43	50	1	5	3	83	45	13	24	90	91	5	1
ACC_08810	conserved hypothetical protein			0	0	1	1	0	0	0	0	1	0	0	0	0	1	0	0
ACC_08811	conserved hypothetical protein			28	12	10	72	29	34	59	61	561	474	110	569	2050	2823	493	71
ACC_08812	conserved hypothetical protein		KOG1239	270	135	188	965	422	76	91	40	3986	5277	34	81	330	248	23	14
ACC_08813	conserved hypothetical protein		KOG3627	468	267	336	762	399	79	108	129	6944	7587	37	33	628	541	191	52
ACC_08814	conserved hypothetical protein		KOG1887	173	218	145	61	20	30	28	38	382	181	20	60	178	223	172	48
ACC_08815	cathepsin J-like		KOG1542	24	26	54	115	55	0	4	4	28	10	1	1	2	3	5	4
ACC_08816	protein NDUFAF4 homolog		KOG4481	53	39	41	106	110	6	9	9	36	28	55	113	129	145	10	8
ACC_08817	LOW QUALITY PROTEIN		KOG1847	200	151	140	295	86	26	44	63	302	218	62	40	138	161	67	51
ACC_08818	inositol oxygenase-like	K00469	KOG1573	851	774	984	1657	632	134	171	215	3383	11013	250	96	1834	617	521	440
ACC_08819	tetratricopeptide repeat protein 39C-like		KOG2687	2	3	3	23	14	0	1	0	1	0	1	0	0	0	0	0
ACC_08820	probable protein BRICK1-B-like	K05752		48	64	115	111	27	1	11	8	59	19	146	76	69	85	11	12
ACC_08821	nuclear protein localization protein 4 homolog	K14015	KOG2834	257	169	151	306	263	40	38	63	410	359	104	399	556	579	119	34
ACC_08822	conserved hypothetical protein			95	43	76	101	56	11	16	12	92	58	9	8	118	514	874	17
ACC_08823	LOW QUALITY PROTEIN			351	258	258	629	637	45	75	93	412	206	304	356	275	339	66	25
ACC_08824	beta-1,3-galactosyltransferase 5-like		KOG2287	100	46	46	92	71	4	5	9	199	130	37	215	126	102	16	5
ACC_08825	LOW QUALITY PROTEIN		KOG1847	1127	1035	1210	1848	479	158	244	288	2043	4792	366	135	927	741	730	587
ACC_08826	activin receptor type-1-like	K04675	KOG2052	191	86	92	152	161	11	11	12	350	264	67	312	272	242	14	3
ACC_08827	lachesin-like		KOG3513	207	100	92	171	48	12	21	34	107	85	51	60	6	1	2	3
ACC_08828	conserved hypothetical protein	K09396	KOG3563	1	4	3	3	1	2	4	2	3	1	0	0	2	2	18	10
ACC_08829	transmembrane 7 superfamily member 3-like			217	170	155	185	122	13	9	34	140	159	76	53	176	131	21	8
ACC_08830	ubiquitin carboxyl-terminal hydrolase 47-like isofor	K11857	KOG4598	448	231	194	384	291	50	106	104	901	1261	124	319	812	754	301	121
ACC_08831	LOW QUALITY PROTEIN		KOG1308	250	156	149	270	185	27	49	51	649	448	170	581	689	783	283	165
ACC_08832	patatin-like phospholipase domain-containing protein 2-like			237	117	135	226	270	9	23	29	285	294	58	102	180	110	30	9

ACC_08833	LOW QUALITY PROTEIN	K08883	KOG1474	349	383	399	450	268	36	53	73	464	436	89	131	344	394	146	51
ACC_08834	conserved hypothetical protein		KOG0528	57	47	58	69	11	3	4	4	108	151	5	7	30	29	123	31
ACC_08835	conserved hypothetical protein			67	27	13	131	63	16	18	23	39	28	41	14	11	11	37	9
ACC_08836	leucine-rich repeats and immunoglobulin-like domains prote		KOG4194	5	22	4	0	4	0	0	0	355	2000	0	0	7	3	1	0
ACC_08837	conserved hypothetical protein			92	73	82	139	149	8	10	10	54	45	69	112	104	86	21	11
ACC_08838	ATP-dependent zinc metalloprotease YME1 homolo	K08955	KOG0734	1186	729	649	1119	955	96	139	179	1566	1408	389	1534	1878	1860	269	113
ACC_08839	conserved hypothetical protein		KOG1437	4	7	1	5	7	0	2	2	19	4	74	191	209	697	76	96
ACC_08840	fibronectin type-III domain-containing protein C4orf31 homo		KOG4806	1	2	0	0	0	2	3	2	52	29	0	1	29	15	5	4
ACC_08841	conserved hypothetical protein	K10718	KOG1540	41	36	104	450	187	7	17	18	64	84	14	0	86	3085	700	423
ACC_08842	LOW QUALITY PROTEIN	K09259	KOG0507	119	74	63	254	167	18	21	32	433	172	89	752	325	377	90	14
ACC_08843	actin-related protein 2/3 complex subunit 3-like isc	K05756	KOG3155	221	172	166	343	334	34	88	120	385	132	400	387	527	877	192	138
ACC_08844	transient receptor potential cation channel subfamily V mem		KOG3676	10	10	12	18	10	3	4	2	108	14	26	37	0	2	2	1
ACC_08845	probable phospholipid-transporting ATPase IIB-like			0	0	0	0	0	0	0	0	3	0	37	8	0	0	0	0
ACC_08846	e3 ubiquitin-protein ligase TRIM9	K10649	KOG4367	65	36	24	113	29	5	11	14	48	18	11	11	16	17	86	33
ACC_08847	organic cation transporter 1-like		KOG0255	84	15	19	58	14	1	0	2	68	45	16	55	36	4	16	13
ACC_08848	tubulin beta-2 chain-like		KOG1375	32	7	13	41	9	0	0	1	35	30	14	1	3	3	0	3
ACC_08849	serine/threonine-protein phosphatase 6 regulatory ankyrin r		KOG4177	40	28	37	61	21	16	13	17	211	576	53	14	71	31	37	27
ACC_08850	coiled-coil domain-containing protein C6orf97-like		KOG0161	37	8	13	21	4	2	0	1	51	40	377	53	11	23	15	3
ACC_08851	e3 ubiquitin-protein ligase MYLIP, partial	K10637	KOG3530	221	121	94	243	164	12	24	33	481	438	218	227	141	93	88	41
ACC_08852	conserved hypothetical protein		KOG1924	1254	1060	1083	949	346	89	79	101	867	715	182	129	297	381	387	179
ACC_08853	conserved hypothetical protein			128	96	79	185	206	13	21	18	152	64	73	164	158	169	24	4
ACC_08854	conserved hypothetical protein		KOG4157	2482	1751	1450	1732	1728	313	774	752	1557	1149	949	485	779	1071	1170	314
ACC_08855	lactosylceramide 4-alpha-galactosyltransferase-like	K01988	KOG1928	80	23	29	51	31	4	5	6	75	95	74	422	53	5	1	2
ACC_08856	UPF0532 protein CG3570-like			54	56	44	54	82	12	14	19	78	77	60	116	219	209	33	20
ACC_08857	conserved hypothetical protein			19	14	14	102	96	2	13	23	38	54	95	395	67	54	26	19
ACC_08858	conserved hypothetical protein		KOG0779	52	35	23	66	46	7	8	16	95	52	24	44	105	183	22	7
ACC_08859	putative inorganic phosphate cotransporter-like	K08193	KOG2532	16	8	22	22	22	0	3	10	45	17	5	57	335	334	42	7
ACC_08860	testis-expressed sequence 2 protein-like		KOG2238	3181	2371	2375	1708	2098	843	1845	2098	2765	2052	996	732	1616	1852	3474	2529
ACC_08861	putative piggyBac transposase Uribo1			373	181	150	188	263	35	115	96	498	434	188	261	487	330	104	39
ACC_08862	cellular retinaldehyde binding protein		KOG1471	78	25	27	192	62	7	11	17	142	37	29	103	706	946	161	280
ACC_08863	conserved hypothetical protein		KOG2591	87	36	42	74	25	9	4	9	94	81	24	19	28	18	37	15
ACC_08864	ubiquitin carboxyl-terminal hydrolase 20-like	K11848	KOG1870	217	162	129	174	155	24	23	17	916	742	169	661	711	837	55	16
ACC_08865	putative homeodomain transcription factor-like			136	63	57	105	129	8	12	15	136	83	93	48	89	135	14	1
ACC_08866	g1/S-specific cyclin-D2			86	56	65	89	24	3	9	11	51	45	15	12	44	45	96	19
ACC_08867	octopamine receptor beta-2R isoform 1		KOG4220	14	9	13	3	1	0	1	0	25	20	2	1	1	0	2	0
ACC_08868	conserved hypothetical protein			4	0	0	1	0	0	0	1	4	0	0	35	34	80	43	79
ACC_08869	serine protease nudel		KOG3627	3	2	1	1	0	0	0	1	2	0	0	0	2	0	0	0
ACC_08870	serine protease nudel		KOG1215	36	29	10	38	15	10	18	20	72	25	80	89	61	66	103	72
ACC_08871	conserved hypothetical protein		KOG1217	111	20	11	21	7	7	11	13	390	171	35	163	146	489	409	139
ACC_08872	cyclic AMP response element-binding protein A-like		KOG0709	35	35	40	29	8	5	8	11	79	231	9	55	75	74	68	22
ACC_08873	conserved hypothetical protein			197	11	16	47	16	2	1	4	60	5	21	583	3	0	2	3
ACC_08874	BTB/POZ domain-containing protein 7-like			13	2	2	12	4	1	1	1	13	21	2	7	25	10	5	0
ACC_08875	conserved hypothetical protein			27	40	12	20	5	27	40	58	275	103	13	19	3	4	1	0
ACC_08876	alpha-glucosidase isozyme II	K01187	KOG0471	14	13	10	69	10	7	4	3	228	1576	2	0	14076	6435	1201	72
ACC_08877	conserved hypothetical protein			25	14	11	35	64	1	1	2	11	12	10	35	64	60	3	2
ACC_08878	conserved hypothetical protein			0	0	0	0	2	0	0	0	1	0	12	8	0	0	0	0
ACC_08879	conserved hypothetical protein			7	0	0	3	0	0	0	0	0	1	1	2	0	0	0	0
ACC_08880	chorion peroxidase		KOG2408	10	2	3	14	9	2	5	5	57	45	21	34	24	51	5	3
ACC_08881	protein FAM46A-like isoform 4		KOG3852	216	374	401	574	83	72	103	240	275	609	248	71	49	108	873	458
ACC_08882	conserved hypothetical protein	K09299	KOG3623	740	355	533	1232	437	71	134	167	805	772	68	68	128	189	731	282
ACC_08883	organic cation transporter-like protein-like		KOG0255	3	1	1	7	5	0	1	1	48	104	217	253	88	66	5	0
ACC_08884	pancreatic lipase-related protein 2-like	K14073		45	30	26	103	64	3	2	4	175	486	1	7	384	138	38	5
ACC_08885	ran-binding protein 9-like			48	10	10	38	12	2	0	0	61	78	22	55	37	18	4	1
ACC_08886	conserved hypothetical protein		KOG4701	194	123	187	155	38	21	26	29	760	564	97	184	179	222	269	177
ACC_08887	Cytochrome c1-2, heme protein, mitochondrial		KOG3052	120	57	74	113	76	16	45	45	85	46	55	76	56	44	121	131
ACC_08888	conserved hypothetical protein			66	52	29	151	82	8	19	18	119	79	80	185	137	137	54	8
ACC_08889	Trophoblast glycoprotein		KOG4194	269	126	186	333	250	10	35	47	266	140	92	526	568	387	71	34

ACC_08890	BMP-binding endothelial regulator protein isoform 1			193	73	113	342	64	36	56	61	784	830	247	299	191	141	357	178
ACC_08891	U3 small nucleolar ribonucleoprotein protein IMP3	K14560	KOG4655	436	226	223	399	334	55	168	156	399	197	276	584	540	673	286	252
ACC_08892	conserved hypothetical protein			52	57	58	55	47	0	17	19	51	43	22	28	41	40	9	7
ACC_08893	nucleoside diphosphate-linked moiety X motif 8, mitochondr		KOG3069	134	85	53	94	92	4	19	10	152	179	117	247	246	234	27	14
ACC_08894	DNA ligase 3	K10776	KOG4437	400	649	178	254	134	326	411	669	154	311	82	83	187	242	99	61
ACC_08895	conserved hypothetical protein	K02916	KOG4316	175	128	102	236	227	21	42	60	208	105	158	365	509	841	169	100
ACC_08896	conserved hypothetical protein			2	1	0	9	2	0	0	1	11	1	0	0	1294	704	159	3
ACC_08897	conserved hypothetical protein	K06091	KOG0609	2740	1615	2184	4207	996	449	749	993	1679	1392	1042	459	466	560	1592	832
ACC_08898	6-phosphofructo-2-kinase/fructose-2,6-biphosphat	K01103	KOG0234	142	92	72	160	118	13	16	21	411	203	158	274	304	358	40	13
ACC_08899	lysyl oxidase homolog 4	K00280		60	79	92	148	49	8	13	18	298	215	46	637	1	0	4	1
ACC_08900	myeloid leukemia factor		KOG4049	806	528	597	1088	769	49	66	110	612	369	273	700	576	846	343	195
ACC_08901	LOW QUALITY PROTEIN	K10408	KOG3595	6	6	8	15	6	5	7	6	34	13	17	1	3	1	2	5
ACC_08902	ceramide glucosyltransferase	K00720	KOG2547	42	18	20	43	15	6	6	9	105	91	40	49	8	11	18	4
ACC_08903	conserved hypothetical protein			68	46	33	87	104	0	7	5	57	48	46	136	127	128	10	3
ACC_08904	echinoderm microtubule-associated protein-like CG42247-lil		KOG2106	108	41	47	81	26	14	9	15	75	146	90	28	7	23	153	179
ACC_08905	metabotropic glutamate receptor 1	K04605	KOG1056	205	86	88	110	26	4	5	9	84	83	4	6	34	11	11	26
ACC_08906	lipoamide acyltransferase component of branched-chain alp		KOG0558	5	2	4	5	5	0	0	0	66	178	16	37	52	103	27	8
ACC_08907	one cut domain family member 2		KOG2252	682	410	588	846	350	54	79	96	248	286	139	32	12	23	134	358
ACC_08908	vesicular glutamate transporter 2.1-like		KOG2532	363	380	276	334	216	142	166	210	3594	1300	2376	3601	43	67	101	38
ACC_08909	prohormone-2			976	757	1410	6590	2261	86	102	168	349	427	39	76	7	23	22	7
ACC_08910	alpha-tocopherol transfer protein-like isoform 2		KOG1471	67	42	20	56	37	35	35	35	1503	1401	359	561	263	102	87	32
ACC_08911	reactive oxygen species modulator 1-like isoform 1		KOG4096	470	276	256	803	473	63	74	72	489	345	364	1043	1159	1033	247	125
ACC_08912	dipeptidyl aminopeptidase-like protein 6-like		KOG2100	350	187	213	414	72	21	34	49	425	387	356	76	9	6	49	43
ACC_08913	E3 ubiquitin-protein ligase AMFR-like		KOG0802	599	257	196	355	198	44	40	54	822	417	396	1229	191	176	29	7
ACC_08914	sodium/potassium/calcium exchanger 4-like		KOG1307	67	54	46	115	58	1	6	3	76	51	4	48	1	2	2	0
ACC_08915	Copia protein			12	6	3	5	3	1	2	5	7	7	0	2	5	8	12	2
ACC_08916	conserved hypothetical protein	K10624	KOG0147	4169	2803	3175	3316	3411	815	1708	1899	2249	1698	1359	510	1532	2946	4430	2434
ACC_08917	blood vessel epicardial substance-like			231	113	108	182	135	6	17	20	342	295	48	236	221	134	38	6
ACC_08918	conserved hypothetical protein		KOG2504	521	275	300	827	251	32	50	52	558	392	73	31	26	64	122	18
ACC_08919	mariner transposase			71	65	41	103	99	15	24	38	72	36	23	22	6	10	18	13
ACC_08920	glycerate kinase-like	K15788	KOG3935	372	206	190	522	455	110	199	224	590	450	459	576	802	1326	497	273
ACC_08921	conserved hypothetical protein			29	12	24	37	5	2	7	7	143	31	0	0	2	3	20	10
ACC_08922	glutamate	K05210	KOG1053	104	34	41	83	17	0	3	2	124	67	4	0	1	10	33	13
ACC_08923	LOW QUALITY PROTEIN	K14772	KOG1823	1676	1032	854	1617	1766	220	723	738	2952	2046	785	547	2210	1617	1734	660
ACC_08924	probable cytochrome P450 305a1		KOG0156	159	76	66	197	185	110	149	140	4077	172	5	11	3286	582	33	45
ACC_08925	retinoid-inducible serine carboxypeptidase-like	K09646	KOG1283	8	4	4	9	7	3	3	2	16	20	6	6	4372	313	128	110
ACC_08926	fork head domain-containing protein FD2-like	K09411	KOG3563	8	4	9	18	10	5	5	10	5	10	3	0	24	52	51	40
ACC_08927	conserved hypothetical protein		KOG1474	2766	3010	2761	2866	1691	717	1353	1390	3212	1900	1011	214	748	1014	3478	2270
ACC_08928	l-lactate dehydrogenase A-like 6A-like		KOG1495	19	33	25	61	10	4	1	2	5	14	1	0	1	2	0	0
ACC_08929	ran-binding protein 9-like		KOG1477	287	164	123	203	150	22	35	46	545	534	70	314	280	209	46	24
ACC_08930	LOW QUALITY PROTEIN		KOG1385	268	184	164	577	502	23	17	34	1289	1120	500	2051	728	466	37	6
ACC_08931	homeobox protein araucan		KOG0773	23	10	36	46	11	0	1	5	10	20	0	1	10	22	240	55
ACC_08932	conserved hypothetical protein		KOG4004	16	5	9	23	0	0	0	1	6	2	2	1	1	1	1	0
ACC_08933	major royal jelly protein 5-like isoform 2			13	705	334	121	107	229	203	367	1082	320	585	15	1	0	0	0
ACC_08934	isocitrate dehydrogenase	K00030	KOG0784	618	305	291	527	485	36	57	75	1859	713	189	902	918	1105	97	41
ACC_08935	conserved hypothetical protein			207	159	213	297	74	22	39	46	530	567	78	162	31	23	120	46
ACC_08936	disco-interacting protein 2		KOG3628	59	35	27	34	15	6	13	11	162	279	29	65	69	44	30	14
ACC_08937	conserved hypothetical protein			25	22	20	15	2	5	1	1	27	31	2	10	3	3	23	5
ACC_08938	neprilysin 2		KOG3624	609	349	54	158	121	223	227	336	1688	745	1436	2870	3074	1121	298	245
ACC_08939	elongation factor 1-alpha	K03231	KOG0052	815	331	350	2340	577	52	71	132	663	677	234	429	37	23	17	22
ACC_08940	glycerol-3-phosphate dehydrogenase		KOG2711	61	54	60	170	126	4	20	15	331	143	55	195	63	46	2	2
ACC_08941	glycerol-3-phosphate dehydrogenase	K00057	KOG2711	151	153	107	298	332	22	26	26	1144	303	144	361	138	81	5	7
ACC_08942	rho guanine nucleotide exchange factor 10-like	K16727	KOG3522	580	303	348	440	293	58	89	99	1612	1320	174	618	698	542	224	76
ACC_08943	LOW QUALITY PROTEIN			21	4	3	15	9	4	8	7	29	30	172	66	6	11	13	10
ACC_08944	conserved hypothetical protein			68	78	35	60	22	34	55	69	492	113	45	18	22	49	59	27
ACC_08945	conserved hypothetical protein		KOG3598	205	203	204	364	115	21	33	30	65	49	48	38	4	6	8	11
ACC_08946	AFG3-like protein 2-like	K08956	KOG0731	1227	797	805	1255	1048	103	163	180	4561	3131	820	2302	3624	4691	563	158

ACC_08947	conserved hypothetical protein			21	4	11	23	7	1	1	0	7	15	7	7	2	2	1	3
ACC_08948	conserved hypothetical protein			23	15	16	24	1	2	1	4	14	11	8	7	1	4	3	5
ACC_08949	conserved hypothetical protein			13	4	3	13	2	0	2	0	10	6	2	9	2	3	2	1
ACC_08950	conserved hypothetical protein			60	30	52	70	26	10	13	13	240	99	60	108	30	23	11	3
ACC_08951	conserved hypothetical protein			30966	4262	1645	4877	1942	21874	21218	34459	5484	18866	2572	12546	226	143	203653	129700
ACC_08952	bifunctional arginine demethylase and lysyl-hydrox	K11323	KOG2130	321	284	291	432	336	78	150	192	408	221	280	403	515	653	352	348
ACC_08953	conserved hypothetical protein		KOG4381	286	203	243	344	271	44	43	51	647	526	150	365	320	278	62	22
ACC_08954	UDP-galactose translocator 1-like		KOG2234	98	68	60	82	170	0	7	3	46	60	49	144	182	102	10	0
ACC_08955	conserved hypothetical protein		KOG3621	313	164	167	339	370	16	30	38	339	316	142	230	140	129	19	7
ACC_08956	transient receptor potential cation channel protein painless		KOG0510	124	90	94	170	195	18	32	22	526	282	151	461	86	54	6	5
ACC_08957	zinc finger protein 714-like		KOG2462	6	7	12	21	5	3	3	9	30	59	5	5	22	17	36	12
ACC_08958	dihydrolipoyllysine-residue acetyltransferase comp	K01183	KOG0557	296	103	133	162	129	9	13	17	690	345	72	514	1586	1574	347	151
ACC_08959	neurotrimin-like		KOG3513	287	191	200	419	92	99	142	155	186	76	67	40	13	35	85	56
ACC_08960	serine/threonine-protein kinase tricorner	K08790	KOG0605	428	175	174	515	191	72	93	137	891	622	151	111	120	122	110	42
ACC_08961	scavenger receptor class B member 1-like		KOG3776	55	10	11	7	9	25	30	33	566	245	1410	2681	154	241	74	44
ACC_08962	glycyl-tRNA synthetase	K01880	KOG2298	401	332	272	523	523	44	62	72	582	657	272	821	2123	1389	136	45
ACC_08963	conserved hypothetical protein		KOG4701	968	408	615	638	211	70	83	133	297	448	109	126	34	51	431	466
ACC_08964	DCN1-like protein 4-like		KOG3077	122	63	57	129	111	9	8	13	245	158	61	242	238	298	30	11
ACC_08965	conserved hypothetical protein			5	7	4	5	3	1	5	7	326	18	112	28	78	127	40	3
ACC_08966	conserved hypothetical protein	K08706		18	6	6	9	3	0	1	1	63	122	27	42	39	47	79	38
ACC_08967	cytochrome P450 6k1-like		KOG0158	230	57	15	201	117	35	35	43	5160	2012	56158	112151	29421	866	8	2
ACC_08968	conserved hypothetical protein			3	1	4	5	1	0	0	0	4	6	0	1	1	4	3	0
ACC_08969	venom acid phosphatase Acph-1-like		KOG3720	13	16	5	16	11	12	17	15	1951	485	103	271	58	54	4	9
ACC_08970	conserved hypothetical protein	K16315	KOG2464	755	500	404	728	1005	77	170	184	568	554	594	573	404	350	184	86
ACC_08971	transmembrane 9 superfamily member 2-like isoform 1		KOG1278	513	312	230	569	600	56	83	124	1538	1588	227	1301	1872	1147	63	27
ACC_08972	exportin-6		KOG2020	282	166	145	241	210	32	23	24	404	299	120	237	481	403	27	12
ACC_08973	feminizer		KOG0415	564	388	350	318	325	85	197	181	445	333	224	104	546	479	285	294
ACC_08974	conserved hypothetical protein	K08739	KOG1977	888	1232	2407	2959	3015	72	79	100	649	344	386	1034	94	118	9	2
ACC_08975	conserved hypothetical protein			191	79	90	168	150	15	26	12	405	270	156	269	323	150	28	13
ACC_08976	probable 2-oxoglutarate dehydrogenase E1 compo	K15791	KOG0451	45	32	27	51	39	3	10	7	285	522	40	16	2196	1195	127	51
ACC_08977	zinc finger protein weckle-like		KOG2462	447	296	258	311	322	51	105	117	257	151	138	34	36	36	67	30
ACC_08978	myosin-IXb isoform 1			0	0	0	1	0	0	1	1	1	5	0	1	0	1	6	5
ACC_08979	ribokinase-like	K00852	KOG2855	131	47	47	108	116	13	7	14	249	284	34	108	2142	534	20	17
ACC_08980	conserved hypothetical protein			302	193	156	240	261	28	50	53	729	377	367	584	473	492	24	14
ACC_08981	cystinosin homolog		KOG3145	273	234	336	548	688	16	21	27	548	338	118	355	601	538	52	10
ACC_08982	BTB/POZ domain-containing protein KCTD15-like		KOG2723	4	2	5	5	3	0	0	1	2	6	0	1	1	2	1	0
ACC_08983	aminopeptidase N-like		KOG1046	0	0	0	0	0	0	0	0	2	0	0	0	75	63	14	2
ACC_08984	ribonuclease Z, mitochondrial-like	K00784	KOG2121	774	353	388	678	767	61	252	211	908	730	656	756	3199	3124	1059	511
ACC_08985	LOW QUALITY PROTEIN	K09669	KOG2619	306	235	306	348	477	18	23	37	109	132	154	220	124	142	56	22
ACC_08986	venom acid phosphatase Acph-1-like	K01078	KOG3720	8	12	6	21	10	5	6	12	5	25	3	3	196	113	19	2
ACC_08987	conserved hypothetical protein	K05766	KOG1716	642	334	420	630	133	102	169	221	1026	881	218	77	156	219	957	1248
ACC_08988	signal recognition particle receptor subunit alpha h	K13431	KOG0781	904	514	350	900	551	269	465	507	1322	1120	638	1054	1589	1614	971	540
ACC_08989	zinc finger protein 622-like isoform 1	K14816	KOG2785	229	254	282	312	302	36	83	90	488	277	210	274	513	594	181	119
ACC_08990	proto-oncogene c-Fos-like		KOG1414	258	100	120	228	81	30	35	61	922	521	175	539	185	94	259	51
ACC_08991	conserved hypothetical protein			23	15	27	38	14	2	11	15	22	40	44	74	3	57	147	83
ACC_08992	peroxiredoxin-like		KOG0852	169	68	69	96	50	15	42	30	93	44	19	6	23	28	69	17
ACC_08993	pyrazinamidase/nicotinamidase-like isoform 2		KOG4003	133	82	87	90	96	21	41	24	329	103	246	434	165	204	44	20
ACC_08994	nicotinic acetylcholine receptor alpha9 subunit precursor		KOG3645	33	16	15	144	30	2	3	7	13	29	11	18	276	125	95	35
ACC_08995	reticulon-4-interacting protein 1, mitochondrial-like isoform		KOG1198	341	246	299	484	364	44	79	126	276	177	196	268	275	322	69	40
ACC_08996	p21-activated protein kinase-interacting protein 1-	K14830	KOG0294	296	203	192	349	460	26	79	100	176	142	256	132	511	897	580	373
ACC_08997	conserved hypothetical protein		KOG0116	194	146	119	68	48	8	23	26	277	470	27	102	526	590	436	261
ACC_08998	conserved hypothetical protein			24	28	21	47	32	8	10	6	57	26	29	36	48	74	13	4
ACC_08999	Longitudinals lacking protein, isoforms F/I/K/T		KOG2462	188	181	192	292	92	38	60	100	46	130	68	22	8	13	211	192
ACC_09000	conserved hypothetical protein		KOG3993	334	286	333	516	244	41	74	108	129	141	83	13	26	38	268	149
ACC_09001	conserved hypothetical protein		KOG2462	271	206	247	577	192	43	55	98	67	83	58	3	9	26	164	124
ACC_09002	zinc finger protein 41-like		KOG3623	292	192	205	533	173	40	76	67	106	92	60	19	23	44	237	118
ACC_09003	conserved hypothetical protein			70	102	149	102	28	4	6	17	154	150	36	88	6	8	5	10

ACC_09004	basement membrane-specific heparan sulfate proteoglycan		KOG3513	134	58	67	173	36	3	8	12	34	33	17	6	4	12	10	5
ACC_09005	D2-like dopamine receptor	K14049	KOG4220	9	5	7	11	5	0	0	1	31	31	2	1	1	0	2	3
ACC_09006	conserved hypothetical protein			7	4	3	19	5	0	1	1	7	0	13	4	3	4	1	8
ACC_09007	cuticle protein 8			0	2	3	11	0	0	0	0	0	1	0	0	13	4	22	0
ACC_09008	odorant receptor 49b-like			8	1	4	0	2	7	1	3	1	0	10	18	1	1	0	0
ACC_09009	odorant receptor 49b-like			3	0	1	3	2	0	0	3	1	2	38	50	0	1	0	0
ACC_09010	kinesin 9	K10397	KOG4280	195	71	125	1223	891	13	23	84	10	79	1	0	0	13	12	2
ACC_09011	organic solute transporter alpha-like protein-like	K14360	KOG2641	20	11	13	89	41	1	1	0	7	1	3	25	123	118	37	7
ACC_09012	conserved hypothetical protein	K02919	KOG4122	101	101	106	123	163	9	8	17	29	24	78	129	114	187	38	23
ACC_09013	beta-hexosaminidase subunit beta-like	K12373	KOG2499	112	88	114	554	162	19	10	14	634	524	93	361	1121	2162	1214	166
ACC_09014	conserved hypothetical protein			6	4	2	6	2	1	1	3	11	1	3	4	2303	815	3348	1106
ACC_09015	conserved hypothetical protein		KOG0044	37	18	23	51	20	5	4	8	26	8	7	9	2	0	4	8
ACC_09016	conserved hypothetical protein			1	0	0	1	0	0	0	0	0	5	0	1	6	2	1	24
ACC_09017	conserved hypothetical protein			3	1	0	1	0	0	1	0	4	1	0	0	0	1	6	64
ACC_09018	guanylate cyclase, soluble, beta 1	K12319	KOG4171	362	156	156	496	138	19	22	26	137	93	79	103	2	34	10	3
ACC_09019	conserved hypothetical protein		KOG2824	37	11	10	85	20	16	31	26	124	80	69	63	90	184	420	378
ACC_09020	protein mab-21-like isoform 1		KOG3963	79	47	45	99	38	29	20	31	17	17	15	2	5	5	18	4
ACC_09021	actin 5C		KOG0676	18	8	3	30	4	2	1	1	979	123	73	459	20	91	86	28
ACC_09022	myosin-IB isoform 1	K10356	KOG0164	466	527	252	178	255	187	212	253	1301	1011	137	679	3323	3153	217	26
ACC_09023	conserved hypothetical protein		KOG3469	8	9	20	38	20	0	3	3	20	9	30	4	4	3	3	4
ACC_09024	protein outspread		KOG0161	506	321	259	144	62	75	111	127	568	402	87	202	241	177	213	177
ACC_09025	alba-like protein C9orf23 homolog		KOG2567	51	17	16	37	31	17	28	26	22	18	39	15	108	208	293	264
ACC_09026	conserved hypothetical protein		KOG0587	345	392	558	2352	548	76	82	107	4235	11115	838	870	84	31	119	51
ACC_09027	5'-nucleotidase domain-containing protein 3-like		KOG2470	449	182	200	421	412	23	48	56	641	246	77	193	78	129	25	6
ACC_09028	esterase E4-like		KOG1516	18	9	13	25	27	19	9	19	233	300	1	60	3	6	2	1
ACC_09029	LOW QUALITY PROTEIN			111	60	67	183	113	31	29	27	629	347	114	186	140	236	93	9
ACC_09030	sphingomyelin phosphodiesterase 4-like	K12353	KOG4396	140	114	104	115	88	10	15	20	163	141	49	56	268	464	63	17
ACC_09031	conserved hypothetical protein		KOG2504	3	2	0	12	8	3	8	7	194	125	4	9	147	201	56	35
ACC_09032	protein disulfide-isomerase TMX3-like	K09585	KOG4277	68	69	39	49	54	6	12	8	103	89	33	84	65	84	3	3
ACC_09033	protein disulfide-isomerase TMX3-like		KOG4277	84	41	46	122	100	6	6	16	110	84	54	69	49	93	16	2
ACC_09034	UDP-N-acetylglucosamine transferase subunit ALG	K07441	KOG3339	144	107	97	158	203	4	19	18	163	129	129	230	243	326	17	14
ACC_09035	triple functional domain protein		KOG0689	1900	1187	1159	2490	963	169	270	313	2352	1373	627	629	426	470	260	209
ACC_09036	probable ATP-dependent RNA helicase pitchoune		KOG0342	35	28	41	56	59	2	4	3	58	44	41	50	82	142	17	4
ACC_09037	conserved hypothetical protein			94	86	60	120	65	15	20	25	140	70	40	27	60	86	61	10
ACC_09038	C-myc promoter-binding protein-like			24	11	14	15	24	0	2	8	69	86	15	31	41	42	1	2
ACC_09039	LOW QUALITY PROTEIN		KOG2127	493	338	271	441	359	35	59	61	994	920	200	396	535	527	70	25
ACC_09040	GABA-gated ion channel isoform 1	K05175	KOG3642	277	96	101	136	67	5	6	5	194	59	27	50	6	14	17	3
ACC_09041	epithelial splicing regulatory protein 2	K14947	KOG1365	84	87	61	68	36	23	44	55	1370	1657	146	370	952	522	239	100
ACC_09042	centromere/kinetochore protein zw10 homolog	K11578	KOG2163	352	303	249	512	553	55	133	112	429	285	362	349	590	666	136	50
ACC_09043	conserved hypothetical protein		KOG3676	22	11	13	41	9	8	8	11	162	155	23	70	20	7	2	3
ACC_09044	conserved hypothetical protein			16	5	2	3	0	3	1	0	4	7	0	0	0	0	2	0
ACC_09045	40S ribosomal protein S28-like	K02979	KOG3502	744	761	424	665	340	212	352	398	218	476	269	636	1001	2356	1235	840
ACC_09046	polyubiquitin-A-like isoform 1		KOG0004	548	238	339	1077	612	24	35	56	384	160	265	414	126	114	22	5
ACC_09047	conserved hypothetical protein		KOG0905	0	1	2	10	7	0	2	1	1	1	0	8	1	1	5	0
ACC_09048	conserved hypothetical protein			6	9	4	9	5	1	1	3	12	43	13	17	12	10	1	1
ACC_09049	Nucleolysin TIA-1 isoform p40		KOG0148	519	357	288	494	107	75	64	110	126	143	63	23	7	8	55	42
ACC_09050	conserved hypothetical protein			7	4	3	5	2	1	1	0	2	13	0	10	1	28	50	23
ACC_09051	high affinity copper uptake protein 1-like		KOG3386	35	27	27	103	86	9	33	25	84	28	151	62	138	158	42	6
ACC_09052	conserved hypothetical protein			215	106	139	545	479	44	85	68	9114	11923	6	4	2328	253	3	3
ACC_09053	histone-lysine N-methyltransferase SETMAR-like			12	7	4	9	9	0	3	2	20	8	2	5	1	4	3	0
ACC_09054	protein TAPT1 homolog		KOG2490	105	72	48	120	120	10	29	29	143	141	86	146	95	90	64	22
ACC_09055	sodium channel protein 60E-like		KOG2301	1158	568	526	729	402	70	113	118	672	382	77	52	34	22	60	20
ACC_09056	exocyst complex component 7-like, partial	K07195	KOG2344	351	316	337	534	421	42	55	70	466	290	251	476	464	658	80	29
ACC_09057	conserved hypothetical protein			0	0	0	0	0	0	0	0	0	0	0	1	0	0	0	0
ACC_09058	protein turtle homolog B			106	35	47	103	32	2	4	8	32	16	16	12	0	7	18	4
ACC_09059	probable multidrug resistance-associated protein lethal(2)03		KOG0054	375	181	146	442	330	33	76	63	866	721	181	233	1146	1388	345	128
ACC_09060	sushi, von Willebrand factor type A, EGF and pentraxin doma		KOG1219	82	84	79	64	64	18	47	31	462	551	62	62	85	116	60	25

ACC_09061	lethal(2)neighbour of tid protein-like	K03845	KOG2762	156	106	97	158	179	11	24	26	185	175	170	179	339	578	35	19
ACC_09062	conserved hypothetical protein			32	24	13	21	3	2	1	5	12	31	9	23	18	17	68	62
ACC_09063	conserved hypothetical protein			118	74	104	83	119	2	14	8	75	141	45	205	156	138	9	6
ACC_09064	pikachurin-like		KOG3509	366	198	159	571	119	26	28	52	51	72	249	34	3	7	95	69
ACC_09065	conserved hypothetical protein			22	8	17	21	22	0	0	1	6	4	3	9	4	8	0	0
ACC_09066	flexible cuticle protein 12			32	17	18	43	31	2	2	3	524	101	177	1457	329	55	7	5
ACC_09067	conserved hypothetical protein		KOG1095	3675	2047	1730	2296	2475	552	1030	1090	2706	1999	1688	1103	1215	1335	1492	1069
ACC_09068	conserved hypothetical protein		KOG3598	343	281	363	184	47	4	5	3	222	180	15	14	10	11	18	12
ACC_09069	conserved hypothetical protein			1	0	0	1	0	0	0	0	0	1	0	0	14	7	4	4
ACC_09070	arrestin domain-containing protein 2		KOG3780	460	328	299	1158	374	35	40	71	130	48	35	10	1	0	1	3
ACC_09071	EF-hand calcium-binding domain-containing protein 1-like		KOG0044	1	0	0	0	0	0	0	0	1	0	0	0	1	0	0	0
ACC_09072	conserved hypothetical protein		KOG3802	351	292	341	582	131	40	73	117	257	204	47	22	5	17	85	66
ACC_09073	arrestin domain-containing protein 3		KOG3780	9	61	16	36	28	10	20	21	339	279	14	18	252	224	44	8
ACC_09074	CCAAT/enhancer-binding protein	K10052	KOG3119	106	32	53	98	18	29	91	64	428	786	16	63	315	250	1113	1327
ACC_09075	ubiquitin carboxyl-terminal hydrolase 46-like	K11842	KOG1864	154	98	82	207	200	15	21	37	293	152	197	446	258	355	48	17
ACC_09076	cyclin-dependent kinase 10	K02449	KOG0663	279	193	179	431	332	39	42	69	315	292	178	458	726	1199	135	53
ACC_09077	probable oligoribonuclease-like, partial	K13288	KOG3242	61	31	48	73	123	3	9	8	114	72	126	334	170	181	13	4
ACC_09078	conserved hypothetical protein		KOG3259	1130	805	737	828	666	225	469	465	1675	1027	510	515	1053	1052	711	693
ACC_09079	short spindle protein 4-like isoform 1		KOG3654	193	189	192	150	55	29	44	44	459	410	32	73	97	110	108	72
ACC_09080	leucine-rich repeat and immunoglobulin-like domain contair		KOG4194	79	64	72	101	34	13	25	20	42	65	19	4	2	5	52	10
ACC_09081	conserved hypothetical protein			80	39	64	98	119	1	5	2	12	25	66	95	58	44	42	36
ACC_09082	conserved hypothetical protein			20	26	20	28	23	3	2	2	38	39	14	18	16	26	7	2
ACC_09083	conserved hypothetical protein			133	31	18	26	48	8	41	32	282	728	1	15	903	739	329	82
ACC_09084	kidney mitochondrial carrier protein 1-like	K15106	KOG0753	357	270	270	444	488	32	71	87	526	283	359	508	484	531	60	22
ACC_09085	ankyrin repeat and zinc finger domain-containing protein 1-l		KOG2505	246	184	132	326	296	57	126	168	247	178	130	120	240	220	217	189
ACC_09086	LOW QUALITY PROTEIN	K10586	KOG0895	1675	1221	1147	1883	909	346	463	631	3150	2953	544	500	717	809	504	188
ACC_09087	conserved hypothetical protein			669	180	282	373	99	8	9	13	261	275	28	30	3	4	5	6
ACC_09088	ribosome biogenesis protein WDR12 homolog	K14863	KOG0313	364	261	234	314	281	38	43	43	326	328	226	421	1004	1495	246	128
ACC_09089	translocator protein-like	K05770	KOG3797	212	148	166	432	390	27	38	48	780	306	958	3623	89	92	23	4
ACC_09090	LOW QUALITY PROTEIN		KOG4436	293	255	204	382	160	45	75	88	407	323	70	214	134	82	76	40
ACC_09091	conserved hypothetical protein		KOG4244	1907	1033	688	1389	550	246	446	494	691	376	541	957	950	586	1794	1026
ACC_09092	monocarboxylate transporter 10-like		KOG2504	63	41	56	76	25	2	5	4	74	121	6	5	3	4	2	2
ACC_09093	scavenger receptor class B member 1-like		KOG3776	43	18	10	10	7	15	50	53	629	301	1495	3106	189	282	71	37
ACC_09094	lachesin-like			93	66	89	102	25	6	9	20	49	38	4	10	3	11	11	10
ACC_09095	conserved hypothetical protein		KOG4441	697	580	598	655	348	48	68	77	618	401	191	222	298	373	137	63
ACC_09096	CUE domain-containing protein 2-like			499	202	225	192	80	10	18	16	256	198	58	168	111	87	30	13
ACC_09097	receptor-type tyrosine-protein phosphatase R-like	K04458	KOG0791	753	190	249	528	266	80	101	163	563	357	128	186	135	124	125	18
ACC_09098	conserved hypothetical protein			7	3	6	11	4	3	1	6	10	22	4	4	4	3	5	5
ACC_09099	conserved hypothetical protein			1	1	1	1	1	1	1	1	2	4	1	2	1	2	3	4
ACC_09100	NADH dehydrogenase subunit 4			3962	1487	1250	1439	1583	47	155	116	1055	1072	1919	2873	2529	2519	1888	1460
ACC_09101	ubiquitin-conjugating enzyme E2 H-like			1010	328	394	1222	924	229	491	481	554	217	558	215	110	153	337	195
ACC_09102	conserved hypothetical protein		KOG1015	4120	2178	1427	2246	1413	558	1132	1185	2072	1701	1147	973	9498	19137	25852	22100
ACC_09103	probable sodium/potassium/calcium exchanger CG1090-like		KOG1307	903	412	419	682	385	65	67	100	413	172	108	70	20	30	60	59
ACC_09104	differentially expressed in FDCP 8 homolog		KOG1829	194	107	112	311	320	31	33	50	382	265	230	337	315	304	61	12
ACC_09105	odorant receptor 2a-like			34	34	28	51	53	2	1	5	87	89	75	94	182	243	33	6
ACC_09106	radial spoke head protein 3 homolog			32	40	36	75	32	7	3	13	15	10	173	83	0	3	6	5
ACC_09107	mRNA turnover protein 4 homolog	K14815	KOG0816	92	86	61	147	162	22	31	24	105	62	92	134	337	755	43	32
ACC_09108	conserved hypothetical protein		KOG0921	158	181	202	439	217	49	91	126	286	163	234	181	209	241	401	237
ACC_09109	conserved hypothetical protein			16	6	9	58	29	2	1	1	30	29	8	38	12	17	5	1
ACC_09110	conserved hypothetical protein		KOG0956	160	133	167	138	49	4	4	8	191	98	3	8	37	32	28	12
ACC_09111	UDP-glucose	K14689	KOG1482	17	9	12	51	42	3	1	4	70	67	38	136	245	455	73	8
ACC_09112	LOW QUALITY PROTEIN			92	53	60	113	150	1	6	4	55	56	105	136	84	148	9	1
ACC_09113	conserved hypothetical protein			31	26	35	43	37	7	7	3	18	25	19	18	21	49	0	0
ACC_09114	geranylgeranyl transferase type-2 subunit alpha-like		KOG0529	10	9	14	15	16	0	2	4	12	11	29	40	13	12	9	5
ACC_09115	e3 ubiquitin-protein ligase RNF19A-like	K11972	KOG1815	107	50	61	112	74	12	16	12	259	163	58	94	94	91	19	8
ACC_09116	glutathione S-transferase D1	K00799	KOG0867	23	57	41	411	175	33	41	40	1182	1812	699	521	57	73	13	3
ACC_09117	glutathione S-transferase 1-1-like		KOG0867	3	5	1	24	12	1	1	0	45	115	717	896	0	2	0	0

ACC_09118	forkhead box protein J1-like		KOG3563	0	0	1	1	0	0	1	0	34	27	232	589	0	2	3	1
ACC_09119	glycine receptor subunit alpha-3-like		KOG3644	119	113	77	142	146	7	8	9	207	117	45	287	377	254	36	4
ACC_09120	conserved hypothetical protein		KOG4723	80	84	100	88	160	1	1	2	21	78	49	269	102	112	12	4
ACC_09121	conserved hypothetical protein		KOG3598	348	224	243	287	76	34	38	23	521	616	70	72	104	103	134	50
ACC_09122	conserved hypothetical protein		KOG3627	1	1	0	1	1	0	0	0	8	3	0	0	7270	2743	430	438
ACC_09123	sodium/potassium-transporting ATPase subunit be	K01540	KOG3927	37	27	18	22	17	6	10	11	336	191	85	148	256	224	45	8
ACC_09124	serine/threonine-protein kinase GD17699-like	K08805	KOG0032	224	152	143	168	120	21	28	39	412	342	98	233	175	167	28	6
ACC_09125	protein sickie-like			76	70	85	62	16	19	16	22	80	68	22	15	6	9	13	8
ACC_09126	SET and MYND domain-containing protein 4-like		KOG0551	17	7	9	34	28	1	9	2	366	101	26	29	4	6	13	6
ACC_09127	conserved hypothetical protein		KOG1056	244	85	147	626	139	14	5	20	86	67	16	7	17	56	109	130
ACC_09128	conserved hypothetical protein			23540	3875	1711	7535	2066	4211	11197	17784	3534	12553	2782	15938	259	154	94776	84936
ACC_09129	protein will die slowly-like	K14963	KOG0271	155	124	93	182	154	19	25	32	220	177	75	300	374	238	28	11
ACC_09130	translocon-associated protein subunit alpha-like	K13249	KOG1631	247	183	49	126	149	24	28	41	108	87	99	107	817	813	44	13
ACC_09131	tRNA dimethylallyltransferase, mitochondrial-like, partial			14	15	20	37	16	3	14	16	28	14	12	10	20	42	39	33
ACC_09132	potassium voltage-gated channel protein Shab-like			450	409	310	483	143	34	52	68	258	189	51	49	20	18	6	7
ACC_09133	conserved hypothetical protein			248	155	129	303	85	16	48	46	134	76	31	15	18	8	8	3
ACC_09134	conserved hypothetical protein			940	406	379	794	477	68	182	196	333	187	109	52	26	21	36	25
ACC_09135	conserved hypothetical protein			351	195	170	333	450	17	17	17	613	392	275	752	932	474	48	8
ACC_09136	UPF0184 protein C9orf16 homolog			385	217	200	393	161	17	47	28	145	87	129	184	312	189	119	115
ACC_09137	conserved hypothetical protein			78	39	75	63	78	0	4	5	29	47	42	110	78	43	15	0
ACC_09138	conserved hypothetical protein			85	71	29	113	47	21	24	24	677	766	115	184	311	133	49	25
ACC_09139	heparan sulfate glucosamine 3-O-sulfotransferase :	K07809	KOG3704	268	150	161	329	264	9	26	22	111	69	53	28	8	9	3	0
ACC_09140	conserved hypothetical protein			398	317	265	427	140	48	87	106	510	969	78	394	662	558	293	107
ACC_09141	solute carrier family 22 member 8-like isoform 2		KOG0255	23	0	4	3	3	0	0	0	17	5	2	12	112	119	105	68
ACC_09142	conserved hypothetical protein		KOG3608	262	181	143	192	192	46	85	122	422	314	187	180	308	350	235	134
ACC_09143	RING finger protein 121-like isoform 1	K15698	KOG1734	116	94	78	114	111	13	21	30	299	171	144	249	311	247	23	5
ACC_09144	nephrin		KOG3515	160	91	83	36	22	2	5	9	109	108	14	8	19	6	26	15
ACC_09145	e3 ubiquitin-protein ligase SMURF2		KOG0939	76	55	43	143	101	9	8	13	244	237	84	389	139	85	25	15
ACC_09146	26S proteasome non-ATPase regulatory subunit 9-l	K06693	KOG3129	227	151	169	420	451	24	37	49	358	148	157	350	398	358	42	20
ACC_09147	conserved hypothetical protein		KOG3598	842	875	1251	1284	166	37	23	74	319	504	35	34	19	34	141	120
ACC_09148	Homeobox protein prospero		KOG3779	46	39	45	111	19	7	3	7	15	21	3	2	1	4	14	7
ACC_09149	carboxypeptidase B-like		KOG2650	3	1	0	4	3	0	0	4	10	3	0	0	13	381	146	215
ACC_09150	voltage-dependent L-type calcium channel subunit	K05317	KOG3812	1442	745	825	2414	549	89	158	233	880	398	127	38	9	13	66	44
ACC_09151	conserved hypothetical protein	K16682	KOG1701	218	210	145	133	69	30	27	38	372	219	59	102	241	201	71	26
ACC_09152	FMRFamide-related neuropeptides-like			31	102	258	570	118	3	7	11	20	9	63	4858	1	0	2	2
ACC_09153	distal-less	K09316	KOG0850	28	15	11	45	7	1	1	1	29	54	57	55	0	2	4	1
ACC_09154	conserved hypothetical protein	K02864	KOG4241	842	616	610	1391	806	150	264	282	1052	584	579	647	1130	1493	600	282
ACC_09155	fibrillin-2-like		KOG1214	398	34	26	118	47	12	14	20	826	188	57	180	242	212	208	91
ACC_09156	conserved hypothetical protein		KOG0307	2249	1839	1600	1472	925	263	444	503	1700	926	524	284	910	990	798	373
ACC_09157	transmembrane protein 177-like isoform 1			89	64	64	84	135	4	2	4	40	67	52	184	168	132	7	3
ACC_09158	conserved hypothetical protein			8	13	2	6	2	0	0	0	27	18	4	12	11	7	9	10
ACC_09159	conserved hypothetical protein			276	212	194	287	94	42	79	89	474	417	109	166	115	169	354	96
ACC_09160	protein trachealess-like isoform 1		KOG3558	2	1	1	1	0	0	0	0	5	2	0	0	4	7	10	10
ACC_09161	TD and POZ domain-containing protein 2-like		KOG4441	129	74	78	228	187	14	33	23	723	393	63	57	231	134	15	11
ACC_09162	conserved hypothetical protein			192	87	83	238	136	5	8	16	70	52	15	4	2	11	21	14
ACC_09163	ecdysis triggering hormone preproprotein			2	1	0	4	0	0	0	0	3	0	6	81	27	59	45	31
ACC_09164	poly(A)-specific ribonuclease PARN-like domain-containing p		KOG1990	180	129	118	145	170	10	33	24	160	128	71	63	167	169	29	10
ACC_09165	xanthine dehydrogenase			16	13	10	10	7	0	3	1	34	75	5	15	16	14	1	0
ACC_09166	conserved hypothetical protein			8	4	3	9	2	0	0	0	9	2	0	0	0	0	0	0
ACC_09167	conserved hypothetical protein			14	7	10	8	6	0	1	5	3	7	1	0	0	0	5	2
ACC_09168	slit homolog 2 protein		KOG4194	167	35	38	161	42	9	10	13	63	50	18	26	5	4	29	14
ACC_09169	mediator of RNA polymerase II transcription subun	K15164	KOG3600	183	152	174	295	118	28	18	40	169	132	55	45	112	106	142	28
ACC_09170	conserved hypothetical protein		KOG0250	95	118	109	312	308	46	81	98	110	234	941	110	13	19	95	68
ACC_09171	conserved hypothetical protein			63	71	75	156	103	15	35	36	121	205	476	180	46	34	40	17
ACC_09172	tetratricopeptide repeat protein 18-like		KOG1173	37	24	22	53	39	4	12	10	18	43	58	19	11	8	0	1
ACC_09173	protein FAM206A-like		KOG3266	70	63	73	87	137	4	1	5	39	64	40	101	186	341	32	6
ACC_09174	nose resistant to fluoxetine protein 6-like		KOG3700	52	25	8	12	11	4	11	15	162	245	5	57	81	37	1	0

ACC_09175	RNA-binding protein 28-like	K14573	KOG0127	1299	686	526	677	1072	199	538	566	768	628	698	521	1467	2239	2554	2349
ACC_09176	probable cytochrome P450 49a1		KOG0159	33	27	17	28	25	3	6	7	48	15	34	331	6	73	13	2
ACC_09177	conserved hypothetical protein			326	239	325	1123	376	101	151	219	232	187	75	76	53	68	553	225
ACC_09178	conserved hypothetical protein			127	106	146	384	160	9	12	18	201	183	18	49	57	72	111	14
ACC_09179	conserved hypothetical protein			62	37	38	154	52	9	5	7	124	104	43	892	3	4	0	0
ACC_09180	conserved hypothetical protein			1	0	0	0	0	0	0	0	4	40	0	1	0	0	1	0
ACC_09181	dihydrolipoyllysine-residue acetyltransferase comp	K00627	KOG0557	191	74	58	97	70	5	8	14	446	263	48	334	858	595	80	36
ACC_09182	A disintegrin and metalloproteinase with thrombospondin m		KOG3538	15	9	8	13	5	1	2	0	17	20	9	16	6	0	6	2
ACC_09183	neurotrimin-like		KOG3513	122	87	118	234	64	64	61	92	128	58	47	30	11	20	46	25
ACC_09184	glucosylceramidase-like isoform 1	K01201	KOG2566	512	1126	686	1064	682	1084	915	2013	15720	3065	433	2081	582	354	67	14
ACC_09185	fez family zinc finger protein 2-like		KOG2462	40	45	63	69	21	1	1	2	17	11	3	0	2	0	4	2
ACC_09186	glutamate receptor delta-2 subunit-like			1	0	1	0	1	0	1	1	34	33	33	12	4	0	0	0
ACC_09187	proclotting enzyme		KOG3627	60	27	49	124	27	16	19	25	113	190	19	41	13	10	34	2
ACC_09188	conserved hypothetical protein			22	22	34	61	66	4	3	0	24	29	19	37	38	36	3	5
ACC_09190	conserved hypothetical protein			28	8	13	60	42	4	8	8	127	87	26	45	26	30	29	10
ACC_09191	trehalase-like	K01194	KOG0602	74	134	12	39	13	65	60	69	941	1919	117	452	462	295	40	11
ACC_09192	Full=Secapin; Flags			0	0	0	0	0	0	0	0	0	0	0	0	0	0	0	0
ACC_09193	conserved hypothetical protein			1	3	1	1	0	0	0	0	0	0	0	4	0	0	0	0
ACC_09194	segmentation polarity homeobox protein engrailed		KOG0493	1	0	0	0	0	0	0	0	1	1	4	5	0	9	2	3
ACC_09195	conserved hypothetical protein			273	110	163	369	321	48	107	102	135	63	104	36	52	82	181	105
ACC_09196	conserved hypothetical protein		KOG4228	210	65	98	80	22	5	23	22	404	433	67	125	36	32	32	35
ACC_09197	bumetanide-sensitive sodium-(potassium)-chloride cotransp		KOG2083	70	36	42	103	88	6	4	9	413	466	85	347	1044	524	78	5
ACC_09198	probable cytochrome P450 6a14-like		KOG0158	0	0	0	4	1	0	0	0	7	1	0	1	1946	993	28	2
ACC_09199	probable cytochrome P450 6a14 isoform 1	K15002	KOG0158	789	940	1263	2421	1813	108	129	218	2848	1317	640	869	1135	936	106	6
ACC_09200	cytochrome P450 6A1		KOG0158	0	0	0	0	0	0	0	0	4	4	0	1	0	5	0	0
ACC_09201	probable cytochrome P450 6a14		KOG0158	1	6	12	9	15	0	3	0	42	143	2	10	10	274	20	1
ACC_09202	conserved hypothetical protein			810	491	496	451	73	33	36	68	338	439	77	19	7	9	117	81
ACC_09203	spermidine synthase	K00797	KOG1562	59	32	40	80	53	14	28	22	88	75	19	20	675	470	204	51
ACC_09204	probable chitinase 2-like		KOG2806	9	6	5	7	9	1	0	1	14	6	6	47	112	210	12	19
ACC_09205	NAD-dependent deacetylase sirtuin-5	K11415	KOG2684	224	129	127	291	321	15	9	20	628	264	131	627	362	310	11	4
ACC_09206	glyoxylate/hydroxypyruvate reductase A-like		KOG0069	98	75	78	102	109	3	18	14	166	88	97	107	145	249	25	15
ACC_09207	conserved hypothetical protein			63	11	25	53	9	6	3	3	149	76	9	15	186	198	233	208
ACC_09208	mediator of RNA polymerase II transcription subun	K15167		228	126	128	423	298	25	32	47	281	273	156	305	600	718	149	19
ACC_09209	fasciclin-3			119	55	51	108	39	14	20	25	154	71	86	131	26	53	162	134
ACC_09210	LOW QUALITY PROTEIN			57	29	35	63	42	9	17	24	83	53	76	136	38	61	123	41
ACC_09211	conserved hypothetical protein			1	2	0	3	1	1	0	0	4	0	0	0	0	1	2	2
ACC_09212	conserved hypothetical protein	K04536	KOG0286	1246	508	580	1305	342	79	104	146	894	683	319	318	354	415	1040	278
ACC_09213	cytochrome P450 307a1	K14939	KOG0156	2	1	3	10	1	1	1	1	9	10	1	1	2	5	24	32
ACC_09214	LOW QUALITY PROTEIN		KOG4583	388	259	295	381	258	36	48	40	713	339	269	521	502	477	40	9
ACC_09215	ras-related protein M-Ras-like	K07831	KOG0395	82	54	40	34	21	11	23	29	132	116	8	69	59	48	37	23
ACC_09216	CDGSH iron-sulfur domain-containing protein 3, mitochondr		KOG4605	161	147	124	81	128	4	2	11	102	105	54	218	300	436	42	8
ACC_09217	malate dehydrogenase-like		KOG4157	4	1	2	4	1	1	3	4	13	17	3	1	2	2	8	6
ACC_09218	chloride channel protein 2-like	K05011	KOG0476	17	7	16	16	7	6	12	6	53	78	4	11	37	28	97	47
ACC_09219	facilitated trehalose transporter Tret1-like		KOG0254	281	148	204	410	305	64	74	107	1597	1368	94	98	828	162	42	8
ACC_09220	conserved hypothetical protein			3	1	2	12	2	1	0	1	9	2	0	3	3	12	62	20
ACC_09221	receptor-type tyrosine-protein phosphatase S-like		KOG4228	38	28	24	52	30	7	18	17	216	317	13	68	252	143	21	8
ACC_09222	upstream-binding protein 1	K09275	KOG4091	1375	941	1101	1430	878	167	196	221	2709	1913	547	764	478	557	370	100
ACC_09223	conserved hypothetical protein		KOG2306	1585	700	657	1236	451	217	242	373	2382	1310	370	369	108	79	120	58
ACC_09224	conserved hypothetical protein			132	50	89	293	113	17	18	14	267	222	48	166	836	84	22	4
ACC_09225	zinc finger protein 396-like	K09212	KOG3623	34	15	11	29	5	6	18	15	36	40	12	7	7	5	114	100
ACC_09226	dolichol kinase-like	K00902	KOG2468	154	89	94	113	123	4	8	18	319	220	73	237	151	133	5	2
ACC_09227	arrestin domain-containing protein 3		KOG3780	119	133	135	267	143	26	33	46	319	238	175	298	312	225	56	35
ACC_09228	conserved hypothetical protein		KOG4572	679	580	750	649	172	120	117	254	1439	1717	130	124	76	73	151	131
ACC_09229	cytochrome P450 9e2		KOG0158	1508	1520	1827	4947	1420	115	120	199	2923	2617	2916	3494	841	890	258	78
ACC_09230	slit homolog 1 protein-like			58	32	26	22	10	4	12	9	48	39	8	37	3	13	14	1
ACC_09231	conserved hypothetical protein		KOG3660	18	10	11	54	10	1	4	5	297	124	128	313	222	128	40	11
ACC_09232	conserved hypothetical protein		KOG4713	594	471	614	849	527	106	211	234	545	499	248	423	670	1041	1014	337

ACC_09233	cyclin-dependent kinase 2-associated protein 2-like		KOG4713	47	30	13	21	19	2	1	2	40	15	18	20	26	46	6	2
ACC_09234	conserved hypothetical protein			12	0	1	2	0	0	0	0	2	0	21	154	229	165	115	365
ACC_09235	conserved hypothetical protein			13	1	4	2	0	2	2	1	2	7	1	5	1	3	3	7
ACC_09236	conserved hypothetical protein			2283	1597	1694	599	857	426	986	842	1388	507	379	235	227	135	530	426
ACC_09237	GPI inositol-deacylase-like	K05294	KOG3724	424	268	273	385	527	16	37	36	459	298	239	315	313	314	24	9
ACC_09238	sodium-independent sulfate anion transporter-like	K14708	KOG0236	2	4	4	8	6	1	0	1	12	67	50	144	30	32	5	0
ACC_09239	conserved hypothetical protein		KOG2408	0	1	0	1	0	1	2	2	1	14	3	6	12	9	9	7
ACC_09240	conserved hypothetical protein			172	107	89	148	198	17	23	19	26	39	60	26	12	22	32	10
ACC_09241	discoidin domain-containing receptor 2-like		KOG1094	157	89	92	171	18	6	6	10	55	33	9	12	5	7	42	14
ACC_09242	conserved hypothetical protein		KOG4193	10	5	5	23	10	2	1	2	192	773	2	4	69	55	23	2
ACC_09243	G-protein coupled receptor Mth2-like		KOG4193	8	6	9	38	33	2	2	0	115	315	19	156	43	36	20	1
ACC_09244	myb protein	K09420	KOG0048	249	207	183	299	111	12	28	25	192	188	24	85	120	163	169	35
ACC_09245	conserved hypothetical protein		KOG2418	527	329	339	843	404	207	211	334	917	604	410	275	319	665	237	84
ACC_09246	Ubiquitin carboxyl-terminal hydrolase 46	K11842	KOG1864	101	60	47	156	175	12	14	12	206	97	107	232	155	243	43	16
ACC_09247	short spindle protein 4-like isoform 1		KOG3654	58	43	44	43	18	4	16	9	112	106	16	33	22	17	35	33
ACC_09248	TP53-regulated inhibitor of apoptosis 1		KOG3481	39	38	60	73	86	0	3	5	10	15	25	44	37	47	4	1
ACC_09249	protein scarlet		KOG0061	16	5	8	18	18	15	34	40	111	53	7	14	1	1	0	2
ACC_09250	zinc finger protein 236		KOG2462	1238	960	858	1155	890	180	336	339	1069	1114	422	348	735	656	423	177
ACC_09251	FAD synthase-like	K00953	KOG2644	336	185	197	297	320	25	47	69	481	234	236	190	291	330	43	17
ACC_09252	syntaxin-1A	K04560	KOG0810	1112	435	506	941	276	75	135	159	915	603	147	150	125	103	397	293
ACC_09253	glutamate receptor delta-2 subunit-like		KOG1052	70	61	68	204	87	5	17	19	108	67	55	82	39	59	56	30
ACC_09254	conserved hypothetical protein		KOG1213	190	108	98	388	276	18	93	95	209	213	202	520	53	123	174	102
ACC_09255	low affinity cationic amino acid transporter 2-like		KOG1286	415	368	334	1001	621	67	98	124	262	228	87	112	586	529	109	35
ACC_09256	LOW QUALITY PROTEIN	K04427		41	17	20	39	37	3	3	3	197	57	30	58	84	60	7	0
ACC_09257	LOW QUALITY PROTEIN		KOG0668	148	107	105	208	133	10	17	31	139	192	94	118	290	452	219	72
ACC_09258	conserved hypothetical protein		KOG4790	775	627	612	504	368	43	35	48	619	401	179	118	333	166	39	2
ACC_09259	conserved hypothetical protein			105	58	75	206	62	7	7	20	91	53	72	159	23	36	34	27
ACC_09260	probable RNA-binding protein 19-like	K14787	KOG0110	835	502	330	730	572	164	339	419	379	422	351	419	1052	1455	2521	1756
ACC_09261	RCC1 and BTB domain-containing protein 1-like	K11494	KOG1427	113	64	72	131	147	13	13	24	218	131	84	121	190	227	27	5
ACC_09262	single Ig IL-1-related receptor-like			21	5	9	27	7	1	3	1	61	85	15	15	13	18	24	5
ACC_09263	protein stoned-B		KOG2677	1472	804	1086	974	368	48	50	56	1255	795	118	58	39	69	58	22
ACC_09264	6-phosphofructo-2-kinase/fructose-2,6-biphosphat	K01103	KOG0234	107	58	53	121	83	7	10	14	255	132	108	186	210	252	43	10
ACC_09265	TLD domain-containing protein KIAA1609 homolog isoform :		KOG2557	470	260	243	595	339	102	182	203	515	288	185	282	573	677	458	222
ACC_09266	conserved hypothetical protein		KOG4167	90	71	63	80	28	10	11	22	100	66	5	5	5	5	13	7
ACC_09267	conserved hypothetical protein		KOG3598	332	169	153	316	272	50	90	98	199	80	39	20	10	17	45	32
ACC_09268	fatty acyl-CoA reductase 1-like		KOG1221	156	132	121	162	145	21	27	44	98	57	91	29	1099	2538	190	81
ACC_09269	LOW QUALITY PROTEIN		KOG1998	916	553	466	394	290	93	195	202	1712	1155	327	396	561	547	247	96
ACC_09270	evolutionarily conserved signaling intermediate in '	K04405	KOG3941	186	110	117	180	187	13	18	23	131	154	80	206	299	314	39	17
ACC_09271	putative Dol-P-Glc	K03850	KOG2642	135	130	143	147	216	5	11	10	73	105	151	256	169	145	18	9
ACC_09272	myosuppressin			10	9	15	85	43	2	0	2	21	6	3	2	1	5	3	2
ACC_09273	protein claret segregational	K10405	KOG0239	58	41	15	34	31	6	9	15	32	33	37	27	286	519	427	234
ACC_09274	actin-related protein 2/3 complex subunit 4	K05755	KOG1876	237	156	124	421	272	14	29	22	175	134	56	188	809	741	184	50
ACC_09275	conserved hypothetical protein	K09172	KOG3598	189	188	251	250	21	8	17	14	320	439	27	9	6	7	83	81
ACC_09276	lipoamide acyltransferase component of branched-	K09699	KOG0558	14	6	12	18	20	1	2	1	78	163	48	60	104	171	36	12
ACC_09277	conserved hypothetical protein	K12114	KOG3119	303	168	215	314	67	32	25	64	407	626	193	129	23	47	142	36
ACC_09278	conserved hypothetical protein			661	285	261	446	161	80	137	128	1330	741	827	729	964	463	527	531
ACC_09279	protein singed wings 2-like		KOG4237	5	3	0	0	0	1	3	0	17	21	8	0	70	38	29	16
ACC_09280	putative DNA helicase Ino80-like	K11665	KOG0388	290	167	146	371	268	62	138	128	335	317	167	113	169	207	264	173
ACC_09281	leucine-rich repeat-containing protein 23-like		KOG2123	0	0	0	5	2	0	1	0	1	0	1	3	0	0	0	0
ACC_09282	prolactin regulatory element-binding protein-like	K14003	KOG0771	174	106	71	181	128	18	35	40	451	308	75	350	459	457	81	44
ACC_09283	alpha-tocopherol transfer protein-like isoform 2		KOG1471	202	98	51	213	102	79	119	109	3967	3812	1132	1429	1043	309	222	69
ACC_09284	mTERF domain-containing protein 1, mitochondrial	K15032	KOG1267	157	93	102	117	153	9	16	18	135	125	108	232	308	476	16	8
ACC_09285	venom dipeptidyl peptidase 4 precursor	K01278	KOG2100	583	421	316	790	237	190	199	291	820	1847	28	272	202	299	733	82
ACC_09286	probable ATP-dependent RNA helicase DDX47-like	K14777	KOG0330	395	255	252	529	506	66	152	155	299	198	253	361	711	1176	952	793
ACC_09287	nuclear transcription factor Y subunit gamma-like	K08066	KOG1657	264	246	326	363	174	32	36	53	384	293	128	518	624	730	263	48
ACC_09288	conserved hypothetical protein			33	28	24	54	31	2	6	4	66	83	16	42	40	36	13	5
ACC_09289	LIM/homeobox protein Awh		KOG4577	13	7	11	12	3	1	1	1	6	2	2	0	5	6	5	2

ACC_09290	dnaJ homolog subfamily C member 30-like		KOG0715	102	71	73	87	122	3	9	6	43	43	88	114	79	88	30	11
ACC_09291	conserved hypothetical protein	K14972	KOG1878	480	285	270	424	106	104	200	207	470	609	124	105	122	149	1372	900
ACC_09292	conserved hypothetical protein		KOG3598	413	399	361	281	83	27	45	48	610	611	49	102	239	254	255	128
ACC_09293	furin-like protease 2-like		KOG3526	504	279	374	871	149	65	100	151	657	655	433	194	56	122	295	308
ACC_09294	conserved hypothetical protein			107	43	47	127	102	10	31	21	111	62	77	143	117	162	61	36
ACC_09295	conserved hypothetical protein	K08031	KOG1304	1	1	1	1	1	0	0	0	0	0	0	0	0	0	1	1
ACC_09296	conserved hypothetical protein		KOG0849	14	28	30	38	4	1	0	5	18	17	0	0	18	6	11	18
ACC_09297	translation initiation factor eIF-2B subunit epsilon-l	K03240	KOG1461	166	116	129	142	180	9	15	11	399	344	112	453	485	368	27	3
ACC_09298	bleomycin hydrolase-like	K01372	KOG4128	436	219	171	563	607	38	66	67	1145	754	403	990	1458	1444	87	18
ACC_09299	conserved hypothetical protein		KOG3598	321	1144	187	159	48	448	415	808	5059	3807	2087	4203	682	727	294	184
ACC_09300	WD repeat-containing protein 47-like		KOG1265	59	61	74	122	21	7	6	12	125	168	21	33	8	6	23	2
ACC_09301	synaptic vesicle membrane protein VAT-1 homolog-like			446	298	162	810	382	483	1351	1111	258	258	230	176	121	116	1548	1051
ACC_09302	serine protease easter		KOG3627	120	66	103	433	288	32	50	66	9229	8550	250	686	588	81	17	19
ACC_09303	conserved hypothetical protein		KOG3598	120	64	50	80	56	19	46	54	28	21	40	5	6	8	138	111
ACC_09304	transglutaminase			14	10	6	23	6	1	2	0	169	178	19	63	4	4	4	6
ACC_09305	t-box protein H15-like isoform 1	K10185	KOG3586	2	0	0	3	0	1	0	1	10	5	50	15	2	4	14	11
ACC_09306	major royal jelly protein MRJP1 precursor			13062	374047	7145	1611	4780	330253	420480	843767	2	56	21	25	21	5	0	1
ACC_09307	major royal jelly protein MRJP5			3590	23826	2389	586	1153	14741	5126	13106	11	26	2	8	2	0	0	0
ACC_09308	2-oxoisovalerate dehydrogenase subunit alpha, mi	K00166	KOG1182	121	84	81	148	141	64	62	100	691	591	210	421	870	679	58	14
ACC_09309	l-xylulose reductase		KOG1207	58	51	29	184	181	6	3	6	274	297	66	268	2334	538	41	26
ACC_09310	conserved hypothetical protein			31	24	44	76	34	3	3	6	50	10	20	22	6	7	7	4
ACC_09311	3-hydroxyacyl-CoA dehydrogenase type-2-like	K08683	KOG1199	538	397	282	702	695	84	151	176	753	633	599	1083	27394	27927	2192	1484
ACC_09312	nurim homolog			59	52	45	71	64	4	4	6	39	81	87	187	58	50	4	2
ACC_09313	disco-interacting protein 2			23	13	9	21	5	3	0	3	28	75	28	37	14	6	37	17
ACC_09314	disco-interacting protein 2		KOG3628	76	49	41	48	19	11	4	9	265	417	22	76	106	66	31	15
ACC_09315	conserved hypothetical protein		KOG0161	125	52	61	104	35	19	31	32	301	101	16	49	59	63	58	22
ACC_09316	conserved hypothetical protein			48	23	20	39	18	5	3	6	7	3	1	0	0	3	2	4
ACC_09317	potassium voltage-gated channel protein Shaker-lil	K05318	KOG1545	458	148	128	370	99	23	31	67	258	694	62	18	11	10	40	30
ACC_09318	conserved hypothetical protein			37	13	11	34	10	16	22	19	76	64	63	169	40	26	87	75
ACC_09319	villin-like protein quail		KOG0443	123	44	52	182	150	6	13	16	65	42	294	270	43	70	23	8
ACC_09320	conserved hypothetical protein		KOG3624	176	97	101	176	222	16	22	32	378	635	122	171	344	266	39	21
ACC_09321	conserved hypothetical protein	K04446	KOG3598	528	464	397	363	124	91	65	92	936	893	178	371	812	562	398	64
ACC_09322	conserved hypothetical protein			119	51	83	48	12	0	0	0	35	32	0	0	0	1	1	2
ACC_09323	Voltage-dependent L-type calcium channel subunit alpha-1C		KOG2301	3026	1799	2151	3117	847	201	264	326	1167	880	248	71	6	19	87	39
ACC_09324	sex-regulated protein janus-A-like			27	28	19	44	40	3	2	9	53	19	19	40	98	103	9	7
ACC_09325	conserved hypothetical protein		KOG3598	1079	1022	1219	1240	234	63	104	128	820	617	71	77	210	525	605	224
ACC_09326	conserved hypothetical protein			511	451	582	574	108	32	43	97	378	234	66	43	103	113	480	185
ACC_09327	protein transport protein Sec24C-like	K14007	KOG1984	1437	1193	906	851	608	255	350	378	2400	2446	556	1560	4293	4211	517	168
ACC_09328	putative inorganic phosphate cotransporter-like	K08193	KOG2532	6	1	6	7	2	2	0	0	1	3	1	3	0	0	3	0
ACC_09329	conserved hypothetical protein			408	259	259	551	615	61	129	168	277	219	265	270	331	355	284	153
ACC_09330	conserved hypothetical protein		KOG2591	370	325	195	157	81	39	54	77	795	1482	73	393	1040	794	218	73
ACC_09331	protein unc-50 homolog		KOG3012	108	81	76	136	153	6	8	8	66	50	66	196	186	123	5	4
ACC_09332	bifunctional purine biosynthesis protein PURH-like	K00602	KOG2555	222	137	151	351	394	26	33	36	1106	3009	223	654	1167	1252	128	74
ACC_09333	conserved hypothetical protein			265	252	228	523	353	57	167	145	163	99	142	24	91	174	323	181
ACC_09334	vesicular acetylcholine transporter-like		KOG3764	375	199	281	185	69	9	9	16	181	169	33	31	0	3	3	0
ACC_09335	conserved hypothetical protein	K13096	KOG0965	2296	2013	2232	3974	1969	441	718	962	1303	647	816	176	588	856	306	223
ACC_09336	conserved hypothetical protein			66	49	64	157	113	4	12	4	103	68	9	30	192	280	21	13
ACC_09337	conserved hypothetical protein		KOG1029	904	658	491	631	612	195	483	438	538	296	294	166	568	1195	5388	2565
ACC_09338	Golgi SNAP receptor complex member 1 isoform 1	K08495	KOG3208	109	94	85	176	169	25	32	32	282	137	127	322	239	193	28	14
ACC_09339	conserved hypothetical protein		KOG3598	773	504	552	731	168	59	122	150	330	141	62	18	11	38	205	152
ACC_09340	conserved hypothetical protein		KOG3598	1038	803	1212	824	224	55	99	114	223	157	43	11	15	40	172	150
ACC_09341	conserved hypothetical protein			28	104	34	82	78	5	53	5	345	83	744	1063	157	106	8	6
ACC_09342	conserved hypothetical protein			54	13	13	16	23	1	0	2	58	12	1	13	14	9	3	2
ACC_09343	Bardet-Biedl syndrome 7 protein-like	K16749		33	24	43	42	29	6	10	2	107	35	253	168	37	28	10	2
ACC_09344	chitinase-like protein Idgf4-like		KOG2806	3728	2215	2396	12671	3115	502	492	716	15139	22625	2403	5495	5855	3335	1859	1299
ACC_09345	conserved hypothetical protein		KOG3568	39	14	28	70	14	6	8	10	84	16	8	10	65	100	256	103
ACC_09346	NADH dehydrogenase	K03961	KOG4632	670	339	413	1118	1096	57	76	79	1248	361	746	1855	868	949	86	32

ACC_09347	facilitated trehalose transporter Tret1-like		KOG0254	21	31	35	228	94	3	3	4	104	72	574	560	270	85	23	7
ACC_09348	conserved hypothetical protein			63	37	35	114	58	7	15	16	148	55	71	36	31	28	11	10
ACC_09349	conserved hypothetical protein		KOG0161	4954	5590	5629	5219	3095	1124	2155	2441	3043	2143	955	111	17	142	1483	538
ACC_09350	vesicular glutamate transporter 2.1-like		KOG2532	280	117	98	347	155	4	7	8	211	70	2	61	173	166	54	10
ACC_09351	conserved hypothetical protein		KOG4701	80	13	23	26	9	0	2	5	30	32	3	15	6	10	10	9
ACC_09352	conserved hypothetical protein		KOG3598	5	4	1	0	0	0	0	0	9	3	1	44	2	1	1	1
ACC_09353	conserved hypothetical protein	K09851	KOG4239	199	77	76	144	135	15	13	27	209	171	25	110	94	78	36	6
ACC_09354	small conductance calcium-activated potassium ch	K05325	KOG3684	98	27	22	57	13	0	4	1	27	21	10	1	0	0	3	1
ACC_09355	fibrillin-2-like		KOG1214	506	44	38	139	49	11	12	22	1156	261	65	250	280	245	250	166
ACC_09356	conserved hypothetical protein			36	39	33	56	21	14	18	26	353	415	61	77	168	78	18	17
ACC_09357	conserved hypothetical protein			252	125	97	113	164	6	26	18	259	264	56	403	393	217	18	11
ACC_09358	conserved hypothetical protein		KOG0161	4583	3890	3398	2585	2139	1535	2634	3357	9446	5791	1870	1210	1929	2016	2889	1736
ACC_09359	LOW QUALITY PROTEIN		KOG0161	2371	1089	1058	1166	1028	316	610	677	1976	1688	659	681	1652	1279	845	325
ACC_09360	conserved hypothetical protein		KOG0943	938	648	959	1081	398	112	193	227	1182	412	236	61	93	156	437	257
ACC_09361	short spindle protein 4-like isoform 1		KOG3654	191	123	86	183	91	39	81	79	373	267	63	41	70	109	369	265
ACC_09362	tetraspanin-11-like		KOG3882	720	112	113	615	691	40	68	95	200	71	22	120	5	21	41	15
ACC_09363	conserved hypothetical protein		KOG4818	2	0	2	6	2	0	0	0	4	4	1	0	682	1172	101	1
ACC_09364	conserved hypothetical protein			264	134	139	356	177	19	48	55	149	43	134	107	5	38	120	26
ACC_09365	recQ-mediated genome instability protein 1-like		KOG3683	327	194	219	269	419	32	68	65	184	131	276	267	191	179	66	42
ACC_09366	conserved hypothetical protein		KOG2039	638	406	252	432	426	182	399	366	708	757	286	226	537	478	782	397
ACC_09367	conserved hypothetical protein		KOG2465	430	244	312	801	166	55	69	116	413	391	109	97	112	126	407	307
ACC_09368	conserved hypothetical protein		KOG3209	247	84	108	235	54	7	15	13	92	101	19	8	2	6	30	45
ACC_09369	conserved hypothetical protein		KOG2308	454	237	217	561	226	61	75	101	1489	1533	400	779	491	531	255	45
ACC_09370	casein kinase I isoform gamma-3	K08958	KOG1165	244	217	164	186	60	27	22	62	270	242	47	57	109	157	109	29
ACC_09371	battenin	K12389	KOG3880	348	116	91	196	79	111	161	197	473	398	109	101	185	115	214	231
ACC_09372	conserved hypothetical protein			37	17	41	75	50	2	1	4	17	15	60	88	45	44	13	8
ACC_09373	latrophilin Cirl-like isoform 1			148	33	40	108	24	5	6	18	16	38	23	5	1	4	81	44
ACC_09374	conserved hypothetical protein	K09100	KOG3559	250	247	297	235	61	11	15	25	82	35	1	0	1	7	25	20
ACC_09375	proto-oncogene tyrosine-protein kinase ROS	K05088	KOG1095	925	321	339	493	391	32	35	53	805	1169	363	299	268	221	64	31
ACC_09376	conserved hypothetical protein	K13156		538	482	511	462	422	68	148	119	443	227	456	177	254	412	142	62
ACC_09377	cytochrome P450 306a1	K10720	KOG0156	26	40	64	98	34	10	8	9	20	28	29	15	38	59	70	75
ACC_09378	conserved hypothetical protein		KOG0123	96	48	62	126	20	6	4	15	103	159	41	4	19	15	27	39
ACC_09379	putative methyltransferase NSUN4-like		KOG2198	158	89	85	316	358	25	32	43	263	154	117	138	341	469	106	37
ACC_09380	dnaJ homolog subfamily B member 6-like			201	162	209	552	336	37	100	113	260	180	300	199	151	148	158	70
ACC_09381	chemosensory protein 2 precursor			829	292	359	1153	282	65	74	121	564	96	113	768	172	396	467	643
ACC_09382	UPF0687 protein C20orf27 homolog			34	32	19	30	31	3	5	5	19	23	14	32	66	84	20	4
ACC_09383	conserved hypothetical protein		KOG1418	310	221	264	200	72	10	23	25	540	263	23	60	28	13	18	13
ACC_09384	conserved hypothetical protein			11	8	11	39	15	6	1	5	44	15	18	3	3	3	0	3
ACC_09385	conserved hypothetical protein			0	0	0	0	0	0	0	0	1	0	30	68	12	3	0	0
ACC_09386	UPF0465 protein C5orf33-like		KOG4180	0	0	1	0	0	0	0	0	0	0	0	0	0	0	0	0
ACC_09387	protein expanded-like	K16683	KOG4371	172	217	185	119	49	9	17	18	237	171	56	65	107	209	122	31
ACC_09388	dual specificity tyrosine-phosphorylation-regulated	K08825	KOG0667	425	235	230	278	90	37	38	74	310	376	57	156	84	88	148	76
ACC_09389	conserved hypothetical protein		KOG0119	1010	624	543	666	424	154	334	350	1197	740	358	198	289	276	412	178
ACC_09390	netrin-B-like, partial		KOG3512	49	21	15	14	12	10	9	8	49	21	2	7	3	6	32	27
ACC_09391	LOW QUALITY PROTEIN			41	27	25	88	75	1	11	8	31	47	28	20	45	68	5	2
ACC_09392	UDP-glucuronosyltransferase 1-3-like	K00699	KOG1192	60	47	51	192	72	21	34	25	3643	3364	122	281	217	511	175	40
ACC_09393	probable phospholipid-transporting ATPase IIB-like			7	8	5	11	2	1	2	2	13	7	22	13	5	0	1	0
ACC_09394	pancreatic triacylglycerol lipase-like			122	90	104	316	313	11	12	22	74	34	55	8	46	165	8	2
ACC_09395	conserved hypothetical protein		KOG0260	148	117	138	350	127	45	67	59	241	249	164	194	8238	23884	9241	400
ACC_09396	snRNA-activating protein complex subunit 4	K09453	KOG0049	856	529	529	1065	1311	58	203	199	503	340	519	340	189	118	97	35
ACC_09397	LOW QUALITY PROTEIN	K16734	KOG1860	143	76	93	173	188	15	28	31	135	80	123	281	292	274	42	18
ACC_09398	agrin-like		KOG4578	598	324	459	1580	627	35	26	51	521	421	59	347	66	317	91	41
ACC_09399	conserved hypothetical protein			202	185	204	290	280	7	133	123	107	69	269	367	294	350	160	124
ACC_09400	phosrestin-2-like	K13808	KOG3865	68	74	82	1718	374	490	472	984	2303	1852	3	94	0	1	4	1
ACC_09401	crossover junction endonuclease EME1-like	K10882	KOG2379	727	343	269	661	942	51	143	147	302	335	514	843	788	758	326	138
ACC_09402	vinculin-like isoform 2		KOG3681	186	142	113	202	133	21	20	27	869	861	84	334	814	681	78	45
ACC_09403	ADIPOR-like receptor CG5315-like	K07297	KOG0748	167	106	77	128	144	17	11	14	902	523	203	633	680	487	41	5

ACC_09404	chymotrypsin inhibitor-like			112	117	42	11	28	56	82	126	13	8	1	0	19	23	13	1
ACC_09405	conserved hypothetical protein			1	0	0	0	0	0	0	0	1	0	0	0	0	0	0	0
ACC_09406	conserved hypothetical protein		KOG3882	3	0	0	2	0	0	1	1	3	6	1	0	0	0	1	0
ACC_09407	LOW QUALITY PROTEIN		KOG3595	1	1	1	5	2	0	0	0	2	2	0	0	1	0	1	0
ACC_09408	LOW QUALITY PROTEIN			145	113	100	112	32	20	49	42	344	511	29	184	330	304	95	81
ACC_09409	chitooligosaccharidolytic beta-N-acetylglucosamini	K12373	KOG2499	69	91	86	266	295	14	19	31	151	197	100	231	935	176	27	20
ACC_09410	arginine-glutamic acid dipeptide repeats protein-like		KOG3554	0	0	0	2	1	0	1	1	0	0	0	0	11	8	4	1
ACC_09411	RNA-binding protein 39-like	K13091	KOG0147	556	288	274	416	295	28	27	28	627	505	200	401	584	858	185	46
ACC_09412	calsyntenin-1-like		KOG1834	1599	690	682	847	284	51	45	57	1408	1532	247	478	810	494	236	74
ACC_09413	conserved hypothetical protein			313	199	193	160	224	10	34	27	102	166	125	316	752	786	94	28
ACC_09414	protein gooseberry-like	K09381	KOG3517	0	0	0	0	0	0	0	0	0	0	0	0	3	3	1	1
ACC_09415	zinc finger BED domain-containing protein 4-like		KOG1121	0	0	0	0	1	0	0	0	0	0	0	0	101	80	13	3
ACC_09416	diablo isoform 1	K10457	KOG4441	113	62	40	128	78	7	1	5	158	133	45	106	131	90	23	7
ACC_09417	probable chitinase 3-like	K01183	KOG2806	224	21	12	66	25	52	77	114	392	120	23	686	319	511	278	180
ACC_09418	dynein intermediate chain 3, ciliary-like	K11143	KOG1587	20	59	59	63	20	15	48	41	90	65	95	3	5	22	189	118
ACC_09419	multiple inositol polyphosphate phosphatase 1-like		KOG1382	6	5	1	1	1	3	5	4	13	4	0	2	0	1	2	2
ACC_09420	Longitudinals lacking protein, isoforms F/I/K/T		KOG4441	1503	945	881	1674	646	150	185	262	1136	979	438	615	505	527	813	549
ACC_09421	peroxidasin-like isoform 1		KOG4221	90	62	72	195	39	7	10	5	63	15	5	1	28	31	39	4
ACC_09422	visual system homeobox 1-like		KOG0494	57	35	37	32	15	1	4	7	38	7	3	0	1	4	8	9
ACC_09423	conserved hypothetical protein			1	0	1	4	1	0	0	2	40	21	92	12	2426	819	2539	1893
ACC_09424	LOW QUALITY PROTEIN	K04157	KOG4220	347	226	293	364	296	49	96	78	248	129	639	225	147	209	224	144
ACC_09425	conserved hypothetical protein			19	18	26	29	18	1	3	0	4	3	16	12	21	41	11	9
ACC_09426	mitochondrial folate transporter/carrier-like		KOG0764	89	69	97	184	140	15	21	37	83	76	30	31	224	101	35	14
ACC_09427	kinesin B		KOG0161	525	416	393	309	293	70	155	153	351	259	109	132	258	342	324	115
ACC_09428	conserved hypothetical protein			12	11	37	31	21	3	3	4	38	45	5	11	10	46	11	3
ACC_09429	delta(3,5)-Delta(2,4)-dienoyl-CoA isomerase, mitoc	K12663	KOG1681	126	98	119	152	181	9	21	28	245	164	305	579	539	521	21	11
ACC_09430	sorting nexin-24-like isoform 1			145	67	68	116	88	44	54	60	94	51	46	26	8	33	52	5
ACC_09431	glucose dehydrogenase		KOG1238	92	31	28	186	96	10	18	9	260	120	127	70	215	116	34	4
ACC_09432	conserved hypothetical protein			0	0	2	2	3	0	0	0	0	1	0	0	0	0	0	0
ACC_09433	retinol dehydrogenase 14-like		KOG1208	18	2	5	26	9	6	6	11	163	49	97	457	61	133	54	129
ACC_09434	conserved hypothetical protein		KOG1726	266	163	185	197	69	24	33	56	396	319	51	110	43	22	46	10
ACC_09435	lactosylceramide 4-alpha-galactosyltransferase-like, partia		KOG1928	27	4	8	19	7	2	3	5	17	16	35	85	16	1	0	4
ACC_09436	lactosylceramide 4-alpha-galactosyltransferase-like		KOG1928	1	0	0	0	0	0	0	0	0	1	0	1	41	44	2	0
ACC_09437	RING finger protein nhl-1-like	K12035	KOG2177	1381	633	647	1403	1104	234	513	505	4236	4273	316	83	179	141	292	203
ACC_09438	cytochrome P450 6j1-like isoform 1		KOG0158	37	19	24	70	40	13	17	20	76	120	39	59	89	58	15	3
ACC_09439	high-affinity choline transporter 1-like	K14387	KOG3761	1470	497	583	1014	366	20	51	52	603	409	137	132	0	3	2	1
ACC_09440	anosmin-1-like		KOG4802	7	5	10	7	4	0	0	0	17	8	1	0	55	83	14	2
ACC_09441	DNA polymerase subunit gamma-2, mitochondrial-	K02333	KOG2298	626	433	399	546	542	79	200	193	504	364	446	950	1499	1643	300	139
ACC_09442	general transcription factor IIF subunit 1-like	K03138	KOG2393	415	337	303	353	371	159	292	302	589	238	315	245	435	647	910	617
ACC_09443	integrin-linked protein kinase-like	K06272	KOG0195	160	73	99	272	220	14	16	16	400	423	116	422	631	516	44	5
ACC_09444	carboxylesterase		KOG1516	1	3	2	19	11	2	1	0	94	200	1	18	2143	1521	130	17
ACC_09445	LOW QUALITY PROTEIN		KOG3513	1236	786	289	497	127	714	1107	823	49669	29764	459	477	495	361	719	801
ACC_09446	LOW QUALITY PROTEIN		KOG4221	412	207	80	214	93	218	318	222	22174	9145	158	86	546	300	77	34
ACC_09447	protein RRNAD1-like		KOG2651	35	40	39	65	68	5	10	12	35	74	52	106	115	107	15	8
ACC_09448	calcium channel flower-like isoform 1			18	6	6	3	5	0	0	0	5	2	0	6	7	5	0	0
ACC_09449	calcitonin receptor-like			26	12	11	38	29	2	2	0	31	47	25	36	17	12	4	4
ACC_09450	ecdysone-induced protein 75B, isoforms C/D-like is	K08701	KOG4216	609	797	1404	1777	636	480	1010	902	996	1642	872	155	232	447	1829	1576
ACC_09451	conserved hypothetical protein			660	465	210	75	60	225	525	691	19	62	1	4	59	262	289	106
ACC_09452	malonyl-CoA decarboxylase, mitochondrial-like	K01578	KOG3018	363	266	247	323	428	17	54	60	409	330	317	730	515	542	50	20
ACC_09453	Cell death activator CIDE-B			324	183	170	279	61	28	21	59	162	76	30	17	4	12	34	30
ACC_09454	conserved hypothetical protein		KOG3607	1855	847	921	1459	466	101	112	161	749	398	59	28	15	27	89	46
ACC_09455	protein phosphatase PTC7 homolog		KOG1379	82	44	33	162	131	8	12	15	113	68	43	100	77	95	26	7
ACC_09456	procollagen-lysine,2-oxoglutarate 5-dioxygenase 3-like		KOG1971	175	77	87	127	112	6	16	14	481	363	85	327	607	448	25	5
ACC_09457	LOW QUALITY PROTEIN			146	26	32	189	29	72	70	109	1265	689	557	3176	113	2	2	3
ACC_09458	conserved hypothetical protein			4	0	1	8	6	3	13	8	52	8	0	1	0	0	1	1
ACC_09459	conserved hypothetical protein		KOG0161	354	272	190	270	133	82	100	122	1200	599	203	605	525	533	374	197
ACC_09460	methyltransferase-like protein 9-like			31	28	28	41	6	1	6	6	37	62	15	168	26	17	22	7

ACC_09461	homogentisate 1,2-dioxygenase-like		KOG1417	1	1	0	1	2	0	0	0	19	20	6	42	18	16	3	0
ACC_09462	conserved hypothetical protein			47	31	13	102	19	98	149	212	39	140	92	37	16	24	833	606
ACC_09463	conserved hypothetical protein		KOG1512	3023	1949	1549	2324	1519	516	803	1036	2500	2529	754	775	1067	1038	1013	402
ACC_09464	paired box protein Pax-1	K09382	KOG3517	9	1	0	4	7	1	3	2	23	11	2	0	20	12	44	40
ACC_09465	heat shock factor protein-like	K09418	KOG0627	962	661	594	687	506	81	138	130	1570	1712	430	522	1063	893	254	120
ACC_09466	pseudouridine-metabolizing bifunctional protein C1861.05-like			41	36	35	135	76	4	3	5	53	29	24	42	49	42	14	2
ACC_09467	myosin-IXb isoform 1	K16677	KOG0164	17	10	14	18	3	1	1	0	35	75	4	5	25	30	60	38
ACC_09468	LOW QUALITY PROTEIN	K09614	KOG0161	81	29	22	40	10	9	19	28	12	33	5	2	7	5	18	12
ACC_09469	lipoma HMGIC fusion partner-like 2 protein-like		KOG4026	7	6	6	11	4	0	1	2	18	8	21	11	25	97	455	289
ACC_09470	conserved hypothetical protein		KOG0818	102	72	83	92	42	9	10	15	326	228	37	107	132	221	237	54
ACC_09471	conserved hypothetical protein		KOG0962	2307	1471	1029	1468	2287	90	253	277	694	1066	788	567	572	441	603	339
ACC_09472	transient receptor potential protein	K05328	KOG3609	246	328	285	472	307	1193	2407	2640	2382	1021	11	128	76	317	534	413
ACC_09473	conserved hypothetical protein	K09680	KOG2201	191	143	192	182	197	14	29	28	534	428	316	630	560	498	45	16
ACC_09474	protein shifted isoform 3	K01691	KOG1225	163	149	145	199	165	16	45	68	46	18	68	69	72	61	32	7
ACC_09475	transcription factor IIIB 90 kDa subunit-like isoform	K15196	KOG1598	477	432	416	455	491	140	351	306	438	264	284	179	373	364	305	205
ACC_09476	erlin-1-like		KOG2962	382	274	282	392	372	14	53	57	432	245	213	837	503	559	92	71
ACC_09477	poly(U)-binding-splicing factor half pint-like	K12838	KOG0124	2410	1223	1317	1353	813	266	422	368	1781	817	1055	452	1193	1948	998	425
ACC_09478	calcium-independent protein kinase C-like isoform	K06068	KOG0694	1210	673	651	1542	1207	86	139	182	633	333	142	77	33	30	55	19
ACC_09479	multidrug resistance-associated protein 1-like		KOG0054	1356	864	841	1274	1344	104	156	208	2235	1420	841	805	1631	2035	231	87
ACC_09480	flexible cuticle protein 12			0	0	0	2	2	0	0	0	15	1	4	32	7889	2656	1101	2398
ACC_09481	dystrobrevin beta isoform 1		KOG4301	367	391	440	647	257	44	68	70	1022	664	141	191	148	181	103	52
ACC_09482	conserved hypothetical protein		KOG1028	452	270	293	582	226	78	86	117	1173	817	400	529	283	424	292	66
ACC_09483	mediator of RNA polymerase II transcription subun	K15159		410	270	254	626	546	41	55	64	559	320	146	249	339	431	38	16
ACC_09484	conserved hypothetical protein		KOG1703	2252	760	331	308	106	713	1277	1120	8759	3871	269	206	171	163	441	547
ACC_09485	methyltransferase-like protein 4-like		KOG2356	141	115	113	158	140	4	27	26	117	49	58	110	121	115	22	14
ACC_09486	vacuolar protein sorting-associated protein 53 homolog		KOG2180	451	287	293	609	549	57	63	67	593	486	255	636	465	425	55	27
ACC_09487	conserved hypothetical protein		KOG3993	368	280	264	213	423	48	117	158	177	394	436	420	339	767	808	353
ACC_09488	ftsJ methyltransferase domain-containing protein 1-like		KOG1800	286	204	215	257	344	19	42	37	418	250	151	318	384	358	24	10
ACC_09489	transmembrane and coiled-coil domains protein 1-like		KOG3850	643	322	231	460	243	85	132	142	1128	610	710	1445	532	169	125	62
ACC_09490	conserved hypothetical protein		KOG2462	80	54	37	65	23	10	11	12	99	52	45	27	49	40	50	16
ACC_09491	conserved hypothetical protein		KOG2462	300	172	163	256	101	39	56	51	446	225	90	42	111	110	209	108
ACC_09492	conserved hypothetical protein	K16831		40	53	45	42	49	5	8	10	31	19	32	19	108	147	54	17
ACC_09493	charged multivesicular body protein 2a-like	K12191	KOG3230	331	188	182	286	260	32	70	66	526	222	197	437	274	304	92	84
ACC_09494	slit homolog 3 protein-like		KOG4194	30	139	40	96	56	10	11	14	4160	2576	5	2	908	16	5	3
ACC_09495	microtubule-associated protein RP/EB family meml	K10436	KOG3000	490	240	239	505	360	26	33	32	453	400	155	483	803	1027	188	51
ACC_09496	prohibitin-2-like		KOG3090	486	420	356	903	662	76	106	129	543	362	523	772	2931	5423	886	527
ACC_09497	conserved hypothetical protein		KOG1874	248	112	124	706	275	30	25	18	545	28	31	2458	17	7	9	9
ACC_09498	pre-mRNA-splicing factor 38B-like	K12850	KOG2888	640	513	517	726	520	96	154	229	900	461	500	372	669	1061	354	266
ACC_09499	e3 ubiquitin-protein ligase MARCH8-like	K10656	KOG1609	378	287	281	725	442	33	39	51	399	274	174	443	447	432	107	26
ACC_09500	adenosine kinase-like isoform 2	K00856	KOG2854	44	39	35	51	43	1	4	10	60	37	61	76	29	26	3	2
ACC_09501	conserved hypothetical protein			57	16	21	34	28	3	10	3	57	22	29	3	39	13	23	15
ACC_09502	conserved hypothetical protein		KOG0712	328	164	196	242	270	85	126	146	536	359	221	290	307	365	257	106
ACC_09503	conserved hypothetical protein	K11252	KOG1744	258	342	185	521	218	94	69	165	250	151	103	245	681	406	625	678
ACC_09504	cadherin-related tumor suppressor	K16669	KOG1219	7	11	20	34	13	4	3	6	14	26	9	12	34	58	108	56
ACC_09505	rhophilin-2-like		KOG1924	1108	806	713	1355	981	214	294	345	2019	1524	687	934	1038	884	298	205
ACC_09506	AP-3 complex subunit delta-1	K12396	KOG1059	779	521	317	927	861	207	451	522	822	852	380	624	695	512	404	181
ACC_09507	conserved hypothetical protein			389	251	237	292	385	55	87	129	990	340	244	280	239	208	90	38
ACC_09508	conserved hypothetical protein		KOG2494	577	350	457	333	54	26	38	58	349	368	81	47	35	62	329	153
ACC_09509	protein kinase C iota type	K06069	KOG0695	755	557	480	863	550	78	115	153	685	736	253	514	559	1032	520	189
ACC_09510	sperm-associated antigen 6-like isoform 1		KOG0166	40	25	28	29	28	3	3	3	21	11	11	15	14	17	4	1
ACC_09511	mediator of RNA polymerase II transcription subun	K15157	KOG4274	336	237	230	357	226	26	48	50	397	288	196	356	551	710	176	102
ACC_09512	TBC1 domain family member 24-like isoform 2		KOG2801	288	100	82	116	77	11	18	23	470	264	40	164	43	53	17	2
ACC_09513	conserved hypothetical protein			1628	1113	870	1280	1005	654	1063	1375	2374	2257	1291	929	292	307	280	85
ACC_09514	peptide deformylase, mitochondrial-like	K01462	KOG3137	216	120	143	288	338	16	36	42	446	251	268	506	699	1057	56	36
ACC_09515	putative inorganic phosphate cotransporter-like	K08193	KOG2532	172	258	302	758	415	17	20	20	1210	561	67	80	2	3	2	1
ACC_09516	ubiquitin conjugation factor E4 A-like	K10596	KOG2042	1114	654	547	1071	1033	91	139	149	1266	1165	496	946	1519	1536	216	62
ACC_09517	dual oxidase	K13411	KOG0039	7	8	7	40	30	6	3	11	109	59	25	23	180	425	52	20

ACC_09518	NMDA receptor 1	K05208	KOG4440	1078	534	604	1453	947	56	77	78	523	232	31	7	20	39	6	4
ACC_09519	UPF0547 protein C16orf87 homolog			241	140	121	154	133	34	70	81	211	121	138	146	104	164	109	64
ACC_09520	neurexin-4, partial		KOG3516	1057	611	532	676	604	92	110	123	2516	1444	372	870	1066	1121	121	26
ACC_09521	conserved hypothetical protein		KOG0585	264	76	96	85	27	4	12	10	361	467	48	114	64	80	32	19
ACC_09522	dynamin-like isoform 1	K01528	KOG0446	3393	1521	1380	1933	1144	100	121	172	1672	1285	234	366	419	492	153	60
ACC_09523	protein KRI1 homolog	K14786	KOG2409	863	662	539	788	893	171	457	538	309	298	451	329	492	849	1272	787
ACC_09524	conserved hypothetical protein			199	156	127	246	293	18	37	46	138	143	201	119	248	239	147	70
ACC_09525	RING finger protein 17-like		KOG2039	385	196	173	231	217	43	80	66	316	273	145	158	518	435	182	87
ACC_09526	NHL repeat-containing protein 2			1094	722	766	908	934	142	206	244	1205	555	533	729	727	731	91	38
ACC_09527	conserved hypothetical protein	K14763	KOG2236	467	392	348	568	571	54	87	96	361	302	222	306	580	870	344	138
ACC_09528	putative ATP-dependent RNA helicase DHX57-like		KOG0922	1200	584	503	956	1193	114	329	300	684	704	631	718	883	1011	766	401
ACC_09529	conserved hypothetical protein			2	2	3	2	3	0	1	0	2	0	0	1	16	13	0	0
ACC_09530	conserved hypothetical protein	K13765	KOG3589	465	235	230	307	104	16	27	36	526	430	72	121	39	67	47	27
ACC_09531	conserved hypothetical protein		KOG0759	18	13	13	32	25	1	3	2	33	26	95	81	17	12	24	21
ACC_09532	traB domain-containing protein-like		KOG2860	545	340	286	427	531	85	156	126	2211	1122	222	819	1397	1192	94	22
ACC_09533	conserved hypothetical protein		KOG0670	3930	2571	2112	2800	2135	820	1782	1871	2754	1524	1769	684	2662	4784	14473	13238
ACC_09534	LOW QUALITY PROTEIN	K03125	KOG0008	983	642	608	909	708	152	280	332	1220	1156	519	578	740	711	548	308
ACC_09535	monocarboxylate transporter 9-like		KOG2504	476	151	182	91	108	41	108	83	684	223	158	127	210	336	98	7
ACC_09536	conserved hypothetical protein			75	32	31	33	56	4	1	4	35	45	20	71	111	85	7	2
ACC_09537	conserved hypothetical protein	K14398	KOG4849	488	359	416	420	132	39	67	62	479	649	175	378	576	993	824	500
ACC_09538	conserved hypothetical protein			36	23	38	37	5	1	2	2	6	100	12	3	0	4	15	6
ACC_09539	chromatin assembly factor 1 subunit A-B	K10750	KOG4364	587	450	386	597	511	129	264	287	403	191	233	104	509	1066	2294	1689
ACC_09540	ADAMTS-like protein 3-like		KOG3539	35	41	64	42	32	1	3	3	22	13	10	70	27	26	4	0
ACC_09541	conserved hypothetical protein	K11320	KOG0391	804	622	473	417	241	124	212	253	916	1046	155	198	1437	1820	1154	554
ACC_09542	conserved hypothetical protein			187	120	150	317	126	58	94	107	538	421	193	290	78	147	246	82
ACC_09543	conserved hypothetical protein	K13131	KOG4284	53	48	51	70	63	2	7	8	48	66	23	45	86	124	35	16
ACC_09544	conserved hypothetical protein			4	4	1	7	2	0	0	0	3	3	3	2	0	1	1	2
ACC_09545	conserved hypothetical protein		KOG0500	208	130	147	138	30	7	7	15	260	131	3	1	5	7	5	5
ACC_09546	conserved hypothetical protein		KOG4441	113	75	90	151	89	11	7	15	137	82	58	91	97	117	34	10
ACC_09547	segmentation protein cap'n'collar-like	K09041	KOG3863	245	215	151	155	86	102	90	129	1264	1980	544	1784	1390	917	371	137
ACC_09548	conserved hypothetical protein		KOG3514	1732	965	961	877	623	161	298	330	1731	1401	344	557	931	1617	2172	1488
ACC_09549	dyslexia-associated protein KIAA0319-like protein-like		KOG4367	573	308	322	513	483	60	76	103	690	640	309	398	789	634	83	41
ACC_09550	conserved hypothetical protein			25	10	7	33	32	0	0	1	6	7	31	62	1	1	0	0
ACC_09551	RNA-binding protein Musashi homolog Rbp6-like	K14411	KOG4205	1491	753	1007	1304	296	82	123	164	349	254	261	81	8	32	180	85
ACC_09552	conserved hypothetical protein		KOG2548	1014	1497	1084	1007	245	120	81	164	865	245	43	58	62	127	7	31
ACC_09553	rho guanine nucleotide exchange factor 12		KOG3520	1053	746	689	683	425	120	172	175	3082	2107	329	756	931	697	202	70
ACC_09554	transient receptor potential channel pyrexia		KOG0510	232	174	200	635	372	30	31	54	286	220	150	216	243	390	156	55
ACC_09555	conserved hypothetical protein		KOG1052	41	29	28	81	21	6	8	8	110	102	228	1251	13	16	15	29
ACC_09556	endocuticle structural glycoprotein SgAbd-8			74	4	3	23	6	14	6	6	599	421	306	1477	27810	5479	4338	4932
ACC_09557	tight junction protein ZO-1		KOG3580	436	305	250	366	244	174	270	267	1312	589	268	334	267	350	199	56
ACC_09558	n-alpha-acetyltransferase 25, NatB auxiliary subunit		KOG2053	132	116	94	173	143	10	14	11	189	197	50	198	457	491	87	26
ACC_09559	radial spoke head 10 homolog B-like		KOG0231	1	1	0	1	0	0	0	1	2	0	2	0	1	1	1	1
ACC_09560	LOW QUALITY PROTEIN		KOG3595	147	118	141	171	212	12	17	20	239	273	161	113	67	70	24	11
ACC_09561	conserved hypothetical protein		KOG1913	974	907	692	734	379	137	278	317	1586	2173	254	525	1297	897	427	193
ACC_09562	nuclear migration protein nudC-like		KOG2265	387	158	135	583	308	92	168	184	261	190	293	530	949	1267	3160	3887
ACC_09563	conserved hypothetical protein		KOG2977	1369	983	827	916	816	282	542	629	1031	833	488	348	823	864	648	308
ACC_09564	conserved hypothetical protein		KOG3237	173	204	193	223	153	29	49	54	158	202	92	134	308	472	170	120
ACC_09565	Basement membrane-specific heparan sulfate proteoglycan		KOG3509	1849	500	453	445	312	63	91	90	2402	1127	313	1203	3043	2648	797	278
ACC_09566	conserved hypothetical protein		KOG3665	3400	1965	1937	3714	2530	868	1738	1826	4293	1686	1947	1358	2155	3782	13503	12675
ACC_09567	conserved hypothetical protein		KOG0509	235	127	173	227	88	33	68	83	168	53	16	86	109	65	106	139
ACC_09568	translation initiation factor eIF-2B subunit alpha-lik	K03239	KOG1466	104	61	52	122	106	6	21	38	81	59	73	105	123	165	39	24
ACC_09569	conserved hypothetical protein		KOG0995	307	223	204	249	265	51	72	86	456	267	240	335	620	650	290	138
ACC_09570	peroxisomal N(1)-acetyl-spermine/spermidine oxidase-like		KOG0685	634	385	309	614	603	40	72	68	257	393	88	140	1471	1565	150	46
ACC_09571	conserved hypothetical protein	K11310	KOG1015	2837	1835	1614	2213	2956	332	871	1017	1879	1906	1390	1268	1324	1399	887	416
ACC_09572	conserved hypothetical protein			165	117	95	161	212	9	26	27	177	166	211	280	189	188	52	21
ACC_09573	similar to nucleolar protein c7b		KOG2372	1828	1163	998	1168	540	323	555	715	2551	1988	419	587	758	428	1118	678
ACC_09574	conserved hypothetical protein		KOG4383	495	276	214	292	224	18	29	44	530	331	81	91	222	171	38	15

ACC_09575	conserved hypothetical protein		KOG0092	200	83	104	112	116	16	23	15	285	164	206	704	216	201	27	9
ACC_09576	ubiquinone biosynthesis monooxygenase COQ6-lik	K06126	KOG3855	376	208	203	391	432	25	38	40	672	254	333	801	507	658	50	28
ACC_09577	MutS protein-like protein 5	K08741	KOG0221	8	2	3	14	5	1	2	1	13	70	2	6	9	11	10	15
ACC_09578	LOW QUALITY PROTEIN		KOG0614	176	285	211	105	52	165	175	197	366	167	220	171	440	801	182	22
ACC_09579	conserved hypothetical protein			112	274	322	465	330	6	18	19	79	60	116	105	0	0	0	0
ACC_09580	Mitogen-activated protein kinase kinase kinase 15	K04426	KOG4279	324	242	249	190	143	17	17	30	1160	762	203	214	606	508	52	12
ACC_09581	CLIP-associating protein 1-A isoform 1	K16578	KOG2956	783	481	362	456	240	90	130	165	1465	1060	196	359	479	380	151	50
ACC_09582	conserved hypothetical protein		KOG0996	601	353	391	806	475	125	190	278	558	422	271	173	269	391	837	450
ACC_09583	conserved hypothetical protein		KOG3545	869	637	647	892	925	87	149	160	779	431	507	260	582	580	108	57
ACC_09584	histone-lysine N-methyltransferase setd3-like		KOG1337	429	279	283	313	204	27	32	48	359	345	73	325	313	323	105	17
ACC_09585	lysosome membrane protein 2		KOG3776	12	9	5	13	2	3	1	3	717	339	1794	2177	1	89	18	17
ACC_09586	ribose-phosphate pyrophosphokinase 1-like isoforr	K00948	KOG1448	196	133	118	274	248	16	32	37	854	2152	72	334	774	660	115	37
ACC_09587	conserved hypothetical protein		KOG0548	75	69	46	169	98	9	13	14	186	82	45	86	113	109	60	18
ACC_09588	dual specificity mitogen-activated protein kinase ki	K04368	KOG0581	152	110	115	102	88	9	34	32	110	145	36	84	95	102	52	28
ACC_09589	protein lunapark-B-like		KOG2846	337	246	215	314	239	43	58	57	523	335	120	299	469	433	126	66
ACC_09590	cytoplasmic dynein 1 intermediate chain	K10415	KOG1587	1510	659	731	1714	710	117	197	264	1003	698	422	341	569	678	599	364
ACC_09591	deoxyhypusine hydroxylase-like	K06072	KOG0567	310	236	295	349	378	32	55	48	283	161	171	313	281	382	67	35
ACC_09592	codanin-1-like			322	178	195	300	360	19	36	32	427	347	123	173	231	264	14	5
ACC_09593	tubulin polyglutamylase TTLL4-like		KOG2156	13	21	19	24	11	4	4	3	58	22	15	11	13	41	50	8
ACC_09594	conserved hypothetical protein		KOG4701	166	138	174	154	109	19	28	22	156	240	38	167	331	1148	319	169
ACC_09595	serine/threonine-protein kinase mig-15	K04413	KOG0587	438	328	305	168	91	32	33	39	757	701	98	284	308	295	111	48
ACC_09596	phosphatidylinositol-4,5-bisphosphate 3-kinase cat	K00922	KOG0904	424	300	247	506	343	56	76	77	459	419	188	206	426	402	103	15
ACC_09597	conserved hypothetical protein		KOG3531	1646	702	731	1776	720	125	190	241	1186	839	748	591	804	610	251	135
ACC_09598	conserved hypothetical protein			204	132	120	238	145	33	33	37	239	135	129	163	111	183	39	32
ACC_09599	26S proteasome non-ATPase regulatory subunit 6-like		KOG0687	78	64	58	190	204	4	12	24	72	82	112	150	193	257	21	12
ACC_09600	putative methyltransferase NSUN5-like	K15264	KOG2360	676	527	465	943	677	73	120	168	453	482	386	687	1281	1665	750	330
ACC_09601	catenin delta-2-like		KOG1048	227	193	192	309	99	44	53	85	257	229	91	77	141	267	117	69
ACC_09602	HIV Tat-specific factor 1 homolog	K13093	KOG1548	516	341	396	523	377	85	94	113	487	227	205	273	287	312	247	134
ACC_09603	UDP-N-acetylglucosamine--dolichyl-phosphate N-a	K01001	KOG2788	111	63	71	164	179	3	4	3	61	80	52	162	222	232	8	3
ACC_09604	conserved hypothetical protein		KOG0161	883	239	242	256	89	38	49	62	659	536	89	93	94	145	134	111
ACC_09605	synaptic vesicle glycoprotein 2B-like	K06258	KOG3288	285	183	150	562	426	27	46	67	1024	889	367	1366	1362	2311	732	88
ACC_09606	syntaxin-binding protein 5 isoform 1	K08518	KOG1983	1651	844	833	1388	459	79	130	184	1068	734	207	142	80	107	101	61
ACC_09607	cGMP-dependent protein kinase 1 foraging	K07376	KOG0614	198	121	111	273	151	30	33	43	1083	746	311	760	563	813	234	48
ACC_09608	conserved hypothetical protein	K08064	KOG1561	217	156	128	253	175	33	39	66	227	220	105	230	390	375	253	124
ACC_09609	conserved hypothetical protein			337	249	246	552	523	57	90	118	1023	421	1375	2401	587	556	133	133
ACC_09610	conserved hypothetical protein	K04459	KOG1717	286	193	184	246	225	15	27	44	438	260	179	554	178	145	18	11
ACC_09611	Dihydrolipoyllysine-residue acetyltransferase comp	K00627	KOG0557	449	199	190	250	195	28	52	50	1746	809	217	718	2235	1942	241	113
ACC_09612	conserved hypothetical protein			32	22	11	41	22	2	9	5	19	22	9	3	24	10	18	20
ACC_09613	derlin-1-like	K11519	KOG1631	496	307	159	428	495	48	54	76	664	373	359	1188	1401	1555	99	27
ACC_09614	potassium voltage-gated channel protein Shab-like		KOG3713	1121	704	616	999	441	75	146	182	607	439	180	136	45	26	47	33
ACC_09615	conserved hypothetical protein		KOG4743	114	61	52	129	105	25	46	39	175	189	36	64	48	64	84	16
ACC_09616	conserved hypothetical protein			6	4	10	19	11	2	1	3	8	8	1	8	3	132	36	15
ACC_09617	hemocytin-like		KOG2246	422	305	341	461	549	64	92	119	959	3394	217	1021	858	500	30	24
ACC_09618	conserved hypothetical protein	K10643	KOG2068	577	445	463	606	252	43	103	110	952	699	201	480	544	562	191	133
ACC_09619	conserved hypothetical protein		KOG4606	4598	2532	1058	581	742	788	1687	2041	928	583	589	600	2114	1760	1265	854
ACC_09620	Y+L amino acid transporter 2-like		KOG1287	217	168	137	154	78	11	9	9	588	329	16	44	25	100	22	12
ACC_09621	zinc finger protein 521-like		KOG2462	145	87	112	194	196	8	22	29	95	77	73	85	49	112	63	18
ACC_09622	conserved hypothetical protein		KOG0612	237	153	128	299	258	35	75	75	184	96	115	66	158	260	170	72
ACC_09623	putative odorant receptor 13a-like			0	0	1	0	0	0	0	4	25	4	59	46	5	4	10	4
ACC_09624	conserved hypothetical protein		KOG0612	559	364	307	530	362	67	116	115	1117	585	353	250	345	314	133	66
ACC_09625	amyloid beta A4 precursor protein-binding family B member		KOG3537	1126	530	597	1944	377	54	95	169	499	310	420	222	12	26	113	87
ACC_09626	protein cortex		KOG0305	22	42	34	25	4	20	30	28	41	73	66	42	8	10	22	13
ACC_09627	congested-like trachea protein-like	K15109	KOG0758	191	113	101	168	191	14	23	25	357	264	180	541	375	478	28	15
ACC_09628	phosphatidylinositol 4-kinase alpha-like isoform 2	K00888	KOG0902	1005	733	705	1036	1012	41	108	103	1087	944	504	982	945	928	224	177
ACC_09629	probable ATP-dependent RNA helicase DHX35 isofc	K13117	KOG0922	160	152	150	158	157	10	22	19	348	236	124	169	441	433	78	20
ACC_09630	fibrillin-1		KOG1214	10	8	13	38	6	2	2	3	13	21	0	2	0	1	7	3
ACC_09631	conserved hypothetical protein		KOG3627	22	10	14	45	44	2	5	6	24	22	7	0	4449	10297	1114	40

ACC_09632	putative polypeptide N-acetylgalactosaminyltransf	K00710	KOG3736	193	67	136	69	18	5	10	16	196	254	5	37	190	141	131	27
ACC_09633	conserved hypothetical protein		KOG4338	453	113	100	242	111	26	39	39	1906	5808	14	23	888	601	426	194
ACC_09634	coiled-coil domain-containing protein 147-like		KOG0161	3	0	0	1	0	1	1	0	1	0	11	5	0	1	1	0
ACC_09635	A disintegrin and metalloproteinase with thrombos	K08624	KOG3538	91	50	32	40	28	17	19	22	230	182	22	48	171	88	80	57
ACC_09636	GTPase activating Rap/RanGAP domain-like 3		KOG3686	710	577	577	945	502	65	86	112	900	813	38	83	690	127	71	33
ACC_09637	conserved hypothetical protein	K09237	KOG4441	1349	703	772	1572	315	96	133	208	712	1143	357	194	153	383	1224	803
ACC_09638	elongation of very long chain fatty acids protein AAEL00800		KOG3071	194	88	104	155	96	27	43	40	236	166	35	153	158	318	315	149
ACC_09639	leucine-rich repeat and calponin homology domain-containi		KOG0532	573	265	242	586	113	35	44	75	301	157	94	87	96	76	147	93
ACC_09640	probable cytochrome P450 6a14-like		KOG0158	3	0	1	2	2	0	1	0	32	6	728	317	5100	1106	23	27
ACC_09641	conserved hypothetical protein	K16546		1	1	1	4	3	1	1	0	1	2	1	0	24	18	3	0
ACC_09642	unc-112-related protein-like isoform 1		KOG3727	431	140	147	366	152	38	38	47	1057	832	162	691	365	407	231	64
ACC_09643	hairless			219	237	238	254	198	19	24	40	202	172	81	273	284	321	81	21
ACC_09644	conserved hypothetical protein		KOG2462	249	172	160	272	167	46	63	79	331	357	114	167	317	328	312	134
ACC_09645	transportin-1-like		KOG2023	721	516	416	523	292	35	37	52	1506	1391	154	306	718	714	147	41
ACC_09646	cysteine dioxygenase type 1-like	K00456	KOG4064	14	4	10	37	35	17	17	12	699	1162	2	0	419	305	63	112
ACC_09647	ATP-dependent RNA helicase WM6-like	K12812	KOG0329	348	159	162	409	288	45	23	40	521	432	245	406	1542	2558	366	119
ACC_09648	conserved hypothetical protein		KOG3632	359	273	227	363	187	20	47	47	387	421	73	202	214	135	36	28
ACC_09649	FCH domain only protein 2-like isoform 2		KOG2398	999	739	695	1074	650	171	335	382	870	736	272	215	397	285	866	618
ACC_09650	zinc finger protein 658-like	K10896	KOG0354	522	373	360	715	519	100	173	212	651	430	461	633	564	624	276	130
ACC_09651	conserved hypothetical protein			152	106	123	137	122	11	19	34	353	302	105	188	113	155	46	13
ACC_09652	sodium channel protein para-like	K05388	KOG2301	1839	908	924	1628	791	99	159	192	889	409	331	97	4	10	38	17
ACC_09653	myosin heavy chain, muscle-like isoform 5	K10352	KOG0161	2635	605	234	1258	232	474	886	673	37553	21375	819	733	2073	2026	3073	3609
ACC_09654	putative odorant receptor 13a-like			0	0	0	0	0	0	0	0	5	3	35	5	0	0	0	0
ACC_09655	conserved hypothetical protein		KOG2139	117	74	58	154	83	10	15	11	241	263	109	91	381	335	39	35
ACC_09656	serine/threonine-protein kinase ULK3-like isoform	K08269	KOG0595	245	129	105	266	274	16	23	26	207	179	73	272	174	168	34	8
ACC_09657	conserved hypothetical protein			0	2	0	2	0	0	0	1	5	3	1	1	0	0	0	0
ACC_09658	poly(rC)-binding protein 3-like	K13162	KOG2190	1640	1579	1797	1516	271	164	292	438	579	537	78	66	47	72	388	155
ACC_09659	putative odorant receptor 13a-like			0	0	1	0	0	0	1	0	7	0	24	12	0	0	0	0
ACC_09660	conserved hypothetical protein		KOG4285	256	217	245	265	150	16	18	24	211	153	95	207	273	366	62	29
ACC_09661	cytoplasmic aconitate hydratase-like isoform 1	K01681	KOG0452	218	144	143	263	180	27	43	47	583	534	237	688	1469	718	82	25
ACC_09662	cationic amino acid transporter 4-like	K13866	KOG1286	973	496	593	1508	622	43	55	83	374	257	125	170	13	24	22	10
ACC_09663	diacylglycerol kinase 1	K00901	KOG1169	115	77	63	94	36	1	5	6	132	80	4	4	40	48	11	4
ACC_09664	conserved hypothetical protein		KOG0251	1196	714	800	670	248	88	104	133	1701	1413	389	673	310	270	196	63
ACC_09665	conserved hypothetical protein			2	0	2	3	0	0	1	0	5	20	0	1	14	16	2	5
ACC_09666	LOW QUALITY PROTEIN		KOG3209	394	264	251	330	193	57	76	97	902	545	100	153	233	445	238	95
ACC_09667	conserved hypothetical protein	K05326	KOG0500	194	149	146	146	63	11	6	19	110	97	0	13	2	8	5	12
ACC_09668	hemicentin-1-like		KOG3515	370	156	182	640	133	22	33	29	114	54	43	92	14	18	80	56
ACC_09669	sorting nexin-25-like		KOG2101	108	70	66	65	95	5	12	8	217	139	39	139	190	127	11	1
ACC_09670	proteasomal ubiquitin receptor ADRM1-like		KOG3037	490	407	410	1054	461	81	114	139	963	750	563	853	1206	1736	518	370
ACC_09671	conserved hypothetical protein		KOG4194	22	10	4	23	8	0	2	1	5	3	0	0	0	0	2	2
ACC_09672	synaptic vesicle glycoprotein 2B-like			7	18	15	43	32	2	1	1	19	7	16	5	1	1	2	0
ACC_09673	putative odorant receptor 13a-like			1	0	0	0	1	0	0	0	2	0	88	6	0	0	0	0
ACC_09674	SIFamide receptor		KOG4219	84	11	27	18	4	0	1	2	32	12	10	3	12	2	2	1
ACC_09675	conserved hypothetical protein			10	6	12	26	9	0	0	1	50	16	0	78	10	55	8	21
ACC_09676	acetyl-coenzyme A synthetase-like	K01895	KOG1175	390	164	160	286	222	11	10	25	348	215	137	282	1833	480	87	66
ACC_09677	ecdysteroid-regulated gene E74	K09428	KOG3804	269	89	74	90	21	16	9	17	153	136	25	70	200	65	83	79
ACC_09678	CAD protein isoform 1	K11540	KOG0370	396	205	90	271	135	28	38	46	217	404	177	260	1097	2257	1380	415
ACC_09679	conserved hypothetical protein		KOG0334	76	29	22	44	7	20	18	25	528	368	81	151	23	26	43	17
ACC_09680	solute carrier family 41 member 1-like isoform 1	K15122	KOG3788	361	211	153	423	275	41	48	53	300	259	143	215	341	406	110	35
ACC_09681	conserved hypothetical protein		KOG3539	128	47	50	80	12	0	0	3	39	70	3	3	2	3	6	6
ACC_09682	conserved hypothetical protein		KOG3130	593	410	341	413	674	94	279	282	244	190	240	235	321	471	442	390
ACC_09683	Down syndrome cell adhesion molecule-like protein 1		KOG3513	3029	1512	1833	1813	364	120	119	203	981	733	278	261	224	214	554	362
ACC_09684	leucokinin receptor	K14071	KOG4219	37	30	30	62	56	5	12	8	95	25	85	30	8	8	11	5
ACC_09685	pyrokinin-like receptor 1		KOG4219	5	1	7	8	3	0	0	1	10	7	0	4	13	14	1	2
ACC_09686	neutral ceramidase	K12349	KOG2232	161	128	176	880	281	25	21	19	297	233	104	80	901	511	154	38
ACC_09687	protein amnionless-like			3	3	1	2	1	1	0	0	20	30	0	0	143	30	17	3
ACC_09688	conserved hypothetical protein		KOG4701	59	19	49	111	63	28	19	23	1396	1194	14	159	60	43	27	16

ACC_09689	type II inositol-1,4,5-trisphosphate 5-phosphatase-	K01099	KOG0566	252	171	202	167	199	19	33	32	249	165	72	90	169	171	14	8
ACC_09690	conserved hypothetical protein			0	0	0	0	0	0	0	0	2	2	63	31	0	0	0	0
ACC_09691	conserved hypothetical protein			370	238	256	721	206	32	54	87	335	488	307	551	42	51	92	47
ACC_09692	Single-stranded DNA-binding protein 3		KOG4594	664	266	327	318	111	42	66	87	1162	835	183	386	256	289	143	57
ACC_09693	conserved hypothetical protein		KOG0032	411	220	105	348	107	170	261	225	17400	7537	238	137	267	290	307	192
ACC_09694	Period delta	K02633	KOG3753	525	782	420	479	206	159	262	346	406	866	158	302	167	84	46	31
ACC_09695	innexin shaking-B-like			204	64	63	179	86	13	10	12	312	185	9	40	207	142	61	13
ACC_09696	homeobox protein cut	K09313	KOG2252	1164	599	838	1735	258	40	76	125	475	418	305	133	36	132	638	645
ACC_09697	inositol monophosphatase 2-like		KOG2951	1	3	0	3	1	2	6	4	48	13	48	292	720	593	147	11
ACC_09698	elongation of very long chain fatty acids protein AAEL00800		KOG3071	5	2	7	7	1	1	0	1	41	26	86	96	77	97	1	1
ACC_09699	peptidoglycan-recognition protein LC-like	K01446		156	110	101	230	163	20	26	20	414	268	164	451	76	53	7	2
ACC_09700	conserved hypothetical protein			0	1	0	0	0	0	0	0	0	0	0	2	0	0	0	0
ACC_09701	odorant receptor 115			0	1	1	0	0	0	0	0	0	2	37	38	4	3	3	1
ACC_09702	protocadherin Fat 4-like		KOG4289	5	3	12	8	9	2	0	2	30	24	21	16	44	79	43	20
ACC_09703	probable beta-hexosaminidase fdl-like	K12373	KOG2499	86	28	38	89	11	6	3	7	94	123	132	84	36	43	149	20
ACC_09704	dynein heavy chain 10, axonemal-like		KOG3595	28	20	10	24	17	7	5	6	65	47	74	10	35	23	36	18
ACC_09705	pipsqueak		KOG4441	233	242	240	296	105	28	23	40	225	98	35	37	153	184	95	23
ACC_09706	conserved hypothetical protein		KOG2361	69	56	35	93	58	7	24	28	57	28	46	35	88	507	213	169
ACC_09707	neuroligin 4 precursor	K07378	KOG4389	83	21	25	75	13	2	7	10	26	21	4	5	1	1	4	4
ACC_09708	heterogeneous nuclear ribonucleoprotein K-like	K12886	KOG2192	1274	577	789	1394	461	180	288	473	1149	708	3830	1749	471	752	1716	1186
ACC_09709	conserved hypothetical protein		KOG4194	11	4	5	8	0	0	0	0	10	21	3	1	2	1	7	10
ACC_09710	glutamyl aminopeptidase-like		KOG1046	0	2	1	2	2	0	0	0	3	1	0	0	6	4	1	2
ACC_09711	calcium-dependent protein kinase 4-like	K00907	KOG0032	76	26	33	121	40	19	34	31	566	260	25	72	199	100	69	33
ACC_09712	conserved hypothetical protein		KOG1015	4742	3306	2412	3420	3095	606	1448	1663	3810	4224	2217	926	1797	2269	2463	971
ACC_09713	mitochondrial sodium/hydrogen exchanger NHA2-like		KOG3826	27	15	13	47	12	2	3	0	205	133	54	572	19	19	16	3
ACC_09714	conserved hypothetical protein			135	79	84	103	44	14	28	37	221	301	20	131	119	81	72	27
ACC_09715	ubiquitin carboxyl-terminal hydrolase 45-like	K11844	KOG1873	532	363	284	767	451	93	152	196	513	427	199	402	802	982	539	296
ACC_09716	ATP-dependent RNA helicase SUV3 homolog, mitochondrial-		KOG0953	683	398	331	562	559	72	105	122	741	700	435	994	1854	2160	332	111
ACC_09717	facilitated trehalose transporter Tret1-like		KOG0254	17	7	9	20	3	1	3	6	6	7	3	0	13	1	3	1
ACC_09718	tyrosine-protein phosphatase 10D-like		KOG4221	279	199	140	224	70	54	51	65	651	747	238	163	25	57	241	231
ACC_09719	la-related protein 1		KOG2591	949	549	392	635	402	372	682	676	1262	1449	533	314	837	1133	5079	4092
ACC_09720	fatty acyl-CoA reductase 1-like		KOG1221	17	27	31	215	73	8	3	16	50	85	221	364	17	52	9	36
ACC_09721	conserved hypothetical protein			641	274	79	344	142	187	340	226	1818	842	216	497	577	224	561	525
ACC_09722	LOW QUALITY PROTEIN		KOG1836	1051	853	748	1074	784	136	255	276	1796	3799	339	646	3130	2499	673	271
ACC_09723	interference hedgehog-like		KOG3513	302	209	200	339	403	30	104	94	455	303	215	460	368	460	159	57
ACC_09724	potassium voltage-gated channel protein eag-like	K05322	KOG0501	994	335	392	678	205	113	164	257	678	726	208	82	28	35	50	49
ACC_09725	conserved hypothetical protein			52	48	58	75	82	8	19	15	74	35	47	10	65	99	11	3
ACC_09726	conserved hypothetical protein			405	255	247	330	319	20	38	35	689	629	164	293	528	588	85	24
ACC_09727	conserved hypothetical protein		KOG0324	1148	666	573	1408	1209	470	992	941	3775	1485	625	903	1001	716	1422	1430
ACC_09728	fibrillin-1-like		KOG1508	435	353	323	456	240	36	68	80	606	476	351	343	880	949	505	517
ACC_09729	heterogeneous nuclear ribonucleoprotein Q-like		KOG0117	1190	888	806	1093	581	140	280	335	1076	1522	386	431	1483	1387	2290	2356
ACC_09730	methyltransferase-like protein 9-like		KOG3987	115	83	77	344	177	15	28	31	101	106	113	278	85	88	56	9
ACC_09731	beta-1,3-glucan-binding protein 1-like			53	40	33	85	51	20	17	12	2979	2122	181	64	62	84	18	49
ACC_09732	conserved hypothetical protein	K03652	KOG0254	149	148	125	172	160	19	29	40	58	70	97	108	145	217	92	36
ACC_09733	conserved hypothetical protein		KOG4729	157	36	29	52	22	3	5	16	74	35	3	22	32	20	29	12
ACC_09734	melanotransferrin			308	161	150	265	243	18	32	36	407	236	143	466	439	515	43	15
ACC_09735	peptide transporter family 1-like		KOG1237	96	64	52	112	55	13	5	10	284	1149	101	258	2020	1103	112	3
ACC_09736	conserved hypothetical protein		KOG3209	140	54	69	140	34	15	19	23	501	325	28	195	41	54	168	56
ACC_09737	battenin	K12389	KOG3880	179	47	75	107	66	27	33	51	247	221	34	44	115	48	71	46
ACC_09738	4-hydroxyphenylpyruvate dioxygenase-like	K00457	KOG0638	36	42	70	120	120	32	41	13	427	2076	57	54	403	1910	152	108
ACC_09739	PH domain leucine-rich repeat-containing protein p	K16340	KOG0618	6	8	4	15	5	2	1	4	35	98	14	36	32	101	27	5
ACC_09740	conserved hypothetical protein			1	2	2	15	4	1	0	2	8	5	5	1	0	0	1	0
ACC_09741	conserved hypothetical protein			58	25	47	60	17	3	8	6	79	136	18	14	31	39	90	69
ACC_09742	putative aminopeptidase W07G4.4-like	K01255	KOG2597	5	6	5	19	7	4	5	10	27	36	15	6	29	15	10	56
ACC_09743	gamma-aminobutyric acid type B receptor subunit	K04615	KOG1055	705	388	444	836	417	17	27	40	401	282	11	4	4	11	12	2
ACC_09744	conserved hypothetical protein			201	77	92	142	31	9	9	13	310	180	23	59	73	96	200	84
ACC_09745	Down syndrome cell adhesion molecule		KOG3513	548	452	387	205	103	13	22	22	290	197	1	2	3	5	11	8

ACC_09746	conserved hypothetical protein		KOG0956	282	272	288	546	205	57	52	82	427	274	212	241	345	217	320	133
ACC_09747	dedicator of cytokinesis protein 3-like	K05727	KOG1998	499	278	260	636	210	23	22	47	589	363	147	150	95	130	97	20
ACC_09748	serine/threonine kinase SAD-1-like	K08796	KOG0588	159	106	96	110	51	4	12	26	206	203	30	28	0	5	3	3
ACC_09749	fatty-acid amide hydrolase 2-A-like isoform 2		KOG1212	29	82	36	141	140	14	34	30	318	438	957	109	4	14	4	8
ACC_09750	conserved hypothetical protein		KOG1144	461	133	127	197	53	33	70	115	448	232	55	82	51	66	238	249
ACC_09751	zinc transporter ZIP13-like		KOG2694	505	326	200	711	625	85	90	94	2244	1687	530	825	710	585	68	10
ACC_09752	conserved hypothetical protein	K05336	KOG3660	321	192	191	193	132	18	18	27	446	249	431	436	285	570	89	16
ACC_09753	conserved hypothetical protein		KOG1026	100	57	146	433	165	60	45	65	1947	1360	148	287	121	190	101	24
ACC_09754	putative protein kinase C delta type homolog	K06068	KOG0694	38	26	38	76	52	8	12	10	757	509	32	12	19	31	18	9
ACC_09755	WD repeat-containing protein 60-like		KOG1592	650	348	268	522	443	59	163	166	459	367	346	420	368	249	175	84
ACC_09756	protein tipE-like			752	226	322	1033	208	40	59	82	188	94	153	51	0	4	13	8
ACC_09757	polypeptide N-acetylgalactosaminyltransferase 2-li	K00710	KOG3738	55	15	7	34	5	1	2	12	44	32	11	7	9	13	8	6
ACC_09758	nogo-B receptor-like		KOG2818	92	76	58	163	84	9	17	20	83	48	30	65	152	141	48	10
ACC_09759	conserved hypothetical protein	K01090	KOG0698	133	94	81	142	66	7	22	26	241	262	44	133	102	126	116	86
ACC_09760	putative glycerophosphocholine phosphodiesterase GPCPD1		KOG2421	1172	754	483	502	433	225	247	244	2211	1257	879	1010	1734	1028	166	32
ACC_09761	conserved hypothetical protein		KOG2462	201	144	185	224	34	8	9	16	40	47	1	8	28	27	84	90
ACC_09762	conserved hypothetical protein			533	464	549	918	196	35	44	65	286	200	52	32	57	72	387	199
ACC_09763	conserved hypothetical protein		KOG3598	6	1	0	0	0	1	0	0	9	2	2	21	2	0	0	0
ACC_09764	ATP-binding cassette sub-family G member 4-like		KOG0061	161	126	136	355	224	15	27	34	628	832	72	104	601	387	93	21
ACC_09765	LOW QUALITY PROTEIN		KOG1587	451	332	297	454	319	94	195	166	319	307	318	564	504	564	557	281
ACC_09766	conserved hypothetical protein		KOG3443	270	165	184	443	266	21	36	50	209	188	141	184	147	163	204	86
ACC_09767	conserved hypothetical protein		KOG0161	1645	1277	1169	704	426	384	621	766	2902	1657	703	964	706	1013	787	371
ACC_09768	conserved hypothetical protein			119	82	70	74	86	11	30	36	178	158	43	95	304	280	139	43
ACC_09769	protein unc-13 homolog D		KOG1328	639	215	218	505	228	47	49	67	2127	1691	229	626	1104	411	74	46
ACC_09770	protein slit	K06839	KOG4237	209	76	70	135	33	11	18	24	121	76	25	12	5	19	150	95
ACC_09771	conserved hypothetical protein	K07607	KOG3882	2	1	1	3	0	0	0	0	6	15	0	0	0	2	4	2
ACC_09772	putative DNA helicase Ino80-like	K11665	KOG0388	374	226	186	395	327	89	211	207	408	386	219	127	227	270	490	281
ACC_09773	prestin-like		KOG0236	343	342	180	265	154	138	146	198	114	49	162	247	319	442	106	55
ACC_09774	sorting nexin-14-like		KOG2273	370	229	240	223	276	12	12	11	638	537	77	355	383	247	11	7
ACC_09775	conserved hypothetical protein		KOG4441	1491	879	959	1565	590	141	170	254	1173	1176	446	698	497	495	747	441
ACC_09776	zinc finger protein 808-like		KOG2462	412	289	272	428	181	79	83	104	257	275	98	39	44	62	187	82
ACC_09777	neuroligin 3 precursor		KOG4389	170	50	45	119	19	4	3	11	48	21	26	12	1	1	17	4
ACC_09778	conserved hypothetical protein		KOG0125	306	197	200	224	85	47	92	97	135	108	41	24	34	29	304	282
ACC_09779	jouberin-like	K16740	KOG1587	1250	703	604	886	1156	120	438	364	647	460	562	494	463	403	357	197
ACC_09780	neurotrimin-like		KOG3513	45	48	48	55	14	2	9	13	78	89	12	24	23	24	17	1
ACC_09781	coronin-2B-like		KOG0303	633	269	234	308	268	97	105	182	454	346	146	262	200	202	46	15
ACC_09782	synapsin-like		KOG3895	7627	2970	3951	8423	2816	235	507	623	1741	1209	261	101	9	61	102	58
ACC_09783	conserved hypothetical protein		KOG4193	7	10	22	72	41	6	2	2	418	1977	6	1	166	122	55	3
ACC_09784	conserved hypothetical protein		KOG3371	335	383	437	626	676	77	168	215	390	343	1390	789	199	284	83	34
ACC_09785	protein TANC2-like isoform 1		KOG4177	261	132	140	297	122	7	18	16	424	443	43	176	30	63	64	13
ACC_09786	LOW QUALITY PROTEIN			280	202	253	396	176	85	90	104	381	318	111	149	89	82	122	55
ACC_09787	conserved hypothetical protein		KOG3598	193	114	122	230	53	41	60	69	125	272	69	51	20	33	326	249
ACC_09788	synaptic vesicular amine transporter	K08155	KOG3764	148	92	80	123	58	90	113	116	386	511	24	20	96	32	17	30
ACC_09789	LOW QUALITY PROTEIN		KOG0158	0	0	0	0	0	0	0	0	1	0	0	1	1	0	0	0
ACC_09790	conserved hypothetical protein			317	150	141	151	24	7	6	13	126	177	11	8	1	2	3	4
ACC_09791	conserved hypothetical protein			344	156	152	296	103	27	34	45	245	207	38	10	10	15	20	12
ACC_09792	carboxypeptidase B-like		KOG2650	3	6	7	8	10	1	0	1	32	8	1	1	6034	2272	950	25
ACC_09793	neuropeptide Y receptor-like		KOG4219	161	65	76	108	88	27	65	72	89	80	93	115	35	72	184	114
ACC_09794	aryl hydrocarbon receptor nuclear translocator-like protein :		KOG3561	19	31	28	32	12	8	9	12	86	104	29	39	5	11	18	6
ACC_09795	conserved hypothetical protein			284	107	112	222	61	13	16	25	84	125	60	28	6	4	8	8
ACC_09796	catenin delta-2-like		KOG1048	306	204	237	408	181	35	58	92	289	231	154	114	225	400	270	188
ACC_09797	neurogenic locus protein delta	K06051		17	11	15	12	4	2	1	0	29	55	4	11	2	4	10	2
ACC_09798	junctophilin-1-like isoform 2		KOG0231	782	338	349	432	81	25	22	47	793	851	53	22	16	18	65	40
ACC_09799	conserved hypothetical protein		KOG1809	222	156	138	162	170	26	71	89	425	311	79	144	214	191	37	28
ACC_09800	pancreatic triacylglycerol lipase-like		KOG2792	184	142	145	227	317	12	11	24	219	150	237	393	422	611	26	13
ACC_09801	procollagen-lysine,2-oxoglutarate 5-dioxygenase 3-	K13647	KOG1971	260	131	111	332	230	18	22	31	637	433	147	411	720	602	85	33
ACC_09802	conserved hypothetical protein			154	186	186	385	331	9	17	20	160	342	156	262	541	361	32	17

ACC_09803	parathyroid hormone/parathyroid hormone-related peptide		KOG4564	13	6	3	13	6	0	4	1	21	34	15	126	69	43	4	2
ACC_09804	acetylcholinesterase	K01049	KOG4389	373	187	247	680	113	8	10	16	320	253	37	114	3	6	16	5
ACC_09805	UPF0472 protein C16orf72 homolog			139	80	79	98	54	21	15	14	486	319	34	157	190	124	33	10
ACC_09806	conserved hypothetical protein	K09108	KOG0561	167	151	162	261	34	18	18	66	170	280	48	35	106	68	209	271
ACC_09807	SEC14 domain and spectrin repeat-containing protein 1		KOG3513	1111	559	356	724	198	353	661	543	17831	10158	391	521	779	621	1388	1249
ACC_09808	coiled-coil domain-containing protein CG32809-like		KOG1984	1394	763	704	1303	236	153	162	278	921	618	226	230	268	420	616	235
ACC_09809	conserved hypothetical protein	K11583	KOG2562	347	199	253	332	125	43	53	92	997	885	65	109	128	192	158	167
ACC_09810	helix-loop-helix protein 1-like	K09075	KOG4029	1	0	1	9	1	2	0	1	0	0	0	0	0	1	1	0
ACC_09811	conserved hypothetical protein			7	0	0	0	0	0	0	0	0	1	0	1	2	218	803	157
ACC_09812	structural maintenance of chromosomes protein 1A-like		KOG0018	2	7	3	4	12	0	1	1	1	5	3	0	4	1	0	1
ACC_09813	ras-GEF domain-containing family member 1B-like		KOG3541	431	169	182	330	129	23	35	60	123	78	25	32	2	1	59	22
ACC_09814	conserved hypothetical protein			25	26	25	7	8	1	0	2	22	10	17	21	21	8	4	1
ACC_09815	soluble guanylate cyclase 89Da-like		KOG4171	16	3	5	11	2	0	1	0	10	10	2	0	3	8	5	1
ACC_09816	conserved hypothetical protein		KOG0161	46	29	33	30	4	2	0	6	59	143	27	23	7	8	3	5
ACC_09817	conserved hypothetical protein			301	87	84	125	42	73	113	140	358	198	905	1252	116	332	603	503
ACC_09818	LOW QUALITY PROTEIN		KOG2302	31	42	64	31	26	0	4	7	13	10	10	3	9	4	2	0
ACC_09819	hypothetical protein			40	18	22	50	44	0	3	3	14	11	37	84	45	48	5	7
ACC_09820	conserved hypothetical protein	K00791	KOG1384	86	49	52	147	58	46	25	56	158	120	55	43	112	163	86	86
ACC_09821	conserved hypothetical protein			26	15	32	46	16	8	8	11	28	47	1	2	0	0	4	7
ACC_09822	cytochrome b-c1 complex subunit 9-like	K00419	KOG3494	295	109	114	484	327	0	12	9	106	46	237	350	271	205	14	4
ACC_09823	conserved hypothetical protein			1	0	0	0	0	0	1	0	1	2	0	0	1	0	0	1
ACC_09824	conserved hypothetical protein		KOG0994	0	1	0	2	0	0	0	0	0	0	0	1	0	0	3	0
ACC_09825	synaptic vesicle glycoprotein 2C-like		KOG0253	28	412	4	10	14	262	198	198	23	20	3	2	131	48	3	2
ACC_09826	protein Wnt-4		KOG3913	19	10	14	22	6	12	20	20	15	16	23	9	10	8	52	19
ACC_09827	UPF0488 protein CG14286-like			30	30	38	34	38	1	6	7	57	33	70	145	134	111	16	9
ACC_09828	conserved hypothetical protein	K09267	KOG0527	1	3	0	0	0	0	1	1	0	1	0	0	1	1	1	0
ACC_09829	otopetrin-3-like		KOG4740	31	16	24	46	23	2	6	11	57	28	11	36	56	244	47	25
ACC_09830	conserved hypothetical protein			72	41	61	71	76	0	1	4	67	34	13	47	17	19	3	2
ACC_09831	conserved hypothetical protein			4	3	4	38	3	2	0	3	7	11	5	1	0	0	4	3
ACC_09832	conserved hypothetical protein			17	2	1	8	1	13	9	16	8	1	1	9	2	10	8	9
ACC_09833	conserved hypothetical protein			11	2	4	11	6	0	0	0	4	15	1	6	0	0	1	0
ACC_09834	conserved hypothetical protein			69	74	47	34	15	11	10	14	189	166	32	129	69	52	25	17
ACC_09835	serine/threonine-protein kinase STK11-like	K07298	KOG0583	33	20	18	67	57	5	10	15	58	45	22	22	76	71	39	41
ACC_09836	voltage-dependent calcium channel subunit alpha-2/delta-3-		KOG2353	1078	535	574	928	505	74	96	100	670	490	141	100	10	44	33	11
ACC_09837	E3 ubiquitin-protein ligase RNF220-like		KOG0978	19	5	8	19	6	1	3	3	13	20	9	11	14	15	146	136
ACC_09838	conserved hypothetical protein		KOG0775	1	0	0	0	0	0	0	0	1	1	0	0	4	3	4	0
ACC_09839	conserved hypothetical protein			54	16	21	39	4	1	0	0	11	20	0	0	0	0	3	2
ACC_09840	serine-protein kinase ATM	K04728	KOG0892	1051	631	504	617	684	49	67	98	420	396	241	226	295	289	33	9
ACC_09841	neuroligin 4 precursor			53	24	24	52	7	7	11	15	19	23	7	6	2	1	9	8
ACC_09842	conserved hypothetical protein			128	77	125	143	67	21	35	52	259	238	24	72	52	70	79	61
ACC_09843	division abnormally delayed protein-like	K02306	KOG3821	340	113	149	223	62	3	3	6	272	308	13	466	63	42	37	36
ACC_09844	odorant binding protein 17 precursor			122	53	39	287	214	62	91	88	9027	2588	20381	15198	6	2	0	1
ACC_09845	facilitated trehalose transporter Tret1-like		KOG0569	68	183	76	78	64	61	42	81	136	200	40	16	6	10	3	0
ACC_09846	zinc finger Ran-binding domain-containing protein 2-like		KOG1995	533	573	736	524	324	111	139	165	723	345	180	190	338	431	195	202
ACC_09847	Myosin class II heavy chain		KOG0161	27	13	19	57	37	0	4	1	7	15	12	0	35	18	7	44
ACC_09848	conserved hypothetical protein			4	12	12	33	13	0	0	6	1	1	10	4	0	0	0	3
ACC_09849	conserved hypothetical protein			12	14	13	23	24	2	7	4	73	41	16	214	243	304	38	11
ACC_09850	peptidyl-prolyl cis-trans isomerase, rhodopsin-specific isozyr		KOG0880	50	33	43	133	86	147	115	203	680	405	0	37	2	1	1	1
ACC_09851	major facilitator superfamily domain-containing protein 6-lik		KOG3762	253	153	170	278	142	5	13	10	825	483	55	379	116	35	8	8
ACC_09852	conserved hypothetical protein			33	23	29	117	36	13	14	22	132	54	13	11	4	15	35	18
ACC_09853	catalase	K03781	KOG0047	52	52	31	114	81	12	16	14	997	563	514	2279	715	1994	120	49
ACC_09854	catalase	K03781	KOG0047	189	136	139	391	310	52	58	111	1601	787	1593	3706	1348	4388	484	134
ACC_09855	conserved hypothetical protein			11	11	13	22	14	0	1	4	23	25	9	56	16	128	5	0
ACC_09856	sushi, von Willebrand factor type A, EGF and pentraxin domain-contain			34	10	14	12	10	13	7	10	172	94	22	430	146	312	39	10
ACC_09857	conserved hypothetical protein	K09411	KOG3562	2	0	1	0	0	0	0	0	9	6	84	132	1	2	4	1
ACC_09858	hypothetical protein			4	4	5	0	0	0	0	1	8	80	1	13	5	1	3	5
ACC_09859	conserved hypothetical protein			18	5	18	56	41	1	1	6	29	36	21	81	117	48	154	52

ACC_09860	zinc finger protein 813-like		KOG3608	64	58	53	108	134	5	31	21	118	44	102	145	136	114	26	8
ACC_09861	conserved hypothetical protein			11	16	20	11	47	3	4	4	25	20	18	65	34	42	6	3
ACC_09862	protein FAM116A-like		KOG2432	166	96	74	148	130	6	12	4	198	153	45	115	204	231	32	5
ACC_09863	phosphatidylinositol N-acetylglucosaminyltransfera	K03859	KOG3059	14	15	34	29	48	0	2	0	12	15	17	13	18	8	3	1
ACC_09864	brain protein 44-like protein-like		KOG1590	1	0	0	4	1	0	1	0	0	1	1	0	1	2	1	0
ACC_09865	membrane-bound transcription factor site-1 protea	K08653	KOG4266	225	137	123	202	170	36	51	59	369	335	134	325	350	384	92	36
ACC_09866	LOW QUALITY PROTEIN		KOG0161	0	0	0	2	1	0	0	1	1	9	2	2	3	1	3	1
ACC_09867	conserved hypothetical protein		KOG0190	219	128	46	131	41	118	217	203	2320	1226	69	56	251	123	100	47
ACC_09868	conserved hypothetical protein		KOG2147	196	105	93	74	63	48	34	45	370	171	142	600	619	561	136	62
ACC_09869	conserved hypothetical protein			20	9	11	25	6	14	18	15	190	156	13	16	2	2	6	2
ACC_09870	conserved hypothetical protein			97	34	12	30	19	24	28	49	880	173	6781	1063	0	1	0	0
ACC_09871	Pyruvate dehydrogenase	K00898	KOG0787	218	145	190	520	200	35	59	64	701	551	506	294	114	35	30	13
ACC_09872	conserved hypothetical protein			20	4	1	12	3	0	2	0	2	2	0	0	0	1	4	0
ACC_09873	conserved hypothetical protein			19	15	13	17	25	1	2	3	34	37	30	23	99	58	31	14
ACC_09874	stromal cell-derived factor 2-like protein 1-like isoform 2		KOG3358	884	905	1011	1015	1193	112	145	162	867	633	555	2056	1963	1791	156	75
ACC_09875	conserved hypothetical protein			336	255	195	214	192	22	35	39	344	182	116	77	260	478	74	16
ACC_09876	APD-3-like protein		KOG2385	6	4	1	0	0	0	0	0	1	1	1	14	2	2	2	0
ACC_09877	decaprenyl-diphosphate synthase subunit 1-like	K12504	KOG0776	52	11	8	40	24	3	6	10	46	23	27	59	141	158	168	64
ACC_09878	maltase 1		KOG0471	20	4	9	6	7	0	2	3	70	3	12	8	226	610	69	10
ACC_09879	lachesin-like, partial		KOG3513	21	7	2	3	1	0	0	1	11	2	26	52	0	0	0	0
ACC_09880	hydrocephalus-inducing protein-like			9	4	3	8	2	4	7	1	23	13	9	16	4	9	11	6
ACC_09881	hypothetical protein			142	25	76	183	54	7	9	16	180	197	28	433	78	37	46	13
ACC_09882	LOW QUALITY PROTEIN	K02375	KOG3577	72	67	65	95	12	3	2	3	60	139	2	13	24	15	27	11
ACC_09883	tubulin polyglutamylase TTLL4-like		KOG2156	50	17	20	114	71	9	9	13	26	8	11	3	1	4	12	7
ACC_09884	conserved hypothetical protein	K09218	KOG2462	22	6	6	12	1	0	3	2	3	6	1	3	2	15	158	110
ACC_09885	lysyl-tRNA synthetase	K04567	KOG1885	1487	768	585	1297	969	265	550	498	823	869	571	801	4079	6535	3416	2733
ACC_09886	conserved hypothetical protein			224	41	47	137	34	24	15	18	693	589	282	961	199	178	236	68
ACC_09887	CD109 antigen-like		KOG1366	55	39	40	90	84	6	6	4	394	164	208	149	51	64	11	4
ACC_09888	dimethylaniline monooxygenase		KOG1399	25	4	3	13	10	3	7	4	139	375	5	112	996	804	63	29
ACC_09889	DNA replication licensing factor MCM8		KOG1924	18	5	18	5	2	0	1	0	6	11	2	4	13	8	37	12
ACC_09890	Leucine-rich repeat-containing protein C10orf92-like protein			0	2	1	0	0	0	0	1	0	2	0	0	0	0	1	1
ACC_09891	conserved hypothetical protein			1	1	1	3	1	1	1	0	14	0	0	0	48	15	4	6
ACC_09892	protein atonal-like	K09083	KOG4395	0	0	0	1	0	0	0	1	0	1	1	0	0	0	4	7
ACC_09893	conserved hypothetical protein	K09321	KOG0489	1	0	1	0	0	0	0	1	11	2	46	23	0	1	6	4
ACC_09894	conserved hypothetical protein			15	85	97	211	107	318	526	641	725	374	0	14	1	0	1	0
ACC_09895	peroxisome biogenesis factor 2-like	K06664	KOG2879	40	43	27	53	28	26	22	30	145	59	82	133	143	124	25	16
ACC_09896	conserved hypothetical protein			424	235	266	296	55	10	15	24	286	197	31	37	52	9	21	30
ACC_09897	phospholipase A2 precursor			7	0	1	1	0	0	0	0	3	1	0	0	0	0	1	2
ACC_09898	conserved hypothetical protein		KOG0248	674	427	454	519	283	59	76	81	1038	371	162	174	197	352	133	57
ACC_09899	conserved hypothetical protein			20	3	3	5	4	1	1	2	1	3	0	1	0	0	1	3
ACC_09900	esterase FE4-like		KOG1516	220	138	137	451	294	51	68	79	12037	5618	4771	7263	4927	4331	846	318
ACC_09901	hexamerin			11	3	0	4	6	3	3	7	510	72	1	2	188856	4508	14	52
ACC_09902	dynein heavy chain 1, axonemal		KOG3595	51	57	46	64	33	12	13	24	11	25	16	6	3	6	12	0
ACC_09903	conserved hypothetical protein			2	2	5	9	6	0	1	2	2	3	0	5	12	26	12	11
ACC_09904	Segment polarity protein dishevelled-like protein DVL-3			38	37	44	98	53	12	32	35	22	25	24	15	8	10	42	7
ACC_09905	conserved hypothetical protein		KOG4838	125	123	101	53	89	2	6	1	50	128	91	385	156	62	1	1
ACC_09906	conserved hypothetical protein			48	37	42	72	5	0	1	4	19	24	17	0	0	0	13	7
ACC_09907	hyperpolarization-activated ion channel		KOG0498	483	255	267	363	142	47	97	120	153	107	219	61	86	22	73	24
ACC_09908	conserved hypothetical protein		KOG2462	78	33	41	44	14	15	13	13	85	98	27	42	58	61	39	13
ACC_09909	conserved hypothetical protein			18	1	4	19	10	0	0	0	1	1	1	0	0	0	0	1
ACC_09910	nuclear receptor subfamily 2 group E member 1-lik	K08546	KOG4218	1	8	7	25	14	0	1	1	2	3	0	0	6	5	2	0
ACC_09911	ras-specific guanine nucleotide-releasing factor 1-like			19	34	29	28	13	1	6	7	16	10	2	7	0	0	0	0
ACC_09912	blood vessel epicardial substance-like			43	37	42	134	16	2	3	3	17	31	3	10	1	5	10	6
ACC_09913	conserved hypothetical protein	K00420		139	72	61	162	166	3	8	10	64	18	158	291	117	127	2	7
ACC_09914	Down syndrome cell adhesion molecule-like protein CG4225		KOG3513	399	194	221	354	106	33	53	72	103	178	38	41	4	16	139	175
ACC_09915	NAD kinase domain-containing protein 1-like		KOG4180	79	83	64	187	173	9	32	25	231	169	135	396	326	446	83	45
ACC_09916	dolichyl-diphosphooligosaccharide--protein glycosyltransferase subuni			41	26	10	36	11	0	2	16	9	16	21	57	187	68	10	1

ACC_09917	putative ferric-chelate reductase 1-like			2	2	0	6	2	3	0	1	4	1	4	17	78	64	18	24
ACC_09918	conserved hypothetical protein		KOG4847	255	116	134	232	41	16	14	34	153	91	15	19	22	11	24	28
ACC_09919	conserved hypothetical protein		KOG3608	297	133	88	331	419	19	64	73	81	170	222	139	165	192	275	129
ACC_09920	conserved hypothetical protein	K00419	KOG3494	24	23	27	54	55	2	3	5	28	11	19	12	22	36	8	2
ACC_09921	zinc finger FYVE domain-containing protein 9	K04679	KOG1841	840	450	400	796	651	50	136	130	981	1276	297	546	626	507	142	58
ACC_09922	zinc finger protein 234-like		KOG3608	122	106	114	198	97	16	32	43	109	104	67	28	95	65	134	108
ACC_09923	hypothetical protein			39	39	32	44	16	6	20	17	56	104	14	15	16	13	46	16
ACC_09924	conserved hypothetical protein			0	0	0	0	0	0	0	0	0	1	0	0	0	4	17	3
ACC_09925	conserved hypothetical protein	K08858	KOG1345	6	2	1	5	2	1	1	1	221	203	2	9	1	6	1	1
ACC_09926	conserved hypothetical protein		KOG1980	11	3	4	3	3	2	1	1	3	1	1	4	5	10	18	42
ACC_09927	cytochrome P450 302a1, mitochondrial	K10721	KOG0159	63	49	31	36	34	17	17	24	71	36	82	228	109	174	38	14
ACC_09928	conserved hypothetical protein			3	1	0	3	4	2	2	1	5	4	9	0	0	0	4	0
ACC_09929	ADP/ATP translocase 4-like		KOG0749	46	25	19	17	26	2	4	6	40	27	13	23	6	11	1	2
ACC_09930	optomotor-blind protein-like, partial	K10177	KOG3586	73	44	51	140	34	3	4	4	62	107	84	56	12	10	27	24
ACC_09931	F-box/SPRY domain-containing protein 1-like	K10319	KOG3953	45	53	23	21	26	21	20	17	44	23	21	22	43	32	15	2
ACC_09932	choline O-acetyltransferase-like	K00623	KOG3717	273	212	213	175	111	9	8	18	299	144	69	128	4	7	4	0
ACC_09933	conserved hypothetical protein		KOG4063	322	229	277	2293	1045	24	30	51	918	1856	13	28	665	373	224	83
ACC_09934	PRA1 family protein 3-like isoform 1		KOG4050	306	134	129	376	373	24	16	30	566	194	128	397	388	422	25	5
ACC_09935	tetraspanin-5		KOG3882	213	66	55	158	94	8	13	18	79	57	3	53	9	12	14	6
ACC_09936	ubiquitin-like protein 3-like isoform 2			278	105	81	79	35	22	50	47	233	252	28	157	41	33	74	50
ACC_09937	conserved hypothetical protein	K15614	KOG0775	7	2	0	6	0	7	9	7	95	84	0	6	5	2	31	15
ACC_09938	conserved hypothetical protein			0	0	0	0	1	0	0	0	0	1	3	2	0	3	1	5
ACC_09939	conserved hypothetical protein			0	0	1	2	0	1	0	1	2	3	36	91	0	0	0	0
ACC_09940	clusterin-associated protein 1-like		KOG3647	20	19	30	57	43	4	7	5	24	22	57	74	5	22	12	10
ACC_09941	LOW QUALITY PROTEIN	K00501	KOG3820	115	39	35	94	15	12	16	13	3089	447	186	1837	27	22	17	10
ACC_09942	conserved hypothetical protein			40	15	20	27	20	0	0	0	30	8	34	97	1	0	2	0
ACC_09943	conserved hypothetical protein	K01183		122	108	136	223	92	12	13	7	206	189	85	101	549	692	187	44
ACC_09944	TAF5-like RNA polymerase II p300/CBP-associated factor-ass		KOG0263	61	42	41	106	83	5	16	10	55	50	46	65	94	98	13	4
ACC_09945	conserved hypothetical protein		KOG3605	60	27	16	31	21	4	2	5	33	27	17	7	10	12	13	3
ACC_09946	nucleolar MIF4G domain-containing protein 1-like		KOG2141	627	367	308	610	924	24	97	103	302	264	324	364	509	548	261	190
ACC_09947	paired box protein Pax-3-B		KOG0849	3	1	2	11	0	0	2	2	38	23	1	0	0	0	4	1
ACC_09948	trafficking protein particle complex subunit 6B-like		KOG3316	109	60	90	101	155	7	6	11	60	64	63	239	293	261	14	3
ACC_09949	conserved hypothetical protein		KOG0161	46	18	19	21	11	4	11	14	135	110	24	29	18	24	62	8
ACC_09950	Basic helix-loop-helix transcription factor amos	K09083	KOG4395	0	0	0	2	0	0	0	0	1	0	1	0	0	0	0	0
ACC_09951	conserved hypothetical protein		KOG4721	212	168	179	130	33	28	29	34	103	121	45	66	36	37	137	40
ACC_09952	conserved hypothetical protein			199	60	52	142	120	14	22	28	248	205	139	284	82	18	8	5
ACC_09953	conserved hypothetical protein			4	1	1	1	1	0	0	0	12	16	4	5	9	108	45	20
ACC_09954	protein abrupt-like			68	76	109	60	13	6	4	5	64	96	7	7	5	4	53	33
ACC_09955	zinc finger protein 426-like	K09207	KOG2462	150	102	85	264	43	17	16	33	108	69	52	34	25	32	127	45
ACC_09956	conserved hypothetical protein			0	0	0	0	0	0	0	0	0	0	0	0	5	438	565	117
ACC_09957	RNA-binding protein 38-like isoform 1		KOG0149	62	59	50	38	10	60	99	128	238	283	17	5	29	34	207	218
ACC_09958	conserved hypothetical protein			8	1	6	11	0	1	0	3	1	0	0	0	0	1	4	10
ACC_09959	conserved hypothetical protein		KOG3811	272	216	221	302	83	16	19	15	402	248	52	46	70	57	57	7
ACC_09960	lactosylceramide 4-alpha-galactosyltransferase-like		KOG1928	5	0	1	2	1	0	1	0	0	3	1	2	121	143	24	0
ACC_09961	conserved hypothetical protein			74	22	32	55	10	3	3	7	17	18	0	0	0	3	21	35
ACC_09962	serine protease HTRA2, mitochondrial	K08669	KOG1320	318	169	174	427	303	26	38	54	535	305	102	403	399	454	91	26
ACC_09963	conserved hypothetical protein		KOG4331	3	2	4	6	3	3	9	12	29	12	5	29	2	2	0	1
ACC_09964	arfaptin-2-like		KOG3876	131	60	60	76	45	1	2	3	100	85	16	70	90	119	6	2
ACC_09965	conserved hypothetical protein			43	39	53	39	19	6	3	11	48	44	10	6	4	5	14	6
ACC_09966	probable G-protein coupled receptor B0563.6-like		KOG4219	23	27	18	50	14	3	5	10	36	10	4	0	7	2	4	3
ACC_09967	conserved hypothetical protein		KOG2200	620	368	402	1277	269	51	80	109	658	417	298	300	183	130	256	144
ACC_09968	probable Rho GTPase-activating protein CG5521-like		KOG3652	311	280	210	128	79	30	44	50	892	1093	104	230	448	309	39	15
ACC_09969	kin of IRRE-like protein 3-like		KOG3513	59	17	29	39	21	3	4	6	20	10	1	0	0	3	8	13
ACC_09970	tubulin polyglutamylase TTLL5-like			48	40	32	26	25	2	5	7	49	41	2	26	29	13	6	3
ACC_09971	conserved hypothetical protein			0	0	0	1	0	0	0	0	0	0	0	0	0	0	3	0
ACC_09972	hypothetical protein			18	11	11	37	14	4	0	6	8	3	10	5	0	0	2	6
ACC_09973	conserved hypothetical protein		KOG0192	53	67	69	49	14	19	27	31	236	210	39	310	278	182	29	18

ACC_09974	conserved hypothetical protein		KOG4220	46	22	29	37	19	4	2	2	30	18	3	5	5	2	4	0
ACC_09975	glutamate receptor, ionotropic kainate 1-like		KOG1054	41	21	26	42	9	3	2	3	32	25	2	2	0	1	2	0
ACC_09976	conserved hypothetical protein			111	56	47	211	47	16	18	30	34	5	3	1	0	1	3	1
ACC_09977	facilitated trehalose transporter Tret1-like			40	31	25	98	57	8	9	12	720	1750	55	131	912	303	99	24
ACC_09978	LOW QUALITY PROTEIN		KOG1029	1238	694	662	796	534	253	539	597	1079	883	442	258	396	275	1314	902
ACC_09979	conserved hypothetical protein			70	90	77	441	191	193	236	306	2705	699	488	517	154	238	207	69
ACC_09980	u7 snRNA-associated Sm-like protein LSm11-like		KOG3460	13	8	16	50	14	1	7	8	15	15	8	9	16	22	51	40
ACC_09981	conserved hypothetical protein			13	6	7	24	16	0	1	1	17	1	0	17	80	297	90	67
ACC_09982	homeobox protein Nkx-2.1-like		KOG0842	82	52	78	120	24	2	1	3	19	38	0	0	0	0	1	3
ACC_09983	diacylglycerol kinase theta-like isoform 2	K00901	KOG1169	4360	2830	3222	6170	1572	237	417	647	2058	934	181	109	63	51	153	60
ACC_09984	conserved hypothetical protein		KOG4126	38	13	10	50	18	2	4	6	12	4	6	0	2	4	12	7
ACC_09985	LOW QUALITY PROTEIN	K16506	KOG1219	293	176	148	156	59	28	34	44	323	299	112	45	102	158	243	83
ACC_09986	conserved hypothetical protein		KOG1052	14	12	8	24	12	1	0	2	56	26	28	12	8	13	5	1
ACC_09987	frequenin-1-like		KOG0044	108	42	53	170	56	8	15	21	29	17	26	17	1	0	4	7
ACC_09988	conserved hypothetical protein		KOG4307	103	78	104	186	70	6	5	10	188	123	23	36	54	72	77	16
ACC_09989	conserved hypothetical protein			5	2	2	10	12	1	2	3	22	10	9	34	4	4	0	0
ACC_09990	transmembrane protease serine 9-like		KOG3627	29	16	20	13	7	14	7	13	6	2	4	20	44	190	180	91
ACC_09991	conserved hypothetical protein			334	203	192	326	138	61	68	97	225	74	38	89	60	65	85	22
ACC_09992	conserved hypothetical protein			0	0	0	1	0	0	0	0	1	1	0	2	7	4	2	14
ACC_09993	conserved hypothetical protein			31	22	60	57	22	8	6	13	41	96	10	5	5	6	104	127
ACC_09994	tyrosine-protein kinase Drl-like		KOG1024	4	3	0	7	4	0	0	3	1	6	0	2	1	0	0	0
ACC_09995	zinc finger protein 568-like		KOG2462	84	54	50	117	83	5	28	31	82	47	40	40	40	66	28	13
ACC_09996	conserved hypothetical protein		KOG0260	2	2	5	18	5	0	1	3	16	17	4	5	19	203	225	315
ACC_09997	conserved hypothetical protein	K05641	KOG0059	124	44	44	118	24	48	55	73	2352	2990	1000	769	196	56	41	17
ACC_09998	cytochrome b5-like		KOG0537	13	26	9	8	10	13	10	10	93	307	23	10	5	9	4	0
ACC_09999	conserved hypothetical protein			14	7	8	28	6	1	3	6	10	17	6	7	6	1	11	9
ACC_10000	neurobeachin-like		KOG1787	1110	637	641	975	343	80	140	197	505	566	85	44	76	75	140	50
ACC_10001	conserved hypothetical protein			0	0	1	0	0	0	0	0	4	2	1	0	0	0	0	1
ACC_10002	beta-2 adrenergic receptor-like		KOG4219	188	130	154	162	52	11	7	19	103	150	95	118	0	3	7	3
ACC_10003	peroxisome assembly protein 12-like isoform 2	K13345	KOG0826	172	159	125	272	293	10	14	12	126	149	152	387	335	261	27	13
ACC_10004	peripheral plasma membrane protein CASK-like	K06103	KOG0033	135	52	56	117	39	13	19	24	304	308	13	30	32	28	8	7
ACC_10005	conserved hypothetical protein		KOG3627	100	75	70	79	45	29	33	56	931	1339	34	33	273	209	118	45
ACC_10006	conserved hypothetical protein		KOG0112	384	268	294	863	247	93	76	109	416	237	166	49	56	99	209	43
ACC_10007	adenylate cyclase type 10-like	K11265		1	1	1	5	1	3	0	1	2	3	50	27	11	3	5	3
ACC_10008	probable elongator complex protein 2-like	K11374	KOG1063	267	235	224	171	232	15	29	33	325	341	93	200	501	633	39	16
ACC_10009	COUP transcription factor 2-like		KOG4215	1	2	2	2	0	1	0	2	4	7	2	1	2	10	28	16
ACC_10010	glutamate receptor, ionotropic kainate 2-like		KOG1054	134	95	102	73	13	10	7	16	74	30	2	3	0	0	0	0
ACC_10011	probable nuclear hormone receptor HR38	K08558	KOG4217	117	124	286	1027	169	10	14	29	85	90	31	51	23	7	21	28
ACC_10012	nicotinic acetylcholine receptor beta 1	K05312	KOG3645	142	57	59	126	44	6	8	10	29	35	1	1	0	1	4	1
ACC_10013	conserved hypothetical protein	K04008		171	54	48	87	21	3	2	2	51	42	3	8	0	1	2	0
ACC_10014	leucine-rich repeat-containing protein 51-like		KOG1644	33	25	17	30	24	1	6	5	27	9	2	44	31	43	0	0
ACC_10015	conserved hypothetical protein			110	75	85	50	84	3	35	42	67	49	25	41	37	27	26	18
ACC_10016	26S proteasome non-ATPase regulatory subunit 1-like isofor		KOG2062	39	14	18	80	22	6	6	5	57	63	27	30	51	94	32	12
ACC_10017	conserved hypothetical protein		KOG0994	174	16	2	9	2	8	16	5	298	61	52	1872	3	5	7	23
ACC_10018	LOW QUALITY PROTEIN	K00962	KOG1067	885	771	727	874	1107	81	135	126	785	607	332	552	950	1359	132	41
ACC_10019	dopamine receptor 2-like		KOG4220	41	38	41	47	5	1	1	1	18	19	1	0	1	2	1	0
ACC_10020	low affinity immunoglobulin epsilon Fc receptor			12	2	1	11	0	0	1	0	21	10	6	32	48	86	72	18
ACC_10021	ubiquitin carboxyl-terminal hydrolase 22-like	K11366	KOG1867	213	121	113	215	140	14	16	13	266	273	56	163	496	411	126	20
ACC_10022	potassium/sodium hyperpolarization-activated cyc	K04955	KOG0498	618	231	229	504	131	47	37	76	248	164	211	93	134	41	105	20
ACC_10023	Glutamate receptor, ionotropic kainate 2		KOG1054	221	53	38	339	105	15	33	15	532	539	15	19	22	27	27	36
ACC_10024	Full=Apamin; Flags			0	0	0	0	0	0	0	0	0	0	0	0	0	0	0	0
ACC_10025	protein yellow			2	0	2	1	0	0	0	0	1	10	4	3	4	2	0	0
ACC_10026	probable G-protein coupled receptor 158-like		KOG4418	26	6	17	22	13	4	2	4	42	29	64	15	43	18	11	14
ACC_10027	probable proline--tRNA ligase, mitochondrial-like	K01881	KOG2324	233	205	168	223	284	30	67	72	265	203	116	324	528	491	77	34
ACC_10028	putative odorant receptor 13a-like	K08471		0	0	0	0	0	0	0	0	1	0	9	0	0	0	1	0
ACC_10029	conserved hypothetical protein		KOG1601	109	103	78	77	30	7	8	18	322	419	16	74	48	57	50	15
ACC_10030	orphan myosuppressin-like receptor		KOG4220	16	8	6	30	4	1	0	0	11	6	1	1	1	0	3	0

ACC_10031	twist	K09069	KOG4447	1	1	3	4	3	0	0	1	8	19	2	6	19	8	5	4
ACC_10032	probable enoyl-CoA hydratase, mitochondrial	K07511	KOG1680	318	191	140	467	418	37	58	89	908	453	372	1495	5325	5594	704	243
ACC_10033	voltage-dependent T-type calcium channel subunit alpha-1G		KOG2302	14	8	6	16	7	0	1	0	12	5	2	2	25	1	0	0
ACC_10034	acetylcholine receptor subunit alpha-like 2-like isof	K05312	KOG3646	533	259	294	610	151	23	19	30	127	49	2	1	2	13	18	3
ACC_10035	conserved hypothetical protein			166	128	136	202	133	48	36	61	465	232	739	1153	44	31	10	10
ACC_10036	conserved hypothetical protein		KOG4221	36	22	31	27	8	3	3	2	23	32	1	2	2	1	2	4
ACC_10037	conserved hypothetical protein			5	3	12	33	25	1	3	8	3	3	6	0	117	9	5	11
ACC_10038	conserved hypothetical protein			37	26	7	35	7	7	8	10	70	31	7	18	34	26	38	7
ACC_10039	adenylate kinase isoenzyme 5-like		KOG3079	0	0	2	7	0	0	0	2	4	1	0	0	1	0	0	0
ACC_10040	glypican-4-like	K08110	KOG3821	74	72	77	132	35	6	7	17	49	52	5	9	74	140	212	23
ACC_10041	hypothetical protein			14	10	7	20	7	5	3	10	8	18	4	4	4	13	16	2
ACC_10042	serine/threonine-protein phosphatase PP1-gamma	K06269	KOG0374	1429	729	591	1134	787	212	264	390	1450	1302	461	1704	1938	2074	1001	271
ACC_10043	thioredoxin domain-containing protein 11-like isoform 1		KOG0190	207	187	130	203	161	31	51	44	454	350	205	284	468	349	49	18
ACC_10044	conserved hypothetical protein			1	1	0	2	1	1	3	3	18	12	3	2	2	85	103	10
ACC_10045	isocitrate dehydrogenase	K00030	KOG0784	629	256	255	763	836	49	48	78	2072	668	312	1885	1151	1381	102	28
ACC_10046	conserved hypothetical protein		KOG1259	13	11	10	15	12	0	2	2	43	54	24	10	9	19	21	24
ACC_10047	allatostatin-A receptor-like	K04233	KOG4219	232	98	114	235	64	7	12	13	65	28	16	1	5	14	28	5
ACC_10048	hypothetical protein			9	0	1	0	0	0	0	0	1	1	0	239	626	618	556	703
ACC_10049	RING finger protein 207-like		KOG2177	625	265	281	628	217	35	31	38	250	133	87	59	8	35	18	6
ACC_10050	LOW QUALITY PROTEIN		KOG3508	684	532	629	325	95	12	14	29	784	559	79	223	104	123	105	32
ACC_10051	mothers against decapentaplegic homolog 6-like	K04677	KOG3701	47	25	23	27	21	4	4	5	106	95	14	14	24	22	25	7
ACC_10052	probable tRNA (uracil-O(2)-)-methyltransferase-like	K15447	KOG3790	755	406	418	619	565	107	241	195	853	555	475	558	750	886	284	108
ACC_10053	conserved hypothetical protein	K08802	KOG4717	129	115	119	116	29	5	2	12	410	405	19	38	37	42	39	11
ACC_10054	conserved hypothetical protein			217	63	72	54	36	2	4	8	99	58	20	38	23	112	24	3
ACC_10055	vesicle transport protein SEC20-like	K08497		52	52	40	138	95	6	14	15	80	56	54	149	135	183	34	9
ACC_10056	conserved hypothetical protein			0	0	0	0	0	0	0	0	1	0	2	0	0	753	1810	212
ACC_10057	conserved hypothetical protein		KOG1144	271	143	181	177	69	65	69	88	1179	514	193	55	648	886	6477	15035
ACC_10058	conserved hypothetical protein			425	421	397	490	123	23	40	56	313	310	37	29	24	25	17	7
ACC_10059	putative amino acid permease F13H10.3-like			27	20	16	16	20	2	1	1	270	353	65	225	487	294	9	2
ACC_10060	conserved hypothetical protein			39	56	81	409	79	211	246	345	1914	1385	15	65	1	3	6	26
ACC_10061	conserved hypothetical protein			211	228	112	81	23	48	51	75	507	494	61	206	133	133	116	46
ACC_10062	conserved hypothetical protein			82	6	3	38	10	2	3	2	25	9	17	25	0	6	6	9
ACC_10063	retinal homeobox protein Rx-like		KOG0484	4	0	5	9	0	0	0	0	2	0	1	1	0	0	2	2
ACC_10064	conserved hypothetical protein		KOG2462	5	0	2	2	5	8	7	16	132	37	34	27	75	110	11	1
ACC_10065	conserved hypothetical protein		KOG0527	12	3	1	15	4	0	1	2	1	4	4	1	2	0	1	3
ACC_10066	glutathione synthetase-like	K01920	KOG0021	50	27	18	46	58	0	4	6	25	40	11	58	122	112	19	1
ACC_10067	hypothetical protein			0	0	0	0	0	0	0	0	1	0	1	0	1	1	5	2
ACC_10068	UPF0670 protein CG4666-like		KOG4366	28	20	13	147	72	9	12	24	116	116	142	249	174	478	117	40
ACC_10069	voltage-dependent T-type calcium channel subunit	K05315	KOG2302	91	45	37	73	36	4	3	4	27	22	3	3	31	4	0	0
ACC_10070	beta-1,3-galactosyltransferase 1-like	K07819	KOG2287	124	80	64	79	99	5	8	4	260	277	33	310	230	118	6	1
ACC_10071	conserved hypothetical protein		KOG3513	67	21	25	79	14	2	5	6	17	27	8	5	6	5	21	7
ACC_10072	conserved hypothetical protein	K09216	KOG3576	24	20	14	39	22	9	11	21	251	263	195	154	33	111	314	244
ACC_10073	rho guanine nucleotide exchange factor 10-like			3	1	2	3	1	0	0	2	17	20	0	9	14	13	11	3
ACC_10074	lachesin-like		KOG3513	46	19	20	60	19	7	2	12	20	9	0	4	3	1	6	2
ACC_10075	conserved hypothetical protein			16	1	0	2	2	1	0	1	8	1	1	30	0	0	2	7
ACC_10076	fasciclin-1			37	6	4	4	3	0	1	1	28	13	2	1	1	5	0	0
ACC_10077	conserved hypothetical protein		KOG4701	107	113	136	110	29	28	30	26	358	442	19	54	43	53	51	22
ACC_10078	conserved hypothetical protein		KOG0921	3	1	0	3	0	2	0	3	20	31	1	4	1	8	3	5
ACC_10079	LOW QUALITY PROTEIN		KOG3679	69	25	32	96	24	4	4	2	54	129	328	98	4	3	24	16
ACC_10080	conserved hypothetical protein			89	67	80	118	113	6	11	10	97	83	56	112	108	79	21	3
ACC_10081	conserved hypothetical protein			39	6	11	21	3	1	1	2	17	19	1	2	0	0	3	1
ACC_10082	dynein heavy chain 5, axonemal		KOG3595	145	104	90	103	92	11	23	27	180	105	15	11	24	8	6	4
ACC_10083	conserved hypothetical protein		KOG1883	348	328	353	243	103	39	44	55	698	408	72	172	304	235	90	34
ACC_10084	tumor protein D54-like isoform 1		KOG4010	157	120	87	326	129	69	101	184	389	186	158	381	375	233	135	85
ACC_10085	conserved hypothetical protein			23	2	4	15	7	1	5	1	37	120	9	43	18	27	28	14
ACC_10086	semaphorin-1A-like, partial		KOG3611	1040	482	518	1326	895	228	527	484	441	207	434	105	84	131	587	294
ACC_10087	alanine--tRNA ligase, mitochondrial-like	K01872	KOG0188	303	187	183	337	371	23	53	58	229	249	177	189	345	270	71	30

ACC_10088	conserved hypothetical protein		KOG2849	155	53	66	126	142	20	16	30	1565	1239	21	42	294	36	8	1
ACC_10089	conserved hypothetical protein		KOG1052	4	3	3	1	3	0	0	0	9	7	2	1	1	5	2	1
ACC_10090	conserved hypothetical protein			0	0	2	1	2	0	0	0	3	0	1	0	0	0	1	2
ACC_10091	conserved hypothetical protein			1	0	0	2	3	1	0	0	1	0	0	0	1	1	1	1
ACC_10092	conserved hypothetical protein			0	0	2	3	2	0	1	1	2	13	1	0	3	5	3	2
ACC_10093	conserved hypothetical protein		KOG4289	87	57	66	56	41	12	34	30	92	51	82	6	16	33	108	62
ACC_10094	conserved hypothetical protein		KOG0596	48	23	14	6	2	14	24	22	230	52	30	41	1672	20	8	15
ACC_10095	conserved hypothetical protein			4	2	1	1	2	0	1	1	15	0	2	34	9	34	2	2
ACC_10096	conserved hypothetical protein		KOG1015	4790	3673	3255	4029	2367	1052	2088	2402	5292	2296	936	307	877	1027	3591	2683
ACC_10097	putative amino acid permease F13H10.3-like	K14995		36	20	21	22	45	3	6	2	297	276	88	189	464	292	18	3
ACC_10098	conserved hypothetical protein			145	84	96	399	66	8	15	20	32	54	5	10	6	8	32	13
ACC_10099	neurogenic locus Notch protein-like	K02599	KOG4177	99	69	75	50	18	3	5	7	119	96	12	19	44	64	75	30
ACC_10100	laccase-4-like		KOG1263	167	108	103	168	120	17	19	31	146	85	48	57	0	20	21	17
ACC_10101	conserved hypothetical protein	K13168	KOG2548	1127	874	845	909	769	81	111	149	1072	913	343	371	653	715	166	84
ACC_10102	zinc finger protein 346-like			79	76	79	170	46	7	13	20	56	45	21	29	21	40	55	18
ACC_10103	conserved hypothetical protein			15	8	15	49	15	1	0	3	62	20	16	13	46	43	33	19
ACC_10104	Longitudinals lacking protein, isoforms A/B/D/L		KOG3623	49	47	49	71	26	13	13	22	24	38	13	3	1	3	45	28
ACC_10105	conserved hypothetical protein			11	7	6	17	1	2	0	2	62	72	5	15	101	50	53	94
ACC_10106	LOW QUALITY PROTEIN		KOG2041	545	402	384	748	182	57	81	99	427	226	173	54	7	13	36	10
ACC_10107	conserved hypothetical protein	K16529	KOG3520	361	249	179	208	108	18	28	51	480	424	66	83	191	164	61	30
ACC_10108	conserved hypothetical protein		KOG0613	45	16	12	35	4	4	8	6	616	202	19	9	60	73	81	35
ACC_10109	laccase-2-like		KOG1263	14	7	6	64	14	6	8	12	47	37	5	4	276	370	608	402
ACC_10110	collagen alpha-1(XI) chain-like isoform 1	K06236	KOG3546	22	3	5	3	2	0	0	0	34	1	0	3	0	0	1	2
ACC_10111	conserved hypothetical protein	K12494	KOG0932	209	147	135	153	80	17	25	19	294	223	33	67	107	60	44	21
ACC_10112	hypothetical protein			32	12	23	13	14	6	10	6	363	548	11	55	688	136	34	16
ACC_10113	transmembrane protein 35-like			368	122	86	219	264	27	32	56	356	94	168	712	165	125	36	15
ACC_10114	conserved hypothetical protein			280	113	129	145	38	20	22	23	163	88	113	29	95	91	81	52
ACC_10115	conserved hypothetical protein			162	113	98	324	144	15	55	30	62	23	436	126	357	383	45	49
ACC_10116	conserved hypothetical protein		KOG1375	369	180	192	787	326	27	26	33	216	92	52	182	1067	1884	791	6322
ACC_10117	28S ribosomal protein S18a, mitochondrial isoform	K02963	KOG3162	111	68	76	196	240	7	17	23	91	60	180	203	267	332	71	68
ACC_10118	conserved hypothetical protein			69669	14658	7217	30612	11136	23682	40178	49095	19802	28571	10222	35026	878	471	300442	247161
ACC_10119	potassium voltage-gated channel protein Shab-like		KOG1924	952	464	420	807	302	116	274	323	285	239	170	84	17	11	142	144
ACC_10120	conserved hypothetical protein	K09228	KOG2462	72	34	34	44	15	6	7	11	183	105	21	14	47	50	48	21
ACC_10121	metalloendopeptidase OMA1, mitochondrial-like		KOG2661	309	212	198	252	304	27	45	46	178	145	378	312	94	77	14	8
ACC_10122	conserved hypothetical protein			5	1	3	7	5	1	0	0	8	12	3	0	0	0	2	0
ACC_10123	conserved hypothetical protein			54	57	43	43	10	6	5	3	11	11	1	1	0	1	3	1
ACC_10124	conserved hypothetical protein	K09228	KOG2462	89	47	42	50	17	8	7	14	210	151	17	26	70	51	60	41
ACC_10125	DNA mismatch repair protein Msh2-like			10	5	8	8	10	0	3	0	14	41	16	7	14	6	0	0
ACC_10126	mitochondrial sodium/hydrogen exchanger NHA2-like			9	3	4	21	2	2	1	1	31	40	9	146	3	2	1	2
ACC_10127	conserved hypothetical protein			233	161	194	132	55	15	18	24	226	207	31	52	157	147	44	17
ACC_10128	LOW QUALITY PROTEIN			227	238	221	114	101	7	21	11	235	131	58	46	81	113	26	8
ACC_10129	conserved hypothetical protein			213	48	43	86	41	15	26	35	200	64	26	30	40	73	102	81
ACC_10130	conserved hypothetical protein		KOG1808	49	36	24	26	20	3	8	5	137	182	6	51	240	241	12	12
ACC_10131	conserved hypothetical protein			55	31	36	38	6	4	2	1	157	162	12	53	6	21	20	15
ACC_10132	conserved hypothetical protein			15	10	10	18	4	6	3	7	31	50	4	8	7	5	11	18
ACC_10133	protein distal antenna-like isoform 1			60	0	61	102	28	4	11	6	60	27	11	27	36	79	88	71
ACC_10134	calcium-transporting ATPase sarcoplasmic/endopla	K05853	KOG0202	801	0	308	301	86	74	106	106	4527	5818	124	781	415	382	216	100
ACC_10135	cyclin-dependent kinase 7-like	K02202	KOG0659	181	0	140	335	285	19	26	27	248	134	110	289	252	347	34	13
ACC_10136	conserved hypothetical protein			162	0	149	230	77	9	7	11	136	68	9	2	4	5	2	1
ACC_10137	multiple inositol polyphosphate phosphatase 1-like		KOG1382	151	0	56	167	98	7	10	8	353	91	521	1081	27	20	9	6
ACC_10138	zinc finger protein 717-like			54	0	59	23	12	6	5	10	59	45	8	5	7	6	15	5
ACC_10139	conserved hypothetical protein		KOG4485	94	0	70	72	22	5	6	6	99	70	6	13	7	12	22	6
ACC_10140	conserved hypothetical protein		KOG2462	46	0	26	47	25	7	25	13	88	61	25	14	20	29	96	30
ACC_10141	conserved hypothetical protein			97	0	89	139	49	3	1	5	51	50	8	24	4	4	2	0
ACC_10142	Prohormone-3			719	0	378	922	539	39	66	56	478	351	98	72	0	6	5	2
ACC_10143	general transcription factor IIH subunit 5-like	K10845	KOG3451	70	0	55	146	88	5	4	8	57	36	68	125	49	142	24	9
ACC_10144	ras-specific guanine nucleotide-releasing factor 1-li	K12326	KOG3629	508	0	420	770	425	95	168	208	321	75	20	14	0	4	4	2

ACC_10145	acetylcholine receptor subunit alpha-like isoform 2		KOG3645	26	0	5	9	4	1	2	0	7	7	2	1	0	0	0	1
ACC_10146	conserved hypothetical protein		KOG0517	798	0	304	1368	239	203	349	317	1609	1805	118	97	19	11	298	163
ACC_10147	dynactin subunit 6	K10428	KOG4042	114	0	98	156	130	7	6	14	75	24	48	134	122	130	19	16
ACC_10148	conserved hypothetical protein			123	0	88	244	51	13	13	16	45	132	21	4	1	1	2	1
ACC_10149	conserved hypothetical protein			22	0	6	71	11	1	4	2	95	220	29	137	4	11	41	4
ACC_10150	protein prune homolog	K01514	KOG4129	209	0	189	407	416	20	36	48	257	214	172	543	62	93	21	4
ACC_10151	putative SERF-like protein-like			385	0	169	618	270	59	99	116	391	115	246	300	279	223	292	306
ACC_10152	neuropeptides capa receptor-like			14	0	7	31	28	0	4	2	51	6	30	16	25	57	16	8
ACC_10153	protein SZT2-like, partial			63	0	32	36	23	4	3	12	44	105	6	26	109	89	12	3
ACC_10154	conserved hypothetical protein			64	0	27	28	24	5	5	3	121	116	8	8	10	7	8	3
ACC_10155	serine/threonine-protein kinase NLK-like	K04468	KOG0664	144	0	142	155	43	33	69	90	58	55	32	13	10	13	312	324
ACC_10156	conserved hypothetical protein			23	0	4	75	19	2	5	1	192	291	36	13	2	2	14	22
ACC_10157	EF-hand domain-containing family member A2-like		KOG2643	55	0	53	162	83	7	8	14	50	23	57	25	61	44	9	3
ACC_10158	glutamate decarboxylase-like	K01580	KOG0629	802	0	216	315	111	10	11	16	193	108	8	11	0	6	7	1
ACC_10159	conserved hypothetical protein			30	0	35	62	27	6	10	12	17	16	13	0	0	4	5	0
ACC_10160	endophilin-A isoform 1	K11247	KOG1118	875	0	421	877	313	25	32	67	703	485	32	101	97	100	43	20
ACC_10161	probable E3 ubiquitin-protein ligase MYCBP2-like	K10693	KOG1428	107	0	61	153	84	7	8	11	192	86	44	13	34	45	19	6
ACC_10162	conserved hypothetical protein		KOG3915	141	0	128	378	51	5	4	16	73	49	1	1	0	1	11	9
ACC_10163	vacuolar protein sorting-associated protein 35 isoform 1		KOG1107	534	0	424	645	789	49	145	133	613	447	392	505	835	896	192	97
ACC_10164	cubilin-like		KOG4586	94	0	35	59	15	2	6	9	52	57	23	4	8	0	9	4
ACC_10165	talin-1-like	K06271	KOG4261	709	617	505	420	123	61	104	108	1888	2159	97	263	253	257	405	151
ACC_10166	inositol polyphosphate 1-phosphatase	K01107	KOG3099	378	264	272	409	426	25	27	34	687	401	96	225	128	125	23	3
ACC_10167	pancreatic triacylglycerol lipase-like			9	56	14	241	98	14	17	15	609	218	252	1180	3	329	51	8
ACC_10168	conserved hypothetical protein			4	3	3	7	8	1	1	4	6	3	30	40	176	7	2	1
ACC_10169	DNA methyltransferase 3	K00558		104	66	78	177	74	0	42	54	245	136	148	75	65	43	106	76
ACC_10170	odorant binding protein ASP4			26	32	41	209	90	31	65	135	397	152	6717	1039	0	1	9	1
ACC_10171	hyaluronidase	K01197		9	6	8	19	4	69	8	2	59	14	18	4	2	4	18	13
ACC_10172	silk protein 4		KOG0994	3	7	9	27	5	5	1	1	9	2	13	3	1	0	5	25
ACC_10173	silk protein 2		KOG0994	2	6	3	44	9	0	2	1	10	6	5	3	3	3	6	14
ACC_10174	silk protein 1		KOG0994	15	6	7	49	8	3	5	1	28	13	8	5	6	1	3	10
ACC_10175	silk protein 3		KOG0994	1	0	0	3	2	1	1	0	0	0	4	1	5	0	0	20
ACC_10176	Melittin			1	2	1	7	0	2	1	2	8	1	5	4	0	2	0	0
ACC_10177	homeobox protein	K09300	KOG0487	1	0	1	0	0	0	0	0	1	2	0	0	10	17	73	28
ACC_10178	homeobox protein		KOG1168	20	8	5	30	1	0	0	1	6	4	42	14	0	0	1	0
ACC_10179	homeobox protein	K09301	KOG0844	348	261	330	263	71	0	45	53	53	52	29	24	106	210	470	17
ACC_10180	Sex combs reduced homeotic protein	K09311	KOG0489	5	0	11	6	0	25	1	2	7	29	1	0	12	8	48	24
ACC_10181	homeotic protein antennapedia-like	K09307	KOG0489	17	5	9	14	0	1	1	0	22	36	4	2	25	77	1118	748
ACC_10182	odorant binding protein			1	0	1	0	1	1	0	0	1	0	2	0	3	6	9	0

Supplementary Table 6. Enrichment analysis of methylation genes of *A.cerana*

	Gene Number in each Pathway			Gene Number in all the Pathway			Enrichment of Higher methylation gene		Enrichment of Rare methylation gene	
Pathway	Higher methylation	Rare methylation	Total Acc	Higher methylation	Rare methylation	Total Acc	P value	FDR	P value	FDR
00072 Synthesis and degradation of ketone b	4	0	4	793	248	2237	0	0	0.37521615	0.536023072
00362 Benzoate degradation	6	0	6	793	248	2237	0	0	0.506315991	0.654228527
00626 Naphthalene degradation	1	0	1	793	248	2237	0	0	0.110862763	0.247557625
00930 Caprolactam degradation	4	0	4	793	248	2237	0	0	0.37521615	0.536023072
00510 N-Glycan biosynthesis	24	2	34	793	248	2237	5.86E-06	0.000269355	0.745203817	0.878958348
04141 Protein processing in endoplasmic reti	57	3	104	793	248	2237	1.17E-05	0.000449072	0.998134808	0.998134808
00401 Novobiocin biosynthesis	0	1	1	793	248	2237	0.354492624	0.885503665	0	0
00624 Polycyclic aromatic hydrocarbon degra	0	1	1	793	248	2237	0.354492624	0.885503665	0	0
00944 Flavone and flavonol biosynthesis	0	1	1	793	248	2237	0.354492624	0.885503665	0	0
00311 Penicillin and cephalosporin biosynthe	0	2	2	793	248	2237	0.583422566	0.999990869	0	0
00472 D-Arginine and D-ornithine metabolism	0	2	2	793	248	2237	0.583422566	0.999990869	0	0
04080 Neuroactive ligand-receptor interactio	1	14	33	793	248	2237	0.999990869	0.999990869	5.15E-07	1.97E-05
04514 Cell adhesion molecules (CAMs)	1	5	6	793	248	2237	0.689597392	0.999990869	1.76E-06	5.78E-05
00981 Insect hormone biosynthesis	1	6	9	793	248	2237	0.88485671	0.999990869	5.63E-06	0.000161881
00982 Drug metabolism - cytochrome P450	3	6	11	793	248	2237	0.587186701	0.999990869	4.23E-05	0.001082089
04726 Serotonergic synapse	3	10	27	793	248	2237	0.995716714	0.999990869	6.45E-05	0.001483419
00140 Steroid hormone biosynthesis	1	4	6	793	248	2237	0.689597392	0.999990869	8.81E-05	0.001842611
04728 Dopaminergic synapse	3	13	44	793	248	2237	0.999990731	0.999990869	0.000157901	0.002793626
04744 Phototransduction	0	3	4	793	248	2237	0.826633858	0.999990869	0.000147826	0.002793626
04360 Axon guidance	6	10	30	793	248	2237	0.948199189	0.999990869	0.000199078	0.00327056
00950 Isoquinoline alkaloid biosynthesis	2	4	7	793	248	2237	0.478085923	0.973095242	0.000280523	0.004012019
00500 Starch and sucrose metabolism	3	8	22	793	248	2237	0.978281729	0.999990869	0.000296839	0.004012019
04730 Long-term depression	2	7	18	793	248	2237	0.978796079	0.999990869	0.000331428	0.004012019
04960 Aldosterone-regulated sodium reabsor	1	6	14	793	248	2237	0.981324158	0.999990869	0.000327562	0.004012019
04974 Protein digestion and absorption	2	7	18	793	248	2237	0.978796079	0.999990869	0.000331428	0.004012019
00627 Aminobenzoate degradation	3	5	11	793	248	2237	0.587186701	0.999990869	0.000499689	0.005472786
04973 Carbohydrate digestion and absorption	0	5	11	793	248	2237	0.99200118	0.999990869	0.000499689	0.005472786
00980 Metabolism of xenobiotics by cytochro	4	5	12	793	248	2237	0.429693747	0.915088534	0.000907315	0.009485568
04970 Salivary secretion	1	8	26	793	248	2237	0.999836465	0.999990869	0.001248565	0.01248565
00053 Ascorbate and aldarate metabolism	4	4	9	793	248	2237	0.178939901	0.674691429	0.001393337	0.012818701
00830 Retinol metabolism	2	4	9	793	248	2237	0.67353218	0.999990869	0.001393337	0.012818701
04540 Gap junction	4	9	32	793	248	2237	0.99666259	0.999990869	0.001645812	0.014019881
04662 B cell receptor signaling pathway	6	7	22	793	248	2237	0.714117683	0.999990869	0.001627991	0.014019881
00591 Linoleic acid metabolism	0	3	6	793	248	2237	0.927921679	0.999990869	0.001847307	0.015174309
00983 Drug metabolism - other enzymes	3	6	18	793	248	2237	0.928040851	0.999990869	0.002051561	0.016270998
04020 Calcium signaling pathway	3	11	44	793	248	2237	0.999990731	0.999990869	0.002152855	0.016505224
04370 VEGF signaling pathway	3	7	23	793	248	2237	0.984148706	0.999990869	0.002261014	0.016775266
00350 Tyrosine metabolism	4	5	14	793	248	2237	0.591596211	0.999990869	0.002432003	0.017480022
00740 Riboflavin metabolism	3	3	7	793	248	2237	0.206988931	0.74386647	0.003936353	0.027435185
04512 ECM-receptor interaction	1	4	11	793	248	2237	0.943344484	0.999990869	0.004233745	0.028640038

04614 Renin-angiotensin system	0	2	4	793	248	2237	0.826633858	0.999990869	0.004948255	0.032517104
04650 Natural killer cell mediated cytotoxicity	5	6	21	793	248	2237	0.812091646	0.999990869	0.005594834	0.03574477
04310 Wnt signaling pathway	10	12	58	793	248	2237	0.998313361	0.999990869	0.008962314	0.055711684
00562 Inositol phosphate metabolism	10	9	40	793	248	2237	0.892020722	0.999990869	0.009696171	0.057290464
04971 Gastric acid secretion	1	6	23	793	248	2237	0.999448904	0.999990869	0.00971447	0.057290464
00290 Valine, leucine and isoleucine biosynth	1	1	2	793	248	2237	0.125562683	0.56291021	0.012246468	0.059929524
00340 Histidine metabolism	3	3	9	793	248	2237	0.402298075	0.898335508	0.01183124	0.059929524
00430 Taurine and hypotaurine metabolism	0	2	5	793	248	2237	0.888200965	0.999990869	0.011358859	0.059929524
00901 Indole alkaloid biosynthesis	0	1	2	793	248	2237	0.583422566	0.999990869	0.012246468	0.059929524
00908 Zeatin biosynthesis	0	1	2	793	248	2237	0.583422566	0.999990869	0.012246468	0.059929524
00965 Betalain biosynthesis	0	1	2	793	248	2237	0.583422566	0.999990869	0.012246468	0.059929524
04060 Cytokine-cytokine receptor interaction	0	2	5	793	248	2237	0.888200965	0.999990869	0.011358859	0.059929524
04640 Hematopoietic cell lineage	0	1	2	793	248	2237	0.583422566	0.999990869	0.012246468	0.059929524
00040 Pentose and glucuronate interconversi	3	4	14	793	248	2237	0.790536531	0.999990869	0.01386931	0.064010965
04270 Vascular smooth muscle contraction	3	8	36	793	248	2237	0.999817899	0.999990869	0.013915427	0.064010965
04976 Bile secretion	0	5	19	793	248	2237	0.999765705	0.999990869	0.013625455	0.064010965
00260 Glycine, serine and threonine metaboli	11	6	25	793	248	2237	0.134368617	0.56291021	0.015698993	0.066866081
00480 Glutathione metabolism	9	6	25	793	248	2237	0.387420104	0.898335508	0.015698993	0.066866081
04380 Osteoclast differentiation	6	6	25	793	248	2237	0.839661104	0.999990869	0.015698993	0.066866081
04723 Retrograde endocannabinoid signaling	3	6	25	793	248	2237	0.991684824	0.999990869	0.015698993	0.066866081
00360 Phenylalanine metabolism	2	3	10	793	248	2237	0.748558079	0.999990869	0.018027769	0.071489429
00790 Folate biosynthesis	1	3	10	793	248	2237	0.918931368	0.999990869	0.018027769	0.071489429
04210 Apoptosis	5	5	20	793	248	2237	0.768682609	0.999990869	0.017702993	0.071489429
04961 Endocrine and other factor-regulated c	1	5	20	793	248	2237	0.998174128	0.999990869	0.017702993	0.071489429
00511 Other glycan degradation	3	4	15	793	248	2237	0.837202448	0.999990869	0.018962847	0.073922963
00920 Sulfur metabolism	4	2	6	793	248	2237	0.023513716	0.200302024	0.020870411	0.075232696
00532 Glycosaminoglycan biosynthesis - chon	1	2	6	793	248	2237	0.689597392	0.999990869	0.020870411	0.075232696
04151 PI3K-Akt signaling pathway	20	13	71	793	248	2237	0.881494172	0.999990869	0.020934315	0.075232696
04727 GABAergic synapse	4	7	32	793	248	2237	0.99666259	0.999990869	0.020035934	0.075232696
04742 Taste transduction	0	2	6	793	248	2237	0.927921679	0.999990869	0.020870411	0.075232696
04745 Phototransduction - fly	0	5	21	793	248	2237	0.999903315	0.999990869	0.022546257	0.079779062
00785 Lipoic acid metabolism	2	1	3	793	248	2237	0.044438515	0.300613487	0.034043537	0.107260459
00960 Tropane, piperidine and pyridine alkalo	2	1	3	793	248	2237	0.044438515	0.300613487	0.034043537	0.107260459
00130 Ubiquinone and other terpenoid-quino	4	2	7	793	248	2237	0.05836532	0.353263777	0.033570955	0.107260459
00460 Cyanoamino acid metabolism	1	1	3	793	248	2237	0.287811017	0.827456674	0.034043537	0.107260459
00073 Cutin, suberine and wax biosynthesis	0	1	3	793	248	2237	0.73122834	0.999990869	0.034043537	0.107260459
00604 Glycosphingolipid biosynthesis - gangli	1	2	7	793	248	2237	0.77420198	0.999990869	0.033570955	0.107260459
04711 Circadian rhythm - fly	0	2	7	793	248	2237	0.953541629	0.999990869	0.033570955	0.107260459
04725 Cholinergic synapse	4	7	35	793	248	2237	0.998746753	0.999990869	0.033488041	0.107260459
00514 Other types of O-glycan biosynthesis	6	3	12	793	248	2237	0.089477758	0.496616561	0.03556022	0.107616456
00590 Arachidonic acid metabolism	4	3	12	793	248	2237	0.429693747	0.915088534	0.03556022	0.107616456
04964 Proximal tubule bicarbonate reclamati	2	3	12	793	248	2237	0.856785401	0.999990869	0.03556022	0.107616456
04724 Glutamatergic synapse	4	7	36	793	248	2237	0.999102056	0.999990869	0.039080217	0.116733116
00531 Glycosaminoglycan degradation	4	3	13	793	248	2237	0.513465842	0.999990869	0.04701607	0.138637131
00330 Arginine and proline metabolism	8	6	31	793	248	2237	0.826102075	0.999990869	0.048315923	0.138908279
04070 Phosphatidylinositol signaling system	11	8	44	793	248	2237	0.906161709	0.999990869	0.047762983	0.138908279

04122 Sulfur relay system	1	2	8	793	248	2237	0.837895703	0.999990869	0.049398224	0.140266562
04660 T cell receptor signaling pathway	7	6	32	793	248	2237	0.927232835	0.999990869	0.05623432	0.15773041
00910 Nitrogen metabolism	5	3	14	793	248	2237	0.372831177	0.89180277	0.060274775	0.167026484
04340 Hedgehog signaling pathway	1	4	20	793	248	2237	0.998174128	0.999990869	0.0618903	0.16746787
04510 Focal adhesion	11	9	53	793	248	2237	0.985493752	0.999990869	0.061570804	0.16746787
00400 Phenylalanine, tyrosine and tryptophar	2	1	4	793	248	2237	0.130610247	0.56291021	0.063138819	0.168859633
02010 ABC transporters	1	2	9	793	248	2237	0.88485671	0.999990869	0.068181716	0.180250513
04978 Mineral absorption	4	3	15	793	248	2237	0.662205259	0.999990869	0.075297294	0.196799746
04620 Toll-like receptor signaling pathway	7	4	22	793	248	2237	0.544536438	0.999990869	0.087707805	0.226660619
00052 Galactose metabolism	7	3	16	793	248	2237	0.168227211	0.655800991	0.092013396	0.23053328
00410 beta-Alanine metabolism	6	3	16	793	248	2237	0.325175756	0.885503665	0.092013396	0.23053328
04142 Lysosome	20	9	57	793	248	2237	0.462060279	0.964806757	0.092213312	0.23053328
04012 ErbB signaling pathway	5	5	29	793	248	2237	0.973615482	0.999990869	0.093450994	0.231115363
00061 Fatty acid biosynthesis	2	1	5	793	248	2237	0.241981325	0.806604418	0.097658793	0.236437077
00750 Vitamin B6 metabolism	1	1	5	793	248	2237	0.580365428	0.999990869	0.097658793	0.236437077
00600 Sphingolipid metabolism	6	4	23	793	248	2237	0.761982277	0.999990869	0.102422121	0.245386331
00280 Valine, leucine and isoleucine degradat	17	5	30	793	248	2237	0.004992388	0.0820178	0.106527012	0.247557625
00524 Butirosin and neomycin biosynthesis	0	0	1	793	248	2237	0.354492624	0.885503665	0.110862763	0.247557625
00623 Toluene degradation	0	0	1	793	248	2237	0.354492624	0.885503665	0.110862763	0.247557625
00780 Biotin metabolism	0	0	1	793	248	2237	0.354492624	0.885503665	0.110862763	0.247557625
01051 Biosynthesis of ansamycins	0	0	1	793	248	2237	0.354492624	0.885503665	0.110862763	0.247557625
04610 Complement and coagulation cascades	0	0	1	793	248	2237	0.354492624	0.885503665	0.110862763	0.247557625
04350 TGF-beta signaling pathway	9	4	24	793	248	2237	0.329469241	0.885503665	0.118279204	0.259087781
04062 Chemokine signaling pathway	12	7	45	793	248	2237	0.862179443	0.999990869	0.117445484	0.259087781
04664 Fc epsilon RI signaling pathway	5	3	18	793	248	2237	0.660562452	0.999990869	0.13012042	0.282336761
04977 Vitamin digestion and absorption	2	1	6	793	248	2237	0.361901498	0.885503665	0.136052984	0.28447442
00592 alpha-Linolenic acid metabolism	0	1	6	793	248	2237	0.927921679	0.999990869	0.136052984	0.28447442
04260 Cardiac muscle contraction	5	4	25	793	248	2237	0.925277389	0.999990869	0.135218267	0.28447442
04972 Pancreatic secretion	4	5	32	793	248	2237	0.99666259	0.999990869	0.135327678	0.28447442
04013 MAPK signaling pathway - fly	3	2	12	793	248	2237	0.665933179	0.999990869	0.139598044	0.289257207
00513 Various types of N-glycan biosynthesis	15	4	26	793	248	2237	0.005707486	0.082045111	0.153168524	0.309024215
00640 Propanoate metabolism	9	3	19	793	248	2237	0.093496851	0.500099435	0.15126094	0.309024215
04670 Leukocyte transendothelial migration	4	4	26	793	248	2237	0.978844684	0.999990869	0.153168524	0.309024215
04720 Long-term potentiation	2	4	27	793	248	2237	0.999151367	0.999990869	0.172050946	0.344101892
00380 Tryptophan metabolism	6	3	20	793	248	2237	0.600434643	0.999990869	0.173603599	0.344214033
00603 Glycosphingolipid biosynthesis - globo	1	1	7	793	248	2237	0.77420198	0.999990869	0.177045795	0.345089262
04740 Olfactory transduction	0	1	7	793	248	2237	0.953541629	0.999990869	0.177045795	0.345089262
04010 MAPK signaling pathway	14	9	66	793	248	2237	0.99172025	0.999990869	0.188752239	0.364815251
00860 Porphyrin and chlorophyll metabolism	12	3	21	793	248	2237	0.011764088	0.135287008	0.196996137	0.374455468
00250 Alanine, aspartate and glutamate meta	3	3	21	793	248	2237	0.970411281	0.999990869	0.196996137	0.374455468
00903 Limonene and pinene degradation	1	0	2	793	248	2237	0.125562683	0.56291021	0.209479057	0.376407681
04112 Cell cycle - Caulobacter	1	0	2	793	248	2237	0.125562683	0.56291021	0.209479057	0.376407681
00100 Steroid biosynthesis	0	0	2	793	248	2237	0.583422566	0.999990869	0.209479057	0.376407681
00232 Caffeine metabolism	0	0	2	793	248	2237	0.583422566	0.999990869	0.209479057	0.376407681
00471 D-Glutamine and D-glutamate metabo	0	0	2	793	248	2237	0.583422566	0.999990869	0.209479057	0.376407681
00533 Glycosaminoglycan biosynthesis - kerat	0	0	2	793	248	2237	0.583422566	0.999990869	0.209479057	0.376407681

00940 Phenylpropanoid biosynthesis	0	0	2	793	248	2237	0.583422566	0.999990869	0.209479057	0.376407681
00512 Mucin type O-glycan biosynthesis	4	1	8	793	248	2237	0.110950086	0.554750431	0.219594986	0.388514205
04011 MAPK signaling pathway - yeast	2	1	8	793	248	2237	0.583120812	0.999990869	0.219594986	0.388514205
04630 Jak-STAT signaling pathway	5	2	15	793	248	2237	0.450378117	0.950339145	0.226902105	0.398275899
04910 Insulin signaling pathway	12	8	61	793	248	2237	0.994882692	0.999990869	0.228575733	0.398275899
04721 Synaptic vesicle cycle	8	4	30	793	248	2237	0.792002874	0.999990869	0.233412813	0.403646218
00620 Pyruvate metabolism	12	4	31	793	248	2237	0.280159671	0.827456674	0.255127263	0.434661262
04912 GnRH signaling pathway	3	4	31	793	248	2237	0.998914411	0.999990869	0.255127263	0.434661262
00710 Carbon fixation in photosynthetic orga	8	2	16	793	248	2237	0.071582538	0.422153427	0.258030269	0.436374719
00450 Selenocompound metabolism	3	1	9	793	248	2237	0.402298075	0.898335508	0.26285592	0.44129096
00051 Fructose and mannose metabolism	9	2	17	793	248	2237	0.041219411	0.300613487	0.289681006	0.458388964
00120 Primary bile acid biosynthesis	2	0	3	793	248	2237	0.044438515	0.300613487	0.297196817	0.458388964
00440 Phosphonate and phosphinate metabo	2	0	3	793	248	2237	0.044438515	0.300613487	0.297196817	0.458388964
00601 Glycosphingolipid biosynthesis - lacto a	2	0	3	793	248	2237	0.044438515	0.300613487	0.297196817	0.458388964
00625 Chloroalkane and chloroalkene degrad	2	0	3	793	248	2237	0.044438515	0.300613487	0.297196817	0.458388964
00300 Lysine biosynthesis	1	0	3	793	248	2237	0.287811017	0.827456674	0.297196817	0.458388964
00643 Styrene degradation	1	0	3	793	248	2237	0.287811017	0.827456674	0.297196817	0.458388964
00730 Thiamine metabolism	1	0	3	793	248	2237	0.287811017	0.827456674	0.297196817	0.458388964
03070 Bacterial secretion system	1	0	3	793	248	2237	0.287811017	0.827456674	0.297196817	0.458388964
04712 Circadian rhythm - plant	1	0	3	793	248	2237	0.287811017	0.827456674	0.297196817	0.458388964
04150 mTOR signaling pathway	9	4	32	793	248	2237	0.750391337	0.999990869	0.277312371	0.458388964
04722 Neurotrophin signaling pathway	10	5	41	793	248	2237	0.910603816	0.999990869	0.298949324	0.458388964
04962 Vasopressin-regulated water reabsorpt	8	3	25	793	248	2237	0.552462719	0.999990869	0.297947649	0.458388964
00770 Pantothenate and CoA biosynthesis	5	1	10	793	248	2237	0.099842235	0.52176564	0.306150834	0.460226744
00760 Nicotinate and nicotinamide metabolis	4	1	10	793	248	2237	0.258037567	0.827456674	0.306150834	0.460226744
04710 Circadian rhythm - mammal	2	1	10	793	248	2237	0.748558079	0.999990869	0.306150834	0.460226744
04330 Notch signaling pathway	6	2	18	793	248	2237	0.467021084	0.964806757	0.321593123	0.480301418
04626 Plant-pathogen interaction	6	1	11	793	248	2237	0.053195804	0.349572427	0.348942444	0.507954191
00670 One carbon pool by folate	5	1	11	793	248	2237	0.155817952	0.617898774	0.348942444	0.507954191
02020 Two-component system	4	1	11	793	248	2237	0.343220579	0.885503665	0.348942444	0.507954191
00534 Glycosaminoglycan biosynthesis - hepa	3	1	11	793	248	2237	0.587186701	0.999990869	0.348942444	0.507954191
04810 Regulation of actin cytoskeleton	10	6	52	793	248	2237	0.992108507	0.999990869	0.352823385	0.510373449
04115 p53 signaling pathway	10	2	20	793	248	2237	0.057244213	0.353263777	0.385282197	0.543176868
00270 Cysteine and methionine metabolism	9	2	20	793	248	2237	0.129749489	0.56291021	0.385282197	0.543176868
04966 Collecting duct acid secretion	4	1	12	793	248	2237	0.429693747	0.915088534	0.390811324	0.543176868
04114 Oocyte meiosis	14	6	54	793	248	2237	0.911490595	0.999990869	0.390960792	0.543176868
04666 Fc gamma R-mediated phagocytosis	8	4	37	793	248	2237	0.948892097	0.999990869	0.392032	0.543176868
00564 Glycerophospholipid metabolism	19	5	47	793	248	2237	0.189941163	0.704620444	0.42315488	0.582788158
04621 NOD-like receptor signaling pathway	8	1	13	793	248	2237	0.013547723	0.135477231	0.43143654	0.587162155
00565 Ether lipid metabolism	2	1	13	793	248	2237	0.893712089	0.999990869	0.43143654	0.587162155
00230 Purine metabolism	39	11	101	793	248	2237	0.214692873	0.750926392	0.445777973	0.603111375
04916 Melanogenesis	3	3	31	793	248	2237	0.998914411	0.999990869	0.455702463	0.612933137
00520 Amino sugar and nucleotide sugar met	17	4	40	793	248	2237	0.134608963	0.56291021	0.460831147	0.616227696
00650 Butanoate metabolism	9	1	14	793	248	2237	0.006546367	0.086086782	0.470579106	0.622029853
04622 RIG-I-like receptor signaling pathway	5	1	14	793	248	2237	0.372831177	0.89180277	0.470579106	0.622029853
04914 Progesterone-mediated oocyte matura	14	4	41	793	248	2237	0.498270077	0.999990869	0.483314733	0.63521365

04146 Peroxisome	20	5	51	793	248	2237	0.234804715	0.794192417	0.504882368	0.654228527
03450 Non-homologous end-joining	2	0	6	793	248	2237	0.361901498	0.885503665	0.506315991	0.654228527
04320 Dorso-ventral axis formation	2	1	16	793	248	2237	0.9587494	0.999990869	0.543787534	0.698721412
00521 Streptomycin biosynthesis	3	0	7	793	248	2237	0.206988931	0.74386647	0.561194357	0.717081678
04520 Adherens junction	6	2	27	793	248	2237	0.895644277	0.999990869	0.589731234	0.749382231
04920 Adipocytokine signaling pathway	7	1	18	793	248	2237	0.284805485	0.827456674	0.609677815	0.770471963
03010 Ribosome	14	7	76	793	248	2237	0.99923799	0.999990869	0.618945277	0.777909365
04145 Phagosome	20	4	48	793	248	2237	0.14421527	0.592312715	0.629003786	0.786254732
00630 Glyoxylate and dicarboxylate metaboli	7	1	19	793	248	2237	0.34934873	0.885503665	0.639812804	0.795442945
00062 Fatty acid elongation	7	0	9	793	248	2237	0.00150691	0.038509919	0.65338656	0.803630528
01040 Biosynthesis of unsaturated fatty acids	3	0	9	793	248	2237	0.402298075	0.898335508	0.65338656	0.803630528
03410 Base excision repair	10	1	20	793	248	2237	0.057244213	0.353263777	0.668093982	0.813024423
03060 Protein export	6	1	20	793	248	2237	0.600434643	0.999990869	0.668093982	0.813024423
03430 Mismatch repair	9	1	21	793	248	2237	0.172383911	0.660804992	0.694560379	0.840783617
04130 SNARE interactions in vesicular transpo	9	1	22	793	248	2237	0.220631417	0.75739143	0.719265137	0.866130793
00010 Glycolysis / Gluconeogenesis	14	2	33	793	248	2237	0.152250105	0.614342527	0.726273462	0.870015085
04113 Meiosis - yeast	16	3	44	793	248	2237	0.381924335	0.896353031	0.73631469	0.877473465
03030 DNA replication	18	2	34	793	248	2237	0.011256691	0.135287008	0.745203817	0.878958348
00720 Carbon fixation pathways in prokaryot	4	0	12	793	248	2237	0.429693747	0.915088534	0.756767218	0.883535331
04975 Fat digestion and absorption	2	0	12	793	248	2237	0.856785401	0.999990869	0.756767218	0.883535331
04140 Regulation of autophagy	9	0	13	793	248	2237	0.002734518	0.055055008	0.783878108	0.905989773
04612 Antigen processing and presentation	8	0	13	793	248	2237	0.013547723	0.135477231	0.783878108	0.905989773
03020 RNA polymerase	14	1	26	793	248	2237	0.016419773	0.145251835	0.801840322	0.92211637
00240 Pyrimidine metabolism	30	5	71	793	248	2237	0.090686503	0.496616561	0.817063713	0.934948528
04064 NF-kappa B signaling pathway	7	0	15	793	248	2237	0.119904874	0.56291021	0.829400185	0.944366548
04530 Tight junction	6	2	41	793	248	2237	0.997428707	0.999990869	0.849974687	0.963025508
04623 Cytosolic DNA-sensing pathway	6	0	17	793	248	2237	0.396159462	0.898335508	0.865364318	0.975655848
00900 Terpenoid backbone biosynthesis	14	0	19	793	248	2237	0.000127612	0.004192962	0.893770917	0.98357565
00030 Pentose phosphate pathway	11	0	18	793	248	2237	0.006737226	0.086086782	0.8804047	0.98357565
00563 Glycosylphosphatidylinositol(GPI)-anch	11	0	19	793	248	2237	0.012465091	0.135477231	0.893770917	0.98357565
03460 Fanconi anemia pathway	11	1	32	793	248	2237	0.469818942	0.964806757	0.885598117	0.98357565
00680 Methane metabolism	6	0	19	793	248	2237	0.535663291	0.999990869	0.893770917	0.98357565
00071 Fatty acid metabolism	15	0	24	793	248	2237	0.001732546	0.039848551	0.94131596	0.985921015
03420 Nucleotide excision repair	21	1	38	793	248	2237	0.003642631	0.064446544	0.935567933	0.985921015
00970 Aminoacyl-tRNA biosynthesis	21	1	39	793	248	2237	0.005613919	0.082045111	0.941563352	0.985921015
04110 Cell cycle	30	3	63	793	248	2237	0.015853338	0.145251835	0.93191725	0.985921015
03440 Homologous recombination	11	0	23	793	248	2237	0.073553851	0.422934645	0.933913294	0.985921015
03008 Ribosome biogenesis in eukaryotes	27	3	64	793	248	2237	0.102084582	0.52176564	0.937050377	0.985921015
03050 Proteasome	16	1	37	793	248	2237	0.121382622	0.56291021	0.928994979	0.985921015
00561 Glycerolipid metabolism	11	0	27	793	248	2237	0.215483226	0.750926392	0.958923913	0.985921015
04144 Endocytosis	27	3	70	793	248	2237	0.245853279	0.807803632	0.961141979	0.985921015
03018 RNA degradation	21	2	55	793	248	2237	0.280947048	0.827456674	0.953620279	0.985921015
00020 Citrate cycle (TCA cycle)	12	1	34	793	248	2237	0.429389017	0.915088534	0.905294208	0.985921015
00190 Oxidative phosphorylation	29	5	88	793	248	2237	0.646698499	0.999990869	0.938181122	0.985921015
00310 Lysine degradation	7	0	25	793	248	2237	0.711408219	0.999990869	0.947892391	0.985921015
03015 mRNA surveillance pathway	19	2	58	793	248	2237	0.611317133	0.999990869	0.96448795	0.985921015

03040 Spliceosome	30	6	102	793	248	2237	0.885628811	0.999990869	0.947701229	0.985921015
03320 PPAR signaling pathway	7	0	26	793	248	2237	0.756980898	0.999990869	0.953734474	0.985921015
03013 RNA transport	40	6	110	793	248	2237	0.376108125	0.89180277	0.969829515	0.986994639
03022 Basal transcription factors	20	0	33	793	248	2237	0.000833171	0.023953654	0.979906472	0.992856778
04111 Cell cycle - yeast	28	1	53	793	248	2237	0.002872435	0.055055008	0.985785714	0.994432958
04120 Ubiquitin mediated proteolysis	40	3	87	793	248	2237	0.0147146	0.141014917	0.990999054	0.995326561

Supplementary Table 7
Unique domains for *A.cerana* and *Apidae*

Unique domain	PF	Pfam description
unique for Apis cerana	PF03067	Chitin_bind_3, Chitin binding domain. This domain is found associated with a wide variety of cellulose binding domain. This domain however is a chitin binding domain. This domain is found in isolation in baculoviral spheroidins and spindolins, protein of unknown function.
unique for Apis cerana	PF04732	Filament_head, Intermediate filament head (DNA binding) region. This family represents the N-terminal head region of intermediate filaments. Intermediate filament heads bind DNA. Vimentin heads are able to alter nuclear architecture and chromatin distribution, and the liberation of heads by HIV-1 protease liberates may play an important role in HIV-1 associated cytopathogenesis and carcinogenesis. Phosphorylation of the head region can affect filament stability. The head has been shown to interaction with the rod domain of the same protein.
unique for Apis cerana	PF06372	Gemin6, Gemin6 protein. This family consists of several mammalian Gemin6 proteins. The exact function of Gemin6 is unknown but it has been found to form part of the pfam06003 complex. The SMN complex plays a key role in the biogenesis of spliceosomal small nuclear ribonucleoproteins (snRNPs) and other ribonucleoprotein particles.
unique for nonsocial insect	PF00355	Rieske, Rieske [2Fe-2S] domain. The rieske domain has a [2Fe-2S] centre. Two conserved cysteines that one Fe ion while the other Fe ion is coordinated by two conserved histidines.
unique for Apis	PF1372	Melittin, Melittin.
unique for Apis	PF12336	SOXp, SOX transcription factor. This domain family is found in eukaryotes, and is approximately 80 amino acids in length. The family is found in association with pfam00505. There are two conserved sequence motifs: KKDK and LPG. This family is made up of SOX transcription factors. These are involved in upregulation of nestin, a neural promoter.
unique for Apidae	PF00386	C1q, C1q domain. C1q is a subunit of the C1 enzyme complex that activates the serum complement system.
unique for Apidae	PF03801	Ndc80_HEC, HEC/Ndc80p family. Members of this family are components of the mitotic spindle. It has been shown that Ndc80/HEC from yeast is part of a complex called the Ndc80p complex. This complex is thought to bind to the microtubules of the spindle.

unique for Apidae	PF05458	Siva, Cd27 binding protein (Siva). Siva binds to the CD27 cytoplasmic tail. It has a DD homology region, a box-B-like ring finger, and a zinc finger-like domain. Overexpression of Siva in various cell lines induces apoptosis, suggesting an important role for Siva in the CD27-transduced apoptotic pathway. Siva-1 binds to and inhibits BCL-X(L)-mediated protection against UV radiation-induced apoptosis. Indeed, the unique amphipathic helical region (SAH) present in Siva-1 is required for its binding to BCL-X(L) and sensitising cells to UV radiation. Natural complexes of Siva-1/BCL-X(L) are detected in HUT78 and murine thymocyte, suggesting a potential role for Siva-1 in regulating T cell homeostasis. This family contains both Siva-1 and the shorter Siva-2 lacking the sequence coded by exon 2 It has been suggested that Siva-2 could regulate the function of Siva-1.

Supplementary Table 8
Comparison of KEGG pathways among insects

Pathway Class	Represented KOs in social insects	conserved KOs in social insects	Only conserved KOs in social insects	Apis cerana		Apis mellifera		Apis florea		Bombus impatiens		Camponotus floridanus		Harpegnathos saltator		Represented KOs in non-social insects	conserved KOs in non-social insects	Only conserved KOs in non-social insects	Anopheles gambiae		Bombyx mori		Drosophila melanogaster		Nasonia vitripennis		M.rotundata	
				Genes	KOs	Genes	KOs	Genes	KOs	Genes	KOs	Genes	KOs	Genes	KOs				Genes	KOs	Genes	KOs	Genes	KOs	Genes	KOs	Genes	KOs
**Cellular Processes**	**423**	**327**	**0**	**443**	**385**	**439**	**387**	**456**	**374**	**483**	**390**	**446**	**395**	**454**	**399**	**464**	**303**	**0**	**475**	**410**	**486**	**408**	**582**	**413**	**441**	**378**	**476**	**386**
Cell Communication	102	83	0	120	98	120	96	124	91	129	99	114	96	118	97	117	71	0	121	99	133	104	144	97	113	93	132	96
Cell Growth and Death	134	104	0	134	120	130	120	136	113	142	123	130	120	132	125	144	96	0	142	131	145	129	167	127	124	116	133	119
Cell Motility	48	39	0	54	44	50	44	58	46	59	45	50	46	49	44	52	27	0	52	43	48	46	59	45	45	37	54	42
Transport and Catabolism	209	158	0	224	187	227	193	228	187	252	191	229	198	238	200	227	155	0	245	203	244	197	311	211	237	190	248	192
**Environmental Information Processing**	**426**	**305**	**0**	**420**	**355**	**419**	**363**	**433**	**352**	**481**	**379**	**418**	**362**	**434**	**370**	**439**	**254**	**1**	**438**	**363**	**457**	**374**	**507**	**369**	**386**	**339**	**433**	**357**
Membrane Transport	41	9	0	11	11	15	15	13	13	30	29	13	13	22	16	19	5	0	15	13	18	15	12	11	11	11	12	12
Signal Transduction	352	274	0	375	315	372	322	390	313	411	321	369	321	371	323	382	231	1	384	323	391	330	449	331	338	299	385	317
Signaling Molecules and Interaction	50	34	0	51	44	50	41	47	40	56	45	55	45	58	47	60	29	0	63	46	68	45	64	44	58	47	55	44
**Genetic Information Processing**	**915**	**703**	**0**	**894**	**834**	**899**	**833**	**921**	**802**	**1023**	**852**	**892**	**832**	**948**	**857**	**902**	**644**	**0**	**893**	**821**	**862**	**798**	**986**	**845**	**875**	**797**	**920**	**834**
Folding, Sorting and Degradation	321	256	0	313	297	314	295	317	288	348	301	308	294	325	307	319	229	0	305	284	301	279	348	292	304	283	328	298
Replication and Repair	121	78	0	104	100	109	101	101	95	117	107	103	98	104	100	112	65	0	98	91	94	89	97	91	102	92	103	97
Transcription	165	133	0	161	154	161	155	166	150	173	153	160	153	171	157	165	123	0	168	157	159	152	183	162	169	147	166	153
Translation	353	275	0	361	327	359	324	384	311	430	332	366	331	395	338	351	260	0	367	330	348	315	414	343	351	316	369	328
**Metabolism**	**904**	**601**	**0**	**860**	**719**	**886**	**763**	**885**	**718**	**1063**	**801**	**868**	**725**	**872**	**730**	**875**	**575**	**0**	**897**	**740**	**962**	**765**	**1178**	**770**	**824**	**702**	**897**	**723**
Amino Acid Metabolism	179	104	0	143	128	154	142	142	129	200	157	147	128	145	128	160	107	0	147	130	163	144	192	142	140	129	147	129
Biosynthesis of Other Secondary Metabolites	22	12	0	20	16	18	16	19	15	23	17	23	18	20	16	22	12	0	19	16	24	18	31	18	22	17	21	16
Carbohydrate Metabolism	191	139	0	200	154	196	161	203	155	255	172	195	153	197	154	178	129	0	191	147	220	163	288	158	186	147	204	153
Energy Metabolism	167	105	0	148	133	151	136	162	138	194	152	138	127	142	130	167	113	0	169	146	158	139	227	152	147	136	158	132
Glycan Biosynthesis and Metabolism	114	75	0	117	98	122	106	119	95	119	97	118	101	117	102	116	76	0	123	106	117	97	141	105	107	93	120	101
Lipid Metabolism	134	103	0	141	109	148	115	151	114	181	126	155	117	154	114	138	95	0	154	111	176	127	199	112	140	110	155	111
Metabolism of Cofactors and Vitamins	113	66	0	95	81	95	86	98	81	131	99	94	80	106	84	96	63	0	98	83	105	85	141	86	88	75	95	81
Metabolism of Other Amino Acids	60	40	0	58	48	58	49	61	47	80	54	67	51	61	48	54	41	0	72	46	76	50	100	49	59	45	64	49
Metabolism of Terpenoids and Polyketides	42	27	0	34	31	35	32	37	33	44	38	36	32	37	34	39	26	0	37	31	42	34	39	33	32	30	37	32
Nucleotide Metabolism	129	89	0	121	104	130	111	131	105	161	113	125	107	120	103	123	82	0	121	109	136	111	151	110	128	104	127	104
Xenobiotics Biodegradation and Metabolism	44	30	0	48	35	47	35	54	35	65	37	53	35	57	34	47	25	0	55	30	79	42	130	38	53	30	57	33
**Organismal Systems**	**456**	**351**	**0**	**489**	**415**	**492**	**417**	**508**	**406**	**546**	**409**	**507**	**425**	**503**	**426**	**513**	**299**	**0**	**526**	**429**	**537**	**429**	**641**	**441**	**468**	**393**	**525**	**412**
Circulatory System	50	38	0	62	48	63	47	75	45	57	46	60	45	58	44	59	36	0	66	51	65	47	78	51	61	44	67	47
Development	61	43	0	60	52	64	58	59	53	67	54	59	53	60	54	70	33	0	68	57	68	59	75	63	56	49	59	51
Digestive System	79	65	0	88	70	89	71	96	69	97	70	97	75	91	73	87	48	0	98	74	113	76	120	71	85	64	99	70
Endocrine System	150	122	0	177	141	172	139	191	138	193	137	183	145	178	146	169	108	0	191	147	178	139	223	148	171	139	193	138
Environmental Adaptation	54	46	0	60	52	57	51	68	49	70	50	59	51	60	52	57	36	0	65	50	55	48	87	49	54	46	63	49
Excretory System	58	46	0	66	55	65	54	71	52	69	51	68	56	68	56	58	44	0	63	51	66	54	88	52	64	54	80	54
Immune System	130	104	0	144	124	138	121	143	118	160	121	139	124	139	123	144	83	0	144	122	144	125	169	125	127	107	146	119
Nervous System	143	120	0	160	135	155	135	164	131	179	136	157	135	164	140	149	91	0	158	131	150	129	176	128	136	119	177	134
Sensory System	24	21	0	31	23	31	23	41	22	39	22	30	22	30	21	25	18	0	35	24	27	22	38	24	29	21	37	22

Supplementary Table 9. The list of single copy gene used for phylogenetic Analysis

protein	A. cerana	A. mellifera	C. floridanus	H. saltator	A. gambiae	B. mori	D. melanogaster	N. vitripennis	A.florea	B.impatiens	M.rotundata
40S ribosomal protein S15	ACC_00002	GB10353	EFN64700	EFN80699	AgaP_AGAP001274	BGIBMGA001471	Dmel_CG8332	nvi:100113507	XP_003698926	XP_003488107	XP_003703554
Alpha,alpha-trehalose-phosphate synthase [UDP-forming] A	ACC_00264	GB12797	EFN65057	EFN84652	AgaP_AGAP008227	BGIBMGA005182	Dmel_CG4104	nvi:100120006	XP_003689723	XP_003490305	XP_003702415
Ubiquitin-conjugating enzyme E2 variant	ACC_00362	GB18381	EFN67604	EFN88620	AgaP_AGAP006937	BGIBMGA011803	Dmel_CG10640	nvi:100122032	XP_003691588	XP_003488993	XP_003699713
40S ribosomal protein S12	ACC_00367	GB15605	EFN72731	EFN80764	AgaP_AGAP011077	BGIBMGA004374	Dmel_CG11271	nvi:100114141	XP_003689761	XP_003490307	XP_003702416
Dihydropyrimidine dehydrogenase [NADP+]	ACC_00420	GB19753	EFN63986	EFN81057	AgaP_AGAP001021	BGIBMGA007111	Dmel_CG2194	nvi:100121034	XP_003697321	XP_003488363	XP_003707058
UDP-glucose 6-dehydrogenase	ACC_00443	GB15000	EFN63018	EFN75169	AgaP_AGAP006532	BGIBMGA012188	Dmel_CG10072	nvi:100114791	XP_003692490	XP_003485726	XP_003701772
Transcription factor MafK	ACC_00491	GB16096	EFN62061	EFN87250	AgaP_AGAP010405	BGIBMGA000157	Dmel_CG9954	nvi:100115495	XP_003694393	XP_003485153	XP_003708285
Putative glycogen [starch] synthase	ACC_00522	GB12399	EFN70285	EFN85082	AgaP_AGAP002586	BGIBMGA011360	Dmel_CG6904	nvi:100119226	XP_003691183	XP_003493758	XP_003700416
Neurofibromin	ACC_00583	GB12613	EFN68416	EFN88549	AgaP_AGAP003140	BGIBMGA011404	Dmel_CG8318	nvi:100118811	XP_003697188	XP_003485458	XP_003707231
40S ribosomal protein S21	ACC_00602	GB18769	EFN68619	EFN77591	AgaP_AGAP009431	BGIBMGA001363	Dmel_CG2986	nvi:100114107	XP_003698795	XP_003489486	XP_003708178
UPF0027 protein C22orf28 homolog	ACC_00605	GB14373	EFN69663	EFN82990	AgaP_AGAP008147	BGIBMGA006828	Dmel_CG9987	nvi:100123449	XP_003698805	XP_003486446	XP_003706516
Tyrosyl-tRNA synthetase, cytoplasmic	ACC_00744	GB10789	EFN69060	EFN77433	AgaP_AGAP003003	BGIBMGA001525	Dmel_CG4561	nvi:100119473	XP_003694790	XP_003493983	XP_003702906
Probable actin-related protein complex subunit	ACC_00828	GB10826	EFN61769	EFN89508	AgaP_AGAP009154	BGIBMGA008927	Dmel_CG10954	nvi:100116506	XP_003692890	XP_003484406	XP_003706888
Transcription elongation factor SPT4	ACC_00890	GB16091	EFN74205	EFN87657	AgaP_AGAP005428	BGIBMGA005413	Dmel_CG12372	nvi:100120596	XP_003698741	XP_003486507	XP_003706128
Probable nucleolar GTP-binding protein	ACC_00942	GB17562	EFN64128	EFN79973	AgaP_AGAP007050	BGIBMGA003221	Dmel_CG8801	nvi:100117603	XP_003696957	XP_003490060	XP_003701539
60S ribosomal protein L31	ACC_01002	GB18564	EFN65940	EFN80500	AgaP_AGAP011896	BGIBMGA000959	Dmel_CG1821	nvi:100117921	XP_003694064	XP_003491807	XP_003706446
Parafibromin	ACC_01014	GB16148	EFN63113	EFN77978	AgaP_AGAP008885	BGIBMGA007821	Dmel_CG11990	nvi:100118523	XP_003694187	XP_003489299	XP_003705099
Probable deoxyhypusine synthase	ACC_01019	GB18573	EFN73018	EFN80600	AgaP_AGAP006531	BGIBMGA000138	Dmel_CG8005	nvi:100114756	XP_003693918	XP_003494156	XP_003705705
40S ribosomal protein S17	ACC_01241	GB10913	EFN61397	EFN83368	AgaP_AGAP004887	BGIBMGA001483	Dmel_CG3922	nvi:100115357	XP_003690603	XP_003487192	XP_003702574
60S ribosomal protein L17	ACC_01322	GB17564	EFN69274	EFN89579	AgaP_AGAP001459	BGIBMGA012414	Dmel_CG3203	nvi:100118206	XP_003697592	XP_003486076	XP_003700278
Mediator of RNA polymerase II transcription subunit	ACC_01414	GB18959	EFN63605	EFN88135	AgaP_AGAP009141	BGIBMGA005197	Dmel_CG7957	nvi:100115667	XP_003689699	XP_003492267	XP_003699442
Sorting nexin-12	ACC_01447	GB11979	EFN74540	EFN88331	AgaP_AGAP003437	BGIBMGA011317	Dmel_CG6359	nvi:100120195	XP_003698720	XP_003486485	XP_003706075
Membrane-associated protein Hem	ACC_01523	GB13021	EFN65681	EFN75387	AgaP_AGAP004946	BGIBMGA002428	Dmel_CG5837	nvi:100118019	XP_003692077	XP_003486951	XP_003706729
F-actin-capping protein subunit beta	ACC_01566	GB18637	EFN68150	EFN81165	AgaP_AGAP007864	BGIBMGA009430	Dmel_CG17158	nvi:100123576	XP_003689805	XP_003488718	XP_003705273
60S ribosomal protein L23	ACC_01582	GB12633	EFN62772	EFN77642	AgaP_AGAP010252	BGIBMGA009751	Dmel_CG3661	nvi:100118197	XP_003697800	XP_003494934	XP_003702854
Protein BUD31 homolog	ACC_01603	GB11160	EFN74888	EFN83793	AgaP_AGAP002301	BGIBMGA006984	Dmel_CG1639	nvi:100114288	XP_003698345	XP_003490246	XP_003699807
40S ribosomal protein S9	ACC_01644	GB12637	EFN69456	EFN81577	AgaP_AGAP005061	BGIBMGA012626	Dmel_CG3395	nvi:100115465	XP_003697513	XP_003493048	XP_003702864
Transmembrane and coiled-coil domains protein	ACC_01668	GB16413	EFN73770	EFN88102	AgaP_AGAP009081	BGIBMGA006200	Dmel_CG10470	nvi:100119904	XP_003693469	XP_003489083	XP_003706793
Putative glutamate synthase [NADPH]	ACC_01676	GB16028	EFN72550	EFN86827	AgaP_AGAP006360	BGIBMGA012701	Dmel_CG9674	nvi:100122109	XP_003693435	XP_003487603	XP_003708150
Eukaryotic translation initiation factor	ACC_01776	GB11201	EFN70839	EFN78983	AgaP_AGAP006130	BGIBMGA012126	Dmel_CG5642	nvi:100114220	XP_003698167	XP_003485076	XP_003707707
Vacuolar protein sorting-associated protein	ACC_01786	GB16280	EFN70827	EFN76467	AgaP_AGAP009739	BGIBMGA002965	Dmel_CG7371	nvi:100121071	XP_003698188	XP_003485035	XP_003705329
60S ribosomal protein L15	ACC_01801	GB16952	EFN65755	EFN78553	AgaP_AGAP004919	BGIBMGA006414	Dmel_CG17420	nvi:100115408	XP_003690013	XP_003488628	XP_003706183
Prohormone-4	ACC_01898	GB18206	EFN61985	EFN80209	AgaP_AGAP010260	BGIBMGA010065	Dmel_CG31221	nvi:100120249	XP_003691879	XP_003487876	XP_003699280
Kinesin light chain	ACC_02132	GB13592	EFN61596	EFN90059	AgaP_AGAP010258	BGIBMGA008408	Dmel_CG5433	nvi:100116045	XP_003690335	XP_003484440	XP_003700120
DNA-directed RNA polymerase II subunit RPB11	ACC_02176	GB11510	EFN73365	EFN84774	AgaP_AGAP009209	BGIBMGA006752	Dmel_CG6840	nvi:100114414	XP_003698760	XP_003489439	XP_003705797
Transaldolase	ACC_02184	GB11283	EFN67761	EFN90010	AgaP_AGAP011800	BGIBMGA000926	Dmel_CG2827	nvi:100115508	XP_003690749	XP_003490341	XP_003706459
60S ribosomal protein L21	ACC_02268	GB16182	EFN68322	EFN85491	AgaP_AGAP012138	BGIBMGA001106	Dmel_CG12775	nvi:100115144	XP_003695520	XP_003489932	XP_003703759

Uncharacterized protein C594.04c	ACC_02306	GB14389	EFN65575	EFN83194	AgaP_AGAP005737	BGIBMGA013819	Dmel_CG6282	nvi:100123185	XP_003698844	XP_003485350	XP_003705012
Uncharacterized protein C45G9.7	ACC_02308	GB17020	EFN74782	EFN83959	AgaP_AGAP005623	BGIBMGA003632	Dmel_CG3402	nvi:100122504	XP_003698849	XP_003485348	XP_003705013
SWI/SNF-related matrix-associated actin-dependent regulator of chromatin subfamily B member	ACC_02375	GB19041	EFN68863	EFN84532	AgaP_AGAP001154	BGIBMGA013255	Dmel_CG1064	nvi:100118240	XP_003691366	XP_003490780	XP_003701943
60S ribosomal protein L18	ACC_02395	GB11029	EFN68908	EFN87206	AgaP_AGAP007580	BGIBMGA011620	Dmel_CG8615	nvi:100120168	XP_003691391	XP_003485273	XP_003707499
6-phosphofructokinase	ACC_02409	GB17113	EFN72043	EFN85262	AgaP_AGAP007642	BGIBMGA003717	Dmel_CG4001	nvi:100115310	XP_003692805	XP_003493467	XP_003706922
Probable eukaryotic translation initiation factor	ACC_02568	GB11906	EFN64229	EFN81318	AgaP_AGAP001380	BGIBMGA009830	Dmel_CG17611	nvi:100115084	XP_003694943	XP_003492156	XP_003707998
Cleavage and polyadenylation specificity factor subunit	ACC_02585	GB18630	EFN67838	EFN76452	AgaP_AGAP007242	BGIBMGA001806	Dmel_CG3689	nvi:100116257	XP_003693499	XP_003484601	XP_003702048
Lipoyl synthase, mitochondrial	ACC_02587	GB16729	EFN68320	EFN85489	AgaP_AGAP010568	BGIBMGA001093	Dmel_CG5231	nvi:100121854	XP_003695502	XP_003489933	XP_003703828
Probable 26S proteasome non-ATPase regulatory subunit	ACC_02601	GB12827	EFN67747	EFN82224	AgaP_AGAP009082	BGIBMGA002953	Dmel_CG42641	nvi:100114055	XP_003689802	XP_003488721	XP_003705277
Translocon-associated protein subunit gamma	ACC_02633	GB16049	EFN72903	EFN76870	AgaP_AGAP008761	BGIBMGA005328	Dmel_CG5885	nvi:100120928	XP_003691811	XP_003490421	XP_003705748
Clathrin heavy chain	ACC_02691	GB19359	EFN61414	EFN80585	AgaP_AGAP003021	BGIBMGA012935	Dmel_CG9012	nvi:100114717	XP_003690808	XP_003486702	XP_003706348
U6 snRNA-associated Sm-like protein LSm5	ACC_02717	GB18491	EFN66930	EFN78665	AgaP_AGAP006604	BGIBMGA007068	Dmel_CG6610	nvi:100117891	XP_003693769	XP_003484674	XP_003708516
60S ribosomal protein L12	ACC_02800	GB19465	EFN72686	EFN87561	AgaP_AGAP010065	BGIBMGA003978	Dmel_CG3195	nvi:100117287	XP_003695708	XP_003489012	XP_003706877
Ribonucleoside-diphosphate reductase subunit M2	ACC_02981	GB13424	EFN63309	EFN75622	AgaP_AGAP006818	BGIBMGA013510	Dmel_CG8975	nvi:100114323	XP_003695935	XP_003493620	XP_003706010
U2 small nuclear ribonucleoprotein B''	ACC_02996	GB15338	EFN74543	EFN77329	AgaP_AGAP011637	BGIBMGA012118	Dmel_CG4528	nvi:100122364	XP_003695965	XP_003493658	XP_003699629
40S ribosomal protein S8	ACC_03026	GB13741	EFN66603	EFN82821	AgaP_AGAP002437	BGIBMGA003397	Dmel_CG7808	nvi:100117715	XP_003690352	XP_003485784	XP_003707560
Protein transport protein Sec23A	ACC_03034	GB19531	EFN69453	EFN78937	AgaP_AGAP001759	BGIBMGA004852	Dmel_CG1250	nvi:100114786	XP_003690328	XP_003484429	XP_003707641
Coatomer subunit zeta-1	ACC_03047	GB16284	EFN60230	EFN77651	AgaP_AGAP006522	BGIBMGA009760	Dmel_CG3948	nvi:100115298	XP_003697516	XP_003493043	XP_003702859
Protein phosphatase PP2A	ACC_03152	GB18138	EFN72063	EFN87073	AgaP_AGAP004502	BGIBMGA010831	Dmel_CG6235	nvi:100124058	XP_003696131	XP_003490537	XP_003704760
40S ribosomal protein S3	ACC_03170	GB15637	EFN61127	EFN84837	AgaP_AGAP001910	BGIBMGA009319	Dmel_CG6779	nvi:100114225	XP_003696828	XP_003494719	XP_003701515
60S ribosomal protein L27	ACC_03234	GB15660	EFN65525	EFN86316	AgaP_AGAP011687	BGIBMGA009863	Dmel_CG4759	nvi:100114558	XP_003691054	XP_003491548	XP_003704014
BTB/POZ domain-containing protein KCTD3	ACC_03237	GB18915	EFN65918	EFN82811	AgaP_AGAP004087	BGIBMGA005002	Dmel_CG9467	nvi:100115552	XP_003696745	XP_003491457	XP_003702636
Hydroxymethylglutaryl-CoA synthase	ACC_03245	GB12497	EFN62647	EFN75750	AgaP_AGAP005847	BGIBMGA004001	Dmel_CG4311	nvi:100116401	XP_003696720	XP_003489261	XP_003702686
60S ribosomal protein L3	ACC_03344	GB15261	EFN62081	EFN83156	AgaP_AGAP003556	BGIBMGA013567	Dmel_CG4863	nvi:100120962	XP_003690092	XP_003493892	XP_003699836
33 kDa inner dynein arm light chain, axonemal	ACC_03425	GB13866	EFN68663	EFN83011	AgaP_AGAP004030	BGIBMGA006363	Dmel_CG6971	nvi:100119377	XP_003691737	XP_003491247	XP_003703946
60S ribosomal protein L32	ACC_03501	GB10903	EFN68969	EFN80090	AgaP_AGAP002122	BGIBMGA000066	Dmel_CG7939	nvi:100115795	XP_003698446	XP_003485519	XP_003702457
26S proteasome non-ATPase regulatory subunit	ACC_03601	GB10959	EFN63481	EFN80757	AgaP_AGAP001745	BGIBMGA008425	Dmel_CG11888	nvi:100114985	XP_003698288	XP_003488307	XP_003706695
General transcription factor IIH subunit	ACC_03639	GB10106	EFN66420	EFN87943	AgaP_AGAP010984	BGIBMGA012623	Dmel_CG7764	nvi:100118816	XP_003691121	XP_003490125	XP_003701820
Ubiquitin-fold modifier-conjugating enzyme	ACC_03746	GB16801	EFN69605	EFN77979	AgaP_AGAP008099	BGIBMGA011117	Dmel_CG8386	nvi:100116885	XP_003697897	XP_003489542	XP_003705375
Transmembrane protein	ACC_03751	GB15423	EFN66974	EFN87320	AgaP_AGAP007885	BGIBMGA006981	Dmel_CG6750	nvi:100121821	XP_003697847	XP_003489523	XP_003705355
Protein pelota	ACC_03870	GB10750	EFN69659	EFN82985	AgaP_AGAP008269	BGIBMGA006754	Dmel_CG3959	nvi:100113863	XP_003698803	XP_003489492	XP_003708181
Protein FAM50 homolog	ACC_03873	GB17262	EFN61011	EFN87424	AgaP_AGAP002062	BGIBMGA000865	Dmel_CG12259	nvi:100116858	XP_003696765	XP_003491379	XP_003705210
L-threonine 3-dehydrogenase, mitochondrial	ACC_03909	GB18964	EFN69849	EFN75542	AgaP_AGAP011948	BGIBMGA006160	Dmel_CG5955	nvi:100122557	XP_003690874	XP_003494460	XP_003704319
Transmembrane protein	ACC_03929	GB10104	EFN67172	EFN77594	AgaP_AGAP009703	BGIBMGA006224	Dmel_CG10723	nvi:100120491	XP_003697709	XP_003488820	XP_003704827
ATPase ASNA1 homolog	ACC_03941	GB18636	EFN66662	EFN83600	AgaP_AGAP005782	BGIBMGA001943	Dmel_CG1598	nvi:100123275	XP_003693290	XP_003488032	XP_003703703
GMP synthase [glutamine-hydrolyzing]	ACC_03991	GB10491	EFN73755	EFN88703	AgaP_AGAP010139	BGIBMGA007436	Dmel_CG9242	nvi:100120653	XP_003695077	XP_003494403	XP_003704219
Importin subunit beta-1	ACC_04024	GB16423	EFN73181	EFN81834	AgaP_AGAP009921	BGIBMGA002972	Dmel_CG2637	nvi:100114333	XP_003692699	XP_003494863	XP_003704389

Deoxyuridine 5'-triphosphate nucleotidohydrolase, mitochondrial	ACC_04076	GB19868	EFN62181	EFN80235	AgaP_AGAP010159	BGIBMGA010154	Dmel_CG4584	nvi:100120364	XP_003698958	XP_003494435	XP_003704191
Glycogen phosphorylase	ACC_04087	GB11892	EFN73448	EFN79902	AgaP_AGAP007939	BGIBMGA010361	Dmel_CG7254	nvi:100117812	XP_003690485	XP_003488962	XP_003707637
Cyclin-Y	ACC_04133	GB17196	EFN73682	EFN79755	AgaP_AGAP008969	BGIBMGA005969	Dmel_CG14939	nvi:100121471	XP_003693365	XP_003492844	XP_003706449
PHD finger-like domain-containing protein	ACC_04144	GB18336	EFN64088	EFN89957	AgaP_AGAP009905	BGIBMGA009855	Dmel_CG9548	nvi:100114630	XP_003694734	XP_003484388	XP_003701008
60S ribosomal protein L38	ACC_04266	GB14397	EFN61045	EFN79053	AgaP_AGAP010163	BGIBMGA005684	Dmel_CG18001	nvi:100118041	XP_003697582	XP_003487129	XP_003705671
Vacuolar protein sorting-associated protein	ACC_04402	GB14744	EFN69227	EFN75577	AgaP_AGAP006139	BGIBMGA000120	Dmel_CG14804	nvi:100121352	XP_003693683	XP_003487056	XP_003705643
Intron-binding protein aquarius	ACC_04443	GB19403	EFN66172	EFN87169	AgaP_AGAP004028	BGIBMGA004963	Dmel_CG31368	nvi:100116368	XP_003696210	XP_003493110	XP_003705196
Short coiled-coil protein A	ACC_04483	GB17865	EFN70369	EFN88403	AgaP_AGAP001277	BGIBMGA010197	Dmel_CG5934	nvi:100123511	XP_003694958	XP_003493166	XP_003706671
40S ribosomal protein S20	ACC_04541	GB17488	EFN60921	EFN89834	AgaP_AGAP010591	BGIBMGA009130	Dmel_CG15693	nvi:100115087	XP_003691551	XP_003490947	XP_003707482
Serine hydroxymethyltransferase	ACC_04662	GB14485	EFN60414	EFN84412	AgaP_AGAP001065	BGIBMGA007079	Dmel_CG3011	nvi:100117303	XP_003698113	XP_003485067	XP_003708127
Peptidylprolyl isomerase domain and WD repeat-containing protein	ACC_04690	GB10843	EFN71628	EFN85461	AgaP_AGAP008660	BGIBMGA004163	Dmel_CG3511	nvi:100122629	XP_003691649	XP_003488313	XP_003701040
Negative elongation factor D	ACC_04742	GB16924	EFN70979	EFN85695	AgaP_AGAP000391	BGIBMGA003465	Dmel_CG9984	nvi:100170020	XP_003689608	XP_003492420	XP_003708374
Eukaryotic translation initiation factor	ACC_04753	GB13540	EFN71096	EFN78082	AgaP_AGAP003119	BGIBMGA006840	Dmel_CG8053	nvi:100117365	XP_003694597	XP_003492370	XP_003703907
Programmed cell death protein	ACC_04793	GB15770	EFN73667	EFN82915	AgaP_AGAP008287	BGIBMGA006228	Dmel_CG5073	nvi:100118333	XP_003698728	XP_003486488	XP_003704038
Surfeit locus protein	ACC_04867	GB14656	EFN69719	EFN79536	AgaP_AGAP001069	BGIBMGA007639	Dmel_CG6202	nvi:100121984	XP_003698807	XP_003489461	XP_003708170
60S ribosomal protein L26	ACC_04896	GB13731	EFN69610	EFN77088	AgaP_AGAP002468	BGIBMGA010970	Dmel_CG6846	nvi:100119535	XP_003692076	XP_003486898	XP_003705919
Inositol-3-phosphate synthase 1-B	ACC_04905	GB11572	EFN71722	EFN79046	AgaP_AGAP011830	BGIBMGA007441	Dmel_CG11143	nvi:100120488	XP_003692777	XP_003484397	XP_003706882
Eukaryotic translation initiation factor	ACC_04952	GB18770	EFN68149	EFN81163	AgaP_AGAP011284	BGIBMGA007469	Dmel_CG3186	nvi:100115070	XP_003689841	XP_003488724	XP_003705274
Anaphase-promoting complex subunit	ACC_05008	GB16034	EFN74840	EFN77387	AgaP_AGAP011799	BGIBMGA000948	Dmel_CG11419	nvi:100116985	XP_003695365	XP_003489394	XP_003705068
Brefeldin A-inhibited guanine nucleotide-exchange protein	ACC_05038	GB12468	EFN72227	EFN81580	AgaP_AGAP008906	BGIBMGA004647	Dmel_CG7578	nvi:100122365	XP_003692583	XP_003489352	XP_003702494
ATP-binding cassette sub-family E member	ACC_05050	GB11485	EFN63715	EFN87340	AgaP_AGAP002182	BGIBMGA010129	Dmel_CG5651	nvi:100124231	XP_003690922	XP_003487517	XP_003701716
60S ribosomal protein L30	ACC_05060	GB12284	EFN63895	EFN82382	AgaP_AGAP003816	BGIBMGA007795	Dmel_CG10652	nvi:100119002	XP_003694261	XP_003491148	XP_003704598
1,4-alpha-glucan-branching enzyme	ACC_05143	GB12103	EFN68446	EFN78864	AgaP_AGAP010428	BGIBMGA007451	Dmel_CG33138	nvi:100118463	XP_003697146	XP_003488932	XP_003707245
60S ribosomal protein L44	ACC_05150	GB15143	EFN68839	EFN89049	AgaP_AGAP003538	BGIBMGA010487	Dmel_CG7424	nvi:100118856	XP_003694635	XP_003486659	XP_003708411
RNA polymerase II subunit	ACC_05216	GB18768	EFN64495	EFN76114	AgaP_AGAP011470	BGIBMGA000925	Dmel_CG14216	nvi:100119231	XP_003691566	XP_003489976	XP_003703439
Soluble NSF attachment protein	ACC_05292	GB15709	EFN72978	EFN79607	AgaP_AGAP003192	BGIBMGA010068	Dmel_CG6625	nvi:100115630	XP_003691911	XP_003487834	XP_003700564
DNA excision repair protein haywire	ACC_05332	GB16621	EFN62432	EFN80971	AgaP_AGAP012169	BGIBMGA011797	Dmel_CG8019	nvi:100117640	XP_003691832	XP_003489748	XP_003700549
Repressor of RNA polymerase III transcription MAF1 homolog	ACC_05363	GB10178	EFN71953	EFN84453	AgaP_AGAP008411	BGIBMGA003005	Dmel_CG40196	nvi:100115733	XP_003691900	XP_003488216	XP_003707792
DNA-directed RNA polymerase III subunit RPC8	ACC_05392	GB17735	EFN71480	EFN81651	AgaP_AGAP012122	BGIBMGA011753	Dmel_CG7339	nvi:100121620	XP_003693065	XP_003484613	XP_003702067
40S ribosomal protein S11	ACC_05454	GB15755	EFN72597	EFN80564	AgaP_AGAP009998	BGIBMGA013792	Dmel_CG8857	nvi:100113518	XP_003696299	XP_003491397	XP_003700346
UPF0183 protein CG7083	ACC_05663	GB12786	EFN66273	EFN79698	AgaP_AGAP003820	BGIBMGA004348	Dmel_CG7083	nvi:100115487	XP_003698750	XP_003486521	XP_003705019
Glucosamine-6-phosphate isomerase	ACC_05672	GB12628	EFN66295	EFN78711	AgaP_AGAP009305	BGIBMGA009467	Dmel_CG6957	nvi:100115436	XP_003690742	XP_003490383	XP_003706495
H(+)/Cl(-) exchange transporter	ACC_05710	GB14095	EFN70548	EFN79810	AgaP_AGAP005777	BGIBMGA003862	Dmel_CG5284	nvi:100120186	XP_003697653	XP_003493337	XP_003704513
WD repeat-containing protein	ACC_05769	GB11315	EFN70366	EFN88399	AgaP_AGAP008003	BGIBMGA009657	Dmel_CG17766	nvi:100118200	XP_003695525	XP_003489813	XP_003706378
UPF0663 transmembrane protein C17orf28 homolog	ACC_05807	GB16139	EFN72818	EFN77341	AgaP_AGAP011570	BGIBMGA009898	Dmel_CG8841	nvi:100117947	XP_003698858	XP_003485368	XP_003706063

Inositol 1,4,5-trisphosphate receptor	ACC_05838	GB11927	EFN73871	EFN76414	AgaP_AGAP006475	BGIBMGA013392	Dmel_CG1063	nvi:100123596	XP_003691335	XP_003485954	XP_003706771
NADH dehydrogenase [ubiquinone] iron-sulfur protein mitochondrial	ACC_05901	GB17476	EFN69628	EFN77575	AgaP_AGAP001711	BGIBMGA003924	Dmel_CG3944	nvi:100123160	XP_003692105	XP_003487022	XP_003701368
APAF1-interacting protein homolog	ACC_06007	GB11537	EFN63871	EFN76715	AgaP_AGAP000470	BGIBMGA011049	Dmel_CG11134	nvi:100169980	XP_003689792	XP_003494877	XP_003706680
Protein suppressor of forked	ACC_06034	GB18168	EFN62227	EFN79573	AgaP_AGAP003019	BGIBMGA011133	Dmel_CG17170	nvi:100115839	XP_003697399	XP_003494077	XP_003701280
Polymerase delta-interacting protein	ACC_06188	GB10300	EFN64395	EFN89414	AgaP_AGAP001153	BGIBMGA013121	Dmel_CG12162	nvi:100117524	XP_003690565	XP_003485599	XP_003700238
Hexuronate transporter	ACC_06189	GB12819	EFN64402	EFN89407	AgaP_AGAP001336	BGIBMGA013242	Dmel_CG14439	nvi:100118311	XP_003690554	XP_003485595	XP_003700242
Alpha-aminoadipic semialdehyde synthase, mitochondrial	ACC_06254	GB12781	EFN69520	EFN79642	AgaP_AGAP008632	BGIBMGA010338	Dmel_CG7144	nvi:100123521	XP_003692916	XP_003487153	XP_003705284
1,2-dihydroxy-3-keto-5-methylthiopentene dioxygenase	ACC_06427	GB19130	EFN63310	EFN89384	AgaP_AGAP005618	BGIBMGA005810	Dmel_CG32068	nvi:100122447	XP_003695805	XP_003493626	XP_003705948
Adenylosuccinate lyase	ACC_06619	GB14254	EFN63519	EFN85616	AgaP_AGAP002378	BGIBMGA007119	Dmel_CG3590	nvi:100124133	XP_003695043	XP_003491171	XP_003705472
General transcription factor IIE subunit	ACC_06717	GB17516	EFN66966	EFN87330	AgaP_AGAP006355	BGIBMGA000355	Dmel_CG10415	nvi:100123640	XP_003697324	XP_003488350	XP_003699188
Inosine triphosphate pyrophosphatase	ACC_06776	GB17688	EFN69846	EFN75538	AgaP_AGAP008374	BGIBMGA006159	Dmel_CG8891	nvi:100114157	XP_003690863	XP_003494501	XP_003704176
Delta-1-pyrroline-5-carboxylate synthetase	ACC_06838	GB15049	EFN61839	EFN76788	AgaP_AGAP002518	BGIBMGA011064	Dmel_CG7470	nvi:100120123	XP_003697979	XP_003489845	XP_003703727
rRNA-processing protein FCF1 homolog	ACC_06848	GB12622	EFN74433	EFN76850	AgaP_AGAP009504	BGIBMGA010318	Dmel_CG4539	nvi:100118730	XP_003698031	XP_003492025	XP_003708010
Chloride intracellular channel exc-4	ACC_06875	GB17906	EFN62862	EFN82179	AgaP_AGAP000943	BGIBMGA009045	Dmel_CG10997	nvi:100116483	XP_003694770	XP_003488548	XP_003702780
Protein NipSnap	ACC_06909	GB16972	EFN65056	EFN85510	AgaP_AGAP003200	BGIBMGA012859	Dmel_CG9212	nvi:100114375	XP_003692276	XP_003490961	XP_003707196
Beta carbonic anhydrase	ACC_06911	GB19829	EFN64927	EFN90033	AgaP_AGAP002992	BGIBMGA003357	Dmel_CG11967	nvi:100118017	XP_003692734	XP_003488528	XP_003699501
Protein mago nashi homolog	ACC_06935	GB10361	EFN74207	EFN83218	AgaP_AGAP010755	BGIBMGA003398	Dmel_CG9401	nvi:100120567	XP_003698742	XP_003486509	XP_003706130
Probable small nuclear ribonucleoprotein Sm D2	ACC_07043	GB10555	EFN66506	EFN76821	AgaP_AGAP003936	BGIBMGA010723	Dmel_CG1249	nvi:100118225	XP_003697276	XP_003489020	XP_003706428
40S ribosomal protein S24	ACC_07172	GB19487	EFN71100	EFN78087	AgaP_AGAP005131	BGIBMGA003923	Dmel_CG3751	nvi:100122507	XP_003694598	XP_003494214	XP_003699211
Cullin-associated NEDD8-dissociated protein	ACC_07411	GB11107	EFN67101	EFN80451	AgaP_AGAP009970	BGIBMGA002649	Dmel_CG5366	nvi:100114294	XP_003695932	XP_003493628	XP_003706013
Dolichyl-diphosphooligosaccharide--protein glycosyltransferase subunit DAD1	ACC_07444	GB18984	EFN64462	EFN82391	AgaP_AGAP008491	BGIBMGA000188	Dmel_CG13393	nvi:100115214	XP_003695753	XP_003492909	XP_003707533
Eukaryotic peptide chain release factor subunit	ACC_07453	GB20018	EFN71190	EFN81945	AgaP_AGAP010310	BGIBMGA014398	Dmel_CG5605	nvi:100118208	XP_003693023	XP_003488503	XP_003702070
Endothelial differentiation-related factor	ACC_07459	GB16042	EFN62723	EFN89227	AgaP_AGAP004990	BGIBMGA007702	Dmel_CG4143	nvi:100116799	XP_003693044	XP_003484733	XP_003702055
Histone chaperone asf1	ACC_07478	GB10442	EFN66107	EFN78698	AgaP_AGAP005920	BGIBMGA001637	Dmel_CG9383	nvi:100123569	XP_003693589	XP_003491727	XP_003706603
39S ribosomal protein L15, mitochondrial	ACC_07533	GB14192	EFN73054	EFN83714	AgaP_AGAP011946	BGIBMGA002975	Dmel_CG5219	nvi:100122526	XP_003692427	XP_003485656	XP_003700760
Set1/Ash2 histone methyltransferase complex subunit ASH2	ACC_07646	GB15983	EFN69077	EFN84212	AgaP_AGAP003705	BGIBMGA008025	Dmel_CG6677	nvi:100122254	XP_003697600	XP_003486312	XP_003703199
Electron transfer flavoprotein subunit beta	ACC_07745	GB16132	EFN66681	EFN88718	AgaP_AGAP004653	BGIBMGA004824	Dmel_CG7834	nvi:100114751	XP_003698462	XP_003485538	XP_003699376
Triosephosphate isomerase	ACC_07757	GB17473	EFN66403	EFN79773	AgaP_AGAP001630	BGIBMGA000559	Dmel_CG2171	nvi:100114064	XP_003691157	XP_003491978	XP_003700534
Retinal rod rhodopsin-sensitive cGMP	ACC_07828	GB11312	EFN65728	EFN77486	AgaP_AGAP011756	BGIBMGA000879	Dmel_CG9296	nvi:100114143	XP_003695003	XP_003484957	XP_003702792
B-cell CLL/lymphoma B	ACC_07913	GB17186	EFN67122	EFN85972	AgaP_AGAP000566	BGIBMGA002884	Dmel_CG17252	nvi:100119536	XP_003690697	XP_003485383	XP_003702516
Eukaryotic translation initiation factor	ACC_08019	GB19077	EFN70768	EFN88651	AgaP_AGAP006944	BGIBMGA003669	Dmel_CG9677	nvi:100115641	XP_003695661	XP_003492501	XP_003706056

Electron transfer flavoprotein-ubiquinone oxidoreductase, mitochondrial	ACC_08052	GB19888	EFN71831	EFN78847	AgaP_AGAP005894	BGIBMGA009420	Dmel_CG12140	nvi:100119918	XP_003697189	XP_003486591	XP_003707243
Eukaryotic translation initiation factor	ACC_08268	GB13583	EFN70162	EFN77223	AgaP_AGAP012281	BGIBMGA000903	Dmel_CG8309	nvi:100114669	XP_003698098	XP_003491277	XP_003701750
Calcium-activated potassium channel slowpoke	ACC_08498	GB17365	EFN73397	EFN86120	AgaP_AGAP003709	BGIBMGA002400	Dmel_CG10693	nvi:100122182	XP_003697598	XP_003486304	XP_003703218
Dolichyl-diphosphooligosaccharide--protein glycosyltransferase	ACC_08539	GB18042	EFN68961	EFN80101	AgaP_AGAP006383	BGIBMGA002814	Dmel_CG9022	nvi:100115033	XP_003694146	XP_003486393	XP_003701131
Glutamate--cysteine ligase catalytic subunit	ACC_08760	GB10922	EFN64509	EFN80028	AgaP_AGAP001512	BGIBMGA007582	Dmel_CG2259	nvi:100121797	XP_003695679	XP_003488364	XP_003706896
60S ribosomal protein L35	ACC_08802	GB15361	EFN70961	EFN85326	AgaP_AGAP001408	BGIBMGA005928	Dmel_CG4111	nvi:100121722	XP_003692590	XP_003489359	XP_003702500
Transient receptor potential cation channel subfamily V member	ACC_08844	GB19362	EFN65752	EFN81068	AgaP_AGAP012241	BGIBMGA003369	Dmel_CG5842	nvi:100117331	XP_003690006	XP_003488629	XP_003706172
Fibropellin-1	ACC_08871	GB20122	EFN68216	EFN86689	AgaP_AGAP002739	BGIBMGA012488	Dmel_CG9138	nvi:100122612	XP_003692306	XP_003489961	XP_003703612
Echinoderm microtubule-associated protein-like CG42247	ACC_08904	GB14446	EFN69469	EFN79049	AgaP_AGAP000502	BGIBMGA002785	Dmel_CG42247	nvi:100122165	XP_003691996	XP_003490463	XP_003705730
Glycyl-tRNA synthetase	ACC_08962	GB13467	EFN67695	EFN89098	AgaP_AGAP008604	BGIBMGA007637	Dmel_CG6778	nvi:100123216	XP_003697406	XP_003494106	XP_003700775
Bifunctional purine biosynthesis protein PURH	ACC_09332	GB14677	EFN72513	EFN82228	AgaP_AGAP001423	BGIBMGA002462	Dmel_CG11089	nvi:100116524	XP_003697710	XP_003486251	XP_003704896
TBC1 domain family member	ACC_09512	GB12914	EFN63823	EFN82365	AgaP_AGAP010235	BGIBMGA006128	Dmel_CG9339	nvi:100120644	XP_003690887	XP_003486135	XP_003707720
Deoxyhypusine hydroxylase	ACC_09591	GB17967	EFN68288	EFN82005	AgaP_AGAP002129	BGIBMGA005590	Dmel_CG2245	nvi:100124181	XP_003690823	XP_003494221	XP_003707183
4-hydroxyphenylpyruvate dioxygenase	ACC_09738	GB18360	EFN68260	EFN84336	AgaP_AGAP004802	BGIBMGA005262	Dmel_CG11796	nvi:100116430	XP_003691052	XP_003491549	XP_003703989
Uncharacterized protein CG42248	ACC_09898	GB13445	EFN66764	EFN79995	AgaP_AGAP001935	BGIBMGA008378	Dmel_CG42248	nvi:100123626	XP_003698513	XP_003485980	XP_003706840

Supplementary Table 10. Genes under positiveselection after divergence between A.cerana and A.mellifera

***A.cerana* Gene**	***A.mellifera* Gene**	**Ka**	**Ks**	**Ka/Ks**	**P-Value(Fisher)**	**Product**	**KOG**	**KOG Definition**	**KOG Class**
ACC_00073	GB15474	0.0148423	0.010628	1.39653	0.862711	N-acetylated-alpha-linked acidic	KOG2195	Transferrin receptor and	Posttranslational modification,
ACC_00839	GB14778	0.0517369	0.0331044	1.56284	0.74426	Probable 28S ribosomal protein	KOG4079	Putative mitochondrial	Translation, ribosomal structure
ACC_01259	GB12313	1.01608	0.953883	1.06521	0.108856	Zinc finger protein	KOG3598	Thyroid hormone receptor-associated	Transcription
ACC_01705	GB15268	0.0565205	0.042313	1.33577	0.620328	voltage-dependent T-type calcium	KOG2302	T-type voltage-gated Ca2+	Inorganic ion transport and
ACC_01882	GB18344	0.00300079	6.00E-05	50	0.367879	Proline-rich nuclear receptor			
ACC_02180	GB18076	0.0201444	0.0165949	1.21389	0.809982	RNA-binding protein	KOG0109	RNA-binding protein LARK,	RNA processing and modification,
ACC_02589	GB13471	0.0197588	0.019593	1.00846	0.784277	Fas-binding factor	KOG0161	Myosin class II heavy chain	Cytoskeleton
ACC_03845	GB13078	0.0328667	0.0265472	1.23805	0.73522	Mitochondrial import receptor	KOG4111	Translocase of outer mitochondrial	Intracellular trafficking,
ACC_04879	GB16037	0.0128155	0.00509156	2.51701	0.252084	conserved hypothetical			
ACC_05913	GB18652	0.0347775	0.0269344	1.29119	0.461614	conserved hypothetical			
ACC_07212	GB20010	0.373919	0.333237	1.12208	0.451378	conserved hypothetical			
ACC_07942	GB18386	0.024232	0.00550158	4.40456	0.0671128	SAP30-binding protein	KOG2959	Transcriptional regulator	Transcription
ACC_08178	GB12718	0.0492702	0.0473662	1.0402	0.998623	conserved hypothetical			
ACC_09413	GB16626	0.200323	0.159098	1.25912	0.372712	ribonuclease P protein			
ACC_09730	GB19326	0.123118	0.122666	1.00368	1	Methyltransferase-like protein	KOG3987	Uncharacterized conserved protein	Function unknown
ACC_09783	GB10044	0.187948	0.182826	1.02802	0.877539	G-protein coupled receptor Mth2	KOG4193	G protein-coupled receptors	Signal transduction mechanisms
ACC_09847	GB11199	0.0667712	0.0630873	1.05839	0.705669	putative leucine-rich repeat-	KOG0161	Myosin class II heavy chain	Cytoskeleton
ACC_09917	GB14589	0.0168171	0.0162958	1.03199	0.937007	Putative defense protein			
ACC_10170	GB13587	0.122903	0.116278	1.05697	0.85462	odorant binding protein ASP4			

Table 11. ***A.cerana*** **unique genes relative to A.mellifera**

					GO		
**Gene**	**Annotation**	**Best Hit Organism**	**KO**	**KOG**	**Cellular Component**	**Molecular Function**	**Biological Process**
ACC_06721	5'-AMP-activated protein kinase subunit	Acromyrmex echinatior					
ACC_04608	Predicted ubiquitin-protein ligase/hyperplastic discs	Acromyrmex echinatior		KOG0943			
ACC_00082	conserved hypothetical protein	Acromyrmex echinatior					
ACC_05365	conserved hypothetical protein	Acromyrmex echinatior					
ACC_07725	conserved hypothetical protein	Acromyrmex echinatior					
ACC_07571	conserved hypothetical protein	Acromyrmex echinatior					
ACC_06528	ras-related protein Rab-8A-like isoform 2	Acromyrmex echinatior					
ACC_07945	testican-2-like	Acromyrmex echinatior			GO:0005737:cytoplasm	GO:0008603:cAMP-dependent protein kinase regulator activity	GO:0007338:single fertilization
ACC_00706	WD repeat-containing protein	Acromyrmex echinatior		KOG1587			
ACC_08229	conserved hypothetical protein	Acyrthosiphon pisum					
ACC_07323	methyltransferase-like protein 9-like	Andrena erigeniae					
ACC_06836	Cytochrome b	Anolis sabanus		KOG4663	GO:0016020:membrane		GO:0022904:respiratory electron transport
ACC_05063	ankyrin repeat domain-containing protein 39-like isoform 3	Anopheles gambiae str. PEST					
ACC_09189	conserved hypothetical protein	Apis cerana					
ACC_07632	Icarapin-like	Apis cerana			GO:0005576:extracellular region		
ACC_07042	NADH-ubiquinone oxidoreductase chain	Apis cerana		KOG4845			
ACC_09100	NADH-ubiquinone oxidoreductase chain	Apis cerana			GO:0016021:integral to membrane	GO:0008137:NADH dehydrogenase (ubiquinone) activity	GO:0042773:ATP synthesis coupled electron transport

ACC_08805	NADH-ubiquinone oxidoreductase chain	Apis cerana			GO:0044464:cell part		
ACC_08766	70 kDa peptidyl-prolyl isomerase	Bombus impatiens		KOG0543			
ACC_07609	band 4.1-like protein 4A-like	Bombus impatiens					
ACC_06502	calcium channel flower-like isoform 1	Bombus impatiens					
ACC_09290	DnaJ homolog subfamily B member	Bombus impatiens		KOG0715	GO:0005783:endoplasmic reticulum	GO:0031072:heat shock protein binding	GO:0030433:ER-associated protein
ACC_04990	Histone deacetylase complex subunit SAP18	Bombus impatiens	K14324	KOG3391			
ACC_06779	histone-lysine N-methyltransferase SETMAR-like	Bombus impatiens					
ACC_07278	conserved hypothetical protein	Bombus impatiens					
ACC_04728	conserved hypothetical protein	Bombus impatiens					
ACC_03868	conserved hypothetical protein	Bombus impatiens					
ACC_04782	conserved hypothetical protein	Bombus impatiens			GO:0016020:membrane	GO:0004984:olfactory receptor activity	GO:0007608:sensory perception of smell
ACC_01497	conserved hypothetical protein	Bombus impatiens					
ACC_01498	conserved hypothetical protein	Bombus impatiens					
ACC_06104	conserved hypothetical protein	Bombus impatiens				GO:0005549:odorant binding	
ACC_07456	conserved hypothetical protein	Bombus impatiens					
ACC_03009	conserved hypothetical protein	Bombus impatiens					
ACC_02725	conserved hypothetical protein	Bombus impatiens					
ACC_05313	conserved hypothetical protein	Bombus impatiens					
ACC_02211	conserved hypothetical protein	Bombus impatiens					
ACC_05754	conserved hypothetical protein	Bombus impatiens					
ACC_06136	conserved hypothetical protein	Bombus impatiens					
ACC_02171	conserved hypothetical protein	Bombus impatiens					

ACC_05133	conserved hypothetical protein	Bombus impatiens					
ACC_06286	JmjC domain-containing protein	Bombus impatiens		KOG2132	GO:0005634:nucleus	GO:0003682:chromatin binding	GO:0000086:G2/M transition of mitotic cell
ACC_09001	Longitudinals lacking protein, isoforms	Bombus impatiens		KOG2462	GO:0005622:intracellular	GO:0008270:zinc ion binding	
ACC_02244	Magnesium-dependent phosphatase	Bombus impatiens		KOG4549	GO:0042645:mitochondrial nucleoid	GO:0002020:protease binding	GO:0006287:base-excision repair, gap-
ACC_07467	Mariner Mos1	Bombus impatiens					
ACC_06154	Matrix metalloproteinase-24	Bombus impatiens					GO:0008152:metabolic process
ACC_03013	Mediator of RNA polymerase II transcription subunit	Bombus impatiens	K15141	KOG1883			GO:0006351:transcription, DNA-dependent
ACC_06174	PRKR-interacting	Bombus impatiens		KOG4055	GO:0005634:nucleus		
ACC_06777	Protein ACN9 homolog, mitochondrial	Bombus impatiens		KOG4100	GO:0005739:mitochondrion		
ACC_01062	Protein G12	Bombus impatiens					
ACC_07923	Protein muscleblind	Bombus impatiens		KOG2494		GO:0003676:nucleic acid binding	
ACC_04825	Putative RNA polymerase II subunit B1 CTD phosphatase	Bombus impatiens		KOG4780			
ACC_06167	Replication protein A	Bombus impatiens	K10739	KOG3108			
ACC_06173	Synaptic vesicle 2-related protein	Bombus impatiens		KOG0253	GO:0016021:integral to membrane	GO:0022857:transmembrane transporter	GO:0055085:transmembrane transport
ACC_05461	Trafficking protein particle complex	Bombus impatiens		KOG3487	GO:0005622:intracellular		GO:0006888:ER to Golgi vesicle-mediated
ACC_04546	Translation initiation factor IF-3, chloroplastic	Bombus impatiens				GO:0003743:translation initiation factor activity	GO:0006413:translational initiation
ACC_00474	Transmembrane	Bombus impatiens		KOG4694			
ACC_05400	Myosin class II heavy	Bombus impatiens		KOG0161			
ACC_06312	WD repeat-containing protein	Bombus impatiens		KOG2247	GO:0031514:motile cilium		
ACC_00920	39S ribosomal protein L42, mitochondrial	Bombus terrestris		KOG4106	GO:0005763:mitochondrial small ribosomal		
ACC_04417	Actin-related protein 2-	Bombus terrestris		KOG0676			
ACC_05712	AP-2 complex subunit sigma	Bombus terrestris	K11827	KOG0935	GO:0030121:AP-1 adaptor complex	GO:0008565:protein transporter activity	GO:0007411:axon guidance
ACC_07740	Bifunctional heparan sulfate N-deacetylase/N-sulfotransferase	Bombus terrestris					
ACC_01467	Coiled-coil domain-containing protein	Bombus terrestris					
ACC_03070	cytohesin-1-like, partial	Bombus terrestris					

ACC_06179	dnaJ homolog subfamily B member 6-like	Bombus terrestris			GO:0005634:nucleus	GO:0003677:DNA binding	GO:0006310:DNA recombination
ACC_06518	DnaJ homolog subfamily C member	Bombus terrestris		KOG0715		GO:0031072:heat shock protein binding	GO:0006457:protein folding
ACC_02829	FAST kinase domain-containing protein	Bombus terrestris					
ACC_05124	homeobox protein rough-like	Bombus terrestris					
ACC_09240	conserved hypothetical protein	Bombus terrestris					
ACC_00813	conserved hypothetical protein	Bombus terrestris					
ACC_02593	conserved hypothetical protein	Bombus terrestris					
ACC_02886	conserved hypothetical protein	Bombus terrestris					
ACC_05353	conserved hypothetical protein	Bombus terrestris					
ACC_08694	conserved hypothetical protein	Bombus terrestris					
ACC_09462	conserved hypothetical protein	Bombus terrestris					
ACC_00085	conserved hypothetical protein	Bombus terrestris					
ACC_00010	conserved hypothetical protein	Bombus terrestris					
ACC_07984	conserved hypothetical protein	Bombus terrestris					
ACC_04002	conserved hypothetical protein	Bombus terrestris					
ACC_01675	conserved hypothetical protein	Bombus terrestris					
ACC_02682	conserved hypothetical protein	Bombus terrestris					
ACC_06092	conserved hypothetical protein	Bombus terrestris					
ACC_09081	conserved hypothetical protein	Bombus terrestris					
ACC_01017	conserved hypothetical protein	Bombus terrestris					
ACC_08764	conserved hypothetical protein	Bombus terrestris					
ACC_09065	conserved hypothetical protein	Bombus terrestris					
ACC_04111	conserved hypothetical protein	Bombus terrestris					

ACC_06860	conserved hypothetical protein	Bombus terrestris					
ACC_05887	conserved hypothetical protein	Bombus terrestris					
ACC_07627	conserved hypothetical protein	Bombus terrestris					
ACC_00629	conserved hypothetical protein	Bombus terrestris				GO:0003677:DNA binding	
ACC_01052	conserved hypothetical protein	Bombus terrestris					
ACC_05065	Kinetochore-associated protein	Bombus terrestris		KOG4256	GO:0043232:intracellular non-membrane-bounded organelle		GO:0022402:cell cycle process
ACC_06139	Leucine-rich repeat neuronal protein	Bombus terrestris		KOG4194			
ACC_09132	potassium voltage-gated channel subfamily KQT member	Bombus terrestris					
ACC_04020	Primary ciliary dyskinesia protein	Bombus terrestris					
ACC_08820	Probable protein	Bombus terrestris	K05752		GO:0005737:cytoplasm		
ACC_08039	protein FAM46A-like isoform 4	Bombus terrestris					
ACC_07288	Regulator of differentiation	Bombus terrestris		KOG1190	GO:0005634:nucleus	GO:0003697:single-stranded DNA binding	GO:0008380:RNA splicing
ACC_06905	reverse transcriptase	Bombus terrestris					
ACC_02899	Transcription initiation protein SPT3 homolog	Bombus terrestris	K11313	KOG3902	GO:0070461:SAGA-type complex	GO:0046982:protein heterodimerization	GO:0016570:histone modification
ACC_01777	Vacuolar import and degradation protein	Bombus terrestris		KOG4635			
ACC_08140	Vacuolar import and degradation protein	Bombus terrestris		KOG4635			
ACC_09101	conserved hypothetical protein	Bombus terrestris					
ACC_00458	UPF0672 protein C3orf58 homolog	Bombus terrestris					
ACC_01948	Retrovirus-related Pol polyprotein from type-1 retrotransposable element R1	Bombyx mori				GO:0003824:catalytic activity	
ACC_05897	cytohesin-1-like isoform	Camponotus floridanus					
ACC_00467	FAST kinase domain-containing protein	Camponotus floridanus				GO:0004672:protein kinase activity	
ACC_04485	conserved hypothetical protein	Camponotus floridanus					

ACC_07709	conserved hypothetical protein	Camponotus floridanus					
ACC_07468	conserved hypothetical protein	Camponotus floridanus					
ACC_07509	conserved hypothetical protein	Camponotus floridanus					
ACC_05116	conserved hypothetical protein	Camponotus floridanus					
ACC_00232	conserved hypothetical protein	Camponotus floridanus					
ACC_02798	conserved hypothetical protein	Camponotus floridanus					
ACC_08439	conserved hypothetical protein	Camponotus floridanus					
ACC_04090	conserved hypothetical protein	Camponotus floridanus					
ACC_04242	Lipopolysaccharide-induced tumor necrosis factor-alpha factor	Camponotus floridanus					
ACC_01093	TPPP family protein	Camponotus floridanus		KOG4070			
ACC_08341	Transmembrane	Camponotus floridanus					
ACC_01534	WD repeat-containing protein	Camponotus floridanus		KOG4497			
ACC_04985	Superoxide dismutase [Cu-Zn], chloroplastic	Cu-Zn		KOG0441		GO:0004784:superoxide dismutase activity	GO:0055114:oxidation-reduction process
ACC_08861	PiggyBac transposable element-derived	Danaus plexippus					
ACC_04844	conserved hypothetical protein	Danaus plexippus					
ACC_10118	conserved hypothetical protein	Daphnia pulex					
ACC_06915	Cytochrome P450 4C1	Harpegnathos saltator					
ACC_00190	Dosage compensation complex, subunit MLE	Harpegnathos saltator		KOG0921			
ACC_01960	conserved hypothetical protein	Harpegnathos saltator					
ACC_01338	conserved hypothetical protein	Harpegnathos saltator					
ACC_06227	conserved hypothetical protein	Harpegnathos saltator					
ACC_05766	conserved hypothetical protein	Harpegnathos saltator					
ACC_08710	conserved hypothetical protein	Harpegnathos saltator					
ACC_04602	conserved hypothetical protein	Harpegnathos saltator					

ACC_02550	conserved hypothetical protein	Harpegnathos saltator					
ACC_09318	conserved hypothetical protein	Harpegnathos saltator					
ACC_07962	conserved hypothetical protein	Harpegnathos saltator					
ACC_03294	conserved hypothetical protein	Harpegnathos saltator					
ACC_01797	conserved hypothetical protein	Harpegnathos saltator			GO:0005576:extracellular region	GO:0008061:chitin binding	GO:0006030:chitin metabolic process
ACC_06790	conserved hypothetical protein	Harpegnathos saltator					
ACC_02887	conserved hypothetical protein	Harpegnathos saltator					
ACC_04376	Neurobeachin	Harpegnathos saltator					
ACC_01696	tRNA dimethylallyltransferase, mitochondrial-like,	Harpegnathos saltator					
ACC_02399	Proteasome assembly chaperone	Ixodes scapularis		KOG4828			
ACC_07486	reverse transcriptase	Limulus polyphemus					
ACC_02644	Cytochrome c oxidase assembly protein	Megachile rotundata		KOG3477			
ACC_10143	General transcription factor IIH subunit	Megachile rotundata	K10845	KOG3451	GO:0005634:nucleus	GO:0003677:DNA binding	GO:0006289:nucleotide-excision repair
ACC_09461	Homogentisate 1,2-dioxygenase	Megachile rotundata		KOG1417			GO:0006559:L-phenylalanine catabolic process
ACC_04389	Max dimerization	Megachile rotundata	K09114	KOG2483			
ACC_09384	Max dimerization	Megachile rotundata					
ACC_02457	putative RNA-binding protein EEED8.10-like	Megachile rotundata		KOG4341			
ACC_01187	Ribonuclease P protein subunit p14	Megachile rotundata	K14529			GO:0004540:ribonuclease activity	GO:0008033:tRNA processing
ACC_00827	Transmembrane inner ear expressed protein	Megachile rotundata					
ACC_07074	conserved hypothetical protein	Megachile rotundata					
ACC_01407	zinc finger protein 717-	Megachile rotundata					
ACC_05489	Zinc finger protein basonuclin-2	Megachile rotundata			GO:0005737:cytoplasm	GO:0046872:metal ion binding	
ACC_02413	Hemocyanin C chain	Metacarcinus magister			GO:0005576:extracellular region		GO:0008152:metabolic process
ACC_02154	conserved hypothetical protein	Mixia osmundae IAM 14324					

ACC_04857	High mobility group B protein	Nasonia vitripennis					
ACC_00370	Transcription factor IIB	Nasonia vitripennis		KOG3213			
ACC_07400	conserved hypothetical protein	Nasonia vitripennis					
ACC_00941	conserved hypothetical protein	Nasonia vitripennis					
ACC_07447	Retrovirus-related Pol polyprotein from type-1 retrotransposable element R2	Nasonia vitripennis				GO:0003723:RNA binding	GO:0006278:RNA-dependent DNA replication
ACC_02003	reverse transcriptase	Nasonia vitripennis				GO:0003723:RNA binding	GO:0006278:RNA-dependent DNA
ACC_07542	reverse transcriptase	Nasonia vitripennis					
ACC_07618	rhomboid family	Nasonia vitripennis					
ACC_05427	probable calcium-binding protein CML13-	Nematostella vectensis					
ACC_05737	Cytochrome c-2	Neptunea arthritica					
ACC_02617	ADP-ribosylation factor	Oryctolagus cuniculus	K07941	KOG0071	GO:0005938:cell cortex	GO:0005525:GTP binding	GO:0030866:cortical actin cytoskeleton
ACC_05466	cysteine-rich motor neuron 1 protein-like	Pediculus humanus corporis					
ACC_09128	conserved hypothetical protein	Pediculus humanus corporis					
ACC_05522	conserved hypothetical protein	Pediculus humanus corporis					
ACC_04243	Sulfur controller	Pediculus humanus		KOG0274			
ACC_02243	Cytochrome c oxidase subunit	Scylla paramamosain	K02256		GO:0016021:integral to membrane	GO:0004129:cytochrome-c oxidase activity	GO:0009060:aerobic respiration
ACC_05894	Ferritin	Scylla paramamosain	K00522	KOG2332	GO:0005737:cytoplasm	GO:0004322:ferroxidase activity	GO:0006879:cellular iron ion homeostasis
ACC_07575	Gamma-aminobutyric acid receptor subunit	Solenopsis invicta			GO:0030054:cell junction	GO:0004890:GABA-A receptor activity	GO:0006821:chloride transport
ACC_00957	Thyroid hormone receptor-associated protein complex,	Solenopsis invicta		KOG3598			
ACC_10159	conserved hypothetical protein	Solenopsis invicta					
ACC_03353	conserved hypothetical protein	Solenopsis invicta					
ACC_00363	conserved hypothetical protein	Solenopsis invicta				GO:0004045:aminoacyl-tRNA hydrolase activity	
ACC_04523	conserved hypothetical protein	Solenopsis invicta					
ACC_04933	conserved hypothetical protein	Solenopsis invicta					

ACC_02465	Nucleolar protein of	Solenopsis invicta		KOG0922	GO:0022625:cytosolic large ribosomal subunit	GO:0003723:RNA binding	
ACC_08219	Putative gustatory receptor	Solenopsis invicta	K08471		GO:0016021:integral to membrane		GO:0050909:sensory perception of taste
ACC_07155	RING finger protein	Solenopsis invicta	K15708		GO:0044464:cell part		
ACC_07485	conserved hypothetical protein	Tribolium castaneum					
ACC_08198	conserved hypothetical protein	Tribolium castaneum					
ACC_04988	conserved hypothetical protein	Tribolium castaneum					
ACC_05366	conserved hypothetical protein	Tribolium castaneum					
ACC_07466	conserved hypothetical protein	Tribolium castaneum					
ACC_07556	conserved hypothetical protein	Tribolium castaneum					
ACC_01348	lactosylceramide 4-alpha-	Vitis vinifera					
ACC_04984	conserved hypothetical protein	Xenopus (Silurana) tropicalis					
ACC_00857	Cytidylate kinase						
ACC_00844	Cytochrome b						
ACC_01436	Dosage compensation complex, subunit MLE			KOG0921			
ACC_04893	Dosage compensation complex, subunit MLE			KOG0921			
ACC_02727	Serine/threonine protein kinase			KOG2268			
ACC_00011	hypothetical protein						
ACC_00116	hypothetical protein						
ACC_00140	hypothetical protein						
ACC_00231	hypothetical protein						
ACC_00234	hypothetical protein						
ACC_00242	hypothetical protein						
ACC_00254	hypothetical protein						
ACC_00259	hypothetical protein						
ACC_00260	hypothetical protein						
ACC_00275	hypothetical protein						
ACC_00287	hypothetical protein						
ACC_00314	hypothetical protein						
ACC_00354	hypothetical protein						
ACC_00364	hypothetical protein						
ACC_00365	hypothetical protein						
ACC_00376	hypothetical protein						
ACC_00378	hypothetical protein						

ACC_00379	hypothetical protein
ACC_00404	hypothetical protein
ACC_00405	hypothetical protein
ACC_00518	hypothetical protein
ACC_00523	hypothetical protein
ACC_00530	hypothetical protein
ACC_00537	hypothetical protein
ACC_00538	hypothetical protein
ACC_00542	hypothetical protein
ACC_00578	hypothetical protein
ACC_00581	hypothetical protein
ACC_00585	hypothetical protein
ACC_00607	hypothetical protein
ACC_00725	hypothetical protein
ACC_00766	hypothetical protein
ACC_00880	hypothetical protein
ACC_00926	hypothetical protein
ACC_00928	hypothetical protein
ACC_00930	hypothetical protein
ACC_00931	hypothetical protein
ACC_00932	hypothetical protein
ACC_00952	hypothetical protein
ACC_00962	hypothetical protein
ACC_01008	hypothetical protein
ACC_01009	hypothetical protein
ACC_01047	hypothetical protein
ACC_01056	hypothetical protein
ACC_01066	hypothetical protein
ACC_01095	hypothetical protein
ACC_01110	hypothetical protein
ACC_01247	hypothetical protein
ACC_01272	hypothetical protein
ACC_01285	hypothetical protein
ACC_01313	hypothetical protein
ACC_01329	hypothetical protein
ACC_01347	hypothetical protein
ACC_01384	hypothetical protein
ACC_01403	hypothetical protein
ACC_01422	hypothetical protein
ACC_01454	hypothetical protein
ACC_01456	hypothetical protein
ACC_01501	hypothetical protein
ACC_01503	hypothetical protein
ACC_01512	hypothetical protein
ACC_01522	hypothetical protein
ACC_01557	hypothetical protein

ACC_01583	hypothetical protein
ACC_01594	hypothetical protein
ACC_01597	hypothetical protein
ACC_01612	hypothetical protein
ACC_01626	hypothetical protein
ACC_01642	hypothetical protein
ACC_01657	hypothetical protein
ACC_01658	hypothetical protein
ACC_01682	hypothetical protein
ACC_01689	hypothetical protein
ACC_01691	hypothetical protein
ACC_01703	hypothetical protein
ACC_01704	hypothetical protein
ACC_01708	hypothetical protein
ACC_01760	hypothetical protein
ACC_01767	hypothetical protein
ACC_01826	hypothetical protein
ACC_01833	hypothetical protein
ACC_01947	hypothetical protein
ACC_01954	hypothetical protein
ACC_01965	hypothetical protein
ACC_02008	hypothetical protein
ACC_02025	hypothetical protein
ACC_02077	hypothetical protein
ACC_02093	hypothetical protein
ACC_02105	hypothetical protein
ACC_02128	hypothetical protein
ACC_02129	hypothetical protein
ACC_02131	hypothetical protein
ACC_02174	hypothetical protein
ACC_02217	hypothetical protein
ACC_02257	hypothetical protein
ACC_02288	hypothetical protein
ACC_02352	hypothetical protein
ACC_02356	hypothetical protein
ACC_02363	hypothetical protein
ACC_02366	hypothetical protein
ACC_02367	hypothetical protein
ACC_02368	hypothetical protein
ACC_02381	hypothetical protein
ACC_02382	hypothetical protein
ACC_02386	hypothetical protein
ACC_02436	hypothetical protein
ACC_02440	hypothetical protein
ACC_02446	hypothetical protein
ACC_02467	hypothetical protein

ACC_02468	hypothetical protein
ACC_02469	hypothetical protein
ACC_02470	hypothetical protein
ACC_02489	hypothetical protein
ACC_02490	hypothetical protein
ACC_02492	hypothetical protein
ACC_02524	hypothetical protein
ACC_02548	hypothetical protein
ACC_02563	hypothetical protein
ACC_02623	hypothetical protein
ACC_02642	hypothetical protein
ACC_02663	hypothetical protein
ACC_02704	hypothetical protein
ACC_02791	hypothetical protein
ACC_02792	hypothetical protein
ACC_02812	hypothetical protein
ACC_02849	hypothetical protein
ACC_02901	hypothetical protein
ACC_02908	hypothetical protein
ACC_02921	hypothetical protein
ACC_02978	hypothetical protein
ACC_03006	hypothetical protein
ACC_03023	hypothetical protein
ACC_03120	hypothetical protein
ACC_03240	hypothetical protein
ACC_03285	hypothetical protein
ACC_03286	hypothetical protein
ACC_03336	hypothetical protein
ACC_03352	hypothetical protein
ACC_03416	hypothetical protein
ACC_03448	hypothetical protein
ACC_03454	hypothetical protein
ACC_03531	hypothetical protein
ACC_03545	hypothetical protein
ACC_03757	hypothetical protein
ACC_03760	hypothetical protein
ACC_03779	hypothetical protein
ACC_03780	hypothetical protein
ACC_03793	hypothetical protein
ACC_03797	hypothetical protein
ACC_03801	hypothetical protein
ACC_03912	hypothetical protein
ACC_04049	hypothetical protein
ACC_04062	hypothetical protein
ACC_04220	hypothetical protein
ACC_04237	hypothetical protein

ACC_04239	hypothetical protein	
ACC_04244	hypothetical protein	
ACC_04249	hypothetical protein	
ACC_04273	hypothetical protein	
ACC_04281	hypothetical protein	
ACC_04285	hypothetical protein	
ACC_04316	hypothetical protein	
ACC_04353	hypothetical protein	
ACC_04407	hypothetical protein	
ACC_04408	hypothetical protein	
ACC_04474	hypothetical protein	
ACC_04536	hypothetical protein	
ACC_04587	hypothetical protein	GO:0003677:DNA
ACC_04676	hypothetical protein	
ACC_04767	hypothetical protein	
ACC_04799	hypothetical protein	
ACC_04800	hypothetical protein	
ACC_04801	hypothetical protein	
ACC_04903	hypothetical protein	
ACC_04950	hypothetical protein	
ACC_04954	hypothetical protein	
ACC_04957	hypothetical protein	
ACC_05000	hypothetical protein	
ACC_05013	hypothetical protein	
ACC_05078	hypothetical protein	
ACC_05081	hypothetical protein	
ACC_05108	hypothetical protein	
ACC_05112	hypothetical protein	
ACC_05196	hypothetical protein	
ACC_05225	hypothetical protein	
ACC_05231	hypothetical protein	
ACC_05240	hypothetical protein	
ACC_05328	hypothetical protein	
ACC_05330	hypothetical protein	
ACC_05385	hypothetical protein	
ACC_05433	hypothetical protein	
ACC_05442	hypothetical protein	
ACC_05497	hypothetical protein	
ACC_05527	hypothetical protein	
ACC_05544	hypothetical protein	
ACC_05568	hypothetical protein	
ACC_05585	hypothetical protein	
ACC_05593	hypothetical protein	
ACC_05631	hypothetical protein	
ACC_05674	hypothetical protein	
ACC_05748	hypothetical protein	

ACC_05751	hypothetical protein
ACC_05757	hypothetical protein
ACC_05758	hypothetical protein
ACC_05805	hypothetical protein
ACC_05829	hypothetical protein
ACC_05832	hypothetical protein
ACC_05839	hypothetical protein
ACC_05848	hypothetical protein
ACC_05849	hypothetical protein
ACC_05851	hypothetical protein
ACC_05860	hypothetical protein
ACC_05867	hypothetical protein
ACC_05877	hypothetical protein
ACC_05878	hypothetical protein
ACC_05888	hypothetical protein
ACC_05942	hypothetical protein
ACC_05951	hypothetical protein
ACC_05956	hypothetical protein
ACC_05978	hypothetical protein
ACC_06022	hypothetical protein
ACC_06053	hypothetical protein
ACC_06085	hypothetical protein
ACC_06159	hypothetical protein
ACC_06172	hypothetical protein
ACC_06225	hypothetical protein
ACC_06244	hypothetical protein
ACC_06365	hypothetical protein
ACC_06373	hypothetical protein
ACC_06379	hypothetical protein
ACC_06388	hypothetical protein
ACC_06389	hypothetical protein
ACC_06399	hypothetical protein
ACC_06400	hypothetical protein
ACC_06461	hypothetical protein
ACC_06506	hypothetical protein

Supplementary Table 12. Homeobox protein genes of A.cerana

Gene	Scaffold	Start	End	Chromosome	Strand	IPR No.	IPR Description	Domain	Annotation	Homolog in A.mellifera
ACC_09464	scaffold98.1	695909	704222	Ac10	+	IPR009057	Homeodomain-like	SSF46689 (SUPERFAMILY)	Paired box protein Pax-1	GB10772
ACC_06221	scaffold72.1	518270	520155	Ac3	+	IPR001356	Homeodomain	PF00046 (PFAM)	Paired box protein Pax-3	GB16151
ACC_01021	scaffold18.1	966239	1009288	Ac6	-	IPR009057	Homeodomain-like	SSF46689 (SUPERFAMILY)	Paired box protein Pax-6 (twin of eyeless-toy)	GB11714
ACC_09296	scaffold70.1	3164619	3225778	Ac1	+	IPR009057	Homeodomain-like	SSF46689 (SUPERFAMILY)	Paired box protein Pax-6 (eyeless)	GB12355
ACC_07211	scaffold292.1	148218	154470	Ac13	-	IPR001356	Homeodomain	PF00046 (PFAM)	Paired box protein Pax-7	GB20010
ACC_02837	scaffold8.1	1333046	1345308	Ac2	-	IPR009057	Homeodomain-like	GO:0003677	Pax-6 protein (eyegone)	GB15698
ACC_03634	scaffold124.1	880754	887511	Ac14	+	IPR009057	Homeodomain-like	SSF46689 (SUPERFAMILY)	Arginine-glutamic acid dipeptide repeats protein	GB18685
ACC_08434	scaffold162.1	359329	361546	Ac9	+	IPR009057	Homeodomain-like	SSF46689 (SUPERFAMILY)	Arginine-glutamic acid dipeptide repeats protein	GB18456
ACC_07827	scaffold64.1	183952	185147	Ac14	-	IPR001356	Homeodomain	PF00046 (PFAM)	Aristaless-related homeobox protein	GB16661
ACC_02487	scaffold58.1	1023029	1031576	Ac13	-	IPR001356	Homeodomain	PF00046 (PFAM)	BarH-like 1 homeobox protein	GB15934
ACC_07697	scaffold36.1	312384	316472	Ac12	-	IPR001356	Homeodomain	PF00046 (PFAM)	Brain-specific homeobox protein	GB15683
ACC_02574	scaffold69.1	262130	395875	Ac1	-	IPR009057	Homeodomain-like	SSF46689 (SUPERFAMILY)	Bromodomain adjacent to zinc finger domain protein	GB16601
ACC_04846	scaffold234.1	1738015	1741250	Ac4	+	IPR009057	Homeodomain-like	SSF46689 (SUPERFAMILY)	Cell division cycle 5-like protein	GB13156
ACC_02066	scaffold36.1	436687	439716	Ac12	-	IPR009057	Homeodomain-like	SSF46689 (SUPERFAMILY)	Chromatin-remodeling complex ATPase chain Iswi	GB14577
ACC_06603	scaffold101.1	540035	562636	Ac12	-	IPR009057	Homeodomain-like	SSF46689 (SUPERFAMILY)	Chromodomain-helicase-DNA-binding protein	GB17138
ACC_01173	scaffold196.1	247294	248466	Ac5	-	IPR009057	Homeodomain-like	SSF46689 (SUPERFAMILY)	conserved hypothetical protein	
ACC_03663	scaffold126.1	1120677	1123234	Ac10	+	IPR009057	Homeodomain-like	SSF46689 (SUPERFAMILY)	conserved hypothetical protein	GB10399
ACC_04561	scaffold70.1	1283390	1292419	Ac1	+	IPR009057	Homeodomain-like	SSF46689 (SUPERFAMILY)	conserved hypothetical protein	GB12686
ACC_09292	scaffold70.1	1286714	1293101	Ac1	+	IPR009057	Homeodomain-like	SSF46689 (SUPERFAMILY)	conserved hypothetical protein	GB12686
ACC_09842	scaffold72.1	1433107	1435878	Ac3	+	IPR009057	Homeodomain-like	SSF46689 (SUPERFAMILY)	conserved hypothetical protein	GB10994
ACC_05435	scaffold181.1	414120	415183	Ac1	+	IPR009057	Homeodomain-like	GO:0003677	DNA-directed RNA polymerases I, II, and III subunit RPABC5	GB15729
ACC_03533	scaffold173.1	386736	394822	Ac2	+	IPR009057	Homeodomain-like	SSF46689 (SUPERFAMILY)	DnaJ homolog subfamily C member	GB17700
ACC_07013	scaffold82.1	613095	614857	Ac8	-	IPR009057	Homeodomain-like	SSF46689 (SUPERFAMILY)	DnaJ homolog subfamily C member	GB15674

ACC_08029	scaffold8.1	2831774	2857871	Ac2	-	IPR001356	Homeodomain	PF00046 (PFAM)	GS homeobox	GB14802
ACC_07593	scaffold9.1	2522246	2524140	Ac9	-	IPR001356	Homeodomain	PF00046 (PFAM)	H2.0-like homeobox protein	GB13745
ACC_08907	scaffold31.1	2549238	2654663	Ac11	+	IPR001356	Homeodomain	PS50071 (PROFILE)	Hepatocyte nuclear factor	GB13918
ACC_10177	scaffold38.1	1026751	1020126	Ac16	-	IPR001356	Homeodomain	PF00046 (PFAM)	homeobox protein	GB10341
ACC_10178	scaffold231.1	318452	350244	Ac16	+	IPR001356	Homeodomain	PF00046 (PFAM)	homeobox protein	GB18833
ACC_10179	scaffold141.1	988789	970698	Ac16	-	IPR001356	Homeodomain	PF00046 (PFAM)	homeobox protein	GB14027
ACC_02085	scaffold38.1	779996	809498	Ac16	-	IPR001356	Homeodomain	PF00046 (PFAM)	Homeobox protein abdominal-A homolog	GB19738
ACC_00577	scaffold138.1	811030	831268	Ac5	-	IPR001356	Homeodomain	PS50071 (PROFILE)	Homeobox protein AKR	GB14146
ACC_08661	scaffold200.1	198611	200294	Ac15	+	IPR001356	Homeodomain	PF00046 (PFAM)	Homeobox protein ceh-19	GB11720
ACC_09696	scaffold127.1	1137889	1238282	Ac6	-	IPR003350	Homeodomain protein C	PF02376 (PFAM)	Homeobox protein cut	GB17945
ACC_07227	scaffold72.1	1205825	1209246	Ac3	-	IPR001356	Homeodomain	PF00046 (PFAM)	Homeobox protein extradenticle	GB15837
ACC_09893	scaffold19.1	423408	424088	Ac1	-	IPR001356	Homeodomain	PF00046 (PFAM)	Homeobox protein GBX-2	GB13163
ACC_06689	scaffold109.1	1550388	1558626	Ac6	-	IPR001356	Homeodomain	PF00046 (PFAM)	Homeobox protein goosecoid	GB12726
ACC_06748	scaffold38.1	51157	55198	Ac16	-	IPR001356	Homeodomain	PF00046 (PFAM)	Homeobox protein Hox-A3	GB18792
ACC_02757	scaffold75.1	256934	262617	Ac7	-	IPR001356	Homeodomain	PF00046 (PFAM)	Homeobox protein MOX-2	GB18266
ACC_00391	scaffold124.1	252768	253301	Ac14	-	IPR001356	Homeodomain	PF00046 (PFAM)	Homeobox protein MSH-B	GB15571
ACC_09982	scaffold10.1	2362133	2381494	Ac5	-	IPR001356	Homeodomain	PF00046 (PFAM)	Homeobox protein Nkx-2.4	GB16784
ACC_01473	scaffold227.1	29273	60962	Ac8	-	IPR001356	Homeodomain	PF00046 (PFAM)	Homeobox protein Nkx-6.1	GB13581
ACC_07095	scaffold2.1	1613041	1615245	Ac8	-	IPR001356	Homeodomain	PF00046 (PFAM)	Homeobox protein not2	GB11491
ACC_07390	scaffold2.1	1613037	1615197	Ac8	-	IPR001356	Homeodomain	PF00046 (PFAM)	Homeobox protein not2	GB11491
ACC_06561	scaffold1.1	2405054	2431196	Ac1	-	IPR001356	Homeodomain	PF00046 (PFAM)	Homeobox protein orthopedia	GB10285
ACC_05205	scaffold216.1	40769	44943	Ac1	+	IPR001356	Homeodomain	PF00046 (PFAM)	Homeobox protein OTX2	GB11566
ACC_08467	scaffold68.1	338453	343466	Ac1	+	IPR001356	Homeodomain	PF00046 (PFAM)	Homeobox protein OTX2	GB16866
ACC_05511	scaffold36.1	330258	333709	Ac12	+	IPR001356	Homeodomain	PS50071 (PROFILE)	Homeobox protein PKNOX2	GB13662
ACC_05946	scaffold55.1	910040	948846	Ac5	+	IPR009057	Homeodomain-like	SSF46689 (SUPERFAMILY)	Homeobox protein prospero	GB14533
ACC_09148	scaffold55.1	879385	884985	Ac5	+	IPR009057	Homeodomain-like	SSF46689 (SUPERFAMILY)	Homeobox protein prospero	GB14533
ACC_01994	scaffold330.1	134967	143512	Ac9	-	IPR001356	Homeodomain	PF00046 (PFAM)	Homeobox protein rough	GB20009
ACC_09838	scaffold19.1	2130705	2136237	Ac1	-	IPR001356	Homeodomain	PF00046 (PFAM)	Homeobox protein SIX4	GB10752
ACC_03355	scaffold104.1	941056	987422	Ac14	+	IPR001356	Homeodomain	PF00046 (PFAM)	Homeobox protein unc-4	GB10221
ACC_07850	scaffold147.1	427151	430083	Ac9	-	IPR001356	Homeodomain	PF00046 (PFAM)	Homeobox protein XENK-2	GB15027
ACC_04207	scaffold1.1	970720	971291	Ac1	+	IPR001356	Homeodomain	PF00046 (PFAM)	Homeodomain-only protein	GB16549
ACC_10181	scaffold38.1	361237	348766	Ac16	-	IPR001356	Homeodomain	PF00046 (PFAM)	homeotic protein antennapedia-like	GB18813
ACC_02748	scaffold74.1	371220	381124	Ac1	-	IPR001356	Homeodomain	PF00046 (PFAM)	Homeotic protein caudal	GB10821
ACC_06749	scaffold38.1	105338	114231	Ac16	-	IPR001356	Homeodomain	PF00046 (PFAM)	Homeotic protein deformed	GB13409
ACC_09153	scaffold58.1	857660	865528	Ac13	+	IPR001356	Homeodomain	PF00046 (PFAM)	Homeotic protein distal-less	GB14516
ACC_04241	scaffold171.1	511171	513401	Ac7	+	IPR001356	Homeodomain	PF00046 (PFAM)	Insulin gene enhancer protein ISL-1	GB12465
ACC_08931	scaffold71.1	335130	336422	Ac11	+	IPR001356	Homeodomain	PS50071 (PROFILE)	Iroquois-class homeodomain protein irx-5	GB11441
ACC_03497	scaffold111.1	356050	356908	Ac7	+	IPR009057	Homeodomain-like	SSF46689 (SUPERFAMILY)	Jerky protein homolog-like	GB18608

ACC_05142	scaffold288.1	73652	80698		+	IPR009057	Homeodomain-like	SSF46689 (SUPERFAMILY)	LAG1 longevity assurance homolog	GB17180
ACC_04169	scaffold189.1	73138	79918	Ac3	-	IPR001356	Homeodomain	PF00046 (PFAM)	LIM homeobox transcription factor	GB19877
ACC_08618	scaffold70.1	972602	980136	Ac1	+	IPR001356	Homeodomain	PF00046 (PFAM)	LIM/homeobox protein Awh	GB14318
ACC_09289	scaffold70.1	967364	979992	Ac1	+	IPR001356	Homeodomain	PF00046 (PFAM)	LIM/homeobox protein Awh	GB14318
ACC_06338	scaffold8.1	2966017	2987858	Ac2	+	IPR001356	Homeodomain	PF00046 (PFAM)	LIM/homeobox protein Lhx1	GB12408
ACC_02463	scaffold55.1	326429	331149	Ac5	-	IPR001356	Homeodomain	PF00046 (PFAM)	LIM/homeobox protein Lhx3	GB15651
ACC_07974	scaffold77.1	238695	295696	Ac11	-	IPR001356	Homeodomain	PF00046 (PFAM)	LIM/homeobox protein Lhx9	GB16957
ACC_04168	scaffold189.1	107803	111288	Ac3	-	IPR009057	Homeodomain-like	SSF46689 (SUPERFAMILY)	Longitudinals lacking protein, isoform G	GB14194
ACC_09420	scaffold9.1	1446410	1466325	Ac9	+	IPR009057	Homeodomain-like	SSF46689 (SUPERFAMILY)	Longitudinals lacking protein, isoforms F/I/K/T	GB12094
ACC_07158	scaffold52.1	133685	145131	Ac10	-	IPR009057	Homeodomain-like	SSF46689 (SUPERFAMILY)	Lysine-specific histone demethylase	GB12774
ACC_08919	scaffold28.1	115682	116692	Ac7	+	IPR009057	Homeodomain-like	SSF46689 (SUPERFAMILY)	Mariner Mos1 transposase	GB17021
ACC_03983	scaffold19.1	1815745	1818247	Ac1	+	IPR009057	Homeodomain-like	SSF46689 (SUPERFAMILY)	Mesoderm induction early response protein	GB16534
ACC_06811	scaffold10.1	2156315	2161183	Ac5	-	IPR009057	Homeodomain-like	SSF46689 (SUPERFAMILY)	Metastasis-associated protein MTA1	GB15896
ACC_02583	scaffold69.1	837018	838462	Ac1	-	IPR009057	Homeodomain-like	SSF46689 (SUPERFAMILY)	MLL1/MLL complex subunit C17orf49 homolog	GB19313
ACC_06738	scaffold128.1	191381	203080	Ac3	-	IPR001356	Homeodomain	PF00046 (PFAM)	Motor neuron and pancreas homeobox protein	GB11098
ACC_08585	scaffold19.1	1013714	1022027	Ac1	+	IPR001356	Homeodomain	PF00046 (PFAM)	Muscle segmentation homeobox	GB13830
ACC_09244	scaffold17.1	633415	642023	Ac1	+	IPR009057	Homeodomain-like	SSF46689 (SUPERFAMILY)	Myb protein	GB12498
ACC_09558	scaffold70.1	983404	1032681	Ac1	-	IPR001356	Homeodomain	PF00046 (PFAM)	N-terminal acetyltransferase B complex subunit NAA25	GB14837
ACC_09291	scaffold70.1	1240170	1265643	Ac1	+	IPR009057	Homeodomain-like	SSF46689 (SUPERFAMILY)	Nuclear receptor corepressor	GB10619
ACC_06144	scaffold31.1	2582416	2646033	Ac11	+	IPR001356	Homeodomain	PF00046 (PFAM)	One cut domain family member	GB13918
ACC_07718	scaffold126.1	752159	752596	Ac10	-	IPR001356	Homeodomain	PF00046 (PFAM)	POU domain protein CF1A	GB16085
ACC_03097	scaffold97.1	6262	19328	Ac4	-	IPR001356	Homeodomain	PF00046 (PFAM)	POU domain, class 2, transcription factor	GB16262
ACC_07233	scaffold97.1	8184	22066	Ac4	-	IPR001356	Homeodomain	PF00046 (PFAM)	POU domain, class 2, transcription factor	GB16262
ACC_09072	scaffold46.1	2907937	2930730	Ac13	+	IPR001356	Homeodomain	PF00046 (PFAM)	POU domain, class 6, transcription factor	GB15643
ACC_08079	scaffold15.1	7966	16400	Ac15	-	IPR001356	Homeodomain	PF00046 (PFAM)	Protein apterous	GB18585
ACC_01098	scaffold187.1	161965	188996	Ac8	-	IPR009057	Homeodomain-like	SSF46689 (SUPERFAMILY)	Protein bric-a-brac	GB14649
ACC_05438	scaffold181.1	475062	477285	Ac1	+	IPR009057	Homeodomain-like	SSF46689 (SUPERFAMILY)	Protein bric-a-brac	GB15091

ACC_07240	scaffold259.1	22655	24713	Ac8	-	IPR009057	Homeodomain-like	SSF46689 (SUPERFAMILY)	Protein bric-a-brac	GB16756
ACC_09705	scaffold9.1	1431759	1445995	Ac9	-	IPR009057	Homeodomain-like	SSF46689 (SUPERFAMILY)	Protein bric-a-brac	GB17640
ACC_09414	scaffold26.1	348837	358808	Ac14	+	IPR001356	Homeodomain	PF00046 (PFAM)	Protein gooseberry	GB15632
ACC_09937	scaffold108.1	621706	651237	Ac4	+	IPR001356	Homeodomain	PF00046 (PFAM)	Protein sine oculis	GB15213
ACC_04587	scaffold2.1	3817687	3818370	Ac8	+	IPR009057	Homeodomain-like	GO:0003677	putative DNA binding protein	
ACC_04543	scaffold2.1	583386	590172	Ac8	+	IPR021980	Transcription factor hom	PF12129 (PFAM)	Putative homeodomain transcription factor	GB11475
ACC_06522	scaffold98.1	630181	633998	Ac10	+	IPR009057	Homeodomain-like	SSF46689 (SUPERFAMILY)	REST corepressor	GB17956
ACC_10063	scaffold1.1	2467199	2500723	Ac1	+	IPR001356	Homeodomain	PF00046 (PFAM)	Retinal homeobox protein Rx	GB19717
ACC_06370	scaffold82.1	1381090	1385279	Ac8	+	IPR001356	Homeodomain	PF00046 (PFAM)	Retinal homeobox protein Rx2	GB14165
ACC_09194	scaffold63.1	83710	103308	Ac1	+	IPR001356	Homeodomain	PF00046 (PFAM)	Segmentation polarity homeobox protein engrailed	GB15566
ACC_07296	scaffold26.1	1560248	1564336	Ac14	-	IPR001356	Homeodomain	PF00046 (PFAM)	Segmentation protein even-skipped	GB10623
ACC_09947	scaffold105.1	300433	313954	Ac14	+	IPR001356	Homeodomain	PF00046 (PFAM)	Segmentation protein paired	GB15469
ACC_10180	scaffold38.1	208644	207830	Ac16	-	IPR001356	Homeodomain	PF00046 (PFAM)	Sex combs reduced homeotic protein	GB13491
ACC_09396	scaffold85.1	1925227	1927998	Ac4	-	IPR009057	Homeodomain-like	SSF46689 (SUPERFAMILY)	snRNA-activating protein complex subunit	GB15491
ACC_03315	scaffold103.1	437999	441773	Ac15	+	IPR009057	Homeodomain-like	SSF46689 (SUPERFAMILY)	SWI/SNF complex subunit SMARCC2	GB17902
ACC_08741	scaffold72.1	927335	931865	Ac3	+	IPR009057	Homeodomain-like	SSF46689 (SUPERFAMILY)	Tigger transposable element-derived protein	GB14204
ACC_07060	scaffold19.1	1167244	1167609	Ac1	-	IPR001356	Homeodomain	PF00046 (PFAM)	Transcription factor LBX1	GB10613
ACC_04113	scaffold164.1	1123854	1127241	Ac10	+	IPR009057	Homeodomain-like	SSF46689 (SUPERFAMILY)	Transcription factor TFIIIB component B'' homolog	GB13362
ACC_01991	scaffold33.1	1487257	1488884	Ac12	-	IPR009057	Homeodomain-like	SSF46689 (SUPERFAMILY)	Transcriptional adapter 2-alpha	GB16325
ACC_04960	scaffold25.1	867078	878781	Ac5	+	IPR009057	Homeodomain-like	SSF46689 (SUPERFAMILY)	Transcriptional adapter 2B	GB15568
ACC_09266	scaffold7.1	1529273	1532487	Ac5	+	IPR009057	Homeodomain-like	SSF46689 (SUPERFAMILY)	Transcriptional-regulating factor	GB14672
ACC_05615	scaffold9.1	2582391	2632678	Ac9	+	IPR001356	Homeodomain	PF00046 (PFAM)	Visual system homeobox	GB19883
ACC_09422	scaffold9.1	2621773	2638545	Ac9	+	IPR001356	Homeodomain	PS50071 (PROFILE)	Visual system homeobox	GB19883
ACC_08728	scaffold234.1	1873252	1877956	Ac4	+	IPR001356	Homeodomain	PF00046 (PFAM)	Zinc finger protein	GB18918
ACC_08882	scaffold234.1	1873483	1878236	Ac4	+	IPR001356	Homeodomain	PF00046 (PFAM)	Zinc finger protein	GB18918
ACC_01474	scaffold229.1	4747	6226	Ac11	-	IPR009057	Homeodomain-like	SSF46689 (SUPERFAMILY)	ZZ-type zinc finger-containing protein	GB19671

Supplementary Table 13. Venom genes in *A.cerana*

Gene	Venom	Allergen	Homolog in A.mellifera	Identity(%)	Subcellular Localization
ACC_00752	Protein 5NUC	Aed a 1	GB17218	92.23	Extracellular
ACC_08415	apyrase precursor	Aed a 1	GB12259	90.26	Cytoplasmic
ACC_02702	venom acid phosphatase	Api m 3	GB12546	99.23	Extracellular
ACC_09285	venom dipeptidyl peptidase 4	Api m 5	GB14496	89.9	Transmembrane
ACC_09897	phospholipase A2	Api m 1	GB13351	94.61	Extracellular
ACC_10171	Hyaluronidase	Api m 2	GB18543	92.76	SignalPeptide
ACC_10176	Melittin	Api m 4	GB10355	97.14	SignalPeptide
ACC_05072	CUB serine protease	Api m 7	GB11552	91.36	Transmembrane
ACC_03949	Lysosomal aspartic protease	Bla g 2	GB16903	97.4	Extracellular
ACC_03799	Glutathione S-transferase	Bla g 5	GB14372	48.06	Cytoplasmic
ACC_03800	Glutathione S-transferase	Bla g 5	GB16959	97.54	Cytoplasmic
ACC_04756	Glutathione S-transferase	Bla g 5	GB14372	98.06	Cytoplasmic
ACC_04982	Glutathione S-transferase	Bla g 5	GB19254	93.4	Cytoplasmic
ACC_00261	Tropomyosin-2	Chi k 10	GB13279	96.25	Cytoplasmic
ACC_01013	Tropomyosin-1	Chi k 10	GB10939	100	Cytoplasmic
ACC_04038	Tropomyosin	Chi k 10	GB10939	70.46	Cytoplasmic
ACC_06644	Tropomyosin-2	Chi k 10	GB19374	99.65	Cytoplasmic
ACC_00164	Disintegrin and metalloproteinase domain-containing protein	desintegrins	GB12763	82.68	Transmembrane
ACC_07062	ADAM 17-like protease	desintegrins	GB18818	97.82	Cytoplasmic
ACC_09182	A disintegrin and metalloproteinase with thrombospondin motifs	desintegrins	GB18241	88.53	Extracellular
ACC_09454	Disintegrin and metalloproteinase domain-containing protein	desintegrins	GB16304	81.92	Cytoplasmic
ACC_09635	A disintegrin and metalloproteinase with thrombospondin motifs	desintegrins	GB10715	97.22	SignalPeptide
ACC_03433	Lipase member H	Dol m 1	GB11340	44.06	Cytoplasmic
ACC_03774	Pancreatic lipase-related protein	Dol m 1	GB13155	97.52	Cytoplasmic
ACC_09394	Pancreatic triacylglycerol lipase	Dol m 1	GB16669	93.64	Cytoplasmic
ACC_01061	major allergen Bla g	Per a 1	GB10225	99.52	SignalPeptide
ACC_01062	Protein G12	Per a 1	GB14784	34.26	Extracellular
ACC_01063	Protein G12	Per a 1	GB13966	95.44	Extracellular
ACC_01064	Protein G12	Per a 1	GB12716	93.93	SignalPeptide
ACC_01065	Protein G12	Per a 1	GB14426	82.87	Extracellular
ACC_04406	Protein G12	Per a 1	GB14784	83.1	SignalPeptide
ACC_00856	Arylphorin subunit alpha	Per a 3	GB15552	96.76	Extracellular
ACC_02413	Hemocyanin C chain	Per a 3	GB18313	34.25	Cytoplasmic
ACC_03623	Phenoloxidase subunit A3	Per a 3	GB18313	96.45	Cytoplasmic
ACC_05357	Hexamerin	Per a 3	GB14361	93.87	Extracellular
ACC_07564	Hexamerin	Per a 3	GB10869	93.08	Extracellular
ACC_09901	Hexamerin	Per a 3	GB13613	95.34	Extracellular
ACC_05230	Pancreatic triacylglycerol lipase	Pol a 1	GB11855	95.95	Transmembrane
ACC_08565	Pancreatic triacylglycerol lipase	Pol a 1	GB11340	90.18	Cytoplasmic
ACC_08884	Pancreatic lipase-related protein	Pol a 1	GB11945	91.01	Extracellular
ACC_10167	Pancreatic triacylglycerol lipase	Pol a 1	GB17220	69.67	Cytoplasmic
ACC_10168	Lipase member H-B	Pol a 1	GB17220	86.02	Cytoplasmic
ACC_08541	Venom allergen	Pol f 5	GB17780	96.09	Cytoplasmic
ACC_03236	Cysteine-rich secretory protein	Pol g 5	GB19688	97.82	Extracellular
ACC_05677	Venom allergen	Sol i 3	GB13007	100	Cytoplasmic
ACC_00382	Phospholipase A1	Ves v 1	GB20032	32.29	Extracellular
ACC_01546	Exosome component	Ves v 1	GB14820	88.99	Cytoplasmic
ACC_03440	Endothelial lipase	Ves v 1	GB14821	97.3	Cytoplasmic
ACC_03583	Pancreatic lipase-related protein	Ves v 1	GB12400	100	Extracellular
ACC_05852	Phospholipase A1 member A	Ves v 1	GB17774	97.39	Cytoplasmic
ACC_07641	Lipase member H-A	Ves v 1	GB19835	96.05	Cytoplasmic
ACC_07915	Phospholipase A1 member A	Ves v 1	GB13246	98.31	Cytoplasmic
ACC_08013	Lipase member H-A	Ves v 1	GB19835	97.17	Cytoplasmic

ACC_09800	pancreatic triacylglycerol lipase-like	Ves v 1	GB15123	96.62	SignalPeptide
ACC_00031	Snake venom vascular endothelial growth factor toxin		GB12643	80.13	Cytoplasmic
ACC_00402	prostatic acid phosphatase-like		GB15862	94.43	SignalPeptide
ACC_00748	lysosomal acid phosphatase-like isoform 1		GB18728	99.13	Cytoplasmic
ACC_01020	acid phosphatase-like protein 2-like		GB12908	99.04	Transmembrane
ACC_01210	Calcium-independent phospholipase A2-gamma		GB13366	98.05	Cytoplasmic
ACC_01228	Phospholipase A2 homolog		GB12505	99.2	Cytoplasmic
ACC_01274	85 kDa calcium-independent phospholipase		GB19826	99.87	Cytoplasmic
ACC_02434	Golgi-associated plant pathogenesis-related protein		GB12915	98.01	Cytoplasmic
ACC_02458	venom acid phosphatase Acph-1-like		GB15544	93.39	Cytoplasmic
ACC_02613	iron/zinc purple acid phosphatase-like protein-like		GB13071	97.41	Cytoplasmic
ACC_02836	Vascular endothelial growth factor B		GB12643	93.52	Cytoplasmic
ACC_02873	Phospholipase A-2-activating protein		GB14172	99.74	Cytoplasmic
ACC_06261	Golgi-associated plant pathogenesis-related protein		GB14341	99.35	Cytoplasmic
ACC_06786	venom acid phosphatase Acph-1-like		GB10161	68.16	SignalPeptide
ACC_07014	Phospholipase A-2-activating protein		GB14172	99.74	Cytoplasmic
ACC_08333	venom acid phosphatase Acph-1-like		GB12546	31.78	Cytoplasmic
ACC_08969	venom acid phosphatase Acph-1-like		GB12132	91.71	Cytoplasmic
ACC_08986	venom acid phosphatase Acph-1-like		GB19394	90.08	Extracellular
ACC_10024	Apamin(Preproapamin)		GB18161	93.48	SignalPeptide

Supplementary Table 14. Codeml test for position selection of allergen genes in *A.cerana*

Apitoxin	n/Model	M1: Nearly neutral			-lnL	M2:Positive selection				-lnL	M0:ω	-2ln(test-null)*	P
Api m 1	5/K80+G	p:	0.53165	0.46835	1870.66	p:	0.5317	0.32244	0.1459	1870.66	0.2293	0	1
		ω:	0.06738	1		ω:	0.0674	1	1				
Api m 2	7/K80+G	p:	0.73008	0.26992	5407.29	p:	0.7301	0.18551	0.0844	5407.29	0.13394	0	1
		ω:	0.06196	1		ω:	0.062	1	1				
Api m 3	6/K80+G	p:	0.70869	0.29131	4825.49	p:	0.7087	0.20381	0.0875	4825.49	0.19652	0	1
		ω:	0.11001	1		ω:	0.11	1	1				
Api m 4	6/K80+G	p:	0.69414	0.30586	402.93	p:	0.7577	0	0.2423	402.61	0.25495	0.64	0.72
		ω:	0	1		ω:	0	1	1.7483				
Api m 5	7/K80+G	p:	0.00001	0.99999	10253.47	p:	0.1478	0.2264	0.6258	10140.33	2.22572	226.28	0
		ω:	1	1		ω:	1	1	3.2038				

Supplementary Table 15 Immune System of A.cerana

**Pathway**	**Gene**	**KO**	**Product**
ko04062 Chemokine signaling pathway	ACC_05516	K03099	SOS; son of sevenless
	ACC_06514	K08045	ADCY5; adenylate cyclase 5 [EC:4.6.1.1]
	ACC_03579	K05704	SRC; tyrosine-protein kinase Src [EC:2.7.10.2]
	ACC_05659	K04371	ERK1_2; extracellular signal-regulated kinase 1/2 [EC:2.7.11.24]
	ACC_06422	K05725	PTK2; focal adhesion kinase 1 [EC:2.7.10.2]
	ACC_01188	K05760	PXN; paxillin
	ACC_08589	K04513	RHOA; Ras homolog gene family, member A
	ACC_06468	K04547	GNG13; guanine nucleotide-binding protein G(I)/G(S)/G(O) subunit gamma-13
	ACC_02507	K04364	GRB2; growth factor receptor-binding protein 2
	ACC_00796	K08042	ADCY2; adenylate cyclase 2 [EC:4.6.1.1]
	ACC_05588	K00910	ADRBK; beta-adrenergic-receptor kinase [EC:2.7.11.15]
	ACC_09596	K00922	PIK3C; phosphatidylinositol-4,5-bisphosphate 3-kinase [EC:2.7.1.153]
	ACC_03570	K04353	RAP1A; Ras-related protein Rap-1A
	ACC_05279	K04456	AKT; RAC serine/threonine-protein kinase [EC:2.7.11.1]
	ACC_02576	K04514	ROCK; Rho-associated protein kinase [EC:2.7.11.1]
	ACC_02724	K05730	VAV; guanine nucleotide exchange factor VAV
	ACC_04189	K05747	WAS; Wiskott-Aldrich syndrome protein
	ACC_05965	K06279	SHC; src homology 2 domain-containing transforming protein C
	ACC_00659	K07209	IKBKB; inhibitor of nuclear factor kappa-B kinase subunit beta [EC:2.7.11.10]
	ACC_02813	K07827	KRAS; GTPase KRas
	ACC_01531	K08048	ADCY8; adenylate cyclase 8 [EC:4.6.1.1]
	ACC_05166	K12366	ELMO1; engulfment and cell motility protein 1
	ACC_05617	K04439	ARRB; beta-arrestin
	ACC_02140	K05731	TIAM1; T-lymphoma invasion and metastasis-inducing protein 1
	ACC_04262	K04539	GNB5; guanine nucleotide-binding protein subunit beta-5
	ACC_09212	K04536	GNB1; guanine nucleotide-binding protein G(I)/G(S)/G(T) subunit beta-1
	ACC_00311	K08049	ADCY9; adenylate cyclase 9 [EC:4.6.1.1]
	ACC_06459	K03083	GSK3B; glycogen synthase kinase 3 beta [EC:2.7.11.26]
	ACC_06850	K04630	GNAI; guanine nucleotide-binding protein G(i) subunit alpha
	ACC_03341	K04438	CRK; proto-oncogene C-crk
	ACC_04513	K04447	JAK2; Janus kinase 2 [EC:2.7.10.2]
	ACC_08442	K04409	PAK1; p21-activated kinase 1 [EC:2.7.11.1]
	ACC_08503,ACC_(	K04368	MAP2K1; mitogen-activated protein kinase kinase 1 [EC:2.7.12.2]
	ACC_04314,ACC_(	K02649	PIK3R; phosphoinositide-3-kinase, regulatory subunit
	ACC_02640,ACC_(	K08043	ADCY3; adenylate cyclase 3 [EC:4.6.1.1]
	ACC_01700,ACC_(	K04392	RAC1; Ras-related C3 botulinum toxin substrate 1
	ACC_01946,ACC_(	K04393	CDC42; cell division control protein 42
	ACC_02939,ACC_(	K05858	PLCB; phosphatidylinositol phospholipase C, beta [EC:3.1.4.11]
	ACC_04702,ACC_(	K04345	PKA; protein kinase A [EC:2.7.11.11]
ko04612 Antigen processing and presentation	ACC_05976	K08059	IFI30; interferon, gamma-inducible protein 30
	ACC_05391	K08065	NFYB; nuclear transcription Y subunit beta
	ACC_09287	K08066	NFYC; nuclear transcription factor Y, gamma
	ACC_09608	K08064	NFYA; nuclear transcription factor Y, alpha
	ACC_03714	K06698	PSME3; proteasome activator subunit 3 (PA28 gamma)
	ACC_00698	K08056	PDIA3; protein disulfide isomerase family A, member 3 [EC:5.3.4.1]
	ACC_03257	K08057	CALR; calreticulin
	ACC_04937	K08054	CANX; calnexin
	ACC_04912	K01365	CTSL; cathepsin L [EC:3.4.22.15]
	ACC_01484,ACC_(	K04079	htpG; molecular chaperone HtpG
	ACC_03611,ACC_(	K03283	HSPA1_8; heat shock 70kDa protein 1/8
ko04620 Toll-like receptor signaling pathway	ACC_06165	K03175	TRAF6; TNF receptor-associated factor 6

	ACC_05659	K04371	ERK1_2; extracellular signal-regulated kinase 1/2 [EC:2.7.11.24]
	ACC_06441	K04403	MAP3K7IP1; TAK1-binding protein 1
	ACC_02138	K04448	JUN; transcription factor AP-1
	ACC_01623	K05402	TOLLIP; toll-interacting protein
	ACC_09256	K04427	MAP3K7; mitogen-activated protein kinase kinase kinase 7 [EC:2.7.11.25]
	ACC_09596	K00922	PIK3C; phosphatidylinositol-4,5-bisphosphate 3-kinase [EC:2.7.1.153]
	ACC_03561	K04430	MAP2K4; mitogen-activated protein kinase kinase 4 [EC:2.7.12.2]
	ACC_04978	K04432	MAP2K3; mitogen-activated protein kinase kinase 3 [EC:2.7.12.2]
	ACC_05279	K04456	AKT; RAC serine/threonine-protein kinase [EC:2.7.11.1]
	ACC_02175	K04729	MYD88; myeloid differentiation primary response protein MyD88
	ACC_04832	K05410	TBK1; TANK-binding kinase 1 [EC:2.7.11.10]
	ACC_00659	K07209	IKBKB; inhibitor of nuclear factor kappa-B kinase subunit beta [EC:2.7.11.10]
	ACC_06215	K04440	JNK; c-Jun N-terminal kinase [EC:2.7.11.24]
	ACC_04876	K04431	MAP2K7; mitogen-activated protein kinase kinase 7 [EC:2.7.12.2]
	ACC_00669	K04441	P38; p38 MAP kinase [EC:2.7.11.24]
	ACC_08503,ACC_(	K04368	MAP2K1; mitogen-activated protein kinase kinase 1 [EC:2.7.12.2]
	ACC_04314,ACC_(	K02649	PIK3R; phosphoinositide-3-kinase, regulatory subunit
	ACC_01700,ACC_(	K04392	RAC1; Ras-related C3 botulinum toxin substrate 1
ko04621 NOD-like receptor signaling pathway	ACC_02636	K12796	ERBB2IP; erbb2-interacting protein
	ACC_06165	K03175	TRAF6; TNF receptor-associated factor 6
	ACC_05659	K04371	ERK1_2; extracellular signal-regulated kinase 1/2 [EC:2.7.11.24]
	ACC_06441	K04403	MAP3K7IP1; TAK1-binding protein 1
	ACC_01135	K16060	BIRC2_3; baculoviral IAP repeat-containing protein 2/3
	ACC_09256	K04427	MAP3K7; mitogen-activated protein kinase kinase kinase 7 [EC:2.7.11.25]
	ACC_01769	K12795	SUGT1; suppressor of G2 allele of SKP1
	ACC_00659	K07209	IKBKB; inhibitor of nuclear factor kappa-B kinase subunit beta [EC:2.7.11.10]
	ACC_06367	K09487	HSP90B; heat shock protein 90kDa beta
	ACC_06215	K04440	JNK; c-Jun N-terminal kinase [EC:2.7.11.24]
	ACC_00669	K04441	P38; p38 MAP kinase [EC:2.7.11.24]
	ACC_01484,ACC_(	K04079	htpG; molecular chaperone HtpG
ko04622 RIG-I-like receptor signaling pathway	ACC_06165	K03175	TRAF6; TNF receptor-associated factor 6
	ACC_06017	K12655	OTUD5; OTU domain-containing protein 5 [EC:3.1.2.15]
	ACC_02429	K00863	E2.7.1.29; dihydroxyacetone kinase [EC:2.7.1.29]
	ACC_09256	K04427	MAP3K7; mitogen-activated protein kinase kinase kinase 7 [EC:2.7.11.25]
	ACC_04832	K05410	TBK1; TANK-binding kinase 1 [EC:2.7.11.10]
	ACC_00659	K07209	IKBKB; inhibitor of nuclear factor kappa-B kinase subunit beta [EC:2.7.11.10]
	ACC_02731	K08339	ATG5; autophagy-related protein 5
	ACC_07906	K08601	CYLD; ubiquitin thioesterase CYLD [EC:3.1.2.15]
	ACC_03702	K09578	PIN1; peptidyl-prolyl cis-trans isomerase NIMA-interacting 1 [EC:5.2.1.8]
	ACC_01600	K08336	ATG12; autophagy-related protein 12
	ACC_06215	K04440	JNK; c-Jun N-terminal kinase [EC:2.7.11.24]
	ACC_00669	K04441	P38; p38 MAP kinase [EC:2.7.11.24]
	ACC_00817,ACC_(	K11594	DDX3X; ATP-dependent RNA helicase [EC:3.6.4.13]
ko04623 Cytosolic DNA-sensing pathway	ACC_02164	K03019	RPC11; DNA-directed RNA polymerase III subunit RPC10
	ACC_03303	K03027	RPC40; DNA-directed RNA polymerases I and III subunit RPAC1
	ACC_00414	K03026	RPC53; DNA-directed RNA polymerase III subunit RPC4
	ACC_01620	K03020	RPC19; DNA-directed RNA polymerases I and III subunit RPAC2
	ACC_05435	K03007	RPB10; DNA-directed RNA polymerases I, II, and III subunit RPABC5

	ACC_03335	K03013	RPB5; DNA-directed RNA polymerases I, II, and III subunit RPABC1
	ACC_08787	K03016	RPB8; DNA-directed RNA polymerases I, II, and III subunit RPABC3
	ACC_07934	K03018	RPC1; DNA-directed RNA polymerase III subunit RPC1 [EC:2.7.7.6]
	ACC_00426	K03021	RPC2; DNA-directed RNA polymerase III subunit RPC2 [EC:2.7.7.6]
	ACC_01036	K03023	RPC82; DNA-directed RNA polymerase III subunit RPC3
	ACC_00697	K03024	RPC31; DNA-directed RNA polymerase III subunit RPC7
	ACC_03884	K03025	RPC34; DNA-directed RNA polymerase III subunit RPC6
	ACC_04832	K05410	TBK1; TANK-binding kinase 1 [EC:2.7.11.10]
	ACC_00659	K07209	IKBKB; inhibitor of nuclear factor kappa-B kinase subunit beta [EC:2.7.11.10]
	ACC_08118	K14721	RPC37; DNA-directed RNA polymerase III subunit RPC5
	ACC_03373	K03014	RPB6; DNA-directed RNA polymerases I, II, and III subunit RPABC2
	ACC_05392	K03022	RPC25; DNA-directed RNA polymerase III subunit RPC8
ko04650 Natural killer cell mediated cytotoxicity	ACC_05516	K03099	SOS; son of sevenless
	ACC_05659	K04371	ERK1_2; extracellular signal-regulated kinase 1/2 [EC:2.7.11.24]
	ACC_06822	K07293	PTPN11; tyrosine-protein phosphatase non-receptor type 11 [EC:3.1.3.48]
	ACC_03620	K01116	PLCG1; phosphatidylinositol phospholipase C, gamma-1 [EC:3.1.4.11]
	ACC_09321	K04446	NFATC; nuclear factor of activated T-cells, cytoplasmic
	ACC_02507	K04364	GRB2; growth factor receptor-binding protein 2
	ACC_09596	K00922	PIK3C; phosphatidylinositol-4,5-bisphosphate 3-kinase [EC:2.7.1.153]
	ACC_02724	K05730	VAV; guanine nucleotide exchange factor VAV
	ACC_05965	K06279	SHC; src homology 2 domain-containing transforming protein C
	ACC_02813	K07827	KRAS; GTPase KRas
	ACC_02607	K02677	CPKC; classical protein kinase C [EC:2.7.11.13]
	ACC_08442	K04409	PAK1; p21-activated kinase 1 [EC:2.7.11.1]
	ACC_08503,ACC_(	K04368	MAP2K1; mitogen-activated protein kinase kinase 1 [EC:2.7.12.2]
	ACC_04314,ACC_(	K02649	PIK3R; phosphoinositide-3-kinase, regulatory subunit
	ACC_05684,ACC_(	K04348	PPP3C; protein phosphatase 3, catalytic subunit [EC:3.1.3.16]
	ACC_01700,ACC_(	K04392	RAC1; Ras-related C3 botulinum toxin substrate 1
	ACC_05321,ACC_(	K06268	PPP3R; protein phosphatase 3, regulatory subunit
ko04660 T cell receptor signaling pathway	ACC_05516	K03099	SOS; son of sevenless
	ACC_06314	K07369	MALT1; mucosa-associated lymphoid tissue lymphoma translocation protein 1 [EC:3.4.22.-]
	ACC_05659	K04371	ERK1_2; extracellular signal-regulated kinase 1/2 [EC:2.7.11.24]
	ACC_03620	K01116	PLCG1; phosphatidylinositol phospholipase C, gamma-1 [EC:3.1.4.11]
	ACC_02138	K04448	JUN; transcription factor AP-1
	ACC_08589	K04513	RHOA; Ras homolog gene family, member A
	ACC_00510	K07364	TEC; tyrosine-protein kinase Tec [EC:2.7.10.2]
	ACC_09256	K04427	MAP3K7; mitogen-activated protein kinase kinase kinase 7 [EC:2.7.11.25]
	ACC_09321	K04446	NFATC; nuclear factor of activated T-cells, cytoplasmic
	ACC_02507	K04364	GRB2; growth factor receptor-binding protein 2
	ACC_09596	K00922	PIK3C; phosphatidylinositol-4,5-bisphosphate 3-kinase [EC:2.7.1.153]
	ACC_06045	K02089	CDK4; cyclin-dependent kinase 4 [EC:2.7.11.22]
	ACC_05279	K04456	AKT; RAC serine/threonine-protein kinase [EC:2.7.11.1]
	ACC_02724	K05730	VAV; guanine nucleotide exchange factor VAV
	ACC_00659	K07209	IKBKB; inhibitor of nuclear factor kappa-B kinase subunit beta [EC:2.7.11.10]
	ACC_02813	K07827	KRAS; GTPase KRas
	ACC_08623	K12076	DLG1; disks large protein 1
	ACC_06459	K03083	GSK3B; glycogen synthase kinase 3 beta [EC:2.7.11.26]

	ACC_04876	K04431	MAP2K7; mitogen-activated protein kinase kinase 7 [EC:2.7.12.2]
	ACC_02669	K07365	NCK; NCK adaptor protein
	ACC_08442	K04409	PAK1; p21-activated kinase 1 [EC:2.7.11.1]
	ACC_00669	K04441	P38; p38 MAP kinase [EC:2.7.11.24]
	ACC_08503,ACC_(	K04368	MAP2K1; mitogen-activated protein kinase kinase 1 [EC:2.7.12.2]
	ACC_04314,ACC_(	K02649	PIK3R; phosphoinositide-3-kinase, regulatory subunit
	ACC_03970,ACC_(	K04707	CBL; E3 ubiquitin-protein ligase CBL [EC:6.3.2.19]
	ACC_05684,ACC_(	K04348	PPP3C; protein phosphatase 3, catalytic subunit [EC:3.1.3.16]
	ACC_05321,ACC_(	K06268	PPP3R; protein phosphatase 3, regulatory subunit
	ACC_01946,ACC_(	K04393	CDC42; cell division control protein 42
ko04662 B cell receptor signaling pathway	ACC_08596	K12230	PIK3AP1; phosphoinositide 3-kinase adapter protein 1
	ACC_05516	K03099	SOS; son of sevenless
	ACC_06314	K07369	MALT1; mucosa-associated lymphoid tissue lymphoma translocation protein 1 [EC:3.4.22.-]
	ACC_05659	K04371	ERK1_2; extracellular signal-regulated kinase 1/2 [EC:2.7.11.24]
	ACC_02138	K04448	JUN; transcription factor AP-1
	ACC_09321	K04446	NFATC; nuclear factor of activated T-cells, cytoplasmic
	ACC_02507	K04364	GRB2; growth factor receptor-binding protein 2
	ACC_09596	K00922	PIK3C; phosphatidylinositol-4,5-bisphosphate 3-kinase [EC:2.7.1.153]
	ACC_05279	K04456	AKT; RAC serine/threonine-protein kinase [EC:2.7.11.1]
	ACC_02724	K05730	VAV; guanine nucleotide exchange factor VAV
	ACC_00659	K07209	IKBKB; inhibitor of nuclear factor kappa-B kinase subunit beta [EC:2.7.11.10]
	ACC_02813	K07827	KRAS; GTPase KRas
	ACC_06459	K03083	GSK3B; glycogen synthase kinase 3 beta [EC:2.7.11.26]
	ACC_08503,ACC_(	K04368	MAP2K1; mitogen-activated protein kinase kinase 1 [EC:2.7.12.2]
	ACC_04314,ACC_(	K02649	PIK3R; phosphoinositide-3-kinase, regulatory subunit
	ACC_05684,ACC_(	K04348	PPP3C; protein phosphatase 3, catalytic subunit [EC:3.1.3.16]
	ACC_01700,ACC_(	K04392	RAC1; Ras-related C3 botulinum toxin substrate 1
	ACC_05321,ACC_(	K06268	PPP3R; protein phosphatase 3, regulatory subunit
ko04664 Fc epsilon RI signaling pathway	ACC_05516	K03099	SOS; son of sevenless
	ACC_05659	K04371	ERK1_2; extracellular signal-regulated kinase 1/2 [EC:2.7.11.24]
	ACC_03620	K01116	PLCG1; phosphatidylinositol phospholipase C, gamma-1 [EC:3.1.4.11]
	ACC_02507	K04364	GRB2; growth factor receptor-binding protein 2
	ACC_09596	K00922	PIK3C; phosphatidylinositol-4,5-bisphosphate 3-kinase [EC:2.7.1.153]
	ACC_03561	K04430	MAP2K4; mitogen-activated protein kinase kinase 4 [EC:2.7.12.2]
	ACC_04978	K04432	MAP2K3; mitogen-activated protein kinase kinase 3 [EC:2.7.12.2]
	ACC_05279	K04456	AKT; RAC serine/threonine-protein kinase [EC:2.7.11.1]
	ACC_02724	K05730	VAV; guanine nucleotide exchange factor VAV
	ACC_02813	K07827	KRAS; GTPase KRas
	ACC_06215	K04440	JNK; c-Jun N-terminal kinase [EC:2.7.11.24]
	ACC_04876	K04431	MAP2K7; mitogen-activated protein kinase kinase 7 [EC:2.7.12.2]
	ACC_00669	K04441	P38; p38 MAP kinase [EC:2.7.11.24]
	ACC_08503,ACC_(	K04368	MAP2K1; mitogen-activated protein kinase kinase 1 [EC:2.7.12.2]
	ACC_04314,ACC_(	K02649	PIK3R; phosphoinositide-3-kinase, regulatory subunit
	ACC_01700,ACC_(	K04392	RAC1; Ras-related C3 botulinum toxin substrate 1
ko04666 Fc gamma R-mediated phagocytosis	ACC_05659	K04371	ERK1_2; extracellular signal-regulated kinase 1/2 [EC:2.7.11.24]
	ACC_07187	K05754	ARPC5; actin related protein 2/3 complex, subunit 5
	ACC_08280	K05743	LIMK1; LIM domain kinase 1 [EC:2.7.11.1]
	ACC_03620	K01116	PLCG1; phosphatidylinositol phospholipase C, gamma-1 [EC:3.1.4.11]
	ACC_09274	K05755	ARPC4; actin related protein 2/3 complex, subunit 4
	ACC_02617	K07941	ARF6; ADP-ribosylation factor 6

	ACC_09596	K00922	PIK3C; phosphatidylinositol-4,5-bisphosphate 3-kinase [EC:2.7.1.153]
	ACC_05279	K04456	AKT; RAC serine/threonine-protein kinase [EC:2.7.11.1]
	ACC_02724	K05730	VAV; guanine nucleotide exchange factor VAV
	ACC_04189	K05747	WAS; Wiskott-Aldrich syndrome protein
	ACC_01746	K05757	ARPC1A_B; actin related protein 2/3 complex, subunit 1A/1B
	ACC_00828	K05758	ARPC2; actin related protein 2/3 complex, subunit 2
	ACC_02844	K05765	CFL; cofilin
	ACC_05870	K04718	E2.7.1.91; sphingosine kinase [EC:2.7.1.91]
	ACC_01365	K12488	ASAP; Arf-GAP with SH3 domain, ANK repeat and PH domain-containing protein
	ACC_02607	K02677	CPKC; classical protein kinase C [EC:2.7.11.13]
	ACC_08843	K05756	ARPC3; actin related protein 2/3 complex, subunit 3
	ACC_04037	K00889	E2.7.1.68; 1-phosphatidylinositol-4-phosphate 5-kinase [EC:2.7.1.68]
	ACC_03341	K04438	CRK; proto-oncogene C-crk
	ACC_08442	K04409	PAK1; p21-activated kinase 1 [EC:2.7.11.1]
	ACC_05868	K01115	PLD1_2; phospholipase D1/2 [EC:3.1.4.4]
	ACC_08503,ACC_(	K04368	MAP2K1; mitogen-activated protein kinase kinase 1 [EC:2.7.12.2]
	ACC_04314,ACC_(	K02649	PIK3R; phosphoinositide-3-kinase, regulatory subunit
	ACC_01700,ACC_(	K04392	RAC1; Ras-related C3 botulinum toxin substrate 1
	ACC_01430,ACC_(	K04688	RPS6KB; p70 ribosomal S6 kinase [EC:2.7.11.1]
	ACC_01946,ACC_(	K04393	CDC42; cell division control protein 42
	ACC_03981,ACC_(	K01528	DNM; dynamin GTPase [EC:3.6.5.5]
	ACC_06916,ACC_(	K01080	PPAP2; phosphatidate phosphatase [EC:3.1.3.4]
ko04670 Leukocyte transendothelial migration			
	ACC_01871	K05699	ACTN; actinin alpha
	ACC_06422	K05725	PTK2; focal adhesion kinase 1 [EC:2.7.10.2]
	ACC_06822	K07293	PTPN11; tyrosine-protein phosphatase non-receptor type 11 [EC:3.1.3.48]
	ACC_01188	K05760	PXN; paxillin
	ACC_03620	K01116	PLCG1; phosphatidylinositol phospholipase C, gamma-1 [EC:3.1.4.11]
	ACC_00682	K05691	CTNNA; catenin alpha
	ACC_08589	K04513	RHOA; Ras homolog gene family, member A
	ACC_09596	K00922	PIK3C; phosphatidylinositol-4,5-bisphosphate 3-kinase [EC:2.7.1.153]
	ACC_03570	K04353	RAP1A; Ras-related protein Rap-1A
	ACC_02576	K04514	ROCK; Rho-associated protein kinase [EC:2.7.11.1]
	ACC_02724	K05730	VAV; guanine nucleotide exchange factor VAV
	ACC_01595	K02105	CTNNB1; catenin beta 1
	ACC_01677	K05702	AF6; afadin
	ACC_02607	K02677	CPKC; classical protein kinase C [EC:2.7.11.13]
	ACC_06850	K04630	GNAI; guanine nucleotide-binding protein G(i) subunit alpha
	ACC_08318	K05719	ITGB1; integrin beta 1
	ACC_00669	K04441	P38; p38 MAP kinase [EC:2.7.11.24]
	ACC_04314,ACC_(	K02649	PIK3R; phosphoinositide-3-kinase, regulatory subunit
	ACC_01700,ACC_(	K04392	RAC1; Ras-related C3 botulinum toxin substrate 1
	ACC_01946,ACC_(	K04393	CDC42; cell division control protein 42
	ACC_04920,ACC_(	K05692	ACTB_G1; actin beta/gamma 1

Supplementary Table 16 G Protein-Coupled Receptors in A.cerana

Class A. Rhodopsin family	Gene	KO	Product
**Biogenic amine**	Acetylcholine (muscarinic)		
	ACC_06927	K04131	muscarinic acetylcholine receptor M3
	ACC_06681	K04134	muscarinic acetylcholine receptor
	Dopamine		
	ACC_07797	K04148	dopamine D1-like receptor
	ACC_09005	K14049	dopamine D2-like receptor
	Serotonin		
	ACC_08581	K04153	5-hydroxytryptamine receptor 1
	ACC_09424	K04157	5-hydroxytryptamine receptor 2
	ACC_06642	K04163	5-hydroxytryptamine receptor 7
	Octopamine		
	ACC_08088	K04165	Octopamine receptor
**Neuropeptide**	Tachykinin		
	ACC_04644	K04225	tachykinin-like receptor
	ACC_08659	K04225	tachykinin-like receptor
	ACC_09684	K14071	leucokinin receptor
	ACC_07076	K14072	neuropeptide Y receptor
	Galanin		
	ACC_10047	K04233	allatostatin receptor
	Neuropeptide Y		
	ACC_00795	K04209	neuropeptide Y receptor, invertebrate
	Neuropeptide S		
	ACC_08311	K08376	neuropeptide S receptor 1
**Hypothalamic hormone**	Gonadotropin-releasing hormone		
	ACC_02676	K04280	gonadotropin-releasing hormone receptor
**Pancreatic peptide**	Relaxin		
	ACC_07605	K04307	leucine-rich repeat-containing G protein-coupled receptor 8
**Vision**	Opsin		
	ACC_02501	K04255	r-opsin
	ACC_02803	K04255	r-opsin
	ACC_03326	K04255	r-opsin
	ACC_08639	K04255	r-opsin
**Class B. Secretin receptor family**			
**Calcium-regulating peptide**	Calcitonin		
	ACC_06505	K04577	calcitonin receptor-like
**Hypothalamic hormone**	Corticotropin-releasing hormone		
	ACC_08643	K04578	corticotropin releasing hormone receptor 1
**Cell adhesion**	Cadherin EGF LAG (CELSR)		
	ACC_06752	K04600	cadherin EGF LAG seven-pass G-type receptor 1 (flamingo)
**Other**	Methuselah-like proteins (MTH)		
	ACC_00226	K04599	G protein-coupled receptor Mth (Methuselah protein)
**Class C. Metabotropic glutamate receptor family**			
**Biogenic amine**	Glutamate (metabotropic)		
	ACC_08905	K04605	metabotropic glutamate receptor 2/3
	ACC_08691	K04608	metabotropic glutamate receptor 6/7/8
	ACC_01549	K04611	metabotropic X receptor
	ACC_08403	K04611	metabotropic X receptor
	GABA		
	ACC_01515	K04615	gamma-aminobutyric acid type B receptor
	ACC_08078	K04615	gamma-aminobutyric acid type B receptor

	ACC_09743	K04615	gamma-aminobutyric acid type B receptor
**Others**			
**Chemoreception**	Insect odorant		
	ACC_01311	K08471	gustatory receptor
	ACC_02322	K08471	gustatory receptor
	ACC_06781	K08471	gustatory receptor
	ACC_08009	K08471	gustatory receptor
	ACC_08219	K08471	gustatory receptor
	ACC_08361	K08471	gustatory receptor
	ACC_10028	K08471	gustatory receptor
**Wnt signaling**	Frizzled / Smoothened family		
	ACC_00484	K02432	frizzled 1/7
	ACC_09882	K02375	frizzled 5/8
	ACC_05811	K06226	smoothened

Supplementary Table 17 Xenobiotic detoxifying genes in A.cerana

Gene	SwissProt	Nr	KO	KOG	Name
ACC_00300	Carboxylesterase	LOW QUALITY PROTEIN		KOG4389	CCE
ACC_01989	Esterase FE4	venom carboxylesterase-6-like		KOG1516	CCE
ACC_02285	Esterase FE4	venom carboxylesterase-6 precursor		KOG1516	CCE
ACC_02286	Esterase FE4	venom carboxylesterase-6-like		KOG1516	CCE
ACC_02612	Acetylcholinesterase	acetylcholinesterase	K01049	KOG4389	CCE
ACC_04101	Carboxylesterase	esterase E4-like		KOG1516	CCE
ACC_05066	Esterase FE4	venom carboxylesterase-6-like		KOG1516	CCE
ACC_07422	Acetylcholinesterase	acetylcholinesterase	K01049	KOG4389	CCE
ACC_09028	Carboxylesterase	esterase E4-like		KOG1516	CCE
ACC_09444	Esterase FE4	carboxylesterase		KOG1516	CCE
ACC_09804	Acetylcholinesterase	acetylcholinesterase	K01049	KOG4389	CCE
ACC_03338	Neurotactin	neurotactin		KOG1516	CCE
ACC_03348	Esterase E4	esterase A2		KOG1516	CCE
ACC_03349	Esterase E4	esterase E4-like, partial		KOG1516	CCE
ACC_04071	Neuroligin-4, Y-linked	neuroligin 5		KOG1516	CCE
ACC_04401	Acetylcholinesterase	acetylcholinesterase-like		KOG1516	CCE
ACC_06498	Esterase FE4	esterase FE4-like		KOG1516	CCE
ACC_07596	Neuroligin-3	hypothetical protein LOC724358		KOG4389	CCE
ACC_07865	Neuroligin-1	neuroligin-1		KOG4389	CCE
ACC_07889	Neuroligin-4, Y-linked	neuroligin-4, Y-linked		KOG4389	CCE
ACC_08121	Neuroligin-1	hypothetical protein LOC724358		KOG4389	CCE
ACC_08128	Esterase E4	esterase E4-like		KOG1516	CCE
ACC_08428	Neuroligin-1	neuroligin 5		KOG4389	CCE
ACC_08793	Neuroligin-4, Y-linked	juvenile hormone esterase-like, partial		KOG1516	CCE
ACC_09707	Neuroligin-3	neuroligin 4 precursor	K07378	KOG4389	CCE
ACC_09777	Neuroligin-1	neuroligin 3 precursor		KOG4389	CCE
ACC_09900	Esterase B1	esterase FE4-like		KOG1516	CCE
ACC_01408	Glutathione S-transferase	LOW QUALITY PROTEIN	K00799	KOG0867	GSTs
ACC_01763	Glutathione S-transferase ome	glutathione S-transferase omega-1-like		KOG0406	GSTs
ACC_02021	Glutathione S-transferase thet	glutathione S-transferase theta-1-like	K00799	KOG0867	GSTs
ACC_03148	Microsomal glutathione S-tran	microsomal glutathione S-transferase 1 isof	K00799		GSTs
ACC_03799	Glutathione S-transferase	glutathione S-transferase-like isoform 1		KOG1695	GSTs
ACC_03800	Glutathione S-transferase	glutathione S-transferase S1		KOG1695	GSTs
ACC_04205	Glutathione S-transferase ome	glutathione S-transferase omega-1	K00799	KOG0406	GSTs
ACC_04756	Glutathione S-transferase	glutathione S-transferase S4		KOG1695	GSTs
ACC_04982	Glutathione S-transferase	glutathione S-transferase-like	K01830	KOG1695	GSTs
ACC_08158	Glutathione S-transferase C-te	glutathione S-transferase C-terminal domain-containi		KOG2811	GSTs
ACC_09116	Glutathione S-transferase	glutathione S-transferase D1	K00799	KOG0867	GSTs
ACC_09117	Glutathione S-transferase	glutathione S-transferase 1-1-like		KOG0867	GSTs
ACC_00312	Cytochrome P450 4C1	cytochrome P450 4C1, partial		KOG0157	P450
ACC_00317	Cytochrome P450 4C1	cytochrome P450 4C1, partial		KOG0157	P450
ACC_00383	Cytochrome P450	cytochrome P450 9e2	K15003	KOG0158	P450
ACC_00385	Cytochrome P450	cytochrome P450 9e2-like	K15003	KOG0158	P450
ACC_00983	Probable cytochrome P450 6a	cytochrome P450 6k1-like	K07424	KOG0158	P450
ACC_00984	Cytochrome P450 6j1	probable cytochrome P450 6a13		KOG0158	P450
ACC_01585	Probable cytochrome P450 30	probable cytochrome P450 305a1		KOG0156	P450
ACC_01649	Cytochrome P450 6B1	probable cytochrome P450 6a14		KOG0158	P450
ACC_01735	Probable cytochrome P450 30	probable cytochrome P450 305a1	K14937	KOG0156	P450
ACC_01812	Probable cytochrome P450 30	LOW QUALITY PROTEIN		KOG0156	P450
ACC_02036	Probable cytochrome P450 30	probable cytochrome P450 301a1, mitochondrial		KOG0159	P450
ACC_02143	Probable cytochrome P450	probable cytochrome P450 12a5, mitochondrial-like		KOG0159	P450
ACC_02303	Cytochrome P450 315a1, mito	cytochrome P450 315a1, mitochondrial isof	K10722	KOG0159	P450

ACC_03022	Cytochrome P450 4c3	cytochrome P450 4c3		KOG0157	P450
ACC_03792	Probable cytochrome P450 30	probable cytochrome P450 304a1		KOG0156	P450
ACC_04304	Cytochrome P450 6a2	LOW QUALITY PROTEIN		KOG0158	P450
ACC_04305	Probable cytochrome P450 6a	probable cytochrome P450 6a14		KOG0158	P450
ACC_04306	Probable cytochrome P450 6a	probable cytochrome P450 6a13		KOG0158	P450
ACC_04307	Cytochrome P450 6A1	cytochrome P450 6A1		KOG0158	P450
ACC_04308	Cytochrome P450 6a2	cytochrome P450 6AS5	K07424	KOG0158	P450
ACC_04309	Probable cytochrome P450 6a	probable cytochrome P450 6a14-like		KOG0158	P450
ACC_05376	Probable cytochrome P450 4a	LOW QUALITY PROTEIN	K15001	KOG0157	P450
ACC_05562	Cytochrome P450 4g15	cytochrome P450 4G11	K15001	KOG0157	P450
ACC_05897		Cytochrome P450 4C1			P450
ACC_06707	Cytochrome P450	cytochrome P450 9e2		KOG0158	P450
ACC_06708	Cytochrome P450	cytochrome P450 9e2		KOG0158	P450
ACC_06711	Cytochrome P450	cytochrome P450 9e2-like		KOG0158	P450
ACC_06737	Cytochrome P450 18a1	cytochrome P450 18a1	K14985	KOG0156	P450
ACC_06770	Cytochrome P450 302a1, mitc	cytochrome P450 302a1, mitochondrial		KOG0159	P450
ACC_07300	Probable cytochrome P450 6a	probable cytochrome P450 6a14		KOG0158	P450
ACC_07562	NADPH--cytochrome P450 red	NADPH--cytochrome P450 reductase-like	K00327	KOG1158	P450
ACC_07563	Ras-related protein Rab-34	NADPH--cytochrome P450 reductase-like	K07921	KOG0094	P450
ACC_07777	Probable cytochrome P450 30	probable cytochrome P450 301a1, mitochondrial		KOG0159	P450
ACC_07812	Cytochrome P450 6a2	probable cytochrome P450 6a14 isoform 1		KOG0158	P450
ACC_08166	Cytochrome P450	cytochrome P450 9e2		KOG0158	P450
ACC_08279	Cytochrome P450	cytochrome P450 9e2 isoform 4		KOG0158	P450
ACC_08522	Cytochrome P450 6B1	probable cytochrome P450 6a14		KOG0158	P450
ACC_08774	Cytochrome P450 6k1	cytochrome P450 6k1		KOG0158	P450
ACC_08924	Probable cytochrome P450 30	probable cytochrome P450 305a1		KOG0156	P450
ACC_08967	Cytochrome P450 6k1	cytochrome P450 6k1-like		KOG0158	P450
ACC_09176	Probable cytochrome P450 49	probable cytochrome P450 49a1		KOG0159	P450
ACC_09198	Probable cytochrome P450 6a	probable cytochrome P450 6a14-like		KOG0158	P450
ACC_09199	Probable cytochrome P450 6a	probable cytochrome P450 6a14 isoform 1	K15002	KOG0158	P450
ACC_09200	Cytochrome P450 6A1	cytochrome P450 6A1		KOG0158	P450
ACC_09201	Cytochrome P450 6a8	probable cytochrome P450 6a14		KOG0158	P450
ACC_09213	Cytochrome P450 307a1	cytochrome P450 307a1	K14939	KOG0156	P450
ACC_09229	Cytochrome P450	cytochrome P450 9e2		KOG0158	P450
ACC_09377	Cytochrome P450 306a1	cytochrome P450 306a1	K10720	KOG0156	P450
ACC_09438	Cytochrome P450 6j1	cytochrome P450 6j1-like isoform 1		KOG0158	P450
ACC_09640	Probable cytochrome P450 6a	probable cytochrome P450 6a14-like		KOG0158	P450
ACC_09789	Cytochrome P450 6A1	LOW QUALITY PROTEIN		KOG0158	P450
ACC_09927	Cytochrome P450 302a1, mitc	cytochrome P450 302a1, mitochondrial	K10721	KOG0159	P450

Supplementary Table 18 Genes encoding antimicrobial peptide in A.cerana

				RPM			
**Gene**	**SwissProt**	**Nr**	**KOG**	**New emerged**	**Nurse**	**Guarder**	**Forager**
ACC_00645	Apidaecins type	apidaecins type 22 precursor	KOG0566	8.38	30.82	238.90	29.27
ACC_03450	Hymenoptaecin	hymenoptaecin		0.82	2.50	27.40	3.54
ACC_03585	Hymenoptaecin	hymenoptaecin		3.27	3.66	55.30	12.30
ACC_04356	Royalisin	defensin1 precursor		800.81	3072.95	1225.12	267.87
ACC_05494		apisimin		7986.28	172877.61	207656.16	18508.78
ACC_07626	Abaecin	abaecin		0.20	1.73	11.96	13.05
ACC_08301	Defensin	defensin2		0.41	1.35	3.74	25.54

Supplementary Table 19. Significantly up/down regulated genes in

		RPM		clear-grooming vs control				
Gene	Product	clear-grooming bees	control	log2 (Fold change) normalized	p-value	FDR	Regulation	GO Category
ACC_00031	Snake venom vascular endothelial growth factor toxin	10.78	26.15	-1.2785853	1.24E-05	0.00010852	Down	
ACC_00048	Actin-related protein	21.87	9.10	1.26603828	7.30E-05	0.00055519	Up	
ACC_00061	Uncharacterized protein C14orf50	19.97	5.31	1.91240132	3.08E-07	3.48E-06	Up	
ACC_00078	RNA-binding protein	220.02	98.16	1.16445988	5.19E-32	3.79E-30	Up	
ACC_00092	Rho GTPase-activating protein	34.24	14.02	1.28791046	4.93E-07	5.42E-06	Up	
ACC_00105	Dimethyladenosine transferase 2, mitochondrial	11.73	2.65	2.14457476	2.39E-05	0.00019812	Up	
ACC_00121	Class A basic helix-loop-helix protein	76.09	28.42	1.42054822	6.43E-16	1.87E-14	Up	
ACC_00124	Nucleolar pre-ribosomal-associated protein	39.95	18.19	1.13479374	8.89E-07	9.43E-06	Up	
ACC_00126	Mitochondrial ribonuclease P protein	39.95	11.75	1.76555993	7.14E-12	1.41E-10	Up	
ACC_00135	Golgi apparatus protein	29.48	13.26	1.15235211	1.91E-05	0.0001618	Up	
ACC_00139	Zinc finger CCHC domain-containing protein	109.69	50.40	1.12182211	7.06E-16	2.04E-14	Up	
ACC_00177	YEATS domain-containing protein	93.21	45.86	1.02328543	5.13E-12	1.02E-10	Up	
ACC_00178	RNA methyltransferase-like protein	25.36	10.23	1.30951691	1.16E-05	0.00010215	Up	
ACC_00217	GRIP and coiled-coil domain-containing protein	66.89	31.08	1.1060235	4.67E-10	7.47E-09	Up	
ACC_00227	Adipocyte plasma membrane-associated protein	94.47	34.49	1.4538502	5.06E-20	1.98E-18	Up	
ACC_00228	Beta-mannosidase	76.40	31.83	1.26304823	1.37E-13	3.22E-12	Up	
ACC_00244	NK-tumor recognition protein	305.61	141.36	1.11233384	8.10E-41	7.97E-39	Up	
ACC_00278	hypothetical protein LOC100577894	32.02	12.89	1.31322496	7.93E-07	8.48E-06	Up	
ACC_00296	Uncharacterized protein C3orf19 homolog	87.82	36.38	1.27125598	1.55E-15	4.31E-14	Up	
ACC_00304	Putative rRNA methyltransferase	168.66	77.69	1.11827866	1.85E-23	8.77E-22	Up	
ACC_00324	DnaJ homolog subfamily C member	87.82	38.66	1.18379314	4.79E-14	1.17E-12	Up	
ACC_00339	Probable ATP-dependent RNA helicase CG8611	54.85	23.50	1.22290824	9.84E-10	1.52E-08	Up	
ACC_00355		45.02	17.81	1.33763459	2.89E-09	4.18E-08	Up	
ACC_00374	Leucine-rich repeat-containing protein	49.77	15.54	1.67954506	1.49E-13	3.47E-12	Up	
ACC_00375	Nucleosomal histone kinase	42.80	16.30	1.39302716	2.35E-09	3.46E-08	Up	
ACC_00394	BAG domain-containing protein Samui	271.06	126.20	1.10287856	6.38E-36	5.50E-34	Up	
ACC_00399	Probable cation-transporting ATPase	38.04	18.95	1.00551073	1.39E-05	0.00012001	Up	
ACC_00416	Sorting nexin-17	32.34	15.54	1.05734966	2.97E-05	0.00024387	Up	
ACC_00430	Uncharacterized protein KIAA1704	198.46	87.17	1.18700512	6.91E-30	4.53E-28	Up	
ACC_00434	hypothetical protein LOC100577102	206.70	89.06	1.21468753	3.76E-32	2.79E-30	Up	
ACC_00441	Prostaglandin E synthase	40.90	14.40	1.50577606	6.10E-10	9.66E-09	Up	
ACC_00451	AMY-1-associating protein expressed in testis	213.99	102.70	1.05907097	5.48E-27	3.20E-25	Up	
ACC_00462	Death domain-associated protein	102.08	44.34	1.20302848	1.85E-16	5.60E-15	Up	
ACC_00499	Uncharacterized protein C7orf50 homolog	106.20	30.70	1.7906436	1.52E-29	9.84E-28	Up	
ACC_00501	Lysophospholipid acyltransferase	25.68	10.23	1.32743882	8.27E-06	7.53E-05	Up	

ACC_00504	Uncharacterized protein KIAA0406 homolog	27.26	9.47	1.52488488	3.41E-07	3.82E-06	Up	
ACC_00506	AP-1 complex subunit sigma-2	19.97	7.58	1.39782815	4.31E-05	0.00034358	Up	response to stress
ACC_00529	Coiled-coil domain-containing protein	51.99	14.78	1.81462611	1.59E-15	4.40E-14	Up	
ACC_00546	hypothetical protein LOC726379 isoform 1	114.76	50.40	1.18703977	5.84E-18	1.95E-16	Up	
ACC_00557	N(6)-adenine-specific DNA methyltransferase	8.88	1.14	2.96486874	1.04E-05	9.30E-05	Up	
ACC_00563	Protein FAM161A	43.75	20.46	1.09611327	5.69E-07	6.20E-06	Up	
ACC_00603	Condensin-2 complex subunit D3	37.73	15.54	1.27974208	1.50E-07	1.78E-06	Up	
ACC_00623	General transcription factor 3C polypeptide	93.52	41.31	1.17886314	8.98E-15	2.34E-13	Up	
ACC_00624	Sine oculis-binding protein homolog	13.00	3.03	2.10002832	1.14E-05	0.0001003	Up	
ACC_00625	Golgin-45	25.68	9.85	1.3818866	4.28E-06	4.10E-05	Up	
ACC_00631	SAP domain-containing ribonucleoprotein	31.07	15.16	1.03525807	5.75E-05	0.00044611	Up	
ACC_00634	Bifunctional aminoacyl-tRNA synthetase	471.42	178.88	1.3980222	6.65E-88	2.32E-85	Up	
ACC_00636	tRNA-splicing endonuclease subunit Sen54	24.73	7.96	1.63556112	3.22E-07	3.63E-06	Up	
ACC_00639	Nitrilase and fragile histidine triad fusion protein NitFhit	22.51	9.10	1.30726094	3.71E-05	0.00029984	Up	
ACC_00644	uncharacterized protein LOC100865162	38.99	10.61	1.8776359	1.62E-12	3.38E-11	Up	
ACC_00645	Apidaecins type	42.16	108.77	-1.3671482	6.29E-21	2.62E-19	Down	
ACC_00657	Fanconi anemia group D2 protein	71.33	29.18	1.28947097	3.72E-13	8.27E-12	Up	
ACC_00683	Nucleolar complex protein	30.75	7.20	2.09446165	1.59E-11	3.01E-10	Up	
ACC_00719	Leukocyte receptor cluster member	44.70	21.60	1.04913766	1.07E-06	1.11E-05	Up	
ACC_00722	Protein SDA1 homolog	195.29	84.13	1.21484699	1.79E-30	1.23E-28	Up	
ACC_00724	hypothetical protein LOC100578271	39.95	12.89	1.6322934	8.85E-11	1.54E-09	Up	
ACC_00731	Uncharacterized protein KIAA1467 homolog	294.83	106.11	1.47428021	3.88E-60	7.47E-58	Up	
ACC_00785	La protein homolog	44.07	21.22	1.05406247	1.16E-06	1.20E-05	Up	
ACC_00786	Ribosomal RNA-processing protein	95.74	43.20	1.14799104	1.59E-14	4.08E-13	Up	
ACC_00825	Nuclear nucleic acid-binding protein C1D	32.34	10.99	1.55692067	1.71E-08	2.29E-07	Up	
ACC_00864	TRAF-interacting protein	32.65	10.99	1.57099585	1.17E-08	1.60E-07	Up	
ACC_00890	Transcription elongation factor SPT4	42.48	20.09	1.08064506	1.10E-06	1.15E-05	Up	
ACC_00896	inner nuclear membrane protein Man1-like	35.19	14.40	1.28896467	3.37E-07	3.78E-06	Up	
ACC_00903		19.97	3.79	2.39782815	3.64E-09	5.22E-08	Up	
ACC_00910	Uncharacterized protein CG1785	196.56	85.65	1.19842176	4.81E-30	3.23E-28	Up	
ACC_00914	Polycomb complex protein BMI-1	416.89	204.27	1.02918194	1.04E-48	1.47E-46	Up	
ACC_00953	Myosin class II heavy chain	64.67	26.15	1.3063772	2.78E-12	5.66E-11	Up	
ACC_00956	Coiled-coil domain-containing protein	29.48	14.02	1.07218177	5.51E-05	0.00042916	Up	
ACC_00988	Protein FAM114A2	118.57	52.68	1.17042971	3.97E-18	1.36E-16	Up	
ACC_01050	Structural maintenance of chromosome protein 4 (chromosome condensation complex Condensin, subunit C)	18.39	6.82	1.43053231	6.51E-05	0.00049909	Up	
ACC_01052	hypothetical protein LOC100652119	6.97	0.00	5.20190794	3.28E-06	3.21E-05	Up	
ACC_01075	Uncharacterized protein C9orf114 homolog	35.51	12.13	1.54983124	3.88E-09	5.54E-08	Up	
ACC_01088	Intraflagellar transport protein	215.58	101.57	1.08577807	3.12E-28	1.95E-26	Up	
ACC_01090	DNA2-like helicase	32.97	13.26	1.31363302	5.41E-07	5.92E-06	Up	
ACC_01127	Dysbindin-A	44.07	17.43	1.33785544	4.22E-09	5.99E-08	Up	
ACC_01132	Protein xmas-2	72.92	25.01	1.54357225	3.95E-17	1.26E-15	Up	

ACC_01133	Protein SON	498.36	237.24	1.07084297	1.22E-61	2.45E-59	Up	
ACC_01152	DNA polymerase beta	53.26	20.46	1.37990624	3.74E-11	6.82E-10	Up	response to stress
ACC_01180	hypothetical protein LOC100578298	54.53	20.09	1.44082062	4.70E-12	9.40E-11	Up	
ACC_01196	Outer dense fiber protein	79.57	37.52	1.08466325	2.26E-11	4.22E-10	Up	
ACC_01210	Calcium-independent phospholipase A2-gamma	21.56	7.58	1.50801107	6.88E-06	6.34E-05	Up	
ACC_01214	ATP-dependent RNA helicase DDX24	57.06	25.77	1.14686657	3.14E-09	4.52E-08	Up	
ACC_01224	Coiled-coil domain-containing protein	47.24	19.33	1.2892195	3.39E-09	4.88E-08	Up	
ACC_01225	28S ribosomal protein S27, mitochondrial	91.94	43.58	1.07689536	8.86E-13	1.90E-11	Up	
ACC_01246	RRP15-like protein	69.43	30.70	1.17741338	2.50E-11	4.65E-10	Up	
ACC_01249	ATP-dependent RNA helicase DDX54	143.93	68.60	1.06917892	6.08E-19	2.27E-17	Up	
ACC_01300	Sodium-coupled monocarboxylate transporter	56.11	28.04	1.0006285	1.46E-07	1.74E-06	Up	
ACC_01309	Exocyst complex component	16.80	6.06	1.47039677	9.75E-05	0.00072614	Up	
ACC_01319	Ubiquitin-related modifier	49.46	20.09	1.29995808	1.14E-09	1.76E-08	Up	
ACC_01388	Uncharacterized protein C1orf26 homolog	95.74	42.45	1.17352614	5.45E-15	1.45E-13	Up	
ACC_01392	Protein FAM60A	26.31	7.96	1.72519833	4.39E-08	5.57E-07	Up	
ACC_01418	Double-strand break repair protein MRE11	35.19	14.02	1.32743882	1.80E-07	2.12E-06	Up	response to stress
ACC_01420	MRG-binding protein	27.90	12.13	1.20190794	1.71E-05	0.00014497	Up	
ACC_01421	Zinc finger CCHC domain-containing protein	22.19	9.47	1.22790315	9.61E-05	0.00071687	Up	
ACC_01437	hypothetical protein LOC100648343	55.80	25.39	1.13581875	6.14E-09	8.62E-08	Up	
ACC_01442	Protein asteroid	17.75	4.55	1.96486874	8.97E-07	9.49E-06	Up	
ACC_01459	Uncharacterized ATP-dependent helicase C29A10.10c	54.53	26.53	1.03945806	8.92E-08	1.08E-06	Up	response to stress
ACC_01489	Probable enoyl-CoA hydratase, mitochondrial	23.46	7.58	1.63000159	6.87E-07	7.41E-06	Up	
ACC_01490	DNA-dependent protein kinase catalytic subunit	68.79	29.18	1.23724101	4.90E-12	9.77E-11	Up	response to stress
ACC_01504	Endonuclease III-like protein	21.87	8.34	1.39156916	1.99E-05	0.00016757	Up	response to stress
ACC_01505	Ubiquitin carboxyl-terminal hydrolase	17.12	5.68	1.59047323	3.09E-05	0.0002526	Up	
ACC_01537	NADH dehydrogenase [ubiquinone] iron-sulfur protein mitochondrial	82.11	30.70	1.4194346	4.87E-17	1.53E-15	Up	
ACC_01545	Something about silencing protein	78.31	39.03	1.00434302	4.68E-10	7.47E-09	Up	
ACC_01546	Exosome component	187.68	92.85	1.01529175	2.22E-22	1.00E-20	Up	
ACC_01560	UPF0399 protein v1g245966	67.21	31.83	1.07807935	9.58E-10	1.49E-08	Up	
ACC_01561	Transmembrane protein C3orf1 homolog	39.31	19.33	1.02424729	7.27E-06	6.67E-05	Up	
ACC_01567	hypothetical protein LOC100576886	27.26	12.51	1.12434695	5.57E-05	0.00043367	Up	
ACC_01569	Enolase-phosphatase E1	36.77	17.05	1.10860422	3.70E-06	3.58E-05	Up	
ACC_01588	Transcription factor containing C2HC type Zn finger	403.26	198.96	1.01918566	1.89E-46	2.44E-44	Up	
ACC_01599	Nibrin	21.56	7.58	1.50801107	6.88E-06	6.34E-05	Up	
ACC_01602	Gamma-tubulin complex component	57.06	25.77	1.14686657	3.14E-09	4.52E-08	Up	
ACC_01606	Transcription regulator XNP/ATRX, DEAD-box superfamily	294.83	112.18	1.39410986	1.95E-55	3.27E-53	Up	
ACC_01622	Bifunctional dihydrofolate reductase-thymidylate synthase	65.94	26.53	1.31363302	1.37E-12	2.89E-11	Up	

ACC_01643	Zinc transporter foi	16.49	4.55	1.85795354	5.10E-06	4.84E-05	Up	
ACC_01668	Transmembrane and coiled-coil domains protein	35.82	8.72	2.03909333	8.67E-13	1.86E-11	Up	
ACC_01699	Uncharacterized protein C6orf203 homolog	19.97	4.55	2.13479374	3.85E-08	4.93E-07	Up	
ACC_01712	Poly(A) RNA polymerase, mitochondrial	21.24	6.06	1.80856551	3.73E-07	4.15E-06	Up	
ACC_01720	hypothetical protein LOC724168	18.07	6.06	1.57536633	2.12E-05	0.00017745	Up	
ACC_01787	Bipolar kinesin KRP-130	12.05	3.41	1.82047883	0.00012068	0.000891	Up	
ACC_01845	Ribonuclease H1	30.43	13.26	1.1981558	7.46E-06	6.84E-05	Up	
ACC_01856	Zinc finger protein DZIP1L	37.41	7.20	2.37719186	9.48E-16	2.69E-14	Up	
ACC_01872	E3 ubiquitin-protein ligase UHRF2	52.94	21.22	1.31882569	1.90E-10	3.14E-09	Up	
ACC_01893	Transcription regulator XNP/ATRX, DEAD-box superfamily	148.68	62.15	1.25836843	7.64E-25	3.82E-23	Up	
ACC_01895	Tumor suppressor p53-binding protein	116.67	46.61	1.32352377	2.52E-21	1.08E-19	Up	
ACC_01909	hypothetical protein LOC724842	17.44	2.27	2.93887353	7.67E-10	1.20E-08	Up	
ACC_01942	Cyclic AMP-dependent transcription factor ATF-6 alpha	76.72	36.76	1.06142671	1.10E-10	1.89E-09	Up	response to stress
ACC_01963	Ribosome production factor	64.99	21.22	1.6146015	2.25E-16	6.79E-15	Up	
ACC_01967	Ras-related protein Rab-8A	31.39	9.47	1.72797675	2.16E-09	3.20E-08	Up	
ACC_01971	Periodic tryptophan protein	25.68	10.61	1.2749714	1.55E-05	0.00013286	Up	
ACC_01980	Prefoldin subunit	34.24	5.31	2.6900089	1.75E-16	5.34E-15	Up	
ACC_02020	Coiled-coil domain-containing protein	41.85	14.78	1.50146822	4.21E-10	6.79E-09	Up	
ACC_02028	E3 ubiquitin-protein ligase RNF14	18.07	6.06	1.57536633	2.12E-05	0.00017745	Up	
ACC_02092	Chromosome-associated kinesin KIF4A	26.31	10.23	1.36262825	4.14E-06	3.98E-05	Up	
ACC_02099	Protein FAM32A	167.07	76.55	1.12592399	1.71E-23	8.16E-22	Up	
ACC_02125	Bifunctional coenzyme A synthase	80.84	28.04	1.52737639	1.47E-18	5.27E-17	Up	
ACC_02151	Transmembrane protein	363.94	176.23	1.04629634	7.19E-44	8.23E-42	Up	
ACC_02183	Zinc finger protein	71.96	26.53	1.43974179	2.04E-15	5.60E-14	Up	
ACC_02188	Regulator of telomere elongation helicase	19.02	5.68	1.74247632	2.77E-06	2.73E-05	Up	response to stress
ACC_02208		44.38	17.81	1.31717048	5.67E-09	7.97E-08	Up	
ACC_02229	Mesencephalic astrocyte-derived neurotrophic factor homolog	56.75	21.98	1.3683111	1.18E-11	2.26E-10	Up	
ACC_02236	Radial spoke head protein	22.19	7.20	1.62383182	1.47E-06	1.50E-05	Up	
ACC_02247	Programmed cell death protein	252.99	109.15	1.21279625	7.05E-39	6.51E-37	Up	
ACC_02252	Leucine-rich repeat-containing protein	24.09	9.10	1.40544133	6.46E-06	5.99E-05	Up	
ACC_02256	Zinc finger protein	47.24	21.22	1.15428992	6.03E-08	7.56E-07	Up	
ACC_02271	Telomere length regulation protein TEL2 homolog	28.85	13.26	1.12098794	3.54E-05	0.00028668	Up	
ACC_02272	Blastoderm-specific protein 25D	38.68	15.54	1.31566165	5.51E-08	6.92E-07	Up	
ACC_02334	High affinity cAMP-specific and IBMX-insensitive 3',5'-cyclic phosphodiesterase	2066.05	959.58	1.10640691	3.68E-263	2.78E-260	Up	
ACC_02353	Uncharacterized protein C9orf82 homolog	61.82	26.15	1.24128218	5.18E-11	9.29E-10	Up	
ACC_02435	39S ribosomal protein L46, mitochondrial	89.40	36.38	1.29706517	3.06E-16	9.10E-15	Up	
ACC_02439	Protein odr-4 homolog	37.41	17.81	1.07053052	5.70E-06	5.34E-05	Up	
ACC_02456	hypothetical protein LOC551416	94.79	40.55	1.22501101	8.54E-16	2.43E-14	Up	

ACC_02465	Nucleolar protein of	49.14	23.88	1.0413208	3.70E-07	4.13E-06	Up	
ACC_02493	uncharacterized protein LOC100871978	69.11	19.33	1.8382353	1.86E-20	7.44E-19	Up	
ACC_02528	Enhancer of polycomb homolog	17.44	4.93	1.82339631	3.65E-06	3.54E-05	Up	regulation of gene expression, epigenetic
ACC_02531	60S ribosomal protein L22	254.57	109.15	1.2218075	1.50E-39	1.43E-37	Up	
ACC_02534	uncharacterized protein LOC100872300	125.86	53.82	1.2257244	1.76E-20	7.12E-19	Up	
ACC_02538	ATP-dependent DNA helicase Q5	37.09	14.78	1.32743882	8.41E-08	1.03E-06	Up	
ACC_02543	Dihydrofolate reductase	19.66	5.31	1.88931771	4.75E-07	5.24E-06	Up	
ACC_02554	Neuroguidin-A	34.56	16.30	1.08439589	1.05E-05	9.36E-05	Up	
ACC_02579	DnaJ homolog subfamily C member	111.59	36.76	1.6019951	1.08E-26	6.24E-25	Up	
ACC_02586	Condensin complex subunit	76.09	28.04	1.43991355	3.25E-16	9.62E-15	Up	
ACC_02589	Fas-binding factor	66.89	23.88	1.48629558	4.59E-15	1.22E-13	Up	
ACC_02596	Cytochrome c oxidase assembly protein COX16 homolog, mitochondrial	24.73	5.68	2.12098794	1.11E-09	1.72E-08	Up	
ACC_02667	UBX domain-containing protein	106.84	50.03	1.09468698	5.82E-15	1.53E-13	Up	
ACC_02675	Protein Daple	110.01	53.06	1.05198516	1.72E-14	4.34E-13	Up	
ACC_02678	Vacuolar protein sorting-associated protein	54.85	26.15	1.06858009	4.12E-08	5.25E-07	Up	
ACC_02686	Lymphoid-specific helicase	28.53	13.26	1.1050464	4.79E-05	0.00037693	Up	regulation of gene expression, epigenetic
ACC_02701	tRNA-splicing endonuclease subunit Sen15	19.02	4.93	1.9489272	4.23E-07	4.68E-06	Up	
ACC_02765	hypothetical protein LOC100577958	48.19	11.37	2.08351324	4.15E-17	1.32E-15	Up	
ACC_02768	Serum response factor-binding protein	45.33	14.40	1.65442014	3.04E-12	6.16E-11	Up	
ACC_02805	tRNA (guanine-N(1)-)-methyltransferase	87.50	18.19	2.26603828	7.36E-33	5.70E-31	Up	
ACC_02807	RNA-binding protein NOB1	42.80	12.89	1.73182908	2.55E-12	5.23E-11	Up	
ACC_02810	Golgin subfamily A member	339.85	158.03	1.10466194	1.03E-44	1.23E-42	Up	
ACC_02843	WD repeat-containing protein CG11141	29.48	13.64	1.11171013	3.28E-05	0.00026755	Up	
ACC_02860	Probable ATP-dependent RNA helicase kurz	257.74	89.06	1.53307092	7.80E-56	1.36E-53	Up	
ACC_02935	E3 ubiquitin-protein ligase RNF8	52.63	20.09	1.3895953	3.86E-11	7.01E-10	Up	response to stress
ACC_02960	Probable ATP-dependent RNA helicase DDX28	15.85	3.03	2.38633251	1.60E-07	1.89E-06	Up	
ACC_02979	Heme oxygenase	13.95	3.79	1.87997984	2.35E-05	0.0001955	Up	response to stress
ACC_03006		294.20	125.44	1.22976991	6.94E-46	8.60E-44	Up	
ACC_03028	Uncharacterized protein C18orf8 homolog	28.53	13.64	1.06440441	8.02E-05	0.00060483	Up	
ACC_03038	PKHD domain-containing transmembrane protein C17orf101 homolog	68.79	31.08	1.14647555	7.87E-11	1.38E-09	Up	
ACC_03106	Glyoxalase domain-containing protein	53.58	25.77	1.05589291	7.91E-08	9.73E-07	Up	
ACC_03141	Probable 28S ribosomal protein S26, mitochondrial	33.92	11.37	1.57705271	5.55E-09	7.83E-08	Up	
ACC_03149	Pheromone-binding protein-related protein	19.97	57.23	-1.5186485	1.76E-13	4.09E-12	Down	
ACC_03166	DNA polymerase subunit gamma-1, mitochondrial	38.99	15.54	1.32743882	3.94E-08	5.02E-07	Up	
ACC_03173	hypothetical protein LOC409221	81.79	37.90	1.10984739	4.91E-12	9.78E-11	Up	
ACC_03174	Neuroblastoma-amplified sequence	32.65	9.47	1.78512066	4.24E-10	6.82E-09	Up	

ACC_03176	6-pyruvoyl tetrahydrobiopterin synthase	18.70	6.82	1.45519437	4.51E-05	0.00035633	Up	
ACC_03177	RNA pseudouridylate synthase domain-containing protein	11.73	1.89	2.63000159	1.90E-06	1.92E-05	Up	
ACC_03219	G protein pathway suppressor	32.97	16.30	1.01665128	4.45E-05	0.00035182	Up	response to stress
ACC_03289	Molybdenum cofactor sulfurase	39.63	18.95	1.06440441	3.35E-06	3.27E-05	Up	
ACC_03293	CDK5RAP1-like protein	48.82	22.36	1.12661981	6.59E-08	8.20E-07	Up	
ACC_03319	Origin recognition complex subunit	24.09	9.10	1.40544133	6.46E-06	5.99E-05	Up	
ACC_03322	Mimitin, mitochondrial	45.97	20.84	1.1410257	1.19E-07	1.42E-06	Up	
ACC_03324	Metal transporter CNNM2	78.94	33.73	1.22674482	1.97E-13	4.51E-12	Up	
ACC_03328	Methionyl-tRNA synthetase, mitochondrial	155.66	66.70	1.22262392	7.40E-25	3.72E-23	Up	
ACC_03329	Probable small nuclear ribonucleoprotein G	24.41	9.47	1.36540667	8.93E-06	8.09E-05	Up	
ACC_03333	Pentatricopeptide repeat-containing protein	52.31	21.98	1.25081754	1.23E-09	1.89E-08	Up	
ACC_03385	Heterogeneous nuclear ribonucleoprotein L	58.02	23.50	1.30397985	3.86E-11	7.00E-10	Up	
ACC_03408	Translation initiation factor IF-2, mitochondrial	122.69	58.74	1.06254167	3.17E-16	9.40E-15	Up	
ACC_03430	28S ribosomal protein S31, mitochondrial	60.55	17.05	1.82805205	5.41E-18	1.81E-16	Up	
ACC_03442	TRAF-interacting protein	73.55	20.46	1.84556981	8.87E-22	3.90E-20	Up	
ACC_03443	Zinc finger MYND domain-containing protein	43.75	20.46	1.09611327	5.69E-07	6.20E-06	Up	
ACC_03450	Hymenoptaecin	9.19	31.83	-1.7918601	6.32E-10	9.98E-09	Down	
ACC_03478	NF-kappa-B essential modulator	169.93	75.80	1.16470932	4.16E-25	2.12E-23	Up	
ACC_03491	uncharacterized protein LOC100865970	53.26	18.19	1.54983124	5.48E-13	1.20E-11	Up	
ACC_03493	Enoyl-CoA hydratase domain-containing protein	13.00	3.79	1.77810023	8.52E-05	0.00063914	Up	
ACC_03497	Jerky protein homolog-like	6.66	0.76	3.13479374	8.88E-05	0.00066605	Up	
ACC_03502	Gem-associated protein	22.51	7.20	1.64429593	9.88E-07	1.04E-05	Up	
ACC_03511	DNA ligase	14.90	3.41	2.12714017	2.16E-06	2.16E-05	Up	response to stress
ACC_03521	Breast cancer type 2 susceptibility protein homolog	47.24	21.98	1.10366384	1.73E-07	2.04E-06	Up	response to stress
ACC_03559	Trans-1,2-dihydrobenzene-1,2-diol dehydrogenase	33.92	12.13	1.48394331	2.48E-08	3.22E-07	Up	
ACC_03585	Hymenoptaecin	31.39	73.90	-1.2354974	8.54E-13	1.84E-11	Down	
ACC_03597	Serologically defined colon cancer antigen	136.64	63.67	1.10170296	6.95E-19	2.58E-17	Up	
ACC_03606	ATP synthase subunit e, mitochondrial	63.72	26.15	1.28500355	7.46E-12	1.46E-10	Up	
ACC_03656	Centrosomal protein of	58.02	20.84	1.47681644	3.74E-13	8.30E-12	Up	
ACC_03657	E3 ubiquitin-protein ligase RNF168	62.14	25.39	1.29109697	1.14E-11	2.19E-10	Up	response to stress
ACC_03673	HAUS augmin-like complex subunit	13.95	4.17	1.74247632	5.98E-05	0.00046111	Up	
ACC_03684	Caspase-8	18.70	4.17	2.16568775	7.95E-08	9.76E-07	Up	
ACC_03705	Intraflagellar transport protein	74.50	35.25	1.07983446	1.13E-10	1.94E-09	Up	
ACC_03733	Protein ccsmst1	28.85	4.93	2.54983124	2.07E-13	4.73E-12	Up	
ACC_03736	M-phase phosphoprotein	17.44	6.06	1.52383603	4.58E-05	0.00036125	Up	
ACC_03744	E3 ubiquitin-protein ligase RAD18	15.22	4.55	1.74247632	2.76E-05	0.00022728	Up	response to stress
ACC_03750	TBC1 domain family member	54.85	25.77	1.08964171	2.50E-08	3.25E-07	Up	
ACC_03808	hypothetical protein LOC100578805	7.93	0.76	3.38633251	9.77E-06	8.77E-05	Up	
ACC_03813	Replication factor C subunit	101.13	48.51	1.05988893	1.37E-13	3.22E-12	Up	
ACC_03825	WD40 repeat-containing protein	8.24	1.52	2.44291604	0.00012749	0.00093594	Up	
ACC_03843	39S ribosomal protein L40, mitochondrial	102.40	47.75	1.10058675	1.63E-14	4.14E-13	Up	

ACC_03852	Spindle assembly abnormal protein	21.24	8.72	1.28500355	7.69E-05	0.000583	Up	
ACC_03895	Plastin-3	136.00	60.26	1.1744272	1.15E-20	4.66E-19	Up	
ACC_03921	Translational activator GCN1	66.58	29.56	1.17131962	7.62E-11	1.34E-09	Up	
ACC_03927	Protein LLP homolog	35.51	16.30	1.12356649	4.29E-06	4.11E-05	Up	
ACC_03955	Dehydrodolichyl diphosphate synthase	18.39	4.55	2.01549481	3.69E-07	4.13E-06	Up	
ACC_04001	DNA repair protein RAD50	165.80	62.15	1.41559145	1.21E-32	9.23E-31	Up	response to stress
ACC_04002	hypothetical protein LOC100647857	63.72	9.47	2.74967182	5.93E-30	3.95E-28	Up	
ACC_04012	39 kDa FK506-binding nuclear protein	136.95	61.39	1.15751382	2.32E-20	9.18E-19	Up	
ACC_04016	Protein downstream neighbor of son homolog	11.41	2.27	2.32743882	1.18E-05	0.00010317	Up	
ACC_04029	non-structural maintenance of chromosome element 4-like isoform 2	92.25	41.69	1.14599195	5.14E-14	1.25E-12	Up	
ACC_04034	SET and MYND domain-containing protein	47.24	10.61	2.15428992	1.85E-17	6.06E-16	Up	
ACC_04039	DNA repair protein XRCC1	34.87	11.37	1.61694544	1.76E-09	2.64E-08	Up	response to stress
ACC_04052	Uncharacterized protein C16orf48	26.00	7.96	1.7077109	6.57E-08	8.18E-07	Up	
ACC_04107	Zinc finger protein	144.88	59.88	1.27474593	1.04E-24	5.13E-23	Up	
ACC_04115	DNA (cytosine-5)-methyltransferase PliMCI	27.90	11.37	1.29501734	5.14E-06	4.87E-05	Up	regulation of gene expression, epigenetic
ACC_04135	hypothetical protein LOC100748542	162.32	68.97	1.23468168	3.15E-26	1.76E-24	Up	
ACC_04223	Werner syndrome ATP-dependent helicase	11.73	1.89	2.63000159	1.90E-06	1.92E-05	Up	
ACC_04232	Coiled-coil domain-containing protein	61.19	27.67	1.1451088	8.95E-10	1.39E-08	Up	
ACC_04238		110.96	49.27	1.17131962	4.40E-17	1.39E-15	Up	
ACC_04299	Vacuolar protein sorting-associated protein	97.01	45.10	1.1050464	6.53E-14	1.57E-12	Up	
ACC_04322	G patch domain-containing protein	69.43	26.15	1.40873892	1.71E-14	4.34E-13	Up	
ACC_04373	Cysteinyl-tRNA synthetase, cytoplasmic	46.60	20.84	1.16078895	6.45E-08	8.06E-07	Up	
ACC_04390	Adenylyltransferase and sulfurtransferase MOCS3	45.65	18.19	1.32743882	2.78E-09	4.03E-08	Up	
ACC_04392	rRNA-processing protein UTP23 homolog	129.98	60.64	1.10002832	5.33E-18	1.79E-16	Up	
ACC_04416	Protein GRINL1A	46.29	18.57	1.31759103	2.66E-09	3.88E-08	Up	
ACC_04419	39S ribosomal protein L23, mitochondrial	11.41	2.65	2.1050464	3.81E-05	0.00030651	Up	
ACC_04422	TAR DNA-binding protein	69.75	31.46	1.1487966	5.44E-11	9.72E-10	Up	
ACC_04434	Acylamino-acid-releasing enzyme	39.95	18.57	1.1050464	1.50E-06	1.54E-05	Up	
ACC_04438	Proton-coupled amino acid transporter	159.15	58.74	1.43789547	4.16E-32	3.06E-30	Up	
ACC_04454	Prolyl 3-hydroxylase	68.79	31.46	1.12898812	1.33E-10	2.26E-09	Up	
ACC_04461	Cleft lip and palate transmembrane protein	18.70	6.82	1.45519437	4.51E-05	0.00035633	Up	
ACC_04470	Zinc finger protein Xfin	395.96	195.93	1.01501361	2.39E-45	2.93E-43	Up	
ACC_04473	La-related protein	108.11	53.44	1.0165529	1.44E-13	3.36E-12	Up	
ACC_04476	E3 UFM1-protein ligase	52.31	26.15	1.00027408	3.89E-07	4.31E-06	Up	
ACC_04490	DNA polymerase epsilon subunit	46.60	22.36	1.05950562	5.13E-07	5.63E-06	Up	
ACC_04495	Protein SPT2 homolog	354.43	169.40	1.06504977	5.61E-44	6.51E-42	Up	
ACC_04528	V-type proton ATPase subunit E	67.21	31.83	1.07807935	9.58E-10	1.49E-08	Up	
ACC_04540	Probable ATP-dependent RNA helicase DDX27	72.28	35.25	1.03620752	8.20E-10	1.28E-08	Up	
ACC_04545	Protein MAK16 homolog A	91.62	43.20	1.08451199	7.19E-13	1.56E-11	Up	
ACC_04546	Translation initiation factor IF-3, chloroplastic	66.58	21.60	1.62383182	7.33E-17	2.27E-15	Up	

ACC_04549	Coiled-coil domain-containing protein	62.45	27.29	1.19460314	1.53E-10	2.57E-09	Up	
ACC_04557	Coiled-coil domain-containing protein	168.02	82.62	1.02414054	1.80E-20	7.25E-19	Up	
ACC_04559	Structural maintenance of chromosome protein 2 (chromosome condensation complex Condensin, subunit E)	41.21	20.09	1.03692368	3.49E-06	3.40E-05	Up	
ACC_04566	NudC domain-containing protein	16.17	3.03	2.41490166	9.81E-08	1.19E-06	Up	
ACC_04616	Retinoblastoma-binding protein	31.07	7.96	1.96486874	8.06E-11	1.41E-09	Up	
ACC_04618	Transmembrane protein	68.79	28.42	1.27520886	1.51E-12	3.16E-11	Up	
ACC_04630	Integrator complex subunit	18.07	6.44	1.48790349	4.56E-05	0.00036016	Up	
ACC_04641	Nucleolar protein	73.23	34.87	1.0706634	2.17E-10	3.57E-09	Up	
ACC_04646	Cytochrome b-c1 complex subunit	60.55	29.56	1.03450293	1.99E-08	2.63E-07	Up	
ACC_04667	Digestive organ expansion factor homolog	28.22	11.75	1.26401344	6.83E-06	6.30E-05	Up	
ACC_04690	Peptidylprolyl isomerase domain and WD repeat-containing protein	47.55	21.60	1.138405	7.61E-08	9.40E-07	Up	
ACC_04728	hypothetical protein LOC100742053 isoform 1	259.64	106.87	1.28064461	2.98E-43	3.29E-41	Up	
ACC_04749	Probable DNA mismatch repair protein Msh6	27.26	12.89	1.08127823	9.43E-05	0.00070519	Up	response to stress
ACC_04779	Ecto-NOX disulfide-thiol exchanger	56.43	25.01	1.17381563	1.95E-09	2.92E-08	Up	
ACC_04825	Putative RNA polymerase II subunit B1 CTD phosphatase RPAP2	44.38	13.26	1.74247632	8.08E-13	1.75E-11	Up	
ACC_04859	Nucleolar protein	19.02	6.82	1.47944191	3.11E-05	0.00025443	Up	
ACC_04878	Surfeit locus protein	175.63	75.42	1.21959387	9.82E-28	6.05E-26	Up	
ACC_04893		157.88	76.93	1.03714233	1.06E-19	4.09E-18	Up	
ACC_04894	hypothetical protein LOC409713	37.09	12.89	1.5253782	2.69E-09	3.92E-08	Up	
ACC_04897	uncharacterized protein LOC100867921	78.94	19.71	2.00203853	9.78E-26	5.21E-24	Up	
ACC_04922	10 kDa heat shock protein, mitochondrial	30.12	14.78	1.02692971	8.33E-05	0.0006272	Up	
ACC_04959	Probable dolichyl-P-Man:Man(7)GlcNAc(2)-PP-dolichyl-alpha-1,6-mannosyltransferase	53.58	20.84	1.36199604	5.11E-11	9.17E-10	Up	
ACC_04980	tRNA-splicing endonuclease subunit Sen34	38.36	18.19	1.07637706	3.94E-06	3.80E-05	Up	
ACC_04989	Uncharacterized protein C9orf85 homolog	20.61	2.27	3.17988163	3.92E-12	7.88E-11	Up	
ACC_05002	Heterogeneous nuclear ribonucleoprotein A1,	39.31	19.33	1.02424729	7.27E-06	6.67E-05	Up	
ACC_05007	UPF0609 protein CG1218	83.38	29.56	1.49599309	1.46E-18	5.24E-17	Up	
ACC_05016	Zinc finger CCCH domain-containing protein	95.11	33.73	1.49556158	6.06E-21	2.54E-19	Up	
ACC_05019	Uncharacterized protein YJR142W	58.65	27.67	1.08403322	9.51E-09	1.31E-07	Up	
ACC_05021	B-cell receptor-associated protein	82.43	33.73	1.2891107	5.79E-15	1.53E-13	Up	
ACC_05041	Lebercilin-like protein	29.80	10.99	1.43908418	3.28E-07	3.69E-06	Up	
ACC_05065	Kinetochore-associated protein	32.02	14.78	1.11528558	1.43E-05	0.00012345	Up	
ACC_05105	Centromere protein J	70.70	34.11	1.05152312	7.91E-10	1.24E-08	Up	
ACC_05113	NMDA receptor-regulated protein	45.02	20.84	1.11086373	2.92E-07	3.31E-06	Up	
ACC_05119	UPF0428 protein CXorf56 homolog	24.73	10.61	1.22052362	4.17E-05	0.00033401	Up	
ACC_05129	ATPase family AAA domain-containing protein	109.06	37.14	1.55403123	4.56E-25	2.31E-23	Up	
ACC_05138	ATP-dependent DNA helicase	25.68	7.58	1.76039823	4.14E-08	5.26E-07	Up	response to stress
ACC_05155	Spermatogenesis-associated protein	22.83	6.82	1.74247632	2.83E-07	3.22E-06	Up	
ACC_05167	RNA polymerase II-associated factor	73.87	34.11	1.11480937	4.44E-11	8.04E-10	Up	
ACC_05182	Protein tramtrack, alpha isoform	22.83	9.47	1.26854513	4.95E-05	0.00038908	Up	

ACC_05194	hypothetical protein LOC100576614	17.12	5.31	1.6900089	1.35E-05	0.00011704	Up	
ACC_05211	Microprocessor complex subunit DGCR8	21.24	8.72	1.28500355	7.69E-05	0.000583	Up	
ACC_05226	Zinc finger protein	82.11	40.93	1.00439711	1.78E-10	2.96E-09	Up	
ACC_05270	Bifunctional polynucleotide phosphatase/kinase	54.53	26.91	1.01899395	1.43E-07	1.71E-06	Up	
ACC_05278	Cleft lip and palate transmembrane protein	27.90	10.23	1.44702044	6.99E-07	7.54E-06	Up	
ACC_05319	Conserved oligomeric Golgi complex subunit	23.14	9.85	1.23186116	6.52E-05	0.00049937	Up	
ACC_05322	Serine/threonine-protein phosphatase 4 regulatory subunit	26.00	9.85	1.39958861	3.00E-06	2.95E-05	Up	
ACC_05369	Transcription factor A, mitochondrial	59.92	16.68	1.84528713	4.97E-18	1.68E-16	Up	
ACC_05373	uncharacterized protein LOC100865248	234.60	84.51	1.47295788	3.73E-48	5.11E-46	Up	
ACC_05400	Uncharacterized protein C9orf117 homolog	72.92	36.00	1.01811076	1.21E-09	1.85E-08	Up	
ACC_05401	uncharacterized protein LOC100864676	87.50	8.72	3.32743882	3.43E-48	4.77E-46	Up	
ACC_05429	Zinc finger protein	26.63	9.85	1.43435402	1.47E-06	1.50E-05	Up	
ACC_05502	Ribosome biogenesis protein BRX1 homolog	32.34	13.26	1.28561864	1.06E-06	1.10E-05	Up	
ACC_05508	NADH-cytochrome b5 reductase-like	20.29	7.96	1.3501589	5.95E-05	0.00045996	Up	
ACC_05534	Nuclear pore complex protein Nup88	36.46	17.05	1.09611327	4.98E-06	4.72E-05	Up	
ACC_05541	hypothetical protein LOC100576945	14.27	4.17	1.7748978	3.93E-05	0.00031545	Up	
ACC_05609	Putative sodium-coupled neutral amino acid transporter	92.89	22.36	2.05459012	5.84E-31	4.10E-29	Up	
ACC_05630	Protein mab-21	32.34	71.63	-1.1473408	3.15E-11	5.77E-10	Down	
ACC_05658	CAP-Gly domain-containing linker protein	439.40	202.00	1.12119614	1.23E-58	2.32E-56	Up	
ACC_05664	Polycomb protein suz12-B	228.26	111.80	1.02975827	1.84E-27	1.10E-25	Up	
ACC_05669	Probable ATP-dependent RNA helicase pitchoune	74.18	32.21	1.2034501	2.24E-12	4.60E-11	Up	
ACC_05708	Peroxisome proliferator-activated receptor gamma coactivator-related protein	189.58	80.72	1.23176837	3.00E-30	2.06E-28	Up	
ACC_05763	Uncharacterized protein CG10915	50.72	24.25	1.06440441	1.45E-07	1.73E-06	Up	
ACC_05814	RNA polymerase II-associated protein	55.16	23.50	1.23122351	7.16E-10	1.13E-08	Up	
ACC_05826	30S ribosomal protein S6	21.24	6.06	1.80856551	3.73E-07	4.15E-06	Up	
ACC_05856	Prefoldin subunit	30.75	13.64	1.17246416	9.58E-06	8.63E-05	Up	
ACC_05864	Protein dispatched homolog	19.97	7.58	1.39782815	4.31E-05	0.00034358	Up	
ACC_05883	60S ribosomal protein L29	29.17	13.64	1.09611327	4.43E-05	0.00035099	Up	
ACC_05911	tRNA dimethylallyltransferase, mitochondrial	47.55	17.81	1.41670616	1.82E-10	3.03E-09	Up	
ACC_05915	Kinesin-associated protein	44.07	19.71	1.16097767	1.46E-07	1.74E-06	Up	
ACC_05921	Ribosomal RNA processing protein	113.18	39.41	1.52181687	3.23E-25	1.66E-23	Up	
ACC_05976	GILT-like protein C02D5.2	48.82	23.12	1.07852552	1.84E-07	2.16E-06	Up	
ACC_06005	Coiled-coil domain-containing protein	10.14	2.27	2.15751382	8.01E-05	0.00060484	Up	
ACC_06015	Transcriptional regulator ATRX homolog	261.55	110.66	1.24090207	1.52E-41	1.54E-39	Up	
ACC_06026	Kinesin-like protein KIF23	33.92	11.75	1.529747	1.19E-08	1.62E-07	Up	response to stress
ACC_06033	Coiled-coil domain-containing protein	78.62	36.38	1.11171013	1.19E-11	2.27E-10	Up	
ACC_06050	Mitochondrial inner membrane protein OXA1L	35.82	15.92	1.17033786	1.84E-06	1.86E-05	Up	
ACC_06055	mRNA cap guanine-N7 methyltransferase	28.53	12.13	1.23432942	8.96E-06	8.11E-05	Up	
ACC_06081	Uncharacterized protein CG10915	31.39	15.54	1.01428094	7.01E-05	0.00053455	Up	

ACC_06105	Uncharacterized protein C12orf65	34.87	15.16	1.20190794	1.53E-06	1.56E-05	Up
ACC_06108	RNA polymerase II-associated protein	69.11	33.35	1.05122903	1.23E-09	1.89E-08	Up
ACC_06134	Cell growth-regulating nucleolar protein	46.92	14.78	1.66652747	9.76E-13	2.09E-11	Up
ACC_06174	PRKR-interacting protein	129.66	54.57	1.24850835	1.27E-21	5.52E-20	Up
ACC_06176	Protein AATF	7.93	0.76	3.38633251	9.77E-06	8.77E-05	Up
ACC_06177	Protein AATF	35.82	7.96	2.17033786	1.01E-13	2.41E-12	Up
ACC_06187	Nucleolar GTP-binding protein	70.38	27.29	1.36696718	4.40E-14	1.08E-12	Up
ACC_06207	Major royal jelly protein	10299.19	20845.82	-1.0172239	0	0	Down
ACC_06208	Major royal jelly protein	9674.97	23098.86	-1.2554895	0	0	Down
ACC_06233	Protein penguin	33.29	8.72	1.93315988	2.83E-11	5.24E-10	Up
ACC_06236	Exonuclease 3'-5' domain-containing protein	26.95	11.37	1.24497666	1.39E-05	0.00012061	Up
ACC_06251	MKI67 FHA domain-interacting nucleolar phosphoprotein-like	26.00	10.61	1.2926734	1.11E-05	9.82E-05	Up
ACC_06289	Lysosomal alpha-mannosidase	62.45	135.30	-1.1152521	4.68E-19	1.77E-17	Down
ACC_06291	Translation initiation factor 5B (eIF-5B)	75.13	35.25	1.09206076	6.36E-11	1.13E-09	Up
ACC_06303	TBC domain-containing protein kinase-like protein	75.45	28.80	1.38936657	2.51E-15	6.80E-14	Up
ACC_06306	HIRA-interacting protein	89.72	40.93	1.13224706	1.95E-13	4.47E-12	Up
ACC_06316	ADP-ribosylation factor-like protein 6-interacting protein	247.60	114.83	1.10848107	3.23E-33	2.55E-31	Up
ACC_06322	Zinc finger CCCH domain-containing protein	200.36	98.91	1.01835107	6.80E-24	3.29E-22	Up
ACC_06339	uncharacterized protein LOC100866703	180.70	82.24	1.1357432	1.31E-25	6.92E-24	Up
ACC_06368	ATP-binding cassette sub-family F member	60.23	27.67	1.12250737	2.20E-09	3.26E-08	Up
ACC_06386	Structural maintenance of chromosome protein 4 (chromosome condensation complex Condensin, subunit C)	664.48	321.75	1.04627858	1.29E-78	3.91E-76	Up
ACC_06407	Post-GPI attachment to proteins factor	42.48	17.43	1.28500355	2.25E-08	2.96E-07	Up
ACC_06409	Coiled-coil domain-containing protein	28.85	12.89	1.16280812	2.05E-05	0.0001722	Up
ACC_06414	hypothetical protein LOC100578330	22.19	1.89	3.54983124	4.68E-14	1.15E-12	Up
ACC_06415	Protein LTV1 homolog	73.55	34.11	1.10860422	5.96E-11	1.06E-09	Up
ACC_06417	UBX domain-containing protein	86.23	41.31	1.06175484	7.79E-12	1.52E-10	Up
ACC_06432	Uncharacterized protein C10orf78 homolog	6.02	0.00	4.99040383	1.69E-05	0.00014355	Up
ACC_06439	Protein FRA10AC1	19.34	5.68	1.76632306	1.83E-06	1.86E-05	Up
ACC_06472	HD domain-containing protein	37.73	18.19	1.05233158	7.03E-06	6.46E-05	Up
ACC_06521	ESF1 homolog	71.33	30.70	1.21640751	3.89E-12	7.82E-11	Up
ACC_06545	Probable ATP-dependent RNA helicase DDX55 homolog	42.48	17.43	1.28500355	2.25E-08	2.96E-07	Up
ACC_06581	U3 small nucleolar RNA-associated protein	32.97	15.54	1.08536403	1.65E-05	0.00014097	Up
ACC_06593	uncharacterized protein LOC100872663	13.32	4.17	1.67536212	0.00013642	0.0009983	Up
ACC_06600	Protein KRI1 homolog	79.89	36.38	1.13479374	3.64E-12	7.33E-11	Up
ACC_06626	Myosin class II heavy chain	79.26	39.41	1.00782089	3.28E-10	5.32E-09	Up
ACC_06633	tRNA-specific adenosine deaminase	18.07	5.68	1.66847574	9.39E-06	8.48E-05	Up
ACC_06634	uncharacterized protein LOC100866312	83.38	41.69	1.0000356	1.50E-10	2.53E-09	Up
ACC_06675	Disorganized muscle protein	730.43	334.64	1.12613169	6.32E-97	2.38E-94	Up

ACC_06698	Charged multivesicular body protein	33.29	6.82	2.28679684	1.31E-13	3.09E-12	Up	
ACC_06755	Centromere-associated protein E	75.45	26.15	1.52876963	1.91E-17	6.23E-16	Up	
ACC_06831	Calcineurin-binding protein cabin-1	97.33	46.99	1.05037485	5.78E-13	1.27E-11	Up	
ACC_06844	Bipolar kinesin KRP-130	19.34	6.06	1.67321366	4.37E-06	4.18E-05	Up	
ACC_06848	rRNA-processing protein FCF1 homolog	11.41	3.03	1.91240132	0.00010927	0.00081073	Up	
ACC_06853	Protein PAT1 homolog	22.51	8.72	1.36866148	1.93E-05	0.00016279	Up	
ACC_06861	hypothetical protein LOC551485	47.55	14.40	1.7233675	1.92E-13	4.43E-12	Up	
ACC_06862	hypothetical protein LOC100576464	493.93	242.93	1.02377529	6.67E-57	1.21E-54	Up	
ACC_06877	RNA polymerase II subunit	52.94	25.01	1.08178649	5.22E-08	6.58E-07	Up	
ACC_06903	NF-kappa-B inhibitor-like protein	78.62	38.66	1.02424729	2.24E-10	3.67E-09	Up	
ACC_06923	Vesicle transport through interaction with t-SNAREs homolog	10.78	2.65	2.02258424	9.56E-05	0.00071413	Up	
ACC_06971	Nucleolar protein	113.81	50.40	1.17503392	1.46E-17	4.81E-16	Up	
ACC_07000	DNA repair protein complementing XP-C cells homolog	184.83	79.59	1.21558288	5.88E-29	3.75E-27	Up	response to stress
ACC_07044	Enhancer of mRNA-decapping protein	64.04	26.91	1.25094068	1.78E-11	3.36E-10	Up	
ACC_07047	Protein slit	46.29	21.60	1.09941086	2.51E-07	2.86E-06	Up	
ACC_07053	GRIP and coiled-coil domain-containing protein	43.75	19.71	1.15056106	1.99E-07	2.31E-06	Up	
ACC_07082	Glucose dehydrogenase [acceptor]	534.82	1107.38	-1.0500199	1.64E-130	8.24E-128	Down	
ACC_07127	Solute carrier family	30.43	15.16	1.00551073	0.00010136	0.00075332	Up	
ACC_07147	DNA-directed RNA polymerase II subunit RPB2	20.61	7.96	1.37252671	4.18E-05	0.00033418	Up	
ACC_07159	Uncharacterized protein C1orf112 homolog	7.93	0.38	4.38633251	1.46E-06	1.50E-05	Up	
ACC_07183	Probable palmitoyltransferase ZDHHC11	32.02	14.78	1.11528558	1.43E-05	0.00012345	Up	
ACC_07241	Adenylyl cyclase-associated protein	22.51	9.47	1.24836725	6.91E-05	0.00052773	Up	
ACC_07242	FUN14 domain-containing protein	48.50	19.71	1.29942444	1.67E-09	2.52E-08	Up	
ACC_07252	Deleted in lung and esophageal cancer protein	19.97	7.96	1.32743882	8.43E-05	0.00063465	Up	
ACC_07262	Ras GTPase-activating protein	31.39	12.51	1.32743882	8.28E-07	8.82E-06	Up	
ACC_07275	uncharacterized protein LOC100868194	145.83	53.82	1.43821925	1.43E-29	9.33E-28	Up	
ACC_07316	hypothetical protein LOC411037	31.07	8.72	1.83362421	5.48E-10	8.68E-09	Up	
ACC_07336	Protein spaetzle	51.04	23.50	1.11919689	3.93E-08	5.02E-07	Up	
ACC_07337	Vanin-like protein	22.51	7.20	1.64429593	9.88E-07	1.04E-05	Up	
ACC_07344	hypothetical protein LOC551646	73.55	26.15	1.49193286	1.70E-16	5.19E-15	Up	
ACC_07369	NAD-dependent deacetylase sirtuin-1	110.01	48.13	1.19258349	2.14E-17	6.95E-16	Up	
ACC_07416	uncharacterized protein LOC724290	64.99	29.18	1.15516988	2.03E-10	3.35E-09	Up	
ACC_07428	Uncharacterized protein C10orf118	111.91	50.03	1.16160657	5.21E-17	1.63E-15	Up	
ACC_07474	ATP-dependent DNA helicase Q4	77.35	25.77	1.58575082	9.67E-19	3.52E-17	Up	response to stress
ACC_07547	Programmed cell death protein	44.07	12.51	1.81702327	2.08E-13	4.72E-12	Up	
ACC_07551	Structural maintenance of chromosome protein 1 (sister chromatid cohesion complex Cohesin, subunit SMC1)	213.99	104.22	1.0379322	3.64E-26	2.02E-24	Up	
ACC_07552	Eukaryotic translation initiation factor	385.82	192.52	1.00290509	2.13E-43	2.38E-41	Up	
ACC_07559	uncharacterized protein LOC100872292	6.66	0.76	3.13479374	8.88E-05	0.00066605	Up	
ACC_07590	hypothetical protein LOC725634	52.63	22.36	1.2348727	1.61E-09	2.44E-08	Up	
ACC_07675	Clathrin interactor	95.74	43.96	1.12290006	4.53E-14	1.11E-12	Up	

ACC_07679	DnaJ homolog subfamily C member	56.75	26.15	1.11776764	7.16E-09	1.00E-07	Up	
ACC_07695	Serine/threonine-protein kinase pelle	24.41	9.10	1.42430036	4.51E-06	4.31E-05	Up	
ACC_07714	WW domain-containing transcription regulator protein	75.45	37.14	1.02258424	5.42E-10	8.61E-09	Up	
ACC_07770	Biorientation of chromosomes in cell division protein	221.28	104.98	1.07581738	1.57E-28	9.96E-27	Up	
ACC_07782	Chromatin assembly factor	41.21	19.71	1.06440441	2.14E-06	2.15E-05	Up	
ACC_07784	Thyroid transcription factor	45.65	22.74	1.00551073	1.93E-06	1.94E-05	Up	
ACC_07786	28S ribosomal protein S30, mitochondrial	57.70	17.43	1.726709	4.99E-16	1.46E-14	Up	
ACC_07801	Zinc finger protein	31.07	14.02	1.1477328	1.22E-05	0.0001067	Up	
ACC_07813	Disks large-associated protein	29.48	7.96	1.88931771	6.91E-10	1.09E-08	Up	
ACC_07852	Protein FAM60A	37.09	13.26	1.48355802	5.60E-09	7.88E-08	Up	
ACC_07862	Glutamate receptor U1	44.70	18.19	1.29706517	7.57E-09	1.05E-07	Up	
ACC_07869	Clathrin interactor	78.31	36.38	1.10588105	1.59E-11	3.02E-10	Up	
ACC_07967	hypothetical protein LOC100747105	56.43	20.09	1.4902893	5.47E-13	1.20E-11	Up	
ACC_07980	Protein QN1 homolog	38.99	16.68	1.22555921	2.44E-07	2.79E-06	Up	
ACC_07984	hypothetical protein LOC100647806	25.36	6.82	1.89447941	9.96E-09	1.37E-07	Up	
ACC_08106	Glycogenin-1	175.31	31.08	2.49605999	1.44E-71	3.63E-69	Up	
ACC_08133	Nose resistant to fluoxetine protein	32.34	14.40	1.16697415	6.12E-06	5.70E-05	Up	
ACC_08138	Chitobiosyldiphosphodolichol beta-mannosyltransferase	10.14	2.27	2.15751382	8.01E-05	0.00060484	Up	
ACC_08204	uncharacterized protein LOC100865574	120.47	50.40	1.25704949	2.11E-20	8.41E-19	Up	
ACC_08214	Probable ribosome production factor	41.53	17.81	1.22131047	1.08E-07	1.30E-06	Up	
ACC_08233	PCI domain-containing protein	26.00	8.34	1.64059671	1.51E-07	1.79E-06	Up	
ACC_08235	Pin2-interacting protein X1	71.65	29.56	1.27725306	4.91E-13	1.09E-11	Up	
ACC_08291	Multiple RNA-binding domain-containing protein	976.12	362.68	1.42834736	8.23E-186	4.97E-183	Up	
ACC_08353	uncharacterized protein LOC100872020	145.51	47.37	1.61904238	8.37E-35	6.76E-33	Up	
ACC_08369	DNA polymerase zeta catalytic subunit	71.33	29.56	1.27085529	6.78E-13	1.48E-11	Up	
ACC_08388	Structural maintenance of chromosome protein 4 (chromosome condensation complex Condensin, subunit C)	74.18	32.21	1.2034501	2.24E-12	4.60E-11	Up	
ACC_08411	SLIT and NTRK-like protein	13.63	3.79	1.84681298	3.63E-05	0.00029352	Up	
ACC_08423	DnaJ homolog subfamily C member	106.84	53.06	1.00979808	2.69E-13	6.04E-12	Up	
ACC_08443	S-adenosylmethionine decarboxylase proenzyme	174.68	81.86	1.09349733	1.96E-23	9.23E-22	Up	
ACC_08453	Zinc finger protein	19.34	7.20	1.42528614	4.42E-05	0.00035068	Up	
ACC_08483	Disintegrin and metalloproteinase domain-containing protein	52.63	23.88	1.14023583	1.49E-08	2.00E-07	Up	
ACC_08495	Protein TIPIN homolog	31.39	14.40	1.12390543	1.54E-05	0.00013207	Up	response to stress
ACC_08507	Zinc finger MYM-type protein	41.53	20.09	1.04797887	2.63E-06	2.60E-05	Up	
ACC_08519	Butyrate response factor	54.21	25.77	1.07286599	4.46E-08	5.65E-07	Up	
ACC_08570	Citron Rho-interacting kinase	72.28	29.56	1.28996411	2.56E-13	5.77E-12	Up	
ACC_08616	Arginyl-tRNA synthetase, cytoplasmic	35.51	15.92	1.15751382	2.50E-06	2.48E-05	Up	

ACC_08692	Nucleolar complex protein	36.77	15.92	1.20813989	7.16E-07	7.69E-06	Up	
ACC_08737	hypothetical protein LOC100578734	90.35	34.49	1.38949979	4.72E-18	1.60E-16	Up	
ACC_08754	DNA polymerase alpha catalytic subunit	25.36	10.99	1.20642342	3.92E-05	0.0003151	Up	response to stress
ACC_08766	70 kDa peptidyl-prolyl isomerase	22.51	8.34	1.43279182	9.70E-06	8.73E-05	Up	
ACC_08843	Actin-related protein	27.90	12.89	1.1144451	5.17E-05	0.00040506	Up	
ACC_08854	uncharacterized protein LOC100867228	245.38	118.62	1.04864723	3.08E-30	2.10E-28	Up	
ACC_08861	PiggyBac transposable element-derived protein	36.46	13.26	1.45868335	1.16E-08	1.58E-07	Up	
ACC_08891	U3 small nucleolar ribonucleoprotein protein IMP3	53.26	20.84	1.35343403	7.18E-11	1.27E-09	Up	
ACC_08892	XK-related protein	5.39	0.00	4.82993916	5.12E-05	0.0004015	Up	
ACC_08923	Small subunit processome component	229.21	83.38	1.45896844	1.89E-46	2.42E-44	Up	
ACC_08984	Ribonuclease Z, mitochondrial	79.89	23.12	1.78901891	1.33E-22	6.08E-21	Up	
ACC_08996	p21-activated protein kinase-interacting protein	25.04	9.85	1.34581735	8.61E-06	7.81E-05	Up	
ACC_09042	Centromere/kinetochore protein zw10 homolog	42.16	20.84	1.01639904	3.91E-06	3.77E-05	Up	
ACC_09074	CCAAT/enhancer-binding protein	28.85	10.99	1.39228996	9.54E-07	1.01E-05	Up	
ACC_09083	hypothetical protein LOC100577224	13.00	3.03	2.10002832	1.14E-05	0.0001003	Up	
ACC_09100	NADH-ubiquinone oxidoreductase chain	49.14	17.81	1.46401187	3.08E-11	5.67E-10	Up	
ACC_09128	hypothetical protein Phum_PHUM351820	3549.73	1595.89	1.15335378	0	0	Up	
ACC_09134	hypothetical protein LOC726868	57.70	25.77	1.16280812	1.71E-09	2.57E-08	Up	
ACC_09175	RNA-binding protein	170.56	75.42	1.17731406	1.33E-25	7.00E-24	Up	
ACC_09254	Meiotic recombination protein REC8 homolog	29.48	6.82	2.11171013	3.28E-11	6.00E-10	Up	
ACC_09301	Synaptic vesicle membrane protein VAT-1 homolog-like	428.30	183.05	1.2264089	1.04E-65	2.35E-63	Up	
ACC_09307	Major royal jelly protein	1625.07	5586.55	-1.7814527	0	0	Down	
ACC_09333	hypothetical protein LOC726729	52.94	21.60	1.2932906	3.53E-10	5.72E-09	Up	
ACC_09337	Inner centromere protein A	153.12	73.90	1.05102538	1.53E-19	5.88E-18	Up	
ACC_09341	hypothetical protein LOC551572	16.80	1.89	3.14846868	4.42E-10	7.09E-09	Up	
ACC_09380	dnaJ homolog subfamily B member 6-like	31.70	14.02	1.17687914	6.55E-06	6.06E-05	Up	
ACC_09396	snRNA-activating protein complex subunit	64.36	21.98	1.54983124	2.22E-15	6.04E-14	Up	
ACC_09399	hypothetical protein LOC100577341	42.16	2.65	3.99040383	2.62E-27	1.56E-25	Up	
ACC_09401	Crossover junction endonuclease EME1	45.33	19.33	1.22992231	2.38E-08	3.10E-07	Up	
ACC_09441	DNA polymerase subunit gamma-2, mitochondrial	63.41	29.94	1.08255176	2.51E-09	3.68E-08	Up	
ACC_09471	DNA repair protein RAD50, ABC-type ATPase/SMC superfamily	80.21	34.11	1.2336168	9.90E-14	2.36E-12	Up	
ACC_09475	Transcription factor IIIB	111.28	53.06	1.06852052	5.57E-15	1.47E-13	Up	
ACC_09476	Erlin-1	16.80	5.31	1.66304185	2.02E-05	0.00016963	Up	
ACC_09485	Methyltransferase-like protein	8.56	1.52	2.49736382	7.69E-05	0.0005827	Up	
ACC_09487	Insulinoma-associated protein	37.09	18.19	1.02787854	1.24E-05	0.00010847	Up	
ACC_09523	Protein KRI1 homolog	144.88	64.81	1.16067416	1.56E-21	6.75E-20	Up	
ACC_09528	Putative ATP-dependent RNA helicase DHX57	104.30	43.20	1.27153008	3.61E-18	1.25E-16	Up	
ACC_09535	Monocarboxylate transporter	34.24	15.54	1.13981182	4.96E-06	4.72E-05	Up	
ACC_09571	Transcriptional regulator ATRX homolog	276.13	125.82	1.1339658	3.69E-38	3.38E-36	Up	
ACC_09618	CCR4-NOT transcription complex subunit	32.65	16.30	1.00271209	5.89E-05	0.00045589	Up	

ACC_09628	Phosphatidylinositol 4-kinase alpha	34.24	15.54	1.13981182	4.96E-06	4.72E-05	Up
ACC_09682	Unconventional prefoldin RPB5 interactor	88.45	35.62	1.31200878	2.41E-16	7.21E-15	Up
ACC_09723	Interference hedgehog	32.97	11.37	1.53602544	1.72E-08	2.30E-07	Up
ACC_09755	WD repeat-containing protein	51.68	22.36	1.20856142	4.16E-09	5.91E-08	Up
ACC_09779	Dynein intermediate chain 2, axonemal	138.86	45.48	1.61037278	4.79E-33	3.74E-31	Up
ACC_09799	Vacuolar protein sorting-associated protein	22.51	9.85	1.19178372	0.00012481	0.00091853	Up
ACC_09860	Transcription factor IIIA	9.83	1.89	2.37474454	3.87E-05	0.00031119	Up
ACC_09919	Transcription factor IIIA	20.29	7.20	1.49454881	1.46E-05	0.00012593	Up
ACC_09921	Zinc finger FYVE domain-containing protein	43.12	18.95	1.18608297	1.23E-07	1.47E-06	Up
ACC_09946	Nucleolar MIF4G domain-containing protein	30.75	9.10	1.75742666	2.03E-09	3.04E-08	Up
ACC_10015	hypothetical protein LOC100576792	11.10	1.14	3.28679684	2.38E-07	2.73E-06	Up
ACC_10115	hypothetical protein EAI_16506	17.44	5.68	1.61694544	2.08E-05	0.00017456	Up
ACC_10163	Vacuolar protein sorting-associated protein	45.97	18.57	1.30767557	3.72E-09	5.32E-08	Up

Supplementary Table 20. Significantly up/down regulated genes

Gene	Product	RPM body-shaking bees	RPM control	body-shaking vs control log2(Fold_change) normalized	p-value	FDR	Regulation
ACC_00043	Alpha-(1,3)-fucosyltransferase C	12.54	33.35	-1.41084501	4.19E-10	2.80E-08	Down
ACC_00054	Tetraspanin-11	15.16	31.46	-1.05269201	1.46E-06	5.11E-05	Down
ACC_00121	Class A basic helix-loop-helix protein	56.91	28.42	1.00160625	5.00E-10	3.30E-08	Up
ACC_00126	Mitochondrial ribonuclease P protein	25.83	11.75	1.13682557	3.39E-06	0.00011055	Up
ACC_00227	Adipocyte plasma membrane-associated protein	70.76	34.49	1.03694521	9.62E-13	8.76E-11	Up
ACC_00278	hypothetical protein LOC100577894	29.77	12.89	1.20791754	1.66E-07	6.75E-06	Up
ACC_00291	Peptidoglycan-recognition protein SC2	7.30	18.19	-1.31706286	1.20E-05	0.00032032	Down
ACC_00550	Tyrosine-protein phosphatase non-receptor type	4.49	12.89	-1.52000292	4.04E-05	0.00087604	Down
ACC_00561	Abhydrolase domain-containing protein	27.14	55.71	-1.03726583	2.40E-10	1.63E-08	Down
ACC_00644	uncharacterized protein LOC100865162	23.78	10.61	1.16382719	5.59E-06	0.00016576	Up
ACC_00645	Apidaecins type	16.29	108.77	-2.73946601	8.15E-68	2.97E-65	Down
ACC_00683	Nucleolar complex protein	17.60	7.20	1.28915876	2.29E-05	0.00054661	Up
ACC_00756	NAD kinase	10.48	25.01	-1.25454177	7.71E-07	2.83E-05	Down
ACC_00825	Nuclear nucleic acid-binding protein C1D	24.71	10.99	1.16891055	3.39E-06	0.00011083	Up
ACC_00864	TRAF-interacting protein	27.52	10.99	1.32418877	6.42E-08	2.76E-06	Up
ACC_00889	Proton-coupled amino acid transporter	14.41	42.45	-1.55807096	3.28E-14	3.68E-12	Down
ACC_01096	Serine proteinase stubble	26.96	54.57	-1.01750258	6.84E-10	4.32E-08	Down
ACC_01159	Elongation factor 1-alpha	185.15	424.46	-1.19695888	9.67E-86	4.19E-83	Down
ACC_01201	Solute carrier organic anion transporter family member	7.49	17.81	-1.25016333	3.19E-05	0.00072164	Down
ACC_01212	Ornithine decarboxylase	7.11	18.57	-1.38428491	4.33E-06	0.00013453	Down
ACC_01244	Thioredoxin domain-containing protein	8.42	20.09	-1.25356994	9.58E-06	0.00026645	Down
ACC_01286	Putative phospholipase B-like lamina ancestor	49.61	105.74	-1.09177534	9.28E-20	1.53E-17	Down
ACC_01484	Heat shock protein	1268.50	565.82	1.16471713	9.11E-242	6.37E-239	Up
ACC_01856	Zinc finger protein DZIP1L	18.16	7.20	1.33448275	9.96E-06	0.00027452	Up
ACC_01980	Prefoldin subunit	19.66	5.31	1.88938802	3.39E-09	1.78E-07	Up
ACC_02123	CD63 antigen	46.43	97.40	-1.06893082	9.30E-18	1.34E-15	Down
ACC_02154	hypothetical protein E5Q_03048	3789.96	1710.72	1.14758474	0	0	Up
ACC_02229	Mesencephalic astrocyte-derived neurotrophic factor homolog	46.05	21.98	1.06703093	3.80E-09	1.98E-07	Up
ACC_02371	E3 ubiquitin-protein ligase UBR2	9.92	23.88	-1.26686205	1.14E-06	4.06E-05	Down
ACC_02425	von Willebrand factor D and EGF domain-containing protein	8.05	18.95	-1.23509401	2.14E-05	0.00052088	Down
ACC_02429	Bifunctional ATP-dependent dihydroxyacetone kinase/FAD-AMP lyase (cyclizing)	20.41	42.07	-1.04373412	3.18E-08	1.45E-06	Down
ACC_02493	uncharacterized protein LOC100871978	40.44	19.33	1.06495958	3.53E-08	1.59E-06	Up

ACC_02546	Cubilin	18.91	39.79	-1.07353661	3.70E-08	1.66E-06	Down
ACC_02765	hypothetical protein LOC100577958	29.58	11.37	1.37938758	7.32E-09	3.70E-07	Up
ACC_02768	Serum response factor-binding protein	28.83	14.40	1.00135645	9.62E-06	0.00026694	Up
ACC_02805	tRNA (guanine-N(1)-)-methyltransferase	58.03	18.19	1.67365933	9.60E-21	1.68E-18	Up
ACC_02807	RNA-binding protein NOB1	26.40	12.89	1.03458593	1.36E-05	0.00035498	Up
ACC_02838	hypothetical protein LOC724993	9.73	23.88	-1.29434278	7.50E-07	2.76E-05	Down
ACC_02858	10-formyltetrahydrofolate dehydrogenase	8.61	40.93	-2.24882812	1.43E-21	2.60E-19	Down
ACC_03016	Serine protease snake	8.42	22.74	-1.43254008	1.81E-07	7.29E-06	Down
ACC_03098	Probable signal peptidase complex subunit	19.84	40.93	-1.04446963	4.78E-08	2.10E-06	Down
ACC_03149	Pheromone-binding protein-related protein	19.09	57.23	-1.58348197	4.94E-19	7.88E-17	Down
ACC_03442	TRAF-interacting protein	44.18	20.46	1.11025297	2.51E-09	1.39E-07	Up
ACC_03450	Hymenoptaecin	2.62	31.83	-3.60246508	1.57E-26	3.40E-24	Down
ACC_03585	Hymenoptaecin	3.56	73.90	-4.37690538	1.03E-66	3.61E-64	Down
ACC_03594	39S ribosomal protein L54, mitochondrial	11.61	3.79	1.61476564	4.67E-05	0.00098861	Up
ACC_03623	Phenoloxidase subunit A3	11.42	23.50	-1.04096155	3.71E-05	0.00081332	Down
ACC_03733	Protein ccsmst1	18.16	4.93	1.88197055	1.46E-08	7.12E-07	Up
ACC_03947	Ejaculatory bulb-specific protein	11.23	26.91	-1.2603591	2.67E-07	1.05E-05	Down
ACC_04002	hypothetical protein LOC100647857	32.01	9.47	1.75649375	7.14E-13	6.63E-11	Up
ACC_04039	DNA repair protein XRCC1	25.27	11.37	1.15242242	3.39E-06	0.00011031	Up
ACC_04063	ABC transporter G family member	6.18	15.92	-1.36542588	2.56E-05	0.00060274	Down
ACC_04103	Monocarboxylate transporter	0.94	7.20	-2.943502	3.43E-06	0.00011019	Down
ACC_04163	E3 ubiquitin-protein ligase Nedd-4	23.03	46.24	-1.00572541	1.86E-08	9.00E-07	Down
ACC_04405	Puratrophin-1	17.41	40.17	-1.20626422	1.25E-09	7.25E-08	Down
ACC_04546	Translation initiation factor IF-3, chloroplastic	51.29	21.60	1.24763949	1.82E-12	1.61E-10	Up
ACC_04643	Lysine-specific demethylase 6A	17.97	36.38	-1.01750258	4.72E-07	1.77E-05	Down
ACC_04656	Antithrombin-III	12.73	26.53	-1.05932275	8.75E-06	0.00024638	Down
ACC_04697	Myosin regulatory light chain	9.92	21.22	-1.09693705	4.29E-05	0.0009191	Down
ACC_04755	Vitellogenin-3	116.63	331.61	-1.50755343	3.43E-95	1.64E-92	Down
ACC_04825	Putative RNA polymerase II subunit B1 CTD phosphatase RPAP2	27.33	13.26	1.04303896	8.35E-06	0.00023737	Up
ACC_04897	uncharacterized protein LOC100867921	45.30	19.71	1.20092094	1.30E-10	9.47E-09	Up
ACC_04989	Uncharacterized protein C9orf85 homolog	10.11	2.27	2.15242242	3.94E-06	0.00012548	Up
ACC_05129	ATPase family AAA domain-containing protein	76.01	37.14	1.0331235	1.67E-13	1.71E-11	Up
ACC_05194	hypothetical protein LOC100576614	15.16	5.31	1.5149925	8.90E-06	0.00024919	Up
ACC_05358	Probable palmitoyltransferase ZDHHC8	1.68	7.96	-2.239895	2.71E-05	0.00063168	Down
ACC_05369	Transcription factor A, mitochondrial	36.32	16.68	1.12297865	4.92E-08	2.15E-06	Up
ACC_05401	uncharacterized protein LOC100864676	22.84	8.72	1.3896728	3.21E-07	1.23E-05	Up
ACC_05472	Putative ferric-chelate reductase	14.23	43.20	-1.60246508	5.63E-15	6.65E-13	Down
ACC_05630	Protein mab-21	32.01	71.63	-1.16189249	3.49E-15	4.29E-13	Down
ACC_05755	hypothetical protein LOC100576758 isoform 1	1.68	7.96	-2.239895	2.71E-05	0.00063168	Down
ACC_05817	Putative fatty acyl-CoA reductase CG5065	32.39	66.70	-1.04230597	3.47E-12	2.87E-10	Down

ACC_06000	Glucosylceramidase	195.25	472.21	-1.27406749	8.68E-105	4.39E-102	Down
ACC_06105	Uncharacterized protein C12orf65	30.33	15.16	1.00041933	5.75E-06	0.0001687	Up
ACC_06134	Cell growth-regulating nucleolar protein	33.32	14.78	1.17282863	6.32E-08	2.73E-06	Up
ACC_06142	ATP-binding cassette sub-family G member	17.04	43.96	-1.36768893	2.47E-12	2.12E-10	Down
ACC_06176	Protein AATF	5.99	0.76	2.98249742	2.02E-05	0.00049708	Up
ACC_06362	Succinyl-CoA:3-ketoacid-coenzyme A transferase 1, mitochondrial	14.41	29.56	-1.03611826	4.05E-06	0.00012831	Down
ACC_06414	hypothetical protein LOC100578330	14.04	1.89	2.88938802	1.32E-10	9.46E-09	Up
ACC_06609	Major royal jelly protein	1.68	8.34	-2.30700919	1.23E-05	0.00032543	Down
ACC_06698	Charged multivesicular body protein	22.09	6.82	1.69521547	6.06E-09	3.12E-07	Up
ACC_07075	Cytochrome b5	5.99	18.57	-1.63221242	2.10E-07	8.40E-06	Down
ACC_07159	Uncharacterized protein C1orf112 homolog	5.24	0.38	3.78985234	1.06E-05	0.00028895	Up
ACC_07268	Acyl-CoA Delta(11) desaturase	15.54	36.76	-1.24237599	2.74E-09	1.47E-07	Down
ACC_07316	hypothetical protein LOC411037	20.59	8.72	1.24029518	8.80E-06	0.00024709	Up
ACC_07443	Alpha-tocopherol transfer protein	23.40	49.65	-1.08514129	5.55E-10	3.58E-08	Down
ACC_07626	Abaecin	1.68	13.26	-2.97686059	2.34E-10	1.60E-08	Down
ACC_07733	Protein FAM20B	13.67	4.93	1.47188226	3.66E-05	0.0008084	Up
ACC_07984	hypothetical protein LOC100647806	16.66	6.82	1.28830585	3.82E-05	0.00083448	Up
ACC_08000	Dehydrogenase/reductase SDR family member	27.14	54.57	-1.00751849	9.37E-10	5.72E-08	Down
ACC_08083	Chymotrypsin inhibitor	48.86	110.66	-1.17942114	4.07E-23	8.05E-21	Down
ACC_08085	Chymotrypsin inhibitor	2665.05	1255.56	1.08583115	0	0	Up
ACC_08106	Glycogenin-1	66.83	31.08	1.10472568	2.84E-13	2.87E-11	Up
ACC_08128	Esterase E4	4.68	34.11	-2.86549948	2.02E-23	4.08E-21	Down
ACC_08383	ABC transporter G family member	20.41	43.58	-1.0948083	4.81E-09	2.49E-07	Down
ACC_08483	Disintegrin and metalloproteinase domain-containing protein	51.48	23.88	1.10850531	1.31E-10	9.45E-09	Up
ACC_08812	Mitochondrial inner membrane protein COX18	7.49	28.80	-1.943502	3.05E-13	3.02E-11	Down
ACC_09010	Kinesin-like protein KIF6	15.73	4.93	1.67437513	1.15E-06	4.10E-05	Up
ACC_09121	Trinucleotide repeat-containing gene 6A protein	4.31	12.89	-1.58140346	2.38E-05	0.0005647	Down
ACC_09128	hypothetical protein Phum_PHUM351820	3329.25	1595.89	1.0608425	0	0	Up
ACC_09254	Meiotic recombination protein REC8 homolog	17.78	6.82	1.38242803	7.06E-06	0.00020321	Up
ACC_09307	Major royal jelly protein	2453.51	5586.55	-1.18710954	0	0	Down
ACC_09321	Nuclear factor of activated T-cells	17.22	34.49	-1.00173526	1.29E-06	4.54E-05	Down
ACC_09399	hypothetical protein LOC100577341	23.03	2.65	3.11765701	1.42E-17	2.02E-15	Up
ACC_09497	KEKE-like motif-containing transcription regulator (Rlr1)/suppressor of sin4	3.37	11.37	-1.75446817	1.92E-05	0.00047703	Down
ACC_09738	4-hydroxyphenylpyruvate dioxygenase	2.43	12.13	-2.31706286	1.24E-07	5.15E-06	Down
ACC_09825	Synaptic vesicle glycoprotein 2C	37.07	99.29	-1.42156896	2.20E-27	5.14E-25	Down
ACC_10015	hypothetical protein LOC100576792	7.86	1.14	2.78985234	2.32E-06	7.83E-05	Up

Supplementary Table 21. Enrichment analysis of significantly regulated genes in grooming bees

Pathway/Class	Up-regulated Genes in clear-grooming bees	*A.cerana* Genes	Pvalue
GO:0006950/response to stress	24	303	0.0149365
GO:0003676/nucleic acid binding	90	1064	8.33E-08
GO:0006259/DNA metabolic process	28	172	1.00E-07
GO:0006807/nitrogen compound metabolic process	66	698	1.87E-07
GO:0040029/regulation of gene expression, epigenetic	3	14	4.03E-02
GO:0004518/nuclease activity	10	56	7.69E-04
	Down-regulated Genes in clear-grooming bees		
GO:0009607/response to biotic stimulus	3	36	1.62E-05
	Down-regulated Genes in shaking-grooming bees		
GO:0009607/response to biotic stimulus	5	34	6.36E-06

Supplementary Table 22 Genes involved in Sensory System of A.cerana

Class	Gene	IPR	Product	GO
**Odorant binding protein (OBP)**	ACC_01037	IPR006170	Pheromone/general odorant binding protein, PBP/GOBP	GO:0005549-odorant binding
	ACC_01038	IPR006170	Pheromone/general odorant binding protein, PBP/GOBP	GO:0005549-odorant binding
	ACC_03149	IPR006170	Pheromone/general odorant binding protein, PBP/GOBP	GO:0005549-odorant binding
	ACC_06104	IPR006170	Pheromone/general odorant binding protein, PBP/GOBP	GO:0005549-odorant binding
	ACC_06720	IPR006170	Pheromone/general odorant binding protein, PBP/GOBP	GO:0005549-odorant binding
	ACC_07022	IPR006170	Pheromone/general odorant binding protein, PBP/GOBP	GO:0005549-odorant binding
	ACC_07721	IPR006170	Pheromone/general odorant binding protein, PBP/GOBP	GO:0005549-odorant binding
	ACC_08050	IPR006170	Pheromone/general odorant binding protein, PBP/GOBP	GO:0005549-odorant binding
	ACC_08110	IPR006170	Pheromone/general odorant binding protein, PBP/GOBP	GO:0005549-odorant binding
	ACC_08685	IPR006170	Pheromone/general odorant binding protein, PBP/GOBP	GO:0005549-odorant binding
	ACC_09844	IPR006170	Pheromone/general odorant binding protein, PBP/GOBP	GO:0005549-odorant binding
	ACC_10170	IPR006170	Pheromone/general odorant binding protein, PBP/GOBP	GO:0005549-odorant binding
	ACC_10182	IPR006170	Pheromone/general odorant binding protein, PBP/GOBP	GO:0005549-odorant binding
	ACC_09870	IPR023316	Pheromone/general odorant binding protein, PBP/GOBP, domain	GO:0005549-odorant binding
**Olfactory receptor (Or)**	ACC_00024	IPR004117	Olfactory receptor, Drosophila	GO:0004984-olfactory receptor activity
	ACC_00582	IPR004117	Olfactory receptor, Drosophila	GO:0004984-olfactory receptor activity
	ACC_00740	IPR004117	Olfactory receptor, Drosophila	GO:0004984-olfactory receptor activity
	ACC_00976	IPR004117	Olfactory receptor, Drosophila	GO:0004984-olfactory receptor activity
	ACC_01253	IPR004117	Olfactory receptor, Drosophila	GO:0004984-olfactory receptor activity
	ACC_01795	IPR004117	Olfactory receptor, Drosophila	GO:0004984-olfactory receptor activity
	ACC_01916	IPR004117	Olfactory receptor, Drosophila	GO:0004984-olfactory receptor activity
	ACC_02313	IPR004117	Olfactory receptor, Drosophila	GO:0004984-olfactory receptor activity
	ACC_02314	IPR004117	Olfactory receptor, Drosophila	GO:0004984-olfactory receptor activity
	ACC_02315	IPR004117	Olfactory receptor, Drosophila	GO:0004984-olfactory receptor activity
	ACC_02316			
	ACC_02317	IPR004117	Olfactory receptor, Drosophila	GO:0004984-olfactory receptor activity
	ACC_02318	IPR004117	Olfactory receptor, Drosophila	GO:0004984-olfactory receptor activity
	ACC_02319	IPR004117	Olfactory receptor, Drosophila	GO:0004984-olfactory receptor activity
	ACC_02320	IPR004117	Olfactory receptor, Drosophila	GO:0004984-olfactory receptor activity
	ACC_02321	IPR004117	Olfactory receptor, Drosophila	GO:0004984-olfactory receptor activity
	ACC_02322	IPR004117	Olfactory receptor, Drosophila	GO:0004984-olfactory receptor activity
	ACC_02323	IPR004117	Olfactory receptor, Drosophila	GO:0004984-olfactory receptor activity
	ACC_02324	IPR004117	Olfactory receptor, Drosophila	GO:0004984-olfactory receptor activity
	ACC_02325	IPR004117	Olfactory receptor, Drosophila	GO:0004984-olfactory receptor activity
	ACC_02326	IPR004117	Olfactory receptor, Drosophila	GO:0004984-olfactory receptor activity
	ACC_02327	IPR004117	Olfactory receptor, Drosophila	GO:0004984-olfactory receptor activity
	ACC_02328	IPR004117	Olfactory receptor, Drosophila	GO:0004984-olfactory receptor activity
	ACC_02329	IPR004117	Olfactory receptor, Drosophila	GO:0004984-olfactory receptor activity
	ACC_02330	IPR004117	Olfactory receptor, Drosophila	GO:0004984-olfactory receptor activity
	ACC_02331	IPR004117	Olfactory receptor, Drosophila	GO:0004984-olfactory receptor activity
	ACC_02332	IPR004117	Olfactory receptor, Drosophila	GO:0004984-olfactory receptor activity
	ACC_02333	IPR004117	Olfactory receptor, Drosophila	GO:0004984-olfactory receptor activity
	ACC_02391	IPR004117	Olfactory receptor, Drosophila	GO:0004984-olfactory receptor activity
	ACC_03357	IPR004117	Olfactory receptor, Drosophila	GO:0004984-olfactory receptor activity
	ACC_03358	IPR004117	Olfactory receptor, Drosophila	GO:0004984-olfactory receptor activity
	ACC_03359	IPR004117	Olfactory receptor, Drosophila	GO:0004984-olfactory receptor activity

ACC_03890	IPR004117	Olfactory receptor, Drosophila	GO:0004984-olfactory receptor activity
ACC_03891	IPR004117	Olfactory receptor, Drosophila	GO:0004984-olfactory receptor activity
ACC_04484	IPR004117	Olfactory receptor, Drosophila	GO:0004984-olfactory receptor activity
ACC_04782	IPR004117	Olfactory receptor, Drosophila	GO:0004984-olfactory receptor activity
ACC_05147	IPR004117	Olfactory receptor, Drosophila	GO:0004984-olfactory receptor activity
ACC_05242	IPR004117	Olfactory receptor, Drosophila	GO:0004984-olfactory receptor activity
ACC_05244	IPR004117	Olfactory receptor, Drosophila	GO:0004984-olfactory receptor activity
ACC_05245	IPR004117	Olfactory receptor, Drosophila	GO:0004984-olfactory receptor activity
ACC_05246	IPR004117	Olfactory receptor, Drosophila	GO:0004984-olfactory receptor activity
ACC_05964	IPR004117	Olfactory receptor, Drosophila	GO:0004984-olfactory receptor activity
ACC_06482	IPR004117	Olfactory receptor, Drosophila	GO:0004984-olfactory receptor activity
ACC_06793	IPR004117	Olfactory receptor, Drosophila	GO:0004984-olfactory receptor activity
ACC_06806	IPR004117	Olfactory receptor, Drosophila	GO:0004984-olfactory receptor activity
ACC_06987	IPR004117	Olfactory receptor, Drosophila	GO:0004984-olfactory receptor activity
ACC_06988	IPR004117	Olfactory receptor, Drosophila	GO:0004984-olfactory receptor activity
ACC_07004	IPR004117	Olfactory receptor, Drosophila	GO:0004984-olfactory receptor activity
ACC_07005	IPR004117	Olfactory receptor, Drosophila	GO:0004984-olfactory receptor activity
ACC_07108	IPR004117	Olfactory receptor, Drosophila	GO:0004984-olfactory receptor activity
ACC_07148	IPR004117	Olfactory receptor, Drosophila	GO:0004984-olfactory receptor activity
ACC_07149	IPR004117	Olfactory receptor, Drosophila	GO:0004984-olfactory receptor activity
ACC_07150	IPR004117	Olfactory receptor, Drosophila	GO:0004984-olfactory receptor activity
ACC_07273	IPR004117	Olfactory receptor, Drosophila	GO:0004984-olfactory receptor activity
ACC_07318	IPR004117	Olfactory receptor, Drosophila	GO:0004984-olfactory receptor activity
ACC_07395	IPR004117	Olfactory receptor, Drosophila	GO:0004984-olfactory receptor activity
ACC_08009	IPR004117	Olfactory receptor, Drosophila	GO:0004984-olfactory receptor activity
ACC_08113	IPR004117	Olfactory receptor, Drosophila	GO:0004984-olfactory receptor activity
ACC_08209	IPR004117	Olfactory receptor, Drosophila	GO:0004984-olfactory receptor activity
ACC_08210	IPR004117	Olfactory receptor, Drosophila	GO:0004984-olfactory receptor activity
ACC_08211	IPR004117	Olfactory receptor, Drosophila	GO:0004984-olfactory receptor activity
ACC_08212	IPR004117	Olfactory receptor, Drosophila	GO:0004984-olfactory receptor activity
ACC_08360	IPR004117	Olfactory receptor, Drosophila	GO:0004984-olfactory receptor activity
ACC_08361	IPR004117	Olfactory receptor, Drosophila	GO:0004984-olfactory receptor activity
ACC_08878	IPR004117	Olfactory receptor, Drosophila	GO:0004984-olfactory receptor activity
ACC_09009	IPR004117	Olfactory receptor, Drosophila	GO:0004984-olfactory receptor activity
ACC_09105	IPR004117	Olfactory receptor, Drosophila	GO:0004984-olfactory receptor activity
ACC_09385	IPR004117	Olfactory receptor, Drosophila	GO:0004984-olfactory receptor activity
ACC_09550	IPR004117	Olfactory receptor, Drosophila	GO:0004984-olfactory receptor activity
ACC_09579	IPR004117	Olfactory receptor, Drosophila	GO:0004984-olfactory receptor activity
ACC_09623	IPR004117	Olfactory receptor, Drosophila	GO:0004984-olfactory receptor activity
ACC_09650	IPR004117	Olfactory receptor, Drosophila	GO:0004984-olfactory receptor activity
ACC_09654	IPR004117	Olfactory receptor, Drosophila	GO:0004984-olfactory receptor activity
ACC_09659	IPR004117	Olfactory receptor, Drosophila	GO:0004984-olfactory receptor activity
ACC_09673	IPR004117	Olfactory receptor, Drosophila	GO:0004984-olfactory receptor activity
ACC_09690	IPR004117	Olfactory receptor, Drosophila	GO:0004984-olfactory receptor activity
ACC_09701	IPR004117	Olfactory receptor, Drosophila	GO:0004984-olfactory receptor activity
ACC_09732	IPR004117	Olfactory receptor, Drosophila	GO:0004984-olfactory receptor activity
ACC_09788	IPR004117	Olfactory receptor, Drosophila	GO:0004984-olfactory receptor activity
ACC_09939	IPR004117	Olfactory receptor, Drosophila	GO:0004984-olfactory receptor activity
ACC_10028	IPR004117	Olfactory receptor, Drosophila	GO:0004984-olfactory receptor activity

**Gustatory receptor (Gr)**	ACC_01158	IPR009318	Gustatory receptor	GO:0008527-taste receptor activity
	ACC_01311	IPR013604	7TM chemoreceptor	GO:0050909-sensory perception of taste
	ACC_01455	IPR013604	7TM chemoreceptor	GO:0050909-sensory perception of taste
	ACC_02728	IPR013604	7TM chemoreceptor	GO:0050909-sensory perception of taste
	ACC_04028	IPR019424	7TM GPCR, olfactory receptor/chemoreceptor Srsx	GO:0007186-G-protein coupled receptor signaling pathway
	ACC_04999	IPR013604	7TM chemoreceptor	GO:0050909-sensory perception of taste
	ACC_06781	IPR013604	7TM chemoreceptor	GO:0050909-sensory perception of taste
	ACC_08219	IPR013604	7TM chemoreceptor	GO:0050909-sensory perception of taste
	ACC_08325	IPR013604	7TM chemoreceptor	GO:0050909-sensory perception of taste
	ACC_08781	IPR013604	7TM chemoreceptor	GO:0050909-sensory perception of taste

**Pathway**	**Gene**	**KO**	**Product**
**04744 Phototransduction**	ACC_05617	K04439	beta-arrestin
	ACC_09212	K04536	guanine nucleotide-binding protein G(I)/G(S)/G(T) subunit beta-1
	ACC_09530	K13765	regulator of G-protein signaling 9
	ACC_06604	K02183	calmodulin
**04745 Phototransduction - fly**	ACC_04047	K04634	guanine nucleotide-binding protein G(q) subunit alpha
	ACC_03091	K07972	guanine nucleotide-binding protein subunit beta, other
	ACC_06468	K04547	guanine nucleotide-binding protein G(I)/G(S)/G(O) subunit gamma-13
	ACC_02939	K05858	phosphatidylinositol phospholipase C, beta [EC:3.1.4.11]
	ACC_05612	K05858	phosphatidylinositol phospholipase C, beta [EC:3.1.4.11]
	ACC_08163	K05328	transient receptor potential cation channel subfamily C, invertebrate
	ACC_09472	K05328	transient receptor potential cation channel subfamily C, invertebrate
	ACC_08537	K13804	inactivation no afterpotential D protein
	ACC_02607	K02677	classical protein kinase C [EC:2.7.11.13]
	ACC_06604	K02183	calmodulin
	ACC_03486	K08834	myosin III [EC:2.7.11.1]
	ACC_08469	K08834	myosin III [EC:2.7.11.1]
	ACC_04920	K05692	actin beta/gamma 1
	ACC_05085	K05692	actin beta/gamma 1
	ACC_06195	K05692	actin beta/gamma 1
	ACC_08334	K05692	actin beta/gamma 1
	ACC_03645	K13805	arrestin-2
	ACC_05838	K02522	inositol 1,4,5-triphosphate receptor, invertebrate
	ACC_05588	K00910	beta-adrenergic-receptor kinase [EC:2.7.11.15]
	ACC_05678	K04515	calcium/calmodulin-dependent protein kinase (CaM kinase) II [EC:2.7.11.17]
	ACC_01972	K13806	sn1-specific diacylglycerol lipase [EC:3.1.1.-]
**04740 Olfactory transduction**	ACC_04702	K04345	protein kinase A [EC:2.7.11.11]
	ACC_08577	K04345	protein kinase A [EC:2.7.11.11]
	ACC_09607	K07376	protein kinase, cGMP-dependent [EC:2.7.11.12]
	ACC_02640	K08043	adenylate cyclase 3 [EC:4.6.1.1]
	ACC_07908	K08043	adenylate cyclase 3 [EC:4.6.1.1]
	ACC_05678	K04515	calcium/calmodulin-dependent protein kinase (CaM kinase) II [EC:2.7.11.17]
	ACC_06604	K02183	calmodulin
**04742 Taste transduction**	ACC_09212	K04536	guanine nucleotide-binding protein G(I)/G(S)/G(T) subunit beta-1
	ACC_06468	K04547	guanine nucleotide-binding protein G(I)/G(S)/G(O) subunit gamma-13
	ACC_01798	K04632	guanine nucleotide-binding protein G(s) subunit alpha

ACC_01531	K08048	adenylate cyclase 8 [EC:4.6.1.1]
ACC_04702	K04345	protein kinase A [EC:2.7.11.11]
ACC_08577	K04345	protein kinase A [EC:2.7.11.11]

Supplementary Table 23. Homologous genes involved in learning and memory in A. mellifera and A. cerana.

Learning and memory genes in other species	Gene ID	Homologous gene in *Apis mellifera*	Homologous gene in *Apis cerana*
cAMP response element binding protein (CREB)	NP_034082.1	CAD23079.1	ACC_03168
cAMP-dependent protein kinase catalytic subunit	NP_730576.2	-	ACC_08577
cAMP-dependent protein kinase type I regulatory subunit (Pka-R1)	NP_730574.2	XP_396167.1	ACC_00806
cAMP-dependent protein kinase type II regulatory subunit (Pka-R2)	NP_523671.1	XP_392905.2	ACC_02101
Protein kinase C-alpha (PKC-alpha)	NP_476682.1	NP_001128420.1	ACC_02607
Protein kinase C–delta (PKC-delta)	AAF79208.1	XP_394743.3	ACC_04702
Protein kinase C-iota (PKC-iota)	NP_032883.2	XP_397273.4	ACC_07930 and ACC_09509
Protein kinase C and casein kinase substrate in neurons protein 2-like	NP_032884.2	-	ACC_02560
TIC	NP_077338.2	XP_394708.4	ACC_10108
G q/o-protein-family activate phospholipase C (PLC)	NP_995606.1	XP_003250990.1	ACC_02933
CREB binding protein (CBP)	AAB53050.1	XP_001122076.2	ACC_03143
Adenylate cyclase 2 (Adcy2)	NP_705762.2	XP_624596.3	ACC_00796 and ACC_05096
Adenylate cyclase 3 (Adcy3)	NP_612178.2	NP_001071276.1	ACC_02640 and ACC_07908
Adenylate cyclase 5 (Adcy5)	NP_001012783.3	XP_001122246.2	ACC_06514
Adenylate cyclase 6 (Adcy6)	NP_031431.2	XP_001122906.2	ACC_03958
Adenylate cyclase 8 (Adcy8)	NP_033753.2	XP_001122017.2	ACC_01531
Adenylate cyclase 9 (Adcy9)	NP_033754.2	XP_003250461.1	ACC_00311
Adenylate cyclase 10 (Adcy10)	NP_766617.2	XP_003250825.1	ACC_10007
Raf kinase, effector of Ras (Raf)	NP_525047.1	XP_396892.2	ACC_01516
Mitogen-activated protein kinase (MEK)	AAN17594.1	XP_393416.1	ACC_05659
Mitogen-activated protein kinase (MAPK)	NP_001015122.1	XP_393029.2	ACC_00669
Ribosomal S6 kinase (RSK2)	NP_996354.2	XP_393798.4	ACC_05456
Calcium/calmodulin-dependent protein kinase II (CaMKII)	NP_726633.2	NP_001128422.1	ACC_05678

GRB2	NP_032189.1	XP_623354.1	ACC_06551
GRB4	O43639.2	XP_624281.2	ACC_08006
GRB14	NP_057928.1	XP_003249156.1	ACC_08007
Ras	NP_476699.1	XP_394288.4	ACC_09353
Early growth response gene-1 (Elk-1)	NP_523445.1	-	ACC_07111
Tyramine receptor 1 (Tyr1)	NP_650652.1	ABA39280.1	ACC_03553
Tyramine receptor 2 (Tyr2)	BAB71788.1	NP_001011594.1	ACC_08270
N-methyl-D-aspartate receptor 1 (NMDAR1)	NP_730940.1	NP_001011573.1	ACC_03504
N-methyl-D-aspartate receptor 2 (NMDAR2)	NP_001014714.1	XP_396271.3	ACC_06180
Glycine receptor alpha-2	NP_906272.1	XP_623445.2	ACC_07504 and ACC_09119
Glycine receptor alpha-3	P24524.1	XP_396282.4	ACC_08255
Metabotropic glutamate receptor R1	NP_058672.1	-	ACC_09127
Metabotropic glutamate receptor R2	NP_001153825.1	-	ACC_08905
Metabotropic glutamate receptor R4	NP_001013403.1	BAD08344.1	ACC_08322
Metabotropic glutamate receptor R5	NP_001137306.1	-	ACC_08323
Metabotropic glutamate receptor R6	NP_775548.2	-	ACC_08403
Metabotropic glutamate receptor R7	NP_796302.2	XP_395227.4	ACC_08691
Metabotropic glutamate receptor R8	NP_032200.2	-	ACC_01549
Serotonin receptor (5-HT2alpha)	ACZ94804.1	CBX90120.1	ACC_02213
Serotonin receptor (5-HT2beta)	AAI08973.1	CBX90121.1	ACC_06641
Serotonin receptor (5-HT1)	ACZ94473.1	NP_001164579.1	ACC_06642
Serotonin receptor (5-HT7)	CAQ76702.1	-	ACC_06687
Acetylcholine alpha1	NP_524481.2	NP_001091690.1	ACC_01172
Acetylcholine alpha2	NP_524482.1	NP_001011625.1	ACC_04245
Acetylcholine alpha3	AAS90358.1	NP_001073029.1	ACC_10034
Acetylcholine alpha4	AAS90359.1	NP_001091691.1	ACC_04045
Acetylcholine alpha6	CAB53472.1	NP_001073564.1	ACC_10145
Acetylcholine alpha8	AAU12512.1	NP_001011575.1	ACC_03637
Acetylcholine beta1	AAU12514.1	NP_001073028.1	ACC_10012
Acetylcholine beta2	AAS90363.1	NP_001091699.1	ACC_02612
Muscarinic acetylcholine receptor	AAA37158.1	XP_395760.4	ACC_06927

Octopamine receptor (beta-1R)	Q4LBB6.4	-	ACC_07279
Octopamine receptor (beta-2R)	O77408.1	XP_003249217.1	ACC_08867 and ACC_05982
Octopamine receptor (OA1)	NP_524548.1	NP_001011565.1	ACC_08088
Dopamine receptor 1 (DopR1)	NP_001014758.1	NP_001011595.1	ACC_07797
Dopamine receptor 2 (DopR2)	Q9VCZ3.1	NP_001011567.1	ACC_10019
Dopamine receptor 3 (DopR3)	Q4LBB9.2	NP_001014983.1	ACC_04947

Supplementary Table 24. Genes expressed in brains of behavioral phenotypes of workers

	New emerged	Nurse	Guarder	Forager	Dancer	Total
Gene number	9894	9735	9782	9920	9801	10081
Genes detected ⩾2 times	9723	9533	9548	9758	9570	9999

Supplementary Table 25.Number of genes showing significant expression differences in brains of five distinct behavioral phenotypes of *Apis cerana* workers

Up in new-emerged vs.				Up in nurse vs.				Up in guarder vs.				Up in forager vs.				Up in dancer vs.				In total
Nurse	Guarder	Forager	Dancer	New-emerged	Guarder	Forager	Dancer	New-emerged	Nurse	Forager	Dancer	New-emerged	Nurse	Guarder	Dancer	New-emerged	Nurse	Guarder	Forager	
2003	1041	715	1389	78	92	162	437	134	244	215	694	778	2844	1522	945	1225	3783	2437	418	6722
192								11				95				95				

Supplementary Table 26. Number of the up-regulated genes significantly enriched of in GO functional classes during behavior maturation

Behavioral stages compared		Nurse vs. newly-		guarder vs. nurse		Forager vs. nurse		Dancer vs. nurse		Forager vs. guarder		Dancer vs. guarder		Forager vs. Dancer		Dancer vs. forager	
**No. of** up-regulated genes **assigned to GO terms**		17		67		1005		1503		574		1038		233		118	
**GO term**	Expected gene No.	No.	%	No.	%	No.	%	No.	%	No.	%	No.	%	No.	%	No.	%
Energy Metabolism	147		0%	3	2%	87**	59%	109**	74%	58**	39%	89**	61%	5	3%	3	2%
Carbohydrate Metabolism	200	3	2%	7	4%	78*	39%	110*	55%	54**	27%	80*	40%	8	4%	5	3%
Lipid Metabolism	141	2	1%	6	4%	49	35%	82**	58%	35	25%	68**	48%	6	4%	6	4%
Amino Acid Metabolism	140		0%	9*	6%	57*	41%	82**	59%	32	23%	54	39%	6	4%	4	3%
Metabolism of Cofactors and Vitamins	94		0%	5	5%	31	33%	62**	66%	18	19%	40*	43%		0%	5	5%
Glycan Biosynthesis and Metabolism	119	3	3%	2	2%	26	22%	64	54%	13	11%	33	28%	8	7%	17**	14%
N-Glycan biosynthesis	15		0%		0%	1	7%	11*	73%		0%	8	53%		0%	4**	27%
Circulatory System	61		0%	1	2%	39**	64%	26	43%	19*	31%	20	33%	12**	20%		0%
Digestive System	85	1	1%	2	2%	38*	45%	29	34%	20	24%	19	22%	15**	18%	2	2%
Excretory System	67		0%	2	3%	29*	43%	24	36%	12	18%	17	25%	9	13%	2	3%
Sensory System	31	1	3%	1	3%	14	45%	3	10%	7	23%	3	10%	11**	35%		0%
Nervous System	158		0%	4	3%	61*	39%	45	28%	24	15%	30	19%	25**	16%	5	3%
Signal Transduction	310	1	0%	13	4%	99	32%	103	33%	47	15%	57	18%	35**	11%	3	1%
Cell Communication	114		0%	2	2%	42	37%	22	19%	24	21%	13	11%	23**	20%		0%
Cell Motility	52		0%	1	2%	10	19%	11	21%	8	15%	8	15%	8*	15%		0%
Replication and Repair	106		0%	4	4%	21	20%	63**	59%	5	5%	38	36%	2	2%	8	8%
Transcription	161		0%	6	4%	63*	39%	83	52%	24	15%	48	30%	3	2%	4	2%
Transcription-Eukaryotic Type	166	1	1%	7	4%	36	22%	16	10%	12	7%	6	4%	45**	27%	1	1%
Translation	318		0%	1	0%	77	24%	151	47%	81**	25%	131**	41%	11	3%	11	3%
Folding, Sorting and Degradation	314		0%	7	2%	91	29%	157	50%	54	17%	129**	41%	5	2%	5	2%
Xenobiotics Biodegradation and Metabolism	49	2	4%	1	2%	24*	49%	31*	63%	14	29%	25*	51%	1	2%	2	4%
GPCR-Class A. Rhodopsin family	23	3*	13%	1	4%	12*	52%	7	30%	7	30%	6	26%	6**	26%		
Eukaryotic cytoskeleton proteins	88	1	1%	3	3%	24	27%	24	27%	17	19%	12	14%	13**	15%	3	3%
Signaling Molecules and Interaction	54		0%	2	4%	27*	50%	12	22%	8	15%	8	15%	16**	30%		0%
Tyrosine protein kinases	19		0%	1	5%	5	26%	4	21%	3	16%		0%	4*	21%	1	5%
Serine/threonine protein kinases: AGC group	24		0%	1	4%	14*	58%	5	21%	5	21%	4	17%	3*	13%		0%
Spliceosome-Complex C	88		0%	2	2%	38*	43%	51*	58%	13	15%	27	31%	1	1%	1	1%
Spliceosome-Complex B	92		0%	2	2%	41*	45%	47	51%	16	17%	27	29%	1	1%	2	2%
Aminoacyl-tRNA synthetase	35		0%	1	3%	2	6%	23*	66%	3	9%	19*	54%		0%	4	11%
tRNA-Eukaryotic Type	118		0%	1	1%	20	17%	73**	62%	17	14%	55**	47%	2	2%	13**	11%
Ribosomal Proteins	75		0%		0%	17	23%	14	19%	46**	61%	35*	47%	4	5%		0%
Proteasome-Eukaryotic proteasome	22		0%		0%	13*	59%	19**	86%	7	32%	16**	73%		0%		0%
Threonine Peptidases	17		0%		0%	9	53%	12*	71%	6	35%	10*	59%		0%	1	6%
Number in bold differed significantly from null hypothesis.*, RST＜0.05; **, RST＜0.001																	

Supplementary Table 27. The number of up-regulated genes significantly enriched in KEGG pathways during worker behavior maturation

		Nurse vs. newly-		guarder vs. nurse		Forager vs. nurse		Dancer vs. nurse		Forager vs. guarder		Dancer vs. guarder		Forager vs. Dancer		Dancer vs. forager	
**KEGG pathways**	Expected gene No.	No.	%	No.	%	No.	%	No.	%	No.	%	No.	%	No.	%	No.	%
00010 Glycolysis / Gluconeogenesis	33		0%	1	3%	11	33%	16	48%	3	9%	12	36%	1	3%		0%
00020 Citrate cycle (TCA cycle)	34		0%		0%	11	32%	22	65%	8	24%	14	41%		0%	1	3%
00071 Fatty acid metabolism	24		0%	2	8%	9	38%	15	63%	7	29%	15*	63%		0%	1	4%
**00190 Oxidative phosphorylation**	**88**		**0%**		**0%**	**62****	**70%**	**71****	**81%**	**39****	**44%**	**59****	**67%**	**4**	**5%**	**1**	**1%**
00230 Purine metabolism	101		0%	4	4%	37	37%	48	48%	11	11%	36	36%	7	7%	4	4%
00240 Pyrimidine metabolism	71		0%	3	4%	19	27%	41	58%	10	14%	30	42%		0%	3	4%
00280 Valine, leucine and isoleucine degradation	30		0%	1	3%	11	37%	24**	80%	8	27%	18*	60%		0%		0%
00380 Tryptophan metabolism	20		0%	2	10%	11	55%	13	65%	9*	45%	10	50%		0%		0%
00480 Glutathione metabolism	25	1*	4%		0%	11	44%	14	56%	6	24%	14	56%	1	4%	1	4%
00510 N-Glycan biosynthesis	34		0%		0%	3	9%	18	53%		0%	15	44%	1	3%	5	15%
00561 Glycerolipid metabolism	27		0%	1	4%	8	30%	14	52%	8	30%	11	41%	2	7%		0%
00563 Glycosylphosphatidylinositol(GPI)-anchor biosynthesis	19		0%		0%	3	16%	18**	95%		0%	6	32%		0%	7**	37%
00640 Propanoate metabolism	19		0%	1	5%	7	37%	13	68%	5	26%		0%	1	5%	1	5%
00650 Butanoate metabolism	14		0%	1	7%	8	57%	14**	100%	4	29%	10*	71%		0%		0%
00970 Aminoacyl-tRNA biosynthesis	39		0%	1	3%	2	5%	24	62%	3	8%	20	51%		0%	4	10%
00980 Metabolism of xenobiotics by cytochrome P450	12	1	8%		0%	7	58%	9	75%	5	42%	9*	75%		0%	1	8%
03008 Ribosome biogenesis in eukaryotes	64		0%		0%	18	28%	44*	69%	9	14%	29	45%		0%	5	8%
03010 Ribosome	76		0%		0%	17	22%	15	20%	46**	61%	35	46%	4	5%		0%
03013 RNA transport	110		0%		0%	31	28%	61	55%	20	18%	39	35%	3	3%	4	4%
03018 RNA degradation	55		0%	1	2%	17	31%	32	58%	9	16%	22	40%		0%	1	2%
03020 RNA polymerase	26		0%	2	8%	6	23%	14	54%	3	12%	11	42%		0%	1	4%
03040 Spliceosome	102		0%	2	2%	49**	48%	53	52%	19	19%	30	29%	3	3%	2	2%
03050 Proteasome	37		0%		0%	20*	54%	29*	78%	12	32%	25*	68%		0%	1	3%
03420 Nucleotide excision repair	38		0%	2	5%	10	26%	24	63%	3	8%	15	39%		0%		0%
03430 Mismatch repair	21		0%	1	5%	6	29%	14	67%	2	10%	8	38%		0%	1	5%
03440 Homologous recombination	23		0%		0%	6	26%	18*	78%	2	9%	10	43%		0%	4	17%
03460 Fanconi anemia pathway	32		0%		0%	5	16%	22	69%	1	3%	9	28%		0%	2	6%
04020 Calcium signaling pathway	44	1*	2%	2	5%	19	43%	14	32%	9	20%	7	16%	10**	23%	2	5%
04062 Chemokine signaling pathway	45		0%		0%	16	36%	9	20%	6	13%	5	11%	8	18%	1	2%
04080 Neuroactive ligand-receptor interaction	33		0%	1	3%	20**	61%	9	27%	6	18%	7	21%	10**	30%		0%
04111 Cell cycle - yeast	53		0%		0%	9	17%	29	55%	4	8%	19	36%		0%	4	8%
04140 Regulation of autophagy	13		0%		0%	2	15%	9	69%	1	8%	5	38%		0%		0%
04142 Lysosome	57	3**	5%	2	4%	24	42%	29	51%	13	23%	20	35%	3	5%	2	4%
04146 Peroxisome	51		0%		0%	14	27%	33	65%	10	20%	22	43%		0%	4	8%
04150 mTOR signaling pathway	32		0%	1	3%	11	34%	12	38%	4	13%	7	22%	3	9%	1	3%
04310 Wnt signaling pathway	58		0%	1	2%	17	29%	13	22%	8	14%	9	16%	13**	22%	1	2%
04510 Focal adhesion	53		0%	1	2%	14	26%	6	11%	8	15%	3	6%	10**	19%		0%
04520 Adherens junction	27		0%	1	4%	7	26%	5	19%	6	22%	2	7%	6	22%		0%
04530 Tight junction	41		0%		0%	16	39%	11	27%	12	29%	8	20%	7	17%		0%

04540 Gap junction	32		0%		0%	17*	53%	8	25%	11	34%	4	13%	9**	28%		0%
04720 Long-term potentiation	27		0%	1	4%	11	41%	6	22%	4	15%	4	15%	7**	26%	1	4%
04723 Retrograde endocannabinoid signaling	25		0%		0%	15*	60%	6	24%	4	16%	5	20%	7**	28%		0%
04724 Glutamatergic synapse	36		0%	2	6%	18	50%	10	28%	5	14%	6	17%	11**	31%	1	3%
04725 Cholinergic synapse	35		0%	1	3%	15	43%	6	17%	5	14%	4	11%	9**	26%		0%
04726 Serotonergic synapse	27		0%		0%	12	44%	5	19%	4	15%	2	7%	6	22%		0%
04727 GABAergic synapse	32		0%	2	6%	17*	53%	11	34%	5	16%	7	22%	7**	22%		0%
04728 Dopaminergic synapse	44		0%	1	2%	17	39%	8	18%	5	11%	5	11%	11**	25%		0%
04745 Phototransduction - fly	21	1*	5%	1	5%	8	38%	2	10%	5	24%	2	10%	8**	38%		0%
04810 Regulation of actin cytoskeleton	52		0%	1	2%	10	19%	11	21%	8	15%	8	15%	8	15%		0%
04910 Insulin signaling pathway	61		0%	2	3%	14	23%	12	20%	5	8%	9	15%		0%	1	2%
04912 GnRH signaling pathway	31		0%		0%	13	42%	8	26%	6	19%	6	19%	6	19%		0%
04916 Melanogenesis	31		0%		0%	9	29%	3	10%	5	16%	2	6%	8**	26%		0%

Number in bold differed significantly from null hypothesis.*, RST＜0.05; **, RST＜0.001

Supplementary table 28. Transcriptional abundance of differential expressed genes in brains of different behavioral phenotypes

Role	**Protein**	**Gene**	**Accumulated tag counts (normalized)**				
			Newly-emerged	Nurse	Guard	Forager	Dancer
Key protein for oxidative phosphorylation	Mitochondrial ADP/ATP carrier protein	ACC_05937, ACC_02107	1500	490	542	1129	828
	Mitochondrial phosphate carrier protein	ACC_06473	566	161	217	423	460
	ATP synthase subunit	ACC_00155, ACC_01144, ACC_00488, ACC_00906, ACC_03925, ACC_04477, ACC_04411, ACC_09748, ACC_00544, ACC_09601, ACC_04040, ACC_03885, ACC_07265, ACC_07299, ACC_02532, ACC_04018, ACC_01807, ACC_06665, ACC_04874, ACC_03027, ACC_02598, ACC_07988, ACC_00381, ACC_04514, ACC_03708, ACC_00173, ACC_05565, ACC_01843, ACC_06360, ACC_00552, ACC_03674	7688	3381	4379	7189	9315
Rate limiting enzyme in β-oxidation	Carnitine O-palmitoyltransferase 2	ACC_01384	67	34	44	57	100
	Carnitine O-palmitoyltransferase 1	ACC_00707, ACC_03563	260	107	88	135	190
Rate limiting enzyme in glycolysis	Hexokinase	ACC_06489,ACC_05858,ACC_07907	388	275	274	348	436
	6-**phosphofructokinase**	ACC_02919	303	143	184	144	258
	Pyruvate kinase	ACC_05844	542	249	330	606	849
Rate limiting enzyme in TCA cycle	**Citrate synthase**	ACC_02507,ACC_02680,ACC_00474	597	177	212	358	527
	Isocitrate dehydrogenase	ACC_03571,ACC_11681,X252	397	151	203	318	463
	2-oxoglutarate dehydrogenase complex	ACC_00511,ACC_08054,ACC_03977,ACC_11751	666	323	421	438	515
Key enzyme for gluconeogenesis	Phosphoenolpyruvate carboxykinase	ACC_09737, ACC_10287	1	0	0	4	2
	Pyruvate carboxylase	ACC_05268	102	58	80	113	57
Rate limiting enzyme for fatty acid biosynthesis	Acetyl-CoA carboxylase	ACC_09855	199	114	71	72	75
Insulin-signaling	Insulin-like peptide (ILP)	ACC_04091, X194	12	8	19	22	18
	Insulin-like peptide receptor (InR)	ACC_03454, ACC_12839	121	40	92	169	82
	Insulin receptor substrate (IRS)	ACC_04251	59	56	64	32	23
	Insulin-like growth factor-binding protein complex acid labile subunit	ACC_10591, ACC_11440, ACC_11531, ACC_08541, ACC_08618, ACC_08850, ACC_09193	202	133	177	278	356
	Insulin-degrading enzymes	ACC_08071, ACC_01087, ACC_01354, ACC_03524, ACC_05250, ACC_02622	940	511	628	927	1227
AKH-signaling	Adipokinetic prohormone type (AKH)	ACC_10016	14	8	22	49	29
	Adipokinetic hormone receptor (AKHR)	ACC_03261	6	2	1	4	6
	Triacylglycerol lipase	ACC_11145, ACC_04384, ACC_06430, ACC_12366, ACC_12624-2	37	36	106	173	166
	Glycogen phosphorylase	ACC_05000	342	194	178	357	487
	Anaplastic lymphoma kinase (**Alk**)	ACC_01344	422	253	426	405	251
	Phosphatidylinositol-3,4,5-trisphosphate 3-phosphatase and dual-specificity protein phosphatase **PTEN**	ACC_07681	40	25	30	23	20

TOR signaling	Cationic amino acid transporter (**Slif**)	ACC_06100, ACC_12724	244	115	176	311	213
	Ras-related GTP-binding protein A (**RagA**)	ACC_00995	40	26	34	45	57
	Phosphatidylinositol 3-kinase subunit (**PI3K**)	ACC_12640, ACC_07053, ACC_05283, ACC_05929	496	289	341	558	455
	3-phosphoinositide-dependent protein kinase (**PDK1**)	ACC_00784	87	37	79	96	30
	RAC serine/threonine-protein kinase (**AKT**)	ACC_06492	48	22	32	70	88
	Hamartin (**TSC1**)	ACC_02115	155	100	118	160	188
	Tuberin (**TSC2**)	ACC_08073	172	114	128	115	157
	GTP-binding protein **Rheb**	ACC_07108	107	77	131	137	165
	Target of rapamycin complex subunit LST8 (**GβL**)	ACC_04696	78	51	68	94	124
	Serine/threonine-protein kinase **mTOR**	ACC_07570	121	77	92	115	133
	Regulatory-associated protein of mTOR (**Raptor**)	ACC_09351	139	82	86	95	112
	Rapamycin-insensitive companion of mTOR (**Rictor**)	ACC_09585	120	75	101	184	151
	Hypoxia-inducible factor 1-alpha (**HIF1α**)	ACC_08923	312	157	207	359	125
	Vascular endothelial growth factor B (**VEGF**)	ACC_03459	82	76	84	268	246
	Ribosomal protein S6 kinase beta-1 (**S6K1/2**)	ACC_01751, ACC_01765	133	68	69	66	72
	40S ribosomal protein **S6**	ACC_11223	931	768	556	922	698
	Eukaryotic translation initiation factorr 4B (**eIF4 B**)	ACC_01729	91	50	46	85	88
	Eukaryotic translation initiation factor 4E (**eIF4E**)	ACC_08894, ACC_02955, ACC_03208	140	106	115	181	234
	Serine/threonine-protein kinase ULK (**ATG1**)	ACC_02805, ACC_12718	201	114	145	195	189
	Mitogen-activated protein kinase 1(**ERK1/2**)	ACC_06961	140	59	66	80	95
	Ribosomal protein S6 kinase alpha-3(**RSK**)	ACC_07128, ACC_06700	180	66	84	185	207
	AMP-activated protein kinase subunit (**AMPK**)	ACC_02186, ACC_04086, ACC_02519	341	183	234	368	461
	Serine/threonine-protein kinase STK11 like (**LKB1**)	X027	7	4	4	12	16
	Protein **Mo25**-like	ACC_07091	85	62	68	139	145
	Raf homolog serine/threonine-protein kinase phl (**BRAF**)	ACC_01852	21	13	17	10	12
Juvenile hormone signaling	Farnesyl pyrophosphate synthase	ACC_09253, ACC_09299, ACC_09493, ACC_09539, ACC_09553	206	196	173	161	224
	Juvenile hormone epoxide hydrolase	ACC_04997	66	59	52	104	114
	juvenile hormone acid methyltransferase	ACC_04742, ACC_11539	294	271	304	579	534

	juvenile hormone esterase-like	ACC_11476	8	4	4	9	8
	Ultraspiracle	ACC_08652	56	28	51	57	32
Storage protein	Vitellogenin precursor	ACC_05847	170	505	91	123	70
	Vitellogenin receptor	ACC_06990	5	6	3	4	5
	Hexamerin	ACC_09651, X096, ACC_06582	102	132	47	61	32
	cGMP-dependent protein kinase 1 , foraging	ACC_12651	40	23	28	51	42
	Protein Malvolio	ACC_10747	91	44	58	96	174
	Period circadian protein	ACC_12767	107	151	105	89	57
	Neuropeptide Y	ACC_04782	2	1	6	37	25
	Neuropeptide Y receptor	ACC_08915, ACC_00968, ACC_12887	49	22	25	38	43
	Short neuropeptide F	ACC_09097	8	8	15	43	28
	Cytoplasmic polyadenylation element-binding protein (CPEB1)	ACC_08895	8	7	10	9	10
	cAMP-dependent protein kinase or protein kinase A (PKA)						
	Cyclic AMP-responsive element-binding protein (CREB 1)						
synaptic plasticity and memory	Beta-amyloid-like protein	ACC_05795	1236	758	999	922	332
	Amyloid protein-binding proteins	ACC_12676, ACC_04880, ACC_11086, X143, ACC_10157	514	268	377	603	442
	Tyramine beta-hydroxylase	ACC_07148	10	9	17	20	24
	Octopamine receptor	ACC_11581, ACC_07354, ACC_09255, ACC_10450	114	49	75	206	78
	Dopamine N-acetyltransferase	ACC_10784, ACC_03707, ACC_03801, ACC_12897	299	174	295	520	606
	Dopamine beta-monooxygenases	ACC_09813, ACC_10939, ACC_11137, ACC_12279, ACC_07148	132	104	212	470	275
	Dopamine receptors	X221, ACC_06080, ACC_10024, ACC_11794, ACC_11793, ACC_07608	96	61	89	83	48
	Serotonin receptor	ACC_06080, ACC_08264, ACC_08265	15	5	4	11	7
	5-hydroxytryptamine receptors	ACC_08264, ACC_08265, ACC_08336, ACC_02691, ACC_11164, ACC_12428	131	68	105	119	105
	Gamma-aminobutyric acid receptors	ACC_11846, ACC_08456, ACC_09140, ACC_09665, ACC_09666, ACC_11493, ACC_01849, ACC_10438, ACC_10506, ACC_12828, ACC_09667	1001	414	568	923	580
	Gamma-aminobutyric acid receptor-associated protein	ACC_03088	439	274	320	731	652
	Acetylcholine receptors	ACC_04450, ACC_08030, ACC_09269, ACC_10556, ACC_11026, ACC_11781, X239, M18, ACC_01433, ACC_04952, ACC_05191, ACC_02734, X214, ACC_08685, ACC_08322, ACC_01425	1030	539	610	968	603
	Acetylcholine receptor-associated protein of the synapse (rapsyn)	ACC_08590	205	76	84	226	167
	Acetylcholinesterases	ACC_00365, ACC_03180, ACC_09461, ACC_09696, ACC_10130, ACC_10166, ACC_10501, ACC_10962, ACC_12781, ACC_12869, ACC_12899, ACC_05397	706	350	525	686	345

Supplementary Table 29. Protease/Peptidase in *A.cerana*

Gene	Clan	Family	Subfamily	Evalue	Annotation	Subcellular Localization
ACC_01720	AA	A11	A11A	2.20E-13	conserved hypothetical protein	Cytoplasmic
ACC_01718	AA	A11	A11X	9.90E-35	Copia protein	Cytoplasmic
ACC_05062	AA	A11	A11X	1.20E-12	Retrovirus-related Pol polyprotein from transposon TNT	Cytoplasmic
ACC_05080	AA	A11	A11X	7.50E-29	Retrovirus-related Pol polyprotein from transposon TNT	Cytoplasmic
ACC_05368	AA	A11	A11X	3.80E-15	Uncharacterized mitochondrial protein AtMg00820	Cytoplasmic
ACC_08580	AD	A22	A22A	1.50E-101	Presenilin homolog	Cytoplasmic
ACC_08815	CA	C1	C01A	1.60E-41	Cathepsin J	Cytoplasmic
ACC_05077	CA	C1	C01A	2.40E-18	Growth/differentiation factor	Cytoplasmic
ACC_09298	CA	C1	C01B	2.10E-149	Bleomycin hydrolase	Cytoplasmic
ACC_00900	CA	C2	C02A	7.20E-95	Calpain-7	Cytoplasmic
ACC_05958	CA	C2	C02A	3.90E-28	Calpain-7-like protein	Cytoplasmic
ACC_01111	CA	C2	C02A	9.70E-135	Calpain-A	Cytoplasmic
ACC_03554	CA	C2	C02A	9.90E-137	Calpain-D	Cytoplasmic
ACC_05378	CA	C12		6.30E-60	Ubiquitin carboxyl-terminal hydrolase	Cytoplasmic
ACC_01927	CA	C12		4.30E-99	Ubiquitin carboxyl-terminal hydrolase BAP1	Cytoplasmic
ACC_05927	CA	C12		2.50E-99	Ubiquitin carboxyl-terminal hydrolase isozyme L5	Cytoplasmic
ACC_01151	CD	C14	C14A	1.20E-56	Caspase	Cytoplasmic
ACC_00475	CD	C14	C14A	1.30E-36	Caspase Nc	Cytoplasmic
ACC_01488	CD	C14	C14A	6.00E-107	Caspase-1	Cytoplasmic
ACC_03684	CD	C14	C14A	2.40E-22	Caspase-8	Cytoplasmic
ACC_06314	CD	C14	C14B	4.40E-20	Mucosa-associated lymphoid tissue lymphoma translocation protein	Cytoplasmic
ACC_04459	CF	C15		2.10E-24	Pyroglutamyl-peptidase	Cytoplasmic
ACC_08814	CA	C19		3.80E-83	Inactive ubiquitin carboxyl-terminal hydrolase	Cytoplasmic
ACC_00289	CA	C19		3.60E-55	PAB-dependent poly(A)-specific ribonuclease subunit	Cytoplasmic
ACC_00371	CA	C19		1.00E-175	Probable ubiquitin carboxyl-terminal hydrolase FAF-X	Cytoplasmic
ACC_00513	CA	C19		1.40E-167	U4/U6.U5 tri-snRNP-associated protein	Cytoplasmic
ACC_00191	CA	C19		1.00E-88	Ubiquitin carboxyl-terminal hydrolase	Cytoplasmic
ACC_00424	CA	C19		2.00E-103	Ubiquitin carboxyl-terminal hydrolase	Cytoplasmic
ACC_00936	CA	C19		5.20E-24	Ubiquitin carboxyl-terminal hydrolase	Cytoplasmic
ACC_01240	CA	C19		8.10E-92	Ubiquitin carboxyl-terminal hydrolase	Cytoplasmic
ACC_01325	CA	C19		3.60E-111	Ubiquitin carboxyl-terminal hydrolase	Cytoplasmic
ACC_01505	CA	C19		2.80E-115	Ubiquitin carboxyl-terminal hydrolase	Cytoplasmic
ACC_03406	CA	C19		2.70E-164	Ubiquitin carboxyl-terminal hydrolase	Cytoplasmic
ACC_08830	CA	C19		5.20E-111	Ubiquitin carboxyl-terminal hydrolase	Cytoplasmic
ACC_08864	CA	C19		1.10E-97	Ubiquitin carboxyl-terminal hydrolase	Cytoplasmic
ACC_09075	CA	C19		1.60E-162	Ubiquitin carboxyl-terminal hydrolase	Cytoplasmic
ACC_09246	CA	C19		6.10E-162	Ubiquitin carboxyl-terminal hydrolase	Cytoplasmic

ACC_09715	CA	C19		2.20E-35	Ubiquitin carboxyl-terminal hydrolase	Cytoplasmic
ACC_10021	CA	C19		2.10E-147	Ubiquitin carboxyl-terminal hydrolase	Cytoplasmic
ACC_04263	CA	C19		5.50E-148	Ubiquitin carboxyl-terminal hydrolase	Cytoplasmic
ACC_04503	CA	C19		6.90E-142	Ubiquitin carboxyl-terminal hydrolase	Cytoplasmic
ACC_04577	CA	C19		8.60E-30	Ubiquitin carboxyl-terminal hydrolase	Cytoplasmic
ACC_05388	CA	C19		5.90E-111	Ubiquitin carboxyl-terminal hydrolase	Cytoplasmic
ACC_05398	CA	C19		1.20E-90	Ubiquitin carboxyl-terminal hydrolase	Cytoplasmic
ACC_06449	CA	C19		5.30E-144	Ubiquitin carboxyl-terminal hydrolase	Cytoplasmic
ACC_06976	CA	C19		8.20E-48	Ubiquitin carboxyl-terminal hydrolase	Cytoplasmic
ACC_07017	CA	C19		4.60E-215	Ubiquitin carboxyl-terminal hydrolase	Cytoplasmic
ACC_09678	PC	C26		4.80E-124	CAD protein	Cytoplasmic
ACC_03991	PC	C26		5.40E-76	GMP synthase [glutamine-hydrolyzing]	Cytoplasmic
ACC_01671	PB	C44		8.70E-102	Amidophosphoribosyltransferase	Cytoplasmic
ACC_05528	PB	C44		1.60E-60	Asparagine synthetase [glutamine-hydrolyzing]	Cytoplasmic
ACC_05676	PB	C44		2.80E-113	Glucosamine--fructose-6-phosphate aminotransferase [isomerizing]	Cytoplasmic
ACC_01676	PB	C44		3.50E-89	Putative glutamate synthase [NADPH]	Cytoplasmic
ACC_04276	PD	C46		3.20E-40	Sonic hedgehog protein A	Cytoplasmic
ACC_01584	CE	C48		2.00E-31	Sentrin-specific protease	Cytoplasmic
ACC_02007	CE	C48		5.40E-24	Sentrin-specific protease	Cytoplasmic
ACC_07687	CE	C48		2.80E-58	Sentrin-specific protease	Cytoplasmic
ACC_05783	CA	C54		1.50E-86	Cysteine protease ATG4B	Cytoplasmic
ACC_01925	CA	C54		2.60E-67	Cysteine protease ATG4D	Cytoplasmic
ACC_03821	PC	C56		2.00E-35	Phosphoribosylformylglycinamidine synthase	Cytoplasmic
ACC_00400	PC	C56		1.60E-24	Protein DJ-1	Cytoplasmic
ACC_03146	CA	C64		2.70E-71	OTU domain-containing protein 7B	Cytoplasmic
ACC_01610	CA	C64		1.30E-81	Ubiquitin thioesterase trabid	Cytoplasmic
ACC_01174	CA	C65		5.00E-83	Ubiquitin thioesterase otubain-like	Cytoplasmic
ACC_07906	CA	C67		7.10E-93	Probable ubiquitin carboxyl-terminal hydrolase CYLD	Cytoplasmic
ACC_07679	PA	C74		4.80E-21	DnaJ homolog subfamily C member	Cytoplasmic
ACC_06692	CA	C78		3.20E-76	Probable Ufm1-specific protease	Cytoplasmic
ACC_05601	CA	C78		2.60E-48	Ufm1-specific protease	Cytoplasmic
ACC_06118	CA	C85		3.90E-22	Nuclear factor related to kappa-B-binding protein	Cytoplasmic
ACC_06017	CA	C85		4.70E-45	OTU domain-containing protein	Cytoplasmic
ACC_03462	CA	C86		9.50E-78	Ataxin-3	Cytoplasmic
ACC_04852	CA	C86		2.00E-50	Josephin-like protein	Cytoplasmic
ACC_09710	MA	M1		1.70E-60	Glutamyl aminopeptidase	Cytoplasmic
ACC_06193	MA	M1		2.10E-109	Puromycin-sensitive aminopeptidase	Cytoplasmic
ACC_07678	MA	M1		8.90E-54	Transcription initiation factor TFIID subunit	Cytoplasmic
ACC_07726	MA	M2		2.00E-11	Multiple epidermal growth factor-like domains protein	Cytoplasmic
ACC_00810	MA	M3	M03A	2.20E-187	Mitochondrial intermediate peptidase	Cytoplasmic

ACC_05272	MA	M3	M03A	2.40E-95	Oligopeptidase A	Cytoplasmic
ACC_05706	MA	M8		2.20E-257	Leishmanolysin-like peptidase	Cytoplasmic
ACC_07319	MA	M10	M10A	3.50E-71	Matrix metalloproteinase-14	Cytoplasmic
ACC_06153	MA	M10	M10A	1.30E-49	Matrix metalloproteinase-16	Cytoplasmic
ACC_08195	MA	M12	M12A	2.30E-100	Tolloid-like protein	Cytoplasmic
ACC_00473	MA	M12	M12A	2.00E-35	Zinc metalloproteinase nas-13	Cytoplasmic
ACC_08010	MA	M12	M12A	3.10E-38	Zinc metalloproteinase nas-15	Cytoplasmic
ACC_07640	MA	M12	M12A	5.30E-38	Zinc metalloproteinase nas-15	Cytoplasmic
ACC_07090	MA	M12	M12B	1.20E-13	A disintegrin and metalloproteinase with thrombospondin motifs	Cytoplasmic
ACC_07506	MA	M12	M12B	3.70E-66	A disintegrin and metalloproteinase with thrombospondin motifs	Cytoplasmic
ACC_07785	MA	M12	M12B	4.60E-40	A disintegrin and metalloproteinase with thrombospondin motifs	Cytoplasmic
ACC_07062	MA	M12	M12B	1.00E-70	ADAM 17-like protease	Cytoplasmic
ACC_08483	MA	M12	M12B	1.60E-107	Disintegrin and metalloproteinase domain-containing protein	Cytoplasmic
ACC_08702	MA	M12	M12B	1.10E-116	Disintegrin and metalloproteinase domain-containing protein	Cytoplasmic
ACC_08750	MA	M12	M12B	1.10E-116	Disintegrin and metalloproteinase domain-containing protein	Cytoplasmic
ACC_09454	MA	M12	M12B	1.20E-24	Disintegrin and metalloproteinase domain-containing protein	Cytoplasmic
ACC_03875	MA	M13		4.70E-38	Endothelin-converting enzyme	Cytoplasmic
ACC_01695	MA	M13		5.60E-56	Membrane metallo-endopeptidase-like	Cytoplasmic
ACC_03071	MA	M13		4.70E-62	Membrane metallo-endopeptidase-like	Cytoplasmic
ACC_06750	MA	M13		9.40E-43	Membrane metallo-endopeptidase-like	Cytoplasmic
ACC_07595	MA	M13		7.80E-124	Neprilysin-2	Cytoplasmic
ACC_03624	MC	M14	M14A	3.30E-82	Carboxypeptidase B	Cytoplasmic
ACC_09792	MC	M14	M14A	1.90E-95	Carboxypeptidase B	Cytoplasmic
ACC_07751	MC	M14	M14A	2.70E-77	Tensin	Cytoplasmic
ACC_04973	MC	M14	M14B	3.40E-175	Carboxypeptidase M	Cytoplasmic
ACC_08164	MC	M14	M14B	4.00E-39	Cytosolic carboxypeptidase	Cytoplasmic
ACC_08493	MC	M14	M14B	3.40E-13	Cytosolic carboxypeptidase	Cytoplasmic
ACC_05561	MC	M14	M14B	1.30E-64	Cytosolic carboxypeptidase	Cytoplasmic
ACC_01413	MC	M14	M14B	1.90E-61	Cytosolic carboxypeptidase-like protein	Cytoplasmic
ACC_08217	MC	M14	M14X	1.10E-44	Cytosolic carboxypeptidase	Cytoplasmic
ACC_04290	ME	M16	M16A	1.80E-76	Insulin-degrading enzyme	Cytoplasmic
ACC_02880	ME	M16	M16A	2.10E-57	Nardilysin	Cytoplasmic
ACC_05047	ME	M16	M16B	1.30E-83	Mitochondrial-processing peptidase subunit alpha	Cytoplasmic
ACC_00897	ME	M16	M16B	1.10E-40	Mitochondrial-processing peptidase subunit beta	Cytoplasmic

ACC_01117	ME	M16	M16B	2.50E-99	Mitochondrial-processing peptidase subunit beta	Cytoplasmic
ACC_06501	ME	M16	M16C	6.10E-145	Presequence protease, mitochondrial	Cytoplasmic
ACC_02157	ME	M16	M16C	8.20E-86	Uncharacterized protein C05D11.1	Cytoplasmic
ACC_06440	MF	M17		5.30E-125	Cytosol aminopeptidase	Cytoplasmic
ACC_06558	MJ	M19		4.00E-89	Dipeptidase	Cytoplasmic
ACC_06270	MH	M20	M20A	1.00E-119	Aminoacylase-1	Cytoplasmic
ACC_05377	MH	M20	M20A	2.00E-196	Cytosolic non-specific dipeptidase	Cytoplasmic
ACC_05547	MK	M22		5.00E-11	Acylphosphatase-1	Cytoplasmic
ACC_05910	MK	M22		2.90E-134	Probable O-sialoglycoprotein endopeptidase	Cytoplasmic
ACC_07755	MK	M22		3.50E-54	Probable O-sialoglycoprotein endopeptidase	Cytoplasmic
ACC_02163	MK	M22		7.20E-16	YrdC domain-containing protein, mitochondrial	Cytoplasmic
ACC_00122	MG	M24	M24A	9.20E-74	Methionine aminopeptidase	Cytoplasmic
ACC_03765	MG	M24	M24A	1.90E-152	Methionine aminopeptidase	Cytoplasmic
ACC_02527	MG	M24	M24A	3.30E-80	Methionine aminopeptidase 1D, mitochondrial	Cytoplasmic
ACC_01672	MG	M24	M24B	1.40E-78	Probable Xaa-Pro aminopeptidase	Cytoplasmic
ACC_03131	MG	M24	M24B	4.10E-91	Xaa-Pro aminopeptidase	Cytoplasmic
ACC_07267	MG	M24	M24B	6.00E-60	Xaa-Pro aminopeptidase	Cytoplasmic
ACC_02080	MG	M24	M24B	2.40E-108	Xaa-Pro dipeptidase	Cytoplasmic
ACC_07863	MG	M24	M24X	2.80E-85	FACT complex subunit spt16	Cytoplasmic
ACC_04747	MG	M24	M24X	1.90E-116	Proliferation-associated protein 2G4	Cytoplasmic
ACC_07472	MH	M28	M28A	3.40E-67	Glutaminyl-peptide cyclotransferase	Cytoplasmic
ACC_04282	MH	M28	M28B	1.60E-60	Plasma glutamate carboxypeptidase	Cytoplasmic
ACC_03366	MJ	M38		7.50E-118	Dihydropyrimidinase	Cytoplasmic
ACC_02358	MJ	M38		1.00E-68	Guanine deaminase	Cytoplasmic
ACC_05995	MJ	M38		1.90E-112	Putative N-acetylglucosamine-6-phosphate deacetylase	Cytoplasmic
ACC_08838	MA	M41		5.00E-29	Protein YME1 homolog	Cytoplasmic
ACC_01178	MA	M49		4.30E-64	Dipeptidyl peptidase	Cytoplasmic
ACC_07683	MA	M49		1.40E-65	Dipeptidyl peptidase	Cytoplasmic
ACC_00480	MP	M67	M67A	4.40E-101	26S proteasome non-ATPase regulatory subunit	Cytoplasmic
ACC_03908	MP	M67	M67A	1.80E-57	26S proteasome non-ATPase regulatory subunit	Cytoplasmic
ACC_08041	MP	M67	M67A	2.90E-100	26S proteasome non-ATPase regulatory subunit	Cytoplasmic
ACC_00131	MP	M67	M67A	1.10E-116	COP9 signalosome complex subunit	Cytoplasmic
ACC_03659	MP	M67	M67A	2.80E-41	COP9 signalosome complex subunit	Cytoplasmic
ACC_04182	MP	M67	M67A	1.70E-32	Lys-63-specific deubiquitinase BRCC36	Cytoplasmic
ACC_03993	MP	M67	M67A	2.80E-11	MPN domain-containing protein CG4751	Cytoplasmic
ACC_04064	MP	M67	M67C	2.80E-58	STAM-binding protein	Cytoplasmic
ACC_01885	MP	M67	M67X	4.70E-75	Eukaryotic translation initiation factor	Cytoplasmic
ACC_03488	MP	M67	M67X	5.10E-68	Eukaryotic translation initiation factor	Cytoplasmic
ACC_01598	MA	M76		4.30E-44	Mitochondrial inner membrane protease ATP23 homolog	Cytoplasmic
ACC_00284	PA	S1	S01A	5.60E-36	ATPase family AAA domain-containing protein	Cytoplasmic
ACC_07272	PA	S1	S01A	2.40E-23	Atrial natriuretic peptide-converting enzyme	Cytoplasmic

ACC_03434	PA	S1	S01A	3.80E-47	Chymotrypsin-2	Cytoplasmic
ACC_02167	PA	S1	S01A	6.50E-47	Chymotrypsin-like elastase family member	Cytoplasmic
ACC_06302	PA	S1	S01A	6.00E-143	Chymotrypsinogen B	Cytoplasmic
ACC_07025	PA	S1	S01A	2.60E-57	Inhibitor of growth protein	Cytoplasmic
ACC_08813	PA	S1	S01A	3.10E-70	Neurotrypsin	Cytoplasmic
ACC_09122	PA	S1	S01A	1.10E-18	Ovochymase-1	Cytoplasmic
ACC_08288	PA	S1	S01A	1.70E-32	Ovochymase-2	Cytoplasmic
ACC_06467	PA	S1	S01A	2.20E-19	Ovochymase-2	Cytoplasmic
ACC_04315	PA	S1	S01A	4.40E-48	Plasma kallikrein	Cytoplasmic
ACC_09187	PA	S1	S01A	1.30E-81	Proclotting enzyme	Cytoplasmic
ACC_03827	PA	S1	S01A	3.10E-55	Serine protease easter	Cytoplasmic
ACC_02572	PA	S1	S01A	1.70E-49	Serine protease snake	Cytoplasmic
ACC_03140	PA	S1	S01A	1.40E-59	Serine protease snake	Cytoplasmic
ACC_01097	PA	S1	S01A	5.00E-66	Serine proteinase stubble	Cytoplasmic
ACC_03552	PA	S1	S01A	2.60E-48	Serine proteinase stubble	Cytoplasmic
ACC_08277	PA	S1	S01A	1.20E-105	Serine proteinase stubble	Cytoplasmic
ACC_06778	PA	S1	S01A	2.40E-55	Serine proteinase stubble	Cytoplasmic
ACC_06825	PA	S1	S01A	2.80E-66	Serine proteinase stubble	Cytoplasmic
ACC_03321	PA	S1	S01A	5.40E-40	Trypsin-7	Cytoplasmic
ACC_03787	PA	S1	S01A	1.80E-21	Trypsin-7	Cytoplasmic
ACC_04790	SB	S8	S08A	5.40E-165	Tripeptidyl-peptidase	Cytoplasmic
ACC_09293	SB	S8	S08B	1.40E-144	Furin-like protease	Cytoplasmic
ACC_01634	SB	S8	S08B	1.40E-72	Furin-like protease 1, isoforms	Cytoplasmic
ACC_03874	SB	S8	S08B	7.70E-158	Neuroendocrine convertase	Cytoplasmic
ACC_05787	SC	S9	S09A	2.90E-117	Prolyl endopeptidase	Cytoplasmic
ACC_00541	SC	S9	S09B	3.30E-118	Dipeptidyl peptidase	Cytoplasmic
ACC_00881	SC	S9	S09B	5.70E-65	Dipeptidyl peptidase	Cytoplasmic
ACC_04434	SC	S9	S09C	2.50E-50	Acylamino-acid-releasing enzyme	Cytoplasmic
ACC_00327	SC	S9	S09X	7.80E-105	Abhydrolase domain-containing protein FAM108C1	Cytoplasmic
ACC_09028	SC	S9	S09X	5.50E-44	Carboxylesterase	Cytoplasmic
ACC_09900	SC	S9	S09X	2.20E-66	Esterase B1	Cytoplasmic
ACC_03348	SC	S9	S09X	7.70E-47	Esterase E4	Cytoplasmic
ACC_03349	SC	S9	S09X	2.00E-37	Esterase E4	Cytoplasmic
ACC_08128	SC	S9	S09X	7.60E-48	Esterase E4	Cytoplasmic
ACC_09444	SC	S9	S09X	8.50E-45	Esterase FE4	Cytoplasmic
ACC_06498	SC	S9	S09X	2.10E-43	Esterase FE4	Cytoplasmic
ACC_04689	SC	S9	S09X	3.50E-48	Hormone-sensitive lipase	Cytoplasmic
ACC_08121	SC	S9	S09X	4.10E-51	Neuroligin-1	Cytoplasmic
ACC_07865	SC	S9	S09X	5.90E-42	Neuroligin-1	Cytoplasmic
ACC_09707	SC	S9	S09X	2.30E-53	Neuroligin-3	Cytoplasmic
ACC_07596	SC	S9	S09X	2.70E-37	Neuroligin-3	Cytoplasmic

ACC_03338	SC	S9	S09X	1.20E-25	Neurotactin	Cytoplasmic
ACC_03699	SC	S9	S09X	1.60E-35	Probable arylformamidase	Cytoplasmic
ACC_00464	SC	S9	S09X	8.60E-119	S-formylglutathione hydrolase	Cytoplasmic
ACC_03056	SK	S14		1.80E-87	Putative ATP-dependent Clp protease proteolytic subunit, mitochondrial	Cytoplasmic
ACC_05996	SJ	S16		1.50E-88	Lon protease homolog, mitochondrial	Cytoplasmic
ACC_04158	SF	S26	S26A	1.00E-26	Mitochondrial inner membrane protease subunit	Cytoplasmic
ACC_03399	SC	S28		4.40E-137	Lysosomal Pro-X carboxypeptidase	Cytoplasmic
ACC_01835	SC	S28		6.30E-96	Putative serine protease K12H4.7	Cytoplasmic
ACC_04727	SC	S28		7.20E-167	Putative serine protease K12H4.7	Cytoplasmic
ACC_00561	SC	S33		2.00E-86	Abhydrolase domain-containing protein	Cytoplasmic
ACC_00661	SC	S33		1.10E-39	Abhydrolase domain-containing protein	Cytoplasmic
ACC_04556	SC	S33		8.30E-60	Abhydrolase domain-containing protein	Cytoplasmic
ACC_05821	SC	S33		6.40E-43	Epoxide hydrolase	Cytoplasmic
ACC_04084	SC	S33		5.00E-83	Juvenile hormone epoxide hydrolase	Cytoplasmic
ACC_00604	SC	S33		3.90E-31	Probable serine hydrolase	Cytoplasmic
ACC_06830	SC	S33		6.20E-58	Protein NDRG3	Cytoplasmic
ACC_00038	SC	S33		2.10E-94	Protein phosphatase methylesterase	Cytoplasmic
ACC_07959	SC	S33		1.00E-36	Serine hydrolase-like protein	Cytoplasmic
ACC_07358	SC	S33		1.20E-71	Valacyclovir hydrolase	Cytoplasmic
ACC_02680	ST	S54		1.10E-74	Rhomboid-related protein	Cytoplasmic
ACC_01854	SP	S59		3.80E-58	Nuclear pore complex protein Nup98-Nup96	Cytoplasmic
ACC_02893	SR	S60		1.50E-21	Transferrin	Cytoplasmic
ACC_06347	PB	S63		5.80E-13	Latrophilin-3	Cytoplasmic
ACC_06090	PB	T1	T01A	8.90E-87	Proteasome subunit alpha type-1	Cytoplasmic
ACC_00543	PB	T1	T01A	7.00E-91	Proteasome subunit alpha type-2	Cytoplasmic
ACC_03035	PB	T1	T01A	4.90E-83	Proteasome subunit alpha type-3	Cytoplasmic
ACC_01817	PB	T1	T01A	6.40E-98	Proteasome subunit alpha type-4	Cytoplasmic
ACC_04391	PB	T1	T01A	2.00E-67	Proteasome subunit alpha type-6	Cytoplasmic
ACC_03835	PB	T1	T01A	7.90E-90	Proteasome subunit alpha type-7-1	Cytoplasmic
ACC_05098	PB	T1	T01A	3.00E-70	Proteasome subunit beta type-1	Cytoplasmic
ACC_05282	PB	T1	T01A	3.60E-92	Proteasome subunit beta type-2	Cytoplasmic
ACC_06028	PB	T1	T01A	8.70E-66	Proteasome subunit beta type-4	Cytoplasmic
ACC_04736	PB	T1	T01A	4.80E-77	Proteasome subunit beta type-5	Cytoplasmic
ACC_05405	PB	T1	T01A	1.20E-60	Proteasome subunit beta type-6	Cytoplasmic
ACC_02551	PB	T1	T01A	1.50E-65	Proteasome subunit beta type-7	Cytoplasmic
ACC_03151	PB	T2		7.40E-52	L-asparaginase	Cytoplasmic
ACC_04516	PB	T2		5.00E-64	Threonine aspartase	Cytoplasmic
ACC_09755	PB	T2		6.00E-13	WD repeat-containing protein	Cytoplasmic
ACC_00568	PB	T3		2.60E-67	Gamma-glutamyltranspeptidase	Cytoplasmic
ACC_00286	PA	S1	S01A	2.50E-46	Mite allergen Der p	Extracellular

ACC_00300	SC	S9	S09X	2.10E-53	Carboxylesterase	Extracellular
ACC_00476	PA	S1	S01A	1.50E-86	Serine proteinase stubble	Extracellular
ACC_01096	PA	S1	S01A	2.50E-114	Serine proteinase stubble	Extracellular
ACC_01184	CA	C1	C01A	3.40E-68	Putative cysteine proteinase CG12163	Extracellular
ACC_01989	SC	S9	S09X	9.00E-38	Esterase FE4	Extracellular
ACC_02114	CA	C2	C02A	2.20E-54	Calpain-C	Extracellular
ACC_02231	;ned Serine Pe	S72		3.30E-53	Dystroglycan	Extracellular
ACC_02233	MA	M12	M12B	7.00E-26	A disintegrin and metalloproteinase with thrombospondin motifs	Extracellular
ACC_02240	PA	S1	S01A	3.20E-61	Chymotrypsin-2	Extracellular
ACC_02285	SC	S9	S09X	2.30E-49	Esterase FE4	Extracellular
ACC_02286	SC	S9	S09X	1.20E-52	Esterase FE4	Extracellular
ACC_02483	PA	S1	S01A	1.60E-29	Ovochymase-2	Extracellular
ACC_02612	SC	S9	S09X	3.90E-102	Acetylcholinesterase	Extracellular
ACC_02653	CA	C1	C01A	4.10E-110	Counting factor associated protein D	Extracellular
ACC_02703	MA	M2		6.00E-196	Angiotensin-converting enzyme	Extracellular
ACC_02859	PB	T2		1.40E-42	N(4)-(Beta-N-acetylglucosaminyl)-L-asparaginase (Fragment)	Extracellular
ACC_02952	PA	S1	S01A	2.70E-56	Proclotting enzyme	Extracellular
ACC_03016	PA	S1	S01A	6.40E-62	Serine protease snake	Extracellular
ACC_03553	PA	S1	S01A	1.60E-56	Trypsin-1	Extracellular
ACC_03904	MA	M12	M12B	3.60E-50	A disintegrin and metalloproteinase with thrombospondin motifs	Extracellular
ACC_03949	AA	A1	A01A	4.70E-147	Lysosomal aspartic protease	Extracellular
ACC_04046	MA	M1		1.10E-14	Aminopeptidase N	Extracellular
ACC_04283	MH	M28	M28B	6.40E-62	Plasma glutamate carboxypeptidase	Extracellular
ACC_04284	MH	M28	M28B	9.40E-59	Plasma glutamate carboxypeptidase	Extracellular
ACC_04401	SC	S9	S09X	4.50E-48	Acetylcholinesterase	Extracellular
ACC_04758	PA	S1	S01A	3.80E-64	Proclotting enzyme	Extracellular
ACC_04851	MC	M14	M14A	9.50E-78	Zinc carboxypeptidase A	Extracellular
ACC_04912	CA	C1	C01A	6.10E-109	Cathepsin L	Extracellular
ACC_05066	SC	S9	S09X	1.80E-49	Esterase FE4	Extracellular
ACC_05683	PA	S1	S01A	1.30E-51	Serine protease gd	Extracellular
ACC_06997	PA	S1	S01A	8.30E-60	Proclotting enzyme	Extracellular
ACC_07096	PA	S1	S01A	4.30E-16	Trypsin-5	Extracellular
ACC_07194	MA	M10	M10A	2.30E-68	Matrix metalloproteinase-14	Extracellular
ACC_07232	PA	S1	S01A	1.20E-52	Trypsin-7	Extracellular
ACC_07317	MA	M13		1.20E-11	Membrane metallo-endopeptidase-like	Extracellular
ACC_07422	SC	S9	S09X	3.90E-102	Acetylcholinesterase	Extracellular
ACC_07749	SC	S10		6.20E-111	Vitellogenic carboxypeptidase	Extracellular
ACC_07815	PB	T1	T01A	1.50E-82	Proteasome subunit beta type-3	Extracellular

ACC_07889	SC	S9	S09X	2.40E-52	Neuroligin-4, Y-linked	Extracellular
ACC_07892	CD	C13		2.30E-123	Putative GPI-anchor transamidase	Extracellular
ACC_08027	MJ	M19		8.00E-56	Dipeptidase	Extracellular
ACC_08087	PA	S1	S01A	3.30E-46	Transmembrane protease serine	Extracellular
ACC_08270	PA	S1	S01A	1.20E-54	Trypsin-2	Extracellular
ACC_08271	PA	S1	S01A	3.80E-30	Trypsin-4	Extracellular
ACC_08400	PA	S1	S01A	5.60E-80	Chymotrypsin-1	Extracellular
ACC_08714	MA	M1		1.20E-50	Aminopeptidase N	Extracellular
ACC_08715	MA	M1		3.10E-30	Aminopeptidase N	Extracellular
ACC_08716	MA	M1		2.30E-59	Membrane alanyl aminopeptidase	Extracellular
ACC_08925	SC	S10		4.40E-101	Retinoid-inducible serine carboxypeptidase	Extracellular
ACC_08983	MA	M1		9.50E-15	Aminopeptidase N	Extracellular
ACC_09149	MC	M14	M14A	3.00E-87	Carboxypeptidase B	Extracellular
ACC_09182	MA	M12	M12B	1.00E-92	A disintegrin and metalloproteinase with thrombospondin motifs	Extracellular
ACC_09302	PA	S1	S01A	1.60E-94	Serine protease easter	Extracellular
ACC_09631	PA	S1	S01A	4.90E-46	Trypsin-1	Extracellular
ACC_09635	MA	M12	M12B	1.40E-56	A disintegrin and metalloproteinase with thrombospondin motifs	Signal
ACC_09734	SR	S60		8.60E-69	Melanotransferrin	Extracellular
ACC_09804	SC	S9	S09X	7.20E-59	Acetylcholinesterase	Extracellular
ACC_09865	SB	S8	S08A	3.20E-131	Membrane-bound transcription factor site-1 protease	Extracellular
ACC_01876	AD	A22	A22B	4.00E-123	Minor histocompatibility antigen H13	Transmembrane
ACC_04167	AD	A22	A22B	3.20E-129	Signal peptide peptidase-like	Transmembrane
ACC_02840	CA	C1	C01A	8.40E-62	Cathepsin O	Transmembrane
ACC_02954	CA	C1	C01A	4.40E-67	Uncharacterized peptidase C1-like protein F26E4.3	Transmembrane
ACC_00878	CA	C19		2.90E-24	Ubiquitin carboxyl-terminal hydrolase	Transmembrane
ACC_09605	ıed Cysteine P‹	C88		4.10E-39	Ubiquitin thioesterase OTU1	Transmembrane
ACC_07202	MA	M1		1.40E-80	Aminopeptidase N	Transmembrane
ACC_07269	MA	M1		4.70E-71	Glutamyl aminopeptidase	Transmembrane
ACC_07643	MA	M1		2.70E-73	Glutamyl aminopeptidase	Transmembrane
ACC_00247	MA	M2		1.50E-17	Angiotensin-converting enzyme	Transmembrane
ACC_07508	MA	M12	M12B	1.30E-39	A disintegrin and metalloproteinase with thrombospondin motifs	Transmembrane
ACC_00164	MA	M12	M12B	1.90E-31	Disintegrin and metalloproteinase domain-containing protein	Transmembrane
ACC_03045	MA	M13		4.50E-122	Endothelin-converting enzyme	Transmembrane
ACC_03225	MA	M13		7.40E-205	Endothelin-converting enzyme	Transmembrane
ACC_08324	MA	M13		5.90E-247	Endothelin-converting enzyme	Transmembrane
ACC_08796	MA	M13		0.00E+00	Membrane metallo-endopeptidase-like	Transmembrane
ACC_08938	MA	M13		3.30E-258	Membrane metallo-endopeptidase-like	Transmembrane

ACC_09320	MA	M13		1.90E-15	Membrane metallo-endopeptidase-like	Transmembrane
ACC_05107	MC	M14	M14B	8.30E-113	Carboxypeptidase D	Transmembrane
ACC_01263	ME	M16	M16B	2.90E-45	Cytochrome b-c1 complex subunit	Transmembrane
ACC_09742	MF	M17		1.10E-91	Putative aminopeptidase W07G4.4	Transmembrane
ACC_02141	MJ	M19		4.10E-91	Dipeptidase	Transmembrane
ACC_02650	MJ	M19		9.30E-93	Dipeptidase	Transmembrane
ACC_08679	MJ	M19		4.80E-96	Dipeptidase	Transmembrane
ACC_01085	MH	M28	M28X	1.20E-58	2-amino-3-ketobutyrate coenzyme A ligase	Transmembrane
ACC_01083	MH	M28	M28X	2.30E-87	Endoplasmic reticulum metallopeptidase	Transmembrane
ACC_08946	MA	M41		8.90E-123	AFG3-like protein	Transmembrane
ACC_01389	MA	M41		1.60E-63	Paraplegin	Transmembrane
ACC_04529	MA	M48	M48A	2.30E-51	CAAX prenyl protease	Transmembrane
ACC_10121	MA	M48	M48X	1.20E-27	Metalloendopeptidase OMA1, mitochondrial	Transmembrane
ACC_05847	MM	M50		1.40E-91	Membrane-bound transcription factor site-2 protease	Transmembrane
ACC_00672	PA	S1	S01A	7.00E-29	Atrial natriuretic peptide-converting enzyme	Transmembrane
ACC_08586	PA	S1	S01A	3.90E-23	Coagulation factor X	Transmembrane
ACC_05072	PA	S1	S01A	4.00E-70	Plasma kallikrein	Transmembrane
ACC_09962	PA	S1	S01A	5.60E-63	Serine protease HTRA2, mitochondrial	Transmembrane
ACC_08869	PA	S1	S01A	2.40E-89	Serine protease nudel	Transmembrane
ACC_09990	PA	S1	S01A	1.90E-65	Serine proteinase stubble	Transmembrane
ACC_08067	PA	S1	S01A	1.00E-72	Transmembrane protease serine	Transmembrane
ACC_10005	PA	S1	S01A	1.00E-42	Transmembrane protease serine	Transmembrane
ACC_08200	PA	S1	S01A	3.90E-102	Trypsin-1	Transmembrane
ACC_10116	PA	S1	S01A	1.70E-75	Tubulin beta-4 chain	Transmembrane
ACC_03672	PA	S1	S01A	9.20E-24	WD repeat-containing protein	Transmembrane
ACC_08260	SB	S8	S08B	1.10E-55	Neuroendocrine convertase	Transmembrane
ACC_08912	SC	S9	S09B	5.50E-34	Dipeptidyl aminopeptidase-like protein	Transmembrane
ACC_09285	SC	S9	S09B	7.60E-61	Dipeptidyl peptidase	Transmembrane
ACC_01628	SC	S9	S09B	8.10E-62	Inactive dipeptidyl peptidase	Transmembrane
ACC_02628	SC	S9	S09X	1.00E-53	Abhydrolase domain-containing protein	Transmembrane
ACC_04101	SC	S9	S09X	6.90E-42	Carboxylesterase	Transmembrane
ACC_03180	SC	S9	S09X	1.60E-58	Monoacylglycerol lipase ABHD12	Transmembrane
ACC_08428	SC	S9	S09X	1.60E-13	Neuroligin-1	Transmembrane
ACC_09777	SC	S9	S09X	1.10E-65	Neuroligin-1	Transmembrane
ACC_08793	SC	S9	S09X	1.60E-43	Neuroligin-4, Y-linked	Transmembrane
ACC_04071	SC	S9	S09X	1.10E-25	Neuroligin-4, Y-linked	Transmembrane
ACC_05099	SC	S9	S09X	6.20E-75	Protein BAT5	Transmembrane
ACC_00536	SF	S26	S26B	1.70E-64	Signal peptidase complex catalytic subunit SEC11C	Transmembrane
ACC_03701	ST	S54		4.30E-44	Presenilins-associated rhomboid-like protein, mitochondrial	Transmembrane
ACC_07343	ST	S54		7.40E-46	Protein rhomboid	Transmembrane

ACC_03012	ST	S54	8.50E-28	Rhomboid domain-containing protein	Transmembrane
ACC_00147	SR	S60	4.60E-47	Transferrin	Transmembrane
ACC_06431	PB	T3	7.00E-26	Gamma-glutamyltransferase	Transmembrane
ACC_07118	PB	T3	1.70E-119	Gamma-glutamyltranspeptidase	Transmembrane
ACC_00081	unassigned	U48	4.30E-63	CAAX prenyl protease	Transmembrane

Supplementary Table 30. Protease Inhibitors in *A.cerana*

Gene	Family	Inhibited Peptidase Family	Evalue	Annotation	Subcellular Localization
ACC_08932	I1	S1,S8	8.60E-11	Agrin	Cytoplasmic
ACC_07394	I1	S1,S8	3.10E-12	Follistatin-related protein	Cytoplasmic
ACC_04565	I2	S1	6.00E-14	Neurexin-4	Cytoplasmic
ACC_05467	I2	S1	2.40E-18	Papilin	Cytoplasmic
ACC_01223	I2	S1	3.80E-14	Spondin-1	Cytoplasmic
ACC_01762	I31	C1	1.20E-11	SPARC-related modular calcium-binding protein	Cytoplasmic
ACC_00249	I31	C1	2.70E-14	Testican-2	Cytoplasmic
ACC_01134	I32	C14	8.70E-24	Apoptosis inhibitor IAP	Cytoplasmic
ACC_08215	I32	C14	9.70E-22	Apoptosis inhibitor IAP	Cytoplasmic
ACC_09086	I32	C14	4.70E-50	Baculoviral IAP repeat-containing protein	Cytoplasmic
ACC_07668	I35	M10	9.70E-13	Leucine-rich repeat-containing protein	Cytoplasmic
ACC_01953	I4	S1,S8	4.60E-71	Antitrypsin	Cytoplasmic
ACC_04789	I51	S1	1.10E-51	Phosphatidylethanolamine-binding protein homolog F40A3.3	Cytoplasmic
ACC_08091	I8	S1,M4	3.50E-16	Chymotrypsin inhibitor	Cytoplasmic
ACC_09404	I8	S1,M4	3.50E-16	Chymotrypsin inhibitor	Cytoplasmic
ACC_09617	I8	S1,M4	9.50E-11	Hemocytin	Cytoplasmic
ACC_05408	I1	S1,S8	9.70E-12	Agrin	Extracellular
ACC_01482	I39		5.90E-127	Alpha-2-macroglobulin-like protein	Extracellular
ACC_04590	I39		1.10E-40	CD109 antigen	Extracellular
ACC_01788	I4	S1,S8	6.60E-66	Alaserpin	Extracellular
ACC_04656	I4	S1,S8	7.00E-74	Antithrombin-III	Extracellular
ACC_09555	I4	S1,S8	1.80E-12	Glutamate receptor delta-2 subunit	Extracellular
ACC_01524	I4	S1,S8	2.40E-13	Ovalbumin-related protein Y	Extracellular
ACC_04567	I4	S1,S8	5.30E-38	Uncharacterized serpin-like protein MA_2613/MA_2612	Extracellular

ACC_08703	I51	S1	8.80E-32	Phosphatidylethanolamine-binding protein homolog F40A3.3	Extracellular
ACC_08083	I8	S1,M4	7.80E-35	Chymotrypsin inhibitor	Extracellular
ACC_08085	I8	S1,M4	9.90E-12	Chymotrypsin inhibitor	Extracellular
ACC_01201	I1	S1,S8	4.00E-12	Solute carrier organic anion transporter family member	Transmembrane
ACC_08021	I2	S1	1.60E-14	Spondin-1	Transmembrane
ACC_01135	I32	C14	5.50E-25	Apoptosis 2 inhibitor	Transmembrane

Supplementary Table 31. Heat shock proteins in *A.cerana*

Heat shock	IPR Number	Gene	Domains	Annotation	Expression Profile
**Hsp20**	IPR002068	ACC_00695		Protein lethal(2)essential for life	GCF-down (p value <1e-30)
	IPR002068	ACC_01373	60596-59994	Protein lethal(2)essential for life	
	IPR002068	ACC_01374	64052-63342	Protein lethal(2)essential for life	GCF-down (p value <1e-150)
	IPR002068	ACC_01375	66997-66416	Protein lethal(2)essential for life	
	IPR002068	ACC_01376	69356-68709	Protein lethal(2)essential for life	CJ-up (p value <1e-150)
	IPR002068	ACC_01377	75221-74637	Protein lethal(2)essential for life	CJ-up (p value =0)
	IPR002068	ACC_04703		Protein lethal(2)essential for life	GCF-down (p value <1e-10)
	IPR002068	ACC_04704		Protein lethal(2)essential for life	
	IPR002068	ACC_05492		Heat shock protein beta-1	
	IPR002068	ACC_02853		Protein lethal(2)essential for life	
**HSP40/DnaJ**	IPR001305/IPR001623	ACC_04757		DnaJ homolog subfamily A member	
	IPR001305/IPR001623	ACC_05141		Protein tumorous imaginal discs, mitochondrial	
	IPR001623	ACC_00324		DnaJ homolog subfamily C member	
	IPR001623	ACC_00356		Cysteine string protein	
	IPR001623	ACC_00373		Mitochondrial import inner membrane translocase subunit TIM14	
	IPR001623	ACC_00574		DnaJ homolog subfamily C member	
	IPR001623	ACC_00874		DnaJ homolog subfamily B member	
	IPR001623	ACC_01029		DnaJ homolog subfamily C member	
	IPR001623	ACC_02173		DnaJ homolog subfamily C member	
	IPR001623	ACC_02579		DnaJ homolog subfamily C member	
	IPR001623	ACC_03122		J domain-containing protein	CJ-up(p value <1e-50)
	IPR001623	ACC_03533		DnaJ homolog subfamily C member	
	IPR001623	ACC_03753		DnaJ homolog subfamily B member	
	IPR001623	ACC_03920		Translocation protein SEC63 homolog	
	IPR001623	ACC_04275		DnaJ homolog subfamily C member	
	IPR001623	ACC_04338		DnaJ homolog subfamily C member	
	IPR001623	ACC_04344		Cyclin-G-associated kinase	
	IPR001623	ACC_04345		Putative tyrosine-protein phosphatase auxilin	
	IPR001623	ACC_04586		DnaJ homolog subfamily C member	
	IPR001623	ACC_04780		DnaJ homolog subfamily A member	
	IPR001623	ACC_05453		DnaJ protein homolog	BY-down(p value <1e-30)
	IPR001623	ACC_05980		DnaJ homolog subfamily C member	
	IPR001623	ACC_06366		DnaJ homolog subfamily B member	
	IPR001623	ACC_06518		DnaJ homolog subfamily C member	
	IPR001623	ACC_06629		Iron-sulfur cluster co-chaperone protein HscB, mitochondrial	
	IPR001623	ACC_07013		DnaJ homolog subfamily C member	

	IPR001623	ACC_07679		DnaJ homolog subfamily C member	
	IPR001623	ACC_08423		DnaJ homolog subfamily C member	
	IPR001623	ACC_08768		DnaJ homolog subfamily C member	
	IPR001623	ACC_09290		DnaJ homolog subfamily B member	
	IPR001623	ACC_09502		DnaJ homolog subfamily B member	
	IPR013126	ACC_02737		Heat shock	
	IPR013126	ACC_03611	EEVD	heat shock protein Hsp70Ab-like	
**Hsp70**	IPR013126	ACC_04534	luding GGMP repeat, EEV	Heat shock protein	BY-down (p value=0)
	IPR013126	ACC_05699		heat shock 70 kDa protein cognate 5-like	
	IPR013126	ACC_05859		heat shock 70 kDa protein 4L isoform 1	
	IPR013126	ACC_06529		Hypoxia up-regulated protein	
	IPR001404	ACC_01484	EEVD	heat shock protein 90	BY-down (p value=0)
**Hsp90**	IPR001404	ACC_02014		heat shock protein 75 kDa	
	IPR001404	ACC_04157	EEVD	Heat shock protein	BY-down (p value <1e-100)
	IPR001404	ACC_06367		Endoplasmin	
	IPR001844	ACC_07756		60 kDa heat shock protein, mitochondrial-like	
**Other**	IPR018369	ACC_04922		10 kDa heat shock protein, mitochondrial-like	
	IPR001763	ACC_00316		heat shock protein 67B2-like	
	IPR001763	ACC_07746		heat shock protein 67B2-like	

Supplementary Table 32. Canditate HSP70 genes used for Phylogenetic analysis.

Species	Gene Id
A. gambiae	AgaP_AGAP012891
	AgaP_AGAP004582
	AgaP_AGAP010876
	AgaP_AGAP004944
	AgaP_AGAP004192
	AgaP_AGAP010331
	AgaP_AGAP001827
	AgaP_AGAP007682
A. mellifera	GB19503
	GB14852
	GB15016
	GB19860
	GB10836
	GB10732
A.florea	XP_003690658
	XP_003690659
	XP_003691374
A. cerana	ACC_05859
	ACC_04534
	ACC_02737
	ACC_05699
	ACC_03611
	ACC_06529
B. mori	BGIBMGA004613
	BGIBMGA014618
	BGIBMGA006574
	BGIBMGA004614
	BGIBMGA014536
	BGIBMGA001635
	BGIBMGA002381
	BGIBMGA007950
	BGIBMGA006313
	BGIBMGA001218
	BGIBMGA008551
C. floridanus	EFN73277
	EFN65945
	EFN61604
	EFN69363
	EFN68463
	EFN70757
D. melanogaster	Dmel_CG7182
	Dmel_CG7756
	Dmel_CG5436
	Dmel_CG31359
	Dmel_CG31449
	Dmel_CG5834
	Dmel_CG6489
	Dmel_CG8937
	Dmel_CG18743
	Dmel_CG31366

	Dmel_CG4264
	Dmel_CG4147
	Dmel_CG8542
	Dmel_CG6603
	Dmel_CG2918
	EFN86984
	EFN75098
H. saltator	EFN86831
	EFN87680
	EFN88179
	EFN75861
	nvi:100114095
	nvi:100119971
	nvi:100120280
	nvi:100114493
N. vitripennis	nvi:100118379
	nvi:100114552
	nvi:100114907
	nvi:100123502
	nvi:100122605

Supplementary Table 33 Nuclear receptors in A.cerana

		Gene	KO	Product
**1. Thyroid hormone like**				
	**1D. Rev-Erb**	ACC_01018	K08701	nuclear receptor subfamily 1 group D member 3
		ACC_08343	K08701	nuclear receptor subfamily 1 group D member 3
		ACC_09450	K08701	nuclear receptor subfamily 1 group D member 3
	**1F. RAR-related orphan receptor**	ACC_00692	K14033	nuclear receptor subfamily 1 group F member 4
	**1H. Liver X receptor like receptor**	ACC_01640	K14034	ecdysone receptor
	**1I. Vitamin D3 like receptor**	ACC_03003	K14035	nuclear receptor subfamily 1 group I
**2. Hepatocyte nuclear factor 4 like**				
	**2A. Hepatocyte nuclear factor 4 receptor**	ACC_05199	K07292	hepatocyte nuclear factor 4-alpha
		ACC_00552	K07295	nuclear receptor subfamily 2 group A
	**2B. Retinoid X receptor (RXR)**	ACC_06901	K14030	nuclear receptor subfamily 2 group B member 4
	**2C. Testicular receptor**	ACC_02345	K14031	nuclear receptor subfamily 2 group C
	**2E. Tailless like receptor**	ACC_02796	K08545	nuclear receptor subfamily 2 group E member 1
		ACC_07152	K08546	nuclear receptor subfamily 2 group E member 3
		ACC_09910	K08546	nuclear receptor subfamily 2 group E member 3

	**2F. COUP-TF like receptor**	ACC_07789	K08547	COUP transcription factor 1
**3. Nerve growth factor IB like**				
	**3A. Nerve growth factor IB like receptor**	ACC_10011	K08558	nuclear receptor subfamily 4 group A member 2
**4. Fushi tarazu-F1 like**				
	**5A. Fushi tarazu-F1 like receptor**	ACC_00461	K08705	nuclear receptor subfamily 5 group A member 3
		ACC_04657	K08705	nuclear receptor subfamily 5 group A member 3
**5. Knirps / DAX like**				
	**5A. Knirps like receptor**	ACC_07210	K08706	nuclear receptor subfamily 0 group A
		ACC_08801	K08706	nuclear receptor subfamily 0 group A
		ACC_08966	K08706	nuclear receptor subfamily 0 group A

Supplementary Table 34 Genes involved in Circadian rhythm of A.cerana

Pathway	Gene	KO	Product
**04710 Circadian rhythm**	ACC_07470	K08960	casein kinase 1, epsilon [EC:2.7.11.1]
	ACC_09694	K02633	period circadian protein
	ACC_03658	K02295	cryptochrome
	ACC_03520	K02296	aryl hydrocarbon receptor nuclear translocator-like protein 1
	ACC_01245	K02223	circadian locomoter output cycles kaput protein [EC:2.3.1.48]
	ACC_03198	K03868	RING-box protein 1
	ACC_07120	K03347	cullin 1
	ACC_03137	K03094	S-phase kinase-associated protein 1
	ACC_08081	K03362	F-box and WD-40 domain protein 1/11
	ACC_05816	K10269	F-box and leucine-rich repeat protein 3
	ACC_03340	K07198	5'-AMP-activated protein kinase, catalytic alpha subunit [EC:2.7.11.11]
	ACC_01805	K07199	5'-AMP-activated protein kinase, regulatory beta subunit
**04713 Circadian entrainment**	ACC_06938	K05208	glutamate receptor, ionotropic, N-methyl D-aspartate 1
	ACC_09518	K05208	glutamate receptor, ionotropic, N-methyl D-aspartate 1
	ACC_08922	K05210	glutamate [NMDA] receptor subunit epsilon-2
	ACC_06604	K02183	calmodulin
	ACC_05678	K04515	calcium/calmodulin-dependent protein kinase (CaM kinase) II [EC:2.7.11.17]
	ACC_05659	K04371	extracellular signal-regulated kinase 1/2 [EC:2.7.11.24]
	ACC_04555	K04445	ribosomal protein S6 kinase alpha-5 [EC:2.7.11.1]
	ACC_05838	K04958	inositol 1,4,5-triphosphate receptor type 1
	ACC_03182	K04962	ryanodine receptor 2
	ACC_04817	K16513	carboxyl-terminal PDZ ligand of neuronal nitric oxide synthase protein
	ACC_06850	K04630	guanine nucleotide-binding protein G(i) subunit alpha
	ACC_07382	K04534	guanine nucleotide-binding protein G(o) subunit alpha
	ACC_09212	K04536	guanine nucleotide-binding protein G(I)/G(S)/G(T) subunit beta-1
	ACC_04262	K04539	guanine nucleotide-binding protein subunit beta-5
	ACC_06468	K04547	guanine nucleotide-binding protein G(I)/G(S)/G(O) subunit gamma-13
	ACC_01798	K04632	guanine nucleotide-binding protein G(s) subunit alpha
	ACC_00796	K08042	adenylate cyclase 2 [EC:4.6.1.1]
	ACC_02640	K08043	adenylate cyclase 3 [EC:4.6.1.1]
	ACC_07908	K08043	adenylate cyclase 3 [EC:4.6.1.1]
	ACC_06514	K08045	adenylate cyclase 5 [EC:4.6.1.1]
	ACC_01531	K08048	adenylate cyclase 8 [EC:4.6.1.1]
	ACC_00311	K08049	adenylate cyclase 9 [EC:4.6.1.1]

	ACC_10007	K11265	adenylate cyclase 10 [EC:4.6.1.1]
	ACC_04702	K04345	protein kinase A [EC:2.7.11.11]
	ACC_08577	K04345	protein kinase A [EC:2.7.11.11]
	ACC_02494	K12318	guanylate cyclase soluble subunit alpha [EC:4.6.1.2]
	ACC_09018	K12319	guanylate cyclase soluble subunit beta [EC:4.6.1.2]
	ACC_09607	K07376	protein kinase, cGMP-dependent [EC:2.7.11.12]
	ACC_04047	K04634	guanine nucleotide-binding protein G(q) subunit alpha
	ACC_02939	K05858	phosphatidylinositol phospholipase C, beta [EC:3.1.4.11]
	ACC_05612	K05858	phosphatidylinositol phospholipase C, beta [EC:3.1.4.11]
	ACC_02607	K02677	classical protein kinase C [EC:2.7.11.13]
	ACC_09694	K02633	period circadian protein
**04711 Circadian rhythm - fly**	ACC_07470	K08960	casein kinase 1, epsilon [EC:2.7.11.1]
	ACC_09694	K02633	period circadian protein
	ACC_01245	K02223	circadian locomoter output cycles kaput protein [EC:2.3.1.48]
	ACC_03520	K02296	aryl hydrocarbon receptor nuclear translocator-like protein 1
	ACC_09277	K12114	vrille
	ACC_00165	K09057	hepatic leukemia factor
	ACC_06459	K03083	glycogen synthase kinase 3 beta [EC:2.7.11.26]
**04712 Circadian rhythm - plant**	ACC_05262	K03097	casein kinase II subunit alpha [EC:2.7.11.1]
	ACC_06230	K03115	casein kinase II subunit beta
	ACC_05308	K10143	E3 ubiquitin-protein ligase RFWD2 [EC:6.3.2.19]

Supplementary Table 35 Genes involved in Nervous System of A.cerana

Pathway	Gene	KO	Product
**04724 Glutamatergic synapse**	ACC_02139	K01425	glutaminase [EC:3.5.1.2]
	ACC_06938	K05208	glutamate receptor, ionotropic, N-methyl D-aspartate 1
	ACC_09518	K05208	glutamate receptor, ionotropic, N-methyl D-aspartate 1
	ACC_08922	K05210	glutamate [NMDA] receptor subunit epsilon-2
	ACC_05684	K04348	protein phosphatase 3, catalytic subunit [EC:3.1.3.16]
	ACC_08696	K04348	protein phosphatase 3, catalytic subunit [EC:3.1.3.16]
	ACC_05321	K06268	protein phosphatase 3, regulatory subunit
	ACC_05594	K06268	protein phosphatase 3, regulatory subunit
	ACC_06311	K15009	SH3 and multiple ankyrin repeat domains protein
	ACC_03529	K15010	homer
	ACC_04047	K04634	guanine nucleotide-binding protein G(q) subunit alpha
	ACC_02939	K05858	phosphatidylinositol phospholipase C, beta [EC:3.1.4.11]
	ACC_05612	K05858	phosphatidylinositol phospholipase C, beta [EC:3.1.4.11]
	ACC_02607	K02677	classical protein kinase C [EC:2.7.11.13]
	ACC_05838	K04958	inositol 1,4,5-triphosphate receptor type 1
	ACC_05868	K01115	phospholipase D1/2 [EC:3.1.4.4]
	ACC_05659	K04371	extracellular signal-regulated kinase 1/2 [EC:2.7.11.24]
	ACC_01798	K04632	guanine nucleotide-binding protein G(s) subunit alpha
	ACC_00796	K08042	adenylate cyclase 2 [EC:4.6.1.1]
	ACC_02640	K08043	adenylate cyclase 3 [EC:4.6.1.1]
	ACC_07908	K08043	adenylate cyclase 3 [EC:4.6.1.1]
	ACC_06514	K08045	adenylate cyclase 5 [EC:4.6.1.1]
	ACC_01531	K08048	adenylate cyclase 8 [EC:4.6.1.1]
	ACC_00311	K08049	adenylate cyclase 9 [EC:4.6.1.1]
	ACC_04702	K04345	protein kinase A [EC:2.7.11.11]
	ACC_08577	K04345	protein kinase A [EC:2.7.11.11]
	ACC_08905	K04605	metabotropic glutamate receptor 2/3
	ACC_08691	K04608	metabotropic glutamate receptor 6/7/8
	ACC_06850	K04630	guanine nucleotide-binding protein G(i) subunit alpha
	ACC_07382	K04534	guanine nucleotide-binding protein G(o) subunit alpha
	ACC_09212	K04536	guanine nucleotide-binding protein G(I)/G(S)/G(T) subunit beta-1
	ACC_04262	K04539	guanine nucleotide-binding protein subunit beta-5
	ACC_06468	K04547	guanine nucleotide-binding protein G(I)/G(S)/G(O) subunit gamma-13
	ACC_05588	K00910	beta-adrenergic-receptor kinase [EC:2.7.11.15]
	ACC_03192	K05613	solute carrier family 1 (glial high affinity glutamate transporter), member 2
	ACC_05532	K01915	glutamine synthetase [EC:6.3.1.2]
**04727 GABAergic synapse**	ACC_02139	K01425	glutaminase [EC:3.5.1.2]
	ACC_10158	K01580	glutamate decarboxylase [EC:4.1.1.15]
	ACC_05210	K15015	solute carrier family 32 (vesicular inhibitory amino acid transporter)
	ACC_01468	K13524	4-aminobutyrate aminotransferase / (S)-3-amino-2-methylpropionate transaminase [EC:2.6.1.19 2.6.1.22]
	ACC_06768	K05175	gamma-aminobutyric acid receptor subunit alpha
	ACC_09040	K05175	gamma-aminobutyric acid receptor subunit alpha
	ACC_08806	K05181	gamma-aminobutyric acid receptor subunit beta
	ACC_04702	K04345	protein kinase A [EC:2.7.11.11]
	ACC_08577	K04345	protein kinase A [EC:2.7.11.11]
	ACC_03579	K05704	tyrosine-protein kinase Src [EC:2.7.10.2]
	ACC_02607	K02677	classical protein kinase C [EC:2.7.11.13]
	ACC_02541	K08341	GABA(A) receptor-associated protein (autophagy-related protein 8)
	ACC_01809	K06027	vesicle-fusing ATPase [EC:3.6.4.6]
	ACC_03853	K15376	gephyrin [EC:2.10.1.1 2.7.7.75]

	ACC_01515	K04615	gamma-aminobutyric acid type B receptor
	ACC_08078	K04615	gamma-aminobutyric acid type B receptor
	ACC_09743	K04615	gamma-aminobutyric acid type B receptor
	ACC_06850	K04630	guanine nucleotide-binding protein G(i) subunit alpha
	ACC_07382	K04534	guanine nucleotide-binding protein G(o) subunit alpha
	ACC_09212	K04536	guanine nucleotide-binding protein G(I)/G(S)/G(T) subunit beta-1
	ACC_04262	K04539	guanine nucleotide-binding protein subunit beta-5
	ACC_06468	K04547	guanine nucleotide-binding protein G(I)/G(S)/G(O) subunit gamma-13
	ACC_00796	K08042	adenylate cyclase 2 [EC:4.6.1.1]
	ACC_02640	K08043	adenylate cyclase 3 [EC:4.6.1.1]
	ACC_07908	K08043	adenylate cyclase 3 [EC:4.6.1.1]
	ACC_06514	K08045	adenylate cyclase 5 [EC:4.6.1.1]
	ACC_01531	K08048	adenylate cyclase 8 [EC:4.6.1.1]
	ACC_00311	K08049	adenylate cyclase 9 [EC:4.6.1.1]
	ACC_00266	K05034	solute carrier family 6 (neurotransmitter transporter, GABA) member 1
	ACC_02486	K05034	solute carrier family 6 (neurotransmitter transporter, GABA) member 1
	ACC_05762	K05034	solute carrier family 6 (neurotransmitter transporter, GABA) member 1
	ACC_05532	K01915	glutamine synthetase [EC:6.3.1.2]
**04725 Cholinergic synapse**	ACC_09932	K00623	choline O-acetyltransferase [EC:2.3.1.6]
	ACC_02612	K01049	acetylcholinesterase [EC:3.1.1.7]
	ACC_07422	K01049	acetylcholinesterase [EC:3.1.1.7]
	ACC_09804	K01049	acetylcholinesterase [EC:3.1.1.7]
	ACC_06927	K04131	muscarinic acetylcholine receptor M3
	ACC_04047	K04634	guanine nucleotide-binding protein G(q) subunit alpha
	ACC_02939	K05858	phosphatidylinositol phospholipase C, beta [EC:3.1.4.11]
	ACC_05612	K05858	phosphatidylinositol phospholipase C, beta [EC:3.1.4.11]
	ACC_05838	K04958	inositol 1,4,5-triphosphate receptor type 1
	ACC_02607	K02677	classical protein kinase C [EC:2.7.11.13]
	ACC_06052	K04926	potassium voltage-gated channel KQT-like subfamily member 1
	ACC_06850	K04630	guanine nucleotide-binding protein G(i) subunit alpha
	ACC_07382	K04534	guanine nucleotide-binding protein G(o) subunit alpha
	ACC_09212	K04536	guanine nucleotide-binding protein G(I)/G(S)/G(T) subunit beta-1
	ACC_04262	K04539	guanine nucleotide-binding protein subunit beta-5
	ACC_06468	K04547	guanine nucleotide-binding protein G(I)/G(S)/G(O) subunit gamma-13
	ACC_02813	K07827	GTPase KRas
	ACC_08503	K04368	mitogen-activated protein kinase kinase 1 [EC:2.7.12.2]
	ACC_09588	K04368	mitogen-activated protein kinase kinase 1 [EC:2.7.12.2]
	ACC_05659	K04371	extracellular signal-regulated kinase 1/2 [EC:2.7.11.24]
	ACC_00796	K08042	adenylate cyclase 2 [EC:4.6.1.1]
	ACC_02640	K08043	adenylate cyclase 3 [EC:4.6.1.1]
	ACC_07908	K08043	adenylate cyclase 3 [EC:4.6.1.1]
	ACC_06514	K08045	adenylate cyclase 5 [EC:4.6.1.1]
	ACC_01531	K08048	adenylate cyclase 8 [EC:4.6.1.1]
	ACC_00311	K08049	adenylate cyclase 9 [EC:4.6.1.1]
	ACC_04702	K04345	protein kinase A [EC:2.7.11.11]
	ACC_08577	K04345	protein kinase A [EC:2.7.11.11]
	ACC_05040	K04374	cyclic AMP-dependent transcription factor ATF-4
	ACC_06622	K09048	cyclic AMP-responsive element-binding protein 3
	ACC_05678	K04515	calcium/calmodulin-dependent protein kinase (CaM kinase) II [EC:2.7.11.17]
	ACC_04513	K04447	Janus kinase 2 [EC:2.7.10.2]
	ACC_09596	K00922	phosphatidylinositol-4,5-bisphosphate 3-kinase [EC:2.7.1.153]
	ACC_04314	K02649	phosphoinositide-3-kinase, regulatory subunit

	ACC_04819	K02649	phosphoinositide-3-kinase, regulatory subunit
	ACC_05279	K04456	RAC serine/threonine-protein kinase [EC:2.7.11.1]
	ACC_09439	K14387	solute carrier family 5 (high affinity choline transporter), member 7
**04728 Dopaminergic synapse**	ACC_09941	K00501	tyrosine 3-monooxygenase [EC:1.14.16.2]
	ACC_00334	K01593	aromatic-L-amino-acid decarboxylase [EC:4.1.1.28]
	ACC_07611	K01593	aromatic-L-amino-acid decarboxylase [EC:4.1.1.28]
	ACC_09788	K08155	MFS transporter, DHA1 family, solute carrier family 18 (vesicular amine transporter), member 1/2
	ACC_04047	K04634	guanine nucleotide-binding protein G(q) subunit alpha
	ACC_02939	K05858	phosphatidylinositol phospholipase C, beta [EC:3.1.4.11]
	ACC_05612	K05858	phosphatidylinositol phospholipase C, beta [EC:3.1.4.11]
	ACC_05838	K04958	inositol 1,4,5-triphosphate receptor type 1
	ACC_06604	K02183	calmodulin
	ACC_05678	K04515	calcium/calmodulin-dependent protein kinase (CaM kinase) II [EC:2.7.11.17]
	ACC_05684	K04348	protein phosphatase 3, catalytic subunit [EC:3.1.3.16]
	ACC_08696	K04348	protein phosphatase 3, catalytic subunit [EC:3.1.3.16]
	ACC_02607	K02677	classical protein kinase C [EC:2.7.11.13]
	ACC_01798	K04632	guanine nucleotide-binding protein G(s) subunit alpha
	ACC_06514	K08045	adenylate cyclase 5 [EC:4.6.1.1]
	ACC_04702	K04345	protein kinase A [EC:2.7.11.11]
	ACC_08577	K04345	protein kinase A [EC:2.7.11.11]
	ACC_06042	K04450	cyclic AMP-dependent transcription factor ATF-2
	ACC_05040	K04374	cyclic AMP-dependent transcription factor ATF-4
	ACC_06622	K09048	cyclic AMP-responsive element-binding protein 3
	ACC_00669	K04441	p38 MAP kinase [EC:2.7.11.24]
	ACC_06215	K04440	c-Jun N-terminal kinase [EC:2.7.11.24]
	ACC_06070	K06269	protein phosphatase 1, catalytic subunit [EC:3.1.3.16]
	ACC_10042	K06269	protein phosphatase 1, catalytic subunit [EC:3.1.3.16]
	ACC_06850	K04630	guanine nucleotide-binding protein G(i) subunit alpha
	ACC_07382	K04534	guanine nucleotide-binding protein G(o) subunit alpha
	ACC_09212	K04536	guanine nucleotide-binding protein G(I)/G(S)/G(T) subunit beta-1
	ACC_04262	K04539	guanine nucleotide-binding protein subunit beta-5
	ACC_06468	K04547	guanine nucleotide-binding protein G(I)/G(S)/G(O) subunit gamma-13
	ACC_03696	K04382	protein phosphatase 2 (formerly 2A), catalytic subunit [EC:3.1.3.16]
	ACC_02649	K03456	protein phosphatase 2 (formerly 2A), regulatory subunit A
	ACC_03152	K04354	protein phosphatase 2 (formerly 2A), regulatory subunit B
	ACC_00281	K11583	protein phosphatase 2 (formerly 2A), regulatory subunit B''
	ACC_09809	K11583	protein phosphatase 2 (formerly 2A), regulatory subunit B''
	ACC_00883	K11584	protein phosphatase 2 (formerly 2A), regulatory subunit B'
	ACC_08181	K11584	protein phosphatase 2 (formerly 2A), regulatory subunit B'
	ACC_05279	K04456	RAC serine/threonine-protein kinase [EC:2.7.11.1]
	ACC_06459	K03083	glycogen synthase kinase 3 beta [EC:2.7.11.26]
	ACC_08922	K05210	glutamate [NMDA] receptor subunit epsilon-2
	ACC_06659	K10396	kinesin family member 5
	ACC_01245	K02223	circadian locomoter output cycles kaput protein [EC:2.3.1.48]
	ACC_03520	K02296	aryl hydrocarbon receptor nuclear translocator-like protein 1
	ACC_06157	K05036	solute carrier family 6 (neurotransmitter transporter, dopamine) member 3

	ACC_01485	K00274	monoamine oxidase [EC:1.4.3.4]
**04726 Serotonergic synapse**	ACC_06650	K00502	tryptophan 5-monooxygenase [EC:1.14.16.4]
	ACC_00334	K01593	aromatic-L-amino-acid decarboxylase [EC:4.1.1.28]
	ACC_07611	K01593	aromatic-L-amino-acid decarboxylase [EC:4.1.1.28]
	ACC_09788	K08155	MFS transporter, DHA1 family, solute carrier family 18 (vesicular amine transporter), member 1/2
	ACC_09424	K04157	5-hydroxytryptamine receptor 2
	ACC_04047	K04634	guanine nucleotide-binding protein G(q) subunit alpha
	ACC_02939	K05858	phosphatidylinositol phospholipase C, beta [EC:3.1.4.11]
	ACC_05612	K05858	phosphatidylinositol phospholipase C, beta [EC:3.1.4.11]
	ACC_05838	K04958	inositol 1,4,5-triphosphate receptor type 1
	ACC_02607	K02677	classical protein kinase C [EC:2.7.11.13]
	ACC_05659	K04371	extracellular signal-regulated kinase 1/2 [EC:2.7.11.24]
	ACC_06642	K04163	5-hydroxytryptamine receptor 7
	ACC_01798	K04632	guanine nucleotide-binding protein G(s) subunit alpha
	ACC_06514	K08045	adenylate cyclase 5 [EC:4.6.1.1]
	ACC_04702	K04345	protein kinase A [EC:2.7.11.11]
	ACC_08577	K04345	protein kinase A [EC:2.7.11.11]
	ACC_08806	K05181	gamma-aminobutyric acid receptor subunit beta
	ACC_04712	K04520	amyloid beta A4 protein
	ACC_08581	K04153	5-hydroxytryptamine receptor 1
	ACC_06850	K04630	guanine nucleotide-binding protein G(i) subunit alpha
	ACC_07382	K04534	guanine nucleotide-binding protein G(o) subunit alpha
	ACC_09212	K04536	guanine nucleotide-binding protein G(I)/G(S)/G(T) subunit beta-1
	ACC_04262	K04539	guanine nucleotide-binding protein subunit beta-5
	ACC_06468	K04547	guanine nucleotide-binding protein G(I)/G(S)/G(O) subunit gamma-13
	ACC_02813	K07827	GTPase KRas
	ACC_08503	K04368	mitogen-activated protein kinase kinase 1 [EC:2.7.12.2]
	ACC_09588	K04368	mitogen-activated protein kinase kinase 1 [EC:2.7.12.2]
	ACC_01485	K00274	monoamine oxidase [EC:1.4.3.4]
**04720 Long-term potentiation**	ACC_01531	K08048	adenylate cyclase 8 [EC:4.6.1.1]
	ACC_04702	K04345	protein kinase A [EC:2.7.11.11]
	ACC_08577	K04345	protein kinase A [EC:2.7.11.11]
	ACC_06070	K06269	protein phosphatase 1, catalytic subunit [EC:3.1.3.16]
	ACC_10042	K06269	protein phosphatase 1, catalytic subunit [EC:3.1.3.16]
	ACC_05678	K04515	calcium/calmodulin-dependent protein kinase (CaM kinase) II [EC:2.7.11.17]
	ACC_03570	K04353	Ras-related protein Rap-1A
	ACC_05684	K04348	protein phosphatase 3, catalytic subunit [EC:3.1.3.16]
	ACC_08696	K04348	protein phosphatase 3, catalytic subunit [EC:3.1.3.16]
	ACC_05321	K06268	protein phosphatase 3, regulatory subunit
	ACC_05594	K06268	protein phosphatase 3, regulatory subunit
	ACC_06938	K05208	glutamate receptor, ionotropic, N-methyl D-aspartate 1
	ACC_09518	K05208	glutamate receptor, ionotropic, N-methyl D-aspartate 1
	ACC_08922	K05210	glutamate [NMDA] receptor subunit epsilon-2
	ACC_06604	K02183	calmodulin
	ACC_03143	K04498	E1A/CREB-binding protein [EC:2.3.1.48]
	ACC_05040	K04374	cyclic AMP-dependent transcription factor ATF-4
	ACC_02813	K07827	GTPase KRas
	ACC_08503	K04368	mitogen-activated protein kinase kinase 1 [EC:2.7.12.2]
	ACC_09588	K04368	mitogen-activated protein kinase kinase 1 [EC:2.7.12.2]
	ACC_05659	K04371	extracellular signal-regulated kinase 1/2 [EC:2.7.11.24]
	ACC_05456	K04373	p90 ribosomal S6 kinase [EC:2.7.11.1]
	ACC_05838	K04958	inositol 1,4,5-triphosphate receptor type 1
	ACC_02607	K02677	classical protein kinase C [EC:2.7.11.13]
	ACC_04047	K04634	guanine nucleotide-binding protein G(q) subunit alpha
	ACC_02939	K05858	phosphatidylinositol phospholipase C, beta [EC:3.1.4.11]
	ACC_05612	K05858	phosphatidylinositol phospholipase C, beta [EC:3.1.4.11]

**04730 Long-term depression**	ACC_02494	K12318	guanylate cyclase soluble subunit alpha [EC:4.6.1.2]
	ACC_09018	K12319	guanylate cyclase soluble subunit beta [EC:4.6.1.2]
	ACC_09607	K07376	protein kinase, cGMP-dependent [EC:2.7.11.12]
	ACC_02649	K03456	protein phosphatase 2 (formerly 2A), regulatory subunit A
	ACC_03696	K04382	protein phosphatase 2 (formerly 2A), catalytic subunit [EC:3.1.3.16]
	ACC_02813	K07827	GTPase KRas
	ACC_08503	K04368	mitogen-activated protein kinase kinase 1 [EC:2.7.12.2]
	ACC_09588	K04368	mitogen-activated protein kinase kinase 1 [EC:2.7.12.2]
	ACC_05659	K04371	extracellular signal-regulated kinase 1/2 [EC:2.7.11.24]
	ACC_06850	K04630	guanine nucleotide-binding protein G(i) subunit alpha
	ACC_07382	K04534	guanine nucleotide-binding protein G(o) subunit alpha
	ACC_01798	K04632	guanine nucleotide-binding protein G(s) subunit alpha
	ACC_00771	K04346	guanine nucleotide-binding protein subunit alpha-12
	ACC_02607	K02677	classical protein kinase C [EC:2.7.11.13]
	ACC_04047	K04634	guanine nucleotide-binding protein G(q) subunit alpha
	ACC_02939	K05858	phosphatidylinositol phospholipase C, beta [EC:3.1.4.11]
	ACC_05612	K05858	phosphatidylinositol phospholipase C, beta [EC:3.1.4.11]
	ACC_05838	K04958	inositol 1,4,5-triphosphate receptor type 1
	ACC_08643	K04578	corticotropin releasing hormone receptor 1
**04723 Retrograde endocannabinoid signaling**	ACC_04047	K04634	guanine nucleotide-binding protein G(q) subunit alpha
	ACC_02939	K05858	phosphatidylinositol phospholipase C, beta [EC:3.1.4.11]
	ACC_05612	K05858	phosphatidylinositol phospholipase C, beta [EC:3.1.4.11]
	ACC_05838	K04958	inositol 1,4,5-triphosphate receptor type 1
	ACC_02607	K02677	classical protein kinase C [EC:2.7.11.13]
	ACC_01972	K13806	sn1-specific diacylglycerol lipase [EC:3.1.1.-]
	ACC_06850	K04630	guanine nucleotide-binding protein G(i) subunit alpha
	ACC_07382	K04534	guanine nucleotide-binding protein G(o) subunit alpha
	ACC_09212	K04536	guanine nucleotide-binding protein G(I)/G(S)/G(T) subunit beta-1
	ACC_04262	K04539	guanine nucleotide-binding protein subunit beta-5
	ACC_06468	K04547	guanine nucleotide-binding protein G(I)/G(S)/G(O) subunit gamma-13
	ACC_05659	K04371	extracellular signal-regulated kinase 1/2 [EC:2.7.11.24]
	ACC_06215	K04440	c-Jun N-terminal kinase [EC:2.7.11.24]
	ACC_00669	K04441	p38 MAP kinase [EC:2.7.11.24]
	ACC_00796	K08042	adenylate cyclase 2 [EC:4.6.1.1]
	ACC_02640	K08043	adenylate cyclase 3 [EC:4.6.1.1]
	ACC_07908	K08043	adenylate cyclase 3 [EC:4.6.1.1]
	ACC_06514	K08045	adenylate cyclase 5 [EC:4.6.1.1]
	ACC_01531	K08048	adenylate cyclase 8 [EC:4.6.1.1]
	ACC_00311	K08049	adenylate cyclase 9 [EC:4.6.1.1]
	ACC_04702	K04345	protein kinase A [EC:2.7.11.11]
	ACC_08577	K04345	protein kinase A [EC:2.7.11.11]
	ACC_05210	K15015	solute carrier family 32 (vesicular inhibitory amino acid transporter)
	ACC_06768	K05175	gamma-aminobutyric acid receptor subunit alpha
	ACC_09040	K05175	gamma-aminobutyric acid receptor subunit alpha
	ACC_08806	K05181	gamma-aminobutyric acid receptor subunit beta
**04721 Synaptic vesicle cycle**	ACC_05210	K15015	solute carrier family 32 (vesicular inhibitory amino acid transporter)
	ACC_09788	K08155	MFS transporter, DHA1 family, solute carrier family 18 (vesicular amine transporter), member 1/2
	ACC_04539	K15290	synaptotagmin-1
	ACC_07814	K13504	vesicle-associated membrane protein 2
	ACC_09252	K04560	syntaxin 1A
	ACC_00326	K15292	syntaxin-binding protein 1
	ACC_02920	K15293	protein unc-13

	ACC_01809	K06027	vesicle-fusing ATPase [EC:3.6.4.6]
	ACC_05292	K15296	alpha-soluble NSF attachment protein
	ACC_03981	K01528	dynamin GTPase [EC:3.6.5.5]
	ACC_09522	K01528	dynamin GTPase [EC:3.6.5.5]
	ACC_02691	K04646	clathrin heavy chain
	ACC_02044	K11824	AP-2 complex subunit alpha
	ACC_01012	K11826	AP-2 complex subunit mu-1
	ACC_05712	K11827	AP-2 complex subunit sigma-1
	ACC_02136	K02145	V-type H+-transporting ATPase subunit A [EC:3.6.3.14]
	ACC_06442	K02147	V-type H+-transporting ATPase subunit B [EC:3.6.3.14]
	ACC_00315	K02148	V-type H+-transporting ATPase subunit C [EC:3.6.3.14]
	ACC_03032	K02149	V-type H+-transporting ATPase subunit D [EC:3.6.3.14]
	ACC_04528	K02150	V-type H+-transporting ATPase subunit E [EC:3.6.3.14]
	ACC_01510	K02151	V-type H+-transporting ATPase subunit F [EC:3.6.3.14]
	ACC_05172	K02152	V-type H+-transporting ATPase subunit G [EC:3.6.3.14]
	ACC_00145	K02153	V-type H+-transporting ATPase subunit H [EC:3.6.3.14]
	ACC_01478	K02154	V-type H+-transporting ATPase subunit I [EC:3.6.3.14]
	ACC_05428	K02154	V-type H+-transporting ATPase subunit I [EC:3.6.3.14]
	ACC_03686	K02146	V-type H+-transporting ATPase subunit AC39 [EC:3.6.3.14]
	ACC_00460	K02144	V-type H+-transporting ATPase 54 kD subunit [EC:3.6.3.14]
	ACC_02496	K02155	V-type H+-transporting ATPase 16kDa proteolipid subunit [EC:3.6.3.14]
	ACC_03982	K03661	V-type H+-transporting ATPase 21kDa proteolipid subunit [EC:3.6.3.14]
**04722 Neurotrophin signaling pathway**	ACC_02507	K04364	growth factor receptor-binding protein 2
	ACC_02813	K07827	GTPase KRas
	ACC_08503	K04368	mitogen-activated protein kinase kinase 1 [EC:2.7.12.2]
	ACC_09588	K04368	mitogen-activated protein kinase kinase 1 [EC:2.7.12.2]
	ACC_05659	K04371	extracellular signal-regulated kinase 1/2 [EC:2.7.11.24]
	ACC_05456	K04373	p90 ribosomal S6 kinase [EC:2.7.11.1]
	ACC_04555	K04445	ribosomal protein S6 kinase alpha-5 [EC:2.7.11.1]
	ACC_05040	K04374	cyclic AMP-dependent transcription factor ATF-4
	ACC_08568	K12460	ankyrin repeat-rich membrane spanning protein
	ACC_03341	K04438	proto-oncogene C-crk
	ACC_01287	K06277	Rap guanine nucleotide exchange factor (GEF) 1
	ACC_03570	K04353	Ras-related protein Rap-1A
	ACC_00669	K04441	p38 MAP kinase [EC:2.7.11.24]
	ACC_05965	K06279	src homology 2 domain-containing transforming protein C
	ACC_04314	K02649	phosphoinositide-3-kinase, regulatory subunit
	ACC_04819	K02649	phosphoinositide-3-kinase, regulatory subunit
	ACC_09596	K00922	phosphatidylinositol-4,5-bisphosphate 3-kinase [EC:2.7.1.153]
	ACC_05279	K04456	RAC serine/threonine-protein kinase [EC:2.7.11.1]
	ACC_06459	K03083	glycogen synthase kinase 3 beta [EC:2.7.11.26]
	ACC_03475	K16172	insulin receptor substrate 1
	ACC_03620	K01116	phosphatidylinositol phospholipase C, gamma-1 [EC:3.1.4.11]
	ACC_06604	K02183	calmodulin
	ACC_05678	K04515	calcium/calmodulin-dependent protein kinase (CaM kinase) II [EC:2.7.11.17]
	ACC_00028	K06619	abelson tyrosine-protein kinase 1 [EC:2.7.10.2]
	ACC_06822	K07293	tyrosine-protein phosphatase non-receptor type 11 [EC:3.1.3.48]
	ACC_04235	K12462	Rho GDP-dissociation inhibitor
	ACC_05713	K12462	Rho GDP-dissociation inhibitor
	ACC_08589	K04513	Ras homolog gene family, member A
	ACC_01946	K04393	cell division control protein 42
	ACC_02707	K04393	cell division control protein 42

ACC_01700	K04392	Ras-related C3 botulinum toxin substrate 1
ACC_07652	K04392	Ras-related C3 botulinum toxin substrate 1
ACC_09580	K04426	mitogen-activated protein kinase kinase kinase 5 [EC:2.7.11.25]
ACC_04876	K04431	mitogen-activated protein kinase kinase 7 [EC:2.7.12.2]
ACC_06215	K04440	c-Jun N-terminal kinase [EC:2.7.11.24]
ACC_02138	K04448	transcription factor AP-1
ACC_03524	K10148	tumor protein p73
ACC_06165	K03175	TNF receptor-associated factor 6
ACC_00886	K06630	14-3-3 protein epsilon
ACC_02410	K06630	14-3-3 protein epsilon
ACC_00659	K07209	inhibitor of nuclear factor kappa-B kinase subunit beta [EC:2.7.11.10]
ACC_08580	K04505	presenilin 1 [EC:3.4.23.-]

Supplementary Table 36 Genes involved in Develop of A.cerana

Pathway	Gene	KO	Product
**04320 Dorso-ventral axis formation**	ACC_02507	K04364	growth factor receptor-binding protein 2
	ACC_02813	K07827	GTPase KRas
	ACC_01516	K02644	pole hole
	ACC_08503	K04368	mitogen-activated protein kinase kinase 1 [EC:2.7.12.2]
	ACC_09588	K04368	mitogen-activated protein kinase kinase 1 [EC:2.7.12.2]
	ACC_05659	K04371	extracellular signal-regulated kinase 1/2 [EC:2.7.11.24]
	ACC_03782	K03211	ETS translocation variant 6/7
	ACC_06525	K02678	c-ets proto-oncogene protein
	ACC_08530	K02174	broad
	ACC_10099	K02599	Notch
	ACC_07066	K02602	oo18 RNA-binding protein
	ACC_03384	K02156	aubergine
	ACC_05752	K02156	aubergine
	ACC_03075	K03102	squid
	ACC_03534	K03102	squid
	ACC_06198	K02098	spire
**04360 Axon guidance**	ACC_01700	K04392	Ras-related C3 botulinum toxin substrate 1
	ACC_07652	K04392	Ras-related C3 botulinum toxin substrate 1
	ACC_06728	K07520	actin-binding LIM protein
	ACC_05684	K04348	protein phosphatase 3, catalytic subunit [EC:3.1.3.16]
	ACC_08696	K04348	protein phosphatase 3, catalytic subunit [EC:3.1.3.16]
	ACC_05321	K06268	protein phosphatase 3, regulatory subunit
	ACC_05594	K06268	protein phosphatase 3, regulatory subunit
	ACC_09321	K04446	nuclear factor of activated T-cells, cytoplasmic
	ACC_08589	K04513	Ras homolog gene family, member A
	ACC_02576	K04514	Rho-associated, coiled-coil containing protein kinase [EC:2.7.11.1]
	ACC_05843	K05463	ephrin-B
	ACC_06422	K05725	PTK2 protein tyrosine kinase 2 [EC:2.7.10.2]
	ACC_08177	K05110	Eph receptor B1 [EC:2.7.10.1]
	ACC_00028	K06619	proto-oncogene tyrosine-protein kinase ABL1 [EC:2.7.10.2]
	ACC_01946	K04393	cell division control protein 42
	ACC_02707	K04393	cell division control protein 42
	ACC_08442	K04409	p21-activated kinase 1 [EC:2.7.11.1]
	ACC_03125	K04352	Ras GTPase-activating protein 1
	ACC_02813	K07827	GTPase KRas
	ACC_05659	K04371	extracellular signal-regulated kinase 1/2 [EC:2.7.11.24]
	ACC_06850	K04630	guanine nucleotide-binding protein G(i) subunit alpha
	ACC_09770	K06839	slit 2
	ACC_02081	K07526	SLIT-ROBO Rho GTPase activating protein
	ACC_08488	K06820	plexin A
	ACC_06459	K03083	glycogen synthase kinase 3 beta [EC:2.7.11.26]
	ACC_05256	K02090	cyclin-dependent kinase 5 [EC:2.7.11.22]
	ACC_08280	K05743	LIM domain kinase 1 [EC:2.7.11.1]
	ACC_02844	K05765	cofilin

	ACC_00148	K06841	semaphorin 5
	ACC_08318	K05719	integrin beta 1
**04380 Osteoclast differentiation**	ACC_02507	K04364	growth factor receptor-binding protein 2
	ACC_05659	K04371	extracellular signal-regulated kinase 1/2 [EC:2.7.11.24]
	ACC_09596	K00922	phosphatidylinositol-4,5-bisphosphate 3-kinase [EC:2.7.1.153]
	ACC_04314	K02649	phosphoinositide-3-kinase, regulatory subunit
	ACC_04819	K02649	phosphoinositide-3-kinase, regulatory subunit
	ACC_05279	K04456	RAC serine/threonine-protein kinase [EC:2.7.11.1]
	ACC_06165	K03175	TNF receptor-associated factor 6
	ACC_09256	K04427	mitogen-activated protein kinase kinase kinase 7 [EC:2.7.11.25]
	ACC_06441	K04403	TAK1-binding protein 1
	ACC_00659	K07209	inhibitor of nuclear factor kappa-B kinase subunit beta [EC:2.7.11.10]
	ACC_08503	K04368	mitogen-activated protein kinase kinase 1 [EC:2.7.12.2]
	ACC_09588	K04368	mitogen-activated protein kinase kinase 1 [EC:2.7.12.2]
	ACC_00669	K04441	p38 MAP kinase [EC:2.7.11.24]
	ACC_04876	K04431	mitogen-activated protein kinase kinase 7 [EC:2.7.12.2]
	ACC_06215	K04440	c-Jun N-terminal kinase [EC:2.7.11.24]
	ACC_02138	K04448	transcription factor AP-1
	ACC_01700	K04392	Ras-related C3 botulinum toxin substrate 1
	ACC_07652	K04392	Ras-related C3 botulinum toxin substrate 1
	ACC_00510	K07364	tec protein tyrosine kinase [EC:2.7.10.2]
	ACC_05684	K04348	protein phosphatase 3, catalytic subunit [EC:3.1.3.16]
	ACC_08696	K04348	protein phosphatase 3, catalytic subunit [EC:3.1.3.16]
	ACC_05321	K06268	protein phosphatase 3, regulatory subunit
	ACC_05594	K06268	protein phosphatase 3, regulatory subunit
	ACC_07906	K08601	ubiquitin thioesterase CYLD [EC:3.1.2.15]
	ACC_02853	K14381	sequestosome 1

Supplementary Table 37 Signal Transduction Pathway of A.cerana

Pathway	Gene	KO	Product
ko04020 Calcium signaling pathway	ACC_06516	K05857	PLCD; phosphatidylinositol phospholipase C, delta [EC:3.1.4.11]
	ACC_03306	K15040	VDAC2; voltage-dependent anion channel protein 2
	ACC_09424	K04157	HTR2; 5-hydroxytryptamine receptor 2
	ACC_03182	K04962	RYR2; ryanodine receptor 2
	ACC_03620	K01116	PLCG1; phosphatidylinositol phospholipase C, gamma-1 [EC:3.1.4.11]
	ACC_10134	K05853	ATP2A; Ca2+ transporting ATPase, sarcoplasmic/endoplasmic reticulum [EC:3.6.3.8]
	ACC_07985	K05860	PLCE; phosphatidylinositol phospholipase C, epsilon [EC:3.1.4.11]
	ACC_00796	K08042	ADCY2; adenylate cyclase 2 [EC:4.6.1.1]
	ACC_02557	K00871	PHKG; phosphorylase kinase gamma subunit [EC:2.7.11.19]
	ACC_06604	K02183	CALM; calmodulin
	ACC_06927	K04131	CHRM3; muscarinic acetylcholine receptor M3
	ACC_01531	K08048	ADCY8; adenylate cyclase 8 [EC:4.6.1.1]
	ACC_01798	K04632	GNAS; guanine nucleotide-binding protein G(s) subunit alpha
	ACC_05838	K04958	ITPR1; inositol 1,4,5-triphosphate receptor type 1
	ACC_05870	K04718	E2.7.1.91; sphingosine kinase [EC:2.7.1.91]
	ACC_05678	K04515	CAMK2; calcium/calmodulin-dependent protein kinase (CaM kinase) II [EC:2.7.11.17]
	ACC_06642	K04163	HTR7; 5-hydroxytryptamine receptor 7
	ACC_08346	K05849	SLC8A; solute carrier family 8 (sodium/calcium exchanger)
	ACC_02705	K16059	STIM; stromal interaction molecule
	ACC_00311	K08049	ADCY9; adenylate cyclase 9 [EC:4.6.1.1]
	ACC_02607	K02677	CPKC; classical protein kinase C [EC:2.7.11.13]
	ACC_09711	K00907	MYLK; myosin-light-chain kinase [EC:2.7.11.18]

	ACC_04827	K05863	SLC25A4S; solute carrier family 25 (mitochondrial adenine nucleotide translocator), member 4/5/6/31
	ACC_04047	K04634	GNAQ; guanine nucleotide-binding protein G(q) subunit alpha
	ACC_06938,A	K05208	GRIN1; glutamate receptor ionotropic, NMDA 1
	ACC_02640,A	K08043	ADCY3; adenylate cyclase 3 [EC:4.6.1.1]
	ACC_05684,ACC_08696	K04348	PPP3C; protein phosphatase 3, catalytic subunit [EC:3.1.3.16]
	ACC_03742,ACC_05372	K09565	PPIF; peptidyl-prolyl isomerase F (cyclophilin D) [EC:5.2.1.8]
	ACC_01879,ACC_07739	K00911	E2.7.1.127; 1D-myo-inositol-triphosphate 3-kinase [EC:2.7.1.127]
	ACC_05321,A	K06268	PPP3R; protein phosphatase 3, regulatory subunit
	ACC_00887,ACC_04173	K16056	ORAI1; calcium release-activated calcium channel protein 1
	ACC_01233,A	K07190	PHKA_B; phosphorylase kinase alpha/beta subunit
	ACC_02939,ACC_05612	K05858	PLCB; phosphatidylinositol phospholipase C, beta [EC:3.1.4.11]
	ACC_04702,A	K04345	PKA; protein kinase A [EC:2.7.11.11]
ko04012 ErbB signaling pathway	ACC_05516	K03099	SOS; son of sevenless
	ACC_03579	K05704	SRC; tyrosine-protein kinase Src [EC:2.7.10.2]
	ACC_05659	K04371	ERK1_2; extracellular signal-regulated kinase 1/2 [EC:2.7.11.24]
	ACC_06422	K05725	PTK2; focal adhesion kinase 1 [EC:2.7.10.2]
	ACC_03620	K01116	PLCG1; phosphatidylinositol phospholipase C, gamma-1 [EC:3.1.4.11]
	ACC_02138	K04448	JUN; transcription factor AP-1
	ACC_00849	K07205	EIF4EBP1; eukaryotic translation initiation factor 4E binding protein 1
	ACC_02507	K04364	GRB2; growth factor receptor-binding protein 2
	ACC_09596	K00922	PIK3C; phosphatidylinositol-4,5-bisphosphate 3-kinase [EC:2.7.1.153]

	ACC_03561	K04430	MAP2K4; mitogen-activated protein kinase kinase 4 [EC:2.7.12.2]
	ACC_05279	K04456	AKT; RAC serine/threonine-protein kinase [EC:2.7.11.1]
	ACC_05965	K06279	SHC; src homology 2 domain-containing transforming protein C
	ACC_02813	K07827	KRAS; GTPase KRas
	ACC_00028	K06619	ABL1; abelson tyrosine-protein kinase 1 [EC:2.7.10.2]
	ACC_05678	K04515	CAMK2; calcium/calmodulin-dependent protein kinase (CaM kinase) II [EC:2.7.11.17]
	ACC_06215	K04440	JNK; c-Jun N-terminal kinase [EC:2.7.11.24]
	ACC_06128	K07203	FRAP; FKBP12-rapamycin complex-associated protein
	ACC_06459	K03083	GSK3B; glycogen synthase kinase 3 beta [EC:2.7.11.26]
	ACC_02607	K02677	CPKC; classical protein kinase C [EC:2.7.11.13]
	ACC_04876	K04431	MAP2K7; mitogen-activated protein kinase kinase 7 [EC:2.7.12.2]
	ACC_03341	K04438	CRK; proto-oncogene C-crk
	ACC_02669	K07365	NCK; NCK adaptor protein
	ACC_08442	K04409	PAK1; p21-activated kinase 1 [EC:2.7.11.1]
	ACC_08503,ACC_09588	K04368	MAP2K1; mitogen-activated protein kinase kinase 1 [EC:2.7.12.2]
	ACC_04314,A	K02649	PIK3R; phosphoinositide-3-kinase, regulatory subunit
	ACC_03970,A	K04707	CBL; E3 ubiquitin-protein ligase CBL [EC:6.3.2.19]
	ACC_01430,A	K04688	RPS6KB; p70 ribosomal S6 kinase [EC:2.7.11.1]
ko04340 Hedgehog signaling pathway	ACC_06102	K06232	GAS1; growth arrest-specific 1
	ACC_04962	K06225	PTCH1; patched 1
	ACC_04161	K01357	WNT10; wingless-type MMTV integration site family, member 10
	ACC_07470	K08960	CSNK1E; casein kinase 1, epsilon [EC:2.7.11.1]
	ACC_03414	K16799	CI; transcriptional activator cubitus interruptus
	ACC_07092	K00444	WNT5; wingless-type MMTV integration site family, member 5

	ACC_08081	K03362	FBXW1_11; F-box and WD-40 domain protein 1/11
	ACC_04149	K04662	BMP2_4; bone morphogenetic protein 2/4
	ACC_04276	K06224	HH; hedgehog
	ACC_05811	K06226	SMO; smoothened
	ACC_03709	K06228	FU; fused [EC:2.7.11.1]
	ACC_00963	K06229	SUFU; suppressor of fused
	ACC_08505	K06234	RAB23; Ras-related protein Rab-23
	ACC_06617	K08957	CSNK1A; casein kinase 1, alpha [EC:2.7.11.1]
	ACC_09370	K08958	CSNK1G; casein kinase 1, gamma [EC:2.7.11.1]
	ACC_06459	K03083	GSK3B; glycogen synthase kinase 3 beta [EC:2.7.11.26]
	ACC_04160	K03209	WNT1; wingless-type MMTV integration site family, member 1
	ACC_04702,A	K04345	PKA; protein kinase A [EC:2.7.11.11]
ko04066 HIF-1 signaling pathway	ACC_05968	K04372	MKNK; MAP kinase interacting serine/threonine kinase [EC:2.7.11.1]
	ACC_07676	K03871	VHL; von Hippel-Lindau disease tumor supressor
	ACC_05659	K04371	ERK1_2; extracellular signal-regulated kinase 1/2 [EC:2.7.11.24]
	ACC_05179	K03872	TCEB1; transcription elongation factor B, polypeptide 1
	ACC_03620	K01116	PLCG1; phosphatidylinositol phospholipase C, gamma-1 [EC:3.1.4.11]
	ACC_06030	K03870	CUL2; cullin 2
	ACC_00849	K07205	EIF4EBP1; eukaryotic translation initiation factor 4E binding protein 1
	ACC_07083	K08268	HIF1A; hypoxia-inducible factor 1 alpha
	ACC_03143	K04498	EP300; E1A/CREB-binding protein [EC:2.3.1.48]
	ACC_09596	K00922	PIK3C; phosphatidylinositol-4,5-bisphosphate 3-kinase [EC:2.7.1.153]
	ACC_00239	K03873	TCEB2; transcription elongation factor B, polypeptide 2
	ACC_05279	K04456	AKT; RAC serine/threonine-protein kinase [EC:2.7.11.1]

	ACC_07572	K07299	SLC2A1; MFS transporter, SP family, solute carrier family 2 (facilitated glucose transporter), member 1
	ACC_00303	K09097	ARNT; aryl hydrocarbon receptor nuclear translocator
	ACC_02658	K05096	FLT1; FMS-like tyrosine kinase 1 [EC:2.7.10.1]
	ACC_08626	K02991	RP-S6e; small subunit ribosomal protein S6e
	ACC_03716	K01689	ENO; enolase [EC:4.2.1.11]
	ACC_05678	K04515	CAMK2; calcium/calmodulin-dependent protein kinase (CaM kinase) II [EC:2.7.11.17]
	ACC_06128	K07203	FRAP; FKBP12-rapamycin complex-associated protein
	ACC_02464	K09592	EGLN; hypoxia-inducible factor prolyl hydroxylase [EC:1.14.11.-]
	ACC_02607	K02677	CPKC; classical protein kinase C [EC:2.7.11.13]
	ACC_03198	K03868	RBX1; RING-box protein 1
	ACC_01773	K00161	PDHA; pyruvate dehydrogenase E1 component subunit alpha [EC:1.2.4.1]
	ACC_02832	K04527	INSR; insulin receptor [EC:2.7.10.1]
	ACC_06381	K00844	HK; hexokinase [EC:2.7.1.1]
	ACC_08503,ACC_09588	K04368	MAP2K1; mitogen-activated protein kinase kinase 1 [EC:2.7.12.2]
	ACC_04314,A	K02649	PIK3R; phosphoinositide-3-kinase, regulatory subunit
	ACC_00732,ACC_01070	K00134	GAPDH; glyceraldehyde 3-phosphate dehydrogenase [EC:1.2.1.12]
	ACC_02438,A	K03259	EIF4E; translation initiation factor 4E
	ACC_00654,ACC_01717	K00162	PDHB; pyruvate dehydrogenase E1 component subunit beta [EC:1.2.4.1]
	ACC_01430,A	K04688	RPS6KB; p70 ribosomal S6 kinase [EC:2.7.11.1]
	ACC_03573,ACC_08898,AC	K01103	PFKFB; 6-phosphofructo-2-kinase / fructose-2,6-bisphosphatase [EC:2.7.1.105 3.1.3.46]
ko04390 Hippo signaling pathway	ACC_01836	K02085	APC; adenomatosis polyposis coli protein
	ACC_08326	K16796	SOX2; transcription factor SOX2 (SOX group B)
	ACC_00682	K05691	CTNNA; catenin alpha
	ACC_03524	K10148	TP73; tumor protein p73
	ACC_09509	K06069	APKC; atypical protein kinase C [EC:2.7.11.13]

ACC_05786	K16621	BMP7; bone morphogenetic protein 7
ACC_04161	K01357	WNT10; wingless-type MMTV integration site family, member 10
ACC_00484	K02432	FZD1_7; frizzled 1/7
ACC_07470	K08960	CSNK1E; casein kinase 1, epsilon [EC:2.7.11.1]
ACC_09882	K02375	FZD5_8; frizzled 5/8
ACC_08317	K04500	SMAD2_3; mothers against decapentaplegic homolog 2/3
ACC_07092	K00444	WNT5; wingless-type MMTV integration site family, member 5
ACC_06867	K02353	DVL; segment polarity protein dishevelled
ACC_08081	K03362	FBXW1_11; F-box and WD-40 domain protein 1/11
ACC_03696	K04382	PPP2C; protein phosphatase 2 (formerly 2A), catalytic subunit [EC:3.1.3.16]
ACC_03641	K04412	STK3; serine/threonine kinase 3
ACC_00017	K04501	SMAD4; mothers against decapentaplegic homolog 4
ACC_04149	K04662	BMP2_4; bone morphogenetic protein 2/4
ACC_02936	K04676	SMAD1; mothers against decapentaplegic homolog 1
ACC_02700	K04680	ID; DNA-binding protein inhibitor ID
ACC_08897	K06091	MPP5; MAGUK p55 subfamily member 5
ACC_00453	K06093	PARD6; partitioning defective protein 6
ACC_08330	K06685	MOB1; MOB kinase activator 1
ACC_02971	K08791	LATS1_2; serine/threonine-protein kinase LATS1/2 [EC:2.7.11.1]
ACC_04548	K09448	TEAD; transcriptional enhancer factor
ACC_00777	K16681	CRB; protein crumbs
ACC_06589	K16684	NF2; merlin
ACC_03213	K16686	SAV1; scaffold protein salvador
ACC_00308	K02157	AXIN1; axin 1
ACC_07471	K06094	LLGL; lethal(2) giant larvae protein
ACC_09151	K16682	AJUBA; LIM domain-containing protein
ACC_01595	K02105	CTNNB1; catenin beta 1
ACC_08623	K12076	DLG1; disks large protein 1

	ACC_07714	K16687	YAP1; protein yorkie
	ACC_02649	K03456	PPP2R1; protein phosphatase 2 (formerly 2A), regulatory subunit A
	ACC_06639	K16175	SCRIB; protein scribble
	ACC_06459	K03083	GSK3B; glycogen synthase kinase 3 beta [EC:2.7.11.26]
	ACC_00214	K04671	BMPR2; bone morphogenetic protein receptor type-2 [EC:2.7.11.30]
	ACC_04160	K03209	WNT1; wingless-type MMTV integration site family, member 1
	ACC_03152	K04354	PPP2R2; protein phosphatase 2 (formerly 2A), regulatory subunit B
	ACC_00886,A	K06630	YWHAE; 14-3-3 protein epsilon
	ACC_04318,A	K16685	WWC1; protein KIBRA
	ACC_06070,A CC_10042	K06269	PPP1C; protein phosphatase 1, catalytic subunit [EC:3.1.3.16]
	ACC_04920,A CC_05085,AC	K05692	ACTB_G1; actin beta/gamma 1
ko04391 Hippo signaling pathway - fly	ACC_07309	K16679	WGN; wengen
	ACC_05309	K16678	EGR; eiger
	ACC_08452	K06095	MPDZ; multiple PDZ domain protein
	ACC_09509	K06069	APKC; atypical protein kinase C [EC:2.7.11.13]
	ACC_09843	K02306	DALLY; dally
	ACC_09353	K09851	RASSF2_4; Ras association domain-containing protein 2/4
	ACC_07470	K08960	CSNK1E; casein kinase 1, epsilon [EC:2.7.11.1]
	ACC_03696	K04382	PPP2C; protein phosphatase 2 (formerly 2A), catalytic subunit [EC:3.1.3.16]
	ACC_03641	K04412	STK3; serine/threonine kinase 3
	ACC_02936	K04676	SMAD1; mothers against decapentaplegic homolog 1
	ACC_08897	K06091	MPP5; MAGUK p55 subfamily member 5
	ACC_00453	K06093	PARD6; partitioning defective protein 6
	ACC_03055	K06626	CCNE; cyclin E

ACC_08330	K06685	MOB1; MOB kinase activator 1
ACC_02971	K08791	LATS1_2; serine/threonine-protein kinase LATS1/2 [EC:2.7.11.1]
ACC_04548	K09448	TEAD; transcriptional enhancer factor
ACC_05917	K16675	ZDHHC14; probable palmitoyltransferase ZDHHC14 [EC:2.3.1.-]
ACC_09467	K16677	DACHS; dachs
ACC_00777	K16681	CRB; protein crumbs
ACC_06589	K16684	NF2; merlin
ACC_03213	K16686	SAV1; scaffold protein salvador
ACC_03754	K16691	KRN; keren
ACC_07471	K06094	LLGL; lethal(2) giant larvae protein
ACC_09151	K16682	AJUBA; LIM domain-containing protein
ACC_03634	K05628	RERE; arginine-glutamic acid dipeptide repeats protein
ACC_00401	K16676	LPP; lipoma-prefererred partner
ACC_09504	K16669	FAT4; protocadherin Fat 4
ACC_08623	K12076	DLG1; disks large protein 1
ACC_06215	K04440	JNK; c-Jun N-terminal kinase [EC:2.7.11.24]
ACC_07714	K16687	YAP1; protein yorkie
ACC_02649	K03456	PPP2R1; protein phosphatase 2 (formerly 2A), regulatory subunit A
ACC_06639	K16175	SCRIB; protein scribble
ACC_04160	K03209	WNT1; wingless-type MMTV integration site family, member 1
ACC_03152	K04354	PPP2R2; protein phosphatase 2 (formerly 2A), regulatory subunit B
ACC_05718,A	K16683	EX; protein expanded
ACC_05620,A	K16673	LIX1L; LIX1-like protein
ACC_00886,A	K06630	YWHAE; 14-3-3 protein epsilon
ACC_04318,A	K16685	WWC1; protein KIBRA
ACC_03538,A CC_06013,AC	K16507	DCHS1_2; protocadherin-16/23

	ACC_04920,A CC_05085,AC	K05692	ACTB_G1; actin beta/gamma 1
ko04630 Jak-STAT signaling pathway	ACC_05516	K03099	SOS; son of sevenless
	ACC_05736	K16063	PIAS2; E3 SUMO-protein ligase PIAS2 [EC:6.3.2.-]
	ACC_06822	K07293	PTPN11; tyrosine-protein phosphatase non-receptor type 11 [EC:3.1.3.48]
	ACC_02507	K04364	GRB2; growth factor receptor-binding protein 2
	ACC_03143	K04498	EP300; E1A/CREB-binding protein [EC:2.3.1.48]
	ACC_09596	K00922	PIK3C; phosphatidylinositol-4,5-bisphosphate 3-kinase [EC:2.7.1.153]
	ACC_05279	K04456	AKT; RAC serine/threonine-protein kinase [EC:2.7.11.1]
	ACC_01785	K04705	STAM; signal transducing adaptor molecule
	ACC_06492	K04703	SPRED; sprouty-related, EVH1 domain containing
	ACC_04513	K04447	JAK2; Janus kinase 2 [EC:2.7.10.2]
	ACC_04314,A	K02649	PIK3R; phosphoinositide-3-kinase, regulatory subunit
	ACC_03970,A	K04707	CBL; E3 ubiquitin-protein ligase CBL [EC:6.3.2.19]
	ACC_00701,A	K04700	SOCSN; suppressor of cytokine signaling, invertebrate
ko04010 MAPK signaling pathway	ACC_05516	K03099	SOS; son of sevenless
	ACC_06887	K04349	RASGRF1; Ras-specific guanine nucleotide-releasing factor 1
	ACC_03507	K04378	SRF; serum response factor
	ACC_10144	K12326	RASGRF2; Ras-specific guanine nucleotide-releasing factor 2
	ACC_05968	K04372	MKNK; MAP kinase interacting serine/threonine kinase [EC:2.7.11.1]
	ACC_06165	K03175	TRAF6; TNF receptor-associated factor 6
	ACC_05659	K04371	ERK1_2; extracellular signal-regulated kinase 1/2 [EC:2.7.11.24]
	ACC_09097	K04458	PTP; protein-tyrosine phosphatase [EC:3.1.3.48]
	ACC_05040	K04374	ATF4; cyclic AMP-dependent transcription factor ATF-4

ACC_05469	K04461	PPM1B; protein phosphatase 1B (formerly 2C) [EC:3.1.3.16]
ACC_06441	K04403	MAP3K7IP1; TAK1-binding protein 1
ACC_02138	K04448	JUN; transcription factor AP-1
ACC_06042	K04450	ATF2; cyclic AMP-dependent transcription factor ATF-2
ACC_10155	K04468	NLK; nemo like kinase [EC:2.7.11.24]
ACC_00771	K04346	GNA12; guanine nucleotide-binding protein subunit alpha-12
ACC_04555	K04445	MSK1; ribosomal protein S6 kinase alpha-5 [EC:2.7.11.1]
ACC_09256	K04427	MAP3K7; mitogen-activated protein kinase kinase kinase 7 [EC:2.7.11.25]
ACC_02507	K04364	GRB2; growth factor receptor-binding protein 2
ACC_05576	K04422	MAP3K13; mitogen-activated protein kinase kinase kinase 13 [EC:2.7.11.25]
ACC_03125	K04352	RASA1; Ras GTPase-activating protein 1
ACC_03570	K04353	RAP1A; Ras-related protein Rap-1A
ACC_05456	K04373	RPS6KA; p90 ribosomal S6 kinase [EC:2.7.11.1]
ACC_04287	K04380	MAPT; microtubule-associated protein tau
ACC_09270	K04405	SITPEC; evolutionarily conserved signaling intermediate in Toll pathways
ACC_03641	K04412	STK3; serine/threonine kinase 3
ACC_09580	K04426	MAP3K5; mitogen-activated protein kinase kinase kinase 5 [EC:2.7.11.25]
ACC_03918	K04429	TAO; thousand and one amino acid protein kinase [EC:2.7.11.1]
ACC_03561	K04430	MAP2K4; mitogen-activated protein kinase kinase 4 [EC:2.7.12.2]
ACC_04978	K04432	MAP2K3; mitogen-activated protein kinase kinase 3 [EC:2.7.12.2]
ACC_05279	K04456	AKT; RAC serine/threonine-protein kinase [EC:2.7.11.1]

ACC_06451	K04460	PPP5C; protein phosphatase 5 [EC:3.1.3.16]
ACC_00659	K07209	IKBKB; inhibitor of nuclear factor kappa-B kinase subunit beta [EC:2.7.11.10]
ACC_02813	K07827	KRAS; GTPase KRas
ACC_04129	K07830	RRAS2; Ras-related protein R-Ras2
ACC_00512	K08018	RAPGEF2; Rap guanine nucleotide exchange factor (GEF) 2
ACC_00583	K08052	NF1; neurofibromin 1
ACC_05617	K04439	ARRB; beta-arrestin
ACC_05578	K04453	MAX; Max protein
ACC_06215	K04440	JNK; c-Jun N-terminal kinase [EC:2.7.11.24]
ACC_02607	K02677	CPKC; classical protein kinase C [EC:2.7.11.13]
ACC_07410	K05316	CACNA2DN; voltage-dependent calcium channel alpha-2/delta, invertebrate
ACC_04876	K04431	MAP2K7; mitogen-activated protein kinase kinase 7 [EC:2.7.12.2]
ACC_09150	K05317	CACNBN; voltage-dependent calcium channel beta, invertebrate
ACC_03341	K04438	CRK; proto-oncogene C-crk
ACC_08442	K04409	PAK1; p21-activated kinase 1 [EC:2.7.11.1]
ACC_00669	K04441	P38; p38 MAP kinase [EC:2.7.11.24]
ACC_03660	K04437	FLNA; filamin
ACC_08503,ACC_09588	K04368	MAP2K1; mitogen-activated protein kinase kinase 1 [EC:2.7.12.2]
ACC_04070,A	K07831	MRAS; Ras-related protein M-Ras
ACC_05684,ACC_08696	K04348	PPP3C; protein phosphatase 3, catalytic subunit [EC:3.1.3.16]
ACC_06978,ACC_10069	K05315	CACNA1N; voltage-dependent calcium channel alpha 1, invertebrate
ACC_01700,A	K04392	RAC1; Ras-related C3 botulinum toxin substrate 1
ACC_05321,A	K06268	PPP3R; protein phosphatase 3, regulatory subunit
ACC_01946,A	K04393	CDC42; cell division control protein 42
ACC_04702,A	K04345	PKA; protein kinase A [EC:2.7.11.11]

	ACC_03611,A	K03283	HSPA1_8; heat shock 70kDa protein 1/8
	ACC_06453,A CC_08655,AC	K04459	DUSP; dual specificity phosphatase [EC:3.1.3.16 3.1.3.48]
ko04013 MAPK signaling pathway - fly	ACC_03782	K03211	ETV6_7; ETS translocation variant 6/7
	ACC_05516	K03099	SOS; son of sevenless
	ACC_05659	K04371	ERK1_2; extracellular signal-regulated kinase 1/2 [EC:2.7.11.24]
	ACC_09375	K05088	ROS1; proto-oncogene tyrosine-protein kinase ROS [EC:2.7.10.1]
	ACC_06822	K07293	PTPN11; tyrosine-protein phosphatase non-receptor type 11 [EC:3.1.3.48]
	ACC_07262	K12380	RASA3; Ras GTPase-activating protein 3
	ACC_06525	K02678	ETS; c-ets proto-oncogene protein
	ACC_02507	K04364	GRB2; growth factor receptor-binding protein 2
	ACC_01516	K02644	PHL; pole hole
	ACC_02813	K07827	KRAS; GTPase KRas
	ACC_04359	K12377	TSL; torso-like protein
	ACC_08503,A CC_09588	K04368	MAP2K1; mitogen-activated protein kinase kinase 1 [EC:2.7.12.2]
ko04150 mTOR signaling pathway	ACC_07512	K08267	RICTOR; rapamycin-insensitive companion of mTOR
	ACC_05659	K04371	ERK1_2; extracellular signal-regulated kinase 1/2 [EC:2.7.11.24]
	ACC_03475	K16172	IRS1; insulin receptor substrate 1
	ACC_00849	K07205	EIF4EBP1; eukaryotic translation initiation factor 4E binding protein 1
	ACC_07083	K08268	HIF1A; hypoxia-inducible factor 1 alpha
	ACC_09596	K00922	PIK3C; phosphatidylinositol-4,5-bisphosphate 3-kinase [EC:2.7.1.153]
	ACC_01412	K03258	EIF4B; translation initiation factor 4B
	ACC_05456	K04373	RPS6KA; p90 ribosomal S6 kinase [EC:2.7.11.1]
	ACC_05279	K04456	AKT; RAC serine/threonine-protein kinase [EC:2.7.11.1]

	ACC_00652	K06276	PDPK1; 3-phosphoinositide dependent protein kinase-1 [EC:2.7.11.1]
	ACC_03340	K07198	PRKAA; 5'-AMP-activated protein kinase, catalytic alpha subunit [EC:2.7.11.11]
	ACC_01739	K07206	TSC1; tuberous sclerosis 1
	ACC_06503	K07207	TSC2; tuberous sclerosis 2
	ACC_05775	K07208	RHEB; Ras homolog enriched in brain
[truncated: 89,476 more chars]
